# Dynamics and competition of CRISPR–Cas9 ribonucleoproteins and AAV donor-mediated NHEJ, MMEJ and HDR editing

**DOI:** 10.1093/nar/gkaa1251

**Published:** 2021-01-04

**Authors:** Ya-Wen Fu, Xin-Yue Dai, Wen-Tian Wang, Zhi-Xue Yang, Juan-Juan Zhao, Jian-Ping Zhang, Wei Wen, Feng Zhang, Kerby C Oberg, Lei Zhang, Tao Cheng, Xiao-Bing Zhang

**Affiliations:** State Key Laboratory of Experimental Hematology, National Clinical Research Center for Blood Diseases, Institute of Hematology & Blood Diseases Hospital, Chinese Academy of Medical Sciences & Peking Union Medical College, Tianjin 300020, China; State Key Laboratory of Experimental Hematology, National Clinical Research Center for Blood Diseases, Institute of Hematology & Blood Diseases Hospital, Chinese Academy of Medical Sciences & Peking Union Medical College, Tianjin 300020, China; State Key Laboratory of Experimental Hematology, National Clinical Research Center for Blood Diseases, Institute of Hematology & Blood Diseases Hospital, Chinese Academy of Medical Sciences & Peking Union Medical College, Tianjin 300020, China; State Key Laboratory of Experimental Hematology, National Clinical Research Center for Blood Diseases, Institute of Hematology & Blood Diseases Hospital, Chinese Academy of Medical Sciences & Peking Union Medical College, Tianjin 300020, China; State Key Laboratory of Experimental Hematology, National Clinical Research Center for Blood Diseases, Institute of Hematology & Blood Diseases Hospital, Chinese Academy of Medical Sciences & Peking Union Medical College, Tianjin 300020, China; State Key Laboratory of Experimental Hematology, National Clinical Research Center for Blood Diseases, Institute of Hematology & Blood Diseases Hospital, Chinese Academy of Medical Sciences & Peking Union Medical College, Tianjin 300020, China; State Key Laboratory of Experimental Hematology, National Clinical Research Center for Blood Diseases, Institute of Hematology & Blood Diseases Hospital, Chinese Academy of Medical Sciences & Peking Union Medical College, Tianjin 300020, China; State Key Laboratory of Experimental Hematology, National Clinical Research Center for Blood Diseases, Institute of Hematology & Blood Diseases Hospital, Chinese Academy of Medical Sciences & Peking Union Medical College, Tianjin 300020, China; Department of Pathology and Human Anatomy, Loma Linda University, Loma Linda, CA 92350, USA; State Key Laboratory of Experimental Hematology, National Clinical Research Center for Blood Diseases, Institute of Hematology & Blood Diseases Hospital, Chinese Academy of Medical Sciences & Peking Union Medical College, Tianjin 300020, China; CAMS Key Laboratory of Gene Therapy for Blood Diseases, Tianjin 300020, China; Tianjin Laboratory of Blood Disease Gene Therapy, Tianjin 300020, China; State Key Laboratory of Experimental Hematology, National Clinical Research Center for Blood Diseases, Institute of Hematology & Blood Diseases Hospital, Chinese Academy of Medical Sciences & Peking Union Medical College, Tianjin 300020, China; Center for Stem Cell Medicine, Chinese Academy of Medical Sciences, Tianjin 300020, China; Department of Stem Cell & Regenerative Medicine, Peking Union Medical College, Tianjin 300020, China; State Key Laboratory of Experimental Hematology, National Clinical Research Center for Blood Diseases, Institute of Hematology & Blood Diseases Hospital, Chinese Academy of Medical Sciences & Peking Union Medical College, Tianjin 300020, China; Department of Medicine, Loma Linda University, Loma Linda, CA 92350, USA

## Abstract

Investigations of CRISPR gene knockout editing profiles have contributed to enhanced precision of editing outcomes. However, for homology-directed repair (HDR) in particular, the editing dynamics and patterns in clinically relevant cells, such as human iPSCs and primary T cells, are poorly understood. Here, we explore the editing dynamics and DNA repair profiles after the delivery of Cas9-guide RNA ribonucleoprotein (RNP) with or without the adeno-associated virus serotype 6 (AAV6) as HDR donors in four cell types. We show that editing profiles have distinct differences among cell lines. We also reveal the kinetics of HDR mediated by the AAV6 donor template. Quantification of T_50_ (time to reach half of the maximum editing frequency) indicates that short indels (especially +A/T) occur faster than longer (>2 bp) deletions, while the kinetics of HDR falls between NHEJ (non-homologous end-joining) and MMEJ (microhomology-mediated end-joining). As such, AAV6-mediated HDR effectively outcompetes the longer MMEJ-mediated deletions but not NHEJ-mediated indels. Notably, a combination of small molecular compounds M3814 and Trichostatin A (TSA), which potently inhibits predominant NHEJ repairs, leads to a 3-fold increase in HDR efficiency.

## INTRODUCTION

The CRISPR–Cas9 genome editing technology has transformed the landscape of gene therapy, immunotherapy and regenerative medicine (1). CRISPR-edited hematopoietic stem cells have been used in clinical trials to treat multiple disorders, such as AIDS (2) and hemoglobinopathies (3). The human primary T cell has recently become a dominant player in CAR-T cancer therapies (4). Edited T cells have demonstrated safety and efficacy in clinical trials (5,6). Human-induced pluripotent stem cells (iPSCs) provide an ideal source for regenerative medicine due to their unlimited self-renewal and ability to differentiate into multiple tissues (7). Edited iPSCs may offer a universal donor for cell replacement therapy and immunotherapy (8). However, in these cells of clinical significance, editing efficiency, in particular HDR efficiency, has become a bottleneck for the wide-spread application of these cells in the clinic.

The CRISPR–Cas9 identified in *Streptococcus pyogenes* (*Sp*) has become a dominant player in mammalian cells’ genome editing. In this system, under the direction of guide RNA (gRNA), endonuclease SpCas9 cuts both strands of the cognate DNA, forming double-strand breaks (DSBs). The Cas9–gRNA complex can be delivered to cells by several methods including plasmid or viral editing vectors that contain Cas9 enzymes and gRNA for intracellular construction. However, transfection of editing plasmids into human iPSCs, hematopoietic cells, or T cells induces significant cell death (9,10) due to an overwhelming cytosolic dsDNA-induced innate immune response (11,12). In contrast, *in vitro* combination and subsequent delivery of the Cas9–gRNA ribonucleoprotein (RNP) complex improves editing efficiency and reduces the possibility of off-target editing (13). The gRNA is composed of two parts: CRISPR RNA (crRNA), a ∼20 nucleotide single-stranded RNA complementary to the target DNA, and a trans-activating CRISPR RNA (tracrRNA), a small trans-encoded RNA to form a crRNA-tracrRNA hybrid (14). Moreover, commercial tracrRNAs and crRNAs can be chemically modified to have enhanced intracellular stability (15,16). Thus, we constructed RNPs and delivered them by electroporation to edit iPSCs and T cells. We also studied K562, an easily editable and widely used erythroleukemia cell line and U937, a pro-monocytic, human myeloid leukemia cell line to gain insights into human hematopoietic cell editing.

Following DNA DSBs, the DNA repair machinery is activated to promote DNA ligations through several DNA repair pathways. These include canonical non-homologous end-joining (c-NHEJ/NHEJ), alternative end-joining or microhomology-mediated end joining (alt-EJ/MMEJ), and homology-directed repair (HDR) when a donor template flanked with homologous arms (HAs) is present (17). These processes may disrupt the gene's open reading frame, generating a knockout (KO) allele (18). In contrast, precise gene knockin (KI) is a templated editing process guided by HDR donors. The main HDR donor types are plasmid donors and single-stranded oligodeoxyribonucleotides (ssODNs). However, plasmids often elicit strong immune responses and severe cytotoxicity (19), and ssODNs are less feasible for large sequence length HDR insertions (20,21). Long single-stranded DNA templates have been used for generating transgenic mice (22). However, this type of HDR donor may carry more mutations (23). AAV vectors have become the preferred choice in clinics because of their low immunogenicity. AAV6 has achieved impressive results in the genome editing of iPSCs, T cells and hematopoietic cells (24–26). Therefore, we used RNP nucleofection and an AAV6 HDR donor for precise gene KI, which reportedly achieves high editing efficiency (24,27,28).

CRISPR–Cas9 mediated DSB has been reported to be blunt with error-prone DNA repair systems generating random and unpredictable mutations (29). However, multiple reports have shown that SpCas9 can also cause staggered breaks, leading to nonrandom DNA repair and predictable editing outcomes (30–32). The acquisition of large quantities of editing outcome data has led to the development of machine learning algorithms to predict the editing results of certain gRNAs (33), such as inDelphi (34) and FORECasT (35). Another machine learning model, SPROUT, was trained on human CD4^+^ T cell RNP editing data, but it does not predict precise editing patterns (36). As such, we compared our data with the predictions of inDelphi and FORECasT.

After a comprehensive investigation of over 80 targets in four cell types, we find that editing efficiencies and patterns vary from one gRNA to another, in a gRNA and cell type-specific manner. Quantification of the time to reach half of the maximum editing frequency, T_50_, by dynamics studies shows that NHEJ-mediated small insertions and deletions (indels), especially +A/T, occur faster than MMEJ-mediated long deletions and HDR editings. As such, HDR outcompetes MMEJ-mediated long deletions but not NHEJ-mediated small indels. After screening 14 small molecules, we have identified M3814 (an effective inhibitor of DNA-dependent protein kinase catalytic subunit, DNA-PKcs, involved in the phosphorylation of NHEJ-associated proteins) as the most significant HDR enhancer in both iPSCs and T cells.

## MATERIALS AND METHODS

### gRNA design

Appropriate crRNAs or sgRNAs targeting human *AAVS1*, *BCL11A*, *CD326*, *GATA4*, *HBG1*, *MYH6*, *EEF1A1* and *EEF2* were designed using CHOPCHOP (http://chopchop.cbu.uib.no) (37,38). Sequences of all crRNAs used in this study are listed in Supplementary Figure S1. Chemically modified synthetic crRNAs were purchased from Sythego or Integrated DNA Technologies (IDT), which showed identical efficacy. Plasmid sgRNA vectors were constructed as described previously (39–41).

### AAV HDR donor construction

The AAV HDR vector consisted of a backbone with AAV2 ITR (Inverted Terminal Repeat), and a short insert of 8, 15 bp (for analysis by sequencing), or a fluorescent protein (for detection of HDR efficiency by FACS) flanked by 600 bp homologous arms. The inserted fragments are located at the cutting site of Cas9-gRNA; thus, the donors and HDR-edited targets will not be recognized by the RNP. All the vector components were amplified from human gDNA or plasmids in our lab by PCR using KAPA HiFi polymerase (KAPA Biosystems) and purified using the GeneJET Gel Extraction Kit (Thermo Fisher Scientific). The PCR products were then assembled using NEBuilder HiFi DNA Assembly kit, following the manufacturer's instructions. Multiple colonies were chosen for Sanger sequencing (MCLAB) to identify the correct clones. All the sequences of AAV6 HDR donors used in this study are listed in Supplementary File 1.

### AAV6 packaging

We used a triple plasmid transfection protocol to produce recombinant AAV vectors as detailed previously (42,43). In brief, HEK293T cells at the 80–90% confluency were transfected with the complex of PEI (polyethylenimine) MAX 40K (Polysciences) and AAV plasmids at a mass ratio of 2:1. pAAV-Helper (Cell Biolabs), pR2C6 (AAV6 capsid vector) (Cell Biolabs), pAAV-HDR (transgene vector construct) were added at a ratio of 2:1:1. For one 15-cm culture dish, 40 μg plasmid DNA was used. Five days after transfection, the supernatant was harvested two hours after treating with 500 mM NaCl (Sigma) and 20 U/ml Benzonase (SCBT) (43). The virus-containing supernatant was clarified at 5000 × g for 10 min, sterilized with a 0.22 μm filter, followed by a 20-fold concentration using a Minimate (PALL) tangential flow filtration system with a 300 K molecular weight cut-off (MWCO) capsule. The AAV6 vectors were further purified by iodixanol gradient centrifugation. To deplete iodixanol in the final AAV products, we washed the vectors twice with PBS–0.01% Pluronic F68 using the Vivaspin 20 centrifugal concentrators (MWCO 100 kDa). The AAV6 vector titers were determined by qPCR analysis using the vector plasmids as controls (42). Individual vector-specific primers (also for amplicon sequencing) were used. We also used primers that amplify the plasmid backbone to monitor plasmid contamination, which showed <5% plasmid contamination. To verify the titers further, viral particles were mixed with human cells, followed by DNA extraction for qPCR analysis. β-Actin was used as an internal reference to calculate AAV titers (44,45).

### Human iPSC culture

iPSC lines were generated from anonymous adult donors by peripheral blood (PB) reprogramming using episomal vectors that express OCT4, SOX2, MYC, KLF4, and BCL-XL (39,46). TJ-iPSC-B1, TJ-iPSC-B2, TJ-iPSC-B3 lines were used in this study. The iPSCs used in this study have been published previously (9). iPSCs were grown under feeder-free conditions and maintained in tissue-culture treated six-well plates (BD) coated with 1% Matrigel (Corning) in fresh mTeSR™ E8 medium (Stemcell Technologies). iPSCs were cultured in a humidified atmosphere with 5% CO_2_ at 37°C, and the medium was changed daily with fresh mTeSR™ E8 medium.

### K562 cell and U937 cell culture

K562 (ATCC) and U937 (ATCC) cells were cultured in RPMI-1640 medium (VWR) with 10% fetal bovine serum (Gibco) and 1% penicillin/streptomycin (Invitrogen). Cells were maintained in a 37°C, 5% CO_2_, fully humidified incubator, and passaged twice weekly.

### Human primary T cell culture

All peripheral blood (PB) samples were harvested and handled according to institutional guidelines and in compliance with all relevant ethical standards. PB mononuclear cells (Tianjin, China) were isolated by density gradient centrifugation, and T lymphocytes were purified using an Easysep Human CD3^+^ selection kit II (Stemcell Technologies) following the manufacturer's protocol. Immediately after isolation, CD3^+^ cells were expanded in the ImmunoCult™-XF T Cell Expansion Medium (Stemcell Technologies) supplemented with 10 ng/ml human recombinant IL-2 (Stemcell Technologies), and stimulated with anti-human CD3/CD28 magnetic Dynabeads (Thermo Fisher). Cells were grown at 37°C in a humidified incubator with 5% CO_2_. The medium was changed every two days, and the cell density was maintained below 1 × 10^6^ cells per ml.

### RNPs

TracrRNAs and crRNAs were acquired from Synthego or IDT and annealed following the recommended protocol. In brief, the tracrRNA and crRNA powder was dissolved with TE buffer at a final concentration of 200 μM. To prepare for 30 μM annealed gRNA, crRNA and tracrRNA were mixed in a 2:1 ratio and diluted in an annealing buffer provided by Synthego, followed by treating at 78°C for 10 min, 37°C for 30 min, and room temperature (23°C) for 15 min. Alt-R^®^ SpCas9 Nuclease V3 protein, which contains a nuclear localization sequence, was purchased from IDT. Cas9 RNPs were prepared immediately before electroporation by incubating 60 pmol Cas9 with 120 pmol gRNA (2:1 gRNA to Cas9 molar ratio) at room temperature for 10 min.

### RNP electroporation and AAV donor transduction

The electroporations were performed using a Lonza 2b nucleofector according to the manufacturer's instructions with modifications. 70 μl buffer per reaction was used. For K562 cells, the Amaxa™ Cell Line Nucleofector™ Kit V (Lonza) and program T-016 were used. For U937 cells, the Amaxa™ Cell Line Nucleofector™ Kit V (Lonza) and program W-001 were used. One to two million cells were used for each electroporation. For iPSCs, single-cell suspension of iPSCs was electroporated using Human Stem Cell Nucleofector™ Kit 2 with program B-016. We also added 0.5 μg BCL-XL plasmid to improve iPSC survival (9). After electroporation, the cuvette was incubated at 37°C for ∼5 min. During the first day after transfection, 10 μM ROCK inhibitor Y27632 was added to the iPSC culture. For T cells, 3 d after initiating T-cell activation, the CD3/CD28 beads were magnetically removed, and 1 × 10^6^ T cells were electroporated using the Lonza Nucleofector 2b (program B-016) and the Human T Cell Nucleofector Kit (VPA-1002, Lonza). Following electroporation, cells were cultured in fresh medium with Dynabeads^®^ Human T-Activator CD3/CD28 (Gibco) and incubated at 37 °C and 5% CO_2_. For HDR editing, the AAV6 donor vector was added at an MOI of 3000 to 10 000 to the culture immediately after electroporation. We removed the AAV6-containing medium and refreshed the culture 24 hours after electroporation.

### Target amplification and Illumina amplicon deep sequencing

Approximately 2 × 10^5^ cells were harvested for genomic DNA extraction using 10–20 μl digestion buffer, which consisted of 100 mM NaCl, 10 mM Tris pH 8, 5 mM EDTA, 0.5% Tween 20 (Sigma), and 1% proteinase K (ABM; 10 mg/ml). To lyse cells and extract gDNA, the mixtures were treated at 56°C for 60 min, followed by 95°C for 10 min. After a short spindown, 1 μl of the supernatant was used for PCR amplification. To prevent artifacts induced by AAV6 HDR templates, the primary PCR was conducted using primers targeting genomic sequences flanking the donor's homology arms. Primers for amplifying target sequences are listed in Supplementary Table S1. We used the KAPA HiFi DNA polymerase (Roche Sequencing) for PCR. The thermal cycler program for primary PCR was as follows: 98°C for 2 min, followed by 98°C for 5 s, 64°C for 5 s, 68°C for 5 s and 72°C for 30 s, for 30 cycles. PCR amplifications were verified by electrophoresis on 1% agarose gels. The primary PCR products were diluted 100 times with nanopure water. Real-time qPCR was performed for the secondary PCR using KAPA SYBR^®^ Fast Universal 2 × qPCR Master Mix (Kapa Biosystems). To exclude HDR artifacts, we abandoned products with high threshold cycle (Ct) values (∼15); thus, there were no detectable HDR artifacts. An additional round of PCR did not introduce bias in frequencies of indels or HDR insertion of a short fragment. Barcode-containing primers were used for the secondary PCR (Supplementary Table S1). The thermal cycler program for 2^nd^ PCR was as follows: 98°C for 2 min, followed by 20 cycles of 98°C for 5 s, 64°C for 5 s, 68°C for 5 s and 72°C for 15 s. Amplicons were verified by electrophoresis on 1% agarose gels. 100 ng of PCR products from each sample were pooled for sequencing using Illumina's NovaSeq6000 System (Novogene). Novogene constructed the library and acquired raw data.

### Analysis of editing efficiency and patterns

The 150-bp paired-end high-throughput sequencing reads were merged into a single read with FLASH (47), followed by demultiplexing using the Barcode Splitter Python script (https://pypi.org/project/barcode-splitter/). We then used the web-based tool Cas-Analyzer (48) to determine the editing efficiency and events by uploading the .fastq file for each amplicon. Next, we loaded the generated result text files to Microsoft Excel. We then used an in-house VB (Visual Basic) script to exclude sequencing errors and misalignment. The VB script is available upon request. For clarity, we only presented the top 10 events of DNA repair patterns in the manuscript. To analyze HDR efficiency, we also provided HDR donor sequences. We analyzed some data using CRISPResso2 (49), which showed almost identical results to Cas-Analyzer. These tools return many results, including the number of reads and factions of editing alleles (NHEJ and HDR). However, these tools do not separate MMEJ from NHEJ. To sort out NHEJ and MMEJ, we manually curated all the top 10 editing patterns. All the repair outcomes that are not MMEJ or HDR were considered NHEJ. MMEJ was designated when 2–16 nt microhomologies were observed surrounding the break site, in agreement with the Rational InDel Meta-Analysis (RIMA) (50), which defines indels as MMEJ only if at least two nt microhomology exists. Here we also considered deleting G in G|G or C in C|C (where | indicates SpCas9 cut site) MMEJ, since these occurrences were substantially (>10-fold) higher than deleting G or C in G|C or C|G. In our dataset, deleting T in T|T or A in A|A was rare, thus not included for analysis.

### inDelphi and FORECasT predictions

Sequences of 30 nt flanking Cas9-gRNA cutting sites were input to the inDelphi website (https://indelphi.giffordlab.mit.edu) (34). To predict indel patterns, we chose two different cell types K562 and mESCs. Similarly, for FORECasT predictions, sequences of suitable length (∼50 nt) were input at the FORECasT website (https://partslab.sanger.ac.uk/FORECasT) (35), which does not require information on the cell identity.

### Determination of intracellular AAV6 HDR donor copy numbers

We harvested iPSCs with Accutase at 4, 8, 12, 24 and 48 h after RNP-AAV6 editing and extracted gDNA using the proteinase K lysis method detailed above. qPCR analysis was carried out on a 7500 Fast Real-Time qPCR System (Applied Biosystems) in 96-well plates. ACTB (ACTB-F: TCGTGCGTGACATTAAGGAG, ACTB-R: GGCAGCTCGTAGCTCTTCTC) was used as a reference gene to normalize vector copy numbers. We considered two copies of ACTB per cell. Each 20 μl reaction mixture contained 10 μM of primer pairs and 1–2 μl of DNA templates, 10 μl of KAPA SYBR^®^ FAST Universal 2× qPCR Master Mix (Kapa Biosystems), and nanopure water. The primers for qPCR analysis are listed in Supplementary Table S1. The reaction procedure was as follows: 95°C for 10 min, followed by 40 cycles of 95°C for 15 s for denaturation and 60°C for 30 s for annealing/extension/data acquisition. The melting curve was determined after PCR cycling to reassure the specificity of PCR amplification.

### Small molecules

Commercially available small molecules used in this study were SCR7 (Tocris; 5342), Azidothymidine (AZT; Tocris), B02 (Sigma; SML0364), 6-Hydroxy-DL-DOPA (DOPA; Tocris), Cyclosporin H (CsH) (Sigma; SML 1575), Mirin (Cayman; 13208), Olaparib (LC Labs; O-9201), AZD7762 (Cayman; 11491), VE822 (Cayman; 24198), RS1 (Calbiochem; 553510), NU7026 (Cayman; 13308), NU7441 (Tocris; 3712), Trichostatin A (TSA) (Cayman; 89730), Pevonedistat (MLN4924) (Selleckchem; S7109), and M3814 (MedKoo; 206478). As a control, this study included the CRISPY Mix (51), which is the combination of 20 μM NU7026 (NHEJ inhibitor), 0.01 μM TSA (HDAC inhibitor) and 0.5 μM MLN4924 (improves CtIP) (NSC 15520 was not available). Stock solutions were prepared in dimethylsulfoxide (DMSO) (Sigma) and diluted to working concentrations before use. The medium was changed 24 h after the addition of small molecules. We listed the detailed information and working concentrations of small molecules in Supplementary Table S2.

### Flow cytometry

Flow cytometry was performed to determine HDR efficiency, as described previously (9). Cells were acquired on a BD FACSAria III flow cytometer three days post-nucleofection. For HDR-mediated knockin of mNeonGreen reporter into the target gene (*EEF1A1* or *EEF2*), the fluorescence-positive cell population was considered the HDR edited cells. The FITC channel was used to determine the proportion of mNeonGreen^+^ cells. Electroporation without relevant sgRNA was carried out as a negative control, which showed low-levels of mNeonGreen^+^ cells. The FACS data were analyzed using FlowJo.

### Statistics and reproducibility

The *P* values for different groups were calculated and analyzed using GraphPad Prism 8. Column plots show means with s.d. error bars. Each figure legend denotes the statistic used. All central tendencies and error bar indications are denoted in the figure legends. Levels of statistical significance: *P < 0.05; **P < 0.01; ***P < 0.001; ****P < 0.0001; ns., not significant. Scatterplots of correlation and linear regression analysis were calculated using Pearson's correlation coefficient. Scatterplots of editing efficiency data between two independent experimental replicates for iPSCs showed reproducibility of editing outcomes. Scatterplots of editing efficiency data between two independent Illumina sequencing pools from one editing sample showed reproducibility of Illumina sequencing and data analysis (Supplementary Figure S2A). We used unpaired or paired two-tailed Student's *t*-test for normally distributed data with similar variance for comparing two groups. In all significance tests performed in the study, the data satisfied the normality criteria for *t*-tests.

## RESULTS

### RNP-mediated genome editing at different loci of four cell types

**Figure 1.**
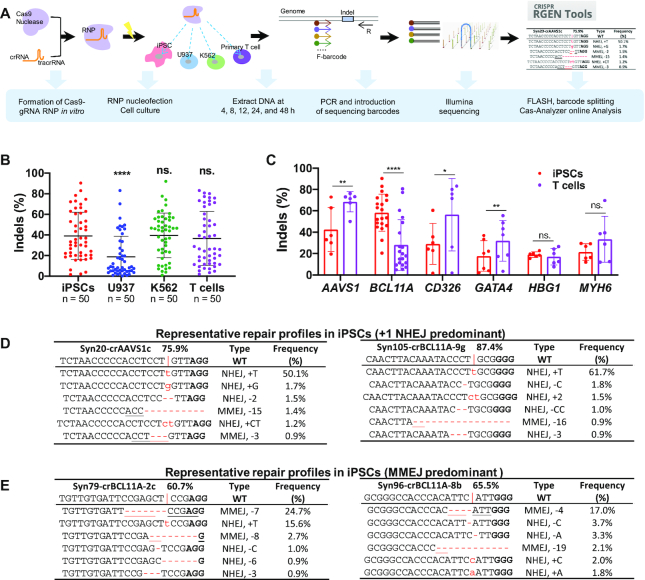
Distinct RNP-mediated editing efficiencies at different loci in iPSCs, U937, K562, and T cells. (**A**) Schematic of genome editing with a CRISPR–Cas9 RNP strategy. (**B**) Summary of indel frequencies of 50 gRNAs targeting six gene loci in 4 cell types. Paired two-sided Student's *t*-tests were conducted. (**C**) Comparison of editing efficiencies in iPSCs and T cells of gRNAs that target 80 different sites at the *AAVS1*, *BCL11A*, *CD326*, *GATA4*, *HBG1* and *MYH6* loci. *P* values were determined using two-tailed paired Student's *t*-tests. (**D**, **E**) Representative editing patterns with a predominant +T NHEJ (**D**) or an MMEJ-mediated deletion (**E**) in iPSCs. The targeting site and wild-type sequence are displayed at the top of each table. The vertical line indicates the Cas9 cleavage site; lower-case red-letter denotes inserted base; micro-homologies are underlined. PAM (NGG) is marked in bold. For **B** and **C**, data are shown as mean ± s.d. ****P < 0.0001; ***P < 0.001; **P < 0.01; *P < 0.05; n.s., *P* ≥ 0.05.

To compare genome editing efficiencies among different cells and gene sites, we designed 80 crRNAs targeting six human genes (*AAVS1*, *BCL11A*, *CD326*, *GATA4*, *MYH6* and *HBG1*) (Supplementary Figure S1). We selected these sites based on their potential clinical interest and differential expression levels in the different cell types (52,53). crRNAs were first annealed with tracrRNA, followed by incubation with SpCas9 protein to form an RNP complex. Compared to *in vitro* transcribed single guide RNAs (sgRNAs), these gRNAs were chemically modified as previously described, to enhance their intracellular stability and minimize immune response (15). Two days after nucleofection of RNP into 4 cell types (iPSCs, U937, K562 and T cells), target sequences were PCR amplified using barcoded primers and pooled for 150-bp paired-end Illumina sequencing. We merged the overlapped reads and demultiplexed the data for analysis, followed by determining the editing patterns and efficiencies using Cas-Analyzer (Figure 1A).

First, we determined the editing efficiencies 4–72 h after electroporation and observed a significant increase from 4 to 24 h (Supplementary Figure S2B). However, after 48 h there was no significant increase in editing efficiency. As such, we harvested cells two days after transfection for analysis in further studies (Supplementary Figure S2C, D). To assess the reproducibility of editing in iPSCs, we compared 57 crRNAs in independent experiments. Pearson correlation analysis showed a coefficient of 0.9 (Supplementary Figure S2E).

We observed widely variable editing efficiencies for each gRNA. To examine whether the nature of individual gRNAs contributed to this difference, we assessed the editing efficiencies of gRNAs targeting *AAVS1*, which is transcriptionally active in all cell types, and *GATA4*, which is transcriptionally silent in the four cell types (Supplementary Figure S3A, B). In each group, we designed 6–8 gRNAs to target a small window (15–20 bp) of the genome sequence (Supplementary Figure S1). High-performing gRNAs targeting *AAVS1* tended to achieve higher cutting efficiencies irrespective of the cell type, and low-performing gRNAs appeared to generate lower efficiencies in all of the cell types (R^2^ range from 0.5 to 0.8) (Supplementary Figure S3C and E). We observed the same effects when editing *GATA4* (Supplementary Figure S3D and F). These data suggest that the relative efficiencies of individual gRNAs was conserved across cell types and not dependent on transcriptional activity of the target. Overall, paired analysis using aggregated data showed that ∼40% of alleles could be edited in iPSCs, K562 and T cells, whereas less than 20% of U937 cells were edited with RNP (Figure 1B). Next, we focused our analysis on iPSCs and T cells. Among the six loci, four showed differential editing efficiencies between the two cell types. Higher efficiencies were observed at *AAVS1*, *CD326* and *GATA4* in T cells compared to iPSCs, whereas *BCL11A* was more editable in iPSCs than in T cells (Figure 1C).

We then analyzed editing patterns after RNP mediated cleavage. Consistent with previous reports (31,32,54), we also observed the strong bias of duplications of the –4 nucleotide before protospacer adjacent motif (PAM) when the –4 position is adenine (A) or thymine (T) (Figure 1D; Supplementary File 2). In addition, a strong microhomology surrounding the predicted cleavage site led to predominant deletions mediated by the MMEJ pathway (Figure 1E; Supplementary File 2). Here we assigned the following editing events as MMEJ—deleting G in G|G, deleting C in C|C (where | indicates cut site), or removal of over 2 nucleotides (usually < 50 nt in our dataset) that are identical in the proximity of cleavage sites (50,55,56).

### Distinct patterns of NHEJ-mediated insertions and MMEJ-mediated deletions in different cell types

**Figure 2.**
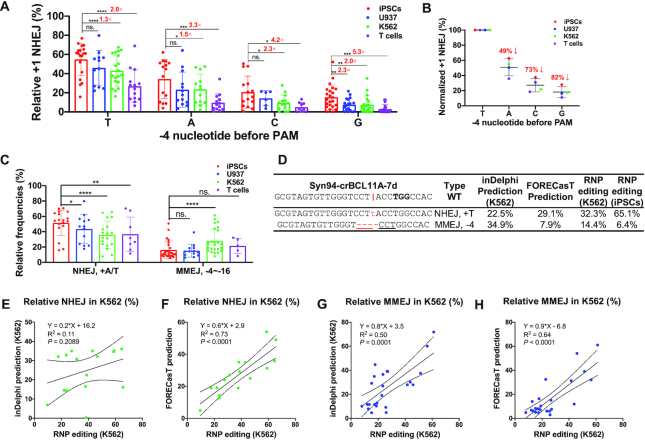
Human iPSCs, U937, K562, and T cells display distinct NHEJ-mediated +1 insertions and MMEJ-mediated deletions. (**A**) Relative frequency of +1 NHEJ in iPSCs, U937, K562 and human primary T cells. +1 insertion data from 80 RNP editing results are divided into four groups according to the –4 nucleotide before PAM (*n* = 6 to 23 independent experiments for each pattern). Paired two-sided Student's *t*-tests were conducted. (**B**) The –4 nucleotide determines the +1 NHEJ frequencies. The raw data in (**A**) were averaged to assess the impact of –4 nucleotide on insertion frequencies. Relative +1 frequencies were normalized to +T insertion. (**C**) Relative frequencies of +A/T NHEJ and –4 to –16 bp MMEJ in four cell types. Data from 25 gRNA editings were summarized. Paired two-sided Student's *t*-tests were conducted. (**D**) Representative patterns of +T NHEJ and –4 MMEJ of inDelphi prediction (K562), FORECasT prediction, and our RNP editings (K562 and iPSCs). The vertical line indicates the Cas9 cleavage site; lower-case red-letter denotes inserted base; micro-homologies are underlined. PAM (NGG) is marked in bold. (**E**–**H**) Comparison of K562 RNP editing data with predictions by inDelphi (**E**, **G**) and FORECasT (**F**, **H**). The predominant +A/T NHEJ (*n* = 16) and –3 to –21 bp MMEJ (*n* = 24) editing outcomes were summarized from RNP editings at 34 different sites. The cell line was set as K562 for inDelphi prediction. Pearson linear regression analyses were conducted in panels **E**–**H**.

We further analyzed the pattern of indel editing from the 80 gRNAs by tabulating editing events with a frequency greater than 1% (absolute value) into seven groups, including NHEJ insertions of +A/T, +C/G, MMEJ deletions ranging from –1 to –30 bp. The most predominant editing patterns were A or T insertions, and other events were relatively lower (Supplementary Figure S4). As such, we focused on the analysis of +1 additions, which refer to duplications of the –4 nucleotide before NGG PAM. After dividing the data into four groups, we found that the +T event was the most predominant pattern in all four cell types, while the paired test showed a higher tendency of +T in iPSCs than other cell types (Figure 2A, B). We also found that iPSCs showed significantly higher frequency in +C/G when the –4 position is C or G, respectively (Figure 2A, B). Paired analysis using aggregated data of 5 (for T cells) or 25 (for K562, U937 and iPSCs) gRNAs showed that the NHEJ insertion repair occurred at ∼40% higher frequency in iPSCs than in K562, U937 and T cells (*P* = 0.01, *P* = 1.0 × 10^−5^ and *P* = 0.006. Figure 2C). Consistent with a previous report on T cell editing (36), the presence of a G or C at –4 position led to low-level insertions of 3–5%, whereas an A or T increased the proportion to 10% and 27% (Figure 2A). Considering the observed differences might be artifacts due to different transfection efficiencies and thereby editing outcomes, we explored and ruled out the possibility by intentionally decreasing the RNP amount to mimic the effects of differential nucleofection efficiencies. As expected, a lower amount of RNP decreased the total editing efficiencies. However, the relative proportion of the top 3 DNA repair patterns showed no significant changes in iPSCs and T cells (Supplementary Figure S5).

Our results and previous reports have shown that a given Cas9–gRNA induces similar indel patterns in different cell types (50,57), but with subtle differences (Supplementary Figure S4; Supplementary File 2). Machine learning models and *in silico* scoring system (33,35,36,58) have been used to predict the frequencies of each repair event, particularly on well-studied cell lines such as K562 (35). inDelphi and FORECasT are web-based tools that were developed using data from pooled lentiviral transduction experiments. We selected 34 gRNAs that performed well in K562, iPSCs and T cells and predicted their editing results using inDelphi and FORECasT (Figure 2D and Supplementary Figure S6). Pearson linear regression analysis showed that FORECasT performed better than inDelphi in predicting our K562 RNP editing outcomes of both NHEJ (*R*^2^ = 0.73 and 0.11 for FORECasT and inDelphi, respectively) and MMEJ (*R*^2^ = 0.64 versus 0.50) (Figure 2E–H). Compared to inDelphi and FORECasT predictions, our RNP editing showed 25% and 28% higher +A/T NHEJ, respectively (Supplementary Figure S6E). The discrepancy may be attributed to differences in CRISPR delivery, as the two models were trained using editing outcomes of lentiviral editing systems (34). Persistent lentiviral vector-mediated Cas9–sgRNA expression might lead to secondary cutting on +1 edits containing single-base DNA bulges (59), reducing the insertion events. We also explored whether these prediction tools could be applied to editing profiles of iPSCs and T cells following RNP delivery. Similar to K562 cells, FORECasT predicted iPSC NHEJ and MMEJ editings more accurately than inDelphi (Supplementary Figure S7A–F); however, both inDelphi and FORECasT failed to predict T cell RNP editing outcomes (*P* > 0.30 for both NHEJ and MMEJ, except FORECasT’s NHEJ predictions) (Supplementary Figure S7G–J).

We compared editing patterns in K562 cells versus iPSCs by analyzing 16 NHEJ and 24 MMEJ paired editing outcomes. We found a 1.6-fold increase in +A/T NHEJ events and a 43% decrease in predominant MMEJ occurrences (Supplementary Figure S6E). These data further consolidate the conclusion that iPSCs have a higher tendency to be repaired by +1 insertion after Cas9–gRNA mediated dsDNA cleavage than other cell types we examined.

### Dynamics of DNA repair after RNP-mediated dsDNA cleavage

**Figure 3.**
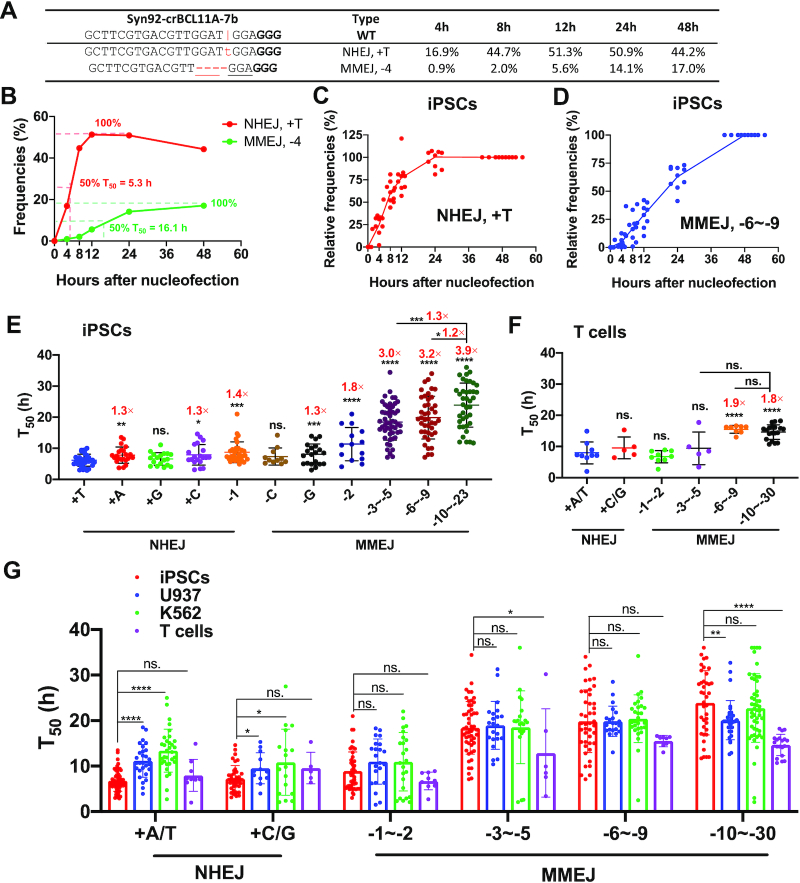
Dynamics and speed of NHEJ or MMEJ editings after Cas9–gRNA RNP-mediated dsDNA cleavage. (**A**) Dynamic changes of +T NHEJ and –4 MMEJ 4–48 h after RNP delivery, as exemplified by a gRNA targeting *BCL11A* in iPSCs. The vertical line indicates the Cas9 cleavage site; lower-case red-letter denotes inserted base; micro-homologies are underlined. PAM (NGG) is marked in bold. (**B**) Methods for calculating the T_50_ values of +1 NHEJ and –4 MMEJ editings. (**C**, **D**) Summary of dynamic changes of NHEJ-mediated +T insertions (**C**) and MMEJ-mediated deletions (–6 to –9 bp) (**D**). Data from 10 gRNA editing patterns in iPSCs were summarized. We normalized editing frequencies relative to the values at 48 h. (**E**, **F**) T_50_ of various editing patterns in iPSCs (**E**) and human primary T cells (**F**) using 80 gRNAs that target different sites at the *AAVS1*, *BCL11A*, *CD326*, *GATA4*, *HBG1* and *MYH6* loci. MMEJ-mediated deletions were categorized into different groups: –3 to –5 bp deletions (iPSCs, *n* = 51; T cells, *n* = 6), –6 to –9 bp deletions (*n* = 43 and 8) and over –10 bp deletions (*n* = 39 and 19). T_50_ of a particular allele was calculated and summarized only when this pattern's events accounted for more than 2% of the total indels. (**G**) Comparison of T_50_ of major NHEJ and MMEJ in iPSCs, U937, K562 and T cells. *n* = 10–50 independent experiments performed at different times. Unpaired two-sided Student's *t*-tests were conducted for **E**–**G**. Data are presented as mean ± s.d. in **E**–**G**. ****P < 0.0001, ***P < 0.001, **P < 0.01, *P < 0.05, n.s., *P* ≥ 0.05.

Previous studies (31) and our RNP editing data have demonstrated overrepresentation of NHEJ-mediated +1 (especially +A/T) editing events in multiple cell types (Figure 2A, B; Supplementary Figure S4 and Supplementary File 2). We hypothesized that this effect is associated with the kinetics of DNA repair by different pathways. In the absence of an HDR template, the indels resulted mainly from NHEJ and MMEJ repairs. To investigate the rates of each DNA repair pattern, we analyzed the editing patterns for *BCL11A* at 4, 8, 12, 24, and 48 h after RNP transfection (Figure 1A). To quantitate the editing and DNA repair speed, we calculated T_50_, which defines as the time for reaching 50% of the highest editing efficiency by regression analysis (Figure 3A, B).

To minimize calculation errors, we only analyzed editing events with maximum absolute values over 1%. In one typical example of iPSC editing, we observed a T_50_ of 5 h for the NHEJ +1 bp event versus 16 h for the –4 MMEJ pattern (Figure 3B). After tabulating all the two major editing events (+T NHEJ versus –6 to –9 MMEJ) using relative value (absolute editings divided by total indels), we observed faster repair by NHEJ and slower repair by MMEJ in iPSCs (Figure 3C, D). U937, K562 and primary T cells exhibited the same pattern (Supplementary Figure S8A–F). These data suggest that NHEJ is the preferential pathway for repairing DSBs in all the four cell types we investigated.

To illustrate each pattern's dynamic changes, we divided all the data into 11 categories based on nucleotide insertion for NHEJ (+A, +T, +G, +C) and various sizes of deletions for MMEJ (–1 to –30). In iPSCs, the fastest repair occurred for +T, followed by other +1 insertions. A random deletion of 1 bp at the cleavage site was slightly slower than +1 insertions. Single nucleotide deletions using C or G microhomology were faster than MMEJ mediated deletions of over six nucleotides, which are 2- to 3-times slower (Figure 3E). In iPSCs, average T_50_ of +T, +A, +C, +G NHEJ were 6.2, 7.8, 7.9, 6.6 h, respectively, while T_50_ of MMEJ-mediated repairs of –3 to –5, –6 to –9 and –10 to –23 were 18.4, 19.9 and 23.9 h, respectively. We observed similar patterns in primary T cells and the other two transformed cell lines (U937 and K562), but the differences were less pronounced among increasing lengths of MMEJ-mediated deletions (Figure 3F; Supplementary Figure S8G–H). These data suggest that longer deletions repair more slowly with MMEJ.

We then compared the T_50_ of each pattern in the four cell types. The salient feature was that iPSCs showed the lowest T_50_ (fastest repair) for NHEJ-mediated +A/T among the cell types tested, especially compared with K562 (2.0-fold, *P* = 7.0 × 10^−14^) and U937 (1.6-fold, *P* = 1.7 × 10^−9^) (Figure 3G). These results suggest that iPSCs have a potent NHEJ pathway. In comparison, the MMEJ editing speed was similar in iPSCs, K562 and U937 cells. Of interest, we observed identical NHEJ editing speed in iPSCs and T cells, while slightly faster MMEJ repair in T cells than in iPSCs (Figure 3G). This result is likely due to the rapid proliferative rate of activated primary T cells.

Taken together, the dsDNA break repair data demonstrate that NHEJ is the predominant repair pathway in all of the cell types we tested. If the DSBs were not bridged promptly, the cells might eventually use the MMEJ pathway (which averages 2–3 times longer) to link the broken dsDNA.

### HDR efficiency is in proportion to indel efficiency but negatively affected by NHEJ frequency

We next explored HDR editing in the presence of an AAV donor template. We used AAV6 because this serotype productively transduces human iPSCs (24), T cells (24), and hematopoietic cells (26). To facilitate the analysis of HDR and indels, we designed AAV6 vectors with ∼600 bp homologous arms and an insertion of a short fragment (8 or 15 bp) at the RNP cleavage site (Supplementary File 1). First, we examined transduction efficiencies by assessing intracellular copies of AAV 24 h after vector addition. qPCR analysis showed that approximately 12%, 10%, 3% and 1% AAV particles entered iPSCs, T cells, U937 and K562, respectively (Supplementary Figure S9A–D). Two days after the AAV6 donor addition, we observed ∼1000 vector genome copies in iPSCs when using an MOI of 30 000 (Supplementary Figure S9E).

**Figure 4.**
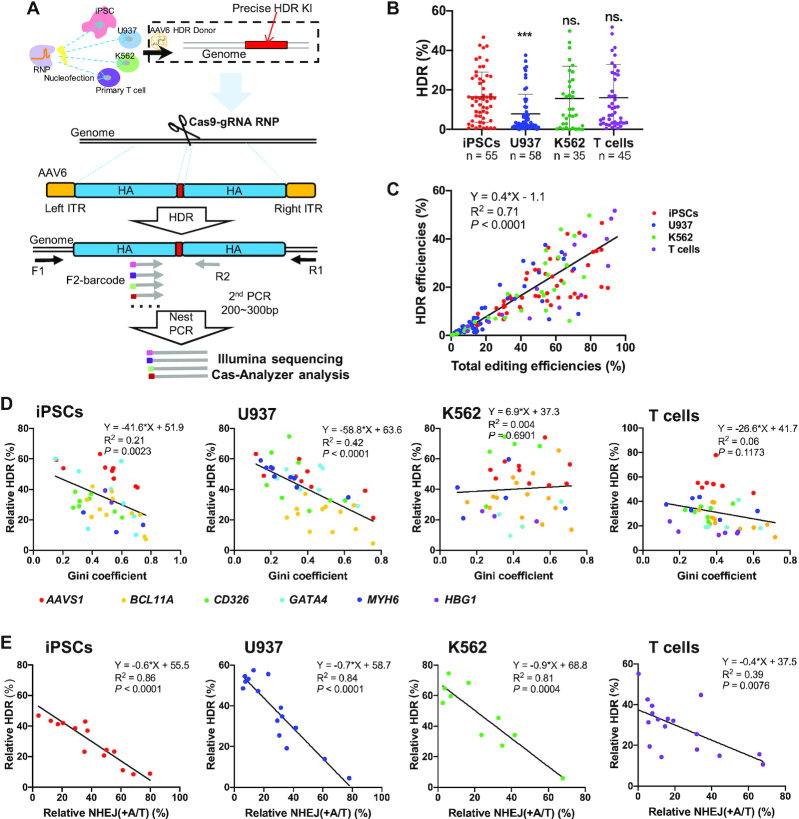
Efficiencies of HDR editing with AAV6 donors are proportional to indel efficiencies, but strong +A/T NHEJ repairs outcompete HDR. (**A**) Schematic of precise HDR editing with RNPs and AAV6 HDR donor templates. (**B**) Summary of HDR efficiencies in iPSCs, U937, K562 and T cells. We compiled editing data of 35∼58 gRNAs that target different sites at *AAVS1*, *BCL11A*, *CD326*, *GATA4*, *HBG1* and *MYH6* loci. Unpaired two-sided Student's *t*-tests were conducted. Data are presented as mean ± s.d.. ***P < 0.001; n.s., *P* ≥ 0.05. (**C**) HDR efficiencies correlate with total indel frequencies. *n* = 155. (**D**) Relative HDR efficiencies weakly correlate with Gini coefficients in iPSCs (*n* = 42) and U937 (*n* = 53), but not K562 (*n* = 36) and T cells (*n* = 44). (**E**) Relative HDR efficiencies strongly correlate with relative NHEJ (+A/T) frequencies in the four cell types. Pearson linear regression analyses were conducted for **D**, **E**.

We analyzed indels and HDR editing 48 h after the delivery of RNP and AAV. We performed nested PCR to prevent HDR editing artifacts induced by residual AAV6 in edited cells (Figure 4A). As shown in Supplementary Figure S10A-C, nested PCR effectively depleted AAV-induced artifacts. Moreover, two rounds of PCR did not bias the editing outcomes, as evidenced by identical editing efficiencies and patterns when compared to data analyzed from only one round of PCR (Supplementary Figure S10D–G). The presence of AAV donors led to a moderate but significant increase in total indels compared to editing without HDR templates (Supplementary Figure S9F). Similar to indel editing efficiencies without AAV donors (Figure 1B), we found that HDR editing efficiencies in iPSC, K562, and T cells were 2-fold higher than in U937 cells (Figure 4B). Analysis showed that HDR editing correlated with total editing (*R*^2^ = 0.71, *P* < 0.0001) (Figure 4C), suggesting that the gRNA targeting efficiency largely dictates the editing efficiencies even for HDR repairs.

Additionally, we observed that certain gRNAs exhibited a strong bias towards specific repair patterns such as +T NHEJ (Supplementary Figure S6A) or –4 bp MMEJ (Supplementary Figure S6B), which is associated with high indel efficiency but relative lower HDR frequency (data not shown). Thus, we hypothesized the presence of a predominant repair pattern would negatively affect HDR editing. To quantitate the distribution disparity, we used the Gini index metric, a measure of income inequality in the economy. Gini coefficient (or Gini index) is a measure of statistical dispersion to reflect income fairness in economics, which is somewhat similar to the concept of entropy. Here we used this metric to assess the distribution evenness of DNA repair patterns. To compute the Gini index, we used the Gini coefficient calculator (http://shlegeris.com/gini) by inputting the percentage of the top 5 indel patterns of each gRNA in the absence of an AAV donor. The high Gini index usually reflects the presence of one or two predominant repair events, such as +T NHEJ or –4 bp MMEJ. Correlation analysis showed a high Gini index correlated with low relative HDR in iPSCs and U937 cells (*R*^2^ = 0.21 and 0.42, respectively). However, regression analysis in K562 and T cells showed a low coefficient (Figure 4D; Supplementary Figure S11A).

We then analyzed whether the relative +A/T NHEJ frequencies affect the HDR ratio in the four cell types (Figure 4E; Supplementary Figure S11B). In iPSCs, U937, and K562 cells, the presence of high-level +A/T NHEJ events coincided with lower relative HDR (*R*^2^ = 0.81–0.86). In primary T cells, a weaker yet robust correlation was also observed (*R*^2^ = 0.39). Therefore, HDR editing is mostly affected by NHEJ but not by MMEJ. An intense +A/T NHEJ repair is associated with reduced HDR editing.

### HDR outcompetes MMEJ, but not NHEJ

Next, we assessed HDR repair dynamics by calculating T_50_ of individual editing events in an HDR donor's presence. Like editing without a donor, we observed fast +1 NHEJ, followed by –1 to –5 MMEJ. It took 2- to 3-times longer to generate –6 to –30 deletions by MMEJ in iPSCs (Supplementary Figure S12A). Of interest, HDR showed a slight but significantly faster repair than –6 to –30 MMEJ in iPSCs (T_50_ of ∼15 h versus ∼20 h, respectively). We also observed similar results in other cell types (Supplementary Figure S12B and C for U937 and K562 cells).

**Figure 5.**
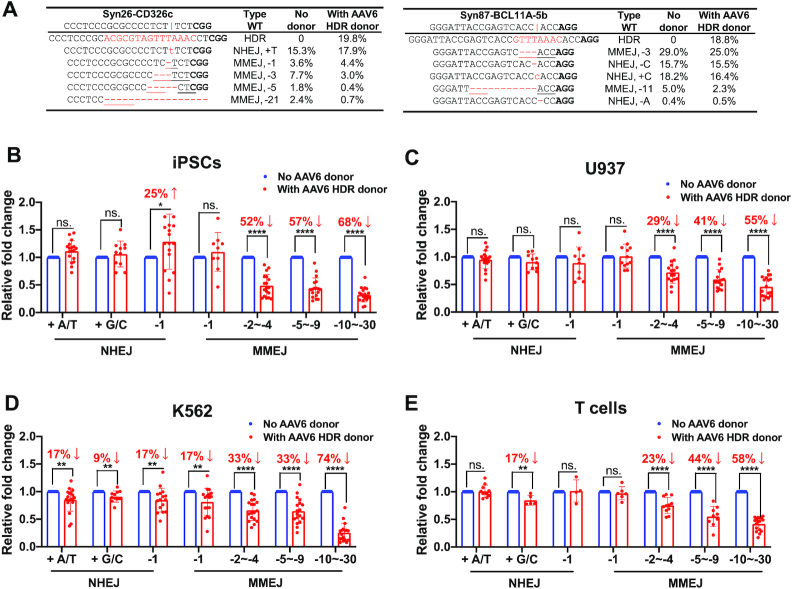
HDR outcompetes MMEJ, but not NHEJ. (**A**) Examples of top five NHEJ and MMEJ editing events 48 h after RNP delivery in the absence or presence of an AAV HDR donor. The vertical line indicates the Cas9 cleavage site; lower-case red-letter denotes inserted base; micro-homologies are underlined. PAM (NGG) is marked in bold. (**B**–**E**) The presence of AAV donors barely changes NHEJ editing events but considerably reduces MMEJ-mediated deletions of –2 to –30 bp. The same patterns were observed in iPSCs, U937 cells, K562 cells and primary T cells. Data are shown as mean ± s.d.. Data of major editing events, ranging from +1 NHEJ to –30 MMEJ, were compiled from editing outcomes of 30 gRNAs targeting different sites at *AAVS1*, *BCL11A*, *CD326*, *GATA4*, *HBG1* and *MYH6* loci. Paired two-sided Student's *t*-tests were conducted. ****P < 0.0001; **P < 0.01; *P < 0.05; n.s., *P* ≥ 0.05.

We hypothesized that the sluggish formation of MMEJ-mediated –6 to –30 bp deletion repairs compared to HDR might lead to a competitive advantage. To assess that, we examined changes in each editing event in AAV6 donors’ absence or presence. In two typical examples of editing in iPSCs at *CD326* and *BCL11A*, we observed that the presence of HDR donors did not affect events of +1 bp or –1 bp NHEJ (Figure 5A, Supplementary File 2). In two cases of –3 bp deletions, the presence of HDR templates led to a slight decrease in MMEJ with strong microhomology (ACC; 29% versus 25%), whereas a 60% decrease in MMEJ with a relatively weaker microhomology (TCT; 8% versus 3%). For longer deletions of –5 to –21 bp, we observed an up to a 3.4-fold decrease in MMEJ frequency when HDR donors were present (Figure 5A). To rule out the possibility that AAV itself induced this effect, we added an unrelated AAV6 donor in the system. There was no significant difference in MMEJ frequencies in the presence or absence of irrelevant AAV6 donors (Supplementary Figure S13).

We collected editing pattern data from 30 edited loci with >1% incidence to assess the statistical significance. Each pattern's relative frequency was computed by dividing the number of individual alleles by overall editing events. Adding HDR donors did not significantly reduce +1 or –1 bp NHEJ frequencies in iPSCs, U937 and T cells (Figure 5A–C and E). In K562, AAV’s presence induced an ∼10% reduction in +1 or –1 bp NHEJ editing (Figure 5D). However, the addition of HDR donors led to a significant decrease in MMEJ editing with deletions over 2 bp in all four cell types. Importantly, MMEJ repairs with longer deletions (over 10 bp) showed the most substantial response to HDR donors with reductions in all four cell types ranging from 55% to 74% (Figure 5B-E). We also evaluated the speed of repairs (T_50_) in the presence of AAV HDR donors and observed a similar pattern of slower MMEJ repairs with longer deletions compared to HDR repairs (Supplementary Figure S12). Therefore, we conclude that HDR repairs effectively outcompete MMEJ-mediated deletion repairs. However, small indels mediated by NHEJ outcompetes HDR editing, and thus is a predominant +A/T NHEJ event that negatively impacts HDR efficiency.

### M3814 increases HDR editing efficiency mainly by blocking NHEJ events

**Figure 6.**
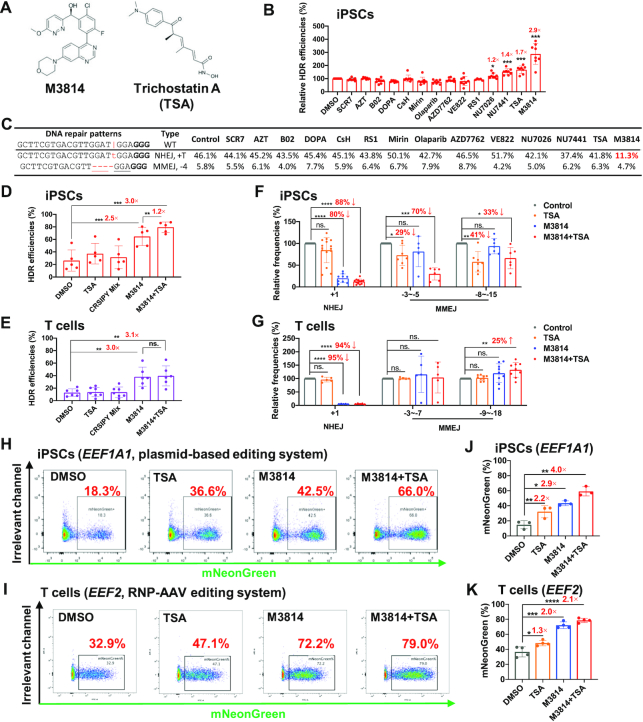
M3814 increases HDR editing efficiency largely by blocking +A/T NHEJ events. (**A**) Molecular structures of M3814 and Trichostatin A (TSA). (**B**) Differential effects of 14 small molecules on improving HDR efficiency. We used AAV6 HDR donors to guide HDR editing after electroporation of RNPs. The HDR efficiencies were normalized to the DMSO control. Data were summarized from eight independent experiments targeting the *BCL11A*, *CD326* and *GATA4* loci. Concentrations of each compound used in this study are listed in Supplementary Table S1. (**C**) Changes in +T NHEJ and –4 MMEJ editing events after treatment with 14 compounds, as exemplified by a gRNA targeting *BCL11A* in iPSCs. The vertical line, Cas9 cleavage site; lower-case red letter, inserted base; underlined letter, micro-homology. PAM (NGG) is marked in bold. We highlighted in red the considerably decreased +T NHEJ frequency after M3814 treatment. (**D**,**E**) A combinatorial effect of M3814 and TSA on enhancing HDR efficiencies in iPSCs (**D**, *n* = 5) and T cells (**E**, *n* = 6). (**F**, **G**) M3814 blocks +1 NHEJ in both iPSCs and T cells, whereas only moderately reduces MMEJ editing in iPSCs. *n* = 5–15 for each editing event in iPSCs (**F**), *n* = 5–9 for each editing event in T cells (**G**). (**H–K**) Combinatorial effects of M3814 and TSA on enhancing HDR insertion of the mNeonGreen reporter gene with a plasmid donor in iPSCs (**H**, **J**) or an AAV donor in T cells (**I**, **K**). HDR efficiencies were determined by FACS 3 days after electroporation (**H**, **I**). Data are shown as mean ± s.d.. Paired two-sided Student's *t*-tests were conducted. ****P < 0.0001; ***P < 0.001; **P < 0.01; *P < 0.05; n.s., *P* ≥ 0.05.

NHEJ is the predominant and fast-acting pathway to repair dsDNA damage and outcompetes HDR editing. As such, NHEJ inhibitors enhance HDR efficiency (60–62). However, in a clinically relevant RNP-AAV editing system, it is still unknown which inhibitor is most effective. Here we tested 14 small molecules reportedly to increase HDR editing in other systems, including SCR7 (63), Azidothymidine (AZT) (64), B02, DOPA (65), NU7026, NU7441 (66), Trichostatin A (TSA) (51), Mirin (67), Olaparib (68), RS1 (69,70), M3814 (71), VE822 (72) and AZD7762 (73,74) (Figure 6A; Supplementary Figure S15A; Supplementary Table S2).

We first investigated the effects of individual compounds on HDR efficiencies in iPSCs using RNP and AAV6 donors. Most of them performed poorly on this screen, likely due to the relatively high-level benchmark (20%) of HDR editing. Only three out of the 14 agents, NU7441 (DNA-PK inhibitor), Trichostatin A (TSA; HDAC inhibitor), and M3814 (DNA-PK inhibitor) were able to increase HDR editing efficiency by 40% or more. Most impressively, M3814 increased HDR repairs by 2.9-fold in iPSCs (Figure 6B).

We further assessed three compounds of particular interest based on their potential to enhance HDR editing (M3814, TSA and SCR7). A 2–3-fold increase or decrease in concentration for these three compounds in this and previous studies showed no significant differences in editing efficiencies (Supplementary Figure S15A–E) (60). The dosage only marginally impacted NHEJ or MMEJ editing events in iPSCs or T cells (Supplementary Figure S15F–K). To explore the mechanism of action, we scrutinized the changes in editing patterns. In a typical example, M3814 decreased +T NHEJ events from 46% to 11%, but only slightly repressed –4 MMEJ from 5.8% to 4.7% (Figure 6C). The effects of SCR7 have been controversial (75,76) and similar to our previous reports (41), we observed no appreciable effects of SCR7 on HDR editing. Interestingly, Olaparib (68), a PARP inhibitor (77) that failed to enhance HDR editing (Figure 6B), inhibited short MMEJ (–3 to –5 nt deletions with a 3-nt micro-homology by 45% (Supplementary Figure S14B left), but had no apparent effect on longer MMEJ (–9 to –12 nt deletions with a 3-nt micro-homology) (Supplementary Figure S14B right, S14C).

We then examined the combinational effects of the inhibitors that enhanced HDR editing. As a control, we included the CRISPY Mix, which previously showed a ∼3-fold increase in HDR efficiency in iPSCs (51); however, in our RNP-AAV system, CRISPY Mix promoted only a modest improvement in HDR editing. In contrast, M3814 increased HDR rates from ∼25% to ∼60%, and the inclusion of TSA further improved editing efficiencies by 20% (*P* = 0.0073, Figure 6D). Similarly, we observed that a combination of M3814 and TSA increased the average HDR repair by 3.1-fold in T cells (Figure 6E). Furthermore, M3814 exhibited variable editing improvements for gRNA that led to predominant MMEJ with more considerable improvements for targets with an intrinsic potential for +A/T NHEJ repair (Supplementary Figure S14D). Linear regression analysis showed that the relative frequency of the predominant NHEJ event is positively associated with HDR improvement by M3814 (*R*^2^ = 0.49, *P* = 0.05) (Supplementary Figure S14E). This result can be explained by potent inhibition of NHEJ and a moderate reduction of MMEJ by M3814 in iPSCs.

Compared to DMSO control, the proportion of NHEJ with M3814 or M3814+TSA showed a striking reduction in both iPSCs (80–88%) and primary T cells (94–95%), suggesting that M3814 increases HDR primarily by blocking NHEJ DNA repair events (Figure 6F, G). Of interest, TSA or M3814+TSA also decreased MMEJ repair by 30–50% in iPSCs, but not in T cells.

In the above studies, HDR editing led to only a ∼10 bp insertion. Next, we assessed the effects of small molecules on the precise knock-in efficiency of a reporter gene. We designed promoterless HDR donors that guided the insertion of mNeonGreen at *EEF1A1* or *EEF2* in iPSCs and T cells. HDR editing led to the expression of mNeonGreen, which can be quantitated by flow cytometry (Supplementary Figure S16). In negative controls, few mNeonGreen-positive cells were detectable when the HDR donors and gRNAs were intentionally switched. We observed that HDR efficiency increased from ∼30% to 80% with the addition of both M3814 and TSA in both iPSCs and T cells (Figure 6I, K; iPSC data not shown). Similarly, in the plasmid donor-based editing system, we observed similar effects of M3814 and TSA in human iPSCs (Figure 6H, J). These data suggest that M3814 and TSA may be a universal combination for enhancing HDR in a variety of cell types and targets.

## DISCUSSION

Herein we report on the editing dynamics and repair principles after RNP delivery in human iPSCs, primary T cells, and transformed cell lines K562 and U937 in the absence or presence of AAV6 HDR templates. After CRISPR-mediated dsDNA cleavage, gRNA and contextual sequences primarily determined the editing efficiencies and NHEJ or MMEJ repair patterns. Yet, we also observed distinct editing patterns in different cell types. The rapid occurrence of +1 bp NHEJ decreases HDR frequencies. However, blockade of +1 bp NHEJ with small molecules considerably increases HDR efficiencies. The DNA repair profiling pattern presented here offers testing strategies for selecting gRNAs for specific editing purposes. We recommend using gRNAs with a strong bias for NHEJ (+A/T) editing in gene knockout applications. The exploitation of the predominant +1 NHEJ or MMEJ editing outcomes has led to donor-free gene therapy (78). Multiple machine learning models for CRISPR editing outcome prediction can also aid in picking the preferable gRNAs.

A large number of comprehensive studies on editing profiles are based on lentiviral library screens (34,35). However, data on the editing dynamics and patterns of therapeutically relevant iPSCs and T cells using RNP are still lacking. The previous database and prediction models are valuable resources. However, our data show that inDelphi could not precisely predict the editing patterns of K562, iPSCs or T cells in our RNP editing system (Figure 2E–H; Supplementary Figures S6 and S7). The FORECasT program performed better than inDelphi in predicting outcomes in K562 and iPSCs. Notably, NHEJ editings were considerably higher than both inDelphi and FORECasT predictions (Supplementary Figure S6E). We attribute this result to the lentiviral-based datasets used for inDelphi and FORECasT development. The constitutive expression of Cas9–gRNA might lead to a considerable decrease in NHEJ because the same gRNA may still identify a single nucleotide insertion or deletion of the target (59). MMEJ frequency predictions by FORECasT were also significantly lower than our RNP editing data (Supplementary Figure S6E). Therefore, current machine learning programs are limited in their predictive accuracy, particularly for RNP editing.

We are the first to report the RNP-mediated editing dynamics by the NHEJ, MMEJ, and HDR pathways to the best of our knowledge. At particular loci, a sizable amount of +A/T NHEJ-mediated repair has been accomplished 4–8 h after RNP delivery. However, MMEJ takes 2–3 times longer (Figure 3E, F; Supplementary Figure S8G, H). Of interest, the HDR repairs using the AAV donors with ∼600-bp of homology occurs faster than MMEJ deletion of over 10-bp (Supplementary Figure S12). We also observed cell-type differences in editing patterns, which might be attributed to differential expressions of the factors involved in DNA damage repair. iPSCs showed higher frequencies of +1 editing compared to other cell types (Figure 2A, B). However, in all these cell types, +1 NHEJ editing is most frequent, with –4 position being T, followed by A. SpCas9 exhibits a strong bias for the +1 insertion at the fourth base from the PAM due to staggered cutting, as evidenced by modeling, structure and functional studies (30,57,79). In clinically relevant and editing-vulnerable cell lines such as human hematopoietic CD34^+^ cells, delayed repair by MMEJ may lead to a hyper-activated TP53 signaling pathway and increased cell death (80,81). We speculate that the preferential selection of gRNA with T/A at the –4 position may lead to greater editing efficiency and better survival in clinical therapy.

AAV6 has been widely used for HDR editing of iPSCs, HSCs, and T cells. Using RNP electroporation with AAV6 HDR donors, we demonstrate that HDR repairs are proportional to indel frequencies (Figure 4C). After DSBs, three primary pathways—NHEJ, MMEJ, and HDR—are competing to repair the double-strand break. Quantification of T_50_ showed speedy repair by NHEJ, followed by HDR and MMEJ (Figure 3E–G; Supplementary Figure S12); thereby, HDR outcompetes MMEJ, but not NHEJ (Figure 5). The presence of a predominant +A/T NHEJ editing pattern decreases HDR efficiency (Figure 4E; Supplementary Figure S11B). Accordingly, the presence of AAV donors considerably decreased MMEJ, but not NHEJ editing.

Many small molecules have been reported to increase HDR efficiency considerably; however, in our system, only M3814 achieved remarkable effects (Figure 6B). High-level baseline HDR levels and cell line differences may explain the discrepancy between our results and previous publications. NU7026 and NU7441, the two commonly used DNA-PK inhibitors with demonstrated effects on inhibiting NHEJ and enhancing HDR (82,83), showed a modest increase of AAV6-mediated HDR in our study. In comparison, Olaparib inhibited MMEJ (84), but did not improve the HDR efficiency. In contrast, M3814 strongly inhibited NHEJ and considerably enhanced HDR. These data support our conclusion that HDR outcompetes MMEJ but not NHEJ.

Many laboratories cannot reproduce the claimed HDR-enhancing effects of multiple small molecule inhibitors or activators. For example, SCR7 and SR1 did not appreciably affect HDR in our systems. Conclusions from editing limited loci in a few cancer cell lines may not be generalized. A low-level HDR baseline editing would heighten the positive effects, whereas a high-level baseline would mask the potential benefits. Our work also offers another explanation for this poor reproducibility: different gRNAs produce specific editing outcomes and bias that is linked to its genetic context and locus. We showed that the NHEJ inhibitor M3814 works best at loci with a strong NHEJ bias (Supplementary Figure S14D, E). Besides, the editing patterns of a gRNA are not identical across cell types (Figure 2A–C), which could lead to poor repeatability of these inhibitors among laboratories using diverse experiment systems and cell types.

We discovered that combined M3814 and TSA treatment was a potent booster of HDR editing in both iPSCs and primary T cells (Figure 6D, E and H–K). The combined M3184 and TSA treatment promote HDR editing by strongly inhibiting +1/–1 bp NHEJ events (Figure 6C and F–G). The combined treatment led to a ∼3-fold increase in HDR efficiency and ∼80% HDR editing in bulk populations of iPSCs or T cells, which may find its application in iPSC-based regenerative medicine and CAR-T therapy. We speculate that TSA’s beneficial effect might be attributed to its ability to decondensate chromatin, which increases mobility and local concentrations of RNPs and HDR donors, thus further increasing M3814-mediated high HDR levels. Alternatively, TSA may also improve the ATM-dependent DNA-damage signaling pathway, vital to HDR (85,86).

In summary, we have illustrated editing dynamics and patterns in multiple cell types using a clinically relevant RNP-AAV editing system. The quantitation of NHEJ, MMEJ, and HDR editing pathway kinetics (T_50_) will have implications for designing novel editing strategies. Our data on RNP-based editing outcomes may also improve machine learning algorithms for predicting CRISPR–Cas9 RNP genome editing. We anticipate that the combination of M3814 and TSA will become a universal strategy for achieving high-level HDR editing efficiency in different cell types, enabling wide-spread applications of CRISPR genome editing technology.

## DATA AVAILABILITY

All other data supporting the findings of this study are available from the corresponding author on reasonable request. Illumina sequencing data are deposited in the GEO database under accession number GSE155891.

## Supplementary Material

gkaa1251_Supplemental_Files
