# Supplementary material for: Dynamics and competition of CRISPR–Cas9 ribonucleoproteins and AAV donor-mediated NHEJ, MMEJ and HDR editing
Source: Nucleic Acids Res. 2021 Jan 4;49(2):969–85. doi: 10.1093/nar/gkaa1251 (PMC7826255; doi:10.1093/nar/gkaa1251)
Supplement: gkaa1251_Supplemental_Files [file gkaa1251_supplemental_files.zip › NAR RNP-AAV editing dynamics - Supplemenary File 1 (AAV donor sequence).docx]

**AAV HDR Donor Sequences**

**AAV6-AAVS1-MluPme (Left HA-Insert-Right HA)**

GGGCATCTCTCCTCCCTCACCCAACCCCATGCCGTCTTCACTCGCTGGGTTCCCTTTTCCTTCTCCTTCTGGGGCCTGTGCCATCTCTCGTTTCTTAGGATGGCCTTCTCCGACGGATGTCTCCCTTGCGTCCCGCCTCCCCTTCTTGTAGGCCTGCATCATCACCGTTTTTCTGGACAACCCCAAAGTACCCCGTCTCCCTGGCTTTAGCCACCTCTCCATCCTCTTGCTTTCTTTGCCTGGACACCCCGTTCTCCTGTGGATTCGGGTCACCTCTCACTCCTTTCATTTGGGCAGCTCCCCTACCCCCCTTACCTCTCTAGTCTGTGCTAGCTCTTCCAGCCCCCTGTCATGGCATCTTCCAGGGGTCCGAGAGCTCAGCTAGTCTTCTTCCTCCAACCCGGGCCCCTATGTCCACTTCAGGACAGCATGTTTGCTGCCTCCAGGGATCCTGTGTCCCCGAGCTGGGACCACCTTATATTCCCAGGGCCGGTTAATGTGGCTCTGGTTCTGGGTACTTTTATCTGTCCCCTCCACCCCACAGTGGGGCCACTAGGGACAGGATTGGTGACAGAAAAGCCCCATCCTTAGGCCTCCTCCTTCCTAGTCTCCTGATATTGGGTCTAACCCACGCGTAGTTTAAACTTAGGCAGATTCCTTATCTGGTGACACACCCCCATTTCCTGGAGCCATCTCTCTCCTTGCCAGAACCTCTAAGGTTTGCTTACGATGGAGCCAGAGAGGATCCTGGGAGGGAGAGCTTGGCAGGGGGTGGGAGGGAAGGGGGGGATGCGTGACCTGCCCGGTTCTCAGTGGCCACCCTGCGCTACCCTCTCCCAGAACCTGAGCTGCTCTGACGCGGCCGTCTGGTGCGTTTCACTGATCCTGGTGCTGCAGCTTCCTTACACTTCCCAAGAGGAGAAGCAGTTTGGAAAAACAAAATCAGAATAAGTTGGTCCTGAGTTCTAACTTTGGCTCTTCACCTTTCTAGTCCCCAATTTATATTGTTCCTCCGTGCGTCAGTTTTACCTGTGAGATAAGGCCAGTAGCCAGCCCCGTCCTGGCAGGGCTGTGGTGAGGAGGGGGGTGTCCGTGTGGAAAACTCCCTTTGTGAGAATGGTGCGTCCTAGGTGTTCACCAGGTCGTGGCCGCCTCTACTCCCTTTCTCTTTCTCCATCCTTCTTTCCTTAAAGAGTCCCCAGTGCTATCTGGGACATATTCCTCCGCCCAGAGCAGGGTCCCGCTTCCCTAAGGCCCTG

**AAV6-CD326a-MluPme (Left HA-Insert-Right HA)**

GGCCCCATTCTTCAAGGCTTCAGAGCAGCGCTCCTCCGGTTAAAAGGAAGTCTCAGCACAGAATCTTCAAACCTCCTCGGAGGCCACCAAAGATCCCTAACGCCGCCATGGAGACGAAGCACCTGGGGCGGGGCGGAGCGGGGCGCGCGGGCCCACACCTGTGGAGAGGGCCGCGCCCCAACTGCAGCGCCGGGGCTGGGGGAGGGGAGCCTACTCACTCCCCCAACTCCCGGGCGGTGACTCATCAACGAGCACCAGCGGCCAGAGGTGAGCAGTCCCGGGAAGGGGCCGAGAGGCGGGGCCGCCAGGTCGGGCAGGTGTGCGCTCCGCCCCGCCGCGCGCACAGAGCGCTAGTCCTTCGGCGAGCGAGCACCTTCGACGCGGTCCGGGGACCCCCTCGTCGCTGTCCTCCCGACGCGGACCCGCGTGCCCCAGGCCTCGCGCTGCCCGGCCGGCTCCTCGTGTCCCACTCCCGGCGCACGCCCTCCCGCGAGTCCCGGGCCCCTCCCGCACGCGTAGTTTAAACCTCGGCGCGCGCGCAGCATGGCGCCCCCGCAGGTCCTCGCGTTCGGGCTTCTGCTTGCCGCGGCGACGGCGACTTTTGCCGCAGCTCAGGAAGGTGAGGCGCGGATTGGAGCAGAGTTGTGGAGCTGGGCTGGGCTGGGGGGCAGCGGCCCCCGGCCCTCGGCCCCCGAAACGGGCATAATAGGGAGGGGACCAAGAGGCCGCGCTTTCCAGCGTGGAGACCGGACGGTGCGGCCGTGCTCCGGCTCAGGCCCTCCGCGCGGTAGGAAACGGCGAGGGCCGTCCCGGGGAGCAGCCTCACTTCGCAGCTTTGCTCGCCTTGGTAGGGAAATGGCCTTGGGCGGAGGCGGGGGACAGGCAGGGAACGGAGTGGCCACGTCCAGGTTTCCTGCGGCCACCGAACCGGTGCCTCGCGCCCTGGCGCACCCACGTCCTCGGTTCGGGGTGGACTTGGGGTTCCAAAACAGCCCCAGCCGGTGGCGGAGTCTTTACGACAGGGACCAGCGGGCTCGCCCTTGTCCTTGCAGCGGGCCCCGGATGTGGGCCTCAGGCGGGGACAGGCGCCCG

**AAV6-BCL11A-GATA-MluPme (Left HA-Insert-Right HA)**

GGGCAATACAGACTGGTTCTGTGATGACAAATAACTCCTAGCTCATTCCTAATGATTTATCACCAAATGTTCTTTCTTCAGCTGGAATTTAAAATATGGACTCATCCGTAAAATAGGAATAATAATAGTATATGCTTCATAGGGTTTGTATGAAAATAAAATGAGTGCGTATTTGTAAAGTTCCTAGAGCAGAGTAAGTGCTCCGAGCTTGTGAACTAAAATGCTGCCTCCTGGTATTTATTAGTTACACCTCAGCAGAAACAAAGTTATCAGGCCCTTTCCCCAATTCCTAGTTTGGGTCAGAAGAAAAGGGAAAAGGGAGAGGAAAAAGGAAAAGAATATGACGTCAGGGGGAGGCAAGTCAGTTGGGAACACAGATCCTAACACAGTAGCTGGTACCTGATAGGTGCCTATATGTGATGGATGGGTGGACAGCCCGACAGATGAAAAATGGACAATTATGAGGAGGGGAGAGTGCAGACAGGGGAAGCTTCACCTCCTTTACAATTTTGGGAGTCCACACGGCATGGCATACAAATTATTTCATTCCCATTGAGAAATAAAATCCAATTCTCCATCACCAAGAGAGCCTTCCGAAAGAGGCCCCCCTGGGCAAACGGCCACCGATGGAGAGGTCTGCCAGTCCTCTTCTACCCCACCCACGCCCCCACCCTAATCAGAACGCGTAGTTTAAACCTGTGATAAAAGCAACTGTTAGCTTGCACTAGACTAGCTTCAAAGTTGTATTGACCCTGGTGTGTTATGTCTAAGAGTAGATGCCATATCTCTTTTCTGGCCTATGTTATTACCTGTATGGACTTTGCACTGGAATCAGCTATCTGCTCTTACTTATGCACACCTGGGGCATAGAGCCAGCCCTGTATCGCTTTTCAGCCATCTCACTACAGATAACTCCCAAGTCCTGTCTAGCTGCCTTCCTTATCACAGGAATAGCACCCAAGGTCCATCAGTACCTCAGAGTAGAACCCCCTATAAACTAGTCTGGTTTGCCCATGGGGCACAGTCAGGCTGTTTTCCAGGGTGGGGTGCAGACATTCTCTGCCTGTTGTGATGCTTACATATAACGTCATAACAGACACACGTATGTGTTGTGATCCCTGTGGTTTGAGAGTTTGGAGCTTCCCTAAAAGTCAAAATATTCTCAATGGGCCCTCAATCAGCACATACACACAAAAGGTACCTGGAAAACTGTAATTCTTTTCCTGCTCAAAGACAGGCAATTCAATACCCCTTCCCCCAACCAAAAACCCT

**AAV6-GATA4-MluPme (Left HA-Insert-Right HA)**

CAGGGGCACCAGGAGCCCCTTCTGGGCCGGGTGGATGGCTTCTTTGTTGGAAAGTGGATGTGGTGGTGATAGGATGGTAGAAAGTGTCTCCTGTAACCATCAGAGCCTTCTGGGCAACCACAGTATCCACAGGGCCACCGGGTCATAGCCCTGGTTGTATACTGTGCTCAGAAGCAGCTGATGCATCACCCAGACCCTTCATGCCTAGATCACCGGGATCAGGAGAAACAGAGAGAAGTGCTCCTTGGTCCCTTCCTGAGGGCTGAAGCCATCCTGGGGACATCTGCATAGCAGGGCACCCTCCCCAGCCTAGACCTCCCAAGCCCTCAGGAGCGTCTCCATGGGCCTCATCGTGTGCTTTCTGCTTTTCAGACGTTCTCAGTCAGTGCGATGTCTGGCCATGGGCCCTCCATCCACCCTGTCCTCTCGGCCCTGAAGCTCTCCCCACAAGGCTATGCGTCTCCCGTCAGCCAGTCTCCACAGACCAGCTCCAAGCAGGACTCTTGGAACAGCCTGGTCTTGGCCGACAGTCACGGGGACATAATCACTGCGTAATCTTCCCTCTTCCCTCACGCGTAGTTTAAACACTTGGAGGATAGCAAAGAAGGAGGCCCTGGGCTCCCAGGGGCCGGCCTCCTCTGCCTGGTAATGACTCCAGAACAACAACTGGGAAGAAACTTGAAGTCGACAATCTGGTTAGGGGAAGCGGGTGTTGGATTTTCTCAGATGCCTTTACACGCTGATGGGACTGGAGGGAGCCCACCCTTCAGCACGAGCACACTGCATCTCTCCTGTGAGTTGGAGACTTCTTTCCCAAGATGTCCTTGTCCCCTGCGTTCCCCACTGTGGCCTAGACCGTGGGTTTTGCATTGTGTTTCTAGCACCGAGGATCTGAGAACAAGCGGAGGGCCGGGCCCTGGGACCCCTGCTCCAGCCCGAATGACGGCATCTGTTTGCCATGTACCTGGATGCGACGGGCCCCTGGGGACAGGCCCTTGCCCCATCCATCCGCTTGAGGCATGGCACCGCCCTGCATCCCTAATACCAAATCTGACTCCAAAATTGTGGGGTGTGACATACAAGTGACTGAACACTTCCTGGGGAGCTACAGGGGCACTTAACCCACCACAGCACAGCCTCATCAAAATGCAGCTGGCAACTTCTCCCCCAGGTGCCTTCCCCCTGCTGCCGGCCTTTGCTCCTTCACTTCCAACATCTCTCAAAATAAAAATCCCT

**AAV6- HBG1-MluPme (Left HA-Insert-Right HA)**

GGTAAATACACATCATCTGGTGTATACATACATACCTGAATATGGAATCAAATATTTTTCTAAGATGAAACAGTCATGATTTATTTCAAATAGGTACGGATAAGTAGATATTGAGGTAAGCATTAGGTCTTATATTATGTAACACTAATCTATTACTGCGCTGAAACTGTGGCTTTATAGAAATTGTTTTCACTGCACTATTGAGAAATTAAGAGATAATGGCAAAAGTCACAAAGAGTATATTCAAAAAGAAGTATAGCACTTTTTCCTTAGAAACCACTGCTAACTGAAAGAGACTAAGATTTGTCCCGTCAAAAATCCTGGACCTATGCCTAAAACACATTTCACAATCCCTGAACTTTTCAAAAATTGGTACATGCTTTAGCTTTAAACTACAGGCCTCACTGGAGCTAGAGACAAGAAGGTAAAAAACGGCTGACAAAAGAAGTCCTGGTATCCTCTATGATGGGAGAAGGAAACTAGCTAAAGGGAAGAATAAATTAGAGAAAAACTGGAATGACTGAATCGGAACAAGGCAAAGGCTATAAAAAAAATTAGCAGTATCCACGCGTAGTTTAAACCTATCTCAATGCAAATATCTGTCTGAAACGGTCCCTGGCTAAACTCCACCCATGGGTTGGCCAGCCTTGCCTTGACCAATAGCCTTGACAAGGCAAACTTGACCAATAGTCTTAGAGTATCCAGTGAGGCCAGGGGCCGGCGGCTGGCTAGGGATGAAGAATAAAAGGAAGCACCCTTCAGCAGTTCCACACACTCGCTTCTGGAACGTCTGAGGTTATCAATAAGCTCCTAGTCCAGACGCCATGGGTCATTTCACAGAGGAGGACAAGGCTACTATCACAAGCCTGTGGGGCAAGGTGAATGTGGAAGATGCTGGAGGAGAAACCCTGGGAAGGTAGGCTCTGGTGACCAGGACAAGGGAGGGAAGGAAGGACCCTGTGCCTGGCAAAAGTCCAGGTCGCTTCTCAGGATTTGTGGCACCTTCTGACTGTCAAACTGTTCTTGTCAATCTCACAGGCTCCTGGTTGTCTACCCATGGACCCAGAGGTTCTTTGACAGCTTTGGCAACCTGTCCTCTGCCTCTGCCATCATGGGCAACCCCAAAGTCAAGGCACATGGCAAGAAGGTGCTGACTTCCTTGGGAGATGCCACAAAGCACCTGGATGATCTCAAGGGCACCTTTGCCC

**AAV6-MYH6-MluPme (Left HA-Insert-Right HA)**

GGGTTGAGAGGCTGAGAATCCCATAGCCCATCTCCAGCTCATTCACCCATCCCCACTGTCCCACCACAGGAGGAGCAAGCCAACACCAACCTGTCCAAGTTCCGCAAGGTGCAGCATGAGCTGGATGAGGCAGAGGAGCGGGCGGACATCGCTGAGTCCCAGGTCAACAAGCTTCGAGCCAAGAGCCGTGACATTGGTGCCAAGGTGGGTCCCTCCCCTGGGCTTCACTAGTCACTTCCACATTAGCATGCCCCCTGATATGGGTGCCCTTCAGAGTGGGCACTGCTTGCCCTATATGTAGGCAGTTCTGAGGGTCCCATAGCTTACATAACCTGAGAATCCACTCTCCTGCTCAAAACAGCCCCCCACTGACTGGAACTTCTGCAGAGATCCCCAGTTCCATCCCCCTAAACCACAAGTGCCTCTAACGTGGGACCACAGGATCCCTGGGGCCCTGCCTCTCCCTCCAAGGGCATCTCCCTTAGGCCTCTGAAAGCCCCAGGGATTTGTCCCCACACACTTCTCCCTCTTGCCAGCTGCCCCCTCACACCTCTTATTCTTTTTGCAGCAAAAAATGCACGATGAGGAGTGACACTGCCTCGGGAACCTCACTCTTGCCAACCTGTAATAAATATGAGTGCCAAACTCTGCCTGAGCCCATCTGTCCTTCCTGCCAGCTGTCCATCCTGGATCAGGGCAGTAGGGGGCCTGAGAGACGCGTAGTTTAAACCAGAGCTGCCAGAGCCAAGGCATCCCATATCCACAAGGAAGGAGACCGAGGTGCAGAGGAGTGAGGTGTTCTGCCTGACGTGGACAGCTGTGAGTCCCAGTTCTGCTCAGTCAACCACACAAGGGTGGAGAGGGAGAAATTTCAAAAGTGAAGAAAACAATGGCATCAAAATGGTGAGCCAGTCCAAGATGCTACGAGATAGAATTTTGGTGCCGAAGTTGAAGCCACTCTCCACCTACCCCACCCCTGAGGCTGCAATGATTAAAGGTGTCCTAGGACACCCATTCTTTCTGAAATGAATTTTCCACCAATGCCAGAAAAGGCTCAGAGCTGGCTGAGAATTAAGGTATGGGAAAGGAGATGGGAGAGATTCCATTCTAGTCAGCCCCTAGGAAGTAACTCCCAGGGAGCCCCCTCCCTG

**AAV6-EEF2-E2A-mNeonGreen (Left HA-Insert-Right HA)**

GGGGGACAGAAGCCCAGTTAAGCTTAGCAAGGTGTTAAAGGAGGCGTCCTGATGGGAGCAGGTGATGGATGGAGCAGGTGGTCCAGTTTCTGACAGCTTGTGGACCCCCTAAATCACTGAATTCCCAGGGGAGGGGCTCTCCTATCCCCAGTGTGAGAAGGGCTCTGGGCCTGGAGCTCTGAAGGCCTACGCCCTGGGCCGGTAGAGCAGCCGAGCTGTAGCACAGGGTTGTCCCAAACGAGCAGCGGCATGAGGCCCATGAGTGGCCTGCTAGGCCCTTCGTGAAGTGCTGGGCACCAGGCCGAGTGTCTGGTCTGCAGGGTGACTCAGGCTGAGGAACTAGCCTGAGCTCCTGACAGGACTTTCCTTCTGCCCTGCCACCTTCTCGATGGCCCAGTGAGCCTCTCGCTTCCCTCTGCAGGCTTCACCGCTGACCTGAGGTCCAACACGGGCGGCCAGGCGTTCCCCCAGTGTGTGTTTGACCACTGGCAGATCCTGCCCGGAGACCCCTTCGACAACAGCAGCCGCCCCAGCCAGGTGGTGGCGGAGACCCGCAAGCGCAAGGGCCTGAAAGAAGGCATCCCTGCCCTGGACAACTTCCTGGACAAATTGCAGTGTACTAATTATGCTCTCTTGAAATTGGCTGGAGATGTTGAGAGCAACCCAGGTCCCATGGTGAGCAAGGGCGAGGAGGATAACATGGCCTCTCTCCCAGCGACACATGAGTTACACATCTTTGGCTCCATCAACGGTGTGGACTTTGACATGGTGGGTCAGGGCACCGGCAATCCAAATGATGGTTATGAGGAGTTAAACCTGAAGTCCACCAAGGGTGACCTCCAGTTCTCCCCCTGGATTCTGGTCCCTCATATCGGGTATGGCTTCCATCAGTACCTGCCCTACCCTGACGGGATGTCGCCTTTCCAGGCCGCCATGGTAGATGGCTCCGGATACCAAGTCCATCGCACAATGCAGTTTGAAGATGGTGCCTCCCTTACTGTTAACTACCGCTACACCTACGAGGGAAGCCACATCAAAGGAGAGGCCCAGGTGAAGGGGACTGGTTTCCCTGCTGACGGTCCTGTGATGACCAACTCGCTGACCGCTGCGGACTGGTGCAGGTCGAAGAAGACTTACCCCAACGACAAAACCATCATCAGTACCTTTAAGTGGAGTTACACCACTGGAAATGGCAAGCGCTACCGGAGCACTGCGCGGACCACCTACACCTTTGCCAAGCCAATGGCGGCTAACTATCTGAAGAACCAGCCGATGTACGTGTTCCGTAAGACGGAGCTCAAGCACTCCAAGACCGAGCTCAACTTCAAGGAGTGGCAAAAGGCCTTTACCGATGTGATGGGCATGGACGAGCTGTACAAGTAACGCGTGCGGCCCTTCCTGCAGCGCCTGCCGCCCCGGGGACTCGCAGCACCCACAGCACCACGTCCTCGAATTCTCAGACGACACCTGGAGACTGTCCCGACACAGCGACGCTCCCCTGAGAGGTTTCTGGGGCCCGCTGCGTGCCATCACTCAACCATAACACTTGATGCCGTTTCTTTCAATATTTATTTCCAGAGTCCGGAGGCAGCAGACACGCCCTCTTAGTAGGGACTTAATGGGCCGGTCGGGGAGGGGGAGGCGGGATGGGACACCCAACACTTTTTCCATTTCTTCAGAGGGAAACTCAGATGTCCAAACTAATTTTAACAAACGCATTAAGAGGTTTATTTGGGTACATGGCCCGCAGTGGCTTTTGCCCCAGAAAGGGGAAAGGAACACGCGGGTAGATGATTTCTAGCAGGCAGGAAGTCCTGTGCGGTGTCACCATGAGCACCTCCAGCTGTACTAGTGCCATTGGAATAATAAATTTGATAAGGTGGTGACTCTGTTCTGCATTTTTCACGGTGTCTTCGCAGGGGAGCGGGGCTGCCCAGTACTGGGCTCCCTGGAGCCTAGAAGGGGACCCGGGCCCT

**AAV6-BCL11A2a-Syn77-Pme (Left HA-Insert-Right HA)**

AGGGGGGGTGTCGGGCTGCAGAGCCGCCCAGCAAGCCAGCCGAGGCCACCGAGGTCTGTGCTTTGCATGGGGGTGAGGGGGTGGAAGAAGTGAGGCTGGAGACCAAGTGCAAACTTGCCATATCCCCCTCTTCGGCCATGCCCCGAAGCCCTAGAGCGCGCACTCCCGGTTCCTCCCTACCAAAAATGCCGGCCCCGAAAGAAAGCGAAAACTGCACCGTGCTCCGAGCCCGGCGCGCGCTGGTCTCAGCTCGCGCACGGGGGTGTCGGCCGCGCGCCCGGTCGCTTCTCCGCGGCCCCGGCCAGAAGCATTTTTAAAGTCAAAATGAAGAACAAAGACATACGGGGTGATGGGGGAGAGAAAGGGAATTCTGCCTAACCGTTCTTTCCCCCCACCCCCACCCTCGCAAAGCTCGTTCCTCCCCGGACGAGAATCGCCGGGCAGGAGGTGGGGGAGGCTCTGGTACAGGTTCAAGTTCGCTGAGCTTTCTTGATCTTGTTCTGCCTCCTGTTTAAACAGCGACCGAATGGCTCATTCAGCCCACATAGCAGAGGCGGGGGGCGGGGGGGTGGTACTGAGGACCGGGATGGCTCCTGCCCCCTCTGAGCACCCAGAAAATGAGGACAGAAAGAAGTGGCCCCCTCACCCCCTCCCAAACTGGCCCTTTAACCCCGGGACAGGAGGAGGGGGTACTAACCCTCCCAGCCTAGAGTGTCCCAAGATGGACCCAGGAGGGAGAGGGGTACGAGGGAGCAGCCTGGACTGCGCGCCCCGGTCTGTCCTTTGAGCCCCCACTGCATCCTTCCGAAGAAGGGCCTGACTTTCCCGAGTCCTGCGAACACTGCCCTTCCTTCCCGCCGGTACCTGGCCGGGCTGTAACTTCACACCGCCGCGCGCCGCGTCTGATCCGCATCCGGCGCGGCCGGGGGAGTTGGGGGCGGGGGAGAGCGGCGAGGGAGGGATGCGAGGGGGTGGGGAGAGGGAGGGCCGCGCAACGTGCCGGGGTGGGGGAGCTTCCGAAGTGTGTGACAAGTTCCAGGGCCCAGTGAAAAATTAAATTCCCTG

**AAV6-BCL11A2b-Syn78-Pme (Left HA-Insert-Right HA)**

GGCAAAGTTCTTGAAAATAACAAGATGATCAAATGTACAGATATAAAACCTGTAACCTCAACATTTTCTATGCTCTTAACGTAAATTATTGCTAATTAACAAGAATTATATTCTAATGTATGAAATACGTTTAACATCGGTCTCCTTGCAAAACATTTGGCTAGTGGTGTTCAGAGAAATACCAAAACGTGTTTTTATCATTGCTGGTATATTATTAAAAATCAGACAAAGTGGGACACAAAGAAAATATGAAAATACAATTCTCATGGTGTAGTCTACTGATTTGCATTGACATCTTTTCTCAGTGGCAATCAACATTTCCTTTCTGGGCATCTGATAATTTCTCTGCATTTTAATGCAAATTCCCCTTTCCCTAAAAACCTTCCTAACCCTCAGCCATTTACCCCCTCCCTCAATTCAACTCCATCCTCCTTCGCCCCAACAATACCAACATTTCTAAGCTCCGGCCTAAACTCCGGCTACAAAGCAGGGAGTCCTCGATTGAAGTGGAAGGGAGTGGGCAAATAATCATAATAATTAGCCGCGAATTCTCTCTACGAGGTTTAAACGGGAGGAAGAAATAGAAAATAAAATGCTTCTACGTTCAAACAGCAAGACAGCCTGGAACGTCTCAATGTCTCAGTGACAGACTGAGATCTCTTTCCTCTCTGTGTAGTTGCAAAAGAATAAATTTAAGGGAAAAAAATAATCCTTATAGGAAATGTTGGTAAGGAGCGAGCAAAACGAGAGCTGCCGGGGAGCGGAGGGGGAGGGGGAAAGGGAGATGTGTCTTCAAGTTTGCTTTCCAAGTCCTTCTCCTTGATTGTCTTAACAGGGTAAAGAAGCAAACGGAGGCTGAAATTCAGAGAGGAAAAGCAAAGAAACAAAAGCAAAGCCGGGGGCTACAGGCCCGGGGCAGGGGCCCCGGCAAGGGTGGACCGTGCAGAAAGTGAAGTTGGGCGCGGGGACTCTGAGGCACAAAGCGAGCAGCGTGCTGGCTGCAGGCTCCCGCCGCGCTTGCTGCGAACACGTGCAGCAGCGGCCGCGGGGCCTTGGGGCCCGGGGGCCCGGAGACGGAGGGCAGCCAGGGGCCCTGACCCTGA

**AAV6-BCL11A2c-Syn79-Pme (Left HA-Insert-Right HA)**

CAGGGTAAAGAAGCAAACGGAGGCTGAAATTCAGAGAGGAAAAGCAAAGAAACAAAAGCAAAGCCGGGGGCTACAGGCCCGGGGCAGGGGCCCCGGCAAGGGTGGACCGTGCAGAAAGTGAAGTTGGGCGCGGGGACTCTGAGGCACAAAGCGAGCAGCGTGCTGGCTGCAGGCTCCCGCCGCGCTTGCTGCGAACACGTGCAGCAGCGGCCGCGGGGCCTTGGGGCCCGGGGGCCCGGAGACGGAGGGCAGCCAGGGGCCCTGACTGCTGCCTCCATGCCCGTCCAGCTCGGCCAGCTCGGCCGCGGGGTGACGGTTCCCTGGGCCTGCCAGTTGGTGAAAAAGGAAGTGAGGTTTGGAAAACCCGGCTGGTTAACCTGGTGCAGTCTCAGTATCTCCGCACACACCTCAAGCGCGGGTCCTGAGATTCATTCTGTGTCCACAAGCTTACACTTTTTACTTTGGGGGATCTCTGCAAATGCATTTCCCCCGCAATTCTGGGCACTGGGTGTGCGCGGCGGCGGCTGCGGTTATTCATTATTAATGACGCTGCCTGCCCGCATCATTATGATGATAACTATTACTATTGTTGTGATTCCGAGCTGTTTAAACCCGAGGCGAGAGGGGGGGTGTCGGGCTGCAGAGCCGCCCAGCAAGCCAGCCGAGGCCACCGAGGTCTGTGCTTTGCATGGGGGTGAGGGGGTGGAAGAAGTGAGGCTGGAGACCAAGTGCAAACTTGCCATATCCCCCTCTTCGGCCATGCCCCGAAGCCCTAGAGCGCGCACTCCCGGTTCCTCCCTACCAAAAATGCCGGCCCCGAAAGAAAGCGAAAACTGCACCGTGCTCCGAGCCCGGCGCGCGCTGGTCTCAGCTCGCGCACGGGGGTGTCGGCCGCGCGCCCGGTCGCTTCTCCGCGGCCCCGGCCAGAAGCATTTTTAAAGTCAAAATGAAGAACAAAGACATACGGGGTGATGGGGGAGAGAAAGGGAATTCTGCCTAACCGTTCTTTCCCCCCACCCCCACCCTCGCAAAGCTCGTTCCTCCCCGGACGAGAATCGCCGGGCAGGAGGTGGGGGAGGCTCTGGTACAGGTTCAAGTTCGCTGAGCTTTCTTGATCTTGTTCTGCCTCCTAGCGACCGAATGGCTCATTCAGCCC

**AAV6-BCL11A4b-Syn83-Pme (Left HA-Insert-Right HA)**

AGGGAAATAAGAAGGGTTCTAAAGCCATGATACGCCTTTTATCATTTCAGCTTTATCATTTCAGTCTAGGAGACCCAGCAAATAAAACTAAGAGCTTCTCTTCCAGGGTGACAAGGGAGAACCACTACACTCTTGACCCAAGGGTATGTAGAGACAATGAAGAGGGCCAAGAGAAGGGGTGAGGGGGAAGGGCTTGCATGACCTCCATTGAGGACGTCCATGCCCCGGGACAGAGTGCGAAGTAAAAGTGAAGCTTTGGAGCTCTTGACCCACATGGATTCTCAGCAGCCACCTCCATCCCACCTCAAACTCAGAAGACAACCTGGATGTGACCGACAACTTCTTTGTGATGGATCTGAACAGCCTCCCTTGGTTCTAGCAGAAGGACTTACCAAGTGAGCAGAGCTCCCATCCGATACTCGCCAGTGCTGAGGCCAACTCTTCCACTCCTCCCACGGTTTAAACGGGCCTCCGCATGAAGTAGGCGCTTCTCAACTACCAAATGATTCTGAAATTCTCTCAGTGGAGGTCTATGTTAGCCTGGCCAATTTGAAAGGATGGAGAATACAGGAAAACAGTAACATTTGGTTTTGGAATACATTGTGTAATCCATCTGCAACGAACCTCAGGTCTTGCTTCAGCAAATATTAATAGCGTCAGGGCAGCCTGTGGAGTGACTGGGAGAGGCCACCACGCAGCTGAAAACTTGCCTCTGGGGAAGCCACATGCGGATCATATGGAGCTGTTGACTGCAGTTTCCATTCAAAACTTCCCTCAAGTTTCCAGCATGCCTTCCGGACTTGCCTTACCACAGCCTTGTTCAGCCAGAAATGAAAACCTCCTCGAAGCAGATTATGACCCCCCAGAACCCATCGTCCTAAGAAACACCACTGCCACGCACACTCACTCACACTCAGTCTCTCCCTCCCTGTATAATTCTGATTCTCCCCAGCCTTTGAAGGTACGATCCGAGAAGTAAATGCCACAGAGACAACACACAGAACTGCTGCAGGCCAAGCCTCACCACTTCTTAAAAGTCCGAGCTTAGTTTCTCATTCAGCCC

**AAV6-BCL11A4c-Syn84-Pme (Left HA-Insert-Right HA)**

GGCTACACTTCCTTTTCCTTTCCTCTCCATTTCATCCCTTTCCAAAAAGTGTTTAGACAAATAGTTTCCCAGACTTGGTTTTATCATGCTGGGTTGACAAAGGTTGTGTACAGAGCTGGAATAATTTTTTCTTCTTTCTACTGTTGGCACATCAATATCTTTTTTTCTGCAAAGAAGGGGCTAAGCTTGCACGAAAACACCGGTGGGAACCCACTGGAGAAGGGCATGGGTGGTTTTTGGTTCGGTTGGTGGCTGATCTGATGGGTGGTATAGGCAGAATGTCTGGCTGCCCCTTCAGATTCCTGTAGATTCCGTAGGCGACCAACATGGGTTTAAACGGTGGGGGTGGGGGACTGTCTGCTCTTTTTGATGCAAATATCTTCACTCTCCTTGGTGCTTCTGTGGTTGTGCCTATGAATTTAGGACCTTATGCCTCATAGTGTTGAGTCAGAGCAAAACAGTTTACATCAAAGGCCACTTATTTAATTTTCCTCAGAAAACTCTGTGAGGATAATTTCTCCTTCTTATAAACGGAATGCGAATGTGTACACAAATGTGTACATTATCCCACCGAAGGGTGATGCTCTTTACTTTTATAATCTAGATTTTAAAAAATTTGGTGGCACTTGATTTCAGGTCCTCAGACTCCAGAATTTATACTCACTGATTAGTAATGCATTATGCAAATAATTCAGTTTACATATCCAAATACTGTTTTGCATCATTCACACCTCATGTCTTGAAAAAAAAAGTGAAATGTTTCCTCAAAATAAACAAAACCTGCAGAAGAGTTTATTGTAAGGGTGAAAATTAAAGAGGAACCCATTTGCCTGGGAACTTTTGAGGAAAATTTTAATACTCCTGCATAAATAATTTTGCAATTATTTTTGATTCCTACCTAATCAATATACCTTGCCACGCAAGGTATATTCTATTATCTTTGGCTTTCTGAATTTTTCAATTTTCAGAAAAAGAAGCC

**AAV6-BCL11A5b-Syn87-Pme (Left HA-Insert-Right HA)**

GGCACCAAGAAGGCACAAAAAATAAAACATGACTCCTGCCCTCCAAAAGGTTATAATTTGAGGAGGAGAAAATACACCCACGAGCCAATGTGAATACCACTGATCCCCTTTTAACTAATCCCAGAGTGACACCTCCTCCAAAGAAAGCCAAGTTGCTGACAGAGTCAGGCCTTCCTAGTTACCTAGAAAAATGGCCTGGGGAGGTCCAGCCCACTAGAAGGAGATACAGAAACAAGCCCTCCATAAATATCTGTGGGACCCAGCTACCTCTCTGAAACACAGCCCAGTGCTTTCAGGCAGGGAGAAAGTGGTGGTGGGTGGCGGACAGAGATGTGAGTGGACTGACACAGACATGGGGGCTTCCAAATGACTCTTGATCACCCAACTTTCCAGCTTCCCCATTTCTGCCCCTGACTTGAGCTCCCAGGCCACTCAACCCACTGTGAGGTCTTACTGTGGGGATTACCGAGTCACCGTTTAAACACCAGGCTGCGCTGAACAGGAAGTGAACAAGTCAATTCGAGAAAGATCCAATAAGGGAATATTCTCTGGGCACCGCTGGGCCAACACCCACGGAGGGGTCCTGGAATACAAATACCCTGGAGTCTGAAGGGCGGTGGCCCCTCTTCTCACCACTTCAATTATGTCTTCATTGAACTGGGTTATGAAAAAGGGAAGCAGGGATTGAAAATCTAGCCTTGTGGCCATAAGCCTGTGATGACTCATGCCAGCTTTGGGTGCTCTAAGATGCTTTCCCATGTCTGTAATGCTTTTCATATCATCCTACACACTCACTGTCACCTCTCCTCACCCCAGCACACAGAAAAGATGTGCTTATTATGTGCACTTAGGTTTTGTTGTACTATTAAACACAATGACCCACTCCTATTATTAAAACACAGGTTTTGCTGGAAACTCAAAAAAGACTTGGCTCTGCTGGCTCACAGGGAATCTGGGCTCCGCTCTTCCAAAACCTTTGTTCTCCAGATGTCAGTGCAAAGTTATGAACAGCTCTCCCCCTGA

**AAV6-BCL11A7bc-Syn92(93)-Pme (Left HA-Insert-Right HA)**

GGAGTTACTGGCTTATGAAATGCAATGGGATGAGGTGGGGTGAGAGGCCATAGCCCAGACTGAACGGCACAGTGACCAGGTAGGGTGGAGCAAGGCCACTCCAGCAAGAATGCCAAGGATAGCCTGCAGTGGGTTATTAGCCCCAGCAGGGCCCATGCCTCTACGGCCATGTTGCTGTCATCTTCGTTCTTGGCTAGCAGATGCTAGTCCCAGGAATGTCCTCCATGCCTTTAAGCTAACTGCAAAAACAGAACGGTGTGAGCCAGGGCAGACCCCATACTCTTTTATATTCTACCTATAAGGGAAAAATAAACCTAGACCTGGAAAACAATGTGTAAAGTGGCAAAAGCCAATCAACTCATTTAAAAAGAAAAACCCTCCGTTTAAACATCCAACGTCACGAAGCTGCTGAATATTAGGTTCCTGGGAAGCTGTCCCAGCAACCTAGATATGAGAAACAACGCTTGCTATTCTGGGGTGAGAAGTGAAGTTGCCTTTCACTCGGGACTGTCAAACACAGTCTATAAATACCAGCAATGCCCCTTGTGAACTACGGGCTGAGCTCAACCCAGAGAAGGCAAGTGTTTCCCAAAGGTCACACAGCAATTCCAGGTCCCCCAGAAGTAGCCCTTCACCAGATGGATCCCATCACCAGATAGCTCTTGGAAATAATACATTGCTGCATCTGATTCTCATTTTCAACAAGAAGGCTGAGGAATGAGATTGCACATTTTAGTTTCCTTCCCCAACTTTCTTTCTCCAACCTTCTGCTCTGGTTCTCCTCTCCCCCACCACACGTACAAAAAAAATCTTTTTAGTATGTTTAGGGATCTATTTCCATATCTACCCAAAATCTCCAAGCCCCT

**AAV6-BCL11A7d-Syn94-Pme (Left HA-Insert-Right HA)**

AGGGCAGCAAACATGGACACTTTTGGCCCCCTTCAGCTATTAAGTACAGTAAAAGGAAGTCCTAACTATCATTTGAAATGCTCCCGGCCAGGCGCTGTGGCTCACACCTGTAATCCCAGCACTTTGGGAGGCCGGGGCAGGAGGATCACCTGAGGTCAGGAGTTCAAGATCAGTTTGGACAACACAGTGAAACCCCGTCTCTACTAAAAATACAAAATTAGCCGGGCATGGTAATACATGCCTGTAATCTCAGCTACTCGGGAGGCTGAGGCAGGAGATCGCTTGAACCTGGGAGGCAGAAGATGCAGTGAGCCGAGATCGCGCCATTGCATTCCAGCCCGGGCAACAAGAGTAAATCTCCGTCTCACCAAAAAAAGAAAAAAGAAAAAAAAAAAAAAGAAAGAAATGCTCCCCTCAGTTCTCCAGGCTGGGCTGACTGCCCACGTCAAAACACCCTAGGCATCATCACTCCGTGTTGAGAGCCAAGTGGCCAGGTGTTTAAACAGGACCCAACACTACGCAGGCACAGACGTTTTCCATGTTAAGAACCCAAATCCATACAGTCCTTAGCACCCAAATAGGTTCAGAAAAGGCAGTCCACACAAATCTGCCAAATAAATGAAGTTCAGATATTGCATTTACGCTAAATACCTTGGAAATTGAGAGGGAATCTGGAGAGGAAGATGGATGTAGGAAAAACAAAAACCCTCACTTTACAGGAATTCAGCACAGGAGATGCTACATTGTTAGCATCTCCGCTTAAGGAGAAGACATACTGATGAATAAGACTGAGTTGGTGACAGGCACAGTGGCTCTCGCCTGTAATCCCAGCACTTTGGGAGGTCAAGGCAGGAGGATCACTGGAGCCCAGGAGTTCAAGACCAGCCTGGGCAACAGGGAGAAACTCCATCTCTACAAAATTAGCCAGGCATGGTGGCATGCACCTGTCGTCCCAGCTACTCAGGAGGCTGAGGTGACAGGATGGCTTGAGCCTAGGAGATCAAGGCTGCAGTGAGCTATGAACATGCCACTACACTCTAGCCTAGGCAACACAGCGAGACCCTG

**AAV6-BCL11A8b-Syn96-Pme (Left HA-Insert-Right HA)**

GGGCAGTACAAAGAAAACACGGACACAGATTTCTCCCACAGCAGGCTTCATTTAGTAGAAAAGGTAAGGCCTGCACAGAAATTGCTCTACCCCCATGTAGAAAGTGTGTCATGAGAGAGGTACAGATGAGCTGCTATGGAAATTCAAAGGAAGAGAAATTATTTCCATTTGGAAGGGAGGGAGAGAGGCTTTGGGAAAGAGGAAAACTTATGAGTTAAACCTGGAGAGCCAAGAGAAAGGATATCCTAGATAAAAAGAAAAAGCAGGTAAAGATGCTTAGAGAGAAGCAAAGCCCAAGGTGGGCACAGAGTAAATGGGGCCCAATAGGCGGTTACATTCACATCAAAATATTGCACACTGATTCATCCAGTTGACAGCGGAGGATACGACCATCTAAATAAATCCTCTGAAGATTTCTGTGACATCTTCAGCTTTCACAAGTGCGTATTTTCCAATTTCACATTTTCTGAAAATAAGGGACACAACCATGTGGACTCTTGTGAAACTCCATGAAGGTTCATCTGTTCTGTTTTCCCCCCACCTCAATTGTTTCTTCCCCCAATGTTTAAACGAATGTGGGTGGCCCGCTAAGGCAAAATTTACTATGTTTTATTCCTCTGAGTCAGACAAACAAGTGTTGATAGCCATGTTCTTCCTTGCAATACTGCCTCCCTGGCTTAAATAATCTATTTCAGATGTCTCATTGCCAGTTTAAGATGGGCTTGTACTTGCTGACACATTTTCAACAAATGACTGTGCAAGAAACATTGAGATTTTCCTACACTACTTTGTCTCTTCAATCATCAACTTGACCATCCCTCCAAATTTCCCCTCTTTCTTCTTTTCAAAGCCATGGGGAGGATAGGGGTGCCCTTACTCCTGCTCTGAGTCCTCAGCTGAGGATCTTGTTTGTGTGTAGTGGGGGCAGGGGGAGGGGTGTAGCCTTGCCTCTATCCGAGGTATTTATCAGGTTATTTGGGAGCTACTTTCAGAGATGCCCTTGCTCTATGTATTCCCACTGCCACCACTCCCCGGATTCTTTTCTTCCTTTAGGAAGCAGGGACTTGTTCCTCTGGAGAGACTGCTAGAGGAAAGTCAGGAACCTAAGTTCTGCAACCCC

**AAV6-BCL11A9a-Syn99-Pme (Left HA-Insert-Right HA)**

CAGGGAGTGGGGCTGGAGGGCGATGGGGAAGGGGAGTGGTGAAAAAGGGGGTGTCAGGTGGGAGTGAGGGAGGGGTATTAATATACCTCTATTCAGTTTTTATATCATTATTCAACACTCGATCACTGTGCCATTTTTTCATGTGTTTCTCCAGGGTACTGTACACGCTAAAAGGCATCTTACAAATTTCACATTTGTAAACGTCCTTCCCCACCTGGCCATGCGTTTTCATGTGCCTGGTGAGCTTGCTACTCTGGGCACAGGCATAGTTGCACAGCTCGCATTTATAAGGCCTTTCGCCCGTGTGGCTTCTCCTGTGGACAGTGAGATTGCTACAGTTCTTGAAGACTTTCCCACAGTACTCACAAGTGTCGCTGCGTCTGCCCTCTTTTGAGCTGGGCCTGCCCGGGCCCGGACCACTAATATGGGGCGTGCTCCCTCCACTTCCCGTGCCGCTGCGCCCCGAGATCCCTCCGTCCAGCTCCCCGGGCGGTGTGGAGAAGCGCAAACTCCCGTTCTGTTTAAACCCGAGGAGTGCTCCGACGAGGAGGCAAAAGGCGATTGTCTGGAGTCTCCGAAGCTAAGGAAGGGATCTTTGAGCTGCCTGGAGGCCGCGTAGCCGGCGAGCCACTGCGAGTACACGTTCTCCGTGTTGGGCATCGCGGCCGGGGGCAGGTCGAACTCCTTCTCGAGCTTGATGCGCTTAGAGAAGGGGCTCAGCGAGCTGGGGCTGCCCAGCAGCAGCTTTTTGGACAGGCCCCCCGAGGCCGACTCGCCCGGGGAGCAGCCGCGGCCATTAACAGTGCCATCGTCTATGCGGTCCGACTCGCCGGCCACCGAGTCTTCGTCGCAAGTGTCCCTG

**AAV6-BCL11A9b-Syn100-Pme (Left HA-Insert-Right HA)**

GGCCCTCGGCCTCGGCCAGGTGGCCGCGCTTATGCTTCTCGCCCAGGACCTGGTGGAAGGCCTCGCTGAAGTGCTGCATGGAGCTGAGCACCATGCCCTGCATGACGTCGGGCAGGGCGCGGCTCTCGTCGCCCACGCCCACGACCGCGCCCCGCGAGCTGTTCTCGTGGTGGCGCGCCGCCTCCAGGCTCAGCCCGAAGCCGTAGTCCACCCTCTCGCTCTCCGTCAGCTCCTCCTCCTCCTCTTCCTCCTCTTCTTCCTCTTCCTCGTCGTCCTCCTCTTCCTCCTCGTCCCCGTTCTCCGGGATCAGGTTGGGGTCGTTCTCGCTCTTGAACTTGGCCACCACGGACTTGAGCGCGCTGCTGGCGCTGCCCACCAAGTCGCTGGTGCCGGGTTCCGGGGAGCTGGCGGTGGAGAGACCGTCGTCGGACTTGACCGTCAGTTTAAACTGGGGGACGATTTGTGCATGTGCGTCTTCATGTGGCGCTTCAGCTTGCTGGCCTGGGTGCACGCGTGGTCGCACAGGTTGCACTTGTAGGGCTTCTCGCCCGTGTGGCTGCGCCGGTGCACCACCAGGTTGCTCTGAAATTTGAACGTCTTGCCGCAGAACTCGCATGACTTGGACTTGACCGGGGGCTGGGAGGGAGGAGGGGCGGATTGCAGAGGAGGGAGGGGGGGCGTCGCCAGGAAGGGCGGCTTGCTACCTGGCTGGAATGGTTGCAGTAACCTTTGCATAGGGCTGGGCCGGCCTGGGGACAGCGGTGGGCTAGACGTGTTCCCTGCCAGCTCTCTAAGTCTCCTAGAGAAATCCATGGCGGGAGGCTCCATAGCCATTGGATTCAACCGCAGCACCCTGTCAAAGGCACTCGGGTGATGGGTGGCCAGGGCCATCTCTTCCGCCCCCAGGCGCTCTATGCGGTGGGGGTCCAAGTGATGTCTCGGTGGTGGACTAAACAGGGGGGGAGTGGGTGGAAAGCGCCCTTCTGCCAGGCCGGAAGCCTCTCTCGATACTGATCCTGGTATTCTTAGCAGGTTAAAGGGGTTATTGTCTGCAATATGAATCCCATGGAGAGGTGGCTGGGAAGGACATTCTGCACCTAGTCCTGAAGGGATACCAACCC

**AAV6-BCL11A9e-Syn103-Pme (Left HA-Insert-Right HA)**

GGCCTCGGCCAGGTGGCCGCGCTTATGCTTCTCGCCCAGGACCTGGTGGAAGGCCTCGCTGAAGTGCTGCATGGAGCTGAGCACCATGCCCTGCATGACGTCGGGCAGGGCGCGGCTCTCGTCGCCCACGCCCACGACCGCGCCCCGCGAGCTGTTCTCGTGGTGGCGCGCCGCCTCCAGGCTCAGCCCGAAGCCGTAGTCCACCCTCTCGCTCTCCGTCAGCTCCTCCTCCTCCTCTTCCTCCTCTTCTTCCTCTTCCTCGTCGTCCTCCTCTTCCTCCTCGTCCCCGTTCTCCGGGATCAGGTTGGGGTCGTTCTCGCTCTTGAACTTGGCCACCACGGACTTGAGCGCGCTGCTGGCGCTGCCCACCAAGTCGCTGGTGCCGGGTTCCGGGGAGCTGGCGGTGGAGAGACCGTCGGTTTAAACTCGGACTTGACCGTCTGGGGGACGATTTGTGCATGTGCGTCTTCATGTGGCGCTTCAGCTTGCTGGCCTGGGTGCACGCGTGGTCGCACAGGTTGCACTTGTAGGGCTTCTCGCCCGTGTGGCTGCGCCGGTGCACCACCAGGTTGCTCTGAAATTTGAACGTCTTGCCGCAGAACTCGCATGACTTGGACTTGACCGGGGGCTGGGAGGGAGGAGGGGCGGATTGCAGAGGAGGGAGGGGGGGCGTCGCCAGGAAGGGCGGCTTGCTACCTGGCTGGAATGGTTGCAGTAACCTTTGCATAGGGCTGGGCCGGCCTGGGGACAGCGGTGGGCTAGACGTGTTCCCTGCCAGCTCTCTAAGTCTCCTAGAGAAATCCATGGCGGGAGGCTCCATAGCCATTGGATTCAACCGCAGCACCCTGTCAAAGGCACTCGGGTGATGGGTGGCCAGGGCCATCTCTTCCGCCCCCAGGCGCTCTATGCGGTGGGGGTCCAAGTGATGTCTCGGTGGTGGACTAAACAGGGGGGGAGTGGGTGGAAAGCGCCCTTCTGCCAGGCCGGAAGCCTCTCTCGATACTGATCCTGGTATTCTTAGCAGGTTAAAGGGGTTATTGTCTGCAATATGAATCCC

**AAV6-BCL11A9f-Syn104-Pme (Left HA-Insert-Right HA)**

GGCCGCGGCTGCTCCCCGGGCGAGTCGGCCTCGGGGGGCCTGTCCAAAAAGCTGCTGCTGGGCAGCCCCAGCTCGCTGAGCCCCTTCTCTAAGCGCATCAAGCTCGAGAAGGAGTTCGACCTGCCCCCGGCCGCGATGCCCAACACGGAGAACGTGTACTCGCAGTGGCTCGCCGGCTACGCGGCCTCCAGGCAGCTCAAAGATCCCTTCCTTAGCTTCGGAGACTCCAGACAATCGCCTTTTGCCTCCTCGTCGGAGCACTCCTCGGAGAACGGGAGTTTGCGCTTCTCCACACCGCCCGGGGAGCTGGACGGAGGGATCTCGGGGCGCAGCGGCACGGGAAGTGGAGGGAGCACGCCCCATATTAGTGGTCCGGGGTTTAAACCCCGGGCAGGCCCAGCTCAAAAGAGGGCAGACGCAGCGACACTTGTGAGTACTGTGGGAAAGTCTTCAAGAACTGTAGCAATCTCACTGTCCACAGGAGAAGCCACACGGGCGAAAGGCCTTATAAATGCGAGCTGTGCAACTATGCCTGTGCCCAGAGTAGCAAGCTCACCAGGCACATGAAAACGCATGGCCAGGTGGGGAAGGACGTTTACAAATGTGAAATTTGTAAGATGCCTTTTAGCGTGTACAGTACCCTGGAGAAACACATGAAAAAATGGCACAGTGATCGAGTGTTGAATAATGATATAAAAACTGAATAGAGGTATATTAATACCCCTCCCTCACTCCCACCTGACACCCCCTTTTTCACCACTCCCCTTCCCCATCGCCCTCCAGCCCCACTCCCTGTAGGATTTTTTTCTAGTCCCATGTGATTTAAACAAACAAACAAACAAACAGAAGTAACGAAGCTAAGAATATGAGAGTGCTTGTCACCAGCACACC

**AAV6-BCL11A9g-Syn105-Pme (Left HA-Insert-Right HA)**

GGTATACCCAAAGTCCTAAATGAGGCCACAGGAGTGAATGTCCAGTAATAAAATGGGCTCCAGAGGCACAGGCATCTTCAATCACTAGCCACGGAGCAAGACAAGACCCTGGGGAGAAGACCCACAGGGATGAGCTACTATCAGCTCCCAGCCTCCAGTGAGGAAAGAAAAAGGGGAGGGAAGAAGTCCCTCTGGGGCTGAGTGGAGTGGGGAGCGGCTGCCAAGTGAGTAATGGAATAATACATATGACAATCTATTTTGGAAGTAACTCCTTCAGTACTTAAAAAGTAAGGGCAATTTCCAGAAATTCTCATCTCTATACACATGGACATTTGTAGAAGAAATAAGGCTCAACTTACAAATACCCTGTTTAAACGCGGGGCATATTCTGCACTCATCCCAGGCGTGGGGATTAGAGCTCCATGTGCAGAACGAGGGGAGGAGAGGCCCCTCCAGTGCAGAAGTTTATCTGTGAAAGAAACCCAAAATCAAGCACTACAGCTACAAACAACGTGCATCATAAACCACAGGATATCACATTTCAATTCCATTAAAATAGATTACAACATCATTACAACAGCCTTGCAGATAGTTAACAGGCCCAAGGCCTGAAACAAAAGAAGAGTGGTGAGTTATTTCCCAGAGTTTCTGTTTTGTTTTCAATTTCGGTCTTTTTGACAAAAGTGAAAGGGTTTGTTTTAGGAAAGGGGAGAGGTGGTCTTTGGTTCATTTGCTTATTTATTTGTTGTTGTTTGATTATTGTCTTGTGGAAAGAAGATGTGTAAGCAGCAAGGGGAAAACTGGTAATATTGTGGGTAGCCTGGAAACATGGGGGGATTTTATCTGCAAGGCTGGTTTACATGCATACATTAACTTTACTTTATAGACAAACCCAAGCAAAAAGCAAAATGCTGATAGGAAATGAGTAGATTTGTGAATCGTTTTTTATGGGACAGAGTCCCACTTTGCTTTCTCTGTCCCTCTGCCCCCCTCTTCCCATGCTATATTTATGATAGCGCCTTCAGTGATAGGGGAAACCTGGGCATTTCTGGACTGAGCTGGGCCATACTCTAGAATCCTGTCAACTGGAACCC
