# Supplementary material for: Dynamics and competition of CRISPR–Cas9 ribonucleoproteins and AAV donor-mediated NHEJ, MMEJ and HDR editing
Source: Nucleic Acids Res. 2021 Jan 4;49(2):969–85. doi: 10.1093/nar/gkaa1251 (PMC7826255; doi:10.1093/nar/gkaa1251)
Supplement: gkaa1251_Supplemental_Files [file gkaa1251_supplemental_files.zip › NAR RNP-AAV editing dynamics - Supplementary File 2.pdf]

P71 Syn23-crAAVS1f KO

4h (P71-BC1) 7.9%	Type	Reads	Percentage
GAATCTGCCTAACAGGA|GGTGGG	WT		
GAATCTGCCTAACAGGAaGGTGGG	+1	220	1.6%
GAATCTGCCTAACAGG---TGGG	-3	148	1.1%
GAATCTGCCTAACAGGA-GTGGG	-1	94	0.7%
GAATCTGCCTAACAGG--GTGGG	-2	82	0.6%
GAATCTGCCTAACAGGAgGGTGGG	+1	27	0.2%
GAATCTGCCTAACAGGA--TGGG	-2	25	0.2%
GAATCTG-----------GTGGG	-11	21	0.2%
GAATCTGCC--------GGTGGG	-8	20	0.1%
GAATCTGCCTAACAGGA---GGG	-3	14	0.1%

8h (P71-BC2) 15.7%	Type	Reads	Percentage
GAATCTGCCTAACAGGA|GGTGGG	WT		
GAATCTGCCTAACAGGAaGGTGGG	+1	768	2.4%
GAATCTGCCTAACAGG---TGGG	-3	492	1.6%
GAATCTGCCTAACAGGA-GTGGG	-1	425	1.4%
GAATCTGCCTAACAGG--GTGGG	-2	304	1.0%
GAATCTGCCTAACAGGA------	-13	82	0.3%
GAATCTG-----------GTGGG	-11	80	0.3%
GAATCTGCC--------GGTGGG	-8	77	0.2%
GAATCTGC---------GGTGGG	-9	73	0.2%
GAATCTGCCTA------GGTGGG	-6	62	0.2%

12h (P71-BC3) 20.7%	Type	Reads	Percentage
GAATCTGCCTAACAGGA|GGTGGG	WT		
GAATCTGCCTAACAGGAaGGTGGG	+1	1252	2.5%
GAATCTGCCTAACAGG---TGGG	-3	1101	2.2%
GAATCTGCCTAACAGGA-GTGGG	-1	768	1.5%
GAATCTGCCTAACAGG--GTGGG	-2	510	1.0%
GAATCTGCCTAACAGG-GGTGGG	-1	487	1.0%
GAATCTGC---------GGTGGG	-9	251	0.5%
GAATCTGCCTA------GGTGGG	-6	224	0.4%
GAATCTGCC--------GGTGGG	-8	212	0.4%
GAATCTGCCTAA-----GGTGGG	-5	195	0.4%

24h (P71-BC4) 26.4%	Type	Reads	Percentage
GAATCTGCCTAACAGGA|GGTGGG	WT		
GAATCTGCCTAACAGG---TGGG	-3	1677	3.4%
GAATCTGCCTAACAGGAaGGTGGG	+1	1349	2.7%
GAATCTGCCTAACAGGA-GTGGG	-1	869	1.8%
GAATCTGCCTAACAGG-GGTGGG	-1	626	1.3%
GAATCTGCCTAACAGG--GTGGG	-2	621	1.3%
GAATCTGCCTAACAGGA------	-13	294	0.6%
GAATCTGCCTA------GGTGGG	-6	238	0.5%
GAATCTG-----------GTGGG	-11	202	0.4%
GAATCTGCC--------------	-23	174	0.4%

48h (P71-BC5) 32.0%	Type	Reads	Percentage
GAATCTGCCTAACAGGA|GGTGGG	WT		
GAATCTGCCTAACAGG---TGGG	-3	2091	5.1%
GAATCTGCCTAACAGGAaGGTGGG	+1	989	2.4%
GAATCTGCCTAACAGGA-GTGGG	-1	649	1.6%
GAATCTGCCTAACAGG--GTGGG	-2	481	1.2%
GAATCTGCCTAACAGG-GGTGGG	-1	461	1.1%
GAATCTGCCTAACA---------GG	-9	250	0.6%
GAATCTG-----------GTGGG	-11	249	0.6%
GAATCTGC---------GGTGGG	-9	221	0.5%
GAATCTGCCTAACA-------GG	-7	186	0.5%

P72 Syn23-crAAVS1f scAAV6 KI

4h (P72-BC1) 16.8% 9.6%

Sequence	Type	Reads	Percentage
GAATCTGCCTAACAGGA|GGTGGG	WT		
GAATCTGCCTAAGTTTAAACTACGCG TGGGTTAGACCC	HDR	2851	9.6%
GAATCTGCCTAACAGGAaGGTGGG	+1	320	1.1%
GAATCTGCCTAACAGGA-GTGGG	-1	184	0.6%
GAATCTGCCTAACAGG---TGGG	-3	148	0.5%
GAATCTGCCTAACAGG--GTGGG	-2	132	0.4%
GAATCTGCCTAACAGG-GGTGGG	-1	117	0.4%
GAATCTGCCTAACAGGA--TGGG	-2	55	0.2%
GAATCTGCCTAACAGGAgGGTGGG	+1	41	0.1%
GAATCTGCCTAACAGGAtGGTGGG	+1	39	0.1%

8h (P72-BC2) 33.4% 21.4%

Sequence	Type	Reads	Percentage
GAATCTGCCTAACAGGA|GGTGGG	WT		
GAATCTGCCTAAGTTTAAACTACGC GTGGGTTAGACCC	HDR	5402	21.4%
GAATCTGCCTAACAGGAaGGTGGG	+1	324	1.3%
GAATCTGCCTAACAGGA-GTGGG	-1	245	1.0%
GAATCTGCCTAACAGG-GGTGGG	-1	153	0.6%
GAATCTGCCTAACAGG---TGGG	-3	148	0.6%
GAATCTGCCTAACAGGA--TGGG	-2	130	0.5%
GAATCTGCCTA------GGTGGG	-6	67	0.3%
GAATCTG-----------GTGGG	-11	56	0.2%
GAATCTGC---------GGTGGG	-9	[illegible]	0.2%

12h (P72-BC3) 38.0% 23.6%

Sequence	Type	Reads	Percentage
GAATCTGCCTAACAGGA|GGTGGG	WT		
GAATCTGCCTAAGTTTAAACTACGCG TGGGTTAGACCC	HDR	7835	23.6%
GAATCTGCCTAACAGGAaGGTGGG	+1	638	1.9%
GAATCTGCCTAACAGGA-GTGGG	-1	336	1.0%
GAATCTGCCTAACAGG---TGGG	-3	222	0.7%
GAATCTGCCTAACAGG--GTGGG	-2	199	0.6%
GAATCTGC---------GGTGGG	-9	163	0.5%
GAATCTGCCTAACAGG-GGTGGG	-1	159	0.5%
GAATCTG-----------GTGGG	-11	135	0.4%
GAATCTGCCTAACAGGA------	-13	134	0.4%

24h (P72-BC4) 49.8% 32.2%

Sequence	Type	Reads	Percentage
GAATCTGCCTAACAGGA|GGTGGG	WT		
GAATCTGCCTAAGTTTAAACTACGCG TGGGTTAGACCC	HDR	7979	32.2%
GAATCTGCCTAACAGGAaGGTGGG	+1	435	1.8%
GAATCTGCCTAACAGG---TGGG	-3	270	1.1%
GAATCTGCCTAACAGGA-GTGGG	-1	235	0.9%
GAATCTGCCTAACAGG-GGTGGG	-1	198	0.8%
GAATCTGCCTAACAGG--GTGGG	-2	192	0.8%
GAATCTG-----------GTGGG	-11	126	0.5%
GAATCTGCCTAACAGGA------	-13	95	0.4%

48h (P72-BC5) 55.8% 36.4%

Sequence	Type	Reads	Percentage
GAATCTGCCTAACAGGA|GGTGGG	WT		
GAATCTGCCTAAGTTTAAACTACGCG TGGGTTAGACCC	HDR	10113	36.4%
GAATCTGCCTAACAGGAaGGTGGG	+1	567	2.0%
GAATCTGCCTAACAGG---TGGG	-3	409	1.5%
GAATCTGCCTAACAGGA-GTGGG	-1	220	0.8%
GAATCTG-----------GTGGG	-11	195	0.7%
GAATCTGCCTAACAGG--GTGGG	-2	171	0.6%
GAATCTGCCTAACAGG-GGTGGG	-1	162	0.6%
GAATCTGCCTAACAGGA------	-13	114	0.4%

P71 Syn25-crAAVS1h RNP KO

4h (P71-BC6) 20.3%	Type	Reads	Percentage
TAAGGAATCTGCCTAAC|AGGAGG	WT		
TAAGGAATCTGCCTAACcAGGAGG	+1	3126	10.2%
TAAGGAATCTGCCTAA-AGGAGG	-1	339	1.1%
TAAGGAATCTGCCTAAC-----G	-5	284	0.9%
TAAGGAATCTGCCTA-CAGGAGG	-1	189	0.6%
TAAGGAATCTGCCTAAC-GGAGG	-1	181	0.6%
TAAGGAATCTGCCTAA--GGAGG	-2	159	0.5%
TAAGGA---------------GG	-15	85	0.3%
TAAGGAATCTGCCTAAC---AGG	-3	54	0.2%
TAAGGAATCTGCCT---AGGAGG	-3	42	0.1%

8h (P71-BC7) 34.1%	Type	Reads	Percentage
TAAGGAATCTGCCTAAC|AGGAGG	WT		
TAAGGAATCTGCCTAACcAGGAGG	+1	5378	16.0%
TAAGGAATCTGCCTAA-AGGAGG	-1	693	2.1%
TAAGGAATCTGCCTAA--GGAGG	-2	595	1.8%
TAAGGA---------------GG	-15	372	1.1%
TAAGGAATCTGCCTA-CAGGAGG	-1	337	1.0%
TAAGGAATCTGCCTAAC-GGAGG	-1	337	1.0%
TAAGGAATCTGCCT---AGGAGG	-3	128	0.4%
TAAGGAATCTGCC----AGGAGG	-4	103	0.3%
TAAGGAATCTGCCTAAC--GAGG	-2	96	0.3%

12h (P71-BC8) 39.2%	Type	Reads	Percentage
TAAGGAATCTGCCTAAC|AGGAGG	WT		
TAAGGAATCTGCCTAACcAGGAGG	+1	8445	17.2%
TAAGGAATCTGCCTAA-AGGAGG	-1	971	2.0%
TAAGGAATCTGCCTAA--GGAGG	-2	914	1.9%
TAAGGA---------------GG	-15	605	1.2%
TAAGGAATCTGCCTAAC-GGAGG	-1	590	1.2%
TAAGGAATCTGCCTA-CAGGAGG	-1	519	1.1%
TAAGGAATCTGCCT---AGGAGG	-3	176	0.4%
TAAGGAATCTGCCTAAC--GAGG	-2	155	0.3%
TAAGGAATCTGCC----AGGAGG	-4	150	0.3%

24h (P71-BC9) 49.3%	Type	Reads	Percentage
TAAGGAATCTGCCTAAC|AGGAGG	WT		
TAAGGAATCTGCCTAACcAGGAGG	+1	8915	16.7%
TAAGGAATCTGCCTAA--GGAGG	-2	1817	3.4%
TAAGGA---------------GG	-15	1689	3.2%
TAAGGAATCTGCCTAA-AGGAGG	-1	1496	2.8%
TAAGGAATCTGCCTAAC-GGAGG	-1	865	1.6%
TAAGGAATCTGCCTA-CAGGAGG	-1	701	1.3%
TAAGGAATCTGCC----AGGAGG	-4	394	0.7%
TAAGGAATCTG--------GAGG	-8	266	0.5%
TAAGGAATCTGCCT---AGGAGG	-3	197	0.4%

48h (P71-BC10) 57.0%	Type	Reads	Percentage
TAAGGAATCTGCCTAAC|AGGAGG	WT		
TAAGGAATCTGCCTAACcAGGAGG	+1	6294	15.6%
TAAGGA---------------GG	-15	2490	6.2%
TAAGGAATCTGCCTAA--GGAGG	-2	1447	3.6%
TAAGGAATCTGCCTAA-AGGAGG	-1	1316	3.3%
TAAGGAATCTGCCTAAC-GGAGG	-1	663	1.6%
TAAGGAATCTGCCTA-CAGGAGG	-1	586	1.5%
TA------------------AGG	-18	270	0.7%
TAAGGAATCTG--------GAGG	-8	259	0.6%
TAAGGAATCTGCCT---AGGAGG	-3	259	0.6%

P72 Syn25-crAAVS1h scAAV6 KI

4h (P72-BC6) 29.9% 11.8%

Sequence	Type	Reads	Percentage
TAAGGAATCTGCCTAAC|AGGAGG	WT		
TAAGGAATCTGCCTAACcAGGAGG	+1	3973	12.2%
TAAGGAATCTGCCTAAGTTTAAACTACGCGTGGGTTA	HDR	3824	11.8%
TAAGGAATCTGCCTAA-AGGAGG	-1	273	0.8%
TAAGGAATCTGCCTAA--GGAGG	-2	231	0.7%
TAAGGAATCTGCCTAAC-GGAGG	-1	168	0.5%
TAAGGA---------------GG	-15	95	0.3%
TAAGGAATCTGCC----AGGAGG	-4	70	0.2%
TAAGGAATCTGCCTAAC----GG	-4	60	0.2%
TAAGGAATCTGCCT---AGGAGG	-3	43	0.1%

8h (P72-BC7) 49.7% 23.8%

Sequence	Type	Reads	Percentage
TAAGGAATCTGCCTAAC|AGGAGG	WT		
TAAGGAATCTGCCTAAGTTTAAACTACGCGTGGGTTA	HDR	8452	23.8%
TAAGGAATCTGCCTAACcAGGAGG	+1	3982	11.2%
TAAGGAATCTGCCTAA--GGAGG	-2	476	1.3%
TAAGGAATCTGCCTAA-AGGAGG	-1	476	1.3%
TAAGGAATCTGCCTAAC-GGAGG	-1	326	0.9%
TAAGGA---------------GG	-15	239	0.7%
TAAGGAATCTGCCTA-CAGGAGG	-1	171	0.5%
TAAGGAATCTGCC----AGGAGG	-4	101	0.3%
TAAGGAATCTGCCTAAC--GAGG	-2	92	0.3%

12h (P72-BC8) 58.2% 29.6%

Sequence	Type	Reads	Percentage
TAAGGAATCTGCCTAAC|AGGAGG	WT		
TAAGGAATCTGCCTAAGTTTAAACTACGCGTGGGTTA	HDR	10640	29.6%
TAAGGAATCTGCCTAACcAGGAGG	+1	4194	11.7%
TAAGGAATCTGCCTAA-AGGAGG	-1	690	1.9%
TAAGGAATCTGCCTAA--GGAGG	-2	350	1.0%
TAA---------------GGAGG	-15	284	0.8%
TAAGGAATCTGCCTAAC-GGAGG	-1	272	0.8%
TAAGGAATCTGCCTA-CAGGAGG	-1	263	0.7%
TAAGGAATCTGCC----AGGAGG	-4	127	0.4%
TAAGGAATCTGC-----AGGAGG	-5	110	0.3%

24h (P72-BC9) 70.2% 36.7%

Sequence	Type	Reads	Percentage
TAAGGAATCTGCCTAAC|AGGAGG	WT		
TAAGGAATCTGCCTAAGTTTAAACTACGCGTGGGTTA	HDR	11885	36.7%
TAAGGAATCTGCCTAACcAGGAGG	+1	4080	12.6%
TAAGGAATCTGCCTAA-AGGAGG	-1	611	1.9%
TAAGGAATCTGCCTAA--GGAGG	-2	442	1.4%
TAAGGAATCTGCCTAAC-GGAGG	-1	346	1.1%
TAAGGA---------------GG	-15	234	0.7%
TAAGGAATCTGCCTA-CAGGAGG	-1	204	0.6%
TAAGGAATCTGCC----AGGAGG	-4	153	0.5%

48h (P72-BC10) 76.9% 41.3%

Sequence	Type	Reads	Percentage
TAAGGAATCTGCCTAAC|AGGAGG	WT		
TAAGGAATCTGCCTAAGTTTAAACTACGCGTGGGTTA	HDR	9768	41.3%
TAAGGAATCTGCCTAACcAGGAGG	+1	3058	12.9%
TAAGGAATCTGCCTAA-AGGAGG	-1	532	2.2%
TAAGGAATCTGCCTAA--GGAGG	-2	432	1.8%
TAAGGA---------------GG	-15	253	1.1%
TAAGGAATCTGCCTA-CAGGAGG	-1	213	0.9%
TAAGGAATCTGCCTAAC-GGAGG	-1	204	0.9%
TAAGGAATCTGCCT---AGGAGG	-3	127	0.5%

P71 Syn29-crCD326f RNP KO

4h (P71-BC1) 14.9%	Type	Reads	Percentage
TGCGCGCGCGCCGAGAA | GAGGGG	WT		
TGCGCGCGCGCCGAGAAaGAGGGG	+1	1989	10.5%
TGCGCGCGCGCCGAGA-GAGGGG	-1	129	0.7%
TGCGCGCGCGCC-----GAGGGG	-5	33	0.2%
TGCGCGCGCGCCGAGAA-AGGGG	-1	31	0.2%
TGCGCGCGCGCCGAGAAgGAGGGG	+1	28	0.1%
TGCGCGCGCGCCGAGA---GGGG	-3	24	0.1%
TGCGCGCGCGCCGAG--GAGGGG	-2	19	0.1%
TGCGCGCGCGCCGAGA--AGGGG	-2	17	0.1%
T---------------------G	-21	17	0.1%

8h (P71-BC2) 23.0%	Type	Reads	Percentage
TGCGCGCGCGCCGAGAA | GAGGGG	WT		
TGCGCGCGCGCCGAGAAaGAGGGG	+1	3733	14.2%
TGCGCGCGCGCCGAGA-GAGGGG	-1	234	0.9%
TGCGCGCGCGCC-----GAGGGG	-5	145	0.6%
TGCGCGCGCGCCGAGA---GGGG	-3	108	0.4%
TGCGCGCGCGCCGAGAA-AGGGG	-1	85	0.3%
T---------------------G	-21	73	0.3%
TGCGCGCGCGCCGAGA--AGGGG	-2	57	0.2%
TGC-------------------G	-19	53	0.2%
TGCGCGCGCGCCGAGAAgGAGGGG	+1	44	0.2%

12h (P71-BC3) 27.6%	Type	Reads	Percentage
TGCGCGCGCGCCGAGAA | GAGGGG	WT		
TGCGCGCGCGCCGAGAAaGAGGGG	+1	13682	27.8%
TGCGCGCGCGCCGAGA-GAGGGG	-1	908	1.2%
TGCGCGCGCGCC-----GAGGGG	-5	623	0.8%
TGCGCGCGCGCCGAGA---GGGG	-3	514	0.7%
T---------------------G	-21	344	0.4%
TGCGCGCGCGCCGAGAA-AGGGG	-1	328	0.4%
TGCGCGCGCGCCGAGAAgGAGGGG	+1	153	0.3%
TGCGCGCGCGCCGAGA-------	-14	153	0.2%
TGCGCGCGCGCCGAGA--AGGGG	-2	136	0.2%

24h (P71-BC4) 41.7%	Type	Reads	Percentage
TGCGCGCGCGCCGAGAA | GAGGGG	WT		
TGCGCGCGCGCCGAGAAaGAGGGG	+1	8849	17.8%
TGCGCGCGCGCC-----GAGGGG	-5	914	1.8%
T---------------------G	-21	775	1.6%
TGCGCGCGCGCCGAGA---GGGG	-3	461	0.9%
T----------------------	-23	336	0.7%
TGC-------------------G	-19	310	0.6%
TGCGCGCGCGCCGAGAA-AGGGG	-1	235	0.5%
TGC--------------------	-32	214	0.4%
TG----------------AGGGG	-16	205	0.4%

48h (P71-BC5) 49.6%	Type	Reads	Percentage
TGCGCGCGCGCCGAGAA | GAGGGG	WT		
TGCGCGCGCGCCGAGAAaGAGGGG	+1	8160	18.0%
TGCGCGCGCGCC-----GAGGGG	-5	1265	2.8%
T---------------------G	-21	977	2.2%
TGCGCGCGCGCCGAGA---GGGG	-3	733	1.6%
TGCGCGCGCGCCGAGA-GAGGGG	-1	650	1.4%
T----------------------	-23	556	1.2%
TGC-------------------G	-19	408	0.9%
TGCGC-----------------G	-17	241	0.5%
TGC--------------------	-32	240	0.5%

P72 Syn29-crCD326f scAAV6 KI

4h (P72-BC1) 16.7% 1.2%

Sequence	Type	Reads	Percentage
TGCGCGCGCGCCGAGAA|GAGGGG	WT		
TGCGCGCGCGCCGAGAAaGAGGGG	+1	6847	10.7%
TGCGCGCGCGCCGAGGTTTAAACTAC GCGTGCGGG	HDR	777	1.2%
TGCGCGCGCGCCGAGA-GAGGGG	-1	456	0.7%
TGCGCGCGCGCCGAGA---GGGG	-3	107	0.2%
TGCGCGCGCGCCGAGAA-AGGGG	-1	84	0.1%
TGCGCGCGCGCCGAGAAgGAGGGG	+1	77	0.1%
T-----------------------	-23	55	0.1%

8h (P72-BC2) 26.7% 5.5%

Sequence	Type	Reads	Percentage
TGCGCGCGCGCCGAGAA|GAGGGG	WT		
TGCGCGCGCGCCGAGAAaGAGGGG	+1	6900	13.2%
TGCGCGCGCGCCGAGGTTTAAACTA CGCGTGCGGG	HDR	2876	5.5%
TGCGCGCGCGCCGAGA-GAGGGG	-1	444	0.8%
TGCGCGCGCGCCGAGA---GGGG	-3	157	0.3%
TGCGCGCGCGCCGAGAA-AGGGG	-1	144	0.3%
T---------------------G	-21	92	0.2%
TGCGCGCGCGCC-----GAGGGG	-5	75	0.1%
TGCGCGCGCGCCGAGAAgGAGGGG	+1	70	0.1%
TGCGCGCGCGCCGAGA[illegible]AGGGG	-2	68	0.1%

12h (P72-BC3) 31.1% 6.8%

Sequence	Type	Reads	Percentage
TGCGCGCGCGCCGAGAA|GAGGGG	WT		
TGCGCGCGCGCCGAGAAaGAGGGG	+1	9979	13.8%
TGCGCGCGCGCCGAGGTTTAAACTAC GCGTGCGGG	HDR	4903	6.8%
TGCGCGCGCGCCGAGA-GAGGGG	-1	610	0.8%
TGCGCGCGCGCCGAGA---GGGG	-3	423	0.6%
T---------------------G	-21	220	0.3%
TGCGCGCGCGCCGAGAA-AGGGG	-1	216	0.3%
TGCGCGCGCGCCGAGAAgGAGGGG	+1	146	0.2%
TGC-------------------G	-19	106	0.1%
TGCGC-----------------G	-17	105	0.1%

24h (P72-BC4) 43.0% 12.3%

Sequence	Type	Reads	Percentage
TGCGCGCGCGCCGAGAA|GAGGGG	WT		
TGCGCGCGCGCCGAGAAaGAGGGG	+1	8757	16.7%
TGCGCGCGCGCCGAGGTTTAAACTAC GCGTGCGGG	HDR	6482	12.3%
TGCGCGCGCGCCGAGA-GAGGGG	-1	764	1.5%
TGCGCGCGCGCCGAGA---GGGG	-3	346	0.7%
TGCGCGCGCGCCGAGAA-AGGGG	-1	204	0.4%
TGCGCGCGCGCC-----GAGGGG	-5	193	0.4%
TGCGCGCGCGCCGAGAAgGAGGGG	+1	152	0.3%
TGCGC-----------------G	-17	148	0.3%

48h (P72-BC5) 55.1% 17.5%

Sequence	Type	Reads	Percentage
TGCGCGCGCGCCGAGAA|GAGGGG	WT		
TGCGCGCGCGCCGAGAAaGAGGGG	+1	8854	19.4%
TGCGCGCGCGCCGAGGTTTAAACTA CGCGTGCGGG	HDR	7974	17.5%
TGCGCGCGCGCCGAGA-GAGGGG	-1	819	1.8%
TGCGCGCGCGCCGAGA---GGGG	-3	445	1.0%
TGCGCGCGCGCC-----GAGGGG	-5	397	0.9%
T---------------------G	-21	315	0.7%
TGCGCGCGCGCCGAGAA-AGGGG	-1	241	0.5%
TGCGC-----------------G	-17	168	0.4%

P71 Syn31-crCD326h RNP KO

4h (P71-BC6) 6.2%

Sequence	Type	Reads	Percentage
GCTGCGCGCGCGCCGAG|AAGAGG	WT		
GCTGCGCGCGCGCCGAGgAAGAGG	+1	1371	2.9%
GCTGCGCGCGCGCCGAGaAAGAGG	+1	333	0.7%
GCTGCGCGCGCGCCGA--AGAGG	-2	117	0.2%
GCTGCGCGCGCGCCGAGA-GAGG	-1	79	0.2%
GCT---------------------	-21	57	0.1%
GCTGCGCGCGCGCC-----GAGG	-5	57	0.1%
GCTGCGCGCGCGCCGA-AAGAGG	-1	50	0.1%
GCTGCGCGCGCGCCGAGagAAGAGG	+2	44	0.1%

8h (P71-BC7) 10.9%

Sequence	Type	Reads	Percentage
GCTGCGCGCGCGCCGAG|AAGAGG	WT		
GCTGCGCGCGCGCCGAGgAAGAGG	+1	2661	4.7%
GCTGCGCGCGCGCCGAGaAAGAGG	+1	286	0.5%
GCTGCGCGCGCGCCGA--AGAGG	-2	198	0.4%
GCTGCGCGCGCGCC-----GAGG	-5	192	0.3%
GCT---------------------	-21	150	0.3%
GCTGCGCGCGCGCCGAGA-GAGG	-1	148	0.3%
GCTGCGCGCGCGCCGA-AAGAGG	-1	145	0.3%
GCT--------------------	-23	68	0.1%
GCTGCGCG---------AAGAGG	-9	60	0.1%

12h (P71-BC8) 13.2%

Sequence	Type	Reads	Percentage
GCTGCGCGCGCGCCGAG|AAGAGG	WT		
GCTGCGCGCGCGCCGAGgAAGAGG	+1	4086	4.7%
GCTGCGCGCGCGCCGAGaAAGAGG	+1	403	0.5%
GCTGCGCGCGCGCCGA-AAGAGG	-1	402	0.5%
GCT---------------------	-21	382	0.4%
GCTGCGCGCGCGCCGA--AGAGG	-2	371	0.4%
GCTGCGCGCGCGCC-----GAGG	-5	312	0.4%
GCT--------------------	-23	233	0.3%
GCTGCGC-----------------	-17	211	0.2%
GCTGCGCGCGCGCCGAGA-GAGG	-1	182	0.2%

24h (P71-BC9) 21.4%

Sequence	Type	Reads	Percentage
GCTGCGCGCGCGCCGAG|AAGAGG	WT		
GCTGCGCGCGCGCCGAGgAAGAGG	+1	3786	4.9%
GCT---------------------	-21	1374	1.8%
GCT--------------------	-23	770	1.0%
GCTGCGCGCGCGCC-----GAGG	-5	676	0.9%
GCTGCGCGCGCGCCGA--AGAGG	-2	548	0.7%
GCTGCGCGCGCGCCGA-AAGAGG	-1	477	0.6%
------------------------GC	-24	343	0.4%
GCTGC-------------------	-19	328	0.4%
GCTGCGCGCGCGCCGAGaAAGAGG	+1	310	0.4%

48h (P71-BC10) 30.7%

Sequence	Type	Reads	Percentage
GCTGCGCGCGCGCCGAG|AAGAGG	WT		
GCTGCGCGCGCGCCGAGgAAGAGG	+1	3123	4.5%
GCT---------------------	-21	1889	2.7%
GCT--------------------	-23	1286	1.9%
GCTGCGCGCGCGCC-----GAGG	-5	1024	1.5%
GCTGC-------------------	-19	826	1.2%
-----------------------	-26	653	0.9%
GCTGCGCGCGCGCCGA--AGAGG	-2	532	0.8%
------------------------ GC	-24	459	0.7%
GCTGCGC-----------------	-17	420	0.6%

P72 Syn31-crCD326h scAAV6 KI

4h (P72-BC6) 6.6% 0.8%	Type	Reads	Percentage
GCTGCGCGCGCGCCGAG|AAGAGG	WT		
GCTGCGCGCGCGCCGAGgAAGAGG	+1	2422	3.1%
GCTGCGCGCGCGCCGAGGTTTAAACT ACGCGTGCGGGAGGGG	HDR	619	0.8%
GCTGCGCGCGCGCCGAGaAAGAGG	+1	250	0.3%
GCTGCGCGCGCGCCGA-AAGAGG	-1	148	0.2%
GCTGCGCGCGCGCC-----GAGG	-5	138	0.2%
GCTGCGCGCGCGCCGA--AGAGG	-2	128	0.2%
GCTGCGCGCGCGCCGAGA-GAGG	-1	65	0.1%
GCTGCGC[illegible]	-17	52	0.1%
GCTGC[illegible]CGAGA---GG	-3	49	0.1%

8h (P72-BC7) 13.6% 4.4%	Type	Reads	Percentage
GCTGCGCGCGCGCCGAG|AAGAGG	WT		
GCTGCGCGCGCGCCGAGGTTTAAAC TACGCGTGCGGGAGGGG	HDR	4093	4.4%
GCTGCGCGCGCGCCGAGgAAGAGG	+1	3475	3.8%
GCT----------------------	-21	285	0.3%
GCTGCGCGCGCGCCGA-AAGAGG	-1	251	0.3%
GCTGCGCGCGCGCCGAGaAAGAGG	+1	236	0.3%
GCTGCGCGCGCGCCGA--AGAGG	-2	220	0.2%
GCTGCGCGCGCGCCGA--------G	-8	147	0.2%
GCTGCGCGCGCGCCGAGagAAGAGG	+2	128	0.1%
GCTGC[illegible]	-19	126	0.1%

12h (P72-BC8) 17.6% 6.1%	Type	Reads	Percentage
GCTGCGCGCGCGCCGAG|AAGAGG	WT		
GCTGCGCGCGCGCCGAGGTTTAAACT ACGCGTGCGGGAGGGG	HDR	5140	6.1%
GCTGCGCGCGCGCCGAGgAAGAGG	+1	3780	4.5%
GCT----------------------	-21	399	0.5%
GCTGCGCGCGCGCCGAGaAAGAGG	+1	299	0.4%
GCTGCGCGCGCGCCGA--AGAGG	-2	249	0.3%
GCTGCGCGCGCGCCGA-AAGAGG	-1	237	0.3%
GCTGCGCGCGCGCCGAGA-GAGG	-1	168	0.2%
GCTGCGCGCGCGCCGAGagAAGAGG	+2	154	0.2%
GCT--------------------	-23	138	0.2%

24h (P72-BC9) 23.5% 10.4%	Type	Reads	Percentage
GCTGCGCGCGCGCCGAG|AAGAGG	WT		
GCTGCGCGCGCGCCGAGGTTTAAACT ACGCGTGCGGGAGGGG	HDR	8177	10.4%
GCTGCGCGCGCGCCGAGgAAGAGG	+1	3101	4.0%
GCTGCGCGCGCGCCGA-AAGAGG	-1	633	0.8%
GCT----------------------	-21	427	0.5%
GCTGC--------------------	-19	278	0.4%
GCTGCGCGCGCGCCGAGaAAGAGG	+1	266	0.3%
GCT--------------------	-23	260	0.3%
GCTGCGCGCGCGCCGA--AGAGG	-2	251	0.3%

48h (P72-BC10) 34.4% 15.3%	Type	Reads	Percentage
GCTGCGCGCGCGCCGAG|AAGAGG	WT		
GCTGCGCGCGCGCCGAGGTTTAAAC TACGCGTGCGGGAGGGG	HDR	7857	15.3%
GCTGCGCGCGCGCCGAGgAAGAGG	+1	2484	4.8%
GCT----------------------	-21	482	0.9%
GCTGCGCGCGCGCCGA--AGAGG	-2	438	0.9%
GCT--------------------	-23	304	0.6%
GCTGCGCGCGCGCCGA-AAGAGG	-1	289	0.6%
GCTGCGC------------------	-17	256	0.5%
GCTGCGCGCGCGCC-----GAGG	-5	241	0.5%

P72 Syn52-crGATA4b scAAV6 KI

4h (P72-BC5) 7.9%

2.1%

Sequence	Type	Reads	Percentage
TCAAATTCCTGCACGGA|CCTGGG	WT		
TCACGCGTAGTTTAAACACTTGGAG	HDR	904	2.1%
TCAAATTCCTGCACGGAaCCTGGG	+1	402	0.9%
TCAAATT----------CCTGGG	-10	173	0.4%
TCAAATTCCTGCAC-------GGAC	-7	108	0.3%
TCAAATTCCTGCACGGA--TGGG	-2	106	0.2%
TCAAATTCCTGCACGG-CCTGGG	-1	81	0.2%
TCAAATTCCTGCACGGA---GGG	-3	65	0.2%
TCAAATTCCTGCACGGAtCCTGGG	+1	62	0.1%
TCAAATTCCTGCACGGAcCCTGGG	+1	55	0.1%
T[illegible]G--CCTGGG	-2	48	0.1%
T[illegible]CTGCACGGA------	-6	44	0.1%

8h (P72-BC1) 15.8%

4.7%

Sequence	Type	Reads	Percentage
TCAAATTCCTGCACGGA|CCTGGG	WT		
TCACGCGTAGTTTAAACACTTGGAG	HDR	1064	4.7%
TCAAATTCCTGCACGGAaCCTGGG	+1	322	1.4%
TCAAATT----------CCTGGG	-10	267	1.2%
TCAAATTCCTGCACGGA-CTGGG	-1	183	0.8%
TCAAATTCCTGCAC-------GGAC	-7	123	0.5%
TCAAATTCCTGCACGG-CCTGGG	-1	85	0.4%
TCAAATTCCTGCACGGA----------	-13	73	0.3%
TCAAATTCCTGC------CTGGG	-6	68	0.3%
TCAAATTCCTGCACGGA--TGGG	-2	58	0.3%
TCAAATTCCTGCACGGAcCCTGGG	+1	33	0.1%
TCAAATTCCTGCACGGA---GGG	-3	33	0.1%
TCAAATTCCTGCAC----CTGGG	-4	32	0.1%

12h (P72-BC2) 20.2%

6.0%

Sequence	Type	Reads	Percentage
TCAAATTCCTGCACGGA|CCTGGG	WT		
TCACGCGTAGTTTAAACACTTGGAG	HDR	1203	6.0%
TCAAATTCCTGCACGGAaCCTGGG	+1	378	1.9%
TCAAATT----------CCTGGG	-10	344	1.7%
TCAAATTCCTGCACGGA-CTGGG	-1	193	1.0%
TCAAATTCCTGCAC-------GGAC	-7	167	0.8%
TCAAATTCCTGCACGGA----------	-13	86	0.4%
TCAAATTCCTGCACGG-CCTGGG	-1	75	0.4%
TCAAATTCCTGCACGGA---GGG	-3	62	0.3%
TCAAATTCCTGC------CTGGG	-6	61	0.3%
TCAAATTCCTGCACGGA--TGGG	-2	58	0.3%
TCAAATTCCTGCACGGAtCCTGGG	+1	49	0.2%
TCAAATTCCTGCACGGAgCCTGGG	+1	40	0.2%

24h (P72-BC3) 30.4%

13.2%

Sequence	Type	Reads	Percentage
TCAAATTCCTGCACGGA|CCTGGG	WT		
TCACGCGTAGTTTAAACACTTGGAG	HDR	4801	13.2%
TCAAATT----------CCTGGG	-10	768	2.1%
TCAAATTCCTGCACGGAaCCTGGG	+1	604	1.7%
TCAAATTCCTGCACGGA-CTGGG	-1	343	0.9%
TCAAATTCCTGCAC-------GGAC	-7	312	0.9%
TCAAATTCCTGCACGGA----------	-13	198	0.5%
TCAAATTCCTGC------CTGGG	-6	175	0.5%
TCAAATTCCTGCACGG-CCTGGG	-1	105	0.3%
TCAAATTCCTGCACGGA[illegible]CCTGGG	+1	85	[illegible]

48h (P72-BC4) 33.5%

16.4%

Sequence	Type	Reads	Percentage
TCAAATTCCTGCACGGA|CCTGGG	WT		
TCACGCGTAGTTTAAACACTTGGAG	HDR	3672	16.4%
TCAAATT----------CCTGGG	-10	458	2.0%
TCAAATTCCTGCACGGAaCCTGGG	+1	439	2.0%
TCAAATTCCTGCAC-------GGAC	-7	217	1.0%
TCAAATTCCTGCACGGA-CTGGG	-1	189	0.8%
TCAAATTCCTGCACGGA----------	-13	140	0.6%
TCAAATTCCTGCACGGA--TGGG	-2	102	0.5%
TCAAATTCC---------CTGGG	-9	82	0.4%

P71 Syn54-crGATA4d RNP KO

<table>
<tr><th>4h (P71-BC6) 22.3%</th><th>Type</th><th>Reads</th><th>Percentage</th></tr>
<tr><td>TCCCTCCTCAAATTCCT|GCACGG</td><td>WT</td><td></td><td></td></tr>
<tr><td>TCCCTCCTCAAATTCCTtGCACGG</td><td>+1</td><td>6193</td><td>14.9%</td></tr>
<tr><td>TCCCTCCTCAAATTCCTctGCACGG</td><td>+2</td><td>663</td><td>1.6%</td></tr>
<tr><td>TCCCTCCTCAAATTCCTG------</td><td>-10</td><td>174</td><td>0.4%</td></tr>
<tr><td>TCCCTCCTCAAATTCCcTGCACGG</td><td>+1</td><td>110</td><td>0.3%</td></tr>
<tr><td>TCCCTCCTCAAATTC-TGCACGG</td><td>-1</td><td>86</td><td>0.2%</td></tr>
<tr><td>TCCCTCCTCAAATTCC-GCACGG</td><td>-1</td><td>57</td><td>0.1%</td></tr>
<tr><td>TCCCTCCTCAAATTCCTcGCACGG</td><td>+1</td><td>43</td><td>0.1%</td></tr>
</table>

<table>
<tr><th>8h (P71-BC7) 33.0%</th><th>Type</th><th>Reads</th><th>Percentage</th></tr>
<tr><td>TCCCTCCTCAAATTCCT|GCACGG</td><td>WT</td><td></td><td></td></tr>
<tr><td>TCCCTCCTCAAATTCCTtGCACGG</td><td>+1</td><td>9348</td><td>20.7%</td></tr>
<tr><td>TCCCTCCTCAAATTCCTctGCACGG</td><td>+2</td><td>1133</td><td>2.5%</td></tr>
<tr><td>TCCCTCCTCAAATTCCTG------</td><td>-10</td><td>247</td><td>0.5%</td></tr>
<tr><td>TCCCTCCTCAAATTC-TGCACGG</td><td>-1</td><td>172</td><td>0.4%</td></tr>
<tr><td>TCCCTCCTCAAATTCCcTGCACGG</td><td>+1</td><td>111</td><td>0.2%</td></tr>
<tr><td>TCCCTCCTCAAATTCC-GCACGG</td><td>-1</td><td>96</td><td>0.2%</td></tr>
<tr><td>TCCCTCCTCAAAT----GCACGG</td><td>-4</td><td>78</td><td>0.2%</td></tr>
<tr><td>TCCCTCCTCAAATTCCTttGCACGG</td><td>+2</td><td>53</td><td>0.1%</td></tr>
</table>

<table>
<tr><th>12h (P71-BC8) 36.8%</th><th>Type</th><th>Reads</th><th>Percentage</th></tr>
<tr><td>TCCCTCCTCAAATTCCT|GCACGG</td><td>WT</td><td></td><td></td></tr>
<tr><td>TCCCTCCTCAAATTCCTtGCACGG</td><td>+1</td><td>1614</td><td>25.2%</td></tr>
<tr><td>TCCCTCCTCAAATTCCTctGCACGG</td><td>+2</td><td>156</td><td>2.4%</td></tr>
<tr><td>TCCCTCCTCAAATTCCTG------</td><td>-10</td><td>68</td><td>1.1%</td></tr>
<tr><td>TCCCTCCTCAAATTC-TGCACGG</td><td>-1</td><td>47</td><td>0.7%</td></tr>
<tr><td>TCCCTCCTCAAATTCCcTGCACGG</td><td>+1</td><td>22</td><td>0.3%</td></tr>
<tr><td>TCCCTCCTCAAATTCC-GCACGG</td><td>-1</td><td>16</td><td>0.2%</td></tr>
<tr><td>TCCCTCCTCAAATT--TGCACGG</td><td>-2</td><td>12</td><td>0.2%</td></tr>
<tr><td>TCCCTCCTCA----------CGG</td><td>-10</td><td>11</td><td>0.2%</td></tr>
</table>

<table>
<tr><th>24h (P71-BC9) 46.0%</th><th>Type</th><th>Reads</th><th>Percentage</th></tr>
<tr><td>TCCCTCCTCAAATTCCT|GCACGG</td><td>WT</td><td></td><td></td></tr>
<tr><td>TCCCTCCTCAAATTCCTtGCACGG</td><td>+1</td><td>21301</td><td>25.9%</td></tr>
<tr><td>TCCCTCCTCAAATTCCTctGCACGG</td><td>+2</td><td>1901</td><td>2.3%</td></tr>
<tr><td>TCCCTCCTCAAATTCCTG------</td><td>-10</td><td>1133</td><td>1.4%</td></tr>
<tr><td>TCCCTCCTCAAATTC-TGCACGG</td><td>-1</td><td>462</td><td>0.6%</td></tr>
<tr><td>TCCCTCCTCAAATT---GCACGG</td><td>-3</td><td>337</td><td>0.4%</td></tr>
<tr><td>TCCCTCCTCAAATTCCcTGCACGG</td><td>+1</td><td>247</td><td>0.3%</td></tr>
<tr><td>TCCCT------------------</td><td>-22</td><td>244</td><td>0.3%</td></tr>
<tr><td>TCCCTCCTCAAATT--TGCACGG</td><td>-2</td><td>232</td><td>0.3%</td></tr>
<tr><td>TCCCTCCTCAAAT----GCACGG</td><td>-4</td><td>189</td><td>0.2%</td></tr>
<tr><td>TCCCTCCTCAAATTCC---ACGG</td><td>-3</td><td>168</td><td>0.2%</td></tr>
</table>

<table>
<tr><th>48h (P71-BC10) 49.2%</th><th>Type</th><th>Reads</th><th>Percentage</th></tr>
<tr><td>TCCCTCCTCAAATTCCT|GCACGG</td><td>WT</td><td></td><td></td></tr>
<tr><td>TCCCTCCTCAAATTCCTtGCACGG</td><td>+1</td><td>11494</td><td>22.6%</td></tr>
<tr><td>TCCCTCCTCAAATTCCTG------</td><td>-10</td><td>1138</td><td>2.2%</td></tr>
<tr><td>TCCCTCCTCAAATTCCTctGCACGG</td><td>+2</td><td>959</td><td>1.9%</td></tr>
<tr><td>TCCCTCCTCAAATTC-TGCACGG</td><td>-1</td><td>303</td><td>0.6%</td></tr>
<tr><td>TCCCTCCTCAAATTCC---ACGG</td><td>-3</td><td>229</td><td>0.5%</td></tr>
<tr><td>TCCCTCCTCAAATTC----ACGG</td><td>-4</td><td>190</td><td>0.4%</td></tr>
<tr><td>TCCCTCCT---------------</td><td>-19</td><td>178</td><td>0.4%</td></tr>
<tr><td>TCCCT------------------</td><td>-22</td><td>165</td><td>0.3%</td></tr>
<tr><td>TCCCTCCTCAAATT--TGCACGG</td><td>-2</td><td>153</td><td>0.3%</td></tr>
<tr><td>TCCCTCCTCAAATTCC-GCACGG</td><td>-1</td><td>147</td><td>0.3%</td></tr>
</table>

P72 Syn54-crGATA4d scAAV6 KI

4h (P72-BC6) 22.7% 2.2%

	Type	Reads	Percentage
TCCCTCCTCAAATTCCT|GCACGG	WT		
TCCCTCCTCAAATTCCTtGCACGG	+1	7681	12.8%
TCCCTCACGCGTAGTTTAAACACTT	HDR	1344	2.2%
TCCCTCCTCAAATTCCTctGCACGG	+2	1116	1.9%
TCCCTCCTCAAATTCCTG------	-10	308	0.5%
TCCCTCCTCAAATTCCcTGCACGG	+1	125	0.2%
TCCCTCCTCAAATTC-TGCACGG	-1	120	0.2%
TCCCTCCTCAAATT---GCACGG	-3	74	0.1%
TCCCTCCTCAAATTCC-GCACGG	-1	70	0.1%

8h (P72-BC7) 34.5% 3.6%

	Type	Reads	Percentage
TCCCTCCTCAAATTCCT|GCACGG	WT		
TCCCTCCTCAAATTCCTtGCACGG	+1	13064	18.7%
TCCCTCACGCGTAGTTTAAACACTT	HDR	2493	3.6%
TCCCTCCTCAAATTCCTctGCACGG	+2	1556	2.2%
TCCCTCCTCAAATTCCTG------	-10	497	0.7%
TCCCTCCTCAAATTC-TGCACGG	-1	233	0.3%
TCCCTCCTCAAATT---GCACGG	-3	172	0.2%
TCCCTCCTCAAATTCCcTGCACGG	+1	150	0.2%
TCCCTCCTCAAATTCCTcGCACGG	+1	97	0.1%
TCCCTCCTCAAAT----GCACGG	-4	85	0.1%
TCCCTCCTCAAATTCC-GCACGG	-1	84	0.1%

12h (P72-BC8) 36.7% 4.2%

	Type	Reads	Percentage
TCCCTCCTCAAATTCCT|GCACGG	WT		
TCCCTCCTCAAATTCCTtGCACGG	+1	1531	21.9%
TCCCTCACGCGTAGTTTAAACACTT	HDR	295	4.2%
TCCCTCCTCAAATTCCTctGCACGG	+2	163	2.3%
TCCCTCCTCAAATTCCTG------	-10	71	1.0%
TCCCTCCTCAAATTC-TGCACGG	-1	28	0.4%
TCCCTCCTCAAATTCCTttGCACGG	+2	15	0.2%
TCCCTCCTCAAATT---GCACGG	-3	13	0.2%
TCCCTCCTCAAATTCCcTGCACGG	+1	13	0.2%
TCCCTCCTCA----------CGG	-10	11	0.2%
TCCCTCCTCAAATTCC-GCACGG	-1	11	0.2%

24h (P72-BC9) 48.5% 9.0%

	Type	Reads	Percentage
TCCCTCCTCAAATTCCT|GCACGG	WT		
TCCCTCCTCAAATTCCTtGCACGG	+1	13081	23.5%
TCCCTCACGCGTAGTTTAAACACTT	HDR	5026	9.0%
TCCCTCCTCAAATTCCTctGCACGG	+2	1294	2.3%
TCCCTCCTCAAATTCCTG------	-10	555	1.0%
TCCCTCCTCAAATTC-TGCACGG	-1	289	0.5%
TCCCTCCTCAAATT---GCACGG	-3	200	0.4%
TCCCTCCTCAAATTCCcTGCACGG	+1	176	0.3%

48h (P72-BC10) 55.1% 14.3%

	Type	Reads	Percentage
TCCCTCCTCAAATTCCT|GCACGG	WT		
TCCCTCCTCAAATTCCTtGCACGG	+1	8060	22.7%
TCCCTCACGCGTAGTTTAAACACTT	HDR	5056	14.3%
TCCCTCCTCAAATTCCTctGCACGG	+2	736	2.1%
TCCCTCCTCAAATTCCTG------	-10	442	1.2%
TCCCTCCTCAAATTC-TGCACGG	-1	167	0.5%
TCCCTCCTCAAATT---GCACGG	-3	148	0.4%
TCCCTCCT-------------------	-19	119	0.3%
TCCCTCCTCAAATTCC-GCACGG	-1	111	0.3%

P71 Syn60-crMYH6b RNP KO

4h (P71-BC1) 5.2%	Type	Reads	Percentage
CAGTAGGGGGCCTGAGA|GGAGGG	WT		
CAGTAGGGGGCCTGAGAaGGAGGG	+1	996	2.8%
CAGTAGGGGGCCTGAG-GGAGGG	-1	148	0.4%
CAGTAGGGGGCCTGAGA-GAGGG	-1	108	0.3%
CAGTAGGGGGCCTGA--GGAGGG	-2	56	0.2%
CAGTAGGGGGCCTGA---GAGGG	-3	51	0.1%
CAGTAGGG----------------GGCC	-16	28	0.1%

8h (P71-BC2) 10.3%	Type	Reads	Percentage
CAGTAGGGGGCCTGAGA|GGAGGG	WT		
CAGTAGGGGGCCTGAGAaGGAGGG	+1	1581	5.1%
CAGTAGGGGGCCTGAG-GGAGGG	-1	176	0.6%
CAGTAGGGGGCCTGAGA-GAGGG	-1	109	0.3%
CAGTAGGGGGCCTGA--GGAGGG	-2	91	0.3%
CAGTAGGGGGCCTGA---GAGGG	-3	89	0.3%
CAGTAGGGGGCCT-----GAGGG	-5	40	0.1%
CAGTAGGGGGCCTGAGA-----G	-5	39	0.1%
CAGTAGG------------AGGG	-12	38	0.1%
CAGTAGGG----------------GGCC	-16	34	0.1%

12h (P71-BC3) 13.2%	Type	Reads	Percentage
CAGTAGGGGGCCTGAGA|GGAGGG	WT		
CAGTAGGGGGCCTGAGAaGGAGGG	+1	5096	5.6%
CAGTAGGGGGCCTGAG-GGAGGG	-1	736	0.8%
CAGTAGGGGGCCTGAGA-GAGGG	-1	467	0.5%
CAGTAGGGGGCCTGA--GGAGGG	-2	330	0.4%
CAGTAGGGGGCCTGA---GAGGG	-3	262	0.3%
CAGTAGGG----------------GGCC	-16	206	0.2%
CAGTAGG------------AGGG	-12	187	0.2%
CAGTAGGGGGCCT-----GAGGG	-5	159	0.2%
CAGTAGGGGGCCTGAGA-----G	-5	144	0.2%
CAGTAGGGGGCCTGAGAtGGAGGG	+1	132	0.1%

24h (P71-BC4) 19.5%	Type	Reads	Percentage
CAGTAGGGGGCCTGAGA|GGAGGG	WT		
CAGTAGGGGGCCTGAGAaGGAGGG	+1	4737	7.0%
CAGTAGGGGGCCTGAG-GGAGGG	-1	959	1.4%
CAGTAGGGGGCCTGA--GGAGGG	-2	469	0.7%
CAGTAGGG----------------GGCC	-16	348	0.5%
GAATCTGCCTAACAGGA-GTGGG	-1	325	0.5%
CAGTAGG------------AGGG	-12	278	0.4%
CAGTAGGGGGCCTGA---GAGGG	-3	217	0.3%
CAGT---------------AGGG	-15	193	0.3%
C---------------AGGAGGG	-15	177	0.3%
CAGTAGGGGGCCT-----GAGGG	-5	137	0.2%

48h (P71-BC5) 26.2%	Type	Reads	Percentage
CAGTAGGGGGCCTGAGA|GGAGGG	WT		
CAGTAGGGGGCCTGAGAaGGAGGG	+1	6541	8.0%
CAGTAGGGGGCCTGAG-GGAGGG	-1	1600	2.0%
CAGTAGGG----------------GGCC	-16	828	1.0%
CAGTAGGGGGCCTGA--GGAGGG	-2	771	0.9%
CAGT---------------AGGG	-15	651	0.8%
GAATCTGCCTAACAGGA-GTGGG	-1	532	0.7%
CAGTAGGGGGCCTGA---GAGGG	-3	499	0.6%
CAGTAGG------------AGGG	-12	483	0.6%
CAGTAGGGGGCCT-----GAGGG	-5	431	0.5%
C---------------AGGAGGG	-15	331	0.4%

P72 Syn60-crMYH6b scAAV6 KI

4h (P72-BC1) 4.3%

0.8%

	Type	Reads	Percentage
CAGTAGGGGGCCTGAGA|GGAGGG	WT		
CAGTAGGGGGCCTGAGAaGGAGGG	+1	435	1.5%
CAGTAGGGGGCCTGAGAGACGCGTAG<br>TTTAAACCAGAGC	HDR	245	0.8%
GAATCTGCCTAACAGGA-GTGGG	-1	61	0.2%
CAGTAGGGGGCCTGAG-GGAGGG	-1	47	0.2%
CAGTAGGGGGCCTGA--GGAGGG	-2	43	0.1%
CAGTAGGGGGCCTGA---GAGGG	-3	16	0.1%
CAGTAGGGGGCCTGA-------GAG	-7	14	0.0%

8h (P72-BC2) 8.8%

1.9%

	Type	Reads	Percentage
CAGTAGGGGGCCTGAGA|GGAGGG	WT		
CAGTAGGGGGCCTGAGAaGGAGGG	+1	690	2.6%
CAGTAGGGGGCCTGAGAGACGCGTAGT<br>TTAAACCAGAGC	HDR	505	1.9%
CAGTAGGGGGCCTGAG-GGAGGG	-1	93	0.3%
GAATCTGCCTAACAGGA-GTGGG	-1	92	0.3%
CAGTAGGGGGCCTGA--GGAGGG	-2	46	0.2%
CAGTAGGGGGCCTGAGA--------G	-8	23	0.1%
CAGTAGGGGGCCTGA---GAGGG	-3	22	0.1%
CAGTAGGGGGCCTGA-------GAG	-7	21	0.1%
CAGTAGG------------AGGG	-12	21	0.1%
[illegible]	-2	19	0.1%

12h (P72-BC3) 12.0%

2.9%

	Type	Reads	Percentage
CAGTAGGGGGCCTGAGA|GGAGGG	WT		
CAGTAGGGGGCCTGAGAaGGAGGG	+1	1590	3.5%
CAGTAGGGGGCCTGAGAGACGCGTAGT<br>TTAAACCAGAGC	HDR	1301	2.9%
CAGTAGGGGGCCTGAG-GGAGGG	-1	133	0.3%
GAATCTGCCTAACAGGA-GTGGG	-1	113	0.3%
CAGTAGGGGGCCTGA--GGAGGG	-2	76	0.2%
CAGTAGGG----------------<br>GGCC	-16	56	0.1%
C---------------AGGAGGG	-15	43	0.1%
CAGTAGGGGGCCTGAGA-----G	-5	39	0.1%
CAGTAGG------------AGGG	-12	39	0.1%
CAGTAGGGGGCCTGAGAtGGAGGG	+1	38	0.1%
CAGT-------------------AGG	-19	27	0.1%

24h (P72-BC4) 19.6%

5.9%

	Type	Reads	Percentage
CAGTAGGGGGCCTGAGA|GGAGGG	WT		
CAGTAGGGGGCCTGAGAaGGAGGG	+1	1733	6.2%
CAGTAGGGGGCCTGAGAGACGCGTAGT<br>TTAAACCAGAGC	HDR	1635	5.9%
CAGTAGGGGGCCTGAG-GGAGGG	-1	176	0.6%
CAGTAGGGGGCCTGA--GGAGGG	-2	96	0.3%
GAATCTGCCTAACAGGA-GTGGG	-1	63	0.2%
CAGTAGG------------AGGG	-12	62	0.2%
CAGTAGGG----------------<br>GGCC	-16	51	0.2%
CAGTAGGGGGCCTGAGAtGGAGGG	+1	51	0.2%
CAGTAGGGGGCCTGAGA--------G	-8	50	0.2%

48h (P72-BC5) 30.8%

12.1%

	Type	Reads	Percentage
CAGTAGGGGGCCTGAGA|GGAGGG	WT		
CAGTAGGGGGCCTGAGAGACGCGTAG<br>TTTAAACCAGAGC	HDR	3955	12.1%
CAGTAGGGGGCCTGAGAaGGAGGG	+1	1633	5.0%
CAGTAGGGGGCCTGAG-GGAGGG	-1	395	1.2%
CAGTAGG------------AGGG	-12	155	0.5%
CAGTAGGGGGCCTGA--GGAGGG	-2	137	0.4%
CAGTAGGGGGCCTGAGAtGGAGGG	+1	99	0.3%
CAGTAGGGGGCCTGAGA-----G	-5	76	0.2%
GAATCTGCCTAACAGGA-GTGGG	-1	74	0.2%
[illegible]	-8	70	[illegible]

P71 Syn61-crMYH6c RNP KO

4h (P71-BC11) 9.2%

Sequence	Type	Reads	Percentage
AGCTCTGATACAGGGGC|TGGGGG	WT		
AGCTCTGATACAGGGGCcTGGGGG	+1	2569	5.6%
AGCTCTGATACAGGGGC-GGGGG	-1	381	0.8%
AGCTCTGATACAGGGGCT-GGGG	-1	231	0.5%
AGCTCTGATACA-------GGGGC	-7	78	0.2%
AGCTCTGATACAGGGG-TGGGGG	-1	59	0.1%
AGCTCTGATACAGGG-CTGGGGG	-1	42	0.1%
AGCTCTGATACAGGGGC------	-11	24	0.1%

8h (P71-BC12) 15.5%

Sequence	Type	Reads	Percentage
AGCTCTGATACAGGGGC|TGGGGG	WT		
AGCTCTGATACAGGGGCcTGGGGG	+1	4664	8.7%
AGCTCTGATACAGGGGC-GGGGG	-1	756	1.4%
AGCTCTGATACAGGGGCT-GGGG	-1	271	0.5%
AGCTCTGATACA-------GGGGC	-7	210	0.4%
AGCTCTGATACAGGGG-TGGGGG	-1	109	0.2%
AGCTCTGATACAGGG-CTGGGGG	-1	83	0.2%
AGCTCTGATACA------GGGGG	-6	73	0.1%
AGCTCTGATACAGGGGCtTGGGGG	+1	69	0.1%
AGCTCTGATACAGGGGC------	-9	46	0.1%

12h (P71-BC13) 16.6%

Sequence	Type	Reads	Percentage
AGCTCTGATACAGGGGC|TGGGGG	WT		
AGCTCTGATACAGGGGCcTGGGGG	+1	9762	9.0%
AGCTCTGATACAGGGGC-GGGGG	-1	1578	1.5%
AGCTCTGATACAGGGGCT-GGGG	-1	556	0.5%
AGCTCTGATACA-------GGGGC	-7	532	0.5%
AGCTCTGATACAGGGG-TGGGGG	-1	184	0.2%
AGCTCTGATACA------GGGGG	-6	164	0.2%
AGCTCTGATACAGGGGCtTGGGGG	+1	137	0.1%
AGCTCTGATACAGGG-CTGGGGG	-1	123	0.1%
AGCTCTGATACAGGGGCT-----	-8	90	0.1%

24h (P71-BC14) 20.3%

Sequence	Type	Reads	Percentage
AGCTCTGATACAGGGGC|TGGGGG	WT		
AGCTCTGATACAGGGGCcTGGGGG	+1	10777	9.9%
AGCTCTGATACAGGGGC-GGGGG	-1	1953	1.8%
AGCTCTGATACA-------GGGGC	-7	991	0.9%
AGCTCTGATACAGGGGCT-GGGG	-1	508	0.5%
AGCTCTGATACA------GGGGG	-6	209	0.2%
AGCTCTGATAC------TGGGGG	-6	120	0.1%
AGCTCTGATACAGGGGCtTGGGGG	+1	117	0.1%
AGCTCTGATACAGGGGC--GGGG	-2	114	0.1%
A---------------------GC	-21	110	0.1%

48h (P71-BC15) 21.9%

Sequence	Type	Reads	Percentage
AGCTCTGATACAGGGGC|TGGGGG	WT		
AGCTCTGATACAGGGGCcTGGGGG	+1	7531	8.9%
AGCTCTGATACAGGGGC-GGGGG	-1	1739	2.1%
AGCTCTGATACA-------GGGGC	-7	1266	1.5%
AGCTCTGATACA------GGGGG	-6	620	0.7%
AGCTCTGATACAGGGGCT-GGGG	-1	392	0.5%
AGCTCTGATACAGGG-CTGGGGG	-1	190	0.2%
AGCTCTGATACAGGGG-TGGGGG	-1	176	0.2%
AGCTCTGATACA--------GGG	-8	168	0.2%
AGCTCTGATACA---------GG	-9	130	0.2%

P72 Syn61-crMYH6c scAAV6 KI

4h (P72-BC11) 10.6%

1.1%

	Type	Reads	Percentage
AGCTCTGATACAGGGGC|TGGGGG	WT		
AGCTCTGATACAGGGGCcTGGGGG	+1	4916	5.6%
AGCTCTGGTTTAAACTACGCGTCTCTC	HDR	987	1.1%
AGCTCTGATACAGGGGC-GGGGG	-1	771	0.9%
AGCTCTGATACAGGGGCT-GGGG	-1	465	0.5%
AGCTCTGATACAGGGG-TGGGGG	-1	131	0.1%
AGCTCTGATACAGGG-CTGGGGG	-1	92	0.1%
AGCTCTGATACAGGGGC--GGGG	-2	47	0.1%

8h (P72-BC12) 17.4%

2.8%

	Type	Reads	Percentage
AGCTCTGATACAGGGGC|TGGGGG	WT		
AGCTCTGATACAGGGGCcTGGGGG	+1	4615	8.2%
AGCTCTGGTTTAAACTACGCGTCTCTC	HDR	1552	2.8%
AGCTCTGATACAGGGGC-GGGGG	-1	557	1.0%
AGCTCTGATACAGGGGCT-GGGG	-1	304	0.5%
AGCTCTGATACAGGGG-TGGGGG	-1	112	0.2%
AGCTCTGATACA-------GGGGC	-7	94	0.2%
AGCTCTGATACAGGG-CTGGGGG	-1	89	0.2%
AGCTCTGATACAGGGGC-----G	-5	53	0.1%
AGCTCTGATACAGGGGC--GGGG	-2	45	0.1%

12h (P72-BC13) 18.5%

3.2%

	Type	Reads	Percentage
AGCTCTGATACAGGGGC|TGGGGG	WT		
AGCTCTGATACAGGGGCcTGGGGG	+1	7053	8.0%
AGCTCTGGTTTAAACTACGCGTCTCTC	HDR	2876	3.2%
AGCTCTGATACAGGGGC-GGGGG	-1	993	1.1%
AGCTCTGATACAGGGGCT-GGGG	-1	438	0.5%
AGCTCTGATACA-------GGGGC	-7	257	0.3%
AGCTCTGATACAGGG-CTGGGGG	-1	126	0.1%
AGCTCTGATACA------GGGGG	-6	110	0.1%
AGCTCTGATACAGGGG-TGGGGG	-1	109	0.1%
AGCTCTGATACAGGGGC------	-11	94	0.1%

24h (P72-BC14) 23.3%

5.4%

	Type	Reads	Percentage
AGCTCTGATACAGGGGC|TGGGGG	WT		
AGCTCTGATACAGGGGCcTGGGGG	+1	7150	9.3%
AGCTCTGGTTTAAACTACGCGTCTCTC	HDR	4191	5.4%
AGCTCTGATACAGGGGC-GGGGG	-1	894	1.2%
AGCTCTGATACA-------GGGGC	-7	352	0.5%
AGCTCTGATACAGGGGCT-GGGG	-1	329	0.4%
AGCTCTGATACAGGGG-TGGGGG	-1	168	0.2%
AGCTCTGATACA------GGGGG	-6	114	0.1%
AGCTCTGATACAGGGGC------	-10	101	0.1%

48h (P72-BC15) 27.7%

8.7%

	Type	Reads	Percentage
AGCTCTGATACAGGGGC|TGGGGG	WT		
AGCTCTGATACAGGGGCcTGGGGG	+1	4502	9.5%
AGCTCTGGTTTAAACTACGCGTCTCTC	HDR	4113	8.7%
AGCTCTGATACA-------GGGGC	-7	270	0.6%
AGCTCTGATACAGGGGCT-GGGG	-1	195	0.4%
AGCTCTGATACA------GGGGG	-6	100	0.2%
AGCTCTGATACAGGGGCtTGGGGG	+1	74	0.2%
A---------------------GC	-21	64	0.1%
AGCTCTGATACAGGGG-TGGGGG	-1	57	0.1%

P226-229 W9 RNP KI with small molecules D2 GGG

DMSO Syn83 94.83 54.46	Type	Reads	Percentage
ACTTCATGCGGAGGCCC|CGT	WT	19807	
ACTTCATGCGGAGGCCC-GT	-1	2558	12.91
ACTTCATGCG---------T	-9	679	3.43
ACTTCATGCGGAGG------	-12	406	2.05
ACTTCATGCGGAGGCC--GT	-2	368	1.86
ACTTCATGCGGAGGCCCcCGT	+1	315	1.59

AZT Syn83 92.51 53.16	Type	Reads	Percentage
ACTTCATGCGGAGGCCC|CGT	WT	18655	
ACTTCATGCGGAGGCCC-GT	-1	2480	13.29
ACTTCATGCG---------T	-9	416	2.23
ACTTCATGCGGAGG------	-12	229	1.23
ACTTCATGCGGAGGCC--GT	-2	724	3.88
ACTTCATGCGGAGGCCCcCGT	+1	255	1.67

AZD7762 Syn83 95.22 43.76	Type	Reads	Percentage
ACTTCATGCGGAGGCCC|CGT	WT	23390	
ACTTCATGCGGAGGCCC-GT	-1	3194	13.66
ACTTCATGCG---------T	-9	974	4.16
ACTTCATGCGGAGG------	-12	987	4.22
ACTTCATGCGGAGGCC--GT	-2	572	2.45
ACTTCATGCGGAGGCCCcCGT	+1	401	1.71

Olaparib Syn83 89.47 49.11	Type	Reads	Percentage
ACTTCATGCGGAGGCCC|CGT	WT	15836	
ACTTCATGCGGAGGCCC-GT	-1	1894	11.96
ACTTCATGCG---------T	-9	670	4.23
ACTTCATGCGGAGG------	-12	307	1.94
ACTTCATGCGGAGGCC--GT	-2	196	1.52
ACTTCATGCGGAGGCCCcCGT	+1	311	1.96

B02 Syn83 86.45 45.67	Type	Reads	Percentage
ACTTCATGCGGAGGCCC|CGT	WT	18805	
ACTTCATGCGGAGGCCC-GT	-1	2529	13.45
ACTTCATGCG---------T	-9	616	3.28
ACTTCATGCGGAGG------	-12	194	1.03
ACTTCATGCGGAGGCC--GT	-2	229	1.22
ACTTCATGCGGAGGCCCcCGT	+1	4.17	2.22

TSA Syn83 96.64 64.75	Type	Reads	Percentage
ACTTCATGCGGAGGCCC|CGT	WT	24255	
ACTTCATGCGGAGGCCC-GT	-1	2169	8.94
ACTTCATGCG---------T	-9	632	2.61
ACTTCATGCGGAGG------	-12	418	1.72
ACTTCATGCGGAGGCC--GT	-2	368	1.52
ACTTCATGCGGAGGCCCcCGT	+1	523	2.16

NU7441 Syn83 96.14 68.05	Type	Reads	Percentage
ACTTCATGCGGAGGCCC|CGT	WT	25828	
ACTTCATGCGGAGGCCC-GT	-1	2081	8.06
ACTTCATGCG---------T	-9	795	3.08
ACTTCATGCGGAGG------	-12	455	1.76
ACTTCATGCGGAGGCC--GT	-2	148	0.57
ACTTCATGCGGAGGCCCcCGT	+1	334	1.29

DOPA Syn83 89.26 45.53	Type	Reads	Percentage
ACTTCATGCGGAGGCCC|CGT	WT	14119	
ACTTCATGCGGAGGCCC-GT	-1	2061	14.60
ACTTCATGCG---------T	-9	1335	9.46
ACTTCATGCGGAGG------	-12	250	1.77
ACTTCATGCGGAGGCC--GT	-2	369	2.61
ACTTCATGCGGAGGCCCcCGT	+1	253	1.79

NaB Syn83 97.35 65.98	Type	Reads	Percentage
ACTTCATGCGGAGGCCC|CGT	WT	14516	
ACTTCATGCGGAGGCCC-GT	-1	1626	11.20
ACTTCATGCG---------T	-9	270	1.86
ACTTCATGCGGAGG------	-12	115	0.79
ACTTCATGCGGAGGCC--GT	-2	212	1.46
ACTTCATGCGGAGGCCCcCGT	+1	193	1.33

SCR7 Syn83 93.36 50.28	Type	Reads	Percentage
ACTTCATGCGGAGGCCC|CGT	WT	24259	
ACTTCATGCGGAGGCCC-GT	-1	3622	14.93
ACTTCATGCG---------T	-9	1010	4.16
ACTTCATGCGGAGG------	-12	409	1.69
ACTTCATGCGGAGGCC--GT	-2	285	1.17
ACTTCATGCGGAGGCCCcCGT	+1	433	1.78

NU7026 Syn83 92.57 60.09	Type	Reads	Percentage
ACTTCATGCGGAGGCCC|CGT	WT	27000	
ACTTCATGCGGAGGCCC-GT	-1	2474	9.16
ACTTCATGCG---------T	-9	785	2.91
ACTTCATGCGGAGG------	-12	566	2.10
ACTTCATGCGGAGGCC--GT	-2	380	1.41
ACTTCATGCGGAGGCCCcCGT	+1	242	0.90

CRISPY Syn83 96.71 60.26	Type	Reads	Percentage
ACTTCATGCGGAGGCCC|CGT	WT	15306	
ACTTCATGCGGAGGCCC-GT	-1	1917	12.52
ACTTCATGCG---------T	-9	524	3.42
ACTTCATGCGGAGG------	-12	126	0.82
ACTTCATGCGGAGGCC--GT	-2	332	2.17
ACTTCATGCGGAGGCCCcCGT	+1	224	1.46

P226-229 W9 RNP KI with small molecules D2 GGG

DMSO Syn83 94.83 54.46	Type	Reads	Percentage
ACTTCATGCGGAGGCCC|CGT	WT	19807	
ACTTCATGCGGAGGCCC-GT	-1	2558	12.91
ACTTCATGCG--------T	-9	679	3.43
ACTTCATGCGGAGG------	-12	406	2.05
ACTTCATGCGGAGGCC--GT	-2	368	1.86
ACTTCATGCGGAGGCCCcCGT	+1	315	1.59

M3814 Syn83 96.35 80.52	Type	Reads	Percentage
ACTTCATGCGGAGGCCC|CGT	WT	24830	
ACTTCATGCGGAGGCCC-GT	-1	757	3.05
ACTTCATGCG--------T	-9	254	1.02
ACTTCATGCGGAGG------	-12	283	1.14
ACTTCATGCGGAGGCC--GT	-2	199	0.8
ACTTCATGCGGAGGCCCcCGT	+1	-?	-?

CsH Syn83 92.17 49.30	Type	Reads	Percentage
ACTTCATGCGGAGGCCC|CGT	WT	19809	
ACTTCATGCGGAGGCCC-GT	-1	2909	14.69
ACTTCATGCG--------T	-9	792	4.00
ACTTCATGCGGAGG------	-12	273	1.38
ACTTCATGCGGAGGCC--GT	-2	383	1.93
ACTTCATGCGGAGGCCCcCGT	+1	319	1.61

Mirin Syn83 88.44 42.17	Type	Reads	Percentage
ACTTCATGCGGAGGCCC|CGT	WT	12602	
ACTTCATGCGGAGGCCC-GT	-1	1786	14.17
ACTTCATGCG--------T	-9	825	6.55
ACTTCATGCGGAGG------	-12	244	1.94
ACTTCATGCGGAGGCC--GT	-2	250	1.98
ACTTCATGCGGAGGCCCcCGT	+1	222	1.76

M+TSA X Syn83 97.46 73.74	Type	Reads	Percentage
ACTTCATGCGGAGGCCC|CGT	WT	26961	
ACTTCATGCGGAGGCCC-GT	-1	2036	7.55c
ACTTCATGCG--------T	-9	634	2.35
ACTTCATGCGGAGG------	-12	318	1.18
ACTTCATGCGGAGGCC--GT	-2	423	1.57
ACTTCATGCGGAGGCCCcCGT	+1	275	1.02

M+NaB Syn83 98.36 87.34	Type	Reads	Percentage
ACTTCATGCGGAGGCCC|CGT	WT	28250	
ACTTCATGCGGAGGCCC-GT	-1	263	0.93
ACTTCATGCG--------T	-9	314	1.11
ACTTCATGCGGAGG------	-12	592	2.10
ACTTCATGCGGAGGCC--GT	-2	86	0.30
ACTTCATGCGGAGGCCCcCGT	+1	-?	-?

RS1 Syn83 92.97 51.57	Type	Reads	Percentage
ACTTCATGCGGAGGCCC|CGT	WT	23087	
ACTTCATGCGGAGGCCC-GT	-1	3025	13.10
ACTTCATGCG--------T	-9	1012	4.38
ACTTCATGCGGAGG------	-12	615	2.66
ACTTCATGCGGAGGCC--GT	-2	462	2.00
ACTTCATGCGGAGGCCCcCGT	+1	508	2.20

VE822 Syn83 89.28 43.88	Type	Reads	Percentage
ACTTCATGCGGAGGCCC|CGT	WT	17762	
ACTTCATGCGGAGGCCC-GT	-1	2834	15.96
ACTTCATGCG--------T	-9	1158	6.52
ACTTCATGCGGAGG------	-12	414	2.33
ACTTCATGCGGAGGCC--GT	-2	433	2.44
ACTTCATGCGGAGGCCCcCGT	+1	454	2.56

P226-229 W9 RNP KI with small molecules

DMSO Syn87 90.67 19.80	Type	Reads	Percentage
GGGATTACCGAGTCACC|ACC	WT	23997	
GGGATTACCGAGTC---ACC	-3	7524	31.35
GGGATTACCGAGTCAC-ACC	-1	3767	15.70
GGGATTACCGAGTCACCcACC	+1	2895	12.06
GGGATT-----------ACC	-11	533	2.22

AZT Syn87 88.76 20.73	Type	Reads	Percentage
GGGATTACCGAGTCACC|ACC	WT	22820	
GGGATTACCGAGTC---ACC	-3	6929	30.36
GGGATTACCGAGTCAC-ACC	-1	3570	15.64
GGGATTACCGAGTCACCcACC	+1	2427	10.64
GGGATT-----------ACC	-11	540	2.37

AZD7762 Syn87 92.65 16.48	Type	Reads	Percentage
GGGATTACCGAGTCACC|ACC	WT	17024	
GGGATTACCGAGTC---ACC	-3	5375	31.57
GGGATTACCGAGTCAC-ACC	-1	2794	16.41
GGGATTACCGAGTCACCcACC	+1	2161	12.69
GGGATT-----------ACC	-11	527	3.10

Olaparib Syn87 84.97 12.49	Type	Reads	Percentage
GGGATTACCGAGTCACC|ACC	WT	23290	
GGGATTACCGAGTC---ACC	-3	7945	34.11
GGGATTACCGAGTCAC-ACC	-1	3919	16.83
GGGATTACCGAGTCACCcACC	+1	2197	9.43
GGGATT-----------ACC	-11	431	1.85

B02 Syn87 88.24 14.89	Type	Reads	Percentage
GGGATTACCGAGTCACC|ACC	WT	25985	
GGGATTACCGAGTC---ACC	-3	9358	36.01
GGGATTACCGAGTCAC-ACC	-1	4521	17.40
GGGATTACCGAGTCACCcACC	+1	2536	9.76
GGGATT-----------ACC	-11	409	1.57

TSA Syn87 96.84 33.14	Type	Reads	Percentage
GGGATTACCGAGTCACC|ACC	WT	16056	
GGGATTACCGAGTC---ACC	**-3**	**4111**	**25.60**
GGGATTACCGAGTCAC-ACC	-1	2243	13.97
GGGATTACCGAGTCACCcACC	+1	1936	12.06
GGGATT-----------ACC	-11	351	2.19

NU7441 Syn87 90.85 30.96	Type	Reads	Percentage
GGGATTACCGAGTCACC|ACC	WT	28405	
GGGATTACCGAGTC---ACC	**-3**	**7280**	**25.63**
GGGATTACCGAGTCAC-ACC	-1	3602	12.68
GGGATTACCGAGTCACCcACC	+1	2447	8.61
GGGATT-----------ACC	-11	628	2.21

DOPA Syn87 89.00 14.36	Type	Reads	Percentage
GGGATTACCGAGTCACC|ACC	WT	17024	
GGGATTACCGAGTC---ACC	-3	5583	32.79
GGGATTACCGAGTCAC-ACC	-1	2691	15.81
GGGATTACCGAGTCACCcACC	+1	2198	12.91
GGGATT-----------ACC	-11	330	1.94

NaB Syn87 97.72 34.86	Type	Reads	Percentage
GGGATTACCGAGTCACC|ACC	WT	26922	
GGGATTACCGAGTC---ACC	-3	5971	22.18
GGGATTACCGAGTCAC-ACC	-1	4100	15.23
GGGATTACCGAGTCACCcACC	+1	3857	14.33
GGGATT-----------ACC	-11	401	1.49

SCR7 Syn87 89.41 17.40	Type	Reads	Percentage
GGGATTACCGAGTCACC|ACC	WT	24532	
GGGATTACCGAGTC---ACC	-3	7814	31.85
GGGATTACCGAGTCAC-ACC	-1	3779	15.40
GGGATTACCGAGTCACCcACC	+1	2999	12.22
GGGATT-----------ACC	-11	517	2.11

NU7026 Syn87 92.19 24.17	Type	Reads	Percentage
GGGATTACCGAGTCACC|ACC	WT	26704	
GGGATTACCGAGTC---ACC	-3	8411	31.50
GGGATTACCGAGTCAC-ACC	-1	3764	14.10
GGGATTACCGAGTCACCcACC	+1	2436	9.12
GGGATT-----------ACC	-11	604	2.26

CRISPY Syn87 95.22 24.79	Type	Reads	Percentage
GGGATTACCGAGTCACC|ACC	WT	24184	
GGGATTACCGAGTC---ACC	-3	8299	34.32
GGGATTACCGAGTCAC-ACC	-1	3258	13.47
GGGATTACCGAGTCACCcACC	+1	2827	11.69
GGGATT-----------ACC	-11	488	2.02

P226-229 W9 RNP KI with small molecules

DMSO Syn87 90.67 19.80	Type	Reads	Percentage
GGGATTACCGAGTCACC|ACC	WT	23997	
GGGATTACCGAGTC---ACC	-3	7524	31.35
GGGATTACCGAGTCAC-ACC	-1	3767	15.70
GGGATTACCGAGTCACCcACC	+1	2895	12.06
GGGATT------------ACC	-11	533	2.22

CsH Syn87 91.36 19.58	Type	Reads	Percentage
GGGATTACCGAGTCACC|ACC	WT	20971	
GGGATTACCGAGTC---ACC	-3	6729	32.09
GGGATTACCGAGTCAC-ACC	-1	3326	15.86
GGGATTACCGAGTCACCcACC	+1	2488	11.86
GGGATT------------ACC	-11	370	1.76

M+TSA Syn87 97.96 82.65	Type	Reads	Percentage
GGGATTACCGAGTCACC|ACC	WT	30956	
GGGATTACCGAGTC---ACC	-3	1174	3.79
GGGATTACCGAGTCAC-ACC	-1	705	2.28
GGGATTACCGAGTCACCcACC	+1	679	2.19
GGGATT------------ACC	-11	394	1.21

M3814 Syn87 96.29 71.29	Type	Reads	Percentage
GGGATTACCGAGTCACC|ACC	WT	21893	
GGGATTACCGAGTC---ACC	-3	1746	7.98
GGGATTACCGAGTCAC-ACC	-1	671	3.06
GGGATTACCGAGTCACCcACC	+1	1004	4.59
GGGATT------------ACC	-11	344	1.57

Mirin Syn87 88.12 15.60	Type	Reads	Percentage
GGGATTACCGAGTCACC|ACC	WT	26550	
GGGATTACCGAGTC---ACC	-3	9165	34.52
GGGATTACCGAGTCAC-ACC	-1	4107	15.47
GGGATTACCGAGTCACCcACC	+1	2645	9.96
GGGATT------------ACC	-11	546	2.06

M+NaB Syn87 98.03 82.79	Type	Reads	Percentage
GGGATTACCGAGTCACC|ACC	WT	20440	
GGGATTACCGAGTC---ACC	-3	983	4.81
GGGATTACCGAGTCAC-ACC	-1	425	2.08
GGGATTACCGAGTCACCcACC	+1	401	1.96
GGGATT------------ACC	-11	277	1.36

VE822 Syn87 90.10 13.44	Type	Reads	Percentage
GGGATTACCGAGTCACC|ACC	WT	27663	
GGGATTACCGAGTC---ACC	-3	9315	33.67
GGGATTACCGAGTCAC-ACC	-1	4857	17.56
GGGATTACCGAGTCACCcACC	+1	3639	13.15
GGGATT------------ACC	-11	354	1.28

VE+AZD Syn87 94.44 9.83	Type	Reads	Percentage
GGGATTACCGAGTCACC|ACC	WT	23978	
GGGATTACCGAGTC---ACC	-3	9463	39.47
GGGATTACCGAGTCAC-ACC	-1	4372	18.23
GGGATTACCGAGTCACCcACC	+1	3518	14.67
GGGATT------------ACC	-11	370	1.54

RS1 Syn87 89.07 18.03	Type	Reads	Percentage
GGGATTACCGAGTCACC|ACC	WT	23271	
GGGATTACCGAGTC---ACC	-3	7339	31.54
GGGATTACCGAGTCAC-ACC	-1	3663	15.74
GGGATTACCGAGTCACCcACC	+1	2626	11.28
GGGATT------------ACC	-11	462	1.99

P226-229 W9 RNP KI with small molecules

DMSO Syn92 92.28 29.28	Type	Reads	Percentage
GCTTCGTGACGTTGGAT|GGA	WT	28623	
GCTTCGTGACGTTGGATtGGA	+1	13205	46.13
GCTTCGTGACGTT----GGA	-4	1652	5.77
GCTTCGTGACGTTGGAT-GA	-1	212	0.74
GCTTCGTGACGTTGGA-GGA	-1	175	0.61

AZT Syn92 92.12 29.03	Type	Reads	Percentage
GCTTCGTGACGTTGGAT|GGA	WT	23922	
GCTTCGTGACGTTGGATtGGA	+1	10823	45.24
GCTTCGTGACGTT----GGA	-4	1457	6.09
GCTTCGTGACGTTGGAT-GA	-1	170	0.71
GCTTCGTGACGTTGGA-GGA	-1	214	0.89

AZD7762 Syn92 91.61 22.33	Type	Reads	Percentage
GCTTCGTGACGTTGGAT|GGA	WT	22663	
GCTTCGTGACGTTGGATtGGA	+1	10539	46.50
GCTTCGTGACGTT----GGA	-4	1968	8.68
GCTTCGTGACGTTGGAT-GA	-1	169	0.66
GCTTCGTGACGTTGGA-GGA	-1	213	0.94

Olaparib Syn92 87.96 25.74	Type	Reads	Percentage
GCTTCGTGACGTTGGAT|GGA	WT	27088	
GCTTCGTGACGTTGGATtGGA	+1	11554	42.65
GCTTCGTGACGTT----GGA	-4	2151	7.94
GCTTCGTGACGTTGGAT-GA	-1	199	0.73
GCTTCGTGACGTTGGA-GGA	-1	164	0.61

B02 Syn92 84.14 26.45	Type	Reads	Percentage
GCTTCGTGACGTTGGAT|GGA	WT	31617	
GCTTCGTGACGTTGGATtGGA	+1	13755	43.51
GCTTCGTGACGTT----GGA	-4	1260	3.99
GCTTCGTGACGTTGGAT-GA	-1	250	0.79
GCTTCGTGACGTTGGA-GGA	-1	229	0.72

TSA Syn92 95.26 38.80	Type	Reads	Percentage
GCTTCGTGACGTTGGAT|GGA	WT	25988	
GCTTCGTGACGTTGGATtGGA	+1	10860	41.79
GCTTCGTGACGTT----GGA	-4	1646	6.33
GCTTCGTGACGTTGGAT-GA	-1	130	0.50
GCTTCGTGACGTTGGA-GGA	-1	168	0.65

NU7441 Syn92 91.38 36.38	Type	Reads	Percentage
GCTTCGTGACGTTGGAT|GGA	WT	36303	
GCTTCGTGACGTTGGATtGGA	+1	13575	37.39
GCTTCGTGACGTT----GGA	-4	2250	6.20
GCTTCGTGACGTTGGAT-GA	-1	122	0.34
GCTTCGTGACGTTGGA-GGA	-1	304	0.84

DOPA Syn92 88.75 22.45	Type	Reads	Percentage
GCTTCGTGACGTTGGAT|GGA	WT	25770	
GCTTCGTGACGTTGGATtGGA	+1	11706	45.42
GCTTCGTGACGTT----GGA	-4	1992	7.73
GCTTCGTGACGTTGGAT-GA	-1	205	0.80
GCTTCGTGACGTTGGA-GGA	-1	290	1.13

NaB Syn92 94.83 41.10	Type	Reads	Percentage
GCTTCGTGACGTTGGAT|GGA	WT	35732	
GCTTCGTGACGTTGGATtGGA	+1	15081	42.21
GCTTCGTGACGTT----GGA	-4	1344	3.76
GCTTCGTGACGTTGGAT-GA	-1	?	?
GCTTCGTGACGTTGGA-GGA	-1	109	0.31

SCR7 Syn92 90.10 28.01	Type	Reads	Percentage
GCTTCGTGACGTTGGAT|GGA	WT		
GCTTCGTGACGTTGGATtGGA	+1	23421	44.05
GCTTCGTGACGTT----GGA	-4	1288	5.50
GCTTCGTGACGTTGGAT-GA	-1	243	1.04
GCTTCGTGACGTTGGA-GGA	-1	202	0.86

NU7026 Syn92 90.70 31.20	Type	Reads	Percentage
GCTTCGTGACGTTGGAT|GGA	WT	33424	
GCTTCGTGACGTTGGATtGGA	+1	14063	42.07
GCTTCGTGACGTT----GGA	-4	1654	4.95
GCTTCGTGACGTTGGAT-GA	-1	284	0.85
GCTTCGTGACGTTGGA-GGA	-1	384	1.15

CRISPY Syn92 92.97 36.48	Type	Reads	Percentage
GCTTCGTGACGTTGGAT|GGA	WT	27072	
GCTTCGTGACGTTGGATtGGA	+1	11152	41.19
GCTTCGTGACGTT----GGA	-4	1837	6.79
GCTTCGTGACGTTGGAT-GA	-1	97	0.36
GCTTCGTGACGTTGGA-GGA	-1	388	1.43

P226-229 W9 RNP KI with small molecules

DMSO Syn92 92.28 29.28	Type	Reads	Percentage
GCTTCGTGACGTTGGAT|GGA	WT	28623	
GCTTCGTGACGTTGGATtGGA	+1	13205	46.13
GCTTCGTGACGTT----GGA	-4	1652	5.77
GCTTCGTGACGTTGGAT-GA	-1	212	0.74
GCTTCGTGACGTTGGA-GGA	-1	175	0.61

M3814 Syn92 97.23 73.43	Type	Reads	Percentage
GCTTCGTGACGTTGGAT|GGA	WT	19865	
GCTTCGTGACGTTGGATtGGA	+1	2251	11.33
GCTTCGTGACGTT----GGA	-4	931	4.69
GCTTCGTGACGTTGGAT-GA	-1	?	?
GCTTCGTGACGTTGGA-GGA	-1	?	?

VE822 Syn92 87.48 20.31	Type	Reads	Percentage
GCTTCGTGACGTTGGAT|GGA	WT	28225	
GCTTCGTGACGTTGGATtGGA	+1	14594	51.71
GCTTCGTGACGTT----GGA	-4	1172	4.15
GCTTCGTGACGTTGGAT-GA	-1	203	0.72
GCTTCGTGACGTTGGA-GGA	-1	284	1.01

CsH Syn92 90.17 27.63	Type	Reads	Percentage
GCTTCGTGACGTTGGAT|GGA	WT	25208	
GCTTCGTGACGTTGGATtGGA	+1	11361	45.07
GCTTCGTGACGTT----GGA	-4	1497	5.94
GCTTCGTGACGTTGGAT-GA	-1	?	?
GCTTCGTGACGTTGGA-GGA	-1	228	0.90

Mirin Syn92 86.87 19.65	Type	Reads	Percentage
GCTTCGTGACGTTGGAT|GGA	WT	33158	
GCTTCGTGACGTTGGATtGGA	+1	16617	50.11
GCTTCGTGACGTT----GGA	-4	2209	6.66
GCTTCGTGACGTTGGAT-GA	-1	185	0.56
GCTTCGTGACGTTGGA-GGA	-1	157	0.47

VE+AZD Syn92 87.51 14.58	Type	Reads	Percentage
GCTTCGTGACGTTGGAT|GGA	WT	27111	
GCTTCGTGACGTTGGATtGGA	+1	15310	56.47
GCTTCGTGACGTT----GGA	-4	991	3.66
GCTTCGTGACGTTGGAT-GA	-1	244	0.90
GCTTCGTGACGTTGGA-GGA	-1	439	1.62

M+TSA Syn92 88.02 86.14	Type	Reads	Percentage
GCTTCGTGACGTTGGAT|GGA	WT	38349	
GCTTCGTGACGTTGGATtGGA	+1	2409	6.28
GCTTCGTGACGTT----GGA	-4	852	2.22
GCTTCGTGACGTTGGAT-GA	-1	?	?
GCTTCGTGACGTTGGA-GGA	-1	?	?

M+NaB Syn92 88.73 86.53	Type	Reads	Percentage
GCTTCGTGACGTTGGAT|GGA	WT	19.58	
GCTTCGTGACGTTGGATtGGA	+1	1183	6.14
GCTTCGTGACGTT----GGA	-4	335	1.74
GCTTCGTGACGTTGGAT-GA	-1	?	?
GCTTCGTGACGTTGGA-GGA	-1	48	0.25

RS1 Syn92 90.30 28.65	Type	Reads	Percentage
GCTTCGTGACGTTGGAT|GGA	WT	26659	
GCTTCGTGACGTTGGATtGGA	+1	11681	43.82
GCTTCGTGACGTT----GGA	-4	1708	6.41
GCTTCGTGACGTTGGAT-GA	-1	171	0.64
GCTTCGTGACGTTGGA-GGA	-1	171	0.64

P226-229 W9 RNP KI with small molecules

DMSO Syn94 47.19 12.88	Type	Reads	Percentage
GCGTAGTGTTGGGTCCT|ACC	WT	19505	
GCGTAGTGTTGGGTCCTtACC	+1	3894	19.96
GCGTAGTGTTGGGTC-TACC	-1	294	1.51
GCGTAGTGTTGGGT----CC	-4	258	1.32
GCGTAGTGTTGGGTCCTctACC	+2	207	1.06

Syn94 51.06 14.37	Type	Reads	Percentage
GCGTAGTGTTGGGTCCT|ACC	WT	13859	
GCGTAGTGTTGGGTCCTtACC	+1	2989	21.57
GCGTAGTGTTGGGTC-TACC	-1	239	1.72
GCGTAGTGTTGGGT----CC	-4	219	1.58
GCGTAGTGTTGGGTCCTctACC	+2	118	0.85

AZD7762 Syn94 49.9 11.34	Type	Reads	Percentage
GCGTAGTGTTGGGTCCT|ACC	WT	18136	
GCGTAGTGTTGGGTCCTtACC	+1	8398	46.31
GCGTAGTGTTGGGTC-TACC	-1	152	0.84
GCGTAGTGTTGGGT----CC	-4	424	2.34
GCGTAGTGTTGGGTCCTctACC	+2	198	1.09

Syn94 42.17 8.14	Type	Reads	Percentage
GCGTAGTGTTGGGTCCT|ACC	WT	18581	
GCGTAGTGTTGGGTCCTtACC	+1	3878	20.87
GCGTAGTGTTGGGTC-TACC	-1	163	0.88
GCGTAGTGTTGGGT----CC	-4	341	1.84
GCGTAGTGTTGGGTCCTctACC	+2	138	0.74

B02 Syn94 35.42 6.8	Type	Reads	Percentage
GCGTAGTGTTGGGTCCT|ACC	WT	18810	
GCGTAGTGTTGGGTCCTtACC	+1	3820	20.31
GCGTAGTGTTGGGTC-TACC	-1	108	0.57
GCGTAGTGTTGGGT----CC	-4	55	0.29
GCGTAGTGTTGGGTCCTctACC	+2	106	0.56

TSA Syn94 70.61 23.19	Type	Reads	Percentage
GCGTAGTGTTGGGTCCT|ACC	WT	13666	
GCGTAGTGTTGGGTCCTtACC	+1	4237	31.00
GCGTAGTGTTGGGTC-TACC	-1	89	0.65
GCGTAGTGTTGGGT----CC	-4	271	1.98
GCGTAGTGTTGGGTCCTctACC	+2	99	0.72

Syn94 55.85 19.82	Type	Reads	Percentage
GCGTAGTGTTGGGTCCT|ACC	WT	20672	
GCGTAGTGTTGGGTCCTtACC	+1	3792	18.34
GCGTAGTGTTGGGTC-TACC	-1	104	0.50
GCGTAGTGTTGGGT----CC	-4	551	2.67
GCGTAGTGTTGGGTCCTctACC	+2	251	1.21

DOPA Syn94 48.73 9.67	Type	Reads	Percentage
GCGTAGTGTTGGGTCCT|ACC	WT	16811	
GCGTAGTGTTGGGTCCTtACC	+1	3701	22.02
GCGTAGTGTTGGGTC-TACC	-1	218	1.30
GCGTAGTGTTGGGT----CC	-4	172	1.02
GCGTAGTGTTGGGTCCTctACC	+2	53	0.32

NaB Syn94 66.67 25.06	Type	Reads	Percentage
GCGTAGTGTTGGGTCCT|ACC	WT	22444	
GCGTAGTGTTGGGTCCTtACC	+1	6808	30.33
GCGTAGTGTTGGGTC-TACC	-1	131	0.58
GCGTAGTGTTGGGT----CC	-4	230	1.02
GCGTAGTGTTGGGTCCTctACC	+2	123	0.55

Syn94 46.92 11.48	Type	Reads	Percentage
GCGTAGTGTTGGGTCCT|ACC	WT	21795	
GCGTAGTGTTGGGTCCTtACC	+1		
GCGTAGTGTTGGGTC-TACC	-1	364	1.67
GCGTAGTGTTGGGT----CC	-4	230	1.06
GCGTAGTGTTGGGTCCTctACC	+2	?	?

NU7026 Syn94 51.92 14.27	Type	Reads	Percentage
GCGTAGTGTTGGGTCCT|ACC	WT	19476	
GCGTAGTGTTGGGTCCTtACC	+1	4724	24.26
GCGTAGTGTTGGGTC-TACC	-1	?	?
GCGTAGTGTTGGGT----CC	-4	116	0.60
GCGTAGTGTTGGGTCCTctACC	+2	148	0.76

CRISPY Syn94 60.25 12.53	Type	Reads	Percentage
GCGTAGTGTTGGGTCCT|ACC	WT	23921	
GCGTAGTGTTGGGTCCTtACC	+1	9110	38.08
GCGTAGTGTTGGGTC-TACC	-1	?	?
GCGTAGTGTTGGGT----CC	-4	170	0.71
GCGTAGTGTTGGGTCCTctACC	+2	382	1.60

P226-229 W9 RNP KI with small molecules

DMSO Syn94 47.19 12.88	Type	Reads	Percentage
GCGTAGTGTTGGGTCCT|ACC	WT	19505	
GCGTAGTGTTGGGTCCTtACC	+1	3894	19.96
GCGTAGTGTTGGGTC-TACC	-1	294	1.51
GCGTAGTGTTGGGT----CC	-4	258	1.32
GCGTAGTGTTGGGTCCTctACC	+2	207	[illegible]

CsH Syn94 49.25 11.71	Type	Reads	Percentage
GCGTAGTGTTGGGTCCT|ACC	WT	13852	
GCGTAGTGTTGGGTCCTtACC	+1	3507	25.32
GCGTAGTGTTGGGTC-TACC	-1	151	1.09
GCGTAGTGTTGGGT----CC	-4	109	0.79
GCGTAGTGTTGGGTCCTctACC	+2	122	0.88

M+TSA Syn94 87.25 77.32	Type	Reads	Percentage
GCGTAGTGTTGGGTCCT|ACC	WT	16277	
GCGTAGTGTTGGGTCCTtACC	+1	899	5.52
GCGTAGTGTTGGGTC-TACC	-1	44	0.27
GCGTAGTGTTGGGT----CC	-4	167	1.03
GCGTAGTGTTGGGTCCTctACC	+2	65	0.40

M3814 Syn94 78.32 49.81	Type	Reads	Percentage
GCGTAGTGTTGGGTCCT|ACC	WT	16918	
GCGTAGTGTTGGGTCCTtACC	+1	1347	7.96
GCGTAGTGTTGGGTC-TACC	-1	79	0.47
GCGTAGTGTTGGGT----CC	-4	?	?
GCGTAGTGTTGGGTCCTctACC	+2	159	0.94

Mirin Syn94 44.99 10.07	Type	Reads	Percentage
GCGTAGTGTTGGGTCCT|ACC	WT	21699	
GCGTAGTGTTGGGTCCTtACC	+1	4712	21.72
GCGTAGTGTTGGGTC-TACC	-1	205	0.94
GCGTAGTGTTGGGT----CC	-4	?	?
GCGTAGTGTTGGGTCCTctACC	+2	312	1.44

M+NaB Syn94 87.15 72.70	Type	Reads	Percentage
GCGTAGTGTTGGGTCCT|ACC	WT	17121	
GCGTAGTGTTGGGTCCTtACC	+1	834	4.87
GCGTAGTGTTGGGTC-TACC	-1	114	0.67
GCGTAGTGTTGGGT----CC	-4	169	0.99
GCGTAGTGTTGGGTCCTctACC	+2	?	?

VE822 Syn94 47.69 7.81	Type	Reads	Percentage
GCGTAGTGTTGGGTCCT|ACC	WT	15409	
GCGTAGTGTTGGGTCCTtACC	+1	4094	26.57
GCGTAGTGTTGGGTC-TACC	-1	214	1.39
GCGTAGTGTTGGGT----CC	-4	81	0.53
GCGTAGTGTTGGGTCCTctACC	+2	363	[illegible]

VE+AZD Syn94 42.26 6.86	Type	Reads	Percentage
GCGTAGTGTTGGGTCCT|ACC	WT	22755	
GCGTAGTGTTGGGTCCTtACC	+1	4893	21.50
GCGTAGTGTTGGGTC-TACC	-1	73	0.32
GCGTAGTGTTGGGT----CC	-4	175	0.77
GCGTAGTGTTGGGTCCTctACC	+2	216	[illegible]

RS1 Syn94 47.77 12.94	Type	Reads	Percentage
GCGTAGTGTTGGGTCCT|ACC	WT	14719	
GCGTAGTGTTGGGTCCTtACC	+1	3596	24.43
GCGTAGTGTTGGGTC-TACC	-1	157	1.07
GCGTAGTGTTGGGT----CC	-4	107	0.72
GCGTAGTGTTGGGTCCTctACC	+2	132	[illegible]

P226-229 W9 RNP KI with small molecules

DMSO Syn100 52.34 14.86	Type	Reads	Percentage
GTCGGACTTGACCGTCA|TGG	WT	19089	
GTCGGACTTGACCGTCAaTGG	+1		14.12
GTCGGACTTGACCGTCA-GG	-1	1421	7.44
GTCGGACTTGACCGTCAtTGG	+1	552	2.89
GTCGGACTTGACCGTC-TGG	-1	304	1.59

AZT Syn100 51.65 13.85	Type	Reads	Percentage
GTCGGACTTGACCGTCA|TGG	WT	16493	
GTCGGACTTGACCGTCAaTGG	+1	2287	13.87
GTCGGACTTGACCGTCA-GG	-1	797	4.83
GTCGGACTTGACCGTCAtTGG	+1	345	2.09
GTCGGACTTGACCGTC-TGG	-1	356	2.16

AZD7762 Syn100 57.92 12.43	Type	Reads	Percentage
GTCGGACTTGACCGTCA|TGG	WT	19132	
GTCGGACTTGACCGTCAaTGG	+1	2973	15.54
GTCGGACTTGACCGTCA-GG	-1	1590	8.31
GTCGGACTTGACCGTCAtTGG	+1	491	2.57
GTCGGACTTGACCGTC-TGG	-1	231	1.21

Syn100 36.26 8.12	Type	Reads	Percentage
GTCGGACTTGACCGTCA|TGG	WT	17429	
GTCGGACTTGACCGTCAaTGG	+1	1980	11.36
GTCGGACTTGACCGTCA-GG	-1	224	1.29
GTCGGACTTGACCGTCAtTGG	+1	567	3.25
GTCGGACTTGACCGTC-TGG	-1	132	0.76

B02 Syn100 39.82 9.18	Type	Reads	Percentage
GTCGGACTTGACCGTCA|TGG	WT	16523	
GTCGGACTTGACCGTCAaTGG	+1	1387	8.39
GTCGGACTTGACCGTCA-GG	-1	1146	6.94
GTCGGACTTGACCGTCAtTGG	+1	432	2.61
GTCGGACTTGACCGTC-TGG	-1	210	1.27

TSA Syn100 68.22 26.75	Type	Reads	Percentage
GTCGGACTTGACCGTCA|TGG	WT	17348	
GTCGGACTTGACCGTCAaTGG	+1	2815	16.23
GTCGGACTTGACCGTCA-GG	-1	1179	6.80
GTCGGACTTGACCGTCAtTGG	+1	809	4.66
GTCGGACTTGACCGTC-TGG	-1	264	1.52

NU7441 Syn100 57.1 22.77	Type	Reads	Percentage
GTCGGACTTGACCGTCA|TGG	WT	13792	
GTCGGACTTGACCGTCAaTGG	+1	1672	12.12
GTCGGACTTGACCGTCA-GG	-1	699	5.07
GTCGGACTTGACCGTCAtTGG	+1	203	1.47
GTCGGACTTGACCGTC-TGG	-1	170	1.23

DOPA Syn100 49.15 10.29	Type	Reads	Percentage
GTCGGACTTGACCGTCA|TGG	WT	15494	
GTCGGACTTGACCGTCAaTGG	+1	2136	13.79
GTCGGACTTGACCGTCA-GG	-1	799	5.16
GTCGGACTTGACCGTCAtTGG	+1	515	3.32
GTCGGACTTGACCGTC-TGG	-1	309	1.99

NaB Syn100 70.54 27.10	Type	Reads	Percentage
GTCGGACTTGACCGTCA|TGG	WT	18612	
GTCGGACTTGACCGTCAaTGG	+1	3419	18.37
GTCGGACTTGACCGTCA-GG	-1	1114	5.99
GTCGGACTTGACCGTCAtTGG	+1	611	3.28
GTCGGACTTGACCGTC-TGG	-1	239	1.28

SCR7 Syn100 51.38 11.99	Type	Reads	Percentage
GTCGGACTTGACCGTCA|TGG	WT	21994	
GTCGGACTTGACCGTCAaTGG	+1	2994	13.61
GTCGGACTTGACCGTCA-GG	-1	1653	7.52
GTCGGACTTGACCGTCAtTGG	+1	546	2.48
GTCGGACTTGACCGTC-TGG	-1	273	1.24

NU7026 Syn100 53.73 14.73	Type	Reads	Percentage
GTCGGACTTGACCGTCA|TGG	WT	15610	
GTCGGACTTGACCGTCAaTGG	+1	1995	12.79
GTCGGACTTGACCGTCA-GG	-1	1216	7.79
GTCGGACTTGACCGTCAtTGG	+1	376	2.41
GTCGGACTTGACCGTC-TGG	-1	276	1.77

CRISPY Syn100 55.97 23.94	Type	Reads	Percentage
GTCGGACTTGACCGTCA|TGG	WT	17141	
GTCGGACTTGACCGTCAaTGG	+1	1535	8.96
GTCGGACTTGACCGTCA-GG	-1	1220	7.12
GTCGGACTTGACCGTCAtTGG	+1	?	?
GTCGGACTTGACCGTC-TGG	-1	426	2.49

P226-229 W9 RNP KI with small molecules

DMSO Syn100 52.34 14.86	Type	Reads	Percentage
GTCGGACTTGACCGTCA|TGG	WT	19089	
GTCGGACTTGACCGTCAaTGG	+1	**8590**	**45.00**
GTCGGACTTGACCGTCA-GG	-1	1421	7.44
GTCGGACTTGACCGTCAtTGG	+1	552	2.89
GTCGGACTTGACCGTC-TGG	-1	304	1.59

CsH Syn100 49.84 11.71	Type	Reads	Percentage
GTCGGACTTGACCGTCA|TGG	WT	16058	
GTCGGACTTGACCGTCAaTGG	+1	2087	13.00
GTCGGACTTGACCGTCA-GG	-1	1119	6.97
GTCGGACTTGACCGTCAtTGG	+1	451	2.81
GTCGGACTTGACCGTC-TGG	-1	332	2.07

M+TSA Syn100 82.95 68.41	Type	Reads	Percentage
GTCGGACTTGACCGTCA|TGG	WT	18731	
GTCGGACTTGACCGTCAaTGG	+1	962	5.14
GTCGGACTTGACCGTCA-GG	-1	635	3.39
GTCGGACTTGACCGTCAtTGG	+1	90	0.48
GTCGGACTTGACCGTC-TGG	-1	77	0.41

M3814 Syn100 76.10 48.64	Type	Reads	Percentage
GTCGGACTTGACCGTCA|TGG	WT	19473	
GTCGGACTTGACCGTCAaTGG	+1	1160	5.96
GTCGGACTTGACCGTCA-GG	-1	538	2.76
GTCGGACTTGACCGTCAtTGG	+1	107	0.55
GTCGGACTTGACCGTC-TGG	-1	123	0.63

Mirin Syn100 46.29 8.01	Type	Reads	Percentage
GTCGGACTTGACCGTCA|TGG	WT	18595	
GTCGGACTTGACCGTCAaTGG	+1	2507	13.48
GTCGGACTTGACCGTCA-GG	-1	1210	6.51
GTCGGACTTGACCGTCAtTGG	+1	549	2.95
GTCGGACTTGACCGTC-TGG	-1	293	1.58

M+NaB Syn100 82.67 67.97	Type	Reads	Percentage
GTCGGACTTGACCGTCA|TGG	WT	22617	
GTCGGACTTGACCGTCAaTGG	+1	758	3.35
GTCGGACTTGACCGTCA-GG	-1	264	1.17
GTCGGACTTGACCGTCAtTGG	+1	57	0.25
GTCGGACTTGACCGTC-TGG	-1	221	0.98

VE822 Syn100 49.32 3.89	Type	Reads	Percentage
GTCGGACTTGACCGTCA|TGG	WT	18625	
GTCGGACTTGACCGTCAaTGG	+1	3426	18.39
GTCGGACTTGACCGTCA-GG	-1	2240	12.03
GTCGGACTTGACCGTCAtTGG	+1	?	?
GTCGGACTTGACCGTC-TGG	-1	?	?

VE+AZD Syn100 54.09 5.99	Type	Reads	Percentage
GTCGGACTTGACCGTCA|TGG	WT	13264	
GTCGGACTTGACCGTCAaTGG	+1	1994	15.03
GTCGGACTTGACCGTCA-GG	-1	1815	13.68
GTCGGACTTGACCGTCAtTGG	+1	348	2.62
GTCGGACTTGACCGTC-TGG	-1	182	1.374

RS1 Syn100 51.27 12.64	Type	Reads	Percentage
GTCGGACTTGACCGTCA|TGG	WT	19387	
GTCGGACTTGACCGTCAaTGG	+1	2501	12.90
GTCGGACTTGACCGTCA-GG	-1	1314	6.78
GTCGGACTTGACCGTCAtTGG	+1	761	3.93
GTCGGACTTGACCGTC-TGG	-1	355	1.83

P258-YW-W9-Syn20crAAVS1c-RNPko

KO Ctrl Syn20 95.77%	Type	Reads	Percentage
TCTAACCCCCACCTCCT|GTTAGG	WT	264	4.23
TCTAACCCCCACCTCCTtGTTAGG	+T	4434	71.07
TCTAACCCCCACCTCCTctGTTAGG	+2	120	1.92
TCTAACCCCCACCTCCT-------	-15	115	1.84
TCTAACCCCCACCTCCT--TAGG	-2	102	1.63

P1664 Syn20 96.22% 24.65%	Type	Reads	Percentage
TCTAACCCCCACCTCCT|GTTAGG	WT	174	3.78
TCTAACCCCCACCTCCTtGTTAGG	+T	2567	55.76
TCTAACCCCCACCTCCTctGTTAGG	+2	85	1.85
TCTAACCCCCACCTCCT--TAGG	-2	50	1.09
TCTAACCCCCACCTCCT-------	-15	24	0.52

M3814-1uM Syn20 78.69%	Type	Reads	Percentage
TCTAACCCCCACCTCCT|GTTAGG	WT	2140	21.31
TCTAACCCCCACCTCCTtGTTAGG	+T	2586	25.76
TCTAACCCCCACCTCCT-------	-15	688	4.85
TCTAACCCCCACCT----------	-18	438	4.36
TCTAACCCCCACCTCCT--TAGG	-2	318	3.14

M3814-2uM Syn20 83.38%	Type	Reads	Percentage
TCTAACCCCCACCTCCT|GTTAGG	WT	2265	16.62
TCTAACCCCCACCTCCTtGTTAGG	+T	3823	28.06
TCTAACCCCCACCTCCT-------	-15	1009	7.41
TCTAACCCCCACCT----------	-18	860	6.31
TCTAACCCCCACCTCCT--TAGG	-2	185	1.36

M3814-4uM Syn20 85.69%	Type	Reads	Percentage
TCTAACCCCCACCTCCT|GTTAGG	WT	2864	14.31x
TCTAACCCCCACCTCCTtGTTAGG	+T	4236	21.17
TCTAACCCCCACCT----------	-18	2121	10.60
TCTAACCCCCACCTCCT-------	-15	1215	6.07
TCTAACCCCCACCTCCT--TAGG	-2	643	3.21

TSA-0.01uM Syn20 96.87%	Type	Reads	Percentage
TCTAACCCCCACCTCCT|GTTAGG	WT	741	3.13
TCTAACCCCCACCTCCTtGTTAGG	+T	16958	71.66
TCTAACCCCCACCTCCT--TAGG	-2	530	2.24
TCTAACCCCCACCTCCTctGTTAGG	+2	460	1.94
TCTAACCCCCACCTCCT-------	-15	420	1.77

TSA-0.05uM Syn20 95.28%	Type	Reads	Percentage
TCTAACCCCCACCTCCT|GTTAGG	WT	1180	4.72
TCTAACCCCCACCTCCTtGTTAGG	+T	17673	70.73
TCTAACCCCCACCTCCTctGTTAGG	+2	454	1.82
TCTAACCCCCACCTCCT-------	-15	402	1.61
TCTAACCCCCACCTCCT--TAGG	-2	331	1.32

TSA-0.1uM Syn20 91.65%	Type	Reads	Percentage
TCTAACCCCCACCTCCT|GTTAGG	WT	2080	8.35
TCTAACCCCCACCTCCTtGTTAGG	+T	17736	71.19
TCTAACCCCCACCTCCT--TAGG	-2	350	1.40
TCTAACCCCCACCTCCTctGTTAGG	+2	345	1.38
TCTAACCCCCACCTCCT-------	-15	319	1.28

SCR7-1uM Syn20 94.50%	Type	Reads	Percentage
TCTAACCCCCACCTCCT|GTTAGG	WT	905	5.50
TCTAACCCCCACCTCCTtGTTAGG	+T	11455	69.67
TCTAACCCCCACCTCCTctGTTAGG	+2	307	1.87
TCTAACCCCCACCTCCT-------	-15	302	1.84
TCTAACCCCCACCTCCT--TAGG	-2	280	1.7

SCR7-5uM Syn20 93.65%	Type	Reads	Percentage
TCTAACCCCCACCTCCT|GTTAGG	WT	1252	6.35
TCTAACCCCCACCTCCTtGTTAGG	+T	14148	71.77
TCTAACCCCCACCTCCT--TAGG	-2	383	1.94
TCTAACCCCCACCTCCTctGTTAGG	+2	351	1.78
TCTAACCCCCACCTCCT-------	-15	254	1.29

SCR7-10uM Syn20 94.15%	Type	Reads	Percentage
TCTAACCCCCACCTCCT|GTTAGG	WT	694	5.85
TCTAACCCCCACCTCCTtGTTAGG	+T	8304	69.97
TCTAACCCCCACCTCCTctGTTAGG	+2	194	1.63
TCTAACCCCCACCTCCT-------	-15	193	1.63
TCTAACCCCCACCTCCT--TAGG	-2	153	1.29

P262-YW-W9-Syn20crAAVS1c-RNPko (P258repeat)

KO Ctrl Syn20 96.07%	Type	Reads	Percentage
TCTAACCCCCACCTCCT|GTTAGG	WT	324	3.93
TCTAACCCCCACCTCCTtGTTAGG	+T	5949	72.22
TCTAACCCCCACCTCCTctGTTAGG	+2	184	2.23
TCTAACCCCCACCTCCT--TAGG	-2	169	2.05
TCTAACCCCCACCTCCT-------	-15	146	1.77

P1664 Syn20 96.89% 25.54%	Type	Reads	Percentage
TCTAACCCCCACCTCCT|GTTAGG	WT	185	3.11
TCTAACCCCCACCTCCTtGTTAGG	+T	3320	55.75
TCTAACCCCCACCTCCTctGTTAGG	+2	120	2.02
TCTAACCCCCACCTCCT--TAGG	-2	84	1.41
TCTAACCCCCACCTCCT-------	-15	39	0.65

M3814-1uM Syn20 77.57%	Type	Reads	Percentage
TCTAACCCCCACCTCCT|GTTAGG	WT	3037	22.43
TCTAACCCCCACCTCCTtGTTAGG	+T	3746	27.66
TCTAACCCCCACCTCCT-------	-15	974	7.19
TCTAACCCCCACCT----------	-18	522	3.85
TCTAACCCCCACCTCCT--TAGG	-2	426	3.15

M3814-2uM Syn20 83.26%	Type	Reads	Percentage
TCTAACCCCCACCTCCT|GTTAGG	WT	2845	16.74
TCTAACCCCCACCTCCTtGTTAGG	+T	4750	27.95
TCTAACCCCCACCTCCT-------	-15	1355	7.97
TCTAACCCCCACCT----------	-18	1088	6.40
TCTAACCCCCACCTCCT--TAGG	-2	233	1.37

M3814-4uM Syn20 85.74%	Type	Reads	Percentage
TCTAACCCCCACCTCCT|GTTAGG	WT	4118	14.26
TCTAACCCCCACCTCCTtGTTAGG	+T	6208	21.50
TCTAACCCCCACCT----------	-18	3009	1042
TCTAACCCCCACCTCCT-------	-15	1849	6.40
TCTAACCCCCACCTCCT--TAGG	-2	494	3.30

TSA-0.01uM Syn20 96.60%	Type	Reads	Percentage
TCTAACCCCCACCTCCT|GTTAGG	WT	1046	3.40
TCTAACCCCCACCTCCTtGTTAGG	+T	22611	73.52
TCTAACCCCCACCTCCT--TAGG	-2	603	1.96
TCTAACCCCCACCTCCTctGTTAGG	+2	599	1.95
TCTAACCCCCACCTCCT-------	-15	479	1.56

TSA-0.05uM Syn20 95.23%	Type	Reads	Percentage
TCTAACCCCCACCTCCT|GTTAGG	WT	1634	4.77
TCTAACCCCCACCTCCTtGTTAGG	+T	24571	71.67
TCTAACCCCCACCTCCTctGTTAGG	+2	681	1.99
TCTAACCCCCACCTCCT-------	-15	521	1.52
TCTAACCCCCACCTCCT--TAGG	-2	486	1.42

TSA-0.1uM Syn20 91.65%	Type	Reads	Percentage
TCTAACCCCCACCTCCT|GTTAGG	WT	2851	8.44
TCTAACCCCCACCTCCTtGTTAGG	+T	24173	71.56
TCTAACCCCCACCTCCT--TAGG	-2	470	1.39
TCTAACCCCCACCTCCTctGTTAGG	+2	459	1.36
TCTAACCCCCACCTCCT-------	-15	437	1.29

SCR7-1uM Syn20 94.65%	Type	Reads	Percentage
TCTAACCCCCACCTCCT|GTTAGG	WT	1117	5.35
TCTAACCCCCACCTCCTtGTTAGG	+T	14924	71.44
TCTAACCCCCACCTCCT-------	-15	397	1.90
TCTAACCCCCACCTCCT--TAGG	-2	382	1.83
TCTAACCCCCACCTCCTctGTTAGG	+2	331	1.58

SCR7-5uM Syn20 93.25%	Type	Reads	Percentage
TCTAACCCCCACCTCCT|GTTAGG	WT	1705	6.75
TCTAACCCCCACCTCCTtGTTAGG	+T	18459	73.04
TCTAACCCCCACCTCCT--TAGG	-2	498	1.97
TCTAACCCCCACCTCCTctGTTAGG	+2	420	1.66
TCTAACCCCCACCTCCT-------	-15	347	1.37

SCR7-10uM Syn20 94.37%	Type	Reads	Percentage
TCTAACCCCCACCTCCT|GTTAGG	WT	1044	5.63
TCTAACCCCCACCTCCTtGTTAGG	+T	13217	71.23
TCTAACCCCCACCTCCT-------	-15	345	1.86
TCTAACCCCCACCTCCTctGTTAGG	+2	299	1.61
TCTAACCCCCACCTCCT--TAGG	-2	264	1.42

P260-YW-TC-Syn20crAAVS1c-RNPko

KO Ctrl Syn20 67.06%	Type	Reads	Percentage
TCTAACCCCCACCTCCT|GTTAGG	WT	5213	32.94
TCTAACCCCCACCTCCTtGTTAGG	+T	5356	33.85
TCTAACCCCCACCT----------	-18	496	3.13
TCTAACCCCCACCTCCT-------	-15	404	2.55
TCTAACCCCCACCTCCT--TAGG	-2	154	0.97

P1664 Syn20 86.22% 31.81%	Type	Reads	Percentage
TCTAACCCCCACCTCCT|GTTAGG	WT	774	13.78
TCTAACCCCCACCTCCTtGTTAGG	+T	1931	34.38
TCTAACCCCCACCTCCT-------	-15	88	1.57
TCTAACCCCCACCT----------	-18	81	1.44
TCTAACCCCCACCTCCT--TAGG	-2	65	1.16

M3814-1uM Syn20 64.49%	Type	Reads	Percentage
TCTAACCCCCACCTCCT|GTTAGG	WT	6775	35.51
TCTAACCCCCACCT----------	-18	1882	9.86
TCTAACCCCCACCTCCT-------	-15	1245	6.52
TCTAACCCCCACCTCCTtGTTAGG	+T	695	3.64
TCTAACC-----------------	-24	366	1.92

M3814-2uM Syn20 64.99%	Type	Reads	Percentage
TCTAACCCCCACCTCCT|GTTAGG	WT	7819	35.01
TCTAACCCCCACCT----------	-18	2722	12.19
TCTAACCCCCACCTCCT-------	-15	1348	6.04
TCTAACCCCCACCTCCTtGTTAGG	+T	775	3.47
TCTAACCCCCACCTCCT--TAGG	-2	126	0.56

M3814-4uM Syn20 64.07%	Type	Reads	Percentage
TCTAACCCCCACCTCCT|GTTAGG	WT	13586	35.93
TCTAACCCCCACCT----------	-18	3518	9.30
TCTAACCCCCACCTCCT-------	-15	2575	6.81
TCTAACCCCCACCTCCTtGTTAGG	+T	879	2.32
TCTAACCCCCACCTCCT--TAGG	-2	149	0.39

TSA-0.01uM Syn20 73.85%	Type	Reads	Percentage
TCTAACCCCCACCTCCT|GTTAGG	WT	10591	26.15
TCTAACCCCCACCTCCTtGTTAGG	+T	14689	36.27
TCTAACCCCCACCT----------	-18	1348	3.33
TCTAACCCCCACCTCCT-------	-15	1255	3.10
TCTAACCCCCACCTCCT--TAGG	-2	660	1.63

TSA-0.05uM Syn20 60.76%	Type	Reads	Percentage
TCTAACCCCCACCTCCT|GTTAGG	WT	17145	39.24
TCTAACCCCCACCTCCTtGTTAGG	+T	12464	28.53
TCTAACCCCCACCT----------	-18	1315	3.01
TCTAACCCCCACCTCCT-------	-15	881	2.02
TCTAACCCCCACCTCCT--TAGG	-2	434	0.99

TSA-0.1uM Syn20 35.95%	Type	Reads	Percentage
TCTAACCCCCACCTCCT|GTTAGG	WT	23402	64.05
TCTAACCCCCACCTCCTtGTTAGG	+T	5963	16.32
TCTAACCCCCACCTCCT-------	-15	658	1.8
TCTAACCCCCACCT----------	-18	457	1.25
TCTAACCCCCACCTCCT--TAGG	-2	300	0.82

SCR7-1uM Syn20 72.57%	Type	Reads	Percentage
TCTAACCCCCACCTCCT|GTTAGG	WT	5919	27.43
TCTAACCCCCACCTCCTtGTTAGG	+T	7346	34.04
TCTAACCCCCACCT----------	-18	962	4.46
TCTAACCCCCACCTCCT-------	-15	6464	2.99
TCTAACCCCCACCTCCT--TAGG	-2	185	0.86

SCR7-5uM Syn20 72.98%	Type	Reads	Percentage
TCTAACCCCCACCTCCT|GTTAGG	WT	7306	27.02
TCTAACCCCCACCTCCTtGTTAGG	+T	9405	34.79
TCTAACCCCCACCT----------	-18	1088	4.02
TCTAACCCCCACCTCCT-------	-15	772	2.83
TCTAACCCCCACCTCCT--TAGG	-2	285	1.05

SCR7-10uM Syn20 67.98%	Type	Reads	Percentage
TCTAACCCCCACCTCCT|GTTAGG	WT	6551	32.02
TCTAACCCCCACCTCCTtGTTAGG	+T	6508	31.81
TCTAACCCCCACCTCCT-------	-15	606	2.96
TCTAACCCCCACCT----------	-18	595	2.91
TCTAACCCCCACCTCCT--TAGG	-2	210	1.03

P259-YW-W9-Syn24crAAVS1g-KO

KO Ctrl Syn24 75.09%	Type	Reads	Percentage
GGAATCTGCCTAACAGG|AGGTGG	WT	4662	24.91
GGAATCTGCCTAACAGG---TGG	-3	5414	28.93
GGAATCTGCCTAACAGG-GGTGG	-A	905	4.84
GGAATCTGCCTAACAG-AGGTGG	-G	582	3.11
GGAATCTGCCTAACAGGgAGGTGG	+G	443	2.37
GGAATCTGCCTAACAGG--GTGG	-2	337	1.80

P1664 Syn24 87.12% 49.65%	Type	Reads	Percentage
GGAATCTGCCTAACAGG|AGGTGG	WT	1337	12.88
GGAATCTGCCTAACAGG---TGG	-3	652	6.28
GGAATCTGCCTAACAGG-GGTGG	-A	516	4.97
GGAATCTGCCTAACAGGgAGGTGG	+G	303	2.92
GGAATCTGCCTAACAG-AGGTGG	-G	224	2.16
GGAATCTGCCTAACAGG--GTGG	-2	79	0.76

M3814-4uM Syn24 85.20%	Type	Reads	Percentage
GGAATCTGCCTAACAGG|AGGTGG	WT	7338	14.80
GGAATCTGCCTAACAGG---TGG	-3	19371	39.07
GGAATCTGCCTAACA---------	-9	1432	2.89
GGAATCTGCC--------------	-14	1071	2.16
GGAATCTGCCTAACAGGgAGGTGG	+G	768	1.55

M3814-1uM Syn24 82.20%	Type	Reads	Percentage
GGAATCTGCCTAACAGG|AGGTGG	WT	5456	17.80
GGAATCTGCCTAACAGG---TGG	-3	13147	42.90
GGAATCTGCCTAACA---------	-9	809	2.64
GGAATCTGCC--------------	-14	435	1.42
GGAATCTGCCTAACA-------G	-7	382	1.25

M3814-2uM Syn24 82.49%	Type	Reads	Percentage
GGAATCTGCCTAACAGG|AGGTGG	WT	6797	17.51
GGAATCTGCCTAACAGG---TGG	-3	14146	36.44
GGAATCTGCCTAACA---------	-9	1236	3.18
GGAATCTGCC--------------	-14	605	1.56
GGAATCTGCCTAACAGGgAGGTGG	+G	592	1.53

TSA-0.1uM Syn24 58.75%	Type	Reads	Percentage
GGAATCTGCCTAACAGG|AGGTG G	WT	20418	41.25
GGAATCTGCCTAACAGG---TGG	-3	7780	15.72
GGAATCTGCCTAACAGG-GGTGG	-A	2188	4.42
GGAATCTGCCTAACAG-AGGTGG	-G	1707	3.45
GGAATCTGCCTAACAGGgAGGTG G	+G	1363	2.75
GGAATCTGCCTAACAGG--GTGG	-2	846	1.71

TSA-0.01uM Syn24 70.61%	Type	Reads	Percentage
GGAATCTGCCTAACAGG|AGGTGG	WT	14554	29.39
GGAATCTGCCTAACAGG---TGG	-3	11453	23.13
GGAATCTGCCTAACAGG-GGTGG	-A	3037	6.13
GGAATCTGCCTAACAGGgAGGTGG	+G	1354	2.73
GGAATCTGCCTAACAG-AGGTGG	-G	1112	2.25
GGAATCTGCCTAACAGG--GTGG	-2	885	1.79

TSA-0.05uM Syn24 60.55%	Type	Reads	Percentage
GGAATCTGCCTAACAGG|AGGTGG	WT	19560	39.45
GGAATCTGCCTAACAGG---TGG	-3	8991	18.13
GGAATCTGCCTAACAGG-GGTGG	-A	2460	4.96
GGAATCTGCCTAACAG-AGGTGG	-G	1546	3.12
GGAATCTGCCTAACAGGgAGGTGG	+G	1324	2.67
GGAATCTGCCTAACAGG--GTGG	-2	773	1.56

SCR7-10uM Syn24 68.74%	Type	Reads	Percentage
GGAATCTGCCTAACAGG|AGGTGG	WT	7918	31.26
GGAATCTGCCTAACAGG---TGG	-3	7028	27.75
GGAATCTGCCTAACAGG-GGTGG	-A	1153	4.55
GGAATCTGCCTAACAG-AGGTGG	-G	589	2.33
GGAATCTGCCTAACAGGgAGGTGG	+G	2.05	519
GGAATCTGCCT------AGGTGG	-6	304	1.20
GGAATCTGCCTA-----AGGTGG	-5	272	1.07

SCR7-1uM Syn24 70.53%	Type	Reads	Percentage
GGAATCTGCCTAACAGG|AGGTGG	WT	11782	29.47
GGAATCTGCCTAACAGG---TGG	-3	10671	26.69
GGAATCTGCCTAACAGG-GGTGG	-A	1575	3.94
GGAATCTGCCTAACAGGgAGGTGG	+G	1160	2.90
GGAATCTGCCTAACAG-AGGTGG	-G	786	1.97
GGAATCTGCCTA-----AGGTGG	-5	487	1.22
GGAATCTGCCTAACA--------	-8	453	1.13
GGAATCTGCCTAACAGG--GTGG	-2	416	1.04

SCR7-5uM Syn24 67.26%	Type	Reads	Percentage
GGAATCTGCCTAACAGG|AGGTGG	WT	15258	32.74
GGAATCTGCCTAACAGG---TGG	-3	12310	26.42
GGAATCTGCCTAACAGG-GGTGG	-A	2032	4.36
GGAATCTGCCTAACAG-AGGTGG	-G	944	2.03
GGAATCTGCCTAACAGGgAGGTGG	+G	884	1.90
GGAATCTGCCTAACAGG--GTGG	-2	623	1.34
GGAATCTGCCTA-----AGGTGG	-5	623	1.34

P261-YW-TC-Syn24crAAVS1g-KO

KO Ctrl Syn24 59.00%	Type	Reads	Percentage
GGAATCTGCCTAACAGG|AGGTGG	WT	13260	58.78
GGAATCTGCCTAACAGG---TGG	-3	2769	12.27
GGAATCTGCCTAACAG-AGGTGG	-G	585	2.59
GGAATCTGCCTAACAGG--GTGG	-2	379	1.68
GGAATCTGCCTAACAGGgAGGTGG	+G	113	0.50

P1664 Syn24 52.88% 22.47%	Type	Reads	Percentage
GGAATCTGCCTAACAGG|AGGTGG	WT	3496	47.12
GGAATCTGCCTAACAGG---TGG	-3	601	8.10
GGAATCTGCCTAACAG-AGGTGG	-G	162	2.18
GGAATCTGCCTAACAGG--GTGG	-2	147	1.98
GGAATCTGCCTA-----AGGTGG	-5	43	0.58

M3814-1uM Syn24 58.15%	Type	Reads	Percentage
GGAATCTGCCTAACAGG|AGGTGG	WT	6377	41.85
GGAATCTGCCTAACAGG---TGG	-3	2992	19.63
GGAATCTGCCTAACA--------	-8	188	1.23
GGAATCTGCCTAACA-------G	-7	162	1.06
GGAATCTGCCTAACA--------	-9	157	1.03

M3814-2uM Syn24 56.94%	Type	Reads	Percentage
GGAATCTGCCTAACAGG|AGGTGG	WT	11072	43.06
GGAATCTGCCTAACAGG---TGG	-3	9883	18.99
GGAATCTGCCTAACA---------	-9	498	1.94
GGAATCTGCC--------------	-14	384	1.49
GGAATCTGCCTAACAGGgAGGTGG	+G	240	0.93

M3814-4uM Syn24 55.78%	Type	Reads	Percentage
GGAATCTGCCTAACAGG|AGGTGG	WT	16033	44.22
GGAATCTGCCTAACAGG---TGG	-3	6827	18.83
GGAATCTGCC--------------	-14	966	2.66
GGAATCTGCCTAACA-------G	-7	424	1.17

TSA-0.01uM Syn24 46.00%	Type	Reads	Percentage
GGAATCTGCCTAACAGG|AGGTGG	WT	22448	54.00
GGAATCTGCCTAACAGG---TGG	-3	4932	11.86
GGAATCTGCCTAACAG-AGGTGG	-G	1057	2.54
GGAATCTGCCTAACAGG--GTGG	-2	981	2.36
GGAATCTGCCTAACA---------	-9	378	0.91

TSA-0.05uM Syn24 36.48%	Type	Reads	Percentage
GGAATCTGCCTAACAGG|AGGTGG	WT	25671	63.52
GGAATCTGCCTAACAGG---TGG	-3	4282	10.60
GGAATCTGCCTAACAG-AGGTGG	-G	730	1.81
GGAATCTGCCTAACAGG--GTGG	-2	557	1.38
GGAATCTGCCTAACA----------	-9	440	1.09

TSA-0.1uM Syn24 18.34%	Type	Reads	Percentage
GGAATCTGCCTAACAGG|AGGTGG	WT	28530	81.66
GGAATCTGCCTAACAGG---TGG	-3	1705	4.88
GGAATCTGCCTAACAG-AGGTGG	-G	379	1.08
GGAATCTGCCTAACA-------G	-7	203	0.58
GGAATCTGCCTAACAGG--GTGG	-2	186	0.53

SCR7-1uM Syn24 42.25%	Type	Reads	Percentage
GGAATCTGCCTAACAGG|AGGTGG	WT	11843	57.75
GGAATCTGCCTAACAGG---TGG	-3	2674	13.04
GGAATCTGCCTAACAG-AGGTGG	-G	504	2.46
GGAATCTGCCTAACAGG--GTGG	-2	316	1.54
GGAATCTGCCTA-----AGGTGG	-5	152	0.74

SCR7-5uM Syn24 43.38%	Type	Reads	Percentage
GGAATCTGCCTAACAGG|AGGTGG	WT	14529	56.62
GGAATCTGCCTAACAGG---TGG	-3	3038	11.84
GGAATCTGCCTAACAG-AGGTGG	-G	704	2.74
GGAATCTGCCTAACAGG--GTGG	-2	373	1.45
GGAATCTGCCTA-----AGGTGG	-5	214	0.83

SCR7-10uM Syn24 42.57%	Type	Reads	Percentage
GGAATCTGCCTAACAGG|AGGTGG	WT	11036	57.43
GGAATCTGCCTAACAGG---TGG	-3	2157	11.22
GGAATCTGCCTAACAG-AGGTGG	-G	540	2.81
GGAATCTGCCTAACA--AGGTGG	-2	360	1.87
GGAATCTGCCTAACAGG--GTGG	-2	139	0.72
GGAATCTGCCTAACAGG-GGTGG	-A	138	0.72

P258-YW-W9-Syn26crCD326c-RNPko

KO Ctrl Syn26 89.97%

Sequence	Type	Reads	Percentage
CCCTCCCGCGCCCCTCT|TCTCGG	WT	727	10.03
CCCTCCCGCGCCCCTCTtTCTCGG	+T	1524	21.03
CCCTCCC------------------	>30	597	8.24
CCCTCCCGCGC------------	-21	436	6.02
CCCTCCCGCGCCCCTCT---CGG	-3	356	4.91
CCCTCCCGCGCCCCTCT-CTCGG	-T	320	4.42
CCCTCCCGC--------------	-23	141	1.95
CCCTCCCGCGCCCCTC-----GG	-5	118	1.63

P1667 Syn26 94.76% 52.71%

Sequence	Type	Reads	Percentage
CCCTCCCGCGCCCCTCT|TCTCGG	WT	202	5.24
CCCTCCCGCGCCCCTCTtTCTCGG	+T	531	13.77
CCCTCCCGCGCCCCTCT-CTCGG	-T	134	3.48
CCCTCCCGCGCCCCTCT---CGG	-3	71	1.84
CCCTCCC------------------	>30	70	1.82
CCCTCCCGCGC------------	-21	52	1.35
CCCTCCCGCGCCCCTC-----GG	-5	29	0.75
CCCTCCCGC--------------	-23	12	0.31

M3814-4uM Syn26 84.05%

Sequence	Type	Reads	Percentage
CCCTCCCGCGCCCCTCT|TCTCGG	WT	6574	15.95
CCCTCCCGCGCCCCTCTtTCTCGG	+T	6558	15.91
CCCTCCC------------------	>30	4480	10.87
CCCTCCCGCGC------------	-21	2433	5.90
CCCTCCCGCGCCCCTCT---CGG	-3	1456	3.53
CCCTCCCGCGCCCCTC-----GG	-5	614	1.49
CCCTCCCGC--------------	-23	522	1.27
CCCTCCCGCGCCCCTCT-CTCGG	-T	415	[illegible]

M3814-1uM Syn26 84.76%

Sequence	Type	Reads	Percentage
CCCTCCCGCGCCCCTCT|TCTCGG	WT	2191	15.24
CCCTCCC------------------	>30	1841	12.80
CCCTCCCGCGCCCCTCTtTCTCGG	+T	1347	9.37
CCCTCCCGCGC------------	-21	593	4.12
CCCTCCCGCGCCCCTCT---CGG	-3	579	4.03
CCCTCCCGC--------------	-23	316	2.20
CCCTCCCGCGCCCCTCT-CTCGG	-T	241	1.68
CCCTCCCGCGCCCCTC-----GG	-5	237	1.65

M3814-2uM Syn26 83.73%

Sequence	Type	Reads	Percentage
CCCTCCCGCGCCCCTCT|TCTCGG	WT	4072	16.27
CCCTCCCGCGCCCCTCTtTCTCGG	+T	2873	11.48
CCCTCCC------------------	>30	2850	11.39
CCCTCCCGCGCCCCTCT---CGG	-3	1617	6.46
CCCTCCCGCGC------------	-21	1410	5.64
CCCTCCCGCGCCCCTC-----GG	-5	710	2.84
CCCTCCCGCGCCCCTCT-CTCGG	-T	435	1.74
CCCTCCCGC--------------	-23	399	1.59

TSA-0.1uM Syn26 84.36%

Sequence	Type	Reads	Percentage
CCCTCCCGCGCCCCTCT|TCTCGG	WT	6310	15.64
CCCTCCCGCGCCCCTCTtTCTCGG	+T	12199	30.23
CCCTCCCGCGCCCCTCT-CTCGG	-T	2885	7.15
CCCTCCC------------------	>30	1980	4.91
CCCTCCCGCGC------------	-21	1187	2.94
CCCTCCCGCGCCCCTCT---CGG	-3	1154	2.86
CCCTCCCGC--------------	-23	551	1.37

TSA-0.01uM Syn26 89.42%

Sequence	Type	Reads	Percentage
CCCTCCCGCGCCCCTCT|TCTCGG	WT	5584	10.58
CCCTCCCGCGCCCCTCTtTCTCGG	+T	16074	30.44
CCCTCCC------------------	>30	3784	7.17
CCCTCCCGCGCCCCTCT-CTCGG	-T	2620	4.96
CCCTCCCGCGC------------	-21	1587	3.01
CCCTCCCGC--------------	-23	1143	2.16

TSA-0.05uM Syn26 90.19%

Sequence	Type	Reads	Percentage
CCCTCCCGCGCCCCTCT|TCTCGG	WT	4076	9.81
CCCTCCCGCGCCCCTCTtTCTCGG	+T	12847	30.93
CCCTCCCGCGCCCCTCT-CTCGG	-T	2718	6.54
CCCTCCC------------------	>30	2189	5.27
CCCTCCCGCGC------------	-21	1221	2.94
CCCTCCCGCGCCCCTCT---CGG	-3	941	2.27
CCCTCCCGC--------------	-23	606	1.49

SCR7-10uM Syn26 86.41%

Sequence	Type	Reads	Percentage
CCCTCCCGCGCCCCTCT|TCTCGG	WT	3149	13.59
CCCTCCCGCGCCCCTCTtTCTCGG	+T	4661	20.11
CCCTCCC------------------	>30	2147	9.26
CCCTCCCGCGCCCCTCT-CTCGG	-T	1307	5.64
CCCTCCCGCGC------------	-21	1107	4.78
CCCTCCCGCGCCCCTCT---CGG	-3	887	3.83
CCCTCCCGC--------------	-23	544	2.35
CCCTCCCGCGCCCCTC-----GG	-5	267	1.15

SCR7-1uM Syn26 86.83%

Sequence	Type	Reads	Percentage
CCCTCCCGCGCCCCTCT|TCTCGG	WT	4027	13.17
CCCTCCCGCGCCCCTCTtTCTCGG	+T	6554	21.44
CCCTCCC------------------	>30	2622	8.58
CCCTCCCGCGCCCCTCT-CTCGG	-T	1635	5.35
CCCTCCCGCGC------------	-21	1414	4.62
CCCTCCCGCGCCCCTCT---CGG	-3	1162	3.80
CCCTCCCGCGCCCCTC-----GG	-5	516	1.69
CCCTCCCGC--------------	-23	456	1.49

SCR7-5uM Syn26 87.57%

Sequence	Type	Reads	Percentage
CCCTCCCGCGCCCCTCT|TCTCGG	WT	3960	12.43
CCCTCCCGCGCCCCTCTtTCTCGG	+T	7327	22.99
CCCTCCC------------------	>30	2332	7.32
CCCTCCCGCGC------------	-21	1684	5.28
CCCTCCCGCGCCCCTCT-CTCGG	-T	1661	5.21
CCCTCCCGCGCCCCTCT---CGG	-3	1239	3.89
CCCTCCCGC--------------	-23	716	2.25
CCCTCCCGCGCCCCTC-----GG	-5	483	1.52

P260-YW-TC-Syn26crCD326c-RNPko

KO Ctrl Syn26 75.88%

Sequence	Type	Reads	Percentage
CCCTCCCGCGCCCCTCT|TCTCGG	WT	2286	24.12
CCCTCCC------------------	>30	1389	14.66
CCCTCCCGCGC-------------	-21	438	4.62
CCCT--------------------	>33	429	4.53
CCCTCCCGCGCCCCTCTtTCTCGG	+T	387	4.08
CCCTCCCG-----------------	>29	225	2.37
CCCTCCCGC----------------	-23	170	1.79
CCCTCCCGCGCCCCTCT-CTCGG	-T	138	1.46
CCCTCCCGCGCCCCTCT---CGG	-3	132	1.39

P1667 Syn26 82.97% 34.40%

Sequence	Type	Reads	Percentage
CCCTCCCGCGCCCCTCT|TCTCGG	WT	819	17.03
CCCTCCC------------------	>30	378	7.86
CCCTCCCGCGCCCCTCTtTCTCGG	+T	168	3.49
CCCTCCCGCGC-------------	-21	157	3.27
CCCT--------------------	>33	100	2.08
CCCTCCCGCGCCCCTCT---CGG	-3	56	1.16
CCCTCCCG-----------------	>29	56	1.16
CCCTCCCGCGCCCCTCT-CTCGG	-T	56	1.16
	-23	35	0.69

M3814-4uM Syn26 71.13%

Sequence	Type	Reads	Percentage
CCCTCCCGCGCCCCTCT|TCTCGG	WT	11183	28.87
CCCTCCC------------------	>30	7667	19.80
CCCTCCCGCGC-------------	-21	2355	6.08
CCCT--------------------	>33	2316	5.98
CCCTCCCG-----------------	>29	1290	3.33
CCCTCCCGC----------------	-23	660	1.70
CCCTCCCGCGCCCCTCT---CGG	-3	203	0.52

M3814-1uM Syn26 76.22%

Sequence	Type	Reads	Percentage
CCCTCCCGCGCCCCTCT|TCTCGG	WT	5265	23.78
CCCTCCC------------------	>30	4776	21.57
CCCT--------------------	>33	1410	6.37
CCCTCCCGCGC-------------	-21	1273	5.75
CCCTCCCG-----------------	>29	772	3.49
CCCTCCCGC----------------	-23	378	1.71
CCCTCCCGCGCCCCTCTtTCTCGG	+T	116	0.52
CCCTCCCGCGCCCCTCT---CGG	-3	[illegible]	0.50

M3814-2uM Syn26 73.59%

Sequence	Type	Reads	Percentage
CCCTCCCGCGCCCCTCT|TCTCGG	WT	9162	26.41
CCCTCCC------------------	>30	7248	20.89
CCCT--------------------	>33	2154	6.21
CCCTCCCGCGC-------------	-21	1930	5.56
CCCTCCCG-----------------	>29	1371	3.95
CCCTCCCGC----------------	-23	616	1.78
CCCTCCCGCGCCCCTCT---CGG	-3	177	0.51

TSA-0.01uM Syn26 74.95%

Sequence	Type	Reads	Percentage
CCCTCCCGCGCCCCTCT|TCTCGG	WT	11386	25.05
CCCTCCC------------------	>30	6914	15.21
CCCTCCCGCGC-------------	-21	2300	5.06
CCCTCCCGCGCCCCTCTtTCTCGG	+T	2187	4.81
CCCT--------------------	>33	1797	3.95
CCCTCCCG-----------------	>29	1035	2.28
CCCTCCCGCGCCCCTCT-CTCGG	-T	924	2.03
CCCTCCCGCGCCCCTCT---CGG	-3	867	1.91

TSA-0.05uM Syn26 60.82%

Sequence	Type	Reads	Percentage
CCCTCCCGCGCCCCTCT|TCTCGG	WT	13880	39.18
CCCTCCC------------------	>30	3831	34.13
CCCTCCCGCGC-------------	-21	1589	4.49
CCCTCCCGCGCCCCTCTtTCTCGG	+T	1377	3.89
CCCT--------------------	>33	1183	3.34
CCCTCCCG-----------------	>29	626	1.77
CCCTCCCGCGCCCCTCT-CTCGG	-T	568	1.60
CCCTCCCGCGCCCCTCT---CGG	-3	565	1.59

SCR7-1uM Syn26 73.78%

Sequence	Type	Reads	Percentage
CCCTCCCGCGCCCCTCT|TCTCGG	WT	10529	26.22
CCCTCCC------------------	>30	6278	15.64
CCCTCCCGCGC-------------	-21	2085	5.19
CCCT--------------------	>33	1869	4.66
CCCTCCCGCGCCCCTCTtTCTCGG	+T	1775	4.42
CCCTCCCGCGCCCCTCT---CGG	-3	832	2.07
CCCTCCCG-----------------	>29	801	2.00
CCCTCCCGC----------------	-23	664	1.65
CCCTCCCGCGCCCCTCT-CTCGG	-T	618	1.54

SCR7-5uM Syn26 71.27%

Sequence	Type	Reads	Percentage
CCCTCCCGCGCCCCTCT|TCTCGG	WT	14641	28.73
CCCTCCC------------------	>30	6921	13.58
CCCTCCCGCGC-------------	-21	2712	5.32
CCCTCCCGCGCCCCTCTtTCTCGG	+T	2188	4.29
CCCT--------------------	>33	1915	3.76
CCCTCCCG-----------------	>29	1103	2.16
CCCTCCCGCGCCCCTCT---CGG	-3	913	1.79
CCCTCCCGCGCCCCTCT-CTCGG	-T	816	1.60
CCCTCCCGC----------------	-23	718	1.41

TSA-0.1uM Syn26 35.04%

Sequence	Type	Reads	Percentage
CCCTCCCGCGCCCCTCT|TCTCGG	WT	29322	64.96
CCCTCCC------------------	>30	2984	6.61
CCCTCCCGCGCCCCTCTtTCTCGG	+T	1192	2.64
CCCTCCCGCGC-------------	-21	1048	2.32
CCCT--------------------	>33	842	1.87
CCCTCCCGCGCCCCTCT-CTCGG	-T	617	1.37
CCCTCCCGCGCCCCTCT---CGG	-3	606	1.34
CCCTCCCG-----------------	>29	558	1.24

SCR7-10uM Syn26 70.32%

Sequence	Type	Reads	Percentage
CCCTCCCGCGCCCCTCT|TCTCGG	WT	9716	29.68
CCCTCCC------------------	>30	4713	14.40
CCCTCCCGCGCCCCTCTtTCTCGG	+T	1473	4.50
CCCTCCCGCGC-------------	-21	1427	4.36
CCCT--------------------	>33	1180	3.61
CCCTCCCG-----------------	>29	701	2.14
CCCTCCCGCGCCCCTCT---CGG	-3	598	1.83
CCCTCCCGCGCCCCTCT-CTCGG	-T	507	1.55
CCCTCCCGC----------------	-23	400	1.22

P259-YW-W9-Syn30crCD326g-KO

KO Ctrl Syn30 83.23%

Sequence	Type	Reads	Percentage
CTGCGCGCGCGCCGAGA|AGAGGG	WT	3183	16.77
-30or>30	-30?	1705	8.98
CTGCGCGCGCGCCGAGA---GGG	-3	1666	8.78
CTGCGCG----------------	-21	1271	6.70
CTGCGCGCGCGCCGAGAaAGAGGG	+A	839	4.42
CTGCGCGCGCGCCGAG-AGAGGG	-A	754	3.97
CTGCG------------------	-23	558	2.94
CTGCGCGCGCGCCGAG-----GG	-5	352	1.85

P1667 Syn30 90.44% 54.32%

Sequence	Type	Reads	Percentage
CTGCGCGCGCGCCGAGA|AGAGGG	WT	526	9.56
CTGCGCGCGCGCCGAGAaAGAGGG	+A	237	4.31
CTGCGCGCGCGCCGAG-AGAGGG	-A	219	3.98
CTGCGCGCGCGCCGAGA---GGG	-3	167	3.03
-30or>30	-30?	128	2.33
CTGCGCG----------------	-21	109	1.98
CTGCG------------------	-23	57	1.04
CTGCGCGCGCGCCGAG-----GG	-5	52	0.94

M3814-4uM Syn30 93.79%

Sequence	Type	Reads	Percentage
CTGCGCGCGCGCCGAGA|AGAGGG	WT	2989	6.21
-30or>30	-30?	9545	19.83
CTGCGCG----------------	-21	4573	9.50
CTGCG------------------	-23	1809	3.76
CTGCGCGCGCGCCGAGA---GGG	-3	1639	3.41
CTGCGCGCGCGCCGAG-AGAGGG	-A	419	0.87
CTGCGCGCGCGCCGAGAaAGAGGG	+A	242	0.50
CTGCGCGCGCGCCGAG-----GG	-5	203	0.42

M3814-1uM Syn30 90.31%

Sequence	Type	Reads	Percentage
CTGCGCGCGCGCCGAGA|AGAGGG	WT	2080	9.69
-30or>30	-30?	4490	20.93
CTGCGCG----------------	-21	1320	6.15
CTGCGCGCGCGCCGAGA---GGG	-3	1069	4.98
CTGCGCGCGCGCCGAG-----GG	-5	263	1.23
CTGCGCGCGCGCCGAGAaAGAGGG	+A	219	1.02
CTGCGCGCGCGCCGAG-AGAGGG	-A	156	0.73

M3814-2uM Syn30 94.81%

Sequence	Type	Reads	Percentage
CTGCGCGCGCGCCGAGA|AGAGGG	WT	1874	5.19
-30or>30	-30?	8073	22.37
CTGCGCG----------------	-21	2583	7.16
CTGCGCGCGCGCCGAGA---GGG	-3	2023	5.61
CTGCG------------------	-23	1143	3.17
CTGCGCGCGCGCCGAG-----GG	-5	322	0.89
CTGCGCGCGCGCCGAG-AGAGGG	-A	[illegible]	0.73
CTGCGCGCGCGCCGAGAaAGAGGG	+A	218	0.60

TSA-0.1uM Syn30 78.38%

Sequence	Type	Reads	Percentage
CTGCGCGCGCGCCGAGA|AGAGGG	WT	12071	21.62
CTGCGCGCGCGCCGAGAaAGAGGG	+A	5884	10.54
CTGCGCGCGCGCCGAG-AGAGGG	-A	5225	9.36
-30or>30	-30?	3599	6.45
CTGCGCGCGCGCCGAGA---GGG	-3	2225	3.98
CTGCGCG----------------	-21	1703	3.05
CTGCG------------------	-23	903	1.62

TSA-0.01uM Syn30 84.12%

Sequence	Type	Reads	Percentage
CTGCGCGCGCGCCGAGA|AGAGGG	WT	7881	15.88
CTGCGCGCGCGCCGAGAaAGAGGG	+A	4636	9.34
-30or>30	-30?	4334	8.73
CTGCGCG----------------	-21	2846	5.74
CTGCGCGCGCGCCGAG-AGAGGG	-A	2392	4.82
CTGCGCGCGCGCCGAGA---GGG	-3	1846	3.72
CTGCG------------------	-23	1082	2.18

TSA-0.05uM Syn30 81.54%

Sequence	Type	Reads	Percentage
CTGCGCGCGCGCCGAGA|AGAGGG	WT	9444	18.46
CTGCGCGCGCGCCGAGAaAGAGGG	+A	4971	9.71
CTGCGCGCGCGCCGAG-AGAGGG	-A	4177	8.16
-30or>30	-30?	3314	6.48
CTGCGCGCGCGCCGAGA---GGG	-3	2475	4.84
CTGCGCG----------------	-21	2179	4.26
CTGCG------------------	-23	1096	2.14

SCR7-10uM Syn30 82.82%

Sequence	Type	Reads	Percentage
CTGCGCGCGCGCCGAGA|AGAGGG	WT	6138	17.18
CTGCGCGCGCGCCGAGA---GGG	-3	3429	9.60
-30or>30	-30?	3020	8.45
CTGCGCG----------------	-21	2364	6.62
CTGCGCGCGCGCCGAG-AGAGGG	-A	1586	4.44
CTGCGCGCGCGCCGAGAaAGAGGG	+A	1533	4.29
CTGCG------------------	-23	741	2.07
CTGCGCGCGCGCCGAG-----GG	-5	660	1.85

SCR7-1uM Syn30 81.26%

Sequence	Type	Reads	Percentage
CTGCGCGCGCGCCGAGA|AGAGGG	WT	8077	18.74
CTGCGCGCGCGCCGAGA---GGG	-3	4105	9.52
-30or>30	-30?	3623	8.41
CTGCGCG----------------	-21	2282	5.29
CTGCGCGCGCGCCGAGAaAGAGGG	+A	2280	5.29
CTGCGCGCGCGCCGAG-AGAGGG	-A	1790	4.15
CTGCGCGCGCGCCGAG-----GG	-5	981	2.28
CTGCG------------------	-23	861	2.00

SCR7-5uM Syn30 81.42%

Sequence	Type	Reads	Percentage
CTGCGCGCGCGCCGAGA|AGAGGG	WT	9618	18.58
-30or>30	-30?	4570	8.83
CTGCGCGCGCGCCGAGA---GGG	-3	4409	8.52
CTGCGCG----------------	-21	2804	5.42
CTGCGCGCGCGCCGAGAaAGAGGG	+A	2538	4.90
CTGCGCGCGCGCCGAG-AGAGGG	-A	2434	4.70
CTGCG------------------	-23	971	1.88
CTGCGCGCGCGCCGAG-----GG	-5	834	1.61

P261-YW-TC-Syn30crCD326g-KO

KO Ctrl Syn30 51.88%

	Type	Reads	Percentage
CTGCGCGCGCGCCGAGA|AGAGGG	WT	8436	48.12
-30or>30	-30?	2098	11.97
CTGCGCG------------------	-21	722	4.12
-33or>33	-33?	390	2.22
CTGCGCGCGCGCCGAGAaAGAGGG	+A	299	1.71
CTGCGCGCGCGCCGAGA---GGG	-3	296	1.69
CTGCGCGCGCGCCGAG-AGAGGG	-A	274	1.56
CTGCGCGCGCGCCGAG-----GG	-5	100	0.57

P1667 Syn30 60.22% 24.88%

	Type	Reads	Percentage
CTGCGCGCGCGCCGAGA|AGAGGG	WT	2080	39.78
-30or>30	-30?	319	6.10
CTGCGCG------------------	-21	115	2.20
CTGCGCGCGCGCCGAG-AGAGGG	-A	98	1.87
CTGCGCGCGCGCCGAGA---GGG	-3	79	1.51
CTGCGCGCGCGCCGAGAaAGAGGG	+A	73	1.40
-33or>33	-33?	44	0.84
CTGCGCGCGCGCCGAG-----GG	-5	42	0.80

M3814-4uM Syn30 58.77%

	Type	Reads	Percentage
CTGCGCGCGCGCCGAGA|AGAGGG	WT	22251	41.23
-30or>30	-30?	9221	17.08
CTGCGCG------------------	-21	2881	5.34
-33or>33	-33?	2189	4.06
CTGCGCGCGCGCCGAGA---GGG	-3	651	1.21
CTGCG--------------------	-23	639	1.18

M3814-1uM Syn30 58.50%

	Type	Reads	Percentage
CTGCGCGCGCGCCGAGA|AGAGGG	WT	12923	41.50
-30or>30	-30?	5608	18.01
CTGCGCG------------------	-21	1614	5.18
-33or>33	-33?	1163	3.73
CTGCG--------------------	-23	492	1.58
CTGCGCGCGCGCCGAGA---GGG	-3	412	1.32

M3814-2uM Syn30 58.57%

	Type	Reads	Percentage
CTGCGCGCGCGCCGAGA|AGAGGG	WT	9786	41.43
-30or>30	-30?	4557	19.29
-33or>33	-33?	1085	4.59
CTGCGCG------------------	-21	1021	4.32
CTGCG--------------------	-23	300	1.27
CTGCGCGCGCGCCGAGA---GGG	-3	234	0.99
CTGCGCGCGCGCCGAG-----GG	-5	136	0.58

TSA-0.1uM Syn30 22.23%

	Type	Reads	Percentage
CTGCGCGCGCGCCGAGA|AGAGGG	WT	44132	77.77
-30or>30	-30?	3250	5.73
CTGCGCGCGCGCCGAG-AGAGGG	-A	832	1.47
CTGCGCGCGCGCCGAGA---GGG	-3	781	1.38
CTGCGCGCGCGCCGAGAaAGAGGG	+A	504	0.89
CTGCGCG------------------	-21	446	0.79
-33or>33	-33?	425	0.75

TSA-0.01uM Syn30 53.18%

	Type	Reads	Percentage
CTGCGCGCGCGCCGAGA|AGAGGG	WT	14398	46.82
-30or>30	-30?	3570	11.61
CTGCGCG------------------	-21	1265	4.11
-33or>33	-33?	775	2.52
CTGCGCGCGCGCCGAGA---GGG	-3	649	2.11
CTGCGCGCGCGCCGAGAaAGAGGG	+A	598	1.94
CTGCGCGCGCGCCGAG-AGAGGG	-A	564	1.83
CTGCG--------------------	-23	303	0.99

TSA-0.05uM Syn30 43.93%

	Type	Reads	Percentage
CTGCGCGCGCGCCGAGA|AGAGGG	WT	30823	56.07
-30or>30	-30?	4726	8.60
CTGCGCG------------------	-21	2415	4.39
CTGCGCGCGCGCCGAG-AGAGGG	-A	1001	1.82
CTGCGCGCGCGCCGAGA---GGG	-3	984	1.79
-33or>33	-33?	943	1.72
CTGCGCGCGCGCCGAGAaAGAGGG	+A	788	1.43
CTGCG--------------------	-23	523	0.95

SCR7-10uM Syn30 46.59%

	Type	Reads	Percentage
CTGCGCGCGCGCCGAGA|AGAGGG	WT	11751	53.41
-30or>30	-30?	2224	10.11
CTGCGCG------------------	-21	582	2.65
CTGCGCGCGCGCCGAGA---GGG	-3	541	2.46
-33or>33	-33?	447	2.03
CTGCGCGCGCGCCGAG-AGAGGG	-A	435	1.98
CTGCGCGCGCGCCGAGAaAGAGGG	+A	419	1.90
CTGCG--------------------	-23	172	0.78

SCR7-1uM Syn30 50.54%

	Type	Reads	Percentage
CTGCGCGCGCGCCGAGA|AGAGGG	WT	16293	49.46
-30or>30	-30?	3954	12.00
CTGCGCG------------------	-21	1101	3.34
CTGCGCGCGCGCCGAGA---GGG	-3	789	2.39
-33or>33	-33?	743	2.26
CTGCGCGCGCGCCGAG-AGAGGG	-A	566	1.72
CTGCGCGCGCGCCGAGAaAGAGGG	+A	535	1.62
CTGCG--------------------	-23	311	0.94

SCR7-5uM Syn30 50.06%

	Type	Reads	Percentage
CTGCGCGCGCGCCGAGA|AGAGGG	WT	15714	49.94
-30or>30	-30?	3221	10.24
CTGCGCG------------------	-21	1324	4.21
CTGCGCGCGCGCCGAGA---GGG	-3	695	2.21
-33or>33	-33?	573	1.82
CTGCGCGCGCGCCGAG-AGAGGG	-A	560	1.78
CTGCGCGCGCGCCGAGAaAGAGGG	+A	486	1.54
CTGCG--------------------	-23	292	0.93

P258-YW-W9-Syn60crMYH6b-RNPko

KO Ctrl Syn60 66.66%

Sequence	Type	Reads	Percentage
CAGTAGGGGGCCTGAGA | GGAGGG	WT	4836	33.34
CAGTAGGGGGCCTGAGAaGGAGGG	+A	3958	27.29
CAGT---------------AGGG	-15	465	3.21
CAGTAGGGGGCC-----------	-16	387	2.67
CAGTAGGGGGCCTGAG--GAGGG	-2	291	2.01
CAG---------------GAGGG	-15	277	1.91
CAGTAGGGGGCCTGAG-GGAGGG	-A	276	1.90
CAGTAGGGGGCCTGAG-----GG	-5	248	1.71
CAGTAGG------------AGGG	-12	197	1.36
CAGTAGG----------------	-16	189	1.30
CAGTAGGGGGCCTGAGA---GGG	-3	187	1.29
CAGTAGGGGGCCTGAGA-GAGGG	-G	150	1.03

M3814-1uM Syn60 65.79%

Sequence	Type	Reads	Percentage
CAGTAGGGGGCCTGAGA | GGAGGG	WT	7559	34.21
CAGTAGGGGGCCTGAGAaGGAGGG	+A	3372	15.26
CAGT---------------AGGG	-15	1477	6.68
CAGTAGGGGGCC-----------	-16	1062	4.81
CAGTAGGGGGCCTGAG-----GG	-5	806	3.65
CAG---------------GAGGG	-15	536	2.43
CAGTAGGG-----------AGGG	-11	386	1.75
CAGTAGG----------------	-16	282	1.28
CAGTAGG------------AGGG	-12	272	1.23
CAGTAGGGGGCCTGAGA---GGG	-3	244	1.10

TSA-0.01uM Syn60 81.28%

Sequence	Type	Reads	Percentage
CAGTAGGGGGCCTGAGA | GGAGGG	WT	9246	18.72
CAGTAGGGGGCCTGAGAaGGAGGG	+A	21382	43.28
CAGTAGGGGGCC-----------	-16	1462	2.96
CAGT---------------AGGG	-15	1021	2.07
CAGTAGGGGGCCTGAG--GAGGG	-2	974	1.97
CAGTAGGGGGCCTGAG-GGAGGG	-A	877	1.78
CAGTAGGGGGCCTGAGA---GGG	-3	794	1.61
CAGTAGG------------AGGG	-12	697	1.41
CAG---------------GAGGG	-15	644	1.60
CAGTAGGGGGCCTGAGA-GAGGG	-G	625	1.27
CAGTAGGGGGCCTGAG-----GG	-5	475	0.96

SCR7-1uM Syn60 63.99%

Sequence	Type	Reads	Percentage
CAGTAGGGGGCCTGAGA | GGAGGG	WT	14243	36.01
CAGTAGGGGGCCTGAGAaGGAGGG	+A	11143	28.18
CAGTAGGGGGCC-----------	-16	823	2.08
CAGT---------------AGGG	-15	759	1.92
CAG---------------GAGGG	-15	736	1.86
CAGTAGG----------------	-16	655	1.66
CAGTAGGGGGCCTGAG-----GG	-5	640	1.62
CAGTAGG------------AGGG	-12	597	1.51
CAGTAGGGGGCCTGAG-GGAGGG	-A	589	1.49
CAGTAGGGGGCCTGAGA---GGG	-3	459	1.16

P1656 Syn60 78.73% 32.10%

Sequence	Type	Reads	Percentage
CAGTAGGGGGCCTGAGA | GGAGGG	WT	2336	21.27
CAGTAGGGGGCCTGAGAaGGAGGG	+A	2653	24.15
CAGTAGGGGGCCTGAG-GGAGGG	-A	236	2.15
CAGTAGG------------AGGG	-12	136	1.24
CAGTAGGGGGCCTGAG--GAGGG	-2	122	1.11
CAGTAGGGGGCC-----------	-16	115	1.05
CAGTAGGGGGCCTGAGAtGGAGGG	+T	102	0.93
CAGTAGGGGGCCTGAGA---GGG	-3	100	0.91
CAGTAGGGGGCCTGAGA-GAGGG	-G	89	0.81
CAGT---------------AGGG	-15	80	0.73

M3814-2uM Syn60 69.40%

Sequence	Type	Reads	Percentage
CAGTAGGGGGCCTGAGA | GGAGGG	WT	10611	30.60
CAGTAGGGGGCCTGAGAaGGAGGG	+A	6467	18.65
CAGT---------------AGGG	-15	2174	6.27
CAGTAGGGGGCC-----------	-16	1813	5.23
CAG---------------GAGGG	-15	1471	4.24
CAGTAGGGGGCCTGAG-----GG	-5	1031	2.97
CAGTAGG------------AGGG	-12	1027	2.96
CAGTAGG----------------	-16	909	2.62

TSA-0.05uM Syn60 75.60%

Sequence	Type	Reads	Percentage
CAGTAGGGGGCCTGAGA | GGAGGG	WT	12825	24.40
CAGTAGGGGGCCTGAGAaGGAGGG	+A	22271	42.37
CAGTAGGGGGCCTGAG-GGAGGG	-A	1173	2.23
CAGTAGGGGGCCTGAG--GAGGG	-2	940	1.79
CAGTAGGGGGCC-----------	-16	653	1.24
CAGT---------------AGGG	-15	648	1.23
CAGTAGGGGGCCTGAGA---GGG	-3	641	1.22
CAGTAGG------------AGGG	-12	614	1.17
CAG---------------GAGGG	-15	586	1.11
CAGTAGGGGGCCTGAG-----GG	-5	520	0.99

SCR7-5uM Syn60 68.07%

Sequence	Type	Reads	Percentage
CAGTAGGGGGCCTGAGA | GGAGGG	WT	11541	31.93
CAGTAGGGGGCCTGAGAaGGAGGG	+A	10581	29.28
CAGT---------------AGGG	-15	1369	3.79
CAGTAGGGGGCC-----------	-16	891	2.47
CAGTAGGGGGCCTGAG-----GG	-5	803	2.22
CAG---------------GAGGG	-15	619	1.71
CAGTAGG----------------	-16	545	1.51
CAGTAGGGGGCCTGAG-GGAGGG	-A	513	1.42
CAGTAGGGGGCCTGAG--GAGGG	-2	407	1.13
CAGTAGG------------AGGG	-12	400	1.11

M3814-4uM Syn60 65.32%

Sequence	Type	Reads	Percentage
CAGTAGGGGGCCTGAGA | GGAGGG	WT	18108	34.68
CAGTAGGGGGCCTGAGAaGGAGGG	+A	9378	17.96
CAGT---------------AGGG	-15	3171	6.07
CAGTAGGGGGCC-----------	-16	3033	5.81
CAGTAGG----------------	-16	1864	3.57
CAG---------------GAGGG	-15	1173	2.25
CAGTAGG------------AGGG	-12	1007	1.93
CAGTAGGGGGCCTGAG-----GG	-5	944	1.81
CAGTAGGG-----------AGGG	-11	681	1.30
CTGCGCGCGCCGAG-AGAGGG	-A	625	1.20

TSA-0.1uM Syn60 72.10%

Sequence	Type	Reads	Percentage
CAGTAGGGGGCCTGAGA | GGAGGG	WT	13827	27.90
CAGTAGGGGGCCTGAGAaGGAGGG	+A	19104	38.55
CAGTAGGGGGCCTGAG-GGAGGG	-A	1324	2.68
CAGTAGGGGGCCTGAG--GAGGG	-2	915	1.85
CAGT---------------AGGG	-15	815	1.64
CAGTAGGGGGCCTGAGA-GAGGG	-G	746	1.51
CAGTAGGGGGCC-----------	-16	638	1.29
CAGTAGGGGGCCTGAG-----GG	-5	550	1.11
CAGTAGG------------AGGG	-12	420	0.85
CAGTAGGGGGCCTGAGA---GGG	-3	411	0.83

SCR7-10uM Syn60 65.07%

Sequence	Type	Reads	Percentage
CAGTAGGGGGCCTGAGA | GGAGGG	WT	8942	34.93
CAGTAGGGGGCCTGAGAaGGAGGG	+A	6784	26.50
CAGTAGGGGGCC-----------	-16	910	3.55
CAGT---------------AGGG	-15	748	2.92
CAG---------------GAGGG	-15	627	2.45
CAGTAGG----------------	-16	486	1.90
CAGTAGGGGGCCTGAG-GGAGGG	-A	403	1.57
CAGTAGGGGGCCTGAG--GAGGG	-2	365	1.43
CAGTAGG------------AGGG	-12	327	1.28
CAGTAGGGGGCCTGAG-----GG	-5	303	1.18

P260-YW-TC-Syn60crMYH6b-RNPko

KO Ctrl Syn60 24.90%	Type	Reads	Percentage
CAGTAGGGGGCCTGAGA|GGAGGG	WT	28470	75.10
CAGTAGGGGGCCTGAGAaGGAGGG	+A	1318	3.48
CAGTAGGGGGCC-----------	-16	1073	2.83
CAGT---------------AGGG	-15	972	2.56
CAGTAGG----------------	-16	587	1.55
CAGTAGGGGGCCTGAG-GGAGGG	-A	475	1.25
CAG---------------GAGGG	-15	386	1.02
CAGTAGGGGGCCTGAG--GAGGG	-2	213	0.56

M3814-1uM Syn60 33.32%	Type	Reads	Percentage
CAGTAGGGGGCCTGAGA|GGAGGG	WT	24796	66.68
CAGTAGGGGGCC-----------	-16	2471	6.64
CAGT---------------AGGG	-15	2324	6.25
CAGTAGG----------------	-16	1286	3.46
CAG---------------GAGGG	-15	850	2.29
CAGTAGGGGGCCTGAGAaGGAGGG	+A	202	0.54

TSA-0.01uM Syn60 28.03%	Type	Reads	Percentage
CAGTAGGGGGCCTGAGA|GGAGGG	WT	35566	71.97
CAGTAGGGGGCCTGAGAaGGAGGG	+A	2071	4.19
CAGTAGGGGGCC-----------	-16	1734	3.51
CAGT---------------AGGG	-15	1138	2.30
CAGTAGG----------------	-16	859	1.74
CAG---------------GAGGG	-15	586	1.19
CAGTAGGGGGCCTGAG-GGAGGG	-A	573	1.16
CAGTAGGGGGCCTGAG--GAGGG	-2	267	0.54

SCR7-1uM Syn60 24.92%	Type	Reads	Percentage
CAGTAGGGGGCCTGAGA|GGAGGG	WT	26845	75.08
CAGTAGGGGGCCTGAGAaGGAGGG	+A	1276	3.57
CAGTAGGGGGCC-----------	-16	1028	2.88
CAGT---------------AGGG	-15	967	2.70
CAGTAGG----------------	-16	579	1.62
CAGTAGGGGGCCTGAG-GGAGGG	-A	383	1.07
CAG---------------GAGGG	-15	352	0.98
CAGTAGGGGGCCTGAG--GAGGG	-2	220	0.62

P1656 Syn60 29.11% 10.08%	Type	Reads	Percentage
CAGTAGGGGGCCTGAGA|GGAGGG	WT	16977	70.89
CAGTAGGGGGCCTGAGAaGGAGGG	+A	1051	4.39
CAGTAGGGGGCC-----------	-16	429	1.79
CAGTAGGGGGCCTGAG-GGAGGG	-A	376	1.57
CAGT---------------AGGG	-15	348	1.45
CAGTAGG----------------	-16	211	0.88
CAGTAGGGGGCCTGAG--GAGGG	-2	150	0.63

M3814-2uM Syn60 33.42%	Type	Reads	Percentage
CAGTAGGGGGCCTGAGA|GGAGGG	WT	28284	66.58
CAGTAGGGGGCC-----------	-16	3085	7.26
CAGT---------------AGGG	-15	2430	5.72
CAGTAGG----------------	-16	1385	3.26
CAG---------------GAGGG	-15	1015	2.39
CAGTAGGGGGCCTGAG-----GG	-5	261	0.61

TSA-0.05uM Syn60 20.30%	Type	Reads	Percentage
CAGTAGGGGGCCTGAGA|GGAGGG	WT	34284	79.70
CAGTAGGGGGCCTGAGAaGGAGGG	+A	1339	3.11
CAGT---------------AGGG	-15	943	2.19
CAGTAGGGGGCC-----------	-16	791	1.84
CAGTAGGGGGCCTGAG-GGAGGG	-A	402	0.93
CAGTAGG----------------	-16	361	0.84
CAG---------------GAGGG	-15	334	0.78

SCR7-5uM Syn60 23.35%	Type	Reads	Percentage
CAGTAGGGGGCCTGAGA|GGAGGG	WT	34918	76.65
CAGTAGGGGGCCTGAGAaGGAGGG	+A	1588	3.49
CAGT---------------AGGG	-15	1120	2.46
CAGTAGGGGGCC-----------	-16	1072	2.35
CAGTAGG----------------	-16	735	1.61
CAGTAGGGGGCCTGAG-GGAGGG	-A	578	1.27
CAG---------------GAGGG	-15	380	0.83

M3814-4uM Syn60 31.85%	Type	Reads	Percentage
CAGTAGGGGGCCTGAGA|GGAGGG	WT	33817	68.15
CAGTAGGGGGCC-----------	-16	3504	7.06
CAGT---------------AGGG	-15	2571	5.18
CAGTAGG----------------	-16	1713	3.45
CAG---------------GAGGG	-15	1137	2.29
CAGTAGGGGGCCTGAG-----GG	-5	308	0.62

TSA-0.1uM Syn60 7.38%	Type	Reads	Percentage
CAGTAGGGGGCCTGAGA|GGAGGG	WT	53060	92.62
CAGTAGGGGGCCTGAGAaGGAGGG	+A	926	1.62
CAGTAGGGGGCCTGAG-GGAGGG	-A	377	0.66
CAGTAGGGGGCC-----------	-16	367	0.64

SCR7-10uM Syn60 24.55%	Type	Reads	Percentage
CAGTAGGGGGCCTGAGA|GGAGGG	WT	27816	75.45
CAGTAGGGGGCCTGAGAaGGAGGG	+A	1279	3.47
CAGTAGGGGGCC-----------	-16	1082	2.93
CAGT---------------AGGG	-15	796	2.16
CAGTAGG----------------	-16	542	1.47
CAGTAGGGGGCCTGAG-GGAGGG	-A	440	1.19
CAG---------------GAGGG	-15	350	0.95

P259-YW-W9-Syn87crBCL11A5b-KO

KO Ctrl Syn87 94.45%

Sequence	Type	Reads	Percentage
GGGATTACCGAGTCACC|ACCAGG	WT	2318	5.55
GGGATTACCGAGTCACC---AGG	-3	11281	26.99
GGGATTACCGAGTCACCcACCAGG	+C	9870	23.62
GGGATTACCGAGTCAC-ACCAGG	-C	6931	16.58
GGGATT-----------ACCAGG	-11	2508	6.00
GGGATTACCGAGTCACCccACCAGG	+CC	761	1.82
GGGATTACCGAGT------CAGG	-6	622	1.49

P1771 Syn87 97.67% 27.42%

Sequence	Type	Reads	Percentage
GGGATTACCGAGTCACC|ACCAGG	WT	783	2.33
GGGATTACCGAGTCACCcACCAGG	+C	7326	21.82
GGGATTACCGAGTCACC---AGG	-3	6826	20.33
GGGATTACCGAGTCAC-ACCAGG	-C	4569	13.61
GGGATT-----------ACCAGG	-11	695	2.07
GGGATTACCGAGTCACCccACCAGG	+CC	428	1.27
GGGATTACCGAGT------CAGG	-6	134	0.40

M3814-4uM Syn87 93.62%

Sequence	Type	Reads	Percentage
GGGATTACCGAGTCACC|ACCAGG	WT	3173	6.38
GGGATT-----------ACCAGG	-11	13341	26.84
GGGATTACCGAGTCACC---AGG	-3	7283	14.65
GGGATTACCGAGTCACCcACCAGG	+C	3060	6.16
GGGATTACCGAGT------CAGG	-6	2891	5.82
GGGATTACCGAGTCAC-ACCAGG	-C	1797	3.62
GGGATTAC---------------	-15	1044	2.10
GGGATTA----------------	-16	876	1.76
GGGATTACCG----------AGG	-10	786	1.58

M3814-1uM Syn87 89.31%

Sequence	Type	Reads	Percentage
GGGATTACCGAGTCACC|ACCAGG	WT	4736	10.69
GGGATT-----------ACCAGG	-11	11120	25.11
GGGATTACCGAGTCACC---AGG	-3	6952	15.70
GGGATTACCGAGT------CAGG	-6	3363	7.59
GGGATTACCGAGTCACCcACCAGG	+C	2214	5.00
GGGATTACCGAGTCAC-ACCAGG	-C	1416	3.20
GGGATTAC---------------	-15	885	2.00
GGGATTACCG----------AGG	-10	863	1.9[illegible]

M3814-2uM Syn87 95.98%

Sequence	Type	Reads	Percentage
GGGATTACCGAGTCACC|ACCAGG	WT	2318	4.02
GGGATT-----------ACCAGG	-11	14437	25.06
GGGATTACCGAGTCACC---AGG	-3	8723	15.14
GGGATTACCGAGT------CAGG	-6	4261	7.40
GGGATTACCGAGTCACCcACCAGG	+C	3498	6.07
GGGATTACCGAGTCAC-ACCAGG	-C	2869	4.98
GGGATTAC---------------	-15	1158	2.01
GGGATTACCG----------AGG	-10	824	1.43

TSA-0.1uM Syn87 95.44%

Sequence	Type	Reads	Percentage
GGGATTACCGAGTCACC|ACCAGG	WT	2264	4.56
GGGATTACCGAGTCACCcACCAGG	+C	13541	27.30
GGGATTACCGAGTCACC---AGG	-3	12302	24.80
GGGATTACCGAGTCAC-ACCAGG	-C	8608	17.35
GGGATT-----------ACCAGG	-11	2189	4.41
GGGATTACCGAGTCACCccACCAGG	+CC	924	1.86
GGGATTACCGAGT------CAGG	-6	640	1.29

TSA-0.01uM Syn87 95.27%

Sequence	Type	Reads	Percentage
GGGATTACCGAGTCACC|ACCAGG	WT	2350	4.73
GGGATTACCGAGTCACCcACCAGG	+C	12996	26.16
GGGATTACCGAGTCACC---AGG	-3	11262	22.67
GGGATTACCGAGTCAC-ACCAGG	-C	8930	17.98
GGGATT-----------ACCAGG	-11	2925	5.89
GGGATTACCGAGT------CAGG	-6	886	1.78
GGGATTACCGAGTCACCccACCAGG	+CC	812	1.63

TSA-0.05uM Syn87 97.02%

Sequence	Type	Reads	Percentage
GGGATTACCGAGTCACC|ACCAGG	WT	1480	2.98
GGGATTACCGAGTCACCcACCAGG	+C	13000	26.13
GGGATTACCGAGTCACC---AGG	-3	12692	25.51
GGGATTACCGAGTCAC-ACCAGG	-C	9456	19.01
GGGATT-----------ACCAGG	-11	2884	5.80
GGGATTACCGAGTCACCccACCAGG	+CC	644	1.29
GGGATTACCGAGT------CAGG	-6	508	1.02

SCR7-10uM Syn87 93.69%

Sequence	Type	Reads	Percentage
GGGATTACCGAGTCACC|ACCAGG	WT	3700	6.31
GGGATTACCGAGTCACC---AGG	-3	14578	24.88
GGGATTACCGAGTCACCcACCAGG	+C	14063	24.00
GGGATTACCGAGTCAC-ACCAGG	-C	9309	15.88
GGGATT-----------ACCAGG	-11	4078	6.96
GGGATTACCGAGT------CAGG	-6	974	1.66
GGGATTACCGAGTCACCccACCAGG	+CC	850	1.45

SCR7-1uM Syn87 93.88%

Sequence	Type	Reads	Percentage
GGGATTACCGAGTCACC|ACCAGG	WT	3045	6.12
GGGATTACCGAGTCACC---AGG	-3	12685	25.49
GGGATTACCGAGTCACCcACCAGG	+C	11853	23.82
GGGATTACCGAGTCAC-ACCAGG	-C	8465	17.01
GGGATT-----------ACCAGG	-11	3142	6.31
GGGATTACCGAGTCACCccACCAGG	+CC	911	1.83
GGGATTACCGAGT------CAGG	-6	836	1.68

SCR7-5uM Syn87 93.52%

Sequence	Type	Reads	Percentage
GGGATTACCGAGTCACC|ACCAGG	WT	3217	6.48
GGGATTACCGAGTCACC---AGG	-3	12216	24.61
GGGATTACCGAGTCACCcACCAGG	+C	11698	23.57
GGGATTACCGAGTCAC-ACCAGG	-C	8234	16.59
GGGATT-----------ACCAGG	-11	3334	6.72
GGGATTACCGAGT------CAGG	-6	1086	2.19
GGGATTACCGAGTCACCccACCAGG	+CC	854	1.72

P261-YW-TC-Syn87crBCL11A5b-KO

KO Ctrl Syn87 67.90%	Type	Reads	Percentage
GGGATTACCGAGTCACC|ACCAGG	WT	12112	32.10
GGGATTACCGAGTCAC-ACCAGG	-C	8227	21.80
GGGATTACCGAGTCACC---AGG	-3	4695	12.44
GGGATT-----------ACCAGG	-11	2959	7.84
GGGATTACCGAGT------CAGG	-6	1047	2.77
GGGATTACCGAGTCACCcACCAGG	+C	1002	2.66

P1771 Syn87 79.54% 14.75%	Type	Reads	Percentage
GGGATTACCGAGTCACC|ACCAGG	WT	6731	20.46
GGGATTACCGAGTCAC-ACCAGG	-C	7473	22.72
GGGATTACCGAGTCACC---AGG	-3	4679	14.23
GGGATT-----------ACCAGG	-11	1956	5.95
GGGATTACCGAGTCACCcACCAGG	+C	953	2.90
GGGATTACCGAGT------CAGG	-6	666	2.02

M3814-4uM Syn87 60.74%	Type	Reads	Percentage
GGGATTACCGAGTCACC|ACCAGG	WT	19427	39.26
GGGATT-----------ACCAGG	-11	7891	15.95
GGGATTACCGAGT------CAGG	-6	3945	7.97
GGGATTACCGAGTCACC---AGG	-3	2496	5.04
GGGATTACCGAGTCAC-ACCAGG	-C	832	1.68
GGGATTAC---------------	-15	507	1.02

M3814-1uM Syn87 63.23%	Type	Reads	Percentage
GGGATTACCGAGTCACC|ACCAGG	WT	11498	36.77
GGGATT-----------ACCAGG	-11	4813	15.39
GGGATTACCGAGT------CAGG	-6	2786	8.91
GGGATTACCGAGTCACC---AGG	-3	1861	5.95
GGGATTACCGAGTCAC-ACCAGG	-C	935	2.99

M3814-2uM Syn87 60.70%	Type	Reads	Percentage
GGGATTACCGAGTCACC|ACCAGG	WT	17054	39.30
GGGATT-----------ACCAGG	-11	7452	17.17
GGGATTACCGAGT------CAGG	-6	3956	9.12
GGGATTACCGAGTCACC---AGG	-3	2125	4.90
GGGATTACCGAGTCAC-ACCAGG	-C	740	1.71

TSA-0.1uM Syn87 24.08%	Type	Reads	Percentage
GGGATTACCGAGTCACC|ACCAGG	WT	37712	75.92
GGGATTACCGAGTCAC-ACCAGG	-C	3956	7.96
GGGATTACCGAGTCACC---AGG	-3	2541	5.12
GGGATT-----------ACCAGG	-11	1141	2.30
GGGATTACCGAGTCACCcACCAGG	+C	343	0.69
GGGATTACCGAGT------CAGG	-6	306	0.62

TSA-0.01uM Syn87 68.06%	Type	Reads	Percentage
GGGATTACCGAGTCACC|ACCAGG	WT	15840	31.94
GGGATTACCGAGTCAC-ACCAGG	-C	9940	20.04
GGGATTACCGAGTCACC---AGG	-3	5975	12.05
GGGATT-----------ACCAGG	-11	4112	8.29
GGGATTACCGAGT------CAGG	-6	1674	3.38
GGGATTACCGAGTCACCcACCAGG	+C	1366	2.75

TSA-0.05uM Syn87 58.46%	Type	Reads	Percentage
GGGATTACCGAGTCACC|ACCAGG	WT	20641	41.54
GGGATTACCGAGTCAC-ACCAGG	-C	8389	16.88
GGGATTACCGAGTCACC---AGG	-3	5956	11.99
GGGATT-----------ACCAGG	-11	3869	7.79
GGGATTACCGAGTCACCcACCAGG	+C	1238	2.49
GGGATTACCGAGT------CAGG	-6	1196	2.41

SCR7-10uM Syn87 68.88%	Type	Reads	Percentage
GGGATTACCGAGTCACC|ACCAGG	WT	15496	31.12
GGGATTACCGAGTCAC-ACCAGG	-C	10940	21.97
GGGATTACCGAGTCACC---AGG	-3	6243	12.54
GGGATT-----------ACCAGG	-11	4082	8.20
GGGATTACCGAGT------CAGG	-6	1437	2.89
GGGATTACCGAGTCACCcACCAGG	+C	1286	2.58

SCR7-1uM Syn87 69.91%	Type	Reads	Percentage
GGGATTACCGAGTCACC|ACCAGG	WT	15558	30.09
GGGATTACCGAGTCAC-ACCAGG	-C	11062	21.39
GGGATTACCGAGTCACC---AGG	-3	6910	13.36
GGGATT-----------ACCAGG	-11	4490	8.68
GGGATTACCGAGT------CAGG	-6	1487	2.88
GGGATTACCGAGTCACCcACCAGG	+C	1310	2.53

SCR7-5uM Syn87 70.34%	Type	Reads	Percentage
GGGATTACCGAGTCACC|ACCAGG	WT	14719	29.66
GGGATTACCGAGTCAC-ACCAGG	-C	10907	21.98
GGGATTACCGAGTCACC---AGG	-3	6748	13.60
GGGATT-----------ACCAGG	-11	4023	8.11
GGGATTACCGAGT------CAGG	-6	1315	2.65
GGGATTACCGAGTCACCcACCAGG	+C	1194	2.41

P51-P55 Syn20-crAAVS1c RNP KO

4h (P51-BC7) 21.7%	Type	Reads	Percentage
TCTAACCCCCACCTCCT|GTTAGG	WT		
TCTAACCCCCACCTCCTtGTTAGG	+1	7022	16.0%
TCTAACCCCCACCTCCTctGTTAGG	+2	201	0.5%
TCTAACCCCCACCTCC--TTAGG	-2	149	0.3%
TCTAACCCCCACCTCCTgGTTAGG	+1	124	0.3%
TCTAACCCCCACCTCC-GTTAGG	-1	111	0.3%
TCTAACCCCCACCTC-TGTTAGG	-1	64	0.1%
TCTAACCCCCACCTCCcTGTTAGG	+1	56	0.1%

8h (P52-BC7) 55.6%	Type	Reads	Percentage
TCTAACCCCCACCTCCT|GTTAGG	WT		
TCTAACCCCCACCTCCTtGTTAGG	+1	10814	40.8%
TCTAACCCCCACCTCCTctGTTAGG	+2	318	1.2%
TCTAACCCCCACCTCC--TTAGG	-2	254	1.0%
TCTAACCCCCACCTCCTgGTTAGG	+1	148	0.6%
TCTAACCCCCACCTC-TGTTAGG	-1	141	0.5%
TCTAACCCCCACCTCC-GTTAGG	-1	140	0.5%
TCTAACCCCCACCTCCT-TTAGG	-1	128	0.5%
TCTAACCCCCACCTCCcTGTTAGG	+1	76	0.3%
TCTAACCCCCACCT---GTTAGG	-3	61	0.2%

12h (P53-BC7) 62.9%	Type	Reads	Percentage
TCTAACCCCCACCTCCT|GTTAGG	WT		
TCTAACCCCCACCTCCTtGTTAGG	+1	14221	41.4%
TCTAACCCCCACCTCC--TTAGG	-2	358	1.0%
TCTAACCCCCACCTCCTgGTTAGG	+1	353	1.0%
TCTAACCCCCACCTCCTctGTTAGG	+2	320	0.9%
TCTAACCCCCACCTCC-GTTAGG	-1	228	0.7%
TCTAACCCCCACCTC-TGTTAGG	-1	178	0.5%
TCTAACCCCCACCTCCT-TTAGG	-1	142	0.4%
TCTAACCCCCA---------------CCT	-15	124	0.4%

24h (P54-BC7) 77.4%	Type	Reads	Percentage
TCTAACCCCCACCTCCT|GTTAGG	WT		
TCTAACCCCCACCTCCTtGTTAGG	+1	7579	52.8%
TCTAACCCCCACCTCC--TTAGG	-2	195	1.4%
TCTAACCCCCACCTCCTgGTTAGG	+1	183	1.3%
TCTAACCCCCACCTCCTctGTTAGG	+2	166	1.2%
TCTAACCCCCACCTCC-GTTAGG	-1	134	0.9%
TCTAACCCCCA---------------CCT	-15	116	0.8%
TCTAACCCCCACCTC-TGTTAGG	-1	84	0.6%
TCTAACCCCCACCT---GTTAGG	-3	77	0.5%
TCTAACCCCCACCTCCT-TTAGG	-1	66	0.5%

48h (P55-BC7) 75.9%	Type	Reads	Percentage
TCTAACCCCCACCTCCT|GTTAGG	WT		
TCTAACCCCCACCTCCTtGTTAGG	+1	1571	50.1%
TCTAACCCCCACCTCCTgGTTAGG	+1	53	1.7%
TCTAACCCCCACCTCC--TTAGG	-2	46	1.5%
TCTAACCCCCA---------------CCT	-15	43	1.4%
TCTAACCCCCACCTCCTctGTTAGG	+2	36	1.1%
TCTAACCCCCACCT---GTTAGG	-3	28	0.9%
TCTAACCCCCACCTCC-GTTAGG	-1	22	0.7%
TCTAACCCCCACCTC-TGTTAGG	-1	21	0.7%
TCTAACCCCCACCTCCT-TTAGG	-1	17	0.5%

P51-P55 Syn20-crAAVS1c scAAV6 KI

4h (P51-BC1) 20.7% 1.3%	Type	Reads	Percentage
TCTAACCCCCACCTCCT|GTTAGG	WT		
TCTAACCCCCACCTCCTtGTTAGG	+1	6514	14.0%
TCTAACCCACGCGTAGTTTAAACTTAGGCAGATTCCTT	HDR	606	1.3%
TCTAACCCCCACCTCCTctGTTAGG	+2	204	0.4%
TCTAACCCCCACCTCCTgGTTAGG	+1	132	0.3%
TCTAACCCCCACCTCC--TTAGG	-2	98	0.2%
TCTAACCCCCACCTCCcTGTTAGG	+1	67	0.1%
TCTAACCCCCACCTCCTcGTTAGG	+1	57	0.1%
TCTAACCCCCACCTCCT-TTAGG	-1	57	0.1%
TCTAA[illegible]C-GTTAGG	-1	48	0.1%

8h (P52-BC1) 58.3% 17.7%	Type	Reads	Percentage
TCTAACCCCCACCTCCT|GTTAGG	WT		
TCTAACCCCCACCTCCTtGTTAGG	+1	13652	25.0%
TCTAACCCACGCGTAGTTTAAACTTAGGCAGATTCCTT	HDR	9662	17.7 %
TCTAACCCCCACCTCC--TTAGG	-2	416	0.8%
TCTAACCCCCACCTCCTctGTTAGG	+2	407	0.7%
TCTAACCCCCACCTCCTgGTTAGG	+1	374	0.7%
TCTAACCCCCACCTCC-GTTAGG	-1	225	0.4%
TCTAACCCCCACCTCCcTGTTAGG	+1	183	0.3%
TCTAA[illegible]C-TGTTAGG	-1	183	0.3%
TCTAACCCCCACCTCCT-TTAGG	-1	155	0.3%

12h (P53-BC1) 66.4% 24.1%	Type	Reads	Percentage
TCTAACCCCCACCTCCT|GTTAGG	WT		
TCTAACCCCCACCTCCTtGTTAGG	+1	8124	25.1%
TCTAACCCACGCGTAGTTTAAACTTAGGCAGATTCCTT	HDR	7801	24.1%
TCTAACCCCCACCTCCTgGTTAGG	+1	359	1.1%
TCTAACCCCCACCTCCTctGTTAGG	+2	223	0.7%
TCTAACCCCCACCTCC--TTAGG	-2	222	0.7%
TCTAACCCCCACCTC-TGTTAGG	-1	168	0.5%
TCTAACCCCCACCTCC-GTTAGG	-1	152	0.5%
TCTAACCCCCACCTCCT-TTAGG	-1	138	0.4%

24h (P54-BC1) 82.6% 32.6%	Type	Reads	Percentage
TCTAACCCCCACCTCCT|GTTAGG	WT		
TCTAACCCACGCGTAGTTTAAACTTAGGCAGATTCCTT	HDR	12249	32.6%
TCTAACCCCCACCTCCTtGTTAGG	+1	11073	29.5%
TCTAACCCCCACCTCCTgGTTAGG	+1	507	1.3%
TCTAACCCCCACCTCC--TTAGG	-2	350	0.9%
TCTAACCCCCACCTCCTctGTTAGG	+2	249	0.7%
TCTAACCCCCACCTC-TGTTAGG	-1	170	0.5%
TCTAACCCCCACCTCC-GTTAGG	-1	162	0.4%
TCTAACCCCCACCT---GTTAGG	-3	139	0.4%

48h (P55-BC1) 86.2% 35.5%	Type	Reads	Percentage
TCTAACCCCCACCTCCT|GTTAGG	WT		
TCTAACCCACGCGTAGTTTAAACTTAGGCAGATTCCTT	HDR	7171	35.5%
TCTAACCCCCACCTCCTtGTTAGG	+1	6207	30.8%
TCTAACCCCCACCTCCTgGTTAGG	+1	240	1.2%
TCTAACCCCCACCTCCTctGTTAGG	+2	168	0.8%
TCTAACCCCCACCTCC--TTAGG	-2	122	0.6%
TCTAACCCCCACCTCC-GTTAGG	-1	118	0.6%
TCTAACCCCCACCT---GTTAGG	-3	118	0.6%
TCTAACCCCCACCTC-TGTTAGG	-1	108	0.5%
TCTAACCCCCA[illegible]	-15	89	[illegible]

P51-P55 Syn21-crAAVS1d RNP KO

4h (P51-BC8) 2.3%	Type	Reads	Percentage
ATCTGCCTAACAGGAGG|TGGGGG	WT		
ATCTGCCTAACAGGAGG-GGGGG	-1	187	0.5%
ATCTGCCTAACAGGAGGT-GGGG	-1	141	0.4%
ATCTGCCTAACAGGAGGgTGGGGG	+1	107	0.3%
ATCTGCCTAACAGGA--TGGGGG	-2	48	0.1%

8h (P52-BC8) 8.4%	Type	Reads	Percentage
ATCTGCCTAACAGGAGG|TGGGGG	WT		
ATCTGCCTAACAGGAGG-GGGGG	-1	352	1.2%
ATCTGCCTAACAGGAGGgTGGGGG	+1	264	0.9%
ATCTGCCTAACAGGA--TGGGGG	-2	161	0.6%
ATCTGCCTAACAGGAG-TGGGGG	-1	114	0.4%
ATCTGCCTAACAGGA------GGT	-6	87	0.3%
ATCTGCCTAACAGGAGG--GGGG	-2	70	0.2%
ATCTGCCTAAC------TGGGGG	-6	55	0.2%
ATCTGCCTAACAGGA-----GGG	-5	50	0.2%
ATCTGCCTAACAG----TGGGGG	-4	49	0.2%

12h (P53-BC8) 11.9%	Type	Reads	Percentage
ATCTGCCTAACAGGAGG|TGGGGG	WT		
ATCTGCCTAACAGGAGG-GGGGG	-1	384	1.2%
ATCTGCCTAACAGGAGGgTGGGGG	+1	315	1.0%
ATCTGCCTAACAGGAG-TGGGGG	-1	199	0.6%
ATCTGCCTAACAGGA--TGGGGG	-2	185	0.6%
ATCTGCCTAACAGGA------GGT	-6	143	0.5%
ATCTGCCTAACAGGAGG--GGGG	-2	81	0.3%
ATCTGCCTAACAGGAGGT-GGGG	-1	76	0.2%
ATCTGCCTAACAGGA---GGGGG	-3	75	0.2%
ATCTGCCTAACAGGA-----GGG	-5	69	0.2%

24h (P54-BC8) 17.8%	Type	Reads	Percentage
ATCTGCCTAACAGGAGG|TGGGGG	WT		
ATCTGCCTAACAGGAGG-GGGGG	-1	245	1.7%
ATCTGCCTAACAGGAGGgTGGGGG	+1	182	1.2%
ATCTGCCTAACAGGA--TGGGGG	-2	151	1.0%
ATCTGCCTAACAGGA------GGT	-6	147	1.0%
ATCTGCCTAACAGGA-----GGG	-5	102	0.7%
ATCTGCCTAACAGGAG-TGGGGG	-1	92	0.6%
ATCTGCCTAACAGGA---GGGGG	-3	53	0.4%
ATCTGCCTAACAGGA----GGGG	-4	51	0.3%
ATCTGCCTAACAGG---TGGGGG	-3	47	0.3%

48h (P55-BC8) 21.9%	Type	Reads	Percentage
ATCTGCCTAACAGGAGG|TGGGGG	WT		
ATCTGCCTAACAGGA------GGT	-6	645	2.3%
ATCTGCCTAACAGGAGG-GGGGG	-1	486	1.7%
ATCTGCCTAACAGGA-----GGG	-5	328	1.2%
ATCTGCCTAACAGGA--TGGGGG	-2	328	1.2%
ATCTGCCTAACAGGAGGgTGGGGG	+1	319	1.1%
ATCTGCCTAACAGGA---GGGGG	-3	199	0.7%
ATCTGCCTAACAGGAG-TGGGGG	-1	176	0.6%
ATCTGCCTAACAGGA----GGGG	-4	139	0.5%
ATCTGCCTAACAGGAGGtTGGGGG	+1	121	0.4%

P51-P55 Syn21-crAAVS1d scAAV6 KI

4h (P51-BC2) 3.1%
0.7%

Sequence	Type	Reads	Percentage
ATCTGCCTAACAGGAGG|TGGGGG	WT		
ATCTGCCTAAGTTTAAACTACGCGTGGGTTAGACCC	HDR	419	0.7%
ATCTGCCTAACAGGAGGT-GGGG	-1	312	0.5%
ATCTGCCTAACAGGAGG-GGGGG	-1	230	0.4%
ATCTGCCTAACAGGAGGgTGGGGG	+1	152	0.3%

8h (P52-BC2) 18.3%
11.4%

Sequence	Type	Reads	Percentage
ATCTGCCTAACAGGAGG|TGGGGG	WT		
ATCTGCCTAAGTTTAAACTACGCGTGGGTTAGACCC	HDR	6684	11.4%
ATCTGCCTAACAGGAGG-GGGGG	-1	504	0.9%
ATCTGCCTAACAGGAGGgTGGGGG	+1	333	0.6%
ATCTGCCTAACAGGAGGT-GGGG	-1	271	0.5%
ATCTGCCTAACAGGA--TGGGGG	-2	218	0.4%
ATCTGCCTAACAGGAG-TGGGGG	-1	201	0.3%
ATCTGCCTAACA-----TGGGGG	-5	139	0.2%
ATCTGCCTAACAGGAGG--GGGG	-2	109	0.2%
ATCTGCCTAACAGGA---GGGGG	-3	[illegible]	0.2%

12h (P53-BC2) 25.5%
15.2%

Sequence	Type	Reads	Percentage
ATCTGCCTAACAGGAGG|TGGGGG	WT		
ATCTGCCTAAGTTTAAACTACGCGTGGGTTAGACCC	HDR	6148	15.2%
ATCTGCCTAACAGGAGG-GGGGG	-1	546	1.4%
ATCTGCCTAACAGGAGGgTGGGGG	+1	453	1.1%
ATCTGCCTAACAGGA--TGGGGG	-2	266	0.7%
ATCTGCCTAACAGGAG-TGGGGG	-1	218	0.5%
ATCTGCCTAACAGGAGGT-GGGG	-1	165	0.4%
ATCTGCCTAACAGGAGG--GGGG	-2	105	0.3%
ATCTGCCTAACAG----TGGGGG	-4	80	0.2%
ATCTGCCTAAC------TGGGGG	-6	75	0.2%

24h (P54-BC2) 38.7%
22.8%

Sequence	Type	Reads	Percentage
ATCTGCCTAACAGGAGG|TGGGGG	WT		
ATCTGCCTAAGTTTAAACTACGCGTGGGTTAGACCC	HDR	10440	22.8%
ATCTGCCTAACAGGAGG-GGGGG	-1	734	1.6%
ATCTGCCTAACAGGA--TGGGGG	-2	550	1.2%
ATCTGCCTAACAGGAGGgTGGGGG	+1	476	1.0%
ATCTGCCTAACAGGA------GGT	-6	282	0.6%
ATCTGCCTAACAGGA---GGGGG	-3	187	0.4%
ATCTGCCTAACAGG---TGGGGG	-3	148	0.3%
ATCTGCCTAACAG----TGGGGG	-4	139	0.3%

48h (P55-BC2) 43.6%
25.9%

Sequence	Type	Reads	Percentage
ATCTGCCTAACAGGAGG|TGGGGG	WT		
ATCTGCCTAAGTTTAAACTACGCGTGGGTTAGACCC	HDR	6674	25.9%
ATCTGCCTAACAGGAGG-GGGGG	-1	447	1.7%
ATCTGCCTAACAGGA--TGGGGG	-2	343	1.3%
ATCTGCCTAACAGGAGGgTGGGGG	+1	318	1.2%
ATCTGCCTAACAGGA------GGT	-6	150	0.6%
ATCTGCCTA--------TGGGGG	-8	148	0.6%
ATCTGCCTAACAGG---TGGGGG	-3	133	0.5%
ATCTGCCTAACAGGAG-TGGGGG	-1	104	0.4%

P51-P55 Syn22-crAAVS1e RNP KO

4h (P51-BC9) 6.4%	Type	Reads	Percentage
AATCTGCCTAACAGGAG|GTGGGG	WT		
AATCTGCCTAACAGGAGgGTGGGG	+1	1123	3.5%
AATCTGCCTAACAGGA-GTGGGG	-1	195	0.6%
AATCTGCCTAACAGG---TGGGG	-3	41	0.1%
AATCTGCCTAACAGGAGagGTGGGG	+2	32	0.1%

8h (P52-BC9) 19.0%	Type	Reads	Percentage
AATCTGCCTAACAGGAG|GTGGGG	WT		
AATCTGCCTAACAGGAGgGTGGGG	+1	2697	9.5%
AATCTGCCTAACAGGA-GTGGGG	-1	454	1.6%
AATCTGCCTAACAGG---TGGGG	-3	135	0.5%
AATCTGCCTAACAGGAGagGTGGGG	+2	88	0.3%
AATCTGCCTAACAGG--GTGGGG	-2	79	0.3%
AATCTGCCTAACAGG-GGTGGGG	-1	79	0.3%
AATCTGCCTAACAGGA-------C	-7	30	0.1%

12h (P53-BC9) 25.9%	Type	Reads	Percentage
AATCTGCCTAACAGGAG|GTGGGG	WT		
AATCTGCCTAACAGGAGgGTGGGG	+1	3072	10.8%
AATCTGCCTAACAGGA-GTGGGG	-1	564	2.0%
AATCTGCCTAACAGG---TGGGG	-3	177	0.6%
AATCTGCCTAACAGG--GTGGGG	-2	125	0.4%
AATCTGCCTAACAGG-GGTGGGG	-1	102	0.4%
AATCTGCCTAACAGGAGagGTGGGG	+2	86	0.3%
AATCTGCCTAACAGGA-------C	-7	51	0.2%
AATCTGC---------GGTGGGG	-9	45	0.2%

24h (P54-BC9) 37.0%	Type	Reads	Percentage
AATCTGCCTAACAGGAG|GTGGGG	WT		
AATCTGCCTAACAGGAGgGTGGGG	+1	3441	13.6%
AATCTGCCTAACAGGA-GTGGGG	-1	704	2.8%
AATCTGCCTAACAGG---TGGGG	-3	308	1.2%
AATCTGCCTAACAGG--GTGGGG	-2	167	0.7%
AATCTGCCTAACAGGAGagGTGGGG	+2	117	0.5%
AATCTGCCTAACAGG-GGTGGGG	-1	102	0.4%
AATCTGC---------GGTGGGG	-9	97	0.4%
AATCTGCCTAACA-------GGG	-7	73	0.3%
AATCTGCCTAACAGGA-------C	-7	72	0.3%
AATCTGCCTAACA--------GG	-8	68	0.3%

48h (P55-BC9) 39.1%	Type	Reads	Percentage
AATCTGCCTAACAGGAG|GTGGGG	WT		
AATCTGCCTAACAGGAGgGTGGGG	+1	1233	13.6%
AATCTGCCTAACAGGA-GTGGGG	-1	269	3.0%
AATCTGCCTAACAGG---TGGGG	-3	137	1.5%
AATCTGCCTAACA---------GG	-9	76	0.8%
AATCTGCCTAACAGGA------GGT	-6	58	0.6%
AATCTGCCTAACAGG--GTGGGG	-2	49	0.5%
AATCTGCCTAACA--------GG	-8	45	0.5%
AATCTGCCTAACA------GGGG	-6	44	0.5%
AATCTGCCTAACAGG-GGTGGGG	-1	42	0.5%
AATCTGCCTAACAGGA-------	-13	38	0.4%

P51-P55 Syn22-crAAVS1e scAAV6 KI

4h (P51-BC3) 6.1% 0.8%

Sequence	Type	Reads	Percentage
AATCTGCCTAACAGGAG|GTGGGG	WT		
AATCTGCCTAACAGGAGgGTGGGG	+1	1459	2.5%
AATCTGCCTAAGTTTAAACTACGCGT GGGTTAGACCC	HDR	467	0.8%
AATCTGCCTAACAGGA-GTGGGG	-1	267	0.5%
AATCTGCCTAACAGG---TGGGG	-3	135	0.2%
AATCTGCCTAACAGG--GTGGGG	-2	79	0.1%

8h (P52-BC3) 28.3% 12.3%

Sequence	Type	Reads	Percentage
AATCTGCCTAACAGGAG|GTGGGG	WT		
AATCTGCCTAAGTTTAAACTACGCG TGGGTTAGACCC	HDR	8476	12.3%
AATCTGCCTAACAGGAGgGTGGGG	+1	4931	7.2%
AATCTGCCTAACAGGA-GTGGGG	-1	862	1.3%
AATCTGCCTAACAGG---TGGGG	-3	303	0.4%
AATCTGCCTAACAGG-GGTGGGG	-1	163	0.2%
AATCTGCCTAACAGGAGagGTGGGG	+2	150	0.2%
AATCTGCCTAACAGG--GTGGGG	-2	136	0.2%
AATCTGCCTAACA---------GG	-9	96	0.1%

12h (P53-BC3) 36.2% 17.1%

Sequence	Type	Reads	Percentage
AATCTGCCTAACAGGAG|GTGGGG	WT		
AATCTGCCTAAGTTTAAACTACGCGT GGGTTAGACCC	HDR	6455	17.1%
AATCTGCCTAACAGGAGgGTGGGG	+1	2975	7.9%
AATCTGCCTAACAGGA-GTGGGG	-1	448	1.2%
AATCTGCCTAACAGG---TGGGG	-3	167	0.4%
AATCTGCCTAACAGG-GGTGGGG	-1	123	0.3%
AATCTGCCTAACAGG--GTGGGG	-2	85	0.2%
AATCTGCCTAACAGGAGagGTGGGG	+2	55	0.1%

24h (P54-BC3) 56.2% 28.5%

Sequence	Type	Reads	Percentage
AATCTGCCTAACAGGAG|GTGGGG	WT		
AATCTGCCTAAGTTTAAACTACGCGT GGGTTAGACCC	HDR	11182	28.5%
AATCTGCCTAACAGGAGgGTGGGG	+1	3836	9.8%
AATCTGCCTAACAGGA-GTGGGG	-1	668	1.7%
AATCTGCCTAACAGG---TGGGG	-3	360	0.9%
AATCTGCCTAACAGG--GTGGGG	-2	290	0.7%
AATCTGCCTAACAGG-GGTGGGG	-1	262	0.7%
AATCTGCC---------------	-23	140	0.4%

48h (P55-BC3) 61.2% 32.0%

Sequence	Type	Reads	Percentage
AATCTGCCTAACAGGAG|GTGGGG	WT		
AATCTGCCTAAGTTTAAACTACGCGT GGGTTAGACCC	HDR	7809	32.0%
AATCTGCCTAACAGGAGgGTGGGG	+1	2702	11.1%
AATCTGCCTAACAGGA-GTGGGG	-1	425	1.7%
AATCTGCCTAACAGG---TGGGG	-3	208	0.9%
AATCTGCCTAACAGG--GTGGGG	-2	159	0.7%
AATCTGCCTAACAGG-GGTGGGG	-1	108	0.4%
AATCTGCCTAACAGGA--------C	-7	82	0.3%
AATCTGCCTAACAGGAGagGTGGGG	+2	70	[illegible]

P51-P55 Syn23-crAAVS1f RNP KO

4h (P51-BC10) 3.4%	Type	Reads	Percentage
GAATCTGCCTAACAGGA|GGTGGG	WT		
GAATCTGCCTAACAGGAaGGTGGG	+1	227	0.7%
GAATCTGCCTAACAGG---TGGG	-3	133	0.4%
GAATCTGCCTAACAGGA-GTGGG	-1	114	0.3%
GAATCTGCCTAACAGG-GGTGGG	-1	55	0.2%
GAATCTGCCTAACAGGAgGGTGGG	+1	40	0.1%

8h (P52-BC10) 12.6%	Type	Reads	Percentage
GAATCTGCCTAACAGGA|GGTGGG	WT		
GAATCTGCCTAACAGGAaGGTGGG	+1	649	2.3%
GAATCTGCCTAACAGG---TGGG	-3	379	1.3%
GAATCTGCCTAACAGGA-GTGGG	-1	264	0.9%
GAATCTGCCTAACAGG-GGTGGG	-1	207	0.7%
GAATCTGCCTAACAGG--GTGGG	-2	166	0.6%
GAATCTGCCTAACAGGAgGGTGGG	+1	94	0.3%
GAATCTGCCTAACAGGA--TGGG	-2	50	0.2%
GAATCTGCC--------GGTGGG	-8	43	0.2%
GAATCTGCCTAACAGGA---GGG	-3	43	0.2%

12h (P53-BC10) 19.8%	Type	Reads	Percentage
GAATCTGCCTAACAGGA|GGTGGG	WT		
GAATCTGCCTAACAGGAaGGTGGG	+1	625	2.9%
GAATCTGCCTAACAGG---TGGG	-3	418	1.9%
GAATCTGCCTAACAGGA-GTGGG	-1	269	1.2%
GAATCTGCCTAACAGG-GGTGGG	-1	194	0.9%
GAATCTGCCTAACAGG--GTGGG	-2	170	0.8%
GAATCTGCCTAACAGGAgGGTGGG	+1	158	0.7%
GAATCTGCC--------GGTGGG	-8	85	0.4%
GAATCTGCCTAACAGGA--TGGG	-2	62	0.3%
GAATCTGCCTA------GGTGGG	-6	52	0.2%

24h (P54-BC10) 34.1%	Type	Reads	Percentage
GAATCTGCCTAACAGGA|GGTGGG	WT		
GAATCTGCCTAACAGG---TGGG	-3	1447	5.0%
GAATCTGCCTAACAGGAaGGTGGG	+1	1218	4.2%
GAATCTGCCTAACAGGA-GTGGG	-1	475	1.7%
GAATCTGCCTAACAGG--GTGGG	-2	331	1.2%
GAATCTGCCTAACAGG-GGTGGG	-1	277	1.0%
GAATCTGCCTAACAGGAgGGTGGG	+1	222	0.8%
GAATCTGC---------GGTGGG	-9	195	0.7%
GAATCTGCCTAACAGGA--TGGG	-2	134	0.5%
GAATCTGCCTA------GGTGGG	-6	128	0.4%

48h (P55-BC10) 41.1%	Type	Reads	Percentage
GAATCTGCCTAACAGGA|GGTGGG	WT		
GAATCTGCCTAACAGG---TGGG	-3	1094	9.7%
GAATCTGCCTAACAGGAaGGTGGG	+1	491	4.4%
GAATCTGCCTAACAGGA-GTGGG	-1	201	1.8%
GAATCTGCCTAACAGG-GGTGGG	-1	124	1.1%
GAATCTGCCTAACAGG--GTGGG	-2	122	1.1%
GAATCTGCCTAACAGGAgGGTGGG	+1	97	0.9%
GAATCTGC---------GGTGGG	-9	88	0.8%
GAATCTGCCTA------GGTGGG	-6	68	0.6%
GAATCTGCCTAACA--------GG	-8	60	0.5%

P51-P55 Syn23-crAAVS1f scAAV6 KI

4h (P51-BC4) 3.9% 1.3%

Sequence	Type	Reads	Percentage
GAATCTGCCTAACAGGA | GGTGGG	WT		
GAATCTGCCTAAGTTTAAACTACGCG TGGGTTAGACCC	HDR	770	1.3%
GAATCTGCCTAACAGGAaGGTGGG	+1	216	0.4%
GAATCTGCCTAACAGG---TGGG	-3	104	0.2%
GAATCTGCCTAACAGG-GGTGGG	-1	96	0.2%
GAATCTGCCTAACAGGA-GTGGG	-1	72	0.1%
GAATCTGCCTAACAGG--GTGGG	-2	62	0.1%
GAATCTGCCTAACAGGAgGGTGGG	+1	57	0.1%

8h (P52-BC4) 26.7% 16.1%

Sequence	Type	Reads	Percentage
GAATCTGCCTAACAGGA | GGTGGG	WT		
GAATCTGCCTAAGTTTAAACTACGC GTGGGTTAGACCC	HDR	1123 1	16.1%
GAATCTGCCTAACAGGAaGGTGGG	+1	1055	1.5%
GAATCTGCCTAACAGGA-GTGGG	-1	765	1.1%
GAATCTGCCTAACAGG-GGTGGG	-1	435	0.6%
GAATCTGCCTAACAGG---TGGG	-3	358	0.5%
GAATCTGCCTAACAGGAgGGTGGG	+1	219	0.3%
GAATCTGCCTAACAGGA--TGGG	-2	151	0.2%
GAATCTGCCTAACAGG--GTGGG	-2	141	0.2%
GAATCTGCCTAACAGGAcGGTGGG	+1	135	0.2%

12h (P53-BC4) 37.2% 23.0%

Sequence	Type	Reads	Percentage
GAATCTGCCTAACAGGA | GGTGGG	WT		
GAATCTGCCTAAGTTTAAACTACGCG TGGGTTAGACCC	HDR	8223	23.0%
GAATCTGCCTAACAGGAaGGTGGG	+1	704	2.0%
GAATCTGCCTAACAGGA-GTGGG	-1	286	0.8%
GAATCTGCCTAACAGG---TGGG	-3	252	0.7%
GAATCTGCCTAACAGGAgGGTGGG	+1	241	0.7%
GAATCTGCCTAACAGG--GTGGG	-2	192	0.5%
GAATCTGCCTAACAGG-GGTGGG	-1	146	0.4%
GAATCTGCCTAACAGGA--TGGG	-2	89	0.2%
GAATCTGC---------GGTGGG	-9	84	0.2%

24h (P54-BC4) 58.6% 34.6%

Sequence	Type	Reads	Percentage
GAATCTGCCTAACAGGA | GGTGGG	WT		
GAATCTGCCTAAGTTTAAACTACGCG TGGGTTAGACCC	HDR	12904	34.6%
GAATCTGCCTAACAGGAaGGTGGG	+1	967	2.6%
GAATCTGCCTAACAGG---TGGG	-3	470	1.3%
GAATCTGCCTAACAGGA-GTGGG	-1	427	1.1%
GAATCTGCCTAACAGGAgGGTGGG	+1	310	0.8%
GAATCTGCCTAACAGG-GGTGGG	-1	283	0.8%
GAATCTGCCTAACAGG--GTGGG	-2	258	0.7%
GAATCTGC---------GGTGGG	-9	186	0.5%

48h (P55-BC4) 66.7% 42.2%

Sequence	Type	Reads	Percentage
GAATCTGCCTAACAGGA | GGTGGG	WT		
GAATCTGCCTAAGTTTAAACTACGCG TGGGTTAGACCC	HDR	6700	42.2%
GAATCTGCCTAACAGGAaGGTGGG	+1	515	3.2%
GAATCTGCCTAACAGG---TGGG	-3	272	1.7%
GAATCTGCCTAACAGGA-GTGGG	-1	253	1.6%
GAATCTGCCTAACAGG--GTGGG	-2	143	0.9%
GAATCTGCCTAACAGGAgGGTGGG	+1	110	0.7%
GAATCTGCCTAACAGG-GGTGGG	-1	110	0.7%
GAATCTGCCTAACAGGA--TGGG	-2	77	0.5%

P51-P55 Syn24-crAAVS1g RNP KO

4h (P51-BC11) 1.8%	Type	Reads	Percentage
GGAATCTGCCTAACAGG|AGGTGG	WT		
GGAATCTGCCTAAC---AGGTGG	-3	92	0.2%
GGAATCTGCCTAACAGG-GGTGG	-1	84	0.2%
GGAATCTGCCTAACAGGAgGGTGG	+1	52	0.1%

8h (P52-BC11) 6.5%	Type	Reads	Percentage
GGAATCTGCCTAACAGG|AGGTGG	WT		
GGAATCTGCCTAAC---AGGTGG	-3	293	0.7%
GGAATCTGCCTAACAGG-GGTGG	-1	228	0.5%
GGAATCTGCCTAACAGGgAGGTGG	+1	196	0.5%
GGAATCTGCCTAACAGGAgGGTGG	+1	82	0.2%
GGAATCTGCCTAACAG-AGGTGG	-1	79	0.2%
GGAATCTGCCTAACAG--GGTGG	-2	57	0.1%
GGAATCTGCCTAA----AGGTGG	-4	41	0.1%

12h (P53-BC11) 11.0%	Type	Reads	Percentage
GGAATCTGCCTAACAGG|AGGTGG	WT		
GGAATCTGCCTAAC---AGGTGG	-3	333	0.9%
GGAATCTGCCTAACAGG-GGTGG	-1	255	0.7%
GGAATCTGCCTAACAGGgAGGTGG	+1	180	0.5%
GGAATCTGCCTAACAGGAgGGTGG	+1	136	0.4%
GGAATCTGCCTAACAG-AGGTGG	-1	86	0.2%
GGAATCTGCCTAACAG--GGTGG	-2	61	0.2%
GGAATCTGCCTAACA--AGGTGG	-2	59	0.2%

24h (P54-BC11) 19.2%	Type	Reads	Percentage
GGAATCTGCCTAACAGG|AGGTGG	WT		
GGAATCTGCCTAAC---AGGTGG	-3	1110	3.4%
GGAATCTGCCTAACAGG-GGTGG	-1	382	1.2%
GGAATCTGCCTAACAGGgAGGTGG	+1	222	0.7%
GGAATCTGCCTAACAG-AGGTGG	-1	207	0.6%
GGAATCTGCCTAACAGGAgGGTGG	+1	149	0.5%
GGAATCTGCCTAACAG--GGTGG	-2	110	0.3%
GGAATCTGCCTAACA--AGGTGG	-2	108	0.3%
GGAATCTGCCTAA----AGGTGG	-4	93	0.3%
GGAATCTGCCTAACAGGcAGGTGG	+1	83	0.3%

48h (P55-BC11) 23.5%	Type	Reads	Percentage
GGAATCTGCCTAACAGG|AGGTGG	WT		
GGAATCTGCCTAAC---AGGTGG	-3	1206	6.9%
GGAATCTGCCTAACAGG-GGTGG	-1	193	1.1%
GGAATCTGCCTAACAGGgAGGTGG	+1	111	0.6%
GGAATCTGCCTAACAG-AGGTGG	-1	100	0.6%
GGAATCTGCCTAACAGGAgGGTGG	+1	77	0.4%
GGAATCTGCCTAACAG--GGTGG	-2	72	0.4%
GGAATCTGCCTAA-----GGTGG	-5	63	0.4%
GGAATCTGCCTAACA--AGGTGG	-2	53	0.3%
GGAATCTGCCTAACAGGcAGGTGG	+1	47	0.3%

P51-P55 Syn24-crAAVS1g scAAV6 KI

4h (P51-BC5) 2.2% 0.4%

	Type	Reads	Percentage
GGAATCTGCCTAACAGG|AGGTGG	WT		
GGAATCTGCCTAAGTTTAAACTACGC GTGGGTTA	HDR	273	0.4%
GGAATCTGCCTAAC---AGGTGG	-3	125	0.2%
GGAATCTGCCTAACAGG-GGTGG	-1	107	0.1%

8h (P52-BC5) 10.5% 5.1%

	Type	Reads	Percentage
GGAATCTGCCTAACAGG|AGGTGG	WT		
GGAATCTGCCTAAGTTTAAACTACG CGTGGGTTA	HDR	4493	5.1%
GGAATCTGCCTAACAGG-GGTGG	-1	497	0.6%
GGAATCTGCCTAACAGGgAGGTGG	+1	386	0.4%
GGAATCTGCCTAAC---AGGTGG	-3	364	0.4%
GGAATCTGCCTAACAG-AGGTGG	-1	203	0.2%
GGAATCTGCCTAACAGGAgGGTGG	+1	188	0.2%
GGAATCTGCCTAACAG--GGTGG	-2	129	0.1%
GGAATCTGCCTAACAGGaAGGTGG	+1	83	0.1%

12h (P53-BC5) 17.9% 8.1%

	Type	Reads	Percentage
GGAATCTGCCTAACAGG|AGGTGG	WT		
GGAATCTGCCTAAGTTTAAACTACGC GTGGGTTA	HDR	4530	8.1%
GGAATCTGCCTAACAGG-GGTGG	-1	442	0.8%
GGAATCTGCCTAAC---AGGTGG	-3	334	0.6%
GGAATCTGCCTAACAGGAgGGTGG	+1	256	0.5%
GGAATCTGCCTAACAGGgAGGTGG	+1	251	0.4%
GGAATCTGCCTAACAG-AGGTGG	-1	231	0.4%
GGAATCTGCCTAACAG--GGTGG	-2	115	0.2%
GGAATCTGCCTAACA--AGGTGG	-2	82	0.1%

24h (P54-BC5) 32.7% 17.7%

	Type	Reads	Percentage
GGAATCTGCCTAACAGG|AGGTGG	WT		
GGAATCTGCCTAAGTTTAAACTACGC GTGGGTTA	HDR	7779	17.7%
GGAATCTGCCTAACAGG-GGTGG	-1	569	1.3%
GGAATCTGCCTAAC---AGGTGG	-3	503	1.1%
GGAATCTGCCTAACAGGgAGGTGG	+1	309	0.7%
GGAATCTGCCTAACAGGAgGGTGG	+1	250	0.6%
GGAATCTGCCTAACA--AGGTGG	-2	187	0.4%
GGAATCTGCCTAACAG-AGGTGG	-1	177	0.4%
GGAATCTGCCTAACAGGaAGGTGG	+1	171	0.4%

48h (P55-BC5) 43.0% 23.3%

	Type	Reads	Percentage
GGAATCTGCCTAACAGG|AGGTGG	WT		
GGAATCTGCCTAAGTTTAAACTACGC GTGGGTTA	HDR	5874	23.3%
GGAATCTGCCTAAC---AGGTGG	-3	389	1.5%
GGAATCTGCCTAACAGG-GGTGG	-1	298	1.2%
GGAATCTGCCTAACAG-AGGTGG	-1	187	0.7%
GGAATCTGCCTAACA--AGGTGG	-2	161	0.6%
GGAATCTGCCTAACAGGAgGGTGG	+1	158	0.6%
GGAATCTGCCTAA-----GGTGG	-5	128	0.5%
GGAATCTGCCTAACAGGgAGGTGG	+1	105	0.4%

P51-P55 Syn25-crAAVS1h RNP KO

4h (P51-BC12) 13.2%	Type	Reads	Percentage
TAAGGAATCTGCCTAAC|AGGAGG	WT		
TAAGGAATCTGCCTAACcAGGAGG	+1	3359	8.1%
TAAGGAATCTGCCTAA--GGAGG	-2	302	0.7%
TAAGGAATCTGCCTAA-AGGAGG	-1	190	0.5%
TAAGGAATCTGCCTAACaAGGAGG	+1	173	0.4%
TAAGGAATCTGCCTA-CAGGAGG	-1	128	0.3%
TAAGGAATCTGCCTAAC-GGAGG	-1	109	0.3%
TAAGGAATCTGCCT---AGGAGG	-3	52	0.1%
TAAGGAATCTGCCTAACtAGGAGG	+1	48	0.1%

8h (P52-BC12) 43.6%	Type	Reads	Percentage
TAAGGAATCTGCCTAAC|AGGAGG	WT		
TAAGGAATCTGCCTAACcAGGAGG	+1	8339	24.6%
TAAGGAATCTGCCTAA-AGGAGG	-1	735	2.2%
TAAGGAATCTGCCTAA--GGAGG	-2	690	2.0%
TAAGGAATCTGCCTAAC-GGAGG	-1	363	1.1%
TAAGGAATCTGCCTA-CAGGAGG	-1	312	0.9%
TAAGGAATCTGCCTAACaAGGAGG	+1	260	0.8%
TAA---------------GGAGG	-15	197	0.6%
TAAGGAATCTGCCT---AGGAGG	-3	132	0.4%
TAAGGAATCTGCCTAAC--GAGG	-2	123	0.4%

12h (P53-BC12) 50.7%	Type	Reads	Percentage
TAAGGAATCTGCCTAAC|AGGAGG	WT		
TAAGGAATCTGCCTAACcAGGAGG	+1	7573	26.1%
TAAGGAATCTGCCTAA--GGAGG	-2	698	2.4%
TAAGGAATCTGCCTAA-AGGAGG	-1	615	2.1%
TAAGGAATCTGCCTAACaAGGAGG	+1	448	1.5%
TAAGGAATCTGCCTAAC-GGAGG	-1	356	1.2%
TAAGGAATCTGCCTA-CAGGAGG	-1	302	1.0%
TAA---------------GGAGG	-15	221	0.8%
TAAGGAATCTGCCTAAC--GAGG	-2	157	0.5%
TAAGGAATCTGCCT---AGGAGG	-3	150	0.5%

24h (P54-BC12) 70.4%	Type	Reads	Percentage
TAAGGAATCTGCCTAAC|AGGAGG	WT		
TAAGGAATCTGCCTAACcAGGAGG	+1	10552	31.7%
TAAGGAATCTGCCTAA--GGAGG	-2	1209	3.6%
TAAGGAATCTGCCTAA-AGGAGG	-1	1117	3.4%
TAA---------------GGAGG	-15	651	2.0%
TAAGGAATCTGCCTAAC-GGAGG	-1	630	1.9%
TAAGGAATCTGCCTA-CAGGAGG	-1	610	1.8%
TAAGGAATCTGCCTAACaAGGAGG	+1	514	1.5%
TAAGGAATCTGCCTAAC--GAGG	-2	306	0.9%
TAAGGAATCTGCCT---AGGAGG	-3	222	0.7%

48h (P55-BC6) 77.2%	Type	Reads	Percentage
TAAGGAATCTGCCTAAC|AGGAGG	WT		
TAAGGAATCTGCCTAACcAGGAGG	+1	4173	34.8%
TAAGGAATCTGCCTAA--GGAGG	-2	551	4.6%
TAA---------------GGAGG	-15	533	4.4%
TAAGGAATCTGCCTAA-AGGAGG	-1	462	3.9%
TAAGGAATCTGCCTA-CAGGAGG	-1	240	2.0%
TAAGGAATCTGCCTAAC-GGAGG	-1	195	1.6%
TAAGGAATCTGCCTAACaAGGAGG	+1	143	1.2%
TAAGGAATCTGCCT---AGGAGG	-3	118	1.0%
TAAGGAATCTGCCTAAC--GAGG	-2	80	0.7%

P51-P55 Syn25-crAAVS1h scAAV6 KI

4h (P51-BC6) 15.2% 2.5%

4h (P51-BC6) 15.2% 2.5%	Type	Reads	Percentage
TAAGGAATCTGCCTAAC|AGGAGG	WT		
TAAGGAATCTGCCTAACcAGGAGG	+1	5673	6.9%
TAAGGAATCTGCCTAAGTTTAAACTACGCGTGGGTTA	HDR	2084	2.5%
TAAGGAATCTGCCTAA-AGGAGG	-1	447	0.5%
TAAGGAATCTGCCTAACaAGGAGG	+1	368	0.4%
TAAGGAATCTGCCTAA--GGAGG	-2	366	0.4%
TAAGGAATCTGCCTA-CAGGAGG	-1	185	0.2%
TAAGGAATCTGCCT---AGGAGG	-3	118	0.1%
TAAGGAATCTGCCTAAC-GGAGG	-1	84	0.1%

8h (P52-BC6) 53.2% 23.1%

8h (P52-BC6) 53.2% 23.1%	Type	Reads	Percentage
TAAGGAATCTGCCTAAC|AGGAGG	WT		
TAAGGAATCTGCCTAAGTTTAAACTACGCGTGGGTTA	HDR	21247	23.1%
TAAGGAATCTGCCTAACcAGGAGG	+1	12780	13.9%
TAAGGAATCTGCCTAA--GGAGG	-2	1373	1.5%
TAAGGAATCTGCCTAA-AGGAGG	-1	1183	1.3%
TAAGGAATCTGCCTAACaAGGAGG	+1	791	0.9%
TAAGGAATCTGCCTA-CAGGAGG	-1	696	0.8%
TAAGGAATCTGCCT---AGGAGG	-3	251	0.3%
TAA---------------GGAGG	-15	201	0.2%
TAAGGAATCTGCC[illegible]AGGAGG	-4	[illegible]	0.2%

12h (P53-BC6) 62.4% 30.1%

12h (P53-BC6) 62.4% 30.1%	Type	Reads	Percentage
TAAGGAATCTGCCTAAC|AGGAGG	WT		
TAAGGAATCTGCCTAAGTTTAAACTACGCGTGGGTTA	HDR	16648	30.1%
TAAGGAATCTGCCTAACcAGGAGG	+1	7667	13.9%
TAAGGAATCTGCCTAACaAGGAGG	+1	935	1.7%
TAAGGAATCTGCCTAA--GGAGG	-2	696	1.3%
TAAGGAATCTGCCTAA-AGGAGG	-1	662	1.2%
TAAGGAATCTGCCT---AGGAGG	-3	310	0.6%
TAA---------------GGAGG	-15	163	0.3%
TAAGGAATCTGCCTAAC--GAGG	-2	161	0.3%
TAAGGAATCTGCC----AGGAGG	-4	153	0.3%

24h (P54-BC6) 80.7% 38.9%

24h (P54-BC6) 80.7% 38.9%	Type	Reads	Percentage
TAAGGAATCTGCCTAAC|AGGAGG	WT		
TAAGGAATCTGCCTAAGTTTAAACTACGCGTGGGTTA	HDR	21419	38.9%
TAAGGAATCTGCCTAACcAGGAGG	+1	9346	17.0%
TAAGGAATCTGCCTAA--GGAGG	-2	958	1.7%
TAAGGAATCTGCCTAA-AGGAGG	-1	844	1.5%
TAAGGAATCTGCCTAACaAGGAGG	+1	834	1.5%
TAA---------------GGAGG	-15	669	1.2%
TAAGGAATCTGCCTAAC-GGAGG	-1	618	1.1%
TAAGGAATCTGCCTA-CAGGAGG	-1	401	0.7%

48h (P55-BC6) 86.5% 46.6%

48h (P55-BC6) 86.5% 46.6%	Type	Reads	Percentage
TAAGGAATCTGCCTAAC|AGGAGG	WT		
TAAGGAATCTGCCTAAGTTTAAACTACGCGTGGGTTA	HDR	17415	46.6%
TAAGGAATCTGCCTAACcAGGAGG	+1	5842	15.6%
TAAGGAATCTGCCTAA-AGGAGG	-1	631	1.7%
TAAGGAATCTGCCTAA--GGAGG	-2	509	1.4%
TAAGGAATCTGCCTAACaAGGAGG	+1	497	1.3%
TAA---------------GGAGG	-15	447	1.2%
TAAGGAATCTGCCTAAC-GGAGG	-1	344	0.9%
TAAGGAATCTGCCTA-CAGGAGG	-1	342	0.9%

P51-P55 Syn26-crCD326a RNP KO

4h (P51-BC7) 3.4%

Sequence	Type	Reads	Percentage
CCCTCCCGCGCCCCTCT|TCTCGG	WT		
CCCTCCCGCGCCCCTCTtTCTCGG	+1	2092	1.7%
CCCTCCCGCGCCCCTC-TCTCGG	-1	392	0.3%
CCCTCCCGCGCCCC---TCTCGG	-3	326	0.3%
CCCTCCCGCGCCCCT--TCTCGG	-2	86	0.1%
CCCTCC------------------	-21	62	0.1%
CCCTCCCGCGCCCCT-TTCTCGG	-1	53	0.0%

8h (P52-BC7) 8.5%

Sequence	Type	Reads	Percentage
CCCTCCCGCGCCCCTCT|TCTCGG	WT		
CCCTCCCGCGCCCCTCTtTCTCGG	+1	4801	3.5%
CCCTCCCGCGCCCCTC-TCTCGG	-1	1191	0.9%
CCCTCCCGCGCCCC---TCTCGG	-3	1009	0.7%
CCCTCCCGCGCCC-----CTCGG	-5	250	0.2%
CCCTCC------------------	-21	183	0.1%
CCCTCCCGCGCCCCT-TTCTCGG	-1	105	0.1%
CCCTCCCGCGCCCCTC--CTCGG	-2	101	0.1%
CCCTCCCGCGCCCCTCTctTCTCGG	+2	85	0.1%

12h (P53-BC7) 10.2%

Sequence	Type	Reads	Percentage
CCCTCCCGCGCCCCTCT|TCTCGG	WT		
CCCTCCCGCGCCCCTCTtTCTCGG	+1	4004	4.1%
CCCTCCCGCGCCCCTC-TCTCGG	-1	1092	1.1%
CCCTCCCGCGCCCC---TCTCGG	-3	841	0.9%
CCCTCC------------------	-21	249	0.3%
CCCTCCCGCGCCC-----CTCGG	-5	191	0.2%
CCCTCCCGCGCCCCT--TCTCGG	-2	134	0.1%
CCCTCC------------------	-23	109	0.1%
CCCTCCCGCGCCCCTCTctTCTCGG	+2	95	0.1%

24h (P54-BC7) 20.6%

Sequence	Type	Reads	Percentage
CCCTCCCGCGCCCCTCT|TCTCGG	WT		
CCCTCCCGCGCCCCTCTtTCTCGG	+1	5753	4.7%
CCCTCCCGCGCCCC---TCTCGG	-3	3998	3.2%
CCCTCCCGCGCCCCTC-TCTCGG	-1	2147	1.7%
CCCTCC------------------	-21	1207	1.0%
CCCTCCCGCGCCC-----CTCGG	-5	1187	1.0%
CCCTCC------------------	-17	389	0.3%
CCCTCCCGCGCCCCT-----------C	-10	358	0.3%
CCCTCC------------------	-23	333	0.3%
CCC--------------TCTCGG	-14	329	0.3%

48h (P55-BC7) 33.3%

Sequence	Type	Reads	Percentage
CCCTCCCGCGCCCCTCT|TCTCGG	WT		
CCCTCCCGCGCCCC---TCTCGG	-3	4093	5.4%
CCCTCCCGCGCCCCTCTtTCTCGG	+1	3884	5.2%
CCCTCC------------------	-21	1782	2.4%
CCCTCCCGCGCCC-----CTCGG	-5	1374	1.8%
CCCTCCCGCGCCCCTC-TCTCGG	-1	1263	1.7%
CCCTCC------------------	-23	732	1.0%
CCCTCC------------------	-19	492	0.7%
CCCTCCCGCGCCCCT-----------C	-10	480	0.6%
CCCTCC------------------	-17	420	0.6%

P51-P55 Syn26-crCD326a scAAV6 KI

4h (P51-BC1) 3.3% 0.1%

	Type	Reads	Percentage
CCCTCCCGCGCCCCTCT|TCTCGG	WT		
CCCTCCCGCGCCCCTCTtTCTCGG	+1	1836	1.6%
CCCTCCCGCGCCCCTC-TCTCGG	-1	433	0.4%
CCCTCCCGCACGCGTAGTTTAAACCTCGG	HDR	120	0.1%
CCCTCCCGCGCCCC---TCTCGG	-3	101	0.1%
CCCTCCCGCGCCCCT-TTCTCGG	-1	44	0.0%
CCCTCC-----------------	-21	42	0.0%

8h (P52-BC1) 8.8% 1.8%

	Type	Reads	Percentage
CCCTCCCGCGCCCCTCT|TCTCGG	WT		
CCCTCCCGCGCCCCTCTtTCTCGG	+1	4140	3.0%
CCCTCCCGCACGCGTAGTTTAAACCTCGG	HDR	2525	1.8%
CCCTCCCGCGCCCCTC-TCTCGG	-1	1238	0.9%
CCCTCCCGCGCCCC---TCTCGG	-3	363	0.3%
CCCTCCCGCGCCCCT--TCTCGG	-2	173	0.1%
CCCTCCCGCGCCCCT-TTCTCGG	-1	146	0.1%
CCCTCC-----------------	-21	133	0.1%
CCCTCC-----------------	-23	76	0.1%
CCCTCCCGCGCCCCTC--CTCGG	-2	55	0.0%

12h (P53-BC1) 12.2% 3.4%

	Type	Reads	Percentage
CCCTCCCGCGCCCCTCT|TCTCGG	WT		
CCCTCCCGCGCCCCTCTtTCTCGG	+1	3099	3.7%
CCCTCCCGCACGCGTAGTTTAAACCTCGG	HDR	2853	3.4%
CCCTCCCGCGCCCCTC-TCTCGG	-1	839	1.0%
CCCTCCCGCGCCCC---TCTCGG	-3	328	0.4%
CCCTCC-----------------	-21	120	0.1%
CCCTCC-----------------	-23	114	0.1%
CCCTCCCGCGCCC-----CTCGG	-5	106	0.1%
CCCTCCCGCGCCCCT-TTCTCGG	-1	80	0.1%
CCCTCCCGCGCCCCTCTcTCTCGG	+1	72	0.1%

24h (P54-BC1) 21.9% 9.0%

	Type	Reads	Percentage
CCCTCCCGCGCCCCTCT|TCTCGG	WT		
CCCTCCCGCACGCGTAGTTTAAACCTCGG	HDR	11342	9.0%
CCCTCCCGCGCCCCTCTtTCTCGG	+1	6119	4.8%
CCCTCCCGCGCCCCTC-TCTCGG	-1	1534	1.2%
CCCTCCCGCGCCCC---TCTCGG	-3	724	0.6%
CCCTCC-----------------	-21	294	0.2%
CCCTCC-----------------	-19	219	0.2%
CCCTCC-----------------	-23	209	0.2%
CCCTCCCGCGCCCCT--TCTCGG	-2	206	0.2%

48h (P55-BC1) 30.3% 12.5%

	Type	Reads	Percentage
CCCTCCCGCGCCCCTCT|TCTCGG	WT		
CCCTCCCGCACGCGTAGTTTAAACCTCGG	HDR	11038	12.5%
CCCTCCCGCGCCCCTCTtTCTCGG	+1	4613	5.2%
CCCTCCCGCGCCCCTC-TCTCGG	-1	1245	1.4%
CCCTCCCGCGCCCC---TCTCGG	-3	1043	1.2%
CCCTCC-----------------	-21	585	0.7%
CCCTCCCGCGCCC-----CTCGG	-5	392	0.4%
CCCTCCCGCGCCCCT-TTCTCGG	-1	320	0.4%
CCCTCC-----------------	-19	319	0.4%

P51-P55 Syn27-crCD326d RNP KO

4h (P51-BC8) 0.2%	Type	Reads	Percentage
CGCGCCGAGAAGAGGGG|CGCGGG	WT		
CGCGCCGAGAAGAGGG-CGCGGG	-1	68	0.1%
CGCGCCGAGAAGAGGGgCGCGGG	+1	29	0.0%

8h (P52-BC8) 0.5%	Type	Reads	Percentage
CGCGCCGAGAAGAGGGG|CGCGGG	WT		
CGCGCCGAGAAGAGGG-CGCGGG	-1	94	0.1%
CGCGCCGAGAAGAGGGgCGCGGG	+1	30	0.0%
CGCGCCGAGAAGAGGGG-GCGGG	-1	24	0.0%

12h (P53-BC8) 1.0%	Type	Reads	Percentage
CGCGCCGAGAAGAGGGG|CGCGGG	WT		
CGCGCCGAGAAGAGGG-CGCGGG	-1	84	0.1%
---------------------GG	-21	36	0.0%
CGCGCCGAGAAGAGGGgCGCGGG	+1	29	0.0%

24h (P54-BC8) 1.7%	Type	Reads	Percentage
CGCGCCGAGAAGAGGGG|CGCGGG	WT		
CGCGCCGAGAAGAGGG-CGCGGG	-1	133	0.1%
CGCGCCGAGAAG-----------	-11	95	0.1%
---------------------GG	-21	63	0.0%
CGCGCCGAGAAGAGGGgCGCGGG	+1	64	0.0%

48h (P55-BC8) 2.5%	Type	Reads	Percentage
CGCGCCGAGAAGAGGGG|CGCGGG	WT		
CGCGCCGAGAAG-----------	-11	218	0.2%
---------------------GG	-21	121	0.1%
CGCGCCGAGAAGAGGG-CGCGGG	-1	84	0.1%
CGCGCCGAGAAG-----------	-11	69	0.1%
CGCGCCGAGAAGAGGGgCGCGGG	+1	63	0.1%

P51-P55 Syn27-crCD326d scAAV6 KI

4h (P51-BC2) 0.4% 0

	Type	Reads	Percentage
CGCGCCGAGAAGAGGGG|CGCGGG	WT		
CGCGCCGAGAAGAGGG-CGCGGG	-1	101	0.1%

8h (P52-BC2) 0.7% 0.2%

	Type	Reads	Percentage
CGCGCCGAGAAGAGGGG|CGCGGG	WT		
CGCGCCGAGGTTTAAACTACGCGTGCGGG	HDR	265	0.2%
CGCGCCGAGAAGAGGG-CGCGGG	-1	132	0.1%
CGCGCCGAGAAGAGGGgCGCGGG	+1	42	0.0%

12h (P53-BC2) 1.0% 0.5%

	Type	Reads	Percentage
CGCGCCGAGAAGAGGGG|CGCGGG	WT		
CGCGCCGAGGTTTAAACTACGCGTGCGGG	HDR	411	0.5%
CGCGCCGAGAAGAGGG-CGCGGG	-1	103	0.1%
CGCGCCGAGAAGAGGGgCGCGGG	+1	37	0.0%
CGCGCCGAGAAG-----------	-11	28	0.0%

24h (P54-BC2) 1.9% 1.1%

	Type	Reads	Percentage
CGCGCCGAGAAGAGGGG|CGCGGG	WT		
CGCGCCGAGGTTTAAACTACGCGTGCGGG	HDR	1454	1.1%
CGCGCCGAGAAGAGGG-CGCGGG	-1	132	0.1%
---------------------GG	-21	86	0.1%
CGCGCCGAGAAGAGGGgCGCGGG	+1	86	0.1%

48h (P55-BC2) 2.9% 1.6%

	Type	Reads	Percentage
CGCGCCGAGAAGAGGGG|CGCGGG	WT		
CGCGCCGAGGTTTAAACTACGCGTGCGGG	HDR	1356	1.6%
CGCGCCGAGAAGAGGG-CGCGGG	-1	123	0.1%
---------------------GG	-21	101	0.1%
CGCGCCGAGAAG-----------	-11	89	0.1%
CGCGCCGAGAAGAGGGgCGCGGG	+1	52	0.1%

P51-P55 Syn28-crCD326e RNP KO

4h (P51-BC9) 1.6%	Type	Reads	Percentage
GCGCGCCGAGAAGAGGG|GCGCGG	WT		
GCGCGCCGAGAAGAGGGgGCGCGG	+1	389	0.6%
GCGCGCCGAGAAGAGGG-CGCGG	-1	272	0.4%
GCGCGCCGAGAAGAGG--CGCGG	-2	62	0.1%
GCGCGCCGAGAAGAGGG-------	-7	48	0.1%

8h (P52-BC9) 6.9%	Type	Reads	Percentage
GCGCGCCGAGAAGAGGG|GCGCGG	WT		
GCGCGCCGAGAAGAGGGgGCGCGG	+1	739	1.6%
GCGCGCCGAGAAGAGGG-CGCGG	-1	452	1.0%
GCGCGCCGAGAAGAGG--CGCGG	-2	137	0.3%
GCGCGCCGAGAAGAGGG-------	-7	94	0.2%
GCGCGG-----------------	-21	44	0.1%
GCGCGCCGAGAAGAGGGGC----	-11	43	0.1%

12h (P53-BC9) 7.3%	Type	Reads	Percentage
GCGCGCCGAGAAGAGGG|GCGCGG	WT		
GCGCGCCGAGAAGAGGGgGCGCGG	+1	1111	1.9%
GCGCGCCGAGAAGAGGG-CGCGG	-1	578	1.0%
GCGCGCCGAGAAGAGGG-------	-7	165	0.3%
GCGCGCCGAGAAGAGG--CGCGG	-2	163	0.3%
GCGCGG-----------------	-21	99	0.2%
GCGCGCCGAGAAGAGGGGC----	-11	61	0.1%

24h (P54-BC9) 10.3%	Type	Reads	Percentage
GCGCGCCGAGAAGAGGG|GCGCGG	WT		
GCGCGCCGAGAAGAGGGgGCGCGG	+1	1618	2.7%
GCGCGCCGAGAAGAGGG-CGCGG	-1	1316	2.2%
GCGCGCCGAGAAGAGGG-------	-7	377	0.6%
GCGCGCCGAGAAGAGG--CGCGG	-2	338	0.6%
GCGCGG-----------------	-21	288	0.5%
GCGCGCCGAGAAGAGGGGC----	-11	232	0.4%
GCGCGG-----------------	-19	98	0.2%
GCGCGG-----------------	-17	93	0.2%

48h (P55-BC9) 12.7%	Type	Reads	Percentage
GCGCGCCGAGAAGAGGG|GCGCGG	WT		
GCGCGCCGAGAAGAGGGgGCGCGG	+1	532	2.2%
GCGCGCCGAGAAGAGGG-CGCGG	-1	425	1.7%
GCGCGCCGAGAAGAGGG-------	-7	381	1.6%
GCGCGG-----------------	-21	237	1.0%
GCGCGCCGAGAAGAGGGGC----	-11	152	0.6%
GCGCGCCGAGAAGAGG--CGCGG	-2	141	0.6%
GCGCGG-----------------	-17	73	0.3%
GCGCGG-----------------	-19	57	0.2%
GCGCGCCGAGAAGAGGG------	-12	52	0.2%

P51-P55 Syn28-crCD326e scAAV6 KI

4h (P51-BC3) 1.6% 0.0%

Sequence	Type	Reads	Percentage
GCGCGCCGAGAAGAGGG|GCGCGG	WT		
GCGCGCCGAGAAGAGGGgGCGCGG	+1	707	0.5%
GCGCGCCGAGAAGAGGG-CGCGG	-1	458	0.3%
GCGCGCCGAGAAGAGG--CGCGG	-2	130	0.1%
GCGCGCCGAGAAG----GCGCGG	-4	48	0.0%
GCGCGCCGAGGTTTAAACTACGCGTGCGG	HDR	43	0.0%

8h (P52-BC3) 4.4% 0.6%

Sequence	Type	Reads	Percentage
GCGCGCCGAGAAGAGGG|GCGCGG	WT		
GCGCGCCGAGAAGAGGGgGCGCGG	+1	2154	1.4%
GCGCGCCGAGAAGAGGG-CGCGG	-1	1234	0.8%
GCGCGCCGAGGTTTAAACTACGCGTGCGG	HDR	845	0.6%
GCGCGCCGAGAAGAGG--CGCGG	-2	295	0.2%
GCGCGCCGAGAAGAGGG-------	-7	172	0.1%
GCGCGCCGAGAAGAGGGGC----	-11	102	0.1%
GCGCGG-----------------	-17	89	0.1%

12h (P53-BC3) 5.7% 1.1%

Sequence	Type	Reads	Percentage
GCGCGCCGAGAAGAGGG|GCGCGG	WT		
GCGCGCCGAGAAGAGGGgGCGCGG	+1	1570	1.6%
GCGCGCCGAGGTTTAAACTACGCGTGCGG	HDR	1070	1.1%
GCGCGCCGAGAAGAGGG-CGCGG	-1	966	1.0%
GCGCGCCGAGAAGAGG--CGCGG	-2	340	0.3%
GCGCGG-----------------	-21	158	0.2%
GCGCGCCGAGAAGAG---CGCGG	-3	74	0.1%
GCGCGCCGAGAAGAGGG------	-12	57	0.1%
GCGCGCCGAGAAGAGGG---CGG	-11	43	0.0%

24h (P54-BC3) 21.9% 9.0%

Sequence	Type	Reads	Percentage
GCGCGCCGAGAAGAGGG|GCGCGG	WT		
GCGCGCCGAGGTTTAAACTACGCGTGCGG	HDR	4354	3.1%
GCGCGCCGAGAAGAGGGgGCGCGG	+1	2636	1.9%
GCGCGCCGAGAAGAGGG-CGCGG	-1	1815	1.3%
GCGCGCCGAGAAGAGG--CGCGG	-2	539	0.4%
GCGCGG-----------------	-21	193	0.1%
GCGCGCCGAGAAGAGGG------	-12	171	0.1%
GCGCGG-----------------	-17	135	0.1%
GCGCGCCGAGAAGAGGGGC----	-11	135	0.1%

48h (P55-BC3) 14.7% 4.4%

Sequence	Type	Reads	Percentage
GCGCGCCGAGAAGAGGG|GCGCGG	WT		
GCGCGCCGAGGTTTAAACTACGCGTGCGG	HDR	3338	4.4%
GCGCGCCGAGAAGAGGGgGCGCGG	+1	1756	2.3%
GCGCGCCGAGAAGAGGG-CGCGG	-1	1610	2.1%
GCGCGCCGAGAAGAGG--CGCGG	-2	499	0.7%
GCGCGG-----------------	-21	238	0.3%
GCGCGCCGAGAAGAGGGGC----	-11	201	0.3%
GCGCGCCGAGAAGAGGG-------	-7	172	0.2%
GCGCGC---------------GG	-15	83	0.1%

P51-P55 Syn29-crCD326f RNP KO

4h (P51-BC10) 15.9%	Type	Reads	Percentage
TGCGCGCGCGCCGAGAA|GAGGGG	WT		
TGCGCGCGCGCCGAGAAaGAGGGG	+1	7165	11.8%
TGCGCGCGCGCCGAGA-GAGGGG	-1	281	0.5%
TGCGCGCGCGCC-----GAGGGG	-5	144	0.2%
TGCGCGCGCGCCGAGA---GGGG	-3	85	0.1%
TGCGCGCGCGCCGAGAA-AGGGG	-1	83	0.1%
TGCGCGCGCGCCGAGAAgGAGGGG	+1	80	0.1%

8h (P52-BC10) 34.8%	Type	Reads	Percentage
TGCGCGCGCGCCGAGAA|GAGGGG	WT		
TGCGCGCGCGCCGAGAAaGAGGGG	+1	11168	23.9%
TGCGCGCGCGCCGAGA-GAGGGG	-1	529	1.1%
TGCGCGCGCGCC-----GAGGGG	-5	295	0.6%
TGCGCGCGCGCCGAGA---GGGG	-3	236	0.5%
TGCGCGCGCGCCGAGAA-AGGGG	-1	149	0.3%
T----------------------	-21	125	0.3%
TGCGCGCGCGCCGAGAAgGAGGGG	+1	120	0.3%
TGCGCGCGCGCCGAGA--AGGGG	-2	88	0.2%

12h (P53-BC10) 40.1%	Type	Reads	Percentage
TGCGCGCGCGCCGAGAA|GAGGGG	WT		
TGCGCGCGCGCCGAGAAaGAGGGG	+1	10244	27.8%
TGCGCGCGCGCCGAGA-GAGGGG	-1	459	1.2%
TGCGCGCGCGCC-----GAGGGG	-5	300	0.8%
TGCGCGCGCGCCGAGA---GGGG	-3	245	0.7%
T----------------------	-21	153	0.4%
TGCGCGCGCGCCGAGAA-AGGGG	-1	130	0.4%
TGCGCGCGCGCCGAGAAgGAGGGG	+1	108	0.3%
TGCGCGCGCGCCGAGA--AGGGG	-2	75	0.2%
T----------------------	-23	60	0.2%

24h (P54-BC10) 59.1%	Type	Reads	Percentage
TGCGCGCGCGCCGAGAA|GAGGGG	WT		
TGCGCGCGCGCCGAGAAaGAGGGG	+1	17067	39.9%
TGCGCGCGCGCC-----GAGGGG	-5	979	2.3%
TGCGCGCGCGCCGAGA-GAGGGG	-1	889	2.1%
TGCGCGCGCGCCGAGA---GGGG	-3	520	1.2%
T----------------------	-21	423	1.0%
TGCGCGCGCGCCGAGAA-AGGGG	-1	258	0.6%
TGCGCGCG----------AGGGG	-10	171	0.4%
TGCGCGCGCGCCGAGA--AGGGG	-2	139	0.3%
TGCGCGCGCGCCGAGA-------	-14	128	0.3%

48h (P55-BC10) 69.8%	Type	Reads	Percentage
TGCGCGCGCGCCGAGAA|GAGGGG	WT		
TGCGCGCGCGCCGAGAAaGAGGGG	+1	5910	37.9%
TGCGCGCGCGCC-----GAGGGG	-5	640	4.1%
TGCGCGCGCGCCGAGA---GGGG	-3	336	2.2%
TGCGCGCGCGCCGAGA-GAGGGG	-1	306	2.0%
T----------------------	-21	286	1.8%
T----------------------	-23	123	0.8%
TGCGCGCGCGCCGAGAA-AGGGG	-1	115	0.7%
TGCGCGCG----------AGGGG	-10	83	0.5%
TGC---------------------- -	-19	58	0.4%

P51-P55 Syn29-crCD326f scAAV6 KI

4h (P51-BC4) 16.0% 0.6%

4h (P51-BC4) 16.0% 0.6%	Type	Reads	Percentage
TGCGCGCGCGCCGAGAA|GAGGGG	WT		
TGCGCGCGCGCCGAGAAaGAGGGG	+1	11521	10.4%
TGCGCGCGCGCCGAGGTTTAAACTAC GCGTGCGGG	HDR	657	0.6%
TGCGCGCGCGCCGAGA-GAGGGG	-1	517	0.5%
TGCGCGCGCGCCGAGAAgGAGGGG	+1	318	0.3%
TGCGCGCGCGCCGAGA---GGGG	-3	177	0.2%
TGCGCGCGCGCCGAGAA-AGGGG	-1	147	0.1%
TGCGCGCGCGCC-----GAGGGG	-5	125	0.1%

8h (P52-BC4) 41.0% 7.1%

8h (P52-BC4) 41.0% 7.1%	Type	Reads	Percentage
TGCGCGCGCGCCGAGAA|GAGGGG	WT		
TGCGCGCGCGCCGAGAAaGAGGGG	+1	24992	21.8%
TGCGCGCGCGCCGAGGTTTAAACTA CGCGTGCGGG	HDR	8072	7.1%
TGCGCGCGCGCCGAGA-GAGGGG	-1	1404	1.2%
TGCGCGCGCGCCGAGA---GGGG	-3	643	0.6%
TGCGCGCGCGCCGAGAAgGAGGGG	+1	426	0.4%
TGCGCGCGCGCCGAGA--AGGGG	-2	352	0.3%
TGCGCGCGCGCC-----GAGGGG	-5	282	0.2%
TGCGCGCGCGCCGAGAA-AGGGG	-1	222	0.2%
[illegible]GAGAAaaGAGGGG	+2	205	0.2%

12h (P53-BC4) 48.2% 12.2%

12h (P53-BC4) 48.2% 12.2%	Type	Reads	Percentage
TGCGCGCGCGCCGAGAA|GAGGGG	WT		
TGCGCGCGCGCCGAGAAaGAGGGG	+1	16794	22.2%
TGCGCGCGCGCCGAGGTTTAAACTAC GCGTGCGGG	HDR	9188	12.2%
TGCGCGCGCGCCGAGA-GAGGGG	-1	760	1.0%
TGCGCGCGCGCCGAGA---GGGG	-3	467	0.6%
TGCGCGCGCGCCGAGAAgGAGGGG	+1	242	0.3%
T---------------------	-21	194	0.3%
TGCGCGCGCGCCGAGA--AGGGG	-2	190	0.3%
TGCGCGCGCGCC-----GAGGGG	-5	175	0.2%
TGCGCGCGCGCCGAGAA-AGGGG	-1	171	0.2%

24h (P54-BC4) 70.1% 25.3%

24h (P54-BC4) 70.1% 25.3%	Type	Reads	Percentage
TGCGCGCGCGCCGAGAA|GAGGGG	WT		
TGCGCGCGCGCCGAGAAaGAGGGG	+1	24567	25.4%
TGCGCGCGCGCCGAGGTTTAAACTAC GCGTGCGGG	HDR	24475	25.3%
TGCGCGCGCGCCGAGA-GAGGGG	-1	1331	1.4%
TGCGCGCGCGCCGAGA---GGGG	-3	668	0.7%
TGCGCGCGCGCCGAGAAgGAGGGG	+1	424	0.4%
TGCGCGCGCGCCGAGAA-AGGGG	-1	387	0.4%
TGCGCGCGCGCC-----GAGGGG	-5	335	0.3%
T[illegible]	[illegible]	[illegible]	[illegible]

48h (P55-BC4) 78.6% 29.1%

48h (P55-BC4) 78.6% 29.1%	Type	Reads	Percentage
TGCGCGCGCGCCGAGAA|GAGGGG	WT		
TGCGCGCGCGCCGAGAAaGAGGGG	+1	13789	29.3%
TGCGCGCGCGCCGAGGTTTAAACTA CGCGTGCGGG	HDR	13677	29.1%
TGCGCGCGCGCCGAGA-GAGGGG	-1	727	1.5%
TGCGCGCGCGCCGAGA---GGGG	-3	393	0.8%
TGCGCGCGCGCC-----GAGGGG	-5	261	0.6%
T---------------------	-21	250	0.5%
TGCGCGCGCGCCGAGAA-AGGGG	-1	235	0.5%
[illegible]	[illegible]	[illegible]	[illegible]

P51-P55 Syn30-crCD326g RNP KO

4h (P51-BC11) 1.5%	Type	Reads	Percentage
CTGCGCGCGCGCCGAGA|AGAGGG	WT		
CTGCGCGCGCGCCGAGAaAGAGGG	+1	411	0.7%
CTGCGCGCGCGCCGAGA-GAGGG	-1	152	0.3%
CTGCGCGCGCGCCG---AGAGGG	-3	71	0.1%

8h (P52-BC11) 4.9%	Type	Reads	Percentage
CTGCGCGCGCGCCGAGA|AGAGGG	WT		
CTGCGCGCGCGCCGAGAaAGAGGG	+1	745	1.5%
CTGCGCGCGCGCCGAGA-GAGGG	-1	446	0.9%
CTGCGCGCGCGCCG---AGAGGG	-3	248	0.5%
CT-----------------------	-23	39	0.1%

12h (P53-BC11) 6.8%	Type	Reads	Percentage
CTGCGCGCGCGCCGAGA|AGAGGG	WT		
CTGCGCGCGCGCCGAGAaAGAGGG	+1	1034	1.8%
CTGCGCGCGCGCCG---AGAGGG	-3	567	1.0%
CTGCGCGCGCGCCGAGA-GAGGG	-1	533	0.9%
CTGCGCGCGCGCC-----GAGGG	-5	114	0.2%
CT-----------------------	-21	101	0.2%
CT-----------------------	-23	71	0.1%
CTGCGCGCGCGCCGAGAcAGAGGG	+1	65	0.1%
CTGCGCGCGCGCCGAGAtAGAGGG	+1	64	0.1%

24h (P54-BC11) 16.1%	Type	Reads	Percentage
CTGCGCGCGCGCCGAGA|AGAGGG	WT		
CTGCGCGCGCGCCG---AGAGGG	-3	2303	3.2%
CTGCGCGCGCGCCGAGAaAGAGGG	+1	2148	3.0%
CTGCGCGCGCGCCGAGA-GAGGG	-1	1065	1.5%
CT-----------------------	-21	664	0.9%
CTGCGCGCGCGCC-----GAGGG	-5	412	0.6%
CT-----------------------	-23	188	0.3%
CTGC---------------------	-19	185	0.3%
CTGCGCGC----------GAGGG	-10	150	0.2%
CTGCGC-------------------	-17	143	0.2%

48h (P55-BC11) 27.4%	Type	Reads	Percentage
CTGCGCGCGCGCCGAGA|AGAGGG	WT		
CTGCGCGCGCGCCG---AGAGGG	-3	1188	6.7%
CTGCGCGCGCGCCGAGAaAGAGGG	+1	507	2.9%
CT-----------------------	-21	408	2.3%
CTGCGCGCGCGCCGAGA-GAGGG	-1	299	1.7%
CTGCGCGCGCGCC-----GAGGG	-5	209	1.2%
CT-----------------------	-23	166	0.9%
CTGC---------------------	-19	114	0.6%
CTGCGCGC----------GAGGG	-10	94	0.5%
CTGCGC-------------------	-17	73	0.4%

P51-P55 Syn30-crCD326g scAAV6 KI

4h (P51-BC5) 1.3% 0.1%

	Type	Reads	Percentage
CTGCGCGCGCGCCGAGA|AGAGGG	WT		
CTGCGCGCGCGCCGAGAaAGAGGG	+1	439	0.5%
CTGCGCGCGCGCCGAGA-GAGGG	-1	244	0.3%
CTGCGCGCGCGCCGAGGTTTAAACTA CGCGTGCGGG	HDR	112	0.1%
CTGCGCGCGCGCCG---AGAGGG	-3	59	0.1%

8h (P52-BC5) 5.9% 2.1%

	Type	Reads	Percentage
CTGCGCGCGCGCCGAGA|AGAGGG	WT		
CTGCGCGCGCGCCGAGGTTTAAACT ACGCGTGCGGG	HDR	2546	2.1%
CTGCGCGCGCGCCGAGAaAGAGGG	+1	1454	1.2%
CTGCGCGCGCGCCGAGA-GAGGG	-1	797	0.6%
CTGCGCGCGCGCCG---AGAGGG	-3	277	0.2%
CTGCGCGCGCGCCGAGAtAGAGGG	+1	135	0.1%
CTGCGCGCGCGCCGAGAcAGAGGG	+1	100	0.1%

12h (P53-BC5) 7.6% 2.3%

	Type	Reads	Percentage
CTGCGCGCGCGCCGAGA|AGAGGG	WT		
CTGCGCGCGCGCCGAGGTTTAAACTA CGCGTGCGGG	HDR	1719	2.3%
CTGCGCGCGCGCCGAGAaAGAGGG	+1	1330	1.8%
CTGCGCGCGCGCCGAGA-GAGGG	-1	733	1.0%
CTGCGCGCGCGCCG---AGAGGG	-3	231	0.3%
CTGCGCGCGC-------AGAGGG	-7	79	0.1%
CTGCGCGCGCGCCGAGAtAGAGGG	+1	72	0.1%
CT---------------------	-21	68	0.1%
CTGCGCGCGCGCCGAGAgAGAGGG	+1	42	0.1%

24h (P54-BC5) 15.9% 7.7%

	Type	Reads	Percentage
CTGCGCGCGCGCCGAGA|AGAGGG	WT		
CTGCGCGCGCGCCGAGGTTTAAACTA CGCGTGCGGG	HDR	7611	7.7%
CTGCGCGCGCGCCGAGAaAGAGGG	+1	2118	2.1%
CTGCGCGCGCGCCGAGA-GAGGG	-1	1105	1.1%
CTGCGCGCGCGCCG---AGAGGG	-3	528	0.5%
CT---------------------	-21	288	0.3%
CTGCGCGCGCGCCGAGAtAGAGGG	+1	166	0.2%
CTGCGCGCGCGCCGAGAcAGAGGG	+1	133	0.1%
CTGCGCGCGCGCCGA--AGAGGG	-2	126	0.1%

48h (P55-BC5) 26.0% 11.3%

	Type	Reads	Percentage
CTGCGCGCGCGCCGAGA|AGAGGG	WT		
CTGCGCGCGCGCCGAGGTTTAAACT ACGCGTGCGGG	HDR	4839	11.3%
CTGCGCGCGCGCCGAGAaAGAGGG	+1	1380	3.2%
CTGCGCGCGCGCCGAGA-GAGGG	-1	695	1.6%
CTGCGCGCGCGCCG---AGAGGG	-3	537	1.3%
CT---------------------	-21	209	0.5%
CTGCGCGCGCGCCGAGAtAGAGGG	+1	186	0.4%
CTGCGCGCGCGCCGAGAcAGAGGG	+1	156	0.4%
CTGC-------------------	-19	152	0.4%

P51-P55 Syn31-crCD326h RNP KO

4h (P51-BC12) 1.9%	Type	Reads	Percentage
GCTGCGCGCGCGCCGAG | AAGAGG	WT		
GCTGCGCGCGCGCCGAGgAAGAGG	+1	472	1.0%
GCTGCGCGCGCGCCGAGaAAGAGG	+1	97	0.2%
GCTGCGCGCGCGCC-----GAGG	-5	47	0.1%

8h (P52-BC12) 6.1%	Type	Reads	Percentage
GCTGCGCGCGCGCCGAG | AAGAGG	WT		
GCTGCGCGCGCGCCGAGgAAGAGG	+1	884	2.0%
GCTGCGCGCGCGCC-----GAGG	-5	198	0.4%
GCTGCGCGCGCGCCGAGaAAGAGG	+1	134	0.3%
GCTGCGCGCGCGCCGA--AGAGG	-2	98	0.2%
GCTGCGCGCGCGCCGAGA-GAGG	-1	90	0.2%
GCTGCGCGCGCGCCGA-AAGAGG	-1	87	0.2%
GCTGCGCGCGCGCCGAGagAAGAGG	+2	77	0.2%

12h (P53-BC12) 7.1%	Type	Reads	Percentage
GCTGCGCGCGCGCCGAG | AAGAGG	WT		
GCTGCGCGCGCGCCGAGgAAGAGG	+1	1044	2.5%
GCTGCGCGCGCGCC-----GAGG	-5	221	0.5%
GCT----------------------	-21	137	0.3%
GCTGCGCGCGCGCCGA--AGAGG	-2	78	0.2%
GCTGCGCGCGCGCCGA-AAGAGG	-1	73	0.2%
GCTGCGCGCGCGCCGAGA-GAGG	-1	72	0.2%
GCT----------------------	-23	60	0.1%
GCTGCGCGCGCGCCGAGagAAGAGG	+2	58	0.1%
GCTGCGC-----------------	-17	55	0.1%

24h (P54-BC12) 18.6%	Type	Reads	Percentage
GCTGCGCGCGCGCCGAG | AAGAGG	WT		
GCTGCGCGCGCGCCGAGgAAGAGG	+1	2546	4.0%
GCTGCGCGCGCGCC-----GAGG	-5	1116	1.7%
GCT----------------------	-21	929	1.5%
GCT----------------------	-23	384	0.6%
GCTGCGCGCGCGCCGAGaAAGAGG	+1	340	0.5%
GCTGC-------------------	-19	323	0.5%
GCTGCGCGCGCGCCGA-AAGAGG	-1	299	0.5%
GCTGCGCGCGCGCCGA--AGAGG	-2	222	0.3%
GCTGCGCGCGCGCCGAGA---GG	-3	208	0.3%

48h (P55-BC12) 31.3%	Type	Reads	Percentage
GCTGCGCGCGCGCCGAG | AAGAGG	WT		
GCTGCGCGCGCGCC-----GAGG	-5	746	3.8%
GCT----------------------	-21	743	3.8%
GCTGCGCGCGCGCCGAGgAAGAGG	+1	624	3.2%
GCT----------------------	-23	318	1.6%
GCTGC-------------------	-19	251	1.3%
GCTGCGC-----------------	-17	161	0.8%
GCTGCGCGCG----------AGG	-10	157	0.8%
GCTGCGCGCGCGCCGAGA---GG	-3	138	0.7%
-------------------------GC	-24	135	0.7%

P51-P55 Syn31-crCD326h scAAV6 KI

4h (P51-BC6) 2.1% 0.2%	Type	Reads	Percentage
GCTGCGCGCGCGCCGAG | AAGAGG	WT		
GCTGCGCGCGCGCCGAGgAAGAGG	+1	972	0.8%
GCTGCGCGCGCGCCGAGaAAGAGG	+1	468	0.4%
GCTGCGCGCGCGCCGAGGTTTAAACT ACGCGTGCGGGAGGGG	HDR	187	0.2%
GCTGCGCGCGCGCCGAGA-GAGG	-1	77	0.1%

8h (P52-BC6) 10.6% 4.8%	Type	Reads	Percentage
GCTGCGCGCGCGCCGAG | AAGAGG	WT		
GCTGCGCGCGCGCCGAGGTTTAAAC TACGCGTGCGGGAGGGG	HDR	6917	4.8%
GCTGCGCGCGCGCCGAGgAAGAGG	+1	3021	2.1%
GCTGCGCGCGCGCCGAGaAAGAGG	+1	971	0.7%
GCTGCGCGCGCGCCGAGA-GAGG	-1	311	0.2%
GCTGCGCGCGCGCCGA--AGAGG	-2	198	0.1%
GCTGCGCGCGCGCC-----GAGG	-5	157	0.1%
GCT---------------------	-21	155	0.1%

12h (P53-BC6) 10.5% 4.3%	Type	Reads	Percentage
GCTGCGCGCGCGCCGAG | AAGAGG	WT		
GCTGCGCGCGCGCCGAGGTTTAAACT ACGCGTGCGGGAGGGG	HDR	4617	4.3%
GCTGCGCGCGCGCCGAGgAAGAGG	+1	2373	2.2%
GCTGCGCGCGCGCCGAGaAAGAGG	+1	831	0.8%
GCTGCGCGCGCGCCGAGA-GAGG	-1	210	0.2%
GCTGCGCGCGCGCCGA-AAGAGG	-1	184	0.2%
GCT-----------------------	-23	118	0.1%
GCTGCGCGCGCGCC-----GAGG	-5	115	0.1%
GCT---------------------	-21	92	0.1%
GCTGCGCGCGCGCCGA--AGAGG	-2	88	0.1%

24h (P54-BC6) 21.5% 11.5%	Type	Reads	Percentage
GCTGCGCGCGCGCCGAG | AAGAGG	WT		
GCTGCGCGCGCGCCGAGGTTTAAACT ACGCGTGCGGGAGGGG	HDR	18192	11.5%
GCTGCGCGCGCGCCGAGgAAGAGG	+1	4089	2.6%
GCTGCGCGCGCGCCGAGaAAGAGG	+1	1684	1.1%
GCTGCGCGCGCGCCGA--AGAGG	-2	451	0.3%
GCT---------------------	-21	420	0.3%
GCTGCGCGCGCGCCGAGA-GAGG	-1	387	0.2%
GCTGCGCGCGCGCCGA-AAGAGG	-1	372	0.2%
GCTGCGCGCGCGCC-----GAGG	-5	368	0.2%

48h (P55-BC6) 30.3% 14.2%	Type	Reads	Percentage
GCTGCGCGCGCGCCGAG | AAGAGG	WT		
GCTGCGCGCGCGCCGAGGTTTAAAC TACGCGTGCGGGAGGGG	HDR	13193	14.2%
GCTGCGCGCGCGCCGAGgAAGAGG	+1	3217	3.5%
GCTGCGCGCGCGCCGAGaAAGAGG	+1	1154	1.2%
GCTGCGCGCGCGCC-----GAGG	-5	658	0.7%
GCT---------------------	-21	644	0.7%
GCTGCGCGCGCGCCGA--AGAGG	-2	375	0.4%
GCTGCGCGCGCGCCGA-AAGAGG	-1	368	0.4%
GCTGCGCGCGCGCCGAGA---GG	-3	357	0.4%

P51-P57-YW-W3-Syn20crAAVS1c-AAV6KI

4h

Total | INDEL	Ins	Del	HDR
46690 | 20.66%	18.34%	1.02%	1.30%

Typical seqs	Reads	Type	pct.
TCTAACCCCACCTCCT|GTT**AGG**CAGA	37046	WT	79.34%
TCTAACCCCACCTCCTtGTTAGGCAGA	7070	+1 t	15.14%
TCTAACCCACGCGTAGTTTAAACTTAGGCAGA	575	+5 HDR	1.23%
TCTAACCCCACCTCCTCTGTTAGGCAGA	215	+2	0.46%
TCTAACCCCACCTCCTgGTTAGGCAGA	139	+1 g	0.30%
TCTAACCCCACCTCC--TTAGGCAGA	110	-2	0.24%
CCTAACCCCCACCTCCTTGTTAGGCAGA	77	+1 Ins	0.16%
TCTAGCCCCACCTCCTTGTTAGGCAGA	74	+1 Ins	0.16%
TCTAACCCCACCTCCTtGTTAGGCGGA	72	+1 t	0.15%
TCTAACCCCACCTCCCTGTTAGGCAGA	67	+1 Ins	0.14%

8h

Total | INDEL	Ins	Del	HDR
54598 | 58.33%	36.47%	4.16%	17.70%

Typical seqs	Reads	Type	pct.
TCTAACCCCACCTCCT|GTT**AGG**CAGA	22752	WT	41.67%
TCTAACCCCACCTCCTtGTTAGGCAGA	16070	+1 t	29.43%
TCTAACCCACGCGTAGTTTAAACTTAGGCAGA	9008	+5 HDR	16.50%
TCTAACCCCACCTCC--TTAGGCAGA	466	-2	0.85%
TCTAACCCCACCTCCTCTGTTAGGCAGA	444	+2	0.81%
TCTAACCCCACCTCCTgGTTAGGCAGA	429	+1 g	0.79%
TCTAACCCCACCTCC-GTTAGGCAGA	251	-1	0.46%
TCTAACCCCACCTCCCTGTTAGGCAGA	199	+1 Ins	0.36%
TCTAACCCCACCTC-TGTTAGGCAGA	191	-1	0.35%
TCTAACCCCACCTCCT-TTAGGCAGA	161	-1	0.29%

12h

Total | INDEL	Ins	Del	HDR
32417 | 66.37%	36.57%	5.74%	24.06%

Typical seqs	Reads	Type	pct.
TCTAACCCCACCTCCT|GTT**AGG**CAGA	10901	WT	33.63%
TCTAACCCCACCTCCTtGTTAGGCAGA	9284	+1 t	28.64%
TCTAACCCACGCGTAGTTTAAACTTAGGCAGA	7220	+5 HDR	22.27%
TCTAACCCCACCTCCTgGTTAGGCAGA	400	+1 g	1.23%
TCTAACCCCACCTCCTCTGTTAGGCAGA	247	+2	0.76%
TCTAACCCCACCTCC--TTAGGCAGA	241	-2	0.74%
TCTAACCCCACCTC-TGTTAGGCAGA	177	-1	0.55%
TCTAACCCCACCTCC-GTTAGGCAGA	159	-1	0.49%
TCTAACCCCACCTCCT-TTAGGCAGA	148	-1	0.46%
TCTAACCCCACCT---GTTAGGCAGA	112	-3	0.35%

24h

Total | INDEL	Ins	Del	HDR
37570 | 82.60%	42.70%	7.30%	32.60%

Typical seqs	Reads	Type	pct.
TCTAACCCCACCTCCT|GTT**AGG**CAGA	6537	WT	17.40%
TCTAACCCCACCTCCTtGTTAGGCAGA	12797	+1 t	34.06%
TCTAACCCACGCGTAGTTTAAACTTAGGCA GA	11486	+5 HDR	30.57%
TCTAACCCCACCTCCTgGTTAGGCAGA	561	+1 g	1.49%
TCTAACCCCACCTCC--TTAGGCAGA	388	-2	1.03%
TCTAACCCCACCTCCTCTGTTAGGCAGA	266	+2	0.71%
TCTAACCCCACCTC-TGTTAGGCAGA	170	-1	0.45%
TCTAACCCCACCTCC-GTTAGGCAGA	174	-1	0.46%
TCTAACCCCACCT---GTTAGGCAGA	145	-3	0.39%
TCTAACCCCACCTCCT-TTAGGCAGA	136	-1	0.36%

48h

Total | INDEL	Ins	Del	HDR
20184 | 86.18%	43.27%	7.38%	35.53%

Typical seqs	Reads	Type	pct.
TCTAACCCCACCTCCT|GTT**AGG**CAGA	2790	WT	13.82%
TCTAACCCCACCTCCTtGTTAGGCAGA	6934	+1 t	34.35%
TCTAACCCACGCGTAGTTTAAACTTAGGCA GA	6703	+5 HDR	33.21%
TCTAACCCCACCTCCTgGTTAGGCAGA	264	+1 g	1.31%
TCTAACCCCACCTCCTCTGTTAGGCAGA	175	+2	0.87%
TCTAACCCCACCTCC--TTAGGCAGA	129	-2	0.64%
TCTAACCCCACCTCC-GTTAGGCAGA	128	-1	0.63%
TCTAACCCCACCT---GTTAGGCAGA	118	-3	0.58%
TCTAACCCCACCTC-TGTTAGGCAGA	108	-1	0.54%
TCTAACCCCACC--------------	105	-15	0.52%

P51-P57-YW-W3-Syn21crAAVS1d-AAV6KI

4h

Total | INDEL	Ins	Del	HDR
60196 | 3.15%	0.48%	1.98%	0.70%

Typical seqs	Reads	Type	pct.
ATCTGCCTAACAGGAGG|TGG**GGG**TTAG	58300	WT	96.85%
ATCTGCCTAAGTTTAAACTACGCGTGGGTTAG	409	+5 HDR	0.68%
ATCTGCCTAACAGGAGGT-GGGGTTAG	336	-1	0.56%
ATCTGCCTAACAGGAGG-GGGGGTTAG	257	-1	0.43%
ATCTGCCTAACAGGAGGgTGGGGGTTAG	155	+1 g	0.26%
ATCTGCCTAACAGGAG-TGGGGGTTAG	66	-1	0.11%
ATCTGCCTAACAGGA--TGGGGGTTAG	60	-2	0.10%
ATCTGCCTAACA-----TGGGGGTTAG	56	-5	0.09%
ATCTGCCTAACAGGAG--GGGGGTTAG	44	-2	0.07%
ATCTGCCTAAC---AGGTGGGGGTTAG	41	-3	0.07%

8h

Total | INDEL	Ins	Del	HDR
58756 | 18.27%	1.89%	5.00%	11.38%

Typical seqs	Reads	Type	pct.
ATCTGCCTAACAGGAGG|TGG**GGG**TTAG	48022	WT	81.73%
ATCTGCCTAAGTTTAAACTACGCGTGGGTTAG	6163	+5 HDR	10.49%
ATCTGCCTAACAGGAGG-GGGGGTTAG	565	-1	0.96%
ATCTGCCTAACAGGAGGgTGGGGGTTAG	354	+1 g	0.60%
ATCTGCCTAACAGGAGGT-GGGGTTAG	299	-1	0.51%
ATCTGCCTAACAGGA--TGGGGGTTAG	233	-2	0.40%
ATCTGCCTAACAGGAG-TGGGGGTTAG	207	-1	0.35%
ATCTGCCTAACA-----TGGGGGTTAG	146	-5	0.25%
ATCTGCCTAACAGGAG--GGGGGTTAG	118	-2	0.20%
ATCTGCCTAACAGGA---GGGGGTTAG	99	-3	0.17%

12h

Total | INDEL	Ins	Del	HDR
40363 | 25.48%	3.11%	7.14%	15.23%

Typical seqs	Reads	Type	pct.
ATCTGCCTAACAGGAGG|TGG**GGG**TTAG	30079	WT	74.52%
ATCTGCCTAAGTTTAAACTACGCGTGGGTTAG	5717	+5 HDR	14.16%
ATCTGCCTAACAGGAGG-GGGGGTTAG	595	-1	1.47%
ATCTGCCTAACAGGAGGgTGGGGGTTAG	510	+1 g	1.26%
ATCTGCCTAACAGGA--TGGGGGTTAG	290	-2	0.72%
ATCTGCCTAACAGGAG-TGGGGGTTAG	240	-1	0.59%
ATCTGCCTAACAGGAGGT-GGGGTTAG	174	-1	0.43%
ATCTGCCTAACAGGAG--GGGGGTTAG	108	-2	0.27%
ATCTGCCTAACA----GTGGGGGTTAG	80	-4	0.20%
ATCTGCCTAAC------TGGGGGTTAG	83	-6	0.21%

24h

Total | INDEL	Ins	Del	HDR
45889 | 38.68%	3.89%	12.03%	22.75%

Typical seqs	Reads	Type	pct.
ATCTGCCTAACAGGAGG|TGG**GGG**TTAG	28141	WT	61.32%
ATCTGCCTAAGTTTAAACTACGCGTGGGTTAG	9729	+5 HDR	21.20%
ATCTGCCTAACAGGAGG-GGGGGTTAG	841	-1	1.83%
ATCTGCCTAACAGGA--TGGGGGTTAG	610	-2	1.33%
ATCTGCCTAACAGGAGGgTGGGGGTTAG	522	+1 g	1.14%
ATCTGCCTAACAGGAG-TGGGGGTTAG	357	-1	0.78%
ATCTGCCTAACAGGA------GGTTAG	306	-6	0.67%
ATCTGCCTAACAGGA---GGGGGTTAG	204	-3	0.44%
ATCTGCCTAAC---AGGTGGGGGTTAG	154	-3	0.34%
ATCTGCCTAACA----GTGGGGGTTAG	143	-4	0.31%

48h

Total | INDEL	Ins	Del	HDR
25773 | 43.63%	4.12%	13.61%	25.90%

Typical seqs	Reads	Type	pct.
ATCTGCCTAACAGGAGG|TGG**GGG**TTAG	14529	WT	56.37%
ATCTGCCTAAGTTTAAACTACGCGTGGGTTAG	6255	+5 HDR	24.27%
ATCTGCCTAACAGGAGG-GGGGGTTAG	483	-1	1.87%
ATCTGCCTAACAGGA--TGGGGGTTAG	381	-2	1.48%
ATCTGCCTAACAGGAGGgTGGGGGTTAG	353	+1 g	1.37%
ATCTGCCTAACAGGA------GGTTAG	156	-6	0.61%
ATCTGCCTA--------TGGGGGTTAG	151	-8	0.59%
ATCTGCCTAAC---AGGTGGGGGTTAG	133	-3	0.52%
ATCTGCCTAACAGGAG-TGGGGGTTAG	104	-1	0.40%
ATCTGCCTAACAGGA---GGGGGTTAG	81	-3	0.31%

P51-P57-YW-W3-Syn22crAAVS1e-AAV6KI

4h

Total	INDEL	Ins	Del	HDR
58844	6.07%	**3.89%**	**1.39%**	**0.79%**

Typical seqs	Reads	Type	pct.
AATCTGCCTAACAGGAG|GTG**GGG**GTTA	55270	WT	93.93%
AATCTGCCTAACAGGAGgGTGGGGGTTA	1677	+1 g	2.85%
AATCTGCCTAAGTTTAAACTACGCGTGGGTTA	445	+5 HDR	0.76%
AATCTGCCTAACAGGA-GTGGGGGTTA	298	-1	0.51%
AATCTGCCTAACAAGGAGGTGGGGGTTA	248	+1 Ins	0.42%
AATCTGCCTAACAGG---TGGGGGTTA	138	-3	0.23%
AATCTGCCTAACAG--GGTGGGGGTTA	82	-2	0.14%
AATCTGCCTAACAGG-GGTGGGGGTTA	64	-1	0.11%
AATCTGCCTAACAGGAGAGGTGGGGGTTA	39	+2	0.07%
AATCTGCCTAACAGGAAGGTGGGGGTTA	31	+1 Ins	0.05%

8h

Total	INDEL	Ins	Del	HDR
68822	28.30%	**11.95%**	**4.03%**	**12.32%**

Typical seqs	Reads	Type	pct.
AATCTGCCTAACAGGAG|GTG**GGG**GTTA	49347	WT	71.70%
AATCTGCCTAAGTTTAAACTACGCGTGGGTTA	7880	+5 HDR	11.45%
AATCTGCCTAACAGGAGgGTGGGGGTTA	5842	+1 g	8.49%
AATCTGCCTAACAGGA-GTGGGGGTTA	980	-1	1.42%
AATCTGCCTAACAAGGAGGTGGGGGTTA	591	+1 Ins	0.86%
AATCTGCCTAACAGG---TGGGGGTTA	333	-3	0.48%
AATCTGCCTAACAGG-GGTGGGGGTTA	169	-1	0.25%
AATCTGCCTAACAGGAGAGGTGGGGGTTA	164	+2	0.24%
AATCTGCCTAACAG--GGTGGGGGTTA	142	-2	0.21%
AATCTGCCTAACA---------GGTTA	102	-9	0.15%

12h

Total	INDEL	Ins	Del	HDR
37759	36.24%	**13.82%**	**5.32%**	**17.10%**

Typical seqs	Reads	Type	pct.
AATCTGCCTAACAGGAG|GTG**GGG**GTTA	24077	WT	63.76%
AATCTGCCTAAGTTTAAACTACGCGTGGGTTA	6064	+5 HDR	16.06%
AATCTGCCTAACAGGAGgGTGGGGGTTA	3429	+1 g	9.08%
AATCTGCCTAACAAGGAGGTGGGGGTTA	680	+1 Ins	1.80%
AATCTGCCTAACAGGA-GTGGGGGTTA	487	-1	1.29%
AATCTGCCTAACAGG---TGGGGGTTA	176	-3	0.47%
AATCTGCCTAACAGG-GGTGGGGGTTA	123	-1	0.33%
AATCTGCCTAACAG--GGTGGGGGTTA	85	-2	0.23%
AATCTGCCTAACAGGAGAGGTGGGGGTTA	55	+2	0.15%
AATCTGCCTA--AGGAGGTGGGGGTTA	47	-2	0.12%

24h

Total	INDEL	Ins	Del	HDR
39279	56.18%	**16.86%**	**10.85%**	**28.47%**

Typical seqs	Reads	Type	pct.
AATCTGCCTAACAGGAG|GTG**GGG**GTTA	17212	WT	43.82%
AATCTGCCTAAGTTTAAACTACGCGTGGGTTA	10494	+5 HDR	26.72%
AATCTGCCTAACAGGAGgGTGGGGGTTA	4471	+1 g	11.38%
AATCTGCCTAACAGGA-GTGGGGGTTA	755	-1	1.92%
AATCTGCCTAACAAGGAGGTGGGGGTTA	634	+1 Ins	1.61%
AATCTGCCTAACAGG---TGGGGGTTA	390	-3	0.99%
AATCTGCCTAACAG--GGTGGGGGTTA	302	-2	0.77%
AATCTGCCTAACAGG-GGTGGGGGTTA	275	-1	0.70%
AATCTG---------------------	147	-23	0.37%
AATCTGCCTAACAGGAAGGTGGGGGTTA	99	+1 Ins	0.25%

48h

Total	INDEL	Ins	Del	HDR
24430	61.22%	**18.42%**	**10.84%**	**31.96%**

Typical seqs	Reads	Type	pct.
AATCTGCCTAACAGGAG|GTG**GGG**GTTA	9474	WT	38.78%
AATCTGCCTAAGTTTAAACTACGCGTGGGTTA	7284	+5 HDR	29.82%
AATCTGCCTAACAGGAGgGTGGGGGTTA	3075	+1 g	12.59%
AATCTGCCTAACAGGA-GTGGGGGTTA	465	-1	1.90%
AATCTGCCTAACAAGGAGGTGGGGGTTA	337	+1 Ins	1.38%
AATCTGCCTAACAGG---TGGGGGTTA	234	-3	0.96%
AATCTGCCTAACAG--GGTGGGGGTTA	163	-2	0.67%
AATCTGCCTAACAGG-GGTGGGGGTTA	108	-1	0.44%
AATCTGCCTAACAGGA-------GTTA	85	-7	0.35%
AATCTGCCTAACAGGAGAGGTGGGGGTTA	73	+2	0.30%

P51-P57-YW-W3-Syn23crAAVS1f-AAV6KI

4h

Total | INDEL	Ins	Del	HDR
59061 | 3.90%	1.55%	1.05%	1.30%

Typical seqs	Reads	Type	pct.
GAATCTGCCTAACAGGA|GGT**GGG**GGTT	56757	WT	96.10%
GAATCTGCCTAAGTTTAAACTACGCGTGGGTT	725	+5 HDR	1.23%
GAATCTGCCTAACAAGGAGGTGGGGGTT	296	+1 Ins	0.50%
GAATCTGCCTAACAGGAaGGTGGGGGTT	234	+1 a	0.40%
GAATCTGCCTAACCAGGAGGTGGGGGTT	201	+1 Ins	0.34%
GAATCTGCCTAACA---GGTGGGGGTT	110	-3	0.19%
GAATCTGCCTAACAGG-GGTGGGGGTT	99	-1	0.17%
GAATCTGCCTAACAGGA-GTGGGGGTT	75	-1	0.13%
GAATCTGCCTAACAG--GGTGGGGGTT	62	-2	0.10%
GAATCTGCCTAACAGGAgGGTGGGGGTT	57	+1 g	0.10%

8h

Total | INDEL	Ins	Del	HDR
69803 | 26.66%	5.52%	5.06%	16.09%

Typical seqs	Reads	Type	pct.
GAATCTGCCTAACAGGA|GGT**GGG**GGTT	51193	WT	73.34%
GAATCTGCCTAAGTTTAAACTACGCGTGGGTT	10488	+5 HDR	15.03%
GAATCTGCCTAACAGGAaGGTGGGGGTT	1254	+1 a	1.80%
GAATCTGCCTAACAGGA-GTGGGGGTT	877	-1	1.26%
GAATCTGCCTAACAAGGAGGTGGGGGTT	614	+1 Ins	0.88%
GAATCTGCCTAACAGG-GGTGGGGGTT	494	-1	0.71%
GAATCTGCCTAACCAGGAGGTGGGGGTT	447	+1 Ins	0.64%
GAATCTGCCTAACA---GGTGGGGGTT	397	-3	0.57%
GAATCTGCCTAACAGGAgGGTGGGGGTT	226	+1 g	0.32%
GAATCTGCCTAACAGGA--TGGGGGTT	151	-2	0.22%

12h

Total | INDEL	Ins	Del	HDR
35774 | 37.16%	7.91%	6.26%	22.99%

Typical seqs	Reads	Type	pct.
GAATCTGCCTAACAGGA|GGT**GGG**GGTT	22481	WT	62.84%
GAATCTGCCTAAGTTTAAACTACGCGTGGGTT	7631	+5 HDR	21.33%
GAATCTGCCTAACAGGAaGGTGGGGGTT	759	+1 a	2.12%
GAATCTGCCTAACAAGGAGGTGGGGGTT	646	+1 Ins	1.81%
GAATCTGCCTAACCAGGAGGTGGGGGTT	385	+1 Ins	1.08%
GAATCTGCCTAACAGGA-GTGGGGGTT	316	-1	0.88%
GAATCTGCCTAACA---GGTGGGGGTT	264	-3	0.74%
GAATCTGCCTAACAGGAgGGTGGGGGTT	250	+1 g	0.70%
GAATCTGCCTAACAG--GGTGGGGGTT	195	-2	0.55%
GAATCTGCCTAACAGG-GGTGGGGGTT	152	-1	0.42%

24h

Total | INDEL	Ins	Del	HDR
37334 | 58.60%	10.46%	13.58%	34.56%

Typical seqs	Reads	Type	pct.
GAATCTGCCTAACAGGA|GGT**GGG**GGTT	15456	WT	41.40%
GAATCTGCCTAAGTTTAAACTACGCGTGGGTT	12011	+5 HDR	32.17%
GAATCTGCCTAACAGGAaGGTGGGGGTT	1107	+1 a	2.97%
GAATCTGCCTAACAAGGAGGTGGGGGTT	662	+1 Ins	1.77%
GAATCTGCCTAACA---GGTGGGGGTT	527	-3	1.41%
GAATCTGCCTAACCAGGAGGTGGGGGTT	505	+1 Ins	1.35%
GAATCTGCCTAACAGGA-GTGGGGGTT	468	-1	1.25%
GAATCTGCCTAACAGGAgGGTGGGGGTT	326	+1 g	0.87%
GAATCTGCCTAACAGG-GGTGGGGGTT	303	-1	0.81%
GAATCTGCCTAACAG--GGTGGGGGTT	274	-2	0.73%

48h

Total | INDEL	Ins	Del	HDR
15879 | 66.65%	10.66%	13.80%	42.19%

Typical seqs	Reads	Type	pct.
GAATCTGCCTAACAGGA|GGT**GGG**GGTT	5295	WT	33.35%
GAATCTGCCTAAGTTTAAACTACGCGTGGGTT	6268	+5 HDR	39.47%
GAATCTGCCTAACAGGAaGGTGGGGGTT	582	+1 a	3.67%
GAATCTGCCTAACA---GGTGGGGGTT	282	-3	1.78%
GAATCTGCCTAACAGGA-GTGGGGGTT	253	-1	1.59%
GAATCTGCCTAACAAGGAGGTGGGGGTT	226	+1 Ins	1.42%
GAATCTGCCTAACCAGGAGGTGGGGGTT	150	+1 Ins	0.94%
GAATCTGCCTAACAG--GGTGGGGGTT	146	-2	0.92%
GAATCTGCCTAACAGGAgGGTGGGGGTT	110	+1 g	0.69%
GAATCTGCCTAACAGG-GGTGGGGGTT	110	-1	0.69%

P51-P57-YW-W3-Syn24crAAVS1g-AAV6KI

4h

Total	INDEL	Ins	Del	HDR
71993	2.23%	1.11%	0.74%	0.38%

Typical seqs	Reads	Type	pct.
GGAATCTGCCTAACAGG|AGG**TGG**GGGT	70389	WT	97.77%
GGAATCTGCCTAACAAGGAGGTGGGGGT	375	+1 Ins	0.52%
GGAATCTGCCTAAGTTTAAACTACGCGTGGGT	263	+5 HDR	0.37%
GGAATCTGCCTAACCAGGAGGTGGGGGT	245	+1 Ins	0.34%
GGAATCTGCCTAAC---AGGTGGGGGT	131	-3	0.18%
GGAATCTGCCTAACAGG-GGTGGGGGT	113	-1	0.16%
GGAATCTGCCTAACAG-AGGTGGGGGT	63	-1	0.09%
GGAATCTGCCTAACAGGgAGGTGGGGGT	56	+1 g	0.08%
GGAATCTGCCTAACAGGaGGGTGGGGGT	55	+1 a	0.08%
GGAATCTGCCT-ACAGGAGGTGGGGGT	27	-1	0.04%

8h

Total	INDEL	Ins	Del	HDR
88574	10.51%	2.92%	2.51%	5.07%

Typical seqs	Reads	Type	pct.
GGAATCTGCCTAACAGG|AGG**TGG**GGGT	79265	WT	89.49%
GGAATCTGCCTAAGTTTAAACTACGCGTGGGT	4189	+5 HDR	4.73%
GGAATCTGCCTAACAAGGAGGTGGGGGT	778	+1 Ins	0.88%
GGAATCTGCCTAACAGG-GGTGGGGGT	582	-1	0.66%
GGAATCTGCCTAACCAGGAGGTGGGGGT	556	+1 Ins	0.63%
GGAATCTGCCTAACAGGgAGGTGGGGGT	448	+1 g	0.51%
GGAATCTGCCTAAC---AGGTGGGGGT	432	-3	0.49%
GGAATCTGCCTAACAG-AGGTGGGGGT	211	-1	0.24%
GGAATCTGCCTAACAGGaGGGTGGGGGT	200	+1 a	0.23%
GGAATCTGCCTAACAG--GGTGGGGGT	129	-2	0.15%

12h

Total	INDEL	Ins	Del	HDR
56120	17.90%	4.96%	4.87%	8.07%

Typical seqs	Reads	Type	pct.
GGAATCTGCCTAACAGG|AGG**TGG**GGGT	46073	WT	82.10%
GGAATCTGCCTAAGTTTAAACTACGCGTGGGT	4224	+5 HDR	7.53%
GGAATCTGCCTAACAAGGAGGTGGGGGT	979	+1 Ins	1.74%
GGAATCTGCCTAACCAGGAGGTGGGGGT	651	+1 Ins	1.16%
GGAATCTGCCTAACAGG-GGTGGGGGT	510	-1	0.91%
GGAATCTGCCTAAC---AGGTGGGGGT	360	-3	0.64%
GGAATCTGCCTAACAGGaGGGTGGGGGT	268	+1 a	0.48%
GGAATCTGCCTAACAGGgAGGTGGGGGT	279	+1 g	0.50%
GGAATCTGCCTAACAG-AGGTGGGGGT	257	-1	0.46%
GGAATCTGCCTAACAG--GGTGGGGGT	129	-2	0.23%

24h

Total	INDEL	Ins	Del	HDR
44006	32.72%	6.48%	8.56%	17.68%

Typical seqs	Reads	Type	pct.
GGAATCTGCCTAACAGG|AGG**TGG**GGGT	29609	WT	67.28%
GGAATCTGCCTAAGTTTAAACTACGCGTGGGT	7220	+5 HDR	16.41%
GGAATCTGCCTAACAAGGAGGTGGGGGT	800	+1 Ins	1.82%
GGAATCTGCCTAACAGG-GGTGGGGGT	653	-1	1.48%
GGAATCTGCCTAAC---AGGTGGGGGT	555	-3	1.26%
GGAATCTGCCTAACCAGGAGGTGGGGGT	533	+1 Ins	1.21%
GGAATCTGCCTAACAGGgAGGTGGGGGT	322	+1 g	0.73%
GGAATCTGCCTAACAGGaGGGTGGGGGT	253	+1 a	0.57%
GGAATCTGCCTAACA--AGGTGGGGGT	217	-2	0.49%
GGAATCTGCCTAACAG-AGGTGGGGGT	187	-1	0.42%

48h

Total	INDEL	Ins	Del	HDR
25168	42.96%	6.39%	13.23%	23.34%

Typical seqs	Reads	Type	pct.
GGAATCTGCCTAACAGG|AGG**TGG**GGGT	14356	WT	57.04%
GGAATCTGCCTAAGTTTAAACTACGCGTGGGT	5458	+5 HDR	21.69%
GGAATCTGCCTAACAAGGAGGTGGGGGT	451	+1 Ins	1.79%
GGAATCTGCCTAAC---AGGTGGGGGT	478	-3	1.90%
GGAATCTGCCTAACAGG-GGTGGGGGT	323	-1	1.28%
GGAATCTGCCTAACCAGGAGGTGGGGGT	245	+1 Ins	0.97%
GGAATCTGCCTAACAG-AGGTGGGGGT	191	-1	0.76%
GGAATCTGCCTAACA--AGGTGGGGGT	175	-2	0.70%
GGAATCTGCCTAACAGGaGGGTGGGGGT	158	+1 a	0.63%
GGAATCTGCCTA-----AGGTGGGGGT	128	-5	0.51%

P51-P57-YW-W3-Syn25crAAVS1h-AAV6KI

Total | INDEL	4h	Ins	Del	HDR
82265 | 15.23%		10.47%	2.22%	2.53%

Typical seqs	Reads	Type	pct.
TAAGGAATCTGCCTAAC|AGG**AGG**TGGG	69740	WT	84.77%
TAAGGAATCTGCCTAACcAGGAGGTGGG	6954	+1 c	8.45%
TAAGGAATCTGCCTAAGTTTAAACTACGCGTG	1957	+5 HDR	2.38%
TAAGGAATCTGCCTAA-AGGAGGTGGG	520	-1	0.63%
TAAGGAATCTGCCTAACaAGGAGGTGGG	403	+1 a	0.49%
TAAGGAATCTGCCTA--AGGAGGTGGG	412	-2	0.50%
TAAGGAATCTGCCTA-CAGGAGGTGGG	213	-1	0.26%
TAAGGAATCTGCCT---AGGAGGTGGG	128	-3	0.16%
TAAGGAATCTGCCTAAC-GGAGGTGGG	84	-1	0.10%
TAAGGAGTCTGCCTAACCAGGAGGTGGG	63	+1 Ins	0.08%

Total | INDEL	8h	Ins	Del	HDR
92060 | 53.16%		22.46%	7.62%	23.08%

Typical seqs	Reads	Type	pct.
TAAGGAATCTGCCTAAC|AGG**AGG**TGGG	43120	WT	46.84%
TAAGGAATCTGCCTAAGTTTAAACTACGCGTG	19582	+5 HDR	21.27%
TAAGGAATCTGCCTAACcAGGAGGTGGG	15579	+1 c	16.92%
TAAGGAATCTGCCTA--AGGAGGTGGG	1626	-2	1.77%
TAAGGAATCTGCCTAA-AGGAGGTGGG	1398	-1	1.52%
TAAGGAATCTGCCTAACaAGGAGGTGGG	908	+1 a	0.99%
TAAGGAATCTGCCTA-CAGGAGGTGGG	819	-1	0.89%
TAAGGAATCTGCCTAAC-GGAGGTGGG	585	-1	0.64%
TAAGGAATCTGCCT---AGGAGGTGGG	285	-3	0.31%
TA---------------AGGAGGTGGG	214	-15	0.23%

Total | INDEL	12h	Ins	Del	HDR
55234 | 62.39%		23.43%	8.82%	30.14%

Typical seqs	Reads	Type	pct.
TAAGGAATCTGCCTAAC|AGG**AGG**TGGG	20774	WT	37.61%
TAAGGAATCTGCCTAAGTTTAAACTACGCGTG	15313	+5 HDR	27.72%
TAAGGAATCTGCCTAACcAGGAGGTGGG	9290	+1 c	16.82%
TAAGGAATCTGCCTAACaAGGAGGTGGG	1072	+1 a	1.94%
TAAGGAATCTGCCTA--AGGAGGTGGG	800	-2	1.45%
TAAGGAATCTGCCTAA-AGGAGGTGGG	775	-1	1.40%
TAAGGAATCTGCCTAAC-GGAGGTGGG	399	-1	0.72%
TAAGGAATCTGCCTA-CAGGAGGTGGG	383	-1	0.69%
TAAGGAATCTGCCT---AGGAGGTGGG	351	-3	0.64%
TA---------------AGGAGGTGGG	170	-15	0.31%

Total | INDEL	24h	Ins	Del	HDR
55087 | 80.65%		27.42%	14.35%	38.88%

Typical seqs	Reads	Type	pct.
TAAGGAATCTGCCTAAC|AGG**AGG**TGGG	10657	WT	19.35%
TAAGGAATCTGCCTAAGTTTAAACTACGCG TG	19881	+5 HDR	36.09%
TAAGGAATCTGCCTAACcAGGAGGTGGG	11077	+1 c	20.11%
TAAGGAATCTGCCTA--AGGAGGTGGG	1136	-2	2.06%
TAAGGAATCTGCCTAA-AGGAGGTGGG	948	-1	1.72%
TAAGGAATCTGCCTAACaAGGAGGTGGG	941	+1 a	1.71%
TA---------------AGGAGGTGGG	764	-15	1.39%
TAAGGAATCTGCCTAAC-GGAGGTGGG	684	-1	1.24%
TAAGGAATCTGCCTA-CAGGAGGTGGG	445	-1	0.81%
TAAGGAATCTGCCT---AGGAGGTGGG	253	-3	0.46%

Total | INDEL	48h	Ins	Del	HDR
37387 | 86.46%		24.95%	14.93%	46.58%

Typical seqs	Reads	Type	pct.
TAAGGAATCTGCCTAAC|AGG**AGG**TGGG	5063	WT	13.54%
TAAGGAATCTGCCTAAGTTTAAACTACGCG TG	16344	+5 HDR	43.72%
TAAGGAATCTGCCTAACcAGGAGGTGGG	6766	+1 c	18.10%
TAAGGAATCTGCCTAA-AGGAGGTGGG	725	-1	1.94%
TAAGGAATCTGCCTA--AGGAGGTGGG	595	-2	1.59%
TAAGGAATCTGCCTAACaAGGAGGTGGG	555	+1 a	1.48%
TA---------------AGGAGGTGGG	499	-15	1.33%
TAAGGAATCTGCCTAAC-GGAGGTGGG	382	-1	1.02%
TAAGGAATCTGCCTA-CAGGAGGTGGG	365	-1	0.98%
TAAGGAATCTGCCTAAC--GAGGTGGG	209	-2	0.56%

P84-P88-YW-W9-Syn79crBCL11A2c-KO

4h

Total	INDEL	Ins	Del	HDR
49144	4.68%	4.07%	0.61%	0.00%

Typical seqs	Reads	Type	pct.
TGTTGTGATTCCGAGCTCCG**AGG**CGAG	46844	WT	95.32%
TGTTGTGATTCCGAGCTtCCGAGGCGAG	1743	+1 t	3.55%
TGTTGTGATTCCGAGC-CCGAGGCGAG	63	-1	0.13%
TGTTGTGAT-------TCCGAGGCGAG	46	-7	0.09%
TGTTGTGATTCCGAGCTGTCCGAGGCGAG	45	+2	0.09%
TGTTGTGATTCCGAG--CCGAGGCGAG	30	-2	0.06%
TGTTGTGATTCCGAGCCCCGAGGCGAG	373	-1	0.76%
TGTTGTGATTCCGAGCTTTCCGAGGCGAG	26	+2	0.05%
TGTCGTGATTCCGAGCTTCCGAGGCGAG	21	+1 Ins	0.04%
TGTTGTGATTCCGAGCTCCGGGGCGAG	333	-1	0.68%

8h

Total	INDEL	Ins	Del	HDR
33366	15.17%	12.99%	2.18%	0.00%

Typical seqs	Reads	Type	pct.
TGTTGTGATTCCGAGCTCCG**AGG**CGAG	28304	WT	84.83%
TGTTGTGATTCCGAGCTtCCGAGGCGAG	3798	+1 t	11.38%
TGTTGTGATTCCGAGC-CCGAGGCGAG	161	-1	0.48%
TGTTGTGATTCCGAG-TCCGAGGCGAG	113	-1	0.34%
TGTTGTGATTCCGAG--CCGAGGCGAG	106	-2	0.32%
TGTTGTGAT-------TCCGAGGCGAG	80	-7	0.24%
TGTTGTGATTCCG---TCCGAGGCGAG	51	-3	0.15%
TGTTGTGATTCCGAGCT-CGAGGCGAG	47	-1	0.14%
TGTTGTGATTCCGAGCTtCCGAGGCGGG	42	+1 t	0.13%
TGTTGTGATTCCGA----CGAGGCGAG	34	-4	0.10%

12h

Total	INDEL	Ins	Del	HDR
33799	27.76%	21.14%	6.61%	0.00%

Typical seqs	Reads	Type	pct.
TGTTGTGATTCCGAGCTCCG**AGG**CGAG	24418	WT	72.24%
TGTTGTGATTCCGAGCTtCCGAGGCGAG	6403	+1 t	18.94%
TGTTGTGAT-------TCCGAGGCGAG	842	-7	2.49%
TGTTGTGATTCCGAG--CCGAGGCGAG	177	-2	0.52%
TGTTGTGATTCCGAGC-CCGAGGCGAG	156	-1	0.46%
TGTTGTGATTCCGA--TCCGAGGCGAG	90	-2	0.27%
TGTTGTGATTCCG---TCCGAGGCGAG	74	-3	0.22%
TGTTGTGATTCCGAGCTtCCGAGGCGGG	88	+1 t	0.26%
TGTTGTGATTCCGAGCTTCCGAGGCGAGA	70	+2	0.21%
TGTTGTGATTCCGAG-TCCGAGGCGAG	63	-1	0.19%

24h

Total	INDEL	Ins	Del	HDR
18933	42.28%	22.82%	19.46%	0.00%

Typical seqs	Reads	Type	pct.
TGTTGTGATTCCGAGCTCCG**AGG**CGAG	10929	WT	57.72%
TGTTGTGATTCCGAGCTtCCGAGGCGAG	3741	+1 t	19.76%
TGTTGTGAT-------TCCGAGGCGAG	1936	-7	10.23%
TGTTGTGATTCCGAG-TCCGAGGCGAG	154	-1	0.81%
TGTTGTGATTCCGA--------GCGAG	137	-8	0.72%
TGTTGTGATTCCGAGC-CCGAGGCGAG	135	-1	0.71%
TGTTGTGATTCCGA--TCCGAGGCGAG	105	-2	0.55%
TGTTGTGAT-------TCCGAGGCGAG	1936	-8	10.23%
TGTTGTGATTCCGAGCT--GAGGCGAG	72	-2	0.38%
TGTTGTGATTC------------CGAG	65	-12	0.34%

48h

Total	INDEL	Ins	Del	HDR
15903	60.71%	19.12%	41.59%	0.00%

Typical seqs	Reads	Type	pct.
TGTTGTGATTCCGAGCTCCG**AGG**CGAG	6249	WT	39.29%
TGTTGTGAT-------TCCGAGGCGAG	3933	-7	24.73%
TGTTGTGATTCCGAGCTtCCGAGGCGAG	2478	+1 t	15.58%
TGTTGTGATTCCGA--------GCGAG	431	-8	2.71%
TGTTGTGAT-------TCCGAGGCGAG	3933	-8	24.73%
TGTTGTGATTCCGAG-TCCGAGGCGAG	159	-1	1.00%
TGTTGTGATTCCGAGC------GCGAG	150	-6	0.94%
TGTTGTGATTCCG---TCCGAGGCGAG	137	-3	0.86%
TGTTGTGATTCCGAGCTCTCCGAGGCGAG	110	+2	0.69%
TGTTGTGATTCCGA--TCCGAGGCGAG	93	-2	0.58%

P84-P88-YW-W9-Syn83crBCL11A4b-KO

4h

Total | INDEL	Ins	Del	HDR
93691 | 4.39%	0.40%	3.99%	0.00%

Typical seqs	Reads	Type	pct.
ACTTCATGCGGAGGCCC|CGT**GGG**AGGA	89582	WT	95.61%
ACTTCATGCGGAGG-CCCGTGGGAGGA	2236	-1	2.39%
ACTTCATGCGGAGGCCCCGT-GGAGGA	307	-1	0.33%
ACTTCATGCGGAGGCCC-----GAGGA	284	-5	0.30%
ACTTCATGCGGAGG--CCGTGGGAGGA	230	-2	0.25%
ACTTCATGCGGAGG---CGTGGGAGGA	221	-3	0.24%
ACTTCATGCGGAGGCCCcCGTGGGAGGA	185	+1 c	0.20%
ACTTCATGCGGAGGCCCtCGTGGGAGGA	109	+1 t	0.12%
ACTTCATGCGGAGGCCC--TGGGAGGA	80	-2	0.09%
ACTTCATGCGGAGGCCCTTCGTGGGAGGA	71	+2	0.08%

8h

Total | INDEL	Ins	Del	HDR
43604 | 30.74%	4.77%	25.97%	0.00%

Typical seqs	Reads	Type	pct.
ACTTCATGCGGAGGCCC|CGT**GGG**AGGA	30201	WT	69.26%
ACTTCATGCGGAGG-CCCGTGGGAGGA	4667	-1	10.70%
ACTTCATGCGGAGGCCCcCGTGGGAGGA	979	+1 c	2.25%
ACTTCATG---------CGTGGGAGGA	631	-9	1.45%
ACTTCATGCGGAGG---CGTGGGAGGA	577	-3	1.32%
ACTTCATGC------------GGAGGA	551	-12	1.26%
ACTTCATGCGGAGG--CCGTGGGAGGA	475	-2	1.09%
ACTTCATGCGGAGGCCC-----GAGGA	288	-5	0.66%
ACTTCATGCGGAGGCCCtCGTGGGAGGA	237	+1 t	0.54%
ACTTCATGC---------------GGA	211	-15	0.48%

12h

Total | INDEL	Ins	Del	HDR
36478 | 57.59%	8.28%	49.31%	0.00%

Typical seqs	Reads	Type	pct.
ACTTCATGCGGAGGCCC|CGT**GGG**AGGA	15471	WT	42.41%
ACTTCATGCGGAGG-CCCGTGGGAGGA	5049	-1	13.84%
ACTTCATG---------CGTGGGAGGA	1496	-9	4.10%
ACTTCATGCGGAGGCCCcCGTGGGAGGA	1197	+1 c	3.28%
ACTTCATGC------------GGAGGA	1293	-12	3.54%
ACTTCAT-----------GTGGGAGGA	1120	-11	3.07%
ACTTCATGCGGAGG--CCGTGGGAGGA	970	-2	2.66%
ACTTCATGCGGAGGC------------	545	-12	1.49%
ACTTCATGCGGAGG---CGTGGGAGGA	523	-3	1.43%
ACTTCATGC---------------GGA	520	-15	1.43%

24h

Total | INDEL	Ins	Del	HDR
47157 | 19.50%	4.32%	15.18%	0.00%

Typical seqs	Reads	Type	pct.
ACTTCATGCGGAGGCCC|CGT**GGG**AGGA	37960	WT	80.50%
ACTTCATGCGGAGG-CCCGTGGGAGGA	2842	-1	6.03%
ACTTCATGCGGAGGCCCcCGTGGGAGGA	1099	+1 c	2.33%
ACTTCATGC------------GGAGGA	508	-12	1.08%
ACTTCATGCGGAGG---CGTGGGAGGA	506	-3	1.07%
ACTTCATGC---------------GGA	377	-15	0.80%
ACTTCATG---------CGTGGGAGGA	308	-9	0.65%
ACTTCATGCGGAGGCCC---------A	269	-9	0.57%
ACTTCATGCGGAGGCCC-----GAGGA	213	-5	0.45%
ACTTCATGCGGAGG--CCGTGGGAGGA	207	-2	0.44%

48h

Total | INDEL	Ins	Del	HDR
26119 | 72.60%	10.10%	62.51%	0.00%

Typical seqs	Reads	Type	pct.
ACTTCATGCGGAGGCCC|CGT**GGG**AGGA	7156	WT	27.40%
ACTTCATGCGGAGG-CCCGTGGGAGGA	3470	-1	13.29%
ACTTCATGC------------GGAGGA	2558	-12	9.79%
ACTTCATG---------CGTGGGAGGA	1276	-9	4.89%
ACTTCATGCGGAGGCCCcCGTGGGAGGA	1063	+1 c	4.07%
ACTTCATGCGGAGG--CCGTGGGAGGA	604	-2	2.31%
ACTTCAT-----------GTGGGAGGA	611	-11	2.34%
ACTTCA-------------TGGGAGGA	607	-13	2.32%
ACTTCATGCGGAGG---CGTGGGAGGA	557	-3	2.13%
ACTTCATGC---------------GGA	492	-15	1.88%

P84-P88-YW-W9-Syn87crBCL11A5b-KO

Total | INDEL	4h	Ins	Del	HDR
66459 | 24.01%		2.42%	21.59%	0.00%

Typical seqs	Reads	Type	pct.
GGGATTACCGAGTCACC|ACC**AGG**CTGC	50503	WT	75.99%
GGGATTACCGAGT---CACCAGGCTGC	6980	-3	10.50%
GGGATTACCGAGTCA-CACCAGGCTGC	4994	-1	7.51%
GGGATTACCGAGTCACCcACCAGGCTGC	1407	+1 c	2.12%
GGGATTACCGAGTCAC-------CTGC	228	-7	0.34%
GGGATTACCGAGTCAC--CCAGGCTGC	235	-2	0.35%
GGGATTACCGAGTCACC-CCAGGCTGC	139	-1	0.21%
GGGATT--------ACCACCAGGCTGC	96	-8	0.14%
GGGATTACCGAGTCACCA-CAGGCTGC	80	-1	0.12%
GGGACTACCGAGT---CACCAGGCTGC	77	-3	0.12%

Total | INDEL	8h	Ins	Del	HDR
38982 | 47.93%		9.68%	38.25%	0.00%

Typical seqs	Reads	Type	pct.
GGGATTACCGAGTCACC|ACC**AGG**CTGC	20296	WT	52.07%
GGGATTACCGAGT---CACCAGGCTGC	7275	-3	18.66%
GGGATTACCGAGTCA-CACCAGGCTGC	4938	-1	12.67%
GGGATTACCGAGTCACCcACCAGGCTGC	2501	+1 c	6.42%
GGGATT-----------ACCAGGCTGC	506	-11	1.30%
GGGATTACCGAGTCACCtACCAGGCTGC	461	+1 t	1.18%
GGGATTACCGAGTCA--ACCAGGCTGC	175	-2	0.45%
GGGATTACCGAGTCA-CATCAGGCTGC	145	-1	0.37%
GGGATTACCGAGTCACCCCACCAGGCTGC	125	+2	0.32%
GGGATTACCGAGT--CCACCAGGCTGC	92	-2	0.24%

Total | INDEL	12h	Ins	Del	HDR
19357 | 71.05%		11.61%	59.44%	0.00%

Typical seqs	Reads	Type	pct.
GGGATTACCGAGTCACC|ACC**AGG**CTGC	5604	WT	28.95%
GGGATTACCGAGT---CACCAGGCTGC	5207	-3	26.90%
GGGATTACCGAGTCA-CACCAGGCTGC	3745	-1	19.35%
GGGATTACCGAGTCACCcACCAGGCTGC	1680	+1 c	8.68%
GGGATT-----------ACCAGGCTGC	567	-11	2.93%
GGGATTACCGAGT------CAGGCTGC	112	-6	0.58%
GGGATTACCGAGTCACCtACCAGGCTGC	106	+1 t	0.55%
GGGA--------------------TGC	95	-20	0.49%
G--------------------GGCTGC	96	-20	0.50%
GGGATTACCGAGTCACC-CCAGGCTGC	86	-1	0.44%

Total | INDEL	24h	Ins	Del	HDR
27527 | 90.64%		14.06%	76.58%	0.00%

Typical seqs	Reads	Type	pct.
GGGATTACCGAGTCACC|ACC**AGG**CTGC	2576	WT	9.36%
GGGATTACCGAGT---CACCAGGCTGC	9476	-3	34.42%
GGGATTACCGAGTCA-CACCAGGCTGC	5333	-1	19.37%
GGGATTACCGAGTCACCcACCAGGCTGC	2790	+1 c	10.14%
GGGATT-----------ACCAGGCTGC	1554	-11	5.65%
GGGATTACCGAGT------CAGGCTGC	299	-6	1.09%
GGGATTACCGAGT----------CTGC	226	-10	0.82%
GGGATTACCGAGTCACCtACCAGGCTGC	185	+1 t	0.67%
GGGATTACCG----------AGGCTGC	168	-10	0.61%
GGGATT--------------AGGCTGC	145	-14	0.53%

Total | INDEL	48h	Ins	Del	HDR
14476 | 89.30%		11.48%	77.82%	0.00%

Typical seqs	Reads	Type	pct.
GGGATTACCGAGTCACC|ACC**AGG**CTGC	1549	WT	10.70%
GGGATTACCGAGT---CACCAGGCTGC	4974	-3	34.36%
GGGATTACCGAGTCA-CACCAGGCTGC	2446	-1	16.90%
GGGATT-----------ACCAGGCTGC	1189	-11	8.21%
GGGATTACCGAGTCACCcACCAGGCTGC	1190	+1 c	8.22%
GGGATTACCGAGT------CAGGCTGC	222	-6	1.53%
GGGATTACCG----------AGGCTGC	116	-10	0.80%
GGGATTACCGAGTC-------------	82	-18	0.57%
GGGATT--------------AGGCTGC	56	-14	0.39%
GGGATTACCGAGTCACCtACCAGGCTGC	59	+1 t	0.41%

P84-P88-YW-W9-Syn92crBCL11A7b-KO

Time	Total	INDEL	Ins	Del	HDR
4h	110144	21.81%	19.54%	2.27%	0.00%
8h	68221	56.86%	51.93%	4.94%	0.00%
12h	31817	67.46%	58.10%	9.36%	0.00%
24h	48474	88.94%	61.90%	27.04%	0.00%
48h	46834	90.98%	53.13%	37.85%	0.00%

4h

Typical seqs	Reads	Type	pct.
GCTTCGTGACGTTGGAT|GGA**GGG**TTTT	86120	WT	78.19%
GCTTCGTGACGTTGGATtGGAGGGTTTT	17830	+1 t	16.19%
GCTTCGTGACGT----TGGAGGGTTTT	1003	-4	0.91%
GCTTCGTGACGTTGGAT-GAGGGTTTT	475	-1	0.43%
GCTTCGTGACGTTGGATaGGAGGGTTTT	365	+1 a	0.33%
GCTTCGTGACGTTGGA-GGAGGGTTTT	276	-1	0.25%
GCTTCGTGACGTTG--TGGAGGGTTTT	403	-2	0.37%
GCTTCGCGACGTTGGATTGGAGGGTTTT	276	+1 Ins	0.25%
GCTTCGTGACGCTGGATTGGAGGGTTTT	266	+1 Ins	0.24%
GCTTCGTGGCGTTGGATTGGAGGGTTTT	213	+1 Ins	0.19%

8h

Typical seqs	Reads	Type	pct.
GCTTCGTGACGTTGGAT|GGA**GGG**TTTT	29429	WT	43.14%
GCTTCGTGACGTTGGATtGGAGGGTTTT	28633	+1 t	41.97%
GCTTCGTGACGT----TGGAGGGTTTT	1386	-4	2.03%
GCTTC------------GGAGGGTTTT	542	-12	0.79%
GCTTCGTGACGCTGGATTGGAGGGTTTT	454	+1 Ins	0.67%
GCTTCGTGGCGTTGGATTGGAGGGTTTT	375	+1 Ins	0.55%
GCTTCGCGACGTTGGATTGGAGGGTTTT	370	+1 Ins	0.54%
GCTTCGTGACGTTGGATtGGAGGGTTCT	347	+1 t	0.51%
GCTTCGTGACGTTGGA-------TTTT	333	-7	0.49%
GCTTCGTGACGTTGGATtGGAGGGTTTC	316	+1 t	0.46%

12h

Typical seqs	Reads	Type	pct.
GCTTCGTGACGTTGGAT|GGA**GGG**TTTT	10352	WT	32.54%
GCTTCGTGACGTTGGATtGGAGGGTTTT	15555	+1 t	48.89%
GCTTCGTGACGT----TGGAGGGTTTT	1780	-4	5.59%
GCTT------------TGGAGGGTTTT	357	-12	1.12%
GCTTCGTGACGTTGGACTGGAGGGTTTT	249	+1 Ins	0.78%
GCTCCGTGACGTTGGATTGGAGGGTTTT	205	+1 Ins	0.64%
GCTTCGTGACGTTGGATtGGGGGGTTTT	170	+1 t	0.53%
GCTTCGTGACGTTGGAT-----TTTTT	155	-5	0.49%
GCTTCGTGACGTTGGA--GAGGGTTTT	160	-2	0.50%
GCTTCGTGACGTTGGATtGGAGGGTTCT	137	+1 t	0.43%

24h

Typical seqs	Reads	Type	pct.
GCTTCGTGACGTTGGAT|GGA**GGG**TTTT	5361	WT	11.06%
GCTTCGTGACGTTGGATtGGAGGGTTTT	24184	+1 t	49.89%
GCTTCGTGACGT----TGGAGGGTTTT	6854	-4	14.14%
GCTTCGTGACGTT-------GGGTTTT	450	-7	0.93%
GCTTCGTGACGTTGGA-GGAGGGTTTT	442	-1	0.91%
GCTTCGTGAC------------GTTTT	339	-12	0.70%
GCTTCG----------TGGAGGGTTTT	334	-10	0.69%
GCTTCGTGACGTTGGATtGGGGGGTTTT	255	+1 t	0.53%
GCTTCGTGACGTTGGATtGGAGGGTTCT	250	+1 t	0.52%
GCTTCGT-----------GAGGGTTTT	223	-11	0.46%

48h

Typical seqs	Reads	Type	pct.
GCTTCGTGACGTTGGAT|GGA**GGG**TTTT	4226	WT	9.02%
GCTTCGTGACGTTGGATtGGAGGGTTTT	20263	+1 t	43.27%
GCTTCGTGACGT----TGGAGGGTTTT	7957	-4	16.99%
GCTTCGTGACGTT-------GGGTTTT	1178	-7	2.52%
GCTTCGTGAC------------GTTTT	1089	-12	2.33%
GCTTCGTGACGTT--------GGTTTT	596	-8	1.27%
GCTTCGTGACGTTGGA-------TTTT	259	-7	0.55%
GCTTCGT-----------GAGGGTTTT	250	-11	0.53%
GCTTCGTGACGTTGGATtGGAGGGTTCT	229	+1 t	0.49%
GCTTCGTGGCGTTGGATTGGAGGGTTTT	226	+1 Ins	0.48%

P84-P88-YW-W9-Syn94crBCL11A7d-KO

4h

Total | INDEL	Ins	Del	HDR
21567 | 15.93%	13.72%	2.21%	0.00%

Typical seqs	Reads	Type	pct.
GCGTAGTGTTGGGTCCT|ACC**TGG**CCAC	18132	WT	84.07%
GCGTAGTGTTGGGTCCTtACCTGGCCAC	2244	+1 t	10.40%
GCGTAGTGTTGGGTCCTCTACCTGGCCAC	281	+2	1.30%
GCGTAGTGTTGGGT----CCTGGCCAC	98	-4	0.45%
GCGTAGTGTTGGGT-CTACCTGGCCAC	74	-1	0.34%
GCGTAGTGTTGGGTCC-ACCTGGCCAC	70	-1	0.32%
GCGTAGTGTTGGGTCCT-CCTGGCCAC	54	-1	0.25%
GCGTAGTGTTGGGTCCCTACCTGGCCAC	36	+1 Ins	0.17%
GCGTAGTGTTGGGTCCTACCT-GCCAC	26	-1	0.12%
GCGTAGTGTTGGGTCCTgACCTGGCCAC	25	+1 g	0.12%

8h

Total | INDEL	Ins	Del	HDR
14762 | 35.98%	29.72%	6.26%	0.00%

Typical seqs	Reads	Type	pct.
GCGTAGTGTTGGGTCCT|ACC**TGG**CCAC	9451	WT	64.02%
GCGTAGTGTTGGGTCCTtACCTGGCCAC	3499	+1 t	23.70%
GCGTAGTGTTGGGTCCTCTACCTGGCCAC	235	+2	1.59%
GCGTAGTGTTGGGT----CCTGGCCAC	181	-4	1.23%
GCGTAGTGTTGGGTCC-ACCTGGCCAC	113	-1	0.77%
GCGTAGTGTTGGGTC--ACCTGGCCAC	77	-2	0.52%
GCGTAGTGTTGGGT-CTACCTGGCCAC	68	-1	0.46%
GCGTAGTGTTGGGTC-----------C	56	-11	0.38%
GCGTAGTGTTGGGTCCCTACCTGGCCAC	46	+1 Ins	0.31%
GCGTAGTGTTGGG---TACCTGGCCAC	45	-3	0.30%

12h

Total | INDEL	Ins	Del	HDR
9034 | 52.73%	43.13%	9.61%	0.00%

Typical seqs	Reads	Type	pct.
GCGTAGTGTTGGGTCCT|ACC**TGG**CCAC	4270	WT	47.27%
GCGTAGTGTTGGGTCCTtACCTGGCCAC	3163	+1 t	35.01%
GCGTAGTGTTGGGT----CCTGGCCAC	208	-4	2.30%
GCGTAGTGTTGGGTCCTCTACCTGGCCAC	174	+2	1.93%
GCGTAGTGTTGGGTCC-ACCTGGCCAC	88	-1	0.97%
GCGTAGTGT-----------TGGCCAC	70	-11	0.77%
GCGTAGTGTTGGGTC-----------C	48	-11	0.53%
GCGTAGTGTTGGGT----------CAC	42	-10	0.46%
GCGTAGTGTTGGGTCCCTACCTGGCCAC	40	+1 Ins	0.44%
GCGTAGTGTTGGGTCCT--------AC	37	-8	0.41%

24h

Total | INDEL	Ins	Del	HDR
9577 | 70.23%	54.87%	15.36%	0.00%

Typical seqs	Reads	Type	pct.
GCGTAGTGTTGGGTCCT|ACC**TGG**CCAC	2851	WT	29.77%
GCGTAGTGTTGGGTCCTtACCTGGCCAC	4351	+1 t	45.43%
GCGTAGTGTTGGGT----CCTGGCCAC	319	-4	3.33%
GCGTAGTGTTGGGTCCTCTACCTGGCCAC	213	+2	2.22%
GCGTAGTGTTGGGTCC-ACCTGGCCAC	108	-1	1.13%
GCGTAGTGTTGGGT-CTACCTGGCCAC	92	-1	0.96%
GCGTAGTGTTGGGTC-----------C	85	-11	0.89%
GCGTAGTGTTGGGT------------C	68	-12	0.71%
GCGTAGTGTTGGGTCCTaACCTGGCCAC	59	+1 a	0.62%
GCGTAGTGTTGGGTC--ACCTGGCCAC	57	-2	0.60%

48h

Total | INDEL	Ins	Del	HDR
8109 | 80.10%	54.15%	25.95%	0.00%

Typical seqs	Reads	Type	pct.
GCGTAGTGTTGGGTCCT|ACC**TGG**CCAC	1614	WT	19.90%
GCGTAGTGTTGGGTCCTtACCTGGCCAC	3596	+1 t	44.35%
GCGTAGTGTTGGGT----CCTGGCCAC	546	-4	6.73%
GCGTAGTGT-----------TGGCCAC	135	-11	1.66%
GCGTAGTGTTGGGTCCTCTACCTGGCCAC	108	+2	1.33%
GCGTAGTGTTGGGTCC-ACCTGGCCAC	97	-1	1.20%
GCGTAGTGTTGGGT-CTACCTGGCCAC	95	-1	1.17%
GCGTAGTGTTGGGTC-----------C	89	-11	1.10%
GCGTAGTGTTGGGTC--ACCTGGCCAC	75	-2	0.92%
GCGTAGTGTTGGGT----------CAC	71	-10	0.88%

P84-P88-YW-W9-Syn96crBCL11A8b-KO

Total | INDEL	4h	Ins	Del	HDR
68652 | 4.56%		0.99%	3.57%	0.00%

Typical seqs	Reads	Type	pct.
GCGGGCCACCCACATTC|ATT**GGG**GGAA	65523	WT	95.44%
GCGGGCCACCCA----CATTGGGGGAA	800	-4	1.17%
GCGGGCCACCCACATT-ATTGGGGGAA	626	-1	0.91%
GCGGGCCACCCACATTC-TTGGGGGAA	227	-1	0.33%
GCGGGCCACCCACATTCcATTGGGGGAA	235	+1 c	0.34%
GCGGGCCACCCACATTCtATTGGGGGAA	128	+1 t	0.19%
GCGGGCCACCCACATTCaATTGGGGGAA	110	+1 a	0.16%
GCGGGCCACCCACA---ATTGGGGGAA	94	-3	0.14%
GCGGGCCACCCACATTC--TGGGGGAA	89	-2	0.13%
GCGGGCCACCCACAT---TTGGGGGAA	61	-3	0.09%

Total | INDEL	8h	Ins	Del	HDR
13251 | 11.24%		1.58%	9.66%	0.00%

Typical seqs	Reads	Type	pct.
GCGGGCCACCCACATTC|ATT**GGG**GGAA	11761	WT	88.76%
GCGGGCCACCCA----CATTGGGGGAA	596	-4	4.50%
GCGGGCCACCCACATT-ATTGGGGGAA	202	-1	1.52%
GCGGGCCACCCACATTC-TTGGGGGAA	172	-1	1.30%
GCGGGCCACCCACATTCcATTGGGGGAA	111	+1 c	0.84%
GCGGGCCACCCACATTCaATTGGGGGAA	46	+1 a	0.35%
GC--------------------GGGAA	30	-20	0.23%
GCGGGCCACCCAC--TAA--GGTGGAA	29	-4	0.22%
GCGGGCCACCCACA---ATTGGGGGAA	24	-3	0.18%
GCGGGCCACCCACATTC--TGGGGGAA	23	-2	0.17%

Total | INDEL	12h	Ins	Del	HDR
25632 | 22.78%		3.53%	19.25%	0.00%

Typical seqs	Reads	Type	pct.
GCGGGCCACCCACATTC|ATT**GGG**GGAA	19793	WT	77.22%
GCGGGCCACCCA----CATTGGGGGAA	1527	-4	5.96%
GCGGGCCACCCACATTC-TTGGGGGAA	557	-1	2.17%
GCGGGCCACCCACATT-ATTGGGGGAA	518	-1	2.02%
GCGGGCCACCCACATTCcATTGGGGGAA	238	+1 c	0.93%
GCGGGCCACCCACATTCaATTGGGGGAA	231	+1 a	0.90%
GCGGGCCACCCACAT--ATTGGGGGAA	212	-2	0.83%
GCGGGCCACCCACA---ATTGGGGGAA	128	-3	0.50%
GCGGGCCACCCACATTC---GGGGGAA	102	-3	0.40%
GCGGGCCA--------CATTGGGGGAA	115	-8	0.45%

Total | INDEL	24h	Ins	Del	HDR
38945 | 50.29%		6.54%	43.75%	0.00%

Typical seqs	Reads	Type	pct.
GCGGGCCACCCACATTC|ATT**GGG**GGAA	19360	WT	49.71%
GCGGGCCACCCA----CATTGGGGGAA	5009	-4	12.86%
GCGGGCCACCCACATT-ATTGGGGGAA	1546	-1	3.97%
GCGGGCCACCCACATTC-TTGGGGGAA	1292	-1	3.32%
GCGGGCCACCCACATTCaATTGGGGGAA	685	+1 a	1.76%
GCGGGCCACCCACATTCcATTGGGGGAA	656	+1 c	1.68%
GCGGGCCACCCACAT--ATTGGGGGAA	412	-2	1.06%
GCGGGCCACCCACA---ATTGGGGGAA	387	-3	0.99%
GCGGGCCA--------CATTGGGGGAA	412	-8	1.06%
GCGGG-----------CATTGGGGGAA	452	-11	1.16%

Total | INDEL	48h	Ins	Del	HDR
30516 | 65.51%		9.01%	56.50%	0.00%

Typical seqs	Reads	Type	pct.
GCGGGCCACCCACATTC|ATT**GGG**GGAA	10526	WT	34.49%
GCGGGCCACCCA----CATTGGGGGAA	5185	-4	16.99%
GCGGGCCACCCACATT-ATTGGGGGAA	1120	-1	3.67%
GCGGGCCACCCACATTC-TTGGGGGAA	1005	-1	3.29%
GCGGGCCACCC----------------	636	-19	2.08%
GCGGGCCACCCACATTCcATTGGGGGAA	616	+1 c	2.02%
GCGGGCCACCCACATTCaATTGGGGGAA	554	+1 a	1.82%
GCGGG-----------CATTGGGGGAA	532	-11	1.74%
GCGGGCCA--------CATTGGGGGAA	415	-8	1.36%
GCGGGCCACCCACAT--ATTGGGGGAA	410	-2	1.34%

P84-P88-YW-W9-Syn99crBCL11A9a-KO

Total | INDEL	4h	Ins	Del	HDR
65674 | 22.78%		21.64%	1.14%	0.00%

Typical seqs	Reads	Type	pct.
GCGCAAGCTCCCGTTCT|CCGAGGAGTG	50715	WT	77.22%
GCGCAAACTCCCGTTCTTCCGAGGAGTG	6137	+1 Ins	9.34%
GCGCAAGCTCCCGTTCTtCCGAGGAGTG	6017	+1 t	9.16%
GCGCAAGCTCCCGTTCTTTCCGAGGAGTG	128	+2	0.19%
GCGCAAACTCCCGTTC-CCGAGGAGTG	125	-1	0.19%
GCGCAAACTCCCGT--TCCGAGGAGTG	130	-2	0.20%
GCGCAAACTC-------CCGAGGAGTG	112	-7	0.17%
GCGCAAACTCCCGTT-TCCGAGGAGTG	78	-1	0.12%
GCGCAAACTCCCGTTCTCTCCGAGGAGTG	74	+2	0.11%
GCGCAAACTCCCGTTCTTTCCGAGGAGTG	64	+2	0.10%

Total | INDEL	8h	Ins	Del	HDR
45942 | 46.01%		41.20%	4.81%	0.00%

Typical seqs	Reads	Type	pct.
GCGCAAGCTCCCGTTCT|CCGAGGAGTG	24804	WT	53.99%
GCGCAAGCTCCCGTTCTtCCGAGGAGTG	8492	+1 t	18.48%
GCGCAAACTCCCGTTCTTCCGAGGAGTG	7583	+1 Ins	16.51%
GCGCAAACTC-------CCGAGGAGTG	607	-7	1.32%
GCGCAAACTCCCGT--TCCGAGGAGTG	354	-2	0.77%
GCGCAAGCTC-------CCGAGGAGTG	264	-7	0.57%
GCGCAAACTCCCGTTCTTTCCGAGGAGTG	162	+2	0.35%
GCGCAAACTCCCG---TCCGAGGAGTG	121	-3	0.26%
GCGCAAGCTCCCGTTCTTTCCGAGGAGTG	108	+2	0.24%
GCGCAAA--------CTCCGAGGAGTG	91	-8	0.20%

Total | INDEL	12h	Ins	Del	HDR
32887 | 62.55%		54.51%	8.03%	0.00%

Typical seqs	Reads	Type	pct.
GCGCAAGCTCCCGTTCT|CCGAGGAGTG	12317	WT	37.45%
GCGCAAGCTCCCGTTCTtCCGAGGAGTG	8419	+1 t	25.60%
GCGCAAACTCCCGTTCTTCCGAGGAGTG	6916	+1 Ins	21.03%
GCGCAAACTC-------CCGAGGAGTG	768	-7	2.34%
GCGCAAACTCCCGTTCTTTCCGAGGAGTG	236	+2	0.72%
GCGCAAGCTCCCGT--TCCGAGGAGTG	248	-2	0.75%
GCGCAAGCTC-------CCGAGGAGTG	215	-7	0.65%
GCGCAAACTCCCGT--TCCGAGGAGTG	184	-2	0.56%
GCGCAAGCTCCCGTTC-CCGAGGAGTG	86	-1	0.26%
GCGCAAACTCCCG---TCCGAGGAGTG	76	-3	0.23%

Total | INDEL	24h	Ins	Del	HDR
25150 | 84.95%		70.99%	13.97%	0.00%

Typical seqs	Reads	Type	pct.
GCGCAAGCTCCCGTTCT|CCGAGGAGTG	3784	WT	15.05%
GCGCAAGCTCCCGTTCTtCCGAGGAGTG	8355	+1 t	33.22%
GCGCAAACTCCCGTTCTTCCGAGGAGTG	7101	+1 Ins	28.23%
GCGCAAACTC-------CCGAGGAGTG	793	-7	3.15%
GCGCAAGCTC-------CCGAGGAGTG	483	-7	1.92%
GCGCAAACTCCCGT--TCCGAGGAGTG	229	-2	0.91%
GCGCAAA--------CTCCGAGGAGTG	159	-8	0.63%
GCGCAAGCTCCCGT--TCCGAGGAGTG	149	-2	0.59%
GCGCAAGCTCCCGTTCTTTCCGAGGAGTG	126	+2	0.50%
GCGCAAACTCCCGTT-TCCGAGGAGTG	106	-1	0.42%

Total | INDEL	48h	Ins	Del	HDR
30950 | 90.21%		68.61%	21.60%	0.00%

Typical seqs	Reads	Type	pct.
GCGCAAGCTCCCGTTCT|CCGAGGAGTG	3031	WT	9.79%
GCGCAAGCTCCCGTTCTtCCGAGGAGTG	9719	+1 t	31.40%
GCGCAAACTCCCGTTCTTCCGAGGAGTG	8173	+1 Ins	26.41%
GCGCAAACTC-------CCGAGGAGTG	1908	-7	6.16%
GCGCAAGCTC-------CCGAGGAGTG	1210	-7	3.91%
GCGCAAACTCCCGT--TCCGAGGAGTG	302	-2	0.98%
GCGCAAACTCCCGTTCTTTCCGAGGAGTG	227	+2	0.73%
GCGCAAGCTCCCGT--TCCGAGGAGTG	213	-2	0.69%
GCGCAAA--------CTCCGAGGAGTG	151	-8	0.49%
GCGCAAACTCCCG---TCCGAGGAGTG	127	-3	0.41%

P84-P88-YW-W9-Syn103crBCL11A9e-KO

Total | INDEL	4h	Ins	Del	HDR
9510 | 0.58%		**0.21%**	**0.37%**	**0.00%**
**Typical seqs**		**Reads**	**Type**	**pct.**
ATGACGGTCAAGTCCGA|CGA**CGG**TCTC		9455	WT	99.42%
ATGACGGTCAAGTC---CGACGGTCTC		27	-3	0.28%
ATGACGGTCAAGTCCGAaCGACGGTCTC		14	+1 a	0.15%
ATGACGGTCAAGTCCGACGACGACGGTCTC		6	+3	0.06%
ATGACGGTCAAGTCCGA-GACGGTCTC		5	-1	0.05%
AT----------------GACGGTCTC		3	-16	0.03%

Total | INDEL	8h	Ins	Del	HDR
6292 | 5.12%		**1.46%**	**3.66%**	**0.00%**
**Typical seqs**		**Reads**	**Type**	**pct.**
ATGACGGTCAAGTCCGA|CGA**CGG**TCTC		5970	WT	94.88%
ATGACGGTCAAGTC---CGACGGTCTC		95	-3	1.51%
ATGACGGTCAAGTCCGAaCGACGGTCTC		74	+1 a	1.18%
AT----------------GACGGTCTC		63	-16	1.00%
ATGACGGTCAAGTCCGA-GACGGTCTC		30	-1	0.48%
ATGACGGTCAAGTCCG-CGACGGTCTC		9	-1	0.14%
ATGACGGTCAAGTCCGAgCGACGGTCTC		8	+1 g	0.13%
ATGACGGTCAAGTCCGAcCGACGGTCTC		7	+1 c	0.11%
ATGACGGTCAAGTCCG-----------		5	-15	0.08%
ATGACGGTCAAGTCCGAC-ACGGTCTC		4	-1	0.06%

Total | INDEL	12h	Ins	Del	HDR
5334 | 12.56%		**4.61%**	**7.95%**	**0.00%**
**Typical seqs**		**Reads**	**Type**	**pct.**
ATGACGGTCAAGTCCGA|CGA**CGG**TCTC		4664	WT	87.44%
ATGACGGTCAAGTCCGAaCGACGGTCTC		187	+1 a	3.51%
ATGACGGTCAAGTC---CGACGGTCTC		129	-3	2.42%
AT----------------GACGGTCTC		65	-16	1.22%
ATGACGGTCAAGTCCG-CGACGGTCTC		36	-1	0.67%
ATGACGGTCAAGTCCGA-GACGGTCTC		30	-1	0.56%
ATGACGGTCA------ACGACGGTCTC		16	-6	0.30%
ATGACGGTCAAGTCCGAtCGACGGTCTC		18	+1 t	0.34%
AT----------------GGCGGTCTC		11	-16	0.21%
ATGACGGTC-------ACGACGGTCTC		11	-7	0.21%

Total | INDEL	24h	Ins	Del	HDR
6917 | 38.62%		**11.52%**	**27.09%**	**0.00%**
**Typical seqs**		**Reads**	**Type**	**pct.**
ATGACGGTCAAGTCCGA|CGA**CGG**TCTC		4246	WT	61.38%
ATGACGGTCAAGTCCGAaCGACGGTCTC		552	+1 a	7.98%
ATGACGGTCAAGTC---CGACGGTCTC		441	-3	6.38%
AT----------------GACGGTCTC		414	-16	5.99%
ATGACGGTCAAGTCCGA-GACGGTCTC		98	-1	1.42%
ATGACGGTCAAGTCCG-CGACGGTCTC		95	-1	1.37%
ATGACGGTCAAG---------------		67	-15	0.97%
ATGACGGTCAAGTCCGAcCGACGGTCTC		53	+1 c	0.77%
ATGACGGTCAAGTCCGA----GGTCTC		48	-4	0.69%
ATGACGGTCA------ACGACGGTCTC		45	-6	0.65%

Total | INDEL	48h	Ins	Del	HDR
4340 | 58.41%		**13.69%**	**44.72%**	**0.00%**
**Typical seqs**		**Reads**	**Type**	**pct.**
ATGACGGTCAAGTCCGA|CGA**CGG**TCTC		1805	WT	41.59%
AT----------------GACGGTCTC		574	-16	13.23%
ATGACGGTCAAGTC---CGACGGTCTC		476	-3	10.97%
ATGACGGTCAAGTCCGAaCGACGGTCTC		436	+1 a	10.05%
ATGACGGTCAA-----------GTCTC		83	-11	1.91%
ATGACGGTCAAG---------------		49	-15	1.13%
ATGACGGTCAAGTCCG-CGACGGTCTC		48	-1	1.11%
ATGACGGTCAAGTCCGA-GACGGTCTC		43	-1	0.99%
ATGACGGTCAAGTCCGAcCGACGGTCTC		41	+1 c	0.94%
AT----------------GGCGGTCTC		37	-16	0.85%

P84-P88-YW-W9-Syn105crBCL11A9g-KO

4h

Total	INDEL	Ins	Del	HDR
109878	14.55%	13.88%	0.67%	0.00%

Typical seqs	Reads	Type	pct.
CAACTTACAAATACCCTGCG**GGG**CATA	93895	WT	85.45%
CAACTTACAAATACCCTtGCGGGGCATA	13071	+1 t	11.90%
CAACTTACAAATACCCCTGCGGGGCATA	218	+1 Ins	0.20%
CAACTTACAAATACCCTGC-GGGCATA	198	-1	0.18%
CAACTTACAAATA-CCTGCGGGGCATA	180	-1	0.16%
CAACTTACAAATA--CTGCGGGGCATA	157	-2	0.14%
CAACTTACAAATACCCTCCTGCGGGGCATA	157	+3	0.14%
CAACTTACAAATACCC-GCGGGGCATA	120	-1	0.11%
CAACTTACGAATACCCTTGCGGGGCATA	113	+1 Ins	0.10%
CAACTTACAAATACCCTTTGCGGGGCATA	118	+2	0.11%

8h

Total	INDEL	Ins	Del	HDR
64126	33.19%	30.97%	2.22%	0.00%

Typical seqs	Reads	Type	pct.
CAACTTACAAATACCCTGCG**GGG**CATA	42841	WT	66.81%
CAACTTACAAATACCCTtGCGGGGCATA	16675	+1 t	26.00%
CAACTTACAAATA-CCTGCGGGGCATA	347	-1	0.54%
CAACTTACAAATA--CTGCGGGGCATA	297	-2	0.46%
CAACTTACAAATACCCCTGCGGGGCATA	197	+1 Ins	0.31%
CAACTTACAAATACCCTTTGCGGGGCATA	179	+2	0.28%
CAACTTACGAATACCCTTGCGGGGCATA	144	+1 Ins	0.22%
CAACTTACAAATACCCTCTGCGGGGCATA	144	+2	0.22%
CAGCTTACAAATACCCTTGCGGGGCATA	136	+1 Ins	0.21%
CAACTTACAAATACCCTcGCGGGGCATA	127	+1 c	0.20%

12h

Total	INDEL	Ins	Del	HDR
44851	62.73%	56.87%	5.86%	0.00%

Typical seqs	Reads	Type	pct.
CAACTTACAAATACCCTGCG**GGG**CATA	16716	WT	37.27%
CAACTTACAAATACCCTtGCGGGGCATA	21486	+1 t	47.91%
CAACTTACAAATA-CCTGCGGGGCATA	538	-1	1.20%
CAACTTACAAATA--CTGCGGGGCATA	516	-2	1.15%
CAACTTACAAATACCCTCTGCGGGGCATA	372	+2	0.83%
CAACTTACAAATACCC-GCGGGGCATA	238	-1	0.53%
CAACTTACAAATACCCTTTGCGGGGCATA	192	+2	0.43%
CAGCTTACAAATACCCTTGCGGGGCATA	190	+1 Ins	0.42%
CAACTTACAAATA---TGCGGGGCATA	186	-3	0.41%
CAACTTACGAATACCCTTGCGGGGCATA	174	+1 Ins	0.39%

24h

Total	INDEL	Ins	Del	HDR
66399	84.68%	74.45%	10.23%	0.00%

Typical seqs	Reads	Type	pct.
CAACTTACAAATACCCTGCG**GGG**CATA	10175	WT	15.32%
CAACTTACAAATACCCTtGCGGGGCATA	41760	+1 t	62.89%
CAACTTACAAATA-CCTGCGGGGCATA	1127	-1	1.70%
CAACTTACAAATACCCTCTGCGGGGCATA	760	+2	1.14%
CAACTTACAAATA--CTGCGGGGCATA	691	-2	1.04%
CAACTTACAAATACCCCTGCGGGGCATA	615	+1 Ins	0.93%
CAACTTACAAATACCC-GCGGGGCATA	388	-1	0.58%
CAACTTACAA--------------ATA	367	-14	0.55%
CAACTTACAAATACCCTTTGCGGGGCATA	333	+2	0.50%
CAACTTACAAATA---TGCGGGGCATA	326	-3	0.49%

48h

Total	INDEL	Ins	Del	HDR
40308	87.43%	73.55%	13.87%	0.00%

Typical seqs	Reads	Type	pct.
CAACTTACAAATACCCTGCG**GGG**CATA	5068	WT	12.57%
CAACTTACAAATACCCTtGCGGGGCATA	24882	+1 t	61.73%
CAACTTACAAATA-CCTGCGGGGCATA	737	-1	1.83%
CAACTTACAAATACCCTCTGCGGGGCATA	613	+2	1.52%
CAACTTACAAATA--CTGCGGGGCATA	409	-2	1.01%
CAACTTA----------------CATA	369	-16	0.92%
CAACTTACAAATA---TGCGGGGCATA	341	-3	0.85%
CAACTTACAAATACCC-GCGGGGCATA	293	-1	0.73%
CAACTTACAAATACCCCTGCGGGGCATA	289	+1 Ins	0.72%
CAACTTACAAATACCCT-CGGGGCATA	250	-1	0.62%

P92-P99-YW-W9-Syn20crAAVS1c-KO

Total | INDEL	4h	Ins	Del	HDR
22878 | 35.06%		31.08%	3.93%	0.04%

Typical seqs	Reads	Type	pct.
TCTAACCCCCACCTCCT|GTTAGGCAGA	14858	WT	64.94%
TCTAACCCCCACCTCCTtGTTAGGCAGA	6204	+1 t	27.12%
TCTAACCCCCACCTCC--TTAGGCAGA	343	-2	1.50%
TCTAACCCCCACCTCCTGCTGTTAGGCAGA	119	+3	0.52%
TCTAACCCCA-CTCCTGTTAGGCAGA	59	-1	0.26%
TCTAACCCCCACCTCCCTGTTAGGCAGA	49	+1 Ins	0.21%
TCTAACCCCCACCTCCTtGTTGGGCAGA	46	+1 t	0.20%
TCTAACCCCGCCTCCTTGTTAGGCAGA	52	+1 Ins	0.23%
TCTAGCCCCCACCTCCTTGTTAGGCAGA	42	+1 Ins	0.18%
TCTAACCCCCACCTCCTcGTTAGGCAGA	46	+1 c	0.20%

Total | INDEL	8h	Ins	Del	HDR
47601 | 57.76%		47.65%	10.10%	0.01%

Typical seqs	Reads	Type	pct.
TCTAACCCCCACCTCCT|GTTAGGCAGA	20106	WT	42.24%
TCTAACCCCCACCTCCTtGTTAGGCAGA	19064	+1 t	40.05%
TCTAACCCCCACCTC-TGTTAGGCAGA	1033	-1	2.17%
TCTAACCCCCACCTCC--TTAGGCAGA	590	-2	1.24%
TCTAACCCCCACCTCC-GTTAGGCAGA	538	-1	1.13%
TCTAACCCCCACCTCCTCTGTTAGGCAGA	294	+2	0.62%
TCTAACCCCCACCTCCT---------A	216	-9	0.45%
TCTAACCCCCACCTCCTcGTTAGGCAGA	217	+1 c	0.46%
CCTAACCCCCACCTCCTTGTTAGGCAGA	192	+1 Ins	0.40%
TCTAACCC----------------AAT	184	-16	0.39%

Total | INDEL	12h	Ins	Del	HDR
76366 | 62.59%		48.83%	13.74%	0.02%

Typical seqs	Reads	Type	pct.
TCTAACCCCCACCTCCT|GTTAGGCAGA	28569	WT	37.41%
TCTAACCCCCACCTCCTtGTTAGGCAGA	30507	+1 t	39.95%
TCTAACCCCCACCTC-TGTTAGGCAGA	1048	-1	1.37%
TCTAACCCCCACC--------------	912	-15	1.19%
TCTAACCCCCACCTCC-GTTAGGCAGA	903	-1	1.18%
TCTAACCCCCACCTC--------CAGA	877	-8	1.15%
TCTAACCCCCACCTCCTCTGTTAGGCAGA	780	+2	1.02%
TCTAACCCCCACCTCC--TTAGGCAGA	630	-2	0.82%
TCTAA------------------CAGA	609	-18	0.80%
TCTAACCCTCACCTCCTTGTTAGGCAGA	382	+1 Ins	0.50%

Total | INDEL	24h	Ins	Del	HDR
72875 | 68.25%		51.42%	16.49%	0.33%

Typical seqs	Reads	Type	pct.
TCTAACCCCCACCTCCT|GTTAGGCAGA	23141	WT	31.75%
TCTAACCCCCACCTCCTtGTTAGGCAGA	31174	+1 t	42.78%
TCTAACCCCCACCTCC--TTAGGCAGA	1090	-2	1.50%
TCTAACCCCCACCTCC-GTTAGGCAGA	962	-1	1.32%
TCTAACCCCCACC--------------	852	-15	1.17%
TCTAACCCCCACCT---GTTAGGCAGA	702	-3	0.96%
TCTAACCCCCACCTC--GTTAGGCAGA	678	-2	0.93%
TCTAACCCCCACCTC--------CAGA	577	-8	0.79%
TCTAACCCCA----CTGTTAGGCAGA	507	-4	0.70%
TCTAACCCCCACCTCCTCTGTTAGGCAGA	536	+2	0.74%

Total | INDEL	48h	Ins	Del	HDR
50803 | 67.16%		47.66%	19.49%	0.01%

Typical seqs	Reads	Type	pct.
TCTAACCCCCACCTCCT|GTTAGGCAGA	16683	WT	32.84%
TCTAACCCCCACCTCCTtGTTAGGCAGA	20237	+1 t	39.83%
TCTAACCCCCACC--------------	1517	-15	2.99%
TCTAACCCCA-----------------	550	-18	1.08%
TCTAACCCCCACCTCC--TTAGGCAGA	525	-2	1.03%
TCTAACCCCCACCTC-TGTTAGGCAGA	480	-1	0.94%
TCTAACCCCCACCTCCTCTGTTAGGCAGA	465	+2	0.92%
TCTAACCCCCACCTCC-GTTAGGCAGA	446	-1	0.88%
TCTAACCCCCAC-----------CAGA	312	-11	0.61%
TCTAACCCCCACCTC--------CAGA	260	-8	0.51%

P92-P99-YW-W9-Syn20crAAVS1c-AAV6KI

4h — Total 34920 | INDEL 44.26% | Ins 33.93% | Del 2.59% | HDR 7.73%

Typical seqs	Reads	Type	pct.
TCTAACCCCCACCTCCT|GTTAGGCAGA	19465	WT	55.74%
TCTAACCCCCACCTCCTtGTTAGGCAGA	10047	+1 t	28.77%
TCTAACCCACGCGTAGTTTAAACTTAGGCAGA	2523	+5 HDR	7.23%
TCTAACCCCCACCTCC--TTAGGCAGA	208	-2	0.60%
TCTAACCCCCACCTCC-GTTAGGCAGA	209	-1	0.60%
TCTAACCCCCACCTCCCTGTTAGGCAGA	98	+1 Ins	0.28%
TCTAACCCCCACCTCCTtGCTAGGCAGA	84	+1 t	0.24%
CCTAACCCCCACCTCCTTGTTAGGCAGA	78	+1 Ins	0.22%
TCTAACCCCCACCCCCTTGTTAGGCAGA	74	+1 Ins	0.21%
TCTAACCCCCACCTCCTcGTTAGGCAGA	76	+1 c	0.22%

8h — Total 35729 | INDEL 72.80% | Ins 41.46% | Del 4.15% | HDR 27.20%

Typical seqs	Reads	Type	pct.
TCTAACCCCCACCTCCT|GTTAGGCAGA	9718	WT	27.20%
TCTAACCCCCACCTCCTtGTTAGGCAGA	11648	+1 t	32.60%
TCTAACCCACGCGTAGTTTAAACTTAGGCAGA	8997	+5 HDR	25.18%
TCTAACCCCCACCTCC-GTTAGGCAGA	306	-1	0.86%
TCTAACCCCCACCTCCTCTGTTAGGCAGA	223	+2	0.62%
TCTAACCCCCACCT---GTTAGGCAGA	181	-3	0.51%
TCTAACCCCCACCTCCT-----GCAGA	149	-5	0.42%
TCTAACCCCCACCTCCTcGTTAGGCAGA	148	+1 c	0.41%
TCTAGCCCCCACCTCCTTGTTAGGCAGA	97	+1 Ins	0.27%
TCTAACCCCCACCTCCT---------A	96	-9	0.27%

12h — Total 20574 | INDEL 75.39% | Ins 34.54% | Del 11.38% | HDR 29.47%

Typical seqs	Reads	Type	pct.
TCTAACCCCCACCTCCT|GTTAGGCAGA	5063	WT	24.61%
TCTAACCCCCACCTCCTtGTTAGGCAGA	5773	+1 t	28.06%
TCTAACCCACGCGTAGTTTAAACTTAGGCAGA	5616	+5 HDR	27.30%
TCTAACCCCCACCTCC--TTAGGCAGA	348	-2	1.69%
TCTAACCCCCACCTC-TGTTAGGCAGA	225	-1	1.09%
TCTAACCCCCACC--------------	170	-15	0.83%
TCTAACCC---------------CAGA	113	-15	0.55%
TCTAACCCCCACCTCCT---------A	103	-9	0.50%
------------------GTTAGGCAGA	88	-18	0.43%
TCTAACCCCCACCTCC-GTTAGGCAGA	94	-1	0.46%

24h — Total 37197 | INDEL 84.85% | Ins 37.94% | Del 7.10% | HDR 39.81%

Typical seqs	Reads	Type	pct.
TCTAACCCCCACCTCCT|GTTAGGCAGA	5635	WT	15.15%
TCTAACCCACGCGTAGTTTAAACTTAGGCAGA	13356	+5 HDR	35.91%
TCTAACCCCCACCTCCTtGTTAGGCAGA	10555	+1 t	28.38%
TCTAACCCCCACCTCCTCTGTTAGGCAGA	290	+2	0.78%
TCTAACCCCCACCTCCT-TTAGGCAGA	275	-1	0.74%
TCTAACCCCCACCTCC-GTTAGGCAGA	210	-1	0.56%
TCTAA------------------CAGA	167	-18	0.45%
TCTAACCCCCACCTC-TGTTAGGCAGA	167	-1	0.45%
TCTAACCCACGCGTAGTTTAGACTTAGGCAGA	156	+5	0.42%
TCTAACCCCCACCTCC--TTAGGTGGCAGA	150	+1 Ins	0.40%

48h — Total 27978 | INDEL 83.38% | Ins 38.03% | Del 10.39% | HDR 34.96%

Typical seqs	Reads	Type	pct.
TCTAACCCCCACCTCCT|GTTAGGCAGA	4651	WT	16.62%
TCTAACCCACGCGTAGTTTAAACTTAGGCAGA	8997	+5 HDR	32.16%
TCTAACCCCCACCTCCTtGTTAGGCAGA	8072	+1 t	28.85%
TCTAACCCCCACC--------------	532	-15	1.90%
TCTAACCCCCACCTCCTCTGTTAGGCAGA	350	+2	1.25%
TCTAACCCCCACCTCC---TAGGCAGA	235	-3	0.84%
TCTAACCCCCACCTCC-GTTAGGCAGA	154	-1	0.55%
TCTAAC-----------------CAGA	149	-17	0.53%
TCTAACCC---------GTTAGGCAGA	136	-9	0.49%
TCTAACCCCCACCT--TGTTAGGCAGA	138	-2	0.49%

P92-P99-YW-W9-Syn25crAAVS1h-KO

Total | INDEL	4h	Ins	Del	HDR
70799 | 20.78%		12.34%	8.41%	0.03%

Typical seqs	Reads	Type	pct.
TCTAACCCCCACCTCCT|GTTAGGCAGA	56084	WT	79.22%
TCTAACCCCCACCTCCTgGTTAGGCAGA	7483	+1 g	10.57%
TCTAACCCCCACCTCCT-TTAGGCAGA	1291	-1	1.82%
TCTAACCCCCACCTCC--TTAGGCAGA	1196	-2	1.69%
TCTAA-CCCCACCTCCT-TTAGGCAGA	322	-2	0.45%
TCTAACCCCCACCTCCT-----GCAAA	292	-5	0.41%
TCTAACCCCCACCTCC-----------	276	-12	0.39%
TCTAACCCCCACC--------------	262	-15	0.37%
TCTAACCCCCACCT---GTTAGGCAGA	253	-3	0.36%
TCTAACCCCCACCTCC-GTTAGGCAGA	163	-1	0.23%

Total | INDEL	8h	Ins	Del	HDR
90823 | 31.75%		15.06%	16.58%	0.11%

Typical seqs	Reads	Type	pct.
TCTAACCCCCACCTCCT|GTTAGGCAGA	61991	WT	68.25%
TCTAACCCCCACCTCCTgGTTAGGCAGA	11294	+1 g	12.44%
TCTAACCCCCACCTCC--TTAGGCAGA	2798	-2	3.08%
TCTAACCCCCACCTCC-GTTAGGCAGA	1701	-1	1.87%
TCTAACCCCCACC--------------	1670	-15	1.84%
TCTAACCCCCACCTCCT-TTAGGCAGA	1482	-1	1.63%
TCTAACCCCCACCTCCTG-TAGGCAGA	1158	-1	1.28%
TCTAA------------GTTAGGCAGA	743	-12	0.82%
TCTAACCCCCACC----GTTAGGCAGA	657	-4	0.72%
TCTAACCCCCACCTCC----AGGCAGA	413	-4	0.45%

Total | INDEL	12h	Ins	Del	HDR
104198 | 39.63%		17.37%	22.19%	0.07%

Typical seqs	Reads	Type	pct.
TCTAACCCCCACCTCCT|GTTAGGCAGA	62905	WT	60.37%
TCTAACCCCCACCTCCTgGTTAGGCAGA	14247	+1 g	13.67%
TCTAACCCCCACCTCC--TTAGGCAGA	5064	-2	4.86%
TCTAACCCCCACCTCCT-TTAGGCAGA	3763	-1	3.61%
TCTAACCCCCACCTCC-GTTAGGCAGA	2194	-1	2.11%
TCTAACCCCCACC--------------	1395	-15	1.34%
TCTAACCCCCACCTCCTG-TAGGCAGA	1131	-1	1.09%
TCTAACCCCCACCTCCT----GGCAGA	944	-4	0.91%
TCTAACCCCCA----------------	887	-18	0.85%
TCTAACCCCCACCTCCTG-------GA	865	-7	0.83%

Total | INDEL	24h	Ins	Del	HDR
80620 | 52.35%		17.83%	34.45%	0.07%

Typical seqs	Reads	Type	pct.
TCTAACCCCCACCTCCT|GTTAGGCAGA	38412	WT	47.65%
TCTAACCCCCACCTCCTgGTTAGGCAGA	11738	+1 g	14.56%
TCTAACCCCCACC--------------	3996	-15	4.96%
TCTAACCCCCACCTCC--TTAGGCAGA	3445	-2	4.27%
TCTAACCCCCACCTCCT-TTAGGCAGA	2688	-1	3.33%
TCTAACCCCCACCTCC-GTTAGGCAGA	2042	-1	2.53%
TCTAACCCCCA----------------	1092	-18	1.35%
TCTAACCCCCACCTCCTG-TAGGCAGA	866	-1	1.07%
TCTAACCCCCACCTCCT----GGCAGA	832	-4	1.03%
TCTAACCCCCACCTCC---TAGGCAGA	517	-3	0.64%

Total | INDEL	48h	Ins	Del	HDR
95475 | 56.09%		15.03%	40.96%	0.09%

Typical seqs	Reads	Type	pct.
TCTAACCCCCACCTCCT|GTTAGGCAGA	41922	WT	43.91%
TCTAACCCCCACCTCCTgGTTAGGCAGA	11367	+1 g	11.91%
TCTAACCCCCACC--------------	10623	-15	11.13%
TCTAACCCCCACCTCCT-TTAGGCAGA	3700	-1	3.88%
TCTAACCCCCACCTCC--TTAGGCAGA	3134	-2	3.28%
TCTAACCCCCA----------------	1601	-18	1.68%
TCTAACCCCCACCTCCTG-TAGGCAGA	1433	-1	1.50%
TCTAACCCCCACCTCC-GTTAGGCAGA	1338	-1	1.40%
TCTAACCCC-------------CAGA	718	-14	0.75%
TCTAAC----------------CAGA	669	-17	0.70%

P92-P99-YW-W9-Syn25crAAVS1h-AAV6KI

Total | INDEL	4h	Ins	Del	HDR
162815 | 21.73%		9.38%	5.90%	6.45%

Typical seqs	Reads	Type	pct.
TCTAACCCCCACCTCCT|GTT**AGG**CAGA	127434	WT	78.27%
TCTAACCCCCACCTCCTgGTTAGGCAGA	11832	+1 g	7.27%
TCTAACCCACGCGTAGTTTAAACTTAGGCA GA	9390	+5 HDR	5.77%
TCTAACCCCCACC--------------	1526	-15	0.94%
TCTAACCCCCACCTCCTG---------	1338	-10	0.82%
TCTAACCCCCACCTCC--TTAGGCAGA	880	-2	0.54%
TCTAACCCCCACCTCC---TAGGCAGA	1265	-3	0.78%
TCTAACCCCCACCTCCT-TTAGGCAGA	673	-1	0.41%
TCTAACCCCCACCTCC-GTTAGGCAGA	493	-1	0.30%
TCTAACCCCCACCTCCT----GGCAGA	416	-4	0.26%

Total | INDEL	8h	Ins	Del	HDR
150315 | 45.85%		14.07%	9.48%	22.29%

Typical seqs	Reads	Type	pct.
TCTAACCCCCACCTCCT|GTT**AGG**CAGA	81402	WT	54.15%
TCTAACCCACGCGTAGTTTAAACTTAGGCA GA	30424	+5 HDR	20.24%
TCTAACCCCCACCTCCTgGTTAGGCAGA	13033	+1 g	8.67%
TCTAACCCCCACCTCC--TTAGGCAGA	3562	-2	2.37%
TCTAACCCCCACCTCCTG-TAGGCAGA	2082	-1	1.39%
TCTAACCCCCACCTCCTG---AGCAGA	908	-3	0.60%
TCTAACCCACGCGTAGTTTAGACTTAGGCA GA	796	+5	0.53%
TCTAACCCCCACC--------------	738	-15	0.49%
TCTAACCCCCACCTCCTGGAGTTAGGCAGA	665	+3	0.44%
-----------------GTTAGGCAGA	602	-18	0.40%

Total | INDEL	12h	Ins	Del	HDR
99577 | 55.85%		14.43%	15.02%	26.40%

Typical seqs	Reads	Type	pct.
TCTAACCCCCACCTCCT|GTT**AGG**CAGA	43962	WT	44.15%
TCTAACCCACGCGTAGTTTAAACTTAGGCA GA	23566	+5 HDR	23.67%
TCTAACCCCCACCTCCTgGTTAGGCAGA	8923	+1 g	8.96%
TCTAACCCCCACCTCCT-TTAGGCAGA	2933	-1	2.95%
TCTAACCCCCACCTC--GTTAGGCAGA	1300	-2	1.31%
TCTAACCCCCACCTCCTG---------	1220	-10	1.23%
TCTAACCCCA--------TTAGGCAGA	1264	-7	1.27%
TCTA-------------GTTAGGCAGA	1180	-13	1.19%
TCTAACCCCCACCTCCT----GGCAGA	1114	-4	1.12%
TCTAACCCCCACC--------------	1160	-15	1.16%

Total | INDEL	24h	Ins	Del	HDR
72486 | 68.52%		18.20%	16.56%	33.76%

Typical seqs	Reads	Type	pct.
TCTAACCCCCACCTCCT|GTT**AGG**CAGA	22819	WT	31.48%
TCTAACCCACGCGTAGTTTAAACTTAGGCA GA	22308	+5 HDR	30.78%
TCTAACCCCCACCTCCTgGTTAGGCAGA	8816	+1 g	12.16%
TCTAACCCCCACCTCC--TTAGGCAGA	2123	-2	2.93%
TCTAACCCCCACCTCCT--------GA	1230	-8	1.70%
TCTAACCCCCACC--------------	1075	-15	1.48%
TCTAACCCCCACCTC--------CAGA	885	-8	1.22%
TCTAACCCCCACCTCCT-TTAGGCAGA	872	-1	1.20%
TCTAACCCCA-----------------	709	-18	0.98%
TCTAACCC------------AGGCAGA	709	-12	0.98%

Total | INDEL	48h	Ins	Del	HDR
77381 | 73.62%		20.68%	10.45%	42.49%

Typical seqs	Reads	Type	pct.
TCTAACCCCCACCTCCT|GTT**AGG**CAGA	20413	WT	26.38%
TCTAACCCACGCGTAGTTTAAACTTAGGCA GA	29893	+5 HDR	38.63%
TCTAACCCCCACCTCCTgGTTAGGCAGA	11345	+1 g	14.66%
-------------------TAGGCAGA	1247	-19	1.61%
TCTAACCCCCACCTCCT-TTAGGCAGA	1051	-1	1.36%
TCTAACCCCCACCTC--GTTAGGCAGA	913	-2	1.18%
TCTAACCCCCACCTCC-----------	734	-16	0.95%
TCTAACCCCCACC--------------	849	-15	1.10%
TCTAACCCCCACCTCC--TTAGGCAGA	656	-2	0.85%
TCTAACCCCCACCTCCTG-TAGGCAGA	561	-1	0.72%

P108-P115-YW-W9-Syn26crCD326c-KO

Ctrl | INDEL<br>107568 | 47.92%	Ins<br>17.65%	Del<br>30.27%	HDR<br>0.00%
**Typical seqs**	**Reads**	**Type**	**pct.**
CCCTCCCGCGCCCCTCT|TCT**CGG**CGCG	56018	WT	52.08%
CCCTCCCGCGCCCCTCTtTCTCGGCGCG	16487	+1 t	15.33%
CCCTCCCGCGCCCC---TCTCGGCGCG	8250	-3	7.67%
CCCTCCCGCGCCCCTC-TCTCGGCGCG	3822	-1	3.55%
CCCTCCCGCGCCC-----CTCGGCGCG	2983	-5	2.77%
CCCTCC---------------------	2784	-21	2.59%
CCCTCC---------------------	2784	-23	2.59%
CCCTCC-----------------CGCG	662	-17	0.62%
CCCTCCCGCGCCCCT-TTCTCGGCGCG	511	-1	0.48%
CC---------------CTCGGCGCG	504	-16	0.47%

AZD7762 | INDEL<br>111532 | 61.69%	Ins<br>18.55%	Del<br>43.14%	HDR<br>0.00%
**Typical seqs**	**Reads**	**Type**	**pct.**
CCCTCCCGCGCCCCTCT|TCT**CGG**CGCG	42731	WT	38.31%
CCCTCCCGCGCCCCTCTtTCTCGGCGCG	17958	+1 t	16.10%
CCCTCCCGCGCCCC---TCTCGGCGCG	11008	-3	9.87%
CCCTCC---------------------	7365	-21	6.60%
CCCTCCCGCGCCCCTC-TCTCGGCGCG	5319	-1	4.77%
CCCTCCCGCGCCC-----CTCGGCGCG	4109	-5	3.68%
CCCTCC---------------------	7365	-23	6.60%
CCCTCC-------------------CG	1474	-19	1.32%
CCCTCC-----------------CGCG	940	-17	0.84%
CCCTCCCGC------------------	855	-24	0.77%

AZD7762+VE822 | INDEL<br>132429 | 53.58%	Ins<br>22.96%	Del<br>30.59%	HDR<br>0.03%
**Typical seqs**	**Reads**	**Type**	**pct.**
CCCTCCCGCGCCCCTCT|TCT**CGG**CGCG	61477	WT	46.42%
CCCTCCCGCGCCCCTCTtTCTCGGCGCG	26867	+1 t	20.29%
CCCTCCCGCGCCCC---TCTCGGCGCG	9667	-3	7.30%
CCCTCCCGCGCCCCTC-TCTCGGCGCG	7128	-1	5.38%
CCCTCC---------------------	4926	-21	3.72%
CCCTCCCGCGCCC-----CTCGGCGCG	2274	-5	1.72%
CCCTCC---------------------	4926	-23	3.72%
CCCTCC-----------------CGCG	977	-17	0.74%
CCCTCCCGCGCCCCT--TCTCGGCGCG	873	-2	0.66%
CCC--------------TCTCGGCGCG	843	-14	0.64%

AZT | INDEL<br>85296 | 46.50%	Ins<br>16.81%	Del<br>29.69%	HDR<br>0.00%
**Typical seqs**	**Reads**	**Type**	**pct.**
CCCTCCCGCGCCCCTCT|TCT**CGG**CGCG	45633	WT	53.50%
CCCTCCCGCGCCCCTCTtTCTCGGCGCG	12066	+1 t	14.15%
CCCTCCCGCGCCCC---TCTCGGCGCG	5780	-3	6.78%
CCCTCCCGCGCCCCTC-TCTCGGCGCG	3338	-1	3.91%
CCCTCCCGCGCCC-----CTCGGCGCG	2176	-5	2.55%
CCCTCC---------------------	2705	-21	3.17%
CCCTCC---------------------	2705	-23	3.17%
CCCTCC-------------------CG	673	-19	0.79%
CCC--------------TCTCGGCGCG	538	-14	0.63%
CCCTCC-----------------CGCG	552	-17	0.65%

CsH | INDEL<br>111868 | 47.98%	Ins<br>21.64%	Del<br>26.29%	HDR<br>0.05%
**Typical seqs**	**Reads**	**Type**	**pct.**
CCCTCCCGCGCCCCTCT|TCT**CGG**CGCG	58194	WT	52.02%
CCCTCCCGCGCCCCTCTtTCTCGGCGCG	21002	+1 t	18.77%
CCCTCCCGCGCCCCTC-TCTCGGCGCG	5800	-1	5.18%
CCCTCCCGCGCCCC---TCTCGGCGCG	4486	-3	4.01%
CCCTCC---------------------	3347	-21	2.99%
CCCTCCCGCGCCC-----CTCGGCGCG	1915	-5	1.71%
CCCTCCCGCGCCCCT--TCTCGGCGCG	858	-2	0.77%
CCCTCC---------------------	3347	-23	2.99%
CCCTCCCGCGCCCCTCTgTCTCGGCGCG	537	+1 g	0.48%
CCCTCCCGCGCCCCT--------CCCG	451	-8	0.40%

B02 | INDEL<br>95505 | 44.01%	Ins<br>16.00%	Del<br>28.00%	HDR<br>0.00%
**Typical seqs**	**Reads**	**Type**	**pct.**
CCCTCCCGCGCCCCTCT|TCT**CGG**CGCG	53476	WT	55.99%
CCCTCCCGCGCCCCTCTtTCTCGGCGCG	13045	+1 t	13.66%
CCCTCCCGCGCCCC---TCTCGGCGCG	5203	-3	5.45%
CCCTCCCGCGCCCCTC-TCTCGGCGCG	4335	-1	4.54%
CCCTCC---------------------	2779	-21	2.91%
CCCTCCCGCGCCC-----CTCGGCGCG	1731	-5	1.81%
CCCTCC-------------------CG	874	-19	0.92%
CCCTCC-----------------CGCG	610	-17	0.64%
CCCTCCCGCGCCCCT-TTCTCGGCGCG	598	-1	0.63%
CCC--------------TCTCGGCGCG	534	-14	0.56%

P108-P115-YW-W9-Syn26crCD326c-KO

DOPA | INDEL<br>142607 | 47.71%	Ins<br>16.82%	Del<br>30.88%	HDR<br>0.00%	M3814 | INDEL<br>133754 | 49.47%	Ins<br>13.60%	Del<br>35.84%	HDR<br>0.03%	Mirin | INDEL<br>97367 | 47.18%	Ins<br>20.31%	Del<br>26.84%	HDR<br>0.03%
**Typical seqs**	**Reads**	**Type**	**pct.**	**Typical seqs**	**Reads**	**Type**	**pct.**	**Typical seqs**	**Reads**	**Type**	**pct.**
CCCTCCCGCGCCCCTCT|TCT**CGG**CGCG	74574	WT	52.29%	CCCTCCCGCGCCCCTCT|TCT**CGG**CGCG	67586	WT	50.53%	CCCTCCCGCGCCCCTCT|TCT**CGG**CGCG	51430	WT	52.82%
CCCTCCCGCGCCCCTCTtTCTCGGCGCG	21454	+1 t	15.04%	CCCTCCCGCGCCCC---TCTCGGCGCG	14572	-3	10.89%	CCCTCCCGCGCCCCTCTtTCTCGGCGCG	17362	+1 t	17.83%
CCCTCCCGCGCCCC---TCTCGGCGCG	9920	-3	6.96%	CCCTCCCGCGCCCCTCTtTCTCGGCGCG	12986	+1 t	9.71%	CCCTCCCGCGCCCC---TCTCGGCGCG	5027	-3	5.16%
CCCTCCCGCGCCCCTC-TCTCGGCGCG	6779	-1	4.75%	CCCTCCCGCGCCCCTC-TCTCGGCGCG	4459	-1	3.33%	CCCTCCCGCGCCCCTC-TCTCGGCGCG	3808	-1	3.91%
CCCTCC---------------------	5248	-21	3.68%	CCCTCCCGCGCCC-----CTCGGCGCG	4394	-5	3.29%	CCCTCCCGCGCCC-----CTCGGCGCG	1995	-5	2.05%
CCCTCCCGCGCCC-----CTCGGCGCG	3156	-5	2.21%	CCCTCC---------------------	4106	-21	3.07%	CCCTCC---------------------	2544	-21	2.61%
CCCTCC---------------------	5248	-23	3.68%	CCCTCC-----------------CGCG	1768	-17	1.32%	CCCTCCCGCGCCCCT-TTCTCGGCGCG	717	-1	0.74%
CCCTCC-------------------CG	948	-19	0.66%	CCCTCC--------------CGGCGCG	1708	-14	1.28%	CCCTCC-------------------CG	727	-19	0.75%
CCC--------------TCTCGGCGCG	805	-14	0.56%	CC----------------CTCGGCGCG	1257	-16	0.94%	CCCTCC---------------------	2544	-23	2.61%
CCCTCC-----------------CGCG	685	-17	0.48%	CCCTCC---------------------	4106	-23	3.07%	CCC--------------TCTCGGCGCG	615	-14	0.63%

Nu7026 | INDEL<br>126760 | 44.96%	Ins<br>15.67%	Del<br>29.24%	HDR<br>0.05%	Nu7026+Tri A | INDEL<br>103994 | 51.14%	Ins<br>19.99%	Del<br>31.11%	HDR<br>0.04%	Nu7441 | INDEL<br>98513 | 44.90%	Ins<br>17.24%	Del<br>27.65%	HDR<br>0.00%
**Typical seqs**	**Reads**	**Type**	**pct.**	**Typical seqs**	**Reads**	**Type**	**pct.**	**Typical seqs**	**Reads**	**Type**	**pct.**
CCCTCCCGCGCCCCTCT|TCT**CGG**CGCG	69771	WT	55.04%	CCCTCCCGCGCCCCTCT|TCT**CGG**CGCG	50810	WT	48.86%	CCCTCCCGCGCCCCTCT|TCT**CGG**CGCG	54283	WT	55.10%
CCCTCCCGCGCCCCTCTtTCTCGGCGCG	17506	+1 t	13.81%	CCCTCCCGCGCCCCTCTtTCTCGGCGCG	17722	+1 t	17.04%	CCCTCCCGCGCCCCTCTtTCTCGGCGCG	14677	+1 t	14.90%
CCCTCCCGCGCCCC---TCTCGGCGCG	8840	-3	6.97%	CCCTCCCGCGCCCC---TCTCGGCGCG	8347	-3	8.03%	CCCTCCCGCGCCCC---TCTCGGCGCG	8380	-3	8.51%
CCCTCCCGCGCCCCTC-TCTCGGCGCG	5733	-1	4.52%	CCCTCCCGCGCCCCTC-TCTCGGCGCG	4335	-1	4.17%	CCCTCCCGCGCCCCTC-TCTCGGCGCG	2867	-1	2.91%
CCCTCCCGCGCCC-----CTCGGCGCG	3236	-5	2.55%	CCCTCCCGCGCCC-----CTCGGCGCG	2064	-5	1.98%	CCCTCCCGCGCCC-----CTCGGCGCG	2718	-5	2.76%
CCCTCC---------------------	3455	-21	2.73%	CCCTCC---------------------	3408	-21	3.28%	CCCTCC---------------------	2487	-21	2.52%
CCCTCC---------------------	3455	-23	2.73%	CCCTCC-----------------CGCG	1606	-17	1.54%	CCCTCC---------------------	2487	-23	2.52%
CC----------------CTCGGCGCG	871	-16	0.69%	CCCTCC---------------------	3408	-23	3.28%	CCC--------------TCTCGGCGCG	599	-14	0.61%
CCCTCCCGCGCCCCT----------CC	708	-10	0.56%	CCCTCCCGCGCCCCT-TTCTCGGCGCG	1217	-1	1.17%	CCCTCC-----------------CGCG	585	-17	0.59%
CCCTCCCGCGCCC----TCTCGGCGCG	685	-4	0.54%	CCCTCC-------------------CG	1129	-19	1.09%	CCCTCC-------------------CG	520	-19	0.53%

P108-P115-YW-W9-Syn26crCD326c-KO

Olaparib | INDEL<br>80004 | 44.57%	Ins<br>20.53%	Del<br>24.04%	HDR<br>0.00%
**Typical seqs**	**Reads**	**Type**	**pct.**
CCCTCCCGCGCCCCTCT|TCT**CGG**CGCG	44344	WT	55.43%
CCCTCCCGCGCCCCTCTtTCTCGGCGCG	13794	+1 t	17.24%
CCCTCCCGCGCCCC---TCTCGGCGCG	3731	-3	4.66%
CCCTCCCGCGCCCCTC-TCTCGGCGCG	3662	-1	4.58%
CCCTCC---------------------	2092	-21	2.61%
CCCTCCCGCGCCC-----CTCGGCGCG	1111	-5	1.39%
CCCTCCCGCGCCCCTCTaTCTCGGCGCG	682	+1 a	0.85%
CCCTCCCGCGCC-----TCTCGGCGCG	623	-5	0.78%
CCCTCC-----------------CGCG	527	-17	0.66%
CCCTCCCGC------------------	499	-24	0.62%

SCR7 | INDEL<br>95372 | 48.38%	Ins<br>18.13%	Del<br>30.24%	HDR<br>0.01%
**Typical seqs**	**Reads**	**Type**	**pct.**
CCCTCCCGCGCCCCTCT|TCT**CGG**CGCG	49233	WT	51.62%
CCCTCCCGCGCCCCTCTtTCTCGGCGCG	14867	+1 t	15.59%
CCCTCCCGCGCCCC---TCTCGGCGCG	6805	-3	7.14%
CCCTCCCGCGCCCCTC-TCTCGGCGCG	4039	-1	4.23%
CCCTCCCGCGCCC-----CTCGGCGCG	2562	-5	2.69%
CCCTCC---------------------	3123	-21	3.27%
CCCTCC-------------------CG	783	-19	0.82%
CCC--------------TCTCGGCGCG	746	-14	0.78%
CCCTCC-----------------CGCG	604	-17	0.63%
CCCTCC---------------------	3123	-23	3.27%

VE822 | INDEL<br>117188 | 50.61%	Ins<br>20.57%	Del<br>30.01%	HDR<br>0.03%
**Typical seqs**	**Reads**	**Type**	**pct.**
CCCTCCCGCGCCCCTCT|TCT**CGG**CGCG	57884	WT	49.39%
CCCTCCCGCGCCCCTCTtTCTCGGCGCG	21537	+1 t	18.38%
CCCTCCCGCGCCCC---TCTCGGCGCG	6542	-3	5.58%
CCCTCCCGCGCCCCTC-TCTCGGCGCG	6318	-1	5.39%
CCCTCC---------------------	4116	-21	3.51%
CCCTCCCGCGCCC-----CTCGGCGCG	1822	-5	1.55%
CCCTCC---------------------	4116	-23	3.51%
CCCTCC-----------------CGCG	1044	-17	0.89%
CCC--------------TCTCGGCGCG	1021	-14	0.87%
CCCTCC-------------------CG	824	-19	0.70%

Tri A | INDEL<br>88098 | 51.71%	Ins<br>22.03%	Del<br>29.66%	HDR<br>0.01%
**Typical seqs**	**Reads**	**Type**	**pct.**
CCCTCCCGCGCCCCTCT|TCT**CGG**CGCG	42545	WT	48.29%
CCCTCCCGCGCCCCTCTtTCTCGGCGCG	16915	+1 t	19.20%
CCCTCCCGCGCCCC---TCTCGGCGCG	4999	-3	5.67%
CCCTCCCGCGCCCCTC-TCTCGGCGCG	4437	-1	5.04%
CCCTCCCGCGCCC-----CTCGGCGCG	2444	-5	2.77%
CCCTCC---------------------	2546	-21	2.89%
CCCTCCCGC------------------	624	-24	0.71%
CCCTCC-----------------CGCG	554	-17	0.63%
CCCTCC---------------------	2546	-23	2.89%
CCCTCC-------------------CG	488	-19	0.55%

P108-P115-YW-W9-Syn26crCD326c-AAV6KI

Ctrl | INDEL 149455 | 60.71%	Ins 21.78%	Del 18.36%	HDR 20.56%	AZD7762 | INDEL 156311 | 67.00%	Ins 27.34%	Del 19.61%	HDR 20.05%	AZD7762+VE822 | INDEL 73803 | 66.40%	Ins 28.30%	Del 19.86%	HDR 18.24%
**Typical seqs**	**Reads**	**Type**	**pct.**	**Typical seqs**	**Reads**	**Type**	**pct.**	**Typical seqs**	**Reads**	**Type**	**pct.**
CCCTCCCGCGCCCCTCT|TCT**CGG**CGCG	58726	WT	39.29%	CCCTCCCGCGCCCCTCT|TCT**CGG**CGCG	51586	WT	33.00%	CCCTCCCGCGCCCCTCT|TCT**CGG**CGCG	24800	WT	33.60%
CCCTCCCGCACGCGTAGTTTAAACCTCGGCGCG	29555	+6 HDR	19.78%	CCCTCCCGCGCCCCTCTtTCTCGGCGCG	34828	+1 t	22.28%	CCCTCCCGCGCCCCTCTtTCTCGGCGCG	17918	+1 t	24.28%
CCCTCCCGCGCCCCTCTtTCTCGGCGCG	26695	+1 t	17.86%	CCCTCCCGCACGCGTAGTTTAAACCTCGGCGCG	30179	+6 HDR	19.31%	CCCTCCCGCACGCGTAGTTTAAACCTCGGCGCG	13092	+6 HDR	17.74%
CCCTCCCGCGCCCCTC-TCTCGGCGCG	6613	-1	4.42%	CCCTCCCGCGCCCCTC-TCTCGGCGCG	8243	-1	5.27%	CCCTCCCGCGCCCCTC-TCTCGGCGCG	6014	-1	8.15%
CCCTCCCGCGCCCC---TCTCGGCGCG	4408	-3	2.95%	CCCTCCCGCGCCCC---TCTCGGCGCG	6038	-3	3.86%	CCCTCCCGCGCCCC---TCTCGGCGCG	2251	-3	3.05%
CCCTCCCGCGCCC-----CTCGGCGCG	1728	-5	1.16%	CCCTCCCGCGCCC-----CTCGGCGCG	1849	-5	1.18%	CCCTCC----------------------	1058	-21	1.43%
CCCTCCCGCGCCCCT--TCTCGGCGCG	1005	-2	0.67%	CCCTCC---------------------	1501	-21	0.96%	CC----------------CTCGGCGCG	532	-16	0.72%
CCCTCC---------------------	1635	-21	1.09%	CCCTCCCGCGCCCCT--TCTCGGCGCG	724	-2	0.46%	CCCTCCCGCGC----------------	477	-18	0.65%
CCC--------------TCTCGGCGCG	719	-14	0.48%	CCCTCCC---------------GCGCG	712	-15	0.46%	CCCTCCCGCGCCCCTCTaTCTCGGCGCG	434	+1 a	0.59%
CCCTCC---------------------	1635	-23	1.09%	CCCTCCCGCGCC----TTCTCGGCGCG	702	-4	0.45%	CCCTCCCGCGCCCCT-TTCTCGGCGCG	396	-1	0.54%

AZT | INDEL 123663 | 59.65%	Ins 20.18%	Del 19.86%	HDR 19.61%	CsH | INDEL 74500 | 57.72%	Ins 21.34%	Del 16.15%	HDR 20.23%	B02 | INDEL 144282 | 56.15%	Ins 22.07%	Del 16.76%	HDR 17.32%
**Typical seqs**	**Reads**	**Type**	**pct.**	**Typical seqs**	**Reads**	**Type**	**pct.**	**Typical seqs**	**Reads**	**Type**	**pct.**
CCCTCCCGCGCCCCTCT|TCT**CGG**CGCG	49903	WT	40.35%	CCCTCCCGCGCCCCTCT|TCT**CGG**CGCG	31497	WT	42.28%	CCCTCCCGCGCCCCTCT|TCT**CGG**CGCG	63262	WT	43.85%
CCCTCCCGCACGCGTAGTTTAAACCTCGGCGCG	23367	+6 HDR	18.90%	CCCTCCCGCACGCGTAGTTTAAACCTCGGCGCG	14617	+6 HDR	19.62%	CCCTCCCGCGCCCCTCTtTCTCGGCGCG	26168	+1 t	18.14%
CCCTCCCGCGCCCCTCTtTCTCGGCGCG	20860	+1 t	16.87%	CCCTCCCGCGCCCCTCTtTCTCGGCGCG	13707	+1 t	18.40%	CCCTCCCGCACGCGTAGTTTAAACCTCGGCGCG	24016	+6 HDR	16.65%
CCCTCCCGCGCCCC---TCTCGGCGCG	5549	-3	4.49%	CCCTCCCGCGCCCCTC-TCTCGGCGCG	3148	-1	4.23%	CCCTCCCGCGCCCCTC-TCTCGGCGCG	8082	-1	5.60%
CCCTCCCGCGCCCCTC-TCTCGGCGCG	5230	-1	4.23%	CCCTCCCGCGCCCC---TCTCGGCGCG	2120	-3	2.85%	CCCTCCCGCGCCCC---TCTCGGCGCG	3447	-3	2.39%
CCCTCCCGCGCCC-----CTCGGCGCG	1201	-5	0.97%	CCCTCCCGCGCCC-----CTCGGCGCG	594	-5	0.80%	CCCTCCCGCGCCC-----CTCGGCGCG	1219	-5	0.84%
CCC--------------TCTCGGCGCG	1198	-14	0.97%	CCCTCCCGCGCCCCT--TCTCGGCGCG	526	-2	0.71%	CCC--------------TCTCGGCGCG	928	-14	0.64%
CCCTCC---------------------	967	-21	0.78%	CCCTCCCGCGCCCCTCTgTCTCGGCGCG	368	+1 g	0.49%	CCCTCCCGCGCCCCT--TCTCGGCGCG	866	-2	0.60%
CCCTCC-----------------CGCG	698	-17	0.56%	CCCTCCCGCGC------TCTCGGCGCG	286	-6	0.38%	CCCTCCCGCGCCCCT-TTCTCGGCGCG	854	-1	0.59%
CCCTCC-------------------CG	481	-19	0.39%	CCCTCC---------------------	352	-21	0.47%	CCCTCC---------------------	1113	-21	0.77%

P108-P115-YW-W9-Syn26crCD326c-AAV6KI

DOPA | INDEL	Ins	Del	HDR
187616 | 58.58%	21.56%	19.75%	17.28%
**Typical seqs**	**Reads**	**Type**	**pct.**
CCCTCCCGCGCCCCTCT|TCT**CGG**CGCG	77705	WT	41.42%
CCCTCCCGCGCCCCTCTtTCTCGGCGCG	33719	+1 t	17.97%
CCCTCCCGCACGCGTAGTTTAAACCTCGGCGCG	31238	+6 HDR	16.65%
CCCTCCCGCGCCCCTC-TCTCGGCGCG	7549	-1	4.02%
CCCTCCCGCGCCCC---TCTCGGCGCG	6611	-3	3.52%
CCCTCCCGCGCCC-----CTCGGCGCG	2149	-5	1.15%
CCCTCC---------------------	2604	-21	1.39%
CCCTCCCGCGCCCCT-TTCTCGGCGCG	1153	-1	0.61%
CCCTCCCGCGCCCCT------------	1753	-14	0.93%
CCCTCC-------------------CG	1114	-19	0.59%

M3814 | INDEL	Ins	Del	HDR
77385 | 71.47%	9.76%	13.02%	48.69%
**Typical seqs**	**Reads**	**Type**	**pct.**
CCCTCCCGCGCCCCTCT|TCT**CGG**CGCG	22080	WT	28.53%
CCCTCCCGCACGCGTAGTTTAAACCTCGGCGCG	36467	+6 HDR	47.12%
CCCTCCCGCGCCCCTCTtTCTCGGCGCG	5000	+1 t	6.46%
CCCTCCCGCGCCCC---TCTCGGCGCG	2404	-3	3.11%
CCCTCCCGCGCCCCTC-TCTCGGCGCG	1849	-1	2.39%
CCCTCC---------------------	1762	-23	2.28%
CCCTCC-----------------CGCG	790	-17	1.02%
CCCTCCCG------------CGGCGCG	646	-12	0.83%
CCCTCCCGCGCGCGTAGTTTAAACCTCGGCGCG	499	+6	0.64%
CCCTCCCGCGCCCCTCT---------G	434	-9	0.56%

Mirin | INDEL	Ins	Del	HDR
59701 | 58.18%	22.67%	17.27%	18.23%
**Typical seqs**	**Reads**	**Type**	**pct.**
CCCTCCCGCGCCCCTCT|TCT**CGG**CGCG	24967	WT	41.82%
CCCTCCCGCGCCCCTCTtTCTCGGCGCG	11745	+1 t	19.67%
CCCTCCCGCACGCGTAGTTTAAACCTCGGCGCG	10266	+6 HDR	17.20%
CCCTCCCGCGCCCCTC-TCTCGGCGCG	2907	-1	4.87%
CCCTCC---------------------	1727	-23	2.89%
CCCTCCCGCGCCCC---TCTCGGCGCG	911	-3	1.53%
CCCTCC---------------------	1727	-21	2.89%
---------------------------	449	-30	0.75%
CCCTCCCGCGCCCCT-TTCTCGGCGCG	337	-1	0.56%
CCCTCCCGCGCCCCTCTCTTCTCGGCGCG	293	+2	0.49%

Nu7026+Tri A | INDEL	Ins	Del	HDR
75172 | 76.73%	13.00%	10.80%	52.92%
**Typical seqs**	**Reads**	**Type**	**pct.**
CCCTCCCGCGCCCCTCT|TCT**CGG**CGCG	17492	WT	23.27%
CCCTCCCGCACGCGTAGTTTAAACCTCGGCGCG	38481	+6 HDR	51.19%
CCCTCCCGCGCCCCTCTtTCTCGGCGCG	7115	+1 t	9.46%
CCCTCCCGCGCCCCTC-TCTCGGCGCG	1903	-1	2.53%
CCCTCCCGCGCCCC---TCTCGGCGCG	1074	-3	1.43%
CCCTCCCGCGCCCCT-TTCTCGGCGCG	674	-1	0.90%
CCCTCCCGC------------------	399	-24	0.53%
CCCTCCCGCGCCCCT--TCTCGGCGCG	359	-2	0.48%
CCCTCCCGCGCCCCTCTACTCTCGGCGCG	340	+2	0.45%
CCCTCC-----------------CGCG	337	-17	0.45%

Nu7026 | INDEL	Ins	Del	HDR
95449 | 59.73%	14.39%	20.76%	24.57%
**Typical seqs**	**Reads**	**Type**	**pct.**
CCCTCCCGCGCCCCTCT|TCT**CGG**CGCG	38439	WT	40.27%
CCCTCCCGCACGCGTAGTTTAAACCTCGGCGCG	22625	+6 HDR	23.70%
CCCTCCCGCGCCCCTCTtTCTCGGCGCG	11909	+1 t	12.48%
CCCTCCCGCGCCCC---TCTCGGCGCG	3728	-3	3.91%
CCCTCCCGCGCCCCTC-TCTCGGCGCG	3495	-1	3.66%
CCC--------------TCTCGGCGCG	1307	-14	1.37%
CCCTCC---------------------	1655	-21	1.73%
CCCTCCC---------------GCGCG	854	-15	0.89%
CCCTCC---------------------	1655	-23	1.73%
CCCTCCCGCGCCC-----CTCGGCGCG	650	-5	0.68%

Nu7441+SCR7 | INDEL	Ins	Del	HDR
164617 | 64.95%	15.58%	16.85%	32.52%
**Typical seqs**	**Reads**	**Type**	**pct.**
CCCTCCCGCGCCCCTCT|TCT**CGG**CGCG	57700	WT	35.05%
CCCTCCCGCACGCGTAGTTTAAACCTCGGCGCG	51560	+6 HDR	31.32%
CCCTCCCGCGCCCCTCTtTCTCGGCGCG	19647	+1 t	11.93%
CCCTCCCGCGCCCC---TCTCGGCGCG	4693	-3	2.85%
CCCTCCCGCGCCCCTC-TCTCGGCGCG	3463	-1	2.10%
CCCTCC---------------------	3178	-21	1.93%
CCCTCCCGCGCCC-----CTCGGCGCG	1998	-5	1.21%
CCC--------------TCTCGGCGCG	1743	-14	1.06%
CCCTCCC---------------GCGCG	1026	-15	0.62%
CCCTCC-------------------CG	936	-19	0.57%

P108-P115-YW-W9-Syn26crCD326c-AAV6KI

Olaparib | INDEL	Ins	Del	HDR
112379 | 54.24%	**20.19%**	**18.27%**	**15.78%**
**Typical seqs**	**Reads**	**Type**	**pct.**
CCCTCCCGCGCCCCTCT|TCT**CGG**CGCG	51430	WT	45.76%
CCCTCCCGCGCCCCTCTtTCTCGGCGCG	18968	+1 t	16.88%
CCCTCCCGCACGCGTAGTTTAAACCTCGGC GCG	17068	+6 HDR	15.19%
CCCTCCCGCGCCCCTC-TCTCGGCGCG	5426	-1	4.83%
CCCTCCCGCGCCCC---TCTCGGCGCG	2265	-3	2.02%
CCCTCC---------------------	1968	-21	1.75%
CCCTCCCGCGCCC-----CTCGGCGCG	930	-5	0.83%
CCCTCC-----------------CGCG	718	-17	0.64%
CCCTCCCGCGCCCCT--TCTCGGCGCG	571	-2	0.51%
CCCTCCCGCGCC-----TCTCGGCGCG	503	-5	0.45%

NU7441 | INDEL	Ins	Del	HDR
141564 | 66.67%	**16.06%**	**15.73%**	**34.88%**
**Typical seqs**	**Reads**	**Type**	**pct.**
CCCTCCCGCGCCCCTCT|TCT**CGG**CGCG	47187	WT	33.33%
CCCTCCCGCACGCGTAGTTTAAACCTCGGC GCG	47551	+6 HDR	33.59%
CCCTCCCGCGCCCCTCTtTCTCGGCGCG	17851	+1 t	12.61%
CCCTCCCGCGCCCCTC-TCTCGGCGCG	4426	-1	3.13%
CCCTCCCGCGCCCC---TCTCGGCGCG	3912	-3	2.76%
CCCTCCCGCGCCC-----CTCGGCGCG	2018	-5	1.43%
CCCTCC---------------------	2485	-21	1.76%
CCCTCC---------------------	2485	-23	1.76%
CCC--------------TCTCGGCGCG	754	-14	0.53%
CCCTCCCGCGCCCCT----------CC	719	-10	0.51%

VE822 | INDEL	Ins	Del	HDR
71247 | 58.56%	**22.26%**	**15.90%**	**20.40%**
**Typical seqs**	**Reads**	**Type**	**pct.**
CCCTCCCGCGCCCCTCT|TCT**CGG**CGCG	29525	WT	41.44%
CCCTCCCGCACGCGTAGTTTAAACCTCGGC GCG	14085	+6 HDR	19.77%
CCCTCCCGCGCCCCTCTtTCTCGGCGCG	13540	+1 t	19.00%
CCCTCCCGCGCCCCTC-TCTCGGCGCG	3387	-1	4.75%
CCCTCCCGCGCCCC---TCTCGGCGCG	1517	-3	2.13%
CCCTCC---------------------	1042	-21	1.46%
CCCTCCCGCGCCCCT--TCTCGGCGCG	585	-2	0.82%
CCC--------------TCTCGGCGCG	431	-14	0.60%
CCCTCCCGCGCCC-----CTCGGCGCG	408	-5	0.57%
CCCTCCCGCGCCCCT-TTCTCGGCGCG	388	-1	0.54%

Tri A | INDEL	Ins	Del	HDR
49188 | 68.59%	**19.34%**	**11.05%**	**38.20%**
**Typical seqs**	**Reads**	**Type**	**pct.**
CCCTCCCGCGCCCCTCT|TCT**CGG**CGCG	15448	WT	31.41%
CCCTCCCGCACGCGTAGTTTAAACCTCGGC GCG	18165	+6 HDR	36.93%
CCCTCCCGCGCCCCTCTtTCTCGGCGCG	7954	+1 t	16.17%
CCCTCCCGCGCCCCTC-TCTCGGCGCG	2050	-1	4.17%
CCCTCCCGCGCCCC---TCTCGGCGCG	714	-3	1.45%
CCCTCC---------------------	599	-23	1.22%
CCCTCCCGCGCCC-----CTCGGCGCG	345	-5	0.70%
CCCTCCCGCGCCCCTCTaTCTCGGCGCG	220	+1 a	0.45%
CCCTCC---------------------	599	-21	1.22%
CCCTCCCGCGC----------------	129	-16	0.26%

YW-W9-Syn29crCD326f-KO-48h

Ctrl | INDEL	Ins	Del	HDR
150522 | 62.53%	39.98%	22.54%	0.00%
**Typical seqs**	**Reads**	**Type**	**pct.**
TGCGCGCGCGCCGAGAA|GAG**GGG**CGCG	56406	WT	37.47%
TGCGCGCGCGCCGAGAAaGAGGGGCGCG	55169	+1 a	36.65%
TGCGCGCGCGCC-----GAGGGGCGCG	5576	-5	3.70%
TGCGCGCGCGCCGA---GAGGGGCGCG	4096	-3	2.72%
TGCGCGCGCGCCGAGA-GAGGGGCGCG	3028	-1	2.01%
T---------------------GCGCG	1888	-21	1.25%
TGCGCGCGCGCCGA-------------	1386	-14	0.92%
T-----------------------GCG	916	-23	0.61%
TGCGCGCGCGCCGAGAA-AGGGGCGCG	732	-1	0.49%
TGCGCGCGCGCCGAGAAaGGGGGGCGCG	792	+1 a	0.53%

AZD7762 | INDEL	Ins	Del	HDR
73385 | 70.11%	36.50%	33.61%	0.00%
**Typical seqs**	**Reads**	**Type**	**pct.**
TGCGCGCGCGCCGAGAA|GAG**GGG**CGCG	21936	WT	29.89%
TGCGCGCGCGCCGAGAAaGAGGGGCGCG	24413	+1 a	33.27%
TGCGCGCGCGCC-----GAGGGGCGCG	5080	-5	6.92%
TGCGCGCGCGCCGA---GAGGGGCGCG	2746	-3	3.74%
TGCGCGCGCGCCGAGA-GAGGGGCGCG	1971	-1	2.69%
T---------------------GCGCG	2087	-21	2.84%
TGCGCGCGCC-----------------	914	-16	1.25%
TGC-------------------GCGCG	667	-19	0.91%
T-----------------------GCG	584	-23	0.80%
TGCGC-----------------GCGCG	553	-17	0.75%

AZD7762+VE822 | INDEL	Ins	Del	HDR
111717 | 67.17%	41.86%	25.27%	0.03%
**Typical seqs**	**Reads**	**Type**	**pct.**
TGCGCGCGCGCCGAGAA|GAG**GGG**CGCG	36680	WT	32.83%
TGCGCGCGCGCCGAGAAaGAGGGGCGCG	42783	+1 a	38.30%
TGCGCGCGCGCCGAGA-GAGGGGCGCG	4076	-1	3.65%
TGCGCGCGCGCCGA---GAGGGGCGCG	3841	-3	3.44%
TGCGCGCGCGCC-----GAGGGGCGCG	2984	-5	2.67%
T---------------------GCGCG	1981	-21	1.77%
TGCGCGCGCGCCGAGAA-AGGGGCGCG	1516	-1	1.36%
TGCGCGCGCGCCGAGA--AGGGGCGCG	1325	-2	1.19%
T-----------------------GCG	1102	-23	0.99%
TGCGC-----------------GCGCG	979	-17	0.88%

AZT | INDEL	Ins	Del	HDR
119190 | 62.16%	37.86%	24.30%	0.00%
**Typical seqs**	**Reads**	**Type**	**pct.**
TGCGCGCGCGCCGAGAA|GAG**GGG**CGCG	45096	WT	37.84%
TGCGCGCGCGCCGAGAAaGAGGGGCGCG	41521	+1 a	34.84%
TGCGCGCGCGCC-----GAGGGGCGCG	5674	-5	4.76%
TGCGCGCGCGCCGAGA-GAGGGGCGCG	2570	-1	2.16%
TGCGCGCGCGCCGA---GAGGGGCGCG	2019	-3	1.69%
T---------------------GCGCG	1898	-21	1.59%
TGC-------------------GCGCG	1062	-19	0.89%
T-----------------------GCG	911	-23	0.76%
TGCGCGCGCGCCGAGAA-AGGGGCGCG	841	-1	0.71%
TGCGCGCGCGCCGAGA--AGGGGCGCG	688	-2	0.58%

B02 | INDEL	Ins	Del	HDR
139640 | 56.68%	32.05%	24.62%	0.00%
**Typical seqs**	**Reads**	**Type**	**pct.**
TGCGCGCGCGCCGAGAA|GAG**GGG**CGCG	60494	WT	43.32%
TGCGCGCGCGCCGAGAAaGAGGGGCGCG	41252	+1 a	29.54%
TGCGCGCGCGCCGAGA-GAGGGGCGCG	5383	-1	3.85%
T---------------------GCGCG	3520	-21	2.52%
T-----------------------GCG	3179	-23	2.28%
TGCGCGCGCGCCGA---GAGGGGCGCG	2869	-3	2.05%
TGCGCGCGCGCC-----GAGGGGCGCG	1793	-5	1.28%
TGC------------------------	1664	-30	1.19%
TGCGCGCGCGCCGAGA--AGGGGCGCG	882	-2	0.63%
T--------------------------	747	-32	0.53%

CsH | INDEL	Ins	Del	HDR
91008 | 60.42%	42.50%	17.88%	0.04%
**Typical seqs**	**Reads**	**Type**	**pct.**
TGCGCGCGCGCCGAGAA|GAG**GGG**CGCG	36024	WT	39.58%
TGCGCGCGCGCCGAGAAaGAGGGGCGCG	35889	+1 a	39.43%
TGCGCGCGCGCC-----GAGGGGCGCG	1961	-5	2.15%
TGCGCGCGCGCCGAGA-GAGGGGCGCG	1742	-1	1.91%
T-----------------------GCG	1153	-23	1.27%
T---------------------GCGCG	1172	-21	1.29%
TGCGCGCGCGCCGAGA----GGGCGCG	1092	-4	1.20%
TGCGCGCGCGCCGA---GAGGGGCGCG	891	-3	0.98%
TGCGCGCGCGCCGA-------------	842	-14	0.93%
TGCGCGCGCGCCGAGAA-AGGGGCGCG	800	-1	0.88%

YW-W9-Syn29crCD326f-KO-48h

DOPA | INDEL<br>163382 | 62.96%	Ins<br>40.59%	Del<br>22.37%	HDR<br>0.00%
**Typical seqs**	**Reads**	**Type**	**pct.**
TGCGCGCGCGCCGAGAA|GAG**GGG**CGCG	60511	WT	37.04%
TGCGCGCGCGCCGAGAAaGAGGGGCGCG	60720	+1 a	37.16%
TGCGCGCGCGCC-----GAGGGGCGCG	6548	-5	4.01%
TGCGCGCGCGCCGA---GAGGGGCGCG	3245	-3	1.99%
TGCGCGCGCGCCGAGA-GAGGGGCGCG	2998	-1	1.83%
T---------------------GCGCG	2091	-21	1.28%
TGCGCGCGCGCCGAGA--AGGGGCGCG	1284	-2	0.79%
T-----------------------GCG	1019	-23	0.62%
TGCGCGCGCGCCGAGAA-AGGGGCGCG	890	-1	0.54%
TGC-------------------GCGCG	826	-19	0.51%

M3814 | INDEL<br>202046 | 58.86%	Ins<br>24.80%	Del<br>34.04%	HDR<br>0.02%
**Typical seqs**	**Reads**	**Type**	**pct.**
TGCGCGCGCGCCGAGAA|GAG**GGG**CGCG	83128	WT	41.14%
TGCGCGCGCGCCGAGAAaGAGGGGCGCG	44014	+1 a	21.78%
TGCGCGCGCGCC-----GAGGGGCGCG	9775	-5	4.84%
TGCGCGCGCGCCGA---GAGGGGCGCG	8574	-3	4.24%
T---------------------GCGCG	5989	-21	2.96%
TGCGCGCGCGCCGAGA-GAGGGGCGCG	5204	-1	2.58%
TGC-------------------GCGCG	3146	-19	1.56%
TGCGCGC---------------GCGCG	2691	-15	1.33%
TGCGCGCGC-------------GCGCG	1899	-13	0.94%
TGCGC-----------------GCGCG	1890	-17	0.94%

Mirin | INDEL<br>74679 | 64.00%	Ins<br>43.14%	Del<br>20.82%	HDR<br>0.04%
**Typical seqs**	**Reads**	**Type**	**pct.**
TGCGCGCGCGCCGAGAA|GAG**GGG**CGCG	26881	WT	36.00%
TGCGCGCGCGCCGAGAAaGAGGGGCGCG	29356	+1 a	39.31%
TGCGCGCGCGCCGAGA-GAGGGGCGCG	2419	-1	3.24%
TGCGCGCGCGCC-----GAGGGGCGCG	1753	-5	2.35%
TGCGCGCGCGCCGA---GAGGGGCGCG	937	-3	1.25%
T---------------------GCGCG	800	-21	1.07%
T-----------------------GCG	802	-23	1.07%
TGCGCGCGCGCCGAGAA-AGGGGCGCG	568	-1	0.76%
TGCGCGCGCGCCGA-------------	711	-14	0.95%
TGC-------------------GCGCG	478	-19	0.64%

Nu7026+Tri A | INDEL<br>150226 | 58.90%	Ins<br>38.44%	Del<br>20.44%	HDR<br>0.02%
**Typical seqs**	**Reads**	**Type**	**pct.**
TGCGCGCGCGCCGAGAA|GAG**GGG**CGCG	61742	WT	41.10%
TGCGCGCGCGCCGAGAAaGAGGGGCGCG	51246	+1 a	34.11%
TGCGCGCGCGCCGAGA-GAGGGGCGCG	3279	-1	2.18%
TGCGCGCGCGCCGA---GAGGGGCGCG	2757	-3	1.84%
TGCGCGCGCGCC-----GAGGGGCGCG	2671	-5	1.78%
T--------------------------	2700	-30	1.80%
T-----------------------GCG	2176	-23	1.45%
TGCG---------------GGAGGCGCG	1920	-14	1.28%
TGCGCGCG--------AGAGGGGCGCG	1623	-8	1.08%
TGCGCGCGCGCCGAG--GAGGGGCGCG	1576	-2	1.05%

Nu7026 | INDEL<br>190613 | 59.76%	Ins<br>34.56%	Del<br>25.16%	HDR<br>0.03%
**Typical seqs**	**Reads**	**Type**	**pct.**
TGCGCGCGCGCCGAGAA|GAG**GGG**CGCG	76709	WT	40.24%
TGCGCGCGCGCCGAGAAaGAGGGGCGCG	59379	+1 a	31.15%
TGCGCGCGCGCC-----GAGGGGCGCG	8374	-5	4.39%
TGCGCGCGCGCCGA---GAGGGGCGCG	4113	-3	2.16%
T---------------------GCGCG	4087	-21	2.14%
TGCGCGCGCGCCGAGA-GAGGGGCGCG	2832	-1	1.49%
T-----------------------GCG	1930	-23	1.01%
TGC-------------------GCGCG	1886	-19	0.99%
TGCGC-----------------GCGCG	1478	-17	0.78%
TGCGCGCGCGCC---------------	1213	-16	0.64%

Nu7441 | INDEL<br>154931 | 62.70%	Ins<br>37.28%	Del<br>25.42%	HDR<br>0.00%
**Typical seqs**	**Reads**	**Type**	**pct.**
TGCGCGCGCGCCGAGAA|GAG**GGG**CGCG	57785	WT	37.30%
TGCGCGCGCGCCGAGAAaGAGGGGCGCG	52427	+1 a	33.84%
TGCGCGCGCGCC-----GAGGGGCGCG	8519	-5	5.50%
TGCGCGCGCGCCGA---GAGGGGCGCG	3438	-3	2.22%
T---------------------GCGCG	3360	-21	2.17%
TGCGCGCGCGCCGAGA-GAGGGGCGCG	3151	-1	2.03%
TGC-------------------GCGCG	1222	-19	0.79%
TGCGCGCGCGCC---------------	1398	-16	0.90%
TGCGC-----------------GCGCG	1123	-17	0.72%
T-----------------------GCG	1018	-23	0.66%

YW-W9-Syn29crCD326f-KO-48h

Olaparib | INDEL	Ins	Del	HDR
98103 | 60.43%	**41.90%**	**18.53%**	**0.00%**
**Typical seqs**	**Reads**	**Type**	**pct.**
TGCGCGCGCGCCGAGAA|GAG**GGG**CGCG	38819	WT	39.57%
TGCGCGCGCGCCGAGAAaGAGGGGCGCG	37868	+1 a	38.60%
TGCGCGCGCGCCGAGA-GAGGGGCGCG	2722	-1	2.77%
TGCGCGCGCGCC-----GAGGGGCGCG	1934	-5	1.97%
T---------------------GCGCG	1604	-21	1.64%
TGCGCGCGCGCCGA---GAGGGGCGCG	1169	-3	1.19%
TGCGCGCGCGCCGAGA--AGGGGCGCG	774	-2	0.79%
TGCGCGCGCGCCGAGAA-AGGGGCGCG	759	-1	0.77%
T-----------------------GCG	731	-23	0.75%
TGC-------------------GCGCG	566	-19	0.58%

SCR7 | INDEL	Ins	Del	HDR
129090 | 64.43%	**38.76%**	**25.67%**	**0.00%**
**Typical seqs**	**Reads**	**Type**	**pct.**
TGCGCGCGCGCCGAGAA|GAG**GGG**CGCG	45914	WT	35.57%
TGCGCGCGCGCCGAGAAaGAGGGGCGCG	46358	+1 a	35.91%
TGCGCGCGCGCC-----GAGGGGCGCG	5994	-5	4.64%
TGCGCGCGCGCCGAGA-GAGGGGCGCG	3498	-1	2.71%
T---------------------GCGCG	2687	-21	2.08%
TGCGCGCGCGCCGA---GAGGGGCGCG	2097	-3	1.62%
TGCGCGCGCGCCGAGAA-AGGGGCGCG	1526	-1	1.18%
TGC-------------------GCGCG	1057	-19	0.82%
T-----------------------GCG	840	-23	0.65%
TGCGC-----------------GCGCG	783	-17	0.61%

Tri A | INDEL	Ins	Del	HDR
58983 | 65.98%	**42.73%**	**23.19%**	**0.05%**
**Typical seqs**	**Reads**	**Type**	**pct.**
TGCGCGCGCGCCGAGAA|GAG**GGG**CGCG	20068	WT	34.02%
TGCGCGCGCGCCGAGAAaGAGGGGCGCG	22983	+1 a	38.97%
TGCGCGCGCGCC-----GAGGGGCGCG	1839	-5	3.12%
TGCGCGCGCGCCGAGA-GAGGGGCGCG	1101	-1	1.87%
T---------------------GCGCG	1106	-21	1.88%
T-----------------------GCG	715	-23	1.21%
TGCGCGCGCGCCGAGAA-AGGGGCGCG	652	-1	1.11%
TGCGCGCGCGCCGAGA--AGGGGCGCG	565	-2	0.96%
TGCG------------GGAGGGGCGCG	532	-12	0.90%
TGCGCGCGCGCCGA---GAGGGGCGCG	400	-3	0.68%

VE822 | INDEL	Ins	Del	HDR
103715 | 63.08%	**42.93%**	**20.12%**	**0.02%**
**Typical seqs**	**Reads**	**Type**	**pct.**
TGCGCGCGCGCCGAGAA|GAG**GGG**CGCG	38296	WT	36.92%
TGCGCGCGCGCCGAGAAaGAGGGGCGCG	40719	+1 a	39.26%
TGCGCGCGCGCCGAGA-GAGGGGCGCG	2351	-1	2.27%
TGCGCGCGCGCC-----GAGGGGCGCG	2142	-5	2.07%
T---------------------GCGCG	2031	-21	1.96%
TGCGCGCGCGCCGA---GAGGGGCGCG	1562	-3	1.51%
T-----------------------GCG	933	-23	0.90%
TGCGC-----------------GCGCG	889	-17	0.86%
TGC-------------------GCGCG	713	-19	0.69%
TGCGCGCGCGCCGAGAA-AGGGGCGCG	534	-1	0.51%

YW-W9-Syn29crCD326f-AAV6KI-48h

Ctrl | INDEL<br>148983 | 72.79%	Ins<br>**43.80%**	Del<br>**13.30%**	HDR<br>**15.69%**
**Typical seqs**	**Reads**	**Type**	**pct.**
TGCGCGCGCGCCGAGAA|GAG**GGG**CGCG	40535	WT	27.21%
TGCGCGCGCGCCGAGAAaGAGGGGCGCG	58660	+1 a	39.37%
TGCGCGCGCGCCGAGGTTTAAACTACGCGT GCG	22430	+6 HDR	15.06%
TGCGCGCGCGCC-----GAGGGGCGCG	2999	-5	2.01%
TGCGCGCGCGCCGAGA-GAGGGGCGCG	2936	-1	1.97%
TGCGCGCGCGCCGA---GAGGGGCGCG	1400	-3	0.94%
TGC-------------------GCGCG	709	-19	0.48%
T---------------------GCGCG	653	-21	0.44%
TGCGCGCGCGCCGAGAAaGGGGGGCGCG	689	+1 a	0.46%
TGCGCGCGCGCCGA-------------	554	-14	0.37%

AZD7762 | INDEL<br>159280 | 76.14%	Ins<br>**46.73%**	Del<br>**12.35%**	HDR<br>**17.06%**
**Typical seqs**	**Reads**	**Type**	**pct.**
TGCGCGCGCGCCGAGAA|GAG**GGG**CGCG	38006	WT	23.86%
TGCGCGCGCGCCGAGAAaGAGGGGCGCG	66957	+1 a	42.04%
TGCGCGCGCGCCGAGGTTTAAACTACGCGT GCG	26053	+6 HDR	16.36%
TGCGCGCGCGCCGAGA-GAGGGGCGCG	2417	-1	1.52%
TGCGCGCGCGCCGAGA--AGGGGCGCG	2112	-2	1.33%
TGCGCGCGCGCCGAGAA-----GCGCG	2056	-5	1.29%
TGCGCGCGCGCC---------------	1440	-16	0.90%
TGCGCGCGCGCCGAGAA-AGGGGCGCG	1222	-1	0.77%
TGCGCGCGCGCC-----GAGGGGCGCG	1158	-5	0.73%
TGC-------------------GCGCG	1111	-19	0.70%

AZD7762+VE822 | INDEL<br>128567 | 72.65%	Ins<br>**48.61%**	Del<br>**8.96%**	HDR<br>**15.08%**
**Typical seqs**	**Reads**	**Type**	**pct.**
TGCGCGCGCGCCGAGAA|GAG**GGG**CGCG	35168	WT	27.35%
TGCGCGCGCGCCGAGAAaGAGGGGCGCG	55607	+1 a	43.25%
TGCGCGCGCGCCGAGGTTTAAACTACGCGT GCG	18602	+6 HDR	14.47%
TGCGCGCGCGCCGAGA-GAGGGGCGCG	3392	-1	2.64%
TGCGCGCGCGCCGAGAA-AGGGGCGCG	1600	-1	1.24%
TGCGCGCGCGCCGAGA--AGGGGCGCG	982	-2	0.76%
TGCGCGCGCGCCGAGAAAAGAGGGGCGCG	639	+2	0.50%
TGCGCGCGCGCCGA----AGGGGCGCG	631	-4	0.49%
TGCGCGCGCGCCGAGAATTAGAGGGGCGCG	620	+3	0.48%
T--------------------------	558	-32	0.43%

AZT | INDEL<br>124344 | 73.39%	Ins<br>**41.93%**	Del<br>**14.36%**	HDR<br>**17.11%**
**Typical seqs**	**Reads**	**Type**	**pct.**
TGCGCGCGCGCCGAGAA|GAG**GGG**CGCG	33087	WT	26.61%
TGCGCGCGCGCCGAGAAaGAGGGGCGCG	46309	+1 a	37.24%
TGCGCGCGCGCCGAGGTTTAAACTACGCGT GCG	20415	+6 HDR	16.42%
TGCGCGCGCGCCGAGA-GAGGGGCGCG	3498	-1	2.81%
TGCGCGCGCGCC-----GAGGGGCGCG	2609	-5	2.10%
TGCGCGCGCGCCGA---GAGGGGCGCG	1624	-3	1.31%
T-----------------------GCG	997	-23	0.80%
TGCGCGCGCGCCGAGA--AGGGGCGCG	584	-2	0.47%
TGCGCGCGCGCCGAGAA-AGGGGCGCG	500	-1	0.40%
TGCGCGCGCGCC---------------	631	-16	0.51%

B02 | INDEL<br>140374 | 69.47%	Ins<br>**44.54%**	Del<br>**9.97%**	HDR<br>**14.96%**
**Typical seqs**	**Reads**	**Type**	**pct.**
TGCGCGCGCGCCGAGAA|GAG**GGG**CGCG	42853	WT	30.53%
TGCGCGCGCGCCGAGAAaGAGGGGCGCG	55823	+1 a	39.77%
TGCGCGCGCGCCGAGGTTTAAACTACGCGT GCG	19864	+6 HDR	14.15%
TGCGCGCGCGCCGAGA-GAGGGGCGCG	3087	-1	2.20%
TGCGCGCGCGCCGAGAA-AGGGGCGCG	1421	-1	1.01%
TGCGCGCGCGCCGA--AGAGGGGCGCG	986	-2	0.70%
TGCGCGCGCGCCGA---GAGGGGCGCG	982	-3	0.70%
TGCGC-----------------GCGCG	819	-17	0.58%
TGCGCGCGCGCCGAGAAAAGAGGGGCGCG	779	+2	0.55%
TGCGCGCGCGCCGAGAA----------	530	-12	0.38%

CsH | INDEL<br>104774 | 71.15%	Ins<br>**43.03%**	Del<br>**12.57%**	HDR<br>**15.55%**
**Typical seqs**	**Reads**	**Type**	**pct.**
TGCGCGCGCGCCGAGAA|GAG**GGG**CGCG	30228	WT	28.85%
TGCGCGCGCGCCGAGAAaGAGGGGCGCG	40446	+1 a	38.60%
TGCGCGCGCGCCGAGGTTTAAACTACGCGT GCG	15549	+6 HDR	14.84%
TGCGCGCGCGCCGAGA-GAGGGGCGCG	2194	-1	2.09%
TGCGCGCGCGCC-----GAGGGGCGCG	1434	-5	1.37%
TGCGCGCGCGCCGA---GAGGGGCGCG	894	-3	0.85%
TGCGCGCGCGCCGA-------------	872	-14	0.83%
T-----------------------GCG	646	-23	0.62%
TGCGCGCGC------------GGCGCG	623	-12	0.59%
TGC------------------------	638	-24	0.61%

YW-W9-Syn29crCD326f-AAV6KI-48h

DOPA | INDEL	Ins	Del	HDR
173292 | 72.84%	**45.87%**	**13.12%**	**13.84%**
**Typical seqs**	**Reads**	**Type**	**pct.**
TGCGCGCGCGCCGAGAA|GAG**GGG**CGCG	47065	WT	27.16%
TGCGCGCGCGCCGAGAAaGAGGGGCGCG	71833	+1 a	41.45%
TGCGCGCGCGCCGAGGTTTAAACTACGCGT<br>GCG	23031	+6 HDR	13.29%
TGCGCGCGCGCCGAGA-GAGGGGCGCG	3487	-1	2.01%
TGCGCGCGCGCCGA---GAGGGGCGCG	2458	-3	1.42%
TGCGCGCGCGCC-----GAGGGGCGCG	1261	-5	0.73%
TGCGCGCGCGCCGA-------------	1553	-14	0.90%
TGC-------------------GCGCG	917	-19	0.53%
TGCGC-----------AGAGGGGCGCG	860	-11	0.50%
TGCGCGC----------GAGGGGCGCG	737	-10	0.43%

M3814 | INDEL	Ins	Del	HDR
156032 | 81.87%	**14.26%**	**14.84%**	**52.77%**
**Typical seqs**	**Reads**	**Type**	**pct.**
TGCGCGCGCGCCGAGAA|GAG**GGG**CGCG	28284	WT	18.13%
TGCGCGCGCGCCGAGGTTTAAACTACGCGT<br>GCG	78644	+6 HDR	50.40%
TGCGCGCGCGCCGAGAAaGAGGGGCGCG	16436	+1 a	10.53%
TGCGCGCGCGCC-----GAGGGGCGCG	3511	-5	2.25%
T---------------------GCGCG	1731	-21	1.11%
TGCGC------------GAGGGGCGCG	1338	-12	0.86%
TGC-------------------GCGCG	1144	-19	0.73%
TGCGCGCG---------GAGGGGCGCG	1121	-9	0.72%
TGCGCGC---------------GCGCG	1093	-15	0.70%
TGCGCGCGCGCCGA---GAGGGGCGCG	1039	-3	0.67%

Mirin | INDEL	Ins	Del	HDR
99027 | 70.12%	**43.59%**	**14.78%**	**11.74%**
**Typical seqs**	**Reads**	**Type**	**pct.**
TGCGCGCGCGCCGAGAA|GAG**GGG**CGCG	29588	WT	29.88%
TGCGCGCGCGCCGAGAAaGAGGGGCGCG	38580	+1 a	38.96%
TGCGCGCGCGCCGAGGTTTAAACTACGCGT<br>GCG	11134	+6 HDR	11.24%
TGCGCGCGCGCCGAGA-GAGGGGCGCG	3273	-1	3.31%
TGCGC-----------------GCGCG	1485	-17	1.50%
T---------------------GCGCG	1215	-21	1.23%
TGCGCGCGCGCCGAGAA-AGGGGCGCG	766	-1	0.77%
TGCGCGCGCGC--------------CG	675	-14	0.68%
T--------------------GGCGCG	508	-20	0.51%
TGCGCGCGCGCC-----GAGGGGCGCG	481	-5	0.49%

Nu7026+Tri A | INDEL	Ins	Del	HDR
117763 | 81.67%	**33.45%**	**8.12%**	**40.10%**
**Typical seqs**	**Reads**	**Type**	**pct.**
TGCGCGCGCGCCGAGAA|GAG**GGG**CGCG	21585	WT	18.33%
TGCGCGCGCGCCGAGGTTTAAACTACGCGT<br>GCG	45242	+6 HDR	38.42%
TGCGCGCGCGCCGAGAAaGAGGGGCGCG	33930	+1 a	28.81%
TGCGCGCGCGCCGAGA-GAGGGGCGCG	3341	-1	2.84%
TGCGCGCGCGCCGA---GAGGGGCGCG	1307	-3	1.11%
TGCGC-----------------GCGCG	608	-17	0.52%
TGCGCGCGCGCC---------------	545	-16	0.46%
TGC----------------GGGGCGCG	501	-16	0.43%
TGCGCGCGCGCCGA-AAGAGGGGCGCG	383	-1	0.33%
TGCGCGCGCGCCGAGAAaGGGGGGCGCG	402	+1 a	0.34%

Nu7026 | INDEL	Ins	Del	HDR
135209 | 74.69%	**38.43%**	**13.94%**	**22.32%**
**Typical seqs**	**Reads**	**Type**	**pct.**
TGCGCGCGCGCCGAGAA|GAG**GGG**CGCG	34215	WT	25.31%
TGCGCGCGCGCCGAGAAaGAGGGGCGCG	44700	+1 a	33.06%
TGCGCGCGCGCCGAGGTTTAAACTACGCGT<br>GCG	28904	+6 HDR	21.38%
TGCGCGCGCGCCGAGA-GAGGGGCGCG	2394	-1	1.77%
T--------------------------	1193	-32	0.88%
TGCGCGCGCGCC-----GAGGGGCGCG	1003	-5	0.74%
TGCGCGCGCGCCGAGAA-AGGGGCGCG	989	-1	0.73%
TGCGCGCGCGCCGAGAACAGAGGGGCGCG	960	+2	0.71%
TGCGCGCGCGCC---------------	964	-16	0.71%
T----------------GAGGGGCGCG	906	-16	0.67%

Nu7441 | INDEL	Ins	Del	HDR
159888 | 78.42%	**31.90%**	**16.00%**	**30.52%**
**Typical seqs**	**Reads**	**Type**	**pct.**
TGCGCGCGCGCCGAGAA|GAG**GGG**CGCG	34508	WT	21.58%
TGCGCGCGCGCCGAGGTTTAAACTACGCGT<br>GCG	46692	+6 HDR	29.20%
TGCGCGCGCGCCGAGAAaGAGGGGCGCG	43413	+1 a	27.15%
T---------------------GCGCG	2792	-21	1.75%
TGCGCGCGCGCC-----GAGGGGCGCG	2217	-5	1.39%
TGCGCGCGCGCCGA---GAGGGGCGCG	2039	-3	1.28%
TGCGCGCGCGCCGAGA-GAGGGGCGCG	1719	-1	1.08%
TGCGCGCGCGCCGA-------------	1310	-14	0.82%
TGCGCGC---------------GCGCG	1064	-15	0.67%
TGCGCGCGCGCCGAGAA-AGGGGCGCG	884	-1	0.55%

YW-W9-Syn29crCD326f-AAV6KI-48h

Olaparib | INDEL	Ins	Del	HDR
121592 | 68.09%	**39.25%**	**17.16%**	**11.68%**
**Typical seqs**	**Reads**	**Type**	**pct.**
TGCGCGCGCGCCGAGAA|GAG**GGG**CGCG	38796	WT	31.91%
TGCGCGCGCGCCGAGAAaGAGGGGCGCG	43786	+1 a	36.01%
TGCGCGCGCGCCGAGGTTTAAACTACGCGT GCG	13696	+6 HDR	11.26%
TGCGCGCGCGCCGAGA-GAGGGGCGCG	3999	-1	3.29%
TGCGCGCGCGCCGA---GAGGGGCGCG	2364	-3	1.94%
TGCGCGCGCGCC-----GAGGGGCGCG	1428	-5	1.17%
T---------------------GCGCG	1215	-21	1.00%
TGCGCGCGCGCCGAGAA-AGGGGCGCG	1035	-1	0.85%
TGC-------------------GCGCG	1003	-19	0.82%
TGCGCGCGCGCCGAGA--AGGGGCGCG	702	-2	0.58%

SCR7 | INDEL	Ins	Del	HDR
148507 | 72.81%	**42.82%**	**14.09%**	**15.91%**
**Typical seqs**	**Reads**	**Type**	**pct.**
TGCGCGCGCGCCGAGAA|GAG**GGG**CGCG	40374	WT	27.19%
TGCGCGCGCGCCGAGAAaGAGGGGCGCG	57368	+1 a	38.63%
TGCGCGCGCGCCGAGGTTTAAACTACGCGT GCG	22621	+6 HDR	15.23%
TGCGCGCGCGCCGA---GAGGGGCGCG	2711	-3	1.83%
TGCGCGCGCGCCGAGA-GAGGGGCGCG	2630	-1	1.77%
TGCGCGCGCGCC-----GAGGGGCGCG	1768	-5	1.19%
TGCGCGCGCGCCGAGAA-AGGGGCGCG	862	-1	0.58%
TGCGCGCGCGCCGAGA--AGGGGCGCG	801	-2	0.54%
TGC--------------GAGGGGCGCG	710	-14	0.48%
T-----------------------GCG	1010	-23	0.68%

Tris A | INDEL	Ins	Del	HDR
96591 | 82.86%	**42.14%**	**8.30%**	**32.42%**
**Typical seqs**	**Reads**	**Type**	**pct.**
TGCGCGCGCGCCGAGAA|GAG**GGG**CGCG	16559	WT	17.14%
TGCGCGCGCGCCGAGAAaGAGGGGCGCG	35855	+1 a	37.12%
TGCGCGCGCGCCGAGGTTTAAACTACGCGT GCG	29935	+6 HDR	30.99%
TGCGCGCGCGCCGAGA-GAGGGGCGCG	1692	-1	1.75%
TGCGC-----------AGAGGGGCGCG	789	-11	0.82%
TGCGC-----------------GCGCG	715	-17	0.74%
T------------------GGGGCGCG	708	-18	0.73%
TGCGCGCGCGCCGAGA----GGGCGCG	680	-4	0.70%
TGCGCGCGCGCCGAGA--AGGGGCGCG	640	-2	0.66%
TGCGCGCGCGCCGA---GAGGGGCGCG	604	-3	0.63%

VE822| INDEL	Ins	Del	HDR
132440 | 71.91%	**45.05%**	**9.84%**	**17.03%**
**Typical seqs**	**Reads**	**Type**	**pct.**
TGCGCGCGCGCCGAGAA|GAG**GGG**CGCG	37196	WT	28.09%
TGCGCGCGCGCCGAGAAaGAGGGGCGCG	53549	+1 a	40.43%
TGCGCGCGCGCCGAGGTTTAAACTACGCGT GCG	21636	+6 HDR	16.34%
TGCGCGCGCGCCGAGA-GAGGGGCGCG	2759	-1	2.08%
TGCGCGCGCGCCGAGAA-AGGGGCGCG	1147	-1	0.87%
TGCGCGCGCGCC-----GAGGGGCGCG	841	-5	0.64%
TGCGCGCGCGCCGAGA--AGGGGCGCG	791	-2	0.60%
T---------------------GCGCG	797	-21	0.60%
TGCGCGCGCGCCGAGAAaGGGGGGCGCG	838	+1 a	0.63%
---------------------GGCGCG	664	-21	0.50%

YW-W9-Syn54crGATA4d-KO-48h

Ctrl | INDEL<br>80468 | 72.88%	Ins<br>58.59%	Del<br>14.29%	HDR<br>0.00%	AZD7762 | INDEL<br>24012 | 75.28%	Ins<br>57.38%	Del<br>17.90%	HDR<br>0.00%	AZD7762+VE822 | INDEL<br>28398 | 71.90%	Ins<br>58.19%	Del<br>13.67%	HDR<br>0.04%
**Typical seqs**	**Reads**	**Type**	**pct.**	**Typical seqs**	**Reads**	**Type**	**pct.**	**Typical seqs**	**Reads**	**Type**	**pct.**
TCCCTCCTCAAATTCCT|GCA**CGG**ACCT	21822	WT	27.12%	TCCCTCCTCAAATTCCT|GCA**CGG**ACCT	5935	WT	24.72%	TCCCTCCTCAAATTCCT|GCA**CGG**ACCT	7981	WT	28.10%
TCCCTCCTCAAATTCCTtGCACGGACCT	39627	+1 t	49.25%	TCCCTCCTCAAATTCCTtGCACGGACCT	11582	+1 t	48.23%	TCCCTCCTCAAATTCCTtGCACGGACCT	14095	+1 t	49.63%
TCCCTCCTCAAATTCCTCTGCACGGACCT	3768	+2	4.68%	TCCCTCCTCAAATTCCTCTGCACGGACCT	1048	+2	4.36%	TCCCTCCTCAAATTCCTCTGCACGGACCT	1183	+2	4.17%
TCCCTCCTCAAATT----------CCT	1703	-10	2.12%	TCCCTCCTCAAATT----------CCT	696	-10	2.90%	TCCCTCCTCAAATT----------CCT	554	-10	1.95%
TCCCTCCTCAAATTC-TGCACGGACCT	829	-1	1.03%	TCCCTCCTCAAATT--TG--AGGACCT	196	-4	0.82%	TCCCTCCTCAAATTCC-GCACGGACCT	273	-1	0.96%
TCCCTCCTCAAATTCC-GCACGGACCT	538	-1	0.67%	TCCCTCCTCAAATTC-TGCACGGACCT	177	-1	0.74%	TCCCTCCTCAAATTC-TGCACGGACCT	185	-1	0.65%
TCCCTCCTCAAATT---GCACGGACCT	429	-3	0.53%	TCCCT-------------------CCT	169	-19	0.70%	TCCCTCCTCAAATTCCCTGCACGGACCT	174	+1 Ins	0.61%
TCCCT-------------------CCT	383	-19	0.48%	TCCCTCCT----------CACGGACCT	129	-10	0.54%	TCCCTCCTCAAATT---GCACGGACCT	170	-3	0.60%
TCCCTCCTCAAATTCCCTGCACGGACCT	349	+1 Ins	0.43%	TCCCTCCTCAAATTC---CACGGACCT	127	-3	0.53%	TC----------------CACGGACCT	146	-16	0.51%
TCCCTCCTCAAATT--TG--AGGACCT	336	-4	0.42%	TCCCTCCTCAAATTCC-GCACGGACCT	116	-1	0.48%	TCCCTCCTCAAATTC---CACGGACCT	131	-3	0.46%

AZT | INDEL<br>100822 | 73.98%	Ins<br>59.97%	Del<br>14.01%	HDR<br>0.00%	B02 | INDEL<br>93791 | 71.56%	Ins<br>56.24%	Del<br>15.32%	HDR<br>0.00%	CsH | INDEL<br>101716 | 77.67%	Ins<br>64.99%	Del<br>12.67%	HDR<br>0.01%
**Typical seqs**	**Reads**	**Type**	**pct.**	**Typical seqs**	**Reads**	**Type**	**pct.**	**Typical seqs**	**Reads**	**Type**	**pct.**
TCCCTCCTCAAATTCCT|GCA**CGG**ACCT	26229	WT	26.02%	TCCCTCCTCAAATTCCT|GCA**CGG**ACCT	26675	WT	28.44%	TCCCTCCTCAAATTCCT|GCA**CGG**ACCT	22710	WT	22.33%
TCCCTCCTCAAATTCCTtGCACGGACCT	50047	+1 t	49.64%	TCCCTCCTCAAATTCCTtGCACGGACCT	44920	+1 t	47.89%	TCCCTCCTCAAATTCCTtGCACGGACCT	55946	+1 t	55.00%
TCCCTCCTCAAATTCCTCTGCACGGACCT	5510	+2	5.47%	TCCCTCCTCAAATTCCTCTGCACGGACCT	3383	+2	3.61%	TCCCTCCTCAAATTCCTCTGCACGGACCT	5642	+2	5.55%
TCCCTCCTCAAATT----------CCT	2149	-10	2.13%	TCCCTCCTCAAATT----------CCT	2310	-10	2.46%	TCCCTCCTCAAATT----------CCT	1726	-10	1.70%
TCCCTCCTCAAATTC-TGCACGGACCT	984	-1	0.98%	TCCCTCCTCAAATTC-TGCACGGACCT	1039	-1	1.11%	TCCCTCCTCAAATTC-TGCACGGACCT	968	-1	0.95%
TCCCTCCTCAAATTCC-GCACGGACCT	557	-1	0.55%	TCCCTCCTCAAAT----GCACGGACCT	629	-4	0.67%	TCCCTCCTCAAATTCC-GCACGGACCT	900	-1	0.88%
TCCCTCCTCAAATT---GCACGGACCT	471	-3	0.47%	TCCCTCCTCAAATTCCCTGCACGGACCT	578	+1 Ins	0.62%	TCCCT-------------------CCT	716	-19	0.70%
TCCCTCCTCAAATTCCCTGCACGGACCT	464	+1 Ins	0.46%	TCCCT-------------------CCT	489	-19	0.52%	TCCCTCCTCAAATTCCCTGCACGGACCT	677	+1 Ins	0.67%
TCCCTCCTCAAATTC---CACGGACCT	422	-3	0.42%	TCCCTCCTCAAATT---GCACGGACCT	485	-3	0.52%	TCCCTCCTCAAATT---GCACGGACCT	467	-3	0.46%
TCCCT-------------------CCT	377	-19	0.37%	TCCCTCCTCAAATTCC-GCACGGACCT	466	-1	0.50%	TCCCTCCTCAA-----TGCACGGACCT	430	-5	0.42%

YW-W9-Syn54crGATA4d-KO-48h

DOPA | INDEL	Ins	Del	HDR
122693 | 73.65%	59.58%	14.06%	0.00%
**Typical seqs**	**Reads**	**Type**	**pct.**
TCCCTCCTCAAATTCCT|GCACGGACCT	32335	WT	26.35%
TCCCTCCTCAAATTCCTtGCACGGACCT	60918	+1 t	49.65%
TCCCTCCTCAAATTCCTCTGCACGGACCT	6078	+2	4.95%
TCCCTCCTCAAATT----------CCT	2523	-10	2.06%
TCCCTCCTCAAATTC-TGCACGGACCT	1137	-1	0.93%
TCCCTCCTCAAATTCC-GCACGGACCT	683	-1	0.56%
TCCCT-------------------CCT	632	-19	0.52%
TCCCTCCTCAAATTCCCTGCACGGACCT	587	+1 Ins	0.48%
TCCCTCCTCAAATTC---CACGGACCT	502	-3	0.41%
TCCCTCCTCAAAT----GCACGGACCT	480	-4	0.39%

M3814 | INDEL	Ins	Del	HDR
123516 | 65.38%	45.79%	19.57%	0.01%
**Typical seqs**	**Reads**	**Type**	**pct.**
TCCCTCCTCAAATTCCT|GCACGGACCT	42766	WT	34.62%
TCCCTCCTCAAATTCCTtGCACGGACCT	45595	+1 t	36.91%
TCCCTCCTCAAATTCCTCTGCACGGACCT	5215	+2	4.22%
TCCCTCCTCAAATT----------CCT	3551	-10	2.87%
TCCCTCCT----------CACGGACCT	1770	-10	1.43%
TCCCTCCTCAAATTC-TGCACGGACCT	1385	-1	1.12%
TCCCT-------------------CCT	1322	-19	1.07%
TCCCTCCTCAAATTC---CACGGACCT	1130	-3	0.91%
TCCCTCCTCAAATTCCGTGCACGGACCT	783	+1 Ins	0.63%
TCCCTCCTCAAATTCC-GCACGGACCT	766	-1	0.62%

Mirin | INDEL	Ins	Del	HDR
118816 | 73.92%	59.47%	14.43%	0.02%
**Typical seqs**	**Reads**	**Type**	**pct.**
TCCCTCCTCAAATTCCT|GCACGGACCT	30982	WT	26.08%
TCCCTCCTCAAATTCCTtGCACGGACCT	60543	+1 t	50.96%
TCCCTCCTCAAATTCCTCTGCACGGACCT	5158	+2	4.34%
TCCCTCCTCAAATT----------CCT	1926	-10	1.62%
TCCCTCCTCAAATTC-TGCACGGACCT	1295	-1	1.09%
TCCCTCCTCAAATTCC-GCACGGACCT	968	-1	0.81%
TCCCT-------------------CCT	636	-19	0.54%
TCCCTCCT----------CACGGACCT	561	-10	0.47%
TCCCTCCTCAAATT---GCACGGACCT	545	-3	0.46%
TCCCTCCTCAAATTC---CACGGACCT	498	-3	0.42%

Nu7026+TrichostatinA | INDEL	Ins	Del	HDR
99160 | 75.50%	58.47%	17.01%	0.02%
**Typical seqs**	**Reads**	**Type**	**pct.**
TCCCTCCTCAAATTCCT|GCACGGACCT	24299	WT	24.50%
TCCCTCCTCAAATTCCTtGCACGGACCT	49442	+1 t	49.86%
TCCCTCCTCAAATTCCTCTGCACGGACCT	4158	+2	4.19%
TCCCTCCTCAAATT----------CCT	3438	-10	3.47%
TCCCTCCTCAAATTC-TGCACGGACCT	963	-1	0.97%
TCCCTCCTCAAATTCC-GCACGGACCT	614	-1	0.62%
TCCCT-------------------CCT	513	-19	0.52%
TCCCTCCTCAAATTC---CACGGACCT	511	-3	0.52%
TCCCTCCTCAAATT---GCACGGACCT	480	-3	0.48%
TCCCTCCTCAAATTCCTG---GGACCT	439	-3	0.44%

Nu7026 | INDEL	Ins	Del	HDR
115454 | 74.11%	59.23%	14.86%	0.02%
**Typical seqs**	**Reads**	**Type**	**pct.**
TCCCTCCTCAAATTCCT|GCACGGACCT	29894	WT	25.89%
TCCCTCCTCAAATTCCTtGCACGGACCT	58439	+1 t	50.62%
TCCCTCCTCAAATTCCTCTGCACGGACCT	4091	+2	3.54%
TCCCTCCTCAAATT----------CCT	2253	-10	1.95%
TCCCTCCTCAAATTC-TGCACGGACCT	1443	-1	1.25%
TCCCTCCTCAAATTCC-GCACGGACCT	996	-1	0.86%
TCCCTCCTCAAATTC---CACGGACCT	824	-3	0.71%
TCCCTCCTCAAATT--TG--AGGACCT	809	-4	0.70%
TCCCTCCTCAAATTCCCTGCACGGACCT	671	+1 Ins	0.58%
TCCCTCCTCAAATTCCT-CACGGACCT	631	-1	0.55%

Nu7441 | INDEL	Ins	Del	HDR
100135 | 69.79%	52.88%	16.91%	0.00%
**Typical seqs**	**Reads**	**Type**	**pct.**
TCCCTCCTCAAATTCCT|GCACGGACCT	30253	WT	30.21%
TCCCTCCTCAAATTCCTtGCACGGACCT	42981	+1 t	42.92%
TCCCTCCTCAAATTCCTCTGCACGGACCT	5069	+2	5.06%
TCCCTCCTCAAATT----------CCT	2629	-10	2.63%
TCCCTCCTCAAATTC-TGCACGGACCT	1102	-1	1.10%
TCCCTCCTCAAATT--TG--AGGACCT	1048	-4	1.05%
TCCCT-------------------CCT	715	-19	0.71%
TCCCTCCTCAAATTCCCTGCACGGACCT	612	+1 Ins	0.61%
TCCCTCCTCAAATTC---CACGGACCT	586	-3	0.59%
TCCCTCCTCAAATTCC-GCACGGACCT	581	-1	0.58%

YW-W9-Syn54crGATA4d-KO-48h

Olaparib | INDEL	Ins	Del	HDR
72804 | 74.38%	**59.06%**	**15.32%**	**0.00%**
**Typical seqs**	**Reads**	**Type**	**pct.**
TCCCTCCTCAAATTCCT|GCA**CGG**ACCT	18655	WT	25.62%
TCCCTCCTCAAATTCCTtGCACGGACCT	35943	+1 t	49.37%
TCCCTCCTCAAATTCCTCTGCACGGACCT	3745	+2	5.14%
TCCCTCCTCAAATT----------CCT	1694	-10	2.33%
TCCCTCCTCAAATTC-TGCACGGACCT	821	-1	1.13%
TCCCTCCTCAAATTCC-GCACGGACCT	379	-1	0.52%
TCCCTCCTCAAATTCCCTGCACGGACCT	318	+1 Ins	0.44%
TCCCTCCTCAAATT--TG--AGGACCT	319	-4	0.44%
TCCCTCCTCAAATT--TGCACGGACCT	314	-2	0.43%
TCCCTCCTCAAATT---GCACGGACCT	287	-3	0.39%

SCR7 | INDEL	Ins	Del	HDR
86601 | 72.72%	**58.21%**	**14.50%**	**0.00%**
**Typical seqs**	**Reads**	**Type**	**pct.**
TCCCTCCTCAAATTCCT|GCA**CGG**ACCT	23628	WT	27.28%
TCCCTCCTCAAATTCCTtGCACGGACCT	42507	+1 t	49.08%
TCCCTCCTCAAATTCCTCTGCACGGACCT	3944	+2	4.55%
TCCCTCCTCAAATT----------CCT	1824	-10	2.11%
TCCCTCCTCAAATTC-TGCACGGACCT	953	-1	1.10%
TCCCT-------------------CCT	605	-19	0.70%
TCCCTCCTCAAATTCC-GCACGGACCT	504	-1	0.58%
TCCCTCCT----------CACGGACCT	422	-10	0.49%
TCCCTCCTCAAATTCCCTGCACGGACCT	397	+1 Ins	0.46%
TCCCTCCTCAAATTC---CACGGACCT	352	-3	0.41%

Trichostatin A | INDEL	Ins	Del	HDR
75630 | 75.89%	**59.70%**	**16.17%**	**0.01%**
**Typical seqs**	**Reads**	**Type**	**pct.**
TCCCTCCTCAAATTCCT|GCA**CGG**ACCT	18238	WT	24.11%
TCCCTCCTCAAATTCCTtGCACGGACCT	38346	+1 t	50.70%
TCCCTCCTCAAATTCCTCTGCACGGACCT	3273	+2	4.33%
TCCCTCCTCAAATT----------CCT	2212	-10	2.92%
TCCCTCCTCAAATTC-TGCACGGACCT	582	-1	0.77%
TCCCTCCTCAA-----TGCACGGACCT	536	-5	0.71%
TCCCTCCTCAAATT--TG--AGGACCT	511	-4	0.68%
TCCCT-------------------CCT	425	-19	0.56%
TCCCTCCTCAAATTCC-GCACGGACCT	427	-1	0.56%
TCCCTCCTCAAATTC---CACGGACCT	435	-3	0.58%

VE822 | INDEL	Ins	Del	HDR
92240 | 72.53%	**59.26%**	**13.25%**	**0.02%**
**Typical seqs**	**Reads**	**Type**	**pct.**
TCCCTCCTCAAATTCCT|GCA**CGG**ACCT	25341	WT	27.47%
TCCCTCCTCAAATTCCTtGCACGGACCT	46478	+1 t	50.39%
TCCCTCCTCAAATTCCTCTGCACGGACCT	4104	+2	4.45%
TCCCTCCTCAAATT----------CCT	2039	-10	2.21%
TCCCTCCTCAAATTC-TGCACGGACCT	968	-1	1.05%
TCCCTCCTCAAATTCC-GCACGGACCT	687	-1	0.74%
TCCCTCCTCAAATT---GCACGGACCT	590	-3	0.64%
TCCCTCCTCAAATTCCCTGCACGGACCT	515	+1 Ins	0.56%
TCCCTCCTCAAATTC---CACGGACCT	404	-3	0.44%
TCCCTCCT----------CACGGACCT	370	-10	0.40%

YW-W9-Syn54crGATA4d-AAV6KI-48h

Ctrl | INDEL	Ins	Del	HDR
101050 | 77.29%	**57.13%**	**12.40%**	**7.76%**
**Typical seqs**	**Reads**	**Type**	**pct.**
TCCCTCCTCAAATTCCT|GCA**CGG**ACCT	22947	WT	22.71%
TCCCTCCTCAAATTCCTtGCACGGACCT	48838	+1 t	48.33%
TCCCT-------------CACG--CGTAGTTTA	7770	-9 HDR	7.69%
TCCCTCCTCAAATTCCTCTGCACGGACCT	5162	+2	5.11%
TCCCTCCTCAAATT----------CCT	1089	-10	1.08%
TCCCTCCTCAAATTCC-GCACGGACCT	897	-1	0.89%
TCCCTCCTCAAATTC-TGCACGGACCT	643	-1	0.64%
TCCCTCCTCAAATTCCT--ACGGACCT	524	-2	0.52%
TCCCTCCTCAAATTCCCTGCACGGACCT	506	+1 Ins	0.50%
TCCCTCCTCAAATT---GCACGGACCT	473	-3	0.47%

AZD7762 | INDEL	Ins	Del	HDR
28787 | 82.37%	**58.70%**	**14.84%**	**8.83%**
**Typical seqs**	**Reads**	**Type**	**pct.**
TCCCTCCTCAAATTCCT|GCA**CGG**ACCT	5076	WT	17.63%
TCCCTCCTCAAATTCCTtGCACGGACCT	14333	+1 t	49.79%
TCCCT-------------CACG--CGTAGTTTA	2520	-9 HDR	8.75%
TCCCTCCTCAAATTCCTCTGCACGGACCT	1516	+2	5.27%
TCCCTCCTCAAATTC-TGCACGGACCT	701	-1	2.44%
TCCCT-------------------CCT	378	-19	1.31%
TCCCTCCTCAAATT---GCACGGACCT	299	-3	1.04%
TCCCTCCTCAAATT----------CCT	209	-10	0.73%
T-----------------------CCT	164	-23	0.57%
TCCCTCCTC------------------	145	-21	0.50%

AZD7762+VE822 | INDEL	Ins	Del	HDR
12593 | 72.45%	**54.09%**	**8.18%**	**10.17%**
**Typical seqs**	**Reads**	**Type**	**pct.**
TCCCTCCTCAAATTCCT|GCA**CGG**ACCT	3470	WT	27.55%
TCCCTCCTCAAATTCCTtGCACGGACCT	5809	+1 t	46.13%
TCCCT-------------CACG--CGTAGTTTA	1269	-9 HDR	10.08%
TCCCTCCTCAAATTCCTCTGCACGGACCT	448	+2	3.56%
TCCCTCCTCAAATT----------CCT	139	-10	1.10%
TCCCTCCTCAAATTC-TGCACGGACCT	137	-1	1.09%
TCCCTCCTCAAATTCCTTTGCACGGACCT	105	+2	0.83%
TCCCTCCTCAAATTCCTATGCACGGACCT	95	+2	0.75%
TCCCTCCTCAAATTCCTG---GGACCT	45	-3	0.36%
TCCCTCCTCAAATT--TGCACGGACCT	39	-2	0.31%

AZT | INDEL	Ins	Del	HDR
121287 | 79.11%	**59.53%**	**11.53%**	**8.05%**
**Typical seqs**	**Reads**	**Type**	**pct.**
TCCCTCCTCAAATTCCT|GCA**CGG**ACCT	25337	WT	20.89%
TCCCTCCTCAAATTCCTtGCACGGACCT	60543	+1 t	49.92%
TCCCT-------------CACG--CGTAGTTTA	9693	-9 HDR	7.99%
TCCCTCCTCAAATTCCTCTGCACGGACCT	6771	+2	5.58%
TCCCTCCTCAAATTC-TGCACGGACCT	1314	-1	1.08%
TCCCTCCTCAAATT----------CCT	1206	-10	0.99%
TCCCTCCTCAAATTCC-GCACGGACCT	650	-1	0.54%
TCCCTCCTCAAATTCCCTGCACGGACCT	632	+1 Ins	0.52%
TCCCTCCTCAAATT---GCACGGACCT	585	-3	0.48%
TCCCTCCTCAAATTCCTTTGCACGGACCT	480	+2	0.40%

B02 | INDEL	Ins	Del	HDR
113764 | 79.45%	**58.66%**	**11.96%**	**8.84%**
**Typical seqs**	**Reads**	**Type**	**pct.**
TCCCTCCTCAAATTCCT|GCA**CGG**ACCT	23380	WT	20.55%
TCCCTCCTCAAATTCCTtGCACGGACCT	57309	+1 t	50.38%
TCCCT-------------CACG--CGTAGTTTA	9988	-9 HDR	8.78%
TCCCTCCTCAAATTCCTCTGCACGGACCT	5106	+2	4.49%
TCCCTCCTCAAATTC-TGCACGGACCT	1483	-1	1.30%
TCCCTCCTCAAATT---GCACGGACCT	1259	-3	1.11%
TCCCTCCTCAAATT----------CCT	812	-10	0.71%
TCCCTCCTCAAATTCCCTGCACGGACCT	706	+1 Ins	0.62%
TCCCTCCTCAAATTCCT--ACGGACCT	530	-2	0.47%
TCCCTCCTCAAATTCCTTTGCACGGACCT	521	+2	0.46%

CsH | INDEL	Ins	Del	HDR
60895 | 77.88%	**53.62%**	**11.62%**	**12.65%**
**Typical seqs**	**Reads**	**Type**	**pct.**
TCCCTCCTCAAATTCCT|GCA**CGG**ACCT	13467	WT	22.12%
TCCCTCCTCAAATTCCTtGCACGGACCT	28158	+1 t	46.24%
TCCCT-------------CACG--CGTAGTTTA	7597	-9 HDR	12.48%
TCCCTCCTCAAATTCCTCTGCACGGACCT	1980	+2	3.25%
TCCCTCCTCAAATT----------CCT	822	-10	1.35%
TCCCTCCTCAAATTC-TGCACGGACCT	423	-1	0.69%
TCCCTCCTCAAATT--TGCACGGACCT	397	-2	0.65%
TCCCTCCTCAAATTCC-GCACGGACCT	373	-1	0.61%
TCCCTCCTCAAATT---GCACGGACCT	375	-3	0.62%
TCCCTCCTCAAATTCCT--ACGGACCT	298	-2	0.49%

YW-W9-Syn54crGATA4d-AAV6KI-48h

DOPA | INDEL	Ins	Del	HDR
139619 | 78.26%	59.66%	11.79%	6.80%
**Typical seqs**	**Reads**	**Type**	**pct.**
TCCCTCCTCAAATTCCT|GCA**CGG**ACCT	30354	WT	21.74%
TCCCTCCTCAAATTCCTtGCACGGACCT	70578	+1 t	50.55%
TCCCT-------------CACG--CGTAGTTTA	9432	-9 HDR	6.76%
TCCCTCCTCAAATTCCTCTGCACGGACCT	6768	+2	4.85%
TCCCTCCTCAAATT----------CCT	2678	-10	1.92%
TCCCTCCTCAAATTC-TGCACGGACCT	1204	-1	0.86%
TCCCTCCTCAAATTCCCTGCACGGACCT	728	+1 Ins	0.52%
TCCCTCCTCAAATTCC-GCACGGACCT	697	-1	0.50%
TCCCT-------------------CCT	674	-19	0.48%
TC------------CCTGCACGGACCT	569	-12	0.41%

M3814 | INDEL	Ins	Del	HDR
70765 | 74.69%	41.90%	12.64%	20.15%
**Typical seqs**	**Reads**	**Type**	**pct.**
TCCCTCCTCAAATTCCT|GCA**CGG**ACCT	17914	WT	25.31%
TCCCTCCTCAAATTCCTtGCACGGACCT	24369	+1 t	34.44%
TCCCT-------------CACG--CGTAGTTTA	13947	-9 HDR	19.71%
TCCCTCCTCAAATTCCTCTGCACGGACCT	2437	+2	3.44%
TCCCTCCTCAAATT----------CCT	903	-10	1.28%
TCCCTCCTCAAATTC-TGCACGGACCT	384	-1	0.54%
TCCCTCCTCAAATT----CACGGACCT	367	-4	0.52%
TCCCTCCTCAAATT---GCACGGACCT	345	-3	0.49%
TC----------------CACGGACCT	316	-16	0.45%
TCCCTCCTCAAATT--TGCACGGACCT	307	-2	0.43%

Mirin | INDEL	Ins	Del	HDR
67392 | 76.42%	54.91%	9.99%	11.51%
**Typical seqs**	**Reads**	**Type**	**pct.**
TCCCTCCTCAAATTCCT|GCA**CGG**ACCT	15892	WT	23.58%
TCCCTCCTCAAATTCCTtGCACGGACCT	31146	+1 t	46.22%
TCCCT-------------CACG--CGTAGTTTA	7651	-9 HDR	11.35%
TCCCTCCTCAAATTCCTCTGCACGGACCT	2784	+2	4.13%
TCCCTCCTCAAATTC-TGCACGGACCT	534	-1	0.79%
TCCCTCCTCAAATTCCTTTGCACGGACCT	547	+2	0.81%
TCCCTCCTCAAATT----------CCT	522	-10	0.77%
TCCCTCCTCAAATT---GCACGGACCT	370	-3	0.55%
TCCCTCCT---------GCACGGACCT	322	-9	0.48%
TCCCTCCTCAAATTCCTATGCACGGACCT	316	+2	0.47%

Nu7026+TrichostatinA | INDEL	Ins	Del	HDR
59288 | 80.86%	51.18%	10.77%	18.92%
**Typical seqs**	**Reads**	**Type**	**pct.**
TCCCTCCTCAAATTCCT|GCA**CGG**ACCT	11346	WT	19.14%
TCCCTCCTCAAATTCCTtGCACGGACCT	26089	+1 t	44.00%
TCCCT-------------CACG--CGTAGTTTA	11049	-9 HDR	18.64%
TCCCTCCTCAAATTCCTCTGCACGGACCT	1774	+2	2.99%
TCCCTCCTCAAATT----------CCT	682	-10	1.15%
TCCCTCCTCAAATTC-TGCACGGACCT	429	-1	0.72%
TCCCTCCTCAAATTCCTTTGCACGGACCT	345	+2	0.58%
TCCCTCCTCAAATT--TGCACGGACCT	338	-2	0.57%
TCCCTCCTCAAAT----GCACGGACCT	282	-4	0.48%
TCCCTCCTCAAATTCCTATGCACGGACCT	262	+2	0.44%

Nu7026 | INDEL	Ins	Del	HDR
66605 | 77.99%	54.17%	11.18%	12.64%
**Typical seqs**	**Reads**	**Type**	**pct.**
TCCCTCCTCAAATTCCT|GCA**CGG**ACCT	14658	WT	22.01%
TCCCTCCTCAAATTCCTtGCACGGACCT	30527	+1 t	45.83%
TCCCT-------------CACG--CGTAGTTTA	8305	-9 HDR	12.47%
TCCCTCCTCAAATTCCTCTGCACGGACCT	2552	+2	3.83%
TCCCTCCTCAAATT----------CCT	814	-10	1.22%
TCCCTCCTCAAATTC-TGCACGGACCT	549	-1	0.82%
TCCCTCCTCAAATT----CACGGACCT	387	-4	0.58%
TCCCTCCTCAAATT--TGCACGGACCT	378	-2	0.57%
TCCCTCCTCAAATTCCTTTGCACGGACCT	364	+2	0.55%
TCCCTCCTCAAATT---GCACGGACCT	292	-3	0.44%

Nu7441 | INDEL	Ins	Del	HDR
128594 | 75.56%	51.86%	12.87%	10.83%
**Typical seqs**	**Reads**	**Type**	**pct.**
TCCCTCCTCAAATTCCT|GCA**CGG**ACCT	31432	WT	24.44%
TCCCTCCTCAAATTCCTtGCACGGACCT	56070	+1 t	43.60%
TCCCT-------------CACG--CGTAGTTTA	13814	-9 HDR	10.74%
TCCCTCCTCAAATTCCTCTGCACGGACCT	6235	+2	4.85%
TCCCTCCTCAAATT----------CCT	2222	-10	1.73%
TCCCTCCTCAAATTC-TGCACGGACCT	938	-1	0.73%
TCCCTCCTCAAATTCC-GCACGGACCT	789	-1	0.61%
TCCCTCCTCAAATT---GCACGGACCT	763	-3	0.59%
TCCCT-------------------CCT	662	-19	0.51%
T-----------------------CCT	484	-23	0.38%

YW-W9-Syn54crGATA4d-AAV6KI-48h

Olaparib | INDEL<br>83079 | 77.03%	Ins<br>56.68%	Del<br>13.09%	HDR<br>7.26%
**Typical seqs**	**Reads**	**Type**	**pct.**
TCCCTCCTCAAATTCCT|GCA**CGG**ACCT	19086	WT	22.97%
TCCCTCCTCAAATTCCTtGCACGGACCT	40230	+1 t	48.42%
TCCCT-------------CACG--CGTAGTTTA	5988	-9 HDR	7.21%
TCCCTCCTCAAATTCCTCTGCACGGACCT	4195	+2	5.05%
TCCCTCCTCAAATT----------CCT	1463	-10	1.76%
TCCCTCCTCAAATTC-TGCACGGACCT	933	-1	1.12%
TCCCTCCTCAAATTCC-GCACGGACCT	522	-1	0.63%
TCCCTCCTCAAATT---GCACGGACCT	500	-3	0.60%
TC----------------------CCT	493	-22	0.59%
TCCCT-------------------CCT	393	-19	0.47%

SCR7 | INDEL<br>104945 | 80.67%	Ins<br>57.74%	Del<br>14.07%	HDR<br>8.86%
**Typical seqs**	**Reads**	**Type**	**pct.**
TCCCTCCTCAAATTCCT|GCA**CGG**ACCT	20290	WT	19.33%
TCCCTCCTCAAATTCCTtGCACGGACCT	51934	+1 t	49.49%
TCCCT-------------CACG--CGTAGTTTA	9238	-9 HDR	8.80%
TCCCTCCTCAAATTCCTCTGCACGGACCT	4579	+2	4.36%
TCCCTCCTCAAATTC-TGCACGGACCT	1439	-1	1.37%
TCCCTCCTCAAATT----------CCT	1098	-10	1.05%
TCCCT-------------------CCT	736	-19	0.70%
TCCCTCCTCAAATT---GCACGGACCT	702	-3	0.67%
TCCCTCCTCAAATTCC-GCACGGACCT	620	-1	0.59%
TCCCTCCTCAAATTCCCTGCACGGACCT	593	+1 Ins	0.57%

TrichostatinA | INDEL<br>38146 | 80.57%	Ins<br>56.70%	Del<br>10.26%	HDR<br>13.62%
**Typical seqs**	**Reads**	**Type**	**pct.**
TCCCTCCTCAAATTCCT|GCA**CGG**ACCT	7411	WT	19.43%
TCCCTCCTCAAATTCCTtGCACGGACCT	18216	+1 t	47.75%
TCCCT-------------CACG--CGTAGTTTA	5111	-9 HDR	13.40%
TCCCTCCTCAAATTCCTCTGCACGGACCT	1495	+2	3.92%
TCCCTCCTCAAATT----------CCT	387	-10	1.01%
TCCCTCCTCAAATTCCTTTGCACGGACCT	301	+2	0.79%
TCCCTCCTCAAATTC-TGCACGGACCT	280	-1	0.73%
TCCCTCCTCAAATTCCT--ACGGACCT	205	-2	0.54%
TCCCTCCTCAAATTCCTATGCACGGACCT	182	+2	0.48%
TCCCTCCTCAAATTCCCTGCACGGACCT	156	+1 Ins	0.41%

VE822 | INDEL<br>40567 | 77.35%	Ins<br>56.46%	Del<br>9.94%	HDR<br>10.95%
**Typical seqs**	**Reads**	**Type**	**pct.**
TCCCTCCTCAAATTCCT|GCA**CGG**ACCT	9187	WT	22.65%
TCCCTCCTCAAATTCCTtGCACGGACCT	19470	+1 t	47.99%
TCCCT-------------CACG--CGTAGTTTA	4370	-9 HDR	10.77%
TCCCTCCTCAAATTCCTCTGCACGGACCT	1675	+2	4.13%
TCCCTCCTCAAATT----------CCT	451	-10	1.11%
TCCCTCCTCAAATTC-TGCACGGACCT	344	-1	0.85%
TCCCTCCTCAAATTCCTTTGCACGGACCT	194	+2	0.48%
TCCCTCCTCAAATT---GCACGGACCT	191	-3	0.47%
TCCCTCCTCAAATT--TGCACGGACCT	181	-2	0.45%
TCCCTCCTCAAATTCC-GCACGGACCT	170	-1	0.42%

YW-W9-Syn57crGATA4h-KO-48h

Ctrl | INDEL	Ins	Del	HDR
81565 | 43.56%	3.14%	40.43%	0.00%
**Typical seqs**	**Reads**	**Type**	**pct.**
GTCCGTGCAGGAATTTG|AGGAGGGAAG	46032	WT	56.44%
GTCCGTGC---------AGGAGGGAAG	11868	-9	14.55%
GTCCGTGCAGGAATTTG-GGAGGGAAG	5641	-1	6.92%
GTCCGTGCAGGAATTT--GGAGGGAAG	1368	-2	1.68%
GTCCGTGCAGGAATTT-AGGAGGGAAG	1216	-1	1.49%
GTCCGTGC------------AGGGAAG	1176	-12	1.44%
GTCCGTGCA-------------GGAAG	997	-13	1.22%
-----------------AGGAGGGAAG	783	-19	0.96%
GTCCGTGCAGGAATT--AGGAGGGAAG	687	-2	0.84%
GTCCGTGCAGGAATTT---GAGGGAAG	685	-3	0.84%

AZD7762 | INDEL	Ins	Del	HDR
20901 | 48.90%	3.39%	45.51%	0.00%
**Typical seqs**	**Reads**	**Type**	**pct.**
GTCCGTGCAGGAATTTG|AGGAGGGAAG	10681	WT	51.10%
GTCCGTGC---------AGGAGGGAAG	4149	-9	19.85%
GTCCGTGCAGGAATTTG-GGAGGGAAG	1206	-1	5.77%
GTCCGTGCAGGAATTT--GGAGGGAAG	384	-2	1.84%
GTCCGTGCA-------------GGAAG	353	-13	1.69%
GTCCGTGC------------AGGGAAG	344	-12	1.65%
GTCCGTGCAGGAATTT-AGGAGGGAAG	256	-1	1.22%
-----------------AGGAGGGAAG	230	-19	1.10%
GTCCGTGCAGGAATTTGgAGGAGGGAAG	201	+1 g	0.96%
GTCCGTGCAGGAATT--AGGAGGGAAG	200	-2	0.96%

AZD7762+VE822 | INDEL	Ins	Del	HDR
6912 | 42.35%	3.60%	38.74%	0.00%
**Typical seqs**	**Reads**	**Type**	**pct.**
GTCCGTGCAGGAATTTG|AGGAGG	3985	WT	57.65%
GTCCGTGC---------AGGAGG	892	-9	12.91%
GTCCGTGCAGGAATTTG-GGAGG	656	-1	9.49%
GTCCGTGCAGGAATTT-AGGAGG	125	-1	1.81%
GTCCGTGCAGGAATTT--GGAGG	104	-2	1.50%
GTCCGTGCAGGAATTTGTAGGAGG	87	+1	1.26%
GTCCGTGCA-------------G	80	-13	1.16%
-----------------AGGAGG	80	-19	1.16%
GTCCGTGCAGGAATTT---GAGG	75	-3	1.09%
GTCCGTGC------------AGG	67	-12	0.97%

AZT | INDEL	Ins	Del	HDR
120595 | 43.36%	3.31%	40.04%	0.00%
**Typical seqs**	**Reads**	**Type**	**pct.**
GTCCGTGCAGGAATTTG|AGGAGGGAAG	68307	WT	56.64%
GTCCGTGC---------AGGAGGGAAG	17807	-9	14.77%
GTCCGTGCAGGAATTTG-GGAGGGAAG	8050	-1	6.68%
GTCCGTGCAGGAATTT--GGAGGGAAG	2258	-2	1.87%
GTCCGTGCAGGAATTT-AGGAGGGAAG	1812	-1	1.50%
GTCCGTGC------------AGGGAAG	1679	-12	1.39%
GTCCGTGCA-------------GGAAG	1345	-13	1.12%
GTCCGTGCAGGAATTT---GAGGGAAG	1186	-3	0.98%
-----------------AGGAGGGAAG	1102	-19	0.91%
GTCCGTGCAGGAATTTGgAGGAGGGAAG	1058	+1 g	0.88%

B02 | INDEL	Ins	Del	HDR
102570 | 43.10%	3.09%	40.01%	0.00%
**Typical seqs**	**Reads**	**Type**	**pct.**
GTCCGTGCAGGAATTTG|AGGAGGGAAG	58364	WT	56.90%
GTCCGTGC---------AGGAGGGAAG	14633	-9	14.27%
GTCCGTGCAGGAATTTG-GGAGGGAAG	6717	-1	6.55%
GTCCGTGCAGGAATTT--GGAGGGAAG	1957	-2	1.91%
GTCCGTGCAGGAATTT-AGGAGGGAAG	1459	-1	1.42%
GTCCGTGCA-------------GGAAG	1388	-13	1.35%
GTCCGTGC------------AGGGAAG	1282	-12	1.25%
GTCCGTGCAGGAATTT---GAGGGAAG	1140	-3	1.11%
GTCCGTGCAGGAATT--AGGAGGGAAG	941	-2	0.92%
GTCCGTGCAGGAATTTGgAGGAGGGAAG	857	+1 g	0.84%

CsH | INDEL	Ins	Del	HDR
56929 | 45.41%	3.98%	41.44%	0.00%
**Typical seqs**	**Reads**	**Type**	**pct.**
GTCCGTGCAGGAATTTG|AGGAGG	31075	WT	54.59%
GTCCGTGC---------AGGAGG	6022	-9	10.58%
GTCCGTGCAGGAATTTG-GGAGG	4776	-1	8.39%
GTCCGTGCAGGAATTT--GGAGG	1487	-2	2.61%
GTCCGTGCAGGAATTT-AGGAGG	1108	-1	1.95%
GTCCGTGCAGGAATTT-------	1353	-10	2.38%
GTCCGTGCAGGAATTT---GAGG	1043	-3	1.83%
GTCCGTGCA-------------G	784	-13	1.38%
GTCCGTGC------------AGG	780	-12	1.37%
GTCCGTGCAGGAATT--AGGAGG	624	-2	1.10%

YW-W9-Syn57crGATA4h-KO-48h

DOPA | INDEL	Ins	Del	HDR
138754 | 43.94%	3.65%	40.29%	0.00%
**Typical seqs**	**Reads**	**Type**	**pct.**
GTCCGTGCAGGAATTTG|AGGAGGGAAG	77788	WT	56.06%
GTCCGTGC---------AGGAGGGAAG	19676	-9	14.18%
GTCCGTGCAGGAATTTG-GGAGGGAAG	8950	-1	6.45%
GTCCGTGCAGGAATTT--GGAGGGAAG	2771	-2	2.00%
GTCCGTGCAGGAATTT-AGGAGGGAAG	2094	-1	1.51%
GTCCGTGCA-------------GGAAG	1887	-13	1.36%
GTCCGTGCAGGAATTT---GAGGGAAG	1763	-3	1.27%
GTCCGTGC------------AGGGAAG	1548	-12	1.12%
-------------------AGGAGGGAAG	1366	-19	0.98%
GTCCGTGCAGGAATTTGgAGGAGGGAAG	1340	+1 g	0.97%

M3814 | INDEL	Ins	Del	HDR
123913 | 41.29%	3.01%	38.28%	0.00%
**Typical seqs**	**Reads**	**Type**	**pct.**
GTCCGTGCAGGAATTTG|AGGAGG	72748	WT	58.71%
GTCCGTGC---------AGGAGG	20079	-9	16.20%
GTCCGTGCAGGAATTTG-GGAGG	5719	-1	4.62%
GTCCGTGCA-------------G	2734	-13	2.21%
GTCCGTGCAGGAATTT--GGAGG	2701	-2	2.18%
GTCCGTGCAGGAATTT-AGGAGG	2599	-1	2.10%
GTCCGTGCAGGAATTT---GAGG	1320	-3	1.07%
GTCCGTGCAGGAATTTGTAGGAGG	1038	+1	0.84%
GTCCGT-------------GAGG	837	-13	0.68%
---------------------GG	802	-21	0.65%

Mirin | INDEL	Ins	Del	HDR
113654 | 41.78%	3.39%	38.39%	0.00%
**Typical seqs**	**Reads**	**Type**	**pct.**
GTCCGTGCAGGAATTTG|AGGAGG	66170	WT	58.22%
GTCCGTGC---------AGGAGG	11484	-9	10.10%
GTCCGTGCAGGAATTTG-GGAGG	10164	-1	8.94%
GTCCGTGCAGGAATTT--GGAGG	2508	-2	2.21%
GTCCGTGCAGGAATTT-AGGAGG	2143	-1	1.89%
GTCCGTGCA-------------G	1437	-13	1.26%
GTCCGTGCAGGAATTT-------	2443	-10	2.15%
GTCCGTGC------------AGG	1238	-12	1.09%
GTCCGTGCAGGAATTTGTAGGAGG	1189	+1	1.05%
GTCCGTGCAGGAATTT---GAGG	1100	-3	0.97%

Nu7026 | INDEL	Ins	Del	HDR
104682 | 41.31%	3.44%	37.86%	0.00%
**Typical seqs**	**Reads**	**Type**	**pct.**
GTCCGTGCAGGAATTTG|AGGAGG	61441	WT	58.69%
GTCCGTGC---------AGGAGG	14798	-9	14.14%
GTCCGTGCAGGAATTTG-GGAGG	5952	-1	5.69%
GTCCGTGCAGGAATTT-AGGAGG	1775	-1	1.70%
GTCCGTGCAGGAATTT--GGAGG	1685	-2	1.61%
GTCCGTGCA-------------G	1644	-13	1.57%
GTCCGTGCAGGAATTT---GAGG	1355	-3	1.29%
GTCCGTGC------------AGG	1265	-12	1.21%
-------------------AGGAGG	998	-19	0.95%
GTCCGTGCAGGAATTTGTAGGAGG	859	+1	0.82%

Nu7026+TrichostatinA | INDEL	Ins	Del	HDR
97558 | 40.10%	4.87%	35.22%	0.00%
**Typical seqs**	**Reads**	**Type**	**pct.**
GTCCGTGCAGGAATTTG|AGGAGG	58441	WT	59.90%
GTCCGTGC---------AGGAGG	13009	-9	13.33%
GTCCGTGCAGGAATTTG-GGAGG	6627	-1	6.79%
GTCCGTGCAGGAATTT-------	2445	-10	2.51%
GTCCGTGCAGGAATTT-AGGAGG	1449	-1	1.49%
GTCCGTGCAGGAATTT--GGAGG	1364	-2	1.40%
GTCCGTGCAGGAATTTGGAGGAGG	1113	+1	1.14%
-------------------AGGAGG	958	-19	0.98%
GTCCGTGCAGGAATTT---GAGG	902	-3	0.92%
GTCCGTGCAGGAATTTGTAGGAGG	866	+1	0.89%

Nu7441 | INDEL	Ins	Del	HDR
128845 | 41.18%	3.19%	37.99%	0.00%
**Typical seqs**	**Reads**	**Type**	**pct.**
GTCCGTGCAGGAATTTG|AGGAGGGAAG	75787	WT	58.82%
GTCCGTGC---------AGGAGGGAAG	20648	-9	16.03%
GTCCGTGCAGGAATTTG-GGAGGGAAG	7940	-1	6.16%
GTCCGTGCA-------------GGAAG	1697	-13	1.32%
GTCCGTGC------------AGGGAAG	1673	-12	1.30%
GTCCGTGCAGGAATTT--GGAGGGAAG	1604	-2	1.24%
GTCCGTGCAGGAATTT-AGGAGGGAAG	1448	-1	1.12%
GTCCGTGCAGGAATTT---GAGGGAAG	1174	-3	0.91%
GTCCGTGCAGGAATTTGgAGGAGGGAAG	912	+1 g	0.71%
GTCCGTGCAGGAATT--AGGAGGGAAG	834	-2	0.65%

YW-W9-Syn57crGATA4h-KO-48h

Olaparib | INDEL	Ins	Del	HDR
65357 | 41.00%	4.32%	36.68%	0.00%
**Typical seqs**	**Reads**	**Type**	**pct.**
GTCCGTGCAGGAATTTG|AGGAGGGAAG	38558	WT	59.00%
GTCCGTGC---------AGGAGGGAAG	8727	-9	13.35%
GTCCGTGCAGGAATTTG-GGAGGGAAG	3085	-1	4.72%
GTCCGTGCAGGAATTT--GGAGGGAAG	1174	-2	1.80%
GTCCGTGCAGGAATTT-AGGAGGGAAG	1038	-1	1.59%
GTCCGTGCAGGAATTT---GAGGGAAG	712	-3	1.09%
GTCCGTGCAGGAATT--AGGAGGGAAG	667	-2	1.02%
GTCCGTGC------------AGGGAAG	688	-12	1.05%
GTCCGTGCAGGAATTTGgAGGAGGGAAG	634	+1 g	0.97%
GTCCGTGCA-------------GGAAG	591	-13	0.90%

SCR7 | INDEL	Ins	Del	HDR
98408 | 44.32%	3.74%	40.58%	0.00%
**Typical seqs**	**Reads**	**Type**	**pct.**
GTCCGTGCAGGAATTTG|AGGAGGGAAG	54798	WT	55.68%
GTCCGTGC---------AGGAGGGAAG	14271	-9	14.50%
GTCCGTGCAGGAATTTG-GGAGGGAAG	7323	-1	7.44%
GTCCGTGCAGGAATTT--GGAGGGAAG	2041	-2	2.07%
GTCCGTGCAGGAATTT-AGGAGGGAAG	1661	-1	1.69%
GTCCGTGCA-------------GGAAG	1282	-13	1.30%
GTCCGTGC------------AGGGAAG	1124	-12	1.14%
GTCCGTGCAGGAATTTGgAGGAGGGAAG	1008	+1 g	1.02%
GTCCGTGCAGGAATTT---GAGGGAAG	889	-3	0.90%
-------------------AGGAGGGAAG	812	-19	0.83%

TrichostatinA | INDEL	Ins	Del	HDR
34375 | 49.50%	3.56%	45.94%	0.00%
**Typical seqs**	**Reads**	**Type**	**pct.**
GTCCGTGCAGGAATTTG|AGGAGG	17359	WT	50.50%
GTCCGTGC---------AGGAGG	5123	-9	14.90%
GTCCGTGCAGGAATTTG-GGAGG	3347	-1	9.74%
GTCCGTGCAGGAATTT--GGAGG	941	-2	2.74%
GTCCGTGCAGGAATTT-AGGAGG	626	-1	1.82%
GTCCGTGC------------AGG	546	-12	1.59%
GTCCGTGCA-------------G	510	-13	1.48%
-------------------AGGAGG	354	-19	1.03%
GTCCGTGCAGGAATTTGTAGGAGG	326	+1	0.95%
GTCCGTGCAGGAATTT-------	634	-12	1.84%

VE822 | INDEL	Ins	Del	HDR
41645 | 39.99%	3.04%	36.94%	0.00%
**Typical seqs**	**Reads**	**Type**	**pct.**
GTCCGTGCAGGAATTTG|AGGAGG	24992	WT	60.01%
GTCCGTGC---------AGGAGG	4716	-9	11.32%
GTCCGTGCAGGAATTTG-GGAGG	2742	-1	6.58%
GTCCGTGCAGGAATTT--GGAGG	952	-2	2.29%
GTCCGTGCAGGAATTT-AGGAGG	730	-1	1.75%
GTCCGTGCAGGAATTT-------	1097	-10	2.63%
GTCCGTGCA-------------G	560	-13	1.34%
GTCCGTGC------------AGG	443	-12	1.06%
-----------------AGGAGG	340	-19	0.82%
GTCCGTGCAGGAATTT---GAGG	336	-3	0.81%

P108-P115-YW-W9-Syn77crBCL11A-KO

4h

YW-W9-Syn77crBCL11A2a-KO(AAV)-4h	Ins	Del	HDR
38300 | 25.58%	**21.02%**	**4.56%**	**0.00%**
**Typical seqs**	**Reads**	**Type**	**pct.**
TGAGCCATTCGGTCGCT|AGG**AGG**CAGA	28503	WT	74.42%
TGAGCCATTCGGTCGCTtAGGAGGCAGA	7564	+1 t	19.75%
TGAGCCATTCGGTCGC-AGGAGGCAGA	595	-1	1.55%
TGAGCCATTCGGTCGCT-GGAGGCAGA	433	-1	1.13%
TGAGCCATTCGGTC--------GCAGA	215	-8	0.56%
TGAGCC-----------AGGAGGCAGA	163	-11	0.43%
TGAGCCATTCGG-----AGGAGGCAGA	84	-5	0.22%
TGAGCCATTCGGTCGCTcAGGAGGCAGA	74	+1 c	0.19%
TGAGCCATTCGGTCGCTCTAGGAGGCAGA	73	+2	0.19%
TGAGCCATTCGGTCGCT-----GCAGA	71	-5	0.19%

8h

YW-W9-Syn77crBCL11A2a-KO(AAV)-8h	Ins	Del	HDR
31722 | 52.15%	**38.45%**	**13.70%**	**0.00%**
**Typical seqs**	**Reads**	**Type**	**pct.**
TGAGCCATTCGGTCGCT|AGG**AGG**CAGA	15180	WT	47.85%
TGAGCCATTCGGTCGCTtAGGAGGCAGA	11273	+1 t	35.54%
TGAGCCATTCGGTC--------GCAGA	576	-8	1.82%
TGAGCCATTCGGTCGC-AGGAGGCAGA	560	-1	1.77%
TGAGCCATTC--------GGAGGCAGA	446	-8	1.41%
TGAGCCATTCGGTCG--AGGAGGCAGA	368	-2	1.16%
TGAGCCATTCGGTCGCT---GGGCAGA	262	-3	0.83%
TG---------------AGGAGGCAGA	215	-15	0.68%
TGAGCCATTCGGTCGCT--GAGGCAGA	172	-2	0.54%
T------------------GAGGCAGA	159	-18	0.50%

12h

YW-W9-Syn77crBCL11A2a-KO(AAV)-12h	Ins	Del	HDR
30093 | 65.37%	**43.81%**	**21.57%**	**0.00%**
**Typical seqs**	**Reads**	**Type**	**pct.**
TGAGCCATTCGGTCGCT|AGG**AGG**CAGA	10420	WT	34.63%
TGAGCCATTCGGTCGCTtAGGAGGCAGA	11923	+1 t	39.62%
TGAGCCATTCGGTCGC-AGGAGGCAGA	1133	-1	3.76%
TGAGCCATTCGGTC--------GCAGA	705	-8	2.34%
TGAGCCATTC--------GGAGGCAGA	537	-8	1.78%
TGAGCCATTCGGTCGCT-GGAGGCAGA	494	-1	1.64%
TGAGCCATTCGG----TAGGAGGCAGA	364	-4	1.21%
TG---------------AGGAGGCAGA	324	-15	1.08%
TGAGCC-----------AGGAGGCAGA	212	-11	0.70%
TGAGCCATTCGGTCG-TAGGAGGCAGA	208	-1	0.69%

24h

YW-W9-Syn77crBCL11A2a-KO(AAV)-24h	Ins	Del	HDR
12467 | 78.71%	**51.05%**	**27.66%**	**0.00%**
**Typical seqs**	**Reads**	**Type**	**pct.**
TGAGCCATTCGGTCGCT|AGG**AGG**CAGA	2654	WT	21.29%
TGAGCCATTCGGTCGCTtAGGAGGCAGA	5768	+1 t	46.27%
TGAGCCATTCGGTC--------GCAGA	624	-8	5.01%
TGAGCCATTCGGTCGC-AGGAGGCAGA	298	-1	2.39%
TGAGCCATTCGGTCGCT-GGAGGCAGA	221	-1	1.77%
TGAGCCATTCGGTCGC--GGAGGCAGA	198	-2	1.59%
TGAGCCATTC--------GGAGGCAGA	179	-8	1.44%
TGAGCC-----------AGGAGGCAGA	167	-11	1.34%
TGAGCCATTCGGTCG-TAGGAGGCAGA	128	-1	1.03%
TGAGCCATTCGGTCG--AGGAGGCAGA	108	-2	0.87%

48h

YW-W9-Syn77crBCL11A2a-KO(AAV)-48h	Ins	Del	HDR
42874 | 80.65%	**49.62%**	**31.03%**	**0.00%**
**Typical seqs**	**Reads**	**Type**	**pct.**
TGAGCCATTCGGTCGCT|AGG**AGG**CAGA	8295	WT	19.35%
TGAGCCATTCGGTCGCTtAGGAGGCAGA	18576	+1 t	43.33%
TGAGCCATTCGGTC--------GCAGA	2549	-8	5.95%
TGAGCCATTCGGTCGC-AGGAGGCAGA	1162	-1	2.71%
TGAGCCATTC--------GGAGGCAGA	927	-8	2.16%
TGAGCCATTCGGTCGCT-GGAGGCAGA	657	-1	1.53%
TGAGCC-----------AGGAGGCAGA	441	-11	1.03%
TGAGCCATTCGGTCG-TAGGAGGCAGA	419	-1	0.98%
TG---------------AGGAGGCAGA	320	-15	0.75%
T------------------GAGGCAGA	312	-18	0.73%

P108-P115-YW-W9-Syn78crBCL11A-KO

YW-W9-Syn78crBCL11A2b-KO(AAV)-4h **4h**	Ins	Del	HDR
55414 | 16.95%	**4.26%**	**12.68%**	**0.00%**
**Typical seqs**	**Reads**	**Type**	**pct.**
CGAATTCTCTCTACGAG|GGG**AGG**AAGA	46024	WT	83.05%
CGAATTCTCTCTACGA-GGGAGGAAGA	4151	-1	7.49%
CGAATTCTCTCTACGAGgGGGAGGAAGA	1032	+1 g	1.86%
CGAATTCTCTCTACGA--GGAGGAAGA	840	-2	1.52%
CGAATTCTCTCTACGAGAGGGGAGGAAGA	638	+2	1.15%
CGAATTCTCTCTAC-----GAGGAAGA	553	-5	1.00%
CGAATTCTCTCTACGAAGGGGAGGAAGA	309	+1 Ins	0.56%
CGAATTCTCTCTAC----GGAGGAAGA	266	-4	0.48%
CGAATTCTCTCTACG-GGGGAGGAAGA	230	-1	0.42%
CGAATTCTCTCTACGAGAAGGGAGGAAGA	195	+2	0.35%

YW-W9-Syn78crBCL11A2b-KO(AAV)-8h **8h**	Ins	Del	HDR
14080 | 30.51%	**7.28%**	**23.23%**	**0.00%**
**Typical seqs**	**Reads**	**Type**	**pct.**
CGAATTCTCTCTACGAG|GGG**AGG**AAGA	9784	WT	69.49%
CGAATTCTCTCTACGA-GGGAGGAAGA	1908	-1	13.55%
CGAATTCTCTCTACGAGgGGGAGGAAGA	612	+1 g	4.35%
CGAATTCTCTCTAC-----GAGGAAGA	420	-5	2.98%
CGAATTCTCTCTACGA--GGAGGAAGA	234	-2	1.66%
CGAATTCTCTCTACGA---GAGGAAGA	169	-3	1.20%
CGAATTCTCTCTACGAGAGGGGAGGAAGA	154	+2	1.09%
CGAATTCTCTCTAC---GGGAGGAAGA	75	-3	0.53%
CGAATTCTCTCTACG-GGGGAGGAAGA	65	-1	0.46%
CGAATTCTCTCTACGAAGGGGAGGAAGA	50	+1 Ins	0.36%

YW-W9-Syn78crBCL11A2b-KO(AAV)-12h **12h**	Ins	Del	HDR
16595 | 54.05%	**11.67%**	**42.39%**	**0.00%**
**Typical seqs**	**Reads**	**Type**	**pct.**
CGAATTCTCTCTACGAG|GGG**AGG**AAGA	7625	WT	45.95%
CGAATTCTCTCTACGA-GGGAGGAAGA	1962	-1	11.82%
CGAATTCTCTCTAC-----GAGGAAGA	1327	-5	8.00%
CGAATTCTCTCTACGAGgGGGAGGAAGA	749	+1 g	4.51%
CGAATTCTCTCTACGA--GGAGGAAGA	743	-2	4.48%
CGAATTCTCTCTACGA---GAGGAAGA	401	-3	2.42%
CGAATTCTCTCTACGAGAGGGGAGGAAGA	274	+2	1.65%
CGAATTCTCTCTACGAG----GGAAGA	116	-4	0.70%
CGAATTCTCTCTAC---GGGAGGAAGA	103	-3	0.62%
CGAATTCTCTCTACGAGGAATTCTCTACGA GGGAGGAAGA	102	+13	0.61%

YW-W9-Syn78crBCL11A2b-KO(AAV)-24h **24h**	Ins	Del	HDR
6464 | 42.33%	**8.43%**	**33.90%**	**0.00%**
**Typical seqs**	**Reads**	**Type**	**pct.**
CGAATTCTCTCTACGAG|GGG**AGG**AAGA	3728	WT	57.67%
CGAATTCTCTCTACGA-GGGAGGAAGA	933	-1	14.43%
CGAATTCTCTCTAC-----GAGGAAGA	250	-5	3.87%
CGAATTCTCTCTACGAGgGGGAGGAAGA	204	+1 g	3.16%
CGAATTCTCTCTACGAGAGGGGAGGAAGA	129	+2	2.00%
CGAATTCTCTCTACGA--GGAGGAAGA	127	-2	1.96%
CGAATTCTCTCTACGA---GAGGAAGA	100	-3	1.55%
CGAATTCTCTCTAC---GGGAGGAAGA	96	-3	1.49%
CGAATTCTCTCTACG-GGGGAGGAAGA	39	-1	0.60%
CGAATTCTCT-------GGGAGGAAGA	36	-7	0.56%

YW-W9-Syn78crBCL11A2b-KO(AAV)-48h **48h**	Ins	Del	HDR
31221 | 57.64%	**10.64%**	**47.00%**	**0.00%**
**Typical seqs**	**Reads**	**Type**	**pct.**
CGAATTCTCTCTACGAG|GGG**AGG**AAGA	13226	WT	42.36%
CGAATTCTCTCTACGA-GGGAGGAAGA	3750	-1	12.01%
CGAATTCTCTCTAC-----GAGGAAGA	2801	-5	8.97%
CGAATTCTCTCTACGAGgGGGAGGAAGA	1129	+1 g	3.62%
CGAATTCTCTCTACGA--GGAGGAAGA	942	-2	3.02%
CGAATTCTCTCTACGA---GAGGAAGA	796	-3	2.55%
CGAATTCTCTCTACGAGAGGGGAGGAAGA	307	+2	0.98%
CGAATTCTCTCTAC---GGGAGGAAGA	264	-3	0.85%
CGAATTCTCTC------GGGAGGAAGA	248	-6	0.79%
CGAATTCTCTCTACG---------AGA	223	-9	0.71%

P108-P115-YW-W9-Syn79crBCL11A-KO

YW-W9-Syn79crBCL11A2c-KO(AAV)-4h **4h**	Ins	Del	HDR
68550 | 22.80%	**18.17%**	**4.63%**	**0.00%**
**Typical seqs**	**Reads**	**Type**	**pct.**
TGTTGTGATTCCGAGCT|CCG**AGG**CGAG	52921	WT	77.20%
TGTTGTGATTCCGAGCTtCCGAGGCGAG	11953	+1 t	17.44%
TGTTGTGAT-------TCCGAGGCGAG	2265	-7	3.30%
TGTTGTGATTCCGAGCT--------AG	191	-8	0.28%
TGTTGTGATTCCGAGCTTTCCGAGGCGAG	157	+2	0.23%
TGTTGTG---------TCCGAGGCGAG	149	-9	0.22%
TGTTGTGATTCCGAGC-CCGAGGCGAG	104	-1	0.15%
TGTTGTGATTCCGAGCT-GGAGGCGAG	93	-1	0.14%
TGTTGTGATTCCGAG-TCCGAGGCGAG	90	-1	0.13%
TGTTGT-------------GAGGCGAG	95	-13	0.14%

YW-W9-Syn79crBCL11A2c-KO(AAV)-8h **8h**	Ins	Del	HDR
56558 | 43.40%	**29.09%**	**14.31%**	**0.00%**
**Typical seqs**	**Reads**	**Type**	**pct.**
TGTTGTGATTCCGAGCT|CCG**AGG**CGAG	32014	WT	56.60%
TGTTGTGATTCCGAGCTtCCGAGGCGAG	15380	+1 t	27.19%
TGTTGTGAT-------TCCGAGGCGAG	5485	-7	9.70%
TGTTGTGATTCCGA--------GCGAG	788	-8	1.39%
TGTTGTGATTCCGAG-TCCGAGGCGAG	231	-1	0.41%
TGTTGTGATTCCGAGCT--GAGGCGAG	189	-2	0.33%
TGTTGTGAT-------TCCGAGGCGAG	5485	-8	9.70%
TGTTGTGATTCCGAG--CCGAGGCGAG	136	-2	0.24%
TGTTGTGATTCCGAGCCTCCGAGGCGAG	129	+1 Ins	0.23%
TGTTGTGATTCCGAGCTCTCCGAGGCGAG	127	+2	0.22%

YW-W9-Syn79crBCL11A2c-KO(AAV)-12h **12h**	Ins	Del	HDR
16123 | 47.93%	**27.31%**	**20.62%**	**0.00%**
**Typical seqs**	**Reads**	**Type**	**pct.**
TGTTGTGATTCCGAGCT|CCG**AGG**CGAG	8396	WT	52.07%
TGTTGTGATTCCGAGCTtCCGAGGCGAG	4121	+1 t	25.56%
TGTTGTGAT-------TCCGAGGCGAG	1785	-7	11.07%
TGTTGTGATTCCGA--------GCGAG	252	-8	1.56%
TGTTGTGATTCCGAG-TCCGAGGCGAG	173	-1	1.07%
TGTTGTGATTCCGA--TCCGAGGCGAG	145	-2	0.90%
TGTTGTGATTCCGAGC-CCGAGGCGAG	87	-1	0.54%
TGTTGTGATTCCGA-CTCCGAGGCGAG	63	-1	0.39%
TGTTGTGATTCCGAGCTATCTAGCTATCTA GCAGTCACTATTCCGAGGCGAG	55	+25	0.34%
TGTTGTGATTCCGAG--CCGAGGCGAG	54	-2	0.33%

YW-W9-Syn79crBCL11A2c-KO(AAV)-24h **24h**	Ins	Del	HDR
31444 | 59.93%	**30.51%**	**29.41%**	**0.00%**
**Typical seqs**	**Reads**	**Type**	**pct.**
TGTTGTGATTCCGAGCT|CCG**AGG**CGAG	12601	WT	40.07%
TGTTGTGATTCCGAGCTtCCGAGGCGAG	8852	+1 t	28.15%
TGTTGTGAT-------TCCGAGGCGAG	5873	-7	18.68%
TGTTGTGATTCCGA--------GCGAG	536	-8	1.70%
TGTTGTGATTCCGAG--CCGAGGCGAG	199	-2	0.63%
TGTTGTGATTCCGA--TCCGAGGCGAG	188	-2	0.60%
TGTTGTGAT-------TCCGAGGCGAG	5873	-8	18.68%
TGTTGT----------TCCGAGGCGAG	151	-10	0.48%
TGTTGTGATTCCGAG-TCCGAGGCGAG	133	-1	0.42%
TGTTGTGATTCCGAG---CGAGGCGAG	110	-3	0.35%

YW-W9-Syn79crBCL11A2c-KO(AAV)-48h **48h**	Ins	Del	HDR
31182 | 66.96%	**28.49%**	**38.46%**	**0.00%**
**Typical seqs**	**Reads**	**Type**	**pct.**
TGTTGTGATTCCGAGCT|CCG**AGG**CGAG	10304	WT	33.04%
TGTTGTGAT-------TCCGAGGCGAG	8215	-7	26.35%
TGTTGTGATTCCGAGCTtCCGAGGCGAG	8083	+1 t	25.92%
TGTTGTGATTCCGA--------GCGAG	860	-8	2.76%
TGTTGTGATTCCGA--TCCGAGGCGAG	224	-2	0.72%
TGTTGTGATTCCGAG-TCCGAGGCGAG	224	-1	0.72%
TGTTGTGATTCCGAGC-CCGAGGCGAG	181	-1	0.58%
TGTTGTGAT-------TCCGAGGCGAG	8215	-8	26.35%
TGTTGTGATTC------------CGAG	157	-12	0.50%
TGTTGTGATTCCGAGCTCTCCGAGGCGAG	152	+2	0.49%

P108-P115-YW-W9-Syn83crBCL11A-KO

YW-W9-Syn83crBCL11A4b-KO(AAV)-4h 4h	Ins	Del	HDR
150738 | 12.87%	2.42%	10.45%	0.00%
Typical seqs	Reads	Type	pct.
ACTTCATGCGGAGGCCC|CGT**GGG**AGGA	131334	WT	87.13%
ACTTCATGCGGAGG-CCCGTGGGAGGA	11622	-1	7.71%
ACTTCATGCGGAGGCCCcCGTGGGAGGA	2047	+1 c	1.36%
ACTTCATGCGGAGG---CGTGGGAGGA	562	-3	0.37%
ACTTCATGC------------GGAGGA	478	-12	0.32%
ACTTCATGCGGAGGCCCtCGTGGGAGGA	461	+1 t	0.31%
ACTTCA-------------TGGGAGGA	400	-13	0.27%
ACTTCATGCGGAGGCGCTCCTACTTCGTGG GAGGA	261	+8	0.17%
ACTTCATGCGGAGGCCCTCCGCATGCGGA	252	+2	0.17%
ACTT-------------CGTGGGAGGA	243	-13	0.16%

YW-W9-Syn83crBCL11A4b-KO(AAV)-8h 8h	Ins	Del	HDR
111765 | 38.31%	6.34%	31.97%	0.00%
Typical seqs	Reads	Type	pct.
ACTTCATGCGGAGGCCC|CGT**GGG**AGGA	68947	WT	61.69%
ACTTCATGCGGAGG-CCCGTGGGAGGA	15081	-1	13.49%
ACTTCATG---------CGTGGGAGGA	3517	-9	3.15%
ACTTCATGC------------GGAGGA	2880	-12	2.58%
ACTTCATGCGGAGG---CGTGGGAGGA	2730	-3	2.44%
ACTTCATGCGGAGGCCCcCGTGGGAGGA	2596	+1 c	2.32%
ACTTCATGCGGAGG--CCGTGGGAGGA	2097	-2	1.88%
ACTT-------------CGTGGGAGGA	881	-13	0.79%
ACTTCAT-----------GTGGGAGGA	890	-11	0.80%
ACTTCATGCGGAGGCCC-----GAGGA	592	-5	0.53%

YW-W9-Syn83crBCL11A4b-KO(AAV)-12h 12h	Ins	Del	HDR
99117 | 53.17%	8.31%	44.86%	0.00%
Typical seqs	Reads	Type	pct.
ACTTCATGCGGAGGCCC|CGT**GGG**AGGA	46413	WT	46.83%
ACTTCATGCGGAGG-CCCGTGGGAGGA	15739	-1	15.88%
ACTTCATG---------CGTGGGAGGA	4075	-9	4.11%
ACTTCATGC------------GGAGGA	3978	-12	4.01%
ACTTCATGCGGAGGCCCcCGTGGGAGGA	3014	+1 c	3.04%
ACTTCATGCGGAGG---CGTGGGAGGA	2834	-3	2.86%
ACTTCATGCGGAGG--CCGTGGGAGGA	2063	-2	2.08%
ACTT-------------CGTGGGAGGA	1005	-13	1.01%
ACTTCAT-----------GTGGGAGGA	909	-11	0.92%
ACTTCATGCGGAGGCCC----GGAGGA	769	-4	0.78%

YW-W9-Syn83crBCL11A4b-KO(AAV)-24h 24h	Ins	Del	HDR
46765 | 68.43%	10.86%	57.57%	0.00%
Typical seqs	Reads	Type	pct.
ACTTCATGCGGAGGCCC|CGT**GGG**AGGA	14762	WT	31.57%
ACTTCATGCGGAGG-CCCGTGGGAGGA	8518	-1	18.21%
ACTTCATG---------CGTGGGAGGA	2265	-9	4.84%
ACTTCATGC------------GGAGGA	1961	-12	4.19%
ACTTCATGCGGAGG--CCGTGGGAGGA	1874	-2	4.01%
ACTTCATGCGGAGGCCCcCGTGGGAGGA	1799	+1 c	3.85%
ACTTCATGCGGAGG---CGTGGGAGGA	1318	-3	2.82%
ACTT-------------CGTGGGAGGA	912	-13	1.95%
ACTTCATGCGGAGGCCCaCGTGGGAGGA	595	+1 a	1.27%
ACTTCATGCGGAGGCCC-----GAGGA	573	-5	1.23%

YW-W9-Syn83crBCL11A4b-KO(AAV)-48h 48h	Ins	Del	HDR
49405 | 70.23%	11.35%	58.88%	0.00%
Typical seqs	Reads	Type	pct.
ACTTCATGCGGAGGCCC|CGT**GGG**AGGA	14709	WT	29.77%
ACTTCATGCGGAGG-CCCGTGGGAGGA	8648	-1	17.50%
ACTTCATGC------------GGAGGA	3855	-12	7.80%
ACTTCATG---------CGTGGGAGGA	2476	-9	5.01%
ACTTCATGCGGAGGCCCcCGTGGGAGGA	1893	+1 c	3.83%
ACTTCATGCGGAGG--CCGTGGGAGGA	1365	-2	2.76%
ACTTCATGCGGAGG---CGTGGGAGGA	1193	-3	2.41%
ACTTCA-------------TGGGAGGA	844	-13	1.71%
ACTT-------------CGTGGGAGGA	752	-13	1.52%
ACTTCAT-----------GTGGGAGGA	662	-11	1.34%

P108-P115-YW-W9-Syn83crBCL11A-AAVKI

YW-W9-Syn83crBCL11A4b-AAV6KI-4h **4h**	Ins	Del	HDR
159418 | 14.67%	2.07%	11.58%	1.02%
**Typical seqs**	**Reads**	**Type**	**pct.**
ACTTCATGCGGAGGCCC|CGT**GGG**AGGA	136031	WT	85.33%
ACTTCATGCGGAGG-CCCGTGGGAGGA	10473	-1	6.57%
ACTTCATGCGGAGGCCCcCGTGGGAGGA	1787	+1 c	1.12%
ACTTCATGCGGAGGCCCGTTTAAACCGTGGGAGGA	1586	+8 HDR	0.99%
ACTTCATGCGGAGG--CCGTGGGAGGA	1222	-2	0.77%
ACTTCATGC------------GGAGGA	1077	-12	0.68%
ACTTCATGCGGAGG---CGTGGGAGGA	958	-3	0.60%
ACTTCATGCGGAGGCCC--TGGGAGGA	890	-2	0.56%
ACTTCATG---------CGTGGGAGGA	753	-9	0.47%
ACTTCATGCGGAG-----GTGGGAGGA	385	-5	0.24%

YW-W9-Syn83crBCL11A4b-AAV6KI-8h **8h**	Ins	Del	HDR
102920 | 48.37%	5.68%	26.04%	16.64%
**Typical seqs**	**Reads**	**Type**	**pct.**
ACTTCATGCGGAGGCCC|CGT**GGG**AGGA	53138	WT	51.63%
ACTTCATGCGGAGGCCCGTTTAAACCGTGGGAGGA	16234	+8 HDR	15.77%
ACTTCATGCGGAGG-CCCGTGGGAGGA	14248	-1	13.84%
ACTTCATGCGGAGGCCCcCGTGGGAGGA	2512	+1 c	2.44%
ACTTCATG---------CGTGGGAGGA	2252	-9	2.19%
ACTTCATGCGGAGG--CCGTGGGAGGA	1871	-2	1.82%
ACTTCATGC------------GGAGGA	803	-12	0.78%
ACTTCATGCGGAGGCCCaCGTGGGAGGA	742	+1 a	0.72%
ACTT-------------CGTGGGAGGA	640	-13	0.62%
ACTTCATGCGGAGGCCCtCGTGGGAGGA	600	+1 t	0.58%

YW-W9-Syn83crBCL11A4b-AAV6KI-12h **12h**	Ins	Del	HDR
88040 | 69.00%	5.86%	33.41%	29.73%
**Typical seqs**	**Reads**	**Type**	**pct.**
ACTTCATGCGGAGGCCC|CGT**GGG**AGGA	27292	WT	31.00%
ACTTCATGCGGAGGCCCGTTTAAACCGTGGGAGGA	24839	+8 HDR	28.21%
ACTTCATGCGGAGG-CCCGTGGGAGGA	11511	-1	13.07%
ACTTCATG---------CGTGGGAGGA	3921	-9	4.45%
ACTTCATGCGGAGGCCCcCGTGGGAGGA	2409	+1 c	2.74%
ACTTCATGCGGAGG--CCGTGGGAGGA	1955	-2	2.22%
ACTTCATGC------------GGAGGA	1891	-12	2.15%
ACTTCATGCGGAGG---CGTGGGAGGA	1005	-3	1.14%
ACTT-------------CGTGGGAGGA	516	-13	0.59%
ACTTCAT-----------GTGGGAGGA	504	-11	0.57%

YW-W9-Syn83crBCL11A4b-AAV6KI-24h-BC1 **24h**	Ins	Del	HDR
43387 | 81.37%	7.20%	39.59%	34.58%
**Typical seqs**	**Reads**	**Type**	**pct.**
ACTTCATGCGGAGGCCC|CGT**GGG**AGGA	8084	WT	18.63%
ACTTCATGCGGAGGCCCGTTTAAACCGTGGGAGGA	14453	+8 HDR	33.31%
ACTTCATGCGGAGG-CCCGTGGGAGGA	5976	-1	13.77%
ACTTCATG---------CGTGGGAGGA	1998	-9	4.61%
ACTTCATGC------------GGAGGA	1178	-12	2.72%
ACTTCATGCGGAGGCCCcCGTGGGAGGA	1040	+1 c	2.40%
ACTTCATGCGGAGG--CCGTGGGAGGA	782	-2	1.80%
ACTTCATGCGGAGG---CGTGGGAGGA	549	-3	1.27%
ACTTCA-----------CGTGGGAGGA	501	-11	1.15%
ACTTCAT-----------GTGGGAGGA	446	-11	1.03%

YW-W9-Syn83crBCL11A4b-AAV6KI-48h-BC2 **48h**	Ins	Del	HDR
42280 | 22.99%	0.14%	15.22%	7.63%
**Typical seqs**	**Reads**	**Type**	**pct.**
ACTTCATGCGGAGGCCC|CGT**GGG**AGGA	32561	WT	77.01%
ACTTCATGCGGAGGCCCGTTTAAACCGTGGGAGGA	3147	+8 HDR	7.44%
ACTTCATGCGGAGG-CCCGTGGGAGGA	1338	-1	3.16%
ACTTCA---------CTCGTGGGAGGA	740	-9	1.75%
ACTTCATG---------CGTGGGAGGA	734	-9	1.74%
ACTTCATGC------------GGAGGA	731	-12	1.73%
A--------------CACGTGGGAGGA	707	-14	1.67%
ACTTCATGCGGAGG---CGTGGGAGGA	675	-3	1.60%
ACTTCATGCGGAGGCCC-----GAGGA	638	-5	1.51%
ACTTCATGCGG------CGTGGGAGGA	634	-6	1.50%

P108-P115-YW-W9-Syn87crBCL11A-KO

YW-W9-Syn87crBCL11A5b-KO(AAV)-4h 4h	Ins	Del	HDR
149043 | 28.50%	10.44%	18.05%	0.00%
**Typical seqs**	**Reads**	**Type**	**pct.**
GGGATTACCGAGTCACC|ACC**AGG**CTGC	106573	WT	71.50%
GGGATTACCGAGT---CACCAGGCTGC	14212	-3	9.54%
GGGATTACCGAGTCACCcACCAGGCTGC	14025	+1 c	9.41%
GGGATTACCGAGTCA-CACCAGGCTGC	9416	-1	6.32%
GGGATTACCGAGTCA--ACCAGGCTGC	827	-2	0.55%
GGGATTACCGAGTCACC-CCAGGCTGC	391	-1	0.26%
GGGATTACCGAGTCACCtACCAGGCTGC	353	+1 t	0.24%
GGGATTACCGAGTCACCCCACCAGGCTGC	294	+2	0.20%
GGGATT-----------ACCAGGCTGC	208	-11	0.14%
GGGATTACCGAGTCACC----------	158	-14	0.11%

YW-W9-Syn87crBCL11A5b-KO(AAV)-8h 8h	Ins	Del	HDR
144747 | 53.50%	18.05%	35.45%	0.00%
**Typical seqs**	**Reads**	**Type**	**pct.**
GGGATTACCGAGTCACC|ACC**AGG**CTGC	67308	WT	46.50%
GGGATTACCGAGT---CACCAGGCTGC	23067	-3	15.94%
GGGATTACCGAGTCACCcACCAGGCTGC	22534	+1 c	15.57%
GGGATTACCGAGTCA-CACCAGGCTGC	16595	-1	11.46%
GGGATT-----------ACCAGGCTGC	3292	-11	2.27%
GGGATTACCGAGTCACCCCACCAGGCTGC	1345	+2	0.93%
GGGATTACCGAGTCA--ACCAGGCTGC	926	-2	0.64%
GGGATTACCGAGT------CAGGCTGC	895	-6	0.62%
GGGATTACCGAGTCACC-CCAGGCTGC	692	-1	0.48%
GGGATTACCGAGTCAC--CCAGGCTGC	344	-2	0.24%

YW-W9-Syn87crBCL11A5b-KO(AAV)-12h 12h	Ins	Del	HDR
156254 | 63.34%	21.74%	41.60%	0.00%
**Typical seqs**	**Reads**	**Type**	**pct.**
GGGATTACCGAGTCACC|ACC**AGG**CTGC	57281	WT	36.66%
GGGATTACCGAGT---CACCAGGCTGC	29940	-3	19.16%
GGGATTACCGAGTCACCcACCAGGCTGC	28263	+1 c	18.09%
GGGATTACCGAGTCA-CACCAGGCTGC	20242	-1	12.95%
GGGATT-----------ACCAGGCTGC	3773	-11	2.41%
GGGATTACCGAGTCACCCCACCAGGCTGC	2004	+2	1.28%
GGGATTACCGAGT------CAGGCTGC	891	-6	0.57%
GGGATTACCGAGTCACC-CCAGGCTGC	874	-1	0.56%
GGGATTACCGAGTCACCtACCAGGCTGC	723	+1 t	0.46%
GGGATTACCGAGT--CCACCAGGCTGC	468	-2	0.30%

YW-W9-Syn87crBCL11A5b-KO(AAV)-24h 24h	Ins	Del	HDR
92939 | 75.45%	23.03%	52.42%	0.00%
**Typical seqs**	**Reads**	**Type**	**pct.**
GGGATTACCGAGTCACC|ACC**AGG**CTGC	22815	WT	24.55%
GGGATTACCGAGT---CACCAGGCTGC	22980	-3	24.73%
GGGATTACCGAGTCACCcACCAGGCTGC	17717	+1 c	19.06%
GGGATTACCGAGTCA-CACCAGGCTGC	14022	-1	15.09%
GGGATT-----------ACCAGGCTGC	3427	-11	3.69%
GGGATTACCGAGTCACCCCACCAGGCTGC	1125	+2	1.21%
GGGATTACCGAGT------CAGGCTGC	749	-6	0.81%
GGGATTACCGAGTCA--ACCAGGCTGC	622	-2	0.67%
GGGATTACCGAGTCACCtACCAGGCTGC	565	+1 t	0.61%
GGGATTACCGAGTCACCaACCAGGCTGC	362	+1 a	0.39%

YW-W9-Syn87crBCL11A5b-KO(AAV)-48h 48h	Ins	Del	HDR
130793 | 82.70%	22.45%	60.26%	0.00%
**Typical seqs**	**Reads**	**Type**	**pct.**
GGGATTACCGAGTCACC|ACC**AGG**CTGC	22622	WT	17.30%
GGGATTACCGAGT---CACCAGGCTGC	37870	-3	28.95%
GGGATTACCGAGTCACCcACCAGGCTGC	23803	+1 c	18.20%
GGGATTACCGAGTCA-CACCAGGCTGC	20550	-1	15.71%
GGGATT-----------ACCAGGCTGC	6573	-11	5.03%
GGGATTACCGAGT------CAGGCTGC	1739	-6	1.33%
GGGATTACCGAGTCACCCCACCAGGCTGC	1696	+2	1.30%
GGGATTACCG----------AGGCTGC	516	-10	0.39%
GGGATTACCGAGTCACC-CCAGGCTGC	496	-1	0.38%
GGGATTACCGAGTCACCtACCAGGCTGC	488	+1 t	0.37%

P108-P115-YW-W9-Syn87crBCL11A-AAVKI

YW-W9-Syn87crBCL11A5b-AAV6KI-4h **4h**	Ins	Del	HDR
111817 | 29.15%	**11.05%**	**17.99%**	**0.11%**
**Typical seqs**	**Reads**	**Type**	**pct.**
GGGATTACCGAGTCACC|ACC**AGG**CTGC	79224	WT	70.85%
GGGATTACCGAGT---CACCAGGCTGC	11890	-3	10.63%
GGGATTACCGAGTCACCcACCAGGCTGC	10807	+1 c	9.66%
GGGATTACCGAGTCA-CACCAGGCTGC	6389	-1	5.71%
GGGATTACCGAGTCACCCCACCAGGCTGC	866	+2	0.77%
GGGATTACCGAGTCACC-CCAGGCTGC	343	-1	0.31%
GGGATTACCGAGTCACC----------	141	-13	0.13%
GGGATTACTGAGTCACCCACCAGGCTGC	138	+1 Ins	0.12%
GGGATTGGGGATT----ACCAGGCTGC	128	-4	0.11%
GGGATTACCG------CACCAGGCTGC	121	-6	0.11%

YW-W9-Syn87crBCL11A5b-AAV6KI-8h **8h**	Ins	Del	HDR
123901 | 58.13%	**21.69%**	**29.48%**	**6.96%**
**Typical seqs**	**Reads**	**Type**	**pct.**
GGGATTACCGAGTCACC|ACC**AGG**CTGC	51876	WT	41.87%
GGGATTACCGAGTCACCcACCAGGCTGC	23484	+1 c	18.95%
GGGATTACCGAGT---CACCAGGCTGC	19194	-3	15.49%
GGGATTACCGAGTCA-CACCAGGCTGC	12731	-1	10.28%
GGGATTACCGAGTCACCGTTTAAACACCAG GCTGC	8324	+8 HDR	6.72%
GGGATTACCGAGTCACCCCACCAGGCTGC	1651	+2	1.33%
GGGATTACCGAGTCA--ACCAGGCTGC	692	-2	0.56%
GGGATT-----------ACCAGGCTGC	515	-11	0.42%
GGGATTACCGAGTCAC--CCAGGCTGC	516	-2	0.42%
GGGATTACCGAGTCACCgACCAGGCTGC	259	+1 g	0.21%

YW-W9-Syn87crBCL11A5b-AAV6KI-12h **12h**	Ins	Del	HDR
93584 | 70.35%	**21.20%**	**35.39%**	**13.75%**
**Typical seqs**	**Reads**	**Type**	**pct.**
GGGATTACCGAGTCACC|ACC**AGG**CTGC	27752	WT	29.65%
GGGATTACCGAGTCACCcACCAGGCTGC	17003	+1 c	18.17%
GGGATTACCGAGT---CACCAGGCTGC	16341	-3	17.46%
GGGATTACCGAGTCACCGTTTAAACACCAG GCTGC	12364	+8 HDR	13.21%
GGGATTACCGAGTCA-CACCAGGCTGC	10589	-1	11.31%
GGGATT-----------ACCAGGCTGC	1003	-11	1.07%
GGGATTACCGAGTCACCCCACCAGGCTGC	996	+2	1.06%
GGGATTACCGAGTCA--ACCAGGCTGC	433	-2	0.46%
GGGATTACCGAGTCACC-CCAGGCTGC	368	-1	0.39%
GGGATTACCGAGTCACCtACCAGGCTGC	364	+1 t	0.39%

YW-W9-Syn87crBCL11A5b-AAV6KI-24h-BC1 **24h**	Ins	Del	HDR
45523 | 85.93%	**22.33%**	**45.70%**	**17.91%**
**Typical seqs**	**Reads**	**Type**	**pct.**
GGGATTACCGAGTCACC|ACC**AGG**CTGC	6405	WT	14.07%
GGGATTACCGAGT---CACCAGGCTGC	10789	-3	23.70%
GGGATTACCGAGTCACCcACCAGGCTGC	8624	+1 c	18.94%
GGGATTACCGAGTCACCGTTTAAACACCAG GCTGC	7860	+8 HDR	17.27%
GGGATTACCGAGTCA-CACCAGGCTGC	5308	-1	11.66%
GGGATT-----------ACCAGGCTGC	1083	-11	2.38%
GGGATTACCGAGTCACCCCACCAGGCTGC	380	+2	0.83%
GGGATTACCGAGT------CAGGCTGC	213	-6	0.47%
GGGATTACCG----------AGGCTGC	197	-10	0.43%
GGGATTACCGAGTCACC-CCAGGCTGC	199	-1	0.44%

YW-W9-Syn87crBCL11A5b-AAV6KI-48h-BC2 **48h**	Ins	Del	HDR
61222 | 89.88%	**19.49%**	**50.73%**	**19.66%**
**Typical seqs**	**Reads**	**Type**	**pct.**
GGGATTACCGAGTCACC|ACC**AGG**CTGC	6195	WT	10.12%
GGGATTACCGAGT---CACCAGGCTGC	15325	-3	25.03%
GGGATTACCGAGTCACCGTTTAAACACCAG GCTGC	11534	+8 HDR	18.84%
GGGATTACCGAGTCACCcACCAGGCTGC	10051	+1 c	16.42%
GGGATTACCGAGTCA-CACCAGGCTGC	9457	-1	15.45%
GGGATT-----------ACCAGGCTGC	1390	-11	2.27%
GGGATTACCGAGTCACCCCACCAGGCTGC	474	+2	0.77%
GGGATTACCGAGTCACC-CCAGGCTGC	294	-1	0.48%
GGGATTACCGAGT----------CTGC	283	-10	0.46%
GGGATTACCGAGTCACCtACCAGGCTGC	235	+1 t	0.38%

P108-P115-YW-W9-Syn92crBCL11A-KO

4h

YW-W9-Syn92crBCL11A7b-KO(AAV)-4h	Ins	Del	HDR
175339 | 30.29%	26.39%	3.90%	0.00%
**Typical seqs**	**Reads**	**Type**	**pct.**
GCTTCGTGACGTTGGAT|GGA**GGG**TTTT	122224	WT	69.71%
GCTTCGTGACGTTGGATtGGAGGGTTTT	42468	+1 t	24.22%
GCTTCGTGACGT----TGGAGGGTTTT	3086	-4	1.76%
GCTTCGTGACGTTGGATATGGAGGGTTTT	1257	+2	0.72%
GCTTCGTGACGTTGGAT-GAGGGTTTT	1189	-1	0.68%
GCTTCGTGACGTTGG-TGGAGGGTTTT	847	-1	0.48%
GCTTCGTGACGTTGGA-GGAGGGTTTT	646	-1	0.37%
GCTTCGTGACGTTG--TGGAGGGTTTT	641	-2	0.37%
GCTTCGTGACGTT--------GGTTTT	243	-8	0.14%
GCTTCGTGACGTTGGATGTTGGAGGGTTTT	212	+3	0.12%

8h

YW-W9-Syn92crBCL11A7b-KO(AAV)-8h	Ins	Del	HDR
186451 | 54.95%	46.13%	8.82%	0.00%
**Typical seqs**	**Reads**	**Type**	**pct.**
GCTTCGTGACGTTGGAT|GGA**GGG**TTTT	83988	WT	45.05%
GCTTCGTGACGTTGGATtGGAGGGTTTT	79347	+1 t	42.56%
GCTTCGTGACGT----TGGAGGGTTTT	9586	-4	5.14%
GCTTCGTGACGTTGGAT-GAGGGTTTT	1602	-1	0.86%
GCTTCGTGAC-------GGAGGGTTTT	745	-7	0.40%
GCTTCGTGACGTTGGATTTGGAGGGTTTT	572	+2	0.31%
GCTTC------------GGAGGGTTTT	432	-12	0.23%
GCTTCGTGACGTTGGATATGGAGGGTTTT	414	+2	0.22%
GCTTCGTGACGTT--------GGTTTT	408	-8	0.22%
GCTTCGTGACGTTGG-TGGAGGGTTTT	396	-1	0.21%

12h

YW-W9-Syn92crBCL11A7b-KO(AAV)-12h	Ins	Del	HDR
209489 | 63.17%	51.90%	11.27%	0.00%
**Typical seqs**	**Reads**	**Type**	**pct.**
GCTTCGTGACGTTGGAT|GGA**GGG**TTTT	77156	WT	36.83%
GCTTCGTGACGTTGGATtGGAGGGTTTT	99541	+1 t	47.52%
GCTTCGTGACGT----TGGAGGGTTTT	10387	-4	4.96%
GCTTCGTGACGTTGGA-GGAGGGTTTT	1648	-1	0.79%
GCTTCGTGACGTTGG-TGGAGGGTTTT	1018	-1	0.49%
GCTTCGTGACGTTGGATATGGAGGGTTTT	963	+2	0.46%
GCTTCGTGACGTTGGAT-GAGGGTTTT	740	-1	0.35%
GCTTCGTGAC------------GTTTT	692	-12	0.33%
GCTTCGTGACGTTGGATTTGGAGGGTTTT	662	+2	0.32%
GCTTCGTGACGTTGGAT--AGGGTTTT	503	-2	0.24%

24h

YW-W9-Syn92crBCL11A7b-KO(AAV)-24h	Ins	Del	HDR
104307 | 74.29%	57.25%	17.04%	0.00%
**Typical seqs**	**Reads**	**Type**	**pct.**
GCTTCGTGACGTTGGAT|GGA**GGG**TTTT	26820	WT	25.71%
GCTTCGTGACGTTGGATtGGAGGGTTTT	54460	+1 t	52.21%
GCTTCGTGACGT----TGGAGGGTTTT	8512	-4	8.16%
GCTTCGTGACGTTGGATATGGAGGGTTTT	813	+2	0.78%
GCTTCGTGACGTTGGA-GGAGGGTTTT	782	-1	0.75%
GCTTCGTGACGTT--------GGTTTT	639	-8	0.61%
GCTTCGTGACGTTGGAT-GAGGGTTTT	562	-1	0.54%
GCTTCGTGACGTT-------GGGTTTT	525	-7	0.50%
GCTTCGTGACGTTGGA--------TTT	443	-8	0.42%
GCTTCGTGACGTTGG-TGGAGGGTTTT	390	-1	0.37%

48h

YW-W9-Syn92crBCL11A7b-KO(AAV)-48h	Ins	Del	HDR
128713 | 30.89%	27.81%	2.66%	0.42%
**Typical seqs**	**Reads**	**Type**	**pct.**
GCTTCGTGACGTTGGAT|GGA**GGG**TTTT	88951	WT	69.11%
GCTTCGTGACGTTGGATtGGAGGGTTTT	33036	+1 t	25.67%
GCTTCGTGACGT----TGGAGGGTTTT	1181	-4	0.92%
GCTTCGTGACGTTGGA-GGAGGGTTTT	1039	-1	0.81%
GCTTCGTGACGTTGGATGTTTAAACGGAGG GTTTT	524	+8 HDR	0.41%
GCTTCGTGACGTTGGAT-GAGGGTTTT	516	-1	0.40%
GCTTCGTGACGTTGGATATGGAGGGTTTT	493	+2	0.38%
GCTTCGTGACGTTG---GGAGGGTTTT	241	-3	0.19%
GCTTCGTGACGTTGGATTTGGAGGGTTTT	229	+2	0.18%
GCTTCG----------TGGAGGGTTTT	153	-10	0.12%

P108-P115-YW-W9-Syn92crBCL11A-AAVKI

YW-W9-Syn92crBCL11A7b-AAV6KI-4h **4h**	Ins	Del	HDR
128713 | 30.89%	**27.81%**	**2.66%**	**0.42%**
**Typical seqs**	**Reads**	**Type**	**pct.**
GCTTCGTGACGTTGGAT|GGA**GGG**TTTT	88951	WT	69.11%
GCTTCGTGACGTTGGATtGGAGGGTTTT	33036	+1 t	25.67%
GCTTCGTGACGT----TGGAGGGTTTT	1181	-4	0.92%
GCTTCGTGACGTTGGA-GGAGGGTTTT	1039	-1	0.81%
GCTTCGTGACGTTGGATGTTTAAACGGAGG GTTTT	524	+8 HDR	0.41%
GCTTCGTGACGTTGGAT-GAGGGTTTT	516	-1	0.40%
GCTTCGTGACGTTGGATATGGAGGGTTTT	493	+2	0.38%
GCTTCGTGACGTTG---GGAGGGTTTT	241	-3	0.19%
GCTTCGTGACGTTGGATTTGGAGGGTTTT	229	+2	0.18%
GCTTCG----------TGGAGGGTTTT	153	-10	0.12%

YW-W9-Syn92crBCL11A7b-AAV6KI-8h **8h**	Ins	Del	HDR
146315 | 62.20%	**48.74%**	**9.29%**	**4.17%**
**Typical seqs**	**Reads**	**Type**	**pct.**
GCTTCGTGACGTTGGAT|GGA**GGG**TTTT	55312	WT	37.80%
GCTTCGTGACGTTGGATtGGAGGGTTTT	65780	+1 t	44.96%
GCTTCGTGACGTTGGATGTTTAAACGGAGG GTTTT	5780	+8 HDR	3.95%
GCTTCGTGACGT----TGGAGGGTTTT	4783	-4	3.27%
GCTTCGTGACGTTGGA-GGAGGGTTTT	1319	-1	0.90%
GCTTCGTGACGTTGGAT-GAGGGTTTT	1274	-1	0.87%
GCTTCGTGACGTTGGA-------TTTT	683	-7	0.47%
GCTTCGTGACGTTGGATATGGAGGGTTTT	623	+2	0.43%
GCTTCGTGACGTTGG-TGGAGGGTTTT	531	-1	0.36%
GCTTCGTGACGTT--------GGTTTT	416	-8	0.28%

YW-W9-Syn92crBCL11A7b-AAV6KI-12h **12h**	Ins	Del	HDR
110684 | 76.02%	**55.20%**	**8.76%**	**12.06%**
**Typical seqs**	**Reads**	**Type**	**pct.**
GCTTCGTGACGTTGGAT|GGA**GGG**TTTT	26542	WT	23.98%
GCTTCGTGACGTTGGATtGGAGGGTTTT	56164	+1 t	50.74%
GCTTCGTGACGTTGGATGTTTAAACGGAGG GTTTT	12459	+8 HDR	11.26%
GCTTCGTGACGT----TGGAGGGTTTT	4066	-4	3.67%
GCTTCGTGACGTTGGAT-GAGGGTTTT	818	-1	0.74%
GCTTCGTGACGTTGGATATGGAGGGTTTT	591	+2	0.53%
GCTTCGTGACGTTGGA-GGAGGGTTTT	559	-1	0.51%
GCTTCGTGACGTTGGA-------TTTT	410	-7	0.37%
GCTTCGTGACGTTGG-TGGAGGGTTTT	372	-1	0.34%
GCTTCG----------TGGAGGGTTTT	336	-10	0.30%

YW-W9-Syn92crBCL11A7b-AAV6KI-24h-BC1 **24h**	Ins	Del	HDR
57658 | 84.97%	**55.79%**	**12.53%**	**16.66%**
**Typical seqs**	**Reads**	**Type**	**pct.**
GCTTCGTGACGTTGGAT|GGA**GGG**TTTT	8664	WT	15.03%
GCTTCGTGACGTTGGATtGGAGGGTTTT	29229	+1 t	50.69%
GCTTCGTGACGTTGGATGTTTAAACGGAGG GTTTT	9084	+8 HDR	15.75%
GCTTCGTGACGT----TGGAGGGTTTT	2808	-4	4.87%
GCTTCGTGACGTTGGAT-GAGGGTTTT	683	-1	1.18%
GCTTCGTGACGTTGGA-GGAGGGTTTT	680	-1	1.18%
GCTTCGTGACGTTGGATATGGAGGGTTTT	478	+2	0.83%
GCTTCGTGACGTTGG-TGGAGGGTTTT	423	-1	0.73%
GCTTCG----------TGGAGGGTTTT	326	-10	0.57%
GCTTCGTGAC-------GGAGGGTTTT	214	-7	0.37%

YW-W9-Syn92crBCL11A7b-AAV6KI-48h-BC2 **48h**	Ins	Del	HDR
53686 | 86.17%	**51.75%**	**14.32%**	**20.10%**
**Typical seqs**	**Reads**	**Type**	**pct.**
GCTTCGTGACGTTGGAT|GGA**GGG**TTTT	7427	WT	13.83%
GCTTCGTGACGTTGGATtGGAGGGTTTT	25673	+1 t	47.82%
GCTTCGTGACGTTGGATGTTTAAACGGAGG GTTTT	10228	+8 HDR	19.05%
GCTTCGTGACGT----TGGAGGGTTTT	3354	-4	6.25%
GCTTCGTGACGTTGGA-GGAGGGTTTT	505	-1	0.94%
GCTTCGTGACGTTGGAT-GAGGGTTTT	271	-1	0.50%
GCTTCGTGACGTTGGA-------TTTT	245	-7	0.46%
GCTTCG----------TGGAGGGTTTT	187	-10	0.35%
GCTTCGTGACGTTGGATATGGAGGGTTTT	173	+2	0.32%
GCTTCGTGACGTT-------GGGTTTT	167	-7	0.31%

P108-P115-YW-W9-Syn94crBCL11A-KO

YW-W9-Syn94crBCL11A7d-KO(AAV)-4h **4h**	Ins	Del	HDR
100819 | 14.50%	**12.95%**	**1.55%**	**0.00%**
**Typical seqs**	**Reads**	**Type**	**pct.**
GCGTAGTGTTGGGTCCT|ACC**TGG**CCAC	86201	WT	85.50%
GCGTAGTGTTGGGTCCTtACCTGGCCAC	11699	+1 t	11.60%
GCGTAGTGTTGGGTCCTCTACCTGGCCAC	507	+2	0.50%
GCGTAGTGTTGGGT----CCTGGCCAC	431	-4	0.43%
GCGTAGTGTTGGGT-CTACCTGGCCAC	421	-1	0.42%
GCGTAGTGTTGGGTCCTaACCTGGCCAC	165	+1 a	0.16%
GCGTAGTGTTGGGTCCTATACCTGGCCAC	113	+2	0.11%
GCGTAGTGTTGGGTCC-ACCTGGCCAC	88	-1	0.09%
GCGTAGTGTTGGGTC--ACCTGGCCAC	84	-2	0.08%
GCGTAGTGTTGGGTC---CCTGGCCAC	84	-3	0.08%

YW-W9-Syn94crBCL11A7d-KO(AAV)-8h **8h**	Ins	Del	HDR
105066 | 36.83%	**29.08%**	**7.74%**	**0.00%**
**Typical seqs**	**Reads**	**Type**	**pct.**
GCGTAGTGTTGGGTCCT|ACC**TGG**CCAC	66374	WT	63.17%
GCGTAGTGTTGGGTCCTtACCTGGCCAC	26623	+1 t	25.34%
GCGTAGTGTTGGGT----CCTGGCCAC	2072	-4	1.97%
GCGTAGTGTTGGGTCCTCTACCTGGCCAC	1558	+2	1.48%
GCGTAGTGTTGGGT-CTACCTGGCCAC	862	-1	0.82%
GCGTAGTGTTGGGTCC-ACCTGGCCAC	841	-1	0.80%
GCGTAGTGTTGGGTCCT-CCTGGCCAC	555	-1	0.53%
GCGTAGTGTTGGG---TACCTGGCCAC	547	-3	0.52%
GCGTAGTGTTGGGT----------CAC	411	-10	0.39%
GCGTAGTGT-----------TGGCCAC	331	-11	0.32%

YW-W9-Syn94crBCL11A7d-KO(AAV)-12h **12h**	Ins	Del	HDR
112677 | 46.88%	**37.20%**	**9.67%**	**0.00%**
**Typical seqs**	**Reads**	**Type**	**pct.**
GCGTAGTGTTGGGTCCT|ACC**TGG**CCAC	59858	WT	53.12%
GCGTAGTGTTGGGTCCTtACCTGGCCAC	35498	+1 t	31.50%
GCGTAGTGTTGGGTCCTCTACCTGGCCAC	2523	+2	2.24%
GCGTAGTGTTGGGT----CCTGGCCAC	1437	-4	1.28%
GCGTAGTGTTGGGT-CTACCTGGCCAC	1122	-1	1.00%
GCGTAGTGTTGGGTC--ACCTGGCCAC	873	-2	0.77%
GCGTAGTGTTGGGTCC-ACCTGGCCAC	807	-1	0.72%
GCGTAGTGTTGGGTC-----------C	580	-11	0.51%
GCGTAGTGTTGGG---TACCTGGCCAC	552	-3	0.49%
GCGTAGTGTTGG----TACCTGGCCAC	447	-4	0.40%

YW-W9-Syn94crBCL11A7d-KO(AAV)-24h **24h**	Ins	Del	HDR
61300 | 57.99%	**44.27%**	**13.72%**	**0.00%**
**Typical seqs**	**Reads**	**Type**	**pct.**
GCGTAGTGTTGGGTCCT|ACC**TGG**CCAC	25752	WT	42.01%
GCGTAGTGTTGGGTCCTtACCTGGCCAC	23235	+1 t	37.90%
GCGTAGTGTTGGGT----CCTGGCCAC	2246	-4	3.66%
GCGTAGTGTTGGGTCCTCTACCTGGCCAC	1145	+2	1.87%
GCGTAGTGTTGGGT-CTACCTGGCCAC	827	-1	1.35%
GCGTAGTGTTGGGTCC-ACCTGGCCAC	577	-1	0.94%
GCGTAGTGTTGGG---TACCTGGCCAC	575	-3	0.94%
GCGTAGTGTTGGGTCCTaACCTGGCCAC	534	+1 a	0.87%
GCGTAGTGTTGGGTC-----------C	450	-11	0.73%
GCGTAGTGT-----------TGGCCAC	301	-11	0.49%

YW-W9-Syn94crBCL11A7d-KO(AAV)-48h **48h**	Ins	Del	HDR
50460 | 63.82%	**45.82%**	**17.99%**	**0.00%**
**Typical seqs**	**Reads**	**Type**	**pct.**
GCGTAGTGTTGGGTCCT|ACC**TGG**CCAC	18258	WT	36.18%
GCGTAGTGTTGGGTCCTtACCTGGCCAC	19995	+1 t	39.63%
GCGTAGTGTTGGGT----CCTGGCCAC	2202	-4	4.36%
GCGTAGTGTTGGGTCCTCTACCTGGCCAC	878	+2	1.74%
GCGTAGTGT-----------TGGCCAC	598	-11	1.19%
GCGTAGTGTTGGGT-CTACCTGGCCAC	540	-1	1.07%
GCGTAGTGTTGGGT----------CAC	535	-10	1.06%
GCGTAGTGTTGGGTCC-ACCTGGCCAC	530	-1	1.05%
GCGTAGTGTTGGGTC--ACCTGGCCAC	480	-2	0.95%
GCGTAGTGTTGGGTC-----------C	313	-11	0.62%

P108-P115-YW-W9-Syn94crBCL11A-AAVKI

YW-W9-Syn94crBCL11A7d-AAV6KI-4h 4h	Ins	Del	HDR
53467 | 13.75%	10.20%	3.03%	0.53%
**Typical seqs**	**Reads**	**Type**	**pct.**
GCGTAGTGTTGGGTCCT|ACC**TGG**CCAC	46114	WT	86.25%
GCGTAGTGTTGGGTCCTtACCTGGCCAC	5036	+1 t	9.42%
GCGTAGTGTTGGGTCC-ACCTGGCCAC	370	-1	0.69%
GCGTAGTGTTGGGTCCTGTTTAAACACCTG GCCAC	279	+8 HDR	0.52%
GCGTAGTGTTGGGT----CCTGGCCAC	184	-4	0.34%
GCGTAGTGTTGGGTC--ACCTGGCCAC	177	-2	0.33%
GCGTAGTGTTGGGTCCTCTACCTGGCCAC	179	+2	0.33%
GCGTAGTGTTGGG---TACCTGGCCAC	173	-3	0.32%
GCGTAGTGT-------TACCTGGCCAC	113	-7	0.21%
GCGTAGTGTTGGGTCCT---TGGCCAC	104	-3	0.19%

YW-W9-Syn94crBCL11A7d-AAV6KI-8h 8h	Ins	Del	HDR
81331 | 36.11%	26.33%	4.92%	4.86%
**Typical seqs**	**Reads**	**Type**	**pct.**
GCGTAGTGTTGGGTCCT|ACC**TGG**CCAC	51963	WT	63.89%
GCGTAGTGTTGGGTCCTtACCTGGCCAC	19003	+1 t	23.37%
GCGTAGTGTTGGGTCCTGTTTAAACACCTG GCCAC	3845	+8 HDR	4.73%
GCGTAGTGTTGGGTCC-ACCTGGCCAC	1210	-1	1.49%
GCGTAGTGTTGGGTCCTCTACCTGGCCAC	714	+2	0.88%
GCGTAGTGTTGGGT----CCTGGCCAC	582	-4	0.72%
GCGTAGTGTTGGGTC--ACCTGGCCAC	533	-2	0.66%
GCGTAGTGTTGGGT--TACCTGGCCAC	291	-2	0.36%
GCGTAGTGTTGGGT-CTACCTGGCCAC	275	-1	0.34%
GCGTAGTGTTGGGTCCCTACCTGGCCAC	242	+1 Ins	0.30%

YW-W9-Syn94crBCL11A7d-AAV6KI-12h 12h	Ins	Del	HDR
66281 | 50.12%	31.06%	7.18%	11.88%
**Typical seqs**	**Reads**	**Type**	**pct.**
GCGTAGTGTTGGGTCCT|ACC**TGG**CCAC	33061	WT	49.88%
GCGTAGTGTTGGGTCCTtACCTGGCCAC	17847	+1 t	26.93%
GCGTAGTGTTGGGTCCTGTTTAAACACCTG GCCAC	7613	+8 HDR	11.49%
GCGTAGTGTTGGGTCCTCTACCTGGCCAC	980	+2	1.48%
GCGTAGTGTTGGGTCC-ACCTGGCCAC	805	-1	1.21%
GCGTAGTGTTGGGT----CCTGGCCAC	495	-4	0.75%
GCGTAGTGTTGGGT-CTACCTGGCCAC	317	-1	0.48%
GCGTAGTGTTGGGTC--ACCTGGCCAC	301	-2	0.45%
GCGTAGTGTTGGG----ACCTGGCCAC	207	-4	0.31%
GCGTAGTGTTGGGTC-----------C	197	-11	0.30%

YW-W9-Syn94crBCL11A7d-AAV6KI-24h-BC1 24h	Ins	Del	HDR
38582 | 65.57%	38.60%	9.77%	17.20%
**Typical seqs**	**Reads**	**Type**	**pct.**
GCGTAGTGTTGGGTCCT|ACC**TGG**CCAC	13284	WT	34.43%
GCGTAGTGTTGGGTCCTtACCTGGCCAC	13094	+1 t	33.94%
GCGTAGTGTTGGGTCCTGTTTAAACACCTG GCCAC	6443	+8 HDR	16.70%
GCGTAGTGTTGGGTCCTCTACCTGGCCAC	641	+2	1.66%
GCGTAGTGTTGGGTC-----------C	493	-11	1.28%
GCGTAGTGTTGGGT-CTACCTGGCCAC	376	-1	0.97%
GCGTAGTGTTGGGT----CCTGGCCAC	302	-4	0.78%
GCGTAGTGTTGGGTCC-ACCTGGCCAC	262	-1	0.68%
GCGTAGTGTTGGG---TACCTGGCCAC	188	-3	0.49%
GCGTAGTGTTGGGTCCCTACCTGGCCAC	169	+1 Ins	0.44%

YW-W9-Syn94crBCL11A7d-AAV6KI-48h-BC2 48h	Ins	Del	HDR
39469 | 70.91%	40.55%	13.10%	17.25%
**Typical seqs**	**Reads**	**Type**	**pct.**
GCGTAGTGTTGGGTCCT|ACC**TGG**CCAC	11483	WT	29.09%
GCGTAGTGTTGGGTCCTtACCTGGCCAC	14033	+1 t	35.55%
GCGTAGTGTTGGGTCCTGTTTAAACACCTG GCCAC	6619	+8 HDR	16.77%
GCGTAGTGTTGGGTCC-ACCTGGCCAC	638	-1	1.62%
GCGTAGTGTTGGGTCCTCTACCTGGCCAC	609	+2	1.54%
GCGTAGTGTTGGGTC-----------C	530	-11	1.34%
GCGTAGTGTTGGGT----CCTGGCCAC	455	-4	1.15%
GCGTAGTGTTGGGTC--ACCTGGCCAC	307	-2	0.78%
GCGTAGTGTTGGG---TACCTGGCCAC	285	-3	0.72%
GCGTAGTGTTGGGT-CTACCTGGCCAC	275	-1	0.70%

P108-P115-YW-W9-Syn96crBCL11A-KO

YW-W9-Syn96crBCL11A8b-KO(AAV)-4h **4h**	Ins	Del	HDR	YW-W9-Syn96crBCL11A8b-KO(AAV)-8h **8h**	Ins	Del	HDR	YW-W9-Syn96crBCL11A8b-KO(AAV)-12h **12h**	Ins	Del	HDR
160228 | 4.10%	**1.04%**	**3.06%**	**0.00%**	167780 | 17.60%	**3.31%**	**14.29%**	**0.00%**	177480 | 23.42%	**4.14%**	**19.28%**	**0.00%**
**Typical seqs**	**Reads**	**Type**	**pct.**	**Typical seqs**	**Reads**	**Type**	**pct.**	**Typical seqs**	**Reads**	**Type**	**pct.**
GCGGGCCACCCACATTC|ATT**GGG**GGAA	153665	WT	95.90%	GCGGGCCACCCACATTC|ATT**GGG**GGAA	138247	WT	82.40%	GCGGGCCACCCACATTC|ATT**GGG**GGAA	135917	WT	76.58%
GCGGGCCACCCA----CATTGGGGGAA	1711	-4	1.07%	GCGGGCCACCCA----CATTGGGGGAA	6789	-4	4.05%	GCGGGCCACCCA----CATTGGGGGAA	10148	-4	5.72%
GCGGGCCACCCACATTCaATTGGGGGAA	986	+1 a	0.62%	GCGGGCCACCCACATT-ATTGGGGGAA	4705	-1	2.80%	GCGGGCCACCCACATTC-TTGGGGGAA	5254	-1	2.96%
GCGGGCCACCCACATTC-TTGGGGGAA	988	-1	0.62%	GCGGGCCACCCACATTC-TTGGGGGAA	3875	-1	2.31%	GCGGGCCACCCACATT-ATTGGGGGAA	4258	-1	2.40%
GCGGGCCACCCACATT-ATTGGGGGAA	733	-1	0.46%	GCGGGCCACCCACATTCcATTGGGGGAA	2925	+1 c	1.74%	GCGGGCCACCCACATTCcATTGGGGGAA	2709	+1 c	1.53%
GCGGGCCACCCACA---ATTGGGGGAA	672	-3	0.42%	GCGGGCCACCCACA---ATTGGGGGAA	1360	-3	0.81%	GCGGGCCACCCACATTCaATTGGGGGAA	1789	+1 a	1.01%
GCGGGCCACCCACATTCcATTGGGGGAA	651	+1 c	0.41%	GCGGGCCACCCACAT--ATTGGGGGAA	1016	-2	0.61%	GCGGGCCACCCACA---ATTGGGGGAA	1516	-3	0.85%
GCGGGCCACCCA-----ATTGGGGGAA	389	-5	0.24%	GCGGGCCACCCACATT--TTGGGGGAA	511	-2	0.30%	GC-------------------GGGGAA	933	-19	0.53%
GCGGGCCACC----TTCCTTGGGGGAA	276	-4	0.17%	GCGGGCCACCCACATTCaATTGGGGGAA	488	+1 a	0.29%	GCGGGCCACCCACATTCtATTGGGGGAA	708	+1 t	0.40%
GCGGGCCACCCACATTC----------	30	-16	0.02%	GCGGGC------------------AAA	422	-18	0.25%	GCGGGCCACC-----ACATTGGGGGAA	655	-5	0.37%

YW-W9-Syn96crBCL11A8b-KO(AAV)-24h **24h**	Ins	Del	HDR	YW-W9-Syn96crBCL11A8b-KO(AAV)-48h **48h**	Ins	Del	HDR
89100 | 37.48%	**4.50%**	**32.98%**	**0.00%**	158636 | 43.03%	**5.50%**	**37.53%**	**0.00%**
**Typical seqs**	**Reads**	**Type**	**pct.**	**Typical seqs**	**Reads**	**Type**	**pct.**
GCGGGCCACCCACATTC|ATT**GGG**GGAA	55704	WT	62.52%	GCGGGCCACCCACATTC|ATT**GGG**GGAA	90374	WT	56.97%
GCGGGCCACCCA----CATTGGGGGAA	9963	-4	11.18%	GCGGGCCACCCA----CATTGGGGGAA	21943	-4	13.83%
GCGGGCCACCCACATTC-TTGGGGGAA	2853	-1	3.20%	GCGGGCCACCCACATT-ATTGGGGGAA	5376	-1	3.39%
GCGGGCCACCCACATT-ATTGGGGGAA	2753	-1	3.09%	GCGGGCCACCCACATTC-TTGGGGGAA	4413	-1	2.78%
GCGGGCCACCCACATTCcATTGGGGGAA	1161	+1 c	1.30%	GCGGGCCACCCACATTCcATTGGGGGAA	2529	+1 c	1.59%
GCGGGCCACCCACAT--ATTGGGGGAA	1042	-2	1.17%	GCGGGCCACCCACAT--ATTGGGGGAA	1764	-2	1.11%
GCGGGCCACCCACATTCtATTGGGGGAA	667	+1 t	0.75%	GCGGGCCACCCACATTCaATTGGGGGAA	1564	+1 a	0.99%
GCGGGCCACCCACA---ATTGGGGGAA	666	-3	0.75%	GCGGGCCA--------CATTGGGGGAA	1350	-8	0.85%
GCGGGCCACCCACATTC--TGGGGGAA	608	-2	0.68%	GC--------------------GGGAA	1249	-20	0.79%
GCGGGCCACCCACATT-----------	862	-15	0.97%	GCGGGCCACCCACA---ATTGGGGGAA	1208	-3	0.76%

P108-P115-YW-W9-Syn96crBCL11A-AAVKI

YW-W9-Syn96crBCL11A8b-AAV6KI-4h 4h	Ins	Del	HDR
117953 | 3.68%	**0.92%**	**2.76%**	**0.00%**
**Typical seqs**	**Reads**	**Type**	**pct.**
GCGGGCCACCCACATTC|ATT**GGG**GGAA	113614	WT	96.32%
GCGGGCCACCCA----CATTGGGGGAA	921	-4	0.78%
GCGGGCCACCCACATTCcATTGGGGGAA	590	+1 c	0.50%
GCGGGCCACCCACATT-ATTGGGGGAA	456	-1	0.39%
GCGGGCCACCCACATTC-TTGGGGGAA	424	-1	0.36%
GCGGGCCACCCACA---ATTGGGGGAA	417	-3	0.35%
GCGGGCCACCCACA-TCATTGGGGGAA	346	-1	0.29%
GCGGGCCACCCACAT--ATTGGGGGAA	333	-2	0.28%
GCGGGCCACCCACAT------------	183	-19	0.16%
GCGGGCCACCCACATTCTTCACCCACATTG GGGGAA	175	+9	0.15%

YW-W9-Syn96crBCL11A8b-AAV6KI-8h 8h	Ins	Del	HDR
131052 | 12.23%	**2.11%**	**10.12%**	**0.00%**
**Typical seqs**	**Reads**	**Type**	**pct.**
GCGGGCCACCCACATTC|ATT**GGG**GGAA	115018	WT	87.77%
GCGGGCCACCCA----CATTGGGGGAA	3096	-4	2.36%
GCGGGCCACCCACATTC-TTGGGGGAA	2812	-1	2.15%
GCGGGCCACCCACATT-ATTGGGGGAA	2756	-1	2.10%
GCGGGCCACCCACAT--ATTGGGGGAA	1356	-2	1.03%
GCGGGCCACCCACATTCcATTGGGGGAA	912	+1 c	0.70%
GCGGGCCACCCACATTCaATTGGGGGAA	739	+1 a	0.56%
GCGGGCCACCCACATTC--TGGGGGAA	415	-2	0.32%
GCGGGCCA--------CATTGGGGGAA	414	-8	0.32%
GCGGGCCACCCACATT--TTGGGGGAA	375	-2	0.29%

YW-W9-Syn96crBCL11A8b-AAV6KI-12h 12h	Ins	Del	HDR
101222 | 22.20%	**3.49%**	**18.47%**	**0.24%**
**Typical seqs**	**Reads**	**Type**	**pct.**
GCGGGCCACCCACATTC|ATT**GGG**GGAA	78753	WT	77.80%
GCGGGCCACCCACATT-ATTGGGGGAA	4256	-1	4.20%
GCGGGCCACCCACATTC-TTGGGGGAA	3573	-1	3.53%
GCGGGCCACCCA----CATTGGGGGAA	3399	-4	3.36%
GCGGGCCACCCACATTCcATTGGGGGAA	1669	+1 c	1.65%
GCGGGCCACCCACAT--ATTGGGGGAA	981	-2	0.97%
GCGGGCCACCCACATTC---GGGGGAA	597	-3	0.59%
GCGGGCCACCCACA---ATTGGGGGAA	499	-3	0.49%
GCGGGCCACCCACATTCaATTGGGGGAA	471	+1 a	0.47%
GCGGGCCACCCA-----ATTGGGGGAA	398	-5	0.39%

YW-W9-Syn96crBCL11A8b-AAV6KI-24h-BC1 24h	Ins	Del	HDR
38208 | 37.45%	**5.56%**	**30.27%**	**1.61%**
**Typical seqs**	**Reads**	**Type**	**pct.**
GCGGGCCACCCACATTC|ATT**GGG**GGAA	23901	WT	62.55%
GCGGGCCACCCA----CATTGGGGGAA	3832	-4	10.03%
GCGGGCCACCCACATT-ATTGGGGGAA	1763	-1	4.61%
GCGGGCCACCCACATTC-TTGGGGGAA	1206	-1	3.16%
GCGGGCCACCCACATTCcATTGGGGGAA	789	+1 c	2.07%
GCGGGCCACCCACATTCGTTTAAACATTGG GGGAA	603	+8 HDR	1.58%
GCGGGCCACCCACATTCaATTGGGGGAA	468	+1 a	1.22%
GCGGGCCACCCACA---ATTGGGGGAA	443	-3	1.16%
GCGGGCCACCCACAT--ATTGGGGGAA	440	-2	1.15%
GCGG-----------------------	288	-23	0.75%

YW-W9-Syn96crBCL11A8b-AAV6KI-48h-BC2 48h	Ins	Del	HDR
32969 | 45.53%	**5.76%**	**37.13%**	**2.64%**
**Typical seqs**	**Reads**	**Type**	**pct.**
GCGGGCCACCCACATTC|ATT**GGG**GGAA	17959	WT	54.47%
GCGGGCCACCCA----CATTGGGGGAA	3689	-4	11.19%
GCGGGCCACCCACATT-ATTGGGGGAA	1399	-1	4.24%
GCGGGCCACCCACATTC-TTGGGGGAA	1147	-1	3.48%
GCGGGCCACCCACATTCGTTTAAACATTGG GGGAA	847	+8 HDR	2.57%
GCGGGCCACCCACATTCcATTGGGGGAA	636	+1 c	1.93%
GCGGGCCACCCACAT--ATTGGGGGAA	567	-2	1.72%
GCGGGCCACCCACATTCaATTGGGGGAA	485	+1 a	1.47%
GCGGGCCACCCACA---ATTGGGGGAA	490	-3	1.49%
GCGGGCCA--------CATTGGGGGAA	265	-8	0.80%

P118-YW-W9-Syn26crCD326ac-KO

Total | INDEL	4h	Ins	Del	HDR
36350 | 8.81%		5.33%	3.49%	0.00%

Typical seqs	Reads	Type	pct.
CCCTCCCGCGCCCCTCT|TCT**CGG**CGCG	33146	WT	91.19%
CCCTCCCGCGCCCCTCTtTCTCGGCGCG	1656	+1 t	4.56%
CCCTCCCGCGCCCCTC-TCTCGGCGCG	600	-1	1.65%
CCCTCCCGCGCCCC---TCTCGGCGCG	235	-3	0.65%
CCCTCCCGCGCCC-----CTCGGCGCG	51	-5	0.14%
CCCTCCCGCGCCCCTCTTTCTCG--GCG	42	-1	0.12%
CCCTCCCGCGCCCCTCCTTCTCGGCGCG	41	+1 Ins	0.11%
CCCTCC-----------------CGCG	35	-17	0.10%
CCCTCCCGCGCCCCCCTTTCTCGGCGCG	32	+1 Ins	0.09%
CCCTCCCGCGCCCCTCTcTCTCGGCGCG	30	+1 c	0.08%

Total | INDEL	8h	Ins	Del	HDR
55200 | 20.24%		11.21%	8.98%	0.04%

Typical seqs	Reads	Type	pct.
CCCTCCCGCGCCCCTCT|TCT**CGG**CGCG	44027	WT	79.76%
CCCTCCCGCGCCCCTCTtTCTCGGCGCG	5134	+1 t	9.30%
CCCTCCCGCGCCCCTC-TCTCGGCGCG	1339	-1	2.43%
CCCTCCCGCGCCCC---TCTCGGCGCG	678	-3	1.23%
CCCTCCCGCGCCC-----CTCGGCGCG	341	-5	0.62%
CCCTCCCGCGCCCCT--TCTCGGCGCG	225	-2	0.41%
CCCTCCCGCGCCCCT-TTCTCGGCGCG	160	-1	0.29%
CCCTCC-------------------CG	155	-19	0.28%
CCCTCC---------------------	154	-21	0.28%
CCCTCCCGCGCCCCTCTTTCTCG--GCG	89	-1	0.16%

Total | INDEL	12h	Ins	Del	HDR
45500 | 24.51%		11.58%	12.87%	0.06%

Typical seqs	Reads	Type	pct.
CCCTCCCGCGCCCCTCT|TCT**CGG**CGCG	34350	WT	75.49%
CCCTCCCGCGCCCCTCTtTCTCGGCGCG	4276	+1 t	9.40%
CCCTCCCGCGCCCCTC-TCTCGGCGCG	1528	-1	3.36%
CCCTCCCGCGCCCC---TCTCGGCGCG	884	-3	1.94%
CCCTCC---------------------	595	-21	1.31%
CCCTCC---------------------	595	-23	1.31%
CCCTCCCGCGCCC-----CTCGGCGCG	189	-5	0.42%
CCCTCCCGCGCCCCT--TCTCGGCGCG	147	-2	0.32%
CCCTCCCGCGCCCCT-TTCTCGGCGCG	143	-1	0.31%
CCCTCCCGCGCCCCTCTCTTCTCGGCGCG	121	+2	0.27%

Total | INDEL	24h	Ins	Del	HDR
82760 | 30.47%		12.32%	18.05%	0.10%

Typical seqs	Reads	Type	pct.
CCCTCCCGCGCCCCTCT|TCT**CGG**CGCG	57542	WT	69.53%
CCCTCCCGCGCCCCTCTtTCTCGGCGCG	8099	+1 t	9.79%
CCCTCCCGCGCCCC---TCTCGGCGCG	3040	-3	3.67%
CCCTCCCGCGCCCCTC-TCTCGGCGCG	2191	-1	2.65%
CCCTCCCGCGCCC-----CTCGGCGCG	911	-5	1.10%
CCCTCC---------------------	941	-21	1.14%
CCCTCC-----------------CGCG	318	-17	0.38%
CCCTCCCGCGCCCCT--TCTCGGCGCG	305	-2	0.37%
CCCTCCCGCGCCCCT------------	556	-14	0.67%
CCCTCC-------------------CG	235	-19	0.28%

Total | INDEL	48h	Ins	Del	HDR
40933 | 36.26%		11.16%	25.00%	0.10%

Typical seqs	Reads	Type	pct.
CCCTCCCGCGCCCCTCT|TCT**CGG**CGCG	26090	WT	63.74%
CCCTCCCGCGCCCCTCTtTCTCGGCGCG	3662	+1 t	8.95%
CCCTCCCGCGCCCC---TCTCGGCGCG	2214	-3	5.41%
CCCTCCCGCGCCCCTC-TCTCGGCGCG	1325	-1	3.24%
CCCTCC---------------------	1079	-21	2.64%
CCCTCCCGCGCCC-----CTCGGCGCG	706	-5	1.72%
CCCTCC---------------------	1079	-23	2.64%
CCCTCCCGC------------------	248	-24	0.61%
CCCTCC-----------------CGCG	222	-17	0.54%
CCCTCCCGCGCCCCT--TCTCGGCGCG	173	-2	0.42%

P118-YW-W9-Syn26crCD326ac-AAV6-KI

Total | INDEL			Ins	Del	HDR
100399 | 9.92%		4h	6.74%	2.82%	0.36%

Typical seqs	Reads	Type	pct.
CCCTCCCGCGCCCCTCT|TCT**CGG**CGCG	90437	WT	90.08%
CCCTCCCGCGCCCCTCTtTCTCGGCGCG	5690	+1 t	5.67%
CCCTCCCGCGCCCCTC-TCTCGGCGCG	1297	-1	1.29%
CCCTCCCGCGCCCC---TCTCGGCGCG	429	-3	0.43%
CCCTCCCGCACGCGTAGTTTAAACCTCGGC GCG	342	+6 HDR	0.34%
CCCTCC---------------------	119	-21	0.12%
CCCTCCCGCGCCCCTCTTTCTCG--GCG	105	-1	0.10%
CCCTCCCGCGCCCCTCTcTCTCGGCGCG	90	+1 c	0.09%
CCCTCCCGCGCCCCCCTTTCTCGGCGCG	91	+1 Ins	0.09%
CCCTCCCGCGCCC-----CTCGGCGCG	74	-5	0.07%

Total | INDEL			Ins	Del	HDR
120321 | 21.61%		8h	11.87%	6.58%	3.17%

Typical seqs	Reads	Type	pct.
CCCTCCCGCGCCCCTCT|TCT**CGG**CGCG	94317	WT	78.39%
CCCTCCCGCGCCCCTCTtTCTCGGCGCG	11794	+1 t	9.80%
CCCTCCCGCACGCGTAGTTTAAACCTCGGC GCG	3461	+6 HDR	2.88%
CCCTCCCGCGCCCCTC-TCTCGGCGCG	2786	-1	2.32%
CCCTCCCGCGCCCC---TCTCGGCGCG	1293	-3	1.07%
CCC--------------TCTCGGCGCG	348	-14	0.29%
CCCTCCCGCGCCCCT--TCTCGGCGCG	304	-2	0.25%
CCCTCCCGCGCCCCT-TTCTCGGCGCG	227	-1	0.19%
CCCTCCCGCGCCCCTCTTTCTCG--GCG	213	-1	0.18%
CCCTCCCGCGCCC-----CTCGGCGCG	201	-5	0.17%

Total | INDEL			Ins	Del	HDR
83203 | 25.64%		12h	12.91%	8.50%	4.24%

Typical seqs	Reads	Type	pct.
CCCTCCCGCGCCCCTCT|TCT**CGG**CGCG	61868	WT	74.36%
CCCTCCCGCGCCCCTCTtTCTCGGCGCG	8610	+1 t	10.35%
CCCTCCCGCACGCGTAGTTTAAACCTCGGC GCG	3135	+6 HDR	3.77%
CCCTCCCGCGCCCCTC-TCTCGGCGCG	1961	-1	2.36%
CCCTCCCGCGCCCC---TCTCGGCGCG	1253	-3	1.51%
CCCTCCCGCGCCCCTCTCTTCTCGGCGCG	257	+2	0.31%
CCCTCC---------------------	402	-23	0.48%
CCCTCCCGCGCCCCT------------	253	-12	0.30%
CCCTCC-------------------CG	193	-19	0.23%
CCCTCC---------------------	402	-21	0.48%

Total | INDEL			Ins	Del	HDR
60197 | 38.86%		24h	14.72%	14.84%	9.30%

Typical seqs	Reads	Type	pct.
CCCTCCCGCGCCCCTCT|TCT**CGG**CGCG	36807	WT	61.14%
CCCTCCCGCGCCCCTCTtTCTCGGCGCG	6861	+1 t	11.40%
CCCTCCCGCACGCGTAGTTTAAACCTCGGC GCG	5058	+6 HDR	8.40%
CCCTCCCGCGCCCCTC-TCTCGGCGCG	1784	-1	2.96%
CCCTCCCGCGCCCC---TCTCGGCGCG	1430	-3	2.38%
CCCTCC---------------------	602	-21	1.00%
CCCTCCCGCGCCC-----CTCGGCGCG	331	-5	0.55%
CCCTCCCGCGCCCCT--TCTCGGCGCG	307	-2	0.51%
CCC--------------TCTCGGCGCG	273	-14	0.45%
CCCTCC-------------------CG	240	-19	0.40%

Total | INDEL			Ins	Del	HDR
93152 | 50.25%		48h	16.59%	17.46%	16.20%

Typical seqs	Reads	Type	pct.
CCCTCCCGCGCCCCTCT|TCT**CGG**CGCG	46340	WT	49.75%
CCCTCCCGCACGCGTAGTTTAAACCTCGGC GCG	13688	+6 HDR	14.69%
CCCTCCCGCGCCCCTCTtTCTCGGCGCG	11787	+1 t	12.65%
CCCTCCCGCGCCCCTC-TCTCGGCGCG	3526	-1	3.79%
CCCTCCCGCGCCCC---TCTCGGCGCG	2252	-3	2.42%
CCCTCCCGCGCCC-----CTCGGCGCG	1059	-5	1.14%
CCCTCC---------------------	1059	-21	1.14%
CCCTCC---------------------	1059	-23	1.14%
CC----------------CTCGGCGCG	325	-16	0.35%
CCCTCCCGCGCCCCTCT----GGCGCG	304	-4	0.33%

P118-YW-W9-Syn27crCD326ad-KO

4h

Total | INDEL	Ins	Del	HDR
26081 | 0.18%	0.04%	0.14%	0.00%

Typical seqs	Reads	Type	pct.
CGCGCCGAGAAGAGGGG|CGC**GGG**AGGG	26033	WT	99.82%
CGCGCCGAGAAGGGGGGCGCGGGAGGG	397	-2	1.52%
CGCGCCGAGAAGAGGGGgCGCGGGAGGG	11	+1 g	0.04%
CGCGCCGAGAAGA-GGGCGCGGGAGGG	10	-1	0.04%
CGCGCCGAGAAG-GGGGCGCGGGAGGG	9	-1	0.03%
CGCCCC---------TCTTCTCGGCGCGGGAGGG	4	-2	0.02%
CGCGCCGAGAAGAGGGG-GCGGGAGGG	3	-1	0.01%

8h

Total | INDEL	Ins	Del	HDR
14362 | 0.33%	0.05%	0.26%	0.03%

Typical seqs	Reads	Type	pct.
CGCGCCGAGAAGAGGGG|CGC**GGG**AGGG	14314	WT	99.67%
CGCGCCGAGAAGA-GGGCGCGGGAGGG	17	-1	0.12%
CGCGCCGAGAAGGGGGGCGCGGGAGGG	230	-2	1.60%
CGCGCCGAGAAGAGGGGgCGCGGGAGGG	7	+1 g	0.05%
CGCGCCGAGGTTTAAACTACGCGTGCGGGAGGG	4	+6 HDR	0.03%
----------------GCGCGGGAGGG	3	-21	0.02%
CGCGCCGAGAAGAGGGGCGC-GGAGGG	3	-1	0.02%

12h

Total | INDEL	Ins	Del	HDR
29980 | 0.78%	0.05%	0.66%	0.08%

Typical seqs	Reads	Type	pct.
CGCGCCGAGAAGAGGGG|CGC**GGG**AGGG	29745	WT	99.22%
CGCGCCGAGAA-----------GAGGG	64	-11	0.21%
CGCGCCGAGAAGA-GGGCGCGGGAGGG	54	-1	0.18%
CGCGCCGAGGTTTAAACTACGCGTGCGGGAGGG	23	+6 HDR	0.08%
CGCGCCGAGA----------------G	21	-16	0.07%
CGCGCCGAGAAGAGGGGTAAAAGCGCGGGAGGG	10	+6	0.03%
CGCGCCGAGAAGAGGGGCGC-GGAGGG	9	-1	0.03%
CGCCCC---------TCTTCTCGGCGCGGGAGGG	8	-2	0.03%
CGCGCCGAGAAG-GGGGCGCGGGAGGG	8	-1	0.03%
CGCGCCGAGAAGGGGGGCGCGGGAGGG	490	-2	1.63%

24h

Total | INDEL	Ins	Del	HDR
67557 | 1.56%	0.13%	1.33%	0.10%

Typical seqs	Reads	Type	pct.
CGCGCCGAGAAGAGGGG|CGC**GGG**AGGG	66500	WT	98.44%
CGCGCCGAGA-----------------	146	-24	0.22%
----------------GCGCGGGAGGG	195	-21	0.29%
CGCGCCGAGAA-----------GAGGG	104	-11	0.15%
CGCGCCGAGAAGA-GGGCGCGGGAGGG	66	-1	0.10%
CGCGCCGAGGTTTAAACTACGCGTGCGGGAGGG	63	+6 HDR	0.09%
CGCGCCGAGAAGA--GGCGCGGGAGGG	65	-2	0.10%
----------------GCGCGGGAGGG	195	-17	0.29%
CGCGCCGAGAAGAG------GGGAGGG	52	-6	0.08%
CGCGCCGAGAAGAGGGGCGC-GGAGGG	44	-1	0.07%

48h

Total | INDEL	Ins	Del	HDR
64316 | 1.93%	0.22%	1.60%	0.11%

Typical seqs	Reads	Type	pct.
CGCGCCGAGAAGAGGGG|CGC**GGG**AGGG	63075	WT	98.07%
CGCGCCGAGAA-----------GAGGG	311	-11	0.48%
CGCGCCGAGGTTTAAACTACGCGTGCGGGAGGG	70	+6 HDR	0.11%
CGCGCCGAGAAGA-GGGCGCGGGAGGG	68	-1	0.11%
CGCGCCGAGAAGAG------GGGAGGG	61	-6	0.09%
----------------GCGCGGGAGGG	118	-21	0.18%
CGCGCCGAGAAGA-------GGAGGG	47	-7	0.07%
CGCGC------------CGCGGGAGGG	40	-12	0.06%
CGCGCCGAGAAGAGGGG-GCGGGAGGG	36	-1	0.06%
----------------GCGCGGGAGGG	118	-19	0.18%

P118-YW-W9-Syn27crCD326ad-AAV6-KI

4h

Total	INDEL	Ins	Del	HDR
105516	0.45%	0.02%	0.43%	0.00%

Typical seqs	Reads	Type	pct.
CGCGCCGAGAAGAGGGG|CGC<u>**GGG**</u>AGGG	105042	WT	99.55%
CGCGCCGAGAAGA-GGGCGCGGGAGGG	90	-1	0.09%
CGCGCCGAGAAGA-------GGGAGGG	56	-7	0.05%
CGCGCCGAGAAGAGGGGCGC-GGAGGG	50	-1	0.05%
CGCGCCGAGAAGA--GGCGCGGGAGGG	45	-2	0.04%
CGCGCCGAGAAG-GGGGCGCGGGAGGG	38	-1	0.04%
CGCGCCGAGAAGGGGGGCGCGGGAGGG	1610	-2	1.53%
CGCCCC--------- TCTTCTCGGCGCGGGAGGG	30	-2	0.03%
CGCGCCGAGAAGAGGGGgCGCGGGAGGG	23	+1 g	0.02%
CGCGCCGAGAAGAGGGGCGCAGGAGGG	378	-2	0.36%

8h

Total	INDEL	Ins	Del	HDR
125572	0.69%	0.14%	0.51%	0.04%

Typical seqs	Reads	Type	pct.
CGCGCCGAGAAGAGGGG|CGC<u>**GGG**</u>AGGG	124702	WT	99.31%
CGCGCCGAGAAGAGGGGcCGCGGGAGGG	111	+1 c	0.09%
CGCGCCGAGAAGA-GGGCGCGGGAGGG	108	-1	0.09%
CGCGCCGAGAA-----------GAGGG	106	-11	0.08%
CGCGCCGAGAAGAGGGGCGC-GGAGGG	71	-1	0.06%
CGCGCCGAGAA----GGCGCGGGAGGG	78	-4	0.06%
CGCGCCGAGGTTTAAACTACGCGTGCGGGA GGG	51	+6 HDR	0.04%
CGCGCCGAGAAGAGGGGgCGCGGGAGGG	39	+1 g	0.03%
CGCGCCGAGAAG-GGGGCGCGGGAGGG	38	-1	0.03%
CGCGCCGAGAAGGGGGGCGCGGGAGGG	1949	-2	1.55%

12h

Total	INDEL	Ins	Del	HDR
87752	0.85%	0.14%	0.65%	0.06%

Typical seqs	Reads	Type	pct.
CGCGCCGAGAAGAGGGG|CGC<u>**GGG**</u>AGGG	87008	WT	99.15%
CGCGCCGAGAAGA-GGGCGCGGGAGGG	124	-1	0.14%
CGC---------------------GGG	105	-21	0.12%
CGCGCCGAGAAGAGGGGcCGCGGGAGGG	77	+1 c	0.09%
CGCGCCGAGAAGAGGGG-GCGGGAGGG	75	-1	0.09%
CGCGCCGAGGTTTAAACTACGCGTGCGGGA GGG	55	+6 HDR	0.06%
CGCGCCGAGAAGA--GGCGCGGGAGGG	45	-2	0.05%
CGCGCCGAGAAGAGGGGCGC-GGAGGG	39	-1	0.04%
CGCGCCGAGAAGGGGGGCGCGGGAGGG	1399	-2	1.59%
CGCGCCGAGAAGAGGGGgCGCGGGAGGG	24	+1 g	0.03%

24h

Total	INDEL	Ins	Del	HDR
113038	1.15%	0.06%	0.93%	0.16%

Typical seqs	Reads	Type	pct.
CGCGCCGAGAAGAGGGG|CGC<u>**GGG**</u>AGGG	111736	WT	98.85%
CGCGCCGAGAAGA-GGGCGCGGGAGGG	320	-1	0.28%
CGCGCCGAGGTTTAAACTACGCGTGCGGGA GGG	186	+6 HDR	0.16%
----------------GCGCGGGAGGG	150	-17	0.13%
CGCGCCGAGAA-----------GAGGG	112	-11	0.10%
CGCGCCGAGA-----------GGAGGG	59	-11	0.05%
CGCGCCGAGAAGAGGGGCGC-GGAGGG	57	-1	0.05%
CGCGCCGAGAAGAGGGG-GCGGGAGGG	50	-1	0.04%
CGCGCCGAGAAGAGGGGgCGCGGGAGGG	39	+1 g	0.03%
CGCGCCGAGAAGGGGGGCGCGGGAGGG	1757	-2	1.55%

48h

Total	INDEL	Ins	Del	HDR
112538	1.95%	0.12%	1.11%	0.72%

Typical seqs	Reads	Type	pct.
CGCGCCGAGAAGAGGGG|CGC<u>**GGG**</u>AGGG	110346	WT	98.05%
CGCGCCGAGGTTTAAACTACGCGTGCGGGA GGG	788	+6 HDR	0.70%
CGCGCCGAGAAGA-GGGCGCGGGAGGG	222	-1	0.20%
CGCGCCGAGAAGA--------------	147	-18	0.13%
CGCGCCGAGAA-----------GAGGG	113	-11	0.10%
CGCGCCGAGAAGAGGGG----------	116	-12	0.10%
CGCGCCGAGAA-----GCGCGGGAGGG	80	-5	0.07%
CGCGCCGAGAAGAGGGGgCGCGGGAGGG	66	+1 g	0.06%
CGCGCCGAGAAGA-----GCGGGAGGG	54	-5	0.05%
----------------GCGCGGGAGGG	107	-19	0.10%

P118-YW-W9-Syn28crCD326ae-KO

Total	INDEL		Ins	Del	HDR
35281	2.83%	4h	**1.31%**	**1.50%**	**0.02%**

Typical seqs	Reads	Type	pct.
GCGCGCCGAGAAGAGGGGCG**CGG**GAGG	34284	WT	97.17%
GCGCGCCGAGAAGAGGGgGCGCGGGAGG	435	+1 g	1.23%
GCGCGCCGAGAAGA-GGGCGCGGGAGG	330	-1	0.94%
GCGCGCCGAGAAGA-------GGGAGG	43	-7	0.12%
GCGCGCCGAGAAGA--GGCGCGGGAGG	33	-2	0.09%
GCGCGCCGAGAA-----------GAGG	27	-11	0.08%
-----------------GCGCGGGAGG	40	-17	0.11%
-----------------GCGCGGGAGG	40	-21	0.11%
GCGCGCCGAGAAGA-GGGCGCGGGAGG	330	-3	0.94%
GCGCGCCGAGAAGAGGGGGCGCGGGAGG	435	-1	1.23%

Total	INDEL		Ins	Del	HDR
61407	5.93%	8h	**2.71%**	**3.18%**	**0.03%**

Typical seqs	Reads	Type	pct.
GCGCGCCGAGAAGAGGGGCG**CGG**GAGG	57767	WT	94.07%
GCGCGCCGAGAAGAGGGgGCGCGGGAGG	1386	+1 g	2.26%
GCGCGCCGAGAAGA-GGGCGCGGGAGG	732	-1	1.19%
GCGCGCCGAGAAGA--GGCGCGGGAGG	265	-2	0.43%
GCGCGCCGAGAAGA-------GGGAGG	226	-7	0.37%
-----------------GCGCGGGAGG	128	-21	0.21%
GCGCGCCGAGAA-----------GAGG	50	-11	0.08%
---------------------------	34	-30	0.06%
GCGCGCCGAGAAGAGGG--GCGGGAGG	31	-2	0.05%
GCGCGCCGAGAA-----GCGCGGGAGG	27	-5	0.04%

Total	INDEL		Ins	Del	HDR
46294	7.68%	12h	**3.42%**	**4.22%**	**0.05%**

Typical seqs	Reads	Type	pct.
GCGCGCCGAGAAGAGGGGCG**CGG**GAGG	42739	WT	92.32%
GCGCGCCGAGAAGAGGGgGCGCGGGAGG	1400	+1 g	3.02%
GCGCGCCGAGAAGA-GGGCGCGGGAGG	704	-1	1.52%
GCGCGCCGAGAA-----------GAGG	174	-11	0.38%
GCGCGCCGAGAAGA--GGCGCGGGAGG	172	-2	0.37%
GCGCGCCGAGAAGA-------GGGAGG	166	-7	0.36%
-----------------GCGCGGGAGG	179	-21	0.39%
GCGCGCCGAGAAGA-------------	56	-13	0.12%
-----------------GCGCGGGAGG	179	-17	0.39%
GC---------------GCGCGGGAGG	28	-15	0.06%

Total	INDEL		Ins	Del	HDR
104464	9.25%	24h	**3.25%**	**5.93%**	**0.08%**

Typical seqs	Reads	Type	pct.
GCGCGCCGAGAAGAGGGGCG**CGG**GAGG	94796	WT	90.75%
GCGCGCCGAGAAGAGGGgGCGCGGGAGG	2674	+1 g	2.56%
GCGCGCCGAGAAGA-GGGCGCGGGAGG	1675	-1	1.60%
-----------------GCGCGGGAGG	751	-21	0.72%
GCGCGCCGAGAAGA--GGCGCGGGAGG	554	-2	0.53%
GCGCGCCGAGAAGA-------GGGAGG	483	-7	0.46%
GCGCGCCGAGAA-----------GAGG	425	-11	0.41%
GCGCGCCGAGAAGA-------------	217	-13	0.21%
GCGCGCCGAGAAGA------------G	172	-12	0.16%
-----------------GCGCGGGAGG	751	-19	0.72%

Total	INDEL		Ins	Del	HDR
89045	11.40%	48h	**2.91%**	**8.41%**	**0.08%**

Typical seqs	Reads	Type	pct.
GCGCGCCGAGAAGAGGGGCG**CGG**GAGG	78893	WT	88.60%
GCGCGCCGAGAAGAGGGgGCGCGGGAGG	2118	+1 g	2.38%
GCGCGCCGAGAAGA-GGGCGCGGGAGG	1922	-1	2.16%
-----------------GCGCGGGAGG	1089	-21	1.22%
GCGCGCCGAGAAGA-------GGGAGG	742	-7	0.83%
GCGCGCCGAGAAGA--GGCGCGGGAGG	547	-2	0.61%
GCGCGCCGAGAA-----------GAGG	489	-11	0.55%
GCGCGCCGAGAAGA------------G	271	-12	0.30%
GCGCGCCGAGAAGA-------------	278	-13	0.31%
-----------------GCGCGGGAGG	1089	-19	1.22%

P118-YW-W9-Syn28crCD326ae-AAV6-KI

4h

Total	INDEL	Ins	Del	HDR
149158	3.04%	1.53%	1.47%	0.03%

Typical seqs	Reads	Type	pct.
GCGCGCCGAGAAGAGGG|GCG**CGG**GAGG	144631	WT	96.96%
GCGCGCCGAGAAGAGGGgGCGCGGGAGG	1990	+1 g	1.33%
GCGCGCCGAGAAGA-GGGCGCGGGAGG	1164	-1	0.78%
GCGCGCCGAGAAGA--GGCGCGGGAGG	166	-2	0.11%
-----------------GCGCGGGAGG	143	-21	0.10%
GCGCGCCGAGAAGA-------GGGAGG	107	-7	0.07%
GCGCGCCGAGAA-----------GAGG	58	-11	0.04%
GCGCGCCGAGAAGAGGGAGGGGCCCGCGCGGGAGG	57	+8	0.04%
GCGCGCCGAGAAGAGGGGCCCGGGACTCGGGAGG	58	+7	0.04%
GCGCGCCGAGAAGAGGGGGCGCGGGAGG	1990	-1	1.33%

8h

Total	INDEL	Ins	Del	HDR
159250	7.04%	2.89%	3.57%	0.58%

Typical seqs	Reads	Type	pct.
GCGCGCCGAGAAGAGGG|GCG**CGG**GAGG	148044	WT	92.96%
GCGCGCCGAGAAGAGGGgGCGCGGGAGG	3936	+1 g	2.47%
GCGCGCCGAGAAGA-GGGCGCGGGAGG	2598	-1	1.63%
GCGCGCCGAGGTTTAAACTACGCGTGCGGGAGG	863	+6 HDR	0.54%
GCGCGCCGAGAAGA--GGCGCGGGAGG	441	-2	0.28%
GCGCGCCGAGAA----GGCGCGGGAGG	294	-4	0.18%
GCGCGCCGAGAAGA-------GGGAGG	300	-7	0.19%
GCGCGCCGAGAA-----------GAGG	280	-11	0.18%
GCGCGCCGAGAAGA-------------	154	-13	0.10%
-----------------GCGCGGGAGG	254	-21	0.16%

12h

Total	INDEL	Ins	Del	HDR
124632	8.38%	3.12%	3.39%	1.86%

Typical seqs	Reads	Type	pct.
GCGCGCCGAGAAGAGGG|GCG**CGG**GAGG	114190	WT	91.62%
GCGCGCCGAGAAGAGGGgGCGCGGGAGG	3139	+1 g	2.52%
GCGCGCCGAGGTTTAAACTACGCGTGCGGGAGG	2176	+6 HDR	1.75%
GCGCGCCGAGAAGA-GGGCGCGGGAGG	1759	-1	1.41%
GCGCGCCGAGAAGA--GGCGCGGGAGG	432	-2	0.35%
GCGCGCCGAGAAGA-------GGGAGG	198	-7	0.16%
-----------------GCGCGGGAGG	310	-21	0.25%
GCGCGCCGAGAA----GGCGCGGGAGG	132	-4	0.11%
-----------------GCGCGGGAGG	310	-17	0.25%
----------------------GGAGG	87	-22	0.07%

24h

Total	INDEL	Ins	Del	HDR
135393	12.13%	3.52%	5.55%	3.06%

Typical seqs	Reads	Type	pct.
GCGCGCCGAGAAGAGGG|GCG**CGG**GAGG	118971	WT	87.87%
GCGCGCCGAGGTTTAAACTACGCGTGCGGGAGG	3823	+6 HDR	2.82%
GCGCGCCGAGAAGAGGGgGCGCGGGAGG	3738	+1 g	2.76%
GCGCGCCGAGAAGA-GGGCGCGGGAGG	2467	-1	1.82%
GCGCGCCGAGAAGA--GGCGCGGGAGG	852	-2	0.63%
-----------------GCGCGGGAGG	850	-21	0.63%
GCGCGCCGAGAA-----------GAGG	461	-11	0.34%
GCGCGCCGAGAAGA------------G	285	-12	0.21%
GCGCGCCGAGAAGA-------------	322	-13	0.24%
GCGCGCCGAGAAGA-------GGGAGG	205	-7	0.15%

48h

Total	INDEL	Ins	Del	HDR
119204	15.44%	4.01%	7.10%	4.33%

Typical seqs	Reads	Type	pct.
GCGCGCCGAGAAGAGGG|GCG**CGG**GAGG	100800	WT	84.56%
GCGCGCCGAGGTTTAAACTACGCGTGCGGGAGG	4801	+6 HDR	4.03%
GCGCGCCGAGAAGAGGGgGCGCGGGAGG	3420	+1 g	2.87%
GCGCGCCGAGAAGA-GGGCGCGGGAGG	3211	-1	2.69%
GCGCGCCGAGAAGA--GGCGCGGGAGG	947	-2	0.79%
GCGCGCCGAGAAGA-------GGGAGG	423	-7	0.35%
GCGCGCCGAGAA----GGCGCGGGAGG	375	-4	0.31%
GCGCGCCGAGAAGA-------------	256	-13	0.21%
GCGC-----------------------	256	-24	0.21%
-----------------GCGCGGGAGG	645	-19	0.54%

P118-YW-W9-Syn29crCD326af-KO

4h — Total | INDEL: 39718 | 16.35%; Ins 13.39%; Del 2.94%; HDR 0.01%

Typical seqs	Reads	Type	pct.
TGCGCGCGCGCCGAGAA|GAGGGGCGCG	33226	WT	83.65%
TGCGCGCGCGCCGAGAAaGAGGGGCGCG	4612	+1 a	11.61%
TGCGCGCGCGCCGAGA-GAGGGGCGCG	258	-1	0.65%
TGCGCGCGCGCC-----GAGGGGCGCG	123	-5	0.31%
TGCGCGCGCGCCGA---GAGGGGCGCG	116	-3	0.29%
TGCGCGCGCGCCGAGA--AGGGGCGCG	105	-2	0.26%
T--GCGCGCGCCGAGAAAGAGGGGCGCG	80	-1	0.20%
TGC------------------------	73	-30	0.18%
TGCGCGCGCGCCGAGAAgGAGGGGCGCG	74	+1 g	0.19%
TGCGCGCGCGCCGAGAAaGGGGGGCGCG	74	+1 a	0.19%

8h — Total | INDEL: 25054 | 29.37%; Ins 21.93%; Del 7.42%; HDR 0.02%

Typical seqs	Reads	Type	pct.
TGCGCGCGCGCCGAGAA|GAGGGGCGCG	17696	WT	70.63%
TGCGCGCGCGCCGAGAAaGAGGGGCGCG	4903	+1 a	19.57%
TGCGCGCGCGCCGAGA-GAGGGGCGCG	300	-1	1.20%
TGCGCGCGCGCC-----GAGGGGCGCG	282	-5	1.13%
TGCGCGCGCGCCGA---GAGGGGCGCG	202	-3	0.81%
TGCGCGCGCGCCGAGAA-AGGGGCGCG	111	-1	0.44%
T--GCGCGCGCCGAGAAAGAGGGGCGCG	88	-1	0.35%
TGCGCGCGCGCCGAGAAaGGGGGGCGCG	78	+1 a	0.31%
TGCGCGCGCGCCGAGA--AGGGGCGCG	76	-2	0.30%
T---------------------GCGCG	62	-21	0.25%

12h — Total | INDEL: 24362 | 35.72%; Ins 25.72%; Del 9.95%; HDR 0.05%

Typical seqs	Reads	Type	pct.
TGCGCGCGCGCCGAGAA|GAGGGGCGCG	15660	WT	64.28%
TGCGCGCGCGCCGAGAAaGAGGGGCGCG	5467	+1 a	22.44%
TGCGCGCGCGCCGAGA-GAGGGGCGCG	412	-1	1.69%
TGCGCGCGCGCC-----GAGGGGCGCG	265	-5	1.09%
TGCGCGCGCGCCGA---GAGGGGCGCG	209	-3	0.86%
T--GCGCGCGCCGAGAAAGAGGGGCGCG	113	-1	0.46%
T-----------------------GCG	88	-23	0.36%
T---------------------GCGCG	91	-21	0.37%
TGC-------------------GCGCG	83	-19	0.34%
TGCGCGCGCGCCGAGAAaGGGGGGCGCG	80	+1 a	0.33%

24h — Total | INDEL: 52675 | 43.98%; Ins 27.65%; Del 16.20%; HDR 0.13%

Typical seqs	Reads	Type	pct.
TGCGCGCGCGCCGAGAA|GAGGGGCGCG	29509	WT	56.02%
TGCGCGCGCGCCGAGAAaGAGGGGCGCG	12364	+1 a	23.47%
TGCGCGCGCGCC-----GAGGGGCGCG	1865	-5	3.54%
TGCGCGCGCGCCGAGA-GAGGGGCGCG	690	-1	1.31%
T---------------------GCGCG	652	-21	1.24%
TGCGCGCGCGCCGA---GAGGGGCGCG	622	-3	1.18%
TGC-------------------GCGCG	308	-19	0.58%
T--GCGCGCGCCGAGAAAGAGGGGCGCG	255	-1	0.48%
TGCGCGCGCGCCGAGAAaGGGGGGCGCG	170	+1 a	0.32%
TGCGCGCGCGCCGAGAAgGAGGGGCGCG	148	+1 g	0.28%

48h — Total | INDEL: 65078 | 51.34%; Ins 26.51%; Del 24.73%; HDR 0.11%

Typical seqs	Reads	Type	pct.
TGCGCGCGCGCCGAGAA|GAGGGGCGCG	31665	WT	48.66%
TGCGCGCGCGCCGAGAAaGAGGGGCGCG	14450	+1 a	22.20%
TGCGCGCGCGCC-----GAGGGGCGCG	3182	-5	4.89%
T---------------------GCGCG	1320	-21	2.03%
TGCGCGCGCGCCGA---GAGGGGCGCG	1195	-3	1.84%
TGCGCGCGCGCCGAGA-GAGGGGCGCG	909	-1	1.40%
TGCGC-----------------GCGCG	465	-17	0.71%
TGC-------------------GCGCG	398	-19	0.61%
T-----------------------GCG	371	-23	0.57%
TGCGCGCGCGCCGAGAA-AGGGGCGCG	307	-1	0.47%

P118-YW-W9-Syn29crCD326af-AAV6-KI

Total | INDEL	4h	Ins	Del	HDR
107513 | 14.70%		12.54%	2.08%	0.08%

Typical seqs	Reads	Type	pct.
TGCGCGCGCGCCGAGAA|GAG**<u>GGG</u>**CGCG	91710	WT	85.30%
TGCGCGCGCGCCGAGAAaGAGGGGCGCG	11474	+1 a	10.67%
TGCGCGCGCGCCGAGA-GAGGGGCGCG	413	-1	0.38%
T--GCGCGCGCCGAGAAAGAGGGGCGCG	236	-1	0.22%
TGCGCGCGCGCC-----GAGGGGCGCG	242	-5	0.23%
TGCGCGCGCGCCGAGA--AGGGGCGCG	213	-2	0.20%
TGCGCGCGCGCCGA---GAGGGGCGCG	180	-3	0.17%
TGCGCGCGCGCCGAGAAgGAGGGGCGCG	148	+1 g	0.14%
TGCGCGCGCGCCGAGAAAAGAGGGGCGCG	125	+2	0.12%
TGCGCGCGCGCCGAGAGAGAGGGGCGCG	125	+1 Ins	0.12%

Total | INDEL	8h	Ins	Del	HDR
139884 | 30.92%		21.77%	7.07%	2.08%

Typical seqs	Reads	Type	pct.
TGCGCGCGCGCCGAGAA|GAG**<u>GGG</u>**CGCG	96636	WT	69.08%
TGCGCGCGCGCCGAGAAaGAGGGGCGCG	25877	+1 a	18.50%
TGCGCGCGCGCCGAGGTTTAAACTACGCGT GCG	2591	+6 HDR	1.85%
TGCGCGCGCGCCGAGA-GAGGGGCGCG	1334	-1	0.95%
TGCGCGCGCGCC-----GAGGGGCGCG	921	-5	0.66%
TGCGCGCGCGCCGA---GAGGGGCGCG	898	-3	0.64%
TGCGCGCGCGCCGAGAA-AGGGGCGCG	866	-1	0.62%
T--GCGCGCGCCGAGAAAGAGGGGCGCG	608	-1	0.43%
TGC-------------AGAGGGGCGCG	401	-13	0.29%
T----------------GAGGGGCGCG	358	-16	0.26%

Total | INDEL	12h	Ins	Del	HDR
88828 | 40.24%		26.65%	7.21%	6.37%

Typical seqs	Reads	Type	pct.
TGCGCGCGCGCCGAGAA|GAG**<u>GGG</u>**CGCG	53085	WT	59.76%
TGCGCGCGCGCCGAGAAaGAGGGGCGCG	19658	+1 a	22.13%
TGCGCGCGCGCCGAGGTTTAAACTACGCGT GCG	5057	+6 HDR	5.69%
TGCGCGCGCGCCGAGA-GAGGGGCGCG	1145	-1	1.29%
T---------------------GCGCG	746	-21	0.84%
TGCGCGCGCGCC-----GAGGGGCGCG	698	-5	0.79%
T--GCGCGCGCCGAGAAAGAGGGGCGCG	396	-1	0.45%
TGCGCGCGCGCCGAGAA-AGGGGCGCG	380	-1	0.43%
TGCGCGCGCGCCGA---GAGGGGCGCG	321	-3	0.36%
TGCGCGCGCGCCGAGGAAGAGGGGCGCG	271	+1 Ins	0.31%

Total | INDEL	24h	Ins	Del	HDR
102367 | 49.28%		26.03%	13.17%	10.08%

Typical seqs	Reads	Type	pct.
TGCGCGCGCGCCGAGAA|GAG**<u>GGG</u>**CGCG	51920	WT	50.72%
TGCGCGCGCGCCGAGAAaGAGGGGCGCG	21526	+1 a	21.03%
TGCGCGCGCGCCGAGGTTTAAACTACGCGT GCG	9250	+6 HDR	9.04%
T---------------------GCGCG	1485	-21	1.45%
TGCGCGCGCGCCGA---GAGGGGCGCG	1108	-3	1.08%
TGCGCGCGCGCC-----GAGGGGCGCG	1101	-5	1.08%
TGCGCGCGCGCCGAGA-GAGGGGCGCG	959	-1	0.94%
TGC-------------------GCGCG	800	-19	0.78%
TGCGCGCGCGCCGAGA--AGGGGCGCG	463	-2	0.45%
TGCGCGCGCGCCGAGAA-AGGGGCGCG	427	-1	0.42%

Total | INDEL	48h	Ins	Del	HDR
78651 | 61.53%		32.91%	12.96%	15.67%

Typical seqs	Reads	Type	pct.
TGCGCGCGCGCCGAGAA|GAG**<u>GGG</u>**CGCG	30257	WT	38.47%
TGCGCGCGCGCCGAGAAaGAGGGGCGCG	20871	+1 a	26.54%
TGCGCGCGCGCCGAGGTTTAAACTACGCGT GCG	11143	+6 HDR	14.17%
TGCGCGCGCGCCGAGA-GAGGGGCGCG	1299	-1	1.65%
TGCGCGCGCGCC-----GAGGGGCGCG	1191	-5	1.51%
TGCGCGCGCGCCGA---GAGGGGCGCG	594	-3	0.76%
T--GCGCGCGCCGAGAAAGAGGGGCGCG	500	-1	0.64%
TGC-------------------GCGCG	380	-19	0.48%
T---------------------GCGCG	430	-21	0.55%
TGCGCGCGCGCCGA--------------	318	-14	0.40%

P118-YW-W9-Syn30crCD326ag-KO

Total | INDEL	4h	Ins	Del	HDR
47729 | 3.13%		1.13%	2.01%	0.00%

Typical seqs	Reads	Type	pct.
CTGCGCGCGCGCCGAGA|AGA**GGG**GCGC	46233	WT	96.87%
CTGCGCGCGCGCCGAG-AGAGGGGCGC	538	-1	1.13%
CTGCGCGCGCGCCGAGAaAGAGGGGCGC	423	+1 a	0.89%
CTGCGCGCGCGCCG---AGAGGGGCGC	109	-3	0.23%
CTGCGCGCGCGCCGAGAtAGAGGGGCGC	48	+1 t	0.10%
CTGCGCGCGCGCCG---AGAGAGGCGC	45	-3	0.09%
CTGCGCGCGCGCCGAGA--AGGGGCGC	38	-2	0.08%
CTGCGCGCGCGCC-----GAGGGGCGC	35	-5	0.07%
CT--GCGCGCGCCGAG-AGAGGGGCGC	21	-3	0.04%
CTGCGCGCGCGCC--GAAGAGGGGCGC	21	-2	0.04%

Total | INDEL	8h	Ins	Del	HDR
44024 | 6.81%		1.22%	5.57%	0.02%

Typical seqs	Reads	Type	pct.
CTGCGCGCGCGCCGAGA|AGA**GGG**GCGC	41026	WT	93.19%
CTGCGCGCGCGCCGAG-AGAGGGGCGC	861	-1	1.96%
CTGCGCGCGCGCCG---AGAGGGGCGC	543	-3	1.23%
CTGCGCGCGCGCCGAGAaAGAGGGGCGC	385	+1 a	0.87%
CTGCGCGCGCGCC--GAAGAGGGGCGC	94	-2	0.21%
CT-----------------------GC	90	-23	0.20%
CTGCGCGCGCGCC-----GAGGGGCGC	65	-5	0.15%
CTGCGCGCGCGCC--------------	62	-16	0.14%
CTGCGCGCGCGCCGAGA----------	70	-11	0.16%
CTGCG--------------GGAGGCGC	51	-14	0.12%

Total | INDEL	12h	Ins	Del	HDR
30859 | 11.59%		2.54%	9.01%	0.05%

Typical seqs	Reads	Type	pct.
CTGCGCGCGCGCCGAGA|AGA**GGG**GCGC	27281	WT	88.41%
CTGCGCGCGCGCCGAGAaAGAGGGGCGC	630	+1 a	2.04%
CTGCGCGCGCGCCG---AGAGGGGCGC	619	-3	2.01%
CTGCGCGCGCGCCGAG-AGAGGGGCGC	572	-1	1.85%
CT---------------------GCGC	266	-21	0.86%
CTGCGCGCGCGCC-----GAGGGGCGC	145	-5	0.47%
CTGCGCGC---------------GCGC	90	-15	0.29%
CTGC-------------------GCGC	89	-19	0.29%
CTGCGCGCGCGCCGA------------	82	-14	0.27%
CTGCGCGCGCGCC--GAAGAGGGGCGC	79	-2	0.26%

Total | INDEL	24h	Ins	Del	HDR
68819 | 16.67%		3.12%	13.46%	0.09%

Typical seqs	Reads	Type	pct.
CTGCGCGCGCGCCGAGA|AGA**GGG**GCGC	57345	WT	83.33%
CTGCGCGCGCGCCG---AGAGGGGCGC	2024	-3	2.94%
CTGCGCGCGCGCCGAG-AGAGGGGCGC	1606	-1	2.33%
CTGCGCGCGCGCCGAGAaAGAGGGGCGC	1567	+1 a	2.28%
CT---------------------GCGC	801	-21	1.16%
CTGCGCGCGCGCC-----GAGGGGCGC	481	-5	0.70%
CT-----------------------GC	410	-23	0.60%
CTGCGCGCGCGCCGA------------	394	-14	0.57%
CTGCGCGCGCGC-----AGAGGGGCGC	215	-5	0.31%
CTGC------------------GGCGC	200	-18	0.29%

Total | INDEL	48h	Ins	Del	HDR
62068 | 22.28%		2.88%	19.29%	0.11%

Typical seqs	Reads	Type	pct.
CTGCGCGCGCGCCGAGA|AGA**GGG**GCGC	48237	WT	77.72%
CTGCGCGCGCGCCG---AGAGGGGCGC	3377	-3	5.44%
CTGCGCGCGCGCCGAGAaAGAGGGGCGC	1227	+1 a	1.98%
CTGCGCGCGCGCCGAG-AGAGGGGCGC	1141	-1	1.84%
CT---------------------GCGC	852	-21	1.37%
CTGCGCGCGCGCC-----GAGGGGCGC	727	-5	1.17%
CTGCGC-----------------GCGC	434	-17	0.70%
CT-----------------------GC	351	-23	0.57%
CTGC-------------------GCGC	320	-19	0.52%
CTGCGCGCGCGCCGA------------	255	-14	0.41%

P118-YW-W9-Syn30crCD326ag-AAV6-KI

4h

Total | INDEL	Ins	Del	HDR
172248 | 2.33%	1.11%	1.22%	0.00%

Typical seqs	Reads	Type	pct.
CTGCGCGCGCGCCGAGA|AGAGGGGCGC	168240	WT	97.67%
CTGCGCGCGCGCCGAGAaAGAGGGGCGC	1621	+1 a	0.94%
CTGCGCGCGCGCCGAG-AGAGGGGCGC	962	-1	0.56%
CTGC-------------------GCGC	212	-19	0.12%
CTGCGCGCGCGCC--GAAGAGGGGCGC	197	-2	0.11%
CTGCGCGCGCGCCGAG--GAGGGGCGC	170	-2	0.10%
CTGCGCGCGCGCCGAGAAG-GGGGCGC	41	-1	0.02%
CTGCGCGCGCGCCGAGGAAGAGGGGCGC	35	+1 Ins	0.02%
CT--GCGCGCGCCGAGAAAGAGGGGCGC	33	-1	0.02%
CT--GCGCGCGCCGAGAAGGGGGGCGC	30	-2	0.02%

8h

Total | INDEL	Ins	Del	HDR
198224 | 6.64%	2.73%	3.12%	0.79%

Typical seqs	Reads	Type	pct.
CTGCGCGCGCGCCGAGA|AGAGGGGCGC	185056	WT	93.36%
CTGCGCGCGCGCCGAGAaAGAGGGGCGC	4324	+1 a	2.18%
CTGCGCGCGCGCCGAG-AGAGGGGCGC	3029	-1	1.53%
CTGCGCGCGCGCCGAGGTTTAAACTACGCG TGC	1458	+6 HDR	0.74%
CTGCGCGCGCGCCG---AGAGGGGCGC	511	-3	0.26%
CTGCGCGGGCGCGG----GAGGGGCGC	487	-4	0.25%
CTGCGCGCGCGCCGAGA-----GGCGC	258	-5	0.13%
CT----------------GAGGGGCGC	243	-16	0.12%
CTGCGCGC---------------GCGC	210	-15	0.11%
CTGCGCGCGCGCCGAG--GAGGGGCGC	209	-2	0.11%

12h

Total | INDEL	Ins	Del	HDR
131338 | 9.20%	3.01%	4.43%	1.77%

Typical seqs	Reads	Type	pct.
CTGCGCGCGCGCCGAGA|AGAGGGGCGC	119249	WT	90.80%
CTGCGCGCGCGCCGAGAaAGAGGGGCGC	3219	+1 a	2.45%
CTGCGCGCGCGCCGAGGTTTAAACTACGCG TGC	2041	+6 HDR	1.55%
CTGCGCGCGCGCCGAG-AGAGGGGCGC	1510	-1	1.15%
CT---------------------GCGC	653	-21	0.50%
CTGCGCGCGCGCCG---AGAGGGGCGC	462	-3	0.35%
CTGCGCGCGCGCCGA--------GCGC	360	-8	0.27%
CTGCGCGCGCGCC----AGAGGGGCGC	353	-4	0.27%
CT-----------------------GC	327	-23	0.25%
-------------------------GC	340	-26	0.26%

24h

Total | INDEL	Ins	Del	HDR
147345 | 18.80%	3.59%	8.26%	6.95%

Typical seqs	Reads	Type	pct.
CTGCGCGCGCGCCGAGA|AGAGGGGCGC	119645	WT	81.20%
CTGCGCGCGCGCCGAGGTTTAAACTACGCG TGC	9256	+6 HDR	6.28%
CTGCGCGCGCGCCGAGAaAGAGGGGCGC	2784	+1 a	1.89%
CTGCGCGCGCGCCG---AGAGGGGCGC	2667	-3	1.81%
CTGCGCGCGCGCCGAG-AGAGGGGCGC	2500	-1	1.70%
CTGC-------------------GCGC	946	-19	0.64%
CTGCGCGCGCGCCGAGA----------	694	-13	0.47%
CTGCGCGCGCGCCGAGAcAGAGGGGCGC	620	+1 c	0.42%
CTGCGCGCGCGCCGAGA--AGGGGCGC	521	-2	0.35%
CTGCGCGGGAGC--------------C	491	-14	0.33%

48h

Total | INDEL	Ins	Del	HDR
115351 | 31.89%	6.57%	16.09%	9.23%

Typical seqs	Reads	Type	pct.
CTGCGCGCGCGCCGAGA|AGAGGGGCGC	78565	WT	68.11%
CTGCGCGCGCGCCGAGGTTTAAACTACGCG TGC	9646	+6 HDR	8.36%
CTGCGCGCGCGCCGAGAaAGAGGGGCGC	4017	+1 a	3.48%
CTGCGCGCGCGCCGAG-AGAGGGGCGC	3619	-1	3.14%
CTGCGCGCGCGCCG---AGAGGGGCGC	3309	-3	2.87%
CGGC-----------------------	1457	-24	1.26%
CTGCGCGCGCGCCGAGAtAGAGGGGCGC	1274	+1 t	1.10%
CT---------------------GCGC	1197	-21	1.04%
CTGCGC-----------------GCGC	948	-17	0.82%
CTGCGCG----------AGAGGGGCGC	843	-10	0.73%

P118-YW-W9-Syn31crCD326ah-KO

Total | INDEL	4h	Ins	Del	HDR
40664 | 5.16%		3.56%	1.59%	0.01%

Typical seqs	Reads	Type	pct.
GCTGCGCGCGCGCCGAG|AAG**AGG**GGCG	38565	WT	94.84%
GCTGCGCGCGCGCCGAGgAAGAGGGGCG	1245	+1 g	3.06%
GCTGCGCGCGCGCC-----GAGGGGCG	151	-5	0.37%
GCTGCGCGCGCGCCGAG-AGAGGGGCG	88	-1	0.22%
GCTGCGCGCGCGCCG---AGAGGGGCG	60	-3	0.15%
GCTGCGCGCGCGCCGA-----------	46	-12	0.11%
GCTGCGCGCGCGCCGAGaAAGAGGGGCG	38	+1 a	0.09%
GCTGC-----------------ATGCG	35	-17	0.09%
GCTGCGCGCGCGCC--GAAGAGGGGCG	34	-2	0.08%
GCTGCGCGCG--------GGAGGGGCG	35	-8	0.09%

Total | INDEL	8h	Ins	Del	HDR
44071 | 12.22%		7.67%	4.50%	0.04%

Typical seqs	Reads	Type	pct.
GCTGCGCGCGCGCCGAG|AAG**AGG**GGCG	38687	WT	87.78%
GCTGCGCGCGCGCCGAGgAAGAGGGGCG	2856	+1 g	6.48%
GCTGCGCGCGCGCC-----GAGGGGCG	200	-5	0.45%
GCTGCGCGCGCGCCGAG-AGAGGGGCG	189	-1	0.43%
GCT---------------------GCG	188	-21	0.43%
GCTGCGCGCGCGCCGA-AAGAGGGGCG	150	-1	0.34%
GCTGCGCGCGCGCCGAGaAAGAGGGGCG	90	+1 a	0.20%
GCTGCGC-----------------GCG	72	-17	0.16%
GCTGCGCGCGC------AAGAGGGGCG	71	-6	0.16%
GCT--GCGCGCGCCGAGGAAGAGGGGCG	66	-1	0.15%

Total | INDEL	12h	Ins	Del	HDR
45084 | 14.40%		6.37%	7.96%	0.07%

Typical seqs	Reads	Type	pct.
GCTGCGCGCGCGCCGAG|AAG**AGG**GGCG	38593	WT	85.60%
GCTGCGCGCGCGCCGAGgAAGAGGGGCG	2327	+1 g	5.16%
GCTGCGCGCGCGCC-----GAGGGGCG	378	-5	0.84%
GCTGCGCGCGCGCC--GAAGAGGGGCG	309	-2	0.69%
GCT---------------------GCG	318	-21	0.71%
GCTGCGCGCGCGCCGAG-AGAGGGGCG	235	-1	0.52%
GCTGCGCGCGCGCCGA-AAGAGGGGCG	178	-1	0.39%
GCTGCGCGCGCGCCG---AGAGGGGCG	143	-3	0.32%
GCTGCGCGC---------------GCG	139	-15	0.31%
---------------------------	131	-29	0.29%

Total | INDEL	24h	Ins	Del	HDR
51350 | 21.23%		6.31%	14.81%	0.11%

Typical seqs	Reads	Type	pct.
GCTGCGCGCGCGCCGAG|AAG**AGG**GGCG	40450	WT	78.77%
GCTGCGCGCGCGCCGAGgAAGAGGGGCG	2528	+1 g	4.92%
GCTGCGCGCGCGCC-----GAGGGGCG	1250	-5	2.43%
GCT---------------------GCG	1231	-21	2.40%
GCTGC-------------------GCG	282	-19	0.55%
GCTGCGC-----------------GCG	293	-17	0.57%
GCT-----------------------G	254	-23	0.49%
--------------------------G	257	-26	0.50%
GCTGCGCGC---------------GCG	205	-15	0.40%
GCTGCGCGCGCGCC--GAAGAGGGGCG	190	-2	0.37%

Total | INDEL	48h	Ins	Del	HDR
86535 | 28.60%		7.23%	21.27%	0.11%

Typical seqs	Reads	Type	pct.
GCTGCGCGCGCGCCGAG|AAG**AGG**GGCG	61786	WT	71.40%
GCTGCGCGCGCGCCGAGgAAGAGGGGCG	4601	+1 g	5.32%
GCTGCGCGCGCGCC-----GAGGGGCG	2469	-5	2.85%
GCT---------------------GCG	2418	-21	2.79%
GCT-----------------------G	1474	-23	1.70%
GCTGC-------------------GCG	897	-19	1.04%
GCTGCGC-----------------GCG	759	-17	0.88%
GCTGCGCGCGCGCCGA-AAGAGGGGCG	516	-1	0.60%
GCTGCGCGCGCGCC--GAAGAGGGGCG	456	-2	0.53%
--------------------------G	414	-26	0.48%

P118-YW-W9-Syn31crCD326ah-AAV6-KI

4h

Total | INDEL	Ins	Del	HDR
164362 | 3.44%	2.56%	0.88%	0.01%

Typical seqs	Reads	Type	pct.
GCTGCGCGCGCGCCGAG|AAGAGGGGCG	158701	WT	96.56%
GCTGCGCGCGCGCCGAGgAAGAGGGGCG	3311	+1 g	2.01%
GCTGCGCGCGCGCC--GAAGAGGGGCG	208	-2	0.13%
GCTGCGCGCGCGCCGAGaAAGAGGGGCG	212	+1 a	0.13%
GCTGCG---------------GGGGCG	185	-15	0.11%
GCTGCGCGCGCGC----AAGAGGGGCG	121	-4	0.07%
GCTGCGCGCGCGCCGAGtAAGAGGGGCG	116	+1 t	0.07%
GCTGCGCGCGCGCCGAG---------G	120	-9	0.07%
GCTGCGCGCGCGCCGA-AAGAGGGGCG	101	-1	0.06%
GCTGCGCGCG--------GGAGGGGCG	97	-8	0.06%

8h

Total | INDEL	Ins	Del	HDR
193540 | 12.42%	5.96%	4.43%	2.02%

Typical seqs	Reads	Type	pct.
GCTGCGCGCGCGCCGAG|AAGAGGGGCG	169508	WT	87.58%
GCTGCGCGCGCGCCGAGgAAGAGGGGCG	9194	+1 g	4.75%
GCTGCGCGCGCGCCGAGGTTTAAACTACGC GTG	3606	+6 HDR	1.86%
GCTGCGCGCGCGCC--GAAGAGGGGCG	1072	-2	0.55%
GCT---------------------GCG	800	-21	0.41%
GCTGCGCGCGCGCCGA-AAGAGGGGCG	694	-1	0.36%
------------------------GCG	580	-24	0.30%
GCTGCGCGCGCGCC---AAGAGGGGCG	570	-3	0.29%
GCTGCGCGCGCGCCGAG-AGAGGGGCG	379	-1	0.20%
GCTGCGCGCGCGCCGAGaAAGAGGGGCG	287	+1 a	0.15%

12h

Total | INDEL	Ins	Del	HDR
133383 | 18.02%	7.86%	6.84%	3.32%

Typical seqs	Reads	Type	pct.
GCTGCGCGCGCGCCGAG|AAGAGGGGCG	109349	WT	81.98%
GCTGCGCGCGCGCCGAGgAAGAGGGGCG	8143	+1 g	6.10%
GCTGCGCGCGCGCCGAGGTTTAAACTACGC GTG	4000	+6 HDR	3.00%
GCT---------------------GCG	820	-21	0.61%
GCTGCGCGCGCGCC-----GAGGGGCG	682	-5	0.51%
GCTGCGCGCGCGCCGA-------GGCG	637	-7	0.48%
GCTGCGCGCGCGCCGAG----GGGGCG	540	-4	0.40%
GCTGC-------------------GCG	451	-19	0.34%
GCTGCGCGCGCGCC--GAAGAGGGGCG	426	-2	0.32%
GCTGCGCGCGCGCCGAG-AGAGGGGCG	378	-1	0.28%

24h

Total | INDEL	Ins	Del	HDR
150980 | 29.47%	8.57%	12.10%	8.80%

Typical seqs	Reads	Type	pct.
GCTGCGCGCGCGCCGAG|AAGAGGGGCG	106482	WT	70.53%
GCTGCGCGCGCGCCGAGGTTTAAACTACGC GTG	11963	+6 HDR	7.92%
GCTGCGCGCGCGCCGAGgAAGAGGGGCG	8783	+1 g	5.82%
GCT---------------------GCG	1717	-21	1.14%
GCT-----------------------G	1368	-23	0.91%
GCTGCGCGCGCGCC-----GAGGGGCG	1135	-5	0.75%
GCTGC-------------------GCG	1085	-19	0.72%
GCTGCGC-----------------GCG	969	-17	0.64%
GCTGCGCGCGCGCCGA-AAGAGGGGCG	768	-1	0.51%
GCTGCGCGCGCGCCG---AGAGGGGCG	766	-3	0.51%

48h

Total | INDEL	Ins	Del	HDR
107884 | 40.67%	11.16%	13.88%	15.64%

Typical seqs	Reads	Type	pct.
GCTGCGCGCGCGCCGAG|AAGAGGGGCG	64008	WT	59.33%
GCTGCGCGCGCGCCGAGGTTTAAACTACGC GTG	15280	+6 HDR	14.16%
GCTGCGCGCGCGCCGAGgAAGAGGGGCG	8625	+1 g	7.99%
GCT---------------------GCG	1488	-21	1.38%
GCTGCGCGCGCGCC-----GAGGGGCG	1104	-5	1.02%
GCTGCGCGCGCGCC--GAAGAGGGGCG	1071	-2	0.99%
GCTGCGCGC---------------GCG	1052	-15	0.98%
GCTGCGCGCGC------AAGAGGGGCG	734	-6	0.68%
GCTGCGCGCGCGC----AAGAGGGGCG	688	-4	0.64%
GCTGCGCGCGCGCCGAG-AGAGGGGCG	660	-1	0.61%

P118-YW-W9-Syn94crBCL11A7d-KO

Total | INDEL	4h	Ins	Del	HDR
62403 | 17.61%		14.60%	3.01%	0.00%

Typical seqs	Reads	Type	pct.
GCGTAGTGTTGGGTCCT|ACC**TGG**CCAC	51415	WT	82.39%
GCGTAGTGTTGGGTCCTtACCTGGCCAC	8061	+1 t	12.92%
GCGTAGTGTTGGGTCCTCTACCTGGCCAC	464	+2	0.74%
GCGTAGTGTTGGGT----CCTGGCCAC	437	-4	0.70%
GCGTAGTGTTGGGTCC-ACCTGGCCAC	419	-1	0.67%
GCGTAGTGTTGGGTC-TACCTGGCCAC	317	-1	0.51%
GCGTAGTGTTGGGTCCTaACCTGGCCAC	152	+1 a	0.24%
GCGTAGTGTTG----------GGCCAC	137	-10	0.22%
GCGTAGTGTTGGGTC--ACCTGGCCAC	128	-2	0.21%
GCGTAGTGTTGGGTCCT-CCTGGCCAC	90	-1	0.14%

Total | INDEL	8h	Ins	Del	HDR
59034 | 36.73%		30.02%	6.71%	0.00%

Typical seqs	Reads	Type	pct.
GCGTAGTGTTGGGTCCT|ACC**TGG**CCAC	37352	WT	63.27%
GCGTAGTGTTGGGTCCTtACCTGGCCAC	15416	+1 t	26.11%
GCGTAGTGTTGGGTCCTCTACCTGGCCAC	1244	+2	2.11%
GCGTAGTGTTGGGT----CCTGGCCAC	778	-4	1.32%
GCGTAGTGTTGGGT---ACCTGGCCAC	353	-3	0.60%
GCGTAGTGTTGGGTC--ACCTGGCCAC	248	-2	0.42%
GCGTAGTGTTGGGTCC-ACCTGGCCAC	236	-1	0.40%
GCGTAGTGT-----------TGGCCAC	226	-11	0.38%
GCGTAGTGTTGGGTC-TACCTGGCCAC	187	-1	0.32%
GCGTAGTGTTGGGTC-----------C	160	-11	0.27%

Total | INDEL	12h	Ins	Del	HDR
60406 | 45.72%		37.16%	8.57%	0.00%

Typical seqs	Reads	Type	pct.
GCGTAGTGTTGGGTCCT|ACC**TGG**CCAC	32787	WT	54.28%
GCGTAGTGTTGGGTCCTtACCTGGCCAC	19834	+1 t	32.83%
GCGTAGTGTTGGGTCCTCTACCTGGCCAC	1086	+2	1.80%
GCGTAGTGTTGGGT----CCTGGCCAC	1031	-4	1.71%
GCGTAGTGTTGGGTCC-ACCTGGCCAC	726	-1	1.20%
GCGTAGTGTTGGGTC--ACCTGGCCAC	311	-2	0.51%
GCGTAGTGTTGGGTC-TACCTGGCCAC	254	-1	0.42%
GCGTAGTGTTG----------GGCCAC	223	-10	0.37%
GCGTAGTGTTGGGT----------CAC	207	-10	0.34%
GCGTAGTGT-----------TGGCCAC	189	-11	0.31%

Total | INDEL	24h	Ins	Del	HDR
64608 | 57.43%		44.89%	12.54%	0.00%

Typical seqs	Reads	Type	pct.
GCGTAGTGTTGGGTCCT|ACC**TGG**CCAC	27504	WT	42.57%
GCGTAGTGTTGGGTCCTtACCTGGCCAC	25310	+1 t	39.17%
GCGTAGTGTTGGGT----CCTGGCCAC	1816	-4	2.81%
GCGTAGTGTTGGGTCCTCTACCTGGCCAC	1428	+2	2.21%
GCGTAGTGTTGGGTCC-ACCTGGCCAC	691	-1	1.07%
GCGTAGTGT-----------TGGCCAC	611	-11	0.95%
GCGTAGTGTTGGGTC-TACCTGGCCAC	552	-1	0.85%
GCGTAGTGTTGGGT---ACCTGGCCAC	435	-3	0.67%
GCGTAGTGTTGGGTC--ACCTGGCCAC	378	-2	0.59%
GCGTAGTGTTGGGTC-----------C	337	-11	0.52%

Total | INDEL	48h	Ins	Del	HDR
48972 | 62.97%		46.96%	16.02%	0.00%

Typical seqs	Reads	Type	pct.
GCGTAGTGTTGGGTCCT|ACC**TGG**CCAC	18134	WT	37.03%
GCGTAGTGTTGGGTCCTtACCTGGCCAC	20076	+1 t	40.99%
GCGTAGTGTTGGGT----CCTGGCCAC	1963	-4	4.01%
GCGTAGTGTTGGGTCCTCTACCTGGCCAC	1125	+2	2.30%
GCGTAGTGT-----------TGGCCAC	676	-11	1.38%
GCGTAGTGTTGGGTCC-ACCTGGCCAC	522	-1	1.07%
GCGTAGTGTTGGGTC-TACCTGGCCAC	460	-1	0.94%
GCGTAGTGTTGGGTC--ACCTGGCCAC	340	-2	0.69%
GCGTAGTGTTGGGT----------CAC	330	-10	0.67%
GCGTAGTGTTGGGTC-----------C	298	-11	0.61%

P121-YW-W9-Syn47crHBG2-g-KO

4h

Total | INDEL	Ins	Del	HDR
47128 | 1.97%	0.77%	1.20%	0.00%

Typical seqs	Reads	Type	pct.
GCATTGAGATAGTGTGG|GGA**AGG**GGCC	46198	WT	98.03%
GCATTGAGATAGTGTGGgGGAAGGGGCC	269	+1 g	0.57%
GCATTGAGATAGTGTG-GGAAGGGGCC	251	-1	0.53%
GCATTGAGATAGTGTGGGG-AGGGGCC	57	-1	0.12%
GCATTGAGATAGGTTT--------------<br>-AAAC	52	-7	0.11%
GCATTGAGATAGTGTG--GAAGGGGCC	41	-2	0.09%
GCATTGAGATAGTGTGGGG-AGGGGGC	24	-1	0.05%
GCATTGAGATAGTGT------------	22	-12	0.05%
GCATTGAGATAGTTGTGGGGAAGGGGCC	18	+1 Ins	0.04%
GCATTGAGATAGT----GGAAGGGGCC	16	-4	0.03%

8h

Total | INDEL	Ins	Del	HDR
18747 | 6.11%	4.05%	2.06%	0.00%

Typical seqs	Reads	Type	pct.
GCATTGAGATAGTGTGG|GGA**AGG**GGCC	17601	WT	93.89%
GCATTGAGATAGTGTGGgGGAAGGGGCC	683	+1 g	3.64%
GCATTGAGATAGTGTG-GGAAGGGGCC	90	-1	0.48%
GCATTGAGATAGTGTG--GAAGGGGCC	92	-2	0.49%
GCATTGAGATAGTGT-GCAAAGGGGCC	58	-1	0.31%
GCATTGAGATAGT----GGAAGGGGCC	48	-4	0.26%
GCATTGAGATAGTGTGGGG-AGGGGCC	31	-1	0.17%
GCATTGAGATAGGTTT--------------<br>-AAAC	23	-7	0.12%
GCATTGAGACAGTGTGGGGGAAGGGGCC	13	+1 Ins	0.07%
GCATTGAGATAGTGT------------	9	-12	0.05%

12h

Total | INDEL	Ins	Del	HDR
29827 | 12.59%	8.15%	4.44%	0.00%

Typical seqs	Reads	Type	pct.
GCATTGAGATAGTGTGG|GGA**AGG**GGCC	26072	WT	87.41%
GCATTGAGATAGTGTGGgGGAAGGGGCC	2044	+1 g	6.85%
GCATTGAGATAGTGTG-GGAAGGGGCC	701	-1	2.35%
GCATTGAGATAGTGTGGGGGGAAGGGGCC	160	+2	0.54%
GCATTGAGATAGTGTGG----------	92	-12	0.31%
GCATTGAGATAGTGT--------GGCC	77	-8	0.26%
GCATT-------------GAAGGGGCC	70	-13	0.23%
GCATTGAGATAGTGTG-----GGGGCC	76	-5	0.25%
GCATTGAGATAGTGTGGGG-AGGGGGC	34	-1	0.11%
GCATTGAGATAGTGTGGGG-AGGGGCC	33	-1	0.11%

24h

Total | INDEL	Ins	Del	HDR
48584 | 15.28%	7.76%	7.51%	0.00%

Typical seqs	Reads	Type	pct.
GCATTGAGATAGTGTGG|GGA**AGG**GGCC	41161	WT	84.72%
GCATTGAGATAGTGTGGgGGAAGGGGCC	2729	+1 g	5.62%
GCATTGAGATAGTGTG-GGAAGGGGCC	1402	-1	2.89%
GCATTGAGATAGTGTG--GAAGGGGCC	559	-2	1.15%
GCATTGAGATAGTGT-------GGGCC	337	-7	0.69%
GCATTGAGATAGTGTGGTGGGGAAGGGGCC	231	+3	0.48%
GCATTG--------------------C	202	-20	0.42%
GCATTGAGATAGTGT---GAAGGGGCC	179	-3	0.37%
GCATTGA---------GGGAAGGGGCC	157	-9	0.32%
GCATTGAGATAGTGTGGAGGGAAGGGGCC	140	+2	0.29%

48h

Total | INDEL	Ins	Del	HDR
47284 | 25.88%	16.74%	9.15%	0.00%

Typical seqs	Reads	Type	pct.
GCATTGAGATAGTGTGG|GGA**AGG**GGCC	35045	WT	74.12%
GCATTGAGATAGTGTGGgGGAAGGGGCC	5807	+1 g	12.28%
GCATTGAGATAGTGTG--GAAGGGGCC	1071	-2	2.27%
GCATTGAGATAGTGTG-GGAAGGGGCC	740	-1	1.57%
GCATTGAGATAGT----GGAAGGGGCC	352	-4	0.74%
GCATTGAGATAGTGTGG---AGGGGCC	322	-3	0.68%
GCATTGAGATAGTGTGGtGGAAGGGGCC	323	+1 t	0.68%
GCATTGAGATAGTGTG-----GGGGCC	318	-5	0.67%
GCATTGAGATAGT-------------C	310	-13	0.66%
GCATTGAGATAGTGTGGcGGAAGGGGCC	252	+1 c	0.53%

P123-P127-YW-W9-RNP-Syn51GATA4-KO

Total | INDEL	4h	Ins	Del	HDR
8521 | 3.23%		1.35%	1.88%	0.00%

Typical seqs	Reads	Type	pct.
TTGGAACAGCCTGGTCT|TCT**TGG**CCGA	8246	WT	96.77%
TTGGAACAGCCTGGGTCTTGGCCGA	90	+1 Ins	1.06%
TTGGAACAGCCT-GTCTTGGCCGA	50	-1	0.59%
TTGGAACAGCCT--TCTTGGCCGA	49	-2	0.58%
TTGGAACAGCCTGG--TTGGCCGA	32	-2	0.38%
TTGGAACAGCC------TGGCCGA	29	-6	0.34%
TTGGAACAGCCTGGTTCTTGGCCGA	25	+1 Ins	0.29%

Total | INDEL	8h	Ins	Del	HDR
15941 | 3.26%		1.29%	1.96%	0.00%

Typical seqs	Reads	Type	pct.
TTGGAACAGCCTGGTCT|TCT**TGG**CCGA	15422	WT	96.74%
TTGGAACAGCCTGGGTCTTGGCCGA	111	+1 Ins	0.70%
TTGGAACAGCCT-GTCTTGGCCGA	103	-1	0.65%
TTGGAACAGCCTGGTTCTTGGCCGA	85	+1 Ins	0.53%
TTGGAACAGCCTGG-CTTGGCCGA	52	-1	0.33%
TTGGAACA------TCTTGGCCGA	33	-6	0.21%
TTGGAACAGCCTGG-------CGA	19	-7	0.12%
TTGGAACAGCCTG-----GGCCGA	18	-5	0.11%
TTGGAACAGCC------TGGCCGA	17	-6	0.11%
TTGGAACAGCC---TCTTGGCCGA	15	-3	0.09%

Total | INDEL	12h	Ins	Del	HDR
16385 | 11.26%		2.45%	8.81%	0.00%

Typical seqs	Reads	Type	pct.
TTGGAACAGCCTGGTCT|TCT**TGG**CCGA	14540	WT	88.74%
TTGGAACAGCCT-GTCTTGGCCGA	306	-1	1.87%
TTGGAACAGCC------TGGCCGA	176	-6	1.07%
TTGGAACAGCCTGG-------CGA	175	-7	1.07%
TTGGAACAGCCTGG-CTTGGCCGA	162	-1	0.99%
TTGGAACAGCCTGGGTCTTGGCCGA	155	+1 Ins	0.95%
TTGGAACAGCCTGGTTCTTGGCCGA	103	+1 Ins	0.63%
TTGGAACAGCCTG-----GGCCGA	69	-5	0.42%
TTGGAACAGCCT--TCTTGGCCGA	59	-2	0.36%
TTGGAACAG------CTTGGCCGA	58	-6	0.35%

Total | INDEL	24h	Ins	Del	HDR
23924 | 14.10%		2.92%	11.18%	0.00%

Typical seqs	Reads	Type	pct.
TTGGAACAGCCTGGTCT|TCT**TGG**CCGA	20551	WT	85.90%
TTGGAACAGCCT-GTCTTGGCCGA	715	-1	2.99%
TTGGAACAGCC------TGGCCGA	411	-6	1.72%
TTGGAACAGCCTGGGTCTTGGCCGA	262	+1 Ins	1.10%
TTGGAACAGCCTGG-------CGA	188	-7	0.79%
TTGGAACAGCCTGGTTCTTGGCCGA	174	+1 Ins	0.73%
TTGGAACAGCCTGG-CTTGGCCGA	146	-1	0.61%
TTGGAACAGCCTGGATCTTGGCCGA	123	+1 Ins	0.51%
TTGGAACA-----GTCTTGGCCGA	96	-5	0.40%
TTGGAAC-------TCTTGGCCGA	96	-7	0.40%

Total | INDEL	48h	Ins	Del	HDR
68580 | 18.08%		3.01%	15.07%	0.00%

Typical seqs	Reads	Type	pct.
TTGGAACAGCCTGGTCT|TCT**TGG**CCGA	56184	WT	81.92%
TTGGAACAGCC------TGGCCGA	3129	-6	4.56%
TTGGAACAGCCT-GTCTTGGCCGA	1723	-1	2.51%
TTGGAACA-----------GCCGA	768	-11	1.12%
TTGGAACAGCCTGG-CTTGGCCGA	583	-1	0.85%
TTGGAACAGCCTGG-------CGA	570	-7	0.83%
TTGGAACAGCCTGGTTCTTGGCCGA	404	+1 Ins	0.59%
TTGGAACAGCCTGGGTCTTGGCCGA	406	+1 Ins	0.59%
TTGGAACAGC-----CTTGGCCGA	313	-5	0.46%
TTGGAACAG------CTTGGCCGA	226	-6	0.33%

P123-P127-YW-W9-RNP-Syn52GATA4-KO

Total | INDEL	4h	Ins	Del	HDR
7243 | 5.48%		3.00%	2.49%	0.00%

Typical seqs	Reads	Type	pct.
TCAAATTCCTGCACGGA|CCT**GGG**ACTT	6846	WT	94.52%
TCAAATTCCTGCACGGAaCCTGGGACTT	159	+1 a	2.20%
TCAAATTCCTGCACGG-CCTGGGACTT	29	-1	0.40%
TCAAATTCCTGCACGGA----------	28	-10	0.39%
TCAAATTCCTGCAC-------GGACTT	23	-7	0.32%
TCAAATTCCTGCACGGAgCCTGGGACTT	22	+1 g	0.30%
TCAAATTCCTGCACGGA-CTGGGACTT	22	-1	0.30%
TCAAATTCCTGCACGGAtCCTGGGACTT	21	+1 t	0.29%
TCAAATTCCTGCACGGA--TGGGACTT	13	-2	0.18%
TCAAATT----------CCTGGGACTT	10	-10	0.14%

Total | INDEL	8h	Ins	Del	HDR
18830 | 8.59%		4.14%	4.45%	0.00%

Typical seqs	Reads	Type	pct.
TCAAATTCCTGCACGGA|CCT**GGG**ACTT	17212	WT	91.41%
TCAAATTCCTGCACGGAaCCTGGGACTT	657	+1 a	3.49%
TCAAATTCCTGCACGGA-CTGGGACTT	223	-1	1.18%
TCAAATT----------CCTGGGACTT	108	-10	0.57%
TCAAATTCCTGCACGG-CCTGGGACTT	81	-1	0.43%
TCAAATTCCTGCACGGA--TGGGACTT	66	-2	0.35%
TCAAATTCCTGCAC-------GGACTT	62	-7	0.33%
TCAAATTCCTG------CCTGGGACTT	43	-6	0.23%
TCAAATTCCTGCAC------GGGACTT	36	-6	0.19%
TCAAATTCCTGCACGGA---GGGACTT	32	-3	0.17%

Total | INDEL	12h	Ins	Del	HDR
9002 | 20.20%		7.20%	13.00%	0.00%

Typical seqs	Reads	Type	pct.
TCAAATTCCTGCACGGA|CCT**GGG**ACTT	7184	WT	79.80%
TCAAATTCCTGCACGGAaCCTGGGACTT	537	+1 a	5.97%
TCAAATT----------CCTGGGACTT	194	-10	2.16%
TCAAATTCCTGCACGGA-CTGGGACTT	168	-1	1.87%
TCAAATTCCTGCAC-------GGACTT	120	-7	1.33%
TCAAATTCCTGCACGG-CCTGGGACTT	118	-1	1.31%
TCAAATTC---------CCTGGGACTT	67	-9	0.74%
TCAAATTCCTGCACGGA--TGGGACTT	65	-2	0.72%
TCAAATTCCTG------CCTGGGACTT	55	-6	0.61%
TCAAATTCCTGCACGGAtCCTGGGACTT	46	+1 t	0.51%

Total | INDEL	24h	Ins	Del	HDR
20380 | 22.47%		6.83%	15.64%	0.00%

Typical seqs	Reads	Type	pct.
TCAAATTCCTGCACGGA|CCT**GGG**ACTT	15800	WT	77.53%
TCAAATTCCTGCACGGAaCCTGGGACTT	954	+1 a	4.68%
TCAAATT----------CCTGGGACTT	762	-10	3.74%
TCAAATTCCTGCAC-------GGACTT	543	-7	2.66%
TCAAATTCCTGCACGGA-CTGGGACTT	354	-1	1.74%
TCAAATTCCTGCACGG-CCTGGGACTT	203	-1	1.00%
TCAAATTCCTGCACGGAtCCTGGGACTT	106	+1 t	0.52%
TCAAATTCCTG------CCTGGGACTT	100	-6	0.49%
TCAAATTCCTGCACGGA--TGGGACTT	102	-2	0.50%
TCAAATTCCTGCAC------GGGACTT	90	-6	0.44%

Total | INDEL	48h	Ins	Del	HDR
40076 | 22.40%		6.14%	16.26%	0.00%

Typical seqs	Reads	Type	pct.
TCAAATTCCTGCACGGA|CCT**GGG**ACTT	31097	WT	77.60%
TCAAATT----------CCTGGGACTT	1758	-10	4.39%
TCAAATTCCTGCACGGAaCCTGGGACTT	1567	+1 a	3.91%
TCAAATTCCTGCAC-------GGACTT	1322	-7	3.30%
TCAAATTCCTGCACGGA-CTGGGACTT	408	-1	1.02%
TCAAATTCCTGCAC------GGGACTT	325	-6	0.81%
TCAAATTCCTGCACGG-CCTGGGACTT	252	-1	0.63%
TCAAATTCCTG------CCTGGGACTT	198	-6	0.49%
TCAAATTCCTGCACGGAtCCTGGGACTT	150	+1 t	0.37%
TCAAATTCCTGC-----------ACTT	146	-11	0.36%

P123-P127-YW-W9-RNP-Syn53GATA4-KO

Total | INDEL	4h	Ins	Del	HDR
10210 | 1.55%		0.39%	1.16%	0.00%

Typical seqs	Reads	Type	pct.
CTCAAATTCCTGCACGG|ACC**TGG**GACT	10052	WT	98.45%
CTCAAATTCCTGCAC-GACCTGGGACT	46	-1	0.45%
CTCAAATTCCTGCACGGgACCTGGGACT	27	+1 g	0.26%
CTCAAATTCCTGCACGG-CCTGGGACT	19	-1	0.19%
CTCAAATTCCTGCACGG--CTGGGACT	16	-2	0.16%
CTCAAATTCCTGCAC--ACCTGGGACT	13	-2	0.13%
CTCAAATTCCTGCAC-------GGACT	13	-7	0.13%
CTCAAATTCCTGCACG--CCTGGGACT	8	-2	0.08%
CTCAAATTCCTGCACGGACTCAAATTCCTG GGACT	7	+8	0.07%
CTCAAATTCCTGCACGGaACCTGGGACT	6	+1 a	0.06%

Total | INDEL	8h	Ins	Del	HDR
20350 | 2.27%		0.41%	1.86%	0.00%

Typical seqs	Reads	Type	pct.
CTCAAATTCCTGCACGG|ACC**TGG**GACT	19888	WT	97.73%
CTCAAATTCCTGC----ACCTGGGACT	70	-4	0.34%
CTCAAATTCCTGCAC------GGGACT	57	-6	0.28%
CTCAAATTCCTGCAC-GACCTGGGACT	60	-1	0.29%
CTCAAATTCCTGCACGGgACCTGGGACT	39	+1 g	0.19%
CTCAAATTCCTGCACGG-CCTGGGACT	38	-1	0.19%
CTCAAATT----------CCTGGGACT	28	-10	0.14%
CTCAAATTCCTGCACGGaACCTGGGACT	18	+1 a	0.09%
CTCAAATTCCTGCACGG--CTGGGACT	16	-2	0.08%
CTCAAA----------AACCTGGGACT	16	-10	0.08%

Total | INDEL	12h	Ins	Del	HDR
14460 | 5.53%		0.58%	4.95%	0.00%

Typical seqs	Reads	Type	pct.
CTCAAATTCCTGCACGG|ACC**TGG**GACT	13660	WT	94.47%
CTCAAATTCCTGCAC-GACCTGGGACT	100	-1	0.69%
CTCAAATTCCTGC----ACCTGGGACT	86	-4	0.59%
CTCAAATT----------CCTGGGACT	84	-10	0.58%
CTCAAATTCCTGCAC--ACCTGGGACT	54	-2	0.37%
CTCAAATTCCTGCACGG-CCTGGGACT	50	-1	0.35%
CTCAAATTCCTGCAC------GGGACT	43	-6	0.30%
CTCAAATTCCTGCACGGaACCTGGGACT	37	+1 a	0.26%
CTCAAATTCCTGCAC-------GGACT	35	-7	0.24%
CTCAAATTCCTGCACGG--CTGGGACT	29	-2	0.20%

Total | INDEL	24h	Ins	Del	HDR
13384 | 6.26%		0.96%	5.30%	0.00%

Typical seqs	Reads	Type	pct.
CTCAAATTCCTGCACGG|ACC**TGG**GACT	12546	WT	93.74%
CTCAAATTCCTGC----ACCTGGGACT	105	-4	0.78%
CTCAAATT----------CCTGGGACT	77	-10	0.58%
CTCAAAT----------ACCTGGGACT	62	-10	0.46%
CTCAAATTCCTGCAC-GACCTGGGACT	59	-1	0.44%
CTCAAATTCCTGCAC------GGGACT	52	-6	0.39%
CTCAAATTCCTGCACGGgACCTGGGACT	50	+1 g	0.37%
CTCAAATTCCTGCACGGaACCTGGGACT	46	+1 a	0.34%
CTCAAATTCCTGCAC--ACCTGGGACT	37	-2	0.28%
CTCAAATTCC-------ACCTGGGACT	34	-7	0.25%

Total | INDEL	48h	Ins	Del	HDR
81478 | 7.87%		1.02%	6.85%	0.00%

Typical seqs	Reads	Type	pct.
CTCAAATTCCTGCACGG|ACC**TGG**GACT	75064	WT	92.13%
CTCAAATT----------CCTGGGACT	984	-10	1.21%
CTCAAATTCCTGC----ACCTGGGACT	921	-4	1.13%
CTCAAATTCCTGCAC-GACCTGGGACT	580	-1	0.71%
CTCAAATTCCTGCAC------GGGACT	405	-6	0.50%
CTCAAATTCCTGCAC-------GGACT	277	-7	0.34%
CTCAAATTCCTGCACGG-CCTGGGACT	198	-1	0.24%
CTCAAATTCCTGCACGGgACCTGGGACT	189	+1 g	0.23%
CTCAAATTCCTGCAC--ACCTGGGACT	169	-2	0.21%
CTCAAATTCCTGCACGGaACCTGGGACT	164	+1 a	0.20%

P123-P127-YW-W9-RNP-Syn54GATA4-KO

Total | INDEL	4h	Ins	Del	HDR
14409 | 18.85%		18.49%	0.36%	0.00%

Typical seqs	Reads	Type	pct.
TCCCTCCTCAAATTCCT|GCA**CGG**ACCT	11693	WT	81.15%
TCCCTCCTCAAATTCCTtGCACGGACCT	2238	+1 t	15.53%
TCCCTCCTCAAATTCCTCTGCACGGACCT	380	+2	2.64%
TCCCTCCTCAA-----TGCACGGACCT	16	-5	0.11%
TCCCTCCTCAAATTCC-GCACGGACCT	10	-1	0.07%
TCCCTCCTCAAATTC--GCACGGACCT	10	-2	0.07%
TCCCTCCTCAAATT------CGGACCT	9	-6	0.06%
TCCCTCCTCAAATTCCTATGCACGGACCT	7	+2	0.05%
TCCCCCCTCAAATTCCTTGCACGGACCT	6	+1 Ins	0.04%
TCCCTCCTCAAATTCCTtACACGGACCT	4	+1 t	0.03%

Total | INDEL	8h	Ins	Del	HDR
20873 | 27.68%		26.52%	1.15%	0.00%

Typical seqs	Reads	Type	pct.
TCCCTCCTCAAATTCCT|GCA**CGG**ACCT	15096	WT	72.32%
TCCCTCCTCAAATTCCTtGCACGGACCT	4426	+1 t	21.20%
TCCCTCCTCAAATTCCTCTGCACGGACCT	803	+2	3.85%
TCCCTCCTCAAATT----------CCT	60	-10	0.29%
TCCCTCCTCAAATTCCCTGCACGGACCT	50	+1 Ins	0.24%
TCCCTCCTCAAATTCC-GCACGGACCT	38	-1	0.18%
TCCCTCCTCAAAT----GCACGGACCT	21	-4	0.10%
TCCCTCCTCAAATTCCTACTGCACGGACCT	20	+3	0.10%
TCCCTCCTCAAATTCCTTTGCACGGACCT	18	+2	0.09%
TCCCTCCTCAAATTCCTATGCACGGACCT	16	+2	0.08%

Total | INDEL	12h	Ins	Del	HDR
10561 | 36.75%		33.55%	3.20%	0.00%

Typical seqs	Reads	Type	pct.
TCCCTCCTCAAATTCCT|GCA**CGG**ACCT	6680	WT	63.25%
TCCCTCCTCAAATTCCTtGCACGGACCT	3083	+1 t	29.19%
TCCCTCCTCAAATTCCTCTGCACGGACCT	320	+2	3.03%
TCCCTCCTCAAATTC-TGCACGGACCT	79	-1	0.75%
TCCCTCCTCAAATT----------CCT	49	-10	0.46%
TCCCTCCT---------GCACGGACCT	24	-9	0.23%
TCCCTCCTCAAATTCCTTTGCACGGACCT	19	+2	0.18%
TCCCTCCTCAAATTCCCTGCACGGACCT	19	+1 Ins	0.18%
TCCCT-------------CACG--CGTAGTTTA	14	-9	0.13%
TCCCTCCTCAAATT----CACGGACCT	14	-4	0.13%

Total | INDEL	24h	Ins	Del	HDR
22759 | 49.54%		42.61%	6.92%	0.00%

Typical seqs	Reads	Type	pct.
TCCCTCCTCAAATTCCT|GCA**CGG**ACCT	11485	WT	50.46%
TCCCTCCTCAAATTCCTtGCACGGACCT	8049	+1 t	35.37%
TCCCTCCTCAAATTCCTCTGCACGGACCT	893	+2	3.92%
TCCCTCCTCAAATT----------CCT	209	-10	0.92%
TCCCTCCTCAAATTC-TGCACGGACCT	96	-1	0.42%
TCCCTCCTCAAATTCCCTGCACGGACCT	86	+1 Ins	0.38%
TCCCT-------------CACG--CGTAGTTTA	72	-9	0.32%
TCCCT-------------------CCT	66	-19	0.29%
TCCCTCCTCAAAT----GCACGGACCT	54	-4	0.24%
TCCCTCCTCAAATT---GCACGGACCT	51	-3	0.22%

Total | INDEL	48h	Ins	Del	HDR
71602 | 49.59%		39.68%	9.91%	0.00%

Typical seqs	Reads	Type	pct.
TCCCTCCTCAAATTCCT|GCA**CGG**ACCT	36095	WT	50.41%
TCCCTCCTCAAATTCCTtGCACGGACCT	23509	+1 t	32.83%
TCCCTCCTCAAATTCCTCTGCACGGACCT	2565	+2	3.58%
TCCCTCCTCAAATT----------CCT	1078	-10	1.51%
TCCCT-------------CACG--CGTAGTTTA	638	-9	0.89%
TCCCTCCTCAAATTC-TGCACGGACCT	297	-1	0.41%
TCCCTCCT----------CACGGACCT	271	-10	0.38%
TCCCTCCTCAAATTCCCTGCACGGACCT	216	+1 Ins	0.30%
TCCCTCCT---------GCACGGACCT	196	-9	0.27%
TCCCTCCTCAAATTC---CACGGACCT	184	-3	0.26%

P123-P127-YW-W9-RNP-Syn55GATA4-KO

Total | INDEL	4h	Ins	Del	HDR
12348 | 3.13%		2.10%	1.04%	0.00%

Typical seqs	Reads	Type	pct.
TCCAAGTCCCAGGTCCG|TGCAGGAATT	11961	WT	96.87%
TCCAAGTCCCAGGTCCGtTGCAGGAATT	169	+1 t	1.37%
TCCAAGTCCCAGGTCC-TGCAGGAATT	61	-1	0.49%
TCCAAGTCCCAGGTCCG-GCAGGAATT	29	-1	0.23%
TCCAAGTCCCAGGTCCGtGCAAGGAATT	25	+1 t	0.20%
TCCAAGTCCCAGGTCCGTTTGCAGGAATT	21	+2	0.17%
TCCAAGTCCCAGGTCC--GCAGGAATT	18	-2	0.15%
TCCAAGTCCCAGGTCCGCGTGCAGGAATT	15	+2	0.12%
TCCAAGTCCCAGGTCCGaTGCAGGAATT	15	+1 a	0.12%
TCCAAGTCCCAGGT---TGCAGGAATT	13	-3	0.11%

Total | INDEL	8h	Ins	Del	HDR
24985 | 5.70%		2.99%	2.67%	0.04%

Typical seqs	Reads	Type	pct.
TCCAAGTCCCAGGTCCG|TGCAGGAATT	23561	WT	94.30%
TCCAAGTCCCAGGTCCGtTGCAGGAATT	382	+1 t	1.53%
TCCAAGTCCCAGGTCCG-GCAGGAATT	114	-1	0.46%
TCCAAGTCCCAGGTCC--GCAGGAATT	90	-2	0.36%
TCCAAGTCCCAGGTCCGtGCAAGGAATT	73	+1 t	0.29%
TCCAAGTCCCAGGTCC-TGCAGGAATT	70	-1	0.28%
TCCAAGTCCCAGGTCCGaTGCAGGAATT	67	+1 a	0.27%
TCCAAGTCCCAGGTCCGgTGCAGGAATT	67	+1 g	0.27%
TCCAAGTCCCAGGTCCGCGTGCAGGAATT	55	+2	0.22%
TCCAAGTCCCAGGT---TGCAGGAATT	40	-3	0.16%

Total | INDEL	12h	Ins	Del	HDR
12287 | 10.52%		5.57%	4.82%	0.12%

Typical seqs	Reads	Type	pct.
TCCAAGTCCCAGGTCCG|TGCAGGAATT	10995	WT	89.48%
TCCAAGTCCCAGGTCCGtTGCAGGAATT	355	+1 t	2.89%
TCCAAGTCCCAGGTCCG-GCAGGAATT	117	-1	0.95%
TCCAAGTCCCAGGTCCGCGTGCAGGAATT	94	+2	0.77%
TCCAAGTCCCAGGTC--TGCAGGAATT	65	-2	0.53%
TCCAAGTCCCAGGTCC--GCAGGAATT	67	-2	0.55%
TCCAAGTCC----------CAGGAATT	49	-10	0.40%
TCCAAGTCCCAGGTCCGgTGCAGGAATT	48	+1 g	0.39%
TCCAAGTCCCAGGTCC-TGCAGGAATT	46	-1	0.37%
TCCAAGTCCCAGGTCCGtGCAAGGAATT	42	+1 t	0.34%

Total | INDEL	24h	Ins	Del	HDR
17708 | 16.51%		7.74%	8.34%	0.43%

Typical seqs	Reads	Type	pct.
TCCAAGTCCCAGGTCCG|TGCAGGAATT	14785	WT	83.49%
TCCAAGTCCCAGGTCCGtTGCAGGAATT	715	+1 t	4.04%
TCCAAGTCC----------CAGGAATT	223	-10	1.26%
TCCAAGTCCCAGGTCCG-GCAGGAATT	214	-1	1.21%
TCCAAGTCCCAGGTCC-TGCAGGAATT	189	-1	1.07%
TCCAAGTCCCAGGTCC--GCAGGAATT	144	-2	0.81%
TCCAAGTCCCAGGTCCGgTGCAGGAATT	141	+1 g	0.80%
TCCAAGTCCCAGGTCCGCGTGCAGGAATT	107	+2	0.60%
TCCAAGTCCCAGGTCCGtGCAAGGAATT	102	+1 t	0.58%
TCCAAGTCCCAGGTCCGaTGCAGGAATT	101	+1 a	0.57%

Total | INDEL	48h	Ins	Del	HDR
60209 | 18.43%		6.91%	10.60%	0.92%

Typical seqs	Reads	Type	pct.
TCCAAGTCCCAGGTCCG|TGCAGGAATT	49114	WT	81.57%
TCCAAGTCCCAGGTCCGtTGCAGGAATT	1754	+1 t	2.91%
TCCAAGTCC----------CAGGAATT	1742	-10	2.89%
TCCAAGT-----GTTTAAACTACGCGT--- ------	553	-9 HDR	0.92%
TCCAAGTCCCAGGTCC-TGCAGGAATT	553	-1	0.92%
TCCAAGTCCCAGGTCCG-GCAGGAATT	552	-1	0.92%
TCCAAGTCCCAGGTCCGtGCAAGGAATT	536	+1 t	0.89%
TCCAA-----------GTGCAGGAATT	343	-11	0.57%
TCCAAGTCCCAGGTCC--GCAGGAATT	277	-2	0.46%
TCCAAGTCCCAGGTCCGTTTGCAGGAATT	264	+2	0.44%

P123-P127-YW-W9-RNP-Syn56GATA4-KO

Total | INDEL	4h	Ins	Del	HDR
12346 | 11.23%		10.68%	0.54%	0.00%

Typical seqs	Reads	Type	pct.
CAGGTCCGTGCAGGAAT|TTG**AGG**AGGG	10960	WT	88.77%
CAGGTCCGTGCAGGAATtTTGAGGAGGG	1158	+1 t	9.38%
CAGGTCCGTGCAGGAATTTTGAGGAGGG	82	+2	0.66%
CAGGTCCGTGCAGGAATATTTGAGGAGGG	25	+2	0.20%
CAGGTCCGTGCAGG--TTTGAGGAGGG	24	-2	0.19%
CAGGTCCGTGCAGGAATtTGAAGGAGGG	22	+1 t	0.18%
CAGGTCCGTGCAGGAATTTTTTGAGGAGGG	16	+3	0.13%
C-------------TTGATGAGGAGGG	13	-13	0.11%
CAGGTCCGTGCAGAAATTTTGAGGAGGG	9	+1 Ins	0.07%
CAGGTCCGTGCAGGAA----AGGAGGG	9	-4	0.07%

Total | INDEL	8h	Ins	Del	HDR
35633 | 21.43%		19.44%	1.99%	0.00%

Typical seqs	Reads	Type	pct.
CAGGTCCGTGCAGGAAT|TTG**AGG**AGGG	27997	WT	78.57%
CAGGTCCGTGCAGGAATtTTGAGGAGGG	6077	+1 t	17.05%
CAGGTCCGTGCAGGAATATTTGAGGAGGG	261	+2	0.73%
CAGGTCCGTGCAGGAA-TTGAGGAGGG	210	-1	0.59%
CAGGTCCGTGCAGGAATTTTTGAGGAGGG	132	+2	0.37%
CAGGTCCGTGCAGGAATtTGAAGGAGGG	78	+1 t	0.22%
CAGGTCCGTGC---------AGGAGGG	77	-9	0.22%
CAGGTCCGTGCAGGAATT------GGG	56	-6	0.16%
CAGGTCCGTGCAGG-ATTTGAGGAGGG	55	-1	0.15%
CAGGTC-----------TTGAGGAGGG	52	-11	0.15%

Total | INDEL	12h	Ins	Del	HDR
19867 | 29.12%		22.99%	6.13%	0.00%

Typical seqs	Reads	Type	pct.
CAGGTCCGTGCAGGAAT|TTG**AGG**AGGG	14081	WT	70.88%
CAGGTCCGTGCAGGAATtTTGAGGAGGG	3827	+1 t	19.26%
CAGGTCCGTGCAGGAATTTTTGAGGAGGG	246	+2	1.24%
CAGGTCCGTGCAGGAA-TTGAGGAGGG	217	-1	1.09%
CAGGTCCGTGC---------AGGAGGG	119	-9	0.60%
CAGGTCCGTGCA-------------GG	118	-13	0.59%
CAGGTCCGTGCAGG-ATTTGAGGAGGG	102	-1	0.51%
CAGGTCCGTGCAGGAATATTTGAGGAGGG	71	+2	0.36%
CAGGTCCGTGCAGG--TTTGAGGAGGG	74	-2	0.37%
CAGGTCCGTGCAGGAATaTTGAGGAGGG	62	+1 a	0.31%

Total | INDEL	24h	Ins	Del	HDR
24296 | 37.15%		26.60%	10.54%	0.00%

Typical seqs	Reads	Type	pct.
CAGGTCCGTGCAGGAAT|TTG**AGG**AGGG	15271	WT	62.85%
CAGGTCCGTGCAGGAATtTTGAGGAGGG	5513	+1 t	22.69%
CAGGTCCGTGC---------AGGAGGG	446	-9	1.84%
CAGGTCCGTGCAGGAA-TTGAGGAGGG	331	-1	1.36%
CAGGTCCGTGCAGGAATTTTTGAGGAGGG	267	+2	1.10%
CAGGTCCG----------TGAGGAGGG	226	-10	0.93%
CAGGTCCGTGCAGG-ATTTGAGGAGGG	166	-1	0.68%
CAGGTCCGTGCA-------------GG	144	-13	0.59%
CAGGTCCGTGCAGGAATATTTGAGGAGGG	140	+2	0.58%
CAGGTCCGTGCAGGAAATTTGAGGAGGG	109	+1 Ins	0.45%

Total | INDEL	48h	Ins	Del	HDR
100652 | 37.41%		23.02%	14.39%	0.00%

Typical seqs	Reads	Type	pct.
CAGGTCCGTGCAGGAAT|TTG**AGG**AGGG	63003	WT	62.59%
CAGGTCCGTGCAGGAATtTTGAGGAGGG	18842	+1 t	18.72%
CAGGTCCGTGC---------AGGAGGG	3949	-9	3.92%
CAGGTCCG----------TGAGGAGGG	1926	-10	1.91%
CAGGTCCGTGCAGGAA-TTGAGGAGGG	1356	-1	1.35%
CAGGTCCGTGCAGGAATTTTTGAGGAGGG	1221	+2	1.21%
CAGGTCCGTGCAGGAATATTTGAGGAGGG	633	+2	0.63%
CAGGTCCGTGC------------AGGG	574	-12	0.57%
CAGGTCCGTGCAGGAATtTGAAGGAGGG	542	+1 t	0.54%
CAGGTCCGTGCA-------------GG	535	-13	0.53%

P123-P127-YW-W9-RNP-Syn57GATA4-KO

Total | INDEL	4h	Ins	Del	HDR	Total | INDEL	8h	Ins	Del	HDR	Total | INDEL	12h	Ins	Del	HDR
17026 | 4.18%		**0.70%**	**3.47%**	**0.00%**	37677 | 7.01%		**1.18%**	**5.83%**	**0.00%**	27373 | 7.93%		**1.07%**	**6.86%**	**0.00%**

Typical seqs	Reads	Type	pct.	Typical seqs	Reads	Type	pct.	Typical seqs	Reads	Type	pct.
GTCCGTGCAGGAATTTG|AGG**AGG**GAAG	16315	WT	95.82%	GTCCGTGCAGGAATTTG|AGG**AGG**GAAG	35037	WT	92.99%	GTCCGTGCAGGAATTTG|AGG**AGG**GAAG	25203	WT	92.07%
GTCCGTGCAGGAATTTG-GGAGGGAAG	346	-1	2.03%	GTCCGTGCAGGAATTTG-GGAGGGAAG	902	-1	2.39%	GTCCGTGCAGGAATTTG-GGAGGGAAG	419	-1	1.53%
GTCCGTGC---------AGGAGGGAAG	103	-9	0.60%	GTCCGTGC---------AGGAGGGAAG	516	-9	1.37%	GTCCGTGC---------AGGAGGGAAG	371	-9	1.36%
GTCCGTGCAGGAATTTGgAGGAGGGAAG	63	+1 g	0.37%	GTCCGTGCAGGAATTTGgAGGAGGGAAG	192	+1 g	0.51%	GTCCGTGCAGGAATTT--GGAGGGAAG	246	-2	0.90%
GTCCGTGCAGGAATTT--GGAGGGAAG	43	-2	0.25%	GTCCGTGCAGGAATTTTGAGGAGGGAAG	166	+1 Ins	0.44%	GTCCGTGCAGGAATTT------GGAAG	165	-6	0.60%
GTCCGTGCAGGAATTT---GAGGGAAG	35	-3	0.21%	GTCCGTGCAGGAATTT--GGAGGGAAG	172	-2	0.46%	GTCCGTGCAGGAATTT---GAGGGAAG	120	-3	0.44%
GTCCGTGCAGGAA--TGAGGAGGGAAG	31	-2	0.18%	GTCCGTGCAGGAATTT-AGGAGGGAAG	116	-1	0.31%	GTCCGTGCAGGA-----AGGAGGGAAG	84	-5	0.31%
GTCCGTGCAGGAATT--AGGAGGGAAG	30	-2	0.18%	GTCCGTGCAGGAATTTGaAGGAGGGAAG	78	+1 a	0.21%	GTCCGTGCAGGAATTTGaAGGAGGGAAG	69	+1 a	0.25%
GTCCGTGCAGGAATTTGtAGGAGGGAAG	24	+1 t	0.14%	GTCCGTGCAGGAATTT---GAGGGAAG	75	-3	0.20%	GTCCGTGCAGGAATTT-AGGAGGGAAG	58	-1	0.21%
GTCCGTGCAGGAATTTGaAGGAGGGAAG	23	+1 a	0.14%	GTCCGTGCAGGAAT---AGGAGGGAAG	72	-3	0.19%	GTCCGTGCAGGAATT----------AG	54	-10	0.20%

Total | INDEL	24h	Ins	Del	HDR	Total | INDEL	48h	Ins	Del	HDR
38710 | 20.45%		**2.34%**	**18.10%**	**0.00%**	126030 | 25.49%		**2.80%**	**22.69%**	**0.00%**

Typical seqs	Reads	Type	pct.	Typical seqs	Reads	Type	pct.
GTCCGTGCAGGAATTTG|AGG**AGG**GAAG	30795	WT	79.55%	GTCCGTGCAGGAATTTG|AGG**AGG**GAAG	93901	WT	74.51%
GTCCGTGC---------AGGAGGGAAG	1839	-9	4.75%	GTCCGTGC---------AGGAGGGAAG	13615	-9	10.80%
GTCCGTGCAGGAATTTG-GGAGGGAAG	1487	-1	3.84%	GTCCGTGCAGGAATTTG-GGAGGGAAG	3374	-1	2.68%
GTCCGTGCAGGAATTT--GGAGGGAAG	415	-2	1.07%	GTCCGTGCAGGAATTT--GGAGGGAAG	1439	-2	1.14%
GTCCGTGCAGGAATTT-----GGGAAG	266	-5	0.69%	GTCCGTGC------------AGGGAAG	1284	-12	1.02%
GTCCGTGC------------AGGGAAG	235	-12	0.61%	GTCCGTGCA-------------GGAAG	1196	-13	0.95%
GTCCGTGCAGGAATTTGgAGGAGGGAAG	232	+1 g	0.60%	GTCCGTGCAGGAATTTGaAGGAGGGAAG	908	+1 a	0.72%
GTCCGTGCA-------------GGAAG	217	-13	0.56%	GTCCGTGCAGGAATTT-AGGAGGGAAG	662	-1	0.53%
GTCCGTGCAGGAATTT----------G	231	-10	0.60%	GTCCGTGCAGGAATTTGgAGGAGGGAAG	615	+1 g	0.49%
-----------------AGGAGGGAAG	195	-19	0.50%	GTCCGTGCAGGAATTTTGAGGAGGGAAG	516	+1 Ins	0.41%

P123-P127-YW-W9-RNP-Syn58GATA4-KO

4h — Total 5327 | INDEL 12.39% | Ins 10.61% | Del 1.78% | HDR 0.00%

Typical seqs	Reads	Type	pct.
TCCGTGCAGGAATTTGA|GGA**GGG**AAGA	4667	WT	87.61%
TCCGTGCAGGAATTTGAaGGAGGGAAGA	542	+1 a	10.17%
TCCGTGCAGGAATTTGA-GAGGGAAGA	37	-1	0.69%
TCCGTGCA---------GGAGGGAAGA	14	-9	0.26%
TCCGTGC------------AGGGAAGA	13	-12	0.24%
TCCGTGCAGGAATTTG-GGAGGGAAGA	13	-1	0.24%
TCCGTGCAGGAATTT---GAGGGAAGA	9	-3	0.17%
TCCGTGCAGGAATTTGA--AGGGAAGA	9	-2	0.17%
TCCGTGCAGGAATTTGAgGGAGGGAAGA	7	+1 g	0.13%
TCCGTGCAGGAATTTGAtGGAGGGAAGA	7	+1 t	0.13%

8h — Total 10982 | INDEL 21.64% | Ins 18.15% | Del 3.49% | HDR 0.00%

Typical seqs	Reads	Type	pct.
TCCGTGCAGGAATTTGA|GGA**GGG**AAGA	8606	WT	78.36%
TCCGTGCAGGAATTTGAaGGAGGGAAGA	1892	+1 a	17.23%
TCCGTGCAGGAATTTG-GGAGGGAAGA	100	-1	0.91%
TCCGTGCAGGAATTTGA-GAGGGAAGA	58	-1	0.53%
TCCGTGCA---------GGAGGGAAGA	53	-9	0.48%
TCCGTGCAGGAATTT-AGGAGGGAAGA	32	-1	0.29%
TCCGTGCAGGAATTT--GGAGGGAAGA	28	-2	0.25%
TCCGTGCAGGAATTT---GAGGGAAGA	24	-3	0.22%
TCCGTGCAGGAATTTGACAGGAGGGAAGA	21	+2	0.19%
TCCG----------TGAGGAGGGAAGA	17	-10	0.15%

12h — Total 8250 | INDEL 29.39% | Ins 22.38% | Del 7.02% | HDR 0.00%

Typical seqs	Reads	Type	pct.
TCCGTGCAGGAATTTGA|GGA**GGG**AAGA	5825	WT	70.61%
TCCGTGCAGGAATTTGAaGGAGGGAAGA	1751	+1 a	21.22%
TCCGTGCA---------GGAGGGAAGA	113	-9	1.37%
TCCGTGCAGGAATTTGA-GAGGGAAGA	98	-1	1.19%
TCCGTGCAGGAATTT---GAGGGAAGA	69	-3	0.84%
TCCGTGCAGGAATTTG-GGAGGGAAGA	65	-1	0.79%
TCCGTGCAGGAATTT--GGAGGGAAGA	50	-2	0.61%
TCCGTGCAGGAATTT----------GA	37	-10	0.45%
TCCGTGCAGGAATTT------------	32	-12	0.39%
TCCGTGCAGGAATTTGAGGGAAGAGGGAAG A	26	+4	0.32%

24h — Total 17115 | INDEL 43.84% | Ins 31.91% | Del 11.93% | HDR 0.00%

Typical seqs	Reads	Type	pct.
TCCGTGCAGGAATTTGA|GGA**GGG**AAGA	9612	WT	56.16%
TCCGTGCAGGAATTTGAaGGAGGGAAGA	5125	+1 a	29.94%
TCCGTGCA---------GGAGGGAAGA	572	-9	3.34%
TCCGTGCAGGAATTTGA-GAGGGAAGA	266	-1	1.55%
TCCGTGC------------AGGGAAGA	184	-12	1.08%
TCCGTGCAGGAATTTG-GGAGGGAAGA	154	-1	0.90%
TCCGTGCAGGAATTT--GGAGGGAAGA	102	-2	0.60%
TCCGTGCA-------------GGAAGA	98	-13	0.57%
TCCGTGCAGGAATTT---GAGGGAAGA	98	-3	0.57%
TCCGTGCAGGAA----AGGAGGGAAGA	52	-4	0.30%

48h — Total 31616 | INDEL 48.44% | Ins 28.63% | Del 19.82% | HDR 0.00%

Typical seqs	Reads	Type	pct.
TCCGTGCAGGAATTTGA|GGA**GGG**AAGA	16300	WT	51.56%
TCCGTGCAGGAATTTGAaGGAGGGAAGA	8183	+1 a	25.88%
TCCGTGCA---------GGAGGGAAGA	1957	-9	6.19%
TCCGTGC------------AGGGAAGA	610	-12	1.93%
TCCGTGCA-------------GGAAGA	510	-13	1.61%
TCCGTGCAGGAATTTGA-GAGGGAAGA	489	-1	1.55%
TCCGTGCAGGAATTTG-GGAGGGAAGA	420	-1	1.33%
TCCGTGCAGGAATTT---GAGGGAAGA	349	-3	1.10%
TCCGTGCAGGAATTT--GGAGGGAAGA	245	-2	0.77%
TCCGTGCAGGAATTTTGAGGAGGGAAGA	122	+1 Ins	0.39%

P128-P137-YW-W9-Syn32crBCL11Gata-a1-KO

Total	INDEL		Ins	Del	HDR
158062	15.51%	4h	11.67%	3.84%	0.00%

Typical seqs	Reads	Type	pct.
CACGCCCCCACCCTAAT|CAG**AGG**CCAA	133552	WT	84.49%
CACGCCCCCACCCTAATtCAGAGGCCAA	11123	+1 t	7.04%
CACGCCCCC------ATCAGAGGCCAA	3108	-6	1.97%
CACGCCCCC------ATCGGAGGCCAA	1724	-6	1.09%
CACGCCCCCACCCCAATTCAGAGGCCAA	870	+1 Ins	0.55%
TACGCCCCCACCCTAATTCAGAGGCCAA	582	+1 Ins	0.37%
CACGCCCCTACCCTAATTCAGAGGCCAA	598	+1 Ins	0.38%
CACGCCCCCATCCTAATTCAGAGGCCAA	551	+1 Ins	0.35%
CACGCCCTCACCCTAATTCAGAGGCCAA	404	+1 Ins	0.26%
CACGCCTCCACCCTAATTCAGAGGCCAA	319	+1 Ins	0.20%

Total	INDEL		Ins	Del	HDR
4642	28.89%	8h	16.91%	11.98%	0.00%

Typical seqs	Reads	Type	pct.
CACGCCCCCACCCTAAT|CAG**AGG**CCAA	3301	WT	71.11%
CACGCCCCCACCCTAATtCAGAGGCCAA	488	+1 t	10.51%
CACG-------------CAGAGGCCAA	76	-13	1.64%
CA---------------------CCAA	58	-21	1.25%
CACG---------------------AA	46	-21	0.99%
-----------------CAGAGGCCAA	43	-17	0.93%
CACGCCCCCAC------CAGAGGCCAA	50	-6	1.08%
CACGCCCCCAC-----TCAGAGGCCAA	38	-5	0.82%
CACGCCCC-------ATCAGAGGCCAA	34	-7	0.73%
CACGCCCCCACCCCAATTCAGAGGCCAA	33	+1 Ins	0.71%

Total	INDEL		Ins	Del	HDR
6102	38.35%	12h	16.54%	21.81%	0.00%

Typical seqs	Reads	Type	pct.
CACGCCCCCACCCTAAT|CAG**AGG**CCAA	3762	WT	61.65%
CACGCCCCCACCCTAATtCAGAGGCCAA	591	+1 t	9.69%
CACG--------------------CAA	287	-20	4.70%
CACGCGC---------TCAGAGGCCAA	184	-9	3.02%
CACGCCC--------------------	154	-26	2.52%
CACGCCCCCAC-----TCAGAGGCCAA	136	-5	2.23%
CACGCCCCCACCCCAATTCAGAGGCCAA	75	+1 Ins	1.23%
CAC-------------------GCCAA	67	-19	1.10%
CACGCC---------------------	59	-27	0.97%
CAC------------------GGCCAA	56	-18	0.92%

Total	INDEL		Ins	Del	HDR
67612	56.79%	24h	20.45%	36.34%	0.00%

Typical seqs	Reads	Type	pct.
CACGCCCCCACCCTAAT|CAG**AGG**CCAA	29216	WT	43.21%
CACGCCCCCACCCTAATtCAGAGGCCAA	7785	+1 t	11.51%
CACG-------------CAGAGGCCAA	3421	-13	5.06%
CACGCCCC---------CAGAGGCCAA	1788	-9	2.64%
CACG-----------------------	2330	-29	3.45%
CACG----------------------A	1419	-22	2.10%
CACGCCCCCACCCCAATTCAGAGGCCAA	1062	+1 Ins	1.57%
CACGCCC----------CAGAGGCCAA	936	-10	1.38%
CAC----------------GAGGCCAA	956	-16	1.41%
CACGCC-------------GAGGCCAA	692	-13	1.02%

Total	INDEL		Ins	Del	HDR
25268	56.68%	48h	15.62%	41.06%	0.00%

Typical seqs	Reads	Type	pct.
CACGCCCCCACCCTAAT|CAG**AGG**CCAA	10946	WT	43.32%
CACGCCCCCACCCTAATtCAGAGGCCAA	2301	+1 t	9.11%
CACGCCC----------CAGAGGCCAA	570	-10	2.26%
CAC-------------------GCCAA	663	-19	2.62%
CACGCC-----------CAGAGGCCAA	477	-11	1.89%
CACG-----------------------	941	-24	3.72%
CACGC------------CAGAGGCCAA	426	-12	1.69%
CACG---------------------AA	359	-21	1.42%
CACGCCCCCAC------CAGAGGCCAA	342	-6	1.35%
CACG-------------CAGAGGCCAA	311	-13	1.23%

P128-P137-YW-W9-Syn33crBCL11Gata-a2-KO

Total | INDEL	4h	Ins	Del	HDR
7562 | 4.44%		2.95%	1.45%	0.04%

Typical seqs	Reads	Type	pct.
CACAGGCTCCAGGAAGG|GTT**TGG**CCTC	7226	WT	95.56%
CACAGGCTCCAGGAAGGgGTTTGGCCTC	198	+1 g	2.62%
CACAGGCTCCAGGAAG-GTTTGGCCTC	48	-1	0.63%
CACAGGCTCCAGGAA------GGCCTC	17	-6	0.22%
CACAGGCCCCAGGAAGGGGTTTGGCCTC	6	+1 Ins	0.08%
CACAGGCTCCAGGAAGG----GGCCTC	6	-4	0.08%
CACAGGCTCCAGGA---GTTTGGCCTC	6	-3	0.08%
CACAGGCTCCA-----GGTTTGGCCTC	5	-5	0.07%
CACAGGCTCCAGGAA-------GCCTC	5	-7	0.07%
CACAGGCTCCAGGAAGGgGTTTGGCCTT	5	+1 g	0.07%

Total | INDEL	8h	Ins	Del	HDR
2832 | 8.09%		4.27%	3.81%	0.00%

Typical seqs	Reads	Type	pct.
CACAGGCTCCAGGAAGG|GTT**TGG**CCTC	2603	WT	91.91%
CACAGGCTCCAGGAAGGgGTTTGGCCTC	112	+1 g	3.95%
CACAGGCTCCAGGAAG-GTTTGGCCTC	66	-1	2.33%
CACAGGCTCCAGGAAGGAACG---------	19	-7	0.67%
CACAGGCTCCAGGAA------------	5	-19	0.18%
CACAGGCTCCAGGAA-------GCCCC	5	-7	0.18%
CACAGGCTCCAGGAAG-----GGCCTC	4	-5	0.14%
CACAGGCTCCAGGAAGGGGTTTGGCCTC	3	+2	0.11%
CACAGGCTCCAGGAAG--TTTGGCCTC	3	-2	0.11%
CACAGGCTCCAGGAAGGgGTTTGGCCTT	3	+1 g	0.11%

Total | INDEL	12h	Ins	Del	HDR
2921 | 12.98%		6.16%	6.81%	0.00%

Typical seqs	Reads	Type	pct.
CACAGGCTCCAGGAAGG|GTT**TGG**CCTC	2542	WT	87.02%
CACAGGCTCCAGGAAGGgGTTTGGCCTC	168	+1 g	5.75%
CACAGGCTCCAGGAAG-GTTTGGCCTC	77	-1	2.64%
CACAGGCTCCAGGAA------------	24	-19	0.82%
CACAGGCTCCAGGAAG--TTTGGCCTC	18	-2	0.62%
CACA-----------------GGCCTC	12	-17	0.41%
CACAGGCTCCA-----GGTTTGGCCTC	8	-5	0.27%
CACAGGCTCCAG------TTTGGCCTC	6	-6	0.21%
CACAGGCTCCAGGAA------GGCCTC	10	-6	0.34%
CACAGGCTCCAGGAA-------GCCTC	5	-7	0.17%

Total | INDEL	24h	Ins	Del	HDR
40863 | 28.93%		7.77%	21.16%	0.00%

Typical seqs	Reads	Type	pct.
CACAGGCTCCAGGAAGG|GTT**TGG**CCTC	29042	WT	71.07%
CACAGGCTCCAGGAAG-GTTTGGCCTC	2248	-1	5.50%
CACAGGCTCCAGGAAGGgGTTTGGCCTC	2566	+1 g	6.28%
CACAGGCTCCAGGA-------------	506	-22	1.24%
CACAGGCTCCAGGAA------GGCCTC	481	-6	1.18%
CACAGGCTCCAGGAAG--TTTGGCCTC	444	-2	1.09%
CACA-----------------GGCCTC	342	-17	0.84%
CACAGGCTCCA-----GGTTTGGCCTC	293	-5	0.72%
CACAGGCTCCA----------GGCCTC	267	-10	0.65%
CACAGGCTCCAG------TTTGGCCTC	317	-6	0.78%

Total | INDEL	48h	Ins	Del	HDR
12797 | 23.90%		5.31%	18.59%	0.00%

Typical seqs	Reads	Type	pct.
CACAGGCTCCAGGAAGG|GTT**TGG**CCTC	9738	WT	76.10%
CACAGGCTCCAGGAAG-GTTTGGCCTC	639	-1	4.99%
CACAGGCTCCAGGAAGGgGTTTGGCCTC	520	+1 g	4.06%
CACAGGCTCCAGGAA------------	149	-19	1.16%
CACAGGCTCCAGGAA------GGCCTC	181	-6	1.41%
CACA-----------------GGCCTC	151	-17	1.18%
CACAGGCTCCAGGAAG--TTTGGCCTC	94	-2	0.73%
CACAGGCTCCAGGAA-------GCCTC	80	-7	0.63%
CACAGGCTCCA----------GGCCTC	81	-10	0.63%
CACAGGCTCCAGG----GTTTGGCCTC	72	-4	0.56%

P128-P137-YW-W9-Syn34crBCL11Gata-a3-KO

Total | INDEL	4h	Ins	Del	HDR
40106 | 9.50%		6.37%	3.09%	0.04%

Typical seqs	Reads	Type	pct.
TTTATCACAGGCTCCAG|GAA**GGG**TTTG	36297	WT	90.50%
TTTATCACAGGCTCCAGgGAAGGGTTTG	2199	+1 g	5.48%
TTTATCACAGGCTCCA-GAAGGGTTTG	557	-1	1.39%
TTTATCACAGGCTCC-GGAAGGGTTTG	435	-1	1.08%
TTTATCACAGGCTCC----AGGGTTTG	87	-4	0.22%
TTTATCACAGGCTCCAGAGGAAGGGTTTG	75	+2	0.19%
TTTATCACAGGCTCCAAGGAAGGGTTTG	34	+1 Ins	0.08%
TTTATCACAGGCTCCACAGGAAGGGTTTG	26	+2	0.06%
TTTATCACAGGCTCC--GAAGGGTTTG	26	-2	0.06%
TTTATCACAGGCTCCAGCAGGAAGGGTTTG	24	+3	0.06%

Total | INDEL	8h	Ins	Del	HDR
1238 | 13.73%		10.42%	3.31%	0.00%

Typical seqs	Reads	Type	pct.
TTTATCACAGGCTCCAG|GAA**GGG**TTTG	1068	WT	86.27%
TTTATCACAGGCTCCAGgGAAGGGTTTG	118	+1 g	9.53%
TTTATCACAGGCTCC-GGAAGGGTTTG	25	-1	2.02%
TTTATCACAGGCTCCA-GAAGGGTTTG	9	-1	0.73%
TTTATCACAGGCTCCAGAGGAAGGGTTTG	7	+2	0.57%
TTTATCACA-----------------G	4	-17	0.32%
TTTATCACAGGCTCCAAGGAAGGGTTTG	4	+1 Ins	0.32%
TTTATCACAGGC-CCAGGAAGGGTTTG	3	-1	0.24%

Total | INDEL	12h	Ins	Del	HDR
3824 | 25.03%		15.43%	9.60%	0.00%

Typical seqs	Reads	Type	pct.
TTTATCACAGGCTCCAG|GAA**GGG**TTTG	2867	WT	74.97%
TTTATCACAGGCTCCAGgGAAGGGTTTG	466	+1 g	12.19%
TTTATCACAGGCTCC-GGAAGGGTTTG	113	-1	2.96%
TTTATCACAGGCTCCA-GAAGGGTTTG	97	-1	2.54%
TTTATCACAGGCTCCAGCAGGAAGGGTTTG	21	+3	0.55%
TTTATCACAGGCTCCAAGGAAGGGTTTG	17	+1 Ins	0.44%
TTTATCACA-----------------G	17	-17	0.44%
TTTATCACAGGCTCC----AGGGTTTG	16	-4	0.42%
TTTATCACAGGCTCCAGAGGAAGGGTTTG	22	+2	0.58%
TTTATCACAGGCT--------------	18	-15	0.47%

Total | INDEL	24h	Ins	Del	HDR
13857 | 39.16%		15.80%	23.35%	0.00%

Typical seqs	Reads	Type	pct.
TTTATCACAGGCTCCAG|GAA**GGG**TTTG	8431	WT	60.84%
TTTATCACAGGCTCCAGgGAAGGGTTTG	1531	+1 g	11.05%
TTTATCACAGGCTCC-GGAAGGGTTTG	647	-1	4.67%
TTTATCACAGGCTCCA-GAAGGGTTTG	579	-1	4.18%
TTTATCACAGGCTCC----AGGGTTTG	325	-4	2.35%
TTTATCACAGGCT--------------	251	-15	1.81%
TTTATCACAGGCTCCAGAGGAAGGGTTTG	134	+2	0.97%
TTTATCACAGGCTCCAAGGAAGGGTTTG	139	+1 Ins	1.00%
TTTATCACA-----------------G	96	-17	0.69%
TTTATCAC-----------AGGGTTTG	98	-11	0.71%

Total | INDEL	48h	Ins	Del	HDR
17191 | 39.81%		15.73%	24.08%	0.00%

Typical seqs	Reads	Type	pct.
TTTATCACAGGCTCCAG|GAA**GGG**TTTG	10347	WT	60.19%
TTTATCACAGGCTCCAGgGAAGGGTTTG	1678	+1 g	9.76%
TTTATCACAGGCTCCA-GAAGGGTTTG	721	-1	4.19%
TTTATCACAGGCTCC-GGAAGGGTTTG	492	-1	2.86%
TTTATCACAGGCT--------------	386	-15	2.25%
TTTATCACAGGCTCC----AGGGTTTG	355	-4	2.07%
TTTATCAC-----------AGGGTTTG	370	-11	2.15%
TTTATCACAGGCTCCAAGGAAGGGTTTG	261	+1 Ins	1.52%
TTTATCACA-----------------G	202	-17	1.18%
TTTATCACAGGCTCCAGAGGAAGGGTTTG	158	+2	0.92%

P128-P137-YW-W9-Syn35crBCL11Gata-a4-KO

4h — Total | INDEL: 20922 | 11.18%; Ins: 10.08%; Del: 1.06%; HDR: 0.04%

Typical seqs	Reads	Type	pct.
TTTTATCACAGGCTCCA|GGA**AGG**GTTT	18583	WT	88.82%
TTTTATCACAGGCTCCAaGGAAGGGTTT	1819	+1 a	8.69%
TTTTATCACAGGCTCC-GGAAGGGTTT	187	-1	0.89%
TTTTATCACAGGCTCCACAGGAAGGGTTT	105	+2	0.50%
TTTTATCACAGGCTCCAgGGAAGGGTTT	17	+1 g	0.08%
TTTTATCACAGGCTCCAaGGAAGGGTTC	16	+1 a	0.08%
TTTTATCACAGGCCCCAAGGAAGGGTTT	13	+1 Ins	0.06%
TTTTATCACAGGCTCCAaGGGAGGGTTT	14	+1 a	0.07%
TTTTATCACAGGCTCCAaGGAAGAGTTT	11	+1 a	0.05%
TTTTATCACAGGCT-CTGGAAGGGTTT	10	-1	0.05%

8h — Total | INDEL: 1223 | 17.09%; Ins: 15.62%; Del: 1.47%; HDR: 0.00%

Typical seqs	Reads	Type	pct.
TTTTATCACAGGCTCCA|GGA**AGG**GTTT	1014	WT	82.91%
TTTTATCACAGGCTCCAaGGAAGGGTTT	157	+1 a	12.84%
TTTTATCACAGGCTCCACAGGAAGGGTTT	7	+2	0.57%
TTTTATCACAGGCTCC-GGAAGGGTTT	6	-1	0.49%
TTTTATCACAGGCTTCCAGGAAGGGTTT	6	+1 Ins	0.49%
TTTTATCACAGGCCTCCAGGAAGGGTTT	5	+1 Ins	0.41%
TTTTATCACAGGCTC-AGGAAGGGTTT	4	-1	0.33%
TTTTATCACAGGCTCCCAGGAAGGGTTT	4	+1 Ins	0.33%
TTTTATCACAGGCTC--GGAAGGGTTT	4	-2	0.33%
TTTTATCACA-------GGAAGGGTTT	4	-7	0.33%

12h — Total | INDEL: 972 | 18.93%; Ins: 16.05%; Del: 2.88%; HDR: 0.00%

Typical seqs	Reads	Type	pct.
TTTTATCACAGGCTCCA|GGA**AGG**GTTT	788	WT	81.07%
TTTTATCACAGGCTCCAaGGAAGGGTTT	143	+1 a	14.71%
TTTTATCACAGGCTCC-GGAAGGGTTT	8	-1	0.82%
TTTTATCACAGGCTCCAgGGAAGGGTTT	7	+1 g	0.72%
TTTTATCACAGGCTCCA-GAAGGGTTT	6	-1	0.62%
TTTTATCACAGGCTCCACAGGAAGGGTTT	6	+2	0.62%
TTTTATCACA-------GGAAGGGTTT	5	-7	0.51%
TTTTATCACAGGCTC-AGGAAGGGTTT	3	-1	0.31%
TTTTATCACAGGCTCC----AGGGTTT	3	-4	0.31%
TTTTATCAC-----------AGGGTTT	3	-11	0.31%

24h — Total | INDEL: 19093 | 39.97%; Ins: 32.00%; Del: 7.97%; HDR: 0.00%

Typical seqs	Reads	Type	pct.
TTTTATCACAGGCTCCA|GGA**AGG**GTTT	11462	WT	60.03%
TTTTATCACAGGCTCCAaGGAAGGGTTT	5214	+1 a	27.31%
TTTTATCACA-------GGAAGGGTTT	321	-7	1.68%
TTTTATCACAGGCTCCACAGGAAGGGTTT	224	+2	1.17%
TTTTATCACAGGCTCC-GGAAGGGTTT	180	-1	0.94%
TTTTATCACAGGCT--------------	141	-15	0.74%
TTTTATCAC-----------AGGGTTT	119	-11	0.62%
TTTTATCACAGGCTCCA----------	114	-10	0.60%
TTTTATCAC-----------------C	49	-17	0.26%
TTTTATCACAGGCTCCAgGGAAGGGTTT	61	+1 g	0.32%

48h — Total | INDEL: 9731 | 40.75%; Ins: 30.62%; Del: 10.12%; HDR: 0.00%

Typical seqs	Reads	Type	pct.
TTTTATCACAGGCTCCA|GGA**AGG**GTTT	5766	WT	59.25%
TTTTATCACAGGCTCCAaGGAAGGGTTT	2490	+1 a	25.59%
TTTTATCACA-------GGAAGGGTTT	333	-7	3.42%
TTTTATCACAGGCTCCACAGGAAGGGTTT	109	+2	1.12%
TTTTATCACAGGCTCC-GGAAGGGTTT	68	-1	0.70%
TTTTATCACAGGCT--------------	89	-15	0.91%
TTTTATCACA-----------------	75	-17	0.77%
TTTTATCAC-----------AGGGTTT	64	-11	0.66%
TTTTATCACAGGCTCCAgGGAAGGGTTT	55	+1 g	0.57%
TTTTATCACAGG---------------	38	-18	0.39%

P128-P137-YW-W9-Syn36crBCL11Gata-a5-KO

Total | INDEL	4h	Ins	Del	HDR
30038 | 5.70%		3.44%	2.27%	0.00%

Typical seqs	Reads	Type	pct.
TTGCTTTTATCACAGGC|TCCAGGAAGG	28325	WT	94.30%
TTGCTTTTATCACAGGCcTCCAGGAAGG	731	+1 c	2.43%
TTGCTTTTATCACAGGC-CCAGGAAGG	278	-1	0.93%
TTGCTTTTATCACAGGCtTCCAGGAAGG	180	+1 t	0.60%
TTGCTTTTATCACAGG-TCCAGGAAGG	162	-1	0.54%
TTGCTTTTATCACAGG--CCAGGAAGG	70	-2	0.23%
TTGCTTTTA--------TCCAGGAAGG	55	-8	0.18%
TTGCTTTTATCA-------CAGGAAGG	39	-7	0.13%
TTGCTTTTATCACAGGCgTCCAGGAAGG	27	+1 g	0.09%
TTGCTTTTATCACAGGCtCCAAGGAAGG	22	+1 t	0.07%

Total | INDEL	8h	Ins	Del	HDR
2552 | 14.15%		8.62%	5.53%	0.00%

Typical seqs	Reads	Type	pct.
TTGCTTTTATCACAGGC|TCCAGGAAGG	2191	WT	85.85%
TTGCTTTTATCACAGGCcTCCAGGAAGG	113	+1 c	4.43%
TTGCTTTTATCACAGGCtTCCAGGAAGG	67	+1 t	2.63%
TTGCTTTTATCACAGGC-CCAGGAAGG	46	-1	1.80%
TTGCTTTTA--------TCCAGGAAGG	24	-8	0.94%
TTGCTTTTATCACAGG--CCAGGAAGG	20	-2	0.78%
TTGCTTTTATCACAGGCtCCAAGGAAGG	17	+1 t	0.67%
TTGCTTTTATCACAGG-TCCAGGAAGG	16	-1	0.63%
TTGCTTTTATCACAGGCgTCCAGGAAGG	13	+1 g	0.51%
TTGCTTTTATCACAG--TCCAGGAAGG	12	-2	0.47%

Total | INDEL	12h	Ins	Del	HDR
3440 | 18.60%		7.70%	10.90%	0.00%

Typical seqs	Reads	Type	pct.
TTGCTTTTATCACAGGC|TCCAGGAAGG	2800	WT	81.40%
TTGCTTTTATCACAGGCcTCCAGGAAGG	135	+1 c	3.92%
TTGCTTTTATCACAGGC-CCAGGAAGG	98	-1	2.85%
TTGCTTTTATCACAGGCtTCCAGGAAGG	100	+1 t	2.91%
TTGCTTTTA--------TCCAGGAAGG	37	-8	1.08%
TTGCTTTTATCACAGGC-----GAAGG	28	-5	0.81%
TTGCTTTTATCACAGG-TCCAGGAAGG	27	-1	0.78%
TTGCTTTTATCACAGG--CCAGGAAGG	27	-2	0.78%
TTGCTTTTATCA-------CAGGAAGG	26	-7	0.76%
TTGCTTTT---------TCCAGGAAGG	21	-9	0.61%

Total | INDEL	24h	Ins	Del	HDR
13332 | 37.28%		13.41%	23.87%	0.00%

Typical seqs	Reads	Type	pct.
TTGCTTTTATCACAGGC|TCCAGGAAGG	8362	WT	62.72%
TTGCTTTTATCACAGGCcTCCAGGAAGG	849	+1 c	6.37%
TTGCTTTTA--------TCCAGGAAGG	652	-8	4.89%
TTGCTTTTATCA-------CAGGAAGG	624	-7	4.68%
TTGCTTTTATCACAGGCtTCCAGGAAGG	547	+1 t	4.10%
TTGCTTTTATCACAGGC-CCAGGAAGG	369	-1	2.77%
TTGCTTTTATCACAGG--CCAGGAAGG	214	-2	1.61%
TTGCTTTTATCACAGG-TCCAGGAAGG	106	-1	0.80%
TTGCTTTTATCACAGGC-----GAAGG	89	-5	0.67%
TTGCTTTT---------TCCAGGAAGG	81	-9	0.61%

Total | INDEL	48h	Ins	Del	HDR
9974 | 40.86%		11.83%	29.03%	0.00%

Typical seqs	Reads	Type	pct.
TTGCTTTTATCACAGGC|TCCAGGAAGG	5899	WT	59.14%
TTGCTTTTATCA-------CAGGAAGG	922	-7	9.24%
TTGCTTTTA--------TCCAGGAAGG	660	-8	6.62%
TTGCTTTTATCACAGGCcTCCAGGAAGG	479	+1 c	4.80%
TTGCTTTTATCACAGGC-CCAGGAAGG	337	-1	3.38%
TTGCTTTTATCACAGGCtTCCAGGAAGG	348	+1 t	3.49%
TTGCTTTTATCACAGG--CCAGGAAGG	108	-2	1.08%
TTGCTTTTATCACAGGCtCCAAGGAAGG	104	+1 t	1.04%
TTGCTTTTATCACAGG-TCCAGGAAGG	98	-1	0.98%
TTGCTTTTATCACA-------------	62	-17	0.62%

P128-P137-YW-W9-Syn37crBCL11Gata-a6-KO

Total | INDEL	4h	Ins	Del	HDR
11999 | 2.55%		1.33%	1.23%	0.00%
**Typical seqs**		**Reads**	**Type**	**pct.**
CTAACAGTTGCTTTTAT|CAC**AGG**CTCC		11693	WT	97.45%
CTAACAGTTGCTTTTATtCACAGGCTCC		31	+1 t	0.26%
CTAACAGTTGCTTTT------------		22	-13	0.18%
CTAACAGTT------------------		18	-23	0.15%
CTAACAGTTGCTCTTATCACAGGCTCCA		20	+1 Ins	0.17%
CTAACAGTTGCTTTTATcGCAGGCTCCA		16	+1 c	0.13%
CTAACAGTTGCTTTTATcACGGGCTCCA		15	+1 c	0.13%
CTAACAGTTGCT---------------		15	-15	0.13%
CTAACAGTTGCTTTTACCACAGGCTCCA		14	+1 Ins	0.12%
CTAACAGTTGCTTTTA--------TCC		12	-8	0.10%

Total | INDEL	8h	Ins	Del	HDR
30834 | 8.94%		7.75%	1.19%	0.00%
**Typical seqs**		**Reads**	**Type**	**pct.**
CTAACAGTTGCTTTTAT|CAC**AGG**CTCC		28077	WT	91.06%
CTAACAGTTGCTTTTATtCACAGGCTCC		2182	+1 t	7.08%
CTAACAGTTGCTTTTA-CACAGGCTCC		233	-1	0.76%
CTAACAGTTGCTTTTATaCACAGGCTCC		41	+1 a	0.13%
CTAACAGTTGCT------ACAGGCTCC		35	-6	0.11%
CTAACAGTTGC-TTTATCACAGGCTCC		28	-1	0.09%
CTAACAGTTGCTTTTATATCACAGGCTCC		27	+2	0.09%
CTGACAGTTGCTTTTA-CACAGGCTCC		22	-1	0.07%
CTAACAGTTGCTTTTAT-ACAGGCTCC		19	-1	0.06%
CTAACAGTTGCTTTTAATCACAGGCTCC		18	+1 Ins	0.06%

Total | INDEL	12h	Ins	Del	HDR
1884 | 12.37%		11.25%	1.11%	0.00%
**Typical seqs**		**Reads**	**Type**	**pct.**
CTAACAGTTGCTTTTAT|CAC**AGG**CTCC		1651	WT	87.63%
CTAACAGTTGCTTTTATtCACAGGCTCC		203	+1 t	10.77%
CTAACAGTTGCTTTTA-CACAGGCTCC		9	-1	0.48%
CTAACAGTTGCTTTTATATCACAGGCTCC		6	+2	0.32%
CTAACAGTTGCTTTT-TCACAGGCTCC		6	-1	0.32%
CTAACAGTTGCTTTTA--------TCC		3	-8	0.16%
CTAACAGTT-------------GCTCC		3	-13	0.16%
CTAACAGTTGCCTTTATTCACAGGCTCC		3	+1 Ins	0.16%
Total | INDEL		Ins	Del	HDR
1884 | 12.37%		11.25%	1.11%	0.00%

Total | INDEL	24h	Ins	Del	HDR
4329 | 17.72%		14.51%	3.21%	0.00%
**Typical seqs**		**Reads**	**Type**	**pct.**
CTAACAGTTGCTTTTAT|CAC**AGG**CTCC		3562	WT	82.28%
CTAACAGTTGCTTTTATtCACAGGCTCC		565	+1 t	13.05%
CTAACAGTTGCTTTTATATCACAGGCTCC		25	+2	0.58%
CTAACAGTTGCTTTTAT-ACAGGCTCC		22	-1	0.51%
CTAACAGTTGCTTTTA-CACAGGCTCC		16	-1	0.37%
CTA---------------ACAGGCTCC		15	-15	0.35%
CTAACAGTTGCTTTTAATCACAGGCTCC		11	+1 Ins	0.25%
CTAA-------------CACAGGCTCC		11	-13	0.25%
CTAACAGTTGCTTTTA--------TCC		8	-8	0.18%
CTAACAGTTGCTTTT-TCACAGGCTCC		6	-1	0.14%

Total | INDEL	48h	Ins	Del	HDR
7676 | 29.57%		18.94%	10.63%	0.00%
**Typical seqs**		**Reads**	**Type**	**pct.**
CTAACAGTTGCTTTTAT|CAC**AGG**CTCC		5406	WT	70.43%
CTAACAGTTGCTTTTATtCACAGGCTCC		1243	+1 t	16.19%
CTA---------------ACAGGCTCC		198	-15	2.58%
CTAACAGTT-------------GCTCC		83	-13	1.08%
CTAA-------------CACAGGCTCC		52	-13	0.68%
CTAACAGTTGCTTTTATATCACAGGCTCC		48	+2	0.63%
CTAACAGTTGCTTTTAT-ACAGGCTCC		42	-1	0.55%
CTAACAGTTGCTTTTA-CACAGGCTCC		31	-1	0.40%
CTAACAGTTGCTTTT--CACAGGCTCC		30	-2	0.39%
CTAACAGTTGCTTTTAATCACAGGCTCC		32	+1 Ins	0.42%

P128-P137-YW-W9-Syn59crMYH6a-KO

4h

Total	INDEL	Ins	Del	HDR
165651	1.46%	0.83%	0.64%	0.00%

Typical seqs	Reads	Type	pct.
GCAGTAGGGGGCCTGAGAGG**AGG**GAGG	163228	WT	98.54%
GCAGTAGGGGGCCTGAGgAGGAGGGAGG	1027	+1 g	0.62%
GCAGTAGGGGGCCTG--AGGAGGGAGG	602	-2	0.36%
GCAGTAGGGGGCCTGAGCTAGGAGGGAGG	170	+2	0.10%
GCAGTAGGGGGCCTGA-AGGAGGGAGG	160	-1	0.10%
GCAGTAGGGGGCCTGAG-GGAGGGAGG	104	-1	0.06%
GCAGTAGGGGGCCTGAGaAGGAGGGAGG	86	+1 a	0.05%
GCAGTAGGGGG-CTGAGAGGAGGGAGG	55	-1	0.03%
GCAGTAGGGGGCCTGAGA-GAGGGAGG	26	-1	0.02%
GCAGTAGGGGGCCT--GAGGAGGGGGG	15	-2	0.01%

8h

Total	INDEL	Ins	Del	HDR
31480	3.18%	1.08%	2.10%	0.00%

Typical seqs	Reads	Type	pct.
GCAGTAGGGGGCCTGAGAGG**AGG**GAGG	30480	WT	96.82%
GCAGTAGGGGGCCTG--AGGAGGGAGG	239	-2	0.76%
GCAGTAGGGGGCCTGAGaAGGAGGGAGG	170	+1 a	0.54%
GCAGTAGGGGGCCTGAGgAGGAGGGAGG	120	+1 g	0.38%
GCAGTAGGGGGCCTGAG-GGAGGGAGG	106	-1	0.34%
GCAGT---------------AGGGAGG	52	-15	0.17%
GCAGT------------AGGAGGGAGG	44	-12	0.14%
GCAGTAGGGGGCCTGAGtAGGAGGGAGG	39	+1 t	0.12%
GCAGTAGGGGGCCT-----GAGGGAGG	33	-5	0.10%
GCAGTAGGGGGCCTG-GAGGAGGGAGG	29	-1	0.09%

12h

Total	INDEL	Ins	Del	HDR
55293	3.13%	0.34%	2.80%	0.00%

Typical seqs	Reads	Type	pct.
GCAGTAGGGGGCCTGAGAGG**AGG**GAGG	53560	WT	96.87%
GCAGTAGGGGGCCTG--AGGAGGGAGG	514	-2	0.93%
GCAGTAGGGGGCCTGAG-GGAGGGAGG	469	-1	0.85%
GCAGTAGGGGGCCTGAGaAGGAGGGAGG	184	+1 a	0.33%
GCAGTAGGGGGCCTGA-AGGAGGGAGG	124	-1	0.22%
GCAGTAGGGGGCCTGA----------G	81	-10	0.15%
GCAGT------------AGGAGGGAGG	65	-12	0.12%
GCAGTAGGGGGCCT--------GGAGG	40	-8	0.07%
GCAGT----------AGAGGAGGGAGG	38	-10	0.07%
GCAGTAGGGGGCCT-----GAGGGAGG	25	-5	0.05%

24h

Total	INDEL	Ins	Del	HDR
276168	8.19%	1.82%	6.37%	0.00%

Typical seqs	Reads	Type	pct.
GCAGTAGGGGGCCTGAGAGG**AGG**GAGG	253556	WT	91.81%
GCAGTAGGGGGCCTG--AGGAGGGAGG	3846	-2	1.39%
GCAGTAGGGGGCCTGAGgAGGAGGGAGG	2486	+1 g	0.90%
GCAGTAGGGGGCCTGA-AGGAGGGAGG	1950	-1	0.71%
GCAGTAGGGGGCCTGAG-GGAGGGAGG	1989	-1	0.72%
GCAGTAGGGGGCCT-----GAGGGAGG	1403	-5	0.51%
GCAGTAGGGGGCCTGAGaAGGAGGGAGG	1536	+1 a	0.56%
GCAGT------------AGGAGGGAGG	1187	-12	0.43%
GC---------------AGGAGGGAGG	1089	-15	0.39%
GCAGTAGGGGGCCT---------GAGG	839	-9	0.30%

48h

Total	INDEL	Ins	Del	HDR
116111	6.15%	1.66%	4.49%	0.00%

Typical seqs	Reads	Type	pct.
GCAGTAGGGGGCCTGAGAGG**AGG**GAGG	108967	WT	93.85%
GCAGTAGGGGGCCTGAGaAGGAGGGAGG	1064	+1 a	0.92%
GCAGTAGGGGGCCTG--AGGAGGGAGG	841	-2	0.72%
GCAGTAGGGGGCCTGAGgAGGAGGGAGG	599	+1 g	0.52%
GCAGT------------AGGAGGGAGG	564	-12	0.49%
GCAGTAGGGGGCCTGAG-GGAGGGAGG	520	-1	0.45%
GC---------------AGGAGGGAGG	277	-15	0.24%
GCAGTAGGGGGCCT---------GAGG	255	-9	0.22%
GCAGTAGGGGGCCT-----GAGGGAGG	177	-5	0.15%
GCAGTAGGG----------------GG	194	-16	0.17%

P128-P137-YW-W9-Syn60crMYH6b-KO

Total	INDEL		Ins	Del	HDR
105809	3.91%	4h	2.99%	0.92%	0.00%

Typical seqs	Reads	Type	pct.
CAGTAGGGGGCCTGAGAGGA**GGG**AGGC	101667	WT	96.09%
CAGTAGGGGGCCTGAGAaGGAGGGAGGC	2610	+1 a	2.47%
CAGTAGGGGGCCTGAGA-GAGGGAGGC	272	-1	0.26%
CAGTAGGGGGCCTGAG-GGAGGGAGGC	188	-1	0.18%
CAGTAGGGGGCCTGAGAcGGAGGGAGGC	147	+1 c	0.14%
CAGTAGGGGGCCTGA-------GAGGC	136	-7	0.13%
CAGTAGGGGGCCTGA---GAGGGAGGC	128	-3	0.12%
CAGTAGGGGGCCTGAGAgGGAGGGAGGC	98	+1 g	0.09%
CAGTAGGG---------GGAGGGAGGC	93	-9	0.09%
CAGTAGGGGGCCTGAGAAAGGAGGGAGGC	66	+2	0.06%

Total	INDEL		Ins	Del	HDR
23744	11.40%	8h	8.96%	2.43%	0.00%

Typical seqs	Reads	Type	pct.
CAGTAGGGGGCCTGAGAGGA**GGG**AGGC	21038	WT	88.60%
CAGTAGGGGGCCTGAGAaGGAGGGAGGC	1885	+1 a	7.94%
CAGTAGGGGGCCTGAG-GGAGGGAGGC	142	-1	0.60%
CAGTAGGGGGCCTGA---GAGGGAGGC	86	-3	0.36%
CAGTAGGGGGCCT--GAGGAGGGAGGC	60	-2	0.25%
CAGT------------AGGAGGGAGGC	43	-12	0.18%
CAGT---------------AGGGAGGC	37	-15	0.16%
CAGTA----------------GGAGGC	33	-16	0.14%
CAGTAGGGGGCCTGAGAtGGAGGGAGGC	31	+1 t	0.13%
CAGTAGGGGGCCTG---GGAGGGAGGC	23	-3	0.10%

Total	INDEL		Ins	Del	HDR
44025	15.62%	12h	11.29%	4.32%	0.00%

Typical seqs	Reads	Type	pct.
CAGTAGGGGGCCTGAGAGGA**GGG**AGGC	37149	WT	84.38%
CAGTAGGGGGCCTGAGAaGGAGGGAGGC	4313	+1 a	9.80%
CAGTAGGGGGCCTGAG-GGAGGGAGGC	499	-1	1.13%
CAGTAGGGGGCCT--GAGGAGGGAGGC	217	-2	0.49%
CAGTAGGGGGCCTGAGA-GAGGGAGGC	141	-1	0.32%
CAGTAGGGGGCCTGA---GAGGGAGGC	141	-3	0.32%
CAGTAGGGGGCCTGAGA------AGGC	73	-6	0.17%
CAGTAGGGGGCCTGAGAgGGAGGGAGGC	72	+1 g	0.16%
CAGT------------AGGAGGGAGGC	74	-12	0.17%
CAG------------CAGGAGGGAGGC	62	-12	0.14%

Total	INDEL		Ins	Del	HDR
222170	26.01%	24h	16.02%	9.99%	0.00%

Typical seqs	Reads	Type	pct.
CAGTAGGGGGCCTGAGAGGA**GGG**AGGC	164391	WT	73.99%
CAGTAGGGGGCCTGAGAaGGAGGGAGGC	29294	+1 a	13.19%
CAGTAGGGGGCCTGAG-GGAGGGAGGC	2182	-1	0.98%
CAGT------------AGGAGGGAGGC	2087	-12	0.94%
CAGTAGGGGGCCT--GAGGAGGGAGGC	1814	-2	0.82%
CAGTAGGGGGCCTGAGA-GAGGGAGGC	1267	-1	0.57%
CAGTAGGGGGCCTGAGAtGGAGGGAGGC	1060	+1 t	0.48%
CAGTAGGGGGCCTGAGA--------GC	852	-8	0.38%
CAGTAGGGGGCCTGAGA-----GAGGC	774	-5	0.35%
CAGTAGGGGGCCTGA---GAGGGAGGC	771	-3	0.35%

Total	INDEL		Ins	Del	HDR
94949	24.43%	48h	13.20%	11.23%	0.00%

Typical seqs	Reads	Type	pct.
CAGTAGGGGGCCTGAGAGGA**GGG**AGGC	71750	WT	75.57%
CAGTAGGGGGCCTGAGAaGGAGGGAGGC	10466	+1 a	11.02%
CAGTAGGGGGCCTGAG-GGAGGGAGGC	1164	-1	1.23%
CAGT---------------AGGGAGGC	891	-15	0.94%
CAGTAGGGGGCCT-----GAGGGAGGC	714	-5	0.75%
CAGTAGGG----------------GGC	615	-16	0.65%
CAGTA----------------GGAGGC	580	-16	0.61%
CAGTAGGGGGCCT--GAGGAGGGAGGC	580	-2	0.61%
CAGT------------AGGAGGGAGGC	548	-12	0.58%
CAGTAGGGGGCCTGA---GAGGGAGGC	466	-3	0.49%

P128-P137-YW-W9-RNPKO-Syn81crBCL11A3b

4h

Total	INDEL	Ins	Del	HDR
37226	8.36%	2.00%	6.36%	0.00%

Typical seqs	Reads	Type	pct.
TGATAACGCCAGTAACC|CGA**GGG**CCAG	34114	WT	91.64%
TGATAACGCCAGTAA-CCGAGGGCCAG	1944	-1	5.22%
TGATAACGCCAGTAACCcCGAGGGCCAG	593	+1 c	1.59%
TGATAACGCCAGTAA--CGAGGGCCAG	143	-2	0.38%
TGGTAACGCCAGTAA-CCGAGGGCCAG	24	-1	0.06%
TGATAACGCCAGTAACCCCCGAGGGCCAG	23	+2	0.06%
TGATAACGCCAGTAACCgCGAGGGCCAG	17	+1 g	0.05%
TGATAACGCCAGTAACCtCGAGGGCCAG	17	+1 t	0.05%
TGATAACGCCAGT-AGCCGAGGGCCAG	20	-1	0.05%
TGATAACGCCAGTAACCaCGAGGGCCAG	15	+1 a	0.04%

8h

Total	INDEL	Ins	Del	HDR
67699	0.21%	0.01%	0.20%	0.00%

Typical seqs	Reads	Type	pct.
TGATAACGCCAGTAACC|CGA**GGG**CCAG	67559	WT	99.79%
TGATAACGCCAGTAACCCGA-GGCCAG	71	-1	0.10%
TGATAACGCCAGTAA-CCGAGGGCCAG	30	-1	0.04%
TGATAACGCCAGT-ACCCGAGGGCCAG	13	-1	0.02%
TGATAACGCCAGT-AGCCGAGGGCCAG	12	-1	0.02%
TGATAACGCCAGTAACCcCGAGGGCCAG	6	+1 c	0.01%
TGATAACGCCAGTAACCCG-GGGCCAG	5	-1	0.01%
TGATA------------CGAGGGCCAG	3	-12	0.00%

12h

Total	INDEL	Ins	Del	HDR
63398	22.81%	5.74%	17.07%	0.00%

Typical seqs	Reads	Type	pct.
TGATAACGCCAGTAACC|CGA**GGG**CCAG	48939	WT	77.19%
TGATAACGCCAGTAA-CCGAGGGCCAG	7992	-1	12.61%
TGATAACGCCAGTAACCcCGAGGGCCAG	2780	+1 c	4.38%
TGATAACGCCAGTAA--CGAGGGCCAG	1211	-2	1.91%
TGATAACGCCAGTA---CGAGGGCCAG	174	-3	0.27%
TGATAACGCCAGTAACCaCGAGGGCCAG	161	+1 a	0.25%
TGATAACGCCAGTAACCgCGAGGGCCAG	103	+1 g	0.16%
TGATAACGCCAGTAACCCCCGAGGGCCAG	99	+2	0.16%
CGATAACGCCAGTAA-CCGAGGGCCAG	61	-1	0.10%
TGATAACGCCAGTAA-ACGAGGGCCAG	54	-1	0.09%

24h

Total	INDEL	Ins	Del	HDR
45024	67.45%	14.12%	53.33%	0.00%

Typical seqs	Reads	Type	pct.
TGATAACGCCAGTAACC|CGA**GGG**CCAG	14656	WT	32.55%
TGATAACGCCAGTAA-CCGAGGGCCAG	14805	-1	32.88%
TGATAACGCCAGTAACCcCGAGGGCCAG	4173	+1 c	9.27%
TGATAACGCCAGTAA--CGAGGGCCAG	2824	-2	6.27%
TGATAAC---------------GCCAG	737	-15	1.64%
TGATAACGCCAGTAACCaCGAGGGCCAG	466	+1 a	1.04%
TGATAA-----------CGAGGGCCAG	343	-11	0.76%
TGATAACGCCAGTAACCgCGAGGGCCAG	292	+1 g	0.65%
TGATAACGCCAGTAACC--AGGGCCAG	227	-2	0.50%
TGATAACGCCAGTAA-ACGAGGGCCAG	207	-1	0.46%

48h

Total	INDEL	Ins	Del	HDR
21924	73.92%	10.99%	62.93%	0.00%

Typical seqs	Reads	Type	pct.
TGATAACGCCAGTAACC|CGA**GGG**CCAG	5718	WT	26.08%
TGATAACGCCAGTAA-CCGAGGGCCAG	7171	-1	32.71%
TGATAACGCCAGTAACCcCGAGGGCCAG	1595	+1 c	7.28%
TGATAAC---------------GCCAG	1334	-15	6.08%
TGATAACGCCAGTAA--CGAGGGCCAG	1009	-2	4.60%
TGATAA-----------CGAGGGCCAG	552	-11	2.52%
TGATAACGCCAGTAACCaCGAGGGCCAG	180	+1 a	0.82%
TGATAACGCCAGTA---CGAGGGCCAG	144	-3	0.66%
TGATAACGCCAGTAA--------CCAG	144	-8	0.66%
TGATAACGCCAGT--------------	208	-14	0.95%

P128-P137-YW-W9-RNPKO-Syn82crBCL11A4a

4h

Total | INDEL	Ins	Del	HDR
37445 | 3.99%	**3.73%**	**0.27%**	**0.00%**

**Typical seqs**	**Reads**	**Type**	**pct.**
TCGTAACACACCGTCATCCGGGGAAGT	35950	WT	96.01%
TCGTAACACACCGTCATtCCGGGGAAGT	1261	+1 t	3.37%
TCGTAACACACCGTC-TCCGGGGAAGT	35	-1	0.09%
TCGTAACACACCGTCATCC-GGGAAGT	33	-1	0.09%
TCGTAACACACCGTCATATCCGGGGAAGT	27	+2	0.07%
TCGTAACACACCGTCAT-CGGGGAAGT	11	-1	0.03%
TCGTAACACACCGCCATTCCGGGGAAGT	11	+1 Ins	0.03%
TCGTAACACACCGTCATCTCCGGGGAAGT	11	+2	0.03%
TCGTAACACGCCGTCATTCCGGGGAAGT	8	+1 Ins	0.02%
CCGTAACACACCGTCATTCCGGGGAAGT	7	+1 Ins	0.02%

8h

Total | INDEL	Ins	Del	HDR
85174 | 0.13%	**0.03%**	**0.10%**	**0.00%**

**Typical seqs**	**Reads**	**Type**	**pct.**
TCGTAACACACCGTCATCCGGGGAAGT	85062	WT	99.87%
TCGTAACACACCGTCATCC-GGGAAGT	73	-1	0.09%
TCGTAACACACCGTCATtCCGGGGAAGT	23	+1 t	0.03%
TCGTAACACACCGTCAT-CGGGGAAGT	7	-1	0.01%
TCGTAACACACCGTCATcCAGGGGAAGT	6	+1 c	0.01%
TCGTAACACA-------CCGGGGAAGT	3	-7	0.00%

Total | INDEL	Ins	Del	HDR
85174 | 0.13%	**0.03%**	**0.10%**	**0.00%**
**Typical seqs**	**Reads**	**Type**	**pct.**
TCGTAACACACCGTCATCCGGGGAAGT	85062	WT	99.87%

12h

Total | INDEL	Ins	Del	HDR
68918 | 39.10%	**31.94%**	**7.17%**	**0.00%**

**Typical seqs**	**Reads**	**Type**	**pct.**
TCGTAACACACCGTCATCCGGGGAAGT	41969	WT	60.90%
TCGTAACACACCGTCATtCCGGGGAAGT	19026	+1 t	27.61%
TCGTAACACA-------CCGGGGAAGT	1642	-7	2.38%
TCGTAACACACCGTC-TCCGGGGAAGT	814	-1	1.18%
TCGTAACACACCGTCATATCCGGGGAAGT	277	+2	0.40%
TCGTAACACACCGTCAT-CGGGGAAGT	177	-1	0.26%
TCGTAACACGCCGTCATTCCGGGGAAGT	173	+1 Ins	0.25%
TCGTAACACACCG---TCCGGGGAAGT	176	-3	0.26%
TCGTAACACACCGTCA-CCGGGGAAGT	173	-1	0.25%
TCGTAACACACCGTCAT--GGGGAAGT	148	-2	0.21%

24h

Total | INDEL	Ins	Del	HDR
32988 | 12.22%	**11.07%**	**1.15%**	**0.00%**

**Typical seqs**	**Reads**	**Type**	**pct.**
TCGTAACACACCGTCATCCGGGGAAGT	28956	WT	87.78%
TCGTAACACACCGTCATtCCGGGGAAGT	3335	+1 t	10.11%
TCGTAACACACCGTC-TCCGGGGAAGT	250	-1	0.76%
TCGTAACACA-------CCGGGGAAGT	67	-7	0.20%
TCGTAACACACCG---TCCGGGGAAGT	35	-3	0.11%
TCGTAACACGCCGTCATTCCGGGGAAGT	26	+1 Ins	0.08%
TCGTAACGCACCGTCATTCCGGGGAAGT	31	+1 Ins	0.09%
TCGTAACACACCGTCATtCCGGGGAGGT	28	+1 t	0.08%
TCGTAACACACCGTCATCC-GGGAAGT	22	-1	0.07%
TCGTAACACACCGTCGTTCCGGGGAAGT	18	+1 Ins	0.05%

48h

Total | INDEL	Ins	Del	HDR
47983 | 43.67%	**28.93%**	**14.74%**	**0.00%**

**Typical seqs**	**Reads**	**Type**	**pct.**
TCGTAACACACCGTCATCCGGGGAAGT	27027	WT	56.33%
TCGTAACACACCGTCATtCCGGGGAAGT	12006	+1 t	25.02%
TCGTAACACA-------CCGGGGAAGT	3164	-7	6.59%
TCGTAACACACCGTC-TCCGGGGAAGT	388	-1	0.81%
TCGTAACACACCG---TCCGGGGAAGT	263	-3	0.55%
TCGTAACACACCGTCATATCCGGGGAAGT	185	+2	0.39%
TCGTAACACACCGTCA-CCGGGGAAGT	132	-1	0.28%
TCGTAACACACCGTCAT-CGGGGAAGT	133	-1	0.28%
TCGT-------------------AAGT	105	-19	0.22%
TCGTAACACACCGTCAT--GGGGAAGT	92	-2	0.19%

P128-P137-YW-W9-RNPKO-Syn93crBCL11A7c

Total | INDEL	4h	Ins	Del	HDR
121225 | 10.23%		4.98%	5.25%	0.00%

Typical seqs	Reads	Type	pct.
AGCTTCGTGACGTTGGA|TGG**AGG**GTTT	108824	WT	89.77%
AGCTTCGTGACGTTGGAaTGGAGGGTTT	4527	+1 a	3.73%
AGCTTCGTGACGTTGGA-GGAGGGTTT	3248	-1	2.68%
AGCTTCGTGACGT----TGGAGGGTTT	1663	-4	1.37%
AGCTTCGTGACGTTGGAtTGGAGGGTTT	776	+1 t	0.64%
AGCTTCGTGACGTTG--TGGAGGGTTT	189	-2	0.16%
AGCTTCGTGACGTTGG-TGGAGGGTTT	170	-1	0.14%
AGCTTCGTGACGTTGGAcTGGAGGGTTT	98	+1 c	0.08%
AGCTTCGTGACGTTGG-GGAGGGTTT	85	-1	0.07%
AGCTTCGTGACG-----TGGAGGGTTT	86	-5	0.07%

Total | INDEL	8h	Ins	Del	HDR
83894 | 14.07%		7.53%	6.54%	0.00%

Typical seqs	Reads	Type	pct.
AGCTTCGTGACGTTGGA|TGG**AGG**GTTT	72091	WT	85.93%
AGCTTCGTGACGTTGGAaTGGAGGGTTT	5347	+1 a	6.37%
AGCTTCGTGACGTTGGA-GGAGGGTTT	2578	-1	3.07%
AGCTTCGTGACGT----TGGAGGGTTT	1553	-4	1.85%
AGCTTCGTGACGTTG--TGGAGGGTTT	188	-2	0.22%
AGCTTCGTGACGTTGGAtTGGAGGGTTT	145	+1 t	0.17%
AGCTTCGTGACGTTGGA--GAGGGTTT	135	-2	0.16%
AGCTTCG----------TGGAGGGTTT	92	-10	0.11%
AGCTTCGTGACGTTGG-TGGAGGGTTT	86	-1	0.10%
AGCTTCGTGACGTTGG--GGAGGGTTT	71	-2	0.08%

Total | INDEL	12h	Ins	Del	HDR
77121 | 48.99%		33.83%	15.15%	0.00%

Typical seqs	Reads	Type	pct.
AGCTTCGTGACGTTGGA|TGG**AGG**GTTT	39343	WT	51.01%
AGCTTCGTGACGTTGGAtTGGAGGGTTT	17974	+1 t	23.31%
AGCTTCGTGACGTTGGAaTGGAGGGTTT	4655	+1 a	6.04%
AGCTTCGTGACGT----TGGAGGGTTT	4723	-4	6.12%
AGCTTCGTGACGTTGGA-GGAGGGTTT	2022	-1	2.62%
AGCTTCGTGACGTTGG--GGAGGGTTT	590	-2	0.77%
AGCTTCG----------TGGAGGGTTT	456	-10	0.59%
AGCTTCGTGACGTTGGAT---GGGTTT	348	-3	0.45%
AGCTTCGTGACGTTGGA---AGGGTTT	337	-3	0.44%
AGCTTCGTG--------TGGAGGGTTT	309	-8	0.40%

Total | INDEL	24h	Ins	Del	HDR
92046 | 52.48%		22.62%	29.86%	0.00%

Typical seqs	Reads	Type	pct.
AGCTTCGTGACGTTGGA|TGG**AGG**GTTT	43741	WT	47.52%
AGCTTCGTGACGTTGGAaTGGAGGGTTT	14265	+1 a	15.50%
AGCTTCGTGACGT----TGGAGGGTTT	10809	-4	11.74%
AGCTTCGTGACGTTGGA-GGAGGGTTT	6694	-1	7.27%
AGCTTCGTGACGTTGGAtTGGAGGGTTT	3486	+1 t	3.79%
AGCTTCG----------TGGAGGGTTT	577	-10	0.63%
AGCTTCGTGACGTTG--TGGAGGGTTT	553	-2	0.60%
AGCTTCGTGACGTTGGA--GAGGGTTT	512	-2	0.56%
AGCTTCGTGACGTT-------GGGTTT	408	-7	0.44%
AGCTTCGTGACGTTGG-TGGAGGGTTT	382	-1	0.42%

Total | INDEL	48h	Ins	Del	HDR
109179 | 65.50%		25.84%	39.66%	0.00%

Typical seqs	Reads	Type	pct.
AGCTTCGTGACGTTGGA|TGG**AGG**GTTT	37667	WT	34.50%
AGCTTCGTGACGT----TGGAGGGTTT	21093	-4	19.32%
AGCTTCGTGACGTTGGAaTGGAGGGTTT	14840	+1 a	13.59%
AGCTTCGTGACGTTGGAtTGGAGGGTTT	9183	+1 t	8.41%
AGCTTCGTGACGTTGGA-GGAGGGTTT	6525	-1	5.98%
AGCTTCGTGACGTT-------GGGTTT	1087	-7	1.00%
AGCTTCGTGAC------------GTTT	1066	-12	0.98%
AGCTTCGTGACGTT--------GGTTT	843	-8	0.77%
AGCTTCGTGACGTTG--TGGAGGGTTT	724	-2	0.66%
AGCTTCG----------TGGAGGGTTT	658	-10	0.60%

P128-P137-YW-W9-RNPKO-Syn95crBCL11A8a

Total | INDEL	4h	Ins	Del	HDR
108510 | 10.87%		9.85%	1.03%	0.00%

Typical seqs	Reads	Type	pct.
GCTTCTGAGCGTCAAAC|TCGAGGAGCA	96710	WT	89.13%
GCTTCTGAGCGTCAAACcTCGAGGAGCA	8897	+1 c	8.20%
GCTTCTGAGCGTCAAACtTCGAGGAGCA	431	+1 t	0.40%
GCTTCTGAGCGTCAAAC-CGAGGAGCA	234	-1	0.22%
GCTTCTGAGCGTCAAA--CGAGGAGCA	148	-2	0.14%
GCTTCTGAGCG------TCGAGGAGCA	137	-6	0.13%
GCTTCTGAGCGTCAAA-TCGAGGAGCA	134	-1	0.12%
GCTTCTGAGCGTC-AACTCGAGGAGCA	106	-1	0.10%
GCTTCTGAGCGTCAAACACTCGAGGAGCA	106	+2	0.10%
GCTTCTGAGCGTCAAACaTCGAGGAGCA	76	+1 a	0.07%

Total | INDEL	8h	Ins	Del	HDR
108463 | 21.46%		17.85%	3.60%	0.00%

Typical seqs	Reads	Type	pct.
GCTTCTGAGCGTCAAAC|TCGAGGAGCA	85191	WT	78.54%
GCTTCTGAGCGTCAAACcTCGAGGAGCA	15536	+1 c	14.32%
GCTTCTGAGCGTCAAACtTCGAGGAGCA	1148	+1 t	1.06%
GCTTCTGAGCG------TCGAGGAGCA	730	-6	0.67%
GCTTCTGAGCGTCAAAC-CGAGGAGCA	558	-1	0.51%
GCTTCTGAGCGTCAAA--CGAGGAGCA	336	-2	0.31%
GCTTCTGAGCGTCAAACACTCGAGGAGCA	270	+2	0.25%
GCTTCT----------------GAGCA	186	-16	0.17%
GCTTCT-------------GAGGAGCA	175	-13	0.16%
GCT--------------TCGAGGAGCA	155	-14	0.14%

Total | INDEL	12h	Ins	Del	HDR
75183 | 19.89%		14.92%	4.97%	0.00%

Typical seqs	Reads	Type	pct.
GCTTCTGAGCGTCAAAC|TCGAGGAGCA	60231	WT	80.11%
GCTTCTGAGCGTCAAACcTCGAGGAGCA	8152	+1 c	10.84%
GCTTCTGAGCG------TCGAGGAGCA	744	-6	0.99%
GCTTCTGAGCGTCAAACtTCGAGGAGCA	647	+1 t	0.86%
GCTTCTGAGCGTCAAAC-CGAGGAGCA	513	-1	0.68%
GCTTCTGAGCGTCAAACACTCGAGGAGCA	424	+2	0.56%
GCTTCTGAGCGTCAAACgTCGAGGAGCA	371	+1 g	0.49%
GCTTCTG----------TCGAGGAGCA	344	-10	0.46%
GCTTCTGAGCGTCAAACaTCGAGGAGCA	279	+1 a	0.37%
GCTTCTG---------CT----GAGCA	266	-13	0.35%

Total | INDEL	24h	Ins	Del	HDR
85178 | 52.45%		35.22%	17.23%	0.00%

Typical seqs	Reads	Type	pct.
GCTTCTGAGCGTCAAAC|TCGAGGAGCA	40500	WT	47.55%
GCTTCTGAGCGTCAAACcTCGAGGAGCA	21112	+1 c	24.79%
GCTTCTGAGCGTCAAACtTCGAGGAGCA	3305	+1 t	3.88%
GCTTCTGAGCG------TCGAGGAGCA	2163	-6	2.54%
GCTTCTGAGCGTCAAAC-CGAGGAGCA	1003	-1	1.18%
GCT--------------TCGAGGAGCA	855	-14	1.00%
GCTTCT----------------GAGCA	736	-16	0.86%
GCTTCTGAGCGTCAAA--CGAGGAGCA	644	-2	0.76%
GCTTCTGAGCGTC-AACTCGAGGAGCA	534	-1	0.63%
GCTTCTGAGCGTCAAACgTCGAGGAGCA	482	+1 g	0.57%

Total | INDEL	48h	Ins	Del	HDR
112922 | 60.36%		36.58%	23.79%	0.00%

Typical seqs	Reads	Type	pct.
GCTTCTGAGCGTCAAAC|TCGAGGAGCA	44757	WT	39.64%
GCTTCTGAGCGTCAAACcTCGAGGAGCA	28585	+1 c	25.31%
GCTTCTGAGCGTCAAACtTCGAGGAGCA	4843	+1 t	4.29%
GCTTCTGAGCG------TCGAGGAGCA	4874	-6	4.32%
GCTTCT----------------GAGCA	2170	-16	1.92%
GCT--------------TCGAGGAGCA	1363	-14	1.21%
GCTTCTGAGCGTCAAAC-CGAGGAGCA	1109	-1	0.98%
GCTTCTGAGCGTCAAA--CGAGGAGCA	732	-2	0.65%
GCTTCTGAGCGTCA------AGGAGCA	748	-6	0.66%
GCTTCTGAGCGTCAAACACTCGAGGAGCA	744	+2	0.66%

P128-P137-YW-W9-RNPKO-Syn97crBCL11A8c

Total | INDEL	4h	Ins	Del	HDR
40746 | 1.34%		0.58%	0.76%	0.00%

Typical seqs	Reads	Type	pct.
CGCGGCAGAAACCTGTC|GTC**TGG**ACAC	40200	WT	98.66%
CGCGGCAGAAACCTGTCcGTCTGGACAC	213	+1 c	0.52%
CGCGGCAGAAACCT---GTCTGGACAC	182	-3	0.45%
CGCGGCAGAAACCTGTC-TCTGGACAC	29	-1	0.07%
CGCGGCAGAAACCTGTCGTCT-GACAC	26	-1	0.06%
CGCGGCAGAAACCTGT-GTCTGGACAC	16	-1	0.04%
CGCGGCAGAAAC----CGTCTGGACAC	11	-4	0.03%
CGCGGCAGAAACCTG-CGTCTGGACAC	8	-1	0.02%
CGCGGCAGAAAC-------CTGGACAC	7	-7	0.02%
CGCGGCAGAAACCTGTCaGTCTGGACAC	6	+1 a	0.01%

Total | INDEL	8h	Ins	Del	HDR
90548 | 5.38%		2.13%	3.25%	0.00%

Typical seqs	Reads	Type	pct.
CGCGGCAGAAACCTGTC|GTC**TGG**ACAC	85674	WT	94.62%
CGCGGCAGAAACCT---GTCTGGACAC	1684	-3	1.86%
CGCGGCAGAAACCTGTCcGTCTGGACAC	1482	+1 c	1.64%
CGCGGCAGAAACCTGTCGTCT-GACAC	128	-1	0.14%
CGCGGCAGAAACCTGTC-TCTGGACAC	127	-1	0.14%
CGCGGCAGAAACCTGT-GTCTGGACAC	124	-1	0.14%
CGCGGCAGAAACCTG--GTCTGGACAC	86	-2	0.09%
CGCGGCAGAAAC----CGTCTGGACAC	76	-4	0.08%
CGCGGCAGAAACCTGTC--CTGGACAC	63	-2	0.07%
CGCGGCAGAAACCTGTC----GGACAC	60	-4	0.07%

Total | INDEL	12h	Ins	Del	HDR
56984 | 10.00%		3.23%	6.77%	0.00%

Typical seqs	Reads	Type	pct.
CGCGGCAGAAACCTGTC|GTC**TGG**ACAC	51284	WT	90.00%
CGCGGCAGAAACCT---GTCTGGACAC	2139	-3	3.75%
CGCGGCAGAAACCTGTCcGTCTGGACAC	1519	+1 c	2.67%
CGCGGCAGAAACCTGT-GTCTGGACAC	157	-1	0.28%
CGCGGCAGAAACCTGTC-TCTGGACAC	138	-1	0.24%
CGCGGCAGAAAC-------CTGGACAC	107	-7	0.19%
CGCGGCAGAAAC----CGTCTGGACAC	89	-4	0.16%
CGCGGCAGAAACCTG--GTCTGGACAC	96	-2	0.17%
CGCGGCAGAAACCT-------------	80	-15	0.14%
CGCGGCAGAAACCTGTC--CTGGACAC	69	-2	0.12%

Total | INDEL	24h	Ins	Del	HDR
27111 | 16.13%		3.66%	12.46%	0.00%

Typical seqs	Reads	Type	pct.
CGCGGCAGAAACCTGTC|GTC**TGG**ACAC	22739	WT	83.87%
CGCGGCAGAAACCT---GTCTGGACAC	1923	-3	7.09%
CGCGGCAGAAACCTGTCcGTCTGGACAC	788	+1 c	2.91%
CGCGGCAGAAAC-------CTGGACAC	140	-7	0.52%
CGCGGCAGAAACCTGT-GTCTGGACAC	102	-1	0.38%
CGCGGCAGAAACCTGTC-TCTGGACAC	94	-1	0.35%
CGCGGCAGAAAC----CGTCTGGACAC	85	-4	0.31%
CGCGGCAGAAACCT-------------	95	-15	0.35%
CGCGGCAGAAACCTG--GTCTGGACAC	71	-2	0.26%
CGCGGCAGAAACCTGTC----GGACAC	47	-4	0.17%

Total | INDEL	48h	Ins	Del	HDR
75962 | 25.68%		3.45%	22.24%	0.00%

Typical seqs	Reads	Type	pct.
CGCGGCAGAAACCTGTC|GTC**TGG**ACAC	56452	WT	74.32%
CGCGGCAGAAACCT---GTCTGGACAC	10805	-3	14.22%
CGCGGCAGAAACCTGTCcGTCTGGACAC	1828	+1 c	2.41%
CGCGGCAGAAAC-------CTGGACAC	1329	-7	1.75%
CGCGGCAGAAACCTGT-GTCTGGACAC	297	-1	0.39%
CGCGGCAGAAAC----CGTCTGGACAC	278	-4	0.37%
CGCGGCAGAAACCTG--GTCTGGACAC	210	-2	0.28%
CGCGGCAGAAACCT-------------	208	-15	0.27%
CGCGGCAGAAAC--------------C	177	-14	0.23%
CGCGGCAGAA-------------ACAC	165	-13	0.22%

P128-P137-YW-W9-RNPKO-Syn98crBCL11A8d

4h

Total	INDEL	Ins	Del	HDR
85804	10.16%	7.53%	2.63%	0.00%

Typical seqs	Reads	Type	pct.
GCCTTGATTACTCAAAC|AAC**AGG**TTAC	77084	WT	89.84%
GCCTTGATTACTCAAACcAACAGGTTAC	5571	+1 c	6.49%
GCCTTGATTACTCA---AACAGGTTAC	891	-3	1.04%
GCCTTGATTACTCAAA-AACAGGTTAC	323	-1	0.38%
GCCTTGATTACTC-AACAACAGGTTAC	182	-1	0.21%
GCCTTGATTACT----CAACAGGTTAC	154	-4	0.18%
GCCTTGATTACTCAAACaAACAGGTTAC	140	+1 a	0.16%
GCCTTGATTACTCAA--AACAGGTTAC	134	-2	0.16%
GCCTTGATTACTCAAAC-ACAGGTTAC	89	-1	0.10%
GCCTTGATTACTCAAACtAACAGGTTAC	81	+1 t	0.09%

8h

Total	INDEL	Ins	Del	HDR
156749	21.51%	14.04%	7.47%	0.00%

Typical seqs	Reads	Type	pct.
GCCTTGATTACTCAAAC|AAC**AGG**TTAC	123039	WT	78.49%
GCCTTGATTACTCAAACcAACAGGTTAC	18837	+1 c	12.02%
GCCTTGATTACTCA---AACAGGTTAC	4753	-3	3.03%
GCCTTGATTACT----CAACAGGTTAC	706	-4	0.45%
GCCTTGATTACTCAA--AACAGGTTAC	621	-2	0.40%
GCCTTGATTACTCAAA-AACAGGTTAC	547	-1	0.35%
GCCTTGATTACTCAAACaAACAGGTTAC	476	+1 a	0.30%
GCCTTGATTACTC-AACAACAGGTTAC	429	-1	0.27%
GCCTTGATTACTCAAAC-ACAGGTTAC	427	-1	0.27%
GCCTTGA----------------TTAC	295	-16	0.19%

12h

Total	INDEL	Ins	Del	HDR
94643	31.20%	18.60%	12.61%	0.00%

Typical seqs	Reads	Type	pct.
GCCTTGATTACTCAAAC|AAC**AGG**TTAC	65110	WT	68.80%
GCCTTGATTACTCAAACcAACAGGTTAC	14723	+1 c	15.56%
GCCTTGATTACTCA---AACAGGTTAC	4376	-3	4.62%
GCCTTGATTACT----CAACAGGTTAC	660	-4	0.70%
GCCTTGATTACTCAA--AACAGGTTAC	485	-2	0.51%
GCCTTGATTACTCAAAC-ACAGGTTAC	513	-1	0.54%
GCCTTGATTACTCAAACaAACAGGTTAC	475	+1 a	0.50%
GCCTTGATTACTCAAA-AACAGGTTAC	422	-1	0.45%
GCCTTGATTACTC-AACAACAGGTTAC	354	-1	0.37%
GCCTTGATTACTCA-------------	360	-14	0.38%

24h

Total	INDEL	Ins	Del	HDR
101607	52.34%	24.73%	27.61%	0.00%

Typical seqs	Reads	Type	pct.
GCCTTGATTACTCAAAC|AAC**AGG**TTAC	48426	WT	47.66%
GCCTTGATTACTCAAACcAACAGGTTAC	19918	+1 c	19.60%
GCCTTGATTACTCA---AACAGGTTAC	9842	-3	9.69%
GCCTTGATTACT----CAACAGGTTAC	1286	-4	1.27%
GCCTTGATTACTCAA--AACAGGTTAC	1088	-2	1.07%
GCCTTGATTACTCA-------------	1072	-14	1.06%
GCCTTGA----------------TTAC	1020	-16	1.00%
GCCTTGATTACTCAAACaAACAGGTTAC	865	+1 a	0.85%
GCCTTGATTACTCAAAC-ACAGGTTAC	800	-1	0.79%
GCCTTGATTACTC-AACAACAGGTTAC	786	-1	0.77%

48h

Total	INDEL	Ins	Del	HDR
150279	66.59%	23.78%	42.80%	0.00%

Typical seqs	Reads	Type	pct.
GCCTTGATTACTCAAAC|AAC**AGG**TTAC	50215	WT	33.41%
GCCTTGATTACTCAAACcAACAGGTTAC	27483	+1 c	18.29%
GCCTTGATTACTCA---AACAGGTTAC	26258	-3	17.47%
GCCTTGA----------------TTAC	3018	-16	2.01%
GCCTTGATTACT----CAACAGGTTAC	2951	-4	1.96%
GCCTTGATTACTCA-------------	2250	-14	1.50%
GCCTTGATTACTCAA--AACAGGTTAC	1826	-2	1.22%
GCCTTGATT---------ACAGGTTAC	1434	-9	0.95%
GCCTTGATTACTCAA----------AC	1396	-10	0.93%
GCCTTGATTACTCAAACaAACAGGTTAC	1245	+1 a	0.83%

P128-P137-YW-W9-RNPKO-Syn100crBCL11A9b

4h

Total	INDEL	Ins	Del	HDR
24686	12.82%	7.61%	5.21%	0.00%

Typical seqs	Reads	Type	pct.
GTCGGACTTGACCGTCA|TGG**GGG**ACGA	21521	WT	87.18%
GTCGGACTTGACCGTCAaTGGGGGACGA	1466	+1 a	5.94%
GTCGGACTTGACCGTCA-GGGGGACGA	620	-1	2.51%
GTCGGACTTGACCGTC-TGGGGGACGA	248	-1	1.00%
GTCGGACTTGACCGTCA--GGGGACGA	181	-2	0.73%
GTCGGACTTGACCGTCAtTGGGGGACGA	157	+1 t	0.64%
GTCGGACTTGACCGTCAT-GGGGACGA	61	-1	0.25%
GTCGGACTTGACCGTCAgTGGGGGACGA	44	+1 g	0.18%
GTCGGACTTGACCGTCAcTGGGGGACGA	40	+1 c	0.16%
GTCGGACTTGACCGTCCATGGGGGACGA	19	+1 Ins	0.08%

8h

Total	INDEL	Ins	Del	HDR
66294	33.08%	20.61%	12.47%	0.00%

Typical seqs	Reads	Type	pct.
GTCGGACTTGACCGTCA|TGG**GGG**ACGA	44366	WT	66.92%
GTCGGACTTGACCGTCAaTGGGGGACGA	10560	+1 a	15.93%
GTCGGACTTGACCGTCA-GGGGGACGA	3213	-1	4.85%
GTCGGACTTGACCGTC-TGGGGGACGA	1265	-1	1.91%
GTCGGACTTGACCGTCA--GGGGACGA	1174	-2	1.77%
GTCGGACTTGACCGTCAtTGGGGGACGA	1079	+1 t	1.63%
GTCGGACTT-------------GACGA	292	-13	0.44%
GTCGGACTTGACCGTC--GGGGGACGA	244	-2	0.37%
GTCGGACTTGACCGTCAT-GGGGACGA	229	-1	0.35%
GTCGGACTTGACCGTCAAATGGGGGACGA	190	+2	0.29%

12h

Total	INDEL	Ins	Del	HDR
64127	45.95%	25.88%	20.07%	0.00%

Typical seqs	Reads	Type	pct.
GTCGGACTTGACCGTCA|TGG**GGG**ACGA	34659	WT	54.05%
GTCGGACTTGACCGTCAaTGGGGGACGA	12472	+1 a	19.45%
GTCGGACTTGACCGTCA-GGGGGACGA	5504	-1	8.58%
GTCGGACTTGACCGTCAtTGGGGGACGA	1891	+1 t	2.95%
GTCGGACTTGACCGTC-TGGGGGACGA	1290	-1	2.01%
GTCGGACTTGACCGTCA--GGGGACGA	1189	-2	1.85%
GTCGGACTTGACCGTCAgTGGGGGACGA	300	+1 g	0.47%
GTCGGACTTGACCGTCAcTGGGGGACGA	277	+1 c	0.43%
GTCGGACTTGACCGTC--GGGGGACGA	248	-2	0.39%
GTCGGACT---------TGGGGGACGA	242	-9	0.38%

24h

Total	INDEL	Ins	Del	HDR
17473	65.03%	36.57%	28.46%	0.00%

Typical seqs	Reads	Type	pct.
GTCGGACTTGACCGTCA|TGG**GGG**ACGA	6110	WT	34.97%
GTCGGACTTGACCGTCAaTGGGGGACGA	4437	+1 a	25.39%
GTCGGACTTGACCGTCA-GGGGGACGA	1526	-1	8.73%
GTCGGACTTGACCGTCAtTGGGGGACGA	979	+1 t	5.60%
GTCGGACTTGACCGTC-TGGGGGACGA	417	-1	2.39%
GTCGGACTTGACCGTCA--GGGGACGA	361	-2	2.07%
GTC------------------GGACGA	219	-18	1.25%
GTCGGACTT-------------GACGA	176	-13	1.01%
GTCGGACTTGACCGTCAgTGGGGGACGA	149	+1 g	0.85%
GTCGGACTT----------------GA	128	-16	0.73%

48h

Total	INDEL	Ins	Del	HDR
88847	79.71%	42.60%	37.11%	0.00%

Typical seqs	Reads	Type	pct.
GTCGGACTTGACCGTCA|TGG**GGG**ACGA	18025	WT	20.29%
GTCGGACTTGACCGTCAaTGGGGGACGA	26084	+1 a	29.36%
GTCGGACTTGACCGTCA-GGGGGACGA	10492	-1	11.81%
GTCGGACTTGACCGTCAtTGGGGGACGA	5486	+1 t	6.17%
GTCGGACTTGACCGTC-TGGGGGACGA	1924	-1	2.17%
GTCGGACTTGACCGTCA--GGGGACGA	1674	-2	1.88%
GTCGGACTT-------------GACGA	1841	-13	2.07%
GTC------------------GGACGA	1361	-18	1.53%
GTCGGACTTGACCGTCAgTGGGGGACGA	787	+1 g	0.89%
GTCGGACTTGACC---------GACGA	711	-9	0.80%

P128-P137-YW-W9-RNPKO-Syn101crBCL11A9c

Total | INDEL	4h	Ins	Del	HDR
67002 | 5.24%		4.71%	0.54%	0.00%

Typical seqs	Reads	Type	pct.
GAGTACACGTTCTCCGT|GTTGGGCATC	63489	WT	94.76%
GAGTACACGTTCTCCGTtGTTGGGCATC	2816	+1 t	4.20%
GAGTACACGTTCTCC--GTTGGGCATC	128	-2	0.19%
GAGTACACGTTCTCC-TGTTGGGCATC	86	-1	0.13%
GAGTACACGTTCTCCGTGTT-GGCATC	54	-1	0.08%
GAGTACACGTTCTCCG-GTTGGGCATC	38	-1	0.06%
GAGTACACGTTCTCCGTaGTTGGGCATC	36	+1 a	0.05%
GAGTACGCGTTCTCCGTTGTTGGGCATC	31	+1 Ins	0.05%
GAGTACACGTTCTCCGT-TTGGGCATC	22	-1	0.03%
GAGTACACGTTCTC--TGTTGGGCATC	19	-2	0.03%

Total | INDEL	8h	Ins	Del	HDR
44742 | 15.93%		13.51%	2.43%	0.00%

Typical seqs	Reads	Type	pct.
GAGTACACGTTCTCCGT|GTTGGGCATC	37613	WT	84.07%
GAGTACACGTTCTCCGTtGTTGGGCATC	5372	+1 t	12.01%
GAGTACACGTTCTCC--GTTGGGCATC	364	-2	0.81%
GAGTACACGTTCTCC-TGTTGGGCATC	187	-1	0.42%
GAGTACGCGTTCTCCGTTGTTGGGCATC	50	+1 Ins	0.11%
GAGTACAC---------GTTGGGCATC	48	-9	0.11%
GAGTACACGTTCTCCGTGTT-GGCATC	45	-1	0.10%
GAGTACACGTTCTCCGT-TTGGGCATC	40	-1	0.09%
GAGTACACGTTCTC--TGTTGGGCATC	47	-2	0.11%
GAGTACACGTTCTCCTTTGTTGGGCATC	39	+1 Ins	0.09%

Total | INDEL	12h	Ins	Del	HDR
55188 | 28.03%		22.59%	5.45%	0.00%

Typical seqs	Reads	Type	pct.
GAGTACACGTTCTCCGT|GTTGGGCATC	39718	WT	71.97%
GAGTACACGTTCTCCGTtGTTGGGCATC	11208	+1 t	20.31%
GAGTACACGTTCTCC--GTTGGGCATC	798	-2	1.45%
GAGTACACGTTCTCC-TGTTGGGCATC	387	-1	0.70%
GAGTACAC---------GTTGGGCATC	238	-9	0.43%
GAGTACACGTTCTCCG-GTTGGGCATC	164	-1	0.30%
GAGTACACGTTCTCC-----GGGCATC	98	-5	0.18%
GAGTACGCGTTCTCCGTTGTTGGGCATC	71	+1 Ins	0.13%
GAGTACA-------CGTGTTGGGCATC	70	-7	0.13%
GGGTACACGTTCTCCGTTGTTGGGCATC	65	+1 Ins	0.12%

Total | INDEL	24h	Ins	Del	HDR
64243 | 35.41%		27.66%	7.75%	0.00%

Typical seqs	Reads	Type	pct.
GAGTACACGTTCTCCGT|GTTGGGCATC	41497	WT	64.59%
GAGTACACGTTCTCCGTtGTTGGGCATC	15907	+1 t	24.76%
GAGTACACGTTCTCC--GTTGGGCATC	1104	-2	1.72%
GAGTACAC---------GTTGGGCATC	573	-9	0.89%
GAGTACACGTTCTCC-TGTTGGGCATC	567	-1	0.88%
GAGTACACGTTCTCCG-GTTGGGCATC	184	-1	0.29%
GAGTACACGTTCTCCG---TGGGCATC	126	-3	0.20%
GAGTACGCGTTCTCCGTTGTTGGGCATC	126	+1 Ins	0.20%
GA---------------GTTGGGCATC	115	-15	0.18%
GAGTA------------------CATC	110	-18	0.17%

Total | INDEL	48h	Ins	Del	HDR
7470 | 39.97%		24.35%	15.62%	0.00%

Typical seqs	Reads	Type	pct.
GAGTACACGTTCTCCGT|GTTGGGCATC	4484	WT	60.03%
GAGTACACGTTCTCCGTtGTTGGGCATC	1607	+1 t	21.51%
GAGTACACGTTCTCC--GTTGGGCATC	220	-2	2.95%
GAGTACAC---------GTTGGGCATC	189	-9	2.53%
GAGTACACGT---------------TC	52	-15	0.70%
GAGTACACGTTCTCCG---TGGGCATC	44	-3	0.59%
GAGTACACGTTCTC---------CATC	43	-9	0.58%
GAGTACACGTTCT--------------	53	-15	0.71%
GAGT--------------------ATC	34	-20	0.46%
GAGTACACGTTCTCC-TGTTGGGCATC	31	-1	0.41%

P128-P137-YW-W9-RNPKO-Syn105crBCL11A9g

Total | INDEL	4h	Ins	Del	HDR
23562 | 15.20%		14.57%	0.63%	0.00%

Typical seqs	Reads	Type	pct.
CAACTTACAAATACCCT|GCGGGGCATA	19981	WT	84.80%
CAACTTACAAATACCCTtGCGGGGCATA	3026	+1 t	12.84%
CAACTTACAAATACCCTCTGCGGGGCATA	55	+2	0.23%
CAACTTACAAATA-CCTGCGGGGCATA	50	-1	0.21%
CAACTTACAAATACCC-GCGGGGCATA	33	-1	0.14%
CAGCTTACAAATACCCTTGCGGGGCATA	17	+1 Ins	0.07%
CAACTTACAAATACCCTTTGCGGGGCATA	16	+2	0.07%
CAACTTACAAATACCCT--GGGGCATA	15	-2	0.06%
CAACTTACAAATACCCTGC-GGGCATA	14	-1	0.06%
CAACTTACAAATACCCTtGTGGGGCATA	14	+1 t	0.06%

Total | INDEL	8h	Ins	Del	HDR
23512 | 31.26%		28.60%	2.65%	0.00%

Typical seqs	Reads	Type	pct.
CAACTTACAAATACCCT|GCGGGGCATA	16163	WT	68.74%
CAACTTACAAATACCCTtGCGGGGCATA	5733	+1 t	24.38%
CAACTTACAAATA-CCTGCGGGGCATA	196	-1	0.83%
CAACTTACAAATA--CTGCGGGGCATA	113	-2	0.48%
CAACTTACAAATACCCTCTGCGGGGCATA	88	+2	0.37%
CAACTTACAAATACCCCTGCGGGGCATA	68	+1 Ins	0.29%
CAACTTACAAATACCCTTTGCGGGGCATA	62	+2	0.26%
CAACTTACAAATACCC-GCGGGGCATA	60	-1	0.26%
CAACTTACAAATACCCTCCTGCGGGGCATA	46	+3	0.20%
CAACTTACAAATACCCTcGCGGGGCATA	41	+1 c	0.17%

Total | INDEL	12h	Ins	Del	HDR
23384 | 61.74%		57.16%	4.58%	0.00%

Typical seqs	Reads	Type	pct.
CAACTTACAAATACCCT|GCGGGGCATA	8947	WT	38.26%
CAACTTACAAATACCCTtGCGGGGCATA	11641	+1 t	49.78%
CAACTTACAAATA-CCTGCGGGGCATA	249	-1	1.06%
CAACTTACAAATA--CTGCGGGGCATA	190	-2	0.81%
CAACTTACAAATACCCTCTGCGGGGCATA	109	+2	0.47%
CAACTTACAAATACCCTTTGCGGGGCATA	104	+2	0.44%
CAACTTACAAATACCCCTGCGGGGCATA	80	+1 Ins	0.34%
CAACCTACAAATACCCTTGCGGGGCATA	72	+1 Ins	0.31%
CAACTTACAAATACCC-GCGGGGCATA	67	-1	0.29%
CAACTTACAAATACCCTCCTGCGGGGCATA	65	+3	0.28%

Total | INDEL	24h	Ins	Del	HDR
26806 | 87.59%		78.56%	9.03%	0.00%

Typical seqs	Reads	Type	pct.
CAACTTACAAATACCCT|GCGGGGCATA	3327	WT	12.41%
CAACTTACAAATACCCTtGCGGGGCATA	18130	+1 t	67.63%
CAACTTACAAATA-CCTGCGGGGCATA	519	-1	1.94%
CAACTTACAAATACCCTCTGCGGGGCATA	248	+2	0.93%
CAACTTACAAATA--CTGCGGGGCATA	275	-2	1.03%
CAACTTACAAATACCCCTGCGGGGCATA	172	+1 Ins	0.64%
CAACTTACAAATACCCTTTGCGGGGCATA	164	+2	0.61%
CAACTTACAAATA---TGCGGGGCATA	121	-3	0.45%
CAACTTACAAATACCC-GCGGGGCATA	108	-1	0.40%
CAACTTACAA--------------ATA	91	-14	0.34%

Total | INDEL	48h	Ins	Del	HDR
43718 | 82.93%		72.61%	10.32%	0.00%

Typical seqs	Reads	Type	pct.
CAACTTACAAATACCCT|GCGGGGCATA	7463	WT	17.07%
CAACTTACAAATACCCTtGCGGGGCATA	27128	+1 t	62.05%
CAACTTACAAATA-CCTGCGGGGCATA	563	-1	1.29%
CAACTTACAAATA---TGCGGGGCATA	479	-3	1.10%
CAACTTACAAATACCCTTTGCGGGGCATA	410	+2	0.94%
CAACTTACAAATA--CTGCGGGGCATA	432	-2	0.99%
CAACTTACAAATACCC-GCGGGGCATA	343	-1	0.78%
CAACTTACAA--------------ATA	279	-14	0.64%
CAACTTACAAATACCCTCTGCGGGGCATA	242	+2	0.55%
CAACTTA--------------------	196	-25	0.45%

P128-P137-YW-W9-RNPKO-Syn106crBCL11A10a

4h — Total 67371 | INDEL 3.61% | Ins 2.34% | Del 1.26% | HDR 0.00%

Typical seqs	Reads	Type	pct.
GCGCTAAGTCACTGTCG|TCG**AGG**CCCC	64941	WT	96.39%
GCGCTAAGTCACTGTCGgTCGAGGCCCC	1187	+1 g	1.76%
GCGCTAAGTCACTGTC-TCGAGGCCCC	446	-1	0.66%
GCGCTAAGTCACT---GTCGAGGCCCC	268	-3	0.40%
GCGCTAAGTCACTGTCGCGTCGAGGCCCC	152	+2	0.23%
GCGCTAAGTCACTGTCGcTCGAGGCCCC	87	+1 c	0.13%
GCGCTAAGTCACTGTCGtTCGAGGCCCC	38	+1 t	0.06%
GCGCTAAGTCACTGTCTTTAAGG--CC	20	-2	0.03%
GCGCTAAGTCACTGT--TCGAGGCCCC	18	-2	0.03%
GCGCTA-----------TCGAGGCCCC	16	-11	0.02%

8h — Total 68117 | INDEL 13.13% | Ins 7.25% | Del 5.88% | HDR 0.00%

Typical seqs	Reads	Type	pct.
GCGCTAAGTCACTGTCG|TCG**AGG**CCCC	59173	WT	86.87%
GCGCTAAGTCACTGTCGgTCGAGGCCCC	3818	+1 g	5.61%
GCGCTAAGTCACT---GTCGAGGCCCC	1690	-3	2.48%
GCGCTAAGTCACTGTC-TCGAGGCCCC	1353	-1	1.99%
GCGCTAAGTCACTGTCGCGTCGAGGCCCC	360	+2	0.53%
GCGCTAAGTCACTGTCGtTCGAGGCCCC	177	+1 t	0.26%
GCGCTAA---------GTCGAGGCCCC	168	-9	0.25%
GCGCTAAGTCACTGTCGaTCGAGGCCCC	74	+1 a	0.11%
GCGCTAAGTCACTGTCG-CGAGGCCCC	51	-1	0.07%
GCGCTAAGTCACTGT--TCGAGGCCCC	50	-2	0.07%

12h — Total 83702 | INDEL 23.26% | Ins 11.98% | Del 11.28% | HDR 0.00%

Typical seqs	Reads	Type	pct.
GCGCTAAGTCACTGTCG|TCG**AGG**CCCC	64234	WT	76.74%
GCGCTAAGTCACTGTCGgTCGAGGCCCC	7476	+1 g	8.93%
GCGCTAAGTCACT---GTCGAGGCCCC	3606	-3	4.31%
GCGCTAAGTCACTGTC-TCGAGGCCCC	2527	-1	3.02%
GCGCTAAGTCACTGTCGCGTCGAGGCCCC	912	+2	1.09%
GCGCTAAGTCACTGTCGtTCGAGGCCCC	402	+1 t	0.48%
GCGCTAA---------GTCGAGGCCCC	341	-9	0.41%
GCGCTAAGTCA---------------C	216	-15	0.26%
GCGCTAAGTCACTGTCGaTCGAGGCCCC	215	+1 a	0.26%
GCGCTAAGTCACTGT--TCGAGGCCCC	166	-2	0.20%

24h — Total 30365 | INDEL 37.59% | Ins 16.21% | Del 21.38% | HDR 0.00%

Typical seqs	Reads	Type	pct.
GCGCTAAGTCACTGTCG|TCG**AGG**CCCC	18951	WT	62.41%
GCGCTAAGTCACTGTCGgTCGAGGCCCC	3542	+1 g	11.66%
GCGCTAAGTCACT---GTCGAGGCCCC	2688	-3	8.85%
GCGCTAAGTCACTGTC-TCGAGGCCCC	1386	-1	4.56%
GCGCTAAGTCACTGTCGCGTCGAGGCCCC	437	+2	1.44%
GCGCTAA---------GTCGAGGCCCC	321	-9	1.06%
GCGCTAAGTCACTGTCGtTCGAGGCCCC	289	+1 t	0.95%
GCGCTAAGTCA---------------C	143	-15	0.47%
GCGCTAAGTCACTGTCG-CGAGGCCCC	135	-1	0.44%
GCGCTAAGTCACTGT---------CCC	85	-9	0.28%

48h — Total 88485 | INDEL 47.45% | Ins 16.56% | Del 30.89% | HDR 0.00%

Typical seqs	Reads	Type	pct.
GCGCTAAGTCACTGTCG|TCG**AGG**CCCC	46499	WT	52.55%
GCGCTAAGTCACT---GTCGAGGCCCC	13140	-3	14.85%
GCGCTAAGTCACTGTCGgTCGAGGCCCC	10165	+1 g	11.49%
GCGCTAAGTCACTGTC-TCGAGGCCCC	4383	-1	4.95%
GCGCTAA---------GTCGAGGCCCC	1289	-9	1.46%
GCGCTAAGTCACTGTCGCGTCGAGGCCCC	1212	+2	1.37%
GCGCTAAGTCACTGTCGtTCGAGGCCCC	1020	+1 t	1.15%
GCGCTAAGTCA---------------C	457	-15	0.52%
GCGCTAAGTCACTGTCG-CGAGGCCCC	428	-1	0.48%
GCGCTAAGTCACTGTCGcTCGAGGCCCC	275	+1 c	0.31%

P128-P137-YW-W9-RNPKO-Syn107crBCL11A10b

4h

Total	INDEL	Ins	Del	HDR
83243	1.31%	0.64%	0.68%	0.00%

Typical seqs	Reads	Type	pct.
GACTATCATACAATTGG|GCAGGGGTGG	82149	WT	98.69%
GACTATCATACAATT-GGCAGGGGTGG	449	-1	0.54%
GACTATCATACAATTGGgGCAGGGGTGG	470	+1 g	0.56%
GACTATCATACAATTGGgGCAGGGGGGG	40	+1 g	0.05%
GACTATCATACAATT--GCAGGGGTGG	16	-2	0.02%
GACTATCATACAATT-GGCAGGGGGGG	13	-1	0.02%
GACTATCATA-------GCAGGGGTGG	11	-7	0.01%
GACTATCA---------GCAGGGGTGG	10	-9	0.01%
GACTATCATACAATTGGgGCAGGAGTGG	6	+1 g	0.01%
GACTATCATACAATTGGCGGCAGGGGTGG	5	+2	0.01%

8h

Total	INDEL	Ins	Del	HDR
72677	5.47%	2.52%	2.95%	0.00%

Typical seqs	Reads	Type	pct.
GACTATCATACAATTGG|GCAGGGGTGG	68703	WT	94.53%
GACTATCATACAATT-GGCAGGGGTGG	1517	-1	2.09%
GACTATCATACAATTGGgGCAGGGGTGG	1496	+1 g	2.06%
GACTATCATACAATTGGgGCAGGGGGGG	119	+1 g	0.16%
GACTATCATACAATT--GCAGGGGTGG	56	-2	0.08%
GACTATCATACAATT-----GGGGTGG	48	-5	0.07%
GACTATCATACAAT---GCAGGGGTGG	34	-3	0.05%
GACTATCATACAATT-----------G	32	-11	0.04%
GACTATCATACAATT------GGGTGG	31	-6	0.04%
GACTATCATACAATT-------GGTGG	28	-7	0.04%

12h

Total	INDEL	Ins	Del	HDR
40199	12.04%	5.09%	6.95%	0.00%

Typical seqs	Reads	Type	pct.
GACTATCATACAATTGG|GCAGGGGTGG	35361	WT	87.96%
GACTATCATACAATT-GGCAGGGGTGG	1612	-1	4.01%
GACTATCATACAATTGGgGCAGGGGTGG	1708	+1 g	4.25%
GACTATCATACAAT----------TGG	118	-10	0.29%
GACTATCATACAATT-------GGTGG	90	-7	0.22%
GACTATCATA--------CAGGGGTGG	72	-8	0.18%
GACTATCATACAATT--GCAGGGGTGG	71	-2	0.18%
GACTATCATACAATTGGgGCAGGGGGGG	108	+1 g	0.27%
GACTATCATACAATT-----GGGGTGG	40	-5	0.10%
GACTATCATACAATT------GGGTGG	33	-6	0.08%

24h

Total	INDEL	Ins	Del	HDR
54101	23.28%	7.14%	16.14%	0.00%

Typical seqs	Reads	Type	pct.
GACTATCATACAATTGG|GCAGGGGTGG	41508	WT	76.72%
GACTATCATACAATT-GGCAGGGGTGG	3357	-1	6.21%
GACTATCATACAATTGGgGCAGGGGTGG	3035	+1 g	5.61%
GACTATCATACAAT----------TGG	391	-10	0.72%
GACTATCATACAATT------GGGTGG	242	-6	0.45%
GACTATCATACAATT-----GGGGTGG	208	-5	0.38%
GACTATCATA--------CAGGGGTGG	214	-8	0.40%
GACTATCATACAATT------------	337	-13	0.62%
---------------------------	121	-30	0.22%
GACTATCATACAATT-----------G	123	-11	0.23%

48h

Total	INDEL	Ins	Del	HDR
76930	27.01%	5.98%	21.03%	0.00%

Typical seqs	Reads	Type	pct.
GACTATCATACAATTGG|GCAGGGGTGG	56153	WT	72.99%
GACTATCATACAATT-GGCAGGGGTGG	4379	-1	5.69%
GACTATCATACAATTGGgGCAGGGGTGG	3394	+1 g	4.41%
GACTATCATACAAT----------TGG	829	-10	1.08%
GACTATCATACAATT-----GGGGTGG	671	-5	0.87%
GACTATCATACAATT-------GGTGG	578	-7	0.75%
GACTATCATACAATT------GGGTGG	405	-6	0.53%
GACTA-------------------TGG	362	-19	0.47%
GACTATCATA--------CAGGGGTGG	384	-8	0.50%
GACTATCA----------------TGG	252	-16	0.33%

P128-P137-YW-W9-RNPKO-Syn108crBCL11A10c

Total | INDEL	4h	Ins	Del	HDR
59144 | 7.20%		6.30%	0.90%	0.00%

Typical seqs	Reads	Type	pct.
GTGGGAACTTTGCCGAT|ATG**AGG**ATGG	54886	WT	92.80%
GTGGGAACTTTGCCGATtATGAGGATGG	3409	+1 t	5.76%
GTGGGAACTTTGCCG--ATGAGGATGG	129	-2	0.22%
GTGGGAACTTTGCCG-TATGAGGATGG	129	-1	0.22%
GTGGGAACTTTGCC--TATGAGGATGG	84	-2	0.14%
GTGGGAACTTTGCCGA-ATGAGGATGG	42	-1	0.07%
GTGGGAACTTTGCCGAT-TGAGGATGG	31	-1	0.05%
GTGGGAGCTTTGCCGATTATGAGGATGG	27	+1 Ins	0.05%
GTGGGAACTTTGCC-----GAGGATGG	26	-5	0.04%
GTGGGAACTCTGCCGATTATGAGGATGG	17	+1 Ins	0.03%

Total | INDEL	8h	Ins	Del	HDR
37791 | 22.18%		19.18%	2.99%	0.00%

Typical seqs	Reads	Type	pct.
GTGGGAACTTTGCCGAT|ATG**AGG**ATGG	29410	WT	77.82%
GTGGGAACTTTGCCGATtATGAGGATGG	6539	+1 t	17.30%
GTGGGAACTTTGCCG-TATGAGGATGG	271	-1	0.72%
GTGGGAACTTTGCCG--ATGAGGATGG	215	-2	0.57%
GTGGGAACTTTGCC-----GAGGATGG	159	-5	0.42%
GTGGGAACTTTGCC--TATGAGGATGG	92	-2	0.24%
GTGGGAACTTTGCCGA-ATGAGGATGG	62	-1	0.16%
GTGGGAACTTTGCC--------GATGG	78	-8	0.21%
GTGGGAGCTTTGCCGATTATGAGGATGG	42	+1 Ins	0.11%
GTGGGAACTTTGCCGATtATGGGGATGG	46	+1 t	0.12%

Total | INDEL	12h	Ins	Del	HDR
35501 | 47.24%		39.26%	7.97%	0.00%

Typical seqs	Reads	Type	pct.
GTGGGAACTTTGCCGAT|ATG**AGG**ATGG	18731	WT	52.76%
GTGGGAACTTTGCCGATtATGAGGATGG	12551	+1 t	35.35%
GTGGGAACTTTGCCG-TATGAGGATGG	526	-1	1.48%
GTGGGAACTTTGCCG--ATGAGGATGG	502	-2	1.41%
GTGGGAACTTTGCC-----GAGGATGG	231	-5	0.65%
GTGGGAACTTTGCC--TATGAGGATGG	166	-2	0.47%
GTGGGAACTTTGCC--------GATGG	136	-8	0.38%
GTGGGAACTT--------TGAGGATGG	101	-8	0.28%
GTGGGAACTTTGCCGATtATGGGGATGG	88	+1 t	0.25%
GTGGGAACTTTGCCGA-ATGAGGATGG	88	-1	0.25%

Total | INDEL	24h	Ins	Del	HDR
27948 | 73.72%		59.40%	14.32%	0.00%

Typical seqs	Reads	Type	pct.
GTGGGAACTTTGCCGAT|ATG**AGG**ATGG	7344	WT	26.28%
GTGGGAACTTTGCCGATtATGAGGATGG	14785	+1 t	52.90%
GTGGGAACTTTGCCG-TATGAGGATGG	704	-1	2.52%
GTGGGAACTTTGCCG--ATGAGGATGG	616	-2	2.20%
GTGGGAACTTTGCC-----GAGGATGG	369	-5	1.32%
GTGGGAACTTTGCC-------------	182	-13	0.65%
GTGGGAACTTTGCCGA-ATGAGGATGG	156	-1	0.56%
GTGGGAACTTTGCC--------GATGG	143	-8	0.51%
GTGGGAACTTTGCC--TATGAGGATGG	139	-2	0.50%
GTGGGAACTT--------TGAGGATGG	122	-8	0.44%

Total | INDEL	48h	Ins	Del	HDR
42028 | 75.99%		49.04%	26.95%	0.00%

Typical seqs	Reads	Type	pct.
GTGGGAACTTTGCCGAT|ATG**AGG**ATGG	10091	WT	24.01%
GTGGGAACTTTGCCGATtATGAGGATGG	17867	+1 t	42.51%
GTGGGAACTTTGCCG--ATGAGGATGG	1495	-2	3.56%
GTGGGAACTTTGCC-----GAGGATGG	1389	-5	3.30%
GTGGGAACTTTGCCG-TATGAGGATGG	1214	-1	2.89%
GTGGGAACTTTGCC--------GATGG	966	-8	2.30%
GTGGGAACTTTGCC-------------	652	-13	1.55%
GTGGGAACTT--------TGAGGATGG	388	-8	0.92%
GTGGGAACTTTGCCGA-ATGAGGATGG	252	-1	0.60%
G-----------------------TGG	222	-23	0.53%

P138-P142-YW-W9-Syn81crBCL11A3b-KO

YW-W9-KO-4h-Syn81crBCL11A3b (4h)	Ins	Del	HDR
28208 | 21.12%	3.47%	17.65%	0.00%
**Typical seqs**	**Reads**	**Type**	**pct.**
TGATAACGCCAGTAACCCGA**GGG**CCAG	22250	WT	78.88%
TGATAACGCCAGTAA-CCGAGGGCCAG	4237	-1	15.02%
TGATAACGCCAGTAACCcCGAGGGCCAG	669	+1 c	2.37%
TGATAACGCCAGTAA--CGAGGGCCAG	279	-2	0.99%
TGATAACGCCAGTAACCgCGAGGGCCAG	108	+1 g	0.38%
TGATAACGCCAGTAACCaCGAGGGCCAG	51	+1 a	0.18%
TGATAACGCCAGTAACCtCGAGGGCCAG	46	+1 t	0.16%
TGATAACGCCAGTA---CGAGGGCCAG	31	-3	0.11%
TGATAACGCCAGTAACCCCCGAGGGCCAG	27	+2	0.10%
TGATAACGCCA------CGAGGGCCAG	27	-6	0.10%

YW-W9-KO-8h-Syn81crBCL11A3b (8h)	Ins	Del	HDR
53403 | 55.23%	10.67%	44.56%	0.00%
**Typical seqs**	**Reads**	**Type**	**pct.**
TGATAACGCCAGTAACCCGA**GGG**CCAG	23909	WT	44.77%
TGATAACGCCAGTAA-CCGAGGGCCAG	17267	-1	32.33%
TGATAACGCCAGTAACCcCGAGGGCCAG	3890	+1 c	7.28%
TGATAACGCCAGTAA--CGAGGGCCAG	2889	-2	5.41%
TGATAACGCCAGTA---CGAGGGCCAG	384	-3	0.72%
TGATAACGCCAGTAACCgCGAGGGCCAG	356	+1 g	0.67%
TGATAACGCCAGTAACCaCGAGGGCCAG	229	+1 a	0.43%
TGATAACGCCAGTAACCtCGAGGGCCAG	228	+1 t	0.43%
TGATAACGCCAGTA--CCGAGGGCCAG	198	-2	0.37%
TGATAACGCCAGTAACCCCCGAGGGCCAG	195	+2	0.37%

YW-W9-KO-12h-Syn81crBCL11A3b (12h)	Ins	Del	HDR
28629 | 74.49%	15.40%	59.09%	0.00%
**Typical seqs**	**Reads**	**Type**	**pct.**
TGATAACGCCAGTAACCCGA**GGG**CCAG	7302	WT	25.51%
TGATAACGCCAGTAA-CCGAGGGCCAG	11964	-1	41.79%
TGATAACGCCAGTAACCcCGAGGGCCAG	2979	+1 c	10.41%
TGATAACGCCAGTAA--CGAGGGCCAG	1926	-2	6.73%
TGATAACGCCAGTAACCaCGAGGGCCAG	285	+1 a	1.00%
TGATAACGCCAGTAACCgCGAGGGCCAG	257	+1 g	0.90%
TGATAACGCCAGTA---CGAGGGCCAG	249	-3	0.87%
TGATAAC---------------GCCAG	190	-15	0.66%
TGATAACGCCAGTAACCCCCGAGGGCCAG	157	+2	0.55%
TGATAACGCCAGTAACCtCGAGGGCCAG	146	+1 t	0.51%

YW-W9-KO-24h-Syn81crBCL11A3b (24h)	Ins	Del	HDR
8526 | 78.08%	12.32%	65.76%	0.00%
**Typical seqs**	**Reads**	**Type**	**pct.**
TGATAACGCCAGTAACCCGA**GGG**CCAG	1869	WT	21.92%
TGATAACGCCAGTAA-CCGAGGGCCAG	3738	-1	43.84%
TGATAACGCCAGTAACCcCGAGGGCCAG	657	+1 c	7.71%
TGATAACGCCAGTAA--CGAGGGCCAG	522	-2	6.12%
TGATAACGCCAGTA---CGAGGGCCAG	102	-3	1.20%
TGATAACGCCAGTAACCaCGAGGGCCAG	103	+1 a	1.21%
TGATAAC---------------GCCAG	82	-15	0.96%
TGATAA-----------CGAGGGCCAG	78	-11	0.91%
TGATAACGCCAGTAACCgCGAGGGCCAG	75	+1 g	0.88%
TGATAACGCCAGTAACCtCGAGGGCCAG	53	+1 t	0.62%

YW-W9-KO-48h-Syn81crBCL11A3b (48h)	Ins	Del	HDR
26232 | 76.63%	10.20%	66.43%	0.00%
**Typical seqs**	**Reads**	**Type**	**pct.**
TGATAACGCCAGTAACCCGA**GGG**CCAG	6130	WT	23.37%
TGATAACGCCAGTAA-CCGAGGGCCAG	10639	-1	40.56%
TGATAACGCCAGTAACCcCGAGGGCCAG	1732	+1 c	6.60%
TGATAACGCCAGTAA--CGAGGGCCAG	1624	-2	6.19%
TGATAAC---------------GCCAG	861	-15	3.28%
TGATAA-----------CGAGGGCCAG	352	-11	1.34%
TGATAACGCCAGTAACCaCGAGGGCCAG	192	+1 a	0.73%
TGATAACGCCAGTA---CGAGGGCCAG	161	-3	0.61%
TGATAACGCCAGTAA-ACGAGGGCCAG	129	-1	0.49%
TGATAACGCCAGTAACCgCGAGGGCCAG	140	+1 g	0.53%

P138-P142-YW-W9-Syn82crBCL11A4a-KO

YW-W9-KO-4h-Syn82crBCL11A4a 4h	Ins	Del	HDR
42201 | 5.37%	4.92%	0.45%	0.00%
**Typical seqs**	**Reads**	**Type**	**pct.**
TCGTAACACACCGTCATCCG**GGG**AAGT	39935	WT	94.63%
TCGTAACACACCGTCATtCCGGGGAAGT	1875	+1 t	4.44%
TCGTAACACACCGTC-TCCGGGGAAGT	53	-1	0.13%
TCGTAACACACCGTCATATCCGGGGAAGT	50	+2	0.12%
TCGTAACACA-------CCGGGGAAGT	34	-7	0.08%
TCGTAACACACCGTCA-CCGGGGAAGT	29	-1	0.07%
TCGTAACACACCGTCATCC-GGGAAGT	24	-1	0.06%
TCGTAACACACCGTCA--CGGGGAAGT	13	-2	0.03%
TCGTAACACACCG---TCCGGGGAAGT	13	-3	0.03%
TCGTAACACGCCGTCATTCCGGGGAAGT	12	+1 Ins	0.03%

YW-W9-KO-8h-Syn82crBCL11A4a 8h	Ins	Del	HDR
98262 | 32.38%	29.22%	3.17%	0.00%
**Typical seqs**	**Reads**	**Type**	**pct.**
TCGTAACACACCGTCATCCG**GGG**AAGT	66441	WT	67.62%
TCGTAACACACCGTCATtCCGGGGAAGT	25382	+1 t	25.83%
TCGTAACACA-------CCGGGGAAGT	1108	-7	1.13%
TCGTAACACACCGTC-TCCGGGGAAGT	691	-1	0.70%
TCGTAACACACCGTCATATCCGGGGAAGT	415	+2	0.42%
TCGTAACACACCG---TCCGGGGAAGT	271	-3	0.28%
TCGTAACACACCGTCAT-CGGGGAAGT	156	-1	0.16%
TCGTAACACACCGTCA-CCGGGGAAGT	141	-1	0.14%
TCGTAACACGCCGTCATTCCGGGGAAGT	140	+1 Ins	0.14%
TCGTAACGCACCGTCATTCCGGGGAAGT	143	+1 Ins	0.15%

YW-W9-KO-12h-Syn82crBCL11A4a 12h	Ins	Del	HDR
17888 | 42.88%	36.93%	5.95%	0.00%
**Typical seqs**	**Reads**	**Type**	**pct.**
TCGTAACACACCGTCATCCG**GGG**AAGT	10218	WT	57.12%
TCGTAACACACCGTCATtCCGGGGAAGT	5846	+1 t	32.68%
TCGTAACACA-------CCGGGGAAGT	357	-7	2.00%
TCGTAACACACCGTC-TCCGGGGAAGT	190	-1	1.06%
TCGTAACACACCGTCATATCCGGGGAAGT	105	+2	0.59%
TCGTAACACACCG---TCCGGGGAAGT	92	-3	0.51%
TCGTAACACACCGTCA-CCGGGGAAGT	71	-1	0.40%
TCGTAACACACCGTCAT-CGGGGAAGT	55	-1	0.31%
TCGTAACACACCGTCATcCCGGGGAAGT	54	+1 c	0.30%
TCGTAACACACCGTCATtCCGGGGAGGT	44	+1 t	0.25%

YW-W9-KO-24h-Syn82crBCL11A4a 24h	Ins	Del	HDR
25436 | 59.41%	47.67%	11.74%	0.00%
**Typical seqs**	**Reads**	**Type**	**pct.**
TCGTAACACACCGTCATCCG**GGG**AAGT	10325	WT	40.59%
TCGTAACACACCGTCATtCCGGGGAAGT	10271	+1 t	40.38%
TCGTAACACA-------CCGGGGAAGT	908	-7	3.57%
TCGTAACACACCGTC-TCCGGGGAAGT	308	-1	1.21%
TCGTAACACACCG---TCCGGGGAAGT	175	-3	0.69%
TCGTAACACACCGTCATATCCGGGGAAGT	171	+2	0.67%
TCGTAACACACCGTCA-CCGGGGAAGT	135	-1	0.53%
TCGTAACACACCGCCATTCCGGGGAAGT	103	+1 Ins	0.40%
TCGTAACACACCGTCATcCCGGGGAAGT	100	+1 c	0.39%
TCGTAACACACCGTCAT-----GAAGT	83	-5	0.33%

YW-W9-KO-48h-Syn82crBCL11A4a 48h	Ins	Del	HDR
38435 | 61.65%	47.65%	14.00%	0.00%
**Typical seqs**	**Reads**	**Type**	**pct.**
TCGTAACACACCGTCATCCG**GGG**AAGT	14741	WT	38.35%
TCGTAACACACCGTCATtCCGGGGAAGT	15593	+1 t	40.57%
TCGTAACACA-------CCGGGGAAGT	2000	-7	5.20%
TCGTAACACACCGTC-TCCGGGGAAGT	455	-1	1.18%
TCGTAACACACCG---TCCGGGGAAGT	320	-3	0.83%
TCGTAACACACCGTCATATCCGGGGAAGT	298	+2	0.78%
TCGTAACACACCGTCAT-CGGGGAAGT	168	-1	0.44%
TCGTAACACACCGTCAT-----GAAGT	143	-5	0.37%
TCGTAACACACCGTCA-CCGGGGAAGT	150	-1	0.39%
TCGTAACACACCGTCATcCCGGGGAAGT	142	+1 c	0.37%

P138-P142-YW-W9-Syn83crBCL11A4b-KO

YW-W9-KO-4h-Syn83crBCL11A4b 4h<br>78761 | 3.35%	Ins<br>0.86%	Del<br>2.49%	HDR<br>0.00%
**Typical seqs**	**Reads**	**Type**	**pct.**
ACTTCATGCGGAGGCCC|CGT**GGG**AGGA	76121	WT	96.65%
ACTTCATGCGGAGG-CCCGTGGGAGGA	1525	-1	1.94%
ACTTCATGCGGAGGCCCcCGTGGGAGGA	260	+1 c	0.33%
ACTTCATGCGGAGGCCCCGT-GGAGGA	104	-1	0.13%
ACTTCATGCGGAGGCCCtCGTGGGAGGA	93	+1 t	0.12%
ACTTCATGCGGAGG--CCGTGGGAGGA	84	-2	0.11%
ACTTCATGCGGAGGCCCgCGTGGGAGGA	68	+1 g	0.09%
ACTTCATGCGGAGGCCCaCGTGGGAGGA	68	+1 a	0.09%
ACTTCATGCGGAGG---CGTGGGAGGA	48	-3	0.06%
ACTTCATGCGGAGGCCCGACGTGGGAGGA	35	+2	0.04%

YW-W9-KO-8h-Syn83crBCL11A4b 8h<br>118771 | 19.09%	Ins<br>3.00%	Del<br>16.10%	HDR<br>0.00%
**Typical seqs**	**Reads**	**Type**	**pct.**
ACTTCATGCGGAGGCCC|CGT**GGG**AGGA	96096	WT	80.91%
ACTTCATGCGGAGG-CCCGTGGGAGGA	11630	-1	9.79%
ACTTCATGCGGAGGCCCcCGTGGGAGGA	1410	+1 c	1.19%
ACTTCATGCGGAGG--CCGTGGGAGGA	1248	-2	1.05%
ACTTCATGCGGAGG---CGTGGGAGGA	849	-3	0.71%
ACTTCATGCGGAGGCCCtCGTGGGAGGA	657	+1 t	0.55%
ACTTCATG---------CGTGGGAGGA	647	-9	0.54%
ACTTCATGC------------GGAGGA	519	-12	0.44%
ACTTCATGCGGAGGCCC--TGGGAGGA	409	-2	0.34%
ACTTCATGCGGAGGCCCgCGTGGGAGGA	281	+1 g	0.24%

YW-W9-KO-12h-Syn83crBCL11A4b 12h<br>62948 | 39.37%	Ins<br>6.95%	Del<br>32.42%	HDR<br>0.00%
**Typical seqs**	**Reads**	**Type**	**pct.**
ACTTCATGCGGAGGCCC|CGT**GGG**AGGA	38167	WT	60.63%
ACTTCATGCGGAGG-CCCGTGGGAGGA	9664	-1	15.35%
ACTTCATGCGGAGGCCCcCGTGGGAGGA	2003	+1 c	3.18%
ACTTCATG---------CGTGGGAGGA	1559	-9	2.48%
ACTTCATGCGGAGG--CCGTGGGAGGA	1231	-2	1.96%
ACTTCATGC------------GGAGGA	832	-12	1.32%
ACTTCATGCGGAGG---CGTGGGAGGA	656	-3	1.04%
ACTTCATGCGGAGGCCCaCGTGGGAGGA	612	+1 a	0.97%
ACTTCATGCGGAGGCCCtCGTGGGAGGA	488	+1 t	0.78%
ACTTCATGCGGAGGCCCgCGTGGGAGGA	393	+1 g	0.62%

YW-W9-KO-24h-Syn83crBCL11A4b 24h<br>44848 | 55.20%	Ins<br>7.58%	Del<br>47.62%	HDR<br>0.00%
**Typical seqs**	**Reads**	**Type**	**pct.**
ACTTCATGCGGAGGCCC|CGT**GGG**AGGA	20092	WT	44.80%
ACTTCATGCGGAGG-CCCGTGGGAGGA	8987	-1	20.04%
ACTTCATGCGGAGGCCCcCGTGGGAGGA	1423	+1 c	3.17%
ACTTCATGCGGAGG--CCGTGGGAGGA	1337	-2	2.98%
ACTTCATG---------CGTGGGAGGA	1211	-9	2.70%
ACTTCATGC------------GGAGGA	1152	-12	2.57%
ACTTCATGCGGAGG---CGTGGGAGGA	819	-3	1.83%
ACTT-------------CGTGGGAGGA	442	-13	0.99%
ACTTCATGCGGAGGCCC-----GAGGA	419	-5	0.93%
ACTTCATGCGGAGGCCCgCGTGGGAGGA	333	+1 g	0.74%

YW-W9-KO-48h-Syn83crBCL11A4b 48h<br>63238 | 66.82%	Ins<br>7.70%	Del<br>59.12%	HDR<br>0.00%
**Typical seqs**	**Reads**	**Type**	**pct.**
ACTTCATGCGGAGGCCC|CGT**GGG**AGGA	20982	WT	33.18%
ACTTCATGCGGAGG-CCCGTGGGAGGA	13931	-1	22.03%
ACTTCATGC------------GGAGGA	3260	-12	5.16%
ACTTCATG---------CGTGGGAGGA	2524	-9	3.99%
ACTTCATGCGGAGG--CCGTGGGAGGA	2074	-2	3.28%
ACTTCATGCGGAGGCCCcCGTGGGAGGA	1813	+1 c	2.87%
ACTTCATGCGGAGG---CGTGGGAGGA	1452	-3	2.30%
ACTTCAT-----------GTGGGAGGA	747	-11	1.18%
ACTT-------------CGTGGGAGGA	664	-13	1.05%
ACTTCATGCGGAGGCCC-----GAGGA	610	-5	0.96%

P138-P142-YW-W9-Syn84crBCL11A4c-KO

YW-W9-KO-4h-Syn84crBCL11A4c 4h<br>11563 | 25.48%	Ins<br>1.76%	Del<br>23.71%	HDR<br>0.00%
**Typical seqs**	**Reads**	**Type**	**pct.**
GTAGGCGACCAACATGG|GGG**GGG**GGGG	8617	WT	74.52%
GTAGGCGACCAACAT--GGTGGGGGTG	1341	-2	11.60%
GTAGGCGACCAACAT-GGGTGGGGGGG	446	-1	3.86%
GTAGGCGACCAACAT-GGGTGGGGGTG	418	-1	3.61%
GTAGGCGACCAACATGGGG-GGGGGGG	81	-1	0.70%
GTAGGCGACCAACAT--GGTGGGGGGG	52	-2	0.45%
GTAGGCGACCAACATGGcGGTGGGGGTG	48	+1 c	0.42%
GTAGGCGACCAACATGGGGGGGGGGT-	43	-1	0.37%
GTAGGCGACCAACATGGtGGTGGGGGTG	42	+1 t	0.36%
GTAGGCGACCAAC----GGTGGGGGTG	41	-4	0.35%

YW-W9-KO-8h-Syn84crBCL11A4c 8h<br>33426 | 57.86%	Ins<br>2.53%	Del<br>55.32%	HDR<br>0.00%
**Typical seqs**	**Reads**	**Type**	**pct.**
GTAGGCGACCAACATGG|GGG**GGG**GGGG	14087	WT	42.14%
GTAGGCGACCAACAT--GGTGGGGGTG	6227	-2	18.63%
GTAGGCGACCAACAT-GGGTGGGGGGG	3240	-1	9.69%
GTAGGCGACCAACAT-GGGTGGGGGTG	3266	-1	9.77%
GTAGGCGACCAAC----GGTGGGGGTG	488	-4	1.46%
GTAGGCGACCAACA-----TGGGGGTG	333	-5	1.00%
GTAGGCGACCAACAT--GGTGGGGGGG	301	-2	0.90%
GTAGGCGACCAACATGGGG-GGGGGGG	252	-1	0.75%
GTAGGCGACCAACATGGtGGTGGGGGTG	213	+1 t	0.64%
GTAGGCGACCAACAT--------GGTG	159	-8	0.48%

YW-W9-KO-12h-Syn84crBCL11A4c 12h<br>31001 | 68.18%	Ins<br>2.90%	Del<br>65.28%	HDR<br>0.00%
**Typical seqs**	**Reads**	**Type**	**pct.**
GTAGGCGACCAACATGG|GGG**GGG**GGTG	9865	WT	31.82%
GTAGGCGACCAACAT--GGTGGGGGTG	5850	-2	18.87%
GTAGGCGACCAACAT-GGGTGGGGGTG	4910	-1	15.84%
GTAGGCGACCAACAT-GGGTGGGGGGG	1995	-1	6.44%
GTAGGCGACCAAC----GGTGGGGGTG	703	-4	2.27%
GTAGGCGACCAACA-----------TG	300	-11	0.97%
GTAGGCGACCAACA-----TGGGGGTG	289	-5	0.93%
GTA--------------GGTGGGGGTG	252	-14	0.81%
GTAGGCGACCA------GGTGGGGGTG	258	-6	0.83%
GTAGGCGACCAA-----GGTGGGGGTG	219	-5	0.71%

YW-W9-KO-24h-Syn84crBCL11A4c 24h<br>21463 | 79.63%	Ins<br>3.90%	Del<br>75.73%	HDR<br>0.00%
**Typical seqs**	**Reads**	**Type**	**pct.**
GTAGGCGACCAACATGG|GGG**GGG**GGGG	4372	WT	20.37%
GTAGGCGACCAACAT--GGTGGGGGTG	5513	-2	25.69%
GTAGGCGACCAACAT-GGGTGGGGGTG	1585	-1	7.38%
GTAGGCGACCAACAT-GGGTGGGGGGG	1481	-1	6.90%
GTAGGCGACCAAC----GGTGGGGGTG	468	-4	2.18%
GTAGGCGACCAACA-----TGGGGGTG	456	-5	2.12%
GTAGGCGACCAACATGGtGGTGGGGGTG	275	+1 t	1.28%
GTAGGCGACCAACA-----------TG	239	-11	1.11%
GTAGGCGACCAACAT--GGTGGGGGGG	241	-2	1.12%
GTA--------------GGTGGGGGTG	212	-14	0.99%

YW-W9-KO-48h-Syn84crBCL11A4c 48h<br>36677 | 89.15%	Ins<br>2.66%	Del<br>86.49%	HDR<br>0.00%
**Typical seqs**	**Reads**	**Type**	**pct.**
GTAGGCGACCAACATGG|GGG**GGG**GGGG	3978	WT	10.85%
GTAGGCGACCAACAT--GGTGGGGGTG	9711	-2	26.48%
GTAGGCGACCAACAT-GGGTGGGGGGG	2114	-1	5.76%
GTAGGCGACCAACAT-GGGTGGGGGTG	2230	-1	6.08%
GTAGGCGACCAACA-----TGGGGGTG	1623	-5	4.43%
GTAGGCGACCAACA-----------TG	1202	-11	3.28%
GTAGGCGACCAAC----GGTGGGGGTG	911	-4	2.48%
GTA--------------GGTGGGGGTG	692	-14	1.89%
GTAGGCGACCAACAT-------GGGTG	454	-7	1.24%
GTAGGCGACCAACAT------------	1014	-12	2.76%

P138-P142-YW-W9-Syn86crBCL11A5a-KO

YW-W9-KO-4h-Syn86crBCL11A5a 4h	Ins	Del	HDR
4323 | 4.83%	**3.91%**	**0.93%**	**0.00%**
**Typical seqs**	**Reads**	**Type**	**pct.**
TCCGAACAGCCACAATC|GGG**GGG**CTAC	4114	WT	95.17%
TCCGAACAGCCACAATCcGGGGGGCTAC	153	+1 c	3.54%
TCCGAACAGCCACAATC-GGGGGCTAC	21	-1	0.49%
TCCGAACAGCCACAATCTCGGGGGGCTAC	12	+2	0.28%
TCCGAACAGCCACAAT-GGGGGGCTAC	9	-1	0.21%
TCCGAACA---------GGGGGGCTAC	4	-9	0.09%
TCCGAACAGCCACAATCcTGGGGGCTAC	4	+1 c	0.09%
TCCGAACAGCCACAAT--TGTGGCTAC	3	-2	0.07%
TCCGAACAGCCA----CGGGGGGCTAC	3	-4	0.07%
YW-W9-KO-4h-Syn86crBCL11A5a	Ins	Del	HDR

YW-W9-KO-8h-Syn86crBCL11A5a 8h	Ins	Del	HDR
57062 | 14.16%	**9.14%**	**5.02%**	**0.00%**
**Typical seqs**	**Reads**	**Type**	**pct.**
TCCGAACAGCCACAATC|GGG**GGG**CTAC	48984	WT	85.84%
TCCGAACAGCCACAATCcGGGGGGCTAC	3865	+1 c	6.77%
TCCGAACAGCCACAATC-GGGGGCTAC	749	-1	1.31%
TCCGAACAGCCACAAT-GGGGGGCTAC	224	-1	0.39%
TCCGAACAGCCACAATCTCGGGGGGCTAC	225	+2	0.39%
TCCGAACAGCCACAATCCGGGGGGGCTAC	148	+2	0.26%
TCCGAACAGCCACAA------------	170	-12	0.30%
TCCGAACAGCCACAA--GGGGGGCTAC	104	-2	0.18%
TCCGAACAGCCA----CGGGGGGCTAC	98	-4	0.17%
TCCGAACAGCCACAATCtGGGGGGCTAC	96	+1 t	0.17%

YW-W9-KO-12h-Syn86crBCL11A5a 12h	Ins	Del	HDR
25467 | 18.34%	**12.15%**	**6.19%**	**0.00%**
**Typical seqs**	**Reads**	**Type**	**pct.**
TCCGAACAGCCACAATC|GGG**GGG**CTAC	20797	WT	81.66%
TCCGAACAGCCACAATCcGGGGGGCTAC	2253	+1 c	8.85%
TCCGAACAGCCACAATC-GGGGGCTAC	363	-1	1.43%
TCCGAACAGCCACAATCTCGGGGGGCTAC	157	+2	0.62%
TCCGAACAGCCACAATCCGGGGGGGCTAC	149	+2	0.59%
TCCGAACAGCCACAAT-GGGGGGCTAC	145	-1	0.57%
TCCGAACAGCCACAATCtGGGGGGCTAC	78	+1 t	0.31%
TCCGAACAGCCACAAT-------CTAC	76	-7	0.30%
TCCGAACA--------------GCTAC	61	-14	0.24%
TCCGAACAGCCACAA------------	103	-12	0.40%

YW-W9-KO-24h-Syn86crBCL11A5a 24h	Ins	Del	HDR
14557 | 23.55%	**9.85%**	**13.70%**	**0.00%**
**Typical seqs**	**Reads**	**Type**	**pct.**
TCCGAACAGCCACAATC|GGG**GGG**CTAC	11129	WT	76.45%
TCCGAACAGCCACAATCcGGGGGGCTAC	778	+1 c	5.34%
TCCGAACAGCCACAATC-GGGGGCTAC	246	-1	1.69%
TCCGAACAGCCACAAT-GGGGGGCTAC	186	-1	1.28%
TCCGAACAGCCACAATCCGGGGGGGCTAC	175	+2	1.20%
TCCGAACAGCCACAAT----------C	149	-10	1.02%
TCCGAA-----------------CTAC	94	-17	0.65%
TCCGAA----------CGGGGGGCTAC	85	-10	0.58%
TCCGAACAGCCACAA--GGGGGGCTAC	78	-2	0.54%
TCCGAACAGCCACAATC---GGGCTAC	75	-3	0.52%

YW-W9-KO-48h-Syn86crBCL11A5a 48h	Ins	Del	HDR
10576 | 32.17%	**14.79%**	**17.38%**	**0.00%**
**Typical seqs**	**Reads**	**Type**	**pct.**
TCCGAACAGCCACAATC|GGG**GGG**CTAC	7174	WT	67.83%
TCCGAACAGCCACAATCcGGGGGGCTAC	1038	+1 c	9.81%
TCCGAACAGCCACAATC-GGGGGCTAC	175	-1	1.65%
TCCGAACAGCCACAAT-GGGGGGCTAC	118	-1	1.12%
TCCGAACAGCCACAA------------	177	-12	1.67%
TCCGAACAGCCACAATCCGGGGGGGCTAC	96	+2	0.91%
TCCGAACAGCCACAAT----------C	90	-10	0.85%
TCCGAACAGCC--------------AC	78	-14	0.74%
TCCGAACAGCCACAATCTCGGGGGGCTAC	74	+2	0.70%
TCCGAACAGCCACAA------------	177	-14	1.67%

P138-P142-YW-W9-Syn87crBCL11A5b-KO

YW-W9-KO-4h-Syn87crBCL11A5b **4h**

58511 | 25.03%

Ins	Del	HDR
8.12%	16.91%	0.00%

Typical seqs	Reads	Type	pct.
GGGATTACCGAGTCACC|ACC**AGG**CTGC	43863	WT	74.97%
GGGATTACCGAGT---CACCAGGCTGC	5112	-3	8.74%
GGGATTACCGAGTCACCcACCAGGCTGC	4062	+1 c	6.94%
GGGATTACCGAGTCA-CACCAGGCTGC	3596	-1	6.15%
GGGATT-----------ACCAGGCTGC	232	-11	0.40%
GGGATTACCGAGTCACCCCACCAGGCTGC	201	+2	0.34%
GGGATTACCGAGTCA--ACCAGGCTGC	69	-2	0.12%
GGGATTACCGAGTCACCgACCAGGCTGC	52	+1 g	0.09%
GGGATTACCGAGTCACC-CCAGGCTGC	47	-1	0.08%
GGGATTACCGAGTCACCtACCAGGCTGC	44	+1 t	0.08%

YW-W9-KO-8h-Syn87crBCL11A5b **8h**

59857 | 42.94%

Ins	Del	HDR
11.54%	31.40%	0.00%

Typical seqs	Reads	Type	pct.
GGGATTACCGAGTCACC|ACC**AGG**CTGC	34152	WT	57.06%
GGGATTACCGAGT---CACCAGGCTGC	10042	-3	16.78%
GGGATTACCGAGTCA-CACCAGGCTGC	5917	-1	9.89%
GGGATTACCGAGTCACCcACCAGGCTGC	5760	+1 c	9.62%
GGGATT-----------ACCAGGCTGC	587	-11	0.98%
GGGATTACCGAGTCACCCCACCAGGCTGC	282	+2	0.47%
GGGATTACCGAGTCACCtACCAGGCTGC	190	+1 t	0.32%
GGGATTACCGAGTCAC--CCAGGCTGC	176	-2	0.29%
GGGATTACCGAGTCACC-CCAGGCTGC	109	-1	0.18%
GGGATTACCGAGTCA--ACCAGGCTGC	104	-2	0.17%

YW-W9-KO-12h-Syn87crBCL11A5b **12h**

54077 | 54.54%

Ins	Del	HDR
13.29%	41.25%	0.00%

Typical seqs	Reads	Type	pct.
GGGATTACCGAGTCACC|ACC**AGG**CTGC	24583	WT	45.46%
GGGATTACCGAGT---CACCAGGCTGC	11384	-3	21.05%
GGGATTACCGAGTCA-CACCAGGCTGC	7286	-1	13.47%
GGGATTACCGAGTCACCcACCAGGCTGC	5991	+1 c	11.08%
GGGATT-----------ACCAGGCTGC	816	-11	1.51%
GGGATTACCGAGTCACCCCACCAGGCTGC	339	+2	0.63%
GGGATTACCGAGTCACCtACCAGGCTGC	223	+1 t	0.41%
GGGATTACCGAGTCA--ACCAGGCTGC	159	-2	0.29%
GGGATTACCGAGTCACC-CCAGGCTGC	131	-1	0.24%
GGGATTACCGAGTCAC--CCAGGCTGC	136	-2	0.25%

YW-W9-KO-24h-Syn87crBCL11A5b **24h**

33689 | 63.07%

Ins	Del	HDR
12.44%	50.63%	0.00%

Typical seqs	Reads	Type	pct.
GGGATTACCGAGTCACC|ACC**AGG**CTGC	12441	WT	36.93%
GGGATTACCGAGT---CACCAGGCTGC	8984	-3	26.67%
GGGATTACCGAGTCA-CACCAGGCTGC	4952	-1	14.70%
GGGATTACCGAGTCACCcACCAGGCTGC	3444	+1 c	10.22%
GGGATT-----------ACCAGGCTGC	609	-11	1.81%
GGGATTACCGAGTCACCtACCAGGCTGC	136	+1 t	0.40%
GGGATTACCGAGTCACCCCACCAGGCTGC	115	+2	0.34%
GGGATTACCGAGTCACC-CCAGGCTGC	102	-1	0.30%
GGGATTACCGAGTCAC--CCAGGCTGC	102	-2	0.30%
GGGATTACCGAGT------CAGGCTGC	93	-6	0.28%

YW-W9-KO-48h-Syn87crBCL11A5b **48h**

11952 | 76.89%

Ins	Del	HDR
12.88%	64.01%	0.00%

Typical seqs	Reads	Type	pct.
GGGATTACCGAGTCACC|ACC**AGG**CTGC	2762	WT	23.11%
GGGATTACCGAGT---CACCAGGCTGC	4091	-3	34.23%
GGGATTACCGAGTCA-CACCAGGCTGC	1945	-1	16.27%
GGGATTACCGAGTCACCcACCAGGCTGC	1293	+1 c	10.82%
GGGATT-----------ACCAGGCTGC	477	-11	3.99%
GGGATTACCGAGTCACCtACCAGGCTGC	67	+1 t	0.56%
GGGATTACCGAGT------CAGGCTGC	56	-6	0.47%
GGGATTACCGAGTCACCCCACCAGGCTGC	50	+2	0.42%
GGGATTACCGAGT--CCACCAGGCTGC	49	-2	0.41%
GGGATTACCGAGTCACC-CCAGGCTGC	42	-1	0.35%

P138-P142-YW-W9-Syn88crBCL11A6a-KO

YW-W9-KO-4h-Syn88crBCL11A6a 4h<br>32579 | 3.73%	Ins<br>2.81%	Del<br>0.92%	HDR<br>0.00%
**Typical seqs**	**Reads**	**Type**	**pct.**
TCCTCGGCCAATGAAGC|GGT**GGG**TCCA	31365	WT	96.27%
TCCTCGGCCAATGAAGCcGGTGGGTCCA	720	+1 c	2.21%
TCCTCGGCCAATGAAGCaGGTGGGTCCA	60	+1 a	0.18%
TCCTCGGCCAATGAA--GGTGGGTCCA	57	-2	0.17%
TCCTCGGCCAATGAAG-GGTGGGTCCA	50	-1	0.15%
TCCTCGGCCAATGAAGCCGAGGTGGGTCCA	38	+3	0.12%
TCCTCGGCCAATGAAGCGGT-GGTCCA	37	-1	0.11%
TCCTCGGCCAATGAAGCtGGTGGGTCCA	21	+1 t	0.06%
TCCTCGGCCAATGA---GGTGGGTCCA	17	-3	0.05%
TCCTCGGCCAATGAAGC-GTGGGTCCA	13	-1	0.04%

YW-W9-KO-8h-Syn88crBCL11A6a 8h<br>26061 | 8.71%	Ins<br>5.93%	Del<br>2.78%	HDR<br>0.00%
**Typical seqs**	**Reads**	**Type**	**pct.**
TCCTCGGCCAATGAAGC|GGT**GGG**TCCA	23791	WT	91.29%
TCCTCGGCCAATGAAGCcGGTGGGTCCA	1121	+1 c	4.30%
TCCTCGGCCAATGAA--GGTGGGTCCA	105	-2	0.40%
TCCTCGGCCAATGAAGCaGGTGGGTCCA	105	+1 a	0.40%
TCCTCGGCCAATGAAGCtGGTGGGTCCA	69	+1 t	0.26%
TCCTCGGCCAATGAAGCgGGTGGGTCCA	65	+1 g	0.25%
TCCTCGGCCAATGAAGCCGAGGTGGGTCCA	64	+3	0.25%
TCCTCGGCCAATGAAG-GGTGGGTCCA	50	-1	0.19%
TCCTCGGCCAATGAAGC-GTGGGTCCA	45	-1	0.17%
TCCTCGGCCAATGAA------------	37	-13	0.14%

YW-W9-KO-12h-Syn88crBCL11A6a 12h<br>4141 | 12.99%	Ins<br>8.72%	Del<br>4.27%	HDR<br>0.00%
**Typical seqs**	**Reads**	**Type**	**pct.**
TCCTCGGCCAATGAAGC|GGT**GGG**TCCA	3603	WT	87.01%
TCCTCGGCCAATGAAGCcGGTGGGTCCA	277	+1 c	6.69%
TCCTCGGCCAATGAA--GGTGGGTCCA	26	-2	0.63%
TCCTCGGCCAATGAAGCtGGTGGGTCCA	21	+1 t	0.51%
TCCTCGGCCAATGAAGCaGGTGGGTCCA	17	+1 a	0.41%
TCCTCGGCCAATGAAG-GGTGGGTCCA	15	-1	0.36%
TCCTCGGCCAATGAAGCgGGTGGGTCCA	15	+1 g	0.36%
TCCTCGGCCAATGAAGCCGAGGTGGGTCCA	14	+3	0.34%
TCCTCGGCCA-------GGTGGGTCCA	11	-7	0.27%
TCCTCGGCCAA------GGTGGGTCCA	11	-6	0.27%

YW-W9-KO-24h-Syn88crBCL11A6a 24h<br>11243 | 19.26%	Ins<br>9.80%	Del<br>9.45%	HDR<br>0.00%
**Typical seqs**	**Reads**	**Type**	**pct.**
TCCTCGGCCAATGAAGC|GGT**GGG**TCCA	9078	WT	80.74%
TCCTCGGCCAATGAAGCcGGTGGGTCCA	760	+1 c	6.76%
TCCTCGGCCAATGAA--GGTGGGTCCA	103	-2	0.92%
TCCTCGGCCAATGAAGCaGGTGGGTCCA	77	+1 a	0.68%
TCCTCGGCCAATGAA------------	64	-13	0.57%
TCCTCGGCCAATGAAGCtGGTGGGTCCA	56	+1 t	0.50%
TCCTCGGCCAATGAAG-GGTGGGTCCA	55	-1	0.49%
TCCTCGGCCAATGAAGCCGAGGTGGGTCCA	51	+3	0.45%
TCCTCGGCCAATGAAGCgGGTGGGTCCA	50	+1 g	0.44%
TCCTCGGCCAATGA---GGTGGGTCCA	42	-3	0.37%

YW-W9-KO-48h-Syn88crBCL11A6a 48h<br>9324 | 28.20%	Ins<br>14.06%	Del<br>14.14%	HDR<br>0.00%
**Typical seqs**	**Reads**	**Type**	**pct.**
TCCTCGGCCAATGAAGC|GGT**GGG**TCCA	6695	WT	71.80%
TCCTCGGCCAATGAAGCcGGTGGGTCCA	889	+1 c	9.53%
TCCTCGGCCAATGAA--GGTGGGTCCA	124	-2	1.33%
TCCTCGGCCAATGAAGCCGAGGTGGGTCCA	91	+3	0.98%
TCCTCGGCCAATGAAGCaGGTGGGTCCA	73	+1 a	0.78%
TCCTCGGCCAATGAAG-GGTGGGTCCA	73	-1	0.78%
TCCTCGGCCAATGAAGCtGGTGGGTCCA	65	+1 t	0.70%
TCCTCGGCCAATGAA------------	60	-13	0.64%
TCCTCGGCCAAT-----GGTGGGTCCA	55	-5	0.59%
TCCTCGGCCAA--------TGGGTCCA	50	-8	0.54%

P138-P142-YW-W9-Syn89crBCL11A6b-KO

P138-YW-W9-KO-4h-Syn89crBCL11A6b 4h	Ins	Del	HDR
1354 | 4.06%	3.32%	0.74%	0.00%
**Typical seqs**	**Reads**	**Type**	**pct.**
CACCATTAACCGTGCCT|CTG**TGG**GCTG	1299	WT	95.94%
CACCATTAACCGTGCCTtCTGTGGGCTG	24	+1 t	1.77%
CACCATTAACCGTGCCTCTCTGTGGGCTG	21	+2	1.55%
CACCATTAACCGTGCC-CTGTGGGCTG	4	-1	0.30%
CACCATTAACCGTGC------GGGCTG	3	-6	0.22%
CACCATTAACCGTGC--CTGTGGGCTG	3	-2	0.22%

YW-W9-KO-8h-Syn89crBCL11A6b 8h	Ins	Del	HDR
28461 | 13.26%	9.44%	3.81%	0.00%
**Typical seqs**	**Reads**	**Type**	**pct.**
CACCATTAACCGTGCCT|CTG**TGG**GCTG	24688	WT	86.74%
CACCATTAACCGTGCCTtCTGTGGGCTG	1832	+1 t	6.44%
CACCATTAACCGTGCCTCTCTGTGGGCTG	557	+2	1.96%
CACCATTAACCGTGC--CTGTGGGCTG	255	-2	0.90%
CACCATTAACCGTGCC-CTGTGGGCTG	175	-1	0.61%
CACCATTAACCGTG---CTGTGGGCTG	129	-3	0.45%
CACCATTAACC--------GTGGGCTG	78	-8	0.27%
CACCATTAACCGTG-CTCTGTGGGCTG	68	-1	0.24%
CACCATTAA--------CTGTGGGCTG	66	-8	0.23%
CACCATTAACCGTGCCCTCTGTGGGCTG	57	+1 Ins	0.20%

YW-W9-KO-12h-Syn89crBCL11A6b 12h	Ins	Del	HDR
3792 | 17.35%	11.74%	5.62%	0.00%
**Typical seqs**	**Reads**	**Type**	**pct.**
CACCATTAACCGTGCCT|CTG**TGG**GCTG	3134	WT	82.65%
CACCATTAACCGTGCCTtCTGTGGGCTG	309	+1 t	8.15%
CACCATTAACCGTGCCTCTCTGTGGGCTG	107	+2	2.82%
CACCATTAACCGTGC--CTGTGGGCTG	53	-2	1.40%
CACCATTAACCGTGCC-CTGTGGGCTG	34	-1	0.90%
CACCATTAA--------CTGTGGGCTG	21	-8	0.55%
CACCATTAACCGTG-CTCTGTGGGCTG	20	-1	0.53%
CACCATTAACCGTG---CTGTGGGCTG	17	-3	0.45%
CACCATTAACCGTGCCCTCTGTGGGCTG	17	+1 Ins	0.45%
CACCATTAACCGTGCCT-TGTGGGCTG	15	-1	0.40%

YW-W9-KO-24h-Syn89crBCL11A6b 24h	Ins	Del	HDR
8623 | 29.51%	18.20%	11.32%	0.00%
**Typical seqs**	**Reads**	**Type**	**pct.**
CACCATTAACCGTGCCT|CTG**TGG**GCTG	6078	WT	70.49%
CACCATTAACCGTGCCTtCTGTGGGCTG	1079	+1 t	12.51%
CACCATTAACCGTGCCTCTCTGTGGGCTG	278	+2	3.22%
CACCATTAACCGTGC--CTGTGGGCTG	210	-2	2.44%
CACCATTAACCGTGCC-CTGTGGGCTG	95	-1	1.10%
CACCATTAACC--------GTGGGCTG	86	-8	1.00%
CACCATTAA--------CTGTGGGCTG	88	-8	1.02%
CACCATTAACCGTG---CTGTGGGCTG	76	-3	0.88%
CACCATTAACCGTG-CTCTGTGGGCTG	71	-1	0.82%
CACCATTAACCGTGC---------CTG	46	-9	0.53%

YW-W9-KO-48h-Syn89crBCL11A6b 48h	Ins	Del	HDR
8348 | 46.01%	23.68%	22.33%	0.00%
**Typical seqs**	**Reads**	**Type**	**pct.**
CACCATTAACCGTGCCT|CTG**TGG**GCTG	4507	WT	53.99%
CACCATTAACCGTGCCTtCTGTGGGCTG	1341	+1 t	16.06%
CACCATTAACCGTGCCTCTCTGTGGGCTG	359	+2	4.30%
CACCATTAACC--------GTGGGCTG	343	-8	4.11%
CACCATTAACCGTGC--CTGTGGGCTG	289	-2	3.46%
CACCATTAA--------CTGTGGGCTG	226	-8	2.71%
CACCATTAACCGTG---CTGTGGGCTG	158	-3	1.89%
CACCATTAACCGT----------GCTG	93	-10	1.11%
CACCATTAACCGTGCC-CTGTGGGCTG	93	-1	1.11%
CACCATTAACCG------TGTGGGCTG	84	-6	1.01%

P138-P142-YW-W9-Syn90crBCL11A6c-KO

YW-W9-KO-4h-Syn90crBCL11A6c 4h<br>64637 | 4.98%	Ins<br>4.09%	Del<br>0.90%	HDR<br>0.00%
**Typical seqs**	**Reads**	**Type**	**pct.**
CTGCCTACCGGCACGCA|TGC**AGG**ATCA	61415	WT	95.02%
CTGCCTACCGGCACGCAaTGCAGGATCA	1527	+1 a	2.36%
CTGCCTACCGGCACGCAtTGCAGGATCA	778	+1 t	1.20%
CTGCCTACCGGCAC----GCAGGATCA	161	-4	0.25%
CTGCCTACCGGCACGCAgTGCAGGATCA	120	+1 g	0.19%
CTGCCTACCGGCACGC-TGCAGGATCA	109	-1	0.17%
CTGCCTACCGGCACGCA-GCAGGATCA	103	-1	0.16%
CTGCCTACCG--------GCAGGATCA	64	-8	0.10%
CTGCCTACCG----GCATGCAGGATCA	62	-4	0.10%
CTGCCTACCGGCACGCAcTGCAGGATCA	52	+1 c	0.08%

YW-W9-KO-8h-Syn90crBCL11A6c 8h<br>73391 | 11.96%	Ins<br>9.79%	Del<br>2.17%	HDR<br>0.00%
**Typical seqs**	**Reads**	**Type**	**pct.**
CTGCCTACCGGCACGCA|TGC**AGG**ATCA	64612	WT	88.04%
CTGCCTACCGGCACGCAaTGCAGGATCA	3756	+1 a	5.12%
CTGCCTACCGGCACGCAtTGCAGGATCA	2148	+1 t	2.93%
CTGCCTACCGGCAC----GCAGGATCA	447	-4	0.61%
CTGCCTACCGGCACGCAgTGCAGGATCA	357	+1 g	0.49%
CTGCCTACCGGCACGC-TGCAGGATCA	144	-1	0.20%
CTGCCTACCGGCACGCAcTGCAGGATCA	144	+1 c	0.20%
CTGCCTACCG--------GCAGGATCA	137	-8	0.19%
CTGCCTACCGGCACGCA-GCAGGATCA	126	-1	0.17%
CTGCCTACCG----GCATGCAGGATCA	119	-4	0.16%

YW-W9-KO-12h-Syn90crBCL11A6c 12h<br>8997 | 16.93%	Ins<br>14.14%	Del<br>2.79%	HDR<br>0.00%
**Typical seqs**	**Reads**	**Type**	**pct.**
CTGCCTACCGGCACGCA|TGC**AGG**ATCA	7474	WT	83.07%
CTGCCTACCGGCACGCAaTGCAGGATCA	682	+1 a	7.58%
CTGCCTACCGGCACGCAtTGCAGGATCA	438	+1 t	4.87%
CTGCCTACCGGCAC----GCAGGATCA	69	-4	0.77%
CTGCCTACCGGCACGCAgTGCAGGATCA	58	+1 g	0.64%
CTGCCTACCGGCACGCAcTGCAGGATCA	30	+1 c	0.33%
CTGCCTACCGGCACGCA-GCAGGATCA	27	-1	0.30%
CTGCCTACCGGCACGC-TGCAGGATCA	26	-1	0.29%
CTGCCTACCG--------GCAGGATCA	24	-8	0.27%
CTGCCTACCGGCACGCAAATGCAGGATCA	16	+2	0.18%

YW-W9-KO-24h-Syn90crBCL11A6c 24h<br>39911 | 25.77%	Ins<br>19.43%	Del<br>6.35%	HDR<br>0.00%
**Typical seqs**	**Reads**	**Type**	**pct.**
CTGCCTACCGGCACGCA|TGC**AGG**ATCA	29624	WT	74.23%
CTGCCTACCGGCACGCAaTGCAGGATCA	3957	+1 a	9.91%
CTGCCTACCGGCACGCAtTGCAGGATCA	2420	+1 t	6.06%
CTGCCTACCGGCAC----GCAGGATCA	731	-4	1.83%
CTGCCTACCGGCACGCAgTGCAGGATCA	356	+1 g	0.89%
CTGCCTACCGGCACGCA-GCAGGATCA	185	-1	0.46%
CTGCCTACCGGCACGC-TGCAGGATCA	181	-1	0.45%
CTGCCTACCG--------GCAGGATCA	174	-8	0.44%
CTGCCTACCG----GCATGCAGGATCA	128	-4	0.32%
CTGCCTACCGGCACGCAcTGCAGGATCA	113	+1 c	0.28%

YW-W9-KO-48h-Syn90crBCL11A6c 48h<br>24276 | 35.68%	Ins<br>25.14%	Del<br>10.54%	HDR<br>0.00%
**Typical seqs**	**Reads**	**Type**	**pct.**
CTGCCTACCGGCACGCA|TGC**AGG**ATCA	15615	WT	64.32%
CTGCCTACCGGCACGCAaTGCAGGATCA	3005	+1 a	12.38%
CTGCCTACCGGCACGCAtTGCAGGATCA	1956	+1 t	8.06%
CTGCCTACCGGCAC----GCAGGATCA	851	-4	3.51%
CTGCCTACCG--------GCAGGATCA	247	-8	1.02%
CTGCCTACCGGCACGCAgTGCAGGATCA	249	+1 g	1.03%
CTGCCTACCG----GCATGCAGGATCA	185	-4	0.76%
CTGCCTACCGGCACGCA-GCAGGATCA	154	-1	0.63%
CTGCCTACCGGCACGCAcTGCAGGATCA	129	+1 c	0.53%
CTGCCTACCGGCACGC-TGCAGGATCA	97	-1	0.40%

P138-P142-YW-W9-Syn91crBCL11A7a-KO

YW-W9-KO-4h-Syn91crBCL11A7a 4h	Ins	Del	HDR
95677 | 19.99%	19.00%	0.99%	0.00%
**Typical seqs**	**Reads**	**Type**	**pct.**
TGACACTTACCGCGTAT|GAG**GGG**TCTT	76549	WT	80.01%
TGACACTTACCGCGTATtGAGGGGTCTT	16164	+1 t	16.89%
TGACACTTACCGCGTATATGAGGGGTCTT	152	+2	0.16%
TGACGCTTACCGCGTATTGAGGGGTCTT	121	+1 Ins	0.13%
TGACACTTACCGC---------GTCTT	119	-9	0.12%
TGACACTTACCGCGTATtGAGGGGTCTC	106	+1 t	0.11%
TGGCACTTACCGCGTATTGAGGGGTCTT	104	+1 Ins	0.11%
CGACACTTACCGCGTATTGAGGGGTCTT	90	+1 Ins	0.09%
TGACACTTACCGCGT-TGAGGGGTCTT	90	-1	0.09%
TGACACTTACCGCG--TGAGGGGTCTT	89	-2	0.09%

YW-W9-KO-8h-Syn91crBCL11A7a 8h	Ins	Del	HDR
114081 | 35.40%	31.77%	3.63%	0.00%
**Typical seqs**	**Reads**	**Type**	**pct.**
TGACACTTACCGCGTAT|GAG**GGG**TCTT	73696	WT	64.60%
TGACACTTACCGCGTATtGAGGGGTCTT	32029	+1 t	28.08%
TGACACTTACCGC---------GTCTT	375	-9	0.33%
TGACACTTACCGCGTATATGAGGGGTCTT	336	+2	0.29%
TGACACTTACCGCGT-TGAGGGGTCTT	260	-1	0.23%
TGACGCTTACCGCGTATTGAGGGGTCTT	200	+1 Ins	0.18%
TGACACTTACCGCGTATTTGAGGGGTCTT	196	+2	0.17%
TGACACTTACCGCG--TGAGGGGTCTT	193	-2	0.17%
CGACACTTACCGCGTATTGAGGGGTCTT	180	+1 Ins	0.16%
TGGCACTTACCGCGTATTGAGGGGTCTT	183	+1 Ins	0.16%

YW-W9-KO-12h-Syn91crBCL11A7a 12h	Ins	Del	HDR
12439 | 45.17%	39.87%	5.30%	0.00%
**Typical seqs**	**Reads**	**Type**	**pct.**
TGACACTTACCGCGTAT|GAG**GGG**TCTT	6820	WT	54.83%
TGACACTTACCGCGTATtGAGGGGTCTT	4172	+1 t	33.54%
TGAGACTTACCGCGTATTGAGGGGTCTT	60	+1 Ins	0.48%
TGGCACTTACCGCGTATTGAGGGGTCTT	51	+1 Ins	0.41%
TGACACTTACCGC---------GTCTT	45	-9	0.36%
TGACGCTTACCGCGTATTGAGGGGTCTT	43	+1 Ins	0.35%
TGACACTTACCGCGTATATGAGGGGTCTT	36	+2	0.29%
TGACACTTACCGCGTA--AGGGGTCTT	40	-2	0.32%
TGACACTTACCGCGT-TGAGGGGTCTT	32	-1	0.26%
TTACACTTACCGCGTATTGAGGGGTCTT	31	+1 Ins	0.25%

YW-W9-KO-24h-Syn91crBCL11A7a 24h	Ins	Del	HDR
72394 | 48.38%	39.88%	8.51%	0.00%
**Typical seqs**	**Reads**	**Type**	**pct.**
TGACACTTACCGCGTAT|GAG**GGG**TCTT	37367	WT	51.62%
TGACACTTACCGCGTATtGAGGGGTCTT	24236	+1 t	33.48%
TGACACT-------TATGAGGGGTCTT	358	-7	0.49%
TGACACTTACCGC---------GTCTT	347	-9	0.48%
TGACGCTTACCGCGTATTGAGGGGTCTT	236	+1 Ins	0.33%
TGGCACTTACCGCGTATTGAGGGGTCTT	233	+1 Ins	0.32%
TGACACTTACCGCGTATATGAGGGGTCTT	227	+2	0.31%
CGACACTTACCGCGTATTGAGGGGTCTT	209	+1 Ins	0.29%
TGACACTTACCGCGTATtGAGGGGTCTC	220	+1 t	0.30%
TGACACTTACCGCGTATtGGGGGGTCTT	200	+1 t	0.28%

YW-W9-KO-48h-Syn91crBCL11A7a 48h	Ins	Del	HDR
50392 | 60.54%	47.01%	13.53%	0.00%
**Typical seqs**	**Reads**	**Type**	**pct.**
TGACACTTACCGCGTAT|GAG**GGG**TCTT	19887	WT	39.46%
TGACACTTACCGCGTATtGAGGGGTCTT	19837	+1 t	39.37%
TGACACTTACCGC---------GTCTT	476	-9	0.94%
TGACACT-------TATGAGGGGTCTT	325	-7	0.64%
TGACACTTACCGCGT---AAGTGTCTT	309	-3	0.61%
TGACACTTACCGCGTA--AGGGGTCTT	275	-2	0.55%
TGACACTTACCGCGTATATGAGGGGTCTT	196	+2	0.39%
CGACACTTACCGCGTATTGAGGGGTCTT	173	+1 Ins	0.34%
TGACACTTAC------TGAGGGGTCTT	181	-6	0.36%
TGACACTTACCGC----GAGGGGTCTT	165	-4	0.33%

P138-P142-YW-W9-Syn92crBCL11A7b-KO

YW-W9-KO-4h-Syn92crBCL11A7b-BC1 4h	Ins	Del	HDR
128812 | 20.50%	18.44%	2.06%	0.00%
**Typical seqs**	**Reads**	**Type**	**pct.**
GCTTCGTGACGTTGGAT|GGA**GGG**TTTT	102403	WT	79.50%
GCTTCGTGACGTTGGATtGGAGGGTTTT	21099	+1 t	16.38%
GCTTCGTGACGT----TGGAGGGTTTT	1489	-4	1.16%
GCTTCGTGACGTTGGA-GGAGGGTTTT	267	-1	0.21%
GCTTCGTGACGTTGGATATGGAGGGTTTT	231	+2	0.18%
GCTTCGTGACGTTGGAT-GAGGGTTTT	190	-1	0.15%
GCTTCGTGACGTTGGATtGGAGGGTTCT	138	+1 t	0.11%
GCTTCGCGACGTTGGATTGGAGGGTTTT	129	+1 Ins	0.10%
GCTTCGTGACGCTGGATTGGAGGGTTTT	114	+1 Ins	0.09%
GCTTCGTGACGTTGGATtGGAGGGCTTT	114	+1 t	0.09%

YW-W9-KO-8h-Syn92crBCL11A7b-BC1 8h	Ins	Del	HDR
138375 | 38.37%	30.45%	7.91%	0.00%
**Typical seqs**	**Reads**	**Type**	**pct.**
GCTTCGTGACGTTGGAT|GGA**GGG**TTTT	85282	WT	61.63%
GCTTCGTGACGTTGGATtGGAGGGTTTT	34065	+1 t	24.62%
GCTTCGTGACGT----TGGAGGGTTTT	4695	-4	3.39%
GCTTCGTGACGTTGGAATGGAGGGTTTT	3370	+1 Ins	2.44%
GCTTCGTGACGTTGGA-GGAGGGTTTT	2687	-1	1.94%
GCTTCGTGACGTTGGAT-GAGGGTTTT	465	-1	0.34%
GCTTCGTGACGTTGGATATGGAGGGTTTT	246	+2	0.18%
GCTTCGTGGCGTTGGATTGGAGGGTTTT	246	+1 Ins	0.18%
GCTTCGTGACGTTGGATtGGAGGGTTCT	234	+1 t	0.17%
GCTTCGTGACGTTGGATtGGAGGGCTTT	219	+1 t	0.16%

YW-W9-KO-12h-Syn92crBCL11A7b-BC1 12h	Ins	Del	HDR
72954 | 42.45%	32.24%	10.21%	0.00%
**Typical seqs**	**Reads**	**Type**	**pct.**
GCTTCGTGACGTTGGAT|GGA**GGG**TTTT	41985	WT	57.55%
GCTTCGTGACGTTGGATtGGAGGGTTTT	17965	+1 t	24.63%
GCTTCGTGACGTTGGAATGGAGGGTTTT	3140	+1 Ins	4.30%
GCTTCGTGACGT----TGGAGGGTTTT	2826	-4	3.87%
GCTTCGTGACGTTGGA-GGAGGGTTTT	2181	-1	2.99%
GCTTCGTGACGTTGGAT-GAGGGTTTT	239	-1	0.33%
GCTTCGTGACGTTGG-TGGAGGGTTTT	248	-1	0.34%
GCTTCGTGACGTTG--TGGAGGGTTTT	167	-2	0.23%
GCTTCGTGACGTTGGACTGGAGGGTTTT	116	+1 Ins	0.16%
GCCTCGTGACGTTGGATTGGAGGGTTTT	113	+1 Ins	0.15%

YW-W9-KO-24h-Syn92crBCL11A7b-BC1 24h	Ins	Del	HDR
102469 | 55.79%	41.13%	14.65%	0.00%
**Typical seqs**	**Reads**	**Type**	**pct.**
GCTTCGTGACGTTGGAT|GGA**GGG**TTTT	45303	WT	44.21%
GCTTCGTGACGTTGGATtGGAGGGTTTT	33792	+1 t	32.98%
GCTTCGTGACGT----TGGAGGGTTTT	6422	-4	6.27%
GCTTCGTGACGTTGGAATGGAGGGTTTT	3304	+1 Ins	3.22%
GCTTCGTGACGTTGGA-GGAGGGTTTT	2048	-1	2.00%
GCTTCGTGACGTTGG-TGGAGGGTTTT	390	-1	0.38%
GCTTCGTGACGTT-------GGGTTTT	360	-7	0.35%
GCTTCGTGACGTTGGAT-GAGGGTTTT	335	-1	0.33%
GCTTCGTGACGTTGGATATGGAGGGTTTT	308	+2	0.30%
GCTTCGTGGCGTTGGATTGGAGGGTTTT	268	+1 Ins	0.26%

YW-W9-KO-48h-Syn92crBCL11A7b-BC1 48h	Ins	Del	HDR
58872 | 69.76%	49.99%	19.77%	0.00%
**Typical seqs**	**Reads**	**Type**	**pct.**
GCTTCGTGACGTTGGAT|GGA**GGG**TTTT	17803	WT	30.24%
GCTTCGTGACGTTGGATtGGAGGGTTTT	24712	+1 t	41.98%
GCTTCGTGACGT----TGGAGGGTTTT	5704	-4	9.69%
GCTTCGTGACGTTGGAATGGAGGGTTTT	886	+1 Ins	1.50%
GCTTCGTGACGTTGGA-GGAGGGTTTT	613	-1	1.04%
GCTTCGTGACGTT-------GGGTTTT	554	-7	0.94%
GCTTCGTGAC------------GTTTT	362	-12	0.61%
GCTTCGTGACGTT--------GGTTTT	313	-8	0.53%
GCTTCGTGACGTTGGAT-GAGGGTTTT	255	-1	0.43%
GCTTCGTGACGTTGG-TGGAGGGTTTT	237	-1	0.40%

P138-P142-YW-W9-Syn93crBCL11A7c-KO

YW-W9-KO-4h-Syn93crBCL11A7c-BC2 4h	Ins	Del	HDR
29779 | 15.99%	14.29%	1.70%	0.00%
**Typical seqs**	**Reads**	**Type**	**pct.**
AGCTTCGTGACGTTGGA|TGG**AGG**GTTT	25016	WT	84.01%
AGCTTCGTGACGTTGGAtTGGAGGGTTT	3801	+1 t	12.76%
AGCTTCGTGACGT----TGGAGGGTTT	264	-4	0.89%
AGCTTCGTGACGTTGGA-GGAGGGTTT	64	-1	0.21%
AGCTTCGTGACGTTGGAT-GAGGGTTT	42	-1	0.14%
AGCTTCGTGACGTTGGATATGGAGGGTTT	34	+2	0.11%
AGCTTCGTGACGTTGGATTTGGAGGGTTT	32	+2	0.11%
AGCCTCGTGACGTTGGATTGGAGGGTTT	32	+1 Ins	0.11%
AGCTTCGTGGCGTTGGATTGGAGGGTTT	30	+1 Ins	0.10%
AGCTTCGCGACGTTGGATTGGAGGGTTT	26	+1 Ins	0.09%

YW-W9-KO-8h-Syn93crBCL11A7c-BC2 8h	Ins	Del	HDR
61111 | 31.79%	22.43%	9.36%	0.00%
**Typical seqs**	**Reads**	**Type**	**pct.**
AGCTTCGTGACGTTGGA|TGG**AGG**GTTT	41684	WT	68.21%
AGCTTCGTGACGTTGGAtTGGAGGGTTT	9502	+1 t	15.55%
AGCTTCGTGACGTTGGAaTGGAGGGTTT	2512	+1 a	4.11%
AGCTTCGTGACGT----TGGAGGGTTT	2138	-4	3.50%
AGCTTCGTGACGTTGGA-GGAGGGTTT	1937	-1	3.17%
AGCTTCGTGACGTTGGAT-GAGGGTTT	180	-1	0.29%
AGCTTCGTGACGTTGGA--GAGGGTTT	116	-2	0.19%
AGCTTCGTGACGTTGG-TGGAGGGTTT	113	-1	0.18%
AGCTTCGTGACGTTGGAcTGGAGGGTTT	100	+1 c	0.16%
AGCCTCGTGACGTTGGATTGGAGGGTTT	94	+1 Ins	0.15%

YW-W9-KO-12h-Syn93crBCL11A7c-BC2 12h	Ins	Del	HDR
64191 | 36.44%	25.44%	11.00%	0.00%
**Typical seqs**	**Reads**	**Type**	**pct.**
AGCTTCGTGACGTTGGA|TGG**AGG**GTTT	40802	WT	63.56%
AGCTTCGTGACGTTGGAtTGGAGGGTTT	10727	+1 t	16.71%
AGCTTCGTGACGTTGGAaTGGAGGGTTT	3660	+1 a	5.70%
AGCTTCGTGACGTTGGA-GGAGGGTTT	2516	-1	3.92%
AGCTTCGTGACGT----TGGAGGGTTT	2401	-4	3.74%
AGCTTCGTGACGTTGG-TGGAGGGTTT	264	-1	0.41%
AGCTTCGTGACGTTGGAT-GAGGGTTT	164	-1	0.26%
AGCTTCGTGACGTTGGA--GAGGGTTT	108	-2	0.17%
AGCTTCGTGACGTTGGAcTGGAGGGTTT	102	+1 c	0.16%
AGCTTCGTGACGTTG--TGGAGGGTTT	98	-2	0.15%

YW-W9-KO-24h-Syn93crBCL11A7c-BC2 24h	Ins	Del	HDR
34372 | 49.77%	31.48%	18.29%	0.00%
**Typical seqs**	**Reads**	**Type**	**pct.**
AGCTTCGTGACGTTGGA|TGG**AGG**GTTT	17266	WT	50.23%
AGCTTCGTGACGTTGGAtTGGAGGGTTT	6612	+1 t	19.24%
AGCTTCGTGACGTTGGAaTGGAGGGTTT	2822	+1 a	8.21%
AGCTTCGTGACGT----TGGAGGGTTT	2273	-4	6.61%
AGCTTCGTGACGTTGGA-GGAGGGTTT	1512	-1	4.40%
AGCTTCGTGACGTTGG-TGGAGGGTTT	141	-1	0.41%
AGCTTCGTGACGTT-------GGGTTT	106	-7	0.31%
AGCTTCGTGACGTT-GATGGAGGGTTT	103	-1	0.30%
AGCTTCGTGACGTTGGA--GAGGGTTT	103	-2	0.30%
AGCTTCGTGACGTTG--TGGAGGGTTT	93	-2	0.27%

YW-W9-KO-48h-Syn93crBCL11A7c-BC2 48h	Ins	Del	HDR
18824 | 49.66%	25.94%	23.72%	0.00%
**Typical seqs**	**Reads**	**Type**	**pct.**
AGCTTCGTGACGTTGGA|TGG**AGG**GTTT	9476	WT	50.34%
AGCTTCGTGACGTTGGAtTGGAGGGTTT	2207	+1 t	11.72%
AGCTTCGTGACGTTGGAaTGGAGGGTTT	2062	+1 a	10.95%
AGCTTCGTGACGT----TGGAGGGTTT	1883	-4	10.00%
AGCTTCGTGACGTTGGA-GGAGGGTTT	1053	-1	5.59%
AGCTTCGTGAC------------GTTT	136	-12	0.72%
AGCTTCGTGACGTT-------GGGTTT	90	-7	0.48%
AGCTTCGTGACGTTGG-TGGAGGGTTT	85	-1	0.45%
AGCTTCG----------TGGAGGGTTT	72	-10	0.38%
AGCTTCGTGACGTT--------GGTTT	69	-8	0.37%

P138-P142-YW-W9-Syn94crBCL11A7d-KO

YW-W9-KO-4h-Syn94crBCL11A7d 4h 23260 | 2.06%	Ins 1.84%	Del 0.21%	HDR 0.00%
**Typical seqs**	**Reads**	**Type**	**pct.**
GCGTAGTGTTGGGTCCT|ACC**TGG**CCAC	22782	WT	97.94%
GCGTAGTGTTGGGTCCTtACCTGGCCAC	417	+1 t	1.79%
GCGTAGTGTTGGGTCCTACCT-GCCAC	25	-1	0.11%
GCGTAGTGTTGGGTC--ACCTGGCCAC	8	-2	0.03%
GCGTAGTGTTGGGT----CCTGGCCAC	6	-4	0.03%
GCGTAGTGTTGGGTCCTCTACCTGGCCAC	4	+2	0.02%
GCGTAGTGTTGGGTCC-ACCTGGCCAC	4	-1	0.02%
GCGTAGTGTTGGGTCCTaACCTGGCCAC	4	+1 a	0.02%
GCGTAGTGTTG-----TACCTGGCCAC	3	-5	0.01%
GCGTAGTGTTGGGTCCTtACCCGGCCAC	3	+1 t	0.01%

YW-W9-KO-8h-Syn94crBCL11A7d 8h 55199 | 29.76%	Ins 25.27%	Del 4.48%	HDR 0.00%
**Typical seqs**	**Reads**	**Type**	**pct.**
GCGTAGTGTTGGGTCCT|ACC**TGG**CCAC	38774	WT	70.24%
GCGTAGTGTTGGGTCCTtACCTGGCCAC	12024	+1 t	21.78%
GCGTAGTGTTGGGTCC-ACCTGGCCAC	448	-1	0.81%
GCGTAGTGTTGGGT----CCTGGCCAC	388	-4	0.70%
GCGTAGTGTTGGGTCCTCTACCTGGCCAC	357	+2	0.65%
GCGTAGTGTTGGGT-CTACCTGGCCAC	319	-1	0.58%
GCGTAGTGTTGGGTCCTaACCTGGCCAC	180	+1 a	0.33%
GCGTAGTGTTGGGTC-----------C	156	-11	0.28%
GCGTAGTGTTGGG---TACCTGGCCAC	140	-3	0.25%
GCGTAGTGTTGGGTC--ACCTGGCCAC	118	-2	0.21%

YW-W9-KO-12h-Syn94crBCL11A7d 12h 26817 | 37.59%	Ins 31.58%	Del 6.01%	HDR 0.00%
**Typical seqs**	**Reads**	**Type**	**pct.**
GCGTAGTGTTGGGTCCT|ACC**TGG**CCAC	16737	WT	62.41%
GCGTAGTGTTGGGTCCTtACCTGGCCAC	7108	+1 t	26.51%
GCGTAGTGTTGGGT-CTACCTGGCCAC	259	-1	0.97%
GCGTAGTGTTGGGTCCTCTACCTGGCCAC	206	+2	0.77%
GCGTAGTGTTGGGT----CCTGGCCAC	205	-4	0.76%
GCGTAGTGTTGGGTCC-ACCTGGCCAC	152	-1	0.57%
GCGTAGTGTTGGGTC--ACCTGGCCAC	112	-2	0.42%
GCGTAGTGTTGGG---TACCTGGCCAC	109	-3	0.41%
GCGTAGTGTTGGGTCCTaACCTGGCCAC	79	+1 a	0.29%
GCGTAGTGTTGGGTC-----------C	73	-11	0.27%

YW-W9-KO-24h-Syn94crBCL11A7d 24h 18839 | 55.24%	Ins 46.78%	Del 8.46%	HDR 0.00%
**Typical seqs**	**Reads**	**Type**	**pct.**
GCGTAGTGTTGGGTCCT|ACC**TGG**CCAC	8433	WT	44.76%
GCGTAGTGTTGGGTCCTtACCTGGCCAC	7503	+1 t	39.83%
GCGTAGTGTTGGGT----CCTGGCCAC	305	-4	1.62%
GCGTAGTGTTGGGTCC-ACCTGGCCAC	282	-1	1.50%
GCGTAGTGTTGGGTCCTCTACCTGGCCAC	199	+2	1.06%
GCGTAGTGTTGGGT-CTACCTGGCCAC	170	-1	0.90%
GCGTAGTGTTGGGTC--ACCTGGCCAC	87	-2	0.46%
GCGTAGTGTTGGG---TACCTGGCCAC	74	-3	0.39%
GCGTAGTGTTGGGTC-----------C	71	-11	0.38%
GCGTAGTGTTGGGTCCTaACCTGGCCAC	59	+1 a	0.31%

YW-W9-KO-48h-Syn94crBCL11A7d 48h 12825 | 58.90%	Ins 47.57%	Del 11.33%	HDR 0.00%
**Typical seqs**	**Reads**	**Type**	**pct.**
GCGTAGTGTTGGGTCCT|ACC**TGG**CCAC	5271	WT	41.10%
GCGTAGTGTTGGGTCCTtACCTGGCCAC	5198	+1 t	40.53%
GCGTAGTGTTGGGT----CCTGGCCAC	291	-4	2.27%
GCGTAGTGTTGGGT-CTACCTGGCCAC	158	-1	1.23%
GCGTAGTGTTGGGTCC-ACCTGGCCAC	154	-1	1.20%
GCGTAGTGTTGGGTCCTCTACCTGGCCAC	108	+2	0.84%
GCGTAGTGT-----------TGGCCAC	80	-11	0.62%
GCGTAGTGTTGGGTCCTaACCTGGCCAC	67	+1 a	0.52%
GCGTAGTGTTGGGTC--ACCTGGCCAC	61	-2	0.48%
GCGTAGTGTTGGGT----------CAC	56	-10	0.44%

P138-P142-YW-W9-Syn95crBCL11A8a-KO

YW-W9-KO-4h-Syn95crBCL11A8a 4h	Ins	Del	HDR
87424 | 0.76%	0.49%	0.27%	0.00%
**Typical seqs**	**Reads**	**Type**	**pct.**
GCTTCTGAGCGTCAAAC|TCG**AGG**AGCA	86758	WT	99.24%
GCTTCTGAGCGTCAAACcTCGAGGAGCA	320	+1 c	0.37%
GCTTCTGAGCGTCAAACtTCGAGGAGCA	66	+1 t	0.08%
GCTTCTGAGCGTCAAA--CGAGGAGCA	55	-2	0.06%
GCTTCTGAGCGTC-AACTCGAGGAGCA	26	-1	0.03%
GCTTCTGAGCGTCAA--TCGAGGAGCA	26	-2	0.03%
GCTTCTGAGCGTCAAAC-CGAGGAGCA	21	-1	0.02%
GCTTCTGAGCGTCAAAC----GGAGCA	14	-4	0.02%
GCTTCTGAGCG------TCGAGGAGCA	12	-6	0.01%
GCTTCTGAGCGTCAAACgTCGAGGAGCA	11	+1 g	0.01%

YW-W9-KO-8h-Syn95crBCL11A8a 8h	Ins	Del	HDR
81371 | 19.13%	15.23%	3.90%	0.00%
**Typical seqs**	**Reads**	**Type**	**pct.**
GCTTCTGAGCGTCAAAC|TCG**AGG**AGCA	65807	WT	80.87%
GCTTCTGAGCGTCAAACcTCGAGGAGCA	9225	+1 c	11.34%
GCTTCTGAGCGTCAAACtTCGAGGAGCA	1271	+1 t	1.56%
GCTTCTGAGCG------TCGAGGAGCA	671	-6	0.82%
GCTTCTGAGCGTCAAACgTCGAGGAGCA	363	+1 g	0.45%
GCTTCTGAGCGTCAAAC-CGAGGAGCA	252	-1	0.31%
GCTTCT----------------GAGCA	206	-16	0.25%
GCT--------------TCGAGGAGCA	185	-14	0.23%
GCTTCTGAGCGTCAAACACTCGAGGAGCA	165	+2	0.20%
GCTTCTGAGCGTC-AACTCGAGGAGCA	162	-1	0.20%

YW-W9-KO-12h-Syn95crBCL11A8a 12h	Ins	Del	HDR
76060 | 24.43%	18.16%	6.27%	0.00%
**Typical seqs**	**Reads**	**Type**	**pct.**
GCTTCTGAGCGTCAAAC|TCG**AGG**AGCA	57477	WT	75.57%
GCTTCTGAGCGTCAAACcTCGAGGAGCA	10139	+1 c	13.33%
GCTTCTGAGCGTCAAACtTCGAGGAGCA	1658	+1 t	2.18%
GCTTCTGAGCG------TCGAGGAGCA	647	-6	0.85%
GCTTCTGAGCGTCAAAC-CGAGGAGCA	607	-1	0.80%
GCTTCT----------------GAGCA	396	-16	0.52%
GCTTCTGAGCGTCAAA--CGAGGAGCA	254	-2	0.33%
GCTTCTGAGCGTCAA--TCGAGGAGCA	255	-2	0.34%
GCTTCTGAGCGTC-AACTCGAGGAGCA	247	-1	0.32%
GCTTCTGAGCGTCAAACgTCGAGGAGCA	248	+1 g	0.33%

YW-W9-KO-24h-Syn95crBCL11A8a 24h	Ins	Del	HDR
40637 | 37.10%	26.46%	10.64%	0.00%
**Typical seqs**	**Reads**	**Type**	**pct.**
GCTTCTGAGCGTCAAAC|TCG**AGG**AGCA	25559	WT	62.90%
GCTTCTGAGCGTCAAACcTCGAGGAGCA	7238	+1 c	17.81%
GCTTCTGAGCGTCAAACtTCGAGGAGCA	1763	+1 t	4.34%
GCTTCTGAGCG------TCGAGGAGCA	408	-6	1.00%
GCTTCT----------------GAGCA	352	-16	0.87%
GCTTCTGAGCGTCAAAC-CGAGGAGCA	349	-1	0.86%
GCT--------------TCGAGGAGCA	309	-14	0.76%
GCTTCTGAGCGTCAAA-TCGAGGAGCA	259	-1	0.64%
GCTTCTGAGCGTCAAACaTCGAGGAGCA	229	+1 a	0.56%
GCTTCTGAGCGTCAAACgTCGAGGAGCA	172	+1 g	0.42%

YW-W9-KO-48h-Syn95crBCL11A8a 48h	Ins	Del	HDR
32692 | 35.65%	23.18%	12.47%	0.00%
**Typical seqs**	**Reads**	**Type**	**pct.**
GCTTCTGAGCGTCAAAC|TCG**AGG**AGCA	21038	WT	64.35%
GCTTCTGAGCGTCAAACcTCGAGGAGCA	4955	+1 c	15.16%
GCTTCTGAGCGTCAAACtTCGAGGAGCA	1427	+1 t	4.36%
GCTTCTGAGCG------TCGAGGAGCA	538	-6	1.65%
GCTTCTGAGCGTCAAAC-CGAGGAGCA	264	-1	0.81%
GCTTCTGAGCGTCAAACgTCGAGGAGCA	206	+1 g	0.63%
GCTTCT----------------GAGCA	183	-16	0.56%
GCTTCTGAGCGTCAAA-TCGAGGAGCA	154	-1	0.47%
GCT--------------TCGAGGAGCA	157	-14	0.48%
GCTTCTGAGCGTCAA--TCGAGGAGCA	144	-2	0.44%

P138-P142-YW-W9-Syn96crBCL11A8b-KO

YW-W9-KO-4h-Syn96crBCL11A8b 4h	Ins	Del	HDR
93167 | 0.28%	0.05%	0.24%	0.00%
**Typical seqs**	**Reads**	**Type**	**pct.**
GCGGGCCACCCACATTC|ATT**GGG**GGAA	92903	WT	99.72%
GCGGGCCACCCA----CATTGGGGGAA	66	-4	0.07%
GCGGGCCACCCACATTC-TTGGGGGAA	19	-1	0.02%
GCGGGCCACCCACATTC---GGGGGAA	19	-3	0.02%
GCGGGCCACCCACATTC----GGGGAA	18	-4	0.02%
GCGGGCCACCCACAT--ATTGGGGGAA	15	-2	0.02%
GCGGGCCACCCACATTCcATTGGGGGAA	14	+1 c	0.02%
GCGGGCCACCCACA-TCATTGGGGGAA	12	-1	0.01%
GCGGGCCACCCACATTCaATTGGGGGAA	11	+1 a	0.01%
GCGGGCCACCCACATTCA-TGGGGGAA	10	-1	0.01%

YW-W9-KO-8h-Syn96crBCL11A8b 8h	Ins	Del	HDR
86308 | 9.44%	1.46%	7.98%	0.00%
**Typical seqs**	**Reads**	**Type**	**pct.**
GCGGGCCACCCACATTC|ATT**GGG**GGAA	78161	WT	90.56%
GCGGGCCACCCA----CATTGGGGGAA	1955	-4	2.27%
GCGGGCCACCCACATT-ATTGGGGGAA	1247	-1	1.44%
GCGGGCCACCCACATTC-TTGGGGGAA	1151	-1	1.33%
GCGGGCCACCCACATTCcATTGGGGGAA	582	+1 c	0.67%
GCGGGCCACCCACAT--ATTGGGGGAA	497	-2	0.58%
GCGGGCCACCCACATTCaATTGGGGGAA	281	+1 a	0.33%
GCGGGCCACCCACA---ATTGGGGGAA	254	-3	0.29%
GCGGGCCACCCACATTC---GGGGGAA	243	-3	0.28%
GCGGGC----------CATTGGGGGAA	146	-10	0.17%

YW-W9-KO-12h-Syn96crBCL11A8b 12h	Ins	Del	HDR
50538 | 14.58%	2.24%	12.34%	0.00%
**Typical seqs**	**Reads**	**Type**	**pct.**
GCGGGCCACCCACATTC|ATT**GGG**GGAA	43170	WT	85.42%
GCGGGCCACCCA----CATTGGGGGAA	1747	-4	3.46%
GCGGGCCACCCACATT-ATTGGGGGAA	823	-1	1.63%
GCGGGCCACCCACATTC-TTGGGGGAA	720	-1	1.42%
GCGGGCCACCCACATTCcATTGGGGGAA	510	+1 c	1.01%
GCGGGCCACCCACAT--ATTGGGGGAA	429	-2	0.85%
GCGGGCCACCCACA---ATTGGGGGAA	357	-3	0.71%
GCGGGCCACCCACATTCaATTGGGGGAA	190	+1 a	0.38%
GCGGGCCACCCACATT-----------	189	-15	0.37%
GCGGGCCACCCACATTC--TGGGGGAA	146	-2	0.29%

YW-W9-KO-24h-Syn96crBCL11A8b 24h	Ins	Del	HDR
56377 | 26.72%	3.26%	23.46%	0.00%
**Typical seqs**	**Reads**	**Type**	**pct.**
GCGGGCCACCCACATTC|ATT**GGG**GGAA	41311	WT	73.28%
GCGGGCCACCCA----CATTGGGGGAA	3831	-4	6.80%
GCGGGCCACCCACATT-ATTGGGGGAA	1662	-1	2.95%
GCGGGCCACCCACATTC-TTGGGGGAA	1295	-1	2.30%
GCGGGCCACCCACAT--ATTGGGGGAA	725	-2	1.29%
GCGGGCCACCCACA---ATTGGGGGAA	637	-3	1.13%
GCGGGCCACCCACATTCcATTGGGGGAA	503	+1 c	0.89%
GCGGGCCACCCACATTCaATTGGGGGAA	420	+1 a	0.74%
GCGGGCCACCCACATTC---GGGGGAA	395	-3	0.70%
GCGGG-----------CATTGGGGGAA	368	-11	0.65%

YW-W9-KO-48h-Syn96crBCL11A8b 48h	Ins	Del	HDR
37675 | 30.44%	3.39%	27.05%	0.00%
**Typical seqs**	**Reads**	**Type**	**pct.**
GCGGGCCACCCACATTC|ATT**GGG**GGAA	26207	WT	69.56%
GCGGGCCACCCA----CATTGGGGGAA	3281	-4	8.71%
GCGGGCCACCCACATTC-TTGGGGGAA	882	-1	2.34%
GCGGGCCACCCACATT-ATTGGGGGAA	869	-1	2.31%
GCGGGCCACCCACAT--ATTGGGGGAA	728	-2	1.93%
GCGGGCCACCCACATTCcATTGGGGGAA	388	+1 c	1.03%
GCGGGCCA--------CATTGGGGGAA	277	-8	0.74%
GCGGGCCACCCACA---ATTGGGGGAA	248	-3	0.66%
GCGGGCCACCCACATTCaATTGGGGGAA	228	+1 a	0.61%
GCGGGCCACCCACATTCtATTGGGGGAA	231	+1 t	0.61%

P138-P142-YW-W9-Syn97crBCL11A8c-KO

YW-W9-KO-4h-Syn97crBCL11A8c 4h<br>62840 | 0.50%	Ins<br>0.14%	Del<br>0.36%	HDR<br>0.00%
**Typical seqs**	**Reads**	**Type**	**pct.**
CGCGGCAGAAACCTGTC|GTC**TGG**ACAC	62528	WT	99.50%
CGCGGCAGAAACCTGTCGTCT-GACAC	88	-1	0.14%
CGCGGCAGAAACCTGTCcGTCTGGACAC	76	+1 c	0.12%
CGCGGCAGAAACCT---GTCTGGACAC	43	-3	0.07%
CGCGGCAGAAA-----CGTCTGGACAC	17	-5	0.03%
CGCGGCAGAAACCTG--GTCTGGACAC	16	-2	0.03%
CGCGGCAGAAACCTGT-GTCTGGACAC	9	-1	0.01%
CGCGGCAGAAACCT-------------	8	-15	0.01%
CGCGGCAGAAAC-------CTGGACAC	7	-7	0.01%
CGCGGCAGAAACCTGTC-TCTGGACAC	7	-1	0.01%

YW-W9-KO-8h-Syn97crBCL11A8c 8h<br>62232 | 6.47%	Ins<br>2.31%	Del<br>4.15%	HDR<br>0.00%
**Typical seqs**	**Reads**	**Type**	**pct.**
CGCGGCAGAAACCTGTC|GTC**TGG**ACAC	58208	WT	93.53%
CGCGGCAGAAACCT---GTCTGGACAC	1309	-3	2.10%
CGCGGCAGAAACCTGTCcGTCTGGACAC	1208	+1 c	1.94%
CGCGGCAGAAAC----CGTCTGGACAC	172	-4	0.28%
CGCGGCAGAAAC-------CTGGACAC	150	-7	0.24%
CGCGGCAGAAACCTG--GTCTGGACAC	103	-2	0.17%
CGCGGCAGAAACCTGT-GTCTGGACAC	94	-1	0.15%
CGCGGCAGAAACCTGTC-TCTGGACAC	92	-1	0.15%
CGCGGCAGAAACCTGTCGTCT-GACAC	60	-1	0.10%
CGCGGCAGAAACCT-------------	42	-15	0.07%

YW-W9-KO-12h-Syn97crBCL11A8c 12h<br>28937 | 9.34%	Ins<br>3.53%	Del<br>5.82%	HDR<br>0.00%
**Typical seqs**	**Reads**	**Type**	**pct.**
CGCGGCAGAAACCTGTC|GTC**TGG**ACAC	26233	WT	90.66%
CGCGGCAGAAACCTGTCcGTCTGGACAC	857	+1 c	2.96%
CGCGGCAGAAACCT---GTCTGGACAC	760	-3	2.63%
CGCGGCAGAAACCTGT-GTCTGGACAC	167	-1	0.58%
CGCGGCAGAAAC----CGTCTGGACAC	93	-4	0.32%
CGCGGCAGAAAC-------CTGGACAC	74	-7	0.26%
CGCGGCAGAAACCTG-CGTCTGGACAC	48	-1	0.17%
CGCGGCAGAAACCT-------------	37	-15	0.13%
CGCGGCAGAAACCTGTC--CTGGACAC	35	-2	0.12%
CGCGGCAGAAACCTGTCtGTCTGGACAC	30	+1 t	0.10%

YW-W9-KO-24h-Syn97crBCL11A8c 24h<br>45569 | 14.65%	Ins<br>4.75%	Del<br>9.90%	HDR<br>0.00%
**Typical seqs**	**Reads**	**Type**	**pct.**
CGCGGCAGAAACCTGTC|GTC**TGG**ACAC	38893	WT	85.35%
CGCGGCAGAAACCT---GTCTGGACAC	2019	-3	4.43%
CGCGGCAGAAACCTGTCcGTCTGGACAC	1682	+1 c	3.69%
CGCGGCAGAAACCTGT-GTCTGGACAC	244	-1	0.54%
CGCGGCAGAAAC----CGTCTGGACAC	233	-4	0.51%
CGCGGCAGAAAC-------CTGGACAC	198	-7	0.43%
CGCGGCAGAAACCTG--GTCTGGACAC	136	-2	0.30%
CGCGGCAGAAACCT-------------	101	-15	0.22%
CGCGGCAGAAACCTGTC-TCTGGACAC	95	-1	0.21%
CGCGGCAGAA---------------AC	85	-15	0.19%

YW-W9-KO-48h-Syn97crBCL11A8c 48h<br>31088 | 15.16%	Ins<br>3.20%	Del<br>11.96%	HDR<br>0.00%
**Typical seqs**	**Reads**	**Type**	**pct.**
CGCGGCAGAAACCTGTC|GTC**TGG**ACAC	26375	WT	84.84%
CGCGGCAGAAACCT---GTCTGGACAC	1950	-3	6.27%
CGCGGCAGAAACCTGTCcGTCTGGACAC	711	+1 c	2.29%
CGCGGCAGAAAC-------CTGGACAC	207	-7	0.67%
CGCGGCAGAAAC----CGTCTGGACAC	115	-4	0.37%
CGCGGCAGAAACCTGT-GTCTGGACAC	107	-1	0.34%
CGCGGCAGAAACCTG--GTCTGGACAC	78	-2	0.25%
CGCGGCAGAAA-----CGTCTGGACAC	75	-5	0.24%
CGCGGCAGAAAC--------------C	74	-14	0.24%
CGCGGCAGAAACCT-------------	72	-15	0.23%

P138-P142-YW-W9-Syn98crBCL11A8d-KO

YW-W9-KO-4h-Syn98crBCL11A8d 4h	Ins	Del	HDR
119014 | 0.20%	0.07%	0.13%	0.00%
**Typical seqs**	**Reads**	**Type**	**pct.**
GCCTTGATTACTCAAAC|AAC**AGG**TTAC	118775	WT	99.80%
GCCTTGATTACTCAAACcAACAGGTTAC	74	+1 c	0.06%
GCCTTGATTACTCA---AACAGGTTAC	64	-3	0.05%
GCCTTGATTACTC-AACAACAGGTTAC	19	-1	0.02%
GCCTTGATTACTCAAAC-ACAGGTTAC	14	-1	0.01%
GCCTTGA----------------TTAC	12	-16	0.01%
GCCTTGATTACTCAA----------AC	6	-10	0.01%
GCCTTGATTACTCAAA-AACAGGTTAC	5	-1	0.00%
GCCTTGATTACTCA------AGGTTAC	5	-6	0.00%
GCCTTGATTACTC--ACAACAGGTTAC	5	-2	0.00%

YW-W9-KO-8h-Syn98crBCL11A8d 8h	Ins	Del	HDR
103450 | 21.58%	12.27%	9.31%	0.00%
**Typical seqs**	**Reads**	**Type**	**pct.**
GCCTTGATTACTCAAAC|AAC**AGG**TTAC	81124	WT	78.42%
GCCTTGATTACTCAAACcAACAGGTTAC	10712	+1 c	10.35%
GCCTTGATTACTCA---AACAGGTTAC	3032	-3	2.93%
GCCTTGATTACTCAA--AACAGGTTAC	879	-2	0.85%
GCCTTGATTACT----CAACAGGTTAC	840	-4	0.81%
GCCTTGATTACTC-AACAACAGGTTAC	651	-1	0.63%
GCCTTGATTACTCAAA-AACAGGTTAC	621	-1	0.60%
GCCTTGATTACTCAAAC-ACAGGTTAC	304	-1	0.29%
GCCTTGATTACTCA-------------	278	-14	0.27%
GCCTTGATTACTCAAACACAACAGGTTAC	210	+2	0.20%

YW-W9-KO-12h-Syn98crBCL11A8d 12h	Ins	Del	HDR
87367 | 27.19%	15.97%	11.21%	0.00%
**Typical seqs**	**Reads**	**Type**	**pct.**
GCCTTGATTACTCAAAC|AAC**AGG**TTAC	63614	WT	72.81%
GCCTTGATTACTCAAACcAACAGGTTAC	11371	+1 c	13.02%
GCCTTGATTACTCA---AACAGGTTAC	2721	-3	3.11%
GCCTTGATTACTCAA--AACAGGTTAC	1056	-2	1.21%
GCCTTGATTACT----CAACAGGTTAC	664	-4	0.76%
GCCTTGATTACTCAAAC-ACAGGTTAC	427	-1	0.49%
GCCTTGATTACTCAAA-AACAGGTTAC	402	-1	0.46%
GCCTTGATTACTC-AACAACAGGTTAC	388	-1	0.44%
GCCTTGATTACTCAAACaAACAGGTTAC	348	+1 a	0.40%
GCCTTGATTACTCAAACtAACAGGTTAC	313	+1 t	0.36%

YW-W9-KO-24h-Syn98crBCL11A8d 24h	Ins	Del	HDR
98705 | 40.79%	19.48%	21.31%	0.00%
**Typical seqs**	**Reads**	**Type**	**pct.**
GCCTTGATTACTCAAAC|AAC**AGG**TTAC	58442	WT	59.21%
GCCTTGATTACTCAAACcAACAGGTTAC	15318	+1 c	15.52%
GCCTTGATTACTCA---AACAGGTTAC	5413	-3	5.48%
GCCTTGATTACT----CAACAGGTTAC	1225	-4	1.24%
GCCTTGATTACTCAA--AACAGGTTAC	1198	-2	1.21%
GCCTTGATTACTCAAAC-ACAGGTTAC	1122	-1	1.14%
GCCTTGATTACTC-AACAACAGGTTAC	965	-1	0.98%
GCCTTGA----------------TTAC	864	-16	0.88%
GCCTTGATTACTCA-------------	792	-14	0.80%
GCCTTGATTACTC--ACAACAGGTTAC	627	-2	0.64%

YW-W9-KO-48h-Syn98crBCL11A8d 48h	Ins	Del	HDR
41490 | 39.26%	17.81%	21.45%	0.00%
**Typical seqs**	**Reads**	**Type**	**pct.**
GCCTTGATTACTCAAAC|AAC**AGG**TTAC	25199	WT	60.74%
GCCTTGATTACTCAAACcAACAGGTTAC	5747	+1 c	13.85%
GCCTTGATTACTCA---AACAGGTTAC	2726	-3	6.57%
GCCTTGATTACT----CAACAGGTTAC	532	-4	1.28%
GCCTTGATTACTCAA--AACAGGTTAC	550	-2	1.33%
GCCTTGA----------------TTAC	481	-16	1.16%
GCCTTGATTACTC-AACAACAGGTTAC	288	-1	0.69%
GCCTTGATTACTCA-------------	299	-14	0.72%
GCCTTGATTACTCAAA-AACAGGTTAC	255	-1	0.61%
GCCTTGATTACTCAAACaAACAGGTTAC	232	+1 a	0.56%

P138-P142-YW-W9-Syn99crBCL11A9a-KO

YW-W9-KO-4h-Syn99crBCL11A9a 4h<br>103607 | 1.66%	Ins<br>1.51%	Del<br>0.15%	HDR<br>0.00%
Typical seqs	Reads	Type	pct.
GCGCAAACTCCCGTTCT|CCG**AGG**AGTG	101889	WT	98.34%
GCGCAAACTCCCGTTCTtCCGAGGAGTG	1413	+1 t	1.36%
GCGCAAGCTCCCGTTCTTCCGAGGAGTG	31	+1 Ins	0.03%
GCGCAAACTC-------CCGAGGAGTG	24	-7	0.02%
GCGCAAACTCCCG---TCCGAGGAGTG	19	-3	0.02%
GCGCAAA--------------------	15	-20	0.01%
GCGCAAACTCCCGTTCT--GAGGAGTG	15	-2	0.01%
GCGCAAACTCCCGTTCT---AGGAGTG	14	-3	0.01%
GCGCAAACTCCC-----CCGAGGAGTG	12	-5	0.01%
GCGCGAACTCCCGTTCTTCCGAGGAGTG	11	+1 Ins	0.01%

YW-W9-KO-8h-Syn99crBCL11A9a 8h<br>82472 | 36.91%	Ins<br>33.34%	Del<br>3.57%	HDR<br>0.00%
Typical seqs	Reads	Type	pct.
GCGCAAGCTCCCGTTCT|CCG**AGG**AGTG	52028	WT	63.09%
GCGCAAGCTCCCGTTCTtCCGAGGAGTG	13310	+1 t	16.14%
GCGCAAACTCCCGTTCTTCCGAGGAGTG	11409	+1 Ins	13.83%
GCGCAAACTCCCGT--TCCGAGGAGTG	453	-2	0.55%
GCGCAAGCTC-------CCGAGGAGTG	384	-7	0.47%
GCGCAAACTC-------CCGAGGAGTG	380	-7	0.46%
GCGCAAACTCCCGTTCTTTCCGAGGAGTG	330	+2	0.40%
GCGCAAACTCCCGTT-TCCGAGGAGTG	258	-1	0.31%
GCGCAAGCTCCCGTTC-CCGAGGAGTG	218	-1	0.26%
GCGCAAGCTCCCGTTCT-CGAGGAGTG	162	-1	0.20%

YW-W9-KO-12h-Syn99crBCL11A9a 12h<br>46345 | 46.38%	Ins<br>41.41%	Del<br>4.97%	HDR<br>0.00%
Typical seqs	Reads	Type	pct.
GCGCAAGCTCCCGTTCT|CCG**AGG**AGTG	24850	WT	53.62%
GCGCAAGCTCCCGTTCTtCCGAGGAGTG	9119	+1 t	19.68%
GCGCAAACTCCCGTTCTTCCGAGGAGTG	7987	+1 Ins	17.23%
GCGCAAACTC-------CCGAGGAGTG	439	-7	0.95%
GCGCAAGCTC-------CCGAGGAGTG	383	-7	0.83%
GCGCAAGCTCCCGTTC-CCGAGGAGTG	195	-1	0.42%
GCGCAAACTCCCGTTCTTTCCGAGGAGTG	140	+2	0.30%
GCGCAAACTCCCGT--TCCGAGGAGTG	116	-2	0.25%
GCGCAAACTCCCG---TCCGAGGAGTG	66	-3	0.14%
GCGCAAGCTCCCGTT-TCCGAGGAGTG	66	-1	0.14%

YW-W9-KO-24h-Syn99crBCL11A9a 24h<br>67088 | 63.33%	Ins<br>54.86%	Del<br>8.46%	HDR<br>0.00%
Typical seqs	Reads	Type	pct.
GCGCAAGCTCCCGTTCT|CCG**AGG**AGTG	24603	WT	36.67%
GCGCAAGCTCCCGTTCTtCCGAGGAGTG	17553	+1 t	26.16%
GCGCAAACTCCCGTTCTTCCGAGGAGTG	14318	+1 Ins	21.34%
GCGCAAGCTC-------CCGAGGAGTG	957	-7	1.43%
GCGCAAACTC-------CCGAGGAGTG	668	-7	1.00%
GCGCAAACTCCCGT--TCCGAGGAGTG	595	-2	0.89%
GCGCAAACTCCCGTTCTTTCCGAGGAGTG	284	+2	0.42%
GCGCAAGCTCCCGT--TCCGAGGAGTG	227	-2	0.34%
GCGCGAGCTCCCGTTCTTCCGAGGAGTG	141	+1 Ins	0.21%
GCGCAAACCCCCGTTCTTCCGAGGAGTG	136	+1 Ins	0.20%

YW-W9-KO-48h-Syn99crBCL11A9a 48h<br>27444 | 64.76%	Ins<br>52.88%	Del<br>11.88%	HDR<br>0.00%
Typical seqs	Reads	Type	pct.
GCGCAAGCTCCCGTTCT|CCG**AGG**AGTG	9671	WT	35.24%
GCGCAAGCTCCCGTTCTtCCGAGGAGTG	6728	+1 t	24.52%
GCGCAAACTCCCGTTCTTCCGAGGAGTG	5923	+1 Ins	21.58%
GCGCAAACTC-------CCGAGGAGTG	754	-7	2.75%
GCGCAAGCTC-------CCGAGGAGTG	480	-7	1.75%
GCGCAAACTCCCGT--TCCGAGGAGTG	239	-2	0.87%
GCGCAAGCTCCCGT--TCCGAGGAGTG	155	-2	0.56%
GCGCAAACTCCCGTTCTTTCCGAGGAGTG	161	+2	0.59%
GCGCAAACTCCCGT-------------	119	-14	0.43%
GC-----------------------G	82	-24	0.30%

P138-P142-YW-W9-Syn100crBCL11A9b-KO

YW-W9-KO-4h-Syn100crBCL11A9b 4h	Ins	Del	HDR
70713 | 0.78%	0.32%	0.46%	0.00%
**Typical seqs**	**Reads**	**Type**	**pct.**
GTCGGACTTGACCGTCA|TGG**GGG**ACGA	70163	WT	99.22%
GTCGGACTTGACCGTCAT-GGGGACGA	222	-1	0.31%
GTCGGACTTGACCGTCAaTGGGGGACGA	175	+1 a	0.25%
GTCGGACTTGACCGTCAtTGGGGGACGA	37	+1 t	0.05%
GTCGGACTTGACCGTC-TGGGGGACGA	32	-1	0.05%
GTCGGACTTGACCGTCA-GGGGGACGA	13	-1	0.02%
GTCGGACTTGACCGT--TGGGGGACGA	10	-2	0.01%
GTCGGACTTGACCGTCAgTGGGGGACGA	7	+1 g	0.01%
GTCGGACTTGACCGTCA--GGGGACGA	7	-2	0.01%
GTC--------------TGGGGGACGA	6	-14	0.01%

YW-W9-KO-8h-Syn100crBCL11A9b 8h	Ins	Del	HDR
62473 | 13.43%	6.97%	6.45%	0.00%
**Typical seqs**	**Reads**	**Type**	**pct.**
GTCGGACTTGACCGTCA|TGG**GGG**ACGA	54085	WT	86.57%
GTCGGACTTGACCGTCAaTGGGGGACGA	3284	+1 a	5.26%
GTCGGACTTGACCGTCA-GGGGGACGA	2042	-1	3.27%
GTCGGACTTGACCGTCAtTGGGGGACGA	528	+1 t	0.85%
GTCGGACTTGACCGTC-TGGGGGACGA	503	-1	0.81%
GTCGGACTTGACCGTCAT-GGGGACGA	215	-1	0.34%
GTCGGACTTGACCGTCA--GGGGACGA	177	-2	0.28%
GTCGGACTTGACC---------GACGA	99	-9	0.16%
GTCGGACTTGACCGTCAgTGGGGGACGA	91	+1 g	0.15%
GTC------------------GGACGA	60	-18	0.10%

YW-W9-KO-12h-Syn100crBCL11A9b 12h	Ins	Del	HDR
1586 | 17.21%	10.47%	6.75%	0.00%
**Typical seqs**	**Reads**	**Type**	**pct.**
GTCGGACTTGACCGTCA|TGG**GGG**ACGA	1313	WT	82.79%
GTCGGACTTGACCGTCAaTGGGGGACGA	138	+1 a	8.70%
GTCGGACTTGACCGTCA-GGGGGACGA	66	-1	4.16%
GTCGGACTTGACCGTCAtTGGGGGACGA	15	+1 t	0.95%
GTCGGACTTGACCGTCAgTGGGGGACGA	10	+1 g	0.63%
GTCGGACTT-------------GACGA	9	-13	0.57%
GTCGGACTTGACCGTC-TGGGGGACGA	7	-1	0.44%
GTCGGACTTGACCGTCA---GGGACGA	6	-3	0.38%
GTCGGACTTGACCGTCA--GGGGACGA	6	-2	0.38%
GTCGGACTTGACCGTCAT-GGGGACGA	4	-1	0.25%

YW-W9-KO-24h-Syn100crBCL11A9b 24h	Ins	Del	HDR
42825 | 36.67%	21.75%	14.92%	0.00%
**Typical seqs**	**Reads**	**Type**	**pct.**
GTCGGACTTGACCGTCA|TGG**GGG**ACGA	27122	WT	63.33%
GTCGGACTTGACCGTCAaTGGGGGACGA	6348	+1 a	14.82%
GTCGGACTTGACCGTCA-GGGGGACGA	2259	-1	5.27%
GTCGGACTTGACCGTCAtTGGGGGACGA	1264	+1 t	2.95%
GTCGGACTTGACCGTC-TGGGGGACGA	570	-1	1.33%
GTCGGACTTGACCGTCA--GGGGACGA	554	-2	1.29%
GTCGGACTTGACCGTCAgTGGGGGACGA	407	+1 g	0.95%
GTCGGACTT-------------GACGA	239	-13	0.56%
GTCGGACTTGACCG---TGGGGGACGA	199	-3	0.46%
GTCGGACT---------TGGGGGACGA	180	-9	0.42%

YW-W9-KO-48h-Syn100crBCL11A9b 48h	Ins	Del	HDR
24529 | 35.27%	21.22%	14.05%	0.00%
**Typical seqs**	**Reads**	**Type**	**pct.**
GTCGGACTTGACCGTCA|TGG**GGG**ACGA	15878	WT	64.73%
GTCGGACTTGACCGTCAaTGGGGGACGA	3497	+1 a	14.26%
GTCGGACTTGACCGTCA-GGGGGACGA	1228	-1	5.01%
GTCGGACTTGACCGTCAtTGGGGGACGA	778	+1 t	3.17%
GTCGGACTTGACCGTC-TGGGGGACGA	242	-1	0.99%
GTCGGACTTGACCGTCA--GGGGACGA	230	-2	0.94%
GTCGGACTT-------------GACGA	179	-13	0.73%
GTCGGACTTGACCGTCAgTGGGGGACGA	155	+1 g	0.63%
GTC------------------GGACGA	119	-18	0.49%
GTCGGACTTGACC---------GACGA	106	-9	0.43%

P138-P142-YW-W9-Syn101crBCL11A9c-KO

YW-W9-KO-4h-Syn101crBCL11A9c 4h	Ins	Del	HDR
40621 | 6.47%	5.94%	0.53%	0.00%
**Typical seqs**	**Reads**	**Type**	**pct.**
GAGTACACGTTCTCCGT|GTT**GGG**CATC	37991	WT	93.53%
GAGTACACGTTCTCCGTtGTTGGGCATC	2174	+1 t	5.35%
GAGTACACGTTCTCC--GTTGGGCATC	91	-2	0.22%
GAGTACACGTTCTCC-TGTTGGGCATC	33	-1	0.08%
GAGTACACGTTCTCCGTGTT-GGCATC	20	-1	0.05%
GAGTACACGCTCTCCGTTGTTGGGCATC	18	+1 Ins	0.04%
GAGTACACGTTCTCCGTcGTTGGGCATC	17	+1 c	0.04%
GGGTACACGTTCTCCGTTGTTGGGCATC	17	+1 Ins	0.04%
GAGTACGCGTTCTCCGTTGTTGGGCATC	15	+1 Ins	0.04%
GAGTACACGTTCTCCGTtGCTGGGCATC	12	+1 t	0.03%

YW-W9-KO-8h-Syn101crBCL11A9c 8h	Ins	Del	HDR
9837 | 18.06%	15.52%	2.54%	0.00%
**Typical seqs**	**Reads**	**Type**	**pct.**
GAGTACACGTTCTCCGT|GTT**GGG**CATC	8060	WT	81.94%
GAGTACACGTTCTCCGTtGTTGGGCATC	1422	+1 t	14.46%
GAGTACACGTTCTCC--GTTGGGCATC	100	-2	1.02%
GAGTACAC---------GTTGGGCATC	31	-9	0.32%
GAGTACACGT---------------TC	17	-15	0.17%
GAGTACACGTTCTCCGTTTTGTTGGGCATC	17	+3	0.17%
GAGTACAC--------TGCCTGGGCATC	16	-7	0.16%
GAGTACACG----------CGAGCATC	14	-10	0.14%
GAGTACACGTTCTCCGTGTT-GGCATC	13	-1	0.13%
GAGTACACGTTCTCCG---TGGGCATC	11	-3	0.11%

YW-W9-KO-12h-Syn101crBCL11A9c 12h	Ins	Del	HDR
25485 | 21.57%	17.45%	4.12%	0.00%
**Typical seqs**	**Reads**	**Type**	**pct.**
GAGTACACGTTCTCCGT|GTT**GGG**CATC	19987	WT	78.43%
GAGTACACGTTCTCCGTtGTTGGGCATC	3951	+1 t	15.50%
GAGTACACGTTCTCC-TGTTGGGCATC	220	-1	0.86%
GAGTACACGTTCTCC--GTTGGGCATC	167	-2	0.66%
GAGTACACGTTCTCCG---TGGGCATC	81	-3	0.32%
GAGTACAC---------GTTGGGCATC	76	-9	0.30%
GAGTACACGTTCTCCGT-TTGGGCATC	64	-1	0.25%
GAGTACACGTTCTCCG-GTTGGGCATC	62	-1	0.24%
GAGTACACGTTCTC--TGTTGGGCATC	47	-2	0.18%
GAGTACA-------CGTGTTGGGCATC	35	-7	0.14%

YW-W9-KO-24h-Syn101crBCL11A9c 24h	Ins	Del	HDR
23786 | 26.93%	21.03%	5.89%	0.00%
**Typical seqs**	**Reads**	**Type**	**pct.**
GAGTACACGTTCTCCGT|GTT**GGG**CATC	17381	WT	73.07%
GAGTACACGTTCTCCGTtGTTGGGCATC	4467	+1 t	18.78%
GAGTACACGTTCTCC--GTTGGGCATC	253	-2	1.06%
GAGTACACGTTCTCC-TGTTGGGCATC	172	-1	0.72%
GAGTACAC---------GTTGGGCATC	136	-9	0.57%
GAGTACACGTTCTCCGT-TTGGGCATC	66	-1	0.28%
GAGTACACGTTCTC------------C	58	-12	0.24%
GAGTACACGTTCTCCG-GTTGGGCATC	49	-1	0.21%
GAGTACACGTTCTCCG---TGGGCATC	48	-3	0.20%
GAGTACGCGTTCTCCGTTGTTGGGCATC	38	+1 Ins	0.16%

YW-W9-KO-48h-Syn101crBCL11A9c 48h	Ins	Del	HDR
36382 | 24.81%	18.76%	6.05%	0.00%
**Typical seqs**	**Reads**	**Type**	**pct.**
GAGTACACGTTCTCCGT|GTT**GGG**CATC	27356	WT	75.19%
GAGTACACGTTCTCCGTtGTTGGGCATC	6097	+1 t	16.76%
GAGTACACGTTCTCC--GTTGGGCATC	708	-2	1.95%
GAGTACACGTTCTCCG-GTTGGGCATC	235	-1	0.65%
GAGTACACGTTCTCC-TGTTGGGCATC	208	-1	0.57%
GAGTACAC---------GTTGGGCATC	181	-9	0.50%
GAGTACACGTTCT--------------	113	-18	0.31%
GAGTACACGTTC----TGTTGGCATC	97	-4	0.27%
GAGTACACGTTCTCCG---TGGGCATC	78	-3	0.21%
GAGTACACGTTCTCCGTtGTTGGGCGTC	45	+1 t	0.12%

P138-P142-YW-W9-Syn105crBCL11A9g-KO

YW-W9-KO-4h-Syn105crBCL11A9g 31175 | 8.28% 4h	Ins 7.94%	Del 0.33%	HDR 0.00%
**Typical seqs**	**Reads**	**Type**	**pct.**
CAACTTACAAATACCCT|GCG**GGG**CATA	28595	WT	91.72%
CAACTTACAAATACCCTtGCGGGGCATA	2261	+1 t	7.25%
CAACTTACAAATACCCTCTGCGGGGCATA	25	+2	0.08%
CAACTTACAAATACCCTGC-GGGCATA	24	-1	0.08%
CAACTTACAAATA--CTGCGGGGCATA	21	-2	0.07%
CAACTTACAAATACCCCTGCGGGGCATA	15	+1 Ins	0.05%
CAACTTACAAATACCCTcGCGGGGCATA	10	+1 c	0.03%
CAACTTACAAATACCCTtGCGGGGCATG	10	+1 t	0.03%
CGACTTACAAATACCCTTGCGGGGCATA	10	+1 Ins	0.03%
CAACTTACAAATACCCTCCTGCGGGGCATA	9	+3	0.03%

YW-W9-KO-8h-Syn105crBCL11A9g 38952 | 42.33% 8h	Ins 39.82%	Del 2.50%	HDR 0.00%
**Typical seqs**	**Reads**	**Type**	**pct.**
CAACTTACAAATACCCT|GCG**GGG**CATA	22465	WT	57.67%
CAACTTACAAATACCCTtGCGGGGCATA	13628	+1 t	34.99%
CAACTTACAAATA-CCTGCGGGGCATA	435	-1	1.12%
CAACTTACAAATACCCTCTGCGGGGCATA	178	+2	0.46%
CAACTTACAAATACCCTATGCGGGGCATA	164	+2	0.42%
CAACTTACAAATACCC-GCGGGGCATA	143	-1	0.37%
CAACTTACAAATACCCTGTGCGGGGCATA	120	+2	0.31%
CAACTTACAAATACCCTCCTGCGGGGCATA	90	+3	0.23%
CAACTTACAAATACCCCTGCGGGGCATA	88	+1 Ins	0.23%
CAACTTACGAATACCCTTGCGGGGCATA	85	+1 Ins	0.22%

YW-W9-KO-12h-Syn105crBCL11A9g 24115 | 54.29% 12h	Ins 49.82%	Del 4.47%	HDR 0.00%
**Typical seqs**	**Reads**	**Type**	**pct.**
CAACTTACAAATACCCT|GCG**GGG**CATA	11024	WT	45.71%
CAACTTACAAATACCCTtGCGGGGCATA	10698	+1 t	44.36%
CAACTTACAAATA--CTGCGGGGCATA	254	-2	1.05%
CAACTTACAAATACCC-GCGGGGCATA	234	-1	0.97%
CAACTTACAAATA---TGCGGGGCATA	146	-3	0.61%
CAACTTACAAATA-CCTGCGGGGCATA	144	-1	0.60%
CAACTTACAAATACCCTCCTGCGGGGCATA	108	+3	0.45%
CAACTTACAAATACCCCTGCGGGGCATA	91	+1 Ins	0.38%
CAACTTACAAATACCCTTGTGCGGGGCATA	60	+3	0.25%
CAACCTACAAATACCCTTGCGGGGCATA	56	+1 Ins	0.23%

YW-W9-KO-24h-Syn105crBCL11A9g 16224 | 70.35% 24h	Ins 65.21%	Del 5.14%	HDR 0.00%
**Typical seqs**	**Reads**	**Type**	**pct.**
CAACTTACAAATACCCT|GCG**GGG**CATA	4810	WT	29.65%
CAACTTACAAATACCCTtGCGGGGCATA	9685	+1 t	59.70%
CAACTTACAAATACCC-GCGGGGCATA	180	-1	1.11%
CAACTTACAAATA-CCTGCGGGGCATA	120	-1	0.74%
CAACTTA----------------CATA	108	-16	0.67%
CAACTTACAAATAC---GCGGGGCATA	97	-3	0.60%
CAACTTACAA--------------ATA	82	-14	0.51%
CAACTTACAAATA--CTGCGGGGCATA	70	-2	0.43%
CAACTTACAAATACCCTTTGCGGGGCATA	73	+2	0.45%
CAACTTACAAATACCCCTGCGGGGCATA	63	+1 Ins	0.39%

YW-W9-KO-48h-Syn105crBCL11A9g 14446 | 65.14% 48h	Ins 59.01%	Del 6.13%	HDR 0.00%
**Typical seqs**	**Reads**	**Type**	**pct.**
CAACTTACAAATACCCT|GCG**GGG**CATA	5036	WT	34.86%
CAACTTACAAATACCCTtGCGGGGCATA	7519	+1 t	52.05%
CAACTTACAAATA-CCTGCGGGGCATA	236	-1	1.63%
CAACTTACAAATA--CTGCGGGGCATA	111	-2	0.77%
CAACTTACAAATACCCTTTGCGGGGCATA	68	+2	0.47%
CAACTTACAAATACCCTCTGCGGGGCATA	59	+2	0.41%
CAACTTACAAATACCC-GCGGGGCATA	55	-1	0.38%
CAACTTACAAATACCCTtGCGGGGCGTA	53	+1 t	0.37%
CAACTTACAAATACCCTATGCGGGGCATA	43	+2	0.30%
CAACTTACAAATACCCTcGCGGGGCATA	42	+1 c	0.29%

P138-P142-YW-W9-Syn106crBCL11A10-KO

YW-W9-KO-4h-Syn106crBCL11A10a 4h	Ins	Del	HDR
72109 | 0.78%	0.32%	0.46%	0.00%
**Typical seqs**	**Reads**	**Type**	**pct.**
GCGCTAAGTCACTGTCG|TCG**AGG**CCCC	71543	WT	99.22%
GCGCTAAGTCACTGTCGgTCGAGGCCCC	119	+1 g	0.17%
GCGCTAAGTCACT---GTCGAGGCCCC	109	-3	0.15%
GCGCTAAGTCACTGTC-TCGAGGCCCC	70	-1	0.10%
GCGCTAAGTCACTGTCGtTCGAGGCCCC	52	+1 t	0.07%
GCGCTAAGTCACTGT--TCGAGGCCCC	35	-2	0.05%
GCGCTAAGTCACTGTCGcTCGAGGCCCC	29	+1 c	0.04%
GCGCTAAGTCACTGTCGCGTCGAGGCCCC	16	+2	0.02%
GCGCTAAGTCACTGTCG-CGAGGCCCC	16	-1	0.02%
GCGCTAAGTCACTGTCGaTCGAGGCCCC	12	+1 a	0.02%

YW-W9-KO-8h-Syn106crBCL11A10a 8h	Ins	Del	HDR
38875 | 9.76%	5.38%	4.38%	0.00%
**Typical seqs**	**Reads**	**Type**	**pct.**
GCGCTAAGTCACTGTCG|TCG**AGG**CCCC	35080	WT	90.24%
GCGCTAAGTCACTGTCGgTCGAGGCCCC	1507	+1 g	3.88%
GCGCTAAGTCACT---GTCGAGGCCCC	768	-3	1.98%
GCGCTAAGTCACTGTC-TCGAGGCCCC	533	-1	1.37%
GCGCTAAGTCACTGTCGtTCGAGGCCCC	127	+1 t	0.33%
GCGCTAAGTCACTGTCGCGTCGAGGCCCC	131	+2	0.34%
GCGCTAA---------GTCGAGGCCCC	78	-9	0.20%
GCGCTAAGTCACTGTCGcTCGAGGCCCC	80	+1 c	0.21%
GCGCTAAGTCACTGTCGaTCGAGGCCCC	74	+1 a	0.19%
GCGCTAAGTCACTGTCG-CGAGGCCCC	44	-1	0.11%

YW-W9-KO-12h-Syn106crBCL11A10a 12h	Ins	Del	HDR
17935 | 15.09%	7.89%	7.20%	0.00%
**Typical seqs**	**Reads**	**Type**	**pct.**
GCGCTAAGTCACTGTCG|TCG**AGG**CCCC	15229	WT	84.91%
GCGCTAAGTCACTGTCGgTCGAGGCCCC	1023	+1 g	5.70%
GCGCTAAGTCACT---GTCGAGGCCCC	539	-3	3.01%
GCGCTAAGTCACTGTC-TCGAGGCCCC	265	-1	1.48%
GCGCTAAGTCACTGTCGtTCGAGGCCCC	112	+1 t	0.62%
GCGCTAAGTCACTGTCGCGTCGAGGCCCC	85	+2	0.47%
GCGCTAA---------GTCGAGGCCCC	65	-9	0.36%
GCGCTAAGTCACTGTCG-CGAGGCCCC	42	-1	0.23%
GCGCTAAGTCACTGTCGcTCGAGGCCCC	40	+1 c	0.22%
GCGCTAAGTCACTGTCGaTCGAGGCCCC	27	+1 a	0.15%

YW-W9-KO-24h-Syn106crBCL11A10a 24h	Ins	Del	HDR
41315 | 18.59%	7.89%	10.71%	0.00%
**Typical seqs**	**Reads**	**Type**	**pct.**
GCGCTAAGTCACTGTCG|TCG**AGG**CCCC	33633	WT	81.41%
GCGCTAAGTCACTGTCGgTCGAGGCCCC	2161	+1 g	5.23%
GCGCTAAGTCACT---GTCGAGGCCCC	1936	-3	4.69%
GCGCTAAGTCACTGTC-TCGAGGCCCC	661	-1	1.60%
GCGCTAAGTCACTGTCGtTCGAGGCCCC	281	+1 t	0.68%
GCGCTAA---------GTCGAGGCCCC	154	-9	0.37%
GCGCTAAGTCA---------------C	142	-15	0.34%
GCGCTAAGTCACTGTCGCGTCGAGGCCCC	140	+2	0.34%
GCGCTAAGTCACTGTCGcTCGAGGCCCC	117	+1 c	0.28%
GCGCTAA----------TCGAGGCCCC	97	-10	0.23%

YW-W9-KO-48h-Syn106crBCL11A10a 48h	Ins	Del	HDR
32004 | 20.98%	8.17%	12.81%	0.00%
**Typical seqs**	**Reads**	**Type**	**pct.**
GCGCTAAGTCACTGTCG|TCG**AGG**CCCC	25288	WT	79.02%
GCGCTAAGTCACT---GTCGAGGCCCC	1987	-3	6.21%
GCGCTAAGTCACTGTCGgTCGAGGCCCC	1708	+1 g	5.34%
GCGCTAAGTCACTGTC-TCGAGGCCCC	584	-1	1.82%
GCGCTAAGTCACTGTCGtTCGAGGCCCC	255	+1 t	0.80%
GCGCTAA---------GTCGAGGCCCC	199	-9	0.62%
GCGCTAAGTCACTGTCGCGTCGAGGCCCC	147	+2	0.46%
GCGCTAAGTCACTGTCGaTCGAGGCCCC	138	+1 a	0.43%
GCGCTAAGTCACTGTCG-CGAGGCCCC	126	-1	0.39%
GCGCTAAGTCA---------------C	112	-15	0.35%

P138-P142-YW-W9-Syn107crBCL11A10-KO

YW-W9-KO-4h-Syn107crBCL11A10b	Ins	Del	HDR
64235 | 0.06% 4h	**0.01%**	**0.06%**	**0.00%**
**Typical seqs**	**Reads**	**Type**	**pct.**
GACTATCATACAATTGG|GCA**GGG**GTGG	64195	WT	99.94%
GACTATCATACAATT-GGCAGGGGTGG	20	-1	0.03%
GACTATCATACAATT--GCAGGGGTGG	4	-2	0.01%
GACTATCATACAATTGGgGCAGGGGTGG	4	+1 g	0.01%
GACTATCATACGATTGGGCAGGGGT-G	3	-1	0.00%
YW-W9-KO-4h-Syn107crBCL11A10b	Ins	Del	HDR
64235 | 0.06%	**0.01%**	**0.06%**	**0.00%**
**Typical seqs**	**Reads**	**Type**	**pct.**
GACTATCATACAATTGG|GCA**GGG**GTGG	64195	WT	99.94%
GACTATCATACAATT-GGCAGGGGTGG	20	-1	0.03%

YW-W9-KO-8h-Syn107crBCL11A10b	Ins	Del	HDR
61378 | 6.47% 8h	**2.01%**	**4.47%**	**0.00%**
**Typical seqs**	**Reads**	**Type**	**pct.**
GACTATCATACAATTGG|GCA**GGG**GTGG	57406	WT	93.53%
GACTATCATACAATT-GGCAGGGGTGG	1811	-1	2.95%
GACTATCATACAATTGGgGCAGGGGTGG	1053	+1 g	1.72%
GACTATCATACAATT--GCAGGGGTGG	74	-2	0.12%
GACTATCATACAATT-------GGTGG	69	-7	0.11%
GACTATCATACAATT------------	68	-12	0.11%
GACTATCATA--------CAGGGGTGG	71	-8	0.12%
GACTATCATACAATT-----------G	47	-11	0.08%
GACTATCATACAATT-----GGGGTGG	40	-5	0.07%
GACTATCATACAATT------GGGTGG	36	-6	0.06%

YW-W9-KO-12h-Syn107crBCL11A10b	Ins	Del	HDR
3356 | 14.69% 12h	**0.77%**	**13.92%**	**0.00%**
**Typical seqs**	**Reads**	**Type**	**pct.**
GACTATCATACAATTGG|GCA**GGG**GTGG	2863	WT	85.31%
GACTATCATACAATT-GGCAGGGGTGG	317	-1	9.45%
GACTATCATACAAT----------TGG	50	-10	1.49%
----------------------GGTGG	12	-23	0.36%
GACTATCATA----------------G	11	-16	0.33%
GACTATCATACAATTGGgGCAGGGGTGG	20	+1 g	0.60%
GACTAT--------------------G	9	-20	0.27%
GACTATCATACAATTG---------GG	7	-9	0.21%
---------------------GGGTGG	7	-22	0.21%
GACTATCATACAATT--------GTGG	7	-8	0.21%

YW-W9-KO-24h-Syn107crBCL11A10b	Ins	Del	HDR
38263 | 15.29% 24h	**3.57%**	**11.72%**	**0.00%**
**Typical seqs**	**Reads**	**Type**	**pct.**
GACTATCATACAATTGG|GCA**GGG**GTGG	32411	WT	84.71%
GACTATCATACAATT-GGCAGGGGTGG	2367	-1	6.19%
GACTATCATACAATTGGgGCAGGGGTGG	1091	+1 g	2.85%
GACTATCATACAAT----------TGG	183	-10	0.48%
GACTATCATACAATT-----------G	101	-11	0.26%
GACTATCATACAATT------------	222	-14	0.58%
GACTATCATA--------CAGGGGTGG	76	-8	0.20%
GACTATCATACAATT-------GGTGG	69	-7	0.18%
GACTATCATACAATT--GCAGGGGTGG	66	-2	0.17%
GACTATCATACAATT------------	222	-15	0.58%

YW-W9-KO-48h-Syn107crBCL11A10b	Ins	Del	HDR
27503 | 15.12% 48h	**3.29%**	**11.83%**	**0.00%**
**Typical seqs**	**Reads**	**Type**	**pct.**
GACTATCATACAATTGG|GCA**GGG**GTGG	23345	WT	84.88%
GACTATCATACAATT-GGCAGGGGTGG	1461	-1	5.31%
GACTATCATACAATTGGgGCAGGGGTGG	728	+1 g	2.65%
GACTATCATACAAT----------TGG	163	-10	0.59%
GACTATCATACAATT--GCAGGGGTGG	85	-2	0.31%
GACTATCATACAATT------GGGTGG	78	-6	0.28%
GACTATCATA--------CAGGGGTGG	52	-8	0.19%
GACTATCATACAATT-----------G	50	-11	0.18%
GACTATCATACAATT-------GGTGG	46	-7	0.17%
GACTATCATACAATT--------GTGG	41	-8	0.15%

P138-P142-YW-W9-Syn108crBCL11A10-KO

YW-W9-KO-4h-Syn108crBCL11A10c 4h	Ins	Del	HDR
48197 | 6.68%	6.06%	0.62%	0.00%
**Typical seqs**	**Reads**	**Type**	**pct.**
GTGGGAACTTTGCCGAT|ATG**AGG**ATGG	44977	WT	93.32%
GTGGGAACTTTGCCGATtATGAGGATGG	2727	+1 t	5.66%
GTGGGAACTTTGCCG--ATGAGGATGG	64	-2	0.13%
GTGGGAACTTTG----TATGAGGATGG	51	-4	0.11%
GTGGGAACTTTGCCG-TATGAGGATGG	47	-1	0.10%
GTGGGAACTTTGC---TATGAGGATGG	26	-3	0.05%
GTGGGAACTTTGCCGACTATGAGGATGG	17	+1 Ins	0.04%
GTGGGAACTTTGCC--TATGAGGATGG	17	-2	0.04%
GTGGGAGCTTTGCCGATTATGAGGATGG	16	+1 Ins	0.03%
GTGGGAACTTTGCC---ATGAGGATGG	13	-3	0.03%

YW-W9-KO-8h-Syn108crBCL11A10c 8h	Ins	Del	HDR
46342 | 14.54%	11.90%	2.63%	0.00%
**Typical seqs**	**Reads**	**Type**	**pct.**
GTGGGAACTTTGCCGAT|ATG**AGG**ATGG	39605	WT	85.46%
GTGGGAACTTTGCCGATtATGAGGATGG	5054	+1 t	10.91%
GTGGGAACTTTGCCG-TATGAGGATGG	412	-1	0.89%
GTGGGAACTTTGCCG--ATGAGGATGG	165	-2	0.36%
GTGGGAACTTTGCC-----GAGGATGG	100	-5	0.22%
GTGGGAACTTTGCC--TATGAGGATGG	102	-2	0.22%
GTGGGAACTTTGCCGA-ATGAGGATGG	86	-1	0.19%
GTGGGAACTTTGCC-------------	77	-13	0.17%
GTGGGAACTTTGCC--------GATGG	46	-8	0.10%
GTGGGAACTTTGCCGAT-TGAGGATGG	45	-1	0.10%

YW-W9-KO-12h-Syn108crBCL11A10c 12h	Ins	Del	HDR
9271 | 25.40%	20.08%	5.32%	0.00%
**Typical seqs**	**Reads**	**Type**	**pct.**
GTGGGAACTTTGCCGAT|ATG**AGG**ATGG	6916	WT	74.60%
GTGGGAACTTTGCCGATtATGAGGATGG	1673	+1 t	18.05%
GTGGGAACTTTGCCG-TATGAGGATGG	168	-1	1.81%
GTGGGAACTTTGCCG--ATGAGGATGG	88	-2	0.95%
GTGGGAACTTTGCC--TATGAGGATGG	48	-2	0.52%
GTGGGAACTTTGCC-----GAGGATGG	34	-5	0.37%
GTGGGAACTTTGCC---ATGAGGATGG	29	-3	0.31%
GTGGGAACTTTGCCGATcATGAGGATGG	19	+1 c	0.20%
GTGGGAACTTTGCCGATtATGGGGATGG	14	+1 t	0.15%
GTGGGAACTT--------TGAGGATGG	12	-8	0.13%

YW-W9-KO-24h-Syn108crBCL11A10c 24h	Ins	Del	HDR
27164 | 39.50%	30.42%	9.08%	0.00%
**Typical seqs**	**Reads**	**Type**	**pct.**
GTGGGAACTTTGCCGAT|ATG**AGG**ATGG	16435	WT	60.50%
GTGGGAACTTTGCCGATtATGAGGATGG	7213	+1 t	26.55%
GTGGGAACTTTGCCG-TATGAGGATGG	688	-1	2.53%
GTGGGAACTTTGCCG--ATGAGGATGG	458	-2	1.69%
GTGGGAACTTTGCCGA-ATGAGGATGG	198	-1	0.73%
GTGGGAACTTTGCC-----GAGGATGG	107	-5	0.39%
GTGGGAACTTTGCC-------------	106	-13	0.39%
GTGGGAACTTTGCC--------GATGG	91	-8	0.34%
GTGGGAACTTTGCC--TATGAGGATGG	87	-2	0.32%
GTGGGAACTTTGCCGATtATGGGGATGG	68	+1 t	0.25%

YW-W9-KO-48h-Syn108crBCL11A10c 48h	Ins	Del	HDR
19221 | 40.86%	30.65%	10.21%	0.00%
**Typical seqs**	**Reads**	**Type**	**pct.**
GTGGGAACTTTGCCGAT|ATG**AGG**ATGG	11368	WT	59.14%
GTGGGAACTTTGCCGATtATGAGGATGG	5191	+1 t	27.01%
GTGGGAACTTTGCCG-TATGAGGATGG	368	-1	1.91%
GTGGGAACTTTGCCG--ATGAGGATGG	347	-2	1.81%
GTGGGAACTTTGCC-----GAGGATGG	141	-5	0.73%
GTGGGAACTTTGCC--TATGAGGATGG	107	-2	0.56%
GTGGGAACTTTGCCGA-ATGAGGATGG	76	-1	0.40%
GTGGGAACTTTGCCGAT---------G	59	-9	0.31%
GTGGGAACTTTGCC--------GATGG	63	-8	0.33%
GTGGGAACTTTGCC-------------	80	-13	0.42%

P148-P152-YW-W9-RNP-Syn104BCL11A-KO

4h

Total	INDEL	Ins	Del	HDR
2506	1.16%	0.16%	1.00%	0.00%

Typical seqs	Reads	Type	pct.
CATATTAGTGGTCCGGG|CCC**GGG**CAGG	2477	WT	98.84%
CATATTAGTGGT------CCGGGCAGG	12	-6	0.48%
CATATTAGTGGTCC-GGCCCGGGCAGG	5	-1	0.20%
CATATTAGTGGTCCGGGgCCCGGGCAGG	4	+1 g	0.16%
-----------------CCCGGGCAGG	3	-19	0.12%

8h

Total	INDEL	Ins	Del	HDR
16020	5.61%	0.84%	4.77%	0.00%

Typical seqs	Reads	Type	pct.
CATATTAGTGGTCCGGG|CCC**GGG**CAGG	15122	WT	94.39%
CATATTAGTGGT------CCGGGCAGG	344	-6	2.15%
CATATTAGTGGTCCGGGgCCCGGGCAGG	100	+1 g	0.62%
CATATTAGTGGTCC-GGCCCGGGCAGG	92	-1	0.57%
CATATTAGTGGTCCGGG-CCGGGCAGG	86	-1	0.54%
CATATTAGTGGTCCGGG--CGGGCAGG	37	-2	0.23%
CATATTAGTGGTCC--GCCCGGGCAGG	20	-2	0.12%
CATATTAGT------GGCCCGGGCAGG	14	-6	0.09%
CATATTAGTGGTCCGG----GGGCAGG	17	-4	0.11%
-----------------CCCGGGCAGG	21	-18	0.13%

12h

Total	INDEL	Ins	Del	HDR
5660	15.57%	1.40%	14.17%	0.00%

Typical seqs	Reads	Type	pct.
CATATTAGTGGTCCGGG|CCC**GGG**CAGG	4779	WT	84.43%
CATATTAGTGGT------CCGGGCAGG	358	-6	6.33%
CATATTAGTGGTCCGGG-CCGGGCAGG	109	-1	1.93%
CATATTAGTGGTCC-GGCCCGGGCAGG	60	-1	1.06%
CATATTAGTGGTCCGGGgCCCGGGCAGG	63	+1 g	1.11%
CATATTAGTGGTCCGGG--CGGGCAGG	28	-2	0.49%
CATATTAGT------GGCCCGGGCAGG	25	-6	0.44%
CATATTAGTGGTCCG----------GG	23	-10	0.41%
CATATTAGTGGT-----CCCGGGCAGG	18	-5	0.32%
CATATTAGTGGTCCGGGtCCCGGGCAGG	13	+1 t	0.23%

24h

Total	INDEL	Ins	Del	HDR
3014	41.80%	2.75%	39.05%	0.00%

Typical seqs	Reads	Type	pct.
CATATTAGTGGTCCGGG|CCC**GGG**CAGG	1754	WT	58.20%
CATATTAGTGGT------CCGGGCAGG	571	-6	18.94%
CATATTAGTGGTCCGGG-CCGGGCAGG	105	-1	3.48%
CATATTAGTGGTCC-GGCCCGGGCAGG	82	-1	2.72%
CATATTAGTGGTCCGGGgCCCGGGCAGG	57	+1 g	1.89%
CATATTAGTGGTCCGGG--CGGGCAGG	47	-2	1.56%
CATATTAGT------GGCCCGGGCAGG	40	-6	1.33%
CATATTAGTGGTCCG----------GG	34	-10	1.13%
CATATTAGTGGTCCGGG---GGGCAGG	28	-3	0.93%
CATATTAGTGGT-----CCCGGGCAGG	26	-5	0.86%

48h

Total	INDEL	Ins	Del	HDR
18638	46.95%	2.36%	44.59%	0.00%

Typical seqs	Reads	Type	pct.
CATATTAGTGGTCCGGG|CCC**GGG**CAGG	9888	WT	53.05%
CATATTAGTGGT------CCGGGCAGG	4429	-6	23.76%
CATATTAGTGGTCC-GGCCCGGGCAGG	413	-1	2.22%
CATATTAGTGGTCCGGG-CCGGGCAGG	368	-1	1.97%
CATATTAGT------GGCCCGGGCAGG	320	-6	1.72%
CATATTAGTGGTCCG----------GG	184	-10	0.99%
CATATTAGTGGTCCGGGgCCCGGGCAGG	150	+1 g	0.80%
CATATTAGTGGTCCGGG--CGGGCAGG	136	-2	0.73%
CATATTAGTGGTCCG-----GGGCAGG	118	-5	0.63%
CATATTAGTGGTCCGG----GGGCAGG	118	-4	0.63%

P158-P162-YW-W9-Syn85BCL11A-KO

4h

Total | INDEL	Ins	Del	HDR
86888 | 20.47%	1.86%	18.61%	0.00%

Typical seqs	Reads	Type	pct.
TGGTCGCCTACGGAATC|TAC**AGG**AATC	69104	WT	79.53%
TGGTCGCCTAC----------GGAATC	5372	-10	6.18%
TGGTCGCCTACGGAATC-ACAGGAATC	2921	-1	3.36%
TGGTCGC---------CTACAGGAATC	2108	-9	2.43%
TGGTCGCCTACGGAA--TACAGGAATC	2165	-2	2.49%
TGGTCGCCTACGGAAT-TACAGGAATC	1172	-1	1.35%
TGGTCGCCTACGGAATCcTACAGGAATC	620	+1 c	0.71%
TGGTCGCCTACGGA---TACAGGAATC	235	-3	0.27%
TGGTCGCCTACGGAATCtTACAGGAATC	220	+1 t	0.25%
TGGTCGCCTACGGAATCgTACAGGAATC	178	+1 g	0.20%

8h

Total | INDEL	Ins	Del	HDR
77076 | 39.89%	2.75%	37.14%	0.00%

Typical seqs	Reads	Type	pct.
TGGTCGCCTACGGAATC|TAC**AGG**AATC	46333	WT	60.11%
TGGTCGCCTAC----------GGAATC	10601	-10	13.75%
TGGTCGC---------CTACAGGAATC	5276	-9	6.85%
TGGTCGCCTACGGAATC-ACAGGAATC	3802	-1	4.93%
TGGTCGCCTACGGAA--TACAGGAATC	2282	-2	2.96%
TGGTCGCCTACGGAAT-TACAGGAATC	1188	-1	1.54%
TGGTCGCCTACGGAATCcTACAGGAATC	1086	+1 c	1.41%
TGGTCGCCTACGGA---TACAGGAATC	548	-3	0.71%
TGGTCGCCTACGGAATC--CAGGAATC	407	-2	0.53%
TGGTCGCCTACGGAAT---CAGGAATC	364	-3	0.47%

12h

Total | INDEL	Ins	Del	HDR
249176 | 47.24%	3.31%	43.93%	0.00%

Typical seqs	Reads	Type	pct.
TGGTCGCCTACGGAATC|TAC**AGG**AATC	131457	WT	52.76%
TGGTCGCCTAC----------GGAATC	36694	-10	14.73%
TGGTCGC---------CTACAGGAATC	17917	-9	7.19%
TGGTCGCCTACGGAATC-ACAGGAATC	15399	-1	6.18%
TGGTCGCCTACGGAA--TACAGGAATC	8684	-2	3.49%
TGGTCGCCTACGGAAT-TACAGGAATC	6454	-1	2.59%
TGGTCGCCTACGGAATCcTACAGGAATC	3092	+1 c	1.24%
TGGTCGCCTACGGA---TACAGGAATC	1921	-3	0.77%
TGGTCGCCTACGGAATC--CAGGAATC	1783	-2	0.72%
TGGTCGCCTACGGAATCtTACAGGAATC	1819	+1 t	0.73%

24h

Total | INDEL	Ins	Del	HDR
76171 | 65.98%	3.22%	62.75%	0.00%

Typical seqs	Reads	Type	pct.
TGGTCGCCTACGGAATC|TAC**AGG**AATC	25917	WT	34.02%
TGGTCGCCTAC----------GGAATC	18964	-10	24.90%
TGGTCGC---------CTACAGGAATC	10040	-9	13.18%
TGGTCGCCTACGGAATC-ACAGGAATC	4473	-1	5.87%
TGGTCGCCTACGGAA--TACAGGAATC	3127	-2	4.11%
TGGTCGCCTACGGAAT-TACAGGAATC	1889	-1	2.48%
TGGTCGCCTACGGAATCcTACAGGAATC	847	+1 c	1.11%
TGGTCGCCTACGGAATC--CAGGAATC	653	-2	0.86%
TGGTCGCCTACGGAATCtTACAGGAATC	555	+1 t	0.73%
TGGTCGCCTACGGA---TACAGGAATC	419	-3	0.55%

48h

Total | INDEL	Ins	Del	HDR
73959 | 79.27%	3.00%	76.27%	0.00%

Typical seqs	Reads	Type	pct.
TGGTCGCCTACGGAATC|TAC**AGG**AATC	15332	WT	20.73%
TGGTCGCCTAC----------GGAATC	23710	-10	32.06%
TGGTCGC---------CTACAGGAATC	15196	-9	20.55%
TGGTCGCCTACGGAATC-ACAGGAATC	3876	-1	5.24%
TGGTCGCCTACGGAA--TACAGGAATC	2747	-2	3.71%
TGGTCGCCTACGGAAT-TACAGGAATC	1671	-1	2.26%
TGGTCGCCTACGGAATCcTACAGGAATC	692	+1 c	0.94%
TGGTCGCCTACGGAATC----------	818	-11	1.11%
TGGTCGCCTACGGA---TACAGGAATC	481	-3	0.65%
TGGTCGCCTACGGAATC--CAGGAATC	521	-2	0.70%

P158-P162-YW-W9-Syn102BCL11A-KO

4h

Total	INDEL	Ins	Del	HDR
17561	0.26%	0.20%	0.06%	0.00%

Typical seqs	Reads	Type	pct.
TCCGTGTTGGGCATCGC|GGC**CGG**GGGC	17515	WT	99.74%
TCCGTGTTGGGCATCGCcGGCCGGGGGC	17	+1 c	0.10%
TCCGTGTTGGGCAT--CGGCCGGGGGC	7	-2	0.04%
TCCGTGTTGGGCATCGCaGGCCGGGGGC	7	+1 a	0.04%
TCCGTGTTGGGCATCGCgGGCCGGGGGC	5	+1 g	0.03%
TCCGTGTTGGGCATCGCGG-CGGGGGC	4	-1	0.02%
TCCGTGTTGGGCATCGCtGGCCGGGGGC	3	+1 t	0.02%
TCCGTGTTGGGCATCGCTTGGCCGGGGGC	3	+2	0.02%

8h

Total	INDEL	Ins	Del	HDR
15968	13.78%	8.19%	5.60%	0.00%

Typical seqs	Reads	Type	pct.
TCCGTGTTGGGCATCGC|GGC**CGG**GGGC	13767	WT	86.22%
TCCGTGTTGGGCATCGCcGGCCGGGGGC	815	+1 c	5.10%
TCCGTGTTG--------GGCCGGGGGC	187	-8	1.17%
TCCGTGTTGGGCATC---GCCGGGGGC	175	-3	1.10%
TCCGTGTTGGGCATCGCtGGCCGGGGGC	125	+1 t	0.78%
TCCGTGTTGGGCAT--CGGCCGGGGGC	110	-2	0.69%
TCCGTGTTGGGCATCGCgGGCCGGGGGC	96	+1 g	0.60%
TCCGTGTTGGGCATCG-GGCCGGGGGC	92	-1	0.58%
TCCGTGTTGGGCATCGCaGGCCGGGGGC	83	+1 a	0.52%
TCCGTGTTGGGCAT---GGCCGGGGGC	59	-3	0.37%

12h

Total	INDEL	Ins	Del	HDR
63763	19.07%	13.20%	5.87%	0.00%

Typical seqs	Reads	Type	pct.
TCCGTGTTGGGCATCGC|GGC**CGG**GGGC	51602	WT	80.93%
TCCGTGTTGGGCATCGCcGGCCGGGGGC	4977	+1 c	7.81%
TCCGTGTTGGGCATCGCtGGCCGGGGGC	1222	+1 t	1.92%
TCCGTGTTGGGCATC---GCCGGGGGC	858	-3	1.35%
TCCGTGTTGGGCATCGCaGGCCGGGGGC	645	+1 a	1.01%
TCCGTGTTGGGCATCG-GGCCGGGGGC	515	-1	0.81%
TCCGTGTTG--------GGCCGGGGGC	467	-8	0.73%
TCCGTGTTGGGCAT--CGGCCGGGGGC	327	-2	0.51%
TCCGTGTTGGGCATCGCgGGCCGGGGGC	319	+1 g	0.50%
TCCGTGTTGGGCATCGC-GCCGGGGGC	236	-1	0.37%

24h

Total	INDEL	Ins	Del	HDR
37836	29.58%	17.87%	11.72%	0.00%

Typical seqs	Reads	Type	pct.
TCCGTGTTGGGCATCGC|GGC**CGG**GGGC	26643	WT	70.42%
TCCGTGTTGGGCATCGCcGGCCGGGGGC	2835	+1 c	7.49%
TCCGTGTTGGGCATCGCtGGCCGGGGGC	1035	+1 t	2.74%
TCCGTGTTGGGCATCGCaGGCCGGGGGC	798	+1 a	2.11%
TCCGTGTTG--------GGCCGGGGGC	655	-8	1.73%
TCCGTGTTGGGCATC---GCCGGGGGC	645	-3	1.70%
TCCGTGTTGGGCAT--CGGCCGGGGGC	519	-2	1.37%
TCCGTGTTGGGCATCG-GGCCGGGGGC	506	-1	1.34%
TCCGTGTTGGGCATCGCgGGCCGGGGGC	512	+1 g	1.35%
TCCGTGTT---------GGCCGGGGGC	289	-9	0.76%

48h

Total	INDEL	Ins	Del	HDR
29159	29.27%	17.26%	12.00%	0.00%

Typical seqs	Reads	Type	pct.
TCCGTGTTGGGCATCGC|GGC**CGG**GGGC	20625	WT	70.73%
TCCGTGTTGGGCATCGCcGGCCGGGGGC	2264	+1 c	7.76%
TCCGTGTTGGGCATCGCtGGCCGGGGGC	811	+1 t	2.78%
TCCGTGTTGGGCATCGCaGGCCGGGGGC	625	+1 a	2.14%
TCCGTGTTG--------GGCCGGGGGC	513	-8	1.76%
TCCGTGTTGGGCATC---GCCGGGGGC	405	-3	1.39%
TCCGTGTTGGGCAT--CGGCCGGGGGC	388	-2	1.33%
TCCGTGTTGGGCATCGCgGGCCGGGGGC	327	+1 g	1.12%
TCCGTGTT---------GGCCGGGGGC	310	-9	1.06%
TCCGTGTTGGGCATCG-GGCCGGGGGC	280	-1	0.96%

P158-P162-YW-W9-Syn103BCL11A-KO

Total | INDEL	4h	Ins	Del	HDR
10712 | 2.01%		1.30%	0.71%	0.00%
**Typical seqs**		**Reads**	**Type**	**pct.**
ATGACGGTCAAGTCCGA|CGA**CGG**TCTC		10497	WT	97.99%
ATGACGGTCAAGTCCGAaCGACGGTCTC		129	+1 a	1.20%
ATGACGGTCAAGTC---CGACGGTCTC		29	-3	0.27%
ATGACGGTCAAGTCCGA-GACGGTCTC		13	-1	0.12%
ATGACGGTCAAGTCCGA--ACGGTCTC		11	-2	0.10%
ATGACGGTCAAGTCCGAgCGACGGTCTC		6	+1 g	0.06%
ATGACGGTCAAGTCCGAtCGACGGTCTC		4	+1 t	0.04%
ATGACGGTCA------ACGACGGTCTC		4	-6	0.04%
ATGACGGTCAAGTCC--CGACGGTCTC		4	-2	0.04%
ATGACGGTCAAGT----CGACGGTCTC		3	-4	0.03%

Total | INDEL	8h	Ins	Del	HDR
11203 | 7.08%		2.81%	4.27%	0.00%
**Typical seqs**		**Reads**	**Type**	**pct.**
ATGACGGTCAAGTCCGA|CGA**CGG**TCTC		10410	WT	92.92%
ATGACGGTCAAGTCCGAaCGACGGTCTC		212	+1 a	1.89%
ATGACGGTCAAGTC---CGACGGTCTC		127	-3	1.13%
AT----------------GACGGTCTC		103	-16	0.92%
ATGACGGTCAAGTCCGAcCGACGGTCTC		49	+1 c	0.44%
ATGACGGTCAAGTCCG-CGACGGTCTC		46	-1	0.41%
ATGACGGTC-------ACGACGGTCTC		28	-7	0.25%
ATGACGGTCAAG---------------		23	-15	0.21%
ATGACGGTCAAGTCCGA-GACGGTCTC		20	-1	0.18%
ATGACGGTCAAGTCCGAgCGACGGTCTC		18	+1 g	0.16%

Total | INDEL	12h	Ins	Del	HDR
26567 | 12.81%		5.76%	7.05%	0.00%
**Typical seqs**		**Reads**	**Type**	**pct.**
ATGACGGTCAAGTCCGA|CGA**CGG**TCTC		23163	WT	87.19%
ATGACGGTCAAGTCCGAaCGACGGTCTC		965	+1 a	3.63%
ATGACGGTCAAGTC---CGACGGTCTC		575	-3	2.16%
AT----------------GACGGTCTC		196	-16	0.74%
ATGACGGTCAAGTCCGA-GACGGTCTC		184	-1	0.69%
ATGACGGTCAAGTCCG-CGACGGTCTC		137	-1	0.52%
ATGACGGTCAAGTCCGAcCGACGGTCTC		134	+1 c	0.50%
ATGACGGTCAAGTCCGAtCGACGGTCTC		123	+1 t	0.46%
ATGACGGTCAAGTCCGAgCGACGGTCTC		105	+1 g	0.40%
ATGACGGTCAAGT----CGACGGTCTC		58	-4	0.22%

Total | INDEL	24h	Ins	Del	HDR
14287 | 26.28%		10.84%	15.44%	0.00%
**Typical seqs**		**Reads**	**Type**	**pct.**
ATGACGGTCAAGTCCGA|CGA**CGG**TCTC		10533	WT	73.72%
ATGACGGTCAAGTCCGAaCGACGGTCTC		1084	+1 a	7.59%
ATGACGGTCAAGTC---CGACGGTCTC		622	-3	4.35%
AT----------------GACGGTCTC		365	-16	2.55%
ATGACGGTCAAGTCCGAcCGACGGTCTC		122	+1 c	0.85%
ATGACGGTCA------ACGACGGTCTC		101	-6	0.71%
ATGACGGTCAAGTCCGAgCGACGGTCTC		84	+1 g	0.59%
ATGACGGTCAAGTCCGA-GACGGTCTC		82	-1	0.57%
ATGACGGTCAAGTCCG-CGACGGTCTC		74	-1	0.52%
ATGACGGTCAAGT----CGACGGTCTC		62	-4	0.43%

Total | INDEL	48h	Ins	Del	HDR
9745 | 32.32%		9.89%	22.43%	0.00%
**Typical seqs**		**Reads**	**Type**	**pct.**
ATGACGGTCAAGTCCGA|CGA**CGG**TCTC		6595	WT	67.68%
ATGACGGTCAAGTCCGAaCGACGGTCTC		687	+1 a	7.05%
AT----------------GACGGTCTC		616	-16	6.32%
ATGACGGTCAAGTC---CGACGGTCTC		472	-3	4.84%
ATGACGGTCAAGTCCG-CGACGGTCTC		79	-1	0.81%
ATGACGGTCAAG---------------		75	-15	0.77%
ATGACGGTC--------CGACGGTCTC		66	-8	0.68%
ATGACGGTCAAGTCCGAcCGACGGTCTC		63	+1 c	0.65%
ATGACGGTCAAGTCCGA-GACGGTCTC		60	-1	0.62%
ATGACGGTCAAGTCCGAtCGACGGTCTC		56	+1 t	0.57%

P158-P162-YW-W9-Syn104BCL11A-KO

Total | INDEL	4h	Ins	Del	HDR
98337 | 0.52%		**0.02%**	**0.49%**	**0.00%**

Typical seqs	Reads	Type	pct.
CATATTAGTGGTCCGGG|CCCGGGCAGG	97830	WT	99.48%
CATATTAGTGGT------CCGGGCAGG	119	-6	0.12%
CATATTAGTGGTCCGGG--CGGGCAGG	84	-2	0.09%
CATATTAGTGGTCC-GGCCCGGGCAGG	76	-1	0.08%
CATATTAGTGGTCCGGG-CCGGGCAGG	72	-1	0.07%
CATATTAGT------GGCCCGGGCAGG	31	-6	0.03%
CATATTAGTGGTCCGGGgCCCGGGCAGG	21	+1 g	0.02%
CATATTAGTGGTCCGG----GGGCAGG	17	-4	0.02%
CATATTAGTGGTCCGGG----------	9	-13	0.01%
CATAT------------CCCGGGCAGG	9	-12	0.01%

Total | INDEL	8h	Ins	Del	HDR
78367 | 5.89%		**0.54%**	**5.36%**	**0.00%**

Typical seqs	Reads	Type	pct.
CATATTAGTGGTCCGGG|CCCGGGCAGG	73750	WT	94.11%
CATATTAGTGGT------CCGGGCAGG	1895	-6	2.42%
CATATTAGTGGTCCGGG-CCGGGCAGG	564	-1	0.72%
CATATTAGTGGTCC-GGCCCGGGCAGG	483	-1	0.62%
CATATTAGTGGTCCGGGgCCCGGGCAGG	191	+1 g	0.24%
CATATTAGTGGTCCGGG--CGGGCAGG	166	-2	0.21%
CATATTAGTGGTCC---CCCGGGCAGG	104	-3	0.13%
CATATTAGTGGTCCG----------GG	99	-10	0.13%
CATATTAGTGGT-----CCCGGGCAGG	82	-5	0.10%
CATATTAGTGGTCCGGGtCCCGGGCAGG	77	+1 t	0.10%

Total | INDEL	12h	Ins	Del	HDR
278410 | 11.67%		**1.33%**	**10.34%**	**0.00%**

Typical seqs	Reads	Type	pct.
CATATTAGTGGTCCGGG|CCCGGGCAGG	245916	WT	88.33%
CATATTAGTGGT------CCGGGCAGG	12648	-6	4.54%
CATATTAGTGGTCC-GGCCCGGGCAGG	3424	-1	1.23%
CATATTAGTGGTCCGGG-CCGGGCAGG	2659	-1	0.96%
CATATTAGTGGTCCGGGgCCCGGGCAGG	2079	+1 g	0.75%
CATATTAGTGGTCCGGG---GGGCAGG	975	-3	0.35%
CATATTAGT------GGCCCGGGCAGG	974	-6	0.35%
CATATTAGTGGTCCGGG--CGGGCAGG	658	-2	0.24%
CATATTAGTGGT-----CCCGGGCAGG	595	-5	0.21%
CATATTAGTGGTCCGGGcCCCGGGCAGG	540	+1 c	0.19%

Total | INDEL	24h	Ins	Del	HDR
74898 | 23.41%		**1.46%**	**21.95%**	**0.00%**

Typical seqs	Reads	Type	pct.
CATATTAGTGGTCCGGG|CCCGGGCAGG	57367	WT	76.59%
CATATTAGTGGT------CCGGGCAGG	7210	-6	9.63%
CATATTAGTGGTCCGGG-CCGGGCAGG	1206	-1	1.61%
CATATTAGTGGTCC-GGCCCGGGCAGG	952	-1	1.27%
CATATTAGTGGTCCGGGgCCCGGGCAGG	567	+1 g	0.76%
CATATTAGTGGT-----CCCGGGCAGG	549	-5	0.73%
CATATTAGT------GGCCCGGGCAGG	446	-6	0.60%
CATATTAGTGGTCCG----------GG	410	-10	0.55%
CATATTAGTGGTCCG-----GGGCAGG	375	-5	0.50%
CATATTAGTGGTCC--GCCCGGGCAGG	359	-2	0.48%

Total | INDEL	48h	Ins	Del	HDR
81381 | 27.69%		**1.71%**	**25.97%**	**0.00%**

Typical seqs	Reads	Type	pct.
CATATTAGTGGTCCGGG|CCCGGGCAGG	58850	WT	72.31%
CATATTAGTGGT------CCGGGCAGG	10910	-6	13.41%
CATATTAGTGGTCCGGG-CCGGGCAGG	908	-1	1.12%
CATATTAGTGGTCC-GGCCCGGGCAGG	851	-1	1.05%
CATATTAGTGGTCCGGGgCCCGGGCAGG	697	+1 g	0.86%
CATATTAGT------GGCCCGGGCAGG	644	-6	0.79%
CATATTAGTGGTCCG----------GG	557	-10	0.68%
CATATTAGTGGT-----CCCGGGCAGG	498	-5	0.61%
CATATTAGTGGTCCGGG--CGGGCAGG	417	-2	0.51%
CATATTAGT-----------GGGCAGG	219	-11	0.27%

P166-P167-YW-W9-Syn20crAAVS1c-KO

P166-YW-W9-Syn20crAAVS1c-KO-Ctrl-48h-BC1	Ins	Del	HDR
218315 | 64.80%	**51.52%**	**13.23%**	**0.06%**
**Typical seqs**	**Reads**	**Type**	**pct.**
TCTAACCCCCACCTCCT|GTT**AGG**CAGA	76837	WT	35.20%
TCTAACCCCCACCTCCTtGTTAGGCAGA	95038	+1 t	43.53%
TCTAACCCCCACC--------------	3339	-15	1.53%
TCTAACCCCCACCTCC--TTAGGCAGA	2466	-2	1.13%
TCTAACCCCCACCTCCTCTGTTAGGCAGA	1988	+2	0.91%
TCTAACCCCCACCT---------CAGA	1904	-9	0.87%
TCTAACCCCCACCTCCTATGTTAGGCAGA	1713	+2	0.78%
TCTAACCCCCACCTCC-GTTAGGCAGA	1620	-1	0.74%
TCTAACCCCCACCT-CTGTTAGGCAGA	1589	-1	0.73%
TCTAACCCCCA----------------	1510	-18	0.69%

P166-YW-W9-Syn20crAAVS1c-KO-M3814-48h-BC5	Ins	Del	HDR
258164 | 13.40%	**1.47%**	**11.67%**	**0.26%**
**Typical seqs**	**Reads**	**Type**	**pct.**
TCTAACCCCCACCTCCT|GTT**AGG**CAGA	223570	WT	86.60%
TCTAA----------------------	11131	-24	4.31%
TCTAACCCCCACC--------------	8745	-15	3.39%
TCTAACCCCCACCTCC--TTAGGCAGA	4416	-2	1.71%
TCTAACCCCCACCTCCTtGTTAGGCAGA	3222	+1 t	1.25%
TCTAACCCCCA----------------	2556	-18	0.99%
TCTAACCCACGCGTAGTTTAAACTTAGGCAGA	640	+5 HDR	0.25%
TCTAACCCCCA---CCTGTTAGGCAGA	195	-3	0.08%
TCTAACCCCCA-CTCCTGTTAGGCAGA	116	-1	0.04%
TCTAACCCCCACCTCC-GTTAGGCAGA	118	-1	0.05%

P166-YW-W9-Syn20crAAVS1c-KO-M3814+Nu7026-48h-BC6	Ins	Del	HDR
325798 | 11.38%	**1.41%**	**9.79%**	**0.19%**
**Typical seqs**	**Reads**	**Type**	**pct.**
TCTAACCCCCACCTCCT|GTT**AGG**CAGA	288718	WT	88.62%
TCTAACCCCCA---CCTGTTAGGCAGA	25622	-3	7.86%
TCTAACCCCCACCTCCTtGTTAGGCAGA	3796	+1 t	1.17%
TCTAACCCTCA---CCTGTTAGGCAGA	1038	-3	0.32%
TCTAACCCACGCGTAGTTTAAACTTAGGCAGA	587	+5 HDR	0.18%
TCTAACCCCCACC--------------	405	-15	0.12%
TCTAA----------------------	297	-24	0.09%
TCTAACCCCCA---CCTGTTAGGCGGA	189	-3	0.06%
TCTAACC----CCACCTGTTAGGCAGA	168	-4	0.05%
CCTAACCCCCA---CCTGTTAGGCAGA	145	-3	0.04%

P166-YW-W9-Syn20crAAVS1c-KO-M3814+TSA-48h-BC7	Ins	Del	HDR
529397 | 5.40%	**2.46%**	**2.70%**	**0.23%**
**Typical seqs**	**Reads**	**Type**	**pct.**
TCTAACCCCCACCTCCT|GTT**AGG**CAGA	500835	WT	94.60%
TCTAACCCCCACCTCCTtGTTAGGCAGA	10864	+1 t	2.05%
TCTAACCCCCACCTCC-GTTAGGCAGA	8387	-1	1.58%
TCTAACCCACGCGTAGTTTAAACTTAGGCAGA	1169	+5 HDR	0.22%
TCTAACCCCCACC--------------	719	-15	0.14%
TCTAA----------------------	537	-24	0.10%
TCTAACCCCCA---CCTGTTAGGCAGA	416	-3	0.08%
TCTAACCCCCACCTCCTG-TAGGCAGA	261	-1	0.05%
TCTAACCCCCACCTCCTtGCTAGGCAGA	198	+1 t	0.04%
TCTAACCCCCA----------------	197	-18	0.04%

P166-YW-W9-Syn20crAAVS1c-KO-M3814+TSA+Nu7026-48h-BC8	Ins	Del	HDR
262479 | 3.15%	**1.85%**	**0.94%**	**0.37%**
**Typical seqs**	**Reads**	**Type**	**pct.**
TCTAACCCCCACCTCCT|GTT**AGG**CAGA	254199	WT	96.85%
TCTAACCCCCACCTCCTtGTTAGGCAGA	3070	+1 t	1.17%
TCTAACCCCCACCTCCTCTGTTAGGCAGA	1169	+2	0.45%
TCTAACCCACGCGTAGTTTAAACTTAGGCAGA	927	+5 HDR	0.35%
TCTAACCCCCACC--------------	343	-15	0.13%
TCTAA----------------------	238	-24	0.09%
TCTAACCCCCA---CCTGTTAGGCAGA	204	-3	0.08%
TCTAACCCCCACCTCC-GTTAGGCAGA	107	-1	0.04%
TCTAACCCCCACCTCC--TTAGGCAGA	104	-2	0.04%
TCTAACCCCCA----------------	104	-18	0.04%

P166-P167-YW-W9-Syn20crAAVS1c-KO

P166-YW-W9-Syn20crAAVS1c-KO-NaB-48h-BC9	Ins	Del	HDR
79974 | 58.30%	49.92%	8.12%	0.26%
**Typical seqs**	**Reads**	**Type**	**pct.**
TCTAACCCCCACCTCCT|GTT**AGG**CAGA	33351	WT	41.70%
TCTAACCCCCACCTCCTtGTTAGGCAGA	34661	+1 t	43.34%
TCTAACCCCCACCTCCTCTGTTAGGCAGA	950	+2	1.19%
TCTAACCCCCACCTCC--TTAGGCAGA	809	-2	1.01%
TCTAACC----------------CAGA	619	-16	0.77%
TCTAACCCCCACCT---------CAGA	482	-9	0.60%
TCTAACCCCCACCTCC-GTTAGGCAGA	360	-1	0.45%
TCTAACCCCCACCT-CTGTTAGGCAGA	353	-1	0.44%
TCTAACCCCCACC--------------	340	-15	0.43%
TCTAACCCCCA----------------	300	-18	0.38%

P166-YW-W9-Syn20crAAVS1c-KO-NaB+M3814-48h-BC11	Ins	Del	HDR
88558 | 4.02%	1.83%	0.99%	1.19%
**Typical seqs**	**Reads**	**Type**	**pct.**
TCTAACCCCCACCTCCT|GTT**AGG**CAGA	84998	WT	95.98%
TCTAACCCCCACCTCCTtGTTAGGCAGA	1376	+1 t	1.55%
TCTAACCCACGCGTAGTTTAAACTTAGGCAGA	994	+5 HDR	1.12%
TCTAACCCCCACC--------------	114	-15	0.13%
TCTAA----------------------	91	-24	0.10%
TCTAACCCCCA---CCTGTTAGGCAGA	79	-3	0.09%
TCTAACCCCCACCTCC--TTAGGCAGA	43	-2	0.05%
TCTAACCCCCACCTCC-GTTAGGCAGA	39	-1	0.04%
TCTAACCCCCACCTCCTCTGTTAGGCAGA	36	+2	0.04%
TCTAACCCCCACCT---------CAGA	30	-9	0.03%

P166-YW-W9-Syn20crAAVS1c-KO-NaB+Nu7026-48h-BC10	Ins	Del	HDR
173867 | 13.54%	7.72%	5.67%	0.15%
**Typical seqs**	**Reads**	**Type**	**pct.**
TCTAACCCCCACCTCCT|GTT**AGG**CAGA	150321	WT	86.46%
TCTAACCCCCACCTCCTtGTTAGGCAGA	11578	+1 t	6.66%
TCTAA----------CAGTTAGGCAGA	4342	-10	2.50%
TCTAACCCCCACCT-CTGTTAGGCAGA	1715	-1	0.99%
TCTAACCCCCACCTC------------	1716	-14	0.99%
TCTAACCCCCACCTCCTCTGTTAGGCAGA	364	+2	0.21%
TCTAACCCACGCGTAGTTTAAACTTAGGCAGA	237	+5 HDR	0.14%
TCTAACCCCCACC--------------	192	-15	0.11%
TCTAA----------------------	162	-24	0.09%
TCTAACCCCCA---CCTGTTAGGCAGA	169	-3	0.10%

P166-YW-W9-Syn20crAAVS1c-KO-NaB+TSA-48h-BC4	Ins	Del	HDR
386077 | 57.68%	47.29%	10.26%	0.13%
**Typical seqs**	**Reads**	**Type**	**pct.**
TCTAACCCCCACCTCCT|GTT**AGG**CAGA	163370	WT	42.32%
TCTAACCCCCACCTCCTtGTTAGGCAGA	151743	+1 t	39.30%
TCTAACCCCCACCTCCTCTGTTAGGCAGA	5399	+2	1.40%
TCTAACCCCCACC--------------	4169	-15	1.08%
TCTAACCCCCACCTCC-GTTAGGCAGA	3990	-1	1.03%
TCTAACCCCCACCTC--GTTAGGCAGA	2887	-2	0.75%
TCTAACCCCCACCTCC--TTAGGCAGA	2907	-2	0.75%
TCTAACCCCCACCT-CTGTTAGGCAGA	2040	-1	0.53%
TCTAACCCCCACCTCCCTGTTAGGCAGA	1709	+1 Ins	0.44%
TCTAACCCCCA---CCTGTTAGGCAGA	1588	-3	0.41%

P166-YW-W9-Syn20crAAVS1c-KO-NaB+TSA+Nu7026-48h-BC12	Ins	Del	HDR
218997 | 21.41%	16.85%	4.29%	0.27%
**Typical seqs**	**Reads**	**Type**	**pct.**
TCTAACCCCCACCTCCT|GTT**AGG**CAGA	172118	WT	78.59%
TCTAACCCCCACCTCCTtGTTAGGCAGA	32301	+1 t	14.75%
TCTAACCC-------------------	1642	-21	0.75%
TCTAACCCCCAC-----GTTAGGCAGA	1602	-5	0.73%
TCTAACCCCCACCTCC-GTTAGGCAGA	1089	-1	0.50%
TCT---------------------GA	1085	-22	0.50%
TCTAACCCCCACCTCCT-------AGA	640	-7	0.29%
TCTAACCCCCCACC------TAGGCAGA	629	-5	0.29%
TCTAACCCACGCGTAGTTTAAACTTAGGCAGA	568	+5 HDR	0.26%
TCTA------------TGTTAGGCAGA	487	-12	0.22%

P166-P167-YW-W9-Syn20crAAVS1c-KO

P166-YW-W9-Syn20crAAVS1c-KO-Nu7441-48h-BC2	Ins	Del	HDR	P166-YW-W9-Syn20crAAVS1c-KO-Nu7026+NaB+M3814-48h-BC3	Ins	Del	HDR	P166-YW-W9-Syn20crAAVS1c-KO-Nu7026+TSA+NaB+M3814-48h-BC164	Ins	Del	HDR
146324 | 2.53%	**1.47%**	**0.96%**	**0.10%**	154997 | 8.95%	**7.43%**	**1.08%**	**0.44%**	121486 | 2.86%	**1.65%**	**0.96%**	**0.25%**
**Typical seqs**	**Reads**	**Type**	**pct.**	**Typical seqs**	**Reads**	**Type**	**pct.**	**Typical seqs**	**Reads**	**Type**	**pct.**
TCTAACCCCCACCTCCT|GTT**AGG**CAGA	142623	WT	97.47%	TCTAACCCCCACCTCCT|GTT**AGG**CAGA	141121	WT	91.05%	TCTAACCCCCACCTCCT|GTT**AGG**CAGA	118007	WT	97.14%
TCTAACCCCCACCTCCTtGTTAGGCAGA	1823	+1 t	1.25%	TCTAACCCCCACCTCCTtGTTAGGCAGA	10363	+1 t	6.69%	TCTAACCCCCACCTCCTtGTTAGGCAGA	1742	+1 t	1.43%
TCTAACCCCCACC-------------	181	-15	0.12%	TCTAACCCACGCGTAGTTTAAACTTAGGCAGA	664	+5 HDR	0.43%	TCTAACCCACGCGTAGTTTAAACTTAGGCAGA	286	+5 HDR	0.24%
TCTAACCCACGCGTAGTTTAAACTTAGGCAGA	143	+5 HDR	0.10%	TCTAACCCCCACC-------------	242	-15	0.16%	TCTAACCCCCACC-------------	155	-15	0.13%
TCTAA---------------------	140	-24	0.10%	TCTAA---------------------	175	-24	0.11%	TCTAA---------------------	118	-24	0.10%
TCTAACCCCCA---CCTGTTAGGCAGA	123	-3	0.08%	TCTAACCCCCA---CCTGTTAGGCAGA	168	-3	0.11%	TCTAACCCCCA---CCTGTTAGGCAGA	97	-3	0.08%
TCTAACCCCCACCTCC-GTTAGGCAGA	95	-1	0.06%	TCTAACCCCCACCTCC-GTTAGGCAGA	69	-1	0.04%	TCTAACCCCCACCTCC-GTTAGGCAGA	59	-1	0.05%
TCTAACCCCCACCTCCTCTGTTAGGCAGA	66	+2	0.05%	TCTAA----------CAGTTAGGCAGA	63	-10	0.04%	TCTAACCCCCA---------------	60	-18	0.05%
TCTAACCCCCA---------------	58	-18	0.04%	TCTAACCCCCACCTCC--TTAGGCAGA	62	-2	0.04%	TCTAACCCCCACCTCC--TTAGGCAGA	56	-2	0.05%
TCTAACCCCCACCTCC--TTAGGCAGA	51	-2	0.03%	TCTAACCCCCACCTCCCTGTTAGGCAGA	67	+1 Ins	0.04%	TCTAACCCCCACCTCCTCTGTTAGGCAGA	44	+2	0.04%

P166-YW-W9-Syn20crAAVS1c-KO-TSA-48h-BC13	Ins	Del	HDR	P166-YW-W9-Syn20crAAVS1c-KO-TSA+Nu7026-48h-BC14	Ins	Del	HDR	P166-YW-W9-Syn20crAAVS1c-KO-TSA+NaB+M3814-48h-BC15	Ins	Del	HDR
304963 | 56.38%	**46.42%**	**9.86%**	**0.10%**	179150 | 18.04%	**16.90%**	**0.97%**	**0.17%**	386721 | 4.56%	**3.42%**	**0.93%**	**0.21%**
**Typical seqs**	**Reads**	**Type**	**pct.**	**Typical seqs**	**Reads**	**Type**	**pct.**	**Typical seqs**	**Reads**	**Type**	**pct.**
TCTAACCCCCACCTCCT|GTT**AGG**CAGA	133039	WT	43.62%	TCTAACCCCCACCTCCT|GTT**AGG**CAGA	146833	WT	81.96%	TCTAACCCCCACCTCCT|GTT**AGG**CAGA	369094	WT	95.44%
TCTAACCCCCACCTCCTtGTTAGGCAGA	117041	+1 t	38.38%	TCTAACCCCCACCTCCTtGTTAGGCAGA	27007	+1 t	15.08%	TCTAACCCCCACCTCCTtGTTAGGCAGA	11553	+1 t	2.99%
TCTAACCCCCACCTCCT-TTAGGCAGA	5675	-1	1.86%	TCTAACCCACGCGTAGTTTAAACTTAGGCAGA	295	+5 HDR	0.16%	TCTAACCCACGCGTAGTTTAAACTTAGGCAGA	789	+5 HDR	0.20%
TCTAACCCCCACCTCCTCTGTTAGGCAGA	3669	+2	1.20%	TCTAACCCCCACC-------------	257	-15	0.14%	TCTAACCCCCACC-------------	493	-15	0.13%
TCTAACCCCCACCT--TGTTAGGCAGA	3221	-2	1.06%	TCTAACCCCCACCCCCTTGTTAGGCAGA	176	+1 Ins	0.10%	TCTAA---------------------	341	-24	0.09%
TCTAACCCCCACCTCCCTGTTAGGCAGA	2856	+1 Ins	0.94%	TCTAA---------------------	172	-24	0.10%	TCTAACCCCCA---CCTGTTAGGCAGA	324	-3	0.08%
TCTAACCCCCACCTCC--TTAGGCAGA	2499	-2	0.82%	TCTAACCCCCATCTCCTTGTTAGGCAGA	154	+1 Ins	0.09%	TCTAACCCCCACCTCC-GTTAGGCAGA	168	-1	0.04%
TCTAACCCCCACCTCC-GTTAGGCAGA	2250	-1	0.74%	TCTAACCCCCA---CCTGTTAGGCAGA	156	-3	0.09%	TCTAACCCCCA---------------	164	-18	0.04%
TCTAACCC------CCTGTTAGGCAGA	2080	-6	0.68%	TCTACCCCCCACCTCCTTGTTAGGCAGA	159	+1 Ins	0.09%	TCTAACCCCCACCTCC--TTAGGCAGA	155	-2	0.04%
TCT--------------------AGA	1747	-21	0.57%	TCTAGCCCCCACCTCCTTGTTAGGCAGA	148	+1 Ins	0.08%	TCTAACCCCCACCTCCTCTGTTAGGCAGA	122	+2	0.03%

P166-P167-YW-W9-Syn20crAAVS1c-AAVKI

P167-YW-W9-Syn20crAAVS1c-AAV6KI-Ctrl-48h-BC1	Ins	Del	HDR
126316 | 82.25%	**60.34%**	**10.54%**	**11.38%**
**Typical seqs**	**Reads**	**Type**	**pct.**
TCTAACCCCCACCTCCTGTT**AGG**CAGA	22415	WT	17.75%
TCTAACCCCCACCTCCTtGTTAGGCAGA	61793	+1 t	48.92%
TCTAACCCACGCGTAGTTTAAACTTAGGCAGA	13507	+5 HDR	10.69%
TCTAACCCCCACCTCCTCTGTTAGGCAGA	3667	+2	2.90%
TCTAACCCCCACC--------------	2249	-15	1.78%
TCTAACCCCCACCT--TGTTAGGCAGA	1783	-2	1.41%
TCTAACCCCCACCT-CTGTTAGGCAGA	1141	-1	0.90%
TCTAACCCCCACCTCCTATGTTAGGCAGA	1010	+2	0.80%
TCTAACCCCCACCTC--GTTAGGCAGA	890	-2	0.70%
TCT-------------TGTTAGGCAGA	847	-13	0.67%

P167-YW-W9-Syn20crAAVS1c-AAV6KI-M3814-48h-BC5	Ins	Del	HDR
228717 | 67.83%	**9.03%**	**11.09%**	**47.70%**
**Typical seqs**	**Reads**	**Type**	**pct.**
TCTAACCCCCACCTCCTGTT**AGG**CAGA	73578	WT	32.17%
TCTAACCCACGCGTAGTTTAAACTTAGGCAGA	101728	+5 HDR	44.48%
TCTAACCCCCACCTCCTtGTTAGGCAGA	11607	+1 t	5.07%
TCTAACCCCCACCT--TGTTAGGCAGA	9881	-2	4.32%
TCTAACCCCCACC--------------	6374	-15	2.79%
TCTAACCCCCACCTCC---TAGGCAGA	6167	-3	2.70%
TCTAACCCACGCGTAGCTTAAACTTAGGCAGA	630	+5	0.28%
TCTAACCCACGCGTAGTCTAAACTTAGGCAGA	562	+5	0.25%
TCTAACCCGCGCGTAGTTTAAACTTAGGCAGA	439	+5	0.19%
TCTAACCCACGCGTAGTTTAAGCTTAGGCAGA	435	+5	0.19%

P167-YW-W9-Syn20crAAVS1c-AAV6KI-M3814+Nu7026-48h-BC6	Ins	Del	HDR
230650 | 68.16%	**25.15%**	**2.88%**	**40.13%**
**Typical seqs**	**Reads**	**Type**	**pct.**
TCTAACCCCCACCTCCTGTT**AGG**CAGA	73434	WT	31.84%
TCTAACCCACGCGTAGTTTAAACTTAGGCAGA	85750	+5 HDR	37.18%
TCTAACCCCCACCTCCTtGTTAGGCAGA	44715	+1 t	19.39%
TCTAACCCCCACCT-CTGTTAGGCAGA	4717	-1	2.05%
TCTAGCCCCCACCTCCTTGTTAGGCAGA	1377	+1 Ins	0.60%
TCTAACCCCCACCTCCTcGTTAGGCAGA	443	+1 c	0.19%
TCTAACCCACGCGTAGTTTAGACTTAGGCAGA	417	+5	0.18%
TCTAACCCACGCGTAGTCTAAACTTAGGCAGA	394	+5	0.17%
TCTAACCCCCCCTCCTTGTTAGGCAGA	458	+1 Ins	0.20%
TCTAACCCACGCGCAGTTTAAAC------TTAGGCAGA	449	+5	0.19%

P167-YW-W9-Syn20crAAVS1c-AAV6KI-M3814+TSA-48h-BC7	Ins	Del	HDR
267173 | 90.36%	**10.78%**	**6.35%**	**73.24%**
**Typical seqs**	**Reads**	**Type**	**pct.**
TCTAACCCCCACCTCCTGTT**AGG**CAGA	25743	WT	9.64%
TCTAACCCACGCGTAGTTTAAACTTAGGCAGA	181469	+5 HDR	67.92%
TCTAACCCCCACCTCCTtGTTAGGCAGA	11967	+1 t	4.48%
TCTAACCCCCACC----GTTAGGCAGA	6901	-4	2.58%
TCTAACCC------CCTGTTAGGCAGA	2623	-6	0.98%
TCTAACCCCCACCTCC--TTAGGCAGA	2405	-2	0.90%
TCTAACCCCC--------TTAGGCAGA	1858	-8	0.70%
TCTAACCCACGCGTAGTTTAGACTTAGGCAGA	1812	+5	0.68%
TCTAACCCGCGCGTAGTTTAAACTTAGGCAGA	1668	+5	0.62%
TCTAACCCACGCGTAGTCTAAACTTAGGCAGA	1356	+5	0.51%

P167-YW-W9-Syn20crAAVS1c-AAV6KI-M3814+TSA+Nu7026-48h-BC8	Ins	Del	HDR
224776 | 93.10%	**13.32%**	**9.31%**	**70.46%**
**Typical seqs**	**Reads**	**Type**	**pct.**
TCTAACCCCCACCTCCTGTT**AGG**CAGA	15518	WT	6.90%
TCTAACCCACGCGTAGTTTAAACTTAGGCAGA	145615	+5 HDR	64.78%
TCTAACCCCCACCTCCTtGTTAGGCAGA	17660	+1 t	7.86%
TCTAACC-------CCTGTTAGGCAGA	4285	-7	1.91%
TCTAACCCCCACC--------------	3678	-15	1.64%
TCTAACCCCCACCTCC--TTA-----A	2895	-7	1.29%
TCTAACCCCCACCTC------------	2125	-14	0.95%
TCTAACC----------------CAGA	2123	-16	0.94%
TCTA-----------------------	1969	-26	0.88%
TCTAACCCACGCGTAGTTTAAGCTTAGGCAGA	949	+5	0.42%

P166-P167-YW-W9-Syn20crAAVS1c-AAVKI

P167-YW-W9-Syn20crAAVS1c-AAV6KI-NaB-48h-BC9	Ins	Del	HDR
146329 | 92.08%	63.70%	10.03%	18.34%
**Typical seqs**	**Reads**	**Type**	**pct.**
TCTAACCCCCACCTCCT|GTT**AGG**CAGA	11596	WT	7.92%
TCTAACCCCCACCTCCTtGTTAGGCAGA	74962	+1 t	51.23%
TCTAACCCACGCGTAGTTTAAACTTAGGCAGA	25366	+5 HDR	17.33%
TCTAACCCCCACCTCCTCTGTTAGGCAGA	4382	+2	2.99%
TCTAACCC------------------A	3238	-18	2.21%
TCTAACCCCCACCTCC--TTAGGCAGA	1700	-2	1.16%
TCTAACCCCCACCT--TGTTAGGCAGA	1316	-2	0.90%
TCTAA-----------------------	1248	-24	0.85%
TCTAACCC---------------CAGA	1193	-15	0.82%
TCTAACCCCCACCTCC-GTTAGGCAGA	1309	-1	0.89%

P167-YW-W9-Syn20crAAVS1c-AAV6KI-NaB+M3814-48h-BC11	Ins	Del	HDR
228849 | 90.97%	8.73%	5.94%	76.30%
**Typical seqs**	**Reads**	**Type**	**pct.**
TCTAACCCCCACCTCCT|GTT**AGG**CAGA	20666	WT	9.03%
TCTAACCCACGCGTAGTTTAAACTTAGGCAGA	162948	+5 HDR	71.20%
TCTAACCCCCACCTCCTtGTTAGGCAGA	7260	+1 t	3.17%
TCTAACCCC--------------CAGA	2647	-14	1.16%
TCTAA-----------------------	2513	-24	1.10%
TCTAA------------------CAGA	2229	-18	0.97%
TCTAAC----------------------	1988	-22	0.87%
TCTAACCCACGCGCAGTTTAAAC------TTAGGCAGA	1258	+5	0.55%
TCTAACCCCCACC--------------	1186	-14	0.52%
TCTAACCCCCACCTCC-----------	973	-12	0.43%

P167-YW-W9-Syn20crAAVS1c-AAV6KI-NaB+Nu7026-48h-BC10	Ins	Del	HDR
122195 | 81.05%	46.92%	6.94%	27.18%
**Typical seqs**	**Reads**	**Type**	**pct.**
TCTAACCCCCACCTCCT|GTT**AGG**CAGA	23162	WT	18.95%
TCTAACCCCCACCTCCTtGTTAGGCAGA	45659	+1 t	37.37%
TCTAACCCACGCGTAGTTTAAACTTAGGCAGA	31081	+5 HDR	25.44%
TCTAACCCCCACCT-CTGTTAGGCAGA	2526	-1	2.07%
TCTAACCCCCACCTCC-GTTAGGCAGA	1407	-1	1.15%
TCTAAC-----------------CAGA	1341	-17	1.10%
TCTAACCCCCACCTCCTTTGTTAGGCAGA	1238	+2	1.01%
TCTAACCCCCACCT--TGTTAGGCAGA	1117	-2	0.91%
TCTAACCCGCGCGTAGTTTAAACTTAGGCAGA	1011	+5	0.83%
TCTAACCCCCACCTCCTCTGTTAGGCAGA	632	+2	0.52%

P167-YW-W9-Syn20crAAVS1c-AAV6KI-NaB+TSA-48h-BC4	Ins	Del	HDR
288730 | 92.05%	60.71%	7.70%	23.65%
**Typical seqs**	**Reads**	**Type**	**pct.**
TCTAACCCCCACCTCCT|GTT**AGG**CAGA	22954	WT	7.95%
TCTAACCCCCACCTCCTtGTTAGGCAGA	145422	+1 t	50.37%
TCTAACCCACGCGTAGTTTAAACTTAGGCAGA	63415	+5 HDR	21.96%
TCTAACCCCCACCTCCTCTGTTAGGCAGA	4542	+2	1.57%
TCTAACCCCCACCTCC---TAGGCAGA	3797	-3	1.32%
TCTAACCCCCACC--------------	3622	-15	1.25%
TCTAACCCCCACCTC----AAGGCAGA	3297	-4	1.14%
TCTAACCCCCA----CTGTTAGGCAGA	3257	-4	1.13%
TCTAA-----------------------	2103	-30	0.73%
TCTAACCCCCACCT-CTGTTAGGCAGA	1257	-1	0.44%

P167-YW-W9-Syn20crAAVS1c-AAV6KI-NaB+TSA+Nu7026-48h-BC12	Ins	Del	HDR
224546 | 89.15%	42.29%	5.90%	40.96%
**Typical seqs**	**Reads**	**Type**	**pct.**
TCTAACCCCCACCTCCT|GTT**AGG**CAGA	24356	WT	10.85%
TCTAACCCACGCGTAGTTTAAACTTAGGCAGA	84753	+5 HDR	37.74%
TCTAACCCCCACCTCCTtGTTAGGCAGA	78471	+1 t	34.95%
TCTAACCCCCACCTCCT-TTAGGCAGA	3003	-1	1.34%
TCTAACCCCCACCTCC-GTTAGGCAGA	2553	-1	1.14%
TCTAACCCCCA---CCTGTTAGGCAGA	1927	-3	0.86%
TCTAACCCCCA------GTTAGGCAGA	1857	-6	0.83%
TCTAA----------------------	1012	-24	0.45%
TCTAGCCCCCACCTCCTTGTTAGGCAGA	649	+1 Ins	0.29%
TCTAACCCCCACCTCCTAGTGTTAGGCAGA	569	+3	0.25%

P166-P167-YW-W9-Syn20crAAVS1c-AAVKI

P167-YW-W9-Syn20crAAVS1c-AAV6KI-Nu7441-48h-BC2	Ins	Del	HDR
98222 | 69.92%	37.77%	11.42%	20.73%
**Typical seqs**	**Reads**	**Type**	**pct.**
TCTAACCCCCACCTCCT|GTT**AGG**CAGA	29547	WT	30.08%
TCTAACCCCCACCTCCTtGTTAGGCAGA	30018	+1 t	30.56%
TCTAACCCACGCGTAGTTTAAACTTAGGCAGA	18899	+5 HDR	19.24%
TCTAACCCCCACCTCC-GTTAGGCAGA	6727	-1	6.85%
TCTAACCCCCACCTCCTCTGTTAGGCAGA	1771	+2	1.80%
TCTAACC----------------CAGA	1577	-16	1.61%
TCTAACCCCCA----------------	1156	-18	1.18%
TC-----------------TAGGCAGA	458	-17	0.47%
TCTAACCCCCACCTCCTcGTTAGGCAGA	307	+1 c	0.31%
TCTAACCCCCACCTCCCTGTTAGGCAGA	228	+1 Ins	0.23%

P167-YW-W9-Syn20crAAVS1c-AAV6KI-Nu7026+NaB+M3814-48h-BC3	Ins	Del	HDR
164056 | 88.15%	10.28%	7.03%	70.84%
**Typical seqs**	**Reads**	**Type**	**pct.**
TCTAACCCCCACCTCCT|GTT**AGG**CAGA	19434	WT	11.85%
TCTAACCCACGCGTAGTTTAAACTTAGGCAGA	108799	+5 HDR	66.32%
TCTAACCCCCACCTCCTtGTTAGGCAGA	8136	+1 t	4.96%
TCTAA---------------AGGCAGA	7111	-15	4.33%
TCTAACCCCCA---CCTGTTAGGCAGA	2839	-3	1.73%
TCTAACCCGCGCGTAGTTTAAACTTAGGCAGA	555	+5	0.34%
TCTAACCCACGCGTAGTCTAAACTTAGGCAGA	549	+5	0.33%
TCTAACCCACGCGTAGCTTAAACTTAGGCAGA	517	+5	0.32%
TCTAACCCACGCGTAGTTTAAGCTTAGGCAGA	509	+5	0.31%
TCTAACCCACGCGTAGTTTAGACTTAGGCAGA	469	+5	0.29%

P167-YW-W9-Syn20crAAVS1c-AAV6KI-Nu7026+TSA+NaB+M3814-48h-BC16	Ins	Del	HDR
56168 | 96.91%	14.41%	0.62%	81.88%
**Typical seqs**	**Reads**	**Type**	**pct.**
TCTAACCCCCACCTCCT|GTT**AGG**CAGA	1734	WT	3.09%
TCTAACCCACGCGTAGTTTAAACTTAGGCAGA	42137	+5 HDR	75.02%
TCTAACCCCCACCTCCTtGTTAGGCAGA	4798	+1 t	8.54%
TCTAACCCGCGCGTAGTTTAAACTTAGGCAGA	414	+5	0.74%
TCTAACCCACGCGTAGTTTAAGCTTAGGCAGA	186	+5	0.33%
TCTAACCCACGCGTAGTTTGAACTTAGGCAGA	181	+5	0.32%
TCTAACCCACGCGTAGCTTAAACTTAGGCAGA	167	+5	0.30%
TCTAACCCACGCGTAGTCTAAACTTAGGCAGA	163	+5	0.29%
TCTAACCCACGCGTAGTTTCAACTTAGGCAGA	141	+5	0.25%
TCTAACCCACGCGTAGTTTAGACTTAGGCAGA	142	+5	0.25%

P167-YW-W9-Syn20crAAVS1c-AAV6KI-TSA-48h-BC13	Ins	Del	HDR
255769 | 91.79%	71.59%	5.86%	14.33%
**Typical seqs**	**Reads**	**Type**	**pct.**
TCTAACCCCCACCTCCT|GTT**AGG**CAGA	21003	WT	8.21%
TCTAACCCCCACCTCCTtGTTAGGCAGA	153393	+1 t	59.97%
TCTAACCCACGCGTAGTTTAAACTTAGGCAGA	34453	+5 HDR	13.47%
TCTAACCCCCACCTCC--TTAGGCAGA	5778	-2	2.26%
TCTAACCCCCACCTCCTCTGTTAGGCAGA	5653	+2	2.21%
TCTAACCCCA----CTGTTAGGCAGA	2502	-4	0.98%
TCTAACCCCCACCTCCT-TTAGGCAGA	1828	-1	0.71%
TCTAACCCCCACCTCC-GTTAGGCAGA	1555	-1	0.61%
TCTAACCCCA---CCTGTTAGGCAGA	1078	-3	0.42%
TCTAACCCCCACCTCCTcGTTAGGCAGA	1059	+1 c	0.41%

P167-YW-W9-Syn20crAAVS1c-AAV6KI-TSA+Nu7026-48h-BC14	Ins	Del	HDR
154669 | 97.46%	60.34%	5.69%	31.43%
**Typical seqs**	**Reads**	**Type**	**pct.**
TCTAACCCCCACCTCCT|GTT**AGG**CAGA	3928	WT	2.54%
TCTAACCCCCACCTCCTtGTTAGGCAGA	76979	+1 t	49.77%
TCTAACCCACGCGTAGTTTAAACTTAGGCAGA	44792	+5 HDR	28.96%
TCTAACCCCCACCTCC--TTAGGCAGA	3824	-2	2.47%
TCTAACCCC--------------CAGA	2777	-14	1.80%
TCTAACCCCCACCTCCTCTGTTAGGCAGA	2447	+2	1.58%
TCTAATCCCCACCTCC--TTAGGCAGA	886	-2	0.57%
TCTAGCCCCCACCTCCTTGTTAGGCAGA	858	+1 Ins	0.55%
TCTAACCCACGCGTAGTCTAAACTTAGGCAGA	550	+5	0.36%
TCTAACCCCCGCCTCCTTGTTAGGCAGA	484	+1 Ins	0.31%

P167-YW-W9-Syn20crAAVS1c-AAV6KI-TSA+NaB+M3814-48h-BC15	Ins	Del	HDR
192543 | 80.72%	9.77%	3.00%	67.95%
**Typical seqs**	**Reads**	**Type**	**pct.**
TCTAACCCCCACCTCCT|GTT**AGG**CAGA	37124	WT	19.28%
TCTAACCCACGCGTAGTTTAAACTTAGGCAGA	121990	+5 HDR	63.36%
TCTAACCCCCACCTCCTtGTTAGGCAGA	9959	+1 t	5.17%
TCTAACCCCCACCTCCT-----ACAGA	4383	-5	2.28%
TCTAACCCACGCGTAGTCTAAACTTAGGCAGA	757	+5	0.39%
TCTAACCCGCGCGTAGTTTAAACTTAGGCAGA	560	+5	0.29%
TCTAACCCACGCGTAGTTTAAGCTTAGGCAGA	492	+5	0.26%
TCTAACCCACGCGTAGTTTGAACTTAGGCAGA	457	+5	0.24%
TCTAACCCACGCGTGGTTTAAACTTAGGCAGA	419	+5	0.22%
TCTAACCCACGCGCAGTTTAAAC------TTAGGCAGA	401	+5	0.21%

P168-P169-YW-W9-Syn24crAAVS1g-KO

P168-YW-W9-Syn24crAAVS1g-KO-Ctrl-48h-BC1	Ins	Del	HDR
86007 | 18.71%	0.51%	18.16%	0.04%
**Typical seqs**	**Reads**	**Type**	**pct.**
GGAATCTGCCTAACAGG|AGG**TGG**GGGT	69913	WT	81.29%
GGAATCTGCCTAAC---AGGTGGGGT	3064	-3	3.56%
GGAATCTGCCTAACAGG-GGTGGGGT	2469	-1	2.87%
GGAATCTGCCTAACAG--GGTGGGGT	1473	-2	1.71%
GGAATCTGCCTAACA-GAGGTGGGGT	1480	-1	1.72%
GGAATCTGCCTA-----AGGTGGGGT	1116	-5	1.30%
GGAATCTGCC-----------------	992	-17	1.15%
GGAATCTGCCTAACA------GGGGT	524	-6	0.61%
GGAATCTG-------------------	431	-23	0.50%
GGAATCT-----------------GT	439	-18	0.51%

P168-YW-W9-Syn24crAAVS1g-KO-M3814-48h-BC5	Ins	Del	HDR
214852 | 5.08%	4.32%	0.72%	0.05%
**Typical seqs**	**Reads**	**Type**	**pct.**
GGAATCTGCCTAACAGG|AGG**TGG**GGGT	203931	WT	94.92%
GGAATCTGCCTAACAGGaAGGTGGGGT	8185	+1 a	3.81%
GGAATCTGCCTAACAGG-GGTGGGGT	150	-1	0.07%
GGAATCTGCCTAACAGGgAGGTGGGGT	135	+1 g	0.06%
GGAATCTGCCTAAC-------------	107	-14	0.05%
GGAATCTGCCTAA----AGGTGGGGT	112	-4	0.05%
GGAATCTGCCTAAGTTTAAACTACGCGTGG GT	98	+5 HDR	0.05%
GGAATCTGCCTAACA-GAGGTGGGGT	95	-1	0.04%
GGAATCTGCCTAAC---AGGTGGGGT	88	-3	0.04%
GGAATCTGC--------AGGTGGGGT	77	-8	0.04%

P168-YW-W9-Syn24crAAVS1g-KO-M3814+Nu7026-48h-BC6	Ins	Del	HDR
162319 | 2.77%	0.12%	2.59%	0.07%
**Typical seqs**	**Reads**	**Type**	**pct.**
GGAATCTGCCTAACAGG|AGG**TGG**GGGT	157822	WT	97.23%
GGAATCT---------GAGGTGGGGT	2763	-9	1.70%
GGAATCTGCCTAACAGG-GGTGGGGT	124	-1	0.08%
GGAATCTGCCTAAGTTTAAACTACGCGTGG GT	102	+5 HDR	0.06%
GGAATCT---------GAGGTGGGGC	94	-9	0.06%
GGAATCTGCCTAA----AGGTGGGGT	88	-4	0.05%
GGAATCTGCCTAAC-------------	89	-14	0.05%
GGAATCTGCCTAACA-GAGGTGGGGT	80	-1	0.05%
GGAATCTGCCTAAC---AGGTGGGGT	78	-3	0.05%
GGAATCTGCCTAACAAGGAGGTGGGGT	66	+1 Ins	0.04%

P168-YW-W9-Syn24crAAVS1g-KO-M3814+TSA-48h-BC7	Ins	Del	HDR
320562 | 0.92%	0.10%	0.75%	0.07%
**Typical seqs**	**Reads**	**Type**	**pct.**
GGAATCTGCCTAACAGG|AGG**TGG**GGGT	317620	WT	99.08%
GGAATCTGCCTAAGTTTAAACTACGCGTGG GT	213	+5 HDR	0.07%
GGAATCTGCCTAACAGG-GGTGGGGT	224	-1	0.07%
GGAATCTGCCTAACA-GAGGTGGGGT	138	-1	0.04%
GGAATCTGCCTAA----AGGTGGGGT	145	-4	0.05%
GGAATCTGCCTAAC-------------	147	-14	0.05%
GGAATCTGCCTAACAGGgAGGTGGGGT	102	+1 g	0.03%
GGAATCTGC--------AGGTGGGGT	104	-8	0.03%
GGAATC---------------------	101	-21	0.03%
GGAATCTGCCTA---------------	103	-18	0.03%

P168-YW-W9-Syn24crAAVS1g-KO-M3814+TSA+Nu7026-48h-BC8	Ins	Del	HDR
141517 | 0.99%	0.15%	0.70%	0.14%
**Typical seqs**	**Reads**	**Type**	**pct.**
GGAATCTGCCTAACAGG|AGG**TGG**GGGT	140121	WT	99.01%
GGAATCTGCCTAAGTTTAAACTACGCGTGG GT	203	+5 HDR	0.14%
GGAATCTGCCTAACAGG-GGTGGGGT	109	-1	0.08%
GGAATCTGCCTAAC-------------	78	-14	0.06%
GGAATCTGCCTAACAGGgAGGTGGGGT	77	+1 g	0.05%
GGAATCTGCCTAACA-GAGGTGGGGT	69	-1	0.05%
GGAATCTGCCTAACAAGGAGGTGGGGT	55	+1 Ins	0.04%
GGAATCTGCCTAA----AGGTGGGGT	61	-4	0.04%
GGAATCTGCCTA---------------	53	-18	0.04%
GGAATCTGC--------AGGTGGGGT	50	-8	0.04%

P168-P169-YW-W9-Syn24crAAVS1g-KO

P168-YW-W9-Syn24crAAVS1g-KO-NaB-48h-BC9<br>69240 | 18.55%	Ins<br>2.58%	Del<br>15.85%	HDR<br>0.11%
**Typical seqs**	**Reads**	**Type**	**pct.**
GGAATCTGCCTAACAGG|AGG**TGG**GGGT	56398	WT	81.45%
GGAATCTGCCTAACAGG-GGTGGGGGT	2457	-1	3.55%
GGAATCTGCCTAAC---AGGTGGGGGT	2633	-3	3.80%
GGAATCTGCCTAACA-GAGGTGGGGGT	1501	-1	2.17%
GGAATCTGCCTAACAGGtAGGTGGGGGT	777	+1 t	1.12%
GGAATCTGCCTAACAG--GGTGGGGGT	511	-2	0.74%
GGAATCTGCCTAACAGGgAGGTGGGGGT	483	+1 g	0.70%
GGAATCTGCCTAACA--------GGGT	470	-8	0.68%
GGAATCTGC------------------	472	-20	0.68%
GGAATCTGCCTAA----AGGTGGGGGT	359	-4	0.52%

P168-YW-W9-Syn24crAAVS1g-KO-NaB+M3814-48h-BC11<br>151596 | 7.35%	Ins<br>0.13%	Del<br>7.07%	HDR<br>0.15%
**Typical seqs**	**Reads**	**Type**	**pct.**
GGAATCTGCCTAACAGG|AGG**TGG**GGGT	140459	WT	92.65%
GGAATCTGCCTAAC-------------	9089	-14	6.00%
GGAATCTGCCTAAGTTTAAACTACGCGTGG GT	214	+5 HDR	0.14%
GGAATCTGCCTAACAGG-GGTGGGGGT	119	-1	0.08%
GGAATCTGCCTTAC-------------	94	-14	0.06%
GGAATCTGCCTAACAGGgAGGTGGGGGT	88	+1 g	0.06%
GGAATCTGCCTAACA-GAGGTGGGGGT	77	-1	0.05%
GGAATCTGCCTA---------------	64	-18	0.04%
GGAATCTGCCTAAC---AGGTGGGGGT	61	-3	0.04%
GGAATCTGC--------AGGTGGGGGT	59	-8	0.04%

P168-YW-W9-Syn24crAAVS1g-KO-NaB+Nu7026-48h-BC10<br>97572 | 13.66%	Ins<br>0.14%	Del<br>13.44%	HDR<br>0.07%
**Typical seqs**	**Reads**	**Type**	**pct.**
GGAATCTGCCTAACAGG|AGG**TGG**GGGT	84245	WT	86.34%
GGAATCTGCCTAAC---AGGTGGGGGT	4193	-3	4.30%
GGAATCTGCCTAA----AGGTGGGGGT	2957	-4	3.03%
GGAATCTGCCTAACAGG-GGTGGGGGT	1821	-1	1.87%
GGAATCTGCCTAACAG--GCTGGGGGT	1585	-2	1.62%
----------------------GGGGT	645	-22	0.66%
GGAGTCTGCCTAA----AGGTGGGGGT	296	-4	0.30%
GGAATCTGCCTAAGTTTAAACTACGCGTGG GT	73	+5 HDR	0.07%
GGAATCTGCCTAAC-------------	56	-14	0.06%
GGAATCTGCCTAACA-GAGGTGGGGGT	58	-1	0.06%

P168-YW-W9-Syn24crAAVS1g-KO-NaB+TSA-48h-BC4<br>260301 | 27.37%	Ins<br>6.61%	Del<br>20.70%	HDR<br>0.06%
**Typical seqs**	**Reads**	**Type**	**pct.**
GGAATCTGCCTAACAGG|AGG**TGG**GGGT	189055	WT	72.63%
GGAATCTGCCTAACAGG-GGTGGGGGT	11205	-1	4.30%
GGAATCTGCCTAACAGGgAGGTGGGGGT	8246	+1 g	3.17%
GGAATCTGCCTAA----AGGTGGGGGT	7316	-4	2.81%
GGAATCTGCCTAACAGGaAGGTGGGGGT	6753	+1 a	2.59%
GGAATCTGCCTAA-------TGGGGGT	5834	-7	2.24%
GGAATCTGCCTA---------------	5329	-18	2.05%
GGAATC---------------------	4341	-21	1.67%
GGAATCTGC--------AGGTGGGGGT	3764	-8	1.45%
GGAATCTGCCT------AGGTGGGGGT	3480	-6	1.34%

P168-YW-W9-Syn24crAAVS1g-KO-NaB+TSA+Nu7026-48h-BC12<br>95700 | 5.24%	Ins<br>0.19%	Del<br>4.89%	HDR<br>0.16%
**Typical seqs**	**Reads**	**Type**	**pct.**
GGAATCTGCCTAACAGG|AGG**TGG**GGGT	90682	WT	94.76%
GGAA-----------------------	3948	-23	4.13%
GGAATCTGCCTAAGTTTAAACTACGCGTGG GT	156	+5 HDR	0.16%
GGAATCTGCCTAACAAGGAGGTGGGGGT	94	+1 Ins	0.10%
GGAATCTGCCTAACAGG-GGTGGGGGT	87	-1	0.09%
GGAATCTGCCTAACA-GAGGTGGGGGT	54	-1	0.06%
GGAATCTGCCTAAC-------------	51	-14	0.05%
GGAATCTGCCTAACAGGgAGGTGGGGGT	45	+1 g	0.05%
GGAATCTGCCTA---------------	45	-18	0.05%
GGAATCTGCCTAA----AGGTGGGGGT	41	-4	0.04%

P168-P169-YW-W9-Syn24crAAVS1g-KO

P168-YW-W9-Syn24crAAVS1g-KO-Nu7441-48h-BC2	Ins	Del	HDR
88853 | 0.94%	**0.12%**	**0.75%**	**0.07%**
**Typical seqs**	**Reads**	**Type**	**pct.**
GGAATCTGCCTAACAGG|AGG**TGG**GGGT	88021	WT	99.06%
GGAATCTGCCTAAGTTTAAACTACGCGTGGGT	61	+5 HDR	0.07%
GGAATCTGCCTAACAGG-GGTGGGGGT	61	-1	0.07%
GGAATCTGCCTAAC-------------	56	-14	0.06%
GGAATCTGCCTAA----AGGTGGGGGT	46	-4	0.05%
GGAATCT---------GAGGTGGGGGT	39	-9	0.04%
GGAATCTGCCTAACAAGGAGGTGGGGGT	38	+1 Ins	0.04%
GGAATCTGC--------AGGTGGGGGT	38	-8	0.04%
GGAATC---------------------	38	-21	0.04%
GGAATCTGCCTAACA-GAGGTGGGGGT	36	-1	0.04%

P168-YW-W9-Syn24crAAVS1g-KO-Nu7026+NaB+M3814-48h-BC3	Ins	Del	HDR
117834 | 1.14%	**0.13%**	**0.88%**	**0.13%**
**Typical seqs**	**Reads**	**Type**	**pct.**
GGAATCTGCCTAACAGG|AGG**TGG**GGGT	116490	WT	98.86%
GGAATCTGCCTAAGTTTAAACTACGCGTGGGT	154	+5 HDR	0.13%
GGAATCTGCCTAACAGG-GGTGGGGGT	100	-1	0.08%
GGAATCTGCCTAAC-------------	80	-14	0.07%
GGAATCTGCCTAACA-GAGGTGGGGGT	66	-1	0.06%
GGAATCTGCCTAA----AGGTGGGGGT	69	-4	0.06%
GGAATCTGCCTA---------------	58	-18	0.05%
GGAATCTGC--------AGGTGGGGGT	59	-8	0.05%
GGAATCTGCCTAACAGGaAGGTGGGGGT	49	+1 a	0.04%
GGAATC---------------------	43	-21	0.04%

P168-YW-W9-Syn24crAAVS1g-KO-Nu7026+TSA+NaB+M3814-48h-BC16	Ins	Del	HDR
248772 | 0.88%	**0.10%**	**0.73%**	**0.05%**
**Typical seqs**	**Reads**	**Type**	**pct.**
GGAATCTGCCTAACAGG|AGG**TGG**GGGT	246571	WT	99.12%
GGAATCTGCCTAAC-------------	151	-14	0.06%
GGAATCTGCCTAACAGG-GGTGGGGGT	133	-1	0.05%
GGAATCTGCCTAAGTTTAAACTACGCGTGGGT	136	+5 HDR	0.05%
GGAATCTGCCTAA----AGGTGGGGGT	124	-4	0.05%
GGAATCTGCCTA---------------	109	-18	0.04%
GGAATCTGC--------AGGTGGGGGT	107	-8	0.04%
GGAA-----------------------	101	-23	0.04%
GGAATCTGCCTAACA-GAGGTGGGGGT	97	-1	0.04%
GGAATCTGCCTAACAGGgAGGTGGGGGT	100	+1 g	0.04%

P168-YW-W9-Syn24crAAVS1g-KO-TSA-48h-BC13	Ins	Del	HDR
222443 | 23.83%	**2.52%**	**21.25%**	**0.05%**
**Typical seqs**	**Reads**	**Type**	**pct.**
GGAATCTGCCTAACAGG|AGG**TGG**GGGT	169445	WT	76.17%
GGAATCTGCCTAACA-GAGGTGGGGGT	11749	-1	5.28%
GGAATCTGCCTAACAGG-GGTGGGGGT	7606	-1	3.42%
GGAATCTGCCTAACAGGgAGGTGGGGGT	4841	+1 g	2.18%
GGAATCTGCC------AAGGTGGGGGT	3025	-6	1.36%
GGAATCTGCC-------AGGTGGGGGT	2905	-7	1.31%
GGAATCTGCCTAA----AGGTGGGGGT	2609	-4	1.17%
GGAATCTGC--------AGGTGGGGGT	2196	-8	0.99%
GGAATCTGCC----------------T	1935	-16	0.87%
GGAATCTGCCTAAC---AGGTGGGGGT	1775	-3	0.80%

P168-YW-W9-Syn24crAAVS1g-KO-TSA+Nu7026-48h-BC14	Ins	Del	HDR
52324 | 5.28%	**2.37%**	**2.75%**	**0.17%**
**Typical seqs**	**Reads**	**Type**	**pct.**
GGAATCTGCCTAACAGG|AGG**TGG**GGGT	49560	WT	94.72%
GGAATCTGCCTAACAGGCAGGAGGTGGGGGT	1034	+4	1.98%
GGAATCTGCCTAACA-GAGGTGGGGGT	987	-1	1.89%
GGAATCTGCCTAAGTTTAAACTACGCGTGGGT	89	+5 HDR	0.17%
GGAATCTGCCTAACAGG-GGTGGGGGT	55	-1	0.11%
GGAATCTGCCTAACAAGGAGGTGGGGGT	51	+1 Ins	0.10%
GGAATCTGCCTAAC-------------	38	-14	0.07%
GGAATCTGC--------AGGTGGGGGT	29	-8	0.06%
GGAATCTGCCTAAC---AGGTGGGGGT	28	-3	0.05%
GGAATCTGCCTAA----AGGTGGGGGT	27	-4	0.05%

P168-YW-W9-Syn24crAAVS1g-KO-TSA+NaB+M3814-48h-BC15	Ins	Del	HDR
159476 | 2.76%	**0.10%**	**2.52%**	**0.14%**
**Typical seqs**	**Reads**	**Type**	**pct.**
GGAATCTGCCTAACAGG|AGG**TGG**GGGT	155072	WT	97.24%
GGAATCT------------GTGGGGGT	2669	-12	1.67%
GGAATCTGCCTAAGTTTAAACTACGCGTGGGT	221	+5 HDR	0.14%
GGAATCTGCCTAACAGG-GGTGGGGGT	139	-1	0.09%
GGAATCTGCCTAAC-------------	88	-14	0.06%
GGAATCTGCCTAA----AGGTGGGGGT	86	-4	0.05%
GGAATCTGCCTAAC---AGGTGGGGGT	72	-3	0.05%
GGAATCTGCCTAACA-GAGGTGGGGGT	77	-1	0.05%
GGAATCTGC--------AGGTGGGGGT	73	-8	0.05%
GGAATCTGCCTA---------------	64	-18	0.04%

P168-P169-YW-W9-Syn24crAAVS1g-AAVSKI

P169-YW-W9-Syn24crAAVS1g-AAV6KI-Ctrl-48h-BC1	Ins	Del	HDR
136372 | 25.17%	**3.59%**	**13.14%**	**8.44%**
**Typical seqs**	**Reads**	**Type**	**pct.**
GGAATCTGCCTAACAGG|AGG**TGG**GGGT	102048	WT	74.83%
GGAATCTGCCTAAGTTTAAACTACGCGTGGGT	10751	+5 HDR	7.88%
GGAATCTGCCTAACAGG-GGTGGGGGT	2827	-1	2.07%
GGAATCTGCCTAACAGGgAGGTGGGGGT	2529	+1 g	1.85%
GGAATCTGCCTAAC---AGGTGGGGGT	1725	-3	1.26%
GGAATCTGC--------AGGTGGGGGT	1459	-8	1.07%
GGAATCTGCCTAA----AGGTGGGGGT	1193	-4	0.87%
GGAATCTGCCTA-----AGGTGGGGGT	1044	-5	0.77%
GGAATCTGCCTAACA-GAGGTGGGGGT	951	-1	0.70%
GGAATCTGCCTAACA--AGGTGGGGGT	734	-2	0.54%

P169-YW-W9-Syn24crAAVS1g-AAV6KI-M3814-48h-BC5	Ins	Del	HDR
262362 | 21.11%	**1.14%**	**4.31%**	**15.66%**
**Typical seqs**	**Reads**	**Type**	**pct.**
GGAATCTGCCTAACAGG|AGG**TGG**GGGT	206984	WT	78.89%
GGAATCTGCCTAAGTTTAAACTACGCGTGGGT	38421	+5 HDR	14.64%
GGAATCTGCCTAAC---AGGTGGGGGT	3214	-3	1.23%
GGAATCT----------------GGGT	2626	-16	1.00%
GGAATCTGCCTAACAGG-GGTGGGGGT	1953	-1	0.74%
GGAATCTGCCTAACA------GGGGGT	1589	-6	0.61%
GGAATCTGCCTAACAGGgAGGTGGGGGT	270	+1 g	0.10%
GGAATCTGCCTAA------<br>GTTTAAACTGCGCGTGGGT	233	+5	0.09%
GGAATCTGCCTAAGTTTAGACTACGCGTGGGT	189	+5	0.07%
GGAATCTGCCTAACA-GAGGTGGGGGT	189	-1	0.07%

P169-YW-W9-Syn24crAAVS1g-AAV6KI-M3814+Nu7026-48h-BC6	Ins	Del	HDR
251921 | 15.36%	**2.25%**	**3.62%**	**9.49%**
**Typical seqs**	**Reads**	**Type**	**pct.**
GGAATCTGCCTAACAGG|AGG**TGG**GGGT	213231	WT	84.64%
GGAATCTGCCTAAGTTTAAACTACGCGTGGGT	22347	+5 HDR	8.87%
GGAATCTGCCTAACAGGgAGGTGGGGGT	3535	+1 g	1.40%
GGAATCTGCCTAACA--AGGTGGGGGT	3154	-2	1.25%
GGAATCTGCCTAAC---AGGTGGGGGT	1605	-3	0.64%
GGAATCTGCCTAACAG--GGTGGGGGT	2261	-2	0.90%
GGAATCTGCCTAACAGG-GGTGGGGGT	310	-1	0.12%
GGAATCTGCCTAAGTTTGAACTACGCGTGGGT	246	+5	0.10%
GGAATCTGCCTAACA-GAGGTGGGGGT	171	-1	0.07%
GGAATCTGCCTAAGTTTAGACTACGCGTGGGT	133	+5	0.05%

P169-YW-W9-Syn24crAAVS1g-AAV6KI-M3814+TSA-48h-BC7	Ins	Del	HDR
255468 | 46.08%	**2.58%**	**4.33%**	**39.18%**
**Typical seqs**	**Reads**	**Type**	**pct.**
GGAATCTGCCTAACAGG|AGG**TGG**GGGT	137742	WT	53.92%
GGAATCTGCCTAAGTTTAAACTACGCGTGGGT	93034	+5 HDR	36.42%
GGAATCTGCCTAAC---AGGTGGGGGT	5578	-3	2.18%
GGAATCTGCCTAACAGG-GGTGGGGGT	2008	-1	0.79%
GGAATCTGCCTAACA-GAGGTGGGGGT	1459	-1	0.57%
GGAATCTGCCTAAGTTTAGACTACGCGTGGGT	553	+5	0.22%
GGAATCTGCCTAAGCTTAAACTACGCGTGGGT	402	+5	0.16%
GGAATCTGCCTAAGTTTAAACTACGCGCGGGT	401	+5	0.16%
GGAATCTGCCTAAGTTTAAGCTACGCGTGGGT	387	+5	0.15%
GGAATCTGCCTAAGTCTAAACTACGCGTGGGT	366	+5	0.14%

P169-YW-W9-Syn24crAAVS1g-AAV6KI-M3814+TSA+Nu7026-48h-BC8	Ins	Del	HDR
194661 | 43.49%	**4.08%**	**2.56%**	**36.85%**
**Typical seqs**	**Reads**	**Type**	**pct.**
GGAATCTGCCTAACAGG|AGG**TGG**GGGT	109996	WT	56.51%
GGAATCTGCCTAAGTTTAAACTACGCGTGGGT	67362	+5 HDR	34.60%
GGAATCTGCCTAACAGGgAGGTGGGGGT	2927	+1 g	1.50%
GGAATCTGCCT------AGGTGGGGGT	1886	-6	0.97%
GGAATCTGCCTAAC---AGGTGGGGGT	1724	-3	0.89%
GGAATCTGCCTAA------<br>GTTTAAACTGCGCGTGGGT	461	+5	0.24%
GGAATCTGCCTAAGTTTAAACTACGCGCGGGT	331	+5	0.17%
GGAATCTGCCTAAGTTTAGACTACGCGTGGGT	320	+5	0.16%
GGAATCTGCCTAAGCTTAAACTACGCGTGGGT	277	+5	0.14%
GGAATCTGCCTAAGTTTAAGCTACGCGTGGGT	229	+5	0.12%

P168-P169-YW-W9-Syn24crAAVS1g-AAVSKI

P169-YW-W9-Syn24crAAVS1g-AAV6KI-NaB-48h-BC9	Ins	Del	HDR
174397 | 40.10%	3.22%	18.31%	18.57%
**Typical seqs**	**Reads**	**Type**	**pct.**
GGAATCTGCCTAACAGG|AGG**TGG**GGGT	104458	WT	59.90%
GGAATCTGCCTAAGTTTAAACTACGCGTGGGT	30196	+5 HDR	17.31%
GGAATCTGCCTAAC---AGGTGGGGGT	5850	-3	3.35%
GGAATCTGCCTAACAGG-GGTGGGGGT	4604	-1	2.64%
GGAATCTGCCTAACA-GAGGTGGGGGT	3543	-1	2.03%
GGAATCTGCCTAACAG--GGTGGGGGT	3333	-2	1.91%
GGAATCTGCCTAACAGGgAGGTGGGGGT	3131	+1 g	1.80%
GGAATCTGCCTA-----AGGTGGGGGT	2913	-5	1.67%
GGAATCTGCCTAA----AGGTGGGGGT	1890	-4	1.08%
GGAATCTGC--------AGGTGGGGGT	1130	-8	0.65%

P169-YW-W9-Syn24crAAVS1g-AAV6KI-NaB+M3814-48h-BC11	Ins	Del	HDR
262246 | 54.71%	4.27%	6.20%	44.24%
**Typical seqs**	**Reads**	**Type**	**pct.**
GGAATCTGCCTAACAGG|AGG**TGG**GGGT	118761	WT	45.29%
GGAATCTGCCTAAGTTTAAACTACGCGTGGGT	107534	+5 HDR	41.01%
GGAATCTGCCTA-----AGGTGGGGGT	5373	-5	2.05%
GGAATCTGCCTAACAGG-GGTGGGGGT	4314	-1	1.65%
GGAATCTGCCTAACAG--GGTGGGGGT	4026	-2	1.54%
GGAATCTGCCTAACAGGgAGGTGGGGGT	2607	+1 g	0.99%
GGAATCTGCCTAA------GTTTAAACTACACGTGGGT	822	+5	0.31%
GGAATCTGCCTAAGTTTAGACTACGCGTGGGT	724	+5	0.28%
GGAATCTGCCTAAGTTCAAACTACGCGTGGGT	547	+5	0.21%
GGAATCTGCCTAAGTTTAAGCTACGCGTGGGT	531	+5	0.20%

P169-YW-W9-Syn24crAAVS1g-AAV6KI-NaB+Nu7026-48h-BC10	Ins	Del	HDR
171368 | 36.16%	4.38%	9.54%	22.25%
**Typical seqs**	**Reads**	**Type**	**pct.**
GGAATCTGCCTAACAGG|AGG**TGG**GGGT	109395	WT	63.84%
GGAATCTGCCTAAGTTTAAACTACGCGTGGGT	35585	+5 HDR	20.77%
GGAATCTGCCTAAC---AGGTGGGGGT	3034	-3	1.77%
GGAATCTGCCTAACAGGgAGGTGGGGGT	2485	+1 g	1.45%
GGAATCTGCCTAACAGG-GGTGGGGGT	2514	-1	1.47%
GGAATCTGCCTAACAG--GGTGGGGGT	2164	-2	1.26%
GGAATCTGCCTAACAGGaAGGTGGGGGT	1570	+1 a	0.92%
GGAATCTGCCTAA---AAGGTGGGGGT	1199	-3	0.70%
GGAAT-------------GGTGGGGGT	1147	-13	0.67%
GGAATCT-----------GGTGGGGGT	1108	-11	0.65%

P169-YW-W9-Syn24crAAVS1g-AAV6KI-NaB+TSA-48h-BC4	Ins	Del	HDR
300512 | 43.66%	5.42%	18.09%	20.14%
**Typical seqs**	**Reads**	**Type**	**pct.**
GGAATCTGCCTAACAGG|AGG**TGG**GGGT	169313	WT	56.34%
GGAATCTGCCTAAGTTTAAACTACGCGTGGGT	56430	+5 HDR	18.78%
GGAATCTGCCTAACAGG-GGTGGGGGT	12143	-1	4.04%
GGAATCTGCCTAACAGGgAGGTGGGGGT	8046	+1 g	2.68%
GGAATCTGCCTAACA-GAGGTGGGGGT	5592	-1	1.86%
GGAATCTGCCTAAC---AGGTGGGGGT	5146	-3	1.71%
GGAATCTGCCTAA----AGGTGGGGGT	4565	-4	1.52%
GGAATCTGCCTAACA--AGGTGGGGGT	3091	-2	1.03%
GGAATCTGCCTA-----AGGTGGGGGT	3082	-5	1.03%
GGAATCTGCCTAACAG--GGTGGGGGT	2534	-2	0.84%

P169-YW-W9-Syn24crAAVS1g-AAV6KI-NaB+TSA+Nu7026-48h-BC12	Ins	Del	HDR
283308 | 39.76%	6.64%	7.25%	25.87%
**Typical seqs**	**Reads**	**Type**	**pct.**
GGAATCTGCCTAACAGG|AGG**TGG**GGGT	170667	WT	60.24%
GGAATCTGCCTAAGTTTAAACTACGCGTGGGT	68365	+5 HDR	24.13%
GGAATCTGCCTAACAGGgAGGTGGGGGT	11849	+1 g	4.18%
GGAATCTGCCTAACAGG-GGTGGGGGT	5451	-1	1.92%
GGAATCTGCCTAACA-GAGGTGGGGGT	4976	-1	1.76%
GGAATCT---------------GGGGT	4064	-15	1.43%
---------------AGGAGGTGGGGGT	1689	-15	0.60%
GGAATCTGCCT------AGGTGGGGGT	1604	-6	0.57%
GGAATCTGCCTAAGCTTAAACTACGCGTGGGT	602	+5	0.21%
GGAATCTGCCTAAATTTAAACTACGCGTGGGT	355	+5	0.13%

P174-P178-YW-iPSC-W9-Syn36crBCL11Gata-a5-4h-BC12

P174-YW-W9-Syn36crBCL11Gata-a5-4h-KO-BC5 4h	Ins	Del	HDR
133193 | 13.84%	7.95%	5.89%	0.00%
**Typical seqs**	**Reads**	**Type**	**pct.**
TTGCTTTTATCACAGGC|TCC**AGG**AAGG	114755	WT	86.16%
TTGCTTTTATCACAGGCcTCCAGGAAGG	6204	+1 c	4.66%
TTGCTTTTATCACAGGC-CCAGGAAGG	2403	-1	1.80%
TTGCTTTTATCACAGGCtTCCAGGAAGG	2040	+1 t	1.53%
TTGCTTTTATCACAGG--CCAGGAAGG	1063	-2	0.80%
TTGCTTTTATCACAGG-TCCAGGAAGG	997	-1	0.75%
TTGCTTTTA--------TCCAGGAAGG	835	-8	0.63%
TTGCTTTTATCA-------CAGGAAGG	663	-7	0.50%
TTGCTTTTATCACAGGCgTCCAGGAAGG	482	+1 g	0.36%
TTGCTTTTATCACAGGCTTTTATCCAGGAAGG	372	+5	0.28%

P175-YW-W9-Syn36crBCL11Gata-a5-8h-KO-BC5 8h	Ins	Del	HDR
36122 | 23.95%	12.00%	11.95%	0.00%
**Typical seqs**	**Reads**	**Type**	**pct.**
TTGCTTTTATCACAGGC|TCC**AGG**AAGG	27470	WT	76.05%
TTGCTTTTATCACAGGCcTCCAGGAAGG	2164	+1 c	5.99%
TTGCTTTTATCACAGGCtTCCAGGAAGG	1002	+1 t	2.77%
TTGCTTTTATCACAGGC-CCAGGAAGG	831	-1	2.30%
TTGCTTTTATCACAGG--CCAGGAAGG	565	-2	1.56%
TTGCTTTTATCA-------CAGGAAGG	475	-7	1.31%
TTGCTTTTATCACAGG-TCCAGGAAGG	423	-1	1.17%
TTGCTTTTATCACAGGCgTCCAGGAAGG	369	+1 g	1.02%
TTGCTTTTA--------TCCAGGAAGG	401	-8	1.11%
TTGCTTTTATCACAGGC-------AGG	242	-7	0.67%

P176-YW-W9-Syn36crBCL11Gata-a5-12h-KO-BC5 12h	Ins	Del	HDR
18882 | 35.00%	15.28%	19.72%	0.00%
**Typical seqs**	**Reads**	**Type**	**pct.**
TTGCTTTTATCACAGGC|TCC**AGG**AAGG	12273	WT	65.00%
TTGCTTTTATCACAGGCcTCCAGGAAGG	1529	+1 c	8.10%
TTGCTTTTATCACAGGCtTCCAGGAAGG	745	+1 t	3.95%
TTGCTTTTATCACAGGC-CCAGGAAGG	712	-1	3.77%
TTGCTTTTATCACAGG-TCCAGGAAGG	400	-1	2.12%
TTGCTTTTATCACAGG--CCAGGAAGG	337	-2	1.78%
TTGCTTTTATCA-------CAGGAAGG	350	-7	1.85%
TTGCTTTTA--------TCCAGGAAGG	284	-8	1.50%
TTGCTTTTATCACAGGC------AAGG	143	-6	0.76%
TTGCTTTTATCACAGGC-----GAAGG	118	-5	0.62%

P177-YW-W9-Syn36crBCL11Gata-a5-24h-KO-BC5 24h	Ins	Del	HDR
9401 | 49.53%	15.85%	33.68%	0.00%
**Typical seqs**	**Reads**	**Type**	**pct.**
TTGCTTTTATCACAGGC|TCC**AGG**AAGG	4745	WT	50.47%
TTGCTTTTATCACAGGCcTCCAGGAAGG	764	+1 c	8.13%
TTGCTTTTATCA-------CAGGAAGG	744	-7	7.91%
TTGCTTTTA--------TCCAGGAAGG	582	-8	6.19%
TTGCTTTTATCACAGGC-CCAGGAAGG	405	-1	4.31%
TTGCTTTTATCACAGGCtTCCAGGAAGG	413	+1 t	4.39%
TTGCTTTTATCACAGG--CCAGGAAGG	251	-2	2.67%
TTGCTTTTATCACAGG-TCCAGGAAGG	155	-1	1.65%
TTGCTTTTATCACAGGC-----GAAGG	77	-5	0.82%
TTGCTTTTATCACAGGCaTCCAGGAAGG	63	+1 a	0.67%

P178-YW-W9-Syn36crBCL11Gata-a5-48h-KO-BC5 48h	Ins	Del	HDR
1250 | 50.32%	12.72%	37.60%	0.00%
**Typical seqs**	**Reads**	**Type**	**pct.**
TTGCTTTTATCACAGGC|TCC**AGG**AAGG	621	WT	49.68%
TTGCTTTTATCA-------CAGGAAGG	168	-7	13.44%
TTGCTTTTA--------TCCAGGAAGG	114	-8	9.12%
TTGCTTTTATCACAGGCcTCCAGGAAGG	79	+1 c	6.32%
TTGCTTTTATCACAGGCtTCCAGGAAGG	53	+1 t	4.24%
TTGCTTTTATCACAGGC-CCAGGAAGG	40	-1	3.20%
TTGCTTTTATCACAGG--CCAGGAAGG	35	-2	2.80%
TTGCTTTTATCACAGG-TCCAGGAAGG	18	-1	1.44%
TTGCTTTTATCACAG----------GG	12	-10	0.96%
TTGCTTTTATCACAGGCTTTTATCCAGGAAGG	11	+5	0.88%

P184-YW-W9-S93crB7c-ko

P184-YW-W9-S93crB7c-ko-4h-BC1 4h	Ins	Del	HDR
47260 | 3.02%	1.90%	1.12%	0.00%
**Typical seqs**	**Reads**	**Type**	**pct.**
AGCTTCGTGACGTTGGA|TGG**AGG**GTTT	45835	WT	96.98%
AGCTTCGTGACGTTGGAaTGGAGGGTTT	806	+1 a	1.71%
AGCTTCGTGACGTTGGA-GGAGGGTTT	202	-1	0.43%
AGCTTCGTGACGT----TGGAGGGTTT	146	-4	0.31%
AGCTTCGTGACGTTGG-TGGAGGGTTT	60	-1	0.13%
AGCTTCG----------TGGAGGGTTT	16	-10	0.03%
AGCTTCGTGACGTT-------GGGTTT	15	-7	0.03%
AGCTTCGTGACGTTGGAtTGGAGGGTTT	12	+1 t	0.03%
AGCTTCGTGACGTTGGAT-GAGGGTTT	12	-1	0.03%
AGCTTCGTGAC------TGGAGGGTTT	11	-6	0.02%

P184-YW-W9-S93crB7c-ko-8h-BC2 8h	Ins	Del	HDR
51276 | 8.23%	5.05%	3.18%	0.00%
**Typical seqs**	**Reads**	**Type**	**pct.**
AGCTTCGTGACGTTGGA|TGG**AGG**GTTT	47057	WT	91.77%
AGCTTCGTGACGTTGGAaTGGAGGGTTT	2130	+1 a	4.15%
AGCTTCGTGACGT----TGGAGGGTTT	601	-4	1.17%
AGCTTCGTGACGTTGGA-GGAGGGTTT	325	-1	0.63%
AGCTTCGTGACGTTGGAtTGGAGGGTTT	159	+1 t	0.31%
AGCTTCGTGACGTTGG-TGGAGGGTTT	99	-1	0.19%
AGCTTCGTGACGTTG--TGGAGGGTTT	64	-2	0.12%
AGCTTCGTGACGTTGGA--GAGGGTTT	52	-2	0.10%
AGCTTCG----------TGGAGGGTTT	45	-10	0.09%
AGCTTCGTGACGTT-GATGGAGGGTTT	37	-1	0.07%

P184-YW-W9-S93crB7c-ko-12h-BC3 12h	Ins	Del	HDR
49096 | 13.49%	6.76%	6.74%	0.00%
**Typical seqs**	**Reads**	**Type**	**pct.**
AGCTTCGTGACGTTGGA|TGG**AGG**GTTT	42471	WT	86.51%
AGCTTCGTGACGTTGGAaTGGAGGGTTT	2883	+1 a	5.87%
AGCTTCGTGACGT----TGGAGGGTTT	1500	-4	3.06%
AGCTTCGTGACGTTGGA-GGAGGGTTT	648	-1	1.32%
AGCTTCG----------TGGAGGGTTT	107	-10	0.22%
AGCTTCGTGACGTTG--TGGAGGGTTT	98	-2	0.20%
AGCTTCGTGACGTTGGAtTGGAGGGTTT	90	+1 t	0.18%
AGCTTCGTGACGTT--ATGGAGGGTTT	61	-2	0.12%
AGCTTCGTGAC------------GTTT	58	-12	0.12%
AGCTTCGTGACGTT--------GGTTT	55	-8	0.11%

P184-YW-W9-S93crB7c-ko-24h-BC4 24h	Ins	Del	HDR
58845 | 23.17%	8.20%	14.97%	0.00%
**Typical seqs**	**Reads**	**Type**	**pct.**
AGCTTCGTGACGTTGGA|TGG**AGG**GTTT	45212	WT	76.83%
AGCTTCGTGACGT----TGGAGGGTTT	4515	-4	7.67%
AGCTTCGTGACGTTGGAaTGGAGGGTTT	4114	+1 a	6.99%
AGCTTCGTGACGTTGGA-GGAGGGTTT	1410	-1	2.40%
AGCTTCGTGACGTT-------GGGTTT	233	-7	0.40%
AGCTTCG----------TGGAGGGTTT	165	-10	0.28%
AGCTTCGTGAC------------GTTT	146	-12	0.25%
AGCTTCGTGACGTTG--TGGAGGGTTT	121	-2	0.21%
AGCTTCGTGACGTTGGAtTGGAGGGTTT	117	+1 t	0.20%
AGCTTCGTGACGTT--------GGTTT	109	-8	0.19%

P184-YW-W9-S93crB7c-ko-48h-BC5 48h	Ins	Del	HDR
61030 | 26.20%	7.80%	18.40%	0.00%
**Typical seqs**	**Reads**	**Type**	**pct.**
AGCTTCGTGACGTTGGA|TGG**AGG**GTTT	45043	WT	73.80%
AGCTTCGTGACGT----TGGAGGGTTT	6118	-4	10.02%
AGCTTCGTGACGTTGGAaTGGAGGGTTT	3968	+1 a	6.50%
AGCTTCGTGACGTTGGA-GGAGGGTTT	1295	-1	2.12%
AGCTTCGTGAC------------GTTT	301	-12	0.49%
AGCTTCGTGACGTT-------GGGTTT	236	-7	0.39%
AGCTTCGTGACGTTGG-TGGAGGGTTT	182	-1	0.30%
AGCTTCGTGACGTT--------GGTTT	167	-8	0.27%
AGCTTCG----------TGGAGGGTTT	136	-10	0.22%
AGCTTCGT-----------GAGGGTTT	120	-11	0.20%

P184-YW-W9-S95crB8a-ko

P184-YW-W9-S95crB8a-ko-4h-BC1 4h 71848 | 1.61%	Ins 1.39%	Del 0.22%	HDR 0.00%
**Typical seqs**	**Reads**	**Type**	**pct.**
GCTTCTGAGCGTCAAAC|TCG**AGG**AGCA	70693	WT	98.39%
GCTTCTGAGCGTCAAACcTCGAGGAGCA	811	+1 c	1.13%
GCTTCTGAGCGTCAAACtTCGAGGAGCA	71	+1 t	0.10%
GCTTCTGAGCG------TCGAGGAGCA	33	-6	0.05%
GCTTCTGAGCGTC-AACTCGAGGAGCA	28	-1	0.04%
GCTTCTGAGCGTCAAACACTCGAGGAGCA	26	+2	0.04%
GCTTCTGAGCGTCAAAC-CGAGGAGCA	16	-1	0.02%
GCTTCTGAGCGTCAA--TCGAGGAGCA	15	-2	0.02%
GCTTCTGAGCGTCAAA-TCGAGGAGCA	9	-1	0.01%
GCTTCTGAGCGTCAATGCTTCGAGGAGCA	8	+2	0.01%

P184-YW-W9-S95crB8a-ko-8h-BC2 8h 65745 | 5.17%	Ins 4.15%	Del 1.03%	HDR 0.00%
**Typical seqs**	**Reads**	**Type**	**pct.**
GCTTCTGAGCGTCAAAC|TCG**AGG**AGCA	62343	WT	94.83%
GCTTCTGAGCGTCAAACcTCGAGGAGCA	2073	+1 c	3.15%
GCTTCTGAGCGTCAAACtTCGAGGAGCA	359	+1 t	0.55%
GCTTCTGAGCG------TCGAGGAGCA	110	-6	0.17%
GCTTCTGAGCGTCAAAC-CGAGGAGCA	106	-1	0.16%
GCTTCTGAGCGTCAAA-TCGAGGAGCA	73	-1	0.11%
GCT--------------TCGAGGAGCA	61	-14	0.09%
GCTTCT----------------GAGCA	43	-16	0.07%
GCTTCTGAGCGTCAAACACTCGAGGAGCA	39	+2	0.06%
GCTTCTGAGCGTC-AACTCGAGGAGCA	37	-1	0.06%

P184-YW-W9-S95crB8a-ko-12h-BC3 12h 62360 | 6.88%	Ins 4.63%	Del 2.24%	HDR 0.00%
**Typical seqs**	**Reads**	**Type**	**pct.**
GCTTCTGAGCGTCAAAC|TCG**AGG**AGCA	58072	WT	93.12%
GCTTCTGAGCGTCAAACcTCGAGGAGCA	2123	+1 c	3.40%
GCTTCTGAGCGTCAAACtTCGAGGAGCA	391	+1 t	0.63%
GCTTCTGAGCG------TCGAGGAGCA	328	-6	0.53%
GCT--------------TCGAGGAGCA	95	-14	0.15%
GCTTCT----------------GAGCA	83	-16	0.13%
GCTTCTGAGCGTCAAAC-CGAGGAGCA	79	-1	0.13%
GCTTCTGAGCGTC-AACTCGAGGAGCA	60	-1	0.10%
GCTTCTGAGCGTCAAACACTCGAGGAGCA	52	+2	0.08%
GCTTCTGAGCGTCAAACgTCGAGGAGCA	48	+1 g	0.08%

P184-YW-W9-S95crB8a-ko-24h-BC4 24h 68281 | 13.51%	Ins 7.28%	Del 6.22%	HDR 0.00%
**Typical seqs**	**Reads**	**Type**	**pct.**
GCTTCTGAGCGTCAAAC|TCG**AGG**AGCA	59058	WT	86.49%
GCTTCTGAGCGTCAAACcTCGAGGAGCA	3323	+1 c	4.87%
GCTTCTGAGCG------TCGAGGAGCA	903	-6	1.32%
GCTTCTGAGCGTCAAACtTCGAGGAGCA	809	+1 t	1.18%
GCT--------------TCGAGGAGCA	336	-14	0.49%
GCTTCT----------------GAGCA	307	-16	0.45%
GCTTCT-------------GAGGAGCA	144	-13	0.21%
GCTTCTGAGCGTC-AACTCGAGGAGCA	130	-1	0.19%
GCTTCTGAG---------CGAGGAGCA	120	-9	0.18%
GCTTCTGAGCGTCAAA-TCGAGGAGCA	115	-1	0.17%

P184-YW-W9-S95crB8a-ko-48h-BC5 48h 75183 | 17.15%	Ins 7.82%	Del 9.32%	HDR 0.00%
**Typical seqs**	**Reads**	**Type**	**pct.**
GCTTCTGAGCGTCAAAC|TCG**AGG**AGCA	62292	WT	82.85%
GCTTCTGAGCGTCAAACcTCGAGGAGCA	3705	+1 c	4.93%
GCTTCTGAGCG------TCGAGGAGCA	1745	-6	2.32%
GCTTCTGAGCGTCAAACtTCGAGGAGCA	1128	+1 t	1.50%
GCTTCT----------------GAGCA	588	-16	0.78%
GCT--------------TCGAGGAGCA	541	-14	0.72%
GCTTCTGAGCGTCAAAC-CGAGGAGCA	218	-1	0.29%
GCTTCTGAGCGT----------------	236	-16	0.31%
GCTTCTGAG---------CGAGGAGCA	191	-9	0.25%
GCTTCT-------------GAGGAGCA	179	-13	0.24%

P185-YW-W9-S97crB8c-ko

P185-YW-W9-S97crB8c-ko-4h-BC1 4h<br>54769 | 0.79%	Ins<br>0.31%	Del<br>0.48%	HDR<br>0.00%
**Typical seqs**	**Reads**	**Type**	**pct.**
CGCGGCAGAAACCTGTC|GTC**TGG**ACAC	54338	WT	99.21%
CGCGGCAGAAACCTGTCcGTCTGGACAC	147	+1 c	0.27%
CGCGGCAGAAACCTGTCGTCT-GACAC	86	-1	0.16%
CGCGGCAGAAACCT---GTCTGGACAC	77	-3	0.14%
CGCGGCAGAAAC----CGTCTGGACAC	25	-4	0.05%
CGCGGCAGAAACCTGT-GTCTGGACAC	16	-1	0.03%
CGCGGCAGAAACCTGTCtGTCTGGACAC	12	+1 t	0.02%
CGCGGCAGAAACCTG--GTCTGGACAC	11	-2	0.02%
CGCGGCAGAAACC--TCGTCTGGACAC	10	-2	0.02%
CGCGGCAGAAACCT-------------	9	-15	0.02%

P185-YW-W9-S97crB8c-ko-8h-BC2 8h<br>64954 | 2.10%	Ins<br>0.74%	Del<br>1.36%	HDR<br>0.00%
**Typical seqs**	**Reads**	**Type**	**pct.**
CGCGGCAGAAACCTGTC|GTC**TGG**ACAC	63590	WT	97.90%
CGCGGCAGAAACCTGTCcGTCTGGACAC	391	+1 c	0.60%
CGCGGCAGAAACCT---GTCTGGACAC	300	-3	0.46%
CGCGGCAGAAACCTGTCGTCT-GACAC	83	-1	0.13%
CGCGGCAGAAACCTGT-GTCTGGACAC	41	-1	0.06%
CGCGGCAGAAACCT-------------	34	-15	0.05%
CGCGGCAGAAAC----CGTCTGGACAC	33	-4	0.05%
CGCGGCAGAAA-----CGTCTGGACAC	28	-5	0.04%
CGCGGCAGAAACCTGTCaGTCTGGACAC	27	+1 a	0.04%
CGCGGCAG---------GTCTGGACAC	26	-9	0.04%

P185-YW-W9-S97crB8c-ko-12h-BC3 12h<br>62514 | 3.54%	Ins<br>0.94%	Del<br>2.60%	HDR<br>0.00%
**Typical seqs**	**Reads**	**Type**	**pct.**
CGCGGCAGAAACCTGTC|GTC**TGG**ACAC	60302	WT	96.46%
CGCGGCAGAAACCT---GTCTGGACAC	903	-3	1.44%
CGCGGCAGAAACCTGTCcGTCTGGACAC	476	+1 c	0.76%
CGCGGCAGAAACCTGTCGTCT-GACAC	85	-1	0.14%
CGCGGCAGAAAC----CGTCTGGACAC	56	-4	0.09%
CGCGGCAGAAACCT-------------	46	-15	0.07%
CGCGGCAGAAACCTGT-GTCTGGACAC	37	-1	0.06%
CGCGGCAGAAACCTGTCtGTCTGGACAC	36	+1 t	0.06%
CGCGGCAGAAACCTG--GTCTGGACAC	35	-2	0.06%
CGCGGCAGAAACCTG-CGTCTGGACAC	32	-1	0.05%

P185-YW-W9-S97crB8c-ko-24h-BC4 24h<br>58103 | 8.68%	Ins<br>1.57%	Del<br>7.11%	HDR<br>0.00%
**Typical seqs**	**Reads**	**Type**	**pct.**
CGCGGCAGAAACCTGTC|GTC**TGG**ACAC	53062	WT	91.32%
CGCGGCAGAAACCT---GTCTGGACAC	2214	-3	3.81%
CGCGGCAGAAACCTGTCcGTCTGGACAC	650	+1 c	1.12%
CGCGGCAGAAAC-------CTGGACAC	275	-7	0.47%
CGCGGCAGAAACCT-------------	137	-15	0.24%
CGCGGCAGAAACCTGT-GTCTGGACAC	101	-1	0.17%
CGCGGCAGAAACCTGTCGTCT-GACAC	81	-1	0.14%
CGCGGCAGAAACCTGTC-TCTGGACAC	75	-1	0.13%
CGCGGCAGAAACCTG--GTCTGGACAC	60	-2	0.10%
CGCGGCAGAAAC----CGTCTGGACAC	57	-4	0.10%

P185-YW-W9-S97crB8c-ko-48h-BC5 48h<br>70930 | 10.46%	Ins<br>1.25%	Del<br>9.21%	HDR<br>0.00%
**Typical seqs**	**Reads**	**Type**	**pct.**
CGCGGCAGAAACCTGTC|GTC**TGG**ACAC	63511	WT	89.54%
CGCGGCAGAAACCT---GTCTGGACAC	3686	-3	5.20%
CGCGGCAGAAACCTGTCcGTCTGGACAC	623	+1 c	0.88%
CGCGGCAGAAAC-------CTGGACAC	535	-7	0.75%
CGCGGCAGAAACCT-------------	169	-15	0.24%
CGCGGCAGAAACCTGT-GTCTGGACAC	149	-1	0.21%
CGCGGCAGAAAC----CGTCTGGACAC	98	-4	0.14%
CGCGGCAGAAACCTG--GTCTGGACAC	74	-2	0.10%
CGCGGCAGAA-------------ACAC	71	-13	0.10%
CGCGGCAGAAACCTGTC-TCTGGACAC	70	-1	0.10%

P185-YW-W9-S98crB8d-ko

P185-YW-W9-S98crB8d-ko-4h-BC1 4h	Ins	Del	HDR
65699 | 1.75%	**1.32%**	**0.44%**	**0.00%**
**Typical seqs**	**Reads**	**Type**	**pct.**
GCCTTGATTACTCAAAC|AAC**AGG**TTAC	64548	WT	98.25%
GCCTTGATTACTCAAACcAACAGGTTAC	789	+1 c	1.20%
GCCTTGATTACTCA---AACAGGTTAC	89	-3	0.14%
GCCTTGATTACTCAA--AACAGGTTAC	41	-2	0.06%
GCCTTGATTACT----CAACAGGTTAC	37	-4	0.06%
GCCTTGATTACTC-AACAACAGGTTAC	23	-1	0.04%
GCCTTGATTACTC---CAACAGGTTAC	22	-3	0.03%
GCCTTGATTACTCAAA-AACAGGTTAC	19	-1	0.03%
GCCTTGATTACTCAAACaAACAGGTTAC	19	+1 a	0.03%
GCCTTGATTACTC--ACAACAGGTTAC	10	-2	0.02%

P185-YW-W9-S98crB8d-ko-8h-BC2 8h	Ins	Del	HDR
63729 | 3.54%	**2.21%**	**1.33%**	**0.00%**
**Typical seqs**	**Reads**	**Type**	**pct.**
GCCTTGATTACTCAAAC|AAC**AGG**TTAC	61472	WT	96.46%
GCCTTGATTACTCAAACcAACAGGTTAC	1195	+1 c	1.88%
GCCTTGATTACTCA---AACAGGTTAC	205	-3	0.32%
GCCTTGATTACTCAA--AACAGGTTAC	79	-2	0.12%
GCCTTGATTACTCAAA-AACAGGTTAC	77	-1	0.12%
GCCTTGATTACTC-AACAACAGGTTAC	47	-1	0.07%
GCCTTGATTACT----CAACAGGTTAC	49	-4	0.08%
GCCTTGATTACTC--ACAACAGGTTAC	45	-2	0.07%
GCCTTGATTACTC---CAACAGGTTAC	32	-3	0.05%
GCCTTGATTACTCAAACaAACAGGTTAC	28	+1 a	0.04%

P185-YW-W9-S98crB8d-ko-12h-BC3 12h	Ins	Del	HDR
58276 | 6.54%	**3.17%**	**3.37%**	**0.00%**
**Typical seqs**	**Reads**	**Type**	**pct.**
GCCTTGATTACTCAAAC|AAC**AGG**TTAC	54465	WT	93.46%
GCCTTGATTACTCAAACcAACAGGTTAC	1596	+1 c	2.74%
GCCTTGATTACTCA---AACAGGTTAC	559	-3	0.96%
GCCTTGATTACT----CAACAGGTTAC	195	-4	0.33%
GCCTTGATTACTCAA--AACAGGTTAC	111	-2	0.19%
GCCTTGATTACTC-AACAACAGGTTAC	80	-1	0.14%
GCCTTGATTACTCAA----------AC	71	-10	0.12%
GCCTTGATTACTCAAA-AACAGGTTAC	70	-1	0.12%
GCCTTGATTACTCAAAC-ACAGGTTAC	68	-1	0.12%
GCCTTGA----------------TTAC	63	-16	0.11%

P185-YW-W9-S98crB8d-ko-24h-BC4 24h	Ins	Del	HDR
46724 | 18.52%	**6.03%**	**12.48%**	**0.00%**
**Typical seqs**	**Reads**	**Type**	**pct.**
GCCTTGATTACTCAAAC|AAC**AGG**TTAC	38073	WT	81.48%
GCCTTGATTACTCAAACcAACAGGTTAC	2316	+1 c	4.96%
GCCTTGATTACTCA---AACAGGTTAC	2281	-3	4.88%
GCCTTGA----------------TTAC	374	-16	0.80%
GCCTTGATTACT----CAACAGGTTAC	351	-4	0.75%
GCCTTGATTACTCAA--AACAGGTTAC	211	-2	0.45%
GCCTTGATTACTCA-------------	176	-14	0.38%
GCCTTGATT---------ACAGGTTAC	141	-9	0.30%
GCCTTGATTACTCAA----------AC	144	-10	0.31%
GCCTTGATTACTC--------------	196	-16	0.42%

P185-YW-W9-S98crB8d-ko-48h-BC5 48h	Ins	Del	HDR
63248 | 27.07%	**6.49%**	**20.58%**	**0.00%**
**Typical seqs**	**Reads**	**Type**	**pct.**
GCCTTGATTACTCAAAC|AAC**AGG**TTAC	46129	WT	72.93%
GCCTTGATTACTCA---AACAGGTTAC	4634	-3	7.33%
GCCTTGATTACTCAAACcAACAGGTTAC	3287	+1 c	5.20%
GCCTTGA----------------TTAC	1155	-16	1.83%
GCCTTGATTACT----CAACAGGTTAC	746	-4	1.18%
GCCTTGATTACTCA-------------	543	-14	0.86%
GCCTTGATT---------ACAGGTTAC	492	-9	0.78%
GCCTTGATTACTCAA--AACAGGTTAC	413	-2	0.65%
GCCTTGATTACT-------CAGGTTAC	284	-7	0.45%
GCCTTGATTACTC--------------	513	-16	0.81%

P197-P201-YW-W3-Syn52crGATA4b-RNPko

P197-YW-W3-Syn52crGATA4b-RNPko-4h-BC2 4h<br>42723 | 1.08%	Ins<br>0.49%	Del<br>0.59%	HDR<br>0.00%
**Typical seqs**	**Reads**	**Type**	**pct.**
TCAAATTCCTGCACGGA|CCT**GGG**ACTT	42261	WT	98.92%
TCAAATTCCTGCACGGAaCCTGGGACTT	146	+1 a	0.34%
TCAAATTCCTGCAC-------GGACTT	55	-7	0.13%
TCAAATT----------CCTGGGACTT	43	-10	0.10%
TCAAATTCCTGCACGGA-CTGGGACTT	33	-1	0.08%
TCAAATTCCTGCACGGA---GGGACTT	28	-3	0.07%
TCAAATTCCTGCACGGA--TGGGACTT	24	-2	0.06%
TCAAATTCCTGCACGGAgCCTGGGACTT	19	+1 g	0.04%
TCAAATTCCTGCACGG-CCTGGGACTT	14	-1	0.03%
TCAAATTCCTGCACGGAcCCTGGGACTT	13	+1 c	0.03%

P198-YW-W3-Syn52crGATA4b-RNPko-8h-BC2 8h<br>62507 | 5.87%	Ins<br>1.04%	Del<br>4.83%	HDR<br>0.00%
**Typical seqs**	**Reads**	**Type**	**pct.**
TCAAATTCCTGCACGGA|CCT**GGG**ACTT	58836	WT	94.13%
TCAAATTCCTGCACGGA-CTGGGACTT	858	-1	1.37%
TCAAATTCCTGCAC-------GGACTT	410	-7	0.66%
TCAAATTCCTGCACGGA----------	334	-11	0.53%
TCAAATTCCTGCACGGAaCCTGGGACTT	315	+1 a	0.50%
TCAAATT----------CCTGGGACTT	304	-10	0.49%
TCAAATTCCTGCACGGAtCCTGGGACTT	169	+1 t	0.27%
TCAAATTCCTGCACGGA--TGGGACTT	159	-2	0.25%
TCAAATTCCTGCAC------GGGACTT	146	-6	0.23%
TCAAATTCCTGCACGG-CCTGGGACTT	117	-1	0.19%

P199-YW-W3-Syn52crGATA4b-RNPko-12h-BC2 12h<br>58802 | 4.85%	Ins<br>1.67%	Del<br>3.19%	HDR<br>0.00%
**Typical seqs**	**Reads**	**Type**	**pct.**
TCAAATTCCTGCACGGA|CCT**GGG**ACTT	55948	WT	95.15%
TCAAATTCCTGCACGGAaCCTGGGACTT	680	+1 a	1.16%
TCAAATTCCTGCACGGA-CTGGGACTT	381	-1	0.65%
TCAAATT----------CCTGGGACTT	357	-10	0.61%
TCAAATTCCTGCAC-------GGACTT	308	-7	0.52%
TCAAATTCCTGCACGGA---GGGACTT	123	-3	0.21%
TCAAATTCCTGCACGGAtCCTGGGACTT	74	+1 t	0.13%
TCAAATTCCTGCACGGA--TGGGACTT	66	-2	0.11%
TCAAATTCCTG------CCTGGGACTT	66	-6	0.11%
TCAAATTCCTGCACGG-CCTGGGACTT	56	-1	0.10%

P200-YW-W3-Syn52crGATA4b-RNPko-24h-BC2 24h<br>75115 | 8.06%	Ins<br>1.91%	Del<br>6.15%	HDR<br>0.00%
**Typical seqs**	**Reads**	**Type**	**pct.**
TCAAATTCCTGCACGGA|CCT**GGG**ACTT	69058	WT	91.94%
TCAAATT----------CCTGGGACTT	1009	-10	1.34%
TCAAATTCCTGCAC-------GGACTT	941	-7	1.25%
TCAAATTCCTGCACGGAaCCTGGGACTT	855	+1 a	1.14%
TCAAATTCCTGCACGGA-CTGGGACTT	523	-1	0.70%
TCAAATTCCTGCACGGAtCCTGGGACTT	205	+1 t	0.27%
TCAAATTCCTGCACGGA---GGGACTT	186	-3	0.25%
TCAAATTCCTGCACGG-CCTGGGACTT	159	-1	0.21%
TCAAATTCCTGCACGGA--TGGGACTT	154	-2	0.21%
TCAAATTCCTGCAC------GGGACTT	157	-6	0.21%

P201-YW-W3-Syn52crGATA4b-RNPko-48h-BC2 48h<br>128345 | 13.37%	Ins<br>3.00%	Del<br>10.37%	HDR<br>0.00%
**Typical seqs**	**Reads**	**Type**	**pct.**
TCAAATTCCTGCACGGA|CCT**GGG**ACTT	111184	WT	86.63%
TCAAATTCCTGCAC-------GGACTT	4198	-7	3.27%
TCAAATT----------CCTGGGACTT	3464	-10	2.70%
TCAAATTCCTGCACGGAaCCTGGGACTT	1903	+1 a	1.48%
TCAAATTCCTGCACGGA-CTGGGACTT	1256	-1	0.98%
TCAAATTCCTGCACGGA---GGGACTT	481	-3	0.37%
TCAAATTCCTGCACGGAtCCTGGGACTT	408	+1 t	0.32%
TCAAATTCCTGCACGGA----------	365	-10	0.28%
TCAAATTCCTGCACGGAcCCTGGGACTT	291	+1 c	0.23%
TCAAATTCCTGCACGG-CCTGGGACTT	264	-1	0.21%

P197-P201-YW-W3-Syn54crGATA4d-RNPko

P197-YW-W3-Syn54crGATA4d-RNPko-4h-BC4 **4h**

47556 | 4.40%

	Ins	Del	HDR
	**4.04%**	**0.36%**	**0.01%**
**Typical seqs**	**Reads**	**Type**	**pct.**
TCCCTCCTCAAATTCCTGCA**CGG**ACCT	45464	WT	95.60%
TCCCTCCTCAAATTCCTtGCACGGACCT	1671	+1 t	3.51%
TCCCTCCTCAAATTCCTCTGCACGGACCT	173	+2	0.36%
TCCCTCCTCAAATT----------CCT	47	-10	0.10%
TCCCTCCTCAAA----TGCACGGACCT	18	-4	0.04%
TCCCTCCTCAAATTCCTATGCACGGACCT	17	+2	0.04%
TCCCTCCTCAAATT-CTGCACGGACCT	14	-1	0.03%
TCCCTCCTCAA-----------GACCT	12	-11	0.03%
TCCCTCCTCAAATT--TGCACGGACCT	11	-2	0.02%
TCCCTCCTCAAATTCCTtGCACGGACGT	10	+1 t	0.02%

P198-YW-W3-Syn54crGATA4d-RNPko-8h-BC4 **8h**

66836 | 10.32%

	Ins	Del	HDR
	**9.30%**	**0.93%**	**0.09%**
**Typical seqs**	**Reads**	**Type**	**pct.**
TCCCTCCTCAAATTCCTGCA**CGG**ACCT	59936	WT	89.68%
TCCCTCCTCAAATTCCTtGCACGGACCT	5517	+1 t	8.25%
TCCCTCCTCAAATTCCTCTGCACGGACCT	318	+2	0.48%
TCCCTCCTCAAATT----------CCT	234	-10	0.35%
TCCCTCCTCAAATTCCTG---GGACCT	86	-3	0.13%
TCCCT-------------CACG-- CGTAGTTTA	61	-9 HDR	0.09%
TCCCTCCTCAAATTCCT-CACGGACCT	50	-1	0.07%
TCCCTCCTCAAATTCCCTGCACGGACCT	35	+1 Ins	0.05%
TCCCTCCTCAAATTCC-GCACGGACCT	33	-1	0.05%
TCCCTCCT-------CTGCACGGACCT	31	-7	0.05%

P199-YW-W3-Syn54crGATA4d-RNPko-12h-BC4 **12h**

62951 | 13.21%

	Ins	Del	HDR
	**11.75%**	**1.42%**	**0.04%**
**Typical seqs**	**Reads**	**Type**	**pct.**
TCCCTCCTCAAATTCCTGCA**CGG**ACCT	54637	WT	86.79%
TCCCTCCTCAAATTCCTtGCACGGACCT	6510	+1 t	10.34%
TCCCTCCTCAAATTCCTCTGCACGGACCT	377	+2	0.60%
TCCCTCCTCAAATT----------CCT	251	-10	0.40%
TCCCTCCTCAAATTCC-GCACGGACCT	78	-1	0.12%
TCCCTCCTCAAATTCCT--ACGGACCT	74	-2	0.12%
TCCC-----------------------	65	-24	0.10%
TCCCTCCTCAAATT-CTGCACGGACCT	56	-1	0.09%
TCCCTCCTCAAATTC---CACGGACCT	56	-3	0.09%
TCCCTCCTCAAATTCCT---AGGACCT	54	-3	0.09%

P200-YW-W3-Syn54crGATA4d-RNPko-24h-BC4 **24h**

128974 | 22.61%

	Ins	Del	HDR
	**18.52%**	**3.95%**	**0.14%**
**Typical seqs**	**Reads**	**Type**	**pct.**
TCCCTCCTCAAATTCCTGCA**CGG**ACCT	99814	WT	77.39%
TCCCTCCTCAAATTCCTtGCACGGACCT	19774	+1 t	15.33%
TCCCTCCTCAAATTCCTCTGCACGGACCT	1838	+2	1.43%
TCCCTCCTCAAATT----------CCT	1340	-10	1.04%
TCCCTCCTCAAATTC---CACGGACCT	227	-3	0.18%
TCCCTCCTCAAATTCCCTGCACGGACCT	208	+1 Ins	0.16%
TCCCTCCTCAAATTCC--CACGGACCT	193	-2	0.15%
TCCCT-------------CACG-- CGTAGTTTA	184	-9 HDR	0.14%
TCCCTCCTCAAATTCCTG---GGACCT	178	-3	0.14%
TCCCTCCTCAAATT--TGCACGGACCT	170	-2	0.13%

P201-YW-W3-Syn54crGATA4d-RNPko-48h-BC4 **48h**

167620 | 31.98%

	Ins	Del	HDR
	**22.43%**	**9.13%**	**0.41%**
**Typical seqs**	**Reads**	**Type**	**pct.**
TCCCTCCTCAAATTCCTGCA**CGG**ACCT	114017	WT	68.02%
TCCCTCCTCAAATTCCTtGCACGGACCT	31513	+1 t	18.80%
TCCCTCCTCAAATT----------CCT	2359	-10	1.41%
TCCCTCCTCAAATTCCTCTGCACGGACCT	1893	+2	1.13%
TCCCTCCTCAAATTCCTG---GGACCT	783	-3	0.47%
TCCCT-------------CACG-- CGTAGTTTA	694	-9 HDR	0.41%
TCCCTCCTCAAAT---TGCACGGACCT	623	-3	0.37%
TCCCTCCTCAAATTCC--CACGGACCT	461	-2	0.28%
TCCCTCCTCAAATT-CTGCACGGACCT	431	-1	0.26%
TCCCTCCT----------CACGGACCT	431	-10	0.26%

P197-P201-YW-W3-Syn54crGATA4d-RNPaav

P197-YW-W3-Syn54crGATA4d-RNPaav-4h-BC12 **4h**	Ins	Del	HDR
72627 | 4.61%	**4.41%**	**0.19%**	**0.01%**
**Typical seqs**	**Reads**	**Type**	**pct.**
TCCCTCCTCAAATTCCT|GCA**CGG**ACCT	69276	WT	95.39%
TCCCTCCTCAAATTCCTtGCACGGACCT	2797	+1 t	3.85%
TCCCTCCTCAAATTCCTCTGCACGGACCT	217	+2	0.30%
TCCCTCCTCAAATTCCTTTGCACGGACCT	38	+2	0.05%
TCCCTCCTCAAATT----------CCT	25	-10	0.03%
TCCCTCCTCAAATTCCTG---GGACCT	24	-3	0.03%
TCCCTCCTCAAATTCCT-CACGGACCT	21	-1	0.03%
TCCCTCCTCAAATT-CTGCACGGACCT	19	-1	0.03%
TCCCTCCTCAAATTCCCTGCACGGACCT	19	+1 Ins	0.03%
TCCCTCCTCAAATTCCTGTGCACGGACCT	14	+2	0.02%

P198-YW-W3-Syn54crGATA4d-RNPaav-8h-BC12 **8h**	Ins	Del	HDR
64310 | 11.04%	**10.18%**	**0.65%**	**0.21%**
**Typical seqs**	**Reads**	**Type**	**pct.**
TCCCTCCTCAAATTCCT|GCA**CGG**ACCT	57213	WT	88.96%
TCCCTCCTCAAATTCCTtGCACGGACCT	5642	+1 t	8.77%
TCCCTCCTCAAATTCCTCTGCACGGACCT	529	+2	0.82%
TCCCT-------------CACG--CGTAGTTTA	136	-9 HDR	0.21%
TCCCTCCTCAAATTCCCTGCACGGACCT	59	+1 Ins	0.09%
TCCCTCCTCAAAT---TGCACGGACCT	44	-3	0.07%
TCCCTCCTCAAATT----------CCT	51	-10	0.08%
TCCCTCCTCAAATT-CTGCACGGACCT	39	-1	0.06%
TCCCTCCTCAAATTCC--CACGGACCT	31	-2	0.05%
TCCCTCCTCAAATTCCTtTCACGGACCT	30	+1 t	0.05%

P199-YW-W3-Syn54crGATA4d-RNPaav-12h-BC12 **12h**	Ins	Del	HDR
80672 | 15.61%	**13.65%**	**1.32%**	**0.64%**
**Typical seqs**	**Reads**	**Type**	**pct.**
TCCCTCCTCAAATTCCT|GCA**CGG**ACCT	68082	WT	84.39%
TCCCTCCTCAAATTCCTtGCACGGACCT	9306	+1 t	11.54%
TCCCTCCTCAAATTCCTCTGCACGGACCT	1157	+2	1.43%
TCCCT-------------CACG--CGTAGTTTA	511	-9 HDR	0.63%
TCCCTCCTCAAATT----------CCT	288	-10	0.36%
TCCCTCCTCAAATT-CTGCACGGACCT	156	-1	0.19%
TCCCTCCTCAAATTCC-GCACGGACCT	100	-1	0.12%
TCCCTCCTCAAATTCCT----GGACCT	63	-4	0.08%
TCCCTCCTCAAATTCCT-----GACCT	49	-5	0.06%
TCCCTCCTCAAA-TCCTGCACGGACCT	44	-1	0.05%

P200-YW-W3-Syn54crGATA4d-RNPaav-24h-BC12 **24h**	Ins	Del	HDR
74363 | 24.87%	**20.32%**	**2.27%**	**2.28%**
**Typical seqs**	**Reads**	**Type**	**pct.**
TCCCTCCTCAAATTCCT|GCA**CGG**ACCT	55870	WT	75.13%
TCCCTCCTCAAATTCCTtGCACGGACCT	13157	+1 t	17.69%
TCCCT-------------CACG--CGTAGTTTA	1665	-9 HDR	2.24%
TCCCTCCTCAAATTCCTCTGCACGGACCT	657	+2	0.88%
TCCCTCCTCAAATT-CTGCACGGACCT	194	-1	0.26%
TCCCTCCTCAAATT----------CCT	178	-10	0.24%
TCCCTCCTCAAATTCC-GCACGGACCT	147	-1	0.20%
TCCCTCCTCAAATTCCCTGCACGGACCT	112	+1 Ins	0.15%
TCCCTCCTCAAATTCCTTTGCACGGACCT	98	+2	0.13%
TCCCTCCTCAAATTCCTGTGCACGGACCT	65	+2	0.09%

P201-YW-W3-Syn54crGATA4d-RNPaav-48h-BC12 **48h**	Ins	Del	HDR
99271 | 36.82%	**28.30%**	**3.92%**	**4.59%**
**Typical seqs**	**Reads**	**Type**	**pct.**
TCCCTCCTCAAATTCCT|GCA**CGG**ACCT	62721	WT	63.18%
TCCCTCCTCAAATTCCTtGCACGGACCT	24571	+1 t	24.75%
TCCCT-------------CACG--CGTAGTTTA	4474	-9 HDR	4.51%
TCCCTCCTCAAATTCCTCTGCACGGACCT	1416	+2	1.43%
TCCCTCCTCAAATT----------CCT	470	-10	0.47%
TCCCTCCTCAAATT-CTGCACGGACCT	187	-1	0.19%
TCCCTCCTCAAAT---TGCACGGACCT	187	-3	0.19%
TCCCTCCTCAAATTCCTATGCACGGACCT	124	+2	0.12%
TCCCTCCTCAAATTC---CACGGACCT	124	-3	0.12%
TCCCTCCTCAAATTCCCTGCACGGACCT	111	+1 Ins	0.11%

P197-P201-YW-W3-Syn55crGATA4e-RNPko

P197-YW-W3-Syn55crGATA4e-RNPko-4h-BC5 **4h**	Ins	Del	HDR
41923 | 0.18%	**0.13%**	**0.04%**	**0.01%**
**Typical seqs**	**Reads**	**Type**	**pct.**
TCCAAGTCCCAGGTCCG|TGC**AGG**AATT	41849	WT	99.82%
TCCAAGTCCCAGGTCCGtTGCAGGAATT	31	+1 t	0.07%
TCCAAGTCCCAGGTCC-TGCAGGAATT	7	-1	0.02%
TCCAAGTCCCAGGTCCGTATGCAGGAATT	7	+2	0.02%
TCCAAGTCCCAGGTCC--GCAGGAATT	5	-2	0.01%
TCCAAGTCCCAGGTCCGgTGCAGGAATT	4	+1 g	0.01%
TCCAAGTCCCAGGTCCGTTTGCAGGAATT	4	+2	0.01%
TCCAAGTCCCAGGTCCGaTGCAGGAATT	4	+1 a	0.01%
TCCAAGTCCCAGGTCC---CAGGAATT	3	-3	0.01%
TCCAAGTCC----------CAGGAATT	3	-10	0.01%

P198-YW-W3-Syn55crGATA4e-RNPko-8h-BC5 **8h**	Ins	Del	HDR
52196 | 0.89%	**0.38%**	**0.42%**	**0.10%**
**Typical seqs**	**Reads**	**Type**	**pct.**
TCCAAGTCCCAGGTCCG|TGC**AGG**AATT	51732	WT	99.11%
TCCAAGTCCCAGGTCCGtTGCAGGAATT	84	+1 t	0.16%
TCCAAGTCCCAGGTCCG-GCAGGAATT	70	-1	0.13%
TCCAAGT-----GTTTAAACTACGCGT----------	50	-9 HDR	0.10%
TCCAAGTCCCAGGTCCGCGTGCAGGAATT	42	+2	0.08%
TCCAAGTCCCAGGTCCGTTTGCAGGAATT	35	+2	0.07%
TCCAAGTCCCAGGTC--TGCAGGAATT	30	-2	0.06%
TCCAAGTCCCAGGTCC--GCAGGAATT	29	-2	0.06%
TCCAAGTCCCAGGTCC-TGCAGGAATT	22	-1	0.04%
TCCAAGTCCCAGGTCCGaTGCAGGAATT	18	+1 a	0.03%

P199-YW-W3-Syn55crGATA4e-RNPko-12h-BC5 **12h**	Ins	Del	HDR
92471 | 1.68%	**0.89%**	**0.77%**	**0.02%**
**Typical seqs**	**Reads**	**Type**	**pct.**
TCCAAGTCCCAGGTCCG|TGC**AGG**AATT	90919	WT	98.32%
TCCAAGTCCCAGGTCCGtTGCAGGAATT	365	+1 t	0.39%
TCCAAGTCC----------CAGGAATT	180	-10	0.19%
TCCAAGTCCCAGGTCC--GCAGGAATT	168	-2	0.18%
TCCAAGTCCCAGGTCCGaTGCAGGAATT	127	+1 a	0.14%
TCCAAGTCCCAGGTCCG-GCAGGAATT	135	-1	0.15%
TCCAAGTCCCAGGTCCGTCTGCAGGAATT	71	+2	0.08%
TCCAAGTCCCAGGTCCGCGTGCAGGAATT	63	+2	0.07%
TCCAAGTCCCAGGTCCTCAATTGCAGGAATT	59	+4	0.06%
TCCAAGTCCCAGGT---TGCAGGAATT	51	-3	0.06%

P200-YW-W3-Syn55crGATA4e-RNPko-24h-BC5 **24h**	Ins	Del	HDR
100807 | 2.36%	**1.02%**	**1.13%**	**0.21%**
**Typical seqs**	**Reads**	**Type**	**pct.**
TCCAAGTCCCAGGTCCG|TGC**AGG**AATT	98424	WT	97.64%
TCCAAGTCCCAGGTCCGtTGCAGGAATT	554	+1 t	0.55%
TCCAAGT-----GTTTAAACTACGCGT----------	212	-9 HDR	0.21%
TCCAAGTCC----------CAGGAATT	183	-10	0.18%
TCCAAGTCCCAGGTCC-TGCAGGAATT	177	-1	0.18%
TCCAAGTCCCAGGTCCGCGTGCAGGAATT	130	+2	0.13%
TCCAAGTCCCAGGTCCG-GCAGGAATT	119	-1	0.12%
TCCAAGTCCCAGGTC----------TT	86	-10	0.09%
TCCAAGTCCCAGGTC----CAGGAATT	73	-4	0.07%
TCCAAGTCCCAGGTCCGaTGCAGGAATT	65	+1 a	0.06%

P201-YW-W3-Syn55crGATA4e-RNPko-48h-BC5 **48h**	Ins	Del	HDR
138782 | 6.60%	**2.72%**	**3.88%**	**0.00%**
**Typical seqs**	**Reads**	**Type**	**pct.**
TCCAAGTCCCAGGTCCG|TGC**AGG**AATT	129622	WT	93.40%
TCCAAGTCCCAGGTCCGtTGCAGGAATT	2064	+1 t	1.49%
TCCAAGTCC----------CAGGAATT	962	-10	0.69%
TCCAA-----------GTGCAGGAATT	716	-11	0.52%
TCCAAGT-----GTTTAAACTACGCGT----------	575	-9	0.41%
TCCAAGTC----------GCAGGAATT	564	-10	0.41%
TCCAAGTCCCAGGTCC-TGCAGGAATT	464	-1	0.33%
TCCAAGTCCCAGGTCCGAATGCAGGAATT	364	+2	0.26%
TCCAAGTCCCAGGTC----CAGGAATT	350	-4	0.25%
TCCAAGTCC--------TGCAGGAATT	313	-8	0.23%

P197-P201-YW-W3-Syn56crGATA4g-RNPko

P197-YW-W3-Syn56crGATA4g-RNPko-4h-BC6 **4h**	Ins	Del	HDR
43298 | 1.38%	**1.20%**	**0.18%**	**0.00%**
**Typical seqs**	**Reads**	**Type**	**pct.**
CAGGTCCGTGCAGGAAT|TTG**AGG**AGGG	42699	WT	98.62%
CAGGTCCGTGCAGGAATtTTGAGGAGGG	460	+1 t	1.06%
CAGGTCCGTGC---------AGGAGGG	22	-9	0.05%
CAGGTCCGTGCAGGAA-TTGAGGAGGG	21	-1	0.05%
CAGGTCCGTGCAGGAATATTTGAGGAGGG	18	+2	0.04%
CAGGTCCGTGCAGG-ATTTGAGGAGGG	17	-1	0.04%
CAGGTCCGTGCAGGAAATTTGAGGAGGG	15	+1 Ins	0.03%
CAGGTCCGTGCAGGAATaTTGAGGAGGG	7	+1 a	0.02%
CAGGTCCGTGCAGGAATTTTTGAGGAGGG	6	+2	0.01%
CAGGTCCG----------TGAGGAGGG	5	-10	0.01%

P198-YW-W3-Syn56crGATA4g-RNPko-8h-BC6 **8h**	Ins	Del	HDR
49397 | 4.02%	**3.34%**	**0.68%**	**0.00%**
**Typical seqs**	**Reads**	**Type**	**pct.**
CAGGTCCGTGCAGGAAT|TTG**AGG**AGGG	47412	WT	95.98%
CAGGTCCGTGCAGGAATtTTGAGGAGGG	1506	+1 t	3.05%
CAGGTCCGTGCAGGAA-TTGAGGAGGG	92	-1	0.19%
CAGGTCCGTGCAGGAATTTTTGAGGAGGG	34	+2	0.07%
CAGGTCCGTGCAGG-ATTTGAGGAGGG	34	-1	0.07%
CAGGTCCGTGC---------AGGAGGG	34	-9	0.07%
CAGGTCCGTGCAGG--TTTGAGGAGGG	33	-2	0.07%
CAGGTC----------TTTGAGGAGGG	28	-10	0.06%
CAGGTCCGTGCAGGA--------AGGG	25	-8	0.05%
CAGGTCCGTGCAGGAATgTTGAGGAGGG	21	+1 g	0.04%

P199-YW-W3-Syn56crGATA4g-RNPko-12h-BC6 **12h**	Ins	Del	HDR
108493 | 6.58%	**4.95%**	**1.63%**	**0.00%**
**Typical seqs**	**Reads**	**Type**	**pct.**
CAGGTCCGTGCAGGAAT|TTG**AGG**AGGG	101350	WT	93.42%
CAGGTCCGTGCAGGAATtTTGAGGAGGG	4847	+1 t	4.47%
CAGGTCCGTGCAGGAA-TTGAGGAGGG	606	-1	0.56%
CAGGTCCGTGCAGG-ATTTGAGGAGGG	232	-1	0.21%
CAGGTCCGTGCAGGAATATTTGAGGAGGG	165	+2	0.15%
CAGGTCCGTGCAGGAA--TGAGGAGGG	127	-2	0.12%
CAGGTCCGTGCAGGAATTTTTGAGGAGGG	95	+2	0.09%
CAGGTCCGTGC----------------	91	-17	0.08%
CAGGTCCGTGCAGG--TTTGAGGAGGG	81	-2	0.07%
CAGGTCCGTGC------------AGGG	79	-12	0.07%

P200-YW-W3-Syn56crGATA4g-RNPko-24h-BC6 **24h**	Ins	Del	HDR
136696 | 8.14%	**5.77%**	**2.37%**	**0.00%**
**Typical seqs**	**Reads**	**Type**	**pct.**
CAGGTCCGTGCAGGAAT|TTG**AGG**AGGG	125565	WT	91.86%
CAGGTCCGTGCAGGAATtTTGAGGAGGG	6790	+1 t	4.97%
CAGGTCCGTGC---------AGGAGGG	490	-9	0.36%
CAGGTCCG----------TGAGGAGGG	436	-10	0.32%
CAGGTCCGTGCAGGAA-TTGAGGAGGG	402	-1	0.29%
CAGGTCCGTGCAGGAATATTTGAGGAGGG	164	+2	0.12%
CAGGTCCGTGCAGG-ATTTGAGGAGGG	137	-1	0.10%
CAGGTCCGTGCAGGAATTTTTGAGGAGGG	119	+2	0.09%
CAGGTCCGTGC------------AGGG	112	-12	0.08%
CAGGTCCGTGCAG------GAGGAGGG	106	-6	0.08%

P201-YW-W3-Syn56crGATA4g-RNPko-48h-BC6 **48h**	Ins	Del	HDR
168168 | 12.41%	**7.76%**	**4.66%**	**0.00%**
**Typical seqs**	**Reads**	**Type**	**pct.**
CAGGTCCGTGCAGGAAT|TTG**AGG**AGGG	147294	WT	87.59%
CAGGTCCGTGCAGGAATtTTGAGGAGGG	10924	+1 t	6.50%
CAGGTCCGTGC---------AGGAGGG	2154	-9	1.28%
CAGGTCCG----------TGAGGAGGG	665	-10	0.40%
CAGGTCCGTGCA-------------GG	638	-13	0.38%
CAGGTCCGTGCAGGAATATTTGAGGAGGG	557	+2	0.33%
CAGGTCCGT---------------GGG	419	-15	0.25%
CAGGTCCGTGC------------AGGG	398	-12	0.24%
CA-----------------GAGGAGGG	336	-17	0.20%
CAGGTCCGTGCAGGAA--TGAGGAGGG	312	-2	0.19%

P197-P201-YW-W3-Syn57crGATA48h-RNPko

P197-YW-W3-Syn57crGATA4h-RNPko-4h-BC7 4h<br>55555 | 0.55%	Ins<br>0.07%	Del<br>0.48%	HDR<br>0.00%
**Typical seqs**	**Reads**	**Type**	**pct.**
GTCCGTGCAGGAATTTG|AGG**AGG**GAAG	55252	WT	99.45%
GTCCGTGCAGGAATTTG-GGAGGGAAG	103	-1	0.19%
GTCCGTGC---------AGGAGGGAAG	82	-9	0.15%
GTCCGTGCAGGAATTT--GGAGGGAAG	21	-2	0.04%
GTCCGTGCAGGAATT--AGGAGGGAAG	14	-2	0.03%
GTCCGTGCAGGAATTTGgAGGAGGGAAG	13	+1 g	0.02%
GTCCGTGCAGGAAT---AGGAGGGAAG	11	-3	0.02%
GTCCGTGCAGGAATTT-AGGAGGGAAG	10	-1	0.02%
GTCCGTGCAGGAATTT---GAGGGAAG	9	-3	0.02%
GTCCG----------TGAGGAGGGAAG	8	-10	0.01%

P198-YW-W3-Syn57crGATA8h-RNPko-8h-BC7 8h<br>67294 | 2.36%	Ins<br>0.13%	Del<br>2.23%	HDR<br>0.00%
**Typical seqs**	**Reads**	**Type**	**pct.**
GTCCGTGCAGGAATTTG|AGG**AGG**GAAG	65706	WT	97.64%
GTCCGTGC---------AGGAGGGAAG	596	-9	0.89%
GTCCGTGCAGGAATTTG-GGAGGGAAG	390	-1	0.58%
GTCCGTGCAGGAATTT--GGAGGGAAG	125	-2	0.19%
GTCCGTGCAGGAATTT-AGGAGGGAAG	95	-1	0.14%
GTCCGTGCAGGAATTTGgAGGAGGGAAG	65	+1 g	0.10%
GTCCGTGCAGGA-----AGGAGGGAAG	52	-5	0.08%
GTCCGTGC------------AGGGAAG	38	-12	0.06%
G--------------TTAGGAGGGAAG	30	-14	0.04%
GTCCGTGCAGGAA---AAGGAGGGAAG	19	-3	0.03%

P199-YW-W3-Syn57crGATA12h-RNPko-12h-BC7 12h<br>112618 | 3.32%	Ins<br>0.31%	Del<br>3.01%	HDR<br>0.00%
**Typical seqs**	**Reads**	**Type**	**pct.**
GTCCGTGCAGGAATTTG|AGG**AGG**GAAG	108876	WT	96.68%
GTCCGTGC---------AGGAGGGAAG	1299	-9	1.15%
GTCCGTGCAGGAATTTG-GGAGGGAAG	727	-1	0.65%
GTCCGTGCAGGAAT---AGGAGGGAAG	192	-3	0.17%
GTCC-------------AGGAGGGAAG	138	-13	0.12%
GTCCGTGCAGGAA----AGGAGGGAAG	129	-4	0.11%
GTCCGTGCAGGAATTTGtAGGAGGGAAG	83	+1 t	0.07%
GTCCGTGCAGGAATTT--GGAGGGAAG	78	-2	0.07%
GTC--------------AGGAGGGAAG	77	-14	0.07%
GT---------------AGGAGGGAAG	75	-15	0.07%

P200-YW-W3-Syn57crGATA24h-RNPko-24h-BC7 24h<br>163307 | 6.42%	Ins<br>0.55%	Del<br>5.87%	HDR<br>0.00%
**Typical seqs**	**Reads**	**Type**	**pct.**
GTCCGTGCAGGAATTTG|AGG**AGG**GAAG	152822	WT	93.58%
GTCCGTGC---------AGGAGGGAAG	3321	-9	2.03%
GTCCGTGCAGGAATTTG-GGAGGGAAG	2219	-1	1.36%
GTCCGTGCAGGAATTT--GGAGGGAAG	813	-2	0.50%
GTCCGTGCAGGAATTT------GGAAG	340	-6	0.21%
GTCCGTGCAGGAATTTGgAGGAGGGAAG	332	+1 g	0.20%
GTCCGT------------GGAGGGAAG	323	-12	0.20%
GTCCGTGCAGGA-----AGGAGGGAAG	264	-5	0.16%
GTCCGTGCAGGAAT---AGGAGGGAAG	231	-3	0.14%
GTCCGTGCAGGAATTT----------G	220	-10	0.13%

P201-YW-W3-Syn57crGATA48h-RNPko-48h-BC7 48h<br>202013 | 15.85%	Ins<br>1.04%	Del<br>14.81%	HDR<br>0.00%
**Typical seqs**	**Reads**	**Type**	**pct.**
GTCCGTGCAGGAATTTG|AGG**AGG**GAAG	169990	WT	84.15%
GTCCGTGC---------AGGAGGGAAG	18597	-9	9.21%
GTCCGTGCAGGAATTTG-GGAGGGAAG	1850	-1	0.92%
GTCCGTGCAGGAATTT-AGGAGGGAAG	1327	-1	0.66%
GTCCGTGCAGGAATTT----------G	1034	-10	0.51%
GTCCGTGCAGGAATTT--GGAGGGAAG	759	-2	0.38%
GTCCGTGC------------AGGGAAG	511	-12	0.25%
GTCCGTGCAGGAATTTGaAGGAGGGAAG	426	+1 a	0.21%
GTCCGTGCA-------------GGAAG	395	-13	0.20%
GTCCGTGCAGGAATTTGcAGGAGGGAAG	396	+1 c	0.20%

P197-P201-YW-W3-Syn58crGATA4i-RNPko

P197-YW-W3-Syn58crGATA4i-RNPko-4h-BC8 4h	Ins	Del	HDR
32546 | 0.96%	0.75%	0.21%	0.00%
**Typical seqs**	**Reads**	**Type**	**pct.**
TCCGTGCAGGAATTTGA|GGA**GGG**AAGA	32233	WT	99.04%
TCCGTGCAGGAATTTGAaGGAGGGAAGA	236	+1 a	0.73%
TCCGTGCAGGAATTTG-GGAGGGAAGA	16	-1	0.05%
TCCGTGC---------AGGAGGGAAGA	16	-9	0.05%
TCCGTGCAGGAATTTGA-GAGGGAAGA	14	-1	0.04%
TCCGTGC------------AGGGAAGA	7	-12	0.02%
TCCGTGCAGGAATTT---GAGGGAAGA	7	-3	0.02%
TCCGTGCAGGAATTT--GGAGGGAAGA	5	-2	0.02%
TCCGTGCAGGAATTTGACGCGTAGGAGGGA AGA	4	+6	0.01%
TCCGTGCAGGAATTTGGAGGAGGGAAGA	4	+1 Ins	0.01%

P198-YW-W3-Syn58crGATA4i-RNPko-8h-BC8 8h	Ins	Del	HDR
44293 | 3.22%	2.24%	0.98%	0.00%
**Typical seqs**	**Reads**	**Type**	**pct.**
TCCGTGCAGGAATTTGA|GGA**GGG**AAGA	42865	WT	96.78%
TCCGTGCAGGAATTTGAaGGAGGGAAGA	952	+1 a	2.15%
TCCGTGC---------AGGAGGGAAGA	104	-9	0.23%
TCCGTGCAGGAATTTGA-GAGGGAAGA	92	-1	0.21%
TCCGTGCAGGAATTTG-GGAGGGAAGA	58	-1	0.13%
TCCGTG-----------GGAGGGAAGA	40	-11	0.09%
TCCGTGCAGGAAT----CGAGGGAAGA	27	-4	0.06%
TCCGTGCAGGAATTTGCAGGAGGGAAGA	17	+1 Ins	0.04%
TCCGTGCAGGAATTTGA-----GAAGA	16	-5	0.04%
TCCGTGCAG------GAGGAGGGAAGA	15	-6	0.03%

P199-YW-W3-Syn58crGATA4i-RNPko-12h-BC8 12h	Ins	Del	HDR
7260 | 3.07%	2.27%	0.80%	0.00%
**Typical seqs**	**Reads**	**Type**	**pct.**
TCCGTGCAGGAATTTGA|GGA**GGG**AAGA	7037	WT	96.93%
TCCGTGCAGGAATTTGAaGGAGGGAAGA	158	+1 a	2.18%
TCCGTGC---------AGGAGGGAAGA	13	-9	0.18%
TCCGTGCA-------------GGAAGA	13	-13	0.18%
TCCGTGCAGGAATTTG-GGAGGGAAGA	9	-1	0.12%
TCCGTGCAGGAATT--AGGAGGGAAGA	6	-2	0.08%
TCCGTGCAGGAATTTGA-GAGGGAAGA	6	-1	0.08%
TCCGTGCAGGAATTTTGAGGAGGGAAGA	4	+1 Ins	0.06%
TCCGTGCAGGAATTTG---------GA	4	-9	0.06%
TCCGTGCAGGAATTT-----GGGAAGA	4	-5	0.06%

P200-YW-W3-Syn58crGATA4i-RNPko-24h-BC8 24h	Ins	Del	HDR
65167 | 5.95%	3.62%	2.33%	0.00%
**Typical seqs**	**Reads**	**Type**	**pct.**
TCCGTGCAGGAATTTGA|GGA**GGG**AAGA	61291	WT	94.05%
TCCGTGCAGGAATTTGAaGGAGGGAAGA	2133	+1 a	3.27%
TCCGTGC---------AGGAGGGAAGA	512	-9	0.79%
TCCGTGCAGGAATTT---GAGGGAAGA	182	-3	0.28%
TCCGTGCAGGAATTTGA-GAGGGAAGA	154	-1	0.24%
TCCGTGCAGGAATTTG-GGAGGGAAGA	132	-1	0.20%
TCCGTGCAGGAATTT--GGAGGGAAGA	54	-2	0.08%
TCCGTGCAGGAATTT----------GA	47	-10	0.07%
TCCGTGCAGGAATTTGA----GGAAGA	45	-4	0.07%
TCCGTGCAGGAATTT------------	39	-12	0.06%

P201-YW-W3-Syn58crGATA4i-RNPko-48h-BC8 48h	Ins	Del	HDR
109924 | 10.59%	5.13%	5.46%	0.00%
**Typical seqs**	**Reads**	**Type**	**pct.**
TCCGTGCAGGAATTTGA|GGA**GGG**AAGA	98283	WT	89.41%
TCCGTGCAGGAATTTGAaGGAGGGAAGA	4719	+1 a	4.29%
TCCGTGC---------AGGAGGGAAGA	2746	-9	2.50%
TCCGTGCAGGAATTTG-GGAGGGAAGA	519	-1	0.47%
TCCGTGC------------AGGGAAGA	395	-12	0.36%
TCCGTGCAGGAATTT---GAGGGAAGA	354	-3	0.32%
TCCGTGCAGGAATTTGA-GAGGGAAGA	353	-1	0.32%
TCCGTGCAGGAATTT--GGAGGGAAGA	236	-2	0.21%
TCCGTGCAGGAATTTGA----GGAAGA	129	-4	0.12%
TCCGTGCAGGAATTTGGAGGAGGGAAGA	125	+1 Ins	0.11%

P197-P201-YW-W9-Syn32crBCL11Gata-a1

P197-YW-W9-Syn32crBCL11Gata-a1-4h-KO-BC1 **4h**	Ins	Del	HDR
12688 | 20.00%	**17.95%**	**2.05%**	**0.00%**
**Typical seqs**	**Reads**	**Type**	**pct.**
CACGCCCCCACCCTAAT|CAG**AGG**CCAA	10151	WT	80.00%
CACGCCCCCACCCTAATtCAGAGGCCAA	1352	+1 t	10.66%
CACGCCCCCCCCCTAATTCAGAGGCCAA	204	+1 Ins	1.61%
CCCGCCCCCACCCTAATTCAGAGGCCAA	93	+1 Ins	0.73%
CACGCCCCCACCCTAATtCAGAGGCCCA	97	+1 t	0.76%
CACGCCCCCACCCCAATTCAGAGGCCAA	76	+1 Ins	0.60%
CACGCCCCCACCCTAATtTAGAGGCCAA	64	+1 t	0.50%
CACGCCC----------------CCAA	47	-16	0.37%
CACGCCCCCACGCCT--CCAGAGGCCAA	46	-1	0.36%
CCCGCCCCCCCCCTAATTCAGAGGCCAA	32	+1 Ins	0.25%

P198-YW-W9-Syn32crBCL11Gata-a1-8h-KO-BC1 **8h**	Ins	Del	HDR
16303 | 56.30%	**18.19%**	**38.10%**	**0.00%**
**Typical seqs**	**Reads**	**Type**	**pct.**
CACGCCCCCACCCTAAT|CAG**AGG**CCAA	7125	WT	43.70%
CACG--------------AGAGGCCAA	1512	-14	9.27%
CACGCCCCCACCCTAATtCAGAGGCCAA	1664	+1 t	10.21%
CACG----------------------A	841	-22	5.16%
CACGTC-----------CAGAGGCCAA	777	-11	4.77%
CA-----------------------AA	686	-23	4.21%
CACGCCCC--------------------	636	-24	3.90%
CACGCCC----------CAGAGGCCAA	575	-10	3.53%
CACGCCCCCGC-----TCAGAGGCCAA	380	-5	2.33%
CACGCCCCCCCCCTAATTCAGAGGCCAA	242	+1 Ins	1.48%

P199-YW-W9-Syn32crBCL11Gata-a1-12h-KO-BC1 **12h**	Ins	Del	HDR
29081 | 62.39%	**21.09%**	**41.30%**	**0.00%**
**Typical seqs**	**Reads**	**Type**	**pct.**
CACGCCCCCACCCTAAT|CAG**AGG**CCAA	10938	WT	37.61%
CACGCCCCCACCCTAATtCAGAGGCCAA	3491	+1 t	12.00%
CACGCCCC---------CAGAGGCCAA	1885	-9	6.48%
CACG-------------CAGAGGCCAA	1123	-13	3.86%
-----------------CAGAGGCCAA	1018	-17	3.50%
CACGCTC----------CAGAGGCCAA	878	-10	3.02%
CACGC----------------------	877	-27	3.02%
CACG--------------------AA	657	-21	2.26%
CACGCCCTC--------CAGAGGCCAA	577	-8	1.98%
CACGCC-----------CAGAGGCCAA	569	-11	1.96%

P200-YW-W9-Syn32crBCL11Gata-a1-24h-KO-BC1 **24h**	Ins	Del	HDR
20440 | 72.87%	**16.28%**	**56.59%**	**0.00%**
**Typical seqs**	**Reads**	**Type**	**pct.**
CACGCCCCCACCCTAAT|CAG**AGG**CCAA	5545	WT	27.13%
CAC----------------GAGGCCAA	2147	-16	10.50%
CACGCCCCCACCCTAATtCAGAGGCCAA	1830	+1 t	8.95%
CACG-----------------------	768	-29	3.76%
CACG--------------------CAA	420	-20	2.05%
CACGC------------CAGAGGCCAA	331	-12	1.62%
CACGCCC----------CAGAGGCCAA	330	-10	1.61%
CACGCC---------------------	868	-26	4.25%
CACG-----------------------	768	-24	3.76%
CACGCC-----------CAGAGGCCAA	295	-11	1.44%

P201-YW-W9-Syn32crBCL11Gata-a1-48h-KO-BC1 **48h**	Ins	Del	HDR
103485 | 75.61%	**11.62%**	**63.99%**	**0.00%**
**Typical seqs**	**Reads**	**Type**	**pct.**
CACGCCCCCACCCTAAT|CAG**AGG**CCAA	25245	WT	24.39%
CACGCCCCCACCCTAATtCAGAGGCCAA	5955	+1 t	5.75%
CAC----------------GAGGCCAA	4293	-16	4.15%
CACG-------------CAGAGGCCAA	4221	-13	4.08%
CACGCC---------------------	4359	-22	4.21%
CACGCCCC---------CAGAGGCCAA	3028	-9	2.93%
CAC------------------GCCAA	2778	-19	2.68%
CACGCC-----------CAGAGGCCAA	2286	-11	2.21%
CACGC------------CAGAGGCCAA	2215	-12	2.14%
CACG-----------------------	4588	-29	4.43%

P197-P201-YW-W9-Syn33crBCL11Gata-a2

P197-YW-W9-Syn33crBCL11Gata-a2-4h-KO-BC2 **4h**	Ins	Del	HDR
12799 | 9.93%	**5.13%**	**4.81%**	**0.00%**
**Typical seqs**	**Reads**	**Type**	**pct.**
CACAGGCTCCAGGAAGG|GTT**TGG**CCTC	11528	WT	90.07%
CACAGGCTCCAGGAAGGgGTTTGGCCTC	503	+1 g	3.93%
CACAGGCTCCAGGAA-GGTTTGGCCTC	292	-1	2.28%
CACAGGCTCCAGGAA------GGCCTC	70	-6	0.55%
CACAGGCTCCAGGAAGGgGTTGGGCCTC	60	+1 g	0.47%
CACAGGCTCC----AGGGTTTGGCCTC	23	-4	0.18%
CACAGGCTCCAG---GGGTTTGGCCTC	20	-3	0.16%
CACAGGCTCCAGGAAGGGGGTTTGGCCTC	29	+2	0.23%
CACAGGCTCCA-----GGTTTGGCCTC	19	-5	0.15%
CACAGGCTCCAGGAA--GTTTGGCCTC	21	-2	0.16%

P198-YW-W9-Syn33crBCL11Gata-a2-8h-KO-BC2 **8h**	Ins	Del	HDR
9546 | 17.85%	**10.85%**	**7.00%**	**0.00%**
**Typical seqs**	**Reads**	**Type**	**pct.**
CACAGGCTCCAGGAAGG|GTT**TGG**CCTC	7842	WT	82.15%
CACAGGCTCCAGGAAGGgGTTTGGCCTC	780	+1 g	8.17%
CACAGGCTCCAGGAA-GGTTTGGCCTC	298	-1	3.12%
CACAGGCTCCAGGAA------GGCCTC	65	-6	0.68%
CACAGGCTCCAGGAAGGgGTTGGGCCTC	78	+1 g	0.82%
CACAGGCTCCA-----GGTTTGGCCTC	35	-5	0.37%
CACAGGCTCCAGGA--GGTTTGGCCTC	34	-2	0.36%
CACAGGCTCCAGGA---GTTTGGCCTC	26	-3	0.27%
CACAGGCTCCAGGAAGG----------	35	-14	0.37%
CACAGGCTCCAGGAA--GTTTGGCCTC	28	-2	0.29%

P199-YW-W9-Syn33crBCL11Gata-a2-12h-KO-BC2 **12h**	Ins	Del	HDR
12089 | 25.54%	**10.33%**	**15.21%**	**0.00%**
**Typical seqs**	**Reads**	**Type**	**pct.**
CACAGGCTCCAGGAAGG|GTT**TGG**CCTC	9001	WT	74.46%
CACAGGCTCCAGGAAGGgGTTTGGCCTC	894	+1 g	7.40%
CACAGGCTCCAGGAA-GGTTTGGCCTC	645	-1	5.34%
CACAGGCTCCAGGAA------GGCCTC	254	-6	2.10%
CACAGGCTCC----AGGGTTTGGCCTC	102	-4	0.84%
CACAGGCTCCA----------GGCCTC	76	-10	0.63%
CACAGGCTCCAGGAA--GTTTGGCCTC	70	-2	0.58%
CACA-----------------GGCCTC	71	-17	0.59%
CACAGGCTCCAGGAAGGgGTTGGGCCTC	84	+1 g	0.69%
CACAGGCTCCAGGAAGG----AGCCTC	51	-4	0.42%

P200-YW-W9-Syn33crBCL11Gata-a2-24h-KO-BC2 **24h**	Ins	Del	HDR
7160 | 30.04%	**9.55%**	**20.49%**	**0.00%**
**Typical seqs**	**Reads**	**Type**	**pct.**
CACAGGCTCCAGGAAGG|GTT**TGG**CCTC	5009	WT	69.96%
CACAGGCTCCAGGAA-GGTTTGGCCTC	555	-1	7.75%
CACAGGCTCCAGGAAGGgGTTTGGCCTC	524	+1 g	7.32%
CACAGGCTCCAGGAA------GGCCTC	184	-6	2.57%
CACAGGCTCCA----------GGCCTC	76	-10	1.06%
CACAGGCTCCAGGAA-GGTTGGGCCTC	47	-1	0.66%
CACAGGCTCCAGGAA-------GCCTC	45	-7	0.63%
CACAGGCTCC----AGGGTTTGGCCTC	42	-4	0.59%
CACAGGCTCCAGGAAGGgGTTGGGCCTC	58	+1 g	0.81%
CACA-----------------GGCCTC	37	-17	0.52%

P201-YW-W9-Syn33crBCL11Gata-a2-48h-KO-BC2 **48h**	Ins	Del	HDR
6456 | 39.73%	**9.91%**	**29.82%**	**0.00%**
**Typical seqs**	**Reads**	**Type**	**pct.**
CACAGGCTCCAGGAAGG|GTT**TGG**CCTC	3891	WT	60.27%
CACAGGCTCCAGGAAGGAGTTGCTTTTATC ACAGGCCTC	277	+12	4.29%
CACAGGCTCCAGGAA-GGTTTGGCCTC	311	-1	4.82%
CACA-----------------GGCCTC	195	-17	3.02%
CACAGGCTCCAGGAAGGgGTTTGGCCTC	235	+1 g	3.64%
CACAGGCTCCAGGAA------------	139	-19	2.15%
CACAGGCTCCAGGAA------GGCCTC	172	-6	2.66%
CACAGGCTCCA------------CCTC	98	-12	1.52%
CACAGGCTCCA----------GGCCTC	101	-10	1.56%
CACAGGCTCCAG---------------	47	-16	0.73%

P197-P201-YW-W9-Syn34crBCL11Gata-a3

P197-YW-W9-Syn34crBCL11Gata-a3-4h-KO-BC3 4h	Ins	Del	HDR
12880 | 19.13%	13.49%	5.64%	0.00%
**Typical seqs**	**Reads**	**Type**	**pct.**
TTTATCACAGGCTCCAG|GAA**GGG**TTTG	10416	WT	80.87%
TTTATCACAGGCTCCAGgGAAGGGTTTG	1175	+1 g	9.12%
TTTATCACAGGCTCC-GGAAGGGTTTG	227	-1	1.76%
TTTATCACAGGCTCCA-GAAGGGTTTG	243	-1	1.89%
TTTATCACAGGCTCCAGAGGAAGGGTTTG	141	+2	1.09%
TTTATCACAGGCTCCAGgGAAGGGTTGG	139	+1 g	1.08%
TTTATCACAGGCTCCAGGGGAAGGGTTTG	59	+2	0.46%
TTTATCACAGGCTCCAGgGAAGGTTTTG	42	+1 g	0.33%
TTTATCACAGGCTCCAGCCAGGGAAGGGTTTG	31	+5	0.24%
TTTATCACAGGCTCC--GAAGGGTTTG	27	-2	0.21%

P198-YW-W9-Syn34crBCL11Gata-a3-8h-KO-BC3 8h	Ins	Del	HDR
18525 | 28.76%	18.06%	10.70%	0.00%
**Typical seqs**	**Reads**	**Type**	**pct.**
TTTATCACAGGCTCCAG|GAA**GGG**TTTG	13198	WT	71.24%
TTTATCACAGGCTCCAGgGAAGGGTTTG	2246	+1 g	12.12%
TTTATCACAGGCTCC-GGAAGGGTTTG	411	-1	2.22%
TTTATCACAGGCTCCA-GAAGGGTTTG	328	-1	1.77%
TTTATCACAGGCTCCAGgGAAGGGTTGG	268	+1 g	1.45%
TTTATCACAGGCTCCAGAGGAAGGGTTTG	168	+2	0.91%
TTTATCACAGGCT---------------	168	-15	0.91%
TTTATCA-------CAGGAAGGGTTTG	101	-7	0.55%
TTTATCACAGGCTCC----AGGGTTTG	110	-4	0.59%
TTTATCACAGGCTCCA-----------	111	-13	0.60%

P199-YW-W9-Syn34crBCL11Gata-a3-12h-KO-BC3 12h	Ins	Del	HDR
13375 | 41.93%	23.25%	18.68%	0.00%
**Typical seqs**	**Reads**	**Type**	**pct.**
TTTATCACAGGCTCCAG|GAA**GGG**TTTG	7767	WT	58.07%
TTTATCACAGGCTCCAGgGAAGGGTTTG	2019	+1 g	15.10%
TTTATCACAGGCTCCA-GAAGGGTTTG	581	-1	4.34%
TTTATCACAGGCTCC-GGAAGGGTTTG	333	-1	2.49%
TTTATCACAGGCTCC----AGGGTTTG	233	-4	1.74%
TTTATCACAGGCTCCAGAGGAAGGGTTTG	223	+2	1.67%
TTTATCACAGGCTCCAGgGAAGGGTTGG	242	+1 g	1.81%
TTTATCACAGGCT--------------	229	-15	1.71%
TTTATCACA-----------------G	85	-17	0.64%
TTTATCACAGGCTCCA-----------	70	-11	0.52%

P200-YW-W9-Syn34crBCL11Gata-a3-24h-KO-BC3 24h	Ins	Del	HDR
14928 | 50.18%	22.55%	27.63%	0.00%
**Typical seqs**	**Reads**	**Type**	**pct.**
TTTATCACAGGCTCCAG|GAA**GGG**TTTG	7437	WT	49.82%
TTTATCACAGGCTCCAGgGAAGGGTTTG	2080	+1 g	13.93%
TTTATCACAGGCTCCA-GAAGGGTTTG	646	-1	4.33%
TTTATCACAGGCTCC-GGAAGGGTTTG	575	-1	3.85%
TTTATCAC-----------AGGGTTTG	430	-11	2.88%
TTTATCACAGGCT--------------	355	-15	2.38%
TTTATCACAGGCTCC----AGGGTTTG	307	-4	2.06%
TTTATCACAGGCTCCAGAGGAAGGGTTTG	280	+2	1.88%
TTTATCACAGGCTCCAGgGAAGGGTTGG	236	+1 g	1.58%
TTTATCACA-----------------G	157	-17	1.05%

P201-YW-W9-Syn34crBCL11Gata-a3-48h-KO-BC3 48h	Ins	Del	HDR
49351 | 55.50%	18.78%	36.71%	0.00%
**Typical seqs**	**Reads**	**Type**	**pct.**
TTTATCACAGGCTCCAG|GAA**GGG**TTTG	21962	WT	44.50%
TTTATCACAGGCTCCAGgGAAGGGTTTG	5648	+1 g	11.44%
TTTATCAC-----------AGGGTTTG	2237	-11	4.53%
TTTATCACAGGCTCCA-GAAGGGTTTG	1906	-1	3.86%
TTTATCACAGGCTCC-GGAAGGGTTTG	1745	-1	3.54%
TTTATCACAGGCTCC----AGGGTTTG	1732	-4	3.51%
TTTATCACA-----------------G	1110	-17	2.25%
TTTATCACAGGCT--------------	1202	-15	2.44%
TTTATCACAGGCTCCAGgGAAGGGTTGG	731	+1 g	1.48%
TTTATCACAGGCTCCAGAGGAAGGGTTTG	635	+2	1.29%

P197-P201-YW-W9-Syn35crBCL11Gata-a4

P197-YW-W9-Syn35crBCL11Gata-a4-4h-KO-BC4 4h	Ins	Del	HDR
29721 | 23.69%	**20.94%**	**2.75%**	**0.00%**
**Typical seqs**	**Reads**	**Type**	**pct.**
TTTTATCACAGGCTCCA|GGA**AGG**GTTT	22680	WT	76.31%
TTTTATCACAGGCTCCAaGGAAGGGTTT	4833	+1 a	16.26%
TTTTATCACAGGCTCCACAGGAAGGGTTT	410	+2	1.38%
TTTTATCACAGGCTCCAaGGAAGGGTTG	464	+1 a	1.56%
TTTTATCA-------CAGGAAGGGTTT	287	-7	0.97%
TTTTATCACAGGCTCC-GGAAGGGTTT	105	-1	0.35%
TTTTATCACAGGCTCCAaGGAAGGTTTT	110	+1 a	0.37%
TTTTATCACAGGCTC--GGAAGGGTTT	82	-2	0.28%
TTTTATCACAGGCTCCA--AAGGGTTT	49	-2	0.16%
TTTTATCACAGGCT---GGAAGGGTTT	38	-3	0.13%

P198-YW-W9-Syn35crBCL11Gata-a4-8h-KO-BC4 8h	Ins	Del	HDR
17108 | 35.22%	**30.49%**	**4.73%**	**0.00%**
**Typical seqs**	**Reads**	**Type**	**pct.**
TTTTATCACAGGCTCCA|GGA**AGG**GTTT	11082	WT	64.78%
TTTTATCACAGGCTCCAaGGAAGGGTTT	4139	+1 a	24.19%
TTTTATCA-------CAGGAAGGGTTT	300	-7	1.75%
TTTTATCACAGGCTCCAaGGAAGGGTTG	372	+1 a	2.17%
TTTTATCACAGGCTCCACAGGAAGGGTTT	232	+2	1.36%
TTTTATCACAGGCTCCAaGGAAGGTTTT	111	+1 a	0.65%
TTTTATCACAGGCTCC-GGAAGGGTTT	87	-1	0.51%
TTTTATCACAGGCTCCCAGGAAGGGTTT	61	+1 Ins	0.36%
TTTTATCACAGGCTC--GGAAGGGTTT	62	-2	0.36%
TTTTATCACAGGCCCCAAGGAAGGGTTT	45	+1 Ins	0.26%

P199-YW-W9-Syn35crBCL11Gata-a4-12h-KO-BC4 12h	Ins	Del	HDR
23784 | 46.40%	**38.35%**	**8.05%**	**0.00%**
**Typical seqs**	**Reads**	**Type**	**pct.**
TTTTATCACAGGCTCCA|GGA**AGG**GTTT	12748	WT	53.60%
TTTTATCACAGGCTCCAaGGAAGGGTTT	6905	+1 a	29.03%
TTTTATCACAGGCTCCACAGGAAGGGTTT	710	+2	2.99%
TTTTATCACAGGCTCCAaGGAAGGGTTG	508	+1 a	2.14%
TTTTATCA-------CAGGAAGGGTTT	381	-7	1.60%
TTTTATCACAGGCTCC-GGAAGGGTTT	210	-1	0.88%
TTTTATCACAGGCTCCAaGGAAGGTTTT	220	+1 a	0.92%
TTTTATCACAGGCTCCA----------	364	-10	1.53%
TTTTATCACAGGCT-------------	141	-15	0.59%
TTTTATCACAGGCTCCA----------	364	-12	1.53%

P200-YW-W9-Syn35crBCL11Gata-a4-24h-KO-BC4 24h	Ins	Del	HDR
15188 | 57.12%	**44.60%**	**12.52%**	**0.00%**
**Typical seqs**	**Reads**	**Type**	**pct.**
TTTTATCACAGGCTCCA|GGA**AGG**GTTT	6513	WT	42.88%
TTTTATCACAGGCTCCAaGGAAGGGTTT	5221	+1 a	34.38%
TTTTATCA-------CAGGAAGGGTTT	595	-7	3.92%
TTTTATCACAGGCTCCAaGGAAGGGTTG	524	+1 a	3.45%
TTTTATCACAGGCTCCACAGGAAGGGTTT	344	+2	2.26%
TTTTATCACAGGCTCC-GGAAGGGTTT	171	-1	1.13%
TTTTATCACAGGCTCCAaGGAAGGTTTT	171	+1 a	1.13%
TTTTATCACAGGCT-------------	133	-15	0.88%
TTTTATCAC-----------AGGGTTT	118	-11	0.78%
TTTTATCACA-----------------	62	-17	0.41%

P201-YW-W9-Syn35crBCL11Gata-a4-48h-KO-BC4 48h	Ins	Del	HDR
71801 | 57.74%	**39.97%**	**17.77%**	**0.00%**
**Typical seqs**	**Reads**	**Type**	**pct.**
TTTTATCACAGGCTCCA|GGA**AGG**GTTT	30345	WT	42.26%
TTTTATCACAGGCTCCAaGGAAGGGTTT	22276	+1 a	31.02%
TTTTATCA-------CAGGAAGGGTTT	3444	-7	4.80%
TTTTATCACAGGCTCCAaGGAAGGGTTG	1924	+1 a	2.68%
TTTTATCAC-----------AGGGTTT	1599	-11	2.23%
TTTTATCACAGGCTCCACAGGAAGGGTTT	1086	+2	1.51%
TTTTATCACA-----------------	877	-17	1.22%
TTTTATCACAGGCTCC-GGAAGGGTTT	766	-1	1.07%
TTTTATCACAGGCT-------------	714	-15	0.99%
TTTTATCACAGGCTCCAaGGAAGGTTTT	656	+1 a	0.91%

P197-P201-YW-W9-Syn36crBCL11Gata-a5

P197-YW-W9-Syn36crBCL11Gata-a5-4h-KO-BC5 4h	Ins	Del	HDR
33154 | 15.35%	8.39%	6.96%	0.00%
**Typical seqs**	**Reads**	**Type**	**pct.**
TTGCTTTTATCACAGGC|TCCAGGAAGG	28065	WT	84.65%
TTGCTTTTATCACAGGCcTCCAGGAAGG	1899	+1 c	5.73%
TTGCTTTTATCACAGGC-CCAGGAAGG	727	-1	2.19%
TTGCTTTTATCACAGGCtTCCAGGAAGG	513	+1 t	1.55%
TTGCTTTTA--------TCCAGGAAGG	292	-8	0.88%
TTGCTTTTATCACAGG--CCAGGAAGG	300	-2	0.90%
TTGCTTTTATCACAGG-TCCAGGAAGG	185	-1	0.56%
TTGCTTTTATCA-------CAGGAAGG	192	-7	0.58%
TTGCTTTTATCACAGGCaTCCAGGAAGG	133	+1 a	0.40%
TTGCTTTTATCACAGGC-----GAAGG	88	-5	0.27%

P198-YW-W9-Syn36crBCL11Gata-a5-8h-KO-BC5 8h	Ins	Del	HDR
30833 | 28.33%	13.35%	14.98%	0.00%
**Typical seqs**	**Reads**	**Type**	**pct.**
TTGCTTTTATCACAGGC|TCCAGGAAGG	22099	WT	71.67%
TTGCTTTTATCACAGGCcTCCAGGAAGG	2377	+1 c	7.71%
TTGCTTTTATCACAGGCtTCCAGGAAGG	1093	+1 t	3.54%
TTGCTTTTATCACAGGC-CCAGGAAGG	861	-1	2.79%
TTGCTTTTA--------TCCAGGAAGG	643	-8	2.09%
TTGCTTTTATCA-------CAGGAAGG	572	-7	1.86%
TTGCTTTTATCACAGG-TCCAGGAAGG	470	-1	1.52%
TTGCTTTTATCACAGG--CCAGGAAGG	385	-2	1.25%
TTGCTTTTATCACAGGC----------	303	-14	0.98%
TTGCTTTTATCACAGGC----GGAAGG	187	-4	0.61%

P199-YW-W9-Syn36crBCL11Gata-a5-12h-KO-BC5 12h	Ins	Del	HDR
21213 | 36.80%	15.30%	21.50%	0.00%
**Typical seqs**	**Reads**	**Type**	**pct.**
TTGCTTTTATCACAGGC|TCCAGGAAGG	13407	WT	63.20%
TTGCTTTTATCACAGGCcTCCAGGAAGG	1736	+1 c	8.18%
TTGCTTTTATCACAGGC-CCAGGAAGG	906	-1	4.27%
TTGCTTTTA--------TCCAGGAAGG	769	-8	3.63%
TTGCTTTTATCACAGGCtTCCAGGAAGG	847	+1 t	3.99%
TTGCTTTTATCACAGG--CCAGGAAGG	403	-2	1.90%
TTGCTTTTATCA-------CAGGAAGG	335	-7	1.58%
TTGCTTTTATCACAGGCaTCCAGGAAGG	238	+1 a	1.12%
TTGCTTTTATCACAGG-TCCAGGAAGG	221	-1	1.04%
TTGCTTTT---------TCCAGGAAGG	162	-9	0.76%

P200-YW-W9-Syn36crBCL11Gata-a5-24h-KO-BC5 24h	Ins	Del	HDR
23058 | 52.75%	16.72%	36.04%	0.00%
**Typical seqs**	**Reads**	**Type**	**pct.**
TTGCTTTTATCACAGGC|TCCAGGAAGG	10894	WT	47.25%
TTGCTTTTATCA-------CAGGAAGG	1936	-7	8.40%
TTGCTTTTATCACAGGCcTCCAGGAAGG	1820	+1 c	7.89%
TTGCTTTTA--------TCCAGGAAGG	1754	-8	7.61%
TTGCTTTTATCACAGGCtTCCAGGAAGG	1260	+1 t	5.46%
TTGCTTTTATCACAGGC-CCAGGAAGG	1014	-1	4.40%
TTGCTTTTATCACAGG--CCAGGAAGG	530	-2	2.30%
TTGCTTTTATCACAGG-TCCAGGAAGG	351	-1	1.52%
TTGCTTTTATCACAGGC-------AGG	217	-7	0.94%
TTGCTTTTATCACAGGCaTCCAGGAAGG	228	+1 a	0.99%

P201-YW-W9-Syn36crBCL11Gata-a5-48h-KO-BC5 48h	Ins	Del	HDR
53938 | 54.50%	13.66%	40.84%	0.00%
**Typical seqs**	**Reads**	**Type**	**pct.**
TTGCTTTTATCACAGGC|TCCAGGAAGG	24542	WT	45.50%
TTGCTTTTATCA-------CAGGAAGG	7595	-7	14.08%
TTGCTTTTA--------TCCAGGAAGG	4476	-8	8.30%
TTGCTTTTATCACAGGCcTCCAGGAAGG	2835	+1 c	5.26%
TTGCTTTTATCACAGGC-CCAGGAAGG	2173	-1	4.03%
TTGCTTTTATCACAGGCtTCCAGGAAGG	2234	+1 t	4.14%
TTGCTTTTATCACAGG-TCCAGGAAGG	914	-1	1.69%
TTGCTTTTATCACAGG--CCAGGAAGG	709	-2	1.31%
TTGCTTTTATCACAGGC-------AGG	423	-7	0.78%
TTGCTTTTATCACAGGCaTCCAGGAAGG	396	+1 a	0.73%

P208-YW-W9-Syn93crBCL11A7c-RNPko

P208-YW-W9-Syn93crBCL11A7c-4h-RNPko-BC1 4h	Ins	Del	HDR
46086 | 10.04%	6.13%	3.92%	0.00%
**Typical seqs**	**Reads**	**Type**	**pct.**
AGCTTCGTGACGTTGGA|TGG**AGG**GTTT	41457	WT	89.96%
AGCTTCGTGACGTTGGAaTGGAGGGTTT	2658	+1 a	5.77%
AGCTTCGTGACGT----TGGAGGGTTT	858	-4	1.86%
AGCTTCGTGACGTTGGA-GGAGGGTTT	589	-1	1.28%
AGCTTCGTGACGTTGG-TGGAGGGTTT	78	-1	0.17%
AGCTTCGTGACGTTG--TGGAGGGTTT	76	-2	0.16%
AGCTTCGTGACGTTGGAtTGGAGGGTTT	75	+1 t	0.16%
AGCTTCGTGACGTTGGA--GAGGGTTT	35	-2	0.08%
AGCTTCGTGACGTT-------GGGTTT	30	-7	0.07%
AGCTTCGTGACGTT--------GGTTT	24	-8	0.05%

P208-YW-W9-Syn93crBCL11A7c-8h-RNPko-BC2 8h	Ins	Del	HDR
55049 | 25.14%	14.90%	10.24%	0.00%
**Typical seqs**	**Reads**	**Type**	**pct.**
AGCTTCGTGACGTTGGA|TGG**AGG**GTTT	41212	WT	74.86%
AGCTTCGTGACGTTGGAaTGGAGGGTTT	7582	+1 a	13.77%
AGCTTCGTGACGT----TGGAGGGTTT	2145	-4	3.90%
AGCTTCGTGACGTTGGA-GGAGGGTTT	1849	-1	3.36%
AGCTTCGTGACGTTG--TGGAGGGTTT	293	-2	0.53%
AGCTTCGTGACGTTGGAtTGGAGGGTTT	279	+1 t	0.51%
AGCTTCGTGACGTTGG-TGGAGGGTTT	145	-1	0.26%
AGCTTCGTGACGTTGGA--GAGGGTTT	120	-2	0.22%
AGCTTCG----------TGGAGGGTTT	111	-10	0.20%
AGCTTCGTGACGTT-------GGGTTT	107	-7	0.19%

P208-YW-W9-Syn93crBCL11A7c-12h-RNPko-BC3 12h	Ins	Del	HDR
80669 | 34.59%	19.32%	15.27%	0.00%
**Typical seqs**	**Reads**	**Type**	**pct.**
AGCTTCGTGACGTTGGA|TGG**AGG**GTTT	52764	WT	65.41%
AGCTTCGTGACGTTGGAaTGGAGGGTTT	14323	+1 a	17.76%
AGCTTCGTGACGT----TGGAGGGTTT	5512	-4	6.83%
AGCTTCGTGACGTTGGA-GGAGGGTTT	3201	-1	3.97%
AGCTTCGTGACGTTGGAtTGGAGGGTTT	355	+1 t	0.44%
AGCTTCG----------TGGAGGGTTT	352	-10	0.44%
AGCTTCGTGACGTTGGA--GAGGGTTT	279	-2	0.35%
AGCTTCGTGACGTTG--TGGAGGGTTT	272	-2	0.34%
AGCTTCGTGACGTTGG-TGGAGGGTTT	259	-1	0.32%
AGCTTCGTGACGTT--------GGTTT	211	-8	0.26%

P208-YW-W9-Syn93crBCL11A7c-24h-RNPko-BC4 24h	Ins	Del	HDR
92841 | 46.63%	21.44%	25.19%	0.00%
**Typical seqs**	**Reads**	**Type**	**pct.**
AGCTTCGTGACGTTGGA|TGG**AGG**GTTT	49550	WT	53.37%
AGCTTCGTGACGTTGGAaTGGAGGGTTT	17561	+1 a	18.92%
AGCTTCGTGACGT----TGGAGGGTTT	11969	-4	12.89%
AGCTTCGTGACGTTGGA-GGAGGGTTT	3981	-1	4.29%
AGCTTCGTGACGTTGGAtTGGAGGGTTT	701	+1 t	0.76%
AGCTTCGTGACGTTG--TGGAGGGTTT	607	-2	0.65%
AGCTTCGTGACGTTGG-TGGAGGGTTT	495	-1	0.53%
AGCTTCGTGAC------------GTTT	487	-12	0.52%
AGCTTCG----------TGGAGGGTTT	389	-10	0.42%
AGCTTCGT-------GATGGAGGGTTT	314	-7	0.34%

P208-YW-W9-Syn93crBCL11A7c-48h-RNPko-BC5 48h	Ins	Del	HDR
68117 | 59.62%	20.71%	38.90%	0.01%
**Typical seqs**	**Reads**	**Type**	**pct.**
AGCTTCGTGACGTTGGA|TGG**AGG**GTTT	27505	WT	40.38%
AGCTTCGTGACGT----TGGAGGGTTT	16471	-4	24.18%
AGCTTCGTGACGTTGGAaTGGAGGGTTT	12432	+1 a	18.25%
AGCTTCGTGACGTTGGA-GGAGGGTTT	2626	-1	3.86%
AGCTTCGTGACGTT-------GGGTTT	620	-7	0.91%
AGCTTCGTGAC------------GTTT	540	-12	0.79%
AGCTTCGTGACGTTG--TGGAGGGTTT	533	-2	0.78%
AGCTTCGTGACGTTGGAtTGGAGGGTTT	526	+1 t	0.77%
AGCTTCGTGACGTTGG-TGGAGGGTTT	509	-1	0.75%
AGCTTCGTGACG-----TGGAGGGTTT	365	-5	0.54%

P209-YW-W9-Syn93crBCL11A7c-48h-RNPaav

P209-YW-W9-Syn93crBCL11A7c-4h-RNPaav-BC1 **4h**	Ins	Del	HDR
68255 | 10.67%	**5.78%**	**3.05%**	**1.84%**
**Typical seqs**	**Reads**	**Type**	**pct.**
AGCTTCGTGACGTTGGA|TGG**AGG**GTTT	60974	WT	89.33%
AGCTTCGTGACGTTGGAaTGGAGGGTTT	3678	+1 a	5.39%
AGCTTCGTGACGTTGGATGTTTAAACGGAG GGTTT	1254	+8 HDR	1.84%
AGCTTCGTGACGTTGGA-GGAGGGTTT	1173	-1	1.72%
AGCTTCGTGACGT----TGGAGGGTTT	501	-4	0.73%
AGCTTCGTGACGTTGGA--GAGGGTTT	139	-2	0.20%
AGCTTCGTGACGTTGGAtTGGAGGGTTT	115	+1 t	0.17%
AGCTTCGTGACGTTGG-TGGAGGGTTT	57	-1	0.08%
AGCTTCGTGACGTTG--TGGAGGGTTT	37	-2	0.05%
AGCTTCGTGACGTTGGAgTGGAGGGTTT	29	+1 g	0.04%

P209-YW-W9-Syn93crBCL11A7c-8h-RNPaav-BC2 **8h**	Ins	Del	HDR
61809 | 27.56%	**13.65%**	**7.86%**	**6.06%**
**Typical seqs**	**Reads**	**Type**	**pct.**
AGCTTCGTGACGTTGGA|TGG**AGG**GTTT	44775	WT	72.44%
AGCTTCGTGACGTTGGAaTGGAGGGTTT	7679	+1 a	12.42%
AGCTTCGTGACGTTGGATGTTTAAACGGAG GGTTT	3707	+8 HDR	6.00%
AGCTTCGTGACGTTGGA-GGAGGGTTT	2475	-1	4.00%
AGCTTCGTGACGT----TGGAGGGTTT	1264	-4	2.05%
AGCTTCGTGACGTTGGAtTGGAGGGTTT	219	+1 t	0.35%
AGCTTCGTGACGTTGG-TGGAGGGTTT	184	-1	0.30%
AGCTTCGTGACGTTGGA--GAGGGTTT	129	-2	0.21%
AGCTTCGTGACGTT-GATGGAGGGTTT	128	-1	0.21%
AGCTTCGTGACGTTGGAGATGGAGGGTTT	90	+2	0.15%

P209-YW-W9-Syn93crBCL11A7c-12h-RNPaav-BC3 **12h**	Ins	Del	HDR
71363 | 44.50%	**18.13%**	**11.59%**	**14.78%**
**Typical seqs**	**Reads**	**Type**	**pct.**
AGCTTCGTGACGTTGGA|TGG**AGG**GTTT	39608	WT	55.50%
AGCTTCGTGACGTTGGAaTGGAGGGTTT	11625	+1 a	16.29%
AGCTTCGTGACGTTGGATGTTTAAACGGAG GGTTT	10396	+8 HDR	14.57%
AGCTTCGTGACGTTGGA-GGAGGGTTT	3641	-1	5.10%
AGCTTCGTGACGT----TGGAGGGTTT	1980	-4	2.77%
AGCTTCGTGACGTTGGAtTGGAGGGTTT	452	+1 t	0.63%
AGCTTCGTGACGTTGGA--GAGGGTTT	294	-2	0.41%
AGCTTCGTGACGTTG--TGGAGGGTTT	222	-2	0.31%
AGCTTCGTGACGTTGG-TGGAGGGTTT	157	-1	0.22%
AGCTTCGTGACGTT--ATGGAGGGTTT	153	-2	0.21%

P209-YW-W9-Syn93crBCL11A7c-24h-RNPaav-BC4 **24h**	Ins	Del	HDR
76974 | 61.81%	**22.34%**	**16.79%**	**22.68%**
**Typical seqs**	**Reads**	**Type**	**pct.**
AGCTTCGTGACGTTGGA|TGG**AGG**GTTT	29394	WT	38.19%
AGCTTCGTGACGTTGGATGTTTAAACGGAG GGTTT	17121	+8 HDR	22.24%
AGCTTCGTGACGTTGGAaTGGAGGGTTT	14875	+1 a	19.32%
AGCTTCGTGACGTTGGA-GGAGGGTTT	4099	-1	5.33%
AGCTTCGTGACGT----TGGAGGGTTT	3537	-4	4.60%
AGCTTCGTGACGTTG--TGGAGGGTTT	598	-2	0.78%
AGCTTCGTGACGTTGGAtTGGAGGGTTT	477	+1 t	0.62%
AGCTTCG----------TGGAGGGTTT	354	-10	0.46%
AGCTTCGTGACGTTGGA--GAGGGTTT	348	-2	0.45%
AGCTTCGTGACGTTGG-TGGAGGGTTT	313	-1	0.41%

P209-YW-W9-Syn93crBCL11A7c-48h-RNPaav-BC5 **48h**	Ins	Del	HDR
50177 | 69.61%	**23.55%**	**19.24%**	**26.82%**
**Typical seqs**	**Reads**	**Type**	**pct.**
AGCTTCGTGACGTTGGA|TGG**AGG**GTTT	15247	WT	30.39%
AGCTTCGTGACGTTGGATGTTTAAACGGAG GGTTT	13156	+8 HDR	26.22%
AGCTTCGTGACGTTGGAaTGGAGGGTTT	10095	+1 a	20.12%
AGCTTCGTGACGT----TGGAGGGTTT	2987	-4	5.95%
AGCTTCGTGACGTTGGA-GGAGGGTTT	3040	-1	6.06%
AGCTTCGTGACGTTGGAtTGGAGGGTTT	553	+1 t	1.10%
AGCTTCGTGACGTTG--TGGAGGGTTT	371	-2	0.74%
AGCTTCGTGACGTTGGA--GAGGGTTT	325	-2	0.65%
AGCTTCG----------TGGAGGGTTT	277	-10	0.55%
AGCTTCGTGACGTTGG-TGGAGGGTTT	238	-1	0.47%

P208-YW-W9-Syn96crBCL11A8b-RNPko

P208-YW-W9-Syn96crBCL11A8b-4h-RNPko-BC1 4h	Ins	Del	HDR
29885 | 3.75%	1.20%	2.55%	0.00%
**Typical seqs**	**Reads**	**Type**	**pct.**
GCGGGCCACCCACATTC|ATT**GGG**GGAA	28763	WT	96.25%
GCGGGCCACCCA----CATTGGGGGAA	357	-4	1.19%
GCGGGCCACCCACATTCcATTGGGGGAA	215	+1 c	0.72%
GCGGGCCACCCACATT-ATTGGGGGAA	128	-1	0.43%
GCGGGCCACCCACATTC-TTGGGGGAA	93	-1	0.31%
GCGGGCCACCCACAT--ATTGGGGGAA	91	-2	0.30%
GCGGGCCACCCACATTCtATTGGGGGAA	51	+1 t	0.17%
GCGGGCCACCCACATTCCCCCACATTGGGGGAA	34	+6	0.11%
GCGGGCCACCCACATTCCACCCACATTGGGGGAA	17	+7	0.06%
GCGGGCCACC-----ACATTGGGGGAA	15	-5	0.05%

P208-YW-W9-Syn96crBCL11A8b-8h-RNPko-BC2 8h	Ins	Del	HDR
23885 | 12.15%	1.93%	10.21%	0.00%
**Typical seqs**	**Reads**	**Type**	**pct.**
GCGGGCCACCCACATTC|ATT**GGG**GGAA	20984	WT	87.85%
GCGGGCCACCCA----CATTGGGGGAA	1114	-4	4.66%
GCGGGCCACCCACATTC-TTGGGGGAA	365	-1	1.53%
GCGGGCCACCCACATT-ATTGGGGGAA	319	-1	1.34%
GCGGGCCACCCACATTCcATTGGGGGAA	188	+1 c	0.79%
GCGGGCCACCCACATTCaATTGGGGGAA	116	+1 a	0.49%
GCGGGCCACCCACAT--ATTGGGGGAA	85	-2	0.36%
GCGGGCCACCCACATTCtATTGGGGGAA	71	+1 t	0.30%
GCGGGCCACCCACATTC--TGGGGGAA	53	-2	0.22%
GCGGGCCACCCACATTC---GGGGGAA	39	-3	0.16%

P208-YW-W9-Syn96crBCL11A8b-12h-RNPko-BC3 12h	Ins	Del	HDR
47838 | 21.99%	3.58%	18.41%	0.00%
**Typical seqs**	**Reads**	**Type**	**pct.**
GCGGGCCACCCACATTC|ATT**GGG**GGAA	37318	WT	78.01%
GCGGGCCACCCA----CATTGGGGGAA	2911	-4	6.09%
GCGGGCCACCCACATT-ATTGGGGGAA	1225	-1	2.56%
GCGGGCCACCCACATTC-TTGGGGGAA	1062	-1	2.22%
GCGGGCCACCCACATTCcATTGGGGGAA	756	+1 c	1.58%
GCGGGCCACCCACAT--ATTGGGGGAA	416	-2	0.87%
GCGGGCCACCCACATTCaATTGGGGGAA	313	+1 a	0.65%
GCGGGCCACCCACA---ATTGGGGGAA	259	-3	0.54%
GCGGGCCACCCACATTC---GGGGGAA	190	-3	0.40%
GCGGGCCACCCACATTC--TGGGGGAA	189	-2	0.40%

P208-YW-W9-Syn96crBCL11A8b-24h-RNPko-BC4 24h	Ins	Del	HDR
46492 | 41.98%	6.71%	35.27%	0.00%
**Typical seqs**	**Reads**	**Type**	**pct.**
GCGGGCCACCCACATTC|ATT**GGG**GGAA	26974	WT	58.02%
GCGGGCCACCCA----CATTGGGGGAA	5482	-4	11.79%
GCGGGCCACCCACATT-ATTGGGGGAA	1703	-1	3.66%
GCGGGCCACCCACATTC-TTGGGGGAA	1135	-1	2.44%
GCGGGCCACCCACATTCaATTGGGGGAA	922	+1 a	1.98%
GCGGGCCACCCACATTCcATTGGGGGAA	707	+1 c	1.52%
GCGGGCCACCCACAT--ATTGGGGGAA	656	-2	1.41%
GCGGGCCACCCACATTCtATTGGGGGAA	372	+1 t	0.80%
GCGGG-----------CATTGGGGGAA	340	-11	0.73%
GCGGGCCACCCACATTC--TGGGGGAA	327	-2	0.70%

P208-YW-W9-Syn96crBCL11A8b-48h-RNPko-BC5 48h	Ins	Del	HDR
41651 | 52.29%	7.11%	45.17%	0.00%
**Typical seqs**	**Reads**	**Type**	**pct.**
GCGGGCCACCCACATTC|ATT**GGG**GGAA	19873	WT	47.71%
GCGGGCCACCCA----CATTGGGGGAA	8016	-4	19.25%
GCGGGCCACCCACATT-ATTGGGGGAA	1516	-1	3.64%
GCGGGCCACCCACATTC-TTGGGGGAA	1109	-1	2.66%
GCGGGCCACCCACATTCaATTGGGGGAA	601	+1 a	1.44%
GCGGGCCACCCACAT--ATTGGGGGAA	522	-2	1.25%
GCGGGCCACCCACATTCcATTGGGGGAA	452	+1 c	1.09%
GCGGGCCACCCACATTCtATTGGGGGAA	367	+1 t	0.88%
GCGGGCCACCCACA---ATTGGGGGAA	327	-3	0.79%
GCGGGCCACCC----------------	383	-19	0.92%

P208-YW-W9-Syn99crBCL11A9a-RNPko

P208-YW-W9-Syn99crBCL11A9a-4h-RNPko-BC1 4h	Ins	Del	HDR
40743 | 24.32%	23.11%	1.21%	0.00%
**Typical seqs**	**Reads**	**Type**	**pct.**
GCGCAAGCTCCCGTTCT|CCG**AGG**AGTG	30834	WT	75.68%
GCGCAAACTCCCGTTCTTCCGAGGAGTG	4704	+1 Ins	11.55%
GCGCAAGCTCCCGTTCTtCCGAGGAGTG	4379	+1 t	10.75%
GCGCAAACTC-------CCGAGGAGTG	165	-7	0.40%
GCGCAAGCTC-------CCGAGGAGTG	78	-7	0.19%
GCGCAAACTCCCGTTCTTTCCGAGGAGTG	47	+2	0.12%
GCGCAAACTCCCGT--TCCGAGGAGTG	45	-2	0.11%
GCGCAAGCACTC-------CCGAGGAGTG	25	-5	0.06%
GCGCAAACTCCCG---TCCGAGGAGTG	25	-3	0.06%
GCGCAAGCTCCCGTTCTTTCCGAGGAGTG	23	+2	0.06%

P208-YW-W9-Syn99crBCL11A9a-8h-RNPko-BC2 8h	Ins	Del	HDR
27423 | 48.41%	44.69%	3.72%	0.00%
**Typical seqs**	**Reads**	**Type**	**pct.**
GCGCAAGCTCCCGTTCT|CCG**AGG**AGTG	14147	WT	51.59%
GCGCAAACTCCCGTTCTTCCGAGGAGTG	5867	+1 Ins	21.39%
GCGCAAGCTCCCGTTCTtCCGAGGAGTG	5778	+1 t	21.07%
GCGCAAACTC-------CCGAGGAGTG	307	-7	1.12%
GCGCAAGCTC-------CCGAGGAGTG	194	-7	0.71%
GCGCAAACTCCCGTTCTTTCCGAGGAGTG	86	+2	0.31%
GCGCAAGCTCCCGTTCTTTCCGAGGAGTG	70	+2	0.26%
GCGCAAACTCCCGT--TCCGAGGAGTG	68	-2	0.25%
GCGCAAGCTCCCGT--TCCGAGGAGTG	55	-2	0.20%
GCGCAAGCTCCCG---TCCGAGGAGTG	41	-3	0.15%

P208-YW-W9-Syn99crBCL11A9a-12h-RNPko-BC3 12h	Ins	Del	HDR
56054 | 62.34%	55.99%	6.35%	0.00%
**Typical seqs**	**Reads**	**Type**	**pct.**
GCGCAAGCTCCCGTTCT|CCG**AGG**AGTG	21111	WT	37.66%
GCGCAAGCTCCCGTTCTtCCGAGGAGTG	15124	+1 t	26.98%
GCGCAAACTCCCGTTCTTCCGAGGAGTG	14569	+1 Ins	25.99%
GCGCAAACTC-------CCGAGGAGTG	905	-7	1.61%
GCGCAAGCTC-------CCGAGGAGTG	459	-7	0.82%
GCGCAAACTCCCGTTCTTTCCGAGGAGTG	391	+2	0.70%
GCGCAAACTCCCGT--TCCGAGGAGTG	204	-2	0.36%
GCGCAAGCTCCCGT--TCCGAGGAGTG	198	-2	0.35%
GCGCAAGCTCCCGTTCTTTCCGAGGAGTG	134	+2	0.24%
GCGCAAACTCCCG---TCCGAGGAGTG	126	-3	0.22%

P208-YW-W9-Syn99crBCL11A9a-24h-RNPko-BC4 24h	Ins	Del	HDR
56584 | 80.39%	68.89%	11.50%	0.00%
**Typical seqs**	**Reads**	**Type**	**pct.**
GCGCAAGCTCCCGTTCT|CCG**AGG**AGTG	11095	WT	19.61%
GCGCAAGCTCCCGTTCTtCCGAGGAGTG	18846	+1 t	33.31%
GCGCAAACTCCCGTTCTTCCGAGGAGTG	17467	+1 Ins	30.87%
GCGCAAACTC-------CCGAGGAGTG	1608	-7	2.84%
GCGCAAGCTC-------CCGAGGAGTG	1116	-7	1.97%
GCGCAAACTCCCGTTCTTTCCGAGGAGTG	491	+2	0.87%
GCGCAAGCTCCCGTTCTTTCCGAGGAGTG	382	+2	0.68%
GCGCAAACTCCCGT--TCCGAGGAGTG	272	-2	0.48%
GCGCAAGCTCCCGT--TCCGAGGAGTG	183	-2	0.32%
GCGCAAA--------CTCCGAGGAGTG	162	-8	0.29%

P208-YW-W9-Syn99crBCL11A9a-48h-RNPko-BC5 48h	Ins	Del	HDR
48980 | 83.94%	66.09%	17.85%	0.00%
**Typical seqs**	**Reads**	**Type**	**pct.**
GCGCAAGCTCCCGTTCT|CCG**AGG**AGTG	7867	WT	16.06%
GCGCAAGCTCCCGTTCTtCCGAGGAGTG	15623	+1 t	31.90%
GCGCAAACTCCCGTTCTTCCGAGGAGTG	14525	+1 Ins	29.65%
GCGCAAACTC-------CCGAGGAGTG	2767	-7	5.65%
GCGCAAGCTC-------CCGAGGAGTG	1416	-7	2.89%
GCGCAAACTCCCGT--TCCGAGGAGTG	410	-2	0.84%
GCGCAAACTCCCGTTCTTTCCGAGGAGTG	302	+2	0.62%
GCGCAAGCTCCCGT--TCCGAGGAGTG	236	-2	0.48%
GCGCAAGCTCCCGTTCTTTCCGAGGAGTG	230	+2	0.47%
GCGCAA--------GCTCCGAGGAGTG	154	-8	0.31%

P208-YW-W9-Syn100crBCL11A9b-RNPko

P208-YW-W9-Syn100crBCL11A9b-4h-RNPko-BC1 4h	Ins	Del	HDR
28496 | 13.24%	9.52%	3.72%	0.00%
**Typical seqs**	**Reads**	**Type**	**pct.**
GTCGGACTTGACCGTCA TGG**GGG**ACGA	24722	WT	86.76%
GTCGGACTTGACCGTCAaTGGGGGACGA	2200	+1 a	7.72%
GTCGGACTTGACCGTCA-GGGGGACGA	539	-1	1.89%
GTCGGACTTGACCGTCAtTGGGGGACGA	352	+1 t	1.24%
GTCGGACTTGACCGTC-TGGGGGACGA	177	-1	0.62%
GTCGGACTTGACCGTCA--GGGGACGA	87	-2	0.31%
GTCGGACTTGACC---------GACGA	36	-9	0.13%
GTCGGACTTG-------TGGGGGACGA	33	-7	0.12%
GTCGGACTTGAC------------CGA	29	-12	0.10%
GTCGGACTT-------------GACGA	21	-13	0.07%

P208-YW-W9-Syn100crBCL11A9b-8h-RNPko-BC2 8h	Ins	Del	HDR
26286 | 28.19%	19.89%	8.30%	0.00%
**Typical seqs**	**Reads**	**Type**	**pct.**
GTCGGACTTGACCGTCA TGG**GGG**ACGA	18877	WT	71.81%
GTCGGACTTGACCGTCAaTGGGGGACGA	4220	+1 a	16.05%
GTCGGACTTGACCGTCA-GGGGGACGA	931	-1	3.54%
GTCGGACTTGACCGTCAtTGGGGGACGA	591	+1 t	2.25%
GTCGGACTTGACCGTCA--GGGGACGA	336	-2	1.28%
GTCGGACTTGACCGTC-TGGGGGACGA	316	-1	1.20%
GTCGGACTTGACCGTCAgTGGGGGACGA	79	+1 g	0.30%
GTCGGACT---------TGGGGGACGA	47	-9	0.18%
GTCGGACTTGACC---------GACGA	43	-9	0.16%
GTCGGACTTGACCGT--TGGGGGACGA	39	-2	0.15%

P208-YW-W9-Syn100crBCL11A9b-12h-RNPko-BC3 12h	Ins	Del	HDR
36736 | 42.61%	30.00%	12.61%	0.00%
**Typical seqs**	**Reads**	**Type**	**pct.**
GTCGGACTTGACCGTCA TGG**GGG**ACGA	21083	WT	57.39%
GTCGGACTTGACCGTCAaTGGGGGACGA	8341	+1 a	22.71%
GTCGGACTTGACCGTCA-GGGGGACGA	1824	-1	4.97%
GTCGGACTTGACCGTCAtTGGGGGACGA	1605	+1 t	4.37%
GTCGGACTTGACCGTC-TGGGGGACGA	481	-1	1.31%
GTCGGACTTGACCGTCAgTGGGGGACGA	241	+1 g	0.66%
GTCGGACTTGACCGTCA--GGGGACGA	216	-2	0.59%
GTCGGACTTGAC------------CGA	143	-12	0.39%
GTCGGACTTGACC---------GACGA	142	-9	0.39%
GTCGGACTTGACCGTCAcTGGGGGACGA	104	+1 c	0.28%

P208-YW-W9-Syn100crBCL11A9b-24h-RNPko-BC4 24h	Ins	Del	HDR
52681 | 56.45%	34.90%	21.54%	0.01%
**Typical seqs**	**Reads**	**Type**	**pct.**
GTCGGACTTGACCGTCA TGG**GGG**ACGA	22943	WT	43.55%
GTCGGACTTGACCGTCAaTGGGGGACGA	13383	+1 a	25.40%
GTCGGACTTGACCGTCAtTGGGGGACGA	2905	+1 t	5.51%
GTCGGACTTGACCGTCA-GGGGGACGA	2689	-1	5.10%
GTCGGACTTGACCGTC-TGGGGGACGA	751	-1	1.43%
GTCGGACTTGACCGTCA--GGGGACGA	609	-2	1.16%
GTCGGACTT-------------GACGA	573	-13	1.09%
GTC------------------GGACGA	411	-18	0.78%
GTCGGACTTGACCGTCAgTGGGGGACGA	387	+1 g	0.73%
GTCGGACTTGACCGT---------CGA	360	-9	0.68%

P208-YW-W9-Syn100crBCL11A9b-48h-RNPko-BC5 48h	Ins	Del	HDR
39050 | 63.09%	34.90%	28.19%	0.00%
**Typical seqs**	**Reads**	**Type**	**pct.**
GTCGGACTTGACCGTCA TGG**GGG**ACGA	14415	WT	36.91%
GTCGGACTTGACCGTCAaTGGGGGACGA	9209	+1 a	23.58%
GTCGGACTTGACCGTCAtTGGGGGACGA	2332	+1 t	5.97%
GTCGGACTTGACCGTCA-GGGGGACGA	1804	-1	4.62%
GTCGGACTT-------------GACGA	962	-13	2.46%
GTCGGACTTGACCGTC-TGGGGGACGA	622	-1	1.59%
GTCGGACTTGACCGTCAgTGGGGGACGA	581	+1 g	1.49%
GTC------------------GGACGA	517	-18	1.32%
GTCGGACT---------TGGGGGACGA	466	-9	1.19%
GT----------------------CGA	417	-22	1.07%

P209-YW-W9-Syn100crBCL11A9b-48h-RNPaav

P209-YW-W9-Syn100crBCL11A9b-4h-RNPaav-BC1 **4h**	Ins	Del	HDR
49963 | 12.82%	**7.98%**	**4.50%**	**0.34%**
**Typical seqs**	**Reads**	**Type**	**pct.**
GTCGGACTTGACCGTCA|TGG**GGG**ACGA	43560	WT	87.18%
GTCGGACTTGACCGTCAaTGGGGGACGA	3217	+1 a	6.44%
GTCGGACTTGACCGTCA-GGGGGACGA	1036	-1	2.07%
GTCGGACTTGACCGTC-TGGGGGACGA	642	-1	1.28%
GTCGGACTTGACCGTCAtTGGGGGACGA	452	+1 t	0.90%
GTCGGACTTGACCGTCA--GGGGACGA	241	-2	0.48%
GTCGGACTTGACCGTCAGTTTAAACTGGGG GACGA	165	+8 HDR	0.33%
GTCGGACTTGACCGTCAgTGGGGGACGA	75	+1 g	0.15%
GTCGGACTTGACCGTCAT-GGGGACGA	33	-1	0.07%
GTCGGACTTGACCGTCACATGGGGGACGA	27	+2	0.05%

P209-YW-W9-Syn100crBCL11A9b-8h-RNPaav-BC2 **8h**	Ins	Del	HDR
45248 | 33.95%	**20.72%**	**11.19%**	**2.04%**
**Typical seqs**	**Reads**	**Type**	**pct.**
GTCGGACTTGACCGTCA|TGG**GGG**ACGA	29887	WT	66.05%
GTCGGACTTGACCGTCAaTGGGGGACGA	7160	+1 a	15.82%
GTCGGACTTGACCGTCA-GGGGGACGA	2379	-1	5.26%
GTCGGACTTGACCGTCAtTGGGGGACGA	1348	+1 t	2.98%
GTCGGACTTGACCGTCAGTTTAAACTGGGG GACGA	890	+8 HDR	1.97%
GTCGGACTTGACCGTC-TGGGGGACGA	762	-1	1.68%
GTCGGACTTGACCGTCA--GGGGACGA	655	-2	1.45%
GTCGGACTTGACCGTCAgTGGGGGACGA	184	+1 g	0.41%
GTCGGACTTGACCGTCA---GGGACGA	133	-3	0.29%
GTCGGACTTGACCGTCAAATGGGGGACGA	78	+2	0.17%

P209-YW-W9-Syn100crBCL11A9b-12h-RNPaav-BC3 **12h**	Ins	Del	HDR
52344 | 48.26%	**26.18%**	**15.57%**	**6.51%**
**Typical seqs**	**Reads**	**Type**	**pct.**
GTCGGACTTGACCGTCA|TGG**GGG**ACGA	27082	WT	51.74%
GTCGGACTTGACCGTCAaTGGGGGACGA	10533	+1 a	20.12%
GTCGGACTTGACCGTCA-GGGGGACGA	3494	-1	6.68%
GTCGGACTTGACCGTCAGTTTAAACTGGGG GACGA	3253	+8 HDR	6.21%
GTCGGACTTGACCGTCAtTGGGGGACGA	1741	+1 t	3.33%
GTCGGACTTGACCGTC-TGGGGGACGA	801	-1	1.53%
GTCGGACTTGACCGTCA--GGGGACGA	773	-2	1.48%
GTCGGACTTGACCGTCAgTGGGGGACGA	203	+1 g	0.39%
GTCGGACTTGACCGTCA----GGACGA	183	-4	0.35%
GTCGGACTTGACCGTCA---GGGACGA	158	-3	0.30%

P209-YW-W9-Syn100crBCL11A9b-24h-RNPaav-BC4 **24h**	Ins	Del	HDR
45454 | 65.86%	**27.57%**	**21.31%**	**16.97%**
**Typical seqs**	**Reads**	**Type**	**pct.**
GTCGGACTTGACCGTCA|TGG**GGG**ACGA	15519	WT	34.14%
GTCGGACTTGACCGTCAaTGGGGGACGA	9146	+1 a	20.12%
GTCGGACTTGACCGTCAGTTTAAACTGGGG GACGA	7363	+8 HDR	16.20%
GTCGGACTTGACCGTCA-GGGGGACGA	3721	-1	8.19%
GTCGGACTTGACCGTCAtTGGGGGACGA	1766	+1 t	3.89%
GTCGGACTTGACCGTCA--GGGGACGA	799	-2	1.76%
GTCGGACTTGACCGTC-TGGGGGACGA	770	-1	1.69%
GTCGGACTTGACCGTCAgTGGGGGACGA	400	+1 g	0.88%
GTC------------------GGACGA	346	-18	0.76%
GTCGGACTTGACCGTC--GGGGGACGA	333	-2	0.73%

P209-YW-W9-Syn100crBCL11A9b-48h-RNPaav-BC5 **48h**	Ins	Del	HDR
25804 | 68.44%	**33.01%**	**19.55%**	**15.89%**
**Typical seqs**	**Reads**	**Type**	**pct.**
GTCGGACTTGACCGTCA|TGG**GGG**ACGA	8143	WT	31.56%
GTCGGACTTGACCGTCAaTGGGGGACGA	6220	+1 a	24.10%
GTCGGACTTGACCGTCAGTTTAAACTGGGG GACGA	3868	+8 HDR	14.99%
GTCGGACTTGACCGTCA-GGGGGACGA	1687	-1	6.54%
GTCGGACTTGACCGTCAtTGGGGGACGA	1275	+1 t	4.94%
GTCGGACTTGACCGTC-TGGGGGACGA	649	-1	2.52%
GTCGGACTTGACCGTCA--GGGGACGA	352	-2	1.36%
GTCGGACTTGACCGTCA---GGGACGA	151	-3	0.59%
GTCGGACTTGACCGTCAgTGGGGGACGA	147	+1 g	0.57%
GTCGGACTTGACCGTC--GGGGGACGA	122	-2	0.47%

P212-YW-W9-Syn103crBCL11A9e-48h-RNPko

P212-YW-W9-Syn103crBCL11A9e-4h-RNPko-BC1 4h	Ins	Del	HDR
18799 | 4.65%	2.30%	2.35%	0.00%
**Typical seqs**	**Reads**	**Type**	**pct.**
ATGACGGTCAAGTCCGA|CGA**CGG**TCTC	17924	WT	95.35%
ATGACGGTCAAGTCCGAaCGACGGTCTC	334	+1 a	1.78%
ATGACGGTCAAGTC---CGACGGTCTC	242	-3	1.29%
AT----------------GACGGTCTC	103	-16	0.55%
ATGACGGTCAAGTCCGAtCGACGGTCTC	39	+1 t	0.21%
ATGACGGTCAAGTCCGA-GACGGTCTC	36	-1	0.19%
ATGACGGTCAAGTCCGAgCGACGGTCTC	24	+1 g	0.13%
ATGACGGTCAAGTCCGAcCGACGGTCTC	18	+1 c	0.10%
AT---------------CGACGGTCTC	14	-15	0.07%
ATGACG-----------CGACGGTCTC	12	-11	0.06%

P212-YW-W9-Syn103crBCL11A9e-8h-RNPko-BC2 8h	Ins	Del	HDR
13274 | 15.14%	6.51%	8.63%	0.00%
**Typical seqs**	**Reads**	**Type**	**pct.**
ATGACGGTCAAGTCCGA|CGA**CGG**TCTC	11264	WT	84.86%
ATGACGGTCAAGTCCGAaCGACGGTCTC	607	+1 a	4.57%
ATGACGGTCAAGTC---CGACGGTCTC	598	-3	4.51%
AT----------------GACGGTCTC	123	-16	0.93%
ATGACGGTCAAGTCCGA-GACGGTCTC	79	-1	0.60%
ATGACGGTCAAGTCCGAcCGACGGTCTC	78	+1 c	0.59%
ATGACGGTCAAGTCCGAtCGACGGTCTC	73	+1 t	0.55%
ATGACGGTCAAGTCCG-CGACGGTCTC	51	-1	0.38%
ATGACGGTCAAGTCCGAgCGACGGTCTC	47	+1 g	0.35%
ATGACGGTCAAGTCC--CGACGGTCTC	23	-2	0.17%

P212-YW-W9-Syn103crBCL11A9e-12h-RNPko-BC3 12h	Ins	Del	HDR
31894 | 25.68%	11.78%	13.90%	0.00%
**Typical seqs**	**Reads**	**Type**	**pct.**
ATGACGGTCAAGTCCGA|CGA**CGG**TCTC	23704	WT	74.32%
ATGACGGTCAAGTCCGAaCGACGGTCTC	2592	+1 a	8.13%
ATGACGGTCAAGTC---CGACGGTCTC	1475	-3	4.62%
AT----------------GACGGTCTC	476	-16	1.49%
ATGACGGTCAAGTCCGAcCGACGGTCTC	376	+1 c	1.18%
ATGACGGTCAAGTCCGA-GACGGTCTC	343	-1	1.08%
ATGACGGTCAAGTCCGAtCGACGGTCTC	239	+1 t	0.75%
ATGACGGTCAAGTCCG-CGACGGTCTC	195	-1	0.61%
ATGACGGTCAAGTCCGAgCGACGGTCTC	192	+1 g	0.60%
ATGACGGTC--------CGACGGTCTC	189	-8	0.59%

P212-YW-W9-Syn103crBCL11A9e-24h-RNPko-BC4 24h	Ins	Del	HDR
43960 | 49.38%	16.58%	32.79%	0.00%
**Typical seqs**	**Reads**	**Type**	**pct.**
ATGACGGTCAAGTCCGA|CGA**CGG**TCTC	22254	WT	50.62%
ATGACGGTCAAGTCCGAaCGACGGTCTC	4944	+1 a	11.25%
ATGACGGTCAAGTC---CGACGGTCTC	4333	-3	9.86%
AT----------------GACGGTCTC	3031	-16	6.89%
ATGACGGTCAAGTCCGA-GACGGTCTC	485	-1	1.10%
ATGACGGTCAAGTCCGAcCGACGGTCTC	478	+1 c	1.09%
ATGACGGTCAAGTCCGAgCGACGGTCTC	447	+1 g	1.02%
ATGACGGTC-------ACGACGGTCTC	438	-7	1.00%
ATGACGGTCAAGTCCGAtCGACGGTCTC	412	+1 t	0.94%
ATGACGGTCAAGTCCG-CGACGGTCTC	395	-1	0.90%

P212-YW-W9-Syn103crBCL11A9e-48h-RNPko-BC5 48h	Ins	Del	HDR
51802 | 64.89%	13.78%	51.11%	0.00%
**Typical seqs**	**Reads**	**Type**	**pct.**
ATGACGGTCAAGTCCGA|CGA**CGG**TCTC	18187	WT	35.11%
AT----------------GACGGTCTC	9334	-16	18.02%
ATGACGGTCAAGTC---CGACGGTCTC	6858	-3	13.24%
ATGACGGTCAAGTCCGAaCGACGGTCTC	4445	+1 a	8.58%
ATGACGGTCAAG---------------	1260	-15	2.43%
ATGACGGTCAAGTCCGAcCGACGGTCTC	762	+1 c	1.47%
ATGACGGTCAA-----------GTCTC	753	-11	1.45%
ATGACGGTCAAGTCCGA-GACGGTCTC	643	-1	1.24%
ATGACGGTCAAGTCCGAtCGACGGTCTC	468	+1 t	0.90%
ATGACGGTC-------ACGACGGTCTC	454	-7	0.88%

P213-YW-W9-Syn103crBCL11A9e-48h-RNPaav

P213-YW-W9-Syn103crBCL11A9e-4h-RNPaav-BC1 4h	Ins	Del	HDR
4623 | 2.73%	1.19%	1.23%	0.30%
**Typical seqs**	**Reads**	**Type**	**pct.**
ATGACGGTCAAGTCCGA|CGA**CGG**TCTC	4497	WT	97.27%
ATGACGGTCAAGTCCGAaCGACGGTCTC	50	+1 a	1.08%
ATGACGGTCAAGTC---CGACGGTCTC	22	-3	0.48%
ATGACGGTCAAGTCCGAGTTTAAACCGACGGTCTC	14	+8 HDR	0.30%
ATGACGGTCAAGTCCGA-GACGGTCTC	10	-1	0.22%
AT----------------GACGGTCTC	7	-16	0.15%
ATGACGGTCAAGTCCGA----------	6	-10	0.13%
ATGACGGTCAAGTCCGAtCGACGGTCTC	5	+1 t	0.11%
ATGACGGTCAAGTCCG-TGACGGTCTC	5	-1	0.11%
ATGACGGTCAAGTCCGA--ACGGTCTC	4	-2	0.09%

P213-YW-W9-Syn103crBCL11A9e-8h-RNPaav-BC2 8h	Ins	Del	HDR
5172 | 13.48%	6.11%	5.05%	2.32%
**Typical seqs**	**Reads**	**Type**	**pct.**
ATGACGGTCAAGTCCGA|CGA**CGG**TCTC	4475	WT	86.52%
ATGACGGTCAAGTCCGAaCGACGGTCTC	224	+1 a	4.33%
ATGACGGTCAAGTCCGAGTTTAAACCGACGGTCTC	120	+8 HDR	2.32%
ATGACGGTCAAGTC---CGACGGTCTC	88	-3	1.70%
AT----------------GACGGTCTC	51	-16	0.99%
ATGACGGTCAAGTCCGAcCGACGGTCTC	39	+1 c	0.75%
ATGACGGTCAAGTCCGAgCGACGGTCTC	25	+1 g	0.48%
ATGACGGTCAAGTCCG-CGACGGTCTC	23	-1	0.44%
ATGACGGTCAAGTCCGA-GACGGTCTC	21	-1	0.41%
ATGACGGTCAAG---------------	17	-15	0.33%

P213-YW-W9-Syn103crBCL11A9e-12h-RNPaav-BC3 12h	Ins	Del	HDR
7198 | 29.12%	8.21%	10.53%	10.38%
**Typical seqs**	**Reads**	**Type**	**pct.**
ATGACGGTCAAGTCCGA|CGA**CGG**TCTC	5102	WT	70.88%
ATGACGGTCAAGTCCGAGTTTAAACCGACGGTCTC	734	+8 HDR	10.20%
ATGACGGTCAAGTCCGAaCGACGGTCTC	386	+1 a	5.36%
ATGACGGTCAAGTC---CGACGGTCTC	194	-3	2.70%
ATGACGGTCAAGTCCGAcCGACGGTCTC	68	+1 c	0.94%
ATGACGGTCAAGTCCGA-GACGGTCTC	63	-1	0.88%
AT----------------GACGGTCTC	56	-16	0.78%
ATGACGGTCAAGTCCGAgCGACGGTCTC	55	+1 g	0.76%
ATGACGGTCAAGTCCG-CGACGGTCTC	54	-1	0.75%
ATGACGGTCAAGTCCGAtCGACGGTCTC	43	+1 t	0.60%

P213-YW-W9-Syn103crBCL11A9e-24h-RNPaav-BC4 24h	Ins	Del	HDR
4661 | 48.23%	12.89%	16.22%	19.12%
**Typical seqs**	**Reads**	**Type**	**pct.**
ATGACGGTCAAGTCCGA|CGA**CGG**TCTC	2413	WT	51.77%
ATGACGGTCAAGTCCGAGTTTAAACCGACGGTCTC	879	+8 HDR	18.86%
ATGACGGTCAAGTCCGAaCGACGGTCTC	401	+1 a	8.60%
ATGACGGTCAAGTC---CGACGGTCTC	132	-3	2.83%
AT----------------GACGGTCTC	85	-16	1.82%
ATGACGGTCAAGTCCGAcCGACGGTCTC	59	+1 c	1.27%
ATGACGGTCAAGTCCGA-GACGGTCTC	54	-1	1.16%
ATGACGGTCAAGTCCG-CGACGGTCTC	46	-1	0.99%
ATGACGGTCAAGTCCGAtCGACGGTCTC	44	+1 t	0.94%
ATGACGGTCAAGTCCGAgCGACGGTCTC	38	+1 g	0.82%

P213-YW-W9-Syn103crBCL11A9e-48h-RNPaav-BC5 48h	Ins	Del	HDR
2450 | 58.04%	18.04%	15.63%	24.37%
**Typical seqs**	**Reads**	**Type**	**pct.**
ATGACGGTCAAGTCCGA|CGA**CGG**TCTC	1028	WT	41.96%
ATGACGGTCAAGTCCGAGTTTAAACCGACGGTCTC	591	+8 HDR	24.12%
ATGACGGTCAAGTCCGAaCGACGGTCTC	294	+1 a	12.00%
ATGACGGTCAAGTC---CGACGGTCTC	55	-3	2.24%
AT----------------GACGGTCTC	50	-16	2.04%
ATGACGGTCAAGTCCGAcCGACGGTCTC	42	+1 c	1.71%
ATGACGGTCAAGTCCGA-GACGGTCTC	28	-1	1.14%
ATGACGGTCAAGTCCGAtCGACGGTCTC	28	+1 t	1.14%
ATGACGGTCAAGTCCG-CGACGGTCTC	27	-1	1.10%
ATGACGGTCAAGTCCGAgCGACGGTCTC	27	+1 g	1.10%

P208-YW-W9-Syn105crBCL11A9g-RNPko

P208-YW-W9-Syn105crBCL11A9g-4h-RNPko-BC1 4h	Ins	Del	HDR
26171 | 28.36%	27.73%	0.63%	0.00%
**Typical seqs**	**Reads**	**Type**	**pct.**
CAACTTACAAATACCCT|GCG**GGG**CATA	18750	WT	71.64%
CAACTTACAAATACCCTtGCGGGGCATA	6870	+1 t	26.25%
CAACTTACAAATACCCTTTGCGGGGCATA	75	+2	0.29%
CAACTTACAAATACCCTCCTGCGGGGCATA	66	+3	0.25%
CAACTTACAAATACCCTCTGCGGGGCATA	53	+2	0.20%
CAACTTACAAATA--CTGCGGGGCATA	46	-2	0.18%
CAACTTACAAATACCCCTGCGGGGCATA	43	+1 Ins	0.16%
CAACTTACAAATA-CCTGCGGGGCATA	38	-1	0.15%
CAACTTACAAATACCCTTTTGCGGGGCATA	26	+3	0.10%
CAACTTACAAATACCC---------TA	18	-9	0.07%

P208-YW-W9-Syn105crBCL11A9g-8h-RNPko-BC2 8h	Ins	Del	HDR
22973 | 54.40%	52.30%	2.10%	0.00%
**Typical seqs**	**Reads**	**Type**	**pct.**
CAACTTACAAATACCCT|GCG**GGG**CATA	10476	WT	45.60%
CAACTTACAAATACCCTtGCGGGGCATA	11353	+1 t	49.42%
CAACTTACAAATACCCTCTGCGGGGCATA	164	+2	0.71%
CAACTTACAAATACCCTTTGCGGGGCATA	138	+2	0.60%
CAACTTACAAATA-CCTGCGGGGCATA	102	-1	0.44%
CAACTTACAAATA--CTGCGGGGCATA	98	-2	0.43%
CAACTTACAAATACCCCTGCGGGGCATA	49	+1 Ins	0.21%
CAACTTACAAATA---TGCGGGGCATA	51	-3	0.22%
CAACTTACAAATACCCTATGCGGGGCATA	40	+2	0.17%
CAACTTACAA--------------ATA	34	-14	0.15%

P208-YW-W9-Syn105crBCL11A9g-12h-RNPko-BC3 12h	Ins	Del	HDR
41879 | 65.11%	61.88%	3.23%	0.00%
**Typical seqs**	**Reads**	**Type**	**pct.**
CAACTTACAAATACCCT|GCG**GGG**CATA	14610	WT	34.89%
CAACTTACAAATACCCTtGCGGGGCATA	24227	+1 t	57.85%
CAACTTACAAATACCCTCTGCGGGGCATA	347	+2	0.83%
CAACTTACAAATACCCTTTGCGGGGCATA	287	+2	0.69%
CAACTTACAAATA-CCTGCGGGGCATA	234	-1	0.56%
CAACTTACAAATA--CTGCGGGGCATA	150	-2	0.36%
CAACTTACAAATACCCTCCTGCGGGGCATA	129	+3	0.31%
CAACTTACAAATACCC-GCGGGGCATA	94	-1	0.22%
CAACTTACAAATACCCCTGCGGGGCATA	92	+1 Ins	0.22%
CAACTTACAAATACCCTATGCGGGGCATA	92	+2	0.22%

P208-YW-W9-Syn105crBCL11A9g-24h-RNPko-BC4 24h	Ins	Del	HDR
38594 | 81.29%	75.90%	5.39%	0.00%
**Typical seqs**	**Reads**	**Type**	**pct.**
CAACTTACAAATACCCT|GCG**GGG**CATA	7222	WT	18.71%
CAACTTACAAATACCCTtGCGGGGCATA	27317	+1 t	70.78%
CAACTTACAAATACCCTTTGCGGGGCATA	344	+2	0.89%
CAACTTACAAATACCCTCTGCGGGGCATA	340	+2	0.88%
CAACTTACAAATA-CCTGCGGGGCATA	206	-1	0.53%
CAACTTACAAATA--CTGCGGGGCATA	190	-2	0.49%
CAACTTACAAATACCCTCCTGCGGGGCATA	162	+3	0.42%
CAACTTACAAATACCCTATGCGGGGCATA	138	+2	0.36%
CAACTTACAA--------------ATA	123	-14	0.32%
CAACTTACAAATA---TGCGGGGCATA	119	-3	0.31%

P208-YW-W9-Syn105crBCL11A9g-48h-RNPko-BC5 48h	Ins	Del	HDR
52696 | 86.48%	78.01%	8.47%	0.00%
**Typical seqs**	**Reads**	**Type**	**pct.**
CAACTTACAAATACCCT|GCG**GGG**CATA	7124	WT	13.52%
CAACTTACAAATACCCTtGCGGGGCATA	38365	+1 t	72.80%
CAACTTACAAATACCCTTTGCGGGGCATA	736	+2	1.40%
CAACTTACAAATA-CCTGCGGGGCATA	716	-1	1.36%
CAACTTACAAATACCCTCTGCGGGGCATA	373	+2	0.71%
CAACTTACAA--------------ATA	268	-14	0.51%
CAACTTACAAATA---TGCGGGGCATA	185	-3	0.35%
CAACTTACAAATAC---------CATA	183	-9	0.35%
CAACTTACAAATACCCTCCTGCGGGGCATA	150	+3	0.28%
CAACTTACAAATACCCCTGCGGGGCATA	147	+1 Ins	0.28%

P210-YW-W9-Syn84crBCL11A4c-48h-ko-BC5

P210-YW-W9-Syn84crBCL11A4c-4h-ko-BC1 4h	Ins	Del	HDR
3123 | 32.34%	0.35%	31.99%	0.00%
**Typical seqs**	**Reads**	**Type**	**pct.**
GTAGGCGACCAACAGGG|GGG**GGG**GGGG	2113	WT	67.66%
GTAGGCGACCAACAT--GGTGGGGGTG	339	-2	10.85%
GTAGGCGACCAACA-GGGGGGGGGGG	83	-1	2.66%
GTAGGCGACCAAC----GGTGGGGGTG	82	-4	2.63%
GTAGGCGACCA----------------	81	-20	2.59%
GTAGGCGACCAACAT--------GGTG	66	-8	2.11%
GTAGGCGACCAACA-GGGGGGGGGGTG	60	-1	1.92%
GTAGGCGACCAACA-----TGGGGGTG	45	-5	1.44%
GTAGGCGACCAACATGGGG-GGGGGTG	36	-1	1.15%
GTAGGCGACCAACA-GGGGTGGGGGTG	33	-1	1.06%

P210-YW-W9-Syn84crBCL11A4c-8h-ko-BC2 8h	Ins	Del	HDR
3768 | 42.70%	0.77%	41.93%	0.00%
**Typical seqs**	**Reads**	**Type**	**pct.**
GTAGGCGACCAACAGGG|GGG**GGG**GGGG	2159	WT	57.30%
GTAGGCGACCAACAT--GGTGGGGGTG	410	-2	10.88%
GTAGGCGACCAACATG----GGGGGTG	113	-4	3.00%
GTAGGCGACCAACA-GGGGGGGGGGG	112	-1	2.97%
GTAGGCGACCAACAT-------GGGTG	90	-7	2.39%
GTAGGCGACCAACA-GGGGGGGGGGTG	88	-1	2.34%
GTAGGCGACCAAC----GGTGGGGGTG	79	-4	2.10%
GTAGGCG------------GGGGGTG	59	-13	1.57%
GTAGGCGACCAACA--GGGTGGGGGTG	49	-2	1.30%
GTAGGCGACCAACAT--GGTGGGTGTG	47	-2	1.25%

P210-YW-W9-Syn84crBCL11A4c-12h-ko-BC3 12h	Ins	Del	HDR
8266 | 76.41%	2.84%	73.57%	0.00%
**Typical seqs**	**Reads**	**Type**	**pct.**
GTAGGCGACCAACAGGG|GGG**GGG**GGGG	1950	WT	23.59%
GTAGGCGACCAACAT--GGTGGGGGTG	2380	-2	28.79%
GTAGGCGACCAACA-----TGGGGGTG	492	-5	5.95%
GTAGGCGACCAACA--GGGTGGGGGTG	301	-2	3.64%
GTAGGCGACCAACA-GGGGGGGGGGG	293	-1	3.54%
GTAGGCGACCAACA-----------TG	250	-11	3.02%
GTAGGCGACCAAC----GGTGGGGGTG	162	-4	1.96%
GTAGGCGACCAACA-GGGGGGGGGGTG	160	-1	1.94%
GTAGGCGAC----------------TG	135	-16	1.63%
GTAGGCGACCAACATGG---TCGGGTG	128	-3	1.55%

P210-YW-W9-Syn84crBCL11A4c-24h-ko-BC4 24h	Ins	Del	HDR
14944 | 76.02%	2.46%	73.57%	0.00%
**Typical seqs**	**Reads**	**Type**	**pct.**
GTAGGCGACCAACAGGG|GGG**GGG**GGGG	3583	WT	23.98%
GTAGGCGACCAACAT--GGTGGGGGTG	3697	-2	24.74%
GTAGGCGACCAACA-----TGGGGGTG	692	-5	4.63%
GTA--------------GGTGGGGGTG	455	-14	3.04%
GTAGGCGACCAACA--GGGTGGGGGTG	434	-2	2.90%
GTAGGCGACCAACA-GGGGGGGGGGG	331	-1	2.21%
GTAGGCGACCAACAT--------GGTG	318	-8	2.13%
GTAGGCGACCAACA-GGGGGGGGGGTG	271	-1	1.81%
GTAGGCGACCAACA-----------TG	247	-11	1.65%
GTAGGCGACCAAC----GGTGGGGGTG	232	-4	1.55%

P210-YW-W9-Syn84crBCL11A4c-48h-ko-BC5 48h	Ins	Del	HDR
19483 | 83.70%	3.08%	80.61%	0.00%
**Typical seqs**	**Reads**	**Type**	**pct.**
GTAGGCGACCAACAGGG|GGG**GGG**GGGG	3176	WT	16.30%
GTAGGCGACCAACAT--GGTGGGGGTG	4976	-2	25.54%
GTAGGCGACCAACA-----TGGGGGTG	1129	-5	5.79%
GTA--------------GGTGGGGGTG	653	-14	3.35%
GTAGGCGACCAACA-----------TG	563	-11	2.89%
GTAGGCGACCAACAT--------GGTG	543	-8	2.79%
GTAGGCGACCAACA--GGGTGGGGGTG	538	-2	2.76%
GTAGGCGACCAACA-GGGGGGGGGGG	426	-1	2.19%
GTAGGCGACCAACA-GGGGGGGGGGTG	333	-1	1.71%
------------------GTGGGGGTG	303	-18	1.56%

P210-YW-W9-Syn93crBCL11A7c-48h-ko-BC5

P210-YW-W9-Syn93crBCL11A7c-4h-ko-BC1 4h	Ins	Del	HDR
45159 | 10.34%	6.09%	4.25%	0.00%
**Typical seqs**	**Reads**	**Type**	**pct.**
AGCTTCGTGACGTTGGA|TGGAGGGTTT	40488	WT	89.66%
AGCTTCGTGACGTTGGAaTGGAGGGTTT	2614	+1 a	5.79%
AGCTTCGTGACGT----TGGAGGGTTT	1070	-4	2.37%
AGCTTCGTGACGTTGGA-GGAGGGTTT	391	-1	0.87%
AGCTTCGTGACGTTGG-TGGAGGGTTT	65	-1	0.14%
AGCTTC------------GGAGGGTTT	54	-12	0.12%
AGCTTCGTGACGTT-------GGGTTT	49	-7	0.11%
AGCTTCGTGACGTTGGAtTGGAGGGTTT	48	+1 t	0.11%
AGCTTCGTGACGTTGGAAATGGAGGGTTT	45	+2	0.10%
AGCTTCGTGACGTT--------GGTTT	44	-8	0.10%

P210-YW-W9-Syn93crBCL11A7c-8h-ko-BC2 8h	Ins	Del	HDR
14160 | 15.96%	10.22%	5.71%	0.03%
**Typical seqs**	**Reads**	**Type**	**pct.**
AGCTTCGTGACGTTGGA|TGGAGGGTTT	11900	WT	84.04%
AGCTTCGTGACGTTGGAaTGGAGGGTTT	1362	+1 a	9.62%
AGCTTCGTGACGT----TGGAGGGTTT	420	-4	2.97%
AGCTTCGTGACGTTGGA-GGAGGGTTT	164	-1	1.16%
AGCTTCGTGACGTTGGAtTGGAGGGTTT	32	+1 t	0.23%
AGCTTCG----------TGGAGGGTTT	29	-10	0.20%
AGCTTCGTGACG-----TGGAGGGTTT	21	-5	0.15%
AGCTTCGTGACGTTGG-TGGAGGGTTT	18	-1	0.13%
AGCT-------------TGGAGGGTTT	16	-13	0.11%
AGCTTCGTG--------TGGAGGGTTT	16	-8	0.11%

P210-YW-W9-Syn93crBCL11A7c-12h-ko-BC3 12h	Ins	Del	HDR
81716 | 20.57%	10.09%	10.49%	0.00%
**Typical seqs**	**Reads**	**Type**	**pct.**
AGCTTCGTGACGTTGGA|TGGAGGGTTT	64903	WT	79.43%
AGCTTCGTGACGTTGGAaTGGAGGGTTT	7616	+1 a	9.32%
AGCTTCGTGACGT----TGGAGGGTTT	4477	-4	5.48%
AGCTTCGTGACGTTGGA-GGAGGGTTT	1896	-1	2.32%
AGCTTCGTGACGTTG--TGGAGGGTTT	240	-2	0.29%
AGCTTCGTGACGTTGGAtTGGAGGGTTT	144	+1 t	0.18%
AGCTTCGTGACGTTGG-TGGAGGGTTT	141	-1	0.17%
AGCTTCG----------TGGAGGGTTT	133	-10	0.16%
AGCTTCGTGACGTTGGA--GAGGGTTT	110	-2	0.13%
AGCTTCGTGACGTT--ATGGAGGGTTT	95	-2	0.12%

P210-YW-W9-Syn93crBCL11A7c-24h-ko-BC4 24h	Ins	Del	HDR
76483 | 33.98%	12.11%	21.86%	0.01%
**Typical seqs**	**Reads**	**Type**	**pct.**
AGCTTCGTGACGTTGGA|TGGAGGGTTT	50496	WT	66.02%
AGCTTCGTGACGT----TGGAGGGTTT	10645	-4	13.92%
AGCTTCGTGACGTTGGAaTGGAGGGTTT	8119	+1 a	10.62%
AGCTTCGTGACGTTGGA-GGAGGGTTT	1839	-1	2.40%
AGCTTCGTGACGTT--------GGTTT	349	-8	0.46%
AGCTTCGTGACGTT-------GGGTTT	334	-7	0.44%
AGCTTCGTGACGTTGGAtTGGAGGGTTT	237	+1 t	0.31%
AGCTTCG----------TGGAGGGTTT	218	-10	0.29%
AGCTTCGTGACGTTG--TGGAGGGTTT	214	-2	0.28%
AGCTTCGTGAC------------GTTT	195	-12	0.25%

P210-YW-W9-Syn93crBCL11A7c-48h-ko-BC5 48h	Ins	Del	HDR
57205 | 39.07%	11.55%	27.51%	0.00%
**Typical seqs**	**Reads**	**Type**	**pct.**
AGCTTCGTGACGTTGGA|TGGAGGGTTT	34857	WT	60.93%
AGCTTCGTGACGT----TGGAGGGTTT	9902	-4	17.31%
AGCTTCGTGACGTTGGAaTGGAGGGTTT	5743	+1 a	10.04%
AGCTTCGTGACGTTGGA-GGAGGGTTT	1383	-1	2.42%
AGCTTCGTGACGTT-------GGGTTT	581	-7	1.02%
AGCTTCGTGACGTTGGAtTGGAGGGTTT	374	+1 t	0.65%
AGCTTCGTGAC------------GTTT	354	-12	0.62%
AGCTTCG----------TGGAGGGTTT	304	-10	0.53%
AGCTTCGTGACGTT--------GGTTT	278	-8	0.49%
AGCTTCGTGACGTTGG-TGGAGGGTTT	130	-1	0.23%

P211-YW-W9-Syn93crBCL11A7c-48h-aav-BC5

P211-YW-W9-Syn93crBCL11A7c-4h-aav-BC1 4h	Ins	Del	HDR
63152 | 11.70%	**6.44%**	**3.03%**	**2.22%**
**Typical seqs**	**Reads**	**Type**	**pct.**
AGCTTCGTGACGTTGGATGG**AGG**GTTT	55766	WT	88.30%
AGCTTCGTGACGTTGGAaTGGAGGGTTT	3744	+1 a	5.93%
AGCTTCGTGACGTTGGATGTTTAAACGGAGGGTTT	1400	+8 HDR	2.22%
AGCTTCGTGACGTTGGA-GGAGGGTTT	986	-1	1.56%
AGCTTCGTGACGT----TGGAGGGTTT	521	-4	0.82%
AGCTTCGTGACGTTGGAtTGGAGGGTTT	92	+1 t	0.15%
AGCTTCGTGACGTTGG-TGGAGGGTTT	89	-1	0.14%
AGCTTCGTGACGTTG--TGGAGGGTTT	79	-2	0.13%
AGCTTCGTGACGTTGGAgTGGAGGGTTT	51	+1 g	0.08%
AGCTTCGTGACGTTGGAcTGGAGGGTTT	43	+1 c	0.07%

P211-YW-W9-Syn93crBCL11A7c-8h-aav-BC2 8h	Ins	Del	HDR
44238 | 22.46%	**10.50%**	**6.13%**	**5.83%**
**Typical seqs**	**Reads**	**Type**	**pct.**
AGCTTCGTGACGTTGGATGG**AGG**GTTT	34303	WT	77.54%
AGCTTCGTGACGTTGGAaTGGAGGGTTT	4304	+1 a	9.73%
AGCTTCGTGACGTTGGATGTTTAAACGGAGGGTTT	2552	+8 HDR	5.77%
AGCTTCGTGACGTTGGA-GGAGGGTTT	1241	-1	2.81%
AGCTTCGTGACGT----TGGAGGGTTT	610	-4	1.38%
AGCTTCGTGACG-----TGGAGGGTTT	130	-5	0.29%
AGCTTCGTGACGTTGGAtTGGAGGGTTT	98	+1 t	0.22%
AGCTTCGT-------GATGGAGGGTTT	91	-7	0.21%
AGCTTCGTGACGTTGGA--GAGGGTTT	78	-2	0.18%
AGCTTCGTGACGTTGG-TGGAGGGTTT	59	-1	0.13%

P211-YW-W9-Syn93crBCL11A7c-12h-aav-BC3 12h	Ins	Del	HDR
86284 | 31.18%	**12.94%**	**8.63%**	**9.61%**
**Typical seqs**	**Reads**	**Type**	**pct.**
AGCTTCGTGACGTTGGATGG**AGG**GTTT	59379	WT	68.82%
AGCTTCGTGACGTTGGAaTGGAGGGTTT	10044	+1 a	11.64%
AGCTTCGTGACGTTGGATGTTTAAACGGAGGGTTT	8197	+8 HDR	9.50%
AGCTTCGTGACGTTGGA-GGAGGGTTT	3020	-1	3.50%
AGCTTCGTGACGT----TGGAGGGTTT	1819	-4	2.11%
AGCTTCGTGACGTTGGAtTGGAGGGTTT	388	+1 t	0.45%
AGCTTCGTGACGTTGGA--GAGGGTTT	332	-2	0.38%
AGCTTCGTGACGTTGG-TGGAGGGTTT	288	-1	0.33%
AGCTTCGTGACGTTG--TGGAGGGTTT	251	-2	0.29%
AGCTTCGTGACG-----TGGAGGGTTT	235	-5	0.27%

P211-YW-W9-Syn93crBCL11A7c-24h-aav-BC4 24h	Ins	Del	HDR
42705 | 43.42%	**14.30%**	**13.88%**	**15.24%**
**Typical seqs**	**Reads**	**Type**	**pct.**
AGCTTCGTGACGTTGGATGG**AGG**GTTT	24163	WT	56.58%
AGCTTCGTGACGTTGGATGTTTAAACGGAGGGTTT	6419	+8 HDR	15.03%
AGCTTCGTGACGTTGGAaTGGAGGGTTT	5308	+1 a	12.43%
AGCTTCGTGACGT----TGGAGGGTTT	1922	-4	4.50%
AGCTTCGTGACGTTGGA-GGAGGGTTT	1796	-1	4.21%
AGCTTCGTGACGTTGGAAATGGAGGGTTT	286	+2	0.67%
AGCTTCGTGACGTTGGA--GAGGGTTT	205	-2	0.48%
AGCTTCGTG--------TGGAGGGTTT	204	-8	0.48%
AGCTTCG----------TGGAGGGTTT	181	-10	0.42%
AGCTTCGTGACGTTG--TGGAGGGTTT	144	-2	0.34%

P211-YW-W9-Syn93crBCL11A7c-48h-aav-BC5 48h	Ins	Del	HDR
42932 | 48.37%	**18.03%**	**13.93%**	**16.42%**
**Typical seqs**	**Reads**	**Type**	**pct.**
AGCTTCGTGACGTTGGATGG**AGG**GTTT	22165	WT	51.63%
AGCTTCGTGACGTTGGATGTTTAAACGGAGGGTTT	6929	+8 HDR	16.14%
AGCTTCGTGACGTTGGAaTGGAGGGTTT	6536	+1 a	15.22%
AGCTTCGTGACGT----TGGAGGGTTT	1963	-4	4.57%
AGCTTCGTGACGTTGGA-GGAGGGTTT	1803	-1	4.20%
AGCTTCGTGACGTTGGAtTGGAGGGTTT	370	+1 t	0.86%
AGCTTCGTGACGTTG--TGGAGGGTTT	281	-2	0.65%
AGCTTCGTGACGTTGG-TGGAGGGTTT	200	-1	0.47%
AGCTTCG----------TGGAGGGTTT	169	-10	0.39%
AGCTTCGTGACGTTGGAGATGGAGGGTTT	148	+2	0.34%

P210-YW-W9-Syn96crBCL11A8b-48h-ko-BC5

P210-YW-W9-Syn96crBCL11A8b-4h-ko-BC1 4h	Ins	Del	HDR
29024 | 3.07%	**0.92%**	**2.15%**	**0.00%**
**Typical seqs**	**Reads**	**Type**	**pct.**
GCGGGCCACCCACATTC|ATT**GGG**GGAA	28133	WT	96.93%
GCGGGCCACCCA----CATTGGGGGAA	285	-4	0.98%
GCGGGCCACCCACATTCcATTGGGGGAA	143	+1 c	0.49%
GCGGGCCACCCACATT-ATTGGGGGAA	114	-1	0.39%
GCGGGCCACCCACATTC-TTGGGGGAA	60	-1	0.21%
GCGGGCCACCCACATTCaATTGGGGGAA	51	+1 a	0.18%
GCGGGCCACCCACAT--ATTGGGGGAA	41	-2	0.14%
GCGGGCCACCCACA---ATTGGGGGAA	26	-3	0.09%
GCGGGCCACCCACATTC---GGGGGAA	21	-3	0.07%
GCGGGCCA---------ATTGGGGGAA	21	-9	0.07%

P210-YW-W9-Syn96crBCL11A8b-8h-ko-BC2 8h	Ins	Del	HDR
16359 | 4.91%	**1.03%**	**3.88%**	**0.00%**
**Typical seqs**	**Reads**	**Type**	**pct.**
GCGGGCCACCCACATTC|ATT**GGG**GGAA	15556	WT	95.09%
GCGGGCCACCCA----CATTGGGGGAA	154	-4	0.94%
GCGGGCCACCCACATT-ATTGGGGGAA	128	-1	0.78%
GCGGGCCACCCACATTC-TTGGGGGAA	79	-1	0.48%
GCGGGCCA--------CATTGGGGGAA	43	-8	0.26%
GCGGGCCACCCACATTCaATTGGGGGAA	43	+1 a	0.26%
GCGGGCCACCCACATTC---GGGGGAA	32	-3	0.20%
GCGGGCCACCCACATTCcATTGGGGGAA	31	+1 c	0.19%
GCGGGCCACCCACAT--ATTGGGGGAA	28	-2	0.17%
GCGGGCCACCCACATTCCCACATTGGGGGA A	25	+4	0.15%

P210-YW-W9-Syn96crBCL11A8b-12h-ko-BC3 12h	Ins	Del	HDR
40362 | 7.08%	**1.17%**	**5.90%**	**0.00%**
**Typical seqs**	**Reads**	**Type**	**pct.**
GCGGGCCACCCACATTC|ATT**GGG**GGAA	37505	WT	92.92%
GCGGGCCACCCA----CATTGGGGGAA	1164	-4	2.88%
GCGGGCCACCCACATT-ATTGGGGGAA	211	-1	0.52%
GCGGGCCACCCACATTCcATTGGGGGAA	172	+1 c	0.43%
GCGGGCCACCCACATTC-TTGGGGGAA	122	-1	0.30%
GCGGGCCACCCACATTCaATTGGGGGAA	106	+1 a	0.26%
GCGGGCCACCCACAT--ATTGGGGGAA	68	-2	0.17%
GCGGGCCACCCACATTC----------	80	-11	0.20%
GCGGG----------------------	46	-26	0.11%
GCGGGCCACC-----ACATTGGGGGAA	45	-5	0.11%

P210-YW-W9-Syn96crBCL11A8b-24h-ko-BC4 24h	Ins	Del	HDR
42302 | 19.22%	**1.95%**	**17.27%**	**0.00%**
**Typical seqs**	**Reads**	**Type**	**pct.**
GCGGGCCACCCACATTC|ATT**GGG**GGAA	34170	WT	80.78%
GCGGGCCACCCA----CATTGGGGGAA	3764	-4	8.90%
GCGGGCCACCCACATT-ATTGGGGGAA	486	-1	1.15%
GCGGGCCACCCACATTC-TTGGGGGAA	322	-1	0.76%
GCGGGCCACC-----ACATTGGGGGAA	201	-5	0.48%
GCGGGCCACCCACATTCcATTGGGGGAA	195	+1 c	0.46%
GCGGGCCACCCACATTCaATTGGGGGAA	168	+1 a	0.40%
GCGGGCCACCCACAT--ATTGGGGGAA	141	-2	0.33%
GCGGGCCACCCAC--------------	170	-20	0.40%
GCGGGCCACCCACA---ATTGGGGGAA	136	-3	0.32%

P210-YW-W9-Syn96crBCL11A8b-48h-ko-BC5 48h	Ins	Del	HDR
33402 | 26.11%	**3.04%**	**23.08%**	**0.00%**
**Typical seqs**	**Reads**	**Type**	**pct.**
GCGGGCCACCCACATTC|ATT**GGG**GGAA	24680	WT	73.89%
GCGGGCCACCCA----CATTGGGGGAA	3994	-4	11.96%
GCGGGCCACCCACATT-ATTGGGGGAA	311	-1	0.93%
GCGGGCCACCC----------------	272	-19	0.81%
GCGGGCCACCCACATTC-TTGGGGGAA	208	-1	0.62%
GCGGGCCACCCACATTCcATTGGGGGAA	199	+1 c	0.60%
GCGGGCCACCCACATTCaATTGGGGGAA	172	+1 a	0.51%
GCGGGCCACC-----ACATTGGGGGAA	117	-5	0.35%
GCGGGCCA--------CATTGGGGGAA	121	-8	0.36%
GCGGGCC--------------------	194	-23	0.58%

P210-YW-W9-Syn99crBCL11A9a-48h-ko-BC5

P210-YW-W9-Syn99crBCL11A9a-4h-ko-BC1 **4h**	Ins	Del	HDR
33534 | 19.50%	**18.11%**	**1.39%**	**0.00%**
**Typical seqs**	**Reads**	**Type**	**pct.**
GCGCAAGCTCCCGTTCT|CCG**AGG**AGTG	26994	WT	80.50%
GCGCAAGCTCCCGTTCTtCCGAGGAGTG	2945	+1 t	8.78%
GCGCAAACTCCCGTTCTTCCGAGGAGTG	2897	+1 Ins	8.64%
GCGCAAACTC-------CCGAGGAGTG	114	-7	0.34%
GCGCAAACTCCCGT--TCCGAGGAGTG	73	-2	0.22%
GCGCAAGCTC-------CCGAGGAGTG	62	-7	0.18%
GCGCAAGCTCCCGTTC-CCGAGGAGTG	50	-1	0.15%
GCGCAAACTCCCGTTCTTTCCGAGGAGTG	30	+2	0.09%
GCGCAAGCTCCCGTTCTTTCCGAGGAGTG	24	+2	0.07%
GCGCAAACTCCCG---TCCGAGGAGTG	24	-3	0.07%

P210-YW-W9-Syn99crBCL11A9a-8h-ko-BC2 **8h**	Ins	Del	HDR
17023 | 25.65%	**23.52%**	**2.13%**	**0.00%**
**Typical seqs**	**Reads**	**Type**	**pct.**
GCGCAAGCTCCCGTTCT|CCG**AGG**AGTG	12657	WT	74.35%
GCGCAAGCTCCCGTTCTtCCGAGGAGTG	2049	+1 t	12.04%
GCGCAAACTCCCGTTCTTCCGAGGAGTG	1783	+1 Ins	10.47%
GCGCAAACTC-------CCGAGGAGTG	107	-7	0.63%
GCGCAAACTCCCGTTCTTTCCGAGGAGTG	57	+2	0.33%
GCGCAAGCTC-------CCGAGGAGTG	36	-7	0.21%
GCGCAAACTCCCG---TCCGAGGAGTG	31	-3	0.18%
GCGCAAGCTCCCGT--TCCGAGGAGTG	22	-2	0.13%
GCGCAAA--------CTCCGAGGAGTG	17	-8	0.10%
GCGCAAACTCCCGT--TCCGAGGAGTG	14	-2	0.08%

P210-YW-W9-Syn99crBCL11A9a-12h-ko-BC3 **12h**	Ins	Del	HDR
52560 | 26.41%	**22.97%**	**3.44%**	**0.00%**
**Typical seqs**	**Reads**	**Type**	**pct.**
GCGCAAGCTCCCGTTCT|CCG**AGG**AGTG	38677	WT	73.59%
GCGCAAACTCCCGTTCTTCCGAGGAGTG	5599	+1 Ins	10.65%
GCGCAAGCTCCCGTTCTtCCGAGGAGTG	5618	+1 t	10.69%
GCGCAAACTC-------CCGAGGAGTG	540	-7	1.03%
GCGCAAGCTC-------CCGAGGAGTG	210	-7	0.40%
GCGCAAACTCCCGTTCTTTCCGAGGAGTG	183	+2	0.35%
GCGCAAGCTCCCGTTCTTTCCGAGGAGTG	114	+2	0.22%
GCGCAAACTCCCGT--TCCGAGGAGTG	105	-2	0.20%
GCGCAAACTCCCGTTCT-CGAGGAGTG	69	-1	0.13%
GCGCAAACTCCCGTTCTGTCCGAGGAGTG	43	+2	0.08%

P210-YW-W9-Syn99crBCL11A9a-24h-ko-BC4 **24h**	Ins	Del	HDR
46760 | 47.37%	**35.48%**	**11.89%**	**0.00%**
**Typical seqs**	**Reads**	**Type**	**pct.**
GCGCAAACTCCCGTTCT|CCG**AGG**AGTG	24608	WT	52.63%
GCGCAAGCTCCCGTTCTTCCGAGGAGTG	8293	+1 Ins	17.74%
GCGCAAACTCCCGTTCTtCCGAGGAGTG	7023	+1 t	15.02%
GCGCAAACTC-------CCGAGGAGTG	1678	-7	3.59%
GCGCAAGCTC-------CCGAGGAGTG	1011	-7	2.16%
GCGCAAACTCCCGTTCTTTCCGAGGAGTG	332	+2	0.71%
GCGCAAACTCCCGT--TCCGAGGAGTG	249	-2	0.53%
GCGCAA--------------------G	199	-20	0.43%
GCGCAAGCTCCCGT--TCCGAGGAGTG	167	-2	0.36%
GCGCAAGCTCCCGTTCTTTCCGAGGAGTG	139	+2	0.30%

P210-YW-W9-Syn99crBCL11A9a-48h-ko-BC5 **48h**	Ins	Del	HDR
38117 | 49.95%	**34.83%**	**15.13%**	**0.00%**
**Typical seqs**	**Reads**	**Type**	**pct.**
GCGCAAGCTCCCGTTCT|CCG**AGG**AGTG	19076	WT	50.05%
GCGCAAGCTCCCGTTCTtCCGAGGAGTG	6385	+1 t	16.75%
GCGCAAACTCCCGTTCTTCCGAGGAGTG	5916	+1 Ins	15.52%
GCGCAAACTC-------CCGAGGAGTG	2027	-7	5.32%
GCGCAAGCTC-------CCGAGGAGTG	1101	-7	2.89%
GCGCAAA--------CTCCGAGGAGTG	181	-8	0.47%
GCGCAAACTCCCGT--TCCGAGGAGTG	165	-2	0.43%
GCGCAAACTCCCGTTCTTTCCGAGGAGTG	143	+2	0.38%
GCGCAAA--------------------	135	-20	0.35%
GCGCAA--------GCTCCGAGGAGTG	99	-8	0.26%

P210-YW-W9-Syn100crBCL11A9b-48h-ko-BC5

P210-YW-W9-Syn100crBCL11A9b-4h-ko-BC1 4h	Ins	Del	HDR
28513 | 7.67%	6.01%	1.66%	0.00%
**Typical seqs**	**Reads**	**Type**	**pct.**
GTCGGACTTGACCGTCA|TGG**GGG**ACGA	26325	WT	92.33%
GTCGGACTTGACCGTCAaTGGGGGACGA	1430	+1 a	5.02%
GTCGGACTTGACCGTCA-GGGGGACGA	216	-1	0.76%
GTCGGACTTGACCGTCAtTGGGGGACGA	192	+1 t	0.67%
GTCGGACTTGACCGTC-TGGGGGACGA	113	-1	0.40%
GTCGGACTTGACCGTCAgTGGGGGACGA	51	+1 g	0.18%
GTCGGACTTGACCGTCA--GGGGACGA	27	-2	0.09%
GTCGGACTTGACCG---TGGGGGACGA	25	-3	0.09%
GTCGGACTTGACCGTCA----GGACGA	20	-4	0.07%
GTCGGACTTGAC-------GGGGACGA	17	-7	0.06%

P210-YW-W9-Syn100crBCL11A9b-8h-ko-BC2 8h	Ins	Del	HDR
15495 | 12.62%	8.64%	3.98%	0.00%
**Typical seqs**	**Reads**	**Type**	**pct.**
GTCGGACTTGACCGTCA|TGG**GGG**ACGA	13539	WT	87.38%
GTCGGACTTGACCGTCAaTGGGGGACGA	982	+1 a	6.34%
GTCGGACTTGACCGTCAtTGGGGGACGA	265	+1 t	1.71%
GTCGGACTTGACCGTCA-GGGGGACGA	246	-1	1.59%
GTCGGACTTGACCGTC-TGGGGGACGA	46	-1	0.30%
GTCGGACTTGACCGTCA--GGGGACGA	37	-2	0.24%
GTCGGACTTGACCGTCAgTGGGGGACGA	27	+1 g	0.17%
GT----------------------CGA	20	-22	0.13%
GTCGGACTTGACCGTCA---GGGACGA	19	-3	0.12%
GTCGGACTTGACCGTCA----GGACGA	17	-4	0.11%

P210-YW-W9-Syn100crBCL11A9b-12h-ko-BC3 12h	Ins	Del	HDR
34295 | 11.98%	7.75%	4.23%	0.00%
**Typical seqs**	**Reads**	**Type**	**pct.**
GTCGGACTTGACCGTCA|TGG**GGG**ACGA	30188	WT	88.02%
GTCGGACTTGACCGTCAaTGGGGGACGA	2028	+1 a	5.91%
GTCGGACTTGACCGTCAtTGGGGGACGA	348	+1 t	1.01%
GTCGGACTTGACCGTCA-GGGGGACGA	335	-1	0.98%
GTCGGACTTGACCGTC-TGGGGGACGA	174	-1	0.51%
GTCGGACT---------TGGGGGACGA	83	-9	0.24%
GTCGGACTTGACCGTCA--GGGGACGA	74	-2	0.22%
GTCGGACTTGACCGTCAgTGGGGGACGA	58	+1 g	0.17%
GTCGGACTTGACCGT--TGGGGGACGA	56	-2	0.16%
GTC---------------GGGGGACGA	48	-15	0.14%

P210-YW-W9-Syn100crBCL11A9b-24h-ko-BC4 24h	Ins	Del	HDR
41087 | 24.94%	13.47%	11.47%	0.00%
**Typical seqs**	**Reads**	**Type**	**pct.**
GTCGGACTTGACCGTCA|TGG**GGG**ACGA	30840	WT	75.06%
GTCGGACTTGACCGTCAaTGGGGGACGA	3900	+1 a	9.49%
GTCGGACTTGACCGTCAtTGGGGGACGA	974	+1 t	2.37%
GTCGGACTTGACCGTCA-GGGGGACGA	858	-1	2.09%
GTCGGACTT-------------GACGA	369	-13	0.90%
GTC------------------GGACGA	276	-18	0.67%
GTC---------------GGGGGACGA	258	-15	0.63%
GTCGGACTTGACCGTC-TGGGGGACGA	242	-1	0.59%
GTCGGACTTGACC---------GACGA	158	-9	0.38%
GTCGGACTTGACCG---TGGGGGACGA	147	-3	0.36%

P210-YW-W9-Syn100crBCL11A9b-48h-ko-BC5 48h	Ins	Del	HDR
26445 | 26.70%	12.43%	14.27%	0.00%
**Typical seqs**	**Reads**	**Type**	**pct.**
GTCGGACTTGACCGTCA|TGG**GGG**ACGA	19384	WT	73.30%
GTCGGACTTGACCGTCAaTGGGGGACGA	2342	+1 a	8.86%
GTCGGACTT-------------GACGA	447	-13	1.69%
GTCGGACTTGACCGTCAtTGGGGGACGA	395	+1 t	1.49%
GTC------------------GGACGA	304	-18	1.15%
GTCGGACTTGACCGTCA-GGGGGACGA	286	-1	1.08%
GTCGGACTTGACCGTC-TGGGGGACGA	231	-1	0.87%
GTCGGACT---------TGGGGGACGA	170	-9	0.64%
GTCGGACTTGAC------------CGA	137	-12	0.52%
GT----------------------CGA	136	-22	0.51%

P211-YW-W9-Syn100crBCL11A9b-48h-aav-BC5

P211-YW-W9-Syn100crBCL11A9b-4h-aav-BC1 4h	Ins	Del	HDR
37907 | 8.21%	**5.39%**	**2.28%**	**0.54%**
**Typical seqs**	**Reads**	**Type**	**pct.**
GTCGGACTTGACCGTCA|TGG**GGG**ACGA	34794	WT	91.79%
GTCGGACTTGACCGTCAaTGGGGGACGA	1685	+1 a	4.45%
GTCGGACTTGACCGTCA-GGGGGACGA	412	-1	1.09%
GTCGGACTTGACCGTCAGTTTAAACTGGGGGACGA	206	+8 HDR	0.54%
GTCGGACTTGACCGTCAtTGGGGGACGA	184	+1 t	0.49%
GTCGGACTTGACCGTC-TGGGGGACGA	153	-1	0.40%
GTCGGACTTGACCGTCA--GGGGACGA	71	-2	0.19%
GTCGGACTTGACCGTCAgTGGGGGACGA	70	+1 g	0.18%
GTCGGACTTGACCGTC--GGGGGACGA	47	-2	0.12%
GTCGGACTTGACCGT--TGGGGGACGA	29	-2	0.08%

P211-YW-W9-Syn100crBCL11A9b-8h-aav-BC2 8h	Ins	Del	HDR
28384 | 18.08%	**11.09%**	**4.73%**	**2.26%**
**Typical seqs**	**Reads**	**Type**	**pct.**
GTCGGACTTGACCGTCA|TGG**GGG**ACGA	23252	WT	81.92%
GTCGGACTTGACCGTCAaTGGGGGACGA	2596	+1 a	9.15%
GTCGGACTTGACCGTCA-GGGGGACGA	701	-1	2.47%
GTCGGACTTGACCGTCAGTTTAAACTGGGGGACGA	615	+8 HDR	2.17%
GTCGGACTTGACCGTCAtTGGGGGACGA	248	+1 t	0.87%
GTCGGACTTGACCGTC-TGGGGGACGA	188	-1	0.66%
GTCGGACTTGACCGTC--GGGGGACGA	67	-2	0.24%
GTCGGACTTGACCGTCAgTGGGGGACGA	58	+1 g	0.20%
GTCGGACTTGACCGTCATTTGGGGGACGA	51	+2	0.18%
GTCGGACTTGACCGT-ATGGGGGACGA	48	-1	0.17%

P211-YW-W9-Syn100crBCL11A9b-12h-aav-BC3 12h	Ins	Del	HDR
51531 | 20.54%	**11.58%**	**5.91%**	**3.06%**
**Typical seqs**	**Reads**	**Type**	**pct.**
GTCGGACTTGACCGTCA|TGG**GGG**ACGA	40944	WT	79.46%
GTCGGACTTGACCGTCAaTGGGGGACGA	4701	+1 a	9.12%
GTCGGACTTGACCGTCAGTTTAAACTGGGGGACGA	1525	+8 HDR	2.96%
GTCGGACTTGACCGTCA-GGGGGACGA	1140	-1	2.21%
GTCGGACTTGACCGTCAtTGGGGGACGA	623	+1 t	1.21%
GTCGGACTTGACCGTCA--GGGGACGA	279	-2	0.54%
GTCGGACTTGACCGTC-TGGGGGACGA	175	-1	0.34%
GTCGGACTTGACCGTCAgTGGGGGACGA	169	+1 g	0.33%
GTCGGACTTGACCGTC--------CGA	159	-8	0.31%
GTCGGACTT-------------GACGA	149	-13	0.29%

P211-YW-W9-Syn100crBCL11A9b-24h-aav-BC4 24h	Ins	Del	HDR
22397 | 30.69%	**14.65%**	**8.49%**	**7.55%**
**Typical seqs**	**Reads**	**Type**	**pct.**
GTCGGACTTGACCGTCA|TGG**GGG**ACGA	15524	WT	69.31%
GTCGGACTTGACCGTCAaTGGGGGACGA	2625	+1 a	11.72%
GTCGGACTTGACCGTCAGTTTAAACTGGGGGACGA	1632	+8 HDR	7.29%
GTCGGACTTGACCGTCA-GGGGGACGA	790	-1	3.53%
GTCGGACTTGACCGTCAtTGGGGGACGA	304	+1 t	1.36%
GTCGGAC--------------------	137	-21	0.61%
GTCGGACTTGAC------------CGA	91	-12	0.41%
GTCGGACTTGACCGTCAATTGGGGGACGA	85	+2	0.38%
GTCGGACTTGACCGTC-TGGGGGACGA	72	-1	0.32%
GTCGGACTTGACCGTCAT------CGA	55	-6	0.25%

P211-YW-W9-Syn100crBCL11A9b-48h-aav-BC5 48h	Ins	Del	HDR
20446 | 40.21%	**20.18%**	**9.22%**	**10.81%**
**Typical seqs**	**Reads**	**Type**	**pct.**
GTCGGACTTGACCGTCA|TGG**GGG**ACGA	12224	WT	59.79%
GTCGGACTTGACCGTCAaTGGGGGACGA	2841	+1 a	13.90%
GTCGGACTTGACCGTCAGTTTAAACTGGGGGACGA	2058	+8 HDR	10.07%
GTCGGACTTGACCGTCA-GGGGGACGA	686	-1	3.36%
GTCGGACTTGACCGTCAtTGGGGGACGA	660	+1 t	3.23%
GTCGGACTTGACCGTC-TGGGGGACGA	179	-1	0.88%
GTCGGACTTGACCGTCAAATGGGGGACGA	138	+2	0.67%
GTCGGACTTGACCGTCAgTGGGGGACGA	135	+1 g	0.66%
GTCGGACTTGACCG---TGGGGGACGA	106	-3	0.52%
GTCGGACTTGACCGTCA--GGGGACGA	75	-2	0.37%

P214-YW-W9-Syn103crBCL11A9e-48h-ko-BC5

P214-YW-W9-Syn103crBCL11A9e-4h-ko-BC1 4h	Ins	Del	HDR
2673 | 2.51%	1.50%	1.01%	0.00%
**Typical seqs**	**Reads**	**Type**	**pct.**
ATGACGGTCAAGTCCGA|CGA**CGG**TCTC	2606	WT	97.49%
ATGACGGTCAAGTCCGAaCGACGGTCTC	25	+1 a	0.94%
ATGACGGTCAAGTC---CGACGGTCTC	12	-3	0.45%
ATGACGGTCAAGTCCGAtCGACGGTCTC	8	+1 t	0.30%
ATGACGGTCAAGTCCGACGACGACGGTCTC	7	+3	0.26%
ATGACGGTCAAGTCC-------GTCTC	4	-7	0.15%
ATGACGGTCA------ACGACGGTCTC	4	-6	0.15%
ATGACGGTC--------CGACGGTCTC	4	-8	0.15%
ATGACGGTCAAGTCCGA-GACGGTCTC	3	-1	0.11%
P214-YW-W9-Syn103crBCL11A9e-4h-ko-BC1	Ins	Del	HDR

P214-YW-W9-Syn103crBCL11A9e-8h-ko-BC2 8h	Ins	Del	HDR
2521 | 7.06%	3.41%	3.65%	0.00%
**Typical seqs**	**Reads**	**Type**	**pct.**
ATGACGGTCAAGTCCGA|CGA**CGG**TCTC	2343	WT	92.94%
ATGACGGTCAAGTCCGAaCGACGGTCTC	65	+1 a	2.58%
ATGACGGTCAAGTC---CGACGGTCTC	41	-3	1.63%
AT----------------GACGGTCTC	21	-16	0.83%
ATGACGGTCAAGTCCGACGACGACGGTCTC	8	+3	0.32%
ATGACGG----------CGACGGTCTC	7	-10	0.28%
ATGACGGTCAAGT---------------	6	-16	0.24%
ATGACGGTCAAGTCCG-CGACGGTCTC	5	-1	0.20%
ATGACGGTCAAGTCCGAcCGACGGTCTC	5	+1 c	0.20%
ATGACGGT---------CGACGGTCTC	5	-9	0.20%

P214-YW-W9-Syn103crBCL11A9e-12h-ko-BC3 12h	Ins	Del	HDR
5042 | 6.55%	2.50%	4.05%	0.00%
**Typical seqs**	**Reads**	**Type**	**pct.**
ATGACGGTCAAGTCCGA|CGA**CGG**TCTC	4712	WT	93.45%
ATGACGGTCAAGTCCGAaCGACGGTCTC	97	+1 a	1.92%
ATGACGGTCAAGTC---CGACGGTCTC	62	-3	1.23%
AT----------------GACGGTCTC	47	-16	0.93%
ATGACGGTCAAGTCCG-CGACGGTCTC	21	-1	0.42%
ATGACG---------------GGTCTC	15	-15	0.30%
ATGACGGTCAAGTCCGACGACGACGGTCTC	11	+3	0.22%
ATGACGGTCAAGTCCGAtCGACGGTCTC	10	+1 t	0.20%
ATGACGGTCA------ACGACGGTCTC	9	-6	0.18%
ATGACGGTCAAG---------------	9	-15	0.18%

P214-YW-W9-Syn103crBCL11A9e-24h-ko-BC4 24h	Ins	Del	HDR
6432 | 25.31%	4.88%	20.43%	0.00%
**Typical seqs**	**Reads**	**Type**	**pct.**
ATGACGGTCAAGTCCGA|CGA**CGG**TCTC	4804	WT	74.69%
AT----------------GACGGTCTC	532	-16	8.27%
ATGACGGTCAAGTC---CGACGGTCTC	332	-3	5.16%
ATGACGGTCAAGTCCGAaCGACGGTCTC	237	+1 a	3.68%
ATGACGGTCAAG---------------	75	-15	1.17%
ATGACGGTCAAGTCCG-CGACGGTCTC	43	-1	0.67%
AT-------------GACGACGGTCTC	32	-13	0.50%
ATGACGGTCAA-----------GTCTC	30	-11	0.47%
ATGACGGTCAAGTCCGAcCGACGGTCTC	29	+1 c	0.45%
ATGACG------------GACGGTCTC	19	-12	0.30%

P214-YW-W9-Syn103crBCL11A9e-48h-ko-BC5 48h	Ins	Del	HDR
7174 | 31.96%	4.54%	27.42%	0.00%
**Typical seqs**	**Reads**	**Type**	**pct.**
ATGACGGTCAAGTCCGA|CGA**CGG**TCTC	4881	WT	68.04%
AT----------------GACGGTCTC	871	-16	12.14%
ATGACGGTCAAGTC---CGACGGTCTC	446	-3	6.22%
ATGACGGTCAAGTCCGAaCGACGGTCTC	191	+1 a	2.66%
ATGACGGTCAA-----------GTCTC	90	-11	1.25%
ATGACGGTCAAGTCCGAcCGACGGTCTC	47	+1 c	0.66%
ATGACGGTCAAG---------------	68	-15	0.95%
ATGACGGTCAAGTCCG-CGACGGTCTC	33	-1	0.46%
----------------ACGACGGTCTC	28	-16	0.39%
ATGACGGTCAAGTCCGAtCGACGGTCTC	27	+1 t	0.38%

P219-YW-W9-Syn103crBCL11A9e-48h-aav-BC5

P219-YW-W9-Syn103crBCL11A9e-4h-aav-BC1 4h	Ins	Del	HDR
10910 | 2.44%	1.15%	1.06%	0.22%
Typical seqs	Reads	Type	pct.
ATGACGGTCAAGTCCGA|CGACGGTCTC	10644	WT	97.56%
ATGACGGTCAAGTCCGAaCGACGGTCTC	82	+1 a	0.75%
ATGACGGTCAAGTC---CGACGGTCTC	34	-3	0.31%
ATGACGGTCAAGTCCG-CGACGGTCTC	25	-1	0.23%
ATGACGGTCAAGTCCGAGTTTAAACCGACG GTCTC	24	+8 HDR	0.22%
ATGACGGTCAAGTCCGAtCGACGGTCTC	19	+1 t	0.17%
ATGACGGTCAAGTCCGA-GACGGTCTC	19	-1	0.17%
ATGACGGTCAAGTCCGAgCGACGGTCTC	15	+1 g	0.14%
ATGACGGTCAAGTCCGA----------	8	-15	0.07%
ATGACGGTCAAGTCCGAC----GTCTC	7	-4	0.06%

P219-YW-W9-Syn103crBCL11A9e-8h-aav-BC2 8h	Ins	Del	HDR
8261 | 7.57%	3.45%	2.41%	1.71%
Typical seqs	Reads	Type	pct.
ATGACGGTCAAGTCCGA|CGACGGTCTC	7636	WT	92.43%
ATGACGGTCAAGTCCGAaCGACGGTCTC	174	+1 a	2.11%
ATGACGGTCAAGTCCGAGTTTAAACCGACG GTCTC	141	+8 HDR	1.71%
ATGACGGTCAAGTC---CGACGGTCTC	67	-3	0.81%
ATGACGGTCAAGTCCGAcCGACGGTCTC	42	+1 c	0.51%
ATGACGGTCAAGTCCGAtCGACGGTCTC	26	+1 t	0.31%
ATGACGGTCAAGTCCGA-GACGGTCTC	19	-1	0.23%
ATGACGGTCAA----------GGTCTC	16	-10	0.19%
ATGACGGTCAAGTCCGAgCGACGGTCTC	13	+1 g	0.16%
ATGACGGTCAAGTCCGACACGACGGTCTC	12	+2	0.15%

P219-YW-W9-Syn103crBCL11A9e-12h-aav-BC3 12h	Ins	Del	HDR
13231 | 17.94%	5.49%	4.48%	7.97%
Typical seqs	Reads	Type	pct.
ATGACGGTCAAGTCCGA|CGACGGTCTC	10858	WT	82.06%
ATGACGGTCAAGTCCGAGTTTAAACCGACG GTCTC	1038	+8 HDR	7.85%
ATGACGGTCAAGTCCGAaCGACGGTCTC	553	+1 a	4.18%
ATGACGGTCAAGTCCGA-GACGGTCTC	150	-1	1.13%
ATGACGGT--------------------	113	-21	0.85%
ATGACGG------------------TC	101	-18	0.76%
ATGACGGTCAAGTCCGAgCGACGGTCTC	96	+1 g	0.73%
ATGACGGTCAAGTCCG-CGACGGTCTC	77	-1	0.58%
ATGACGGTCA------ACGACGGTCTC	54	-6	0.41%
ATGACGGTCAAGTC---CGACGGTCTC	52	-3	0.39%

P219-YW-W9-Syn103crBCL11A9e-24h-aav-BC4 24h	Ins	Del	HDR
8810 | 23.97%	5.86%	8.67%	9.44%
Typical seqs	Reads	Type	pct.
ATGACGGTCAAGTCCGA|CGACGGTCTC	6698	WT	76.03%
ATGACGGTCAAGTCCGAGTTTAAACCGACG GTCTC	818	+8 HDR	9.28%
ATGACGGTCAAGTCCGAaCGACGGTCTC	265	+1 a	3.01%
ATGACGGTCAAGTCCGA-GACGGTCTC	122	-1	1.38%
ATGACGGTCAAGTCCG-CGACGGTCTC	109	-1	1.24%
AT----------------GACGGTCTC	107	-16	1.21%
ATGACGGTCAAGTC---CGACGGTCTC	69	-3	0.78%
ATGACGGTCAAGTCC--CGACGGTCTC	57	-2	0.65%
ATGACGGTCAAG---------------	53	-15	0.60%
ATGACGGTCAAGTCCGAcCGACGGTCTC	45	+1 c	0.51%

P219-YW-W9-Syn103crBCL11A9e-48h-aav-BC5 48h	Ins	Del	HDR
6413 | 30.16%	8.72%	9.71%	11.73%
Typical seqs	Reads	Type	pct.
ATGACGGTCAAGTCCGA|CGACGGTCTC	4479	WT	69.84%
ATGACGGTCAAGTCCGAGTTTAAACCGACG GTCTC	746	+8 HDR	11.63%
ATGACGGTCAAGTCCGAaCGACGGTCTC	332	+1 a	5.18%
ATGACGGTCAAGTC---CGACGGTCTC	140	-3	2.18%
ATGACGGTCAAGTCCGAtCGACGGTCTC	101	+1 t	1.57%
ATGACGGTCAAGTCCGAC--CGGTCTC	71	-2	1.11%
ATGACGGTCAAGTCCGA----------	117	-12	1.82%
ATGACGGTCAAGTCCG-----------	54	-14	0.84%
ATGACGGTCAAGTCCGAcCGACGGTCTC	46	+1 c	0.72%
ATGACGGTCAAGTCCG-CGACGGTCTC	46	-1	0.72%

P210-YW-W9-Syn105crBCL11A9g-48h-ko-BC5

P210-YW-W9-Syn105crBCL11A9g-4h-ko-BC1 **4h**	Ins	Del	HDR
25548 | 34.33%	33.27%	1.06%	0.00%
**Typical seqs**	**Reads**	**Type**	**pct.**
CAACTTACAAATACCCT|GCG**GGG**CATA	16777	WT	65.67%
CAACTTACAAATACCCTtGCGGGGCATA	7930	+1 t	31.04%
CAACTTACAAATACCCTCTGCGGGGCATA	206	+2	0.81%
CAACTTACAAATACCCTTTGCGGGGCATA	123	+2	0.48%
CAACTTACAAATA-CCTGCGGGGCATA	71	-1	0.28%
CAACTTACAAATA--CTGCGGGGCATA	63	-2	0.25%
CAACTTACAAA-------------ATA	42	-18	0.16%
CAACTTACAAATACCCTGGTGGCGGGGCATA	41	+4	0.16%
CAACTTACAAATACCC-GCGGGGCATA	29	-1	0.11%
CAACTTACAAATACCCTCCTGCGGGGCATA	21	+3	0.08%

P210-YW-W9-Syn105crBCL11A9g-8h-ko-BC2 **8h**	Ins	Del	HDR
18371 | 41.02%	39.50%	1.52%	0.00%
**Typical seqs**	**Reads**	**Type**	**pct.**
CAACTTACAAATACCCT|GCG**GGG**CATA	10836	WT	58.98%
CAACTTACAAATACCCTtGCGGGGCATA	6910	+1 t	37.61%
CAACTTACAAATACCCTCTGCGGGGCATA	92	+2	0.50%
CAACTTACAAATA-CCTGCGGGGCATA	83	-1	0.45%
CAACTTACAAATACCC-GCGGGGCATA	71	-1	0.39%
CAACTTACAAATACCC---------TA	49	-9	0.27%
CAACTTACAAATACCCTTTTGCGGGGCATA	46	+3	0.25%
CAACTTACAAATAC---GCGGGGCATA	41	-3	0.22%
CAACTTACAAATA--CTGCGGGGCATA	35	-2	0.19%
CAACTTACAAATACCCCCTGCGGGGCATA	33	+2	0.18%

P210-YW-W9-Syn105crBCL11A9g-12h-ko-BC3 **12h**	Ins	Del	HDR
39060 | 31.06%	30.18%	0.88%	0.00%
**Typical seqs**	**Reads**	**Type**	**pct.**
CAACTTACAAATACCCT|GCG**GGG**CATA	26927	WT	68.94%
CAACTTACAAATACCCTtGCGGGGCATA	11086	+1 t	28.38%
CAACTTACAAATACCCTCTGCGGGGCATA	112	+2	0.29%
CAACTTACAAATACCCCTGCGGGGCATA	67	+1 Ins	0.17%
CAACTTACAAATACCCTCCTGCGGGGCATA	65	+3	0.17%
CAACTTACAAATACCCTAATGCGGGGCATA	62	+3	0.16%
CAACTTACAAATACCCTTTGCGGGGCATA	49	+2	0.13%
CAACTTACAA--------------ATA	48	-14	0.12%
CAACTTACAAATA-CCTGCGGGGCATA	46	-1	0.12%
CAACTTACAAATACC-----------A	31	-11	0.08%

P210-YW-W9-Syn105crBCL11A9g-24h-ko-BC4 **24h**	Ins	Del	HDR
36831 | 63.69%	58.24%	5.45%	0.00%
**Typical seqs**	**Reads**	**Type**	**pct.**
CAACTTACAAATACCCT|GCG**GGG**CATA	13374	WT	36.31%
CAACTTACAAATACCCTtGCGGGGCATA	19785	+1 t	53.72%
CAACTTACAAATACCCTTTGCGGGGCATA	459	+2	1.25%
CAACTTACAAATACCCTCTGCGGGGCATA	410	+2	1.11%
CAACTTACAAATA-CCTGCGGGGCATA	259	-1	0.70%
CAACTTACAA--------------ATA	205	-14	0.56%
CAACTTACAAATACC------------	170	-14	0.46%
CAACTTACAAATACCCT------CATA	146	-6	0.40%
CAACTTACAAATACC--------CATA	132	-8	0.36%
CAACTTACAAATACCCTATGCGGGGCATA	111	+2	0.30%

P210-YW-W9-Syn105crBCL11A9g-48h-ko-BC5 **48h**	Ins	Del	HDR
39322 | 63.93%	58.07%	5.86%	0.00%
**Typical seqs**	**Reads**	**Type**	**pct.**
CAACTTACAAATACCCT|GCG**GGG**CATA	14182	WT	36.07%
CAACTTACAAATACCCTtGCGGGGCATA	21447	+1 t	54.54%
CAACTTACAA--------------ATA	321	-14	0.82%
CAACTTACAAATACCCTTTGCGGGGCATA	274	+2	0.70%
CAACTTACAAATACCCTCTGCGGGGCATA	250	+2	0.64%
CAACTTACAAATA-CCTGCGGGGCATA	208	-1	0.53%
CAACTTACAAATA---TGCGGGGCATA	175	-3	0.45%
CAACTTACAAATACCCTATGCGGGGCATA	130	+2	0.33%
CAACTTACAAATAC-------------	230	-14	0.58%
CAACTTACAAATA--CTGCGGGGCATA	111	-2	0.28%

P41-P50-WT-U937-Syn32crBC1L11Gata-a1-KO-BC11

Total | INDEL	4h	Ins	Del	HDR
373570 | 3.43%		2.47%	0.96%	0.00%

Typical seqs	Reads	Type	pct.
CACGCCCCCACCCTAAT|CAGAGGCCAA	360761	WT	96.57%
CACGCCCCCACCCTAATtCAGAGGCCAA	7646	+1 t	2.05%
CACGCCCCCACCCTA-TCAGAGGCCAA	788	-1	0.21%
CACGCCCCCACCCT---CAGAGGCCAA	189	-3	0.05%
CACGCCCCCACCCTA--CAGAGGCCAA	125	-2	0.03%
CACGCCCCCACCCTAA-CAGAGGCCAA	115	-1	0.03%
CACGCCCCCACCCTA---AGAGGCCAA	111	-3	0.03%
CACGCCCCCCCCCTAATTCAGAGGCCAA	111	+1 Ins	0.03%
CACGCCCCCACCCT--TCAGAGGCCAA	87	-2	0.02%
CACGCCCCCACCCTAATtCAGAGGCCCA	91	+1 t	0.02%

Total | INDEL	8h	Ins	Del	HDR
431033 | 8.24%		5.31%	2.93%	0.00%

Typical seqs	Reads	Type	pct.
CACGCCCCCACCCTAAT|CAGAGGCCAA	395496	WT	91.76%
CACGCCCCCACCCTAATtCAGAGGCCAA	18716	+1 t	4.34%
CACGCCCCCACCCTA-TCAGAGGCCAA	1704	-1	0.40%
CACGCCCCCACCCT---CAGAGGCCAA	718	-3	0.17%
CACGCCCCCACCCTAA-CAGAGGCCAA	566	-1	0.13%
CACGCCCCC------------------	493	-18	0.11%
CACGCCCCCACCCTA---AGAGGCCAA	445	-3	0.10%
CACGCCCCCACCCTAAT-AGAGGCCAA	431	-1	0.10%
CACGCCCCCACCCT--TCAGAGGCCAA	425	-2	0.10%
CACGCCCCCACCC-----AGAGGCCAA	376	-5	0.09%

Total | INDEL	12h	Ins	Del	HDR
148694 | 14.55%		8.19%	6.35%	0.00%

Typical seqs	Reads	Type	pct.
CACGCCCCCACCCTAAT|CAGAGGCCAA	127064	WT	85.45%
CACGCCCCCACCCTAATtCAGAGGCCAA	9574	+1 t	6.44%
CACGCCCCCACCCTA-TCAGAGGCCAA	1034	-1	0.70%
CACGCCCCCACCCTA---AGAGGCCAA	492	-3	0.33%
CACGCCCCCACCC-----AGAGGCCAA	452	-5	0.30%
CACGCCCCC------------------	437	-18	0.29%
CACGCCCCCACCCTAA-CAGAGGCCAA	382	-1	0.26%
CACGCCCCCACCCT---CAGAGGCCAA	335	-3	0.23%
CACGCCCCCACCCTA—CAGAGGCCAA	326	-2	0.22%
CACGCCCC---------CAGAGGCCAA	280	-9	0.19%

Total | INDEL	24h	Ins	Del	HDR
300207 | 36.45%		14.00%	22.45%	0.00%

Typical seqs	Reads	Type	pct.
CACGCCCCCACCCTAAT|CAGAGGCCAA	190781	WT	63.55%
CACGCCCCCACCCTAATtCAGAGGCCAA	31701	+1 t	10.56%
CACGCCCCC------------------	6125	-18	2.04%
CAC-------------------GCCAA	3943	-19	1.31%
CACGCCCCCACCCTA-TCAGAGGCCAA	2706	-1	0.90%
CACGCCCC---------CAGAGGCCAA	2379	-9	0.79%
CACGCCCCCACCC-----AGAGGCCAA	2279	-5	0.76%
CACGCCCCCACCCTA---AGAGGCCAA	2194	-3	0.73%
CACGCCC----------------CCAA	2085	-16	0.69%
CACGCCCCCA-------------CCAA	1655	-13	0.55%

Total | INDEL	48h	Ins	Del	HDR
175882 | 50.11%		17.04%	33.07%	0.00%

Typical seqs	Reads	Type	pct.
CACGCCCCCACCCTAAT|CAGAGGCCAA	87746	WT	49.89%
CACGCCCCCACCCTAATtCAGAGGCCAA	22191	+1 t	12.62%
CACGCCCCC------------------	5661	-18	3.22%
CAC-------------------GCCAA	3546	-19	2.02%
CACGCCCCCACCC-----AGAGGCCAA	2598	-5	1.48%
CACGCCC----------------CCAA	2069	-16	1.18%
CACGCCCCCACCCTA-TCAGAGGCCAA	2030	-1	1.15%
CACGCCCCCACCCTA---AGAGGCCAA	1885	-3	1.07%
CACGCCCC---------CAGAGGCCAA	1797	-9	1.02%
CACGCCCCCACCCTA-----AGGCCAA	1487	-5	0.85%

P41-P50-WT-U937-Syn33crBC1L11Gata-a2-KO-BC12

Total | INDEL	4h	Ins	Del	HDR
131178 | 2.08%		0.36%	1.72%	0.00%

Typical seqs	Reads	Type	pct.
CACAGGCTCCAGGAAGG|GTT**TGG**CCTC	128449	WT	97.92%
CACAGGCTCCAGGAAG-GTTTGGCCTC	967	-1	0.74%
CACAGGCTCCAGGAAGGgGTTTGGCCTC	362	+1 g	0.28%
CACAGGCTCCAGGAAG--TTTGGCCTC	218	-2	0.17%
CACAGGCTCCAGGA---GTTTGGCCTC	138	-3	0.11%
CACAGGCTCCAGGAAGGG-TTGGCCTC	115	-1	0.09%
CACAGGCTCCAGG----GTTTGGCCTC	83	-4	0.06%
CACAGGCTCCAGGAA-AGTTTGGCCTC	62	-1	0.05%
CACAGGCTCCAGG-AGGGTTTGGCCTC	49	-1	0.04%
CACAGGCTCCAG------TTTGGCCTC	48	-6	0.04%

Total | INDEL	8h	Ins	Del	HDR
237415 | 4.72%		0.86%	3.86%	0.00%

Typical seqs	Reads	Type	pct.
CACAGGCTCCAGGAAGG|GTT**TGG**CCTC	226217	WT	95.28%
CACAGGCTCCAGGAAG-GTTTGGCCTC	3289	-1	1.39%
CACAGGCTCCAGGAAGGgGTTTGGCCTC	1445	+1 g	0.61%
CACAGGCTCCAGGAAG--TTTGGCCTC	1151	-2	0.48%
CACAGGCTCCAGGA---GTTTGGCCTC	612	-3	0.26%
CACAGGCTCCAGG----GTTTGGCCTC	351	-4	0.15%
CACAGGCTCCAGGAA-AGTTTGGCCTC	330	-1	0.14%
CACAGGCTCCAGGAA------GGCCTC	260	-6	0.11%
CACAGGCTCCAG------TTTGGCCTC	215	-6	0.09%
CACAGGCTCCAGGAAGG----AGCCTC	214	-4	0.09%

Total | INDEL	12h	Ins	Del	HDR
89435 | 8.29%		1.05%	7.25%	0.00%

Typical seqs	Reads	Type	pct.
CACAGGCTCCAGGAAGG|GTT**TGG**CCTC	82017	WT	91.71%
CACAGGCTCCAGGAAG-GTTTGGCCTC	2312	-1	2.59%
CACAGGCTCCAGGAAG--TTTGGCCTC	681	-2	0.76%
CACAGGCTCCAGGAAGGgGTTTGGCCTC	625	+1 g	0.70%
CACAGGCTCCAGGA---GTTTGGCCTC	334	-3	0.37%
CACAGGCTCCAGG----GTTTGGCCTC	251	-4	0.28%
CACAGGCTCCAGGAAGG----AGCCTC	210	-4	0.23%
CACAGGCTCCAG------TTTGGCCTC	205	-6	0.23%
CACAGGCTCCAGGAA------GGCCTC	156	-6	0.17%
CACAGGCTCCAGGAA-AGTTTGGCCTC	152	-1	0.17%

Total | INDEL	24h	Ins	Del	HDR
119044 | 19.12%		2.04%	17.08%	0.00%

Typical seqs	Reads	Type	pct.
CACAGGCTCCAGGAAGG|GTT**TGG**CCTC	96281	WT	80.88%
CACAGGCTCCAGGAAG-GTTTGGCCTC	4829	-1	4.06%
CACAGGCTCCAGGAAG--TTTGGCCTC	1576	-2	1.32%
CACAGGCTCCAGGAAGGgGTTTGGCCTC	1365	+1 g	1.15%
CACAGGCTCCAGGAAGG----AGCCTC	1228	-4	1.03%
CACAGGCTCCAGGAA------GGCCTC	990	-6	0.83%
CACA-----------------GGCCTC	923	-17	0.78%
CACAGGCTCCAGGA---GTTTGGCCTC	880	-3	0.74%
CACAGGCTCCA----------GGCCTC	611	-10	0.51%
CACAGGCTCCAG------TTTGGCCTC	558	-6	0.47%

Total | INDEL	48h	Ins	Del	HDR
97719 | 24.92%		2.49%	22.43%	0.00%

Typical seqs	Reads	Type	pct.
CACAGGCTCCAGGAAGG|GTT**TGG**CCTC	73370	WT	75.08%
CACAGGCTCCAGGAAG-GTTTGGCCTC	5134	-1	5.25%
CACAGGCTCCAGGAAG--TTTGGCCTC	1593	-2	1.63%
CACAGGCTCCAGGAAGG----AGCCTC	1466	-4	1.50%
CACAGGCTCCAGGAAGGgGTTTGGCCTC	1252	+1 g	1.28%
CACA-----------------GGCCTC	1217	-17	1.25%
CACAGGCTCCAGGAA------GGCCTC	1086	-6	1.11%
CACAGGCTCCAGGA---GTTTGGCCTC	860	-3	0.88%
CACAGGCTCCA----------GGCCTC	704	-10	0.72%
CACAGGCTCCAGG----GTTTGGCCTC	619	-4	0.63%

P41-P50-WT-U937-Syn34crBC1L11Gata-a3-KO-BC13

4h

Total	INDEL	Ins	Del	HDR
319362	1.55%	0.75%	0.80%	0.00%

Typical seqs	Reads	Type	pct.
TTTATCACAGGCTCCAGGAA**GGG**TTTG	314406	WT	98.45%
TTTATCACAGGCTCCAGgGAAGGGTTTG	1480	+1 g	0.46%
TTTATCACAGGCTCCA-GAAGGGTTTG	898	-1	0.28%
TTTATCACAGGCTCC-GGAAGGGTTTG	229	-1	0.07%
TTTATCACAGGCTCCAGaGAAGGGTTTG	181	+1 a	0.06%
TTTATCACAGGCTCCAAGGAAGGGTTTG	166	+1 Ins	0.05%
TTTATCACAGGCTCC----AGGGTTTG	145	-4	0.05%
TTTATCACAGGCT-CAGGAAGGGTTTG	143	-1	0.04%
TTTATCACAGGCTCCAGG-AGGGTTTG	91	-1	0.03%
TTTATCACAGGCTCCAGAGGAAGGGTTTG	87	+2	0.03%

8h

Total	INDEL	Ins	Del	HDR
586734	3.51%	1.53%	1.98%	0.00%

Typical seqs	Reads	Type	pct.
TTTATCACAGGCTCCAGGAA**GGG**TTTG	566115	WT	96.49%
TTTATCACAGGCTCCAGgGAAGGGTTTG	4691	+1 g	0.80%
TTTATCACAGGCTCCA-GAAGGGTTTG	3010	-1	0.51%
TTTATCACAGGCTCC-GGAAGGGTTTG	1086	-1	0.19%
TTTATCACAGGCTCCAGaGAAGGGTTTG	931	+1 a	0.16%
TTTATCACAGGCTCC----AGGGTTTG	885	-4	0.15%
TTTATCACAGGCTCCAAGGAAGGGTTTG	647	+1 Ins	0.11%
TTTATCACAGGCTCC--GAAGGGTTTG	527	-2	0.09%
TTTATCAC-----------AGGGTTTG	492	-11	0.08%
TTTATCACAGGCT-CAGGAAGGGTTTG	343	-1	0.06%

12h

Total	INDEL	Ins	Del	HDR
228883	6.17%	2.22%	3.95%	0.00%

Typical seqs	Reads	Type	pct.
TTTATCACAGGCTCCAGGAA**GGG**TTTG	214750	WT	93.83%
TTTATCACAGGCTCCAGgGAAGGGTTTG	2654	+1 g	1.16%
TTTATCACAGGCTCCA-GAAGGGTTTG	2171	-1	0.95%
TTTATCACAGGCTCC----AGGGTTTG	820	-4	0.36%
TTTATCACAGGCTCCAGaGAAGGGTTTG	544	+1 a	0.24%
TTTATCAC-----------AGGGTTTG	514	-11	0.22%
TTTATCACAGGCTCC-GGAAGGGTTTG	440	-1	0.19%
TTTATCACAGGCTCCAAGGAAGGGTTTG	378	+1 Ins	0.17%
TTTATCACAGGCTCC--GAAGGGTTTG	364	-2	0.16%
TTTATCA-------CAGGAAGGGTTTG	168	-7	0.07%

24h

Total	INDEL	Ins	Del	HDR
325222	15.69%	4.27%	11.42%	0.00%

Typical seqs	Reads	Type	pct.
TTTATCACAGGCTCCAGGAA**GGG**TTTG	274200	WT	84.31%
TTTATCACAGGCTCCAGgGAAGGGTTTG	6135	+1 g	1.89%
TTTATCACAGGCTCCA-GAAGGGTTTG	5691	-1	1.75%
TTTATCACAGGCTCC----AGGGTTTG	4634	-4	1.42%
TTTATCAC-----------AGGGTTTG	4157	-11	1.28%
TTTATCACA-----------------G	2208	-17	0.68%
TTTATCACAGGCTCCAGaGAAGGGTTTG	1535	+1 a	0.47%
TTTATCACAGG----------------	1485	-18	0.46%
TTTATCACAGGCTCC-GGAAGGGTTTG	1159	-1	0.36%
TTTATCACAGGCTCCAAGGAAGGGTTTG	994	+1 Ins	0.31%

48h

Total	INDEL	Ins	Del	HDR
245437	22.94%	5.87%	17.07%	0.00%

Typical seqs	Reads	Type	pct.
TTTATCACAGGCTCCAGGAA**GGG**TTTG	189136	WT	77.06%
TTTATCACAGGCTCCAGgGAAGGGTTTG	6079	+1 g	2.48%
TTTATCACAGGCTCC----AGGGTTTG	5901	-4	2.40%
TTTATCACAGGCTCCA-GAAGGGTTTG	5589	-1	2.28%
TTTATCAC-----------AGGGTTTG	4473	-11	1.82%
TTTATCACA-----------------G	2078	-17	0.85%
TTTATCACAGG----------------	1868	-18	0.76%
TTTATCACAGGCTCCAGaGAAGGGTTTG	1266	+1 a	0.52%
TTTATCACAGGCTCCAAGGAAGGGTTTG	1210	+1 Ins	0.49%
TTTATCACAGGCTCC-GGAAGGGTTTG	1064	-1	0.43%

P41-P50-WT-U937-Syn35crBC1L11Gata-a4-KO-BC14

Total | INDEL	4h	Ins	Del	HDR
295551 | 5.23%		4.51%	0.72%	0.00%
**Typical seqs**		**Reads**	**Type**	**pct.**
TTTTATCACAGGCTCCA|GGAAGGGTTT		280095	WT	94.77%
TTTTATCACAGGCTCCAaGGAAGGGTTT		11270	+1 a	3.81%
TTTTATCACA-------GGAAGGGTTT		348	-7	0.12%
TTTTATCACAGGCTC-AGGAAGGGTTT		250	-1	0.08%
TTTTATCACAGGCTCCAaGGAAGGGTTG		203	+1 a	0.07%
TTTTATCACAGGCTCCACAGGAAGGGTTT		158	+2	0.05%
TTTTATCACAGGCTC--GGAAGGGTTT		136	-2	0.05%
TTTTATCACAGGCTCCA--AAGGGTTT		128	-2	0.04%
TTTTATCACAGGCTCC-GGAAGGGTTT		121	-1	0.04%
TTTTATCACAGGCTCCA-GAAGGGTTT		107	-1	0.04%

Total | INDEL	8h	Ins	Del	HDR
541688 | 10.67%		8.37%	2.29%	0.00%
**Typical seqs**		**Reads**	**Type**	**pct.**
TTTTATCACAGGCTCCA|GGAAGGGTTT		483901	WT	89.33%
TTTTATCACAGGCTCCAaGGAAGGGTTT		37691	+1 a	6.96%
TTTTATCACA-------GGAAGGGTTT		1518	-7	0.28%
TTTTATCACAGGCTC--GGAAGGGTTT		843	-2	0.16%
TTTTATCACAGGCTCCA--AAGGGTTT		742	-2	0.14%
TTTTATCACAGGCTC-AGGAAGGGTTT		697	-1	0.13%
TTTTATCACAGGCTCC-GGAAGGGTTT		680	-1	0.13%
TTTTATCAC-----------AGGGTTT		662	-11	0.12%
TTTTATCACAGGCTCCACAGGAAGGGTTT		639	+2	0.12%
TTTTATCACAGGCTCCAaGGAAGGGTTG		640	+1 a	0.12%

Total | INDEL	12h	Ins	Del	HDR
22572 | 14.83%		10.96%	3.87%	0.00%
**Typical seqs**		**Reads**	**Type**	**pct.**
TTTTATCACAGGCTCCA|GGAAGGGTTT		19225	WT	85.17%
TTTTATCACAGGCTCCAaGGAAGGGTTT		2090	+1 a	9.26%
TTTTATCACA-------GGAAGGGTTT		111	-7	0.49%
TTTTATCACAGGCTCCA--AAGGGTTT		66	-2	0.29%
TTTTATCAC-----------AGGGTTT		54	-11	0.24%
TTTTATCACAGGCTC--GGAAGGGTTT		40	-2	0.18%
TTTTATCACAGGCTC-AGGAAGGGTTT		38	-1	0.17%
TTTTATCACAGGCTCCACAGGAAGGGTTT		35	+2	0.16%
TTTTATCACAGGCTCC-GGAAGGGTTT		31	-1	0.14%
TTTTATCACAGGCTCCAgGGAAGGGTTT		32	+1 g	0.14%

Total | INDEL	24h	Ins	Del	HDR
87113 | 33.77%		19.70%	14.07%	0.00%
**Typical seqs**		**Reads**	**Type**	**pct.**
TTTTATCACAGGCTCCA|GGAAGGGTTT		57692	WT	66.23%
TTTTATCACAGGCTCCAaGGAAGGGTTT		13949	+1 a	16.01%
TTTTATCACA-------GGAAGGGTTT		2199	-7	2.52%
TTTTATCAC-----------AGGGTTT		1612	-11	1.85%
TTTTATCACA-----------------		929	-17	1.07%
TTTTATCACAGG---------------		478	-18	0.55%
TTTTATCACAGGCTCCA--AAGGGTTT		378	-2	0.43%
TTTTATCACAGGCTC--GGAAGGGTTT		373	-2	0.43%
TTTTATCACA------------GGTTT		360	-12	0.41%
TTTTATCACAGGCTCC-GGAAGGGTTT		294	-1	0.34%

Total | INDEL	48h	Ins	Del	HDR
24850 | 45.33%		24.58%	20.75%	0.00%
**Typical seqs**		**Reads**	**Type**	**pct.**
TTTTATCACAGGCTCCA|GGAAGGGTTT		13586	WT	54.67%
TTTTATCACAGGCTCCAaGGAAGGGTTT		4956	+1 a	19.94%
TTTTATCACA-------GGAAGGGTTT		1126	-7	4.53%
TTTTATCAC-----------AGGGTTT		700	-11	2.82%
TTTTATCACA-----------------		351	-17	1.41%
TTTTATCACAGG---------------		260	-18	1.05%
TTTTATCACAGGCTCCA--AAGGGTTT		147	-2	0.59%
TTTTATCACA------------GGTTT		121	-12	0.49%
TTTTATCACAGGCTC--GGAAGGGTTT		125	-2	0.50%
TTTTATCACAGGCTCC-GGAAGGGTTT		108	-1	0.43%

P41-P50-WT-U937-Syn36crBC1L11Gata-a5-KO-BC15

Total | INDEL	4h	Ins	Del	HDR
86702 | 2.40%		1.41%	0.99%	0.00%

Typical seqs	Reads	Type	pct.
TTGCTTTTATCACAGGC|TCC**AGG**AAGG	84619	WT	97.60%
TTGCTTTTATCACAGGCtTCCAGGAAGG	640	+1 t	0.74%
TTGCTTTTATCACAGGCcTCCAGGAAGG	144	+1 c	0.17%
TTGCTTTTATCACAGGCgTCCAGGAAGG	129	+1 g	0.15%
TTGCTTTTATCACAGG--CCAGGAAGG	113	-2	0.13%
TTGCTTTTATCA-------CAGGAAGG	94	-7	0.11%
TTGCTTTTATCACAGGCaTCCAGGAAGG	84	+1 a	0.10%
TTGCTTTTATCACAGGCtCCAAGGAAGG	84	+1 t	0.10%
TTGCTTTTATCACAGGC----GGAAGG	83	-4	0.10%
TTGCTTTTATCACAGGC-CCAGGAAGG	71	-1	0.08%

Total | INDEL	8h	Ins	Del	HDR
197956 | 7.32%		3.35%	3.97%	0.00%

Typical seqs	Reads	Type	pct.
TTGCTTTTATCACAGGC|TCC**AGG**AAGG	183460	WT	92.68%
TTGCTTTTATCACAGGCtTCCAGGAAGG	3463	+1 t	1.75%
TTGCTTTTATCACAGG--CCAGGAAGG	895	-2	0.45%
TTGCTTTTATCACAGGC-----GAAGG	865	-5	0.44%
TTGCTTTTA--------TCCAGGAAGG	789	-8	0.40%
TTGCTTTTATCACAGGCcTCCAGGAAGG	743	+1 c	0.38%
TTGCTTTTATCACAGGCgTCCAGGAAGG	704	+1 g	0.36%
TTGCTTTTATCACAGGC-CCAGGAAGG	651	-1	0.33%
TTGCTTTTATCA-------CAGGAAGG	630	-7	0.32%
TTGCTTTTATCACAGGC----GGAAGG	453	-4	0.23%

Total | INDEL	12h	Ins	Del	HDR
184792 | 14.02%		5.14%	8.89%	0.00%

Typical seqs	Reads	Type	pct.
TTGCTTTTATCACAGGC|TCC**AGG**AAGG	158878	WT	85.98%
TTGCTTTTATCACAGGCtTCCAGGAAGG	5112	+1 t	2.77%
TTGCTTTTATCA-------CAGGAAGG	2018	-7	1.09%
TTGCTTTTATCACAGG--CCAGGAAGG	1705	-2	0.92%
TTGCTTTTA--------TCCAGGAAGG	1671	-8	0.90%
TTGCTTTTATCACAGGC-----GAAGG	1470	-5	0.80%
TTGCTTTTATCACAGGC-CCAGGAAGG	1076	-1	0.58%
TTGCTTTTATCACAGGCcTCCAGGAAGG	1116	+1 c	0.60%
TTGCTTTTATCACAGGCgTCCAGGAAGG	953	+1 g	0.52%
TTGCTTTTATCACAGGC----GGAAGG	916	-4	0.50%

Total | INDEL	24h	Ins	Del	HDR
116533 | 32.34%		8.78%	23.56%	0.00%

Typical seqs	Reads	Type	pct.
TTGCTTTTATCACAGGC|TCC**AGG**AAGG	78844	WT	67.66%
TTGCTTTTATCA-------CAGGAAGG	6715	-7	5.76%
TTGCTTTTATCACAGGCtTCCAGGAAGG	5150	+1 t	4.42%
TTGCTTTTA--------TCCAGGAAGG	4448	-8	3.82%
TTGCTTTTATCACAGGC-----GAAGG	1756	-5	1.51%
TTGCTTTTATCACAGG--CCAGGAAGG	1635	-2	1.40%
TTGCTTTTATCACAGGC-CCAGGAAGG	1167	-1	1.00%
TTGCTTTTATCACAGGCcTCCAGGAAGG	1021	+1 c	0.88%
TTGCTTTTATCACAGGCgTCCAGGAAGG	948	+1 g	0.81%
TTGCTTTTATCACAGGC------AAGG	796	-6	0.68%

Total | INDEL	48h	Ins	Del	HDR
1079 | 33.18%		9.08%	24.10%	0.00%

Typical seqs	Reads	Type	pct.
TTGCTTTTATCACAGGC|TCC**AGG**AAGG	721	WT	66.82%
TTGCTTTTATCA-------CAGGAAGG	82	-7	7.60%
TTGCTTTTATCACAGGCtTCCAGGAAGG	60	+1 t	5.56%
TTGCTTTTA--------TCCAGGAAGG	46	-8	4.26%
TTGCTTTTATCACAGGC-----GAAGG	20	-5	1.85%
TTGCTTTTATCACAGG--CCAGGAAGG	14	-2	1.30%
TTGCTTTTATCACAGGCgTCCAGGAAGG	13	+1 g	1.20%
TTGCTTTTATCACAGGC-CCAGGAAGG	12	-1	1.11%
TTGCTTTTATCACAGGCcTCCAGGAAGG	11	+1 c	1.02%
TTGCTTTTATCACAGGC------AAGG	8	-6	0.74%

P41-P50-WT-U937-Syn37crBC1L11Gata-a6-KO-BC16

Total | INDEL	4h	Ins	Del	HDR
72787 | 1.00%		0.70%	0.29%	0.00%

Typical seqs	Reads	Type	pct.
CTAACAGTTGCTTTTAT|CAC**AGG**CTCC	72061	WT	99.00%
CTAACAGTTGCTTTTATtCACAGGCTCC	453	+1 t	0.62%
CTAACAGTTGC-TTTATCACAGGCTCC	46	-1	0.06%
CTAACAGTTGCTTTTA-CACAGGCTCC	23	-1	0.03%
CTA---------------ACAGGCTCC	12	-15	0.02%
CTAACAGTTGCTTTTATATCACAGGCTCC	12	+2	0.02%
CTAACAGTTGCTTTTAT--CAGGCTCC	12	-2	0.02%
CTAACAGTTGCTTTT-TCACAGGCTCC	7	-1	0.01%
CTAACAGTT-------------GCTCC	7	-13	0.01%
CTAACAGTTGCTTTT---ACAGGCTCC	6	-3	0.01%

Total | INDEL	8h	Ins	Del	HDR
147440 | 2.52%		1.57%	0.95%	0.00%

Typical seqs	Reads	Type	pct.
CTAACAGTTGCTTTTAT|CAC**AGG**CTCC	143718	WT	97.48%
CTAACAGTTGCTTTTATtCACAGGCTCC	1884	+1 t	1.28%
CTAACAGTTGCTTTT--CACAGGCTCC	129	-2	0.09%
CTA---------------ACAGGCTCC	113	-15	0.08%
CTAACAGTTGCTTTTATATCACAGGCTCC	100	+2	0.07%
CTAACAGTTGC-TTTATCACAGGCTCC	97	-1	0.07%
CTAA-------------CACAGGCTCC	92	-13	0.06%
CTAACAGTTGCTTTTA-CACAGGCTCC	82	-1	0.06%
CTAACAGTTGCTTTTAT--CAGGCTCC	71	-2	0.05%
CTAACAGTTGCTTTTAATCACAGGCTCC	58	+1 Ins	0.04%

Total | INDEL	12h	Ins	Del	HDR
76285 | 4.44%		2.30%	2.14%	0.00%

Typical seqs	Reads	Type	pct.
CTAACAGTTGCTTTTAT|CAC**AGG**CTCC	72900	WT	95.56%
CTAACAGTTGCTTTTATtCACAGGCTCC	1407	+1 t	1.84%
CTA---------------ACAGGCTCC	233	-15	0.31%
CTAACAGTTGCTTTT--CACAGGCTCC	149	-2	0.20%
CTAACAGTT-------------GCTCC	105	-13	0.14%
CTAACAGTTGCTTTTAT-ACAGGCTCC	73	-1	0.10%
CTAACAGTTGCTTTTAT--CAGGCTCC	81	-2	0.11%
CTAACAGTTGCTTTTA-CACAGGCTCC	69	-1	0.09%
CTAA-------------CACAGGCTCC	63	-13	0.08%
CTAACAGTTGCTTTTAATCACAGGCTCC	55	+1 Ins	0.07%

Total | INDEL	24h	Ins	Del	HDR
66550 | 14.62%		5.00%	9.62%	0.00%

Typical seqs	Reads	Type	pct.
CTAACAGTTGCTTTTAT|CAC**AGG**CTCC	56819	WT	85.38%
CTAACAGTTGCTTTTATtCACAGGCTCC	2459	+1 t	3.69%
CTA---------------ACAGGCTCC	1427	-15	2.14%
CTAACAGTT-------------GCTCC	772	-13	1.16%
CTAA-------------CACAGGCTCC	314	-13	0.47%
CTAACAGTTGCTTTT--CACAGGCTCC	256	-2	0.38%
CTAACAGTTGCTTTT-----AGGCTCC	178	-5	0.27%
CTAACAGTTGCTTTTAT--CAGGCTCC	169	-2	0.25%
CTAACAGTTGC-------ACAGGCTCC	124	-7	0.19%
CTAACAGTTGCTTTTA-CACAGGCTCC	130	-1	0.20%

Total | INDEL	48h	Ins	Del	HDR
44420 | 19.92%		6.63%	13.29%	0.00%

Typical seqs	Reads	Type	pct.
CTAACAGTTGCTTTTAT|CAC**AGG**CTCC	35573	WT	80.08%
CTAACAGTTGCTTTTATtCACAGGCTCC	2136	+1 t	4.81%
CTA---------------ACAGGCTCC	1278	-15	2.88%
CTAACAGTT-------------GCTCC	715	-13	1.61%
CTAA-------------CACAGGCTCC	350	-13	0.79%
CTAACAGTTGCTTTT--CACAGGCTCC	223	-2	0.50%
CTAACAGTTGCT-----AACAGGCTCC	153	-5	0.34%
CTAACAGTTGCTTTT-----AGGCTCC	142	-5	0.32%
CTAACAGTTGCTTTTAT--CAGGCTCC	138	-2	0.31%
CTAACAGTTGC-------ACAGGCTCC	128	-7	0.29%

P41-P50-WT-U937-Syn38crBC1L11Gata-b1-KO-BC17

Total | INDEL	4h	Ins	Del	HDR
124947 | 4.79%		1.74%	3.05%	0.00%

Typical seqs	Reads	Type	pct.
CTTCAAAGTTGTATTGA|CCC**TGG**TGTG	118962	WT	95.21%
CTTCAAAGTTGTATTGAaCCCTGGTGTG	560	+1 a	0.45%
CTTCAAAGTTGTATTG-CCCTGGTGTG	478	-1	0.38%
CTTCAAAGTTGTATT--CCCTGGTGTG	418	-2	0.33%
CTTCAAAGTTGTATTGACCCT-GTGTG	267	-1	0.21%
CTTCAAAGTTGTATTGA--CTGGTGTG	232	-2	0.19%
CTTCAAAGTTGTAT------TGGTGTG	192	-6	0.15%
CTTCAAAGTTGTATTGGCCCTGGTTGTG	165	+1 Ins	0.13%
CTTCAAAGTTGTATTGA-CCTGGTGTG	147	-1	0.12%
CTTCAAAGTTGTACTGACCCTGGTTGTG	127	+1 Ins	0.10%

Total | INDEL	8h	Ins	Del	HDR
223445 | 8.36%		2.06%	6.29%	0.00%

Typical seqs	Reads	Type	pct.
CTTCAAAGTTGTATTGA|CCC**TGG**TGTG	204772	WT	91.64%
CTTCAAAGTTGTATTGAaCCCTGGTGTG	2468	+1 a	1.10%
CTTCAAAGTTGTATTG-CCCTGGTGTG	2305	-1	1.03%
CTTCAAAGTTGTATT--CCCTGGTGTG	1738	-2	0.78%
CTTCAAAGTTGTATTGA--CTGGTGTG	1105	-2	0.49%
CTTCAAAGTTGTAT------TGGTGTG	688	-6	0.31%
CTTCAAAGTTGTATTGAcCCCTGGTGTG	516	+1 c	0.23%
CTTCAAAGTTGTATTGA-CCTGGTGTG	463	-1	0.21%
CTTCAAAGTTGTATTGA----GGTGTG	400	-4	0.18%
CTTCAAAGTTGTATTGA---TGGTGTG	403	-3	0.18%

Total | INDEL	12h	Ins	Del	HDR
111855 | 19.20%		3.85%	15.34%	0.00%

Typical seqs	Reads	Type	pct.
CTTCAAAGTTGTATTGA|CCC**TGG**TGTG	90380	WT	80.80%
CTTCAAAGTTGTATTGAaCCCTGGTGTG	2254	+1 a	2.02%
CTTCAAAGTTGTATTG-CCCTGGTGTG	2116	-1	1.89%
CTTCAAAGTTGTATT--CCCTGGTGTG	1461	-2	1.31%
CTTCAAAGTTGTAT------TGGTGTG	1408	-6	1.26%
CTTCAAAGTTGTATTGA--CTGGTGTG	1357	-2	1.21%
CTTCAAAGTTGTATTGA-CCTGGTGTG	573	-1	0.51%
CTTCAAAGTTGTATTGA----GGTGTG	523	-4	0.47%
CTTCAAAGTTGTATTGAcCCCTGGTGTG	477	+1 c	0.43%
CTTCAAAGTTGTAT---CCCTGGTGTG	432	-3	0.39%

Total | INDEL	24h	Ins	Del	HDR
93955 | 41.02%		7.29%	33.73%	0.00%

Typical seqs	Reads	Type	pct.
CTTCAAAGTTGTATTGA|CCC**TGG**TGTG	55415	WT	58.98%
CTTCAAAGTTGTAT------TGGTGTG	3640	-6	3.87%
CTTCAAAGTTGTATTG-CCCTGGTGTG	2730	-1	2.91%
CTTCAAAGTTGTATTGAaCCCTGGTGTG	2748	+1 a	2.92%
CTTCAAAGTTGTATT--CCCTGGTGTG	2144	-2	2.28%
CTTCAAAGTTGTATTGA--CTGGTGTG	1945	-2	2.07%
CTTCAAAGTTGTATTGA-CCTGGTGTG	793	-1	0.84%
CTTCAAAGT--------------TGTG	783	-14	0.83%
CTTCAAAGT-----------TGGTGTG	773	-11	0.82%
CTTCAAAGTTGTATTGAcCCCTGGTGTG	673	+1 c	0.72%

Total | INDEL	48h	Ins	Del	HDR
14636 | 42.53%		6.63%	35.90%	0.00%

Typical seqs	Reads	Type	pct.
CTTCAAAGTTGTATTGA|CCC**TGG**TGTG	8412	WT	57.47%
CTTCAAAGTTGTAT------TGGTGTG	759	-6	5.19%
CTTCAAAGTTGTATTG-CCCTGGTGTG	428	-1	2.92%
CTTCAAAGTTGTATTGAaCCCTGGTGTG	417	+1 a	2.85%
CTTCAAAGTTGTATT--CCCTGGTGTG	326	-2	2.23%
CTTCAAAGTTGTATTGA--CTGGTGTG	280	-2	1.91%
CTTCAAAGT-----------TGGTGTG	178	-11	1.22%
CTTCAAAGTTGTATTGA-CCTGGTGTG	134	-1	0.92%
CTTCAAAGT--------------TGTG	125	-14	0.85%
CTTCAAAGTTGTATTGAcCCCTGGTGTG	104	+1 c	0.71%

P41-P50-WT-U937-Syn39crBC1L11Gata-b2-KO-BC18

Total | INDEL	4h	Ins	Del	HDR
38471 | 28.05%		26.09%	1.97%	0.00%

Typical seqs	Reads	Type	pct.
CTCTTAGACATAACACA|CCAGGGTCAA	27679	WT	71.95%
CTCTTAGACATAACACAaCCAGGGTCAA	8877	+1 a	23.07%
CTCTTAGACATAACA--CCAGGGTCAA	102	-2	0.27%
CTCTTAGACATAACACA-CAGGGTCAA	91	-1	0.24%
CTCTTAGACATAACA---CAGGGTCAA	65	-3	0.17%
CTCTTAGACATAACACAAACCAGGGTCAA	59	+2	0.15%
CTCTTAGACATAACACAaCCAGGGCCAA	62	+1 a	0.16%
CTCTTAGACATAA-----CAGGGTCAA	52	-5	0.14%
CCCTTAGACATAACACAACCAGGGTCAA	47	+1 Ins	0.12%
CTCTTAGACATAACA-ACCAGGGTCAA	44	-1	0.11%

Total | INDEL	8h	Ins	Del	HDR
53373 | 42.31%		37.71%	4.60%	0.00%

Typical seqs	Reads	Type	pct.
CTCTTAGACATAACACA|CCAGGGTCAA	30791	WT	57.69%
CTCTTAGACATAACACAaCCAGGGTCAA	17603	+1 a	32.98%
CTCTTAGACATAACA--CCAGGGTCAA	343	-2	0.64%
CTCTTAGACATAACA---CAGGGTCAA	261	-3	0.49%
CTCTTAGACATAA-----CAGGGTCAA	225	-5	0.42%
CTCTTAGACATAACACA-CAGGGTCAA	205	-1	0.38%
CTCTTAGACATAACACAAACCAGGGTCAA	162	+2	0.30%
CTCTTAGACATAACA-ACCAGGGTCAA	113	-1	0.21%
CTCTTAGACATAACACAaCCAGGGCCAA	114	+1 a	0.21%
CTCTTAGACATAAC----CAGGGTCAA	108	-4	0.20%

Total | INDEL	12h	Ins	Del	HDR
33046 | 53.51%		45.08%	8.44%	0.00%

Typical seqs	Reads	Type	pct.
CTCTTAGACATAACACA|CCAGGGTCAA	15362	WT	46.49%
CTCTTAGACATAACACAaCCAGGGTCAA	13062	+1 a	39.53%
CTCTTAGACATAACA--CCAGGGTCAA	277	-2	0.84%
CTCTTAGACATAACA---CAGGGTCAA	265	-3	0.80%
CTCTTAGACATAACACA-CAGGGTCAA	256	-1	0.77%
CTCTTAGACATAA-----CAGGGTCAA	267	-5	0.81%
CTCTTAGACATAAC----CAGGGTCAA	143	-4	0.43%
CTCTTAGACATAACA-ACCAGGGTCAA	121	-1	0.37%
CTCTTAGACATAACACAAACCAGGGTCAA	114	+2	0.34%
CTCTTAGA----------CAGGGTCAA	95	-10	0.29%

Total | INDEL	24h	Ins	Del	HDR
33668 | 66.97%		51.79%	15.17%	0.00%

Typical seqs	Reads	Type	pct.
CTCTTAGACATAACACA|CCAGGGTCAA	11122	WT	33.03%
CTCTTAGACATAACACAaCCAGGGTCAA	15433	+1 a	45.84%
CTCTTAGACATAA-----CAGGGTCAA	726	-5	2.16%
CTCTTAGACATAACA---CAGGGTCAA	471	-3	1.40%
CTCTTAGACATAACA--CCAGGGTCAA	396	-2	1.18%
CTCTTAGACATAACACA-CAGGGTCAA	309	-1	0.92%
CTCTTAGA----------CAGGGTCAA	275	-10	0.82%
CTCTTAGACATAAC----CAGGGTCAA	164	-4	0.49%
CTCTTAGACATAACACAAACCAGGGTCAA	137	+2	0.41%
CTCTT--------------AGGGTCAA	123	-14	0.37%

Total | INDEL	48h	Ins	Del	HDR
22462 | 76.77%		58.41%	18.37%	0.00%

Typical seqs	Reads	Type	pct.
CTCTTAGACATAACACA|CCAGGGTCAA	5217	WT	23.23%
CTCTTAGACATAACACAaCCAGGGTCAA	11704	+1 a	52.11%
CTCTTAGACATAA-----CAGGGTCAA	572	-5	2.55%
CTCTTAGACATAACA---CAGGGTCAA	342	-3	1.52%
CTCTTAGACATAACA--CCAGGGTCAA	295	-2	1.31%
CTCTTAGA----------CAGGGTCAA	291	-10	1.30%
CTCTTAGACATAACACA-CAGGGTCAA	231	-1	1.03%
CTCTTAGACATAAC----CAGGGTCAA	132	-4	0.59%
CTCTT--------------AGGGTCAA	116	-14	0.52%
CTCTTAGACATAACACAAACCAGGGTCAA	99	+2	0.44%

P41-P50-WT-U937-Syn40crBC1L11Gata-b3-KO-BC19

4h

Total	INDEL	Ins	Del	HDR
97074	2.62%	1.37%	1.24%	0.00%

Typical seqs	Reads	Type	pct.
ACTCTTAGACATAACAC|ACC**AGG**GTCA	94534	WT	97.38%
ACTCTTAGACATAACACaACCAGGGTCA	732	+1 a	0.75%
ACTCTTAGACATAAC--ACCAGGGTCA	460	-2	0.47%
ACTCTTAGACATAACA-ACCAGGGTCA	161	-1	0.17%
ACTCTTAGACATAACACcACCAGGGTCA	167	+1 c	0.17%
ACTCTTAGACATAACACtACCAGGGTCA	159	+1 t	0.16%
ACTCTTAGACATA----ACCAGGGTCA	105	-4	0.11%
ACTCTTAGACATAACACA-CAGGGTCA	83	-1	0.09%
ACTCTTAGACATAACACgACCAGGGTCA	48	+1 g	0.05%
ACTCTTAGACATAA---ACCAGGGTCA	43	-3	0.04%

8h

Total	INDEL	Ins	Del	HDR
91091	4.94%	1.86%	3.08%	0.00%

Typical seqs	Reads	Type	pct.
ACTCTTAGACATAACAC|ACC**AGG**GTCA	86591	WT	95.06%
ACTCTTAGACATAAC--ACCAGGGTCA	1023	-2	1.12%
ACTCTTAGACATAACACaACCAGGGTCA	698	+1 a	0.77%
ACTCTTAGACATAACACcACCAGGGTCA	336	+1 c	0.37%
ACTCTTAGACATAACA-ACCAGGGTCA	307	-1	0.34%
ACTCTTAGACATAACACtACCAGGGTCA	215	+1 t	0.24%
ACTCTTAGACATA----ACCAGGGTCA	212	-4	0.23%
ACTCTTAGACATAACACgACCAGGGTCA	108	+1 g	0.12%
ACTCTTAGACATAA---ACCAGGGTCA	119	-3	0.13%
ACTCTTAGACAT-----ACCAGGGTCA	110	-5	0.12%

12h

Total	INDEL	Ins	Del	HDR
51765	9.15%	3.11%	6.04%	0.00%

Typical seqs	Reads	Type	pct.
ACTCTTAGACATAACAC|ACC**AGG**GTCA	47027	WT	90.85%
ACTCTTAGACATAAC--ACCAGGGTCA	1006	-2	1.94%
ACTCTTAGACATAACACaACCAGGGTCA	684	+1 a	1.32%
ACTCTTAGACATA----ACCAGGGTCA	363	-4	0.70%
ACTCTTAGACATAACA-ACCAGGGTCA	303	-1	0.59%
ACTCTTAGACATAACACcACCAGGGTCA	238	+1 c	0.46%
ACTCTTAGACATAACACtACCAGGGTCA	215	+1 t	0.42%
ACTCTTAGACATAA-----CAGGGTCA	160	-5	0.31%
ACTCTTAGACATAACA---CAGGGTCA	112	-3	0.22%
ACTCTTAGACATAACACgACCAGGGTCA	80	+1 g	0.15%

24h

Total	INDEL	Ins	Del	HDR
79923	25.61%	6.34%	19.27%	0.00%

Typical seqs	Reads	Type	pct.
ACTCTTAGACATAACAC|ACC**AGG**GTCA	59454	WT	74.39%
ACTCTTAGACATAAC--ACCAGGGTCA	3520	-2	4.40%
ACTCTTAGACATA----ACCAGGGTCA	1670	-4	2.09%
ACTCTTAGACATAACACaACCAGGGTCA	1576	+1 a	1.97%
ACTCTTAGACATAA-----CAGGGTCA	1450	-5	1.81%
ACTCTTAGACATAACA-ACCAGGGTCA	904	-1	1.13%
ACTCTTAGACATAACACcACCAGGGTCA	851	+1 c	1.06%
ACTCTTAGACATAACACtACCAGGGTCA	814	+1 t	1.02%
ACTCTTAGA----------CAGGGTCA	638	-10	0.80%
ACTCTTAG---------ACCAGGGTCA	469	-9	0.59%

48h

Total	INDEL	Ins	Del	HDR
68009	34.93%	7.95%	26.98%	0.00%

Typical seqs	Reads	Type	pct.
ACTCTTAGACATAACAC|ACC**AGG**GTCA	44256	WT	65.07%
ACTCTTAGACATAAC--ACCAGGGTCA	4388	-2	6.45%
ACTCTTAGACATA----ACCAGGGTCA	2348	-4	3.45%
ACTCTTAGACATAA-----CAGGGTCA	1850	-5	2.72%
ACTCTTAGACATAACACaACCAGGGTCA	1491	+1 a	2.19%
ACTCTTAGACATAACA-ACCAGGGTCA	1220	-1	1.79%
ACTCTTAGACATAACACtACCAGGGTCA	905	+1 t	1.33%
ACTCTTAGACATAACACcACCAGGGTCA	775	+1 c	1.14%
ACTCTTAGA----------CAGGGTCA	633	-10	0.93%
ACTCTTAG---------ACCAGGGTCA	569	-9	0.84%

P41-P50-WT-U937-Syn59crMYH6a-KO-BC11

Total | INDEL	4h	Ins	Del	HDR
122402 | 0.41%		0.06%	0.36%	0.00%

Typical seqs	Reads	Type	pct.
GCAGTAGGGGGCCTGAG|AGGAGGGAGG	121898	WT	99.59%
GCAGTAGGGGGCCTG--AGGAGGGAGG	128	-2	0.10%
GCAGTAGGGGGCCTGAGaAGGAGGGAGG	31	+1 a	0.03%
GCAGTAGGGGGCCTGAGA-GAGGGAGG	28	-1	0.02%
GCAGTAGGGGG-CTGAGAGGAGGGAGG	16	-1	0.01%
GCAGT------------AGGAGGGAGG	15	-12	0.01%
GCAGTAGGGGGCCTGAGAGG-GGGAGG	20	-1	0.02%
GCAGTAGGGGGCCT-----GAGGGAGG	14	-5	0.01%
GCAGTAGGGGGCCTGA-AGGAGGGAGG	12	-1	0.01%
GCAGTAGGGGGC-----AGGAGGGAGG	10	-5	0.01%

Total | INDEL	8h	Ins	Del	HDR
496381 | 1.97%		0.25%	1.72%	0.00%

Typical seqs	Reads	Type	pct.
GCAGTAGGGGGCCTGAG|AGGAGGGAGG	486600	WT	98.03%
GCAGTAGGGGGCCTG--AGGAGGGAGG	2903	-2	0.58%
GCAGTAGGGGGCCTGAGgAGGAGGGAGG	596	+1 g	0.12%
GCAGTAGGGGGCCT-----GAGGGAGG	568	-5	0.11%
GCAGTAGGGGGCCTGA-AGGAGGGAGG	459	-1	0.09%
GCAGT------------AGGAGGGAGG	318	-12	0.06%
GCAGT---------------AGGGAGG	254	-15	0.05%
GCAGTAGGGGGC-----AGGAGGGAGG	241	-5	0.05%
GCAGTAGGGGGCCT---AGGAGGGAGG	224	-3	0.05%
GCAGTAGGGGGCCTGAGtAGGAGGGAGG	212	+1 t	0.04%

Total | INDEL	12h	Ins	Del	HDR
395045 | 3.38%		0.36%	3.03%	0.00%

Typical seqs	Reads	Type	pct.
GCAGTAGGGGGCCTGAG|AGGAGGGAGG	381676	WT	96.62%
GCAGTAGGGGGCCTG--AGGAGGGAGG	3940	-2	1.00%
GCAGTAGGGGGCCT-----GAGGGAGG	805	-5	0.20%
GCAGTAGGGGGCCTGA-AGGAGGGAGG	797	-1	0.20%
GCAGTAGGGGGCCTGAGgAGGAGGGAGG	504	+1 g	0.13%
GCAGT------------AGGAGGGAGG	454	-12	0.11%
GCAGTAGGGGGCCTGAG-GGAGGGAGG	457	-1	0.12%
GCAGTAGGG----------------GG	407	-16	0.10%
GC---------------AGGAGGGAGG	399	-15	0.10%
GCAGTAGGGGGC-----AGGAGGGAGG	314	-5	0.08%

Total | INDEL	24h	Ins	Del	HDR
358779 | 12.15%		0.97%	11.18%	0.00%

Typical seqs	Reads	Type	pct.
GCAGTAGGGGGCCTGAG|AGGAGGGAGG	315205	WT	87.85%
GCAGTAGGGGGCCTG--AGGAGGGAGG	6253	-2	1.74%
GCAGTAGGG----------------GG	4858	-16	1.35%
GC---------------AGGAGGGAGG	3978	-15	1.11%
GCAGT---------------AGGGAGG	3094	-15	0.86%
GCAGTAGGGGGCCT-----GAGGGAGG	2919	-5	0.81%
GCAGT------------AGGAGGGAGG	1664	-12	0.46%
GCAGTA----------------GGAGG	1180	-16	0.33%
GCAGTAGGGGGCCTGA-AGGAGGGAGG	1040	-1	0.29%
GCAGTAGGGGGCCT---------GAGG	899	-9	0.25%

Total | INDEL	48h	Ins	Del	HDR
339690 | 16.42%		1.32%	15.10%	0.00%

Typical seqs	Reads	Type	pct.
GCAGTAGGGGGCCTGAG|AGGAGGGAGG	283918	WT	83.58%
GCAGTAGGGGGCCTG--AGGAGGGAGG	7517	-2	2.21%
GCAGTAGGG----------------GG	6405	-16	1.89%
GC---------------AGGAGGGAGG	4888	-15	1.44%
GCAGTAGGGGGCCT-----GAGGGAGG	3954	-5	1.16%
GCAGT---------------AGGGAGG	3924	-15	1.16%
GCAGT------------AGGAGGGAGG	2294	-12	0.68%
GCAGTA----------------GGAGG	1794	-16	0.53%
GCAGTAGGGGGCCTGA-AGGAGGGAGG	1256	-1	0.37%
GCAGTAGGGGGCCTGAG-GGAGGGAGG	1134	-1	0.33%

P41-P50-WT-U937-Syn59crMYH6a-AAV-KI-BC11

4h

Total | INDEL	Ins	Del	HDR
325359 | 1.05%	0.13%	0.74%	0.19%

Typical seqs	Reads	Type	pct.
GCAGTAGGGGGCCTGAG|AGGAGGGAGG	321942	WT	98.95%
GCAGTAGGGGGCCTG--AGGAGGGAGG	713	-2	0.22%
GCAGTAGGGGGCCTGAGagacgcgtagttt aaaccAGAGCTGCCAGAGCCAAGG	575	-9 HDR	0.18%
GCAGTAGGGGGCCTGA-AGGAGGGAGG	246	-1	0.08%
GCAGTAGGGGGCCTGAGgAGGAGGGAGG	304	+1 g	0.09%
GCAGTAGGGGGC-----AGGAGGGAGG	152	-5	0.05%
GCAGTAGGGGGCCTGAG----------	142	-14	0.04%
GCAGTAGGGGG-CTGAGAGGAGGGAGG	93	-1	0.03%
GCAGTAGGGGGCCTGAGA-GAGGGAGG	96	-1	0.03%
GCAGTAGGGGGCCTGA------GGAGG	70	-6	0.02%

8h

Total | INDEL	Ins	Del	HDR
380309 | 2.95%	0.30%	1.38%	1.26%

Typical seqs	Reads	Type	pct.
GCAGTAGGGGGCCTGAG|AGGAGGGAGG	369101	WT	97.05%
GCAGTAGGGGGCCTGAGagacgcgtagttt aaaccAGAGCTGCCAGAGCCAAGG	4441	-9 HDR	1.17%
GCAGTAGGGGGCCTG--AGGAGGGAGG	1802	-2	0.47%
GCAGTAGGGGGCCTGAGgAGGAGGGAGG	363	+1 g	0.10%
GCAGTAGGGGGCCTGA-AGGAGGGAGG	337	-1	0.09%
GCAGTAGGGGGCCTGAGaAGGAGGGAGG	191	+1 a	0.05%
GCAGTAGGGGGCCTGAG-GGAGGGAGG	157	-1	0.04%
GCAGTAGGGGGCCTGA---GAGGGAGG	151	-3	0.04%
GCAGT------------AGGAGGGAGG	144	-12	0.04%
GCAGTAGGGGGCCT-----GAGGGAGG	133	-5	0.03%

12h

Total | INDEL	Ins	Del	HDR
297945 | 5.88%	0.32%	2.31%	3.25%

Typical seqs	Reads	Type	pct.
GCAGTAGGGGGCCTGAG|AGGAGGGAGG	280425	WT	94.12%
GCAGTAGGGGGCCTGAGagacgcgtagttt aaaccAGAGCTGCCAGAGCCAAGG	8903	-9 HDR	2.99%
GCAGTAGGGGGCCTG--AGGAGGGAGG	1996	-2	0.67%
GCAGTAGGGGGCCTGA-AGGAGGGAGG	477	-1	0.16%
GCAGTAGGGGGCCTGAG-GGAGGGAGG	363	-1	0.12%
GCAGTAGGGGGCCT-----GAGGGAGG	299	-5	0.10%
GCAGTAGGGGGCCTGAGgAGGAGGGAGG	255	+1 g	0.09%
GCAGTAGGGGGCCTGAGaAGGAGGGAGG	150	+1 a	0.05%
GCAGTAGGGGGCCTGA---GAGGGAGG	140	-3	0.05%
GC---------------AGGAGGGAGG	130	-15	0.04%

24h

Total | INDEL	Ins	Del	HDR
104368 | 16.22%	0.96%	5.09%	10.17%

Typical seqs	Reads	Type	pct.
GCAGTAGGGGGCCTGAG|AGGAGGGAGG	87435	WT	83.78%
GCAGTAGGGGGCCTGAGagacgcgtagttt aaaccAGAGCTGCCAGAGCCAAGG	9748	-9 HDR	9.34%
GCAGTAGGGGGCCTG--AGGAGGGAGG	1167	-2	1.12%
GCAGTAGGGGGCCT-----GAGGGAGG	457	-5	0.44%
GC---------------AGGAGGGAGG	335	-15	0.32%
GCAGTAGGGGGCCTGAGaAGGAGGGAGG	301	+1 a	0.29%
GCAGTAGGGGGCCTGAGgAGGAGGGAGG	240	+1 g	0.23%
GCAGTAGGG----------------GG	218	-16	0.21%
GCAGT---------------AGGGAGG	188	-15	0.18%
GCAGTAGGGGGCCTGA-AGGAGGGAGG	172	-1	0.16%

48h

Total | INDEL	Ins	Del	HDR
98052 | 19.54%	0.96%	7.04%	11.53%

Typical seqs	Reads	Type	pct.
GCAGTAGGGGGCCTGAG|AGGAGGGAGG	78894	WT	80.46%
GCAGTAGGGGGCCTGAGagacgcgtagttt aaaccAGAGCTGCCAGAGCCAAGG	10424	-9 HDR	10.63%
GCAGTAGGGGGCCTG--AGGAGGGAGG	1455	-2	1.48%
GCAGTAGGGGGCCT-----GAGGGAGG	448	-5	0.46%
GCAGTAGGG----------------GG	460	-16	0.47%
GC---------------AGGAGGGAGG	409	-15	0.42%
GCAGT---------------AGGGAGG	304	-15	0.31%
GCAGTAGGGGGCCTGAGgAGGAGGGAGG	240	+1 g	0.24%
GCAGT------------AGGAGGGAGG	236	-12	0.24%
GCAGTAGGGGGCCTGA-AGGAGGGAGG	200	-1	0.20%

P41-P50-WT-U937-Syn60crMYH6b-KO-BC12

Total | INDEL	4h	Ins	Del	HDR
174556 | 2.11%		1.43%	0.68%	0.00%

Typical seqs	Reads	Type	pct.
CAGTAGGGGGCCTGAGA|GGAGGGAGGC	170869	WT	97.89%
CAGTAGGGGGCCTGAGAaGGAGGGAGGC	2041	+1 a	1.17%
CAGTAGGGGGCCTGAG-GGAGGGAGGC	301	-1	0.17%
CAGTAGGGGGCCT--GAGGAGGGAGGC	170	-2	0.10%
CAGTAGGGGGCCTGAGAtGGAGGGAGGC	131	+1 t	0.08%
CAGTAGGGGGCCTGAGAGGA-GGAGGC	81	-1	0.05%
CAGTAGGGGGCCTGAGA-GAGGGAGGC	84	-1	0.05%
CAGT------------AGGAGGGAGGC	70	-12	0.04%
CAGTAGGGGGCCTGAGAgGGAGGGAGGC	63	+1 g	0.04%
CAGTAGGGGGCCTGAGGAGGAGGGAGGC	43	+1 Ins	0.02%

Total | INDEL	8h	Ins	Del	HDR
216241 | 4.25%		2.69%	1.56%	0.00%

Typical seqs	Reads	Type	pct.
CAGTAGGGGGCCTGAGA|GGAGGGAGGC	207054	WT	95.75%
CAGTAGGGGGCCTGAGAaGGAGGGAGGC	4632	+1 a	2.14%
CAGTAGGGGGCCT--GAGGAGGGAGGC	482	-2	0.22%
CAGTAGGGGGCCTGAG-GGAGGGAGGC	387	-1	0.18%
CAGTAGGGGGCCT-----GAGGGAGGC	313	-5	0.14%
CAGTAGGGGGCCTGAGAtGGAGGGAGGC	235	+1 t	0.11%
CAGTAGGGGGCCTGAGAgGGAGGGAGGC	174	+1 g	0.08%
CAGTAGGGGGCCTGA---GAGGGAGGC	150	-3	0.07%
CAGTAGGGGGCCTG---GGAGGGAGGC	143	-3	0.07%
CAGTAGGGGGCCTGAGA--AGGGAGGC	141	-2	0.07%

Total | INDEL	12h	Ins	Del	HDR
3217 | 5.32%		3.29%	2.02%	0.00%

Typical seqs	Reads	Type	pct.
CAGTAGGGGGCCTGAGA|GGAGGGAGGC	3046	WT	94.68%
CAGTAGGGGGCCTGAGAaGGAGGGAGGC	90	+1 a	2.80%
CAGTAGGGGGCCT--GAGGAGGGAGGC	16	-2	0.50%
CAGTAGGGGGCCTGAG-GGAGGGAGGC	11	-1	0.34%
CAGTAGGGGGCCTGAGAtGGAGGGAGGC	4	+1 t	0.12%
CAGTAGGGGGCCT-----GAGGGAGGC	4	-5	0.12%
CAGTAGGGGGCCTGAGA-GAGGGAGGC	4	-1	0.12%
CAGTAGGGGGCCTGAGA----GGAGGC	4	-4	0.12%
CGGTAGGGGGCCTGAGAAGGAGGGAGGC	4	+1 Ins	0.12%
CAGT---------------AGGGAGGC	4	-15	0.12%

Total | INDEL	24h	Ins	Del	HDR
195388 | 19.70%		6.47%	13.23%	0.00%

Typical seqs	Reads	Type	pct.
CAGTAGGGGGCCTGAGA|GGAGGGAGGC	156897	WT	80.30%
CAGTAGGGGGCCTGAGAaGGAGGGAGGC	9993	+1 a	5.11%
CAGTAGGG----------------GGC	3788	-16	1.94%
CAGT---------------AGGGAGGC	2366	-15	1.21%
CAGTAGGGGGCCT-----GAGGGAGGC	1952	-5	1.00%
CAGTAGGGGGCCT--GAGGAGGGAGGC	1566	-2	0.80%
CAGTAGGGGGCCTGAG-GGAGGGAGGC	1420	-1	0.73%
CAGTAGGGGGCCTGA---GAGGGAGGC	1302	-3	0.67%
CAGTA----------------GGAGGC	1196	-16	0.61%
C---------------AGGAGGGAGGC	815	-15	0.42%

Total | INDEL	48h	Ins	Del	HDR
70168 | 24.88%		7.03%	17.85%	0.00%

Typical seqs	Reads	Type	pct.
CAGTAGGGGGCCTGAGA|GGAGGGAGGC	52711	WT	75.12%
CAGTAGGGGGCCTGAGAaGGAGGGAGGC	3802	+1 a	5.42%
CAGTAGGG----------------GGC	1786	-16	2.55%
CAGT---------------AGGGAGGC	1161	-15	1.65%
CAGTAGGGGGCCT-----GAGGGAGGC	1082	-5	1.54%
CAGTAGGGGGCCTGA---GAGGGAGGC	678	-3	0.97%
CAGTAGGGGGCCTGAG-GGAGGGAGGC	649	-1	0.92%
CAGTA----------------GGAGGC	652	-16	0.93%
CAGTAGGGGGCCT--GAGGAGGGAGGC	592	-2	0.84%
CAGTAGGGGGCCT----GGAGGGAGGC	420	-4	0.60%

P41-P50-WT-U937-Syn60crMYH6b-AAV-KI-BC12

4h — Total | INDEL: 141601 | 2.60%; Ins 1.59%; Del 0.76%; HDR 0.25%

Typical seqs	Reads	Type	pct.
CAGTAGGGGGCCTGAGA|GGAGGGAGGC	137922	WT	97.40%
CAGTAGGGGGCCTGAGAaGGAGGGAGGC	1704	+1 a	1.20%
CAGTAGGGGGCCTGAGAgacgcgtagtttaaacCAGAGCTGCCAGAGCCAAGG	338	-9 HDR	0.24%
CAGTAGGGGGCCTGAG-GGAGGGAGGC	168	-1	0.12%
CAGTAGGGGGCCT--GAGGAGGGAGGC	136	-2	0.10%
CAGTAGGGGGCCTGAGAGGA-GGAGGC	101	-1	0.07%
CAGTAGGGGGCCTGAGAgGGAGGGAGGC	93	+1 g	0.07%
CAGTAGGGGGCCTGAGA-GAGGGAGGC	90	-1	0.06%
CAGTAGGGGGCCTGAGAtGGAGGGAGGC	60	+1 t	0.04%
CAGTAGGGGGCCTGA---GAGGGAGGC	60	-3	0.04%

8h — Total | INDEL: 151753 | 5.55%; Ins 2.64%; Del 1.51%; HDR 1.39%

Typical seqs	Reads	Type	pct.
CAGTAGGGGGCCTGAGA|GGAGGGAGGC	143333	WT	94.45%
CAGTAGGGGGCCTGAGAaGGAGGGAGGC	3256	+1 a	2.15%
CAGTAGGGGGCCTGAGAgacgcgtagtttaaacCAGAGCTGCCAGAGCCAAGG	1951	-9 HDR	1.29%
CAGTAGGGGGCCT--GAGGAGGGAGGC	508	-2	0.33%
CAGTAGGGGGCCTGAG-GGAGGGAGGC	327	-1	0.22%
CAGTAGGGGGCCTGAGAtGGAGGGAGGC	117	+1 t	0.08%
CAGTAGGGGGCCTGAGAGGA-GGAGGC	87	-1	0.06%
CAGTAGGGGGCCTGA---GAGGGAGGC	91	-3	0.06%
CAGTAGGGGGCCTGAGAgGGAGGGAGGC	68	+1 g	0.04%
CAGTAGGGGGCCT----GGAGGGAGGC	67	-4	0.04%

12h — Total | INDEL: 5756 | 7.92%; Ins 2.36%; Del 2.14%; HDR 3.42%

Typical seqs	Reads	Type	pct.
CAGTAGGGGGCCTGAGA|GGAGGGAGGC	5300	WT	92.08%
CAGTAGGGGGCCTGAGAgacgcgtagtttaaacCAGAGCTGCCAGAGCCAAGG	191	-9 HDR	3.32%
CAGTAGGGGGCCTGAGAaGGAGGGAGGC	126	+1 a	2.19%
CAGTAGGGGGCCTGAG-GGAGGGAGGC	23	-1	0.40%
CAGTAGGGGGCCT--GAGGAGGGAGGC	20	-2	0.35%
CAGTAGGGGGCCT-----GAGGGAGGC	6	-5	0.10%
CAGTAGGGGGCCTGA-AGGAGGGAGGC	5	-1	0.09%
CAGTAGGGGGCCTGAGAGGCGCGTAG---	5	-9	0.09%
CAGTAGGGGGCCTGA---GAGGGAGGC	5	-3	0.09%
CAGTAGGGGGCCT-----GAGGGAGGC	4	-5	0.07%

24h — Total | INDEL: 142293 | 25.00%; Ins 5.65%; Del 6.94%; HDR 12.41%

Typical seqs	Reads	Type	pct.
CAGTAGGGGGCCTGAGA|GGAGGGAGGC	106723	WT	75.00%
CAGTAGGGGGCCTGAGAgacgcgtagtttaaacCAGAGCTGCCAGAGCCAAGG	16250	-9 HDR	11.42%
CAGTAGGGGGCCTGAGAaGGAGGGAGGC	6247	+1 a	4.39%
CAGTAGGGGGCCTGAG-GGAGGGAGGC	1059	-1	0.74%
CAGTAGGGGGCCT--GAGGAGGGAGGC	628	-2	0.44%
CAGT---------------AGGGAGGC	617	-15	0.43%
CAGTAGGGGGCCT-----GAGGGAGGC	603	-5	0.42%
CAGTAGGGGGCCTGA---GAGGGAGGC	483	-3	0.34%
CAGTAGGG----------------GGC	451	-16	0.32%
CAGTAGGGGGCCT----GGAGGGAGGC	316	-4	0.22%

48h — Total | INDEL: 49920 | 32.37%; Ins 6.74%; Del 10.32%; HDR 15.31%

Typical seqs	Reads	Type	pct.
CAGTAGGGGGCCTGAGA|GGAGGGAGGC	33761	WT	67.63%
CAGTAGGGGGCCTGAGAgacgcgtagtttaaacCAGAGCTGCCAGAGCCAAGG	7014	-9 HDR	14.05%
CAGTAGGGGGCCTGAGAaGGAGGGAGGC	2607	+1 a	5.22%
CAGTAGGGGGCCTGAG-GGAGGGAGGC	522	-1	1.05%
CAGTAGGGGGCCT-----GAGGGAGGC	456	-5	0.91%
CAGTAGGG----------------GGC	369	-16	0.74%
CAGTAGGGGGCCT--GAGGAGGGAGGC	348	-2	0.70%
CAGT---------------AGGGAGGC	286	-15	0.57%
CAGTAGGGGGCCTGA---GAGGGAGGC	273	-3	0.55%
CAGTAGGGGGCCT----GGAGGGAGGC	174	-4	0.35%

P41-P50-WT-U937-Syn61crMYH6c-KO-BC13

4h

Total	INDEL	Ins	Del	HDR
298668	1.75%	0.68%	1.07%	0.00%

Typical seqs	Reads	Type	pct.
AGCTCTGATACAGGGGC|TGGGGGCCTC	293436	WT	98.25%
AGCTCTGATACAGGGGCcTGGGGGCCTC	1743	+1 c	0.58%
AGCTCTGATACAGGGGCT-GGGGCCTC	1543	-1	0.52%
AGCTCTGATACA-GGGCTGGGGGCCTC	175	-1	0.06%
AGCTCTGATACAGGGGC-GGGGGCCTC	149	-1	0.05%
AGCTCTGATA------CTGGGGGCCTC	98	-6	0.03%
AGCTCTGATACA-------GGGGCCTC	74	-7	0.02%
AGCTCTGATAC-GGGGCTGGGGGCCTC	71	-1	0.02%
AG--------------------------	110	-26	0.04%

8h

Total	INDEL	Ins	Del	HDR
443795	2.68%	1.16%	1.52%	0.00%

Typical seqs	Reads	Type	pct.
AGCTCTGATACAGGGGC|TGGGGGCCTC	431891	WT	97.32%
AGCTCTGATACAGGGGCcTGGGGGCCTC	4401	+1 c	0.99%
AGCTCTGATACAGGGGCT-GGGGCCTC	2634	-1	0.59%
AGCTCTGATACA-------GGGGCCTC	646	-7	0.15%
AGCTCTGATACAGGGGC-GGGGGCCTC	387	-1	0.09%
AGCTCTGATACA-GGGCTGGGGGCCTC	285	-1	0.06%
AGCTCTGATACA------GGGGGCCTC	273	-6	0.06%
AGCTCTGATACAGGGG-TGGGGGCCTC	234	-1	0.05%
AGCTCTGATACAGGGGCtTGGGGGCCTC	177	+1 t	0.04%
AGCTCTGATAC-GGGGCTGGGGGCCTC	100	-1	0.02%

12h

Total	INDEL	Ins	Del	HDR
223228	3.18%	1.40%	1.77%	0.00%

Typical seqs	Reads	Type	pct.
AGCTCTGATACAGGGGC|TGGGGGCCTC	216139	WT	96.82%
AGCTCTGATACAGGGGCcTGGGGGCCTC	2491	+1 c	1.12%
AGCTCTGATACAGGGGCT-GGGGCCTC	1141	-1	0.51%
AGCTCTGATACA-------GGGGCCTC	432	-7	0.19%
AGCTCTGATACAGGGGC-GGGGGCCTC	323	-1	0.14%
AGCTCTGATACA------GGGGGCCTC	223	-6	0.10%
AGCTCTGATACAGGGGCtTGGGGGCCTC	195	+1 t	0.09%
AGCTCTGATACA-GGGCTGGGGGCCTC	156	-1	0.07%
AGCTCTGATACAG-----GGGGGCCTC	95	-5	0.04%
AGCTCTGATACA--------GGGCCTC	87	-8	0.04%

24h

Total	INDEL	Ins	Del	HDR
288712	6.70%	1.71%	4.99%	0.00%

Typical seqs	Reads	Type	pct.
AGCTCTGATACAGGGGC|TGGGGGCCTC	269359	WT	93.30%
AGCTCTGATACAGGGGCcTGGGGGCCTC	3750	+1 c	1.30%
AGCTCTGATACA-------GGGGCCTC	3137	-7	1.09%
AGCTCTGATACA------GGGGGCCTC	1419	-6	0.49%
AGCTCTGATACAGGGGCT-GGGGCCTC	1287	-1	0.45%
AGCTCTGATACAGGGGC-GGGGGCCTC	543	-1	0.19%
AGCTCTGATACA---------GGCCTC	524	-9	0.18%
AGCTCTGATACA--------GGGCCTC	455	-8	0.16%
AG----------------------CTC	355	-22	0.12%

48h

Total	INDEL	Ins	Del	HDR
299424	8.15%	2.08%	6.07%	0.00%

Typical seqs	Reads	Type	pct.
AGCTCTGATACAGGGGC|TGGGGGCCTC	275010	WT	91.85%
AGCTCTGATACAGGGGCcTGGGGGCCTC	4785	+1 c	1.60%
AGCTCTGATACA-------GGGGCCTC	4244	-7	1.42%
AGCTCTGATACA------GGGGGCCTC	2255	-6	0.75%
AGCTCTGATACAGGGGCT-GGGGCCTC	1137	-1	0.38%
AGCTCTGATACA--------GGGCCTC	742	-8	0.25%
AGCTCTGATACAGGGGC-GGGGGCCTC	623	-1	0.21%
AGCTCTGATACA---------GGCCTC	623	-9	0.21%
AGCTCTGATACAG-----GGGGGCCTC	539	-5	0.18%
AGCTCTGATACAGGGGCtTGGGGGCCTC	401	+1 t	0.13%

P41-P50-WT-U937-Syn61crMYH6c-AAV-KI-BC13

4h — Total 301697 | INDEL 2.65% | Ins 1.25% | Del 1.15% | HDR 0.25%

Typical seqs	Reads	Type	pct.
AGCTCTGATACAGGGGC|TGGGGGCCTC	293706	WT	97.35%
AGCTCTGATACAGGGGCcTGGGGGCCTC	3148	+1 c	1.04%
AGCTCTGATACAGGGGCT-GGGGCCTC	2164	-1	0.72%
AGCTCTGgtttaaactacgcgtcTCTCAGG CCCCCTACTGCC	719	-9 HDR	0.24%
AGCTCTGATACAGGGGC-GGGGGCCTC	268	-1	0.09%
AGCTCTGATACA-GGGCTGGGGGCCTC	262	-1	0.09%
AGCTCTGATAC-GGGGCTGGGGGCCTC	105	-1	0.03%
AGCTCTGATACAGGGGCgTGGGGGCCTC	81	+1 g	0.03%
AGCTCTGATACAGGGGGCTGGGGGCCTC	68	+1 Ins	0.02%
AGCTCTGATACAGGGGCtTGGGGGCCTC	61	+1 t	0.02%

8h — Total 344821 | INDEL 4.27% | Ins 1.37% | Del 1.60% | HDR 1.30%

Typical seqs	Reads	Type	pct.
AGCTCTGATACAGGGGC|TGGGGGCCTC	330098	WT	95.73%
AGCTCTGgtttaaactacgcgtcTCTCAGG CCCCCTACTGCC	4264	-9 HDR	1.24%
AGCTCTGATACAGGGGCcTGGGGGCCTC	3521	+1 c	1.02%
AGCTCTGATACAGGGGCT-GGGGCCTC	2707	-1	0.79%
AGCTCTGATACA-GGGCTGGGGGCCTC	392	-1	0.11%
AGCTCTGATACAGGGGC-GGGGGCCTC	291	-1	0.08%
AGCTCTGATACAGGGGCtTGGGGGCCTC	237	+1 t	0.07%
AGCTCTGATACAGGGGCaTGGGGGCCTC	175	+1 a	0.05%
AGCTCTGATACA-------GGGGCCTC	151	-7	0.04%
AGCTCTGATACA------GGGGGCCTC	119	-6	0.03%

12h — Total 35996 | INDEL 5.98% | Ins 1.47% | Del 1.95% | HDR 2.56%

Typical seqs	Reads	Type	pct.
AGCTCTGATACAGGGGC|TGGGGGCCTC	33842	WT	94.02%
AGCTCTGgtttaaactacgcgtcTCTCAGG CCCCCTACTGCC	897	-9 HDR	2.49%
AGCTCTGATACAGGGGCcTGGGGGCCTC	390	+1 c	1.08%
AGCTCTGATACAGGGGCT-GGGGCCTC	232	-1	0.64%
AGCTCTGATACA-------GGGGCCTC	56	-7	0.16%
AGCTCTGATACA-GGGCTGGGGGCCTC	43	-1	0.12%
AGCTCTGATACAGGGGCtTGGGGGCCTC	29	+1 t	0.08%
AGCTCTGATACAGGGGC-GGGGGCCTC	29	-1	0.08%
AGCTCTGATACA------GGGGGCCTC	29	-6	0.08%
AGCTCTGATACAGGGG-TGGGGGCCTC	20	-1	0.06%

24h — Total 114073 | INDEL 11.35% | Ins 1.92% | Del 3.57% | HDR 5.86%

Typical seqs	Reads	Type	pct.
AGCTCTGATACAGGGGC|TGGGGGCCTC	101121	WT	88.65%
AGCTCTGgtttaaactacgcgtcTCTCAGG CCCCCTACTGCC	6375	-9 HDR	5.59%
AGCTCTGATACAGGGGCcTGGGGGCCTC	1672	+1 c	1.47%
AGCTCTGATACAGGGGCT-GGGGCCTC	719	-1	0.63%
AGCTCTGATACA-------GGGGCCTC	543	-7	0.48%
AGCTCTGATACAGGGGC-GGGGGCCTC	317	-1	0.28%
AGCTCTGATACA------GGGGGCCTC	150	-6	0.13%
AGCTCTGATACA-GGGCTGGGGGCCTC	127	-1	0.11%
AGCTCTGATACAGGGGCtTGGGGGCCTC	95	+1 t	0.08%
AGCTCTGATACAGGGGCaTGGGGGCCTC	82	+1 a	0.07%

48h — Total 143589 | INDEL 11.69% | Ins 2.29% | Del 4.56% | HDR 4.84%

Typical seqs	Reads	Type	pct.
AGCTCTGATACAGGGGC|TGGGGGCCTC	126810	WT	88.31%
AGCTCTGgtttaaactacgcgtcTCTCAGG CCCCCTACTGCC	6606	-9 HDR	4.60%
AGCTCTGATACAGGGGCcTGGGGGCCTC	2519	+1 c	1.75%
AGCTCTGATACA-------GGGGCCTC	1110	-7	0.77%
AGCTCTGATACAGGGGCT-GGGGCCTC	1024	-1	0.71%
AGCTCTGATACA------GGGGGCCTC	569	-6	0.40%
AGCTCTGATACAGGGGC-GGGGGCCTC	240	-1	0.17%
AGCTCTGATACAG-----GGGGGCCTC	178	-5	0.12%
AGCTCTGATACAGGGGCtTGGGGGCCTC	162	+1 t	0.11%
AGCTCTGATACA--------GGGCCTC	159	-8	0.11%

P41-P50-WT-U937-Syn62crMYH6d-AAV-KI-BC14

4h

Total | INDEL	Ins	Del	HDR
187529 | 0.43%	**0.05%**	**0.28%**	**0.10%**

Typical seqs	Reads	Type	pct.
CAGCTCTGATACAGGGG|CTGGGGGCCT	186719	WT	99.57%
CAGCTCTGgtttaaactacgcgtcTCTCAG GCCCCCTACTGCC	178	-9 HDR	0.09%
CAGCTCTGATACAGGG-CTGGGGGCCT	113	-1	0.06%
CAGCTCTGATACAGGGG--GGGGGCCT	69	-2	0.04%
CAGCTCTGATAC-GGGGCTGGGGGCCT	66	-1	0.04%
CAGCTCTGATACA----CTGGGGGCCT	41	-4	0.02%
CAGCTCTGATAC-----CTGGGGGCCT	33	-5	0.02%
CAGCTCTGATACAGGGGCT--GGGCCT	32	-2	0.02%
CAGCTCTGATACAG---CTGGGGGCCT	30	-3	0.02%
CAGCTCTGATACAGGGGgCTGGGGGCCT	32	+1 g	0.02%

8h

Total | INDEL	Ins	Del	HDR
393212 | 0.96%	**0.09%**	**0.36%**	**0.50%**

Typical seqs	Reads	Type	pct.
CAGCTCTGATACAGGGG|CTGGGGGCCT	389443	WT	99.04%
CAGCTCTGgtttaaactacgcgtcTCTCAG GCCCCCTACTGCC	1910	-9 HDR	0.49%
CAGCTCTGATACAGGG-CTGGGGGCCT	406	-1	0.10%
CAGCTCTGATACAGGGGgCTGGGGGCCT	120	+1 g	0.03%
CAGCTCTGATACAGGGGcCTGGGGGCCT	90	+1 c	0.02%
CAGCTCTGATAC-GGGGCTGGGGGCCT	89	-1	0.02%
CAGCTCTGATAAA----CTGGGGGCCT	64	-4	0.02%
CAGCTCTGATACAGGGGGGGCTGGGGGCCT	60	+3	0.02%
CAGCTCTGATACAGG----GGGGGCCT	49	-4	0.01%
CAGCTCTGATACAG---CTGGGGGCCT	48	-3	0.01%

12h

Total | INDEL	Ins	Del	HDR
58405 | 1.88%	**0.12%**	**0.50%**	**1.25%**

Typical seqs	Reads	Type	pct.
CAGCTCTGATACAGGGG|CTGGGGGCCT	57307	WT	98.12%
CAGCTCTGgtttaaactacgcgtcTCTCAG GCCCCCTACTGCC	706	-9 HDR	1.21%
CAGCTCTGATACAGGG-CTGGGGGCCT	67	-1	0.11%
CAGCTCTGATACAGGGGcCTGGGGGCCT	35	+1 c	0.06%
CAGCTCTGATAC-GGGGCTGGGGGCCT	24	-1	0.04%
CAGCTCTGATACAGGGGgCTGGGGGCCT	19	+1 g	0.03%
CAGCTCTGAT--AGGGGCTGGGGGCCT	18	-2	0.03%
CAGCTCTGATACA----CTGGGGGCCT	12	-4	0.02%
CAGCTCTGATA------CTGGGGGCCT	9	-6	0.02%
CAGCTCTGATACAGGGGC-GGGGGCCT	8	-1	0.01%

24h

Total | INDEL	Ins	Del	HDR
361138 | 2.10%	**0.11%**	**0.61%**	**1.38%**

Typical seqs	Reads	Type	pct.
CAGCTCTGATACAGGGG|CTGGGGGCCT	353561	WT	97.90%
CAGCTCTGgtttaaactacgcgtcTCTCAG GCCCCCTACTGCC	4773	-9 HDR	1.32%
CAGCTCTGATACAGGG-CTGGGGGCCT	313	-1	0.09%
CAGCTCTGATACAGGGGcCTGGGGGCCT	156	+1 c	0.04%
CAGCTCTGATACAGGGGgCTGGGGGCCT	124	+1 g	0.03%
CAGCTCTGATACA-------GGGGCCT	90	-7	0.02%
CAGCTCTGATAC-GGGGCTGGGGGCCT	86	-1	0.02%
CAGCTCTGATACAG-----GGGGGCCT	81	-5	0.02%
CAGCTCTGATACAGGGG--GGGGGCCT	54	-2	0.01%
CAGCTCTGATA------CTGGGGGCCT	52	-6	0.01%

48h

Total | INDEL	Ins	Del	HDR
131531 | 1.90%	**0.11%**	**0.76%**	**1.03%**

Typical seqs	Reads	Type	pct.
CAGCTCTGATACAGGGG|CTGGGGGCCT	129032	WT	98.10%
CAGCTCTGgtttaaactacgcgtcTCTCAG GCCCCCTACTGCC	1304	-9 HDR	0.99%
CAGCTCTGATACAGGG-CTGGGGGCCT	136	-1	0.10%
CAGCTCTGATACAGGGGcCTGGGGGCCT	74	+1 c	0.06%
CAGCTCTGATACA----CTGGGGGCCT	46	-4	0.03%
CAGCTCTGATA------CTGGGGGCCT	38	-6	0.03%
CAGCTCTGATACA------GGGGGCCT	36	-6	0.03%
CAGCTCTGATACAGGGGgCTGGGGGCCT	33	+1 g	0.03%
CAGCTCTGATACA-------GGGGCCT	28	-7	0.02%
CAGCTCTGATACAGG--CTGGGGGCCT	28	-2	0.02%

P41-P50-WT-U937-Syn63crMYH6e-KO-BC15

4h

Total	INDEL	Ins	Del	HDR
59220	0.45%	**0.05%**	**0.40%**	**0.00%**

Typical seqs	Reads	Type	pct.
GCAGCTCTGATACAGGGGCT**GGG**GGCC	58953	WT	99.55%
GCAGCTCTGATACAGG-GCTGGGGGCC	69	-1	0.12%
GCAGCTCTGATAC-GGGGCTGGGGGCC	16	-1	0.03%
GCAGCTCTGATACA---GCTGGGGGCC	15	-3	0.03%
GCAGCTCTGATACAGGGgGCTGGGGGCC	13	+1 g	0.02%
GCAGCTCTGATACAG--GCTGGGGGCC	10	-2	0.02%
GCAGCTCTGATACA---------GGCC	8	-9	0.01%
GCAGCTCTGATAC--CCGCTGGGGGCC	8	-2	0.01%
GCAGCTCTGATACAG-----GGGGGCC	8	-5	0.01%

8h

Total	INDEL	Ins	Del	HDR
144436	0.47%	**0.07%**	**0.40%**	**0.00%**

Typical seqs	Reads	Type	pct.
GCAGCTCTGATACAGGGGCT**GGG**GGCC	143753	WT	99.53%
GCAGCTCTGATACAGG-GCTGGGGGCC	169	-1	0.12%
GCAGCTCTGATACAGGGgGCTGGGGGCC	49	+1 g	0.03%
GCAGCTCTGATACAG--GCTGGGGGCC	44	-2	0.03%
GCAGCTCTGATACAGGGgCCTGGGGGCC	30	+1 g	0.02%
GCAGCTCTGATAC-GGGGCTGGGGGCC	27	-1	0.02%
GCA-------------GGCTGGGGGCC	23	-13	0.02%
GCAGCTCTGAT-CAGGGGCTGGGGGCC	19	-1	0.01%
GCAGCTCTGATAC----GCTGGGGGCC	18	-4	0.01%
GCA--------------GCTGGGGGCC	17	-14	0.01%

12h

Total	INDEL	Ins	Del	HDR
130488	0.58%	**0.10%**	**0.47%**	**0.00%**

Typical seqs	Reads	Type	pct.
GCAGCTCTGATACAGGGGCT**GGG**GGCC	129736	WT	99.42%
GCAGCTCTGATACAGG-GCTGGGGGCC	151	-1	0.12%
GCAGCTCTGATACAG--GCTGGGGGCC	83	-2	0.06%
GCAGCTCTGATACAGGGgCCTGGGGGCC	34	+1 g	0.03%
GCAGCTCTGATAC-GGGGCTGGGGGCC	23	-1	0.02%
GCAGCTCTGAT-CAGGGGCTGGGGGCC	21	-1	0.02%
GCAGCTCTGATACA------GGGGGCC	19	-6	0.01%
GCAGCTCTGATACAGGGgGCTGGGGGCC	19	+1 g	0.01%
GCAGCTCTGAT--AGGGGCTGGGGGCC	19	-2	0.01%
GCAGCTCTGATAC----GCTGGGGGCC	18	-4	0.01%

24h

Total	INDEL	Ins	Del	HDR
184680	1.05%	**0.09%**	**0.95%**	**0.00%**

Typical seqs	Reads	Type	pct.
GCAGCTCTGATACAGGGGCT**GGG**GGCC	182745	WT	98.95%
GCAGCTCTGATACAGG-GCTGGGGGCC	209	-1	0.11%
GCAGCTCTGATACA------GGGGGCC	93	-6	0.05%
GCAGCTCTGATACAG--GCTGGGGGCC	68	-2	0.04%
GCAG----------------------C	56	-22	0.03%
GCAGCTCTGATACAGGGgGCTGGGGGCC	53	+1 g	0.03%
GCAGCTCTGATAC----GCTGGGGGCC	50	-4	0.03%
GCA---------------------GCC	42	-21	0.02%
GCAGCTCTGATACA-------GGGGCC	40	-7	0.02%

48h

Total	INDEL	Ins	Del	HDR
168751	1.70%	**0.11%**	**1.59%**	**0.00%**

Typical seqs	Reads	Type	pct.
GCAGCTCTGATACAGGGGCT**GGG**GGCC	165887	WT	98.30%
GCAGCTCTGATACAGG-GCTGGGGGCC	156	-1	0.09%
GCAGCTCTGATACAG--GCTGGGGGCC	85	-2	0.05%
GCAGCT------------CTGGGGGCC	71	-12	0.04%
GCAGCTCTGATACA------GGGGGCC	72	-6	0.04%
GCAGCTCTGATAC----GCTGGGGGCC	59	-4	0.03%
GCA---------------------GCC	57	-21	0.03%
GCAG----------------------C	63	-22	0.04%
GCAGCTCTGATACAGGGgCCTGGGGGCC	48	+1 g	0.03%

P41-P50-WT-U937-Syn63crMYH6e-AAV-KI-BC15

4h

Total | INDEL	Ins	Del	HDR
75145 | 0.58%	**0.06%**	**0.40%**	**0.13%**

Typical seqs	Reads	Type	pct.
GCAGCTCTGATACAGGG|GCT**GGG**GGCC	74708	WT	99.42%
GCAGCTCTGgtttaaactacgcgtcTCTCA GGCCCCCTACTGCC	94	-9 HDR	0.13%
GCAGCTCTGATACAGG-GCTGGGGGCC	93	-1	0.12%
GCAGCTCTGATACAG--GCTGGGGGCC	40	-2	0.05%
GCAGCTCTGATAC-GGGGCTGGGGGCC	23	-1	0.03%
GCAGCTCTGATACA------GGGGGCC	14	-6	0.02%
GCAGCTCTGATACA---------GGCC	12	-9	0.02%
GCAGCTCTGATACA---GCTGGGGGCC	12	-3	0.02%
GCAGCTCTGATAC---CGCTGGGGGCC	13	-3	0.02%
GCAGCTCTGATACAGGGgCCTGGGGGCC	11	+1 g	0.01%

8h

Total | INDEL	Ins	Del	HDR
133660 | 1.31%	**0.12%**	**0.50%**	**0.69%**

Typical seqs	Reads	Type	pct.
GCAGCTCTGATACAGGG|GCT**GGG**GGCC	131905	WT	98.69%
GCAGCTCTGgtttaaactacgcgtcTCTCA GGCCCCCTACTGCC	882	-9 HDR	0.66%
GCAGCTCTGATACAGG-GCTGGGGGCC	234	-1	0.18%
GCAGCTCTGATACAGGGgGCTGGGGGCC	84	+1 g	0.06%
GCAGCTCTGATACAG--GCTGGGGGCC	68	-2	0.05%
GCAGCTCTGATAC-GGGGCTGGGGGCC	50	-1	0.04%
GCAGCTCTGATACAGGGgCCTGGGGGCC	34	+1 g	0.03%
GCAGCTCTGAT--AGGGGCTGGGGGCC	22	-2	0.02%
GCAGCTCTGATAC----GCTGGGGGCC	19	-4	0.01%
GCAGCTCTGAT-CAGGGGCTGGGGGCC	19	-1	0.01%

12h

Total | INDEL	Ins	Del	HDR
133982 | 1.84%	**0.14%**	**0.67%**	**1.04%**

Typical seqs	Reads	Type	pct.
GCAGCTCTGATACAGGG|GCT**GGG**GGCC	131516	WT	98.16%
GCAGCTCTGgtttaaactacgcgtcTCTCA GGCCCCCTACTGCC	1325	-9 HDR	0.99%
GCAGCTCTGATACAGG-GCTGGGGGCC	165	-1	0.12%
GCAGCTCTGATACAG--GCTGGGGGCC	99	-2	0.07%
GCAGCTCTGATACAGGGgCCTGGGGGCC	71	+1 g	0.05%
GCAGCTCTGATACAGGGgGCTGGGGGCC	38	+1 g	0.03%
GCAGCTCTGATAC-GGGGCTGGGGGCC	35	-1	0.03%
GCAGCTCTGAT-CAGGGGCTGGGGGCC	33	-1	0.02%
GCAGCTCTGAT--AGGGGCTGGGGGCC	32	-2	0.02%
GCAGCT---------------------	26	-24	0.02%

24h

Total | INDEL	Ins	Del	HDR
5460 | 3.21%	**0.11%**	**0.59%**	**2.51%**

Typical seqs	Reads	Type	pct.
GCAGCTCTGATACAGGG|GCT**GGG**GGCC	5285	WT	96.79%
GCAGCTCTGgtttaaactacgcgtcTCTCA GGCCCCCTACTGCC	133	-9 HDR	2.44%
GCAGCTCTGATACAGG-GCTGGGGGCC	6	-1	0.11%
GCAGCTCTGAT-CAGGGGCTGGGGGCC	4	-1	0.07%
GCAGCTCTGATACAGGGgCCTGGGGGCC	4	+1 g	0.07%
GCA---------------------GCC	4	-21	0.07%
GCAGCTCTGAT------GCTGGGGGCC	3	-6	0.05%
GCAGCTCTGATACAGGGGC-GGGGGCC	3	-1	0.05%
GCAGCTCTGATACAG--GCTGGGGGCC	2	-2	0.04%
GCAGCTCTGATAC----GCTGGGGGCC	2	-4	0.04%

48h

Total | INDEL	Ins	Del	HDR
87593 | 1.86%	**0.14%**	**0.72%**	**1.00%**

Typical seqs	Reads	Type	pct.
GCAGCTCTGATACAGGG|GCT**GGG**GGCC	85960	WT	98.14%
GCAGCTCTGgtttaaactacgcgtcTCTCA GGCCCCCTACTGCC	836	-25+16 -9 HDR	0.95%
GCAGCTCTGATACAGG-GCTGGGGGCC	122	-1	0.14%
GCAGCTCTGATACAGGGgCCTGGGGGCC	42	+1 g	0.05%
GCAGCTCTGATACAGGGgGCTGGGGGCC	35	+1 g	0.04%
GCAGCTCTGAT-CAGGGGCTGGGGGCC	26	-1	0.03%
GCAGCTCTGAT------GCTGGGGGCC	23	-6	0.03%
GCAGCTCTGATAC-GGGGCTGGGGGCC	23	-1	0.03%
GCAGCTCTGATACAG--GCTGGGGGCC	19	-2	0.02%
GCAGCTCTGATAC----GCTGGGGGCC	22	-4	0.03%

P41-P50-WT-U937-Syn64crMYH6f-KO-BC16

Total | INDEL	4h	Ins	Del	HDR
9717 | 0.26%		0.07%	0.19%	0.00%

Typical seqs	Reads	Type	pct.
GGCAGCTCTGATACAGG|GGC**TGG**GGGC	9692	WT	99.74%
GGCAGCTCTGATACAG-GGCTGGGGGC	7	-1	0.07%
GGCAGCTCTGATACA--GGCTGGGGGC	5	-2	0.05%
GGCAGCTCTGATAACAGGGGCTGGGGGC	3	+1 Ins	0.03%
GGCAGCTCTGATACAGGtGGCTGGGGGC	2	+1 t	0.02%
GGCAGCTCTGATA----GGCTGGGGGC	2	-4	0.02%
GGCAGCTCTGAT--AGGGGCTGGGGGC	2	-2	0.02%
GGCAGCTCTG--AGAGG----AGGGGC	2	-6	0.02%
GGCAGCTCTGATACAGGgGGCTGGGGGC	2	+1 g	0.02%
Total | INDEL	Ins	Del	HDR

Total | INDEL	8h	Ins	Del	HDR
223137 | 0.48%		0.12%	0.36%	0.00%

Typical seqs	Reads	Type	pct.
GGCAGCTCTGATACAGG|GGC**TGG**GGGC	222073	WT	99.52%
GGCAGCTCTGATACA--GGCTGGGGGC	209	-2	0.09%
GGCAGCTCTGATACAG-GGCTGGGGGC	165	-1	0.07%
GGCAGCTCTGATAACAGGGGCTGGGGGC	80	+1 Ins	0.04%
GGCAGCTCTGATACAGGgGGCTGGGGGC	70	+1 g	0.03%
GGCAGCTCTGATACAGGaGGCTGGGGGC	47	+1 a	0.02%
GGCAGCTCTGA------GGCTGGGGGC	36	-6	0.02%
GGCAGCTCTGATACAGGgGCCTGGGGGC	31	+1 g	0.01%
GGCAGCTCTGATAC-GGGGCTGGGGGC	27	-1	0.01%
GGCAGCTCTGATA----GGCTGGGGGC	21	-4	0.01%

Total | INDEL	12h	Ins	Del	HDR
53217 | 0.78%		0.16%	0.63%	0.00%

Typical seqs	Reads	Type	pct.
GGCAGCTCTGATACAGG|GGC**TGG**GGGC	52800	WT	99.22%
GGCAGCTCTGATACAG-GGCTGGGGGC	87	-1	0.16%
GGCAGCTCTGATACA--GGCTGGGGGC	62	-2	0.12%
GGCAGCTCTGATAACAGGGGCTGGGGGC	28	+1 Ins	0.05%
GGCAGCTCTGATACAGGgGCCTGGGGGC	22	+1 g	0.04%
GGCAGCTCTGATACAGGaGGCTGGGGGC	13	+1 a	0.02%
GGCAGCTCTGATAC-GGGGCTGGGGGC	10	-1	0.02%
GGCAGCTCTGATACAGGgGGCTGGGGGC	9	+1 g	0.02%
GGCAGCTCTGATACA-------GGGGC	9	-7	0.02%
GGCAG----------------------	10	-22	0.02%

Total | INDEL	24h	Ins	Del	HDR
1041 | 1.15%		0.38%	0.77%	0.00%

Typical seqs	Reads	Type	pct.
GGCAGCTCTGATACAGG|GGC**TGG**GGGC	1029	WT	98.85%
GGCAGCTCTGATAACAGGGGCTGGGGGC	4	+1 Ins	0.38%
GGCAGCTCTGATACA--GGCTGGGGGC	4	-2	0.38%
GGCAGCCC----CCAGGGGCTGGGGGC	2	-4	0.19%
------------------------GGC	2	-24	0.19%

Total | INDEL	48h	Ins	Del	HDR
22823 | 2.73%		0.45%	2.28%	0.00%

Typical seqs	Reads	Type	pct.
GGCAGCTCTGATACAGG|GGC**TGG**GGGC	22200	WT	97.27%
GGCAGCTCTGATACA--GGCTGGGGGC	103	-2	0.45%
GGCAGCTCTGATAaCAGGGGCTGGGGGC	51	+1 Ins	0.22%
GGCAGCTCTGATACAG-GGCTGGGGGC	38	-1	0.17%
GGCAG----------------------	30	-22	0.13%
GGCAGCTCTGATACA------GGGGGC	21	-6	0.09%
GGCAGCT------------CTGGGGGC	21	-12	0.09%
GGCAGCTCTGATACA---------GGC	13	-9	0.06%
GGCAGCTCTGATACAGGgGCCTGGGGGC	12	+1 g	0.05%
GGCAGCTCTGAT--AGGGGCTGGGGGC	12	-2	0.05%

P41-P50-WT-U937-Syn64crMYH6f-AAV-KI-BC16

Total | INDEL	4h	Ins	Del	HDR
48975 | 0.46%		**0.10%**	**0.24%**	**0.12%**

Typical seqs	Reads	Type	pct.
GGCAGCTCTGATACAGG|GGC**TGG**GGGC	48750	WT	99.54%
GGCAGCTCTGgtttaaactacgcgtcTCTC AGGCCCCCTACTGCC	55	-9 HDR	0.11%
GGCAGCTCTGATACAG-GGCTGGGGGC	35	-1	0.07%
GGCAGCTCTGATACA--GGCTGGGGGC	29	-2	0.06%
GGCAGCTCTGATACAGGgGGCTGGGGGC	22	+1 g	0.04%
GGCAGCTCTGAT-----GGCTGGGGGC	16	-5	0.03%
GGCAGCTCTGATAC-GGGGCTGGGGGC	15	-1	0.03%
GGCAGCTCTGATAACAGGGGCTGGGGGC	11	+1 Ins	0.02%
GGCAGCTCTGATACAGGgGCCTGGGGGC	7	+1 g	0.01%
GGCAGCTCTGATACAGGGGGCGGCTGGGGGC	5	+3	0.01%

Total | INDEL	8h	Ins	Del	HDR
135580 | 1.25%		**0.12%**	**0.51%**	**0.62%**

Typical seqs	Reads	Type	pct.
GGCAGCTCTGATACAGG|GGC**TGG**GGGC	133891	WT	98.75%
GGCAGCTCTGgtttaaactacgcgtcTCTC AGGCCCCCTACTGCC	810	-9 HDR	0.60%
GGCAGCTCTGATACAG-GGCTGGGGGC	193	-1	0.14%
GGCAGCTCTGATACA--GGCTGGGGGC	116	-2	0.09%
GGCAGCTCTGATAACAGGGGCTGGGGGC	64	+1 Ins	0.05%
GGCAGCTCTGATAC-GGGGCTGGGGGC	44	-1	0.03%
GGCAGCTCTGATACAGGgGGCTGGGGGC	38	+1 g	0.03%
GGCAGCTCTGATA----GGCTGGGGGC	40	-4	0.03%
GGCAGCTCTGATACAGGgGCCTGGGGGC	24	+1 g	0.02%
GGCAGCTCTGAT-CAGGGGCTGGGGGC	18	-1	0.01%

Total | INDEL	12h	Ins	Del	HDR
89794 | 1.75%		**0.19%**	**0.64%**	**0.93%**

Typical seqs	Reads	Type	pct.
GGCAGCTCTGATACAGG|GGC**TGG**GGGC	88220	WT	98.25%
GGCAGCTCTGgtttaaactacgcgtcTCTC AGGCCCCCTACTGCC	803	-9 HDR	0.89%
GGCAGCTCTGATACAG-GGCTGGGGGC	105	-1	0.12%
GGCAGCTCTGATACA--GGCTGGGGGC	92	-2	0.10%
GGCAGCTCTGATAACAGGGGCTGGGGGC	58	+1 Ins	0.06%
GGCAGCTCTGATACAGGgGGCTGGGGGC	32	+1 g	0.04%
GGCAGCTCTGATACAGGgGCCTGGGGGC	31	+1 g	0.03%
GGCAGCTCTGATAC-GGGGCTGGGGGC	29	-1	0.03%
GGCAGCTCTGAT-CAGGGGCTGGGGGC	25	-1	0.03%
GGCAGCTCTGATA----GGCTGGGGGC	22	-4	0.02%

Total | INDEL	24h	Ins	Del	HDR
95807 | 3.69%		**0.28%**	**1.39%**	**2.02%**

Typical seqs	Reads	Type	pct.
GGCAGCTCTGATACAGG|GGC**TGG**GGGC	92267	WT	96.31%
GGCAGCTCTGgtttaaactacgcgtcTCTC AGGCCCCCTACTGCC	1858	-9 HDR	1.94%
GGCAGCTCTGATACA--GGCTGGGGGC	264	-2	0.28%
GGCAGCTCTGATACAG-GGCTGGGGGC	129	-1	0.13%
GGCAGCTCTGATAACAGGGGCTGGGGGC	96	+1 Ins	0.10%
-----------------GGCTGGGGGC	47	-17	0.05%
GGCAGCTCT--------GGCTGGGGGC	46	-8	0.05%
GGCAGCTCTGATACAGGgGGCTGGGGGC	46	+1 g	0.05%
GGCAGCTCTGATACA-------GGGGC	37	-7	0.04%
GGCAGCTCTGATACA------GGGGGC	37	-6	0.04%

Total | INDEL	48h	Ins	Del	HDR
34731 | 4.50%		**0.48%**	**1.86%**	**2.16%**

Typical seqs	Reads	Type	pct.
GGCAGCTCTGATACAGG|GGC**TGG**GGGC	33169	WT	95.50%
GGCAGCTCTGgtttaaactacgcgtcTCTC AGGCCCCCTACTGCC	723	-9 HDR	2.08%
GGCAGCTCTGATACA--GGCTGGGGGC	117	-2	0.34%
GGCAGCTCTGATACAG-GGCTGGGGGC	65	-1	0.19%
GGCAGCTCTGATAaCAGGGGCTGGGGGC	63	+1 Ins	0.18%
GGCAGCTCTGATACAGGgGCCTGGGGGC	20	+1 g	0.06%
GGCAGCTCTGATACA------GGGGGC	19	-6	0.05%
GGCAGCTCTGATACAGGgGGCTGGGGGC	19	+1 g	0.05%
GGCAG----------------------	34	-22	0.10%
GGCAGCTCT--------GGCTGGGGGC	17	-8	0.05%

P41-P50-WT-U937-Syn65crMYH6g-KO-BC17

Total | INDEL	4h	Ins	Del	HDR
109928 | 3.65%		1.43%	2.22%	0.00%
**Typical seqs**		**Reads**	**Type**	**pct.**
CTCTGGCAGCTCTGATA|CAG**GGG**CTGG		105913	WT	96.35%
CTCTGGCAGCTCTGATAaCAGGGGCTGG		1173	+1 a	1.07%
CTCTGGCAGCTCTGAT--AGGGGCTGG		415	-2	0.38%
CTCTGGCAGCTCTGAT-CAGGGGCTGG		208	-1	0.19%
CTCTGGCAGCTCTGA--CAGGGGCTGG		193	-2	0.18%
CTCTGGCAGCT------CAGGGGCTGG		98	-6	0.09%
CTCTGGCAGCTCTG----AGGGGCTGG		96	-4	0.09%
CTCTGGCAGCTCTGATATACAGGGGCTGG		102	+2	0.09%
CTCTGGCAGCTCTGATACA-GGGCTGG		93	-1	0.08%
CTCTGGCAGCTCTGATA-----GCTGG		70	-5	0.06%

Total | INDEL	8h	Ins	Del	HDR
221996 | 8.73%		3.31%	5.42%	0.00%
**Typical seqs**		**Reads**	**Type**	**pct.**
CTCTGGCAGCTCTGATA|CAG**GGG**CTGG		202610	WT	91.27%
CTCTGGCAGCTCTGATAaCAGGGGCTGG		5353	+1 a	2.41%
CTCTGGCAGCTCTGAT--AGGGGCTGG		1899	-2	0.86%
CTCTGGCAGCTCTGA--CAGGGGCTGG		1000	-2	0.45%
CTCTGGCAGCTCTGAT-CAGGGGCTGG		585	-1	0.26%
CTCTGGCAGCTCTGATACA-GGGCTGG		554	-1	0.25%
CTCTGGCAGCT------CAGGGGCTGG		422	-6	0.19%
CTCTGGCAGCTCT----CAGGGGCTGG		361	-4	0.16%
CTCTGGCAGCTCTGATA-AGGGGCTGG		312	-1	0.14%
CTCTGGCAGCTCTG----AGGGGCTGG		304	-4	0.14%

Total | INDEL	12h	Ins	Del	HDR
78783 | 14.42%		4.42%	10.00%	0.00%
**Typical seqs**		**Reads**	**Type**	**pct.**
CTCTGGCAGCTCTGATA|CAG**GGG**CTGG		67422	WT	85.58%
CTCTGGCAGCTCTGATAaCAGGGGCTGG		2533	+1 a	3.22%
CTCTGGCAGCTCTGAT--AGGGGCTGG		1081	-2	1.37%
CTCTGGCAGCTCTGA--CAGGGGCTGG		647	-2	0.82%
CTCTGGCAGCTCTGAT-CAGGGGCTGG		558	-1	0.71%
CTCTGGCAGCTCTGATACA-GGGCTGG		327	-1	0.42%
CTCTGGCAGCT------------CTGG		243	-12	0.31%
CTCTGGCA--------------------		283	-21	0.36%
CTCTGGCAGCTCTG----AGGGGCTGG		211	-4	0.27%
CTCTGG-----------CAGGGGCTGG		194	-11	0.25%

Total | INDEL	24h	Ins	Del	HDR
34939 | 34.93%		7.11%	27.82%	0.00%
**Typical seqs**		**Reads**	**Type**	**pct.**
CTCTGGCAGCTCTGATA|CAG**GGG**CTGG		22735	WT	65.07%
CTCTGGCAGCTCTGATAaCAGGGGCTGG		1675	+1 a	4.79%
CTCTGGCAG-------------------		854	-22	2.44%
CTCTGGCAGCTCTGAT--AGGGGCTGG		758	-2	2.17%
CTCTGGCA--------------------		644	-21	1.84%
CTCTGGCAGCT------------CTGG		564	-12	1.61%
CTCTGGCAGCTCTGAT-CAGGGGCTGG		423	-1	1.21%
CTCTGGCAGCTCTGA--CAGGGGCTGG		376	-2	1.08%
CTCTGGCAGCT------CAGGGGCTGG		279	-6	0.80%
CTCTGGCA--------------GCTGG		262	-14	0.75%

Total | INDEL	48h	Ins	Del	HDR
73731 | 48.72%		9.55%	39.17%	0.00%
**Typical seqs**		**Reads**	**Type**	**pct.**
CTCTGGCAGCTCTGATA|CAG**GGG**CTGG		37806	WT	51.28%
CTCTGGCAGCTCTGATAaCAGGGGCTGG		4374	+1 a	5.93%
CTCTGGCAG-------------------		2540	-22	3.44%
CTCTGGCAGCTCTGAT--AGGGGCTGG		1785	-2	2.42%
CTCTGGCA--------------------		2010	-21	2.73%
CTCTGGCAGCT------------CTGG		1659	-12	2.25%
CTCTGGCAGCTCTGAT-CAGGGGCTGG		1068	-1	1.45%
CTCTGGCAGCTCTGA--CAGGGGCTGG		947	-2	1.28%
CTCTGGCAGCT------CAGGGGCTGG		887	-6	1.20%
CTCTGG-----------CAGGGGCTGG		835	-11	1.13%

P41-P50-WT-U937-Syn65crMYH6g-AAV-KI-BC17

Total | INDEL	4h	Ins	Del	HDR
63027 | 4.13%		1.48%	1.77%	0.88%
**Typical seqs**		**Reads**	**Type**	**pct.**
CTCTGGCAGCTCTGATA|CAGGGGCTGG		60425	WT	95.87%
CTCTGGCAGCTCTGATAaCAGGGGCTGG		665	+1 a	1.06%
CTCTGGCAGCTCTGgtttaaactacgcgtc TCTCAGGCCCCCTACTGCC		526	-9 HDR	0.83%
CTCTGGCAGCTCTGAT--AGGGGCTGG		109	-2	0.17%
CTCTGGCAGCTCTGAT-CAGGGGCTGG		116	-1	0.18%
CTCTGGCAGCTCTGA--CAGGGGCTGG		114	-2	0.18%
CTCTGGCAGCTCTGATACA-GGGCTGG		60	-1	0.10%
CTCTGGCAGCTCTG---CAGGGGCTGG		43	-3	0.07%
CTCTGGCAGCTCT------GGGGCTGG		38	-6	0.06%
CTCTGGCAGCTCTGATATACAGGGGCTGG		37	+2	0.06%

Total | INDEL	8h	Ins	Del	HDR
117692 | 13.59%		3.25%	5.23%	5.11%
**Typical seqs**		**Reads**	**Type**	**pct.**
CTCTGGCAGCTCTGATA|CAGGGGCTGG		101697	WT	86.41%
CTCTGGCAGCTCTGgtttaaactacgcgtc TCTCAGGCCCCCTACTGCC		5660	-9 HDR	4.81%
CTCTGGCAGCTCTGATAaCAGGGGCTGG		2873	+1 a	2.44%
CTCTGGCAGCTCTGAT--AGGGGCTGG		716	-2	0.61%
CTCTGGCAGCTCTGAT-CAGGGGCTGG		486	-1	0.41%
CTCTGGCAGCTCTGA--CAGGGGCTGG		464	-2	0.39%
CTCTGGCAGCTCTGATACA-GGGCTGG		328	-1	0.28%
CTCTGGCAGCT------------CTGG		163	-12	0.14%
CTCTGGCAGCTCTGATA-AGGGGCTGG		184	-1	0.16%
CTCTGGCAGCTCTGATTACAGGGGCTGG		159	+1 Ins	0.14%

Total | INDEL	12h	Ins	Del	HDR
97838 | 25.69%		3.64%	8.58%	13.47%
**Typical seqs**		**Reads**	**Type**	**pct.**
CTCTGGCAGCTCTGATA|CAGGGGCTGG		72701	WT	74.31%
CTCTGGCAGCTCTGgtttaaactacgcgtc TCTCAGGCCCCCTACTGCC		12324	-9 HDR	12.60%
CTCTGGCAGCTCTGATAaCAGGGGCTGG		2363	+1 a	2.42%
CTCTGGCAGCTCTGAT--AGGGGCTGG		1046	-2	1.07%
CTCTGGCAGCTCTGA--CAGGGGCTGG		629	-2	0.64%
CTCTGGCAGCTCTGAT-CAGGGGCTGG		609	-1	0.62%
CTCTGGCAGCTCTGATACA-GGGCTGG		300	-1	0.31%
CTCTGGCAGCTCTG---CAGGGGCTGG		193	-3	0.20%
CTCTGGCAGCTCTGATA-AGGGGCTGG		175	-1	0.18%
CTCTGG-----------CAGGGGCTGG		167	-11	0.17%

Total | INDEL	24h	Ins	Del	HDR
87955 | 50.41%		6.33%	15.33%	28.74%
**Typical seqs**		**Reads**	**Type**	**pct.**
CTCTGGCAGCTCTGATA|CAGGGGCTGG		43620	WT	49.59%
CTCTGGCAGCTCTGgtttaaactacgcgtc TCTCAGGCCCCCTACTGCC		23543	-9 HDR	26.77%
CTCTGGCAGCTCTGATAaCAGGGGCTGG		3625	+1 a	4.12%
CTCTGGCAGCTCTGAT--AGGGGCTGG		1105	-2	1.26%
CTCTGGCAGCTCTGA--CAGGGGCTGG		706	-2	0.80%
CTCTGGCAGCTCTGAT-CAGGGGCTGG		705	-1	0.80%
CTCTGGCAGCTCTGATACA-GGGCTGG		410	-1	0.47%
CTCTGGCAGCT------CAGGGGCTGG		385	-6	0.44%
CTCTGGCAG------------------		521	-22	0.59%
CTCTGGCAGCT------------CTGG		343	-12	0.39%

Total | INDEL	48h	Ins	Del	HDR
55827 | 63.36%		7.00%	21.81%	34.55%
**Typical seqs**		**Reads**	**Type**	**pct.**
CTCTGGCAGCTCTGATA|CAGGGGCTGG		20456	WT	36.64%
CTCTGGCAGCTCTGgtttaaactacgcgtc TCTCAGGCCCCCTACTGCC		17881	-9 HDR	32.03%
CTCTGGCAGCTCTGATAaCAGGGGCTGG		2507	+1 a	4.49%
CTCTGGCAGCTCTGAT--AGGGGCTGG		1017	-2	1.82%
CTCTGGCAGCTCTGA--CAGGGGCTGG		569	-2	1.02%
CTCTGGCAGCT------------CTGG		475	-12	0.85%
CTCTGGCAGCTCTGAT-CAGGGGCTGG		449	-1	0.80%
CTCTGGCAG------------------		565	-22	1.01%
CTCTGG-----------CAGGGGCTGG		402	-11	0.72%
CTCTGGCAGCT------CAGGGGCTGG		328	-6	0.59%

P41-P50-WT-U937-Syn66crMYH6h-KO-BC18

Total | INDEL	4h	Ins	Del	HDR
38088 | 2.15%		1.60%	0.55%	0.00%

Typical seqs	Reads	Type	pct.
GCTCTGGCAGCTCTGAT|ACA**GGG**GCTG	37271	WT	97.85%
GCTCTGGCAGCTCTGATtACAGGGGCTG	527	+1 t	1.38%
GCTCTGGCAGCTCT---ACAGGGGCTG	44	-3	0.12%
GCTCTGGCAGCTCT--TACAGGGGCTG	17	-2	0.04%
GCTCTGGCAGCTCTG-TACAGGGGCTG	14	-1	0.04%
GCTCTGGCAGCTCTGATgACAGGGGCTG	13	+1 g	0.03%
GCTCTGGCAGCTCT------GGGGCTG	13	-6	0.03%
GCTCTGGCAGCTCTGA-ACAGGGGCTG	11	-1	0.03%
GCTCTGGCAGCTCTGATTTACAGGGGCTG	10	+2	0.03%
GCTCTGGCAGCTCTGAT----GGGCTG	9	-4	0.02%

Total | INDEL	8h	Ins	Del	HDR
76108 | 4.26%		3.25%	1.01%	0.00%

Typical seqs	Reads	Type	pct.
GCTCTGGCAGCTCTGAT|ACA**GGG**GCTG	72864	WT	95.74%
GCTCTGGCAGCTCTGATtACAGGGGCTG	2199	+1 t	2.89%
GCTCTGGCAGCTCT---ACAGGGGCTG	86	-3	0.11%
GCTCTGGCAGCTCTGATaACAGGGGCTG	58	+1 a	0.08%
GCTCTGGCAGCTCT--TACAGGGGCTG	50	-2	0.07%
GCTCTGGCAGCTCTGAT--AGGGGCTG	37	-2	0.05%
GCTCTGGCAGCTCTG--ACAGGGGCTG	38	-2	0.05%
GCTCTGGCAGCTCTG-TACAGGGGCTG	34	-1	0.04%
GCTCTGGCAGCT------------CTG	30	-12	0.04%
GCTCTGGCAGCTCTGATAC-GGGGCTG	18	-1	0.02%

Total | INDEL	12h	Ins	Del	HDR
30943 | 5.71%		3.98%	1.73%	0.00%

Typical seqs	Reads	Type	pct.
GCTCTGGCAGCTCTGAT|ACA**GGG**GCTG	29175	WT	94.29%
GCTCTGGCAGCTCTGATtACAGGGGCTG	1083	+1 t	3.50%
GCTCTGGCAGCTCT---ACAGGGGCTG	53	-3	0.17%
GCTCTGGCAGCTCTGATaACAGGGGCTG	48	+1 a	0.16%
GCTCTGG-----------CAGGGGCTG	28	-11	0.09%
GCTCTGGCAGCTCT--TACAGGGGCTG	26	-2	0.08%
GCTCTGGCAGCTCTGA-ACAGGGGCTG	22	-1	0.07%
GCTCTGGCAGCTCT------GGGGCTG	21	-6	0.07%
GCTCTGGCAGCTCTGATgACAGGGGCTG	15	+1 g	0.05%
GCTCTGGCAGCT------CAGGGGCTG	15	-6	0.05%

Total | INDEL	24h	Ins	Del	HDR
28130 | 11.29%		6.16%	5.13%	0.00%

Typical seqs	Reads	Type	pct.
GCTCTGGCAGCTCTGAT|ACA**GGG**GCTG	24953	WT	88.71%
GCTCTGGCAGCTCTGATtACAGGGGCTG	1495	+1 t	5.31%
GCTCTGGCAGCT------------CTG	77	-12	0.27%
GCTCTGGCAG-----------------	70	-22	0.25%
GCTCTGGCA------------------	87	-21	0.31%
GCTCTGG-----------CAGGGGCTG	61	-11	0.22%
GCTCTGGCAGCTCTGATaACAGGGGCTG	60	+1 a	0.21%
GCTCTGGCAGCTCT---ACAGGGGCTG	63	-3	0.22%
GCTCTGGCAGCTCTG----AGGGGCTG	50	-4	0.18%
GCTCTGGCA--------------GCTG	44	-14	0.16%

Total | INDEL	48h	Ins	Del	HDR
27137 | 16.33%		8.45%	7.88%	0.00%

Typical seqs	Reads	Type	pct.
GCTCTGGCAGCTCTGAT|ACA**GGG**GCTG	22705	WT	83.67%
GCTCTGGCAGCTCTGATtACAGGGGCTG	1924	+1 t	7.09%
GCTCTGGCAGCT------------CTG	131	-12	0.48%
GCTCTGGCAGCTCT---ACAGGGGCTG	108	-3	0.40%
GCTCTGGCAGCTCTG----AGGGGCTG	95	-4	0.35%
GCTCTGGCA------------------	118	-21	0.43%
GCTCTGG-----------CAGGGGCTG	79	-11	0.29%
GCTCTGGCA--------------GCTG	79	-14	0.29%
GCTCTGGCAG-----------------	78	-22	0.29%
GCTCTGGCAGCTCTGATaACAGGGGCTG	78	+1 a	0.29%

P41-P50-WT-U937-Syn66crMYH6h-AAV-KI-BC18

Total | INDEL	4h	Ins	Del	HDR
25124 | 2.43%		1.78%	0.39%	0.27%

Typical seqs	Reads	Type	pct.
GCTCTGGCAGCTCTGAT|ACA**GGG**GCTG	24513	WT	97.57%
GCTCTGGCAGCTCTGATtACAGGGGCTG	376	+1 t	1.50%
GCTCTGGCAGCTCTGgtttaaactacgcgt cTCTCAGGCCCCCTACTGCC	61	-9 HDR	0.24%
GCTCTGGCAGCTCTGATaACAGGGGCTG	26	+1 a	0.10%
GCTCTGGCAGCTCT---ACAGGGGCTG	21	-3	0.08%
GCTCTGGCAGCTCTGATAC-GGGGCTG	12	-1	0.05%
GCTCTGGCAGCTCTGA-ACAGGGGCTG	10	-1	0.04%
GCTCTGGCAGCTCTGATgACAGGGGCTG	10	+1 g	0.04%
GCTCTGGCAGCTCTGAT--AGGGGCTG	9	-2	0.04%
GCTCTGGCAGCTCTGATtACAGGGGCCG	6	+1 t	0.02%

Total | INDEL	8h	Ins	Del	HDR
40982 | 5.27%		3.11%	0.88%	1.28%

Typical seqs	Reads	Type	pct.
GCTCTGGCAGCTCTGAT|ACA**GGG**GCTG	38822	WT	94.73%
GCTCTGGCAGCTCTGATtACAGGGGCTG	1027	+1 t	2.51%
GCTCTGGCAGCTCTGgtttaaactacgcgt cTCTCAGGCCCCCTACTGCC	489	-9 HDR	1.19%
GCTCTGGCAGCTCT---ACAGGGGCTG	61	-3	0.15%
GCTCTGGCAGCTCTGATaACAGGGGCTG	58	+1 a	0.14%
GCTCTGGCAGCTCTGAT-CAGGGGCTG	21	-1	0.05%
GCTCTGGCAGCTCTGAT--AGGGGCTG	21	-2	0.05%
GCTCTGGCAGCTCTGATgACAGGGGCTG	18	+1 g	0.04%
GCTCTGGCGGCTCTGATTACAGGGGCTG	21	+1 Ins	0.05%
GCTCTGGCAGCTCTG--ACAGGGGCTG	17	-2	0.04%

Total | INDEL	12h	Ins	Del	HDR
32407 | 8.29%		3.66%	1.60%	3.02%

Typical seqs	Reads	Type	pct.
GCTCTGGCAGCTCTGAT|ACA**GGG**GCTG	29721	WT	91.71%
GCTCTGGCAGCTCTGATtACAGGGGCTG	973	+1 t	3.00%
GCTCTGGCAGCTCTGgtttaaactacgcgt cTCTCAGGCCCCCTACTGCC	923	-9 HDR	2.85%
GCTCTGGCAGCTCT---ACAGGGGCTG	65	-3	0.20%
GCTCTGGCAGCTCTGATaACAGGGGCTG	56	+1 a	0.17%
GCTCTGGCAGCTCT--TACAGGGGCTG	31	-2	0.10%
GCTCTGGCAGCTCTG--ACAGGGGCTG	23	-2	0.07%
GCTCTGGCAGCTCTGATgACAGGGGCTG	21	+1 g	0.06%
GCTCTGGCAGCTCTGAT--AGGGGCTG	20	-2	0.06%
GCTCTGGCAGCTCTGA-ACAGGGGCTG	18	-1	0.06%

Total | INDEL	24h	Ins	Del	HDR
32115 | 16.71%		6.02%	3.84%	6.85%

Typical seqs	Reads	Type	pct.
GCTCTGGCAGCTCTGAT|ACA**GGG**GCTG	26750	WT	83.29%
GCTCTGGCAGCTCTGgtttaaactacgcgt cTCTCAGGCCCCCTACTGCC	2080	-9 HDR	6.48%
GCTCTGGCAGCTCTGATtACAGGGGCTG	1640	+1 t	5.11%
GCTCTGGCAGCTCT---ACAGGGGCTG	111	-3	0.35%
GCTCTGGCAGCT------------CTG	70	-12	0.22%
GCTCTGGCAGCTCT--TACAGGGGCTG	51	-2	0.16%
GCTCTGGCAGCTCTGATaACAGGGGCTG	47	+1 a	0.15%
GCTCTGGCA------------------	52	-21	0.16%
GCTCTGGCAGCTCTG--ACAGGGGCTG	44	-2	0.14%
GCTCTGGCA--------------GCTG	34	-14	0.11%

Total | INDEL	48h	Ins	Del	HDR
17976 | 21.31%		8.16%	5.74%	7.42%

Typical seqs	Reads	Type	pct.
GCTCTGGCAGCTCTGAT|ACA**GGG**GCTG	14145	WT	78.69%
GCTCTGGCAGCTCTGgtttaaactacgcgt cTCTCAGGCCCCCTACTGCC	1264	-9 HDR	7.03%
GCTCTGGCAGCTCTGATtACAGGGGCTG	1243	+1 t	6.91%
GCTCTGGCAGCTCT---ACAGGGGCTG	62	-3	0.34%
GCTCTGGCAGCT------------CTG	51	-12	0.28%
GCTCTGGCAGCTCTGATaACAGGGGCTG	42	+1 a	0.23%
GCTCTGGCAGCTCT------GGGGCTG	39	-6	0.22%
GCTCTGGCA------------------	46	-21	0.26%
GCTCTGGCAGCTCTG--ACAGGGGCTG	37	-2	0.21%
GCTCTGGCAG-----------------	45	-22	0.25%

P41-P50-WT-U937-Syn67crMYH6i-KO-BC19

Total | INDEL	4h	Ins	Del	HDR
33371 | 0.48%		0.19%	0.28%	0.00%

Typical seqs	Reads	Type	pct.
GGCTCTGGCAGCTCTGA|TAC**AGG**GGCT	33212	WT	99.52%
GGCTCTGGCAGCTCTGAaTACAGGGGCT	28	+1 a	0.08%
GGCTCTGGCAGCTCTG--ACAGGGGCT	25	-2	0.07%
GGCTCTGGCAGCTCTGAtTACAGGGGCT	20	+1 t	0.06%
GGCTCTGGCAGCT------------CT	8	-12	0.02%
GGCTCTGGCAGCTCTGAtAACAGGGGCT	8	+1 t	0.02%
GGCTCTGGCAGCTCTG----AGGGGCT	7	-4	0.02%
GGCTCTGGCAGCTC--ATACAGGGGCT	6	-2	0.02%
GGCTCTGGCAGCTCTGAgTACAGGGGCT	5	+1 g	0.01%
GGCTCTGGCAGCTCTGATACGGGGGCT	182	-1	0.55%

Total | INDEL	8h	Ins	Del	HDR
217037 | 1.20%		0.42%	0.77%	0.00%

Typical seqs	Reads	Type	pct.
GGCTCTGGCAGCTCTGA|TAC**AGG**GGCT	214438	WT	98.80%
GGCTCTGGCAGCTCTGAaTACAGGGGCT	411	+1 a	0.19%
GGCTCTGGCAGCTCTG--ACAGGGGCT	277	-2	0.13%
GGCTCTGGCAGCTCTGAtTACAGGGGCT	201	+1 t	0.09%
GGCTCTGGCAGCTCTGA-ACAGGGGCT	144	-1	0.07%
GGCTCTGGCAGCTCTG----AGGGGCT	125	-4	0.06%
GGCTCTGGCAGCTCTGAtAACAGGGGCT	88	+1 t	0.04%
GGCTCTGGCAGCTCTG-TACAGGGGCT	80	-1	0.04%
GGCTCTGGCAGCTC---TACAGGGGCT	83	-3	0.04%
GGCTCTGGCAGCTCTGATAC-GGGGCT	42	-1	0.02%

Total | INDEL	12h	Ins	Del	HDR
125956 | 1.91%		0.64%	1.27%	0.00%

Typical seqs	Reads	Type	pct.
GGCTCTGGCAGCTCTGA|TAC**AGG**GGCT	123546	WT	98.09%
GGCTCTGGCAGCTCTGAaTACAGGGGCT	346	+1 a	0.27%
GGCTCTGGCAGCTCTG--ACAGGGGCT	292	-2	0.23%
GGCTCTGGCAGCTCTGAtTACAGGGGCT	194	+1 t	0.15%
GGCTCTGGCAGCTCTGA-ACAGGGGCT	128	-1	0.10%
GGCTCTGGCAGCTCTG-TACAGGGGCT	92	-1	0.07%
GGCTCTGGCAGCTCTG----AGGGGCT	80	-4	0.06%
GGCTCTGGCAGCTCTGAtAACAGGGGCT	71	+1 t	0.06%
GGCTCTGGCAGCTCTGAcTACAGGGGCT	63	+1 c	0.05%
GGCTCTGGCAGCTC---TACAGGGGCT	52	-3	0.04%

Total | INDEL	24h	Ins	Del	HDR
168812 | 5.87%		1.29%	4.58%	0.00%

Typical seqs	Reads	Type	pct.
GGCTCTGGCAGCTCTGA|TAC**AGG**GGCT	158899	WT	94.13%
GGCTCTGGCAGCTCTGAaTACAGGGGCT	1065	+1 a	0.63%
GGCTCTGGCAGCTCTG--ACAGGGGCT	585	-2	0.35%
GGCTCTGGCAGCTCTGA-ACAGGGGCT	495	-1	0.29%
GGCTCTGGCAGCT------------CT	493	-12	0.29%
GGCTCTGGCAGCTCTG----AGGGGCT	352	-4	0.21%
GGCTCTGGCAGCTCTGAtTACAGGGGCT	356	+1 t	0.21%
GGCTCTGGCAGCTCTG-TACAGGGGCT	313	-1	0.19%
GGCTCTGGCA-----------------	336	-21	0.20%
GGCTCTGG-----------CAGGGGCT	248	-11	0.15%

Total | INDEL	48h	Ins	Del	HDR
196832 | 9.26%		1.90%	7.36%	0.00%

Typical seqs	Reads	Type	pct.
GGCTCTGGCAGCTCTGA|TAC**AGG**GGCT	178607	WT	90.74%
GGCTCTGGCAGCTCTGAaTACAGGGGCT	1824	+1 a	0.93%
GGCTCTGGCAGCTCTGA-ACAGGGGCT	894	-1	0.45%
GGCTCTGGCAGCTCTG--ACAGGGGCT	843	-2	0.43%
GGCTCTGGCAGCT------------CT	817	-12	0.42%
GGCTCTGGCAGCTCTG----AGGGGCT	745	-4	0.38%
GGCTCTGG-----------CAGGGGCT	578	-11	0.29%
GGCTCTGGCA-----------------	794	-21	0.40%
GGCTCTGGCAGCTCTG-TACAGGGGCT	540	-1	0.27%
GGCTCTGGCAGCTCT------GGGGCT	500	-6	0.25%

P41-P50-WT-U937-Syn67crMYH6i-AAV-KI-BC19

4h

Total | INDEL	Ins	Del	HDR
116359 | 2.63%	0.21%	0.42%	2.00%

Typical seqs	Reads	Type	pct.
GGCTCTGGCAGCTCTGA|TAC**AGG**GGCT	113304	WT	97.37%
GGCTCTGGCAGCTCTGgtttaaactacgcg tcTCTCAGGCCCCCTACTGCC	2222	-9 HDR	1.91%
GGCTCTGGCAGCTCTGAtTACAGGGGCT	89	+1 t	0.08%
GGCTCTGGCAGCTCTGAaTACAGGGGCT	87	+1 a	0.07%
GGCTCTGGCAGCTCTG--ACAGGGGCT	69	-2	0.06%
GGCTCTGGCAGCTCTGA-ACAGGGGCT	38	-1	0.03%
GGCTCTGGCAGCTCTGAtAACAGGGGCT	26	+1 t	0.02%
GGCTCTGGCAGCTCTGATAC-GGGGCT	25	-1	0.02%
GGCTCTGGCAGCTCTGAcTACAGGGGCT	21	+1 c	0.02%
GGCTCTGGCAGCTCTG-TACAGGGGCT	20	-1	0.02%

8h

Total | INDEL	Ins	Del	HDR
41429 | 6.60%	0.53%	1.00%	5.07%

Typical seqs	Reads	Type	pct.
GGCTCTGGCAGCTCTGA|TAC**AGG**GGCT	38695	WT	93.40%
GGCTCTGGCAGCTCTGgtttaaactacgcg tcTCTCAGGCCCCCTACTGCC	1991	-9 HDR	4.81%
GGCTCTGGCAGCTCTGAtTACAGGGGCT	91	+1 t	0.22%
GGCTCTGGCAGCTCTG--ACAGGGGCT	65	-2	0.16%
GGCTCTGGCAGCTCTGAaTACAGGGGCT	44	+1 a	0.11%
GGCTCTGGCAGCTCTGAtAACAGGGGCT	43	+1 t	0.10%
GGCTCTGGCAGCTCTGA-ACAGGGGCT	19	-1	0.05%
GGCTCTGGCAGCTC---TACAGGGGCT	19	-3	0.05%
GGCTCTGGCAGCTCTGATAC-GGGGCT	13	-1	0.03%
GGCTCTGGCAGCTCTG----AGGGGCT	12	-4	0.03%

12h

Total | INDEL	Ins	Del	HDR
153106 | 4.87%	0.58%	1.51%	2.77%

Typical seqs	Reads	Type	pct.
GGCTCTGGCAGCTCTGA|TAC**AGG**GGCT	145653	WT	95.13%
GGCTCTGGCAGCTCTGgtttaaactacgcg tcTCTCAGGCCCCCTACTGCC	3974	-9 HDR	2.60%
GGCTCTGGCAGCTCTGAaTACAGGGGCT	375	+1 a	0.24%
GGCTCTGGCAGCTCTG--ACAGGGGCT	382	-2	0.25%
GGCTCTGGCAGCTCTGAtTACAGGGGCT	218	+1 t	0.14%
GGCTCTGGCAGCTCTGA-ACAGGGGCT	179	-1	0.12%
GGCTCTGGCAGCTCTG-TACAGGGGCT	175	-1	0.11%
GGCTCTGGCAGCTCTG----AGGGGCT	134	-4	0.09%
GGCTCTGGCAGCTC---TACAGGGGCT	101	-3	0.07%
GGCTCTGGCAGCTCTGAtAACAGGGGCT	90	+1 t	0.06%

24h

Total | INDEL	Ins	Del	HDR
20755 | 10.06%	1.41%	3.03%	5.63%

Typical seqs	Reads	Type	pct.
GGCTCTGGCAGCTCTGA|TAC**AGG**GGCT	18667	WT	89.94%
GGCTCTGGCAGCTCTGgtttaaactacgcgtcTC TCAGGCCCCCTACTGCC	1116	-9 HDR	5.38%
GGCTCTGGCAGCTCTGAaTACAGGGGCT	126	+1 a	0.61%
GGCTCTGGCAGCTCTG--ACAGGGGCT	76	-2	0.37%
GGCTCTGGCAGCTCTGA-ACAGGGGCT	59	-1	0.28%
GGCTCTGGCAGCTCTGAtTACAGGGGCT	50	+1 t	0.24%
GGCTCTGGCAGCTCTGAtAACAGGGGCT	35	+1 t	0.17%
GGCTCTGGCAGCTCTG-TACAGGGGCT	30	-1	0.14%
GGCTCTGGCAGCTCTG----AGGGGCT	24	-4	0.12%
GGCTCTGGCAGCTC---TACAGGGGCT	20	-3	0.10%

48h

Total | INDEL	Ins	Del	HDR
154332 | 15.12%	2.07%	5.72%	7.34%

Typical seqs	Reads	Type	pct.
GGCTCTGGCAGCTCTGA|TAC**AGG**GGCT	130992	WT	84.88%
GGCTCTGGCAGCTCTGgtttaaactacgcgtcTC TCAGGCCCCCTACTGCC	10635	-9 HDR	6.89%
GGCTCTGGCAGCTCTGAaTACAGGGGCT	1379	+1 a	0.89%
GGCTCTGGCAGCTCTG--ACAGGGGCT	779	-2	0.50%
GGCTCTGGCAGCTCTGA-ACAGGGGCT	663	-1	0.43%
GGCTCTGGCAGCTCTGAtTACAGGGGCT	597	+1 t	0.39%
GGCTCTGGCAGCT------------CT	392	-12	0.25%
GGCTCTGGCAGCTCTG-TACAGGGGCT	354	-1	0.23%
GGCTCTGGCAGCTCTG----AGGGGCT	353	-4	0.23%
GGCTCTGG-----------CAGGGGCT	277	-11	0.18%

P41-P50-WT-U937-Syn45crHBG2-e-KO-BC7

4h

Total	INDEL	Ins	Del	HDR
37086	1.98%	0.38%	1.60%	0.00%

Typical seqs	Reads	Type	pct.
ATTGAGATAGTGTGGGGAAG**GGG**CCCC	36352	WT	98.02%
ATTGAGATAGTGTGGG-AAGGGGCCCC	132	-1	0.36%
ATTGAGATAGTGTGGGGgAAGGGGCCCC	58	+1 g	0.16%
ATTGAGATAGTGTGGGG-AGGGGCCCC	50	-1	0.13%
ATTGAGATAGTGTGG------GGCCCC	35	-6	0.09%
ATTGAGATAGTGTGG--AAGGGGCCCC	32	-2	0.09%
ATTGAGATAGTGTGGGG-AGGGGGCCC	21	-1	0.06%
ATTGAGATAGTGT---GAAGGGGCCCC	21	-3	0.06%
ATTGAGATAGTGTGGGGAA-GGGCCCC	17	-1	0.05%

8h

Total	INDEL	Ins	Del	HDR
31088	3.82%	0.58%	3.24%	0.00%

Typical seqs	Reads	Type	pct.
ATTGAGATAGTGTGGGGAAG**GGG**CCCC	29899	WT	96.18%
ATTGAGATAGTGTGGG-AAGGGGCCCC	153	-1	0.49%
ATTGAGATAGTGTGGGGgAAGGGGCCCC	99	+1 g	0.32%
ATTGAGATAGTGTGG------GGCCCC	95	-6	0.31%
ATTGAGATAGTGT----AAGGGGCCCC	78	-4	0.25%
ATTGAGATAGTGTGGGG-AGGGGCCCC	69	-1	0.22%
ATTGAGATAGTGTGG--AAGGGGCCCC	38	-2	0.12%
ATTGAGATAGTGTGGGGaAAGGGGCCCC	36	+1 a	0.12%
ATTGAGAT---------AAGGGGCCCC	31	-9	0.10%
ATTGAGATAGTGT---GAAGGGGCCCC	28	-3	0.09%

12h

Total	INDEL	Ins	Del	HDR
24636	5.62%	0.67%	4.95%	0.00%

Typical seqs	Reads	Type	pct.
ATTGAGATAGTGTGGGGAAG**GGG**CCCC	23252	WT	94.38%
ATTGAGATAGTGTGG------GGCCCC	165	-6	0.67%
ATTGAGATAGTGTGGG-AAGGGGCCCC	123	-1	0.50%
ATTGAGATAGTGT----AAGGGGCCCC	81	-4	0.33%
ATTGAGATAGTGTGGGGgAAGGGGCCCC	80	+1 g	0.32%
ATTGAGATAGTGTGG--AAGGGGCCCC	69	-2	0.28%
ATTGAGAT---------AAGGGGCCCC	53	-9	0.22%
ATTGAGATAGTGTGGGG-AGGGGCCCC	48	-1	0.19%
ATTGAGATAGTGTG-----GGGGCCCC	41	-5	0.17%
ATTGAGATAGTGT---GAAGGGGCCCC	39	-3	0.16%

24h

Total	INDEL	Ins	Del	HDR
45648	14.03%	1.36%	12.67%	0.00%

Typical seqs	Reads	Type	pct.
ATTGAGATAGTGTGGGGAAG**GGG**CCCC	39243	WT	85.97%
ATTGAGATAGTGTGG------GGCCCC	1219	-6	2.67%
ATTGAGATAGTGTGGG-AAGGGGCCCC	429	-1	0.94%
ATTGAGATAGTGTG-----GGGGCCCC	276	-5	0.60%
ATTGAGATAGTGTGGGGgAAGGGGCCCC	262	+1 g	0.57%
ATTGAGATAGTGT----AAGGGGCCCC	240	-4	0.53%
ATTGAGATAGTGT-------GGGCCCC	217	-7	0.48%
ATTGAGATAGTGTGG--AAGGGGCCCC	200	-2	0.44%
ATTGAGATAGTGTGGGG-AGGGGCCCC	181	-1	0.40%
ATTGAGATAGTGT---GAAGGGGCCCC	145	-3	0.32%

48h

Total	INDEL	Ins	Del	HDR
29002	18.68%	1.67%	17.01%	0.00%

Typical seqs	Reads	Type	pct.
ATTGAGATAGTGTGGGGAAG**GGG**CCCC	23584	WT	81.32%
ATTGAGATAGTGTGG------GGCCCC	1184	-6	4.08%
ATTGAGATAGTGTGGG-AAGGGGCCCC	329	-1	1.13%
ATTGAGATAGTGTG-----GGGGCCCC	305	-5	1.05%
ATTGAGATAGTGT----AAGGGGCCCC	208	-4	0.72%
ATTGAGATAGTGTGGGGgAAGGGGCCCC	190	+1 g	0.66%
ATTGAGATAGTGT-------GGGCCCC	193	-7	0.67%
ATTGAGATAGTGTGG--AAGGGGCCCC	152	-2	0.52%
ATTGAGATAGTGTGGGG-AGGGGCCCC	133	-1	0.46%

P41-P50-WT-U937-Syn46crHBG2-f-KO-BC8

4h

Total | INDEL	Ins	Del	HDR
33508 | 2.18%	0.44%	1.74%	0.00%

Typical seqs	Reads	Type	pct.
CATTGAGATAGTGTGGG|GAAGGGGCCC	32777	WT	97.82%
CATTGAGATAGTGTGG-GAAGGGGCCC	292	-1	0.87%
CATTGAGATAGTGTGGGgGAAGGGGCCC	121	+1 g	0.36%
CATTGAGATAGTGTGG--AAGGGGCCC	43	-2	0.13%
CATTGAGATAGTGTGGGG-AGGGGCCC	31	-1	0.09%
CATTGAGATAGTGT------GGGGCCC	16	-6	0.05%
CATTGAGATAGTGTGGGG-AGGGGGCC	13	-1	0.04%
CATTGAGATAGT-----GAAGGGGCCC	12	-5	0.04%
CATTGAGATAGTGT-------GGGCCC	9	-7	0.03%

8h

Total | INDEL	Ins	Del	HDR
22792 | 3.46%	0.63%	2.83%	0.00%

Typical seqs	Reads	Type	pct.
CATTGAGATAGTGTGGG|GAAGGGGCCC	22004	WT	96.54%
CATTGAGATAGTGTGG-GAAGGGGCCC	270	-1	1.18%
CATTGAGATAGTGTGGGgGAAGGGGCCC	127	+1 g	0.56%
CATTGAGATAGTGTGG--AAGGGGCCC	45	-2	0.20%
CATTGAGATAGTGT------GGGGCCC	44	-6	0.19%
CATTGAGATAGTGT-------GGGCCC	29	-7	0.13%
CATTGAGATAGTGTGGGG-AGGGGCCC	18	-1	0.08%
CATTGAGATAGTGT---GAAGGGGCCC	17	-3	0.07%
CATTGAGATAGT-----GAAGGGGCCC	15	-5	0.07%

12h

Total | INDEL	Ins	Del	HDR
19012 | 4.95%	0.86%	4.09%	0.00%

Typical seqs	Reads	Type	pct.
CATTGAGATAGTGTGGG|GAAGGGGCCC	18070	WT	95.05%
CATTGAGATAGTGTGG-GAAGGGGCCC	282	-1	1.48%
CATTGAGATAGTGTGGGgGAAGGGGCCC	136	+1 g	0.72%
CATTGAGATAGTGTGG--AAGGGGCCC	69	-2	0.36%
CATTGAGATAGTGT------GGGGCCC	59	-6	0.31%
CATTGAGATAGTGT-------GGGCCC	35	-7	0.18%
CATTGAGATAGT-----GAAGGGGCCC	21	-5	0.11%
CATTGAGATAGT----GGAAGGGGCCC	17	-4	0.09%
CATTGAGATAGTGTGGGG-AGGGGCCC	14	-1	0.07%

24h

Total | INDEL	Ins	Del	HDR
38515 | 10.96%	1.96%	9.00%	0.00%

Typical seqs	Reads	Type	pct.
CATTGAGATAGTGTGGG|GAAGGGGCCC	34294	WT	89.04%
CATTGAGATAGTGTGG-GAAGGGGCCC	804	-1	2.09%
CATTGAGATAGTGTGGGgGAAGGGGCCC	559	+1 g	1.45%
CATTGAGATAGTGT------GGGGCCC	433	-6	1.12%
CATTGAGATAGTGT-------GGGCCC	244	-7	0.63%
CATTGAGATAGTGTGG--AAGGGGCCC	208	-2	0.54%
CATTGAGATA-------GAAGGGGCCC	109	-7	0.28%
CATTGAGATAGGTTT---------------AAACT	84	-7	0.22%
CATTGAGATAGTGTG-----GGGGCCC	74	-5	0.19%
CATTGAGATAGT-----GAAGGGGCCC	71	-5	0.18%

48h

Total | INDEL	Ins	Del	HDR
4895 | 11.52%	1.51%	10.01%	0.00%

Typical seqs	Reads	Type	pct.
CATTGAGATAGTGTGGG|GAAGGGGCCC	4331	WT	88.48%
CATTGAGATAGTGTGG-GAAGGGGCCC	87	-1	1.78%
CATTGAGATAGTGT------GGGGCCC	70	-6	1.43%
CATTGAGATAGTGTGGGgGAAGGGGCCC	52	+1 g	1.06%
CATTGAGATAGTGT-------GGGCCC	24	-7	0.49%
CATTGAGATAGTGTGG--AAGGGGCCC	24	-2	0.49%
CATTGAGATAGTGTG-----GGGGCCC	16	-5	0.33%
CATTGAGATAGTGTGGGG-AGGGGCCC	13	-1	0.27%
CATTGAGATA----GGGGAAGGGGCCC	12	-4	0.25%

P41-P50-WT-U937-Syn47crHBG2-g-KO-BC9

4h — Total 17515 | INDEL 1.60% | Ins 0.41% | Del 1.19% | HDR 0.00%

Typical seqs	Reads	Type	pct.
GAGATAGTGTGG|GGA**AGG**GGCC	17234	WT	98.40%
GAGATAGTGTG-GGAAGGGGCC	74	-1	0.42%
GAGATAGTGTGGgGGAAGGGGCC	37	+1 g	0.21%
GAGATAGTTGTGGGGAAGGGGCC	35	+1 Ins	0.20%
GAGATAGTGTGGGG-AGGGGCC	12	-1	0.07%
GAGATAGTGTG--GAAGGGGCC	12	-2	0.07%
GAGATAGTGTGGGG-AGGGGGC	8	-1	0.05%
GAGATA--GTGGGGAAGGGGCC	7	-2	0.04%
GAGATAGTGT------GGGGCC	7	-6	0.04%

8h — Total 28550 | INDEL 2.72% | Ins 0.71% | Del 2.01% | HDR 0.00%

Typical seqs	Reads	Type	pct.
GCATTGAGATAGTGTGG|GGA**AGG**GGCC	27773	WT	97.28%
GCATTGAGATAGTGTG-GGAAGGGGCC	154	-1	0.54%
GCATTGAGATAGTGTG--GAAGGGGCC	116	-2	0.41%
GCATTGAGATAGTGTGGgGGAAGGGGCC	105	+1 g	0.37%
GCATTGAGATAGTTGTGGGGAAGGGGCC	40	+1 Ins	0.14%
GCATTGAGATAGTGTGGGG-AGGGGCC	28	-1	0.10%
GCATTGAGATAGTGT-------GGGCC	14	-7	0.05%
GCATTGAGATAGTGT------GGGGCC	14	-6	0.05%
GCATTGAGATAGT----GGAAGGGGCC	12	-4	0.04%
GCATTGAGATAGTGTGGaGGAAGGGGCC	12	+1 a	0.04%

12h — Total 21432 | INDEL 4.15% | Ins 0.96% | Del 3.19% | HDR 0.00%

Typical seqs	Reads	Type	pct.
GCATTGAGATAGTGTGG|GGA**AGG**GGCC	20542	WT	95.85%
GCATTGAGATAGTGTG-GGAAGGGGCC	155	-1	0.72%
GCATTGAGATAGTGTG--GAAGGGGCC	128	-2	0.60%
GCATTGAGATAGTGTGGgGGAAGGGGCC	112	+1 g	0.52%
GCATTGAGATAGTTGTGGGGAAGGGGCC	46	+1 Ins	0.21%
GCATTGAGATAGTGT------GGGGCC	22	-6	0.10%
GCATTGAGATA------GGAAGGGGCC	20	-6	0.09%
GCATTGAGATAGTGT-------GGGCC	16	-7	0.07%
GCATTGAGATAGT----GGAAGGGGCC	16	-4	0.07%

24h — Total 44948 | INDEL 9.92% | Ins 1.98% | Del 7.95% | HDR 0.00%

Typical seqs	Reads	Type	pct.
GCATTGAGATAGTGTGG|GGA**AGG**GGCC	40487	WT	90.08%
GCATTGAGATAGTGTG--GAAGGGGCC	653	-2	1.45%
GCATTGAGATAGTGTG-GGAAGGGGCC	545	-1	1.21%
GCATTGAGATAGTGTGGgGGAAGGGGCC	433	+1 g	0.96%
GCATTGAGATAGTGT------GGGGCC	307	-6	0.68%
GCATTGAGATAGTTGTGGGGAAGGGGCC	190	+1 Ins	0.42%
GCATTGAGATAGTGT-------GGGCC	156	-7	0.35%
GCATTGAGATA--GTGGGGAAGGGGCC	82	-2	0.18%
GCATTGAGAT----------AGGGGCC	60	-10	0.13%
GCATTGAGATAGTGTG-----GGGGCC	59	-5	0.13%

48h — Total 30482 | INDEL 13.11% | Ins 2.35% | Del 10.76% | HDR 0.00%

Typical seqs	Reads	Type	pct.
GCATTGAGATAGTGTGG|GGA**AGG**GGCC	26486	WT	86.89%
GCATTGAGATAGTGTG--GAAGGGGCC	620	-2	2.03%
GCATTGAGATAGTGTG-GGAAGGGGCC	512	-1	1.68%
GCATTGAGATAGTGTGGgGGAAGGGGCC	390	+1 g	1.28%
GCATTGAGATAGTGT------GGGGCC	318	-6	1.04%
GCATTGAGATAGTGT-------GGGCC	154	-7	0.51%
GCATTGAGATAGT----GGAAGGGGCC	86	-4	0.28%
GCATTGAGATAGTtGTGGGGAAGGGGCC	84	+1 Ins	0.28%
GCATTGAGAT----------AGGGGCC	58	-10	0.19%

P41-P50-WT-U937-Syn48crHBG2-h-KO-BC10

Total | INDEL	4h	Ins	Del	HDR
7686 | 1.48%		0.91%	0.57%	0.00%

Typical seqs	Reads	Type	pct.
ATTTGCATTGAGATAGT|GTGGGGAAGG	7572	WT	98.52%
ATTTGCATTGAGATAGTtGTGGGGAAGG	64	+1 t	0.83%
ATTTGCATTGAGATAGTGT-GGGAAGG	17	-1	0.22%
ATTTGCATTGAGATA-TGTGGGGAAGG	5	-1	0.07%
ATTTGCATTGAGAT--TGTGGGGAAGG	4	-2	0.05%
ATTTGCATT------GTGTGGGGAAGG	4	-6	0.05%
ATTTGCATTGAGATAGT----GGAAGG	4	-4	0.05%
ATTTGCATTGAGATAAGTGTGGGGAAGG	2	+1 Ins	0.03%
ATTTGCATTGAGATA------GGAAGG	2	-6	0.03%
ATTTGCATTGA-----------GAAGG	2	-11	0.03%

Total | INDEL	8h	Ins	Del	HDR
7288 | 2.46%		1.65%	0.81%	0.00%

Typical seqs	Reads	Type	pct.
ATTTGCATTGAGATAGT|GTGGGGAAGG	7109	WT	97.54%
ATTTGCATTGAGATAGTtGTGGGGAAGG	92	+1 t	1.26%
ATTTGCATTGAGATAGTGT-GGGAAGG	20	-1	0.27%
ATTTGCATTGAGATAAGTGTGGGGAAGG	18	+1 Ins	0.25%
ATTTGCATTGAGAT---GTGGGGAAGG	7	-3	0.10%
ATTTGCATTGAGAT--TGTGGGGAAGG	5	-2	0.07%
ATTTGCATTGAGATAG--TGGGGAAGG	4	-2	0.05%
ATTTGCATTGAGATA----GGGGAAGG	4	-4	0.05%
ATTTGCATTGAGATAGTGT--GGAAGG	3	-2	0.04%
ATTTGCATTGAGATAGTTTGTGGGGAAGG	3	+2	0.04%

Total | INDEL	12h	Ins	Del	HDR
7790 | 3.08%		1.82%	1.26%	0.00%

Typical seqs	Reads	Type	pct.
ATTTGCATTGAGATAGT|GTGGGGAAGG	7550	WT	96.92%
ATTTGCATTGAGATAGTtGTGGGGAAGG	136	+1 t	1.75%
ATTTGCATTGAGATAGTGT-GGGAAGG	18	-1	0.23%
ATTTGCATTGAGATAG--TGGGGAAGG	12	-2	0.15%
ATTTGCATTGAGATAGTGT--GGAAGG	8	-2	0.10%
ATTTGCATTGAGATA----GGGGAAGG	7	-4	0.09%
ATTTGCATTGAGATAAGTGTGGGGAAGG	6	+1 Ins	0.08%
ATTTGCATTGAGAT---GTGGGGAAGG	5	-3	0.06%
ATTTGCATTGAGAT----------AGG	4	-10	0.05%
ATTTGCATT------GTGTGGGGAAGG	3	-6	0.04%

Total | INDEL	24h	Ins	Del	HDR
8551 | 6.79%		3.20%	3.59%	0.00%

Typical seqs	Reads	Type	pct.
ATTTGCATTGAGATAGT|GTGGGGAAGG	7970	WT	93.21%
ATTTGCATTGAGATAGTtGTGGGGAAGG	241	+1 t	2.82%
ATTTGCATTGAGATAGTGT-GGGAAGG	42	-1	0.49%
ATTTGCATTGAGATA----GGGGAAGG	32	-4	0.37%
ATTTGCATTGAGATAG--TGGGGAAGG	23	-2	0.27%
ATTTGCATTGAGATAGTGT-------G	20	-7	0.23%
ATTTGCATTGAGATAGTGT--GGAAGG	20	-2	0.23%
ATTTGCATTGAGAT---GTGGGGAAGG	16	-3	0.19%
ATTTGCATTGAGAT----------AGG	13	-10	0.15%
ATTTGCATTGAGATA-----GGGAAGG	10	-5	0.12%

Total | INDEL	48h	Ins	Del	HDR
7383 | 8.61%		4.51%	4.10%	0.00%

Typical seqs	Reads	Type	pct.
ATTTGCATTGAGATAGT|GTGGGGAAGG	6747	WT	91.39%
ATTTGCATTGAGATAGTtGTGGGGAAGG	299	+1 t	4.05%
ATTTGCATTGAGATAGTGT-GGGAAGG	39	-1	0.53%
ATTTGCATTGAGATA----GGGGAAGG	31	-4	0.42%
ATTTGCATTGAGATAGTGT-------G	22	-7	0.30%
ATTTGCATTGAGATAG--TGGGGAAGG	17	-2	0.23%
ATTTGCATT--------GTGGGGAAGG	14	-8	0.19%
ATTTGCATTGAGATAGTGT--GGAAGG	12	-2	0.16%
ATTTGCATTGAGAT----------AGG	12	-10	0.16%
ATTTGCATT------GTGTGGGGAAGG	10	-6	0.14%

P41-P50-WT-U937-Syn49crHBG2-i-KO-BC11

4h — Total | INDEL: 151938 | 0.77% — Ins 0.20% — Del 0.57% — HDR 0.00%

Typical seqs	Reads	Type	pct.
TATTTGCATTGAGATAG|TGT**GGG**GAAG	150767	WT	99.23%
TATTTGCATTGAGATAGtTGTGGGGAAG	123	+1 t	0.08%
TATTTGCATTGAGAT--TGTGGGGAAG	101	-2	0.07%
TATTTGCATTGAGATAAGTGTGGGGAAG	99	+1 Ins	0.07%
TATTTGCATTGAGATA--GTGGGGAAG	89	-2	0.06%
TATTTGCATTGAGA---TGTGGGGAAG	79	-3	0.05%
TATTTGCAT--------TGTGGGGAAG	66	-8	0.04%
TATTTGCATTGAGATA-TGTGGGGAAG	43	-1	0.03%
TATTTGCATTGAGATA----GGGGAAG	42	-4	0.03%
TATTTGCATTG------TGTGGGGAAG	43	-6	0.03%

8h — Total | INDEL: 212951 | 1.57% — Ins 0.36% — Del 1.20% — HDR 0.00%

Typical seqs	Reads	Type	pct.
TATTTGCATTGAGATAG|TGT**GGG**GAAG	209611	WT	98.43%
TATTTGCATTGAGA---TGTGGGGAAG	382	-3	0.18%
TATTTGCATTGAGATAAGTGTGGGGAAG	270	+1 Ins	0.13%
TATTTGCATTGAGATAGtTGTGGGGAAG	259	+1 t	0.12%
TATTTGCATTGAGAT--TGTGGGGAAG	216	-2	0.10%
TATTTGCAT--------TGTGGGGAAG	194	-8	0.09%
TATTTGCATTGAGATA--GTGGGGAAG	180	-2	0.08%
TATTTGCATTGAGATA-TGTGGGGAAG	117	-1	0.05%
TATTTGCATTG------TGTGGGGAAG	111	-6	0.05%
TATTTGCATTGAGATAGTGT--GGAAG	89	-2	0.04%

12h — Total | INDEL: 144367 | 2.16% — Ins 0.57% — Del 1.59% — HDR 0.00%

Typical seqs	Reads	Type	pct.
TATTTGCATTGAGATAG|TGT**GGG**GAAG	141252	WT	97.84%
TATTTGCATTGAGATAGtTGTGGGGAAG	432	+1 t	0.30%
TATTTGCATTGAGA---TGTGGGGAAG	272	-3	0.19%
TATTTGCATTGAGAT--TGTGGGGAAG	260	-2	0.18%
TATTTGCATTGAGATA--GTGGGGAAG	174	-2	0.12%
TATTTGCAT--------TGTGGGGAAG	155	-8	0.11%
TATTTGCATTGAGATAAGTGTGGGGAAG	118	+1 Ins	0.08%
TATTTGCATTG------TGTGGGGAAG	100	-6	0.07%
TATTTGCATTGAGATAGTGT--GGAAG	94	-2	0.07%
TATTTGCATTGAGATA-TGTGGGGAAG	90	-1	0.06%

24h — Total | INDEL: 145896 | 6.31% — Ins 1.19% — Del 5.12% — HDR 0.00%

Typical seqs	Reads	Type	pct.
TATTTGCATTGAGATAG|TGT**GGG**GAAG	136697	WT	93.69%
TATTTGCAT--------TGTGGGGAAG	799	-8	0.55%
TATTTGCATTGAGATAGtTGTGGGGAAG	668	+1 t	0.46%
TATTTGCATTGAGA---TGTGGGGAAG	617	-3	0.42%
TATTTGCATTGAGAT--TGTGGGGAAG	530	-2	0.36%
TATTTGCATTGAGATA----GGGGAAG	440	-4	0.30%
TATTTGCATTGAGATA--GTGGGGAAG	354	-2	0.24%
TATTTGCATTGAGATAGTGT--GGAAG	265	-2	0.18%
TATTTGCATTGAGATA-TGTGGGGAAG	249	-1	0.17%
TATTTGCATTGAGAT----------AG	210	-10	0.14%

48h — Total | INDEL: 145506 | 7.72% — Ins 1.37% — Del 6.35% — HDR 0.00%

Typical seqs	Reads	Type	pct.
TATTTGCATTGAGATAG|TGT**GGG**GAAG	134270	WT	92.28%
TATTTGCAT--------TGTGGGGAAG	1221	-8	0.84%
TATTTGCATTGAGA---TGTGGGGAAG	684	-3	0.47%
TATTTGCATTGAGATA----GGGGAAG	661	-4	0.45%
TATTTGCATTGAGAT--TGTGGGGAAG	649	-2	0.45%
TATTTGCATTGAGATAGtTGTGGGGAAG	619	+1 t	0.43%
TATTTGCATTGAGATA--GTGGGGAAG	425	-2	0.29%
TATTTGCATTGAGAT----------AG	299	-10	0.21%
TATTTGCATTGAGATA-TGTGGGGAAG	287	-1	0.20%
TATTTGCATTGAGATAGTGT--GGAAG	272	-2	0.19%

P41-P50-WT-U937-Syn50crHBG2-j-KO-BC12

Total | INDEL	4h	Ins	Del	HDR
86922 | 0.58%		0.37%	0.21%	0.00%

Typical seqs	Reads	Type	pct.
ATATTTGCATTGAGATA|GTGTGGGGAA	86416	WT	99.42%
ATATTTGCATTGAGATAaGTGTGGGGAA	184	+1 a	0.21%
ATATTTGCATTGAGATAgTTGTGGGGAA	75	+1 g	0.09%
ATATTTGCATTGAGATATAGTGTGGGGAA	22	+2	0.03%
ATATTTGCATTGAGA--GTGTGGGGAA	12	-2	0.01%
ATATTTGCAT--------TGTGGGGAA	13	-8	0.01%
ATATTTGCATTGAGATA--GTGGGGAA	11	-2	0.01%
ATATTTGCATTGA--------GGGGAA	9	-8	0.01%
ATATTTGCATTGAG----TGTGGGGAA	8	-4	0.01%
ATATTTGCATTGAGATAtGTGTGGGGAA	8	+1 t	0.01%

Total | INDEL	8h	Ins	Del	HDR
85927 | 1.42%		0.76%	0.67%	0.00%

Typical seqs	Reads	Type	pct.
ATATTTGCATTGAGATA|GTGTGGGGAA	84706	WT	98.58%
ATATTTGCATTGAGATAaGTGTGGGGAA	300	+1 a	0.35%
ATATTTGCATTGAGATAgTTGTGGGGAA	234	+1 g	0.27%
ATATTTGCAT--------TGTGGGGAA	37	-8	0.04%
ATATTTGCATTGAGATATAGTGTGGGGAA	32	+2	0.04%
ATATTTGCATTGAGATA--GTGGGGAA	30	-2	0.03%
ATATTTGCATTGAGA--GTGTGGGGAA	27	-2	0.03%
ATATTTGCATT------GTGTGGGGAA	26	-6	0.03%
ATATTTGCATTGAG----TGTGGGGAA	26	-4	0.03%
ATATTTGCATTGAGA---TGTGGGGAA	24	-3	0.03%

Total | INDEL	12h	Ins	Del	HDR
109173 | 1.06%		0.54%	0.51%	0.00%

Typical seqs	Reads	Type	pct.
ATATTTGCATTGAGATA|GTGTGGGGAA	108018	WT	98.94%
ATATTTGCATTGAGATAaGTGTGGGGAA	347	+1 a	0.32%
ATATTTGCATTGAGATAgTTGTGGGGAA	124	+1 g	0.11%
ATATTTGCAT--------TGTGGGGAA	38	-8	0.03%
ATATTTGCATTGAG----TGTGGGGAA	35	-4	0.03%
ATATTTGCATTGAGATATAGTGTGGGGAA	31	+2	0.03%
ATATTTGCATTGAGATA--GTGGGGAA	25	-2	0.02%
ATATTTGCATTGAGA---TGTGGGGAA	24	-3	0.02%
ATATTTGCATTGAGA--GTGTGGGGAA	21	-2	0.02%
ATATTTGCATT------GTGTGGGGAA	20	-6	0.02%

Total | INDEL	24h	Ins	Del	HDR
80410 | 3.53%		1.28%	2.26%	0.00%

Typical seqs	Reads	Type	pct.
ATATTTGCATTGAGATA|GTGTGGGGAA	77569	WT	96.47%
ATATTTGCATTGAGATAaGTGTGGGGAA	422	+1 a	0.52%
ATATTTGCATTGAGATAgTTGTGGGGAA	331	+1 g	0.41%
ATATTTGCAT--------TGTGGGGAA	140	-8	0.17%
ATATTTGCATTGAGA---TGTGGGGAA	80	-3	0.10%
ATATTTGCATTGAG----TGTGGGGAA	81	-4	0.10%
ATATTTGCATT------GTGTGGGGAA	67	-6	0.08%
ATATTTGCATTGAGATA-TGTGGGGAA	53	-1	0.07%
ATATTTGCATTGAGATA--GTGGGGAA	48	-2	0.06%
ATATTTGCATTGAGAT-GTGTGGGGAA	48	-1	0.06%

Total | INDEL	48h	Ins	Del	HDR
69250 | 3.81%		1.34%	2.47%	0.00%

Typical seqs	Reads	Type	pct.
ATATTTGCATTGAGATA|GTGTGGGGAA	66615	WT	96.19%
ATATTTGCATTGAGATAaGTGTGGGGAA	473	+1 a	0.68%
ATATTTGCATTGAGATAgTTGTGGGGAA	231	+1 g	0.33%
ATATTTGCAT--------TGTGGGGAA	121	-8	0.17%
ATATTTGCATT------GTGTGGGGAA	81	-6	0.12%
ATATTTGCATTGAG----TGTGGGGAA	81	-4	0.12%
ATATTTGCATTGAGA---TGTGGGGAA	71	-3	0.10%
ATATTTGCATTGAGAT-GTGTGGGGAA	61	-1	0.09%
ATATTTGCATTGA-----------GAA	55	-11	0.08%
ATATTTGCATTGAGA--GTGTGGGGAA	44	-2	0.06%

P58-P67-WT-U937-Syn20crAAVS1c-KO-48h-BC1

4h — Total | INDEL: 81564 | 23.24% — Ins 19.27% — Del 3.98% — HDR 0.00%

Typical seqs	Reads	Type	pct.
TCTAACCCCCACCTCCT|GTTAGGCAGA	61792	WT	75.76%
TCTAACCCCCACCTCCTtGTTAGGCAGA	13726	+1 t	16.83%
TCTAACCCCCACCTCC--TTAGGCAGA	387	-2	0.47%
TCTAACCCCCACCTC-TGTTAGGCAGA	292	-1	0.36%
TCTAACCCCCACCT---GTTAGGCAGA	198	-3	0.24%
TCTAACCCCCACCTCCTgGTTAGGCAGA	193	+1 g	0.24%
TCTAACCCCCACCTC--GTTAGGCAGA	154	-2	0.19%
TCTAACCCCCACCTCC-GTTAGGCAGA	134	-1	0.16%
TCTAACCCCCACCT--TGTTAGGCAGA	129	-2	0.16%

8h — Total | INDEL: 205075 | 50.7% — Ins 34.55% — Del 16.15% — HDR 0.00%

Typical seqs	Reads	Type	pct.
TCTAACCCCCACCTCCT|GTTAGGCAGA	89471	WT	43.63%
TCTAACCCCCACCTCCTtGTTAGGCAGA	58197	+1 t	28.38%
TCTAACCCCCACCTCC--TTAGGCAGA	2287	-2	1.12%
TCTAACCCCCACCTC-TGTTAGGCAGA	2031	-1	0.99%
TCTAACCCCCACCT---GTTAGGCAGA	1505	-3	0.73%
TCTAACCCCCACCTC--GTTAGGCAGA	1352	-2	0.66%
TCTAACCCCCACCTCCTgGTTAGGCAGA	1239	+1 g	0.60%
TCTAACCCCCACCTCCT-TTAGGCAGA	1074	-1	0.52%
TCTAACCCCCACCTCC-GTTAGGCAGA	1037	-1	0.51%

12h — Total | INDEL: 102908 | 60.06% — Ins 35.22% — Del 24.85% — HDR 0.00%

Typical seqs	Reads	Type	pct.
TCTAACCCCCACCTCCT|GTTAGGCAGA	32774	WT	31.85%
TCTAACCCCCACCTCCTtGTTAGGCAGA	29763	+1 t	28.92%
TCTAACCCCCACCT---GTTAGGCAGA	1352	-3	1.31%
TCTAACCCCCACCTCC--TTAGGCAGA	1330	-2	1.29%
TCTAACCCCCACCTC-TGTTAGGCAGA	1015	-1	0.99%
TCTAACCCCCACC--------------	1010	-15	0.98%
TCTAACCCCCACCTCCTgGTTAGGCAGA	731	+1 g	0.71%
TCTAACCCCCA----------------	729	-18	0.71%
TCTAACCCCCACCTC--GTTAGGCAGA	655	-2	0.64%

24h — Total | INDEL: 74311 | 62.97% — Ins 49.65% — Del 13.32% — HDR 0.00%

Typical seqs	Reads	Type	pct.
TCTAACCCCCACCTCCT|GTTAGGCAGA	26449	WT	35.59%
TCTAACCCCCACCTCCTtGTTAGGCAGA	32171	+1 t	43.29%
TCTAACCCACGCGTAGTTTAAACTTAGGCAGA	1070	+5	1.44%
TCTAACCCCCACCTCC--TTAGGCAGA	939	-2	1.26%
TCTAACCCCCACCTC-TGTTAGGCAGA	899	-1	1.21%
TCTACCCCCCACCTCCTTGTTAGGCAGA	662	+1 Ins	0.89%
TCTAACCCCCACCTC--GTTAGGCAGA	500	-2	0.67%
TCTAACCCCCACCTCC-GTTAGGCAGA	465	-1	0.63%
TCTAACCCCCACCT---GTTAGGCAGA	405	-3	0.55%
TCTAACCCCCA----CTGTTAGGCAGA	405	-4	0.55%

48h — Total | INDEL: 42482 | 83.10% — Ins 54.47% — Del 28.63% — HDR 0.00%

Typical seqs	Reads	Type	pct.
TCTAACCCCCACCTCCT|GTTAGGCAGA	6456	WT	15.20%
TCTAACCCCCACCTCCTtGTTAGGCAGA	20030	+1 t	47.15%
TCTAACCCCCACCTCC--TTAGGCAGA	704	-2	1.66%
TCTAACCCCCACCTC-TGTTAGGCAGA	569	-1	1.34%
TCTAACCCCCACC--------------	478	-15	1.13%
TCTAACCCCCA----------------	399	-18	0.94%
TCTAACCCCCAC-----------CAGA	350	-11	0.82%
TCTAACCCCCACCT---GTTAGGCAGA	350	-3	0.82%
TCTAACCCCCACCT-----TAGGCAGA	336	-5	0.79%

P58-P67-WT-U937-Syn20crAAVS1c-AAV6-KI-BC7

Total | INDEL	4h	Ins	Del	HDR
77340 | 29.23%		**20.07%**	**3.50%**	**5.66%**
**Typical seqs**	**Reads**	**Type**	**pct.**	
TCTAACCCCCACCTCCT|GTT**AGG**CAGA	54730	WT	70.77%	
TCTAACCCCCACCTCCTtGTTAGGCAGA	12910	+1 t	16.69%	
TCTAACCCacgcgtagtttaaacTTAGGCAGA	4043	+5 HDR	5.23%	
TCTAACCCCCACCTC-TGTTAGGCAGA	311	-1	0.40%	
TCTAACCCCCACCTCC--TTAGGCAGA	294	-2	0.38%	
TCTAACCCCCACCTCCTgGTTAGGCAGA	219	+1 g	0.28%	
TCTAACCCCCACCT---GTTAGGCAGA	220	-3	0.28%	
TCTAACCCCCACCTC--GTTAGGCAGA	126	-2	0.16%	
TCTAACCCCCACCTCC-GTTAGGCAGA	129	-1	0.17%	
TCTAACCCCCA----CTGTTAGGCAGA	121	-4	0.16%	

Total | INDEL	8h	Ins	Del	HDR
191390 | 51.18%		**25.83%**	**7.87%**	**17.48%**
**Typical seqs**	**Reads**	**Type**	**pct.**	
TCTAACCCCCACCTCCT|GTT**AGG**CAGA	93428	WT	48.82%	
TCTAACCCCCACCTCCTtGTTAGGCAGA	38508	+1 t	20.12%	
TCTAACCCacgcgtagtttaaacTTAGGCAGA	30727	+5 HDR	16.0[illegible]%	
TCTAACCCCCACCTC-TGTTAGGCAGA	1287	-1	0.67%	
TCTAACCCCCACCTCC--TTAGGCAGA	1254	-2	0.66%	
TCTAACCCCCACCTCCTgGTTAGGCAGA	818	+1 g	0.43%	
TCTAACCCCCACCTCCT-TTAGGCAGA	753	-1	0.39%	
TCTAACCCCCACCT---GTTAGGCAGA	717	-3	0.37%	
TCTAACCCCCA----CTGTTAGGCAGA	630	-4	0.33%	
TCTACCCCCCACCTCCTTGTTAGGCAGA	709	+1 Ins	0.37%	

Total | INDEL	12h	Ins	Del	HDR
165145 | 68.09%		**33.41%**	**10.36%**	**24.32%**
**Typical seqs**	**Reads**	**Type**	**pct.**	
TCTAACCCCCACCTCCT|GTT**AGG**CAGA	52696	WT	31.91%	
TCTAACCCCCACCTCCTtGTTAGGCAGA	43406	+1 t	26.28%	
TCTAACCCacgcgtagtttaaacTTAGGCAGA	37066	+5 HDR	22.44%	
TCTAACCCCCACCTCC--TTAGGCAGA	1345	-2	0.81%	
TCTAACCCCCACCTC-TGTTAGGCAGA	1330	-1	0.81%	
TCTAACCCCCACCTC--GTTAGGCAGA	921	-2	0.56%	
TCTAACCCCCACCT---GTTAGGCAGA	834	-3	0.51%	
TCTAACCCCCACCTCCTgGTTAGGCAGA	787	+1 g	0.48%	
TCTAACCCCCACCTCC-GTTAGGCAGA	714	-1	0.43%	
TCTAACCCCCACCTCCT-TTAGGCAGA	643	-1	0.39%	

Total | INDEL	24h	Ins	Del	HDR
202120 | 68.48%		**28.28%**	**13.15%**	**27.05%**
**Typical seqs**	**Reads**	**Type**	**pct.**	
TCTAACCCCCACCTCCT|GTT**AGG**CAGA	63712	WT	31.52%	
TCTAACCCacgcgtagtttaaacTTAGGCAGA	50469	+5 HDR	24.97%	
TCTAACCCCCACCTCCTtGTTAGGCAGA	42917	+1 t	21.23%	
TCTAACCCCCACCTCC--TTAGGCAGA	1874	-2	0.93%	
TCTAACCCCCACCT---GTTAGGCAGA	1749	-3	0.87%	
TCTAACCCCCACCTCCTgGTTAGGCAGA	1393	+1 g	0.69%	
TCTAACCCCCACCTC-TGTTAGGCAGA	1329	-1	0.66%	
TCTAACCCCCACCTCCT-TTAGGCAGA	1041	-1	0.52%	
TCTAACCCCCACCTC--GTTAGGCAGA	928	-2	0.46%	
TCTAACCCCCACCTCC-GTTAGGCAGA	740	-1	0.37%	

Total | INDEL	48h	Ins	Del	HDR
85807 | 70.50%		**28.67%**	**14.27%**	**27.56%**
**Typical seqs**	**Reads**	**Type**	**pct.**	
TCTAACCCCCACCTCCT|GTT**AGG**CAGA	25314	WT	29.50%	
TCTAACCCacgcgtagtttaaacTTAGGCAGA	21971	+5 HDR	25.61%	
TCTAACCCCCACCTCCTtGTTAGGCAGA	19085	+1 t	22.24%	
TCTAACCCCCACCT---GTTAGGCAGA	914	-3	1.07%	
TCTAACCCCCACCTCC--TTAGGCAGA	859	-2	1.00%	
TCTAACCCCCACCTCCTgGTTAGGCAGA	578	+1 g	0.67%	
TCTAACCCCCACCTC-TGTTAGGCAGA	536	-1	0.62%	
TCTAACCCCCACCTC--GTTAGGCAGA	481	-2	0.56%	
TCTAACCCCCACCTCCT-TTAGGCAGA	420	-1	0.49%	
TCTAACCCCCACCTCC-GTTAGGCAGA	419	-1	0.49%	

P58-P67-WT-U937-Syn21crAAVS1d-KO-BC2

Total | INDEL	4h	Ins	Del	HDR
1198 | 5.43%		0.75%	4.67%	0.00%

Typical seqs	Reads	Type	pct.
ATCTGCCTAACAGGAGG|TGGGGGTTAG	1133	WT	94.57%
ATCTGCCTAACAGGAG-TGGGGGTTAG	8	-1	0.67%
ATCTGCCTAACAGGA--TGGGGGTTAG	7	-2	0.58%
ATCTGCCTAACAGGAG--GGGGGTTAG	6	-2	0.50%
ATCTGCCTAACAGGAGGT-GGGGTTAG	6	-1	0.50%
ATCTGCCTAACAGGA----GGGGTTAG	4	-4	0.33%
ATCTGCCTAACAGGA------GGTTAG	4	-6	0.33%
ATCTGCCTAACA-----TGGGGGTTAG	4	-5	0.33%
ATCTGCCTAACAGGA---GGGGGTTAG	4	-3	0.33%
ATCTGCCTAACAGGA-ATGGGGGTTAG	4	-1	0.33%

Total | INDEL	8h	Ins	Del	HDR
191013 | 10.56%		1.98%	8.58%	0.00%

Typical seqs	Reads	Type	pct.
ATCTGCCTAACAGGAGG|TGGGGGTTAG	163222	WT	85.45%
ATCTGCCTAACAGGA--TGGGGGTTAG	2222	-2	1.16%
ATCTGCCTAACAGGAG-TGGGGGTTAG	1204	-1	0.63%
ATCTGCCTAACAGGA---GGGGGTTAG	783	-3	0.41%
ATCTGCCTAACAGGAGGgTGGGGGTTAG	759	+1 g	0.40%
ATCTGCCTAACA-----TGGGGGTTAG	727	-5	0.38%
ATCTGCCTAACA----GTGGGGGTTAG	636	-4	0.33%
ATCTGCCTAACAGGAGGT-GGGGTTAG	644	-1	0.34%
ATCTGCCTAACAGGA-----GGGTTAG	579	-5	0.30%

Total | INDEL	12h	Ins	Del	HDR
226137 | 15.99%		2.57%	13.42%	0.00%

Typical seqs	Reads	Type	pct.
ATCTGCCTAACAGGAGG|TGGGGGTTAG	177703	WT	78.58%
ATCTGCCTAACAGGA--TGGGGGTTAG	3246	-2	1.44%
ATCTGCCTAACAGGAG-TGGGGGTTAG	1966	-1	0.87%
ATCTGCCTAACAGGA---GGGGGTTAG	1610	-3	0.71%
ATCTGCCTAACAGGA------GGTTAG	1392	-6	0.62%
ATCTGCCTAACAGGA-----GGGTTAG	1297	-5	0.57%
ATCTGCCTAACAGGAGGgTGGGGGTTAG	1059	+1 g	0.47%
ATCTGCCTAACA-----TGGGGGTTAG	1061	-5	0.47%
ATCTGCCTAACA----GTGGGGGTTAG	1029	-4	0.46%

Total | INDEL	24h	Ins	Del	HDR
221197 | 26.59%		3.32%	23.26%	0.00%

Typical seqs	Reads	Type	pct.
ATCTGCCTAACAGGAGG|TGGGGGTTAG	149623	WT	67.64%
ATCTGCCTAACAGGA--TGGGGGTTAG	4213	-2	1.90%
ATCTGCCTAACAGGA------GGTTAG	3275	-6	1.48%
ATCTGCCTAACAGGA---GGGGGTTAG	3190	-3	1.44%
ATCTGCCTAACAGGA-----GGGTTAG	2722	-5	1.23%
ATCTGCCTAACAGGAG-TGGGGGTTAG	2249	-1	1.02%
ATCTGCCTAACAGGA----GGGGTTAG	1734	-4	0.78%
ATCTGCCTAAC------TGGGGGTTAG	1489	-6	0.67%
ATCTGCCTAAC---AGGTGGGGGTTAG	1418	-3	0.64%

Total | INDEL	48h	Ins	Del	HDR
61454 | 33.62%		3.06%	30.56%	0.00%

Typical seqs	Reads	Type	pct.
ATCTGCCTAACAGGAGG|TGGGGGTTAG	39639	WT	64.50%
ATCTGCCTAACAGGA--TGGGGGTTAG	1801	-2	2.93%
ATCTGCCTAACAGGA------GGTTAG	1414	-6	2.30%
ATCTGCCTAACAGGA---GGGGGTTAG	1391	-3	2.26%
ATCTGCCTAACAGGA-----GGGTTAG	1145	-5	1.86%
ATCTGCCTAACAGGAG-TGGGGGTTAG	956	-1	1.56%
ATCTGCCTAACAGGA----GGGGTTAG	714	-4	1.16%
ATCTGCCTAAC------TGGGGGTTAG	593	-6	0.96%
ATCTGCCTAACA-----TGGGGGTTAG	537	-5	0.87%

P58-P67-WT-U937-Syn21crAAVS1d-AAV6-KI-BC8

4h — Total 83814 | INDEL 6.53% — Ins 0.87% | Del 3.24% | HDR 2.42%

Typical seqs	Reads	Type	pct.
ATCTGCCTAACAGGAGGTGG**GGG**TTAG	78344	WT	93.47%
ATCTGCCTAAgtttaaactacgcgtGGGTTAGA	1888	+5 HDR	2.25%
ATCTGCCTAACAGGAGGT-GGGGTTAG	383	-1	0.46%
ATCTGCCTAACAGGA--TGGGGGTTAG	367	-2	0.44%
ATCTGCCTAACAGGAG-TGGGGGTTAG	262	-1	0.31%
ATCTGCCTAACAGGAGGgTGGGGGTTAG	214	+1 g	0.26%
ATCTGCCTAACA-----TGGGGGTTAG	121	-5	0.14%
ATCTGCCTAACAGGAGGtTGGGGGTTAG	103	+1 t	0.12%
ATCTGCCTAAC---AGGTGGGGGTTAG	100	-3	0.12%
ATCTGCCTAAC------TGGGGGTTAG	82	-6	0.10%

8h — Total 202546 | INDEL 21.78% — Ins 2.15% | Del 7.73% | HDR 11.91%

Typical seqs	Reads	Type	pct.
ATCTGCCTAACAGGAGGTGG**GGG**TTAG	158425	WT	78.22%
ATCTGCCTAAgtttaaactacgcgtGGGTTAGA	22155	+5 HDR	10.94%
ATCTGCCTAACAGGA--TGGGGGTTAG	1991	-2	0.98%
ATCTGCCTAACAGGAG-TGGGGGTTAG	1309	-1	0.65%
ATCTGCCTAACA----GTGGGGGTTAG	804	-4	0.40%
ATCTGCCTAACAGGAGGgTGGGGGTTAG	777	+1 g	0.38%
ATCTGCCTAAC------TGGGGGTTAG	751	-6	0.37%
ATCTGCCTAACAGGAGGT-GGGGTTAG	706	-1	0.35%
ATCTGCCTAACA-----TGGGGGTTAG	717	-5	0.35%
ATCTGCCTAACAGGA-ATGGGGGTTAG	565	-1	0.28%

12h — Total 171818 | INDEL 31.64% — Ins 2.61% | Del 11.17% | HDR 17.86%

Typical seqs	Reads	Type	pct.
ATCTGCCTAACAGGAGGTGG**GGG**TTAG	117453	WT	68.36%
ATCTGCCTAAgtttaaactacgcgtGGGTTAGA	28298	+5 HDR	16.47%
ATCTGCCTAACAGGA--TGGGGGTTAG	2767	-2	1.61%
ATCTGCCTAACAGGAG-TGGGGGTTAG	1655	-1	0.96%
ATCTGCCTAACA-----TGGGGGTTAG	1137	-5	0.66%
ATCTGCCTAACA----GTGGGGGTTAG	905	-4	0.53%
ATCTGCCTAAC------TGGGGGTTAG	828	-6	0.48%
ATCTGCCTAACAGGAGGgTGGGGGTTAG	690	+1 g	0.40%
ATCTGCCTAACAGGA---GGGGGTTAG	657	-3	0.38%
ATCTGCCTAAC---AGGTGGGGGTTAG	563	-3	0.33%

24h — Total 23735 | INDEL 46.81% — Ins 3.35% | Del 15.12% | HDR 28.34%

Typical seqs	Reads	Type	pct.
ATCTGCCTAACAGGAGGTGG**GGG**TTAG	12624	WT	53.19%
ATCTGCCTAAgtttaaactacgcgtGGGTTAGA	6239	+5 HDR	26.29%
ATCTGCCTAACAGGA--TGGGGGTTAG	580	-2	2.44%
ATCTGCCTAACAGGAG-TGGGGGTTAG	293	-1	1.23%
ATCTGCCTAACA-----TGGGGGTTAG	212	-5	0.89%
ATCTGCCTAAC------TGGGGGTTAG	176	-6	0.74%
ATCTGCCTAACAGGA-ATGGGGGTTAG	148	-1	0.62%
ATCTGCCTAACA----GTGGGGGTTAG	146	-4	0.62%
ATCTGCCTAACAGGA------GGTTAG	140	-6	0.59%
ATCTGCCTAACAGGA---GGGGGTTAG	127	-3	0.54%

48h — Total 376 | INDEL 47.07% — Ins 4.26% | Del 13.03% | HDR 29.79%

Typical seqs	Reads	Type	pct.
ATCTGCCTAACAGGAGGTGG**GGG**TTAG	199	WT	52.93%
ATCTGCCTAAgtttaaactacgcgtGGGTTAGA	112	+5 HDR	29.79%
ATCTGCCTAACAGGA--TGGGGGTTAG	11	-2	2.93%
ATCTGCCTAACAGGAG-TGGGGGTTAG	5	-1	1.33%
ATCTGCCTAACA-----TGGGGGTTAG	5	-5	1.33%
ATCTGCCTAACAGGA-ATGGGGGTTAG	4	-1	1.06%
ATCTGCCTAACAGGAGGgTGGGGGTTAG	3	+1 g	0.80%
ATCTGCCTAACAG-------------G	3	-13	0.80%
ATCTGCCTAACAGGA----GGGGTTAG	3	-4	0.80%

P58-P67-WT-U937-Syn22crAAVS1e-KO-BC3

4h

Total	INDEL	Ins	Del	HDR
94477	5.75%	3.46%	2.29%	0.00%

Typical seqs	Reads	Type	pct.
AATCTGCCTAACAGGAG|GTG**GGG**GTTA	88210	WT	93.37%
AATCTGCCTAACAAGGAGGTGGGGGTTA	1881	+1 Ins	1.99%
AATCTGCCTAACAGGAGgGTGGGGGTTA	726	+1 g	0.77%
AATCTGCCTAACAGGA-GTGGGGGTTA	362	-1	0.38%
AATCTGCCTAACAGG---TGGGGGTTA	254	-3	0.27%
AATCTGCCTA--AGGAGGTGGGGGTTA	99	-2	0.10%
AATCTGCCTAACA----GTGGGGGTTA	77	-4	0.08%
AATCTGCCTAACA-GAGGTGGGGGTTA	68	-1	0.07%
AATCTGCCTAACAGGA--TGGGGGTTA	74	-2	0.08%

8h

Total	INDEL	Ins	Del	HDR
209618	11.87%	6.37%	5.50%	0.00%

Typical seqs	Reads	Type	pct.
AATCTGCCTAACAGGAG|GTG**GGG**GTTA	176494	WT	84.20%
AATCTGCCTAACAAGGAGGTGGGGGTTA	6358	+1 Ins	3.03%
AATCTGCCTAACAGGAGgGTGGGGGTTA	3114	+1 g	1.49%
AATCTGCCTAACAGGA-GTGGGGGTTA	1221	-1	0.58%
AATCTGCCTAACAGG---TGGGGGTTA	1078	-3	0.51%
AATCTGCCTA--AGGAGGTGGGGGTTA	481	-2	0.23%
AATCTGCCTAA-AGGAGGTGGGGGTTA	394	-1	0.19%
AATCTGCCTAACA----GTGGGGGTTA	361	-4	0.17%
AATCTGCCTAACAGGA--TGGGGGTTA	338	-2	0.16%

12h

Total	INDEL	Ins	Del	HDR
231992	16.31%	7.44%	8.87%	0.00%

Typical seqs	Reads	Type	pct.
AATCTGCCTAACAGGAG|GTG**GGG**GTTA	181937	WT	78.42%
AATCTGCCTAACAAGGAGGTGGGGGTTA	7580	+1 Ins	3.27%
AATCTGCCTAACAGGAGgGTGGGGGTTA	3943	+1 g	1.70%
AATCTGCCTAACAGG---TGGGGGTTA	1927	-3	0.83%
AATCTGCCTAACAGGA-GTGGGGGTTA	1792	-1	0.77%
AATCTGCCTA--AGGAGGTGGGGGTTA	642	-2	0.28%
AATCTGCCTAA-AGGAGGTGGGGGTTA	625	-1	0.27%
AATCTGCCTAACA----GTGGGGGTTA	552	-4	0.24%
AATCTGCCTAACAGGA--TGGGGGTTA	537	-2	0.23%

24h

Total	INDEL	Ins	Del	HDR
153823	21.15%	6.3%	14.85%	0.00%

Typical seqs	Reads	Type	pct.
AATCTGCCTAACAGGAG|GTG**GGG**GTTA	119000	WT	77.36%
AATCTGCCTAACAGGAGgGTGGGGGTTA	5155	+1 g	3.35%
AATCTGCCTAACAGGA-GTGGGGGTTA	1923	-1	1.25%
AATCTGCCTAACAGG---TGGGGGTTA	1686	-3	1.10%
AATCTGCCTAAC--------------A	835	-14	0.54%
AATCTGCCTAACA------GGGGGTTA	766	-6	0.50%
AATCTGCCTAACAAGGAGGTGGGGGTTA	819	+1 Ins	0.53%
AATCTGCCTAACA---------GGTTA	771	-9	0.50%
AATCTGCCTAACAGGA------GGTTA	605	-6	0.39%

48h

Total	INDEL	Ins	Del	HDR
121178	27.05%	9.65%	17.39%	0.00%

Typical seqs	Reads	Type	pct.
AATCTGCCTAACAGGAG|GTG**GGG**GTTA	79280	WT	65.42%
AATCTGCCTAACAAGgAGGTGGGGGTTA	4475	+1 Ins	3.69%
AATCTGCCTAACAGGAGgGTGGGGGTTA	2965	+1 g	2.45%
AATCTGCCTAACAGG---TGGGGGTTA	1706	-3	1.41%
AATCTGCCTAACAGGA-GTGGGGGTTA	1404	-1	1.16%
AATCTGCCTAAC--------------A	595	-14	0.49%
AATCTGCCTAACA------GGGGGTTA	604	-6	0.50%
AATCTGCCTAACAGGA------GGTTA	568	-6	0.47%
AATCTGCCTA--AGGAGGTGGGGGTTA	540	-2	0.45%

P58-P67-WT-U937-Syn22crAAVS1e-AAV6-KI-BC9

4h

Total | INDEL	Ins	Del	HDR
96775 | 7.95%	3.48%	2.29%	2.18%

Typical seqs	Reads	Type	pct.
AATCTGCCTAACAGGAG|GTG**GGG**GTTA	89081	WT	92.05%
AATCTGCCTAACAAGGAGGTGGGGGTTA	2096	+1 Ins	2.17%
AATCTGCCTAAgtttaaactacgcgtGGGTTAG	1962	+5 HDR	2.03%
AATCTGCCTAACAGGAGgGTGGGGGTTA	553	+1 g	0.57%
AATCTGCCTAACAGGA-GTGGGGGTTA	333	-1	0.34%
AATCTGCCTAACAGG---TGGGGGTTA	263	-3	0.27%
AATCTGCCTA--AGGAGGTGGGGGTTA	95	-2	0.10%
AATCTGCCTAA-AGGAGGTGGGGGTTA	94	-1	0.10%
AATCTGCCTAACAGGA--TGGGGGTTA	86	-2	0.09%
AATCTGCCTAACA-GAGGTGGGGGTTA	74	-1	0.08%

8h

Total | INDEL	Ins	Del	HDR
220799 | 21.26%	6.26%	5.08%	9.91%

Typical seqs	Reads	Type	pct.
AATCTGCCTAACAGGAG|GTG**GGG**GTTA	173868	WT	78.74%
AATCTGCCTAAgtttaaactacgcgtGGGTTAG	20095	+5 HDR	9.10%
AATCTGCCTAACAAGGAGGTGGGGGTTA	6618	+1 Ins	3.00%
AATCTGCCTAACAGGAGgGTGGGGGTTA	2937	+1 g	1.33%
AATCTGCCTAACAGGA-GTGGGGGTTA	1120	-1	0.51%
AATCTGCCTAACAGG---TGGGGGTTA	989	-3	0.45%
AATCTGCCTA--AGGAGGTGGGGGTTA	489	-2	0.22%
AATCTGCCTAA-AGGAGGTGGGGGTTA	448	-1	0.20%
AATCTGCCTAACAGGA--TGGGGGTTA	404	-2	0.18%
AATCTGCCTAACAGGAAGGTGGGGGTTA	367	+1 Ins	0.17%

12h

Total | INDEL	Ins	Del	HDR
248886 | 29.45%	7.22%	7.63%	14.59%

Typical seqs	Reads	Type	pct.
AATCTGCCTAACAGGAG|GTG**GGG**GTTA	175596	WT	70.55%
AATCTGCCTAAgtttaaactacgcgtGGGTTAG	33535	+5 HDR	13.47%
AATCTGCCTAACAAGGAGGTGGGGGTTA	8304	+1 Ins	3.34%
AATCTGCCTAACAGGAGgGTGGGGGTTA	3627	+1 g	1.46%
AATCTGCCTAACAGGA-GTGGGGGTTA	1976	-1	0.79%
AATCTGCCTAACAGG---TGGGGGTTA	1672	-3	0.67%
AATCTGCCTA--AGGAGGTGGGGGTTA	795	-2	0.32%
AATCTGCCTAA-AGGAGGTGGGGGTTA	712	-1	0.29%
AATCTGCCTAACA----GTGGGGGTTA	583	-4	0.23%
AATCTGCCTAACAGGA--TGGGGGTTA	536	-2	0.22%

24h

Total | INDEL	Ins	Del	HDR
6757 | 34.73%	5.82%	6.26%	22.66%

Typical seqs	Reads	Type	pct.
AATCTGCCTAACAGGAG|GTG**GGG**GTTA	4410	WT	65.27%
AATCTGCCTAAgtttaaactacgcgtGGGTTAG	1448	+5 HDR	21.43%
AATCTGCCTAACAGGAGgGTGGGGGTTA	172	+1 g	2.55%
AATCTGCCTAACAGGA-GTGGGGGTTA	72	-1	1.07%
AATCTGCCTAACAGG---TGGGGGTTA	57	-3	0.84%
AATCTGCCTAACAAGGAGGTGGGGGTTA	33	+1 Ins	0.49%
AATCTGCCTAAC-----GTGGGGGTTA	15	-5	0.22%
AATCTGCCTAACA---------GGTTA	14	-9	0.21%
AATCTGCCTAAGTTGAAACTACGCGTGGGTTA	14	+5	0.21%
AATCTGCCTAAC----GGTGGGGGTTA	13	-4	0.19%

48h

Total | INDEL	Ins	Del	HDR
106996 | 42.13%	8.56%	13.14%	20.42%

Typical seqs	Reads	Type	pct.
AATCTGCCTAACAGGAG|GTG**GGG**GTTA	61921	WT	57.87%
AATCTGCCTAAgtttaaactacgcgtGGGTTAG	20268	+5 HDR	18.94%
AATCTGCCTAACAAGGAGGTGGGGGTTA	3644	+1 Ins	3.41%
AATCTGCCTAACAGGAGgGTGGGGGTTA	2087	+1 g	1.95%
AATCTGCCTAACAGG---TGGGGGTTA	1419	-3	1.33%
AATCTGCCTAACAGGA-GTGGGGGTTA	1049	-1	0.98%
AATCTGCCTA--AGGAGGTGGGGGTTA	479	-2	0.45%
AATCTGCCTAACAGGA--TGGGGGTTA	358	-2	0.33%
------------AGGAGGTGGGGGTTA	360	-15	0.34%
AATCTGCCTAA-AGGAGGTGGGGGTTA	370	-1	0.35%

P58-P67-WT-U937-Syn23crAAVS1f-KO-BC4

Total | INDEL	4h	Ins	Del	HDR
93351 | 5.57%		3.38%	2.19%	0.00%

Typical seqs	Reads	Type	pct.
GAATCTGCCTAACAGGA|GGTGGGGGTT	87283	WT	93.50%
GAATCTGCCTAACAAGGAGGTGGGGGTT	1897	+1 Ins	2.03%
GAATCTGCCTAACAGGAaGGTGGGGGTT	322	+1 a	0.34%
GAATCTGCCTAACA---GGTGGGGGTT	253	-3	0.27%
GAATCTGCCTAACCAGGAGGTGGGGGTT	247	+1 Ins	0.26%
GAATCTGCCTAACAGGA-GTGGGGGTT	173	-1	0.19%
GAATCTGCCTAACAGGA--TGGGGGTT	116	-2	0.12%
GAATCTGCCTAACAGGAgGGTGGGGGTT	115	+1 g	0.12%
GAATCTGCCTA--AGGAGGTGGGGGTT	87	-2	0.09%

Total | INDEL	8h	Ins	Del	HDR
44165 | 9.31%		2.87%	6.44%	0.00%

Typical seqs	Reads	Type	pct.
GAATCTGCCTAACAGGA|GGTGGGGGTT	39415	WT	89.24%
GAATCTGCCTAACAGGAaGGTGGGGGTT	613	+1 a	1.39%
GAATCTGCCTAACA---GGTGGGGGTT	561	-3	1.27%
GAATCTGCCTAACAG--GGTGGGGGTT	225	-2	0.51%
GAATCTGCCTAACAAGGAGGTGGGGGTT	176	+1 Ins	0.40%
GAATCTGCCTAAC----GGTGGGGGTT	121	-4	0.27%
GAATCTGCCTAACAGGA-GTGGGGGTT	121	-1	0.27%
GAATCTGCCTAACAGGA--TGGGGGTT	98	-2	0.22%
GAATCTGCCTAACAGGA--------TT	78	-8	0.18%

Total | INDEL	12h	Ins	Del	HDR
221866 | 19.04%		7.63%	11.41%	0.00%

Typical seqs	Reads	Type	pct.
GAATCTGCCTAACAGGA|GGTGGGGGTT	167512	WT	75.50%
GAATCTGCCTAACAAGGAGGTGGGGGTT	7567	+1 Ins	3.41%
GAATCTGCCTAACA---GGTGGGGGTT	3668	-3	1.65%
GAATCTGCCTAACAGGAaGGTGGGGGTT	2574	+1 a	1.16%
GAATCTGCCTAACCAGGAGGTGGGGGTT	1266	+1 Ins	0.57%
GAATCTGCCTAACAG--GGTGGGGGTT	991	-2	0.45%
GAATCTGCCTAACAGGA-GTGGGGGTT	966	-1	0.44%
GAATCTGCCTAACAGGA--TGGGGGTT	939	-2	0.42%
GAATCTGCCTA--AGGAGGTGGGGGTT	695	-2	0.31%

Total | INDEL	24h	Ins	Del	HDR
45158 | 28.84%		4.99%	23.86%	0.00%

Typical seqs	Reads	Type	pct.
GAATCTGCCTAACAGGA|GGTGGGGGTT	31392	WT	69.52%
GAATCTGCCTAACA---GGTGGGGGTT	2199	-3	4.87%
GAATCTGCCTAACAGGAaGGTGGGGGTT	1006	+1 a	2.23%
GAATCTGCCTAAC--------------	463	-14	1.03%
GAATCTGCCTAACA---------GGTT	395	-9	0.87%
GAATCTGCCTAACA------GGGGGTT	383	-6	0.85%
GAATCTGCCTAACAG--GGTGGGGGTT	330	-2	0.73%
GAATCTGCCTAACA--------GGGTT	301	-8	0.67%
GAATCTGCCTAACAGGA-GTGGGGGTT	253	-1	0.56%

Total | INDEL	48h	Ins	Del	HDR
72137 | 34.15%		7.08%	27.08%	0.00%

Typical seqs	Reads	Type	pct.
GAATCTGCCTAACAGGA|GGTGGGGGTT	44751	WT	62.04%
GAATCTGCCTAACA---GGTGGGGGTT	3909	-3	5.42%
GAATCTGCCTAACAGGAaGGTGGGGGTT	1591	+1 a	2.21%
GAATCTGCCTAACAaGGAGGTGGGGGTT	1211	+1 Ins	1.68%
GAATCTGCCTAACA------GGGGGTT	761	-6	1.05%
GAATCTGCCTAAC--------------	730	-14	1.01%
GAATCTGCCTAACA---------GGTT	580	-9	0.80%
GAATCTGCCTAACA--------GGGTT	531	-8	0.74%
GAATCTGCCTAACAG--GGTGGGGGTT	533	-2	0.74%

P58-P67-WT-U937-Syn23crAAVS1f-AAV6-KI-BC10

4h

Total	INDEL	Ins	Del	HDR
85511	8.16%	3.53%	2.27%	2.37%

Typical seqs	Reads	Type	pct.
GAATCTGCCTAACAGGAGGT**GGG**GGTT	78532	WT	91.84%
GAATCTGCCTAAGTTTAAACTACGCGTGGGTT	1894	+5 HDR	2.21%
GAATCTGCCTAACAAGGAGGTGGGGGTT	1830	+1 Ins	2.14%
GAATCTGCCTAACAGGAaGGTGGGGGTT	283	+1 a	0.33%
GAATCTGCCTAACCAGGAGGTGGGGGTT	213	+1 Ins	0.25%
GAATCTGCCTAACA---GGTGGGGGTT	167	-3	0.20%
GAATCTGCCTAACAGGA-GTGGGGGTT	169	-1	0.20%
GAATCTGCCTAACAG--GGTGGGGGTT	120	-2	0.14%
GAATCTGCCTAACAGGAgGGTGGGGGTT	96	+1 g	0.11%
GAATCTGCCTA--AGGAGGTGGGGGTT	104	-2	0.12%

8h

Total	INDEL	Ins	Del	HDR
207361	24.40%	6.37%	5.71%	12.32%

Typical seqs	Reads	Type	pct.
GAATCTGCCTAACAGGAGGT**GGG**GGTT	156757	WT	75.60%
GAATCTGCCTAAGTTTAAACTACGCGTGGGTT	23467	+5 HDR	11.32%
GAATCTGCCTAACAAGGAGGTGGGGGTT	6251	+1 Ins	3.01%
GAATCTGCCTAACAGGAaGGTGGGGGTT	1529	+1 a	0.74%
GAATCTGCCTAACA---GGTGGGGGTT	1129	-3	0.54%
GAATCTGCCTAACCAGGAGGTGGGGGTT	852	+1 Ins	0.41%
GAATCTGCCTAACAGGA-GTGGGGGTT	736	-1	0.35%
GAATCTGCCTAACAGGA--TGGGGGTT	600	-2	0.29%
GAATCTGCCTAACAGGAgGGTGGGGGTT	611	+1 g	0.29%
GAATCTGCCTAACAG--GGTGGGGGTT	484	-2	0.23%

12h

Total	INDEL	Ins	Del	HDR
225435	34.52%	7.72%	8.34%	18.45%

Typical seqs	Reads	Type	pct.
GAATCTGCCTAACAGGAGGT**GGG**GGTT	147619	WT	65.48%
GAATCTGCCTAAGTTTAAACTACGCGTGGGTT	38510	+5 HDR	17.08%
GAATCTGCCTAACAAGGAGGTGGGGGTT	7575	+1 Ins	3.36%
GAATCTGCCTAACAGGAaGGTGGGGGTT	2151	+1 a	0.95%
GAATCTGCCTAACA---GGTGGGGGTT	2114	-3	0.94%
GAATCTGCCTAACCAGGAGGTGGGGGTT	1207	+1 Ins	0.54%
GAATCTGCCTAACAGGA-GTGGGGGTT	845	-1	0.37%
GAATCTGCCTAACAGGAgGGTGGGGGTT	839	+1 g	0.37%
GAATCTGCCTAACAG--GGTGGGGGTT	830	-2	0.37%
GAATCTGCCTAACAGGA--TGGGGGTT	684	-2	0.30%

24h

Total	INDEL	Ins	Del	HDR
236905	46.78%	8.66%	13.38%	24.74%

Typical seqs	Reads	Type	pct.
GAATCTGCCTAACAGGAGGT**GGG**GGTT	126090	WT	53.22%
GAATCTGCCTAAGTTTAAACTACGCGTGGGTT	53747	+5 HDR	22.69%
GAATCTGCCTAACAAGGAGGTGGGGGTT	7607	+1 Ins	3.21%
GAATCTGCCTAACA---GGTGGGGGTT	3185	-3	1.34%
GAATCTGCCTAACAGGAaGGTGGGGGTT	2567	+1 a	1.08%
GAATCTGCCTAACCAGGAGGTGGGGGTT	1581	+1 Ins	0.67%
GAATCTGCCTAACAGGA-GTGGGGGTT	1222	-1	0.52%
GAATCTGCCTAACAGGA--TGGGGGTT	1157	-2	0.49%
GAATCTGCCTAACAG--GGTGGGGGTT	1138	-2	0.48%
GAATCTGCCTAACAGGAgGGTGGGGGTT	992	+1 g	0.42%

48h

Total	INDEL	Ins	Del	HDR
91400	50.36%	9.50%	14.76%	26.10%

Typical seqs	Reads	Type	pct.
GAATCTGCCTAACAGGAGGT**GGG**GGTT	45368	WT	49.64%
GAATCTGCCTAAGTTTAAACTACGCGTGGGTT	22221	+5 HDR	24.31%
GAATCTGCCTAACAaGGAGGTGGGGGTT	3392	+1 Ins	3.71%
GAATCTGCCTAACA---GGTGGGGGTT	1804	-3	1.97%
GAATCTGCCTAACAGGAaGGTGGGGGTT	1174	+1 a	1.28%
GAATCTGCCTAACCAGGAGGTGGGGGTT	530	+1 Ins	0.58%
GAATCTGCCTAACAGGAgGGTGGGGGTT	486	+1 g	0.53%
GAATCTGCCTAACAGGA--TGGGGGTT	453	-2	0.50%
GAATCTGCCTAACAGGA-GTGGGGGTT	457	-1	0.50%
GAATCTGCCTA--AGGAGGTGGGGGTT	409	-2	0.45%

P58-P67-WT-U937-Syn24crAAVS1g-KO-BC5

Total | INDEL	4h	Ins	Del	HDR
87383 | 4.98%		3.07%	1.92%	0.00%

Typical seqs	Reads	Type	pct.
GGAATCTGCCTAACAGG|AGG**TGG**GGGT	82285	WT	94.17%
GGAATCTGCCTAACAAGGAGGTGGGGGT	1899	+1 Ins	2.17%
GGAATCTGCCTAAC---AGGTGGGGGT	206	-3	0.24%
GGAATCTGCCTAACCAGGAGGTGGGGGT	203	+1 Ins	0.23%
GGAATCTGCCTAACAG-AGGTGGGGGT	125	-1	0.14%
GGAATCTGCCTAACAGGaGGGTGGGGGT	118	+1 a	0.14%
GGAATCTGCCTA--AGGAGGTGGGGGT	104	-2	0.12%
GGAATCTGCCTAACAGGA-GTGGGGGT	82	-1	0.09%
GGAATCTGCCTAA-AGGAGGTGGGGGT	78	-1	0.09%

Total | INDEL	8h	Ins	Del	HDR
168243 | 10.03%		5.19%	4.84%	0.00%

Typical seqs	Reads	Type	pct.
GGAATCTGCCTAACAGG|AGG**TGG**GGGT	144318	WT	85.78%
GGAATCTGCCTAACAAGGAGGTGGGGGT	5081	+1 Ins	3.02%
GGAATCTGCCTAAC---AGGTGGGGGT	892	-3	0.53%
GGAATCTGCCTAACCAGGAGGTGGGGGT	675	+1 Ins	0.40%
GGAATCTGCCTAACAG-AGGTGGGGGT	539	-1	0.32%
GGAATCTGCCTAACAGGaGGGTGGGGGT	417	+1 a	0.25%
GGAATCTGCCTAACA--AGGTGGGGGT	325	-2	0.19%
GGAATCTGCCTA--AGGAGGTGGGGGT	323	-2	0.19%
GGAATCTGCCTAA-AGGAGGTGGGGGT	301	-1	0.18%

Total | INDEL	12h	Ins	Del	HDR
206731 | 13.64%		6.1%	7.54%	0.00%

Typical seqs	Reads	Type	pct.
GGAATCTGCCTAACAGG|AGG**TGG**GGGT	167601	WT	81.07%
GGAATCTGCCTAACAAGGAGGTGGGGGT	7017	+1 Ins	3.39%
GGAATCTGCCTAAC---AGGTGGGGGT	1764	-3	0.85%
GGAATCTGCCTAACCAGGAGGTGGGGGT	1144	+1 Ins	0.55%
GGAATCTGCCTAACAG-AGGTGGGGGT	955	-1	0.46%
GGAATCTGCCTA--AGGAGGTGGGGGT	627	-2	0.30%
GGAATCTGCCTAA-AGGAGGTGGGGGT	613	-1	0.30%
GGAATCTGCCTAACA--AGGTGGGGGT	571	-2	0.28%
GGAATCTGCCTAACAGGaGGGTGGGGGT	541	+1 a	0.26%

Total | INDEL	24h	Ins	Del	HDR
26204 | 13.97%		1.67%	12.30%	0.00%

Typical seqs	Reads	Type	pct.
GGAATCTGCCTAACAGG|AGG**TGG**GGGT	22070	WT	84.22%
GGAATCTGCCTAAC---AGGTGGGGGT	1028	-3	3.92%
GGAATCTGCCTAACAG-AGGTGGGGGT	204	-1	0.78%
GGAATCTGCCTAACA--AGGTGGGGGT	154	-2	0.59%
GGAATCTGCCTAACAAGGAGGTGGGGGT	138	+1 Ins	0.53%
GGAATCTGCCTAACA------GGGGGT	78	-6	0.30%
GGAATCTGCCTAACAG--GGTGGGGGT	67	-2	0.26%
GGAATCTGCCTAA----AGGTGGGGGT	66	-4	0.25%
GGAATCTGCCTAAC-------------	62	-14	0.24%

Total | INDEL	48h	Ins	Del	HDR
164447 | 19.64%		6.58%	13.06%	0.00%

Typical seqs	Reads	Type	pct.
GGAATCTGCCTAACAGG|AGG**TGG**GGGT	120757	WT	73.43%
GGAATCTGCCTAACAaGGAGGTGGGGGT	5299	+1 Ins	3.22%
GGAATCTGCCTAAC---AGGTGGGGGT	3073	-3	1.87%
GGAATCTGCCTAACCAGGAGGTGGGGGT	1033	+1 Ins	0.63%
GGAATCTGCCTAACAG-AGGTGGGGGT	889	-1	0.54%
GGAATCTGCCTA--AGGAGGTGGGGGT	658	-2	0.40%
GGAATCTGCCTAACA--AGGTGGGGGT	614	-2	0.37%
GGAATCTGCCTAACAGGaGGGTGGGGGT	583	+1 a	0.35%
GGAATCTGCCTAA-AGGAGGTGGGGGT	580	-1	0.35%

P58-P67-WT-U937-Syn24crAAVS1g-AAV6-KI-BC11

4h — Total 110697 | INDEL 6.16% — Ins 2.93% — Del 1.98% — HDR 1.25%

Typical seqs	Reads	Type	pct.
GGAATCTGCCTAACAGG|AGGTGGGGGT	103880	WT	93.84%
GGAATCTGCCTAACAAGGAGGTGGGGGT	2325	+1 Ins	2.10%
GGAATCTGCCTAAGTTTAAACTACGCGTGGGT	1303	+5 HDR	1.18%
GGAATCTGCCTAACCAGGAGGTGGGGGT	263	+1 Ins	0.24%
GGAATCTGCCTAAC---AGGTGGGGGT	194	-3	0.18%
GGAATCTGCCTAACAG-AGGTGGGGGT	158	-1	0.14%
GGAATCTGCCTAACA--AGGTGGGGGT	125	-2	0.11%
GGAATCTGCCTAACAGGaGGGTGGGGGT	128	+1 a	0.12%
GGAATCTGCCTAA-AGGAGGTGGGGGT	113	-1	0.10%
GGAATCTGCCTA--AGGAGGTGGGGGT	110	-2	0.10%

8h — Total 166079 | INDEL 13.62% — Ins 3.48% — Del 3.82% — HDR 6.32%

Typical seqs	Reads	Type	pct.
GGAATCTGCCTAACAGG|AGGTGGGGGT	143461	WT	86.38%
GGAATCTGCCTAAGTTTAAACTACGCGTGGGT	9677	+5 HDR	5.83%
GGAATCTGCCTAACAAGGAGGTGGGGGT	3163	+1 Ins	1.90%
GGAATCTGCCTAAC---AGGTGGGGGT	714	-3	0.43%
GGAATCTGCCTAACCAGGAGGTGGGGGT	493	+1 Ins	0.30%
GGAATCTGCCTAACAG-AGGTGGGGGT	415	-1	0.25%
GGAATCTGCCTAACA--AGGTGGGGGT	411	-2	0.25%
GGAATCTGCCTA--AGGAGGTGGGGGT	260	-2	0.16%
GGAATCTGCCTAACAGGaGGGTGGGGGT	231	+1 a	0.14%
GGAATCTGCCTAACAGGA--TGGGGGT	203	-2	0.12%

12h — Total 285297 | INDEL 22.72% — Ins 5.81% — Del 7.39% — HDR 9.52%

Typical seqs	Reads	Type	pct.
GGAATCTGCCTAACAGG|AGGTGGGGGT	220491	WT	77.28%
GGAATCTGCCTAAGTTTAAACTACGCGTGGGT	25290	+5 HDR	8.86%
GGAATCTGCCTAACAAGGAGGTGGGGGT	9292	+1 Ins	3.26%
GGAATCTGCCTAAC---AGGTGGGGGT	2012	-3	0.71%
GGAATCTGCCTAACCAGGAGGTGGGGGT	1616	+1 Ins	0.57%
GGAATCTGCCTAACAG-AGGTGGGGGT	1250	-1	0.44%
GGAATCTGCCTAACA--AGGTGGGGGT	979	-2	0.34%
GGAATCTGCCTA--AGGAGGTGGGGGT	839	-2	0.29%
GGAATCTGCCTAA-AGGAGGTGGGGGT	791	-1	0.28%
GGAATCTGCCTAACAGGaGGGTGGGGGT	749	+1 a	0.26%

24h — Total 307853 | INDEL 31.82% — Ins 6.67% — Del 11.74% — HDR 13.40%

Typical seqs	Reads	Type	pct.
GGAATCTGCCTAACAGG|AGGTGGGGGT	209909	WT	68.18%
GGAATCTGCCTAAGTTTAAACTACGCGTGGGT	38297	+5 HDR	12.44%
GGAATCTGCCTAACAAGGAGGTGGGGGT	9778	+1 Ins	3.18%
GGAATCTGCCTAAC---AGGTGGGGGT	4419	-3	1.44%
GGAATCTGCCTAACCAGGAGGTGGGGGT	1974	+1 Ins	0.64%
GGAATCTGCCTAACAG-AGGTGGGGGT	1491	-1	0.48%
GGAATCTGCCTA--AGGAGGTGGGGGT	1271	-2	0.41%
GGAATCTGCCTAA-AGGAGGTGGGGGT	1109	-1	0.36%
GGAATCTGCCTAACAGGaGGGTGGGGGT	1065	+1 a	0.35%
GGAATCTGCCTAACA--AGGTGGGGGT	1000	-2	0.32%

48h — Total 126651 | INDEL 36.52% — Ins 7.18% — Del 14.10% — HDR 15.24%

Typical seqs	Reads	Type	pct.
GGAATCTGCCTAACAGG|AGGTGGGGGT	80396	WT	63.48%
GGAATCTGCCTAAGTTTAAACTACGCGTGGGT	18005	+5 HDR	14.22%
GGAATCTGCCTAACAaGGAGGTGGGGGT	4518	+1 Ins	3.57%
GGAATCTGCCTAAC---AGGTGGGGGT	2757	-3	2.18%
GGAATCTGCCTAACAG-AGGTGGGGGT	794	-1	0.63%
GGAATCTGCCTAACcAGGAGGTGGGGGT	766	+1 Ins	0.60%
GGAATCTGCCTAACA--AGGTGGGGGT	669	-2	0.53%
GGAATCTGCCTA--AGGAGGTGGGGGT	543	-2	0.43%
GGAATCTGCCTAACAGGaGGGTGGGGGT	537	+1 a	0.42%
GGAATCTGCCTAA-AGGAGGTGGGGGT	449	-1	0.35%

P58-P67-WT-U937-Syn25crAAVS1h-KO-BC6

4h — Total 93752 | INDEL 7.77% | Ins 4.34% | Del 3.43% | HDR 0.00%

Typical seqs	Reads	Type	pct.
TAAGGAATCTGCCTAAC|AGGAGGTGGG	85643	WT	91.35%
TAAGGAATCTGCCTAACaAGGAGGTGGG	1964	+1 a	2.09%
TAAGGAATCTGCCTAACcAGGAGGTGGG	1420	+1 c	1.51%
TAAGGAATCTGCCTAA-AGGAGGTGGG	509	-1	0.54%
TAAGGAATCTGCCTA--AGGAGGTGGG	402	-2	0.43%
TAAGGAATCTGCCTA-CAGGAGGTGGG	170	-1	0.18%
TAAGGAATCTGC-----AGGAGGTGGG	153	-5	0.16%
TAAGGAATCTGCC----AGGAGGTGGG	152	-4	0.16%
TAAGGAATCTGCCT---AGGAGGTGGG	133	-3	0.14%

8h — Total 205042 | INDEL 18.83% | Ins 8.39% | Del 10.37% | HDR 0.00%

Typical seqs	Reads	Type	pct.
TAAGGAATCTGCCTAAC|AGGAGGTGGG	158284	WT	77.20%
TAAGGAATCTGCCTAACaAGGAGGTGGG	6503	+1 a	3.17%
TAAGGAATCTGCCTAACcAGGAGGTGGG	6338	+1 c	3.09%
TAAGGAATCTGCCTAA-AGGAGGTGGG	2435	-1	1.19%
TAAGGAATCTGCCTA--AGGAGGTGGG	2266	-2	1.11%
TAAGGAATCTGCC----AGGAGGTGGG	1076	-4	0.52%
TAAGGAATCTGC-----AGGAGGTGGG	1108	-5	0.54%
TAAGGAATCTGCCTA-CAGGAGGTGGG	946	-1	0.46%
TAAGGAATCTGCCT---AGGAGGTGGG	821	-3	0.40%

12h — Total 207581 | INDEL 29.5% | Ins 10.99% | Del 18.51% | HDR 0.00%

Typical seqs	Reads	Type	pct.
TAAGGAATCTGCCTAAC|AGGAGGTGGG	134969	WT	65.02%
TAAGGAATCTGCCTAACcAGGAGGTGGG	9257	+1 c	4.46%
TAAGGAATCTGCCTAACaAGGAGGTGGG	7593	+1 a	3.66%
TAAGGAATCTGCCTAA-AGGAGGTGGG	4095	-1	1.97%
TAAGGAATCTGCCTA--AGGAGGTGGG	3945	-2	1.90%
TA---------------AGGAGGTGGG	2099	-15	1.01%
TAAGGAATCTGC-----AGGAGGTGGG	1837	-5	0.88%
TAAGGAATCTGCC----AGGAGGTGGG	1696	-4	0.82%
TAAGGAATCTGCCT---AGGAGGTGGG	1606	-3	0.77%

24h — Total 201426 | INDEL 41.65% | Ins 12.45% | Del 29.21% | HDR 0.00%

Typical seqs	Reads	Type	pct.
TAAGGAATCTGCCTAAC|AGGAGGTGGG	99983	WT	49.64%
TAAGGAATCTGCCTAACcAGGAGGTGGG	9665	+1 c	4.80%
TAAGGAATCTGCCTAACaAGGAGGTGGG	7592	+1 a	3.77%
TAAGGAATCTGCCTA--AGGAGGTGGG	5274	-2	2.62%
TAAGGAATCTGCCTAA-AGGAGGTGGG	4824	-1	2.39%
TA---------------AGGAGGTGGG	4519	-15	2.24%
TAAGGAATCTGCCT---AGGAGGTGGG	1919	-3	0.95%
TAAGGAATCTGCC----AGGAGGTGGG	1919	-4	0.95%
TAAGGAATCTGC-----AGGAGGTGGG	1835	-5	0.91%

48h — Total 70804 | INDEL 48.05% | Ins 13.83% | Del 34.22% | HDR 0.00%

Typical seqs	Reads	Type	pct.
TAAGGAATCTGCCTAAC|AGGAGGTGGG	30837	WT	43.55%
TAAGGAATCTGCCTAACcAGGAGGTGGG	4005	+1 c	5.66%
TAAGGAATCTGCCTAACaAGGAGGTGGG	2875	+1 a	4.06%
TAAGGAATCTGCCTA--AGGAGGTGGG	2234	-2	3.16%
TA---------------AGGAGGTGGG	2106	-15	2.97%
TAAGGAATCTGCCTAA-AGGAGGTGGG	1785	-1	2.52%
TAAGGAATCTGC-----AGGAGGTGGG	867	-5	1.22%
TAAGGAATCTGCCT---AGGAGGTGGG	840	-3	1.19%
TAAGGAATCTGCC----AGGAGGTGGG	691	-4	0.98%

P58-P67-WT-U937-Syn25crAAVS1h-AAV6-KI-BC12

4h

Total	INDEL	Ins	Del	HDR
87636	11.00%	4.48%	3.70%	2.82%

Typical seqs	Reads	Type	pct.
TAAGGAATCTGCCTAACAGG**AGG**TGGG	78000	WT	89.00%
TAAGGAATCTGCCTAAGTTTAAACTACGCGTG	2281	+5 HDR	2.60%
TAAGGAATCTGCCTAACaAGGAGGTGGG	1933	+1 a	2.21%
TAAGGAATCTGCCTAACcAGGAGGTGGG	1332	+1 c	1.52%
TAAGGAATCTGCCTA--AGGAGGTGGG	463	-2	0.53%
TAAGGAATCTGCCTAA-AGGAGGTGGG	433	-1	0.49%
TAAGGAATCTGCCTA-CAGGAGGTGGG	226	-1	0.26%
TAAGGAATCTGC-----AGGAGGTGGG	174	-5	0.20%
TAAGGAATCTGCC----AGGAGGTGGG	155	-4	0.18%
TAAGGAATCTGCCT---AGGAGGTGGG	123	-3	0.14%

8h

Total	INDEL	Ins	Del	HDR
61723	39.73%	7.29%	10.62%	21.83%

Typical seqs	Reads	Type	pct.
TAAGGAATCTGCCTAACAGG**AGG**TGGG	37198	WT	60.27%
TAAGGAATCTGCCTAAGTTTAAACTACGCGTG	12387	+5 HDR	20.07%
TAAGGAATCTGCCTAACcAGGAGGTGGG	2425	+1 c	3.93%
TAAGGAATCTGCCTAA-AGGAGGTGGG	939	-1	1.52%
TAAGGAATCTGCCTA--AGGAGGTGGG	826	-2	1.34%
TAAGGAATCTGC-----AGGAGGTGGG	532	-5	0.86%
TAAGGAATCTGCCTAACaAGGAGGTGGG	420	+1 a	0.68%
TAAGGAATCTGCC----AGGAGGTGGG	397	-4	0.64%
TAAGGAATCTGCCTA-CAGGAGGTGGG	355	-1	0.58%
TAAGGAATCTGCCT---AGGAGGTGGG	287	-3	0.46%

12h

Total	INDEL	Ins	Del	HDR
228614	45.64%	10.13%	13.47%	22.03%

Typical seqs	Reads	Type	pct.
TAAGGAATCTGCCTAACAGG**AGG**TGGG	124279	WT	54.36%
TAAGGAATCTGCCTAAGTTTAAACTACGCGTG	46518	+5 HDR	20.35%
TAAGGAATCTGCCTAACaAGGAGGTGGG	7859	+1 a	3.44%
TAAGGAATCTGCCTAACcAGGAGGTGGG	7510	+1 c	3.29%
TAAGGAATCTGCCTAA-AGGAGGTGGG	3860	-1	1.69%
TAAGGAATCTGCCTA--AGGAGGTGGG	2628	-2	1.15%
TAAGGAATCTGCC----AGGAGGTGGG	1589	-4	0.70%
TAAGGAATCTGCCTA-CAGGAGGTGGG	1492	-1	0.65%
TAAGGAATCTGC-----AGGAGGTGGG	1422	-5	0.62%
TAAGGAATCTGCCTAAC--GAGGTGGG	1103	-2	0.48%

24h

Total	INDEL	Ins	Del	HDR
256209	60.39%	11.35%	18.86%	30.18%

Typical seqs	Reads	Type	pct.
TAAGGAATCTGCCTAACAGG**AGG**TGGG	101484	WT	39.61%
TAAGGAATCTGCCTAAGTTTAAACTACGCGTG	70786	+5 HDR	27.63%
TAAGGAATCTGCCTAACcAGGAGGTGGG	9746	+1 c	3.80%
TAAGGAATCTGCCTAACaAGGAGGTGGG	8238	+1 a	3.22%
TAAGGAATCTGCCTAA-AGGAGGTGGG	5019	-1	1.96%
TAAGGAATCTGCCTA--AGGAGGTGGG	4447	-2	1.74%
TAAGGAATCTGCC----AGGAGGTGGG	2760	-4	1.08%
TAAGGAATCTGC-----AGGAGGTGGG	2307	-5	0.90%
TAAGGAATCTGCCTAAC--GAGGTGGG	1721	-2	0.67%
TAAGGAATCTGCCTA-CAGGAGGTGGG	1735	-1	0.68%

48h

Total	INDEL	Ins	Del	HDR
100052	64.64%	12.39%	20.57%	31.69%

Typical seqs	Reads	Type	pct.
TAAGGAATCTGCCTAACAGG**AGG**TGGG	35375	WT	35.36%
TAAGGAATCTGCCTAAGTTTAAACTACGCGTG	29354	+5 HDR	29.34%
TAAGGAATCTGCCTAACaAGGAGGTGGG	3880	+1 a	3.88%
TAAGGAATCTGCCTAACcAGGAGGTGGG	4065	+1 c	4.06%
TAAGGAATCTGCCTAA-AGGAGGTGGG	2246	-1	2.24%
TAAGGAATCTGCCTA--AGGAGGTGGG	2100	-2	2.10%
TAAGGAATCTGC-----AGGAGGTGGG	901	-5	0.90%
TAAGGAATCTGCCTAAC---AGGTGGG	818	-3	0.82%
TAAGGAATCTGCC----AGGAGGTGGG	791	-4	0.79%
TAAGGAATCTGCCT---AGGAGGTGGG	771	-3	0.77%

P58-P67-WT-U937-Syn26crCD326ac-KO-BC1

4h

Total | INDEL	Ins	Del	HDR
149652 | 0.49%	0.22%	0.27%	0.00%

Typical seqs	Reads	Type	pct.
CCCTCCCGCGCCCCTCT|TCT**CGG**CGCG	148912	WT	99.51%
CCCTCCCGCGCCCCTCTtTCTCGGCGCG	301	+1 t	0.20%
CCCTCCCGCGCCCCTC-TCTCGGCGCG	62	-1	0.04%
CCCTCCCGCGCCCC---TCTCGGCGCG	54	-3	0.04%
CCCTCCCGCGCCCCT--TCTCGGCGCG	33	-2	0.02%
CCCTCCCGCGCCCCT-TTCTCGGCGCG	29	-1	0.02%
CCCTCCCGCGCCCC-CTTCTCGGCGCG	27	-1	0.02%
CCCTCCCGCGCCC-----CTCGGCGCG	23	-5	0.02%
CCCTCCCGCGCCC----TCTCGGCGCG	19	-4	0.01%
CCCTCC---------------------	15	-21	0.01%

8h

Total | INDEL	Ins	Del	HDR
200629 | 1.02%	0.58%	0.44%	0.00%

Typical seqs	Reads	Type	pct.
CCCTCCCGCGCCCCTCT|TCT**CGG**CGCG	198579	WT	98.98%
CCCTCCCGCGCCCCTCTtTCTCGGCGCG	1018	+1 t	0.51%
CCCTCCCGCGCCCCTC-TCTCGGCGCG	152	-1	0.08%
CCCTCCCGCGCCCC---TCTCGGCGCG	139	-3	0.07%
CCCTCCCGCGCCCCT-TTCTCGGCGCG	103	-1	0.05%
CCCTCCCGCGCCCCT--TCTCGGCGCG	44	-2	0.02%
CCCTCCCGCGCCC---TTCTCGGCGCG	36	-3	0.02%
CCCTCCCGCGCCC-----CTCGGCGCG	34	-5	0.02%
CCCTCCCGCGCCCCTCTgTCTCGGCGCG	28	+1 g	0.01%
CCCTCCCGCGCCCC-CTTCTCGGCGCG	23	-1	0.01%

12h

Total | INDEL	Ins	Del	HDR
542080 | 1.39%	0.63%	0.76%	0.00%

Typical seqs	Reads	Type	pct.
CCCTCCCGCGCCCCTCT|TCT**CGG**CGCG	534565	WT	98.61%
CCCTCCCGCGCCCCTCTtTCTCGGCGCG	2804	+1 t	0.52%
CCCTCCCGCGCCCCTC-TCTCGGCGCG	613	-1	0.11%
CCCTCCCGCGCCCC---TCTCGGCGCG	579	-3	0.11%
CCCTCCCGCGCCCCT--TCTCGGCGCG	268	-2	0.05%
CCCTCCCGCGCCCCT-TTCTCGGCGCG	230	-1	0.04%
CCCTCC---------------------	304	-21	0.06%
CCCTCCCGCACGCGTAGTTTAAACCTCGGC GCG	169	+6	0.03%
CCCTCCCGCGCCC-----CTCGGCGCG	155	-5	0.03%
CCCTCC---------------------	304	-23	0.06%

24h

Total | INDEL	Ins	Del	HDR
560018 | 2.77%	1.09%	1.68%	0.00%

Typical seqs	Reads	Type	pct.
CCCTCCCGCGCCCCTCT|TCT**CGG**CGCG	544520	WT	97.23%
CCCTCCCGCGCCCCTCTtTCTCGGCGCG	4876	+1 t	0.87%
CCCTCCCGCGCCCC---TCTCGGCGCG	1319	-3	0.24%
CCCTCC---------------------	1382	-21	0.25%
CCCTCCCGCGCCCCTC-TCTCGGCGCG	874	-1	0.16%
CCCTCCCGCGCCCCT-TTCTCGGCGCG	648	-1	0.12%
CCCTCCCGCGCCCCT--TCTCGGCGCG	399	-2	0.07%
CCCTCC---------------------	1382	-23	0.25%
CCCTCCCGCGCCC-----CTCGGCGCG	356	-5	0.06%
CCCTCCCGCACGCGTAGTTTAAACCTCGGC GCG	252	+6	0.04%

48h

Total | INDEL	Ins	Del	HDR
231046 | 3.53%	1.17%	2.36%	0.00%

Typical seqs	Reads	Type	pct.
CCCTCCCGCGCCCCTCT|TCT**CGG**CGCG	222888	WT	96.47%
CCCTCCCGCGCCCCTCTtTCTCGGCGCG	2204	+1 t	0.95%
CCCTCCCGCGCCCC---TCTCGGCGCG	1017	-3	0.44%
CCCTCC---------------------	852	-21	0.37%
CCCTCCCGCGCCCCTC-TCTCGGCGCG	521	-1	0.23%
CCCTCCCGCGCCC-----CTCGGCGCG	311	-5	0.13%
CCCTCCCGCGCCCCT-TTCTCGGCGCG	321	-1	0.14%
CCCTCC---------------------	852	-23	0.37%
CCCTCCCGCGCCCCT--TCTCGGCGCG	143	-2	0.06%
CCCTCC-------------------CG	147	-19	0.06%

P58-P67-WT-U937-Syn26crCD326ac-AAV6-KI-BC7

4h — Total | INDEL: 191387 | 0.66% — Ins 0.31% — Del 0.29% — HDR 0.07%

Typical seqs	Reads	Type	pct.
CCCTCCCGCGCCCCTCTTCT<u>**CGG**</u>CGCG	190118	WT	99.34%
CCCTCCCGCGCCCCTCTtTCTCGGCGCG	472	+1 t	0.25%
CCCTCCCGCACGCGTAGTTTAAACCTCGGCGCG	120	+6 HDR	0.06%
CCCTCCCGCGCCCCTC-TCTCGGCGCG	71	-1	0.04%
CCCTCCCGCGCCCCT-TTCTCGGCGCG	58	-1	0.03%
CCCTCCCGCGCCCCT--TCTCGGCGCG	45	-2	0.02%
CCCTCCCGCGCCCC-CTTCTCGGCGCG	42	-1	0.02%
CCCTCCCGCGCCCCCCTTCTCG--GCG	33	-2	0.02%
CCCTCCCGCGCCCCTCTTCTC--AGCG	27	-2	0.01%
CCCTCCCGCGCCCC---TCTCGGCGCG	20	-3	0.01%

8h — Total | INDEL: 475835 | 1.58% — Ins 0.55% — Del 0.43% — HDR 0.60%

Typical seqs	Reads	Type	pct.
CCCTCCCGCGCCCCTCTTCT<u>**CGG**</u>CGCG	468338	WT	98.42%
CCCTCCCGCACGCGTAGTTTAAACCTCGGCGCG	2687	+6 HDR	0.56%
CCCTCCCGCGCCCCTCTtTCTCGGCGCG	1968	+1 t	0.41%
CCCTCCCGCGCCCC---TCTCGGCGCG	312	-3	0.07%
CCCTCCCGCGCCCCTC-TCTCGGCGCG	232	-1	0.05%
CCCTCCCGCGCCCCT-TTCTCGGCGCG	213	-1	0.04%
CCCTCCCGCGCGCGTAGTTTAAACCTCGGCGCG	162	+6	0.03%
CCCTCCCGCGCCCCT--TCTCGGCGCG	134	-2	0.03%
CCCTCCCGCGCC-----TCTCGGCGCG	74	-5	0.02%
CCCTCCCGCGCCCC-CTTCTCGGCGCG	66	-1	0.01%

12h — Total | INDEL: 400870 | 3.02% — Ins 0.85% — Del 0.65% — HDR 1.51%

Typical seqs	Reads	Type	pct.
CCCTCCCGCGCCCCTCTTCT<u>**CGG**</u>CGCG	388770	WT	96.98%
CCCTCCCGCACGCGTAGTTTAAACCTCGGCGCG	5665	+6 HDR	1.41%
CCCTCCCGCGCCCCTCTtTCTCGGCGCG	2149	+1 t	0.54%
CCCTCCCGCGCGCGTAGTTTAAACCTCGGCGCG	312	+6	0.08%
CCCTCCCGCGCCCC---TCTCGGCGCG	279	-3	0.07%
CCCTCCCGCGCCCCTC-TCTCGGCGCG	280	-1	0.07%
CCCTCCCGCGCCCCT-TTCTCGGCGCG	216	-1	0.05%
CCCTCCCGCGCCCCT--TCTCGGCGCG	214	-2	0.05%
CCCTCC---------------------	144	-21	0.04%
CCCTCCCGCGCCC---TTCTCGGCGCG	113	-3	0.03%

24h — Total | INDEL: 202855 | 5.26% — Ins 1.36% — Del 1.11% — HDR 2.78%

Typical seqs	Reads	Type	pct.
CCCTCCCGCGCCCCTCTTCT<u>**CGG**</u>CGCG	192193	WT	94.74%
CCCTCCCGCACGCGTAGTTTAAACCTCGGCGCG	5308	+6 HDR	2.62%
CCCTCCCGCGCCCCTCTtTCTCGGCGCG	1675	+1 t	0.83%
CCCTCCCGCGCCCC---TCTCGGCGCG	404	-3	0.20%
CCCTCCCGCGCGCGTAGTTTAAACCTCGGCGCG	318	+6	0.16%
CCCTCCCGCGCCCCTC-TCTCGGCGCG	290	-1	0.14%
CCCTCCCGCGCCCCT-TTCTCGGCGCG	204	-1	0.10%
CCCTCCCGCGCCCCT--TCTCGGCGCG	114	-2	0.06%
CCCTCC---------------------	141	-21	0.07%
CCCTCC-----------------CGCG	76	-17	0.04%

48h — Total | INDEL: 16281 | 6.47% — Ins 1.24% — Del 1.51% — HDR 3.72%

Typical seqs	Reads	Type	pct.
CCCTCCCGCGCCCCTCTTCT<u>**CGG**</u>CGCG	15227	WT	93.53%
CCCTCCCGCACGCGTAGTTTAAACCTCGGCGCG	581	+6 HDR	3.57%
CCCTCCCGCGCCCCTCTtTCTCGGCGCG	136	+1 t	0.84%
CCCTCCCGCGCCCC---TCTCGGCGCG	43	-3	0.26%
CCCTCCCGCGCCCCTC-TCTCGGCGCG	32	-1	0.20%
CCCTCC---------------------	34	-21	0.21%
CCCTCCCGCGCGCGTAGTTTAAACCTCGGCGCG	19	+6	0.12%
CCCTCCCGCGCCCCT-TTCTCGGCGCG	15	-1	0.09%
CCCTCCCGCGCCCCT--TCTCGGCGCG	9	-2	0.06%
CCCTCCCGCGCCC-----CTCGGCGCG	10	-5	0.06%

P58-P67-WT-U937-Syn27crCD326ad-KO-BC2

4h

Total | INDEL	Ins	Del	HDR
173904 | 0.10%	0.01%	0.09%	0.00%

Typical seqs	Reads	Type	pct.
CGCGCCGAGAAGAGGGG|CGC**GGG**AGGG	173729	WT	99.90%
CGCGCCGAGAAGA-GGGCGCGGGAGGG	56	-1	0.03%
CGCGCCGAGAAGAGGGGCGC-GGAGGG	29	-1	0.02%
CGCGCCGAGAAG-GGGGCGCGGGAGGG	23	-1	0.01%
CGCGCCGAGAAGGGGGGCGCGGGAGGG	1685	-2	0.97%
CGCGCCGAGAAGAGGGG-GCGGGAGGG	12	-1	0.01%
CGCGCCGAGAAGAGGGGgCGCGGGAGGG	7	+1 g	0.00%
CGCGCCGAGAAGAGGGG----------	7	-13	0.00%
CGCGCCGAGAAGAGGGGCGCAGGAGGG	225	-2	0.13%
CGCGCCGAGAAGAGGGACGCGGGAGGG	170	-2	0.10%

8h

Total | INDEL	Ins	Del	HDR
426785 | 0.14%	0.02%	0.13%	0.00%

Typical seqs	Reads	Type	pct.
CGCGCCGAGAAGAGGGG|CGC**GGG**AGGG	426050	WT	99.83%
CGCGCCGAGAAGA-GGGCGCGGGAGGG	187	-1	0.04%
CGCGCCGAGAAGAGGGGCGC-GGAGGG	83	-1	0.02%
CGCGCCGAGAAG-GGGGCGCGGGAGGG	51	-1	0.01%
CGCGCCGAGA------GCGCGGGAGGG	23	-6	0.01%
CGCGCCGAGAAGAGGGG-GCGGGAGGG	21	-1	0.00%
CGCGCCGAGAAGGGGGGCGCGGGAGGG	2266	-2	0.53%
CGCGCCGAG------------------	19	-20	0.00%
CGCGCCGAGAAGAGGGCCCGCGGGAGGG	17	+1 Ins	0.00%

12h

Total | INDEL	Ins	Del	HDR
442348 | 0.14%	0.01%	0.13%	0.00%

Typical seqs	Reads	Type	pct.
CGCGCCGAGAAGAGGGG|CGC**GGG**AGGG	441592	WT	99.83%
CGCGCCGAGAAGA-GGGCGCGGGAGGG	246	-1	0.06%
CGCGCCGAGAAGAGGGGCGC-GGAGGG	71	-1	0.02%
CGCGCCGAGAAG-GGGGCGCGGGAGGG	66	-1	0.01%
CGCGCCGAGAAGAGGGG-GCGGGAGGG	36	-1	0.01%
CGCGCCGAGAAGGGGGGCGCGGGAGGG	2387	-2	0.54%
CGCGCCGAGAAGAGGGGgCGCGGGAGGG	22	+1 g	0.00%
CGCGCCGAGA--AGGGGCGCGGGAGGG	16	-2	0.00%
CGCGCCGAGAAGAGGGGCGTGGGAGGG	560	-2	0.13%

24h

Total | INDEL	Ins	Del	HDR
132832 | 0.15%	0.04%	0.11%	0.00%

Typical seqs	Reads	Type	pct.
CGCGCCGAGAAGAGGGG|CGC**GGG**AGGG	132629	WT	99.85%
CGCGCCGAGAAGA-GGGCGCGGGAGGG	51	-1	0.04%
CGCGCCGAGAAGAGGGGCGC-GGAGGG	23	-1	0.02%
CGCGCCGAGAAG-GGGGCGCGGGAGGG	10	-1	0.01%
CGCGCCGAGAAGAGGGG-GCGGGAGGG	8	-1	0.01%
CGCGCCGAGAAGGGGGGCGCGGGAGGG	707	-2	0.53%
CGCGCCGAGAAGAGGGGgCGCGGGAGGG	7	+1 g	0.01%
CGCGCCGAGAA-----------GAGGG	7	-11	0.01%
CGCGCCGAGAAGAGGGGTGCGGGAGGG	141	-2	0.11%

48h

Total | INDEL	Ins	Del	HDR
182246 | 0.17%	0.03%	0.14%	0.00%

Typical seqs	Reads	Type	pct.
CGCGCCGAGAAGAGGGG|CGC**GGG**AGGG	181929	WT	99.83%
CGCGCCGAGAAGA-GGGCGCGGGAGGG	70	-1	0.04%
CGCGCCGAGAAGAGGGGCGC-GGAGGG	22	-1	0.01%
CGCGCCGAGAAGA-------GGGAGGG	18	-7	0.01%
CGCGCCGAGAA-----------GAGGG	15	-11	0.01%
CGCGCCGAGAAG-GGGGCGCGGGAGGG	13	-1	0.01%
CGCGCCGAGAAGA--GGCGCGGGAGGG	13	-2	0.01%
----------------GCGCGGGAGGG	11	-21	0.01%
CGCGCCGAGAAGA----CGCGGGAGGG	9	-4	0.00%

P58-P67-WT-U937-Syn27crCD326ad-AAV6-KI-BC8

4h

Total	INDEL	Ins	Del	HDR
201701	0.24%	**0.02%**	**0.22%**	**0.01%**

**Typical seqs**	**Reads**	**Type**	**pct.**
CGCGCCGAGAAGAGGGG|CGC**GGG**AGGG	201210	WT	99.76%
CGCGCCGAGAAGA-GGGCGCGGGAGGG	150	-1	0.07%
CGCGCCGAGAAGAGGGGCGC-GGAGGG	63	-1	0.03%
CGCGCCGAGAAG-GGGGCGCGGGAGGG	41	-1	0.02%
CGCGCCGAGAAGAGGGG-GCGGGAGGG	32	-1	0.02%
CGCGCCGAGAAGGGGGGCGCGGGAGGG	2294	-2	1.14%
CGCGCCGAGGTTTAAACTACGCGTGCGGGAGGG	17	+6 HDR	0.01%
CGCGCCGAGAAGAGGGGgCGCGGGAGGG	15	+1 g	0.01%
CGCGCCGAGAAGAGGGGCGCAGGAGGG	526	-2	0.26%
CGCGCCGAGAAGAGGGGCACGGGAGGG	506	-2	0.25%

8h

Total	INDEL	Ins	Del	HDR
246448	0.23%	**0.02%**	**0.20%**	**0.01%**

**Typical seqs**	**Reads**	**Type**	**pct.**
CGCGCCGAGAAGAGGGG|CGC**GGG**AGGG	245871	WT	99.77%
CGCGCCGAGAAGA-GGGCGCGGGAGGG	173	-1	0.07%
CGCGCCGAGAAGAGGGGCGC-GGAGGG	93	-1	0.04%
CGCGCCGAGAAG-GGGGCGCGGGAGGG	51	-1	0.02%
CGCGCCGAGGTTTAAACTACGCGTGCGGGAGGG	34	+6 HDR	0.01%
CGCGCCGAGAAGGGGGGCGCGGGAGGG	1795	-2	0.73%
CGCGCCGAGAAGAGGGGgCGCGGGAGGG	27	+1 g	0.01%
CGCGCCGAGAAGA--GGCGCGGGAGGG	18	-2	0.01%
CGCGCCGAGAAGAGGGG-GCGGGAGGG	16	-1	0.01%
CGCGCCGAGAAGAGGGGCGCAGGAGGG	608	-2	0.25%

12h

Total	INDEL	Ins	Del	HDR
404568	0.27%	**0.02%**	**0.23%**	**0.02%**

**Typical seqs**	**Reads**	**Type**	**pct.**
CGCGCCGAGAAGAGGGG|CGC**GGG**AGGG	403472	WT	99.73%
CGCGCCGAGAAGA-GGGCGCGGGAGGG	283	-1	0.07%
CGCGCCGAGAAGAGGGGCGC-GGAGGG	134	-1	0.03%
CGCGCCGAGGTTTAAACTACGCGTGCGGGAGGG	78	+6 HDR	0.02%
CGCGCCGAGAAG-GGGGCGCGGGAGGG	74	-1	0.02%
CGCGCCGAGAAGGGGGGCGCGGGAGGG	2822	-2	0.70%
CGCGCCGAGAAGAGGGGgCGCGGGAGGG	49	+1 g	0.01%
CGCGCCGAGAAGAGGGG-GCGGGAGGG	42	-1	0.01%
CGCGCCG--------------------	35	-22	0.01%
CGCGCCGAGAAGA--GGCGCGGGAGGG	30	-2	0.01%

24h

Total	INDEL	Ins	Del	HDR
548967	0.33%	**0.02%**	**0.26%**	**0.05%**

**Typical seqs**	**Reads**	**Type**	**pct.**
CGCGCCGAGAAGAGGGG|CGC**GGG**AGGG	547143	WT	99.67%
CGCGCCGAGAAGA-GGGCGCGGGAGGG	388	-1	0.07%
CGCGCCGAGGTTTAAACTACGCGTGCGGGAGGG	275	+6 HDR	0.05%
CGCGCCGAGAAGAGGGGCGC-GGAGGG	204	-1	0.04%
CGCGCCGAGAAGGGGGGCGCGGGAGGG	8376	-2	1.53%
CGCGCCGAGAAG-GGGGCGCGGGAGGG	97	-1	0.02%
CGCGCCGAGAAGAGGGG-GCGGGAGGG	70	-1	0.01%
CGCGCCGAGAAGAGGGGgCGCGGGAGGG	41	+1 g	0.01%
CGCGCCGAGAAGA--GGCGCGGGAGGG	38	-2	0.01%
CGCGCCGAGAAGAGGGGCACGGGAGGG	1460	-2	0.27%

48h

Total	INDEL	Ins	Del	HDR
169955	0.27%	**0.01%**	**0.21%**	**0.04%**

**Typical seqs**	**Reads**	**Type**	**pct.**
CGCGCCGAGAAGAGGGG|CGC**GGG**AGGG	169500	WT	99.73%
CGCGCCGAGAAGA-GGGCGCGGGAGGG	100	-1	0.06%
CGCGCCGAGGTTTAAACTACGCGTGCGGGAGGG	71	+6 HDR	0.04%
CGCGCCGAGAAGAGGGGCGC-GGAGGG	53	-1	0.03%
CGCGCCGAGAAG-GGGGCGCGGGAGGG	38	-1	0.02%
CGCGCCGAGAAGAGGGG-GCGGGAGGG	23	-1	0.01%
CGCGCCGAGAAGGGGGGCGCGGGAGGG	1192	-2	0.70%
CGCGCCGAGAAGAGGGGCACGGGAGGG	428	-2	0.25%
CGCGCCGAGAAGAGGGGgCGCGGGAGGG	11	+1 g	0.01%
CGCGCCGAGAAGAGGGGTGCGGGAGGG	326	-2	0.19%

P58-P67-WT-U937-Syn28crCD326ae-KO-BC3

Total | INDEL	4h	Ins	Del	HDR
190826 | 0.24%		0.06%	0.18%	0.00%

Typical seqs	Reads	Type	pct.
GCGCGCCGAGAAGAGGG|GCG**CGG**GAGG	190364	WT	99.76%
GCGCGCCGAGAAGA-GGGCGCGGGAGG	198	-1	0.10%
GCGCGCCGAGAAGAGGGgGCGCGGGAGG	111	+1 g	0.06%
GCGCGCCGAGAAG-GGGGCGCGGGAGG	34	-1	0.02%
GCGCGCCGAGAAGGGGGGCGCGGGAGG	1905	-2	1.00%
GCGCGCCGAGAAGAGGGG-GCGGGAGG	9	-1	0.00%
GCGCGCCGAG--GAGGGGCGCGGGAGG	8	-2	0.00%
GCGCGCCGAGAAGA-------GGGAGG	9	-7	0.00%
GCGCGCCGAGA------GCGCGGGAGG	7	-6	0.00%
GCGCGCCGAGAAGCGGGGCGCGGGAGG	217	-2	0.11%

Total | INDEL	8h	Ins	Del	HDR
404641 | 0.39%		0.11%	0.29%	0.00%

Typical seqs	Reads	Type	pct.
GCGCGCCGAGAAGAGGG|GCG**CGG**GAGG	403045	WT	99.61%
GCGCGCCGAGAAGA-GGGCGCGGGAGG	699	-1	0.17%
GCGCGCCGAGAAGAGGGgGCGCGGGAGG	301	+1 g	0.07%
GCGCGCCGAGAAGA--GGCGCGGGAGG	45	-2	0.01%
GCGCGCCGAGAAGA-------GGGAGG	37	-7	0.01%
GCGCGCCGAGAAG-GGGGCGCGGGAGG	36	-1	0.01%
GCGCGCCGAGA------GCGCGGGAGG	27	-6	0.01%
GCGCGCCGAGAAGGGGGGCGCGGGAGG	2101	-2	0.52%
GCGCGCCGAGAA-----------GAGG	22	-11	0.01%

Total | INDEL	12h	Ins	Del	HDR
400905 | 0.42%		0.12%	0.30%	0.00%

Typical seqs	Reads	Type	pct.
GCGCGCCGAGAAGAGGG|GCG**CGG**GAGG	399208	WT	99.58%
GCGCGCCGAGAAGA-GGGCGCGGGAGG	698	-1	0.17%
GCGCGCCGAGAAGAGGGgGCGCGGGAGG	374	+1 g	0.09%
GCGCGCCGAGAA-----------GAGG	63	-11	0.02%
GCGCGCCGAGAAG-GGGGCGCGGGAGG	54	-1	0.01%
GCGCGCCGAGAAGA--GGCGCGGGAGG	35	-2	0.01%
GCGCGCCGAGAAGA---GCGCGGGAGG	33	-3	0.01%
GCGCGCCGAGAAGA-------GGGAGG	32	-7	0.01%
GCGCGCCGAGAA----GGCGCGGGAGG	27	-4	0.01%

Total | INDEL	24h	Ins	Del	HDR
358166 | 0.69%		0.11%	0.57%	0.00%

Typical seqs	Reads	Type	pct.
GCGCGCCGAGAAGAGGG|GCG**CGG**GAGG	355699	WT	99.31%
GCGCGCCGAGAAGA-GGGCGCGGGAGG	997	-1	0.28%
GCGCGCCGAGAAGAGGGgGCGCGGGAGG	267	+1 g	0.07%
GCGCGCCGAGAAGA--GGCGCGGGAGG	163	-2	0.05%
GCGCGCCGAGAAGA-------GGGAGG	119	-7	0.03%
GCGCGCCGAGAA-----------GAGG	79	-11	0.02%
-----------------GCGCGGGAGG	95	-17	0.03%
GCGCGCCGAGAAGGGGGGCGCGGGAGG	5390	-2	1.50%
GCGCGCCGAGAAG-GGGGCGCGGGAGG	47	-1	0.01%

Total | INDEL	48h	Ins	Del	HDR
157753 | 0.81%		0.19%	0.62%	0.00%

Typical seqs	Reads	Type	pct.
GCGCGCCGAGAAGAGGG|GCG**CGG**GAGG	156481	WT	99.19%
GCGCGCCGAGAAGA-GGGCGCGGGAGG	402	-1	0.25%
GCGCGCCGAGAAGAGGGgGCGCGGGAGG	194	+1 g	0.12%
GCGCGCCGAGAAGA--GGCGCGGGAGG	86	-2	0.05%
GCGCGCCGAGAAGA-------GGGAGG	79	-7	0.05%
GCGCGCCGAGAA-----------GAGG	52	-11	0.03%
-----------------GCGCGGGAGG	47	-21	0.03%
GCGCGCCGAGAAG-GGGGCGCGGGAGG	26	-1	0.02%
GCGCGCCGAGAA----GGCGCGGGAGG	21	-4	0.01%

P58-P67-WT-U937-Syn28crCD326ae-AAV6-KI-BC9

4h — Total 199026 | INDEL 0.36% — Ins 0.04% — Del 0.31% — HDR 0.01%

Typical seqs	Reads	Type	pct.
GCGCGCCGAGAAGAGGG|GCG**CGG**GAGG	198311	WT	99.64%
GCGCGCCGAGAAGA-GGGCGCGGGAGG	332	-1	0.17%
GCGCGCCGAGAAGAGGGgGCGCGGGAGG	59	+1 g	0.03%
GCGCGCCGAGAAG-GGGGCGCGGGAGG	32	-1	0.02%
GCGCGCCGAGAAGGGGGGCGCGGGAGG	1317	-2	0.66%
GCGCGCCGAGAAGAGGGG-GCGGGAGG	25	-1	0.01%
GCGCGCCGAG--GAGGGGCGCGGGAGG	25	-2	0.01%
GCGCGCCGAGAGGAGGGGCGCGGGAGG	1324	-2	0.67%
GCGCGCCGAGAAGAGGGGGGCGCGGGAGG	15	+2	0.01%
GCGCGCCGAGAAGA-------GGGAGG	11	-7	0.01%

8h — Total 149977 | INDEL 0.53% — Ins 0.08% — Del 0.39% — HDR 0.06%

Typical seqs	Reads	Type	pct.
GCGCGCCGAGAAGAGGG|GCG**CGG**GAGG	149177	WT	99.47%
GCGCGCCGAGAAGA-GGGCGCGGGAGG	316	-1	0.21%
GCGCGCCGAGGTTTAAACTACGCGTGCGGGAGG	95	+6 HDR	0.06%
GCGCGCCGAGAAGAGGGgGCGCGGGAGG	66	+1 g	0.04%
GCGCGCCGAGAAGA--GGCGCGGGAGG	47	-2	0.03%
GCGCGCCGAGAGGAGGGGCGCGGGAGG	991	-2	0.66%
GCGCGCCGAGAAG-GGGGCGCGGGAGG	18	-1	0.01%
GCGCGCCGAGAAGGGGGGGCGCGGGAGG	15	+1 Ins	0.01%
-----------------GCGCGGGAGG	14	-17	0.01%
GCGCGCCGAGAAGAGGGGGGCGCGGGAGG	12	+2	0.01%

12h — Total 59837 | INDEL 0.68% — Ins 0.12% — Del 0.44% — HDR 0.12%

Typical seqs	Reads	Type	pct.
GCGCGCCGAGAAGAGGG|GCG**CGG**GAGG	59429	WT	99.32%
GCGCGCCGAGAAGA-GGGCGCGGGAGG	137	-1	0.23%
GCGCGCCGAGGTTTAAACTACGCGTGCGGGAGG	68	+6 HDR	0.11%
GCGCGCCGAGAAGAGGGgGCGCGGGAGG	51	+1 g	0.09%
GCGCGCCGAGAAGA--GGCGCGGGAGG	33	-2	0.06%
GCGCGCCGAGAAG-GGGGCGCGGGAGG	9	-1	0.02%
GCGCGCCGAGAAGGGGGGCGCGGGAGG	389	-2	0.65%
GCGCGCCGAGAGGAGGGGCGCGGGAGG	352	-2	0.59%
GCGCGCCGAGA--AGGGGCGCGGGAGG	5	-2	0.01%
GCGCGCCGAGAAGAGGGGCGCGAGAGG	91	-2	0.15%

24h — Total 473805 | INDEL 1.14% — Ins 0.20% — Del 0.61% — HDR 0.33%

Typical seqs	Reads	Type	pct.
GCGCGCCGAGAAGAGGG|GCG**CGG**GAGG	468416	WT	98.86%
GCGCGCCGAGGTTTAAACTACGCGTGCGGGAGG	1500	+6 HDR	0.32%
GCGCGCCGAGAAGA-GGGCGCGGGAGG	1364	-1	0.29%
GCGCGCCGAGAAGAGGGgGCGCGGGAGG	603	+1 g	0.13%
GCGCGCCGAGAAGA--GGCGCGGGAGG	225	-2	0.05%
GCGCGCCGAGAA-----------GAGG	112	-11	0.02%
GCGCGCCGAGAAG-GGGGCGCGGGAGG	79	-1	0.02%
-----------------GCGCGGGAGG	101	-21	0.02%
GCGCGCCGAGAAGA-------GGGAGG	70	-7	0.01%
GCGCGCCGAGAGGAGGGGCGCGGGAGG	3358	-2	0.71%

48h — Total 270677 | INDEL 1.20% — Ins 0.19% — Del 0.70% — HDR 0.31%

Typical seqs	Reads	Type	pct.
GCGCGCCGAGAAGAGGG|GCG**CGG**GAGG	267434	WT	98.80%
GCGCGCCGAGAAGA-GGGCGCGGGAGG	790	-1	0.29%
GCGCGCCGAGGTTTAAACTACGCGTGCGGGAGG	788	+6 HDR	0.29%
GCGCGCCGAGAAGAGGGgGCGCGGGAGG	355	+1 g	0.13%
GCGCGCCGAGAAGA--GGCGCGGGAGG	148	-2	0.05%
-----------------GCGCGGGAGG	115	-21	0.04%
GCGCGCCGAGAAGA-------GGGAGG	64	-7	0.02%
GCGCGCCGAGAAGGGGGGCGCGGGAGG	3493	-2	1.29%
GCGCGCCGAGAAG-GGGGCGCGGGAGG	58	-1	0.02%
-----------------GCGCGGGAGG	115	-19	0.04%

P58-P67-WT-U937-Syn29crCD326af-KO-BC4

4h

Total	INDEL	Ins	Del	HDR
161662	0.55%	0.30%	0.25%	0.00%

Typical seqs	Reads	Type	pct.
TGCGCGCGCGCCGAGAA|GAGGGGCGCG	160769	WT	99.45%
TGCGCGCGCGCCGAGAAaGAGGGGCGCG	458	+1 a	0.28%
TGCGCGCGCGCCGAGA-GAGGGGCGCG	92	-1	0.06%
TGCGCGCGCGCCGAGAAGA-GGGCGCG	52	-1	0.03%
TGCGCGCGCGCCGA---GAGGGGCGCG	41	-3	0.03%
TGCGCGCGCGCCGAGAAG-GGGGCGCG	29	-1	0.02%
TGCGCGCGCGCCGAG--GAGGGGCGCG	21	-2	0.01%
T-----------------------GCG	16	-23	0.01%
TGCGCGCGCGCCGAGA--AGGGGCGCG	15	-2	0.01%
T--GCGCGCGCCGAGAAGGGGGGCGCG	14	-2	0.01%

8h

Total	INDEL	Ins	Del	HDR
394361	1.05%	0.63%	0.42%	0.00%

Typical seqs	Reads	Type	pct.
TGCGCGCGCGCCGAGAA|GAGGGGCGCG	390221	WT	98.95%
TGCGCGCGCGCCGAGAAaGAGGGGCGCG	2238	+1 a	0.57%
TGCGCGCGCGCCGAGA-GAGGGGCGCG	370	-1	0.09%
TGCGCGCGCGCCGAGAAGA-GGGCGCG	220	-1	0.06%
TGCGCGCGCGCCGA---GAGGGGCGCG	125	-3	0.03%
TGCGCGCGCGCC-----GAGGGGCGCG	94	-5	0.02%
T---------------------GCGCG	82	-21	0.02%
TGCGCGCGCGCCGAGA--AGGGGCGCG	73	-2	0.02%
TGCGCGCGCGCCGA--AGAGGGGCGCG	71	-2	0.02%
TGCGCGCGCGCCGAGAA-AGGGGCGCG	67	-1	0.02%

12h

Total	INDEL	Ins	Del	HDR
116422	1.47%	1.02%	0.45%	0.00%

Typical seqs	Reads	Type	pct.
TGCGCGCGCGCCGAGAA|GAGGGGCGCG	114714	WT	98.53%
TGCGCGCGCGCCGAGAAaGAGGGGCGCG	1066	+1 a	0.92%
TGCGCGCGCGCCGAGA-GAGGGGCGCG	130	-1	0.11%
TGCGCGCGCGCCGA---GAGGGGCGCG	66	-3	0.06%
TGCGCGCGCGCCGAGAAGA-GGGCGCG	58	-1	0.05%
TGCGCGCGCGCC-----GAGGGGCGCG	56	-5	0.05%
TGCGCGCGCGCCGAGA--AGGGGCGCG	18	-2	0.02%
TGCGCGCGCGCCGAGAA-AGGGGCGCG	15	-1	0.01%
T---------------------GCGCG	16	-21	0.01%

24h

Total	INDEL	Ins	Del	HDR
359295	3.25%	1.76%	1.49%	0.00%

Typical seqs	Reads	Type	pct.
TGCGCGCGCGCCGAGAA|GAGGGGCGCG	347605	WT	96.75%
TGCGCGCGCGCCGAGAAaGAGGGGCGCG	5648	+1 a	1.57%
TGCGCGCGCGCCGAGA-GAGGGGCGCG	1032	-1	0.29%
TGCGCGCGCGCCGA---GAGGGGCGCG	655	-3	0.18%
T---------------------GCGCG	442	-21	0.12%
TGCGCGCGCGCC-----GAGGGGCGCG	395	-5	0.11%
TGCGCGCGCGCCGAGAAGA-GGGCGCG	220	-1	0.06%
TGCGCGCGCGCCGAGAA-AGGGGCGCG	158	-1	0.04%
TGCGCGCGCGCCGAGA--AGGGGCGCG	143	-2	0.04%

48h

Total	INDEL	Ins	Del	HDR
150945	4.45%	2.32%	2.13%	0.00%

Typical seqs	Reads	Type	pct.
TGCGCGCGCGCCGAGAA|GAGGGGCGCG	144229	WT	95.55%
TGCGCGCGCGCCGAGAAaGAGGGGCGCG	3160	+1 a	2.09%
TGCGCGCGCGCCGAGA-GAGGGGCGCG	448	-1	0.30%
TGCGCGCGCGCCGA---GAGGGGCGCG	321	-3	0.21%
TGCGCGCGCGCC-----GAGGGGCGCG	306	-5	0.20%
T---------------------GCGCG	293	-21	0.19%
T-----------------------GCG	116	-23	0.08%
TGCGCGCGCGCCGAGAAGA-GGGCGCG	114	-1	0.08%
TGC-------------------GCGCG	97	-19	0.06%
TGCGC-----------------GCGCG	68	-17	0.05%

P58-P67-WT-U937-Syn29crCD326af-AAV6-KI-BC10

4h

Total	INDEL	Ins	Del	HDR
186045	0.72%	**0.38%**	**0.32%**	**0.02%**

Typical seqs	Reads	Type	pct.
TGCGCGCGCGCCGAGAA|GAG**GGG**CGCG	184699	WT	99.28%
TGCGCGCGCGCCGAGAAaGAGGGGCGCG	635	+1 a	0.34%
TGCGCGCGCGCCGAGA-GAGGGGCGCG	121	-1	0.07%
TGCGCGCGCGCCGAGAAGA-GGGCGCG	118	-1	0.06%
TGCGCGCGCGCCGAGGTTTAAACTACGCGTGCG	40	+6 HDR	0.02%
TGCGCGCGCGCCGA---GAGGGGCGCG	31	-3	0.02%
TGCGCGCGCGCCGAGAAG-GGGGCGCG	29	-1	0.02%
T--GCGCGCGCCGAGAGGAGGGGCGCG	27	-2	0.01%
TGCGCGCGCGCCGAGA--AGGGGCGCG	25	-2	0.01%
T--GCGCGCGCCGAGGAGAGGGGCGCG	19	-2	0.01%

8h

Total	INDEL	Ins	Del	HDR
396925	1.46%	**0.78%**	**0.44%**	**0.24%**

Typical seqs	Reads	Type	pct.
TGCGCGCGCGCCGAGAA|GAG**GGG**CGCG	391116	WT	98.54%
TGCGCGCGCGCCGAGAAaGAGGGGCGCG	2628	+1 a	0.66%
TGCGCGCGCGCCGAGGTTTAAACTACGCGTGCG	894	+6 HDR	0.23%
TGCGCGCGCGCCGAGA-GAGGGGCGCG	384	-1	0.10%
TGCGCGCGCGCCGAGAAGA-GGGCGCG	296	-1	0.07%
TGCGCGCGCGCCGA---GAGGGGCGCG	154	-3	0.04%
TGCGCGCGCGCCGAG--GAGGGGCGCG	75	-2	0.02%
TGCGCGCGCGCCGAGAAG-GGGGCGCG	64	-1	0.02%
T--GCGCGCGCTGAGAAGAGGGGCGCG	50	-2	0.01%
T---------------------GCGCG	45	-21	0.01%

12h

Total	INDEL	Ins	Del	HDR
292979	2.39%	**1.02%**	**0.62%**	**0.75%**

Typical seqs	Reads	Type	pct.
TGCGCGCGCGCCGAGAA|GAG**GGG**CGCG	285976	WT	97.61%
TGCGCGCGCGCCGAGAAaGAGGGGCGCG	2417	+1 a	0.82%
TGCGCGCGCGCCGAGGTTTAAACTACGCGTGCG	2074	+6 HDR	0.71%
TGCGCGCGCGCCGAGA-GAGGGGCGCG	495	-1	0.17%
TGCGCGCGCGCCGAGAAGA-GGGCGCG	216	-1	0.07%
TGCGCGCGCGCCGA---GAGGGGCGCG	157	-3	0.05%
TGCGCGCGCGCCGAGGTTTAAACTACGCGCGCG	99	+6	0.03%
TGCGCGCGCGCCGAG--GAGGGGCGCG	71	-2	0.02%
TGCGCGCGCGCCGAGA--AGGGGCGCG	64	-2	0.02%
TGCGCGCGCGCCGA--AGAGGGGCGCG	63	-2	0.02%

24h

Total	INDEL	Ins	Del	HDR
231248	5.07%	**1.97%**	**1.33%**	**1.78%**

Typical seqs	Reads	Type	pct.
TGCGCGCGCGCCGAGAA|GAG**GGG**CGCG	219521	WT	94.93%
TGCGCGCGCGCCGAGGTTTAAACTACGCGTGCG	3853	+6 HDR	1.67%
TGCGCGCGCGCCGAGAAaGAGGGGCGCG	3548	+1 a	1.53%
TGCGCGCGCGCCGAGA-GAGGGGCGCG	680	-1	0.29%
TGCGCGCGCGCCGA---GAGGGGCGCG	306	-3	0.13%
TGCGCGCGCGCCGAGGTTTAAACTACGCGCGCG	249	+6	0.11%
TGCGCGCGCGCCGAGAAGA-GGGCGCG	201	-1	0.09%
T---------------------GCGCG	141	-21	0.06%
TGCGCGCGCGCC-----GAGGGGCGCG	116	-5	0.05%
T-----------------------GCG	101	-23	0.04%

48h

Total	INDEL	Ins	Del	HDR
269051	7.18%	**2.69%**	**2.15%**	**2.35%**

Typical seqs	Reads	Type	pct.
TGCGCGCGCGCCGAGAA|GAG**GGG**CGCG	249738	WT	92.82%
TGCGCGCGCGCCGAGGTTTAAACTACGCGTGCG	5853	+6 HDR	2.18%
TGCGCGCGCGCCGAGAAaGAGGGGCGCG	5630	+1 a	2.09%
TGCGCGCGCGCCGAGA-GAGGGGCGCG	1190	-1	0.44%
TGCGCGCGCGCCGA---GAGGGGCGCG	538	-3	0.20%
T---------------------GCGCG	463	-21	0.17%
TGCGCGCGCGCCGAGAAGA-GGGCGCG	268	-1	0.10%
TGCGCGCGCGCC-----GAGGGGCGCG	270	-5	0.10%
TGCGCGCGCGCCGAGGTTTAAACTACGCGCGCG	252	+6	0.09%
T-----------------------GCG	154	-23	0.06%

P58-P67-WT-U937-Syn30crCD326ag-KO-BC5

4h

Total	INDEL	Ins	Del	HDR
14697	0.06%	0.01%	0.05%	0.00%

Typical seqs	Reads	Type	pct.
CTGCGCGCGCGCCGAGA|AGA**GGG**GCGC	14688	WT	99.94%
CTGCGCGCGCGCCGAG-AGAGGGGCAC	3	-1	0.02%
CT--GCGCGCGCCGAGAAGAGAGGCGC	2	-2	0.01%
CTGCGCGCGCGCCGAG-AGAGGGGCGC	2	-1	0.01%
CTGCGCGCGCGCCGAGAaAGAGGGGCGC	2	+1 a	0.01%

8h

Total	INDEL	Ins	Del	HDR
324874	0.17%	0.06%	0.11%	0.00%

Typical seqs	Reads	Type	pct.
CTGCGCGCGCGCCGAGA|AGA**GGG**GCGC	324327	WT	99.83%
CTGCGCGCGCGCCGAGAaAGAGGGGCGC	76	+1 a	0.02%
CTGCGCGCGCGCCG---AGAGGGGCGC	48	-3	0.01%
CTGCGCGCGCGCCGAGAAG-GGGGCGC	45	-1	0.01%
CTGCGCGCGCGCCGAG-AGAGGGGCGC	41	-1	0.01%
CTGCGCGCGCGCCGAGAtAGAGGGGCGC	27	+1 t	0.01%
CT--GCGCGCGCTGAGAAGAGGGGCGC	23	-2	0.01%
CTGCGCGCGCGCCGAGAgAGAGGGGCGC	17	+1 g	0.01%
CTGCGCGCGCGCCGA---------CGC	15	-9	0.00%

12h

Total	INDEL	Ins	Del	HDR
294531	0.22%	0.09%	0.13%	0.00%

Typical seqs	Reads	Type	pct.
CTGCGCGCGCGCCGAGA|AGA**GGG**GCGC	293889	WT	99.78%
CTGCGCGCGCGCCGAGAaAGAGGGGCGC	103	+1 a	0.03%
CTGCGCGCGCGCCGAG-AGAGGGGCGC	63	-1	0.02%
CTGCGCGCGCGCCG---AGAGGGGCGC	59	-3	0.02%
CTGCGCGCGCGCCGAGAAG-GGGGCGC	29	-1	0.01%
CTGCGCGCGCGCCGAGAgAGAGGGGCGC	21	+1 g	0.01%
CTGCGCGCGCGCCGAGAcAGAGGGGCGC	20	+1 c	0.01%
CTGCGCGCGCGCC--------GGGCGC	15	-8	0.01%
CT---------------------GCGC	14	-21	0.00%

24h

Total	INDEL	Ins	Del	HDR
242918	0.39%	0.14%	0.25%	0.00%

Typical seqs	Reads	Type	pct.
CTGCGCGCGCGCCGAGA|AGA**GGG**GCGC	241978	WT	99.61%
CTGCGCGCGCGCCGAGAaAGAGGGGCGC	242	+1 a	0.10%
CTGCGCGCGCGCCG---AGAGGGGCGC	126	-3	0.05%
CT---------------------GCGC	52	-21	0.02%
CT-----------------------GC	50	-23	0.02%
CTGCGCGCGCGCCGAG-AGAGGGGCGC	43	-1	0.02%
CTGCGCGCGCGCCGAGAAG-GGGGCGC	37	-1	0.02%
CTGCGC-----------------GCGC	34	-17	0.01%
CTGC-------------------GCGC	28	-19	0.01%

48h

Total	INDEL	Ins	Del	HDR
174280	0.81%	0.23%	0.58%	0.00%

Typical seqs	Reads	Type	pct.
CTGCGCGCGCGCCGAGA|AGA**GGG**GCGC	172862	WT	99.19%
CTGCGCGCGCGCCGAGAaAGAGGGGCGC	224	+1 a	0.13%
CTGCGCGCGCGCCG---AGAGGGGCGC	181	-3	0.10%
CT---------------------GCGC	106	-21	0.06%
CTGCGCGCGCGCCGAG-AGAGGGGCGC	75	-1	0.04%
CTGCGCGCGCGCC-----GAGGGGCGC	64	-5	0.04%
CTGCGCGCGCGCCGAGAtAGAGGGGCGC	38	+1 t	0.02%
CTGCGC-----------------GCGC	29	-17	0.02%
CTGCGCGCGCGCCGAGAcAGAGGGGCGC	18	+1 c	0.01%

P58-P67-WT-U937-Syn30crCD326ag-AAV6-KI-BC11

4h

Total | INDEL	Ins	Del	HDR
214664 | 0.19%	0.04%	0.14%	0.01%

Typical seqs	Reads	Type	pct.
CTGCGCGCGCGCCGAGA|AGA**GGG**GCGC	214265	WT	99.81%
CTGCGCGCGCGCCGAGAaAGAGGGGCGC	52	+1 a	0.02%
CTGCGCGCGCGCCGAGAAG-GGGGCGC	35	-1	0.02%
CT--GCGCGCGCCGAGAGGAGGGGCGC	34	-2	0.02%
CT--GCGCGCGCCGAGGAGAGGGGCGC	28	-2	0.01%
CT--GCGCGCGCTGAGAAGAGGGGCGC	21	-2	0.01%
CT--GCGCGCGCCGAGAAGGGGGGCGC	18	-2	0.01%
CTGCGCGCGCGCCGAGAgAGAGGGGCGC	16	+1 g	0.01%
CTGCGCGCGCGCCGAGGTTTAAACTACGCGTGC	15	+6 HDR	0.01%
CT--GCGCGCGCCGGGAAGAGGGGCGC	16	-2	0.01%

8h

Total | INDEL	Ins	Del	HDR
465732 | 0.30%	0.04%	0.19%	0.07%

Typical seqs	Reads	Type	pct.
CTGCGCGCGCGCCGAGA|AGA**GGG**GCGC	464315	WT	99.70%
CTGCGCGCGCGCCGAGGTTTAAACTACGCGTGC	300	+6 HDR	0.06%
CTGCGCGCGCGCCGAGAaAGAGGGGCGC	148	+1 a	0.03%
CTGCGCGCGCGCCG---AGAGGGGCGC	113	-3	0.02%
CTGCGCGCGCGCCGAGAAG-GGGGCGC	90	-1	0.02%
CTGCGCGCGCGCCGAG-AGAGGGGCGC	59	-1	0.01%
CT--GCGCGCGCCGAGAGGAGGGGCGC	56	-2	0.01%
CT--GCGCGCGCCGAGAAGGGGGGCGC	49	-2	0.01%
CT--GCGCGCGCTGAGAAGAGGGGCGC	48	-2	0.01%
CTGCGCGCGCGCCGAGA--AGGGGCGC	44	-2	0.01%

12h

Total | INDEL	Ins	Del	HDR
311398 | 0.57%	0.11%	0.28%	0.17%

Typical seqs	Reads	Type	pct.
CTGCGCGCGCGCCGAGA|AGA**GGG**GCGC	309635	WT	99.43%
CTGCGCGCGCGCCGAGGTTTAAACTACGCGTGC	502	+6 HDR	0.16%
CTGCGCGCGCGCCGAGAaAGAGGGGCGC	208	+1 a	0.07%
CTGCGCGCGCGCCG---AGAGGGGCGC	96	-3	0.03%
CTGCGCGCGCGCCGAG-AGAGGGGCGC	89	-1	0.03%
CTGCGCGCGCGCCGAGAAG-GGGGCGC	58	-1	0.02%
CT--GCGCGCGCCGAGAAGGGGGGCGC	48	-2	0.02%
CTGCGCGCGCGCCGAGA----GGGCGC	39	-4	0.01%
CT--GCGCGCGCTGAGAAGAGGGGCGC	40	-2	0.01%
CTGCGCGCGCGCCGAGA--AGGGGCGC	38	-2	0.01%

24h

Total | INDEL	Ins	Del	HDR
445987 | 0.84%	0.19%	0.32%	0.33%

Typical seqs	Reads	Type	pct.
CTGCGCGCGCGCCGAGA|AGA**GGG**GCGC	442241	WT	99.16%
CTGCGCGCGCGCCGAGGTTTAAACTACGCGTGC	1377	+6 HDR	0.31%
CTGCGCGCGCGCCGAGAaAGAGGGGCGC	522	+1 a	0.12%
CTGCGCGCGCGCCG---AGAGGGGCGC	178	-3	0.04%
CTGCGCGCGCGCCGAG-AGAGGGGCGC	176	-1	0.04%
CT-------------------GCGC	117	-21	0.03%
CTGCGCGCGCGCCGAGAtAGAGGGGCGC	59	+1 t	0.01%
CTGCGCGCGCGCCGAGAcAGAGGGGCGC	56	+1 c	0.01%
CTGCGCGCGCGCCGAGAAG-GGGGCGC	55	-1	0.01%
CT--GCGCGCGCTGAGAAGAGGGGCGC	52	-2	0.01%

48h

Total | INDEL	Ins	Del	HDR
214200 | 1.16%	0.24%	0.53%	0.40%

Typical seqs	Reads	Type	pct.
CTGCGCGCGCGCCGAGA|AGA**GGG**GCGC	211712	WT	98.84%
CTGCGCGCGCGCCGAGGTTTAAACTACGCGTGC	808	+6 HDR	0.38%
CTGCGCGCGCGCCGAGAaAGAGGGGCGC	295	+1 a	0.14%
CTGCGCGCGCGCCGAG-AGAGGGGCGC	136	-1	0.06%
CTGCGCGCGCGCCG---AGAGGGGCGC	136	-3	0.06%
CT-------------------GCGC	68	-21	0.03%
CT--GCGCGCGCCGAGAAGGGGGGCGC	55	-2	0.03%
CTGCGCGCGCGCCGAGAAG-GGGGCGC	50	-1	0.02%
CTGCGCGCGCGCC-----GAGGGGCGC	34	-5	0.02%
CTGCGCGCGCGCCGAGGTTTAAACTACGCGCGC	31	+6	0.01%

P58-P67-WT-U937-Syn31crCD326ah-KO-BC6

Total | INDEL	4h	Ins	Del	HDR
130169 | 0.11%		0.03%	0.08%	0.00%

Typical seqs	Reads	Type	pct.
GCTGCGCGCGCGCCGAG|AAG**AGG**GGCG	130021	WT	99.89%
GCTGCGCGCGCGCCGAGaAAGAGGGGCG	19	+1 a	0.01%
GCTGCGCGCGCGCCGAGgAAGAGGGGCG	17	+1 g	0.01%
GCTGCGCGCGCGCCGAGAAG-GGGGCG	16	-1	0.01%
GCTGCGCGCGCGCCGAG--GAGGGGCG	11	-2	0.01%
GCT--GCGCGCGCTGAGAAGAGGGGCG	10	-2	0.01%
GCTGCGCGCGCGCCGAG-AGAGGGGCG	9	-1	0.01%
GCTGCGCG----------GGAGGGGCG	7	-10	0.01%
GCT--GCGCGCGCCGAGAGGAGGGGCG	7	-2	0.01%
GCT--GCGCGCGCCGAGGAGAGGGGCG	6	-2	0.00%

Total | INDEL	8h	Ins	Del	HDR
255137 | 0.23%		0.10%	0.14%	0.00%

Typical seqs	Reads	Type	pct.
GCTGCGCGCGCGCCGAG|AAG**AGG**GGCG	254539	WT	99.77%
GCTGCGCGCGCGCCGAGgAAGAGGGGCG	87	+1 g	0.03%
GCTGCGCGCGCGCCGAGaAAGAGGGGCG	77	+1 a	0.03%
GCTGCGCGCGCGCCGAGAAG-GGGGCG	36	-1	0.01%
GCTGCGCGCGCGCCGAGGA---GGGCG	27	-3	0.01%
GCTGCGCGCGCGCCGAG-AGAGGGGCG	26	-1	0.01%
GCTGCGCGCGCGCC-----GAGGGGCG	20	-5	0.01%
GCTGCGCGCGCGCCGA-AAGAGGGGCG	18	-1	0.01%
GCT--GCGCGCGCCGAGAAGGGGGGCG	20	-2	0.01%

Total | INDEL	12h	Ins	Del	HDR
312476 | 0.27%		0.10%	0.17%	0.00%

Typical seqs	Reads	Type	pct.
GCTGCGCGCGCGCCGAG|AAG**AGG**GGCG	311629	WT	99.73%
GCTGCGCGCGCGCCGAGgAAGAGGGGCG	108	+1 g	0.03%
GCTGCGCGCGCGCCGAGaAAGAGGGGCG	77	+1 a	0.02%
GCT---------------------GCG	57	-21	0.02%
GCTGCGCGCGCGCCGA-AAGAGGGGCG	54	-1	0.02%
GCTGCGCGCGCGCC-----GAGGGGCG	38	-5	0.01%
GCTGCGCGCGCGCCGAGAAG-GGGGCG	38	-1	0.01%
GCTGCGC-----------------GCG	41	-17	0.01%
GCTGCGCGCGCGCCG---AGAGGGGCG	33	-3	0.01%

Total | INDEL	24h	Ins	Del	HDR
340081 | 0.66%		0.16%	0.49%	0.00%

Typical seqs	Reads	Type	pct.
GCTGCGCGCGCGCCGAG|AAG**AGG**GGCG	337839	WT	99.34%
GCTGCGCGCGCGCCGAGaAAGAGGGGCG	175	+1 a	0.05%
GCTGCGCGCGCGCCGAGgAAGAGGGGCG	135	+1 g	0.04%
GCTGCGCGCGCGCC-----GAGGGGCG	129	-5	0.04%
GCT---------------------GCG	140	-21	0.04%
GCTGCGCGCGCGCCG---AGAGGGGCG	98	-3	0.03%
GCTGCGCGCGCGCCGAG--GAGGGGCG	69	-2	0.02%
GCTGCGCGCGCGCC--GAAGAGGGGCG	65	-2	0.02%
GCTGCGCGCGCGCCGAG-AGAGGGGCG	55	-1	0.02%

Total | INDEL	48h	Ins	Del	HDR
177927 | 0.97%		0.19%	0.78%	0.00%

Typical seqs	Reads	Type	pct.
GCTGCGCGCGCGCCGAG|AAG**AGG**GGCG	176201	WT	99.03%
GCTGCGCGCGCGCC-----GAGGGGCG	211	-5	0.12%
GCT---------------------GCG	112	-21	0.06%
GCTGCGCGCGCGCCGAGaAAGAGGGGCG	106	+1 a	0.06%
GCT-----------------------G	95	-23	0.05%
GCTGCGCGCGCGCCG---AGAGGGGCG	86	-3	0.05%
GCTGCGCGCGCGCCGAGgAAGAGGGGCG	82	+1 g	0.05%
GCTGCGCGCGCGCCGAG--GAGGGGCG	74	-2	0.04%
GCTGCGC-----------------GCG	52	-17	0.03%

P58-P67-WT-U937-Syn31crCD326ah-AAV6-KI-BC12

4h

Total	INDEL	Ins	Del	HDR
185041	0.23%	0.05%	0.16%	0.02%

Typical seqs	Reads	Type	pct.
GCTGCGCGCGCGCCGAG|AAG**AGG**GGCG	184624	WT	99.77%
GCTGCGCGCGCGCCGAGgAAGAGGGGCG	49	+1 g	0.03%
GCTGCGCGCGCGCCGAGaAAGAGGGGCG	31	+1 a	0.02%
GCTGCGCGCGCGCCGAGGTTTAAACTACGCGTG	30	+6 HDR	0.02%
GCTGCGCGCGCGCCGAGAAG-GGGGCG	33	-1	0.02%
GCTGCGCGCGCGCCGAG--GAGGGGCG	27	-2	0.01%
GCT--GCGCGCGCCGAGAGGAGGGGCG	19	-2	0.01%
GCT--GCGCGCGCCGAGAAGGGGGGCG	17	-2	0.01%
GCTGCGCGCGCGCCGA-AGGAGGGGCG	12	-1	0.01%
GCTGCGCG--------GAAGAGGGGCG	10	-8	0.01%

8h

Total	INDEL	Ins	Del	HDR
338878	0.34%	0.06%	0.19%	0.09%

Typical seqs	Reads	Type	pct.
GCTGCGCGCGCGCCGAG|AAG**AGG**GGCG	337719	WT	99.66%
GCTGCGCGCGCGCCGAGGTTTAAACTACGCGTG	292	+6 HDR	0.09%
GCTGCGCGCGCGCCGAGaAAGAGGGGCG	67	+1 a	0.02%
GCTGCGCGCGCGCCGAGAAG-GGGGCG	65	-1	0.02%
GCTGCGCGCGCGCCGAG--GAGGGGCG	60	-2	0.02%
GCT--GCGCGCGCCGAGAAGGGGGGCG	50	-2	0.01%
GCT--GCGCGCGCCGAGGAGAGGGGCG	39	-2	0.01%
GCT--GCGCGCGCCGAGAGGAGGGGCG	34	-2	0.01%
GCTGCGCGCGCGCCGAGgAAGAGGGGCG	35	+1 g	0.01%
GCTGCGCGCGCGCCGAG-AGAGGGGCG	30	-1	0.01%

12h

Total	INDEL	Ins	Del	HDR
72425	0.62%	0.10%	0.29%	0.23%

Typical seqs	Reads	Type	pct.
GCTGCGCGCGCGCCGAG|AAG**AGG**GGCG	71974	WT	99.38%
GCTGCGCGCGCGCCGAGGTTTAAACTACGCGTG	160	+6 HDR	0.22%
GCTGCGCGCGCGCCGAGgAAGAGGGGCG	34	+1 g	0.05%
GCTGCGCGCGCGCC-----GAGGGGCG	20	-5	0.03%
GCTGCGCGCGCGCCGAGAAG-GGGGCG	17	-1	0.02%
GCTGCGCGCGCGCCGAG--GAGGGGCG	16	-2	0.02%
GCT--GCGCGCGCTGAGAAGAGGGGCG	14	-2	0.02%
GCTGCGCGCGCGCCGAGaAAGAGGGGCG	15	+1 a	0.02%
GCT---------------------GCG	12	-21	0.02%
GCTGCGCGCGC----CGAAGAGGGGCG	13	-4	0.02%

24h

Total	INDEL	Ins	Del	HDR
122031	1.19%	0.22%	0.47%	0.50%

Typical seqs	Reads	Type	pct.
GCTGCGCGCGCGCCGAG|AAG**AGG**GGCG	120575	WT	98.81%
GCTGCGCGCGCGCCGAGGTTTAAACTACGCGTG	593	+6 HDR	0.49%
GCTGCGCGCGCGCCGAGgAAGAGGGGCG	125	+1 g	0.10%
GCTGCGCGCGCGCC-----GAGGGGCG	85	-5	0.07%
GCTGCGCGCGCGCCGAG--GAGGGGCG	44	-2	0.04%
GCTGCGCGCGCGCC--GAAGAGGGGCG	37	-2	0.03%
GCT---------------------GCG	36	-21	0.03%
GCTGCGCGCGCGCCG---AGAGGGGCG	34	-3	0.03%
GCTGCGCGCGCGCCGAGaAAGAGGGGCG	31	+1 a	0.03%
GCTGCGCGCGCGCCGAGGTTTAAACTACGCGCG	23	+6	0.02%

48h

Total	INDEL	Ins	Del	HDR
277663	1.73%	0.23%	0.76%	0.74%

Typical seqs	Reads	Type	pct.
GCTGCGCGCGCGCCGAG|AAG**AGG**GGCG	272860	WT	98.27%
GCTGCGCGCGCGCCGAGGTTTAAACTACGCGTG	1901	+6 HDR	0.68%
GCTGCGCGCGCGCCGAGgAAGAGGGGCG	196	+1 g	0.07%
GCTGCGCGCGCGCC-----GAGGGGCG	194	-5	0.07%
GCTGCGCGCGCGCCGAGaAAGAGGGGCG	142	+1 a	0.05%
GCT---------------------GCG	124	-21	0.04%
GCTGCGCGCGCGCCGAG--GAGGGGCG	125	-2	0.05%
GCTGCGCGCGCGCCG---AGAGGGGCG	95	-3	0.03%
GCTGCGCGCGCGCCGA-------GGCG	76	-7	0.03%
GCTGC-------------------GCG	48	-19	0.02%

P58-P67-WT-U937-Syn51crGATA4a-KO-BC1

4h — Total | INDEL: 115010 | 0.31% — Ins 0.05% — Del 0.26% — HDR 0.00%

Typical seqs	Reads	Type	pct.
CTCTTGGAACAGCCTGG|TCT**TGG**CCGA	114654	WT	99.69%
CTCTTGGAACAGCCTG-TCTTGGCCGA	189	-1	0.16%
CTCTTGGAACAGCCTGGtTCTTGGCCGA	24	+1 t	0.02%
CTCTTGGAACAGCCTGGTC-TGGCCGA	21	-1	0.02%
CTCTTGGAACAGCCTGGgTCTTGGCCGA	18	+1 g	0.02%
CTCTTGGAACAGCC---TCTTGGCCGA	15	-3	0.01%
CTCTTGGAACAG-CTGGTCTTGGCCGA	13	-1	0.01%
CTCTTGGAACAGCCTGGTCTT-GCCGA	9	-1	0.01%
CTCTTGGAACAGCC------TGGCCGA	8	-6	0.01%
CTCTTGGAACAGCCT--TCTTGGCCGA	7	-2	0.01%

8h — Total | INDEL: 5709 | 0.70% — Ins 0.16% — Del 0.54% — HDR 0.00%

Typical seqs	Reads	Type	pct.
CTCTTGGAACAGCCTGG|TCT**TGG**CCGA	5669	WT	99.30%
CTCTTGGAACAGCCTG-TCTTGGCCGA	8	-1	0.14%
CTCTTGGAACAGCCTGGtTCTTGGCCGA	6	+1 t	0.11%
CTCTTGGAACAGCC------TGGCCGA	7	-6	0.12%
CTCTTGGAACAGCCTGGaTCTTGGCCGA	3	+1 a	0.05%
CTCTTGGAACAGCC---TCTTGGCCGA	3	-3	0.05%
CTCTTGGAACAGCCTGG-CTTGGCCGA	3	-1	0.05%
CTCTTGGAACAGC---GTCTTGGCCGA	2	-3	0.04%
CTCTTGGAACAGCCTG---------GA	2	-9	0.04%
CTCTTGGAAC-------TCTTGGCCGA	2	-7	0.04%

12h — Total | INDEL: 271228 | 1.02% — Ins 0.23% — Del 0.80% — HDR 0.00%

Typical seqs	Reads	Type	pct.
CTCTTGGAACAGCCTGG|TCT**TGG**CCGA	268452	WT	98.98%
CTCTTGGAACAGCCTG-TCTTGGCCGA	700	-1	0.26%
CTCTTGGAACAGCC------TGGCCGA	335	-6	0.12%
CTCTTGGAACAGCCTGGtTCTTGGCCGA	241	+1 t	0.09%
CTCTTGGAACAGCC---TCTTGGCCGA	135	-3	0.05%
CTCTTGGAACAGCCTGGgTCTTGGCCGA	104	+1 g	0.04%
CTCTTGGAACA-----------GCCGA	97	-11	0.04%
CTCTTGGAACAGCCTGGcTCTTGGCCGA	70	+1 c	0.03%
CTCTTGGA------------------A	64	-18	0.02%
CTCTTGGAACAGCCT--TCTTGGCCGA	59	-2	0.02%

24h — Total | INDEL: 173650 | 2.29% — Ins 0.37% — Del 1.93% — HDR 0.00%

Typical seqs	Reads	Type	pct.
CTCTTGGAACAGCCTGG|TCT**TGG**CCGA	169666	WT	97.71%
CTCTTGGAACAGCC------TGGCCGA	883	-6	0.51%
CTCTTGGAACAGCCTG-TCTTGGCCGA	502	-1	0.29%
CTCTTGGAACA-----------GCCGA	280	-11	0.16%
CTCTTGGAACAGCCTGGtTCTTGGCCGA	232	+1 t	0.13%
CTCTTGGA------------------A	186	-18	0.11%
CTCTTGGAACAGCC---TCTTGGCCGA	170	-3	0.10%
CTCTTGGAACAGC-----CTTGGCCGA	156	-5	0.09%
CTCTTGGAACAGCCT--TCTTGGCCGA	91	-2	0.05%
CTCTTGGAACAGCCTGGaTCTTGGCCGA	51	+1 a	0.03%

48h — Total | INDEL: 111887 | 3.49% — Ins 0.60% — Del 2.89% — HDR 0.00%

Typical seqs	Reads	Type	pct.
CTCTTGGAACAGCCTGG|TCT**TGG**CCGA	107980	WT	96.51%
CTCTTGGAACAGCC------TGGCCGA	984	-6	0.88%
CTCTTGGAACAGCCTG-TCTTGGCCGA	411	-1	0.37%
CTCTTGGAACAGCCTGGtTCTTGGCCGA	250	+1 t	0.22%
CTCTTGGAACA-----------GCCGA	220	-11	0.20%
CTCTTGGAACAGC-----CTTGGCCGA	173	-5	0.15%
CTCTTGGA------------------A	168	-18	0.15%
CTCTTGGAACAGCC---TCTTGGCCGA	158	-3	0.14%
CTCTTGGAACAGCCT--TCTTGGCCGA	102	-2	0.09%
CTCTTGGAACAGCCTGGgTCTTGGCCGA	80	+1 g	0.07%

P58-P67-WT-U937-Syn51crGATA4a-AAV6-KI-BC9

4h — Total 127919 | INDEL 0.39% | HDR 0.03%

Typical seqs	Reads	Type	pct.
CTCTTGGAACAGCCTGG|TCT**TGG**CCGA	127418	WT	99.61%
CTCTTGGAACAGCCTG-TCTTGGCCGA	287	-1	0.22%
CTCTTGGAACAGCCTGGtTCTTGGCCGA	24	+1 t	0.02%
CTCTTGGAACAGCCTGGTC-TGGCCGA	17	-1	0.01%
CTCTTGGAACAGCCT--TCTTGGCCGA	15	-2	0.01%
CTCTTGGAACAGCCTGGgTCTTGGCCGA	13	+1 g	0.01%
CTCTTGGAACAGCCTGGGGTCTTGGCCGA	11	+2	0.01%
CTCTTGGAACAGCCTGG-------CGA	9	-7	0.01%
CTCTTGGAACAGCC---TCTTGGCCGA	7	-3	0.01%
CTCTTGGAACAGCC------TGGCCGA	8	-6	0.01%

8h — Total 43116 | INDEL 0.91% | HDR 0.18%

Typical seqs	Reads	Type	pct.
CTCTTGGAACAGCCTGG|TCT**TGG**CCGA	42722	WT	99.09%
CTCTTGGAACAGCCTG-TCTTGGCCGA	170	-1	0.39%
CTCTTGGAACAGCCTGGtTCTTGGCCGA	43	+1 t	0.10%
CTCTTGGAACAGCC---TCTTGGCCGA	22	-3	0.05%
CTCTTGGAACAGCCT--TCTTGGCCGA	19	-2	0.04%
CTCTTGGAACAGCCTGGgTCTTGGCCGA	10	+1 g	0.02%
CTCTTGGAACAGCCTGGaTCTTGGCCGA	10	+1 a	0.02%
CTCTTGGAACAGCCTGG-CTTGGCCGA	7	-1	0.02%
CTCTTGGAACAGCC------TGGCCGA	6	-6	0.01%
CTCTTGGAACAGCC--CTCTTGGCCGA	6	-2	0.01%

12h — Total 319844 | INDEL 0.93% | HDR 0.70%

Typical seqs	Reads	Type	pct.
CTCTTGGAACAGCCTGG|TCT**TGG**CCGA	316880	WT	99.07%
CTCTTGGAACAGCCTG-TCTTGGCCGA	965	-1	0.30%
CTCTTGGAACAGCCTGGtTCTTGGCCGA	206	+1 t	0.06%
CTCTTGGAACAGCC------TGGCCGA	214	-6	0.07%
CTCTTGGAACAGCC---TCTTGGCCGA	123	-3	0.04%
CTCTTGGAACAGCCT--TCTTGGCCGA	103	-2	0.03%
CTCTTGGAACAGCCTGGaTCTTGGCCGA	80	+1 a	0.03%
CTCTTGGAACAGCCTGGgTCTTGGCCGA	60	+1 g	0.02%
CTCTTGGAACAGC-----CTTGGCCGA	55	-5	0.02%
CTCTTGGAACA-----------GCCGA	60	-11	0.02%

24h — Total 322410 | INDEL 2.00% | HDR 2.07%

Typical seqs	Reads	Type	pct.
CTCTTGGAACAGCCTGG|TCT**TGG**CCGA	315969	WT	98.00%
CTCTTGGAACAGCCTG-TCTTGGCCGA	1016	-1	0.32%
CTCTTGGAACAGCC------TGGCCGA	946	-6	0.29%
CTCTTGGAACAGCCTGGtTCTTGGCCGA	456	+1 t	0.14%
CTCTTGGAACA-----------GCCGA	239	-11	0.07%
CTCTTGGAACAGC-----CTTGGCCGA	200	-5	0.06%
CTCTTGGAACAGCCT--TCTTGGCCGA	185	-2	0.06%
CTCTTGGAACAGCC---TCTTGGCCGA	170	-3	0.05%
CTCTTGGAACAGCCTGGgTCTTGGCCGA	119	+1 g	0.04%
CTCTTGGA------------------A	120	-18	0.04%

48h — Total 16891 | INDEL 4.15% | HDR 1.66%

Typical seqs	Reads	Type	pct.
CTCTTGGAACAGCCTGG|TCT**TGG**CCGA	16190	WT	95.85%
CTCTTGGAACAGCC------TGGCCGA	133	-6	0.79%
CTCTTGGAACAGCCTG-TCTTGGCCGA	72	-1	0.43%
CTCTTGGAACAGCCTGGtTCTTGGCCGA	57	+1 t	0.34%
CTCTTGGAACAGC-----CTTGGCCGA	35	-5	0.21%
CTCTTGGA------------------A	30	-18	0.18%
CTCTTGGAACAGCCT--TCTTGGCCGA	27	-2	0.16%
CTCTTGGAACAGCC---TCTTGGCCGA	25	-3	0.15%
CTCTTGGAACAGCCTGGgTCTTGGCCGA	22	+1 g	0.13%
CTCTTGGAACA-----------GCCGA	24	-11	0.14%

P58-P67-WT-U937-Syn52crGATA4b-KO-BC2

Total | INDEL		Ins	Del	HDR
106273 | 0.91%	4h	0.22%	0.69%	0.00%

Typical seqs	Reads	Type	pct.
TCAAATTCCTGCACGGA|CCT**GGG**ACTT	105309	WT	99.09%
TCAAATTCCTGCACGGA-CTGGGACTT	186	-1	0.18%
TCAAATTCCTGCACGGACCT-GGACTT	171	-1	0.16%
TCAAATTCCTGCACGGAaCCTGGGACTT	79	+1 a	0.07%
TCAAATTCCTGCACGGA--TGGGACTT	56	-2	0.05%
TCAAATT----------CCTGGGACTT	42	-10	0.04%
TCAAATTCCTGCACGGAcCCTGGGACTT	45	+1 c	0.04%
TCAAATTCCTGCACGGAtCCTGGGACTT	40	+1 t	0.04%
TCAAATTCCTGCAC-------GGACTT	38	-7	0.04%
TCAAATTCCTGCACGG-CCTGGGACTT	33	-1	0.03%

Total | INDEL		Ins	Del	HDR
178965 | 2.51%	8h	0.43%	2.09%	0.00%

Typical seqs	Reads	Type	pct.
TCAAATTCCTGCACGGA|CCT**GGG**ACTT	174093	WT	97.28%
TCAAATTCCTGCACGGA-CTGGGACTT	850	-1	0.47%
-----------CACG--CGTAGTTTAAACACTT	378	-9	0.21%
TCAAATTCCTGCACGGAaCCTGGGACTT	361	+1 a	0.20%
TCAAATT----------CCTGGGACTT	327	-10	0.18%
TCAAATTCCTGCACGGACCT-GGACTT	333	-1	0.19%
TCAAATTCCTGCACGGA--TGGGACTT	235	-2	0.13%
TCAAATTCCTGCAC-------GGACTT	230	-7	0.13%
TCAAATTCCTGCACGGAtCCTGGGACTT	190	+1 t	0.11%
TCAAATTCCTGCACGGA---GGGACTT	199	-3	0.11%

Total | INDEL		Ins	Del	HDR
277347 | 3.09%	12h	0.21%	2.88%	0.00%

Typical seqs	Reads	Type	pct.
TCAAATTCCTGCACGGA|CCT**GGG**ACTT	267385	WT	96.41%
-----------CACG--CGTAGTTTAAACACTT	1393	-9	0.50%
TCAAATTCCTGCACGGA-CTGGGACTT	1286	-1	0.46%
TCAAATT----------CCTGGGACTT	788	-10	0.28%
TCAAATTCCTGCAC-------GGACTT	736	-7	0.27%
TCAAATTCCTGCACGGAaCCTGGGACTT	551	+1 a	0.20%
TCAAATTCCTGCACGGACCT-GGACTT	480	-1	0.17%
TCAAATTCCTGCACGGA--TGGGACTT	357	-2	0.13%
TCAAATTCCTGCACGGAtCCTGGGACTT	354	+1 t	0.13%
TCAAATTCCTGCACGGAcCCTGGGACTT	305	+1 c	0.11%

Total | INDEL		Ins	Del	HDR
168343 | 7.25%	24h	1.12%	6.60%	0.00%

Typical seqs	Reads	Type	pct.
TCAAATTCCTGCACGGA|CCT**GGG**ACTT	153663	WT	91.28%
TCAAATT----------CCTGGGACTT	1991	-10	1.18%
TCAAATTCCTGCAC-------GGACTT	1465	-7	0.87%
TCAAATTCCTGCACGGA-CTGGGACTT	983	-1	0.58%
TCAAATTCCTGCACGGAaCCTGGGACTT	471	+1 a	0.28%
TCAAATTCCTGCAC------GGGACTT	456	-6	0.27%
TCAAATTCCTGCAC-------------	520	-13	0.31%
TCAAATTCCTGCACGGA--TGGGACTT	312	-2	0.19%
TCAAATTCCTGCACGGA---GGGACTT	274	-3	0.16%

Total | INDEL		Ins	Del	HDR
95644 | 9.28%	48h	1.40%	9.28%	0.00%

Typical seqs	Reads	Type	pct.
TCAAATTCCTGCACGGA|CCT**GGG**ACTT	85434	WT	89.32%
TCAAATT----------CCTGGGACTT	1464	-10	1.53%
TCAAATTCCTGCAC-------GGACTT	1259	-7	1.32%
TCAAATTCCTGCACGGA-CTGGGACTT	741	-1	0.77%
TCAAATTCCTGCAC------GGGACTT	315	-6	0.33%
TCAAATTCCTGCACGGAaCCTGGGACTT	315	+1 a	0.33%
TCAAATTCCTGCACGGA--TGGGACTT	225	-2	0.24%
TCAAATTCCTGCAC-------------	309	-13	0.32%
TCAAATTCCTGCACGGA---GGGACTT	198	-3	0.21%

P58-P67-WT-U937-Syn52crGATA4b-AAV6-KI-BC10

4h — Total 118593 | INDEL 1.03% — Ins 0.22% — Del 0.73% — HDR 0.08%

Typical seqs	Reads	Type	pct.
TCAAATTCCTGCACGGA|CCT**GGG**ACTT	117369	WT	98.97%
TCAAATTCCTGCACGGACCT-GGACTT	275	-1	0.23%
TCAAATTCCTGCACGGA-CTGGGACTT	176	-1	0.15%
-----------CACG--CGTAGTTTAAACACTT	95	-9 HDR	0.08%
TCAAATTCCTGCACGGAaCCTGGGACTT	85	+1 a	0.07%
TCAAATTCCTGCACGGAtCCTGGGACTT	55	+1 t	0.05%
TCAAATTCCTGCACGGA---GGGACTT	52	-3	0.04%
TCAAATTCCTGCACGGA--TGGGACTT	44	-2	0.04%
TCAAATTCCTGCACGGAcCCTGGGACTT	36	+1 c	0.03%
TCAAATTCCTGCACGGAgCCTGGGACTT	25	+1 g	0.02%

8h — Total 52635 | INDEL 4.86% — Ins 0.90% — Del 2.16% — HDR 1.79%

Typical seqs	Reads	Type	pct.
TCAAATTCCTGCACGGA|CCT**GGG**ACTT	50079	WT	95.14%
-----------CACG--CGTAGTTTAAACACTT	921	-9 HDR	1.75%
TCAAATTCCTGCACGGA-CTGGGACTT	351	-1	0.67%
TCAAATTCCTGCACGGAaCCTGGGACTT	159	+1 a	0.30%
TCAAATTCCTGCACGGACCT-GGACTT	105	-1	0.20%
TCAAATTCCTGCACGGAcCCTGGGACTT	98	+1 c	0.19%
TCAAATTCCTGCACGGA--TGGGACTT	74	-2	0.14%
TCAAATTCCTGCACGGAgCCTGGGACTT	70	+1 g	0.13%
TCAAATTCCTGCACGGAtCCTGGGACTT	62	+1 t	0.12%
TCAAATTCCTGCACGGA---GGGACTT	50	-3	0.09%

12h — Total 270800 | INDEL 5.54% — Ins 0.70% — Del 2.21% — HDR 2.63%

Typical seqs	Reads	Type	pct.
TCAAATTCCTGCACGGA|CCT**GGG**ACTT	255809	WT	94.46%
-----------CACG--CGTAGTTTAAACACTT	6845	-9 HDR	2.53%
TCAAATTCCTGCACGGA-CTGGGACTT	1373	-1	0.51%
TCAAATTCCTGCACGGACCT-GGACTT	571	-1	0.21%
TCAAATTCCTGCACGGAaCCTGGGACTT	500	+1 a	0.18%
TCAAATTCCTGCACGGA--TGGGACTT	411	-2	0.15%
TCAAATT----------CCTGGGACTT	391	-10	0.14%
TCAAATTCCTGCACGGA---GGGACTT	335	-3	0.12%
TCAAATTCCTGCAC-------GGACTT	280	-7	0.10%
TCAAATTCCTGCACGGAcCCTGGGACTT	269	+1 c	0.10%

24h — Total 279114 | INDEL 11.21% — Ins 1.03% — Del 4.02% — HDR 6.15%

Typical seqs	Reads	Type	pct.
TCAAATTCCTGCACGGA|CCT**GGG**ACTT	247828	WT	88.79%
-----------CACG--CGTAGTTTAAACACTT	16603	-9 HDR	5.95%
TCAAATTCCTGCACGGA-CTGGGACTT	1757	-1	0.63%
TCAAATT----------CCTGGGACTT	1424	-10	0.51%
TCAAATTCCTGCAC-------GGACTT	898	-7	0.32%
TCAAATTCCTGCACGGA--TGGGACTT	697	-2	0.25%
TCAAATTCCTGCACGGAaCCTGGGACTT	588	+1 a	0.21%
TCAAATTCCTGCACGGACCT-GGACTT	500	-1	0.18%
TCAAATTCCTGCACGGAtCCTGGGACTT	462	+1 t	0.17%
TCAAATTCCTGCACGGA---GGGACTT	445	-3	0.16%

48h — Total 126899 | INDEL 12.87% — Ins 1.31% — Del 5.33% — HDR 6.23%

Typical seqs	Reads	Type	pct.
TCAAATTCCTGCACGGA|CCT**GGG**ACTT	110572	WT	87.13%
-----------CACG--CGTAGTTTAAACACTT	7600	-9 HDR	5.99%
TCAAATTCCTGCACGGA-CTGGGACTT	1203	-1	0.95%
TCAAATT----------CCTGGGACTT	907	-10	0.71%
TCAAATTCCTGCAC-------GGACTT	630	-7	0.50%
TCAAATTCCTGCACGGAaCCTGGGACTT	371	+1 a	0.29%
TCAAATTCCTGCACGGA--TGGGACTT	334	-2	0.26%
TCAAATTCCTGCACGGA---GGGACTT	317	-3	0.25%
TCAAATTCCTGCACGGAcCCTGGGACTT	276	+1 c	0.22%
TCAAATTCCTGCACGGAtCCTGGGACTT	237	+1 t	0.19%

P58-P67-WT-U937-Syn53crGATA4c-KO-BC3

Total | INDEL	4h	Ins	Del	HDR
128854 | 0.17%		0.04%	0.12%	0.00%

Typical seqs	Reads	Type	pct.
CTCAAATTCCTGCACGG|ACCTGGGACT	128638	WT	99.83%
CTCAAATTCCTGCACGGA-CTGGGACT	49	-1	0.04%
CTCAAATTCCTGCAC-GACCTGGGACT	17	-1	0.01%
CTCAAATTCCTGCACGG--CTGGGACT	12	-2	0.01%
CTCAAATTCCTGCACGGaACCTGGGACT	10	+1 a	0.01%
CTCAAATTCCTGCAACGGACCTGGGACT	9	+1 Ins	0.01%
CTCAAATTCCTGCACGGaCCCTGGGACT	9	+1 a	0.01%
CTCAAATTCCTGCACGGA--TGGGACT	8	-2	0.01%
CTCAAATTCCTGCAC------GGGACT	7	-6	0.01%

Total | INDEL	8h	Ins	Del	HDR
78747 | 0.29%		0.01%	0.28%	0.00%

Typical seqs	Reads	Type	pct.
CTCAAATTCCTGCACGG|ACCTGGGACT	78519	WT	99.71%
CTCAAATTCCTGCACGG--CTGGGACT	33	-2	0.04%
CTCAAATTCCTGCACGG-CCTGGGACT	10	-1	0.01%
CTCAAATTCCTGCAC-GACCTGGGACT	12	-1	0.02%
CTCAAATTCCTGCACGGA-CTGGGACT	9	-1	0.01%
CTCAAATTCCTGC----ACCTGGGACT	9	-4	0.01%
CTCAAATTCCTGCAC------GGGACT	7	-6	0.01%
CTCAAATTCCTGCAC-------GGACT	6	-7	0.01%
CTCAAATTCCTGCACGGgACCTGGGACT	6	+1 g	0.01%

Total | INDEL	12h	Ins	Del	HDR
184823 | 0.51%		0.06%	0.45%	0.00%

Typical seqs	Reads	Type	pct.
CTCAAATTCCTGCACGG|ACCTGGGACT	183872	WT	99.49%
CTCAAATTCCTGCACGG--CTGGGACT	96	-2	0.05%
CTCAAATTCCTGCACGGA-CTGGGACT	49	-1	0.03%
CTCAAATTCCTGC----ACCTGGGACT	51	-4	0.03%
CTCAAATT----------CCTGGGACT	36	-10	0.02%
CTCAAATTCCTGCAC-GACCTGGGACT	35	-1	0.02%
CTCAAATTCCTGCACGGtACCTGGGACT	21	+1 t	0.01%
CTCAAATTCCTGCAC------GGGACT	21	-6	0.01%
CTCAAATTCCTGCACGGaACCTGGGACT	21	+1 a	0.01%

Total | INDEL	24h	Ins	Del	HDR
209526 | 1.32%		0.20%	2.52%	0.00%

Typical seqs	Reads	Type	pct.
CTCAAATTCCTGCACGG|ACCTGGGACT	203827	WT	97.28%
CTCAAATT----------CCTGGGACT	306	-10	0.15%
CTCAAATTCCTGCACGGA-CTGGGACT	163	-1	0.08%
CTCAAATTCCTGCAC------GGGACT	130	-6	0.06%
CTCAAATTCCTGCAC-------GGACT	131	-7	0.06%
CTCAAATTCCTGC----ACCTGGGACT	103	-4	0.05%
CTCAAATTCCTGCAACGGACCTGGGACT	76	+1 Ins	0.04%
CTCAAATTCCTGCACGG--CTGGGACT	66	-2	0.03%
CTCAAATTCCTGCACGGaACCTGGGACT	61	+1 a	0.03%

Total | INDEL	48h	Ins	Del	HDR
118323 | 1.65%		0.25%	3.00%	0.00%

Typical seqs	Reads	Type	pct.
CTCAAATTCCTGCACGG|ACCTGGGACT	114480	WT	96.75%
CTCAAATT----------CCTGGGACT	336	-10	0.28%
CTCAAATTCCTGCAC-------GGACT	138	-7	0.12%
CTCAAATTCCTGC----ACCTGGGACT	119	-4	0.10%
CTCAAATTCCTGCACGGA-CTGGGACT	114	-1	0.10%
CTCAAATTCCTGCAC------GGGACT	108	-6	0.09%
CT----------CACGGACCTGGGACT	59	-10	0.05%
CTCAAATTCCTGCACGG--CTGGGACT	54	-2	0.05%
CTCAAATTCCTGCAACGGACCTGGGACT	51	+1 Ins	0.04%

P58-P67-WT-U937-Syn53crGATA4c-AAV6-KI-BC11

Total | INDEL	4h	Ins	Del	HDR
127659 | 0.19%		0.03%	0.14%	0.02%

Typical seqs	Reads	Type	pct.
CTCAAATTCCTGCACGG|ACCTGGGACT	127411	WT	99.81%
CTCAAATTCCTGCACGGA-CTGGGACT	50	-1	0.04%
------------CACG-- CGTAGTTTAAACACT	22	-9 HDR	0.02%
CTCAAATTCCTGCAC-GACCTGGGACT	16	-1	0.01%
CTCAAATTCCTGCACGGaACCTGGGACT	12	+1 a	0.01%
CTCAAATTCCTGCACGG-CCTGGGACT	10	-1	0.01%
CTCAAATTCCTGCACGGA--TGGGACT	9	-2	0.01%
CTCAAATT----------CCTGGGACT	8	-10	0.01%
CTCAAATTCCTGCACGGA---GGGACT	6	-3	0.00%
CTCAAATTCCTGCACGGA-GGGGGACT	4	-1	0.00%

Total | INDEL	8h	Ins	Del	HDR
170236 | 0.42%		0.04%	0.23%	0.15%

Typical seqs	Reads	Type	pct.
CTCAAATTCCTGCACGG|ACCTGGGACT	169527	WT	99.58%
------------CACG-- CGTAGTTTAAACACT	259	-9 HDR	0.15%
CTCAAATTCCTGCACGG--CTGGGACT	76	-2	0.04%
CTCAAATTCCTGCACGGA-CTGGGACT	68	-1	0.04%
CTCAAATTCCTGCAC-GACCTGGGACT	28	-1	0.02%
CTCAAATTCCTGCACGG-CCTGGGACT	26	-1	0.02%
CTCAAATTCCTGCACGGA--TGGGACT	17	-2	0.01%
CTCAAATT----------CCTGGGACT	17	-10	0.01%
CTCAAATTCCTGCACGGaACCTGGGACT	14	+1 a	0.01%
CTCAAATTCCTGCAC-------GGACT	10	-7	0.01%

Total | INDEL	12h	Ins	Del	HDR
290732 | 1.28%		0.16%	0.49%	0.63%

Typical seqs	Reads	Type	pct.
CTCAAATTCCTGCACGG|ACCTGGGACT	287017	WT	98.72%
------------CACG-- CGTAGTTTAAACACT	1781	-9 HDR	0.61%
CTCAAATTCCTGCACGGA-CTGGGACT	216	-1	0.07%
CTCAAATT----------CCTGGGACT	161	-10	0.06%
CTCAAATTCCTGCACGGaACCTGGGACT	111	+1 a	0.04%
CTCAAATTCCTGCAC-------GGACT	89	-7	0.03%
CTCAAATTCCTGCACGGA--TGGGACT	48	-2	0.02%
CTCAAATTCCTGCAACGGACCTGGGACT	41	+1 Ins	0.01%
CTCAAATTCCTGCAC-GACCTGGGACT	40	-1	0.01%
CTCAAATTCCTGCACGGA---GGGACT	34	-3	0.01%

Total | INDEL	24h	Ins	Del	HDR
327990 | 3.08%		0.22%	1.13%	1.73%

Typical seqs	Reads	Type	pct.
CTCAAATTCCTGCACGG|ACCTGGGACT	317874	WT	96.92%
------------CACG-- CGTAGTTTAAACACT	5534	-9 HDR	1.69%
CTCAAATT----------CCTGGGACT	515	-10	0.16%
CTCAAATTCCTGCACGGA-CTGGGACT	266	-1	0.08%
CTCAAATTCCTGCAC-------GGACT	240	-7	0.07%
CTCAAATTCCTGCAC------GGGACT	140	-6	0.04%
CT----------CACGGACCTGGGACT	125	-10	0.04%
CTCAAATTCCTGCAACGGACCTGGGACT	118	+1 Ins	0.04%
CTCAAATTCCTGC----ACCTGGGACT	110	-4	0.03%
------------------CCTGGGACT	168	-19	0.05%

Total | INDEL	48h	Ins	Del	HDR
152595 | 3.66%		0.25%	1.68%	1.73%

Typical seqs	Reads	Type	pct.
CTCAAATTCCTGCACGG|ACCTGGGACT	147003	WT	96.34%
------------CACG-- CGTAGTTTAAACACT	2575	-9 HDR	1.69%
CTCAAATT----------CCTGGGACT	393	-10	0.26%
CTCAAATTCCTGCACGGA-CTGGGACT	197	-1	0.13%
CTCAAATTCCTGCAC-------GGACT	156	-7	0.10%
CTCAAATTCCTGC----ACCTGGGACT	123	-4	0.08%
CTCAAATTCCTGCACGG--CTGGGACT	102	-2	0.07%
CT----------CACGGACCTGGGACT	90	-10	0.06%
CTCAAATTCCTGCAC------GGGACT	80	-6	0.05%
CTCAAATTCCTGCAACGGACCTGGGACT	63	+1 Ins	0.04%

P58-P67-WT-U937-Syn54crGATA4d-KO-BC4

4h

Total | INDEL	Ins	Del	HDR
127015 | 1.65%	1.45%	0.20%	0.00%

Typical seqs	Reads	Type	pct.
TCCCTCCTCAAATTCCT|GCACGGACCT	124921	WT	98.35%
TCCCTCCTCAAATTCCTtGCACGGACCT	1646	+1 t	1.30%
TCCCTCCTCAAATTCCTCTGCACGGACCT	37	+2	0.03%
TCCCTCCTCAAATT---GCACGGACCT	31	-3	0.02%
TCCCTCCTCAAATTC-TGCACGGACCT	32	-1	0.03%
TCCCTCCTCAAATT--TGCACGGACCT	23	-2	0.02%
TCCCTCCTCAAATTCCTTTGCACGGACCT	17	+2	0.01%
TCCCTCCTCAAATTCCCTGCACGGACCT	13	+1 Ins	0.01%
TCCCTCCTCAAATTCCTtGCGCGGACCT	14	+1 t	0.01%

8h

Total | INDEL	Ins	Del	HDR
258398 | 3.34%	2.87%	0.71%	0.00%

Typical seqs	Reads	Type	pct.
TCCCTCCTCAAATTCCT|GCACGGACCT	249155	WT	96.42%
TCCCTCCTCAAATTCCTtGCACGGACCT	6638	+1 t	2.57%
TCCCTCCTCAAATTCCTCTGCACGGACCT	136	+2	0.05%
TCCCTCCTCAAATTC-TGCACGGACCT	104	-1	0.04%
TCCCTCCTCAAATT---GCACGGACCT	93	-3	0.04%
TCCCTCCTCAAATT----------CCT	92	-10	0.04%
TCCCTCCTCAAATT--TGCACGGACCT	75	-2	0.03%
TCCCTCCTCAAATTCCCTGCACGGACCT	51	+1 Ins	0.02%
TC----------------------CCT	39	-22	0.02%

12h

Total | INDEL	Ins	Del	HDR
50705 | 8.13%	6.91%	1.28%	0.00%

Typical seqs	Reads	Type	pct.
TCCCTCCTCAAATTCCT|GCACGGACCT	46551	WT	91.81%
TCCCTCCTCAAATTCCTtGCACGGACCT	3124	+1 t	6.16%
TCCCTCCTCAAATT----------CCT	52	-10	0.10%
TCCCTCCTCAAATTCCTCTGCACGGACCT	56	+2	0.11%
TCCCTCCTCAAATTC-TGCACGGACCT	44	-1	0.09%
TCCCTCCTCAAATT--TGCACGGACCT	38	-2	0.07%
TCCCTCCTCAAATT---GCACGGACCT	34	-3	0.07%
TCCCTCCTCAAATTCC-GCACGGACCT	24	-1	0.05%
TCCCTCCTCAAATTCCTtGCGCGGACCT	23	+1 t	0.05%

24h

Total | INDEL	Ins	Del	HDR
19697 | 15.19%	11.13%	4.62%	0.00%

Typical seqs	Reads	Type	pct.
TCCCTCCTCAAATTCCT|GCACGGACCT	16594	WT	84.25%
TCCCTCCTCAAATTCCTtGCACGGACCT	1954	+1 t	9.92%
TCCCTCCTCAAATT----------CCT	92	-10	0.47%
TCCCTCCT----------CACGGACCT	59	-10	0.30%
TCCCT-------------------CCT	44	-19	0.22%
TCCCTCCTCAAATT------CGGACCT	34	-6	0.17%
TCCCTCCTCAAATTCCTCTGCACGGACCT	35	+2	0.18%
TC----------------------CCT	31	-22	0.16%
TCCCTCCTCAAATTCCCTGCACGGACCT	28	+1 Ins	0.14%

48h

Total | INDEL	Ins	Del	HDR
102306 | 14.14%	8.97%	6.49%	0.00%

Typical seqs	Reads	Type	pct.
TCCCTCCTCAAATTCCT|GCACGGACCT	86489	WT	84.54%
TCCCTCCTCAAATTCCTtGCACGGACCT	8003	+1 t	7.82%
TCCCTCCTCAAATT----------CCT	617	-10	0.60%
TCCCTCCT---------GCACGGACCT	321	-9	0.31%
TCCCTCCT----------CACGGACCT	271	-10	0.26%
TCCCT-------------------CCT	246	-19	0.24%
TCCCTCCTCAAATT------CGGACCT	133	-6	0.13%
TCCCTCCTCAAATTCCTCTGCACGGACCT	110	+2	0.11%
TCCCTCCTCAAATT--TGCACGGACCT	105	-2	0.10%

P58-P67-WT-U937-Syn54crGATA4d-AAV6-KI-BC12

Total | INDEL	4h	Ins	Del	HDR
109700 | 1.63%		1.43%	0.16%	0.04%

Typical seqs	Reads	Type	pct.
TCCCTCCTCAAATTCCT|GCACGGACCT	107917	WT	98.37%
TCCCTCCTCAAATTCCTtGCACGGACCT	1372	+1 t	1.25%
TCCCT-------------CACG--CGTAGTTTA	37	-9 HDR	0.03%
TCCCTCCTCAAATTC-TGCACGGACCT	28	-1	0.03%
TCCCTCCTCAAATTCCTCTGCACGGACCT	21	+2	0.02%
TCCCTCCTCAAATT---GCACGGACCT	17	-3	0.02%
TCCCTCCTCAAATT--TGCACGGACCT	16	-2	0.01%
TCCCTCCTCAAATTCC-GCACGGACCT	15	-1	0.01%
TCCCTCCTCAAATTCCCTGCACGGACCT	13	+1 Ins	0.01%
TCCCTCCTCAAATTCCTGCAC-GACCT	13	-1	0.01%

Total | INDEL	8h	Ins	Del	HDR
58689 | 5.77%		4.17%	0.72%	0.88%

Typical seqs	Reads	Type	pct.
TCCCTCCTCAAATTCCT|GCACGGACCT	55302	WT	94.23%
TCCCTCCTCAAATTCCTtGCACGGACCT	2184	+1 t	3.72%
TCCCT-------------CACG--CGTAGTTTA	505	-9 HDR	0.86%
TCCCTCCTCAAATTC-TGCACGGACCT	53	-1	0.09%
TCCCTCCTCAAATT--TGCACGGACCT	47	-2	0.08%
TCCCTCCTCAAATTCCTCTGCACGGACCT	37	+2	0.06%
TCCCTCCTCAAATT---GCACGGACCT	35	-3	0.06%
TCCCTCCTCAAATTCC-GCACGGACCT	22	-1	0.04%
TCCCTCCCCAAATTCCTTGCACGGACCT	18	+1 Ins	0.03%
TCCCTCCTCAAAT----GCACGGACCT	18	-4	0.03%

Total | INDEL	12h	Ins	Del	HDR
242534 | 6.05%		3.52%	0.75%	1.77%

Typical seqs	Reads	Type	pct.
TCCCTCCTCAAATTCCT|GCACGGACCT	227868	WT	93.95%
TCCCTCCTCAAATTCCTtGCACGGACCT	7319	+1 t	3.02%
TCCCT-------------CACG--CGTAGTTTA	4203	-9 HDR	1.73%
TCCCTCCTCAAATTC-TGCACGGACCT	122	-1	0.05%
TCCCTCCTCAAATTCCTCTGCACGGACCT	112	+2	0.05%
TCCCTCCTCAAATT----------CCT	128	-10	0.05%
TCCCTCCTCAAATT---GCACGGACCT	98	-3	0.04%
TCCCTCCTCAAATT--TGCACGGACCT	102	-2	0.04%
TCCCTCCT---------GCACGGACCT	89	-9	0.04%
TCCCTCCTCAAATTCCCTGCACGGACCT	64	+1 Ins	0.03%

Total | INDEL	24h	Ins	Del	HDR
137832 | 19.69%		9.83%	3.09%	6.78%

Typical seqs	Reads	Type	pct.
TCCCTCCTCAAATTCCT|GCACGGACCT	110686	WT	80.31%
TCCCTCCTCAAATTCCTtGCACGGACCT	11444	+1 t	8.30%
TCCCT-------------CACG--CGTAGTTTA	9093	-9 HDR	6.60%
TCCCTCCTCAAATT--TGCACGGACCT	265	-2	0.19%
TCCCT-------------------CCT	236	-19	0.17%
TCCCTCCTCAAATT----------CCT	235	-10	0.17%
TCCCTCCTCAAATTC-TGCACGGACCT	178	-1	0.13%
TCCCTCCT----------CACGGACCT	130	-10	0.09%
TCCCTCCTCAAAT----GCACGGACCT	132	-4	0.10%
TCCCTCCTCAAATTCCTCTGCACGGACCT	115	+2	0.08%

Total | INDEL	48h	Ins	Del	HDR
114079 | 16.86%		8.26%	3.69%	4.91%

Typical seqs	Reads	Type	pct.
TCCCTCCTCAAATTCCT|GCACGGACCT	94840	WT	83.14%
TCCCTCCTCAAATTCCTtGCACGGACCT	7993	+1 t	7.01%
TCCCT-------------CACG--CGTAGTTTA	5482	-9 HDR	4.81%
TCCCTCCTCAAATT----------CCT	493	-10	0.43%
TCCCTCCT---------GCACGGACCT	318	-9	0.28%
TCCCTCCTCAAATTCCTCTGCACGGACCT	149	+2	0.13%
TCCCTCCTCAAATT------CGGACCT	133	-6	0.12%
TCCCTCCT----------CACGGACCT	129	-10	0.11%
TCCCT-------------------CCT	96	-19	0.08%
TCCCTCCTCAAATTC-TGCACGGACCT	105	-1	0.09%

P58-P67-WT-U937-Syn55crGATA4e-KO-BC5

Total | INDEL	4h	Ins	Del	HDR
66775 | 0.46%		0.29%	0.16%	0.00%

Typical seqs	Reads	Type	pct.
TCCAAGTCCCAGGTCCG|TGC**AGG**AATT	66471	WT	99.54%
TCCAAGTCCCAGGTCCGtGCAAGGAATT	106	+1 t	0.16%
TCCAAGTCCCAGGTCCGtTGCAGGAATT	33	+1 t	0.05%
TCCAAGTCCCA-GTCCGTGCAGGAATT	16	-1	0.02%
TCCAAGTCCCAGGTTCCGTGCAGGAATT	14	+1 Ins	0.02%
TCCAAGTCCCAGGTCC--GCAGGAATT	10	-2	0.01%
TCCAAGTCCCAGGT-CGTGCAGGAATT	9	-1	0.01%
TCCAAGTCCCAGGTC--TGCAGGAATT	9	-2	0.01%
TCCAAGTCCCA--TCCGTGCAGGAATT	7	-2	0.01%

Total | INDEL	8h	Ins	Del	HDR
171274 | 1.0%		0.53%	0.64%	0.00%

Typical seqs	Reads	Type	pct.
TCCAAGTCCCAGGTCCG|TGC**AGG**AATT	169276	WT	98.83%
TCCAAGTCCCAGGTCCGtGCAAGGAATT	491	+1 t	0.29%
TCCAAGTCCCAGGTCCGtTGCAGGAATT	196	+1 t	0.11%
TCCAAGTCCCA-GTCCGTGCAGGAATT	114	-1	0.07%
TCCAAGTCC----------CAGGAATT	68	-10	0.04%
TCCAAGTCCCAGGTC--TGCAGGAATT	50	-2	0.03%
TCCAAGTCCCAGGTCC--GCAGGAATT	44	-2	0.03%
TCCAAGTCCCAGGTCCGaTGCAGGAATT	39	+1 a	0.02%
TCCAAGTCCCAGGTCCGTGCA-GAATT	29	-1	0.02%

Total | INDEL	12h	Ins	Del	HDR
222803 | 1.57%		0.79%	1.28%	0.00%

Typical seqs	Reads	Type	pct.
TCCAAGTCCCAGGTCCG|TGC**AGG**AATT	218189	WT	97.93%
TCCAAGTCCCAGGTCCGtGCAAGGAATT	832	+1 t	0.37%
TCCAAGTCCCAGGTCCGtTGCAGGAATT	363	+1 t	0.16%
TCCAAGTCC----------CAGGAATT	278	-10	0.12%
TCCAAGTCCCA-GTCCGTGCAGGAATT	153	-1	0.07%
TCCAAGTCCCAGGTCC--GCAGGAATT	146	-2	0.07%
TCCAAGTCCCAGGTC--TGCAGGAATT	84	-2	0.04%
TCCAAGTCCCAGGTCC-TGCAGGAATT	63	-1	0.03%
TCCAAGTCCCAGGTCCGTTTGCAGGAATT	60	+2	0.03%

Total | INDEL	24h	Ins	Del	HDR
225493 | 3.60%		1.39%	3.71%	0.00%

Typical seqs	Reads	Type	pct.
TCCAAGTCCCAGGTCCG|TGC**AGG**AATT	213993	WT	94.90%
TCCAAGTCCCAGGTCCGtGCAAGGAATT	1460	+1 t	0.65%
TCCAAGTCC----------CAGGAATT	1261	-10	0.56%
TCCAAGTCCCAGGTCCGtTGCAGGAATT	783	+1 t	0.35%
TCCAAGTCCCAGGTCC--GCAGGAATT	214	-2	0.09%
TCCAAGTCCCA-GTCCGTGCAGGAATT	196	-1	0.09%
TCCAAGTCCCAGGTC--TGCAGGAATT	170	-2	0.08%
TCCAAGTCCCAGGTCC-TGCAGGAATT	172	-1	0.08%
TCCAAGTCCC-----------------	218	-19	0.10%

Total | INDEL	48h	Ins	Del	HDR
184371 | 5.23%		1.68%	4.75%	0.00%

Typical seqs	Reads	Type	pct.
TCCAAGTCCCAGGTCCG|TGC**AGG**AATT	172508	WT	93.57%
TCCAAGTCC----------CAGGAATT	1764	-10	0.96%
TCCAAGTCCCAGGTCCGtGCAAGGAATT	1373	+1 t	0.74%
TCCAAGTCCCAGGTCCGtTGCAGGAATT	720	+1 t	0.39%
TCCAAGTCCCAGGTCC--GCAGGAATT	286	-2	0.16%
TCCAAGTCCCAGGTC--TGCAGGAATT	232	-2	0.13%
TCCAAGTCCCA-GTCCGTGCAGGAATT	198	-1	0.11%
TCCAAGTCCC-----------------	270	-19	0.15%
TCCAAGTCCCAGGTCC-TGCAGGAATT	165	-1	0.09%

P58-P67-WT-U937-Syn55crGATA4e-AAV6-KI-BC13

4h — Total 116063 | INDEL 0.48% | Ins 0.26% | Del 0.18% | HDR 0.04%

Typical seqs	Reads	Type	pct.
TCCAAGTCCCAGGTCCG|TGC**AGG**AATT	115507	WT	99.52%
TCCAAGTCCCAGGTCCGtGCAAGGAATT	180	+1 t	0.16%
TCCAAGT-----GTTTAAACTACGCGT---------	41	-9 HDR	0.04%
TCCAAGTCCCA-GTCCGTGCAGGAATT	41	-1	0.04%
TCCAAGTCCCAGGTCCGtTGCAGGAATT	33	+1 t	0.03%
TCCAAGTCCCAGGTCCGaTGCAGGAATT	21	+1 a	0.02%
TCCAAGTCCCAGGTCC--GCAGGAATT	23	-2	0.02%
TCCAAGTCCCAGGTC--TGCAGGAATT	16	-2	0.01%
TCCAAGTCCCAGGT-CGTGCAGGAATT	11	-1	0.01%
TCCAAGTCCCAGGTCCGgTGCAGGAATT	11	+1 g	0.01%

8h — Total 32326 | INDEL 1.01% | Ins 0.33% | Del 0.31% | HDR 0.37%

Typical seqs	Reads	Type	pct.
TCCAAGTCCCAGGTCCG|TGC**AGG**AATT	32001	WT	98.99%
TCCAAGT-----GTTTAAACTACGCGT---------	115	-9 HDR	0.36%
TCCAAGTCCCAGGTCCGtTGCAGGAATT	60	+1 t	0.19%
TCCAAGTCCCAGGTCC--GCAGGAATT	23	-2	0.07%
TCCAAGTCCCA-GTCCGTGCAGGAATT	15	-1	0.05%
TCCAAGTCCCAGGTCCGaTGCAGGAATT	12	+1 a	0.04%
TCCAAGTCCCAGGTCC-TGCAGGAATT	10	-1	0.03%
TCCAAGTCCCAGGTCCGtGCAAGGAATT	8	+1 t	0.02%
TCCAAGTCCCAGGTCCG-GCAGGAATT	8	-1	0.02%
TCCAAGTCCCAGGTC--TGCAGGAATT	6	-2	0.02%

12h — Total 259094 | INDEL 2.52% | Ins 0.72% | Del 0.71% | HDR 1.09%

Typical seqs	Reads	Type	pct.
TCCAAGTCCCAGGTCCG|TGC**AGG**AATT	252568	WT	97.48%
TCCAAGT-----GTTTAAACTACGCGT---------	2709	-9 HDR	1.05%
TCCAAGTCCCAGGTCCGtGCAAGGAATT	1043	+1 t	0.40%
TCCAAGTCCCAGGTCCGtTGCAGGAATT	255	+1 t	0.10%
TCCAAGTCCCA-GTCCGTGCAGGAATT	214	-1	0.08%
TCCAAGTCC----------CAGGAATT	203	-10	0.08%
TCCAAGTCCCAGGTCC--GCAGGAATT	112	-2	0.04%
TCCAAGTCCCAGGTC--TGCAGGAATT	113	-2	0.04%
TCCAAGTCCCAGGTCC-TGCAGGAATT	87	-1	0.03%
TCCAA-------GTCCGTGCAGGAATT	70	-7	0.03%

24h — Total 319677 | INDEL 6.00% | Ins 1.30% | Del 1.86% | HDR 2.84%

Typical seqs	Reads	Type	pct.
TCCAAGTCCCAGGTCCG|TGC**AGG**AATT	300499	WT	94.00%
TCCAAGT-----GTTTAAACTACGCGT---------	8755	-9 HDR	2.74%
TCCAAGTCCCAGGTCCGtGCAAGGAATT	2004	+1 t	0.63%
TCCAAGTCC----------CAGGAATT	1003	-10	0.31%
TCCAAGTCCCAGGTCCGtTGCAGGAATT	905	+1 t	0.28%
TCCAAGTCCCAGGTCC--GCAGGAATT	324	-2	0.10%
TCCAAGTCCCAGGTC--TGCAGGAATT	316	-2	0.10%
TCCAAGTCCCA-GTCCGTGCAGGAATT	266	-1	0.08%
TCCAA-------GTCCGTGCAGGAATT	175	-7	0.05%
TCCAAGTCCCAGGTCC-TGCAGGAATT	143	-1	0.04%

48h — Total 136298 | INDEL 7.12% | Ins 1.52% | Del 2.77% | HDR 2.83%

Typical seqs	Reads	Type	pct.
TCCAAGTCCCAGGTCCG|TGC**AGG**AATT	126589	WT	92.88%
TCCAAGT-----GTTTAAACTACGCGT---------	3702	-9 HDR	2.72%
TCCAAGTCCCAGGTCCGtGCAAGGAATT	1008	+1 t	0.74%
TCCAAGTCC----------CAGGAATT	889	-10	0.65%
TCCAAGTCCCAGGTCCGtTGCAGGAATT	366	+1 t	0.27%
TCCAAGTCCCAGGTCC--GCAGGAATT	178	-2	0.13%
TCCAAGTCCCA-GTCCGTGCAGGAATT	165	-1	0.12%
TCCAAGTCCCAGGTC--TGCAGGAATT	128	-2	0.09%
TCCAAGTCCCAGGTCC-TGCAGGAATT	95	-1	0.07%
TCCAA-------GTCCGTGCAGGAATT	110	-7	0.08%

P58-P67-WT-U937-Syn56crGATA4g-AAV6-KI-BC14

4h — Total 98227 | INDEL 0.64% | Ins 0.46% | Del 0.16% | HDR 0.03%

Typical seqs	Reads	Type	pct.
CAGGTCCGTGCAGGAAT|TTG**AGG**AGGG	97594	WT	99.36%
CAGGTCCGTGCAGGAATtTTGAGGAGGG	360	+1 t	0.37%
CAGGTCCGTGCAGGAA-TTGAGGAGGG	41	-1	0.04%
CAGGTCCGTGCAGGAATtTGAAGGAGGG	22	+1 t	0.02%
---GTTTAAACTACGCGT-------------GAGGG	21	-9 HDR	0.02%
CAGGTCCGTGCAGGAA--TGAGGAGGG	15	-2	0.02%
CAGGTCCGTGCAGGAATTTTTGAGGAGGG	14	+2	0.01%
CAGGTCCGTGC---------AGGAGGG	14	-9	0.01%
CAGGTCCGTGCAGGAATTTGA-GAGGG	13	-1	0.01%
CAGGTCCGTGCAGGAATTTG-GGAGGG	8	-1	0.01%

8h — Total 214884 | INDEL 2.09% | Ins 0.92% | Del 0.42% | HDR 0.74%

Typical seqs	Reads	Type	pct.
CAGGTCCGTGCAGGAAT|TTG**AGG**AGGG	210398	WT	97.91%
---GTTTAAACTACGCGT-------------GAGGG	1571	-9 HDR	0.73%
CAGGTCCGTGCAGGAATtTTGAGGAGGG	1594	+1 t	0.74%
CAGGTCCGTGCAGGAA-TTGAGGAGGG	216	-1	0.10%
CAGGTCCGTGCAGGAATtTGAAGGAGGG	72	+1 t	0.03%
CAGGTCCGTGC---------AGGAGGG	55	-9	0.03%
CAGGTCCGTGCAGGAATTTTTGAGGAGGG	49	+2	0.02%
CAGGTCCGTGCAGG-ATTTGAGGAGGG	39	-1	0.02%
CAGGTCCGTGCAGGAATTTGA-GAGGG	25	-1	0.01%
CAGGTCCGTGCA-GAATTTGAGGAGGG	27	-1	0.01%

12h — Total 198989 | INDEL 3.82% | Ins 1.21% | Del 0.67% | HDR 1.94%

Typical seqs	Reads	Type	pct.
CAGGTCCGTGCAGGAAT|TTG**AGG**AGGG	191394	WT	96.18%
---GTTTAAACTACGCGT-------------GAGGG	3691	-9 HDR	1.85%
CAGGTCCGTGCAGGAATtTTGAGGAGGG	1824	+1 t	0.92%
CAGGTCCGTGCAGGAA-TTGAGGAGGG	195	-1	0.10%
CAGGTCCGTGC---------AGGAGGG	106	-9	0.05%
CAGGTCCGTGCAGGAATtTGAAGGAGGG	99	+1 t	0.05%
CAGGTCCGTGCAGGAATTTTTGAGGAGGG	65	+2	0.03%
CAGGTCCGTGCAGGAATTTGA-GAGGG	46	-1	0.02%
CAGGTCCGTGCAGGAA--TGAGGAGGG	44	-2	0.02%
CAGGTCCGTGCAGGAAT---AGGAGGG	38	-3	0.02%

24h — Total 225343 | INDEL 9.50% | Ins 2.07% | Del 2.47% | HDR 4.96%

Typical seqs	Reads	Type	pct.
CAGGTCCGTGCAGGAAT|TTG**AGG**AGGG	203941	WT	90.50%
---GTTTAAACTACGCGT-------------GAGGG	10897	-9 HDR	4.84%
CAGGTCCGTGCAGGAATtTTGAGGAGGG	3675	+1 t	1.63%
CAGGTCCGTGC---------AGGAGGG	922	-9	0.41%
CAGGTCCGTGCAGGAA-TTGAGGAGGG	430	-1	0.19%
CAGGTCCG----------TGAGGAGGG	344	-10	0.15%
CAGGTCCGTGCA-------------GG	300	-13	0.13%
CAGGTCCGTGCAGGAATtTGAAGGAGGG	200	+1 t	0.09%
CAGGTCCGTGC------------AGGG	142	-12	0.06%
C-------------------AGGAGGG	102	-19	0.05%

48h — Total 3026 | INDEL 13.12% | Ins 3.44% | Del 2.38% | HDR 7.30%

Typical seqs	Reads	Type	pct.
CAGGTCCGTGCAGGAAT|TTG**AGG**AGGG	2629	WT	86.88%
---GTTTAAACTACGCGT-------------GAGGG	221	-9 HDR	7.30%
CAGGTCCGTGCAGGAATtTTGAGGAGGG	91	+1 t	3.01%
CAGGTCCGTGC---------AGGAGGG	21	-9	0.69%
CAGGTCCGTGCAGGAA-TTGAGGAGGG	6	-1	0.20%
CAGGTCCGTGCA-------------GG	4	-13	0.13%
CAGGTCCG----------TGAGGAGGG	5	-10	0.17%
CAGGTCCGTGCAGGAA------GAGGG	3	-6	0.10%
CAGGTCCGTGCAGGAATtTTGAGGGGGG	3	+1 t	0.10%

P58-P67-WT-U937-Syn57crGATA4h-KO-BC7

4h — Total 98321 | INDEL 0.22% — Ins 0.10% — Del 0.12% — HDR 0.00%

Typical seqs	Reads	Type	pct.
GTCCGTGCAGGAATTTG|AGG**AGG**GAAG	98102	WT	99.78%
GTCCGTGCAGGAATTTTGAGGAGGGAAG	55	+1 Ins	0.06%
GTCCGTGCAGGAATTTGaAGGAGGGAAG	26	+1 a	0.03%
GTCCGTGC---------AGGAGGGAAG	20	-9	0.02%
GTCCGTGCAGGAATTT-AGGAGGGAAG	15	-1	0.02%
GTCCGTGCAGGAATT-GAGGAGGGAAG	14	-1	0.01%
GTCCGTGCAGGAATT--AGGAGGGAAG	10	-2	0.01%
GTCCGTGCAGGAATTTGAGG-GGGAAG	9	-1	0.01%
GTCCGTGCAGGAATTTGgAGGAGGGAAG	6	+1 g	0.01%

8h — Total 240294 | INDEL 0.5% — Ins 0.17% — Del 0.54% — HDR 0.00%

Typical seqs	Reads	Type	pct.
GTCCGTGCAGGAATTTG|AGG**AGG**GAAG	238592	WT	99.29%
GTCCGTGCAGGAATTTTGAGGAGGGAAG	221	+1 Ins	0.09%
GTCCGTGC---------AGGAGGGAAG	127	-9	0.05%
GTCCGTGCAGGAATTTGaAGGAGGGAAG	82	+1 a	0.03%
GTCCGTGCAGGAATTTG-GGAGGGAAG	62	-1	0.03%
GTCCGTGCAGGAATTTGA-GAGGGAAG	43	-1	0.02%
GTCCGTGCAGGAATT-GAGGAGGGAAG	46	-1	0.02%
GTCCGTGCAGGAATT--AGGAGGGAAG	28	-2	0.01%
GTCCGTGCAGGAATTT--GGAGGGAAG	26	-2	0.01%

12h — Total 210492 | INDEL 0.77% — Ins 0.35% — Del 1.22% — HDR 0.00%

Typical seqs	Reads	Type	pct.
GTCCGTGCAGGAATTTG|AGG**AGG**GAAG	207193	WT	98.43%
GTCCGTGCAGGAATTTTGAGGAGGGAAG	394	+1 Ins	0.19%
GTCCGTGC---------AGGAGGGAAG	184	-9	0.09%
GTCCGTGCAGGAATTTGaAGGAGGGAAG	164	+1 a	0.08%
GTCCGTGCAGGAATT-GAGGAGGGAAG	68	-1	0.03%
GTCCGTGCAGGAATTTGA-GAGGGAAG	39	-1	0.02%
GTCCGTGCA-------------GGAAG	30	-13	0.01%
GTCCGTGCAGGAATTTG-GGAGGGAAG	25	-1	0.01%
GTCCGTGC------------AGGGAAG	28	-12	0.01%

24h — Total 299849 | INDEL 3.33% — Ins 0.45% — Del 4.18% — HDR 0.00%

Typical seqs	Reads	Type	pct.
GTCCGTGCAGGAATTTG|AGG**AGG**GAAG	285966	WT	95.37%
GTCCGTGC---------AGGAGGGAAG	2350	-9	0.78%
GTCCGTGCA-------------GGAAG	574	-13	0.19%
GTCCGTGCAGGAATTTTGAGGAGGGAAG	531	+1 Ins	0.18%
GTCCGTGC------------AGGGAAG	420	-12	0.14%
GTCCGTGCA------------------	297	-20	0.10%
GTCCGTGCAGGAATTTGaAGGAGGGAAG	300	+1 a	0.10%
GTCCGTGCAGGAATTT--GGAGGGAAG	226	-2	0.08%
GTCCGT---------------------	235	-33	0.08%

48h — Total 6046 | INDEL 5.16% — Ins 0.22% — Del 4.95% — HDR 0.00%

Typical seqs	Reads	Type	pct.
GTCCGTGCAGGAATTTG|AGG**AGG**GAAG	5734	WT	94.84%
GTCCGTGC---------AGGAGGGAAG	122	-9	2.02%
GTCCGTGC------------AGGGAAG	28	-12	0.46%
GTCCGTGCAGGAATTT--GGAGGGAAG	22	-2	0.36%
GTCCGTGCA-------------GGAAG	14	-13	0.23%
GTCCGTGC-------------------	11	-19	0.18%
GTCCGTGCAGGAATTT-AGGAGGGAAG	11	-1	0.18%
GTCCGTGCA------------------	8	-20	0.13%
GTCCGT---------------------	8	-33	0.13%
GTCCGTGCAGGAATTT---GAGGGAAG	6	-3	0.10%

P58-P67-WT-U937-Syn57crGATA4h-AAV6-KI-BC15

4h

Total | INDEL	Ins	Del	HDR
132253 | 0.23%	0.07%	0.12%	0.03%

Typical seqs	Reads	Type	pct.
GTCCGTGCAGGAATTTG|AGGAGGGAAG	131953	WT	99.77%
GTCCGTGCAGGAATTTTGAGGAGGGAAG	56	+1 Ins	0.04%
GTTTAAACTACGCGT-------------GAGGGAAG	39	-9 HDR	0.03%
GTCCGTGCAGGAATTTGaAGGAGGGAAG	31	+1 a	0.02%
GTCCGTGCAGGAATTTGA-GAGGGAAG	20	-1	0.02%
GTCCGTGCAGGAATT-GAGGAGGGAAG	19	-1	0.01%
GTCCGTGC---------AGGAGGGAAG	15	-9	0.01%
GTCCGTGCAGGAATTTGAGG-GGGAAG	10	-1	0.01%
GTCCGTGCAGGA-----AGGAGGGAAG	9	-5	0.01%
GTCCGTGCAGGAATTT--GGAGGGAAG	9	-2	0.01%

8h

Total | INDEL	Ins	Del	HDR
263180 | 0.91%	0.17%	0.28%	0.46%

Typical seqs	Reads	Type	pct.
GTCCGTGCAGGAATTTG|AGGAGGGAAG	260783	WT	99.09%
GTTTAAACTACGCGT-------------GAGGGAAG	1175	-9 HDR	0.45%
GTCCGTGCAGGAATTTTGAGGAGGGAAG	221	+1 Ins	0.08%
GTCCGTGCAGGAATTTGaAGGAGGGAAG	104	+1 a	0.04%
GTCCGTGC---------AGGAGGGAAG	59	-9	0.02%
GTCCGTGCAGGAATTTGA-GAGGGAAG	52	-1	0.02%
GTCCGTGCAGGAATT--AGGAGGGAAG	47	-2	0.02%
GTCCGTGCAGGAAT---AGGAGGGAAG	48	-3	0.02%
GTCCGTGCAGGAATT-GAGGAGGGAAG	43	-1	0.02%
GTCCGTGCAGGAATTT-AGGAGGGAAG	40	-1	0.02%

12h

Total | INDEL	Ins	Del	HDR
28852 | 2.28%	0.06%	0.63%	1.59%

Typical seqs	Reads	Type	pct.
GTCCGTGCAGGAATTTG|AGGAGGGAAG	28194	WT	97.72%
GTTTAAACTACGCGT-------------GAGGGAAG	418	-9 HDR	1.45%
GTCCGTGC---------AGGAGGGAAG	22	-9	0.08%
GTCCGTGCAGGAATTT--GGAGGGAAG	17	-2	0.06%
GTCCGTGCAGGAATTTG-GGAGGGAAG	14	-1	0.05%
GTCCGTGCAGGAATT--AGGAGGGAAG	13	-2	0.05%
GTCCGTGCAGGAATTT---GAGGGAAG	10	-3	0.03%
-----------------------AAG	9	-26	0.03%
GTCCGTGCAGGAATTT-AGGAGGGAAG	9	-1	0.03%
GTCCGTGCAGGAAT---AGGAGGGAAG	6	-3	0.02%

24h

Total | INDEL	Ins	Del	HDR
309153 | 6.05%	0.45%	1.82%	3.79%

Typical seqs	Reads	Type	pct.
GTCCGTGCAGGAATTTG|AGGAGGGAAG	290436	WT	93.95%
GTTTAAACTACGCGT-------------GAGGGAAG	11203	-9 HDR	3.62%
GTCCGTGC---------AGGAGGGAAG	1402	-9	0.45%
GTCCGTGCAGGAATTTTGAGGAGGGAAG	582	+1 Ins	0.19%
GTCCGTGC------------AGGGAAG	311	-12	0.10%
GTCCGTGCAGGAATTTGaAGGAGGGAAG	258	+1 a	0.08%
GTCCGTGCA-------------GGAAG	246	-13	0.08%
GTCCGTGCAGGAATTTG-GGAGGGAAG	147	-1	0.05%
GTCCGTGCAGGAATTT--GGAGGGAAG	143	-2	0.05%
-----------------AGGAGGGAAG	146	-19	0.05%

48h

Total | INDEL	Ins	Del	HDR
122448 | 7.05%	0.63%	2.62%	3.80%

Typical seqs	Reads	Type	pct.
GTCCGTGCAGGAATTTG|AGGAGGGAAG	113815	WT	92.95%
GTTTAAACTACGCGT-------------GAGGGAAG	4454	-9 HDR	3.64%
GTCCGTGC---------AGGAGGGAAG	1015	-9	0.83%
GTCCGTGCAGGAATTTTGAGGAGGGAAG	367	+1 Ins	0.30%
GTCCGTGCA-------------GGAAG	179	-13	0.15%
GTCCGTGCAGGAATTTGaAGGAGGGAAG	157	+1 a	0.13%
GTCCGTGC------------AGGGAAG	117	-12	0.10%
GTCCGTGCAGGAATTT--GGAGGGAAG	103	-2	0.08%
GTCCGTGCAGGAATTTG-GGAGGGAAG	78	-1	0.06%
GTCCGTGCAGGAATTT---GAGGGAAG	70	-3	0.06%

P58-P67-WT-U937-Syn58crGATA4i-KO-BC8

Total | INDEL	4h	Ins	Del	HDR
13190 | 0.48%		0.35%	0.13%	0.00%

Typical seqs	Reads	Type	pct.
TCCGTGCAGGAATTTGA|GGA**GGG**AAGA	13127	WT	99.52%
TCCGTGCAGGAATTTGAaGGAGGGAAGA	44	+1 a	0.33%
TCCGTGCAGGAATTTGA-GAGGGAAGA	3	-1	0.02%
TCCGTGCA---------GGAGGGAAGA	3	-9	0.02%
TCCGTGCAGGAATTTGAGGGGGGAAGA	39	-1	0.30%
TCCGTGCAGGAATTTG-GGAGGGAAGA	2	-1	0.02%
TCCGTGCAGGAATTTGAaGGAGGGAAGG	2	+1 a	0.02%
TCCGTGCA--------AGGAGGGAAGA	2	-8	0.02%

Total | INDEL	8h	Ins	Del	HDR
90960 | 0.83%		0.46%	0.59%	0.00%

Typical seqs	Reads	Type	pct.
TCCGTGCAGGAATTTGA|GGA**GGG**AAGA	90000	WT	98.94%
TCCGTGCAGGAATTTGAaGGAGGGAAGA	287	+1 a	0.32%
TCCGTGCAGGAATTTTGAGGAGGGAAGA	87	+1 Ins	0.10%
TCCGTGCAGGAATTTG-GGAGGGAAGA	53	-1	0.06%
TCCGTGCA---------GGAGGGAAGA	27	-9	0.03%
TCCGTGCAGGAATTTGAGGA-GGAAGA	24	-1	0.03%
TCCGTGCAGGAA-TTGAGGAGGGAAGA	22	-1	0.02%
TCCGTGCAGGAATTTGA-GAGGGAAGA	20	-1	0.02%
TCCGTGCA-------------GGAAGA	17	-13	0.02%

Total | INDEL	12h	Ins	Del	HDR
93265 | 1.41%		0.66%	1.22%	0.00%

Typical seqs	Reads	Type	pct.
TCCGTGCAGGAATTTGA|GGA**GGG**AAGA	91510	WT	98.12%
TCCGTGCAGGAATTTGAaGGAGGGAAGA	441	+1 a	0.47%
TCCGTGCAGGAATTTTGAGGAGGGAAGA	114	+1 Ins	0.12%
TCCGTGCA---------GGAGGGAAGA	98	-9	0.11%
TCCGTGCAGGAATTTG-GGAGGGAAGA	57	-1	0.06%
TCCGTGC------------AGGGAAGA	50	-12	0.05%
TCCGTGCAGGAATTTGA-GAGGGAAGA	42	-1	0.05%
TCCGTGCAGGAATTT---GAGGGAAGA	40	-3	0.04%
TCCGTGCA-------------GGAAGA	26	-13	0.03%

Total | INDEL	24h	Ins	Del	HDR
10630 | 6.0%		1.65%	4.52%	0.00%

Typical seqs	Reads	Type	pct.
TCCGTGCAGGAATTTGA|GGA**GGG**AAGA	9974	WT	93.83%
TCCGTGCAGGAATTTGAaGGAGGGAAGA	152	+1 a	1.43%
TCCGTGCA---------GGAGGGAAGA	89	-9	0.84%
TCCGTGCA-------------GGAAGA	52	-13	0.49%
TCCGTGCAGGAATTT---GAGGGAAGA	39	-3	0.37%
TCCGTGCAGGAATTTG-GGAGGGAAGA	34	-1	0.32%
TCCGTGC------------AGGGAAGA	32	-12	0.30%
TCCGTGCA-------------------	27	-20	0.25%
TCCGT----------------------	24	-33	0.23%

Total | INDEL	48h	Ins	Del	HDR
51446 | 6.32%		1.69%	5.87%	0.00%

Typical seqs	Reads	Type	pct.
TCCGTGCAGGAATTTGA|GGA**GGG**AAGA	47556	WT	92.44%
TCCGTGCAGGAATTTGAaGGAGGGAAGA	575	+1 a	1.12%
TCCGTGCA---------GGAGGGAAGA	477	-9	0.93%
TCCGTGCA-------------GGAAGA	249	-13	0.48%
TCCGTGC------------AGGGAAGA	194	-12	0.38%
TCCGTGCAGGAATTT---GAGGGAAGA	140	-3	0.27%
TCCGTGCAGGAATTTTGAGGAGGGAAGA	132	+1 Ins	0.26%
TCCGTGCAGGAATTT--GGAGGGAAGA	76	-2	0.15%
TCCGTGCAGGAATTTG-GGAGGGAAGA	80	-1	0.16%

P58-P67-WT-U937-Syn58crGATA4i-AAV6-KI-BC16

Total | INDEL	4h	Ins	Del	HDR
79888 | 0.45%		0.27%	0.16%	0.02%
**Typical seqs**		**Reads**	**Type**	**pct.**
TCCGTGCAGGAATTTGA|GGA**GGG**AAGA		79527	WT	99.55%
TCCGTGCAGGAATTTGAaGGAGGGAAGA		157	+1 a	0.20%
TCCGTGCAGGAATTTTGAGGAGGGAAGA		34	+1 Ins	0.04%
TCCGTGCAGGAATTTGAGGA-GGAAGA		21	-1	0.03%
TTTAAACTACGCGT-------------GAGGGAAGA		15	-9 HDR	0.02%
TCCGTGCAGGAATTTG-GGAGGGAAGA		15	-1	0.02%
TCCGTGCAGGAATTTGA-GAGGGAAGA		13	-1	0.02%
TCCGTGCA---------GGAGGGAAGA		8	-9	0.01%
TC--------------AGGAGGGAAGA		7	-14	0.01%
TCCGTGCAGGAATTTGA----------		11	-13	0.01%

Total | INDEL	8h	Ins	Del	HDR
194326 | 1.28%		0.41%	0.33%	0.54%
**Typical seqs**		**Reads**	**Type**	**pct.**
TCCGTGCAGGAATTTGA|GGA**GGG**AAGA		191838	WT	98.72%
TTTAAACTACGCGT-------------GAGGGAAGA		972	-9 HDR	0.50%
TCCGTGCAGGAATTTGAaGGAGGGAAGA		514	+1 a	0.26%
TCCGTGCAGGAATTTTGAGGAGGGAAGA		160	+1 Ins	0.08%
TCCGTGCAGGAATTTG-GGAGGGAAGA		83	-1	0.04%
TCCGTGCAGGAATTTGAGGA-GGAAGA		77	-1	0.04%
TCCGTGCAGGAATTTGA-GAGGGAAGA		72	-1	0.04%
TCCGTGCA---------GGAGGGAAGA		49	-9	0.03%
TCCGTGCAGGAATTT--GGAGGGAAGA		30	-2	0.02%
TCCGTGCAGGAATTT---GAGGGAAGA		27	-3	0.01%

Total | INDEL	12h	Ins	Del	HDR
31908 | 4.01%		0.90%	0.84%	2.26%
**Typical seqs**		**Reads**	**Type**	**pct.**
TCCGTGCAGGAATTTGA|GGA**GGG**AAGA		30630	WT	95.99%
TTTAAACTACGCGT-------------GAGGGAAGA		675	-9 HDR	2.12%
TCCGTGCAGGAATTTGAaGGAGGGAAGA		230	+1 a	0.72%
TCCGTGCAGGAATTTGA-GAGGGAAGA		36	-1	0.11%
TCCGTGCA---------GGAGGGAAGA		30	-9	0.09%
TCCGTGCAGGAATT--AGGAGGGAAGA		20	-2	0.06%
TCCGTGCAGGAATTTG-GGAGGGAAGA		16	-1	0.05%
TCCGTGCAGGAATTT-AGGAGGGAAGA		6	-1	0.02%
TCCGTGCAGGAA----AGGAGGGAAGA		6	-4	0.02%
TCCGTGCAGGAATTT---GAGGGAAGA		5	-3	0.02%

Total | INDEL	24h	Ins	Del	HDR
231683 | 8.96%		1.30%	2.43%	5.23%
**Typical seqs**		**Reads**	**Type**	**pct.**
TCCGTGCAGGAATTTGA|GGA**GGG**AAGA		210927	WT	91.04%
TTTAAACTACGCGT-------------GAGGGAAGA		11423	-9 HDR	4.93%
TCCGTGCAGGAATTTGAaGGAGGGAAGA		2232	+1 a	0.96%
TCCGTGCA---------GGAGGGAAGA		688	-9	0.30%
TCCGTGCA-------------GGAAGA		436	-13	0.19%
TCCGTGCAGGAATTTG-GGAGGGAAGA		355	-1	0.15%
TCCGTGCAGGAATTT---GAGGGAAGA		339	-3	0.15%
TCCGTGC------------AGGGAAGA		298	-12	0.13%
TCCGTGCAGGAATTTTGAGGAGGGAAGA		267	+1 Ins	0.12%
TCCGTGCAGGAATTTGA-GAGGGAAGA		197	-1	0.09%

Total | INDEL	48h	Ins	Del	HDR
85127 | 10.52%		1.60%	3.32%	5.60%
**Typical seqs**		**Reads**	**Type**	**pct.**
TCCGTGCAGGAATTTGA|GGA**GGG**AAGA		76172	WT	89.48%
TTTAAACTACGCGT-------------GAGGGAAGA		4537	-9 HDR	5.33%
TCCGTGCAGGAATTTGAaGGAGGGAAGA		863	+1 a	1.01%
TCCGTGCA---------GGAGGGAAGA		498	-9	0.59%
TCCGTGCAGGAATTTTGAGGAGGGAAGA		242	+1 Ins	0.28%
TCCGTGC------------AGGGAAGA		225	-12	0.26%
TCCGTGCA-------------GGAAGA		144	-13	0.17%
TCCGTGCAGGAATTT---GAGGGAAGA		133	-3	0.16%
TCCGTGCAGGAATTTG-GGAGGGAAGA		103	-1	0.12%
TCCGTGCAGGAATTT--GGAGGGAAGA		99	-2	0.12%

P68-P70-WT-U937-Syn45crHBG2-e-AAVKI-BC19

4h — Total | INDEL: 312493 | 5.21% — Ins 3.33% — Del 1.52% — HDR 0.35%

Typical seqs	Reads	Type	pct.
ATTGAGATAGTGTGGGG|AAG**GGG**CCCC	296223	WT	94.79%
ATTGAGATAGTGTGGG-AAGGGGCCCC	1164	-1	0.37%
ATTGAGATAGTGTGGGGGAGGGGCCCCCAAG	1159	+4	0.37%
ATTGAGATAGGTTT---------------AAACTA	1026	-7 HDR	0.33%
ATTGAGATAGTGTGGGGAGGGGGCCCCCAAG	936	+4	0.30%
ATTGAGATAGTGCGGGGAAGGGGCCCCCAAG	840	+4	0.27%
ATTGAGATAGTGTGGG-AAGGGGCCCCCAAG	810	+3	0.26%
ATTGAGATAGTGTGGGGGAAGGGGCCCCCAAG	494	+5	0.16%
ATTGAGATAGTGTGGGGgAAGGGGCCCC	377	+1 g	0.12%
ATTGAGATAGTGTGGGG-AGGGGCCCC	327	-1	0.10%

8h — Total | INDEL: 271448 | 5.95% — Ins 3.38% — Del 2.01% — HDR 0.56%

Typical seqs	Reads	Type	pct.
ATTGAGATAGTGTGGGG|AAG**GGG**CCCC	255290	WT	94.05%
ATTGAGATAGGTTT---------------AAACTA	1439	-7 HDR	0.53%
ATTGAGATAGTGTGGG-AAGGGGCCCC	1138	-1	0.42%
ATTGAGATAGTGTGGGGGAGGGGCCCCCAAG	917	+4	0.34%
ATTGAGATAGTGTGGG-AAGGGGCCCCCAAG	843	+3	0.31%
ATTGAGATAGTGTGGGGAGGGGGCCCCCAAG	712	+4	0.26%
ATTGAGATAGTGCGGGGAAGGGGCCCCCAAG	687	+4	0.25%
ATTGAGATAGTGTGGGGGAAGGGGCCCCCAAG	534	+5	0.20%
ATTGAGATAGTGTGGGGgAAGGGGCCCC	431	+1 g	0.16%
ATTGAGATAGTGTGG--AAGGGGCCCC	281	-2	0.10%

12h — Total | INDEL: 352638 | 7.07% — Ins 3.69% — Del 2.62% — HDR 0.76%

Typical seqs	Reads	Type	pct.
ATTGAGATAGTGTGGGG|AAG**GGG**CCCC	327720	WT	92.93%
ATTGAGATAGGTTT---------------AAACTA	2446	-7 HDR	0.69%
ATTGAGATAGTGTGGG-AAGGGGCCCC	1403	-1	0.40%
ATTGAGATAGTGTGGGGGAGGGGCCCCCAAG	1159	+4	0.33%
ATTGAGATAGTGTGGG-AAGGGGCCCCCAAG	1038	+3	0.29%
ATTGAGATAGTGTGGGGAGGGGGCCCCCAAG	1014	+4	0.29%
ATTGAGATAGTGCGGGGAAGGGGCCCCCAAG	909	+4	0.26%
ATTGAGATAGTGTGGGGgAAGGGGCCCC	725	+1 g	0.21%
ATTGAGATAGTGTGGGGGAAGGGGCCCCCAAG	682	+5	0.19%
ATTGAGATAGTGTGG------GGCCCCCAAG	533	-2	0.15%

24h — Total | INDEL: 250631 | 10.72% — Ins 4.04% — Del 5.37% — HDR 1.32%

Typical seqs	Reads	Type	pct.
ATTGAGATAGTGTGGGG|AAG**GGG**CCCC	223757	WT	89.28%
ATTGAGATAGGTTT---------------AAACTA	3072	-7 HDR	1.23%
ATTGAGATAGTGTGGG-AAGGGGCCCC	1525	-1	0.61%
ATTGAGATAGTGTGG------GGCCCC	1110	-6	0.44%
ATTGAGATAGTGTGG------GGCCCCCAAG	1100	-2	0.44%
ATTGAGATAGTGTGGGGGAGGGGCCCCCAAG	784	+4	0.31%
ATTGAGATAGTGTGGG-AAGGGGCCCCCAAG	750	+3	0.30%
ATTGAGATAGTGTGGGGGAAGGGGCCCCCAAG	692	+5	0.28%
ATTGAGATAGTGTGGGGAGGGGGCCCCCAAG	641	+4	0.26%
ATTGAGATAGTGTGGGGgAAGGGGCCCC	594	+1 g	0.24%

48h — Total | INDEL: 486321 | 14.90% — Ins 6.17% — Del 6.90% — HDR 1.83%

Typical seqs	Reads	Type	pct.
ATTGAGATAGTGTGGGG|GGC**CCC**CAAG	413841	WT	85.10%
ATTGAGATAGGTTT---------------AAACTA	8291	-7 HDR	1.70%
ATTGAGATAGTGTGG------GGCCCC	3754	-10	0.77%
ATTGAGATAGTGTGG------GGCCCCCAAG	3575	-6	0.74%
ATTGAGATAGTGTGGGGgGGCCCCCAAG	3494	+1 g	0.72%
ATTGAGATAGTGTGGG-AAGGGGCCCCCAAG	2803	-1	0.58%
ATTGAGATAGTGTGGG-AAGGGGCCCC	2445	-5	0.50%
ATTGAGATAGTGTGGGGGAGGGGCCCCCAAG	2158	0	0.44%
ATTGAGATAGTGTGGGGAGGGGGCCCCCAAG	1704	0	0.35%
ATTGAGATAGTGTGGGGGAAGGGGCCCC	1521	-3	0.31%

P68-P70-WT-U937-Syn46crHBG2-f-AAVKI-BC20

4h

Total	INDEL	Ins	Del	HDR
101884	5.06%	3.75%	1.22%	0.09%

Typical seqs	Reads	Type	pct.
CATTGAGATAGTGTGGG|GAA**GGG**GCCC	96728	WT	94.94%
CATTGAGATAGTGTGG-GAAGGGGCCC	720	-1	0.71%
CATTGAGATAGTGTGGG-AAGGGGCCCCCAA	419	+3	0.41%
CATTGAGATAGTGTGGGGGAGGGGCCCCCAA	369	+4	0.36%
CATTGAGATAGTGTGGGgGAAGGGGCCC	340	+1 g	0.33%
CATTGAGATAGTGTGGGAGGGGGCCCCCAA	300	+4	0.29%
CATTGAGATAGCGTGGGGAAGGGGCCCCCAA	274	+4	0.27%
CATTGAGATAGTGCGGGGAAGGGGCCCCCAA	243	+4	0.24%
CATTGAGATAGTGTGGGGGAAGGGGCCCCCAA	217	+5	0.21%
CATTGAGATAGTGTGGGGAAGGAGCCCCCAA	101	+4	0.10%

8h

Total	INDEL	Ins	Del	HDR
294490	6.43%	4.18%	1.82%	0.43%

Typical seqs	Reads	Type	pct.
CATTGAGATAGTGTGGG|GAA**GGG**GCCC	275546	WT	93.57%
CATTGAGATAGTGTGG-GAAGGGGCCC	1817	-1	0.62%
CATTGAGATAGTGTGGG-AAGGGGCCCCCAA	1281	+3	0.43%
CATTGAGATAGGTTT---------------AAACT	1227	-7 HDR	0.42%
CATTGAGATAGTGTGGGgGAAGGGGCCC	1198	+1 g	0.41%
CATTGAGATAGTGTGGGGGAGGGGCCCCCAA	1037	+4	0.35%
CATTGAGATAGTGTGGGGGAAGGGGCCCCCAA	984	+5	0.33%
CATTGAGATAGCGTGGGGAAGGGGCCCCCAA	894	+4	0.30%
CATTGAGATAGTGTGGGAGGGGGCCCCCAA	852	+4	0.29%
CATTGAGATAGTGCGGGGAAGGGGCCCCCAA	769	+4	0.26%

12h

Total	INDEL	Ins	Del	HDR
10992	7.66%	4.79%	2.19%	0.68%

Typical seqs	Reads	Type	pct.
CATTGAGATAGTGTGGG|GAA**GGG**GCCC	10150	WT	92.34%
CATTGAGATAGTGTGG-GAAGGGGCCC	133	-1	1.21%
CATTGAGATAGTGTGGG-AAGGGGCCCCCAA	96	+3	0.87%
CATTGAGATAGGTTT---------------AAACT	75	-7 HDR	0.68%
CATTGAGATAGTGTGGGgGAAGGGGCCC	70	+1 g	0.64%
CATTGAGATAGTGTGGGGGAAGGGGCCCCCAA	47	+5	0.43%
CATTGAGATAGTGTGGGGGAGGGGCCCCCAA	26	+4	0.24%
CATTGAGATAGCGTGGGGAAGGGGCCCCCAA	29	+4	0.26%
CATTGAGATAGTGTGG--AAGGGGCCC	15	-2	0.14%
CATTGAGATAGTGTGGGGAAGGAGCCCCCAA	13	+4	0.12%

24h

Total	INDEL	Ins	Del	HDR
38924	14.10%	6.65%	6.09%	1.36%

Typical seqs	Reads	Type	pct.
CATTGAGATAGTGTGGG|GAA**GGG**GCCC	33437	WT	85.90%
CATTGAGATAGTGTGG-GAAGGGGCCC	767	-1	1.97%
CATTGAGATAGTGTGGG-AAGGGGCCCCCAA	544	+3	1.40%
CATTGAGATAGGTTT---------------AAACT	503	-7 HDR	1.29%
CATTGAGATAGTGTGGGGGAAGGGGCCCCCAA	476	+5	1.22%
CATTGAGATAGTGTGGGgGAAGGGGCCC	356	+1 g	0.91%
CATTGAGATAGTGTGG------GGCCCCCAA	164	-2	0.42%
CATTGAGATAGTGT-------GGGCCCCCAA	147	-3	0.38%
CATTGAGATAGTGTGG--AAGGGGCCC	114	-2	0.29%
CATTGAGATAGTGT------GGGGCCC	124	-6	0.32%

48h

Total	INDEL	Ins	Del	HDR
467106	14.29%	7.68%	5.34%	1.27%

Typical seqs	Reads	Type	pct.
CATTGAGATAGTGTGGG|GGG**CCC**CCAA	400370	WT	85.71%
CATTGAGATAGTGTGG-GAAGGGGCCC	6197	-5	1.33%
CATTGAGATAGTGTGGG-AAGGGGCCCCCAA	6071	-1	1.30%
CATTGAGATAGGTTT---------------AAACT	5595	-7 HDR	1.20%
CATTGAGATAGTGTGGGgGGGCCCCCAA	5427	+1 g	1.16%
CATTGAGATAGTGTGGGGGAAGGGGCCC	3454	-3	0.74%
CATTGAGATAGTGTGGGGGAGGGGCCCCCAA	2122	0	0.45%
CATTGAGATAGCGTGGGGAAGGGGCCCCCAA	1800	0	0.39%
CATTGAGATAGTGTGGGGAGGGGGCCCCCAA	1676	0	0.36%
CATTGAGATAGTGT------GGGGCCC	1564	-10	0.33%

P68-P70-WT-U937-Syn47crHBG2-g-AAVKI-BC15

4h

Total | INDEL	Ins	Del	HDR
393460 | 6.03%	**4.14%**	**1.53%**	**0.37%**

Typical seqs	Reads	Type	pct.
GCATTGAGATAGTGTGG|GGA**AGG**GGCC	369721	WT	93.97%
GCATTGAGATAGTGTGGGGGAGGGGCCCCCA	1509	+4	0.38%
GCATTGAGATAGGTTT---------------AAAC	1362	-7 HDR	0.35%
GCATTGAGATAGTGTG-GGAAGGGGCC	1373	-1	0.35%
GCATTGAGATAGCGTGGGGAAGGGGCCCCCA	1205	+4	0.31%
GCATTGAGATAGTGCGGGGAAGGGGCCCCCA	1147	+4	0.29%
GCATTGAGATAGTGTGGGGAGGGGGCCCCCA	1116	+4	0.28%
GCATTGAGATAGTGTGGG-AAGGGGCCCCCA	1066	+3	0.27%
GCATTGAGATAGTTGTGGGGAAGGGGCC	754	+1 Ins	0.19%
GCATTGAGATAGTGTGGGGGAAGGGGCCCCCA	705	+5	0.18%

8h

Total | INDEL	Ins	Del	HDR
332836 | 6.47%	**4.22%**	**1.74%**	**0.51%**

Typical seqs	Reads	Type	pct.
GCATTGAGATAGTGTGG|GGA**AGG**GGCC	311313	WT	93.53%
GCATTGAGATAGGTTT---------------AAAC	1595	-7 HDR	0.48%
GCATTGAGATAGTGTG-GGAAGGGGCC	1330	-1	0.40%
GCATTGAGATAGTGTGGGGGAGGGGCCCCCA	1136	+4	0.34%
GCATTGAGATAGTGTGGGGAGGGGGCCCCCA	933	+4	0.28%
GCATTGAGATAGCGTGGGGAAGGGGCCCCCA	905	+4	0.27%
GCATTGAGATAGTGCGGGGAAGGGGCCCCCA	888	+4	0.27%
GCATTGAGATAGTGTGGGGGAAGGGGCCCCCA	854	+5	0.26%
GCATTGAGATAGTGTGGG-AAGGGGCCCCCA	796	+3	0.24%
GCATTGAGATAGTGTGGgGGAAGGGGCC	821	+1 g	0.25%

12h

Total | INDEL	Ins	Del	HDR
15629 | 5.91%	**3.94%**	**1.48%**	**0.48%**

Typical seqs	Reads	Type	pct.
GCATTGAGATAGTGTGG|GGA**AGG**GGCC	14706	WT	94.09%
GCATTGAGATAGTGTGGGGGAAGGGGCCCCCA	85	+5	0.54%
GCATTGAGATAGTGTGGG-AAGGGGCCCCCA	79	+3	0.51%
GCATTGAGATAGTGTG-GGAAGGGGCC	73	-1	0.47%
GCATTGAGATAGGTTT---------------AAAC	73	-7 HDR	0.47%
GCATTGAGATAGTGTGGgGGAAGGGGCC	50	+1 g	0.32%
GCATTGAGATAGTGTG--GAAGGGGCC	42	-2	0.27%
GCATTGAGATAGTGTGGGGGAGGGGCCCCCA	42	+4	0.27%
GCATTGAGATAGTGCGGGGAAGGGGCCCCCA	35	+4	0.22%
GCATTGAGATAGTGTGGGGAGGGGGCCCCCA	32	+4	0.20%

24h

Total | INDEL	Ins	Del	HDR
190952 | 9.45%	**5.29%**	**3.09%**	**1.07%**

Typical seqs	Reads	Type	pct.
GCATTGAGATAGTGTGG|GGA**AGG**GGCC	172914	WT	90.55%
GCATTGAGATAGGTTT---------------AAAC	1879	-7 HDR	0.98%
GCATTGAGATAGTGTG-GGAAGGGGCC	1175	-1	0.62%
GCATTGAGATAGTGTGGgGGAAGGGGCC	965	+1 g	0.51%
GCATTGAGATAGTGTGGG-AAGGGGCCCCCA	889	+3	0.47%
GCATTGAGATAGTGTG--GAAGGGGCC	847	-2	0.44%
GCATTGAGATAGTGTGGGGGAAGGGGCCCCCA	749	+5	0.39%
GCATTGAGATAGTGTGGGGGAGGGGCCCCCA	608	+4	0.32%
GCATTGAGATAGTGTGGGGAGGGGGCCCCCA	583	+4	0.31%
GCATTGAGATAGCGTGGGGAAGGGGCCCCCA	582	+4	0.30%

48h

Total | INDEL	Ins	Del	HDR
347231 | 13.21%	**7.17%**	**4.65%**	**1.38%**

Typical seqs	Reads	Type	pct.
GCATTGAGATAGTGTGG|GGG**GCC**CCCA	301357	WT	86.79%
GCATTGAGATAGGTTT---------------AAAC	4538	-7 HDR	1.31%
GCATTGAGATAGTGTGGgGGGGCCCCCA	3221	+1 g	0.93%
GCATTGAGATAGTGTGGG-AAGGGGCCCCCA	2965	-1	0.85%
GCATTGAGATAGTGTG-GGAAGGGGCC	3060	-5	0.88%
GCATTGAGATAGTGTG--GAAGGGGCC	2652	-6	0.76%
GCATTGAGATAGTGTGGGGGAAGGGGCC	2557	-3	0.74%
GCATTGAGATAGTGTGG--AAGGGGCCCCCA	2071	-2	0.60%
GCATTGAGATAGTGTGGGGGAGGGGCCCCCA	1286	0	0.37%
GCATTGAGATAGCGTGGGGAAGGGGCCCCCA	1084	0	0.31%

P68-P70-WT-U937-Syn48crHBG2-h-AAVKI-BC22

4h

Total	INDEL	Ins	Del	HDR
184761	4.35%	1.54%	2.06%	0.75%

Typical seqs	Reads	Type	pct.
ATTTGCATTGAGATAGT|GTGGGGAAGG	176718	WT	95.65%
ATTTGCATTGAGATAGTtGTGGGGAAGG	2311	+1 t	1.25%
ATTTGCATTGAGATAGGTTT---------------	1305	-7 HDR	0.71%
ATTTGCATTGAGATAGTGT-GGGAAGG	1018	-1	0.55%
ATTTGCATTGAGAT--TGTGGGGAAGG	249	-2	0.13%
ATTTGCATTGAGATAGTGT--GGAAGG	225	-2	0.12%
ATTTGCATTGA-----TGTGGGGAAGG	174	-5	0.09%
ATTTGCATTGAGAT-------------	168	-23	0.09%
ATTTGCATTGAGATA-----GGGAAGG	103	-5	0.06%
ATTTGCATTGAGATAG--TGGGGAAGG	86	-2	0.05%

8h

Total	INDEL	Ins	Del	HDR
38004	5.75%	2.44%	2.18%	1.13%

Typical seqs	Reads	Type	pct.
ATTTGCATTGAGATAGT|GTGGGGAAGG	35817	WT	94.25%
ATTTGCATTGAGATAGTtGTGGGGAAGG	763	+1 t	2.01%
ATTTGCATTGAGATAGGTTT---------------	403	-7 HDR	1.06%
ATTTGCATTGAGATAGTGT-GGGAAGG	176	-1	0.46%
ATTTGCATTGAGAT---GTGGGGAAGG	52	-3	0.14%
ATTTGCATTGAGATAG--TGGGGAAGG	41	-2	0.11%
ATTTGCATTGAGATA-TGTGGGGAAGG	41	-1	0.11%
ATTTGCATT------GTGTGGGGAAGG	34	-6	0.09%
ATTTGCATTGAGAT--TGTGGGGAAGG	30	-2	0.08%
ATTTGCATTGAGATA----GGGGAAGG	20	-4	0.05%

12h

Total	INDEL	Ins	Del	HDR
222164	6.39%	2.97%	2.22%	1.20%

Typical seqs	Reads	Type	pct.
ATTTGCATTGAGATAGT|GTGGGGAAGG	207961	WT	93.61%
ATTTGCATTGAGATAGTtGTGGGGAAGG	5499	+1 t	2.48%
ATTTGCATTGAGATAGGTTT---------------	2469	-7 HDR	1.11%
ATTTGCATTGAGATAGTGT-GGGAAGG	1175	-1	0.53%
ATTTGCATTGAGAT--TGTGGGGAAGG	280	-2	0.13%
ATTTGCATTGAGATAGTGT--GGAAGG	231	-2	0.10%
ATTTGCATTGAGATA-TGTGGGGAAGG	209	-1	0.09%
ATTTGCATTGAGATAG--TGGGGAAGG	217	-2	0.10%
ATTTGCATT------GTGTGGGGAAGG	185	-6	0.08%
ATTTGCATTGAGAT---GTGGGGAAGG	131	-3	0.06%

24h

Total	INDEL	Ins	Del	HDR
95429	9.74%	4.34%	3.60%	1.79%

Typical seqs	Reads	Type	pct.
ATTTGCATTGAGATAGT|GTGGGGAAGG	86138	WT	90.26%
ATTTGCATTGAGATAGTtGTGGGGAAGG	3352	+1 t	3.51%
ATTTGCATTGAGATAGGTTT---------------	1586	-7 HDR	1.66%
ATTTGCATTGAGATAGTGT-GGGAAGG	504	-1	0.53%
ATTTGCATTGAGAT--TGTGGGGAAGG	280	-2	0.29%
ATTTGCATTGAGAT---GTGGGGAAGG	282	-3	0.30%
ATTTGCATTGAGATAG--TGGGGAAGG	207	-2	0.22%
ATTTGCATT--------GTGGGGAAGG	128	-8	0.13%
ATTTGCATTGAGATA-TGTGGGGAAGG	124	-1	0.13%
ATTTGCATT------GTGTGGGGAAGG	103	-6	0.11%

48h

Total	INDEL	Ins	Del	HDR
241420	14.31%	6.47%	5.09%	2.75%

Typical seqs	Reads	Type	pct.
ATTTGCATTGAGATAGT|GTGGGGAAGG	206884	WT	85.69%
ATTTGCATTGAGATAGTtGTGGGGAAGG	12257	+1 t	5.08%
ATTTGCATTGAGATAGGTTT---------------	6119	-7 HDR	2.53%
ATTTGCATTGAGATAGTGT-GGGAAGG	1478	-1	0.61%
ATTTGCATTGAGAT--TGTGGGGAAGG	792	-2	0.33%
ATTTGCATTGAGATAG--TGGGGAAGG	655	-2	0.27%
ATTTGCATTGAGATA-TGTGGGGAAGG	535	-1	0.22%
ATTTGCATTGAGAT---GTGGGGAAGG	475	-3	0.20%
ATTTGCATTGAGAT----------AGG	380	-10	0.16%
ATTTGCATTGAGATAGTGT--GGAAGG	329	-2	0.14%

P68-P70-WT-U937-Syn49crHBG2-i-AAVKI-BC23

4h

Total | INDEL	Ins	Del	HDR
214863 | 2.78%	0.52%	1.63%	0.64%

Typical seqs	Reads	Type	pct.
TATTTGCATTGAGATAG|TGT**GGG**GAAG	208887	WT	97.22%
TATTTGCATTGAGATAGGTTT--------------	1249	-7 HDR	0.58%
TATTTGCATTGAGATAGtTGTGGGGAAG	786	+1 t	0.37%
TATTTGCATTGAGATAGTGT--GGAAG	296	-2	0.14%
TATTTGCATTGAGAT--TGTGGGGAAG	299	-2	0.14%
TATTTGCATTGAGATA--GTGGGGAAG	337	-2	0.16%
TATTTGCATTGAGA---TGTGGGGAAG	263	-3	0.12%
TATTTGCATTGAGATAG-GTGGGGAAG	112	-1	0.05%
TATTTGCATTGAGAT------------	119	-21	0.06%
TATTTGCATTGAGATAGT---GGGAAG	87	-3	0.04%

8h

Total | INDEL	Ins	Del	HDR
209957 | 3.46%	0.53%	1.98%	0.95%

Typical seqs	Reads	Type	pct.
TATTTGCATTGAGATAG|TGT**GGG**GAAG	202692	WT	96.54%
TATTTGCATTGAGATAGGTTT--------------	1860	-7 HDR	0.89%
TATTTGCATTGAGATAGtTGTGGGGAAG	686	+1 t	0.33%
TATTTGCATTGAGAT--TGTGGGGAAG	481	-2	0.23%
TATTTGCATTGAGA---TGTGGGGAAG	340	-3	0.16%
TATTTGCATTGAGATA-TGTGGGGAAG	236	-1	0.11%
TATTTGCATTGAGATAGTGT--GGAAG	219	-2	0.10%
TATTTGCATTGAGATA--GTGGGGAAG	179	-2	0.09%
TATTTGCAT--------TGTGGGGAAG	154	-8	0.07%
TATTTGCATTG----AGTGTGGGGAAG	140	-4	0.07%

12h

Total | INDEL	Ins	Del	HDR
168672 | 4.25%	0.53%	2.20%	1.51%

Typical seqs	Reads	Type	pct.
TATTTGCATTGAGATAG|TGT**GGG**GAAG	161508	WT	95.75%
TATTTGCATTGAGATAGGTTT--------------	2336	-7 HDR	1.38%
TATTTGCATTGAGATAGtTGTGGGGAAG	522	+1 t	0.31%
TATTTGCATTGAGA---TGTGGGGAAG	468	-3	0.28%
TATTTGCATTGAGAT--TGTGGGGAAG	287	-2	0.17%
TATTTGCATTGAGATA--GTGGGGAAG	275	-2	0.16%
TATTTGCAT--------TGTGGGGAAG	218	-8	0.13%
TATTTGCATTGAGATA-TGTGGGGAAG	171	-1	0.10%
TATTTGCATTGAGATAGTGT--GGAAG	131	-2	0.08%
TATTTGCATTGAGATAG---GGGGAAG	122	-3	0.07%

24h

Total | INDEL	Ins	Del	HDR
124272 | 7.18%	0.86%	3.80%	2.52%

Typical seqs	Reads	Type	pct.
TATTTGCATTGAGATAG|TGT**GGG**GAAG	115353	WT	92.82%
TATTTGCATTGAGATAGGTTT--------------	2910	-7 HDR	2.34%
TATTTGCATTGAGATAGtTGTGGGGAAG	539	+1 t	0.43%
TATTTGCATTGAGAT--TGTGGGGAAG	522	-2	0.42%
TATTTGCATTGAGA---TGTGGGGAAG	503	-3	0.40%
TATTTGCAT--------TGTGGGGAAG	331	-8	0.27%
TATTTGCATTGAGATA----GGGGAAG	263	-4	0.21%
TATTTGCATTGAGATA-TGTGGGGAAG	181	-1	0.15%
TATTTGCATTG------TGTGGGGAAG	168	-6	0.14%
TATTTGCATTGAGATA--GTGGGGAAG	191	-2	0.15%

48h

Total | INDEL	Ins	Del	HDR
121192 | 12.28%	1.19%	7.59%	3.50%

Typical seqs	Reads	Type	pct.
TATTTGCATTGAGATAG|TGT**GGG**GAAG	106311	WT	87.72%
TATTTGCATTGAGATAGGTTT--------------	3888	-7 HDR	3.21%
TATTTGCATTGAGAT--TGTGGGGAAG	1372	-2	1.13%
TATTTGCAT--------TGTGGGGAAG	1076	-8	0.89%
TATTTGCATTGAGA---TGTGGGGAAG	960	-3	0.79%
TATTTGCATTGAGATA----GGGGAAG	502	-4	0.41%
TATTTGCATTGAGATA--GTGGGGAAG	477	-2	0.39%
TATTTGCATTG------TGTGGGGAAG	343	-6	0.28%
TATTTGCATTGA-----TGTGGGGAAG	322	-5	0.27%
TATTTGCATTGAGATA-TGTGGGGAAG	261	-1	0.22%

P68-P70-WT-U937-Syn50crHBG2-j-AAVKI-BC24

4h

Total | INDEL	Ins	Del	HDR
180675 | 2.10%	**0.51%**	**0.97%**	**0.62%**

Typical seqs	Reads	Type	pct.
ATATTTGCATTGAGATA|GTG**TGG**GGAA	176888	WT	97.90%
ATATTTGCATTGAGATAGGTTT-------------	1031	-7 HDR	0.57%
ATATTTGCATTGAGATAgTTGTGGGGAA	625	+1 g	0.35%
ATATTTGCATTGAGATAaGTGTGGGGAA	122	+1 a	0.07%
ATATTTGCATTGAGATA--GTGGGGAA	82	-2	0.05%
ATATTTGCATTGAGAT--TGTGGGGAA	79	-2	0.04%
ATATTTGCATTGAGA---TGTGGGGAA	75	-3	0.04%
ATATTTGCATTGAG----TGTGGGGAA	77	-4	0.04%
ATATTTGCATTGAGATAGTGT----AA	49	-4	0.03%
ATATTTGCAT--------TGTGGGGAA	44	-8	0.02%

8h

Total | INDEL	Ins	Del	HDR
50671 | 1.03%	**0.35%**	**0.51%**	**0.18%**

Typical seqs	Reads	Type	pct.
ATATTTGCATTGAGATA|GTG**TGG**GGAA	50147	WT	98.97%
ATATTTGCATTGAGATAaGTGTGGGGAA	118	+1 a	0.23%
ATATTTGCATTGAGATAGGTTT-------------	88	-7 HDR	0.17%
ATATTTGC--------AGTGTGGGGAA	50	-8	0.10%
ATATTTGCATTGAG----TGTGGGGAA	30	-4	0.06%
ATATTTGCATTGAGATA---TGGGGAA	21	-3	0.04%
ATATTT---------TTGTGTGGGGAA	18	-9	0.04%
ATATTTGCATT------GTGTGGGGAA	19	-6	0.04%
ATATTTGCATTGAGATATTTGTGTGGGGAA	18	+3	0.04%
ATATTTGCATTGAGATATAGTGTGGGGAA	14	+2	0.03%

12h

Total | INDEL	Ins	Del	HDR
54242 | 2.98%	**0.67%**	**1.36%**	**0.95%**

Typical seqs	Reads	Type	pct.
ATATTTGCATTGAGATA|GTG**TGG**GGAA	52623	WT	97.02%
ATATTTGCATTGAGATAGGTTT-------------	482	-7 HDR	0.89%
ATATTTGCATTGAGATAgTTGTGGGGAA	259	+1 g	0.48%
ATATTTGCATTGAGAT--TGTGGGGAA	56	-2	0.10%
ATATTTGCATTGAGATA--GTGGGGAA	51	-2	0.09%
ATATTTGCATTGAGATAaGTGTGGGGAA	36	+1 a	0.07%
ATATTTGCATTGAGA---TGTGGGGAA	33	-3	0.06%
ATATTTGCATTGAGATAGT-----GAA	23	-5	0.04%
ATATTTGCATTGAGATAGTGT----AA	24	-4	0.04%
ATATTTGCAT--------TGTGGGGAA	23	-8	0.04%

24h

Total | INDEL	Ins	Del	HDR
133111 | 3.54%	**1.09%**	**1.49%**	**0.96%**

Typical seqs	Reads	Type	pct.
ATATTTGCATTGAGATA|GTG**TGG**GGAA	128402	WT	96.46%
ATATTTGCATTGAGATAGGTTT-------------	1179	-7 HDR	0.89%
ATATTTGCATTGAGATAaGTGTGGGGAA	531	+1 a	0.40%
ATATTTGCATTGAGATAgTTGTGGGGAA	480	+1 g	0.36%
ATATTTGCATTGAG----TGTGGGGAA	130	-4	0.10%
ATATTTGCAT--------TGTGGGGAA	85	-8	0.06%
ATATTTGCATTGAGAT--TGTGGGGAA	72	-2	0.05%
ATATTTGCATTGAGA---TGTGGGGAA	67	-3	0.05%
ATATTTGCATTGAGA--GTGTGGGGAA	68	-2	0.05%
ATATTTGCATTGAGATA--GTGGGGAA	58	-2	0.04%

48h

Total | INDEL	Ins	Del	HDR
20881 | 3.81%	**1.46%**	**1.38%**	**0.97%**

Typical seqs	Reads	Type	pct.
ATATTTGCATTGAGATA|GTG**TGG**GGAA	20086	WT	96.19%
ATATTTGCATTGAGATAaGTGTGGGGAA	243	+1 a	1.16%
ATATTTGCATTGAGATAGGTTT-------------	189	-7 HDR	0.91%
ATATTTGCATTGAGA--GTGTGGGGAA	42	-2	0.20%
ATATTTGCATTGAG----TGTGGGGAA	43	-4	0.21%
ATATTTGCATTGAGAT-GTGTGGGGAA	13	-1	0.06%
ATATTTGCATTGAGA---TGTGGGGAA	13	-3	0.06%
ATATTTGCATT------GTGTGGGGAA	10	-6	0.05%
ATATTTGCAT--------TGTGGGGAA	10	-8	0.05%
ATATTTGCATTGA-----------GAA	9	-11	0.04%

P152-YW-U937-RNP-Syn79crBCL11A2c-KO

4h

Total	INDEL	Ins	Del	HDR
3679	3.15%	1.96%	1.20%	0.00%

Typical seqs	Reads	Type	pct.
TGTTGTGATTCCGAGCTCCG**AGG**CGAG	3563	WT	96.85%
TGTTGTGATTCCGAGCTtCCGAGGCGAG	77	+1 t	2.09%
TGTTGTGATT-------CCGAGGCGAG	23	-7	0.63%
TGTTGTGATTCCGA--------GCGAG	6	-8	0.16%
TGTTGTGATTCCGAG-TCCGAGGCGAG	6	-1	0.16%
TGTTGTGATTCCGAGCT--GAGGCGAG	5	-2	0.14%
TGTTGTGATTCCGA--TCCGAGGCGAG	4	-2	0.11%

8h

Total	INDEL	Ins	Del	HDR
6985	11.17%	5.84%	5.33%	0.00%

Typical seqs	Reads	Type	pct.
TGTTGTGATTCCGAGCTCCG**AGG**CGAG	6205	WT	88.83%
TGTTGTGATTCCGAGCTtCCGAGGCGAG	391	+1 t	5.60%
TGTTGTGATT-------CCGAGGCGAG	193	-7	2.76%
TGTTGTGATTCCGAG-TCCGAGGCGAG	32	-1	0.46%
TGTTGTGATTCCGA--------GCGAG	32	-8	0.46%
TGTTGTGATTCCGA--TCCGAGGCGAG	22	-2	0.31%
TGTTGTGATTCCG---TCCGAGGCGAG	12	-3	0.17%
TGTTGTGATTCCGAGCT-CGAGGCGAG	11	-1	0.16%
TGTTGTGATTC------------CGAG	9	-12	0.13%
TGTTGTGATT-------CCGAGGCGAG	193	-8	2.76%

12h

Total	INDEL	Ins	Del	HDR
21838	23.97%	9.52%	14.45%	0.00%

Typical seqs	Reads	Type	pct.
TGTTGTGATTCCGAGCTCCG**AGG**CGAG	16604	WT	76.03%
TGTTGTGATTCCGAGCTtCCGAGGCGAG	1909	+1 t	8.74%
TGTTGTGATT-------CCGAGGCGAG	1672	-7	7.66%
TGTTGTGATTCCGA--TCCGAGGCGAG	210	-2	0.96%
TGTTGTGATTCCGA--------GCGAG	210	-8	0.96%
TGTTGTGATTCCGAG-TCCGAGGCGAG	134	-1	0.61%
TGTTGTGATTC------------CGAG	87	-12	0.40%
TGTTGTGATTCCG---TCCGAGGCGAG	77	-3	0.35%
TGTTGTGATTCCGAG--CCGAGGCGAG	63	-2	0.29%
TGTTGTGATTCCGAGCT--GAGGCGAG	42	-2	0.19%

24h

Total	INDEL	Ins	Del	HDR
25890	42.94%	13.31%	29.63%	0.00%

Typical seqs	Reads	Type	pct.
TGTTGTGATTCCGAGCTCCG**AGG**CGAG	14774	WT	57.06%
TGTTGTGATT-------CCGAGGCGAG	4447	-7	17.18%
TGTTGTGATTCCGAGCTtCCGAGGCGAG	3164	+1 t	12.22%
TGTTGTGATTCCGA--------GCGAG	597	-8	2.31%
TGTTGTGATTCCGAG-TCCGAGGCGAG	292	-1	1.13%
TGTTGTGATTCCGA--TCCGAGGCGAG	281	-2	1.09%
TGTTGTGATTC------------CGAG	198	-12	0.76%
TGTTGTGATT-------CCGAGGCGAG	4447	-8	17.18%
TGTTGTGATTCCGAGCT--GAGGCGAG	105	-2	0.41%
TGTTGTGATTCCG---TCCGAGGCGAG	85	-3	0.33%

48h

Total	INDEL	Ins	Del	HDR
15698	56.28%	15.61%	40.67%	0.00%

Typical seqs	Reads	Type	pct.
TGTTGTGATTCCGAGCTCCG**AGG**CGAG	6863	WT	43.72%
TGTTGTGATT-------CCGAGGCGAG	3872	-7	24.67%
TGTTGTGATTCCGAGCTtCCGAGGCGAG	2275	+1 t	14.49%
TGTTGTGATTCCGA--------GCGAG	500	-8	3.19%
TGTTGTGATTCCGA--TCCGAGGCGAG	233	-2	1.48%
TGTTGTGATTCCGAG-TCCGAGGCGAG	182	-1	1.16%
TGTTGTGATTC------------CGAG	134	-12	0.85%
TGTTGTGATT-------CCGAGGCGAG	3872	-8	24.67%
TGTTGTGATTCCG---TCCGAGGCGAG	97	-3	0.62%
TGTTGTGATTCCGAGCT--GAGGCGAG	76	-2	0.48%

P152-YW-U937-RNP-Syn83crBCL11A4b-KO

4h

Total | INDEL	Ins	Del	HDR
5682 | 2.53%	0.69%	1.85%	0.00%

Typical seqs	Reads	Type	pct.
ACTTCATGCGGAGGCCC|CGT**GGG**AGGA	5538	WT	97.47%
ACTTCATGCGGAGGCC-CGTGGGAGGA	46	-1	0.81%
ACTTCATGCGGAGGCCCcCGTGGGAGGA	19	+1 c	0.33%
ACTTCATGCGGAGGCCCtCGTGGGAGGA	13	+1 t	0.23%
ACTTCATGC------------GGAGGA	11	-12	0.19%
ACTT-------------CGTGGGAGGA	10	-13	0.18%
ACTTCATGCGGAGG---CGTGGGAGGA	8	-3	0.14%
ACTTCATGCGGAGGCCCCGT-GGAGGA	4	-1	0.07%
ACTTCATGCGGAGGC------------	4	-12	0.07%
ACTTCATGCGGAGGCCTACTTCGTGGGAGGA	4	+4	0.07%

8h

Total | INDEL	Ins	Del	HDR
9374 | 10.26%	1.81%	8.45%	0.00%

Typical seqs	Reads	Type	pct.
ACTTCATGCGGAGGCCC|CGT**GGG**AGGA	8412	WT	89.74%
ACTTCATGCGGAGGCC-CGTGGGAGGA	223	-1	2.38%
ACTTCATGC------------GGAGGA	144	-12	1.54%
ACTTCATGCGGAGGCCCcCGTGGGAGGA	52	+1 c	0.55%
ACTTCATGCGGAGGC--CGTGGGAGGA	36	-2	0.38%
ACTT-------------CGTGGGAGGA	31	-13	0.33%
ACTTCATGC---------------GGA	25	-15	0.27%
ACTTCATGCGGAGGCCC--TGGGAGGA	25	-2	0.27%
ACTTCATG---------CGTGGGAGGA	24	-9	0.26%
ACTTCA-------------TGGGAGGA	24	-13	0.26%

12h

Total | INDEL	Ins	Del	HDR
47458 | 25.64%	4.37%	21.27%	0.00%

Typical seqs	Reads	Type	pct.
ACTTCATGCGGAGGCCC|CGT**GGG**AGGA	35290	WT	74.36%
ACTTCATGC------------GGAGGA	2108	-12	4.44%
ACTTCATGCGGAGGCC-CGTGGGAGGA	1741	-1	3.67%
ACTTCATGCGGAGGCCCcCGTGGGAGGA	628	+1 c	1.32%
ACTT-------------CGTGGGAGGA	454	-13	0.96%
ACTTCATG---------CGTGGGAGGA	383	-9	0.81%
ACTTCA-------------TGGGAGGA	386	-13	0.81%
ACTTCATGCGGAGGC--CGTGGGAGGA	345	-2	0.73%
ACTTCATGC---------------GGA	330	-15	0.70%
ACTTCATGCGGAGG---CGTGGGAGGA	264	-3	0.56%

24h

Total | INDEL	Ins	Del	HDR
36837 | 42.27%	6.38%	35.89%	0.00%

Typical seqs	Reads	Type	pct.
ACTTCATGCGGAGGCCC|CGT**GGG**AGGA	21267	WT	57.73%
ACTTCATGC------------GGAGGA	2809	-12	7.63%
ACTTCATGCGGAGGCC-CGTGGGAGGA	1843	-1	5.00%
ACTT-------------CGTGGGAGGA	702	-13	1.91%
ACTTCATG---------CGTGGGAGGA	650	-9	1.76%
ACTTCA-------------TGGGAGGA	625	-13	1.70%
ACTTCATGCGGAGGCCCcCGTGGGAGGA	586	+1 c	1.59%
ACTTCATGC---------------GGA	532	-15	1.44%
ACTTCATGCGGAGGC--CGTGGGAGGA	409	-2	1.11%
ACTTCAT-----------GTGGGAGGA	352	-11	0.96%

48h

Total | INDEL	Ins	Del	HDR
26341 | 49.64%	7.66%	41.98%	0.00%

Typical seqs	Reads	Type	pct.
ACTTCATGCGGAGGCCC|CGT**GGG**AGGA	13266	WT	50.36%
ACTTCATGC------------GGAGGA	2423	-12	9.20%
ACTTCATGCGGAGGCC-CGTGGGAGGA	1568	-1	5.95%
ACTT-------------CGTGGGAGGA	679	-13	2.58%
ACTTCA-------------TGGGAGGA	562	-13	2.13%
ACTTCATG---------CGTGGGAGGA	494	-9	1.88%
ACTTCATGCGGAGGCCCcCGTGGGAGGA	449	+1 c	1.70%
ACTTCATGC---------------GGA	375	-15	1.42%
ACTTCATGCGGAGG---CGTGGGAGGA	322	-3	1.22%
ACTTCATGCGGAGGC--CGTGGGAGGA	280	-2	1.06%

P148-YW-U937-RNP-Syn83crBCL11A4b-AAV6KI

4h — Total 12089 | INDEL 2.75% — Ins 0.66% — Del 1.64% — HDR 0.45%

Typical seqs	Reads	Type	pct.
ACTTCATGCGGAGGCCC|CGTGGGAGGA	11757	WT	97.25%
ACTTCATGCGGAGGCC-CGTGGGAGGA	85	-1	0.70%
ACTTCATGCGGAGGCCCGTTTAAACCGTGG GAGGA	54	+8 HDR	0.45%
ACTTCATGCGGAGGCCCcCGTGGGAGGA	48	+1 c	0.40%
ACTTCATGC------------GGAGGA	26	-12	0.22%
ACTTCATGCGGAGGCCCCGT-GGAGGA	20	-1	0.17%
ACTTCATGCGGAGG---CGTGGGAGGA	14	-3	0.12%
ACTTCATGCGGAGGC--CGTGGGAGGA	11	-2	0.09%
ACTTCATGCGGAGGCCCtCGTGGGAGGA	9	+1 t	0.07%
ACTTCATGCGGAGGCCCgCGTGGGAGGA	8	+1 g	0.07%

8h — Total 10003 | INDEL 13.80% — Ins 2.46% — Del 6.48% — HDR 4.86%

Typical seqs	Reads	Type	pct.
ACTTCATGCGGAGGCCC|CGTGGGAGGA	8623	WT	86.20%
ACTTCATGCGGAGGCCCGTTTAAACCGTGG GAGGA	471	+8 HDR	4.71%
ACTTCATGCGGAGGCC-CGTGGGAGGA	195	-1	1.95%
ACTTCATGC------------GGAGGA	84	-12	0.84%
ACTTCATGCGGAGGCCCcCGTGGGAGGA	71	+1 c	0.71%
ACTTCATGCGGAGGCCCtCGTGGGAGGA	44	+1 t	0.44%
ACTTCATGCGGAGGC--CGTGGGAGGA	37	-2	0.37%
ACTTCATGCGGAGGCCCaCGTGGGAGGA	30	+1 a	0.30%
ACTT-------------CGTGGGAGGA	30	-13	0.30%
ACTTCATGCGGAGGCCCgCGTGGGAGGA	28	+1 g	0.28%

12h — Total 22660 | INDEL 31.57% — Ins 5.11% — Del 16.08% — HDR 10.38%

Typical seqs	Reads	Type	pct.
ACTTCATGCGGAGGCCC|CGTGGGAGGA	15507	WT	68.43%
ACTTCATGCGGAGGCCCGTTTAAACCGTGG GAGGA	2212	+8 HDR	9.76%
ACTTCATGCGGAGGCC-CGTGGGAGGA	831	-1	3.67%
ACTTCATGC------------GGAGGA	486	-12	2.14%
ACTTCATGCGGAGGCCCcCGTGGGAGGA	298	+1 c	1.32%
ACTTCATGCGGAGGC--CGTGGGAGGA	209	-2	0.92%
ACTTCATGCGGAGGCCCtCGTGGGAGGA	156	+1 t	0.69%
ACTTCATG---------CGTGGGAGGA	152	-9	0.67%
ACTT-------------CGTGGGAGGA	148	-13	0.65%
ACTTCA-------------TGGGAGGA	126	-13	0.56%

24h — Total 9282 | INDEL 45.54% — Ins 7.10% — Del 24.63% — HDR 13.81%

Typical seqs	Reads	Type	pct.
ACTTCATGCGGAGGCCC|CGTGGGAGGA	5055	WT	54.46%
ACTTCATGCGGAGGCCCGTTTAAACCGTGG GAGGA	1220	+8 HDR	13.14%
ACTTCATGCGGAGGCC-CGTGGGAGGA	488	-1	5.26%
ACTTCATGC------------GGAGGA	386	-12	4.16%
ACTTCATGCGGAGGCCCcCGTGGGAGGA	206	+1 c	2.22%
ACTTCATG---------CGTGGGAGGA	141	-9	1.52%
ACTT-------------CGTGGGAGGA	114	-13	1.23%
ACTTCATGCGGAGGC--CGTGGGAGGA	92	-2	0.99%
ACTTCATGCGGAGGCCCtCGTGGGAGGA	91	+1 t	0.98%
ACTTCA-------------TGGGAGGA	85	-13	0.92%

48h — Total 4426 | INDEL 52.26% — Ins 7.03% — Del 31.00% — HDR 14.23%

Typical seqs	Reads	Type	pct.
ACTTCATGCGGAGGCCC|CGTGGGAGGA	2113	WT	47.74%
ACTTCATGCGGAGGCCCGTTTAAACCGTGG GAGGA	602	+8 HDR	13.60%
ACTTCATGC------------GGAGGA	275	-12	6.21%
ACTTCATGCGGAGGCC-CGTGGGAGGA	235	-1	5.31%
ACTTCATGCGGAGGCCCcCGTGGGAGGA	83	+1 c	1.88%
ACTTCATG---------CGTGGGAGGA	72	-9	1.63%
ACTTCA-------------TGGGAGGA	71	-13	1.60%
ACTTCATGCGGAGGC--CGTGGGAGGA	66	-2	1.49%
ACTT-------------CGTGGGAGGA	66	-13	1.49%
ACTTCATGCGGAGG---CGTGGGAGGA	52	-3	1.17%

P152-YW-U937-RNP-Syn92crBCL11A7b-KO

Total	INDEL	4h	Ins	Del	HDR
4393	1.71%		1.57%	0.14%	0.00%

Typical seqs	Reads	Type	pct.
GCTTCGTGACGTTGGAT|GGA**GGG**TTTT	4318	WT	98.29%
GCTTCGTGACGTTGGATtGGAGGGTTTT	69	+1 t	1.57%
GCTTCGTGACGTT----GGAGGGTTTT	6	-4	0.14%

Total	INDEL	8h	Ins	Del	HDR
4279	4.65%		3.97%	0.68%	0.00%

Typical seqs	Reads	Type	pct.
GCTTCGTGACGTTGGAT|GGA**GGG**TTTT	4080	WT	95.35%
GCTTCGTGACGTTGGATtGGAGGGTTTT	167	+1 t	3.90%
GCTTCGTGACGTT----GGAGGGTTTT	13	-4	0.30%
GCTTCGTGACGTTG--TGGAGGGTTTT	7	-2	0.16%
GCTTCGTGACGTT--------GGTTTT	3	-8	0.07%
GCTTCGTGACGTTGGATTTGGAGGGTTTT	3	+2	0.07%
GCTTCGTGACGTT---TGGAGGGTTTT	3	-3	0.07%
GCTTCGTGACGTTGGA-GGAGGGTTTT	3	-1	0.07%

Total	INDEL	12h	Ins	Del	HDR
40861	9.52%		7.11%	2.41%	0.00%

Typical seqs	Reads	Type	pct.
GCTTCGTGACGTTGGAT|GGA**GGG**TTTT	36972	WT	90.48%
GCTTCGTGACGTTGGATtGGAGGGTTTT	2572	+1 t	6.29%
GCTTCGTGACGTT----GGAGGGTTTT	314	-4	0.77%
GCTTCGTGACGTTG--TGGAGGGTTTT	93	-2	0.23%
GCTTCGTGACGTT-------GGGTTTT	66	-7	0.16%
GCTTCGTGAC------------GTTTT	43	-12	0.11%
GCTTCGTGACGTTGGAT-GAGGGTTTT	35	-1	0.09%
GCTTCGTGACGTT--------GGTTTT	31	-8	0.08%
GCTTCGTGACGTTGGA-GGAGGGTTTT	29	-1	0.07%
GCTTCGTGACGTTGGATTTGGAGGGTTTT	24	+2	0.06%

Total	INDEL	24h	Ins	Del	HDR
49092	15.00%		10.56%	4.44%	0.00%

Typical seqs	Reads	Type	pct.
GCTTCGTGACGTTGGAT|GGA**GGG**TTTT	41727	WT	85.00%
GCTTCGTGACGTTGGATtGGAGGGTTTT	4639	+1 t	9.45%
GCTTCGTGACGTT----GGAGGGTTTT	948	-4	1.93%
GCTTCGTGACGTT-------GGGTTTT	160	-7	0.33%
GCTTCGTGACGTTG--TGGAGGGTTTT	101	-2	0.21%
GCTTCGTGAC------------GTTTT	83	-12	0.17%
GCTTCGTGACGTT--------GGTTTT	78	-8	0.16%
GCTTCGTGACGTTGGA-GGAGGGTTTT	56	-1	0.11%
GCTTCGTGACGTTGGA---AGGGTTTT	46	-3	0.09%
GCTTCGTGACGTTGG-TGGAGGGTTTT	40	-1	0.08%

Total	INDEL	48h	Ins	Del	HDR
40124	17.77%		12.35%	5.42%	0.00%

Typical seqs	Reads	Type	pct.
GCTTCGTGACGTTGGAT|GGA**GGG**TTTT	32993	WT	82.23%
GCTTCGTGACGTTGGATtGGAGGGTTTT	4415	+1 t	11.00%
GCTTCGTGACGTT----GGAGGGTTTT	923	-4	2.30%
GCTTCGTGACGTT-------GGGTTTT	192	-7	0.48%
GCTTCGTGACGTTG--TGGAGGGTTTT	104	-2	0.26%
GCTTCGTGAC------------GTTTT	92	-12	0.23%
GCTTCGTGACGTT--------GGTTTT	85	-8	0.21%
GCTTCGTGACGTTGGA-GGAGGGTTTT	55	-1	0.14%
GCTTCGTGACGTTGGAT-GAGGGTTTT	47	-1	0.12%
GCTTCGTGACGTTGGA---AGGGTTTT	38	-3	0.09%

P195-YW-U937-Syn99crBCL11A9a-RNPko

P195-YW-U937-Syn99crBCL11A9a-RNPko-4h-BC1 4h	Ins	Del	HDR
5709 | 1.19%	1.10%	0.09%	0.00%
**Typical seqs**	**Reads**	**Type**	**pct.**
GCGCAAACTCCCGTTCT|CCG**AGG**AGTG	5641	WT	98.81%
GCGCAAACTCCCGTTCTtCCGAGGAGTG	63	+1 t	1.10%
GCGCAAACTC-------CCGAGGAGTG	5	-7	0.09%

P195-YW-U937-Syn99crBCL11A9a-RNPko-8h-BC2 8h	Ins	Del	HDR
8607 | 3.23%	2.63%	0.60%	0.00%
**Typical seqs**	**Reads**	**Type**	**pct.**
GCGCAAACTCCCGTTCT|CCG**AGG**AGTG	8329	WT	96.77%
GCGCAAACTCCCGTTCTtCCGAGGAGTG	208	+1 t	2.42%
GCGCAAACTC-------CCGAGGAGTG	32	-7	0.37%
GCGCAAACTCCCGTTCTTTCCGAGGAGTG	15	+2	0.17%
GCGCAAACTCCCGT--TCCGAGGAGTG	11	-2	0.13%
GCGCAAA--------CTCCGAGGAGTG	3	-8	0.03%
GCGCAAACTCCCGTTC-CCGAGGAGTG	3	-1	0.03%
GCGCAAACTCCCGTTCT--GAGGAGTG	3	-2	0.03%
GCGCAAACTCCCGTTCTATCCGAGGAGTG	3	+2	0.03%

P195-YW-U937-Syn99crBCL11A9a-RNPko-12h-BC3 12h	Ins	Del	HDR
30752 | 6.27%	4.48%	1.78%	0.00%
**Typical seqs**	**Reads**	**Type**	**pct.**
GCGCAAACTCCCGTTCT|CCG**AGG**AGTG	28825	WT	93.73%
GCGCAAACTCCCGTTCTtCCGAGGAGTG	1143	+1 t	3.72%
GCGCAAACTC-------CCGAGGAGTG	283	-7	0.92%
GCGCAAACTCCCGTTCTTTCCGAGGAGTG	83	+2	0.27%
GCGCAAACTCCCGT--TCCGAGGAGTG	32	-2	0.10%
GCGCAAA--------CTCCGAGGAGTG	33	-8	0.11%
GCGCAAACTCCCGTT-TCCGAGGAGTG	23	-1	0.07%
GCGCAAACTCCCGTTCTcCCGAGGAGTG	15	+1 c	0.05%
GCGCAAACTCCCGTTCT---AGGAGTG	14	-3	0.05%
GCGCAAACTCCCGTTC-CCGAGGAGTG	13	-1	0.04%

P195-YW-U937-Syn99crBCL11A9a-RNPko-24h-BC4 24h	Ins	Del	HDR
27682 | 8.68%	5.65%	3.03%	0.00%
**Typical seqs**	**Reads**	**Type**	**pct.**
GCGCAAACTCCCGTTCT|CCG**AGG**AGTG	25279	WT	91.32%
GCGCAAACTCCCGTTCTtCCGAGGAGTG	1346	+1 t	4.86%
GCGCAAACTC-------CCGAGGAGTG	383	-7	1.38%
GCGCAAACTCCCGT--TCCGAGGAGTG	65	-2	0.23%
GCGCAAACTCCCGTTCTTTCCGAGGAGTG	61	+2	0.22%
GCGCAAA--------CTCCGAGGAGTG	53	-8	0.19%
GCGCAAACTCCCGTTCT-CGAGGAGTG	21	-1	0.08%
GCGCAAAC------TCTCCGAGGAGTG	16	-6	0.06%
GCGCAAACTCCCGTTC-CCGAGGAGTG	15	-1	0.05%
GCGCAAACTCCCG---TCCGAGGAGTG	14	-3	0.05%

P195-YW-U937-Syn99crBCL11A9a-RNPko-48h-BC5 48h	Ins	Del	HDR
23495 | 10.26%	6.56%	3.70%	0.00%
**Typical seqs**	**Reads**	**Type**	**pct.**
GCGCAAACTCCCGTTCT|CCG**AGG**AGTG	21084	WT	89.74%
GCGCAAACTCCCGTTCTtCCGAGGAGTG	1293	+1 t	5.50%
GCGCAAACTC-------CCGAGGAGTG	451	-7	1.92%
GCGCAAACTCCCGTTCTTTCCGAGGAGTG	84	+2	0.36%
GCGCAAACTCCCGT--TCCGAGGAGTG	49	-2	0.21%
GCGCAAA--------CTCCGAGGAGTG	41	-8	0.17%
GCGCAAACTCCCGTTCT-CGAGGAGTG	36	-1	0.15%
GCGCAAA--------------------	31	-20	0.13%
GCGCAAACTCCCGTT-TCCGAGGAGTG	27	-1	0.11%
GCGCAAACTCCCGTTCT--GAGGAGTG	16	-2	0.07%

P195-YW-U937-Syn100crBCL11A9b-RNPko

P195-YW-U937-Syn100crBCL11A9b-RNPko-4h-BC1 4h	Ins	Del	HDR
1824 | 0.77%	**0.77%**	**0.00%**	**0.00%**
**Typical seqs**	**Reads**	**Type**	**pct.**
GTCGGACTTGACCGTCA|TGG**GGG**ACGA	1810	WT	99.23%
GTCGGACTTGACCGTCAaTGGGGGACGA	10	+1 a	0.55%
GTCGGACTTGACCGTCAtTGGGGGACGA	4	+1 t	0.22%

P195-YW-U937-Syn100crBCL11A9b-RNPko-8h-BC2 8h	Ins	Del	HDR
5232 | 2.31%	**1.28%**	**1.03%**	**0.00%**
**Typical seqs**	**Reads**	**Type**	**pct.**
GTCGGACTTGACCGTCA|TGG**GGG**ACGA	5111	WT	97.69%
GTCGGACTTGACCGTCAaTGGGGGACGA	52	+1 a	0.99%
GTCGGACTTGACCGTCAT-GGGGACGA	15	-1	0.29%
GTCGGACTTGACCGTCAtTGGGGGACGA	12	+1 t	0.23%
GTCGGACTTGACC---------GACGA	8	-9	0.15%
GTCGGACTTGACCGTCA-GGGGGACGA	8	-1	0.15%
GTCGGACTTGACCGTC-TGGGGGACGA	5	-1	0.10%
GTC------------------------	3	-29	0.06%
GTCGGACTT-------------GACGA	3	-13	0.06%
G----------------TGGGGGACGA	3	-16	0.06%

P195-YW-U937-Syn100crBCL11A9b-RNPko-12h-BC3 12h	Ins	Del	HDR
10570 | 5.85%	**2.74%**	**3.10%**	**0.00%**
**Typical seqs**	**Reads**	**Type**	**pct.**
GTCGGACTTGACCGTCA|TGG**GGG**ACGA	9952	WT	94.15%
GTCGGACTTGACCGTCAaTGGGGGACGA	157	+1 a	1.49%
GTCGGACTTGACCGTCAtTGGGGGACGA	77	+1 t	0.73%
GTCGGACTTGACCGTCA-GGGGGACGA	44	-1	0.42%
GTCGGACTT-------------GACGA	43	-13	0.41%
GTCGGACTTGACCGTCAT-GGGGACGA	43	-1	0.41%
GTCGGACTTGACC---------GACGA	30	-9	0.28%
GTC------------------GGACGA	18	-18	0.17%
GTCGGACTTGAC------------CGA	11	-12	0.10%
GTCGGACTTGACCGTC------GACGA	10	-6	0.09%

P195-YW-U937-Syn100crBCL11A9b-RNPko-24h-BC4 24h	Ins	Del	HDR
15978 | 8.02%	**3.04%**	**4.98%**	**0.00%**
**Typical seqs**	**Reads**	**Type**	**pct.**
GTCGGACTTGACCGTCA|TGG**GGG**ACGA	14696	WT	91.98%
GTCGGACTTGACCGTCAaTGGGGGACGA	267	+1 a	1.67%
GTCGGACTT-------------GACGA	142	-13	0.89%
GTCGGACTTGACCGTCAtTGGGGGACGA	117	+1 t	0.73%
GTCGGACTTGACC---------GACGA	103	-9	0.64%
GTC------------------GGACGA	63	-18	0.39%
GTCGGACTTGAC------------CGA	62	-12	0.39%
GTCGGACTTGACCGTCAT-GGGGACGA	64	-1	0.40%
GTCGGACTT----------------GA	28	-16	0.18%
GTCGGACTTGACCGTCAcTGGGGGACGA	22	+1 c	0.14%

P195-YW-U937-Syn100crBCL11A9b-RNPko-48h-BC5 48h	Ins	Del	HDR
17597 | 9.63%	**3.73%**	**5.90%**	**0.00%**
**Typical seqs**	**Reads**	**Type**	**pct.**
GTCGGACTTGACCGTCA|TGG**GGG**ACGA	15902	WT	90.37%
GTCGGACTTGACCGTCAaTGGGGGACGA	355	+1 a	2.02%
GTCGGACTT-------------GACGA	136	-13	0.77%
GTCGGACTTGACCGTCAtTGGGGGACGA	137	+1 t	0.78%
GTCGGACTTGACC---------GACGA	118	-9	0.67%
GTCGGACTTGACCGTCAT-GGGGACGA	73	-1	0.41%
GTCGGACTTGAC------------CGA	71	-12	0.40%
GTCGGACTTGACCGTCA-GGGGGACGA	72	-1	0.41%
GTC------------------GGACGA	59	-18	0.34%
GTCGGACTTGACCGTC-TGGGGGACGA	28	-1	0.16%

P195-YW-U937-Syn105crBCL11A9g-RNPko

P195-YW-U937-Syn105crBCL11A9g-RNPko-4h-BC1 4h	Ins	Del	HDR
2597 | 7.93%	7.55%	0.39%	0.00%
Typical seqs	Reads	Type	pct.
CAACTTACAAATACCCT|GCGGGGCATA	2391	WT	92.07%
CAACTTACAAATACCCTtGCGGGGCATA	196	+1 t	7.55%
CAACTTACAAATACCCTGC-GGGCATA	7	-1	0.27%
CAACTTACAAATA-CCTGCGGGGCATA	3	-1	0.12%

P195-YW-U937-Syn105crBCL11A9g-RNPko-8h-BC2 8h	Ins	Del	HDR
7429 | 18.31%	17.14%	1.17%	0.00%
Typical seqs	Reads	Type	pct.
CAACTTACAAATACCCT|GCGGGGCATA	6069	WT	81.69%
CAACTTACAAATACCCTtGCGGGGCATA	1151	+1 t	15.49%
CAACTTACAAATACCCTTTGCGGGGCATA	22	+2	0.30%
CAACTTACAAATA-CCTGCGGGGCATA	17	-1	0.23%
CAACTTACAAATA--CTGCGGGGCATA	16	-2	0.22%
CAACTTACAAAT----TGCGGGGCATA	10	-4	0.13%
CAACTTACAAATACCCCTGCGGGGCATA	9	+1 Ins	0.12%
CAACTTACAAATA---TGCGGGGCATA	8	-3	0.11%
CAACTTACAAATACCCTcGCGGGGCATA	7	+1 c	0.09%
CAACTTACGAATACCCTTGCGGGGCATA	7	+1 Ins	0.09%

P195-YW-U937-Syn105crBCL11A9g-RNPko-12h-BC3 12h	Ins	Del	HDR
15902 | 27.69%	25.18%	2.51%	0.00%
Typical seqs	Reads	Type	pct.
CAACTTACAAATACCCT|GCGGGGCATA	11499	WT	72.31%
CAACTTACAAATACCCTtGCGGGGCATA	3653	+1 t	22.97%
CAACTTACAAATA-CCTGCGGGGCATA	63	-1	0.40%
CAACTTACAAATA--CTGCGGGGCATA	51	-2	0.32%
CAACTTACAAATA---TGCGGGGCATA	46	-3	0.29%
CAACTTACAAATACCCTTTGCGGGGCATA	39	+2	0.25%
CAACTTACAAATACCCCTGCGGGGCATA	24	+1 Ins	0.15%
CAACTTACAAATACCCTATGCGGGGCATA	19	+2	0.12%
CAACCTACAAATACCCTTGCGGGGCATA	19	+1 Ins	0.12%
CAACTTA----------------CATA	17	-16	0.11%

P195-YW-U937-Syn105crBCL11A9g-RNPko-24h-BC4 24h	Ins	Del	HDR
22778 | 37.60%	33.48%	4.12%	0.00%
Typical seqs	Reads	Type	pct.
CAACTTACAAATACCCT|GCGGGGCATA	14214	WT	62.40%
CAACTTACAAATACCCTtGCGGGGCATA	6902	+1 t	30.30%
CAACTTACAAATA--CTGCGGGGCATA	108	-2	0.47%
CAACTTACAAATA-CCTGCGGGGCATA	105	-1	0.46%
CAACTTACAAATA---TGCGGGGCATA	90	-3	0.40%
CAACTTACAAATACCCTTTGCGGGGCATA	81	+2	0.36%
CAACTTACAA--------------ATA	57	-14	0.25%
CAACTTACAAATACCCCTGCGGGGCATA	47	+1 Ins	0.21%
CAACTTA----------------CATA	41	-16	0.18%
CAACTTACAAATACCCTCTGCGGGGCATA	39	+2	0.17%

P195-YW-U937-Syn105crBCL11A9g-RNPko-48h-BC5 48h	Ins	Del	HDR
24441 | 45.91%	40.67%	5.25%	0.00%
Typical seqs	Reads	Type	pct.
CAACTTACAAATACCCT|GCGGGGCATA	13220	WT	54.09%
CAACTTACAAATACCCTtGCGGGGCATA	8956	+1 t	36.64%
CAACTTACAAATA-CCTGCGGGGCATA	158	-1	0.65%
CAACTTACAAATACCCTTTGCGGGGCATA	113	+2	0.46%
CAACTTACAAATA--CTGCGGGGCATA	108	-2	0.44%
CAACTTACAAATA---TGCGGGGCATA	86	-3	0.35%
CAACTTACAAATACCCCTGCGGGGCATA	59	+1 Ins	0.24%
CAACTTACAA--------------ATA	57	-14	0.23%
CAACTTACAAATA-----CGGGGCATA	51	-5	0.21%
CAACTTA----------------CATA	43	-16	0.18%

P193-YW-U937-Syn26crCD326ac-RNPaav

P189-YW-U937-Syn26crCD326ac-RNPaav-4h-BC7 **4h**	Ins	Del	HDR
117215 | 2.71%	**0.96%**	**0.74%**	**1.02%**
**Typical seqs**	**Reads**	**Type**	**pct.**
CCCTCCCGCGCCCCTCT|TCT**CGG**CGCG	114033	WT	97.29%
CCCTCCCGCACGCGTAGTTTAAACCTCGGCGCG	1131	+6 HDR	0.96%
CCCTCCCGCGCCCCTCTtTCTCGGCGCG	1006	+1 t	0.86%
CCCTCCCGCGCCCCTC-TCTCGGCGCG	300	-1	0.26%
CCCTCCCGCGCCCC---TCTCGGCGCG	105	-3	0.09%
CCCTCCCGCGCCCCT-TTCTCGGCGCG	44	-1	0.04%
CCCTCCCGCGCCC----TCTCGGCGCG	35	-4	0.03%
CCCTCCCGCGCCCCTCT----------	56	-14	0.05%
CCCTC----------CTTCTCGGCGCG	26	-10	0.02%
CCCTCCCGCGCCC--CTTCTCGGCGCG	25	-2	0.02%

P190-YW-U937-Syn26crCD326ac-RNPaav-8h-BC7 **8h**	Ins	Del	HDR
77907 | 10.37%	**2.26%**	**2.03%**	**6.08%**
**Typical seqs**	**Reads**	**Type**	**pct.**
CCCTCCCGCGCCCCTCT|TCT**CGG**CGCG	69828	WT	89.63%
CCCTCCCGCACGCGTAGTTTAAACCTCGGCGCG	4439	+6 HDR	5.70%
CCCTCCCGCGCCCCTCTtTCTCGGCGCG	1326	+1 t	1.70%
CCCTCCCGCGCCCCTC-TCTCGGCGCG	403	-1	0.52%
CCCTCCCGCGCCCC---TCTCGGCGCG	306	-3	0.39%
CCCTCCCGCGCCCCT-TTCTCGGCGCG	102	-1	0.13%
CCCTCCCGCGCCC--CTTCTCGGCGCG	58	-2	0.07%
CCCTCCCGCGCGCGTAGTTTAAACCTCGGCGCG	47	+6	0.06%
CCCTCCCGCGCCCCTCT--TCGGCGCG	46	-2	0.06%
CCCTCC---------------------	86	-21	0.11%

P191-YW-U937-Syn26crCD326ac-RNPaav-12h-BC7 **12h**	Ins	Del	HDR
117104 | 18.47%	**3.55%**	**3.30%**	**11.62%**
**Typical seqs**	**Reads**	**Type**	**pct.**
CCCTCCCGCGCCCCTCT|TCT**CGG**CGCG	95478	WT	81.53%
CCCTCCCGCACGCGTAGTTTAAACCTCGGCGCG	12744	+6 HDR	10.88%
CCCTCCCGCGCCCCTCTtTCTCGGCGCG	2943	+1 t	2.51%
CCCTCCCGCGCCCCTC-TCTCGGCGCG	1135	-1	0.97%
CCCTCCCGCGCCCC---TCTCGGCGCG	536	-3	0.46%
CCCTCCCGCGCCCCT-TTCTCGGCGCG	220	-1	0.19%
CCCTCCCGCGCGCGTAGTTTAAACCTCGGCGCG	180	+6	0.15%
CCCTCC---------------------	208	-21	0.18%
CCCTCCCGCGCCC--CTTCTCGGCGCG	128	-2	0.11%
CCCTCCCGCGCCC-----CTCGGCGCG	83	-5	0.07%

P192-YW-U937-Syn26crCD326ac-RNPaav-24h-BC7 **24h**	Ins	Del	HDR
71748 | 31.82%	**4.74%**	**4.39%**	**22.69%**
**Typical seqs**	**Reads**	**Type**	**pct.**
CCCTCCCGCGCCCCTCT|TCT**CGG**CGCG	48916	WT	68.18%
CCCTCCCGCACGCGTAGTTTAAACCTCGGCGCG	15223	+6 HDR	21.22%
CCCTCCCGCGCCCCTCTtTCTCGGCGCG	2080	+1 t	2.90%
CCCTCCCGCGCCCCTC-TCTCGGCGCG	660	-1	0.92%
CCCTCC---------------------	540	-21	0.75%
CCCTCCCGCGCCCC---TCTCGGCGCG	277	-3	0.39%
CCCTCCCGCGCGCGTAGTTTAAACCTCGGCGCG	223	+6	0.31%
CCCTCC---------------------	540	-23	0.75%
CCCTCCCGCGCCC----TCTCGGCGCG	101	-4	0.14%
CCCTCCCGCGCCCCT-TTCTCGGCGCG	92	-1	0.13%

P193-YW-U937-Syn26crCD326ac-RNPaav-48h-BC7 **48h**	Ins	Del	HDR
52902 | 41.43%	**4.81%**	**5.70%**	**30.92%**
**Typical seqs**	**Reads**	**Type**	**pct.**
CCCTCCCGCGCCCCTCT|TCT**CGG**CGCG	30986	WT	58.57%
CCCTCCCGCACGCGTAGTTTAAACCTCGGCGCG	15314	+6 HDR	28.95%
CCCTCCCGCGCCCCTCTtTCTCGGCGCG	1352	+1 t	2.56%
CCCTCCCGCGCCCCTC-TCTCGGCGCG	513	-1	0.97%
CCCTCC---------------------	720	-21	1.36%
CCCTCCCGCGCCCC---TCTCGGCGCG	318	-3	0.60%
CCCTCCCGCGCGCGTAGTTTAAACCTCGGCGCG	274	+6	0.52%
CCCTCC---------------------	720	-23	1.36%
CCCTCCCGCGCCCCT-TTCTCGGCGCG	82	-1	0.16%
CCCTCC-------------------CG	79	-19	0.15%

P193-YW-U937-Syn27crCD326ad-RNPaav

P189-YW-U937-Syn27crCD326ad-RNPaav-4h-BC8 4h	Ins	Del	HDR
92300 | 0.17%	0.01%	0.15%	0.01%
**Typical seqs**	**Reads**	**Type**	**pct.**
CGCGCCGAGAAGAGGGG|CGC**GGG**AGGG	92146	WT	99.83%
CGCGCCGAGAAGA-GGGCGCGGGAGGG	40	-1	0.04%
CGCGCCGAGAAGAGGGGCGC-GGAGGG	23	-1	0.02%
CGCGCCGAGAAGA--GGCGCGGGAGGG	11	-2	0.01%
CGCCCC---------TCTTCTCGGCGCGGGAGGG	9	-2	0.01%
CGCGCCGAGAA-----------GAGGG	8	-11	0.01%
CGCGCCGAGGTTTAAACTACGCGTGCGGGAGGG	8	+6 HDR	0.01%
CGCGCCGAGAAGAGGGGgCGCGGGAGGG	8	+1 g	0.01%
CGCGCCGAGA-------CGCGGGAGGG	6	-7	0.01%
CGCGCCGAGAAGAGGGGCGCGGAAGGG	108	-2	0.12%

P190-YW-U937-Syn27crCD326ad-RNPaav-8h-BC8 8h	Ins	Del	HDR
66831 | 0.18%	0.01%	0.16%	0.01%
**Typical seqs**	**Reads**	**Type**	**pct.**
CGCGCCGAGAAGAGGGG|CGC**GGG**AGGG	66714	WT	99.82%
CGCGCCGAGAAGA-GGGCGCGGGAGGG	37	-1	0.06%
CGCGCCGAGAAGAGGGGCGC-GGAGGG	13	-1	0.02%
CGCGCCGAGAAGAGGGGCGCAGGAGGG	234	-2	0.35%
C--------------------------	10	-26	0.01%
CGCGCCGAGAAGGGGGGCGCGGGAGGG	297	-2	0.44%
CGCGCCGAGAAG-GGGGCGCGGGAGGG	9	-1	0.01%
CGCGCCGA--------GCGCGGGAGGG	6	-8	0.01%
CGCGCCGAGGTTTAAACTACGCGTGCGGGAGGG	4	+6 HDR	0.01%
CGCGCCGAGA--AGGGGCGCGGGAGGG	4	-2	0.01%

P191-YW-U937-Syn27crCD326ad-RNPaav-12h-BC8 12h	Ins	Del	HDR
88690 | 0.22%	0.01%	0.16%	0.05%
**Typical seqs**	**Reads**	**Type**	**pct.**
CGCGCCGAGAAGAGGGG|CGC**GGG**AGGG	88499	WT	99.78%
CGCGCCGAGAAGA-GGGCGCGGGAGGG	43	-1	0.05%
CGCGCCGAGGTTTAAACTACGCGTGCGGGAGGG	41	+6 HDR	0.05%
CGCGCCGAGAAGAGGGGCGC-GGAGGG	22	-1	0.02%
CGCGCCGAGAA-----------GAGGG	10	-11	0.01%
CGCGCCGAGAAG-GGGGCGCGGGAGGG	10	-1	0.01%
CGCGCCGAGAAGAGG---------GGG	8	-9	0.01%
CGCGCCGAGAAGGGGGGCGCGGGAGGG	368	-2	0.41%
CGCGCCGAGAAGAGGGGCGCAGGAGGG	255	-2	0.29%
CGCGCCGAGAAGAGGGGgCGCGGGAGGG	6	+1 g	0.01%

P192-YW-U937-Syn27crCD326ad-RNPaav-24h-BC8 24h	Ins	Del	HDR
43300 | 0.39%	0.01%	0.31%	0.07%
**Typical seqs**	**Reads**	**Type**	**pct.**
CGCGCCGAGAAGAGGGG|CGC**GGG**AGGG	43129	WT	99.61%
CGCGCCGAGGTTTAAACTACGCGTGCGGGAGGG	30	+6 HDR	0.07%
CGCGCCGAGAAGAGGGGCGC-GGAGGG	21	-1	0.05%
CGCGCCGAGAAGA-GGGCGCGGGAGGG	20	-1	0.05%
CGCGCCGAGAA-----------GAGGG	21	-11	0.05%
----------------GCGCGGGAGGG	14	-21	0.03%
CGCGC-------------------GGG	9	-19	0.02%
CGCGCCGAGAAGA--GGCGCGGGAGGG	8	-2	0.02%
CGCGCCGAGAAG-GGGGCGCGGGAGGG	8	-1	0.02%
CGCGCCGAGAAGA---GCGCGGGAGGG	7	-3	0.02%

P193-YW-U937-Syn27crCD326ad-RNPaav-48h-BC8 48h	Ins	Del	HDR
27417 | 0.48%	0.01%	0.33%	0.14%
**Typical seqs**	**Reads**	**Type**	**pct.**
CGCGCCGAGAAGAGGGG|CGC**GGG**AGGG	27285	WT	99.52%
CGCGCCGAGGTTTAAACTACGCGTGCGGGAGGG	39	+6 HDR	0.14%
CGCGCCGAGAA-----------GAGGG	17	-11	0.06%
CGCGCCGAGAAGA-GGGCGCGGGAGGG	12	-1	0.04%
CGCGCCGAGAAGA--GGCGCGGGAGGG	10	-2	0.04%
CGCGCCGAGAAGA----CGCGGGAGGG	7	-4	0.03%
CGC------------------------	6	-24	0.02%
CGCGCCGA--------GCGCGGGAGGG	6	-8	0.02%
CGCGCCGAGAAGAGGGGCGAGGGAGGG	38	-2	0.14%
CGCGCCGAGAAGA-------GGGAGGG	5	-7	0.02%

P193-YW-U937-Syn28crCD326ae-RNPaav

P189-YW-U937-Syn28crCD326ae-RNPaav-4h-BC9 **4h**	Ins	Del	HDR
88380 | 0.78%	**0.10%**	**0.66%**	**0.02%**
**Typical seqs**	**Reads**	**Type**	**pct.**
GCGCGCCGAGAAGAGGG|GCG**CGG**GAGG	87688	WT	99.22%
GCGCGCCGAGAAGA-GGGCGCGGGAGG	297	-1	0.34%
GCGCGCCGAGAAGA--GGCGCGGGAGG	130	-2	0.15%
GCGCGCCGAGAAGAGGGgGCGCGGGAGG	77	+1 g	0.09%
GCGCGCCGAGA------GCGCGGGAGG	20	-6	0.02%
GCGCGCCGAGGTTTAAACTACGCGTGCGGGAGG	18	+6 HDR	0.02%
GCGC------------GGCGCGGGAGG	14	-12	0.02%
GCGCGCCGAGA-----GGCGCGGGAGG	11	-5	0.01%
GCGCGCCGAGA--AGGGGCGCGGGAGG	9	-2	0.01%
GCGCGCCGAGAA---GCGCGCGGGAGG	9	-3	0.01%

P190-YW-U937-Syn28crCD326ae-RNPaav-8h-BC9 **8h**	Ins	Del	HDR
69233 | 1.72%	**0.24%**	**1.28%**	**0.21%**
**Typical seqs**	**Reads**	**Type**	**pct.**
GCGCGCCGAGAAGAGGG|GCG**CGG**GAGG	68042	WT	98.28%
GCGCGCCGAGAAGA-GGGCGCGGGAGG	533	-1	0.77%
GCGCGCCGAGAAGAGGGgGCGCGGGAGG	146	+1 g	0.21%
GCGCGCCGAGGTTTAAACTACGCGTGCGGGAGG	142	+6 HDR	0.21%
GCGCGCCGAGAAGA--GGCGCGGGAGG	120	-2	0.17%
GCGCGCCGAGAAG-GGGGCGCGGGAGG	25	-1	0.04%
GCGCGCCGAGAAGA---GCGCGGGAGG	17	-3	0.02%
GCGCGCCGAGAA---GGGCGCGGGAGG	13	-3	0.02%
GCGCGCCGAGA--AGGGGCGCGGGAGG	12	-2	0.02%
GCGCGCCGAGAAGAG------------	12	-17	0.02%

P191-YW-U937-Syn28crCD326ae-RNPaav-12h-BC9 **12h**	Ins	Del	HDR
66907 | 2.99%	**0.37%**	**2.06%**	**0.55%**
**Typical seqs**	**Reads**	**Type**	**pct.**
GCGCGCCGAGAAGAGGG|GCG**CGG**GAGG	64905	WT	97.01%
GCGCGCCGAGAAGA-GGGCGCGGGAGG	643	-1	0.96%
GCGCGCCGAGGTTTAAACTACGCGTGCGGGAGG	367	+6 HDR	0.55%
GCGCGCCGAGAAGAGGGgGCGCGGGAGG	222	+1 g	0.33%
GCGCGCCGAGAAGA--GGCGCGGGAGG	208	-2	0.31%
-----------------GCGCGGGAGG	78	-21	0.12%
GCGCGCCGAGA-----GGCGCGGGAGG	50	-5	0.07%
GCGCGCCGAGAA-----------GAGG	34	-11	0.05%
GCGCGCCGAGAAGA---GCGCGGGAGG	26	-3	0.04%
GCGCGCCGAGAAGA-------GGGAGG	21	-7	0.03%

P192-YW-U937-Syn28crCD326ae-RNPaav-24h-BC9 **24h**	Ins	Del	HDR
49340 | 4.62%	**0.59%**	**2.99%**	**1.04%**
**Typical seqs**	**Reads**	**Type**	**pct.**
GCGCGCCGAGAAGAGGG|GCG**CGG**GAGG	47062	WT	95.38%
GCGCGCCGAGAAGA-GGGCGCGGGAGG	589	-1	1.19%
GCGCGCCGAGGTTTAAACTACGCGTGCGGGAGG	505	+6 HDR	1.02%
GCGCGCCGAGAAGAGGGgGCGCGGGAGG	240	+1 g	0.49%
GCGCGCCGAGAAGA--GGCGCGGGAGG	159	-2	0.32%
-----------------GCGCGGGAGG	130	-21	0.26%
GCGCGCCGAGAA-----------GAGG	83	-11	0.17%
GCGCGCCGAGAAGA-------------	53	-13	0.11%
GCGCGCCGAGAAGA-------GGGAGG	32	-7	0.06%
GCGCGCCGAGA------GCGCGGGAGG	23	-6	0.05%

P193-YW-U937-Syn28crCD326ae-RNPaav-48h-BC9 **48h**	Ins	Del	HDR
32082 | 5.10%	**0.47%**	**3.35%**	**1.28%**
**Typical seqs**	**Reads**	**Type**	**pct.**
GCGCGCCGAGAAGAGGG|GCG**CGG**GAGG	30445	WT	94.90%
GCGCGCCGAGGTTTAAACTACGCGTGCGGGAGG	395	+6 HDR	1.23%
GCGCGCCGAGAAGA-GGGCGCGGGAGG	356	-1	1.11%
GCGCGCCGAGAAGA--GGCGCGGGAGG	132	-2	0.41%
GCGCGCCGAGAAGAGGGgGCGCGGGAGG	128	+1 g	0.40%
-----------------GCGCGGGAGG	119	-21	0.37%
GCGCGCCGAGAA-----------GAGG	47	-11	0.15%
GCGCGCCGAGAAGA-------GGGAGG	39	-7	0.12%
GCGCGCCGAGAAGA-------------	66	-14	0.21%
GCGCGCCGAGAAGA------------G	26	-12	0.08%

P193-YW-U937-Syn29crCD326af-RNPaav

P189-YW-U937-Syn29crCD326af-RNPaav-4h-BC10 — 4h

72512 | 5.43%	Ins	Del	HDR
	**3.05%**	**1.17%**	**1.22%**

Typical seqs	Reads	Type	pct.
TGCGCGCGCGCCGAGAA|GAG**GGG**CGCG	68572	WT	94.57%
TGCGCGCGCGCCGAGAAaGAGGGGCGCG	1982	+1 a	2.73%
TGCGCGCGCGCCGAGGTTTAAACTACGCGTGCG	841	+6 HDR	1.16%
TGCGCGCGCGCCGAG-AGAGGGGCGCG	215	-1	0.30%
TGCGCGCGCGCCG---AGAGGGGCGCG	162	-3	0.22%
TGCGCGCGCGCCGAGAA-AGGGGCGCG	77	-1	0.11%
TGCGCGCGCGCC-----GAGGGGCGCG	45	-5	0.06%
T---------------------GCGCG	39	-21	0.05%
TGCGCGCGCGCCGAGAAGA-GGGCGCG	33	-1	0.05%
TGCGCGCGCGCCGAGA--AGGGGCGCG	30	-2	0.04%

P190-YW-U937-Syn29crCD326af-RNPaav-8h-BC10 — 8h

50338 | 16.53%	Ins	Del	HDR
	**6.51%**	**2.73%**	**7.29%**

Typical seqs	Reads	Type	pct.
TGCGCGCGCGCCGAGAA|GAG**GGG**CGCG	42016	WT	83.47%
TGCGCGCGCGCCGAGGTTTAAACTACGCGTGCG	3419	+6 HDR	6.79%
TGCGCGCGCGCCGAGAAaGAGGGGCGCG	2822	+1 a	5.61%
TGCGCGCGCGCCGAG-AGAGGGGCGCG	345	-1	0.69%
TGCGCGCGCGCCG---AGAGGGGCGCG	169	-3	0.34%
TGCGCGCGCGCCGAGAA-AGGGGCGCG	117	-1	0.23%
T---------------------GCGCG	60	-21	0.12%
TGCGCGCGCGCCGAGAAGA-GGGCGCG	50	-1	0.10%
TGCGCGCGCGCC-----GAGGGGCGCG	41	-5	0.08%
T-----------------------GCG	34	-23	0.07%

P191-YW-U937-Syn29crCD326af-RNPaav-12h-BC10 — 12h

35107 | 31.97%	Ins	Del	HDR
	**9.17%**	**4.86%**	**17.93%**

Typical seqs	Reads	Type	pct.
TGCGCGCGCGCCGAGAA|GAG**GGG**CGCG	23885	WT	68.03%
TGCGCGCGCGCCGAGGTTTAAACTACGCGTGCG	5850	+6 HDR	16.66%
TGCGCGCGCGCCGAGAAaGAGGGGCGCG	2580	+1 a	7.35%
TGCGCGCGCGCCGAG-AGAGGGGCGCG	300	-1	0.85%
TGCGCGCGCGCCG---AGAGGGGCGCG	223	-3	0.64%
T---------------------GCGCG	129	-21	0.37%
TGCGCGCGCGCCGAGAA-AGGGGCGCG	92	-1	0.26%
TGCGCGCGCGCCGAGGTTTAAACTACGCGCGCG	72	+6	0.21%
T-----------------------GCG	56	-23	0.16%
TGCGC-----------AGAGGGGCGCG	39	-11	0.11%

P192-YW-U937-Syn29crCD326af-RNPaav-24h-BC10 — 24h

32962 | 44.71%	Ins	Del	HDR
	**10.82%**	**5.13%**	**28.76%**

Typical seqs	Reads	Type	pct.
TGCGCGCGCGCCGAGAA|GAG**GGG**CGCG	18225	WT	55.29%
TGCGCGCGCGCCGAGGTTTAAACTACGCGTGCG	8827	+6 HDR	26.78%
TGCGCGCGCGCCGAGAAaGAGGGGCGCG	2667	+1 a	8.09%
TGCGCGCGCGCCGAG-AGAGGGGCGCG	301	-1	0.91%
TGCGCGCGCGCCG---AGAGGGGCGCG	210	-3	0.64%
TGCGCGCGCGCCGAGGTTTAAACTACGCGCGCG	183	+6	0.56%
T---------------------GCGCG	156	-21	0.47%
TGCGCGCGCGCCGAGAA-AGGGGCGCG	72	-1	0.22%
T-----------------------GCG	62	-23	0.19%
TGCGCGCGCGCCGAGGTTTAGACTACGCGTGCG	44	+6	0.13%

P193-YW-U937-Syn29crCD326af-RNPaav-48h-BC10 — 48h

15274 | 54.01%	Ins	Del	HDR
	**11.08%**	**5.45%**	**37.48%**

Typical seqs	Reads	Type	pct.
TGCGCGCGCGCCGAGAA|GAG**GGG**CGCG	7024	WT	45.99%
TGCGCGCGCGCCGAGGTTTAAACTACGCGTGCG	5354	+6 HDR	35.05%
TGCGCGCGCGCCGAGAAaGAGGGGCGCG	1231	+1 a	8.06%
TGCGCGCGCGCCGAG-AGAGGGGCGCG	110	-1	0.72%
TGCGCGCGCGCCGAGGTTTAAACTACGCGCGCG	106	+6	0.69%
TGCGCGCGCGCCG---AGAGGGGCGCG	91	-3	0.60%
TGCGCGCGCGCCGAGAA-AGGGGCGCG	52	-1	0.34%
T---------------------GCGCG	68	-21	0.45%
T-----------------------GCG	38	-23	0.25%
TGCGCGCGCGCCGAGA--AGGGGCGCG	27	-2	0.18%

P193-YW-U937-Syn30crCD326ag-RNPko

P189-YW-U937-Syn30crCD326ag-RNPko-4h-BC5 4h	Ins	Del	HDR
49094 | 0.57%	0.10%	0.46%	0.00%
**Typical seqs**	**Reads**	**Type**	**pct.**
CTGCGCGCGCGCCGAGA|AGA**GGG**GCGC	48815	WT	99.43%
CTGCGCGCGCGCCGAG-AGAGGGGCGC	96	-1	0.20%
CTGCGCGCGCGCCG---AGAGGGGCGC	47	-3	0.10%
CTGCGCGCGCGCCGAGAaAGAGGGGCGC	43	+1 a	0.09%
CTGCG------------AGAGGGGCGC	10	-12	0.02%
CTGCGCGCGCGCC-----GAGGGGCGC	8	-5	0.02%
CTGCGCG----------AGAGGGGCGC	8	-10	0.02%
CTGCGCGCGCGCCGAGAcAGAGGGGCGC	8	+1 c	0.02%
CTGCGCGCGCGCCGAGA----GGGCGC	6	-4	0.01%
CTGCGCGCGCGCCGAGAAG-GGGGCGC	6	-1	0.01%

P190-YW-U937-Syn30crCD326ag-RNPko-8h-BC5 8h	Ins	Del	HDR
39908 | 1.17%	0.26%	0.91%	0.00%
**Typical seqs**	**Reads**	**Type**	**pct.**
CTGCGCGCGCGCCGAGA|AGA**GGG**GCGC	39441	WT	98.83%
CTGCGCGCGCGCCGAG-AGAGGGGCGC	151	-1	0.38%
CTGCGCGCGCGCCGAGAaAGAGGGGCGC	99	+1 a	0.25%
CTGCGCGCGCGCCG---AGAGGGGCGC	51	-3	0.13%
CT-----------------------GC	16	-23	0.04%
CT---------------------GCGC	16	-21	0.04%
CTGCGCGCGCGCCGAGA----GGGCGC	15	-4	0.04%
CTGCGCGCGCGCCGAGA--AGGGGCGC	10	-2	0.03%
CTG--------------AGAGGGGCGC	8	-14	0.02%
CTGCGC-----------------GCGC	8	-17	0.02%

P191-YW-U937-Syn30crCD326ag-RNPko-12h-BC5 12h	Ins	Del	HDR
29593 | 2.82%	0.38%	2.43%	0.01%
**Typical seqs**	**Reads**	**Type**	**pct.**
CTGCGCGCGCGCCGAGA|AGA**GGG**GCGC	28759	WT	97.18%
CTGCGCGCGCGCCGAG-AGAGGGGCGC	190	-1	0.64%
CTGCGCGCGCGCCG---AGAGGGGCGC	128	-3	0.43%
CTGCGCGCGCGCCGAGAaAGAGGGGCGC	107	+1 a	0.36%
CT---------------------GCGC	85	-21	0.29%
CT-----------------------GC	34	-23	0.11%
-----------------------GCGC	23	-24	0.08%
CTGCGCGCGCGCCGA------------	22	-14	0.07%
CTGCGC-----------------GCGC	14	-17	0.05%
CTGCGCGCGCGCC-----GAGGGGCGC	13	-5	0.04%

P192-YW-U937-Syn30crCD326ag-RNPko-24h-BC5 24h	Ins	Del	HDR
13360 | 3.98%	0.45%	3.53%	0.00%
**Typical seqs**	**Reads**	**Type**	**pct.**
CTGCGCGCGCGCCGAGA|AGA**GGG**GCGC	12828	WT	96.02%
CTGCGCGCGCGCCGAG-AGAGGGGCGC	110	-1	0.82%
CT---------------------GCGC	84	-21	0.63%
CTGCGCGCGCGCCGAGAaAGAGGGGCGC	56	+1 a	0.42%
CTGCGCGCGCGCCG---AGAGGGGCGC	31	-3	0.23%
CT-----------------------GC	25	-23	0.19%
-----------------------GCGC	20	-24	0.15%
CTGCG------------AGAGGGGCGC	12	-12	0.09%
CTGCGCGCGCGCCGAGA----------	18	-12	0.13%
CTGCGCG----------AGAGGGGCGC	11	-10	0.08%

P193-YW-U937-Syn30crCD326ag-RNPko-48h-BC5 48h	Ins	Del	HDR
6821 | 8.66%	0.62%	8.05%	0.00%
**Typical seqs**	**Reads**	**Type**	**pct.**
CTGCGCGCGCGCCGAGA|AGA**GGG**GCGC	6230	WT	91.34%
CT---------------------GCGC	116	-21	1.70%
CTGCGCGCGCGCCGAG-AGAGGGGCGC	95	-1	1.39%
CT-----------------------GC	60	-23	0.88%
CTGCGCGCGCGCCG---AGAGGGGCGC	56	-3	0.82%
-----------------------GCGC	42	-24	0.62%
CTGCGCGCGCGCCGAGAaAGAGGGGCGC	36	+1 a	0.53%
CTGC-------------------GCGC	11	-19	0.16%
CTGC-----------------------	11	-30	0.16%
CTGCGCGCGCGCC--------------	11	-16	0.16%

P193-YW-U937-Syn30crCD326ag-RNPaav

P189-YW-U937-Syn30crCD326ag-RNPaav-4h-BC11 (4h)	Ins	Del	HDR
63837 | 0.73%	0.08%	0.38%	0.27%
**Typical seqs**	**Reads**	**Type**	**pct.**
CTGCGCGCGCGCCGAGA|AGA**GGG**GCGC	63371	WT	99.27%
CTGCGCGCGCGCCGAGGTTTAAACTACGCGTGC	163	+6 HDR	0.26%
CTGCGCGCGCGCCGAG-AGAGGGGCGC	127	-1	0.20%
CTGCGCGCGCGCCGAGAaAGAGGGGCGC	39	+1 a	0.06%
CTGCGCGCGCGCCG---AGAGGGGCGC	36	-3	0.06%
CTGCGCGCGCGCCGAGA----GGGCGC	13	-4	0.02%
CTGCGCGCGCGCC--GAAGAGGGGCGC	11	-2	0.02%
CTGCGCGCGCGCCGAGAcAGAGGGGCGC	6	+1 c	0.01%
CTGCGCGCGCGCCGAGA------GCGC	6	-6	0.01%
CTGCGCGCGCGCCGAGA-----GGCGC	6	-5	0.01%

P190-YW-U937-Syn30crCD326ag-RNPaav-8h-BC11 (8h)	Ins	Del	HDR
48189 | 3.06%	0.36%	1.06%	1.64%
**Typical seqs**	**Reads**	**Type**	**pct.**
CTGCGCGCGCGCCGAGA|AGA**GGG**GCGC	46714	WT	96.94%
CTGCGCGCGCGCCGAGGTTTAAACTACGCGTGC	759	+6 HDR	1.58%
CTGCGCGCGCGCCGAG-AGAGGGGCGC	220	-1	0.46%
CTGCGCGCGCGCCGAGAaAGAGGGGCGC	122	+1 a	0.25%
CTGCGCGCGCGCCG---AGAGGGGCGC	91	-3	0.19%
CT---------------------GCGC	15	-21	0.03%
CTGCGCGCGCGCCGAGA----------	19	-15	0.04%
CTGCGCGCGCGCC--GAAGAGGGGCGC	11	-2	0.02%
CTGC-------------------GCGC	13	-19	0.03%
CT-----------------------GC	10	-23	0.02%

P191-YW-U937-Syn30crCD326ag-RNPaav-12h-BC11 (12h)	Ins	Del	HDR
44146 | 8.78%	0.85%	3.00%	4.93%
**Typical seqs**	**Reads**	**Type**	**pct.**
CTGCGCGCGCGCCGAGA|AGA**GGG**GCGC	40268	WT	91.22%
CTGCGCGCGCGCCGAGGTTTAAACTACGCGTGC	2051	+6 HDR	4.65%
CTGCGCGCGCGCCGAG-AGAGGGGCGC	414	-1	0.94%
CTGCGCGCGCGCCGAGAaAGAGGGGCGC	216	+1 a	0.49%
CTGCGCGCGCGCCG---AGAGGGGCGC	161	-3	0.36%
CT---------------------GCGC	112	-21	0.25%
CTGCGCGCGCGCC--GAAGAGGGGCGC	43	-2	0.10%
-----------------------GCGC	35	-24	0.08%
CTGCGCGCGCGCCGA------------	36	-14	0.08%
CT-----------------------GC	29	-23	0.07%

P192-YW-U937-Syn30crCD326ag-RNPaav-24h-BC11 (24h)	Ins	Del	HDR
40119 | 13.44%	1.15%	3.44%	8.85%
**Typical seqs**	**Reads**	**Type**	**pct.**
CTGCGCGCGCGCCGAGA|AGA**GGG**GCGC	34726	WT	86.56%
CTGCGCGCGCGCCGAGGTTTAAACTACGCGTGC	3293	+6 HDR	8.21%
CTGCGCGCGCGCCGAG-AGAGGGGCGC	347	-1	0.86%
CTGCGCGCGCGCCGAGAaAGAGGGGCGC	221	+1 a	0.55%
CTGCGCGCGCGCCG---AGAGGGGCGC	196	-3	0.49%
CT---------------------GCGC	163	-21	0.41%
CT-----------------------GC	55	-23	0.14%
CTGCGCGCGCGCC-----GAGGGGCGC	45	-5	0.11%
-----------------------GCGC	40	-24	0.10%
CTGCGCGCGCGCC--GAAGAGGGGCGC	29	-2	0.07%

P193-YW-U937-Syn30crCD326ag-RNPaav-48h-BC11 (48h)	Ins	Del	HDR
27196 | 17.57%	1.40%	4.89%	11.27%
**Typical seqs**	**Reads**	**Type**	**pct.**
CTGCGCGCGCGCCGAGA|AGA**GGG**GCGC	22419	WT	82.43%
CTGCGCGCGCGCCGAGGTTTAAACTACGCGTGC	2896	+6 HDR	10.65%
CTGCGCGCGCGCCGAG-AGAGGGGCGC	286	-1	1.05%
CT---------------------GCGC	218	-21	0.80%
CTGCGCGCGCGCCG---AGAGGGGCGC	163	-3	0.60%
CTGCGCGCGCGCCGAGAaAGAGGGGCGC	160	+1 a	0.59%
CT-----------------------GC	73	-23	0.27%
CTGCGCGCGCGCCGAGGTTTAAACTACGCGCGC	66	+6	0.24%
-----------------------GCGC	55	-24	0.20%
-------------------------GC	28	-26	0.10%

P193-YW-U937-Syn31crCD326ah-RNPko

P189-YW-U937-Syn31crCD326ah-RNPko-4h-BC6 **4h**	Ins	Del	HDR
86593 | 1.00%	**0.41%**	**0.59%**	**0.00%**
**Typical seqs**	**Reads**	**Type**	**pct.**
GCTGCGCGCGCGCCGAG|AAG**AGG**GGCG	85727	WT	99.00%
GCTGCGCGCGCGCCGAGgAAGAGGGGCG	323	+1 g	0.37%
GCTGCGCGCGCGCC--GAAGAGGGGCG	83	-2	0.10%
GCTGCGCGCGCGCCGAG--GAGGGGCG	44	-2	0.05%
GCTGCGCGCGCGCCGA-AAGAGGGGCG	31	-1	0.04%
GCTGCGCGCGCGCCG---AGAGGGGCG	29	-3	0.03%
GCTGC------------AAGAGGGGCG	24	-12	0.03%
GCTGCGCGCGCGCC-----GAGGGGCG	23	-5	0.03%
GCTGCGCGCGCGCC---AAGAGGGGCG	19	-3	0.02%
GCTGCGCGCGCGCCG-GAAGAGGGGCG	19	-1	0.02%

P190-YW-U937-Syn31crCD326ah-RNPko-8h-BC6 **8h**	Ins	Del	HDR
70842 | 2.19%	**0.77%**	**1.41%**	**0.00%**
**Typical seqs**	**Reads**	**Type**	**pct.**
GCTGCGCGCGCGCCGAG|AAG**AGG**GGCG	69293	WT	97.81%
GCTGCGCGCGCGCCGAGgAAGAGGGGCG	479	+1 g	0.68%
GCTGCGCGCGCGCC--GAAGAGGGGCG	155	-2	0.22%
GCTGCGCGCGCGCC-----GAGGGGCG	98	-5	0.14%
GCT---------------------GCG	77	-21	0.11%
GCTGCGCGCGCGCCGAG--GAGGGGCG	41	-2	0.06%
GCTGCGCGCGCGCCGA-AAGAGGGGCG	41	-1	0.06%
GCTGCGCGCGCGCC---AAGAGGGGCG	38	-3	0.05%
GCTGCGC---------GAAGAGGGGCG	31	-9	0.04%
GCTGC------------AAGAGGGGCG	26	-12	0.04%

P191-YW-U937-Syn31crCD326ah-RNPko-12h-BC6 **12h**	Ins	Del	HDR
82791 | 4.00%	**1.02%**	**2.98%**	**0.00%**
**Typical seqs**	**Reads**	**Type**	**pct.**
GCTGCGCGCGCGCCGAG|AAG**AGG**GGCG	79482	WT	96.00%
GCTGCGCGCGCGCCGAGgAAGAGGGGCG	729	+1 g	0.88%
GCT---------------------GCG	339	-21	0.41%
GCTGCGCGCGCGCC--GAAGAGGGGCG	168	-2	0.20%
GCTGCGCGCGCGCC-----GAGGGGCG	166	-5	0.20%
GCTGCGCGCGCGCCGAG--GAGGGGCG	96	-2	0.12%
GCTGC-------------------GCG	83	-19	0.10%
GCT-----------------------G	76	-23	0.09%
GCTGCGCGCGCGCCGA-AAGAGGGGCG	75	-1	0.09%
GCTGCGCGCGCGCC---AAGAGGGGCG	67	-3	0.08%

P192-YW-U937-Syn31crCD326ah-RNPko-24h-BC6 **24h**	Ins	Del	HDR
36401 | 6.44%	**1.17%**	**5.27%**	**0.00%**
**Typical seqs**	**Reads**	**Type**	**pct.**
GCTGCGCGCGCGCCGAG|AAG**AGG**GGCG	34055	WT	93.56%
GCTGCGCGCGCGCCGAGgAAGAGGGGCG	399	+1 g	1.10%
GCT---------------------GCG	365	-21	1.00%
GCT-----------------------G	156	-23	0.43%
GCTGCGCGCGCGCC--GAAGAGGGGCG	122	-2	0.34%
GCTGCGCGCGCGCC-----GAGGGGCG	108	-5	0.30%
GCTGCGCGCGCGCCGA-AAGAGGGGCG	59	-1	0.16%
GCTGCGC-----------------GCG	55	-17	0.15%
GCTGCGCGCGCGCCGA--------GCG	51	-8	0.14%
GCTGCGCGCGCGCCGAG--GAGGGGCG	48	-2	0.13%

P193-YW-U937-Syn31crCD326ah-RNPko-48h-BC6 **48h**	Ins	Del	HDR
30817 | 9.01%	**1.62%**	**7.40%**	**0.00%**
**Typical seqs**	**Reads**	**Type**	**pct.**
GCTGCGCGCGCGCCGAG|AAG**AGG**GGCG	28039	WT	90.99%
GCTGCGCGCGCGCCGAGgAAGAGGGGCG	447	+1 g	1.45%
GCT---------------------GCG	421	-21	1.37%
GCTGCGCGCGCGCC--GAAGAGGGGCG	156	-2	0.51%
GCT-----------------------G	125	-23	0.41%
GCTGCGCGCGCGCC-----GAGGGGCG	121	-5	0.39%
GCTGCGCGCGCGCCGA-AAGAGGGGCG	81	-1	0.26%
------------------------GCG	62	-24	0.20%
GCTGCGC----------AAGAGGGGCG	45	-10	0.15%
GCTGCGCGCGCGCCGAG--GAGGGGCG	47	-2	0.15%

P84-YW-K562-Syn20crAAVS1c-KO

Total | INDEL	4h	Ins	Del	HDR
22987 | 33.33%		26.15%	7.18%	0.00%

Typical seqs	Reads	Type	pct.
TCTAACCCCCACCTCCT|GTT**AGG**CAGA	15326	WT	66.67%
TCTAACCCCCACCTCCTtGTTAGGCAGA	5219	+1 t	22.70%
TCTAACCCCCACCTCC--TTAGGCAGA	201	-2	0.87%
TCTAACCCCCA--TCCTGTTAGGCAGA	111	-2	0.48%
TCTAACCCCCA-CTCCTGTTAGGCAGA	98	-1	0.43%
TCTAACCCCCACC--------------	83	-15	0.36%
TCTAACCCCCA---CCTGTTAGGCAGA	78	-3	0.34%
TCTAACCCCCA----CTGTTAGGCAGA	59	-4	0.26%
TCTAACTCCCACCTCCTTGTTAGGCAGA	51	+1 Ins	0.22%
TCTAACCCCCACCT-CTGTTAGGCAGA	51	-1	0.22%

Total | INDEL	8h	Ins	Del	HDR
41097 | 44.00%		33.91%	10.09%	0.00%

Typical seqs	Reads	Type	pct.
TCTAACCCCCACCTCCT|GTT**AGG**CAGA	23015	WT	56.00%
TCTAACCCCCACCTCCTtGTTAGGCAGA	11797	+1 t	28.71%
TCTAACCCCCACCTCC--TTAGGCAGA	456	-2	1.11%
TCTAACCCCCACC--------------	274	-15	0.67%
TCTAACCCCCA--TCCTGTTAGGCAGA	219	-2	0.53%
TCTAACCCCCA-CTCCTGTTAGGCAGA	212	-1	0.52%
TCTAACCCCCACCT-CTGTTAGGCAGA	158	-1	0.38%
TCTAACCCCCA---CCTGTTAGGCAGA	145	-3	0.35%
TCTAACCCCCACCTCCTCTGTTAGGCAGA	143	+2	0.35%
TCTAACCCCCA----CTGTTAGGCAGA	147	-4	0.36%

Total | INDEL	12h	Ins	Del	HDR
100096 | 47.94%		35.72%	12.23%	0.00%

Typical seqs	Reads	Type	pct.
TCTAACCCCCACCTCCT|GTT**AGG**CAGA	52105	WT	52.06%
TCTAACCCCCACCTCCTtGTTAGGCAGA	30157	+1 t	30.13%
TCTAACCCCCACCTCC--TTAGGCAGA	1147	-2	1.15%
TCTAACCCCCACC--------------	961	-15	0.96%
TCTAACCCCCA--TCCTGTTAGGCAGA	505	-2	0.50%
TCTAACCCCCA-CTCCTGTTAGGCAGA	483	-1	0.48%
TCTAACCCCCA---CCTGTTAGGCAGA	453	-3	0.45%
TCTAACCCCCACCT-CTGTTAGGCAGA	408	-1	0.41%
TCTAACCCCCA----CTGTTAGGCAGA	387	-4	0.39%
TCTAACTCCCACCTCCTTGTTAGGCAGA	349	+1 Ins	0.35%

Total | INDEL	24h	Ins	Del	HDR
44169 | 50.10%		35.85%	14.25%	0.00%

Typical seqs	Reads	Type	pct.
TCTAACCCCCACCTCCT|GTT**AGG**CAGA	22039	WT	49.90%
TCTAACCCCCACCTCCTtGTTAGGCAGA	13432	+1 t	30.41%
TCTAACCCCCACCTCC--TTAGGCAGA	551	-2	1.25%
TCTAACCCCCACC--------------	624	-15	1.41%
TCTAACCCCCA--TCCTGTTAGGCAGA	261	-2	0.59%
TCTAACCCCCA-CTCCTGTTAGGCAGA	232	-1	0.53%
TCTAACCCCCACCT-CTGTTAGGCAGA	209	-1	0.47%
TCTAACCCCCA----CTGTTAGGCAGA	191	-4	0.43%
TCTAACCCCCA---CCTGTTAGGCAGA	185	-3	0.42%
TCTAACCCCCA----------------	186	-18	0.42%

Total | INDEL	48h	Ins	Del	HDR
36053 | 50.90%		35.23%	15.67%	0.00%

Typical seqs	Reads	Type	pct.
TCTAACCCCCACCTCCT|GTT**AGG**CAGA	17701	WT	49.10%
TCTAACCCCCACCTCCTtGTTAGGCAGA	10843	+1 t	30.08%
TCTAACCCCCACC--------------	614	-15	1.70%
TCTAACCCCCACCTCC--TTAGGCAGA	411	-2	1.14%
TCTAACCCCCA----------------	241	-18	0.67%
TCTAACCCCCA----CTGTTAGGCAGA	174	-4	0.48%
TCTAACCCCCA--TCCTGTTAGGCAGA	165	-2	0.46%
TCTAACCCCCA-CTCCTGTTAGGCAGA	147	-1	0.41%
TCTAACCCCCACCT-CTGTTAGGCAGA	152	-1	0.42%
TCTAACCCCCA---CCTGTTAGGCAGA	145	-3	0.40%

P85-YW-K562-Syn20crAAVS1c-AAV6KI

4h

Total	INDEL	Ins	Del	HDR
21404	41.34%	22.21%	5.62%	13.51%

Typical seqs	Reads	Type	pct.
TCTAACCCCCACCTCCTGTT**AGG**CAGA	12556	WT	58.66%
TCTAACCCCCACCTCCTtGTTAGGCAGA	3885	+1 t	18.15%
TCTAACCCACGCGTAGTTTAAACTTAGGCAGA	2685	+5 HDR	12.54%
TCTAACCCCCACCTCC--TTAGGCAGA	165	-2	0.77%
TCTAACCCCCA--TCCTGTTAGGCAGA	107	-2	0.50%
TCTAACCCCCA-CTCCTGTTAGGCAGA	90	-1	0.42%
TCTAACCCCCA----CTGTTAGGCAGA	61	-4	0.28%
TCTAACCCCCA---CCTGTTAGGCAGA	54	-3	0.25%
TCTAACCCCCACCTCCTCTGTTAGGCAGA	53	+2	0.25%
TCTAACCCCCACCTCCTtGTTAGGCGGA	45	+1 t	0.21%

8h

Total	INDEL	Ins	Del	HDR
31839	52.09%	23.99%	7.15%	20.95%

Typical seqs	Reads	Type	pct.
TCTAACCCCCACCTCCTGTT**AGG**CAGA	15255	WT	47.91%
TCTAACCCCCACCTCCTtGTTAGGCAGA	6120	+1 t	19.22%
TCTAACCCACGCGTAGTTTAAACTTAGGCAGA	6120	+5 HDR	19.22%
TCTAACCCCCACCTCC--TTAGGCAGA	236	-2	0.74%
TCTAACCCCCA--TCCTGTTAGGCAGA	193	-2	0.61%
TCTAACCCCCA-CTCCTGTTAGGCAGA	165	-1	0.52%
TCTAACCCCCA----CTGTTAGGCAGA	93	-4	0.29%
TCTAACCCCCA---CCTGTTAGGCAGA	85	-3	0.27%
TCTAACCCCCACCT-CTGTTAGGCAGA	78	-1	0.24%
TCTAACCCCCACC--------------	76	-15	0.24%

12h

Total	INDEL	Ins	Del	HDR
50366	55.53%	25.59%	7.68%	22.26%

Typical seqs	Reads	Type	pct.
TCTAACCCCCACCTCCTGTT**AGG**CAGA	22400	WT	44.47%
TCTAACCCACGCGTAGTTTAAACTTAGGCAGA	10331	+5 HDR	20.51%
TCTAACCCCCACCTCCTtGTTAGGCAGA	10119	+1 t	20.09%
TCTAACCCCCACCTCC--TTAGGCAGA	462	-2	0.92%
TCTAACCCCCA--TCCTGTTAGGCAGA	297	-2	0.59%
TCTAACCCCCA-CTCCTGTTAGGCAGA	231	-1	0.46%
TCTAACCCCCA---CCTGTTAGGCAGA	183	-3	0.36%
TCTAACCCCCA----CTGTTAGGCAGA	158	-4	0.31%
TCTAACCCCCACC--------------	148	-15	0.29%
TCTACCCCCCACCTCCTTGTTAGGCAGA	138	+1 Ins	0.27%

24h

Total	INDEL	Ins	Del	HDR
26263	55.00%	23.66%	7.19%	24.16%

Typical seqs	Reads	Type	pct.
TCTAACCCCCACCTCCTGTT**AGG**CAGA	11818	WT	45.00%
TCTAACCCACGCGTAGTTTAAACTTAGGCAGA	5838	+5 HDR	22.23%
TCTAACCCCCACCTCCTtGTTAGGCAGA	4907	+1 t	18.68%
TCTAACCCCCACCTCC--TTAGGCAGA	161	-2	0.61%
TCTAACCCCCA--TCCTGTTAGGCAGA	146	-2	0.56%
TCTAACCCCCA-CTCCTGTTAGGCAGA	122	-1	0.46%
TCTAACCCCCACC--------------	107	-15	0.41%
TCTAACCCCCA----CTGTTAGGCAGA	82	-4	0.31%
TCTAACCCCCA---CCTGTTAGGCAGA	75	-3	0.29%
TCTAACCCCCACCT-CTGTTAGGCAGA	77	-1	0.29%

48h

Total	INDEL	Ins	Del	HDR
20691	55.37%	23.27%	6.96%	25.13%

Typical seqs	Reads	Type	pct.
TCTAACCCCCACCTCCTGTT**AGG**CAGA	9235	WT	44.63%
TCTAACCCACGCGTAGTTTAAACTTAGGCAGA	4779	+5 HDR	23.10%
TCTAACCCCCACCTCCTtGTTAGGCAGA	3767	+1 t	18.21%
TCTAACCCCCACCTCC--TTAGGCAGA	141	-2	0.68%
TCTAACCCCCA-CTCCTGTTAGGCAGA	109	-1	0.53%
TCTAACCCCCA--TCCTGTTAGGCAGA	109	-2	0.53%
TCTAACCCCCACC--------------	78	-15	0.38%
TCTAACCCCCA----CTGTTAGGCAGA	58	-4	0.28%
TCTAACCCCCA---CCTGTTAGGCAGA	60	-3	0.29%
TCTAACCCCCA-----TGTTAGGCAGA	46	-5	0.22%

P84-YW-K562-Syn21crAAVS1d-KO

Total | INDEL	4h	Ins	Del	HDR
40882 | 9.92%		2.20%	7.71%	0.00%

Typical seqs	Reads	Type	pct.
ATCTGCCTAACAGGAGG|TGGGGGTTAG	36828	WT	90.08%
ATCTGCCTAACAGGAGGgTGGGGGTTAG	467	+1 g	1.14%
ATCTGCCTAACAGGA--TGGGGGTTAG	294	-2	0.72%
ATCTGCCTAACAGGA------GGTTAG	257	-6	0.63%
ATCTGCCTAACAGGA-GTGGGGGTTAG	252	-1	0.62%
ATCTGCCTAACAGGAGGT-GGGGTTAG	159	-1	0.39%
ATCTGCCTAAC---AGGTGGGGGTTAG	110	-3	0.27%
ATCTGCCTAACA-----TGGGGGTTAG	105	-5	0.26%
ATCTGCCTAACAGGAGG-GGGGGTTAG	102	-1	0.25%
ATCTGCCTAACA----GTGGGGGTTAG	101	-4	0.25%

Total | INDEL	8h	Ins	Del	HDR
176070 | 14.09%		2.62%	11.47%	0.00%

Typical seqs	Reads	Type	pct.
ATCTGCCTAACAGGAGG|TGGGGGTTAG	151256	WT	85.91%
ATCTGCCTAACAGGAGGgTGGGGGTTAG	2130	+1 g	1.21%
ATCTGCCTAACAGGA--TGGGGGTTAG	2031	-2	1.15%
ATCTGCCTAACAGGA------GGTTAG	2012	-6	1.14%
ATCTGCCTAACAGGA-GTGGGGGTTAG	1271	-1	0.72%
ATCTGCCTAACA----GTGGGGGTTAG	863	-4	0.49%
ATCTGCCTAACAGGAGG-GGGGGTTAG	749	-1	0.43%
ATCTGCCTAACAGGAGGT-GGGGTTAG	676	-1	0.38%
ATCTGCCTAACA-----TGGGGGTTAG	607	-5	0.34%
ATCTGCCTAAC------TGGGGGTTAG	620	-6	0.35%

Total | INDEL	12h	Ins	Del	HDR
124844 | 16.02%		2.76%	13.27%	0.00%

Typical seqs	Reads	Type	pct.
ATCTGCCTAACAGGAGG|TGGGGGTTAG	104838	WT	83.98%
ATCTGCCTAACAGGA--TGGGGGTTAG	1812	-2	1.45%
ATCTGCCTAACAGGA------GGTTAG	1632	-6	1.31%
ATCTGCCTAACAGGAGGgTGGGGGTTAG	1592	+1 g	1.28%
ATCTGCCTAACAGGA-GTGGGGGTTAG	1140	-1	0.91%
ATCTGCCTAACAGGAGG-GGGGGTTAG	626	-1	0.50%
ATCTGCCTAACA----GTGGGGGTTAG	602	-4	0.48%
ATCTGCCTAACAGGAGGT-GGGGTTAG	530	-1	0.42%
ATCTGCCTAACA-----TGGGGGTTAG	518	-5	0.41%
ATCTGCCTAAC------TGGGGGTTAG	453	-6	0.36%

Total | INDEL	24h	Ins	Del	HDR
104306 | 21.53%		3.11%	18.42%	0.00%

Typical seqs	Reads	Type	pct.
ATCTGCCTAACAGGAGG|TGGGGGTTAG	81853	WT	78.47%
ATCTGCCTAACAGGA------GGTTAG	2878	-6	2.76%
ATCTGCCTAACAGGA--TGGGGGTTAG	1883	-2	1.81%
ATCTGCCTAACAGGAGGgTGGGGGTTAG	1297	+1 g	1.24%
ATCTGCCTAACAGGA-GTGGGGGTTAG	923	-1	0.88%
ATCTGCCTAACAGGAGG-GGGGGTTAG	585	-1	0.56%
ATCTGCCTAAC------TGGGGGTTAG	564	-6	0.54%
ATCTGCCTAACA-----TGGGGGTTAG	557	-5	0.53%
ATCTGCCTAACA----GTGGGGGTTAG	467	-4	0.45%
ATCTGCCTAA-------TGGGGGTTAG	439	-7	0.42%

Total | INDEL	48h	Ins	Del	HDR
65029 | 23.25%		3.06%	20.18%	0.00%

Typical seqs	Reads	Type	pct.
ATCTGCCTAACAGGAGG|TGGGGGTTAG	49913	WT	76.75%
ATCTGCCTAACAGGA------GGTTAG	1931	-6	2.97%
ATCTGCCTAACAGGA--TGGGGGTTAG	1140	-2	1.75%
ATCTGCCTAACAGGAGGgTGGGGGTTAG	846	+1 g	1.30%
ATCTGCCTAACAGGA-GTGGGGGTTAG	641	-1	0.99%
ATCTGCCTAACAGGA-----GGGTTAG	463	-5	0.71%
ATCTGCCTAACAGGAGG-GGGGGTTAG	398	-1	0.61%
ATCTGCCTAACA-----TGGGGGTTAG	387	-5	0.60%
ATCTGCCTAACA----GTGGGGGTTAG	368	-4	0.57%
ATCTGCCTAAC------TGGGGGTTAG	372	-6	0.57%

P85-YW-K562-Syn21crAAVS1d-AAV6KI

4h

Total	INDEL	Ins	Del	HDR
52516	20.44%	2.64%	6.58%	11.22%

Typical seqs	Reads	Type	pct.
ATCTGCCTAACAGGAGG|TGG**GGG**TTAG	41783	WT	79.56%
ATCTGCCTAAGTTTAAACTACGCGTGGGTTAG	5393	+5 HDR	10.27%
ATCTGCCTAACAGGAGGgTGGGGGTTAG	534	+1 g	1.02%
ATCTGCCTAACAGGA--TGGGGGTTAG	477	-2	0.91%
ATCTGCCTAACAGGA-GTGGGGGTTAG	327	-1	0.62%
ATCTGCCTAACAGGAGGT-GGGGTTAG	199	-1	0.38%
ATCTGCCTAACAGGA------GGTTAG	204	-6	0.39%
ATCTGCCTAACA-----TGGGGGTTAG	142	-5	0.27%
ATCTGCCTAAC---AGGTGGGGGTTAG	133	-3	0.25%
ATCTGCCTAAC------TGGGGGTTAG	127	-6	0.24%

8h

Total	INDEL	Ins	Del	HDR
116032	24.36%	2.88%	8.73%	12.75%

Typical seqs	Reads	Type	pct.
ATCTGCCTAACAGGAGG|TGG**GGG**TTAG	87771	WT	75.64%
ATCTGCCTAAGTTTAAACTACGCGTGGGTTAG	13695	+5 HDR	11.80%
ATCTGCCTAACAGGA--TGGGGGTTAG	1579	-2	1.36%
ATCTGCCTAACAGGAGGgTGGGGGTTAG	1312	+1 g	1.13%
ATCTGCCTAACAGGA-GTGGGGGTTAG	985	-1	0.85%
ATCTGCCTAACAGGA------GGTTAG	625	-6	0.54%
ATCTGCCTAACAGGAGGT-GGGGTTAG	433	-1	0.37%
ATCTGCCTAACA----GTGGGGGTTAG	436	-4	0.38%
ATCTGCCTAACA-----TGGGGGTTAG	426	-5	0.37%
ATCTGCCTAACAGGAGG-GGGGGTTAG	414	-1	0.36%

12h

Total	INDEL	Ins	Del	HDR
71863	26.90%	3.07%	9.60%	14.23%

Typical seqs	Reads	Type	pct.
ATCTGCCTAACAGGAGG|TGG**GGG**TTAG	52535	WT	73.10%
ATCTGCCTAAGTTTAAACTACGCGTGGGTTAG	9411	+5 HDR	13.10%
ATCTGCCTAACAGGA--TGGGGGTTAG	1077	-2	1.50%
ATCTGCCTAACAGGAGGgTGGGGGTTAG	824	+1 g	1.15%
ATCTGCCTAACAGGA-GTGGGGGTTAG	560	-1	0.78%
ATCTGCCTAACAGGA------GGTTAG	509	-6	0.71%
ATCTGCCTAACAGGAGG-GGGGGTTAG	293	-1	0.41%
ATCTGCCTAACA-----TGGGGGTTAG	284	-5	0.40%
ATCTGCCTAACA----GTGGGGGTTAG	264	-4	0.37%
ATCTGCCTAAC------TGGGGGTTAG	258	-6	0.36%

24h

Total	INDEL	Ins	Del	HDR
55441	31.96%	3.07%	12.11%	16.78%

Typical seqs	Reads	Type	pct.
ATCTGCCTAACAGGAGG|TGG**GGG**TTAG	37721	WT	68.04%
ATCTGCCTAAGTTTAAACTACGCGTGGGTTAG	8553	+5 HDR	15.43%
ATCTGCCTAACAGGA--TGGGGGTTAG	1008	-2	1.82%
ATCTGCCTAACAGGA------GGTTAG	626	-6	1.13%
ATCTGCCTAACAGGAGGgTGGGGGTTAG	596	+1 g	1.08%
ATCTGCCTAACAGGA-GTGGGGGTTAG	573	-1	1.03%
ATCTGCCTAACAGGAGG-GGGGGTTAG	258	-1	0.47%
ATCTGCCTAAC------TGGGGGTTAG	243	-6	0.44%
ATCTGCCTAACA-----TGGGGGTTAG	243	-5	0.44%
ATCTGCCTAACA----GTGGGGGTTAG	232	-4	0.42%

48h

Total	INDEL	Ins	Del	HDR
31991	32.47%	3.00%	12.31%	17.15%

Typical seqs	Reads	Type	pct.
ATCTGCCTAACAGGAGG|TGG**GGG**TTAG	21604	WT	67.53%
ATCTGCCTAAGTTTAAACTACGCGTGGGTTAG	5110	+5 HDR	15.97%
ATCTGCCTAACAGGA--TGGGGGTTAG	567	-2	1.77%
ATCTGCCTAACAGGA------GGTTAG	428	-6	1.34%
ATCTGCCTAACAGGAGGgTGGGGGTTAG	371	+1 g	1.16%
ATCTGCCTAACAGGA-GTGGGGGTTAG	307	-1	0.96%
ATCTGCCTAACA-----TGGGGGTTAG	180	-5	0.56%
ATCTGCCTAACA----GTGGGGGTTAG	157	-4	0.49%
ATCTGCCTAACAGGAGG-GGGGGTTAG	148	-1	0.46%
ATCTGCCTAAC------TGGGGGTTAG	122	-6	0.38%

P84-YW-K562-Syn22crAAVS1e-KO

4h

Total | INDEL	Ins	Del	HDR
106003 | 22.54%	13.96%	8.58%	0.00%

Typical seqs	Reads	Type	pct.
AATCTGCCTAACAGGAG|GTGGGGGTTA	82113	WT	77.46%
AATCTGCCTAACAAGGAGGTGGGGGTTA	10682	+1 Ins	10.08%
AATCTGCCTAACAGGAGgGTGGGGGTTA	1777	+1 g	1.68%
AATCTGCCTAACAGGA-GTGGGGGTTA	735	-1	0.69%
AATCTGCCTAACAGGA------GGTTA	721	-6	0.68%
AATCTGCCTAACAGGA--TGGGGGTTA	541	-2	0.51%
AATCTGCCTAAC---AGGTGGGGGTTA	421	-3	0.40%
AATCTGCCTA--AGGAGGTGGGGGTTA	401	-2	0.38%
------------AGGAGGTGGGGGTTA	370	-15	0.35%
AATCTGCCTAACA----GTGGGGGTTA	229	-4	0.22%

8h

Total | INDEL	Ins	Del	HDR
116243 | 24.25%	14.54%	9.71%	0.00%

Typical seqs	Reads	Type	pct.
AATCTGCCTAACAGGAG|GTGGGGGTTA	88058	WT	75.75%
AATCTGCCTAACAAGGAGGTGGGGGTTA	11380	+1 Ins	9.79%
AATCTGCCTAACAGGAGgGTGGGGGTTA	2711	+1 g	2.33%
AATCTGCCTAACAGGA-GTGGGGGTTA	998	-1	0.86%
AATCTGCCTAACAGGA------GGTTA	774	-6	0.67%
AATCTGCCTAACAGGA--TGGGGGTTA	598	-2	0.51%
AATCTGCCTAAC---AGGTGGGGGTTA	597	-3	0.51%
AATCTGCCTA--AGGAGGTGGGGGTTA	456	-2	0.39%
------------AGGAGGTGGGGGTTA	427	-15	0.37%
AATCTGCCTAACA----GTGGGGGTTA	265	-4	0.23%

12h

Total | INDEL	Ins	Del	HDR
79780 | 26.44%	15.57%	10.87%	0.00%

Typical seqs	Reads	Type	pct.
AATCTGCCTAACAGGAG|GTGGGGGTTA	58684	WT	73.56%
AATCTGCCTAACAAGGAGGTGGGGGTTA	8210	+1 Ins	10.29%
AATCTGCCTAACAGGAGgGTGGGGGTTA	2101	+1 g	2.63%
AATCTGCCTAACAGGA-GTGGGGGTTA	700	-1	0.88%
AATCTGCCTAACAGGA------GGTTA	593	-6	0.74%
AATCTGCCTAAC---AGGTGGGGGTTA	534	-3	0.67%
AATCTGCCTAACAGGA--TGGGGGTTA	414	-2	0.52%
------------AGGAGGTGGGGGTTA	335	-15	0.42%
AATCTGCCTA--AGGAGGTGGGGGTTA	276	-2	0.35%
AATCTGCCTAACA----GTGGGGGTTA	183	-4	0.23%

24h

Total | INDEL	Ins	Del	HDR
49299 | 30.62%	16.73%	13.89%	0.00%

Typical seqs	Reads	Type	pct.
AATCTGCCTAACAGGAG|GTGGGGGTTA	34203	WT	69.38%
AATCTGCCTAACAAGGAGGTGGGGGTTA	5248	+1 Ins	10.65%
AATCTGCCTAACAGGAGgGTGGGGGTTA	1578	+1 g	3.20%
AATCTGCCTAACAGGA-GTGGGGGTTA	518	-1	1.05%
AATCTGCCTAACAGGA------GGTTA	377	-6	0.76%
AATCTGCCTAAC---AGGTGGGGGTTA	347	-3	0.70%
AATCTGCCTAACAGGA--TGGGGGTTA	264	-2	0.54%
------------AGGAGGTGGGGGTTA	190	-15	0.39%
AATCTGCCTA--AGGAGGTGGGGGTTA	168	-2	0.34%
AATCTGCCTAACA----GTGGGGGTTA	166	-4	0.34%

48h

Total | INDEL	Ins	Del	HDR
40420 | 32.21%	16.95%	15.25%	0.00%

Typical seqs	Reads	Type	pct.
AATCTGCCTAACAGGAG|GTGGGGGTTA	27402	WT	67.79%
AATCTGCCTAACAAGGAGGTGGGGGTTA	4313	+1 Ins	10.67%
AATCTGCCTAACAGGAGgGTGGGGGTTA	1323	+1 g	3.27%
AATCTGCCTAACAGGA-GTGGGGGTTA	445	-1	1.10%
AATCTGCCTAACAGGA------GGTTA	361	-6	0.89%
AATCTGCCTAAC---AGGTGGGGGTTA	327	-3	0.81%
AATCTGCCTAACAGGA--TGGGGGTTA	228	-2	0.56%
AATCTG---------------------	249	-23	0.62%
------------AGGAGGTGGGGGTTA	173	-15	0.43%
AATCTGCCTA--AGGAGGTGGGGGTTA	146	-2	0.36%

P85-YW-K562-Syn22crAAVS1e-AAV6KI

Total | INDEL	4h	Ins	Del	HDR
106098 | 28.21%		11.28%	6.29%	10.63%

Typical seqs	Reads	Type	pct.
AATCTGCCTAACAGGAG|GTG**GGG**GTTA	76171	WT	71.79%
AATCTGCCTAAGTTTAAACTACGCGTGGGT TA	10331	+5 HDR	9.74%
AATCTGCCTAACAAGGAGGTGGGGGTTA	7798	+1 Ins	7.35%
AATCTGCCTAACAGGAGgGTGGGGGTTA	1674	+1 g	1.58%
AATCTGCCTAACAGGA-GTGGGGGTTA	718	-1	0.68%
AATCTGCCTAACAGGA--TGGGGGTTA	630	-2	0.59%
AATCTGCCTAAC---AGGTGGGGGTTA	400	-3	0.38%
AATCTGCCTAACAGGA------GGTTA	346	-6	0.33%
AATCTGCCTA--AGGAGGTGGGGGTTA	306	-2	0.29%
AATCTGCCTAACA-----TGGGGGTTA	205	-5	0.19%

Total | INDEL	8h	Ins	Del	HDR
91973 | 31.61%		12.02%	7.06%	12.54%

Typical seqs	Reads	Type	pct.
AATCTGCCTAACAGGAG|GTG**GGG**GTTA	62896	WT	68.39%
AATCTGCCTAAGTTTAAACTACGCGTGGGT TA	10580	+5 HDR	11.50%
AATCTGCCTAACAAGGAGGTGGGGGTTA	6595	+1 Ins	7.17%
AATCTGCCTAACAGGAGgGTGGGGGTTA	1991	+1 g	2.16%
AATCTGCCTAACAGGA-GTGGGGGTTA	735	-1	0.80%
AATCTGCCTAACAGGA--TGGGGGTTA	484	-2	0.53%
AATCTGCCTAAC---AGGTGGGGGTTA	378	-3	0.41%
AATCTGCCTAACAGGA------GGTTA	303	-6	0.33%
AATCTGCCTA--AGGAGGTGGGGGTTA	274	-2	0.30%
AATCTGCCTAACA----GTGGGGGTTA	200	-4	0.22%

Total | INDEL	12h	Ins	Del	HDR
45968 | 33.36%		12.09%	7.44%	13.83%

Typical seqs	Reads	Type	pct.
AATCTGCCTAACAGGAG|GTG**GGG**GTTA	30632	WT	66.64%
AATCTGCCTAAGTTTAAACTACGCGTGGGT TA	5858	+5 HDR	12.74%
AATCTGCCTAACAAGGAGGTGGGGGTTA	3289	+1 Ins	7.15%
AATCTGCCTAACAGGAGgGTGGGGGTTA	980	+1 g	2.13%
AATCTGCCTAACAGGA-GTGGGGGTTA	412	-1	0.90%
AATCTGCCTAACAGGA--TGGGGGTTA	268	-2	0.58%
AATCTGCCTAAC---AGGTGGGGGTTA	211	-3	0.46%
AATCTGCCTAACAGGA------GGTTA	200	-6	0.44%
AATCTGCCTA--AGGAGGTGGGGGTTA	139	-2	0.30%
AATCTGCCTAACA----GTGGGGGTTA	108	-4	0.23%

Total | INDEL	24h	Ins	Del	HDR
23619 | 37.14%		12.79%	8.62%	15.74%

Typical seqs	Reads	Type	pct.
AATCTGCCTAACAGGAG|GTG**GGG**GTTA	14846	WT	62.86%
AATCTGCCTAAGTTTAAACTACGCGTGGGT TA	3471	+5 HDR	14.70%
AATCTGCCTAACAAGGAGGTGGGGGTTA	1748	+1 Ins	7.40%
AATCTGCCTAACAGGAGgGTGGGGGTTA	623	+1 g	2.64%
AATCTGCCTAACAGGA-GTGGGGGTTA	225	-1	0.95%
AATCTGCCTAACAGGA--TGGGGGTTA	146	-2	0.62%
AATCTGCCTAAC---AGGTGGGGGTTA	144	-3	0.61%
AATCTGCCTAACAGGA------GGTTA	77	-6	0.33%
AATCTGCCTA--AGGAGGTGGGGGTTA	61	-2	0.26%
AATCTGCCTAACA----GTGGGGGTTA	60	-4	0.25%

Total | INDEL	48h	Ins	Del	HDR
19837 | 37.72%		12.59%	8.61%	16.52%

Typical seqs	Reads	Type	pct.
AATCTGCCTAACAGGAG|GTG**GGG**GTTA	12355	WT	62.28%
AATCTGCCTAAGTTTAAACTACGCGTGGGT TA	3071	+5 HDR	15.48%
AATCTGCCTAACAAGGAGGTGGGGGTTA	1457	+1 Ins	7.34%
AATCTGCCTAACAGGAGgGTGGGGGTTA	500	+1 g	2.52%
AATCTGCCTAACAGGA-GTGGGGGTTA	209	-1	1.05%
AATCTGCCTAACAGGA--TGGGGGTTA	116	-2	0.58%
AATCTGCCTAACAGGA------GGTTA	89	-6	0.45%
AATCTGCCTAAC---AGGTGGGGGTTA	86	-3	0.43%
AATCTGCCTAACA----GTGGGGGTTA	58	-4	0.29%
AATCTGCCTA--AGGAGGTGGGGGTTA	56	-2	0.28%

P86-YW-K562-Syn23crAAVS1f-KO

4h

Total | INDEL	Ins	Del	HDR
6859 | 29.67%	6.24%	23.20%	0.23%

Typical seqs	Reads	Type	pct.
GAATCTGCCTAACAGGA|GGTGGGGGTT	4824	WT	70.33%
GAATCTGCCTAAC---AGGTGGGGGTT	429	-3	6.25%
GAATCTGCCTAACCAGGAGGTGGGGGTT	315	+1 Ins	4.59%
-------------AGGAGGTGGGGGTT	143	-15	2.08%
GAATCTGCCTA--AGGAGGTGGGGGTT	81	-2	1.18%
GAATCTGCCTAA-AGGAGGTGGGGGTT	64	-1	0.93%
GAATCTGCCTAACA--AGGTGGGGGTT	46	-2	0.67%
GAATCTGCCTAACA-GAGGTGGGGGTT	44	-1	0.64%
GAATCTGCCTAACAGGAaGGTGGGGGTT	44	+1 a	0.64%
GAATCTGCCTAAC--GAGGTGGGGGTT	36	-2	0.52%

8h

Total | INDEL	Ins	Del	HDR
25919 | 31.71%	6.75%	24.82%	0.14%

Typical seqs	Reads	Type	pct.
GAATCTGCCTAACAGGA|GGTGGGGGTT	17700	WT	68.29%
GAATCTGCCTAAC---AGGTGGGGGTT	1684	-3	6.50%
GAATCTGCCTAACCAGGAGGTGGGGGTT	1237	+1 Ins	4.77%
-------------AGGAGGTGGGGGTT	469	-15	1.81%
GAATCTGCCTA--AGGAGGTGGGGGTT	300	-2	1.16%
GAATCTGCCTAACAGGAaGGTGGGGGTT	173	+1 a	0.67%
GAATCTGCCTAA-AGGAGGTGGGGGTT	179	-1	0.69%
GAATCTGCCTAACA--AGGTGGGGGTT	171	-2	0.66%
GAATCTGCCTAACA-GAGGTGGGGGTT	150	-1	0.58%
GAATCTGCCTAAC--GAGGTGGGGGTT	134	-2	0.52%

12h

Total | INDEL	Ins	Del	HDR
73651 | 33.29%	7.14%	26.01%	0.14%

Typical seqs	Reads	Type	pct.
GAATCTGCCTAACAGGA|GGTGGGGGTT	49135	WT	66.71%
GAATCTGCCTAAC---AGGTGGGGGTT	4713	-3	6.40%
GAATCTGCCTAACCAGGAGGTGGGGGTT	3541	+1 Ins	4.81%
-------------AGGAGGTGGGGGTT	1357	-15	1.84%
GAATCTGCCTA--AGGAGGTGGGGGTT	832	-2	1.13%
GAATCTGCCTAACA--AGGTGGGGGTT	533	-2	0.72%
GAATCTGCCTAA-AGGAGGTGGGGGTT	523	-1	0.71%
GAATCTGCCTAACAGGAaGGTGGGGGTT	495	+1 a	0.67%
GAATCTGCCTAACA-GAGGTGGGGGTT	437	-1	0.59%
GAATCTGCCTAAC--GAGGTGGGGGTT	351	-2	0.48%

24h

Total | INDEL	Ins	Del	HDR
35864 | 35.36%	7.07%	28.16%	0.13%

Typical seqs	Reads	Type	pct.
GAATCTGCCTAACAGGA|GGTGGGGGTT	23184	WT	64.64%
GAATCTGCCTAAC---AGGTGGGGGTT	2735	-3	7.63%
GAATCTGCCTAACCAGGAGGTGGGGGTT	1748	+1 Ins	4.87%
-------------AGGAGGTGGGGGTT	732	-15	2.04%
GAATCTGCCTA--AGGAGGTGGGGGTT	415	-2	1.16%
GAATCTGCCTAACAGGAaGGTGGGGGTT	275	+1 a	0.77%
GAATCTGCCTAACA--AGGTGGGGGTT	250	-2	0.70%
GAATCTGCCTAACA-GAGGTGGGGGTT	229	-1	0.64%
GAATCTGCCTAAC--GAGGTGGGGGTT	215	-2	0.60%
GAATCTGCCTAA-AGGAGGTGGGGGTT	203	-1	0.57%

48h

Total | INDEL	Ins	Del	HDR
29875 | 35.38%	6.61%	28.60%	0.16%

Typical seqs	Reads	Type	pct.
GAATCTGCCTAACAGGA|GGTGGGGGTT	19306	WT	64.62%
GAATCTGCCTAAC---AGGTGGGGGTT	2293	-3	7.68%
GAATCTGCCTAACCAGGAGGTGGGGGTT	1326	+1 Ins	4.44%
-------------AGGAGGTGGGGGTT	639	-15	2.14%
GAATCTGCCTA--AGGAGGTGGGGGTT	353	-2	1.18%
GAATCTGCCTAACA--AGGTGGGGGTT	198	-2	0.66%
GAATCTGCCTAACAGGAaGGTGGGGGTT	201	+1 a	0.67%
GAATCTGCCTAACA-GAGGTGGGGGTT	181	-1	0.61%
GAATCTGCCTAA-AGGAGGTGGGGGTT	180	-1	0.60%
GAATCTGCCTAAC--GAGGTGGGGGTT	171	-2	0.57%

P87-YW-K562-Syn23crAAVS1f-AAV6KI

Total	INDEL	4h	Ins	Del	HDR
42548	23.34%		4.17%	7.26%	11.91%

Typical seqs	Reads	Type	pct.
GAATCTGCCTAACAGGA|GGTGGGGGTT	32617	WT	76.66%
GAATCTGCCTAAGTTTAAACTACGCGTGGG TT	4730	+5 HDR	11.12%
GAATCTGCCTAAC---AGGTGGGGGTT	649	-3	1.53%
GAATCTGCCTAACCAGGAGGTGGGGGTT	612	+1 Ins	1.44%
GAATCTGCCTAACAGGAaGGTGGGGGTT	271	+1 a	0.64%
GAATCTGCCTAACAAGGAGGTGGGGGTT	241	+1 Ins	0.57%
GAATCTGCCTA--AGGAGGTGGGGGTT	143	-2	0.34%
GAATCTGCCTAACA--AGGTGGGGGTT	104	-2	0.24%
GAATCTGCCTAACAGGA-GTGGGGGTT	100	-1	0.24%
GAATCTGCCTAACA-GAGGTGGGGGTT	94	-1	0.22%

Total	INDEL	8h	Ins	Del	HDR
48601	32.90%		5.19%	9.15%	18.56%

Typical seqs	Reads	Type	pct.
GAATCTGCCTAACAGGA|GGTGGGGGTT	32612	WT	67.10%
GAATCTGCCTAAGTTTAAACTACGCGTGGG TT	8305	+5 HDR	17.09%
GAATCTGCCTAAC---AGGTGGGGGTT	1119	-3	2.30%
GAATCTGCCTAACCAGGAGGTGGGGGTT	723	+1 Ins	1.49%
GAATCTGCCTAACAGGAaGGTGGGGGTT	508	+1 a	1.05%
GAATCTGCCTAACAAGGAGGTGGGGGTT	281	+1 Ins	0.58%
GAATCTGCCTA--AGGAGGTGGGGGTT	196	-2	0.40%
GAATCTGCCTAACA--AGGTGGGGGTT	140	-2	0.29%
GAATCTGCCTAACAGGA-GTGGGGGTT	139	-1	0.29%
GAATCTGCCTAACAG--GGTGGGGGTT	139	-2	0.29%

Total	INDEL	12h	Ins	Del	HDR
93283	38.44%		5.76%	11.23%	21.45%

Typical seqs	Reads	Type	pct.
GAATCTGCCTAACAGGA|GGTGGGGGTT	57424	WT	61.56%
GAATCTGCCTAAGTTTAAACTACGCGTGGG TT	18276	+5 HDR	19.59%
GAATCTGCCTAAC---AGGTGGGGGTT	2724	-3	2.92%
GAATCTGCCTAACCAGGAGGTGGGGGTT	1269	+1 Ins	1.36%
GAATCTGCCTAACAGGAaGGTGGGGGTT	1193	+1 a	1.28%
GAATCTGCCTAACAAGGAGGTGGGGGTT	485	+1 Ins	0.52%
GAATCTGCCTAACAGGA-GTGGGGGTT	399	-1	0.43%
GAATCTGCCTAACAG--GGTGGGGGTT	338	-2	0.36%
GAATCTGCCTAACAGG-GGTGGGGGTT	312	-1	0.33%
GAATCTGCCTA--AGGAGGTGGGGGTT	304	-2	0.33%

Total	INDEL	24h	Ins	Del	HDR
44231	49.75%		6.68%	15.24%	27.82%

Typical seqs	Reads	Type	pct.
GAATCTGCCTAACAGGA|GGTGGGGGTT	22227	WT	50.25%
GAATCTGCCTAAGTTTAAACTACGCGTGGG TT	11298	+5 HDR	25.54%
GAATCTGCCTAAC---AGGTGGGGGTT	2054	-3	4.64%
GAATCTGCCTAACAGGAaGGTGGGGGTT	650	+1 a	1.47%
GAATCTGCCTAACCAGGAGGTGGGGGTT	655	+1 Ins	1.48%
GAATCTGCCTAACAG--GGTGGGGGTT	193	-2	0.44%
GAATCTGCCTA--AGGAGGTGGGGGTT	175	-2	0.40%
GAATCTGCCTAACAGG-GGTGGGGGTT	170	-1	0.38%
GAATCTGCCTAACAAGGAGGTGGGGGTT	164	+1 Ins	0.37%
GAATCTGCCTAACAGGA-GTGGGGGTT	146	-1	0.33%

Total	INDEL	48h	Ins	Del	HDR
29908	54.02%		6.66%	17.48%	29.88%

Typical seqs	Reads	Type	pct.
GAATCTGCCTAACAGGA|GGTGGGGGTT	13751	WT	45.98%
GAATCTGCCTAAGTTTAAACTACGCGTGGG TT	8243	+5 HDR	27.56%
GAATCTGCCTAAC---AGGTGGGGGTT	1496	-3	5.00%
GAATCTGCCTAACAGGAaGGTGGGGGTT	422	+1 a	1.41%
GAATCTGCCTAACCAGGAGGTGGGGGTT	412	+1 Ins	1.38%
GAATCTGCCTAACAG--GGTGGGGGTT	160	-2	0.53%
GAATCTGCCTAACAAGGAGGTGGGGGTT	141	+1 Ins	0.47%
GAATCTGCCTAACAGG-GGTGGGGGTT	129	-1	0.43%
GAATCTGCCTAACAGGA-GTGGGGGTT	118	-1	0.39%
GAATCTGCCTA-----AGGTGGGGGTT	106	-5	0.35%

P86-YW-K562-Syn24crAAVS1g-KO

4h

Total | INDEL	Ins	Del	HDR
33088 | 32.14%	6.76%	25.26%	0.12%

Typical seqs	Reads	Type	pct.
GGAATCTGCCTAACAGG|AGG**TGG**GGGT	22452	WT	67.86%
GGAATCTGCCTAAC---AGGTGGGGGT	2156	-3	6.52%
GGAATCTGCCTAACCAGGAGGTGGGGGT	1566	+1 Ins	4.73%
--------------AGGAGGTGGGGGT	629	-15	1.90%
GGAATCTGCCTA--AGGAGGTGGGGGT	424	-2	1.28%
GGAATCTGCCTAACA--AGGTGGGGGT	220	-2	0.66%
GGAATCTGCCTAA-AGGAGGTGGGGGT	216	-1	0.65%
GGAATCTGCCTAACA-GAGGTGGGGGT	186	-1	0.56%
GGAATCTGCCTAACAGGaAGGTGGGGGT	194	+1 a	0.59%
GGAATCTGCCTAAC--GAGGTGGGGGT	156	-2	0.47%

8h

Total | INDEL	Ins	Del	HDR
110479 | 34.41%	7.01%	27.29%	0.11%

Typical seqs	Reads	Type	pct.
GGAATCTGCCTAACAGG|AGG**TGG**GGGT	72464	WT	65.59%
GGAATCTGCCTAAC---AGGTGGGGGT	7144	-3	6.47%
GGAATCTGCCTAACCAGGAGGTGGGGGT	5228	+1 Ins	4.73%
--------------AGGAGGTGGGGGT	2169	-15	1.96%
GGAATCTGCCTA--AGGAGGTGGGGGT	1380	-2	1.25%
GGAATCTGCCTAACA--AGGTGGGGGT	855	-2	0.77%
GGAATCTGCCTAA-AGGAGGTGGGGGT	772	-1	0.70%
GGAATCTGCCTAACA-GAGGTGGGGGT	758	-1	0.69%
GGAATCTGCCTAACAGGaAGGTGGGGGT	641	+1 a	0.58%
GGAATCTGCCTAAC--GAGGTGGGGGT	606	-2	0.55%

12h

Total | INDEL	Ins	Del	HDR
78812 | 34.86%	6.80%	27.94%	0.12%

Typical seqs	Reads	Type	pct.
GGAATCTGCCTAACAGG|AGG**TGG**GGGT	51337	WT	65.14%
GGAATCTGCCTAAC---AGGTGGGGGT	5507	-3	6.99%
GGAATCTGCCTAACCAGGAGGTGGGGGT	3687	+1 Ins	4.68%
--------------AGGAGGTGGGGGT	1590	-15	2.02%
GGAATCTGCCTA--AGGAGGTGGGGGT	990	-2	1.26%
GGAATCTGCCTAACA--AGGTGGGGGT	667	-2	0.85%
GGAATCTGCCTAA-AGGAGGTGGGGGT	605	-1	0.77%
GGAATCTGCCTAACA-GAGGTGGGGGT	519	-1	0.66%
GGAATCTGCCTAACAGGaAGGTGGGGGT	446	+1 a	0.57%
GGAATCTGCCTAAC--GAGGTGGGGGT	425	-2	0.54%

24h

Total | INDEL	Ins	Del	HDR
75280 | 37.24%	6.80%	30.29%	0.15%

Typical seqs	Reads	Type	pct.
GGAATCTGCCTAACAGG|AGG**TGG**GGGT	47244	WT	62.76%
GGAATCTGCCTAAC---AGGTGGGGGT	5917	-3	7.86%
GGAATCTGCCTAACCAGGAGGTGGGGGT	3390	+1 Ins	4.50%
--------------AGGAGGTGGGGGT	1472	-15	1.96%
GGAATCTGCCTA--AGGAGGTGGGGGT	912	-2	1.21%
GGAATCTGCCTAACA--AGGTGGGGGT	670	-2	0.89%
GGAATCTGCCTAACA-GAGGTGGGGGT	613	-1	0.81%
GGAATCTGCCTAA-AGGAGGTGGGGGT	532	-1	0.71%
GGAATCTGCCTAACAGGaAGGTGGGGGT	458	+1 a	0.61%
GGAATCTGCCTAAC--GAGGTGGGGGT	410	-2	0.54%

48h

Total | INDEL	Ins	Del	HDR
40742 | 37.91%	6.54%	31.27%	0.10%

Typical seqs	Reads	Type	pct.
GGAATCTGCCTAACAGG|AGG**TGG**GGGT	25298	WT	62.09%
GGAATCTGCCTAAC---AGGTGGGGGT	3479	-3	8.54%
GGAATCTGCCTAACCAGGAGGTGGGGGT	1809	+1 Ins	4.44%
--------------AGGAGGTGGGGGT	809	-15	1.99%
GGAATCTGCCTA--AGGAGGTGGGGGT	521	-2	1.28%
GGAATCTGCCTAACA--AGGTGGGGGT	353	-2	0.87%
GGAATCTGCCTAACA-GAGGTGGGGGT	290	-1	0.71%
GGAATCTGCCTAA-AGGAGGTGGGGGT	259	-1	0.64%
GGAATCTGCCTAAC--GAGGTGGGGGT	247	-2	0.61%
GGAATCTGCCTA-----AGGTGGGGGT	216	-5	0.53%

P87-YW-K562-Syn24crAAVS1g-AAV6KI

4h

Total | INDEL		Ins	Del	HDR
75892 | 25.12%	4h	3.82%	8.77%	12.54%

Typical seqs	Reads	Type	pct.
GGAATCTGCCTAACAGG|AGG**TGG**GGGT	56825	WT	74.88%
GGAATCTGCCTAAGTTTAAACTACGCGTGG GT	8821	+5 HDR	11.62%
GGAATCTGCCTAAC---AGGTGGGGGT	1526	-3	2.01%
GGAATCTGCCTAACCAGGAGGTGGGGGT	1233	+1 Ins	1.62%
GGAATCTGCCTAACA-GAGGTGGGGGT	419	-1	0.55%
GGAATCTGCCTAACA--AGGTGGGGGT	363	-2	0.48%
GGAATCTGCCTA--AGGAGGTGGGGGT	252	-2	0.33%
GGAATCTGCCTAACAGGaAGGTGGGGGT	243	+1 a	0.32%
GGAATCTGCCTAACAGG-GGTGGGGGT	190	-1	0.25%
GGAATCTGCCTA-----AGGTGGGGGT	178	-5	0.23%

8h

Total | INDEL		Ins	Del	HDR
145254 | 36.59%	8h	4.38%	12.64%	19.56%

Typical seqs	Reads	Type	pct.
GGAATCTGCCTAACAGG|AGG**TGG**GGGT	92109	WT	63.41%
GGAATCTGCCTAAGTTTAAACTACGCGTGG GT	26351	+5 HDR	18.14%
GGAATCTGCCTAAC---AGGTGGGGGT	4063	-3	2.80%
GGAATCTGCCTAACCAGGAGGTGGGGGT	2059	+1 Ins	1.42%
GGAATCTGCCTAACA--AGGTGGGGGT	1436	-2	0.99%
GGAATCTGCCTAACA-GAGGTGGGGGT	1241	-1	0.85%
GGAATCTGCCTA-----AGGTGGGGGT	651	-5	0.45%
GGAATCTGCCTAACAGGgAGGTGGGGGT	565	+1 g	0.39%
GGAATCTGCCTAA----AGGTGGGGGT	575	-4	0.40%
GGAATCTGCCTA--AGGAGGTGGGGGT	513	-2	0.35%

12h

Total | INDEL		Ins	Del	HDR
79792 | 43.44%	12h	4.69%	15.13%	23.62%

Typical seqs	Reads	Type	pct.
GGAATCTGCCTAACAGG|AGG**TGG**GGGT	45132	WT	56.56%
GGAATCTGCCTAAGTTTAAACTACGCGTGG GT	17426	+5 HDR	21.84%
GGAATCTGCCTAAC---AGGTGGGGGT	2991	-3	3.75%
GGAATCTGCCTAACCAGGAGGTGGGGGT	1173	+1 Ins	1.47%
GGAATCTGCCTAACA-GAGGTGGGGGT	849	-1	1.06%
GGAATCTGCCTAACA--AGGTGGGGGT	733	-2	0.92%
GGAATCTGCCTA-----AGGTGGGGGT	449	-5	0.56%
GGAATCTGCCTAA----AGGTGGGGGT	402	-4	0.50%
GGAATCTGCCTAACAGGgAGGTGGGGGT	325	+1 g	0.41%
GGAATCTGCCTAACAGG-GGTGGGGGT	320	-1	0.40%

24h

Total | INDEL		Ins	Del	HDR
93831 | 57.10%	24h	5.17%	21.43%	30.50%

Typical seqs	Reads	Type	pct.
GGAATCTGCCTAACAGG|AGG**TGG**GGGT	40258	WT	42.90%
GGAATCTGCCTAAGTTTAAACTACGCGTGG GT	26293	+5 HDR	28.02%
GGAATCTGCCTAAC---AGGTGGGGGT	5856	-3	6.24%
GGAATCTGCCTAACCAGGAGGTGGGGGT	1314	+1 Ins	1.40%
GGAATCTGCCTAACA--AGGTGGGGGT	1317	-2	1.40%
GGAATCTGCCTAACA-GAGGTGGGGGT	1002	-1	1.07%
GGAATCTGCCTAA----AGGTGGGGGT	773	-4	0.82%
GGAATCTGCCTAACAGG-GGTGGGGGT	521	-1	0.56%
GGAATCTGCCTA-----AGGTGGGGGT	509	-5	0.54%
GGAATCTGCCTAACAG--GGTGGGGGT	492	-2	0.52%

48h

Total | INDEL		Ins	Del	HDR
32904 | 61.59%	48h	4.84%	24.35%	32.40%

Typical seqs	Reads	Type	pct.
GGAATCTGCCTAACAGG|AGG**TGG**GGGT	12639	WT	38.41%
GGAATCTGCCTAAGTTTAAACTACGCGTGG GT	9914	+5 HDR	30.13%
GGAATCTGCCTAAC---AGGTGGGGGT	2615	-3	7.95%
GGAATCTGCCTAACA--AGGTGGGGGT	447	-2	1.36%
GGAATCTGCCTAACCAGGAGGTGGGGGT	439	+1 Ins	1.33%
GGAATCTGCCTAACA-GAGGTGGGGGT	406	-1	1.23%
GGAATCTGCCTAA----AGGTGGGGGT	245	-4	0.74%
GGAATCTGCCTA-----AGGTGGGGGT	201	-5	0.61%
GGAATCTGCCTAACAG--GGTGGGGGT	178	-2	0.54%
GGAATCTGCCT------AGGTGGGGGT	160	-6	0.49%

P86-YW-K562-Syn25crAAVS1h-KO

4h

Total | INDEL	Ins	Del	HDR
83465 | 33.70%	7.25%	26.31%	0.14%

Typical seqs	Reads	Type	pct.
TAAGGAATCTGCCTAAC|AGG**AGG**TGGG	55339	WT	66.30%
TAAGGAATCTGCCTAAC---AGGTGGG	5496	-3	6.58%
TAAGGAATCTGCCTAACcAGGAGGTGGG	4243	+1 c	5.08%
TA---------------AGGAGGTGGG	1690	-15	2.02%
TAAGGAATCTGCCTA--AGGAGGTGGG	1119	-2	1.34%
TAAGGAATCTGCCTAA-AGGAGGTGGG	644	-1	0.77%
TAAGGAATCTGCCTAACA--AGGTGGG	546	-2	0.65%
TAAGGAATCTGCCTAACaGGAAGGTGGG	528	+1 a	0.63%
TAAGGAATCTGCCTAACA-GAGGTGGG	491	-1	0.59%
TAAGGAATCTGCCTAAC--GAGGTGGG	468	-2	0.56%

8h

Total | INDEL	Ins	Del	HDR
77131 | 35.41%	7.86%	27.40%	0.15%

Typical seqs	Reads	Type	pct.
TAAGGAATCTGCCTAAC|AGG**AGG**TGGG	49818	WT	64.59%
TAAGGAATCTGCCTAAC---AGGTGGG	4950	-3	6.42%
TAAGGAATCTGCCTAACcAGGAGGTGGG	4419	+1 c	5.73%
TA---------------AGGAGGTGGG	1524	-15	1.98%
TAAGGAATCTGCCTA--AGGAGGTGGG	1072	-2	1.39%
TAAGGAATCTGCCTAA-AGGAGGTGGG	751	-1	0.97%
TAAGGAATCTGCCTAACA--AGGTGGG	592	-2	0.77%
TAAGGAATCTGCCTAACA-GAGGTGGG	475	-1	0.62%
TAAGGAATCTGCCTAAC--GAGGTGGG	447	-2	0.58%
TAAGGAATCTGCCTAACaGGAAGGTGGG	478	+1 a	0.62%

12h

Total | INDEL	Ins	Del	HDR
65743 | 36.39%	8.37%	27.88%	0.14%

Typical seqs	Reads	Type	pct.
TAAGGAATCTGCCTAAC|AGG**AGG**TGGG	41818	WT	63.61%
TAAGGAATCTGCCTAAC---AGGTGGG	4326	-3	6.58%
TAAGGAATCTGCCTAACcAGGAGGTGGG	4065	+1 c	6.18%
TA---------------AGGAGGTGGG	1457	-15	2.22%
TAAGGAATCTGCCTA--AGGAGGTGGG	985	-2	1.50%
TAAGGAATCTGCCTAA-AGGAGGTGGG	546	-1	0.83%
TAAGGAATCTGCCTAACA--AGGTGGG	459	-2	0.70%
TAAGGAATCTGCCTAAC--GAGGTGGG	439	-2	0.67%
TAAGGAATCTGCCTAACA-GAGGTGGG	406	-1	0.62%
TAAGGAATCTGCCTAACaGGAAGGTGGG	378	+1 a	0.57%

24h

Total | INDEL	Ins	Del	HDR
34278 | 37.84%	8.18%	29.51%	0.15%

Typical seqs	Reads	Type	pct.
TAAGGAATCTGCCTAAC|AGG**AGG**TGGG	21306	WT	62.16%
TAAGGAATCTGCCTAAC---AGGTGGG	2331	-3	6.80%
TAAGGAATCTGCCTAACcAGGAGGTGGG	2115	+1 c	6.17%
TA---------------AGGAGGTGGG	922	-15	2.69%
TAAGGAATCTGCCTA--AGGAGGTGGG	617	-2	1.80%
TAAGGAATCTGCCTAA-AGGAGGTGGG	322	-1	0.94%
TAAGGAATCTGCCTAAC--GAGGTGGG	246	-2	0.72%
TAAGGAATCTGCCTAACA--AGGTGGG	227	-2	0.66%
TAAGGAATCTGCCTAACA-GAGGTGGG	222	-1	0.65%
TAAGGAATCTGCCTAACaGGAAGGTGGG	195	+1 a	0.57%

48h

Total | INDEL	Ins	Del	HDR
28291 | 38.04%	8.28%	29.61%	0.15%

Typical seqs	Reads	Type	pct.
TAAGGAATCTGCCTAAC|AGG**AGG**TGGG	17528	WT	61.96%
TAAGGAATCTGCCTAAC---AGGTGGG	1883	-3	6.66%
TAAGGAATCTGCCTAACcAGGAGGTGGG	1712	+1 c	6.05%
TA---------------AGGAGGTGGG	857	-15	3.03%
TAAGGAATCTGCCTA--AGGAGGTGGG	478	-2	1.69%
TAAGGAATCTGCCTAA-AGGAGGTGGG	231	-1	0.82%
TAAGGAATCTGCCTAACA--AGGTGGG	205	-2	0.72%
TAAGGAATCTGCCTAACaGGAAGGTGGG	174	+1 a	0.62%
TAAGGAATCTGCCTAAC--GAGGTGGG	183	-2	0.65%
TAAGGAATCTGCCTAACA-GAGGTGGG	145	-1	0.51%

P87-YW-K562-Syn25crAAVS1h-AAV6KI

Total | INDEL	4h	Ins	Del	HDR
136981 | 32.28%		7.05%	10.01%	15.21%

Typical seqs	Reads	Type	pct.
TAAGGAATCTGCCTAAC|AGG**AGG**TGGG	92768	WT	67.72%
TAAGGAATCTGCCTAAGTTTAAACTACGCG TG	19172	+5 HDR	14.00%
TAAGGAATCTGCCTAACcAGGAGGTGGG	6223	+1 c	4.54%
TAAGGAATCTGCCTAAC---AGGTGGG	2019	-3	1.47%
TAAGGAATCTGCCTA--AGGAGGTGGG	1393	-2	1.02%
TAAGGAATCTGCCTAA-AGGAGGTGGG	747	-1	0.55%
TAAGGAATCTGCCTAAC--GAGGTGGG	535	-2	0.39%
TAAGGAATCTGCCT-ACAGGAGGTGGG	541	-1	0.39%
TAAGGAATCTG-----CAGGAGGTGGG	508	-5	0.37%
TAAGGAATCTGCCTAAC-GGAGGTGGG	488	-1	0.36%

Total | INDEL	8h	Ins	Del	HDR
97202 | 55.26%		10.74%	14.40%	30.11%

Typical seqs	Reads	Type	pct.
TAAGGAATCTGCCTAAC|AGG**AGG**TGGG	43492	WT	44.74%
TAAGGAATCTGCCTAAGTTTAAACTACGCG TG	26970	+5 HDR	27.75%
TAAGGAATCTGCCTAACcAGGAGGTGGG	6596	+1 c	6.79%
TAAGGAATCTGCCTA--AGGAGGTGGG	1628	-2	1.67%
TAAGGAATCTGCCTAAC---AGGTGGG	1365	-3	1.40%
TAAGGAATCTGCCTAA-AGGAGGTGGG	1049	-1	1.08%
TA---------------AGGAGGTGGG	705	-15	0.73%
TAAGGAATCTGCCTAAC--GAGGTGGG	630	-2	0.65%
TAAGGAATCTG-----CAGGAGGTGGG	608	-5	0.63%
TAAGGAATCTGCCTAAC-GGAGGTGGG	595	-1	0.61%

Total | INDEL	12h	Ins	Del	HDR
57374 | 61.58%		11.82%	15.99%	33.76%

Typical seqs	Reads	Type	pct.
TAAGGAATCTGCCTAAC|AGG**AGG**TGGG	22044	WT	38.42%
TAAGGAATCTGCCTAAGTTTAAACTACGCG TG	17858	+5 HDR	31.13%
TAAGGAATCTGCCTAACcAGGAGGTGGG	4379	+1 c	7.63%
TAAGGAATCTGCCTA--AGGAGGTGGG	1065	-2	1.86%
TAAGGAATCTGCCTAAC---AGGTGGG	775	-3	1.35%
TAAGGAATCTGCCTAA-AGGAGGTGGG	582	-1	1.01%
TA---------------AGGAGGTGGG	583	-15	1.02%
TAAGGAATCTGCCTAAC--GAGGTGGG	519	-2	0.90%
TAAGGAATCTG-----CAGGAGGTGGG	371	-5	0.65%
TAAGGAATCTGC----CAGGAGGTGGG	361	-4	0.63%

Total | INDEL	24h	Ins	Del	HDR
41417 | 68.30%		11.53%	17.23%	39.54%

Typical seqs	Reads	Type	pct.
TAAGGAATCTGCCTAAC|AGG**AGG**TGGG	13128	WT	31.70%
TAAGGAATCTGCCTAAGTTTAAACTACGCG TG	15078	+5 HDR	36.41%
TAAGGAATCTGCCTAACcAGGAGGTGGG	2830	+1 c	6.83%
TAAGGAATCTGCCTA--AGGAGGTGGG	711	-2	1.72%
TAAGGAATCTGCCTAAC---AGGTGGG	630	-3	1.52%
TA---------------AGGAGGTGGG	606	-15	1.46%
TAAGGAATCTGCCTAA-AGGAGGTGGG	410	-1	0.99%
TAAGGAATCTGCCTAAC--GAGGTGGG	386	-2	0.93%
TAAGGAATCTGCCT-ACAGGAGGTGGG	258	-1	0.62%
TAAGGAATCTGCCTAAC-GGAGGTGGG	273	-1	0.66%

Total | INDEL	48h	Ins	Del	HDR
35190 | 70.81%		11.36%	18.18%	41.26%

Typical seqs	Reads	Type	pct.
TAAGGAATCTGCCTAAC|AGG**AGG**TGGG	10273	WT	29.19%
TAAGGAATCTGCCTAAGTTTAAACTACGCG TG	13438	+5 HDR	38.19%
TAAGGAATCTGCCTAACcAGGAGGTGGG	2417	+1 c	6.87%
TAAGGAATCTGCCTA--AGGAGGTGGG	683	-2	1.94%
TA---------------AGGAGGTGGG	528	-15	1.50%
TAAGGAATCTGCCTAAC---AGGTGGG	477	-3	1.36%
TAAGGAATCTGCCTAA-AGGAGGTGGG	440	-1	1.25%
TAAGGAATCTGCCTAAC--GAGGTGGG	329	-2	0.93%
TAAGGAATCTGCCT-ACAGGAGGTGGG	235	-1	0.67%
TAAGGAATCTG-----CAGGAGGTGGG	231	-5	0.66%

P84-YW-K562-Syn45crHBG200e-KO

Total | INDEL	4h	Ins	Del	HDR
9633 | 28.51%		5.20%	23.31%	0.00%

Typical seqs	Reads	Type	pct.
ATTGAGATAGTGTGGGG|AAG**GGG**CCCC	6887	WT	71.49%
ATTGAGATAGTGT------GGGGCCCC	474	-6	4.92%
ATTGAGATAGTGTGGGGgAAGGGGCCCC	364	+1 g	3.78%
ATTGAGATAGTGT-GGGAAGGGGCCCC	267	-1	2.77%
ATTGAGATAGTGT----AAGGGGCCCC	233	-4	2.42%
ATTGAGATAGTGT-------GGGCCCC	144	-7	1.49%
ATTGAGATAGTGTGGGG-AGGGGCCCC	106	-1	1.10%
ATTGAGATAGTGT--GGAAGGGGCCCC	105	-2	1.09%
ATTGAGATAGTGT---GAAGGGGCCCC	94	-3	0.98%
ATTGAGAT---------AAGGGGCCCC	54	-9	0.56%

Total | INDEL	8h	Ins	Del	HDR
7818 | 29.42%		5.09%	24.33%	0.00%

Typical seqs	Reads	Type	pct.
ATTGAGATAGTGTGGGG|AAG**GGG**CCCC	5518	WT	70.58%
ATTGAGATAGTGT------GGGGCCCC	385	-6	4.92%
ATTGAGATAGTGTGGGGgAAGGGGCCCC	306	+1 g	3.91%
ATTGAGATAGTGT-GGGAAGGGGCCCC	240	-1	3.07%
ATTGAGATAGTGT----AAGGGGCCCC	204	-4	2.61%
ATTGAGATAGTGT--GGAAGGGGCCCC	108	-2	1.38%
ATTGAGATAGTGT-------GGGCCCC	105	-7	1.34%
ATTGAGATAGTGTGGGG-AGGGGCCCC	82	-1	1.05%
ATTGAGATAGTGT---GAAGGGGCCCC	79	-3	1.01%
ATTGAGAT---------AAGGGGCCCC	55	-9	0.70%

Total | INDEL	12h	Ins	Del	HDR
23092 | 30.63%		5.21%	25.42%	0.00%

Typical seqs	Reads	Type	pct.
ATTGAGATAGTGTGGGG|AAG**GGG**CCCC	16018	WT	69.37%
ATTGAGATAGTGT------GGGGCCCC	1172	-6	5.08%
ATTGAGATAGTGTGGGGgAAGGGGCCCC	862	+1 g	3.73%
ATTGAGATAGTGT-GGGAAGGGGCCCC	706	-1	3.06%
ATTGAGATAGTGT----AAGGGGCCCC	605	-4	2.62%
ATTGAGATAGTGT-------GGGCCCC	330	-7	1.43%
ATTGAGATAGTGT--GGAAGGGGCCCC	280	-2	1.21%
ATTGAGATAGTGT---GAAGGGGCCCC	271	-3	1.17%
ATTGAGATAGTGTGGGG-AGGGGCCCC	232	-1	1.00%
ATTGAGAT---------AAGGGGCCCC	143	-9	0.62%

Total | INDEL	24h	Ins	Del	HDR
9584 | 32.73%		5.18%	27.56%	0.00%

Typical seqs	Reads	Type	pct.
ATTGAGATAGTGTGGGG|AAG**GGG**CCCC	6447	WT	67.27%
ATTGAGATAGTGT------GGGGCCCC	562	-6	5.86%
ATTGAGATAGTGTGGGGgAAGGGGCCCC	365	+1 g	3.81%
ATTGAGATAGTGT-GGGAAGGGGCCCC	312	-1	3.26%
ATTGAGATAGTGT----AAGGGGCCCC	219	-4	2.29%
ATTGAGATAGTGT-------GGGCCCC	135	-7	1.41%
ATTGAGATAGTGT--GGAAGGGGCCCC	129	-2	1.35%
ATTGAGATAGTGT---GAAGGGGCCCC	121	-3	1.26%
ATTGAGATAGTGTGGGG-AGGGGCCCC	111	-1	1.16%
ATTGAGATAGT-----GAAGGGGCCCC	71	-5	0.74%

Total | INDEL	48h	Ins	Del	HDR
28000 | 34.80%		5.60%	29.20%	0.00%

Typical seqs	Reads	Type	pct.
ATTGAGATAGTGTGGGG|AAG**GGG**CCCC	18257	WT	65.20%
ATTGAGATAGTGT------GGGGCCCC	1734	-6	6.19%
ATTGAGATAGTGTGGGGgAAGGGGCCCC	1083	+1 g	3.87%
ATTGAGATAGTGT-GGGAAGGGGCCCC	942	-1	3.36%
ATTGAGATAGTGT----AAGGGGCCCC	721	-4	2.58%
ATTGAGATAGTGT-------GGGCCCC	487	-7	1.74%
ATTGAGATAGTGT--GGAAGGGGCCCC	385	-2	1.38%
ATTGAGATAGTGTGGGG-AGGGGCCCC	321	-1	1.15%
ATTGAGATAGTGT---GAAGGGGCCCC	301	-3	1.08%
ATTGAGAT---------AAGGGGCCCC	196	-9	0.70%

P85-YW-K562-Syn45crHBG200e-AAV6KI

4h

Total | INDEL	Ins	Del	HDR
5233 | 33.21%	4.68%	20.28%	8.26%

Typical seqs	Reads	Type	pct.
ATTGAGATAGTGTGGGG|AAG**GGG**CCCC	3495	WT	66.79%
ATTGAGATAGGTTT--------------- AAACTA	422	-7 HDR	8.06%
ATTGAGATAGTGT------GGGGCCCC	197	-6	3.76%
ATTGAGATAGTGTGGGGgAAGGGGCCCC	188	+1 g	3.59%
ATTGAGATAGTGT-GGGAAGGGGCCCC	165	-1	3.15%
ATTGAGATAGTGT----AAGGGGCCCC	132	-4	2.52%
ATTGAGATAGTGT--GGAAGGGGCCCC	75	-2	1.43%
ATTGAGATAGTGTGGGG-AGGGGCCCC	64	-1	1.22%
ATTGAGATAGTGT---GAAGGGGCCCC	53	-3	1.01%
ATTGAGATAGT-----GAAGGGGCCCC	38	-5	0.73%

8h

Total | INDEL	Ins	Del	HDR
4814 | 36.31%	5.67%	21.89%	8.75%

Typical seqs	Reads	Type	pct.
ATTGAGATAGTGTGGGG|AAG**GGG**CCCC	3066	WT	63.69%
ATTGAGATAGGTTT--------------- AAACTA	421	-7 HDR	8.75%
ATTGAGATAGTGTGGGGgAAGGGGCCCC	215	+1 g	4.47%
ATTGAGATAGTGT------GGGGCCCC	186	-6	3.86%
ATTGAGATAGTGT-GGGAAGGGGCCCC	163	-1	3.39%
ATTGAGATAGTGT----AAGGGGCCCC	127	-4	2.64%
ATTGAGATAGTGT--GGAAGGGGCCCC	72	-2	1.50%
ATTGAGATAGTGTGGGG-AGGGGCCCC	48	-1	1.00%
ATTGAGATAGTGT---GAAGGGGCCCC	40	-3	0.83%
ATTGAGATAGT-----GAAGGGGCCCC	37	-5	0.77%

12h

Total | INDEL	Ins	Del	HDR
14762 | 36.89%	5.95%	23.06%	7.89%

Typical seqs	Reads	Type	pct.
ATTGAGATAGTGTGGGG|AAG**GGG**CCCC	9316	WT	63.11%
ATTGAGATAGGTTT--------------- AAACTA	1149	-7 HDR	7.78%
ATTGAGATAGTGTGGGGgAAGGGGCCCC	606	+1 g	4.11%
ATTGAGATAGTGT------GGGGCCCC	588	-6	3.98%
ATTGAGATAGTGT-GGGAAGGGGCCCC	532	-1	3.60%
ATTGAGATAGTGT----AAGGGGCCCC	377	-4	2.55%
ATTGAGATAGTGT--GGAAGGGGCCCC	249	-2	1.69%
ATTGAGATAGTGT---GAAGGGGCCCC	179	-3	1.21%
ATTGAGATAGTGTGGGG-AGGGGCCCC	137	-1	0.93%
ATTGAGATAGTGT-------GGGCCCC	93	-7	0.63%

24h

Total | INDEL	Ins	Del	HDR
6866 | 37.61%	5.53%	23.27%	8.80%

Typical seqs	Reads	Type	pct.
ATTGAGATAGTGTGGGG|AAG**GGG**CCCC	4284	WT	62.39%
ATTGAGATAGGTTT--------------- AAACTA	600	-7 HDR	8.74%
ATTGAGATAGTGT------GGGGCCCC	288	-6	4.19%
ATTGAGATAGTGTGGGGgAAGGGGCCCC	263	+1 g	3.83%
ATTGAGATAGTGT-GGGAAGGGGCCCC	220	-1	3.20%
ATTGAGATAGTGT----AAGGGGCCCC	188	-4	2.74%
ATTGAGATAGTGT--GGAAGGGGCCCC	104	-2	1.51%
ATTGAGATAGTGT---GAAGGGGCCCC	85	-3	1.24%
ATTGAGATAGTGTGGGG-AGGGGCCCC	67	-1	0.98%
ATTGAGAT---------AAGGGGCCCC	56	-9	0.82%

48h

Total | INDEL	Ins	Del	HDR
17603 | 40.18%	5.62%	24.27%	10.29%

Typical seqs	Reads	Type	pct.
ATTGAGATAGTGTGGGG|AAG**GGG**CCCC	10530	WT	59.82%
ATTGAGATAGGTTT--------------- AAACTA	1760	-7 HDR	10.00%
ATTGAGATAGTGT------GGGGCCCC	702	-6	3.99%
ATTGAGATAGTGTGGGGgAAGGGGCCCC	680	+1 g	3.86%
ATTGAGATAGTGT-GGGAAGGGGCCCC	571	-1	3.24%
ATTGAGATAGTGT----AAGGGGCCCC	478	-4	2.72%
ATTGAGATAGTGT--GGAAGGGGCCCC	254	-2	1.44%
ATTGAGATAGTGT---GAAGGGGCCCC	208	-3	1.18%
ATTGAGATAGTGTGGGG-AGGGGCCCC	177	-1	1.01%
ATTGAGATAGTGT-------GGGCCCC	148	-7	0.84%

P143-P147-YW(WT)-K562-RNP-KO-Syn80crBCL11A3a

4h — Total 61634 | INDEL 3.64% | Ins 3.15% | Del 0.49% | HDR 0.00%

Typical seqs	Reads	Type	pct.
CGTAAGGCAACACACGTCTA**TGG**GGAG	59392	WT	96.36%
CGTAAGGCAACACACGTtCTATGGGGAG	1818	+1 t	2.95%
CGTAAGGCAACACACG--TATGGGGAG	127	-2	0.21%
CGTAAGGCAACACACGT-TATGGGGAG	34	-1	0.06%
CGTAAGGCAACACACG----TGGGGAG	24	-4	0.04%
CGTAAGGCAACACA---CTATGGGGAG	20	-3	0.03%
CGTAAGGCAACACACGTtCTATGGGGGG	14	+1 t	0.02%
CGTAAGGCAACACGCGTTCTATGGGGAG	13	+1 Ins	0.02%
CGTAAGGCAACACACGTcCTATGGGGAG	11	+1 c	0.02%
CGTAAGGCAA-------CTATGGGGAG	10	-7	0.02%

8h — Total 45720 | INDEL 7.76% | Ins 6.64% | Del 1.12% | HDR 0.00%

Typical seqs	Reads	Type	pct.
CGTAAGGCAACACACGTCTA**TGG**GGAG	42172	WT	92.24%
CGTAAGGCAACACACGTtCTATGGGGAG	2758	+1 t	6.03%
CGTAAGGCAACACACG--TATGGGGAG	133	-2	0.29%
CGTAAGGCAACACACGT-TATGGGGAG	72	-1	0.16%
CGTAAGGCAACACA---CTATGGGGAG	37	-3	0.08%
CGTAAGGCAACACACG----TGGGGAG	33	-4	0.07%
CGTAAGGCAACACACGT---TGGGGAG	23	-3	0.05%
CGTAAGGCAACACACGTcCTATGGGGAG	19	+1 c	0.04%
CGTAAGGCAACACACGT------GGAG	19	-6	0.04%
CGTAAGGCGACACACGTTCTATGGGGAG	18	+1 Ins	0.04%

12h — Total 48289 | INDEL 10.38% | Ins 8.80% | Del 1.58% | HDR 0.00%

Typical seqs	Reads	Type	pct.
CGTAAGGCAACACACGTCTA**TGG**GGAG	43278	WT	89.62%
CGTAAGGCAACACACGTtCTATGGGGAG	3861	+1 t	8.00%
CGTAAGGCAACACACG--TATGGGGAG	196	-2	0.41%
CGTAAGGCAACACACGT-TATGGGGAG	125	-1	0.26%
CGTAAGGCAACACACG----TGGGGAG	64	-4	0.13%
CGTAAGGCAACACA---CTATGGGGAG	49	-3	0.10%
CGTAAGGCAACACAC-TCTATGGGGAG	28	-1	0.06%
CGTAAGGCAACACGCGTTCTATGGGGAG	25	+1 Ins	0.05%
CGTAAGGCAACACACG-CTATGGGGAG	23	-1	0.05%
CGTAAGGCAACACACGTtCTATGGGGGG	22	+1 t	0.05%

24h — Total 53510 | INDEL 16.11% | Ins 12.11% | Del 4.00% | HDR 0.00%

Typical seqs	Reads	Type	pct.
CGTAAGGCAACACACGTCTA**TGG**GGAG	44887	WT	83.89%
CGTAAGGCAACACACGTtCTATGGGGAG	5983	+1 t	11.18%
CGTAAGGCAACACACG--TATGGGGAG	429	-2	0.80%
CGTAAGGCAACACACGT-TATGGGGAG	229	-1	0.43%
CGTAAGGCAACACACG----TGGGGAG	148	-4	0.28%
CGTAAGGCAACACA---CTATGGGGAG	97	-3	0.18%
CG----------------TATGGGGAG	43	-16	0.08%
CGTAAGGCAACA-----CTATGGGGAG	39	-5	0.07%
CGTAAGGCAAC----------------	49	-21	0.09%
CGTAAGGCAACAC------ATGGGGAG	37	-6	0.07%

48h — Total 190367 | INDEL 23.20% | Ins 16.03% | Del 7.17% | HDR 0.00%

Typical seqs	Reads	Type	pct.
CGTAAGGCAACACACGTCTA**TGG**GGAG	146202	WT	76.80%
CGTAAGGCAACACACGTtCTATGGGGAG	27519	+1 t	14.46%
CGTAAGGCAACACACG--TATGGGGAG	2020	-2	1.06%
CGTAAGGCAACACACGT-TATGGGGAG	1029	-1	0.54%
CGTAAGGCAACACACG----TGGGGAG	810	-4	0.43%
CGTAAGGCAACACA---CTATGGGGAG	605	-3	0.32%
CGTAAGGCAACACAC--CTATGGGGAG	255	-2	0.13%
CGTA-----------------------	368	-23	0.19%
CGTAAGGCA------------------	345	-23	0.18%
CGTAAGGCAACACAC---------GAG	261	-9	0.14%

P143-P147-YW(WT)-K562-RNP-KO-Syn81crBCL11A3b

Total | INDEL	4h	Ins	Del	HDR
20100 | 4.72%		0.57%	4.15%	0.00%
**Typical seqs**		**Reads**	**Type**	**pct.**
TGATAACGCCAGTAACC|CGA**GGG**CCAG		19151	WT	95.28%
TGATAACGCCAGTAA-CCGAGGGCCAG		637	-1	3.17%
TGATAACGCCAGTAACCcCGAGGGCCAG		107	+1 c	0.53%
TGATAACGCCAGTAA--CGAGGGCCAG		46	-2	0.23%
TGATAACGCCAGTA---CGAGGGCCAG		26	-3	0.13%
TGATAACGCCA-----CCGAGGGCCAG		14	-5	0.07%
TGATAACGCCAGT----CGAGGGCCAG		9	-4	0.04%
TGATAACGCCAGT---CCGAGGGCCAG		9	-3	0.04%
TGATAACGCCAGTA--CCGAGGGCCAG		8	-2	0.04%
TGATAACGCCAG----CCGAGGGCCAG		7	-4	0.03%

Total | INDEL	8h	Ins	Del	HDR
8078 | 12.55%		1.29%	11.27%	0.00%
**Typical seqs**		**Reads**	**Type**	**pct.**
TGATAACGCCAGTAACC|CGA**GGG**CCAG		7064	WT	87.45%
TGATAACGCCAGTAA-CCGAGGGCCAG		619	-1	7.66%
TGATAACGCCAGTAACCcCGAGGGCCAG		94	+1 c	1.16%
TGATAACGCCAGTAA--CGAGGGCCAG		85	-2	1.05%
TGATAAC---------------GCCAG		23	-15	0.28%
TGATAACGCCAGTA--CCGAGGGCCAG		18	-2	0.22%
TGATAACGCCAGTA---CGAGGGCCAG		18	-3	0.22%
TGATAACG--------CCGAGGGCCAG		16	-8	0.20%
TGATAACGCCA------CGAGGGCCAG		11	-6	0.14%
TGATAACGCCAGT----CGAGGGCCAG		11	-4	0.14%

Total | INDEL	12h	Ins	Del	HDR
13774 | 21.68%		1.91%	19.77%	0.00%
**Typical seqs**		**Reads**	**Type**	**pct.**
TGATAACGCCAGTAACC|CGA**GGG**CCAG		10788	WT	78.32%
TGATAACGCCAGTAA-CCGAGGGCCAG		1689	-1	12.26%
TGATAACGCCAGTAACCcCGAGGGCCAG		198	+1 c	1.44%
TGATAACGCCAGTAA--CGAGGGCCAG		151	-2	1.10%
TGATAAC---------------GCCAG		132	-15	0.96%
TGATAACGCCAGTA---CGAGGGCCAG		45	-3	0.33%
TGATAACGCCAGTA--CCGAGGGCCAG		39	-2	0.28%
TGATAACGCCAGT----CGAGGGCCAG		38	-4	0.28%
TGATAACG--------CCGAGGGCCAG		36	-8	0.26%
TGATAACGCCA------CGAGGGCCAG		32	-6	0.23%

Total | INDEL	24h	Ins	Del	HDR
2990 | 46.92%		2.61%	44.31%	0.00%
**Typical seqs**		**Reads**	**Type**	**pct.**
TGATAACGCCAGTAACC|CGA**GGG**CCAG		1587	WT	53.08%
TGATAACGCCAGTAA-CCGAGGGCCAG		655	-1	21.91%
TGATAAC---------------GCCAG		274	-15	9.16%
TGATAACGCCAGTAA--CGAGGGCCAG		77	-2	2.58%
TGATAACGCCAGTAACCcCGAGGGCCAG		66	+1 c	2.21%
TGATAACGCCAGTA---CGAGGGCCAG		37	-3	1.24%
TGATAA-----------CGAGGGCCAG		25	-11	0.84%
TGATAACGCCAGTA--CCGAGGGCCAG		22	-2	0.74%
TGATAACGCCAGTAACC--AGGGCCAG		21	-2	0.70%
TGATAACGCCAGTAA--------CCAG		14	-8	0.47%

Total | INDEL	48h	Ins	Del	HDR
23765 | 64.44%		3.93%	60.51%	0.00%
**Typical seqs**		**Reads**	**Type**	**pct.**
TGATAACGCCAGTAACC|CGA**GGG**CCAG		8450	WT	35.56%
TGATAACGCCAGTAA-CCGAGGGCCAG		5384	-1	22.66%
TGATAAC---------------GCCAG		3603	-15	15.16%
TGATAACGCCAGTAACCcCGAGGGCCAG		698	+1 c	2.94%
TGATAACGCCAGTAA--CGAGGGCCAG		575	-2	2.42%
TGATAA-----------CGAGGGCCAG		239	-11	1.01%
TGATAACGCCAGTA---CGAGGGCCAG		234	-3	0.98%
TGATAACGCCAGTA--CCGAGGGCCAG		153	-2	0.64%
TGATAACGCCAGTAA--------CCAG		140	-8	0.59%
TGATAACG--------CCGAGGGCCAG		131	-8	0.55%

P143-P147-YW(WT)-K562-RNP-KO-Syn82crBCL11A4a

Total	INDEL		Ins	Del	HDR
23090	1.94%	4h	1.62%	0.32%	0.00%

Typical seqs	Reads	Type	pct.
TCGTAACACACCGTCAT|CCG**GGG**AAGT	22643	WT	98.06%
TCGTAACACACCGTCATtCCGGGGAAGT	361	+1 t	1.56%
TCGTAACACACCGTC-TCCGGGGAAGT	13	-1	0.06%
TCGTAACACA-------CCGGGGAAGT	10	-7	0.04%
TCGTAACACACCGTCATCC-GGGAAGT	10	-1	0.04%
TCGTAACACACCG---TCCGGGGAAGT	10	-3	0.04%
TCGTAACACACCGTCATATCCGGGGAAGT	7	+2	0.03%
TCGTAACACACCGTCAT--GGGGAAGT	5	-2	0.02%
TCGTAACACACC----TCCGGGGAAGT	5	-4	0.02%
TCGTAACGCACCGTCATTCCGGGGAAGT	3	+1 Ins	0.01%

Total	INDEL		Ins	Del	HDR
8782	4.07%	8h	3.15%	0.91%	0.00%

Typical seqs	Reads	Type	pct.
TCGTAACACACCGTCAT|CCG**GGG**AAGT	8425	WT	95.93%
TCGTAACACACCGTCATtCCGGGGAAGT	271	+1 t	3.09%
TCGTAACACA-------CCGGGGAAGT	24	-7	0.27%
TCGTA----------ATCCGGGGAAGT	6	-10	0.07%
TCGTAACACACCGTC-TCCGGGGAAGT	6	-1	0.07%
TCGTAACACACCG---TCCGGGGAAGT	6	-3	0.07%
TCGTAACACACCGTCAT-CGGGGAAGT	5	-1	0.06%
TCGTAACACACCGTCAT--GGGGAAGT	5	-2	0.06%
TCGTAACACACCGTCAT----------	5	-14	0.06%
TCGTAACACACCGTCATCC-GGGAAGT	4	-1	0.05%

Total	INDEL		Ins	Del	HDR
14430	6.84%	12h	4.50%	2.34%	0.00%

Typical seqs	Reads	Type	pct.
TCGTAACACACCGTCAT|CCG**GGG**AAGT	13443	WT	93.16%
TCGTAACACACCGTCATtCCGGGGAAGT	584	+1 t	4.05%
TCGTAACACA-------CCGGGGAAGT	99	-7	0.69%
TCGTAACACACCGTCAT-CGGGGAAGT	27	-1	0.19%
TCGTAACACACCG---TCCGGGGAAGT	22	-3	0.15%
TCGTAACACACCGTCA-CCGGGGAAGT	15	-1	0.10%
TCGTAACACACCGTCATCC-GGGAAGT	11	-1	0.08%
TCGTAACACAC------CCGGGGAAGT	11	-6	0.08%
TCGTAACACACCGTCATcCCGGGGAAGT	10	+1 c	0.07%
TCGTAACACACCGTCATATCCGGGGAAGT	9	+2	0.06%

Total	INDEL		Ins	Del	HDR
4436	12.67%	24h	5.88%	6.79%	0.00%

Typical seqs	Reads	Type	pct.
TCGTAACACACCGTCAT|CCG**GGG**AAGT	3874	WT	87.33%
TCGTAACACACCGTCATtCCGGGGAAGT	254	+1 t	5.73%
TCGTAACACA-------CCGGGGAAGT	174	-7	3.92%
TCGTAACACACCG---TCCGGGGAAGT	15	-3	0.34%
TCGTAACACACCGTC-TCCGGGGAAGT	14	-1	0.32%
TCGTA----------ATCCGGGGAAGT	9	-10	0.20%
TCGTAACACACCGTCAT-CGGGGAAGT	8	-1	0.18%
TCGTAACACACCGTCA-CCGGGGAAGT	7	-1	0.16%
TCGTAA--CACC-----CCGGGGAAGT	7	-7	0.16%
TCGTAAC---------ACCGGGGAAGT	7	-9	0.16%

Total	INDEL		Ins	Del	HDR
38920	20.93%	48h	9.01%	11.92%	0.00%

Typical seqs	Reads	Type	pct.
TCGTAACACACCGTCAT|CCG**GGG**AAGT	30773	WT	79.07%
TCGTAACACACCGTCATtCCGGGGAAGT	3075	+1 t	7.90%
TCGTAACACA-------CCGGGGAAGT	2172	-7	5.58%
TCGTAACACACCG---TCCGGGGAAGT	185	-3	0.48%
TCGTAACACACCGTC-TCCGGGGAAGT	93	-1	0.24%
TCGTAACACACCGTCA-CCGGGGAAGT	87	-1	0.22%
TCGTAACACACCGTCAT-CGGGGAAGT	72	-1	0.18%
TCGTAA-----------CCGGGGAAGT	59	-11	0.15%
TCGTAACACACCGTCAT-----GAAGT	61	-5	0.16%
TCGTA----------ATCCGGGGAAGT	57	-10	0.15%

P143-P147-YW(WT)-K562-RNP-KO-Syn83crBCL11A4b

4h

Total	INDEL	Ins	Del	HDR
44784	1.18%	**0.19%**	**0.99%**	**0.00%**

Typical seqs	Reads	Type	pct.
ACTTCATGCGGAGGCCC|CGT**GGG**AGGA	44256	WT	98.82%
ACTTCATGCGGAGGCC-CGTGGGAGGA	227	-1	0.51%
ACTTCATGCGGAGGCCCcCGTGGGAGGA	59	+1 c	0.13%
ACTTCATG---------CGTGGGAGGA	26	-9	0.06%
ACTTCATGCGGAGGCCCCGT-GGAGGA	26	-1	0.06%
ACTTCATGCGGAGGC--CGTGGGAGGA	23	-2	0.05%
ACTTCATGCGGAGGCCC-----GAGGA	13	-5	0.03%
ACTTCATGC------------GGAGGA	10	-12	0.02%
ACTTCATGCGGAGGCCC----GGAGGA	9	-4	0.02%
ACTT-------------CGTGGGAGGA	9	-13	0.02%

8h

Total	INDEL	Ins	Del	HDR
44214	3.72%	**0.34%**	**3.38%**	**0.00%**

Typical seqs	Reads	Type	pct.
ACTTCATGCGGAGGCCC|CGT**GGG**AGGA	42568	WT	96.28%
ACTTCATGCGGAGGCC-CGTGGGAGGA	567	-1	1.28%
ACTTCATGC------------GGAGGA	149	-12	0.34%
ACTTCATG---------CGTGGGAGGA	103	-9	0.23%
ACTTCATGCGGAGGCCCcCGTGGGAGGA	70	+1 c	0.16%
ACTTCA-------------TGGGAGGA	59	-13	0.13%
ACTT-------------CGTGGGAGGA	45	-13	0.10%
ACTTCATGCGGAGGC--CGTGGGAGGA	41	-2	0.09%
ACTTCATGCGGAGGCCCCGT-GGAGGA	32	-1	0.07%
ACTTCAT-----------GTGGGAGGA	29	-11	0.07%

12h

Total	INDEL	Ins	Del	HDR
59933	8.26%	**0.60%**	**7.67%**	**0.00%**

Typical seqs	Reads	Type	pct.
ACTTCATGCGGAGGCCC|CGT**GGG**AGGA	54982	WT	91.74%
ACTTCATGCGGAGGCC-CGTGGGAGGA	1147	-1	1.91%
ACTTCATGC------------GGAGGA	596	-12	0.99%
ACTTCATG---------CGTGGGAGGA	365	-9	0.61%
ACTTCA-------------TGGGAGGA	237	-13	0.40%
ACTT-------------CGTGGGAGGA	184	-13	0.31%
ACTTCATGCGGAGGCCCcCGTGGGAGGA	175	+1 c	0.29%
ACTTCATGCGGAGGC--CGTGGGAGGA	143	-2	0.24%
ACTTCATGCGGAGG---CGTGGGAGGA	127	-3	0.21%
ACTTCAT-----------GTGGGAGGA	118	-11	0.20%

24h

Total	INDEL	Ins	Del	HDR
8458	37.72%	**0.98%**	**36.73%**	**0.00%**

Typical seqs	Reads	Type	pct.
ACTTCATGCGGAGGCCC|CGT**GGG**AGGA	5268	WT	62.28%
ACTTCATGCGCA---------GGAGGA	662	-9	7.83%
ACTTCA-------------TGGGAGGA	671	-13	7.93%
ACTTCATGC------------GGAGGA	527	-12	6.23%
ACTTCATGCGGAGGCC-CGTGGGAGGA	261	-1	3.09%
ACTTCATG---------CGTGGGAGGA	139	-9	1.64%
ACTT-------------CGTGGGAGGA	120	-13	1.42%
ACTTCAT-----------GTGGGAGGA	87	-11	1.03%
ACTTCATGCGGAGGC--CGTGGGAGGA	48	-2	0.57%
ACTTCATGC---------------GGA	46	-15	0.54%

48h

Total	INDEL	Ins	Del	HDR
161265	46.39%	**2.95%**	**43.44%**	**0.00%**

Typical seqs	Reads	Type	pct.
ACTTCATGCGGAGGCCC|CGT**GGG**AGGA	86455	WT	53.61%
ACTTCATGC------------GGAGGA	17666	-12	10.95%
ACTTCATGCGGAGGCC-CGTGGGAGGA	8077	-1	5.01%
ACTTCA-------------TGGGAGGA	5419	-13	3.36%
ACTTCATG---------CGTGGGAGGA	4710	-9	2.92%
ACTT-------------CGTGGGAGGA	4415	-13	2.74%
ACTTCAT-----------GTGGGAGGA	2746	-11	1.70%
ACTTCATGC---------------GGA	2005	-15	1.24%
ACTTCATGCGGAGG---CGTGGGAGGA	1363	-3	0.85%
ACTTCATGCGGAGGC--CGTGGGAGGA	1110	-2	0.69%

P143-P147-YW(WT)-K562-RNP-KO-Syn84crBCL11A4c

4h — Total | INDEL: 11860 | 7.17%; Ins 0.11%; Del 7.06%; HDR 0.00%

Typical seqs	Reads	Type	pct.
GTAGGCGACCAACATGG|GGG**GGG**GGTG	11010	WT	92.83%
GTAGGCGACCAACAT--GGTGGGGGTG	416	-2	3.51%
GTAGGCGACCAACAT-GGGTGGGGGTG	143	-1	1.21%
GTAGGCGACCAACAT-GGGTGGGGGGG	54	-1	0.46%
GTAGGCGACCAAC----GGTGGGGGTG	51	-4	0.43%
GTAGGCGACCAACA-----TGGGGGTG	21	-5	0.18%
GTAGGCGACCAACATGGGGGGGGGGT-	19	-1	0.16%
GTAGGCGACCAACAT--GGTGGGGGGG	13	-2	0.11%
GTAGGCGACCAA-----GGTGGGGGTG	12	-5	0.10%
GTAGGCGAC--------GGTGGGGGTG	12	-8	0.10%

8h — Total | INDEL: 7780 | 21.66%; Ins 0.23%; Del 21.43%; HDR 0.00%

Typical seqs	Reads	Type	pct.
GTAGGCGACCAACATGG|GGG**GGG**GGtG	6095	WT	78.34%
GTAGGCGACCAACAT--GGTGGGGGTG	714	-2	9.18%
GTAGGCGACCAACAT-GGGTGGGGGGG	186	-1	2.39%
GTAGGCGACCAACAT-GGGTGGGGGTG	139	-1	1.79%
GTAGGCGACCAAC----GGTGGGGGTG	76	-4	0.98%
GTAGGCGACCAACATGGGG-GGGGGGG	51	-1	0.66%
GTAGGCGACCAACAT--GGTGGGGGGG	42	-2	0.54%
GTAGGCGACCAACA-----TGGGGGTG	33	-5	0.42%
GTAGGCGACCAACA---GGTGGGGGTG	30	-3	0.39%
GTAGGCGACCAACA-----------TG	30	-11	0.39%

12h — Total | INDEL: 14992 | 26.91%; Ins 0.33%; Del 26.58%; HDR 0.00%

Typical seqs	Reads	Type	pct.
GTAGGCGACCAACATGG|GGG**GGG**GGtG	10957	WT	73.09%
GTAGGCGACCAACAT--GGTGGGGGTG	1559	-2	10.40%
GTAGGCGACCAACAT-GGGTGGGGGTG	564	-1	3.76%
GTAGGCGACCAACAT-GGGTGGGGGGG	261	-1	1.74%
GTAGGCGACCAACA-----TGGGGGTG	146	-5	0.97%
GTAGGCGACCAAC----GGTGGGGGTG	124	-4	0.83%
GTA--------------GGTGGGGGTG	71	-14	0.47%
GTAGGC---------------------	68	-24	0.45%
GTAGGCGAC--------GGTGGGGGTG	66	-8	0.44%
GTAGGCGACCAACA-----------TG	52	-11	0.35%

24h — Total | INDEL: 60118 | 60.22%; Ins 1.13%; Del 59.09%; HDR 0.00%

Typical seqs	Reads	Type	pct.
GTAGGCGACCAACATGG|GGG**GGG**GGTG	23915	WT	39.78%
GTAGGCGACCAACAT--GGTGGGGGTG	12175	-2	20.25%
GTAGGCGACCAACAT-GGGTGGGGGTG	3233	-1	5.38%
GTAGGCGACCAACA-----TGGGGGTG	2644	-5	4.40%
GTAGGCGACCAACAT-GGGTGGGGGGG	1150	-1	1.91%
GTA--------------GGTGGGGGTG	1121	-14	1.86%
GTAGGCGACCAAC----GGTGGGGGTG	773	-4	1.29%
GTAGGCGACCAACAT--------GGTG	712	-8	1.18%
GTAGGC---------------------	895	-24	1.49%
------------------GTGGGGGTG	429	-18	0.71%

48h — Total | INDEL: 4236 | 74.08%; Ins 1.20%; Del 72.88%; HDR 0.00%

Typical seqs	Reads	Type	pct.
GTAGGCGACCAACATGG|GGG**GGG**GGtG	1098	WT	25.92%
GTAGGCGACCAACAT--GGTGGGGGTG	1273	-2	30.05%
GTAGGCGACCAACA-----TGGGGGTG	391	-5	9.23%
GTAGGCGACCAACAT-GGGTGGGGGTG	183	-1	4.32%
GTAGGCGACCAACAT-GGGTGGGGGGG	124	-1	2.93%
GTAGGCGACCAAC----GGTGGGGGTG	101	-4	2.38%
GTAGGCGACCAACAT--------GGTG	72	-8	1.70%
GTA--------------GGTGGGGGTG	59	-14	1.39%
GTAGGCGACCAACAT--GGTGGGGGGG	43	-2	1.02%
GTAGGCGACCAACA-----------TG	38	-11	0.90%

P143-P147-YW(WT)-K562-RNP-KO-Syn86crBCL11A5a

Total | INDEL	4h	Ins	Del	HDR
15551 | 0.66%		0.05%	0.60%	0.00%
**Typical seqs**		**Reads**	**Type**	**pct.**
TCCGAACAGCCACAATC|GGGGGGCTAC		15449	WT	99.34%
TCCGAACAGCCACAATC-GGGGGCTAC		91	-1	0.59%
TCCGAACAGCCACAATCcGGGGGGCTAC		5	+1 c	0.03%
TCCGAACAGCCACAATCtGGGGGGCTAC		3	+1 t	0.02%
TCCGAACAGCCACAATC-GGGGGTTAC		3	-1	0.02%

Total | INDEL	8h	Ins	Del	HDR
11355 | 1.37%		0.11%	1.27%	0.00%
**Typical seqs**		**Reads**	**Type**	**pct.**
TCCGAACAGCCACAATC|GGGGGGCTAC		11199	WT	98.63%
TCCGAACAGCCACAATC-GGGGGCTAC		118	-1	1.04%
TCCGAACAGCCACAAT-GGGGGGCTAC		12	-1	0.11%
TCCGAACAGCCACAATCcGGGGGGCTAC		12	+1 c	0.11%
TCCGAACAGCCACAAT--GGGGGCTAC		4	-2	0.04%
TCCGAACAGCCA----CGGGGGGCTAC		4	-4	0.04%
TCCGAACGGCCACAATC-GGGGGCTAC		3	-1	0.03%
TCCGA--------------------AC		3	-20	0.03%

Total | INDEL	12h	Ins	Del	HDR
12520 | 1.24%		0.16%	1.08%	0.00%
**Typical seqs**		**Reads**	**Type**	**pct.**
TCCGAACAGCCACAATC|GGGGGGCTAC		12365	WT	98.76%
TCCGAACAGCCACAATC-GGGGGCTAC		95	-1	0.76%
TCCGAACAGCCACAATCcGGGGGGCTAC		20	+1 c	0.16%
TCCGAACAGCCACAAT-GGGGGGCTAC		16	-1	0.13%
TCCGAACAGCCACAA--GGGGGGCTAC		8	-2	0.06%
TCCGAACAGCCACAA------------		7	-14	0.06%
TCCGAACA--------------GCTAC		3	-14	0.02%
TCCGAA----------CGGGGGGCTAC		3	-10	0.02%
TCCGAACAGCCACAATC--GGGGCTAC		3	-2	0.02%

Total | INDEL	24h	Ins	Del	HDR
12144 | 4.85%		0.68%	4.17%	0.00%
**Typical seqs**		**Reads**	**Type**	**pct.**
TCCGAACAGCCACAATC|GGGGGGCTAC		11555	WT	95.15%
TCCGAACAGCCACAATC-GGGGGCTAC		105	-1	0.86%
TCCGAACAGCCACAATCcGGGGGGCTAC		77	+1 c	0.63%
TCCGAACAGCCACAAT-GGGGGGCTAC		46	-1	0.38%
TCCGAACAGCCACAA------------		68	-12	0.56%
TCCGA--------------------AC		34	-20	0.28%
TCCGAACA--------------GCTAC		27	-14	0.22%
TCCGAACAGCC--------------AC		25	-14	0.21%
TCCGAACAGCCACAA--GGGGGGCTAC		25	-2	0.21%
TCCGAACAGCCACAA------------		68	-14	0.56%

Total | INDEL	48h	Ins	Del	HDR
28589 | 8.12%		1.03%	7.09%	0.00%
**Typical seqs**		**Reads**	**Type**	**pct.**
TCCGAACAGCCACAATC|GGGGGGCTAC		26269	WT	91.88%
TCCGAACAGCCACAATC-GGGGGCTAC		247	-1	0.86%
TCCGAACAGCCACAATCcGGGGGGCTAC		223	+1 c	0.78%
TCCGAACAGCCACAA------------		298	-12	1.04%
TCCGAACAGCCACAAT-GGGGGGCTAC		151	-1	0.53%
TCCGA--------------------AC		148	-20	0.52%
TCCGAACAGCCACAA------------		298	-14	1.04%
TCCGAACA--------------GCTAC		93	-14	0.33%
TCCGAACAGCC--------------AC		87	-14	0.30%
TCCGAACAGCCACAAT----------C		83	-10	0.29%

P143-P147-YW(WT)-K562-RNP-KO-Syn87crBCL11A5b

Total | INDEL	4h	Ins	Del	HDR
18573 | 5.69%		1.15%	4.53%	0.00%

Typical seqs	Reads	Type	pct.
GGGATTACCGAGTCACC|ACC**AGG**CTGC	17517	WT	94.31%
GGGATTACCGAGTCAC-ACCAGGCTGC	398	-1	2.14%
GGGATTACCGAGTC---ACCAGGCTGC	363	-3	1.95%
GGGATTACCGAGTCACCcACCAGGCTGC	193	+1 c	1.04%
GGGATTACCGAGTCAC--CCAGGCTGC	15	-2	0.08%
GGGATT-----------ACCAGGCTGC	13	-11	0.07%
GGGATTACCGAGTCACC-CCAGGCTGC	9	-1	0.05%
GGGATTACCGAGTCACCaACCAGGCTGC	9	+1 a	0.05%
GGGATTACCGAGT--CCACCAGGCTGC	6	-2	0.03%
GGGATTACCGAGTCAC-----------	5	-12	0.03%

Total | INDEL	8h	Ins	Del	HDR
18736 | 11.21%		1.98%	9.23%	0.00%

Typical seqs	Reads	Type	pct.
GGGATTACCGAGTCACC|ACC**AGG**CTGC	16636	WT	88.79%
GGGATTACCGAGTCAC-ACCAGGCTGC	890	-1	4.75%
GGGATTACCGAGTC---ACCAGGCTGC	593	-3	3.17%
GGGATTACCGAGTCACCcACCAGGCTGC	330	+1 c	1.76%
GGGATT-----------ACCAGGCTGC	33	-11	0.18%
GGGATTACCGAGTCAC--CCAGGCTGC	30	-2	0.16%
GGGATTACCGAGTCACCtACCAGGCTGC	21	+1 t	0.11%
GGGATTACCGAGTCACC-CCAGGCTGC	11	-1	0.06%
GGGATTACCGAGCCA-CACCAGGCTGC	10	-1	0.05%
GGGATTACCGAGT------CAGGCTGC	10	-6	0.05%

Total | INDEL	12h	Ins	Del	HDR
7897 | 15.49%		2.82%	12.66%	0.00%

Typical seqs	Reads	Type	pct.
GGGATTACCGAGTCACC|ACC**AGG**CTGC	6674	WT	84.51%
GGGATTACCGAGTCAC-ACCAGGCTGC	433	-1	5.48%
GGGATTACCGAGTC---ACCAGGCTGC	339	-3	4.29%
GGGATTACCGAGTCACCcACCAGGCTGC	183	+1 c	2.32%
GGGATT-----------ACCAGGCTGC	65	-11	0.82%
GGGATTACCGAGTCAC--CCAGGCTGC	24	-2	0.30%
GGGATTACCGAGTCACCtACCAGGCTGC	19	+1 t	0.24%
GGGATTACCGAGT------CAGGCTGC	18	-6	0.23%
GGGATTACCGAGTCACC-CCAGGCTGC	14	-1	0.18%
GGGATTACCGAGTCACCCCACCAGGCTGC	11	+2	0.14%

Total | INDEL	24h	Ins	Del	HDR
2372 | 37.98%		4.97%	33.01%	0.00%

Typical seqs	Reads	Type	pct.
GGGATTACCGAGTCACC|ACC**AGG**CTGC	1471	WT	62.02%
GGGATTACCGAGTC---ACCAGGCTGC	263	-3	11.09%
GGGATTACCGAGTCAC-ACCAGGCTGC	255	-1	10.75%
GGGATT-----------ACCAGGCTGC	136	-11	5.73%
GGGATTACCGAGTCACCcACCAGGCTGC	107	+1 c	4.51%
GGGATTACCGAGT------CAGGCTGC	40	-6	1.69%
GGGATTACCGAGTCAC--CCAGGCTGC	12	-2	0.51%
------------------------TGC	7	-26	0.30%
GGGATTACCGAGTCACCCCACCAGGCTGC	6	+2	0.25%
GGGATTACCGAGTCACC-CCAGGCTGC	6	-1	0.25%

Total | INDEL	48h	Ins	Del	HDR
19436 | 56.69%		6.30%	50.39%	0.00%

Typical seqs	Reads	Type	pct.
GGGATTACCGAGTCACC|ACC**AGG**CTGC	8417	WT	43.31%
GGGATTACCGAGTC---ACCAGGCTGC	2915	-3	15.00%
GGGATTACCGAGTCAC-ACCAGGCTGC	2228	-1	11.46%
GGGATT-----------ACCAGGCTGC	1675	-11	8.62%
GGGATTACCGAGTCACCcACCAGGCTGC	1010	+1 c	5.20%
GGGATTACCGAGT------CAGGCTGC	662	-6	3.41%
GGGATTACCGAGTCAC--CCAGGCTGC	133	-2	0.68%
GGGATTACCG----------AGGCTGC	79	-10	0.41%
GGGATTACCGAGTC-------------	67	-18	0.34%
GGGATTACCGAGTCA------------	71	-13	0.37%

P143-P147-YW(WT)-K562-RNP-KO-Syn88crBCL11A6a

Total | INDEL	4h	Ins	Del	HDR
5531 | 0.29%		0.09%	0.20%	0.00%

Typical seqs	Reads	Type	pct.
TCCTCGGCCAATGAAGC|GGT**GGG**TCCA	5515	WT	99.71%
TCCTCGGCCAATGAAGCGGT-GGTCCA	8	-1	0.14%
TCCTCGGCCAATGAAGCcGGTGGGTCCA	5	+1 c	0.09%
TCCTCGGCCAATGAA--GGTGGGTCCA	3	-2	0.05%

Total | INDEL	8h	Ins	Del	HDR
10209 | 0.49%		0.24%	0.24%	0.00%

Typical seqs	Reads	Type	pct.
TCCTCGGCCAATGAAGC|GGT**GGG**TCCA	10159	WT	99.51%
TCCTCGGCCAATGAAGCcGGTGGGTCCA	25	+1 c	0.24%
TCCTCGGCCAATGAAGCGGT-GGTCCA	15	-1	0.15%
TCCTCGGCCAATGAA------------	7	-13	0.07%
TCCTCGGCCAATGAA--GGTGGGTCCA	3	-2	0.03%

Total | INDEL	12h	Ins	Del	HDR
4350 | 0.60%		0.21%	0.39%	0.00%

Typical seqs	Reads	Type	pct.
TCCTCGGCCAATGAAGC|GGT**GGG**TCCA	4324	WT	99.40%
TCCTCGGCCAATGAAGCcGGTGGGTCCA	9	+1 c	0.21%
TCCTCGGCCAATGAA--GGTGGGTCCA	7	-2	0.16%
TCCTC----------------GGTCCA	4	-16	0.09%
TCCTCGGCCAATGAAGC-------CCA	3	-7	0.07%
TCCTCGG-----------------CCA	3	-17	0.07%

Total | INDEL	24h	Ins	Del	HDR
9842 | 3.00%		0.46%	2.54%	0.00%

Typical seqs	Reads	Type	pct.
TCCTCGGCCAATGAAGC|GGT**GGG**TCCA	9547	WT	97.00%
TCCTCGGCCAATGAA------------	35	-13	0.36%
TCCTCGGCCAATGAAGCcGGTGGGTCCA	35	+1 c	0.36%
TCCTC----------------------	28	-22	0.28%
TCCTCGGCCAATGAA--GGTGGGTCCA	17	-2	0.17%
TCCTCGGCCAATGA------------A	17	-12	0.17%
TCCTCGGCCAA--------TGGGTCCA	16	-8	0.16%
TCCTCGGCCAATGA---GGTGGGTCCA	10	-3	0.10%
TCCT------------CGGTGGGTCCA	9	-12	0.09%
TCCTCGG-----------------CCA	9	-17	0.09%

Total | INDEL	48h	Ins	Del	HDR
6549 | 5.57%		0.43%	5.15%	0.00%

Typical seqs	Reads	Type	pct.
TCCTCGGCCAATGAAGC|GGT**GGG**TCCA	6184	WT	94.43%
TCCTCGGCCAATGAA------------	46	-13	0.70%
TCCTC----------------------	31	-22	0.47%
TCCTCGGCCAATGAA--GGTGGGTCCA	29	-2	0.44%
TCCTCGGCCAATGAAGCcGGTGGGTCCA	28	+1 c	0.43%
TCCTCGGCCAA--------TGGGTCCA	23	-8	0.35%
TCCTC----------------GGTCCA	16	-16	0.24%
TCCTCGG-----------------CCA	16	-17	0.24%
TCCTCGGCCAATGA------------A	13	-12	0.20%
TCCT------------CGGTGGGTCCA	12	-12	0.18%

P143-P147-YW(WT)-K562-RNP-KO-Syn90crBCL11A6c

Total | INDEL	4h	Ins	Del	HDR
9463 | 0.10%		**0.10%**	**0.00%**	**0.00%**

Typical seqs	Reads	Type	pct.
CTGCCTACCGGCACGCA|TGC**AGG**ATCA	9454	WT	99.90%
CTGCCTACCGGCACGCAaTGCAGGATCA	5	+1 a	0.05%
CTGCCTACCGGCACGCAtTGCAGGATCA	4	+1 t	0.04%

Total | INDEL	8h	Ins	Del	HDR
13631 | 0.29%		**0.29%**	**0.00%**	**0.00%**

Typical seqs	Reads	Type	pct.
CTGCCTACCGGCACGCA|TGC**AGG**ATCA	13591	WT	99.71%
CTGCCTACCGGCACGCAaTGCAGGATCA	28	+1 a	0.21%
CTGCCTACCGGCACGCAtTGCAGGATCA	8	+1 t	0.06%
CTGCCTACCGGCACGCAgTGCAGGATCA	4	+1 g	0.03%

Total | INDEL	12h	Ins	Del	HDR
7473 | 0.55%		**0.48%**	**0.07%**	**0.00%**

Typical seqs	Reads	Type	pct.
CTGCCTACCGGCACGCA|TGC**AGG**ATCA	7432	WT	99.45%
CTGCCTACCGGCACGCAaTGCAGGATCA	22	+1 a	0.29%
CTGCCTACCGGCACGCAtTGCAGGATCA	14	+1 t	0.19%
CTGCCTACCGGCAC----GCAGGATCA	5	-4	0.07%

Total | INDEL	24h	Ins	Del	HDR
13315 | 2.78%		**1.63%**	**1.15%**	**0.00%**

Typical seqs	Reads	Type	pct.
CTGCCTACCGGCACGCA|TGC**AGG**ATCA	12945	WT	97.22%
CTGCCTACCGGCACGCAaTGCAGGATCA	127	+1 a	0.95%
CTGCCTACCGGCACGCAtTGCAGGATCA	67	+1 t	0.50%
CTGCCTACCGGCAC----GCAGGATCA	44	-4	0.33%
CTGCCTACCG--------GCAGGATCA	21	-8	0.16%
CTGCCTACCGGCACGCAgTGCAGGATCA	12	+1 g	0.09%
-----------------TGCAGGATCA	11	-19	0.08%
CTGCCTACCG----GCATGCAGGATCA	11	-4	0.08%
C----------------TGCAGGATCA	11	-16	0.08%
CTGCCTACCGGCACGCA--CAGGATCA	8	-2	0.06%

Total | INDEL	48h	Ins	Del	HDR
16881 | 4.58%		**2.36%**	**2.22%**	**0.00%**

Typical seqs	Reads	Type	pct.
CTGCCTACCGGCACGCA|TGC**AGG**ATCA	16108	WT	95.42%
CTGCCTACCGGCACGCAaTGCAGGATCA	252	+1 a	1.49%
CTGCCTACCGGCAC----GCAGGATCA	128	-4	0.76%
CTGCCTACCGGCACGCAtTGCAGGATCA	115	+1 t	0.68%
CTGCCTACCG--------GCAGGATCA	60	-8	0.36%
C----------------TGCAGGATCA	23	-16	0.14%
-----------------TGCAGGATCA	18	-19	0.11%
CTGCCTACCGGCACGCA-GCAGGATCA	14	-1	0.08%
C--------------------------	14	-30	0.08%
CTGCCTACCG----GCATGCAGGATCA	12	-4	0.07%

P143-P147-YW(WT)-K562-RNP-KO-Syn91crBCL11A7a

Total | INDEL	4h	Ins	Del	HDR
14828 | 0.79%		**0.67%**	**0.12%**	**0.00%**

Typical seqs	Reads	Type	pct.
TGACACTTACCGCGTAT|GAG**GGG**TCTT	14711	WT	99.21%
TGACACTTACCGCGTATtGAGGGGTCTT	96	+1 t	0.65%
TGACACTTACCGCGTATGA-GGGTCTT	10	-1	0.07%
TGACACT-------TATGAGGGGTCTT	8	-7	0.05%
TGGCACTTACCGCGTATTGAGGGGTCTT	3	+1 Ins	0.02%

Total | INDEL	8h	Ins	Del	HDR
18012 | 2.11%		**1.55%**	**0.56%**	**0.00%**

Typical seqs	Reads	Type	pct.
TGACACTTACCGCGTAT|GAG**GGG**TCTT	17632	WT	97.89%
TGACACTTACCGCGTATtGAGGGGTCTT	263	+1 t	1.46%
TGACACTTACCGCGTAT-----GTCTT	14	-5	0.08%
TGACACTTACCGCGTATGA-GGGTCTT	14	-1	0.08%
TGACACT-------TATGAGGGGTCTT	8	-7	0.04%
TGACACTTACCGCGTA----------T	6	-10	0.03%
TGACACTTAC------TGAGGGGTCTT	6	-6	0.03%
TGACGCTTACCGCGTATTGAGGGGTCTT	5	+1 Ins	0.03%
TGACACTTACCGCGTA--AGGGGTCTT	5	-2	0.03%
TGACACTTACCGCGTATG-GGGGTCTT	5	-1	0.03%

Total | INDEL	12h	Ins	Del	HDR
12804 | 2.98%		**2.17%**	**0.80%**	**0.00%**

Typical seqs	Reads	Type	pct.
TGACACTTACCGCGTAT|GAG**GGG**TCTT	12423	WT	97.02%
TGACACTTACCGCGTATtGAGGGGTCTT	258	+1 t	2.01%
TGACACTTACCGCGTAT-----GTCTT	12	-5	0.09%
TGACACTTACCGC---------GTCTT	10	-9	0.08%
TGACACT-------TATGAGGGGTCTT	9	-7	0.07%
TGACACTTACCGCGTATGA-GGGTCTT	8	-1	0.06%
TGACACT---------TGAGGGGTCTT	8	-9	0.06%
TGACACTTACCGCGTA--AGGGGTCTT	8	-2	0.06%
TGACACTTAC------TGAGGGGTCTT	7	-6	0.05%
TGACACTTACC-----TGAGGGGTCTT	5	-5	0.04%

Total | INDEL	24h	Ins	Del	HDR
18512 | 6.48%		**3.63%**	**2.85%**	**0.00%**

Typical seqs	Reads	Type	pct.
TGACACTTACCGCGTAT|GAG**GGG**TCTT	17313	WT	93.52%
TGACACTTACCGCGTATtGAGGGGTCTT	622	+1 t	3.36%
TGACACTTACCGC---------GTCTT	32	-9	0.17%
TGACA-------------------CTT	28	-19	0.15%
TGACACTTACCGCGTA--AGGGGTCTT	27	-2	0.15%
TGACACTT-------------------	30	-24	0.16%
TGACACTTAC------TGAGGGGTCTT	20	-6	0.11%
TGACACT-------TATGAGGGGTCTT	20	-7	0.11%
TGACACTTACCGCGTA-----AGTCTT	20	-5	0.11%
TGACACTTACCGCGTAT-AGGGGTCTT	18	-1	0.10%

Total | INDEL	48h	Ins	Del	HDR
27889 | 10.38%		**5.35%**	**5.03%**	**0.00%**

Typical seqs	Reads	Type	pct.
TGACACTTACCGCGTAT|GAG**GGG**TCTT	24995	WT	89.62%
TGACACTTACCGCGTATtGAGGGGTCTT	1247	+1 t	4.47%
TGACACTTACCGC---------GTCTT	107	-9	0.38%
TGACACTTACCGCGTA--AGGGGTCTT	71	-2	0.25%
TGACACTT-------------------	79	-24	0.28%
TGACA-------------------CTT	70	-19	0.25%
TGACACTTACCGCGT---AAGTGTCTT	51	-3	0.18%
TGACACT-------TATGAGGGGTCTT	41	-7	0.15%
TGACACT----------------TCTT	34	-16	0.12%
-------------------------TT	33	-28	0.12%

P143-P147-YW(WT)-K562-RNP-KO-Syn92crBCL11A7b-BC1

Total | INDEL	4h	Ins	Del	HDR
31254 | 2.11%		1.62%	0.50%	0.00%

Typical seqs	Reads	Type	pct.
GCTTCGTGACGTTGGAT|GGA**GGG**TTTT	30594	WT	97.89%
GCTTCGTGACGTTGGATtGGAGGGTTTT	457	+1 t	1.46%
GCTTCGTGACGTT----GGAGGGTTTT	42	-4	0.13%
GCTTCGTGACGTTGGAATGGAGGGTTTT	32	+1 Ins	0.10%
GCTTCGTGACGTTGGA-GGAGGGTTTT	30	-1	0.10%
GCTTCGTGACGTTGGAT-GAGGGTTTT	20	-1	0.06%
GCTTCGTGACGTTG--TGGAGGGTTTT	14	-2	0.04%
GCTTCGTGACGTTGGATATGGAGGGTTTT	8	+2	0.03%
GCTTCGTGACGTTGGATGG-GGGTTTT	6	-1	0.02%
GCTTCGTGACGTTGGA--GAGGGTTTT	6	-2	0.02%

Total | INDEL	8h	Ins	Del	HDR
46573 | 4.42%		3.18%	1.23%	0.00%

Typical seqs	Reads	Type	pct.
GCTTCGTGACGTTGGAT|GGA**GGG**TTTT	44515	WT	95.58%
GCTTCGTGACGTTGGATtGGAGGGTTTT	1254	+1 t	2.69%
GCTTCGTGACGTT----GGAGGGTTTT	228	-4	0.49%
GCTTCGTGACGTTGGAATGGAGGGTTTT	107	+1 Ins	0.23%
GCTTCGTGACGTTGGA-GGAGGGTTTT	98	-1	0.21%
GCTTCGTGACGTTG--TGGAGGGTTTT	44	-2	0.09%
GCTTCG----------TGGAGGGTTTT	22	-10	0.05%
GCTTCGTGACGTTGG-TGGAGGGTTTT	16	-1	0.03%
GCCTCGTGACGTTGGATTGGAGGGTTTT	14	+1 Ins	0.03%
GCTTCGTGACGTTGGAT-GAGGGTTTT	13	-1	0.03%

Total | INDEL	12h	Ins	Del	HDR
40896 | 7.58%		4.96%	2.61%	0.00%

Typical seqs	Reads	Type	pct.
GCTTCGTGACGTTGGAT|GGA**GGG**TTTT	37797	WT	92.42%
GCTTCGTGACGTTGGATtGGAGGGTTTT	1759	+1 t	4.30%
GCTTCGTGACGTT----GGAGGGTTTT	474	-4	1.16%
GCTTCGTGACGTTGGAATGGAGGGTTTT	100	+1 Ins	0.24%
GCTTCGTGACGTTG--TGGAGGGTTTT	93	-2	0.23%
GCTTCGTGACGTTGGA-GGAGGGTTTT	71	-1	0.17%
GCTTCG----------TGGAGGGTTTT	36	-10	0.09%
GCTTCGTGACGTTGGAT-GAGGGTTTT	28	-1	0.07%
GCTTCGTGACGTTGGA--------TTT	22	-8	0.05%
GCTTCGTGACGTT-------GGGTTTT	19	-7	0.05%

Total | INDEL	24h	Ins	Del	HDR
21165 | 22.15%		9.09%	13.06%	0.00%

Typical seqs	Reads	Type	pct.
GCTTCGTGACGTTGGAT|GGA**GGG**TTTT	16478	WT	77.85%
GCTTCGTGACGTT----GGAGGGTTTT	1836	-4	8.67%
GCTTCGTGACGTTGGATtGGAGGGTTTT	1588	+1 t	7.50%
GCTTCGTGACGTTGGATcGGAGGGTTTT	145	+1 c	0.69%
GCTTCGTGACGTT-------GGGTTTT	107	-7	0.51%
GCTTCGTGACGTTGGAATGGAGGGTTTT	93	+1 Ins	0.44%
GCTTCGTGACGTTGGA-GGAGGGTTTT	73	-1	0.34%
GCTTCGTGAC------------GTTTT	65	-12	0.31%
GCTTCGTGACGTTG--TGGAGGGTTTT	50	-2	0.24%
GCTTCG----------TGGAGGGTTTT	41	-10	0.19%

Total | INDEL	48h	Ins	Del	HDR
91069 | 29.65%		9.74%	19.90%	0.00%

Typical seqs	Reads	Type	pct.
GCTTCGTGACGTTGGAT|GGA**GGG**TTTT	64070	WT	70.35%
GCTTCGTGACGTT----GGAGGGTTTT	11239	-4	12.34%
GCTTCGTGACGTTGGATtGGAGGGTTTT	7449	+1 t	8.18%
GCTTCGTGACGTT-------GGGTTTT	709	-7	0.78%
GCTTCGTGACGTTGGAATGGAGGGTTTT	498	+1 Ins	0.55%
GCTTCGTGACGTTGGA-GGAGGGTTTT	425	-1	0.47%
GCTTCGTGAC------------GTTTT	358	-12	0.39%
GCTTCGTGACGTTG--TGGAGGGTTTT	328	-2	0.36%
GCTTCGTGACGTT--------GGTTTT	284	-8	0.31%
GCTTCG----------TGGAGGGTTTT	225	-10	0.25%

P143-P147-YW(WT)-K562-RNP-KO-Syn93crBCL11A7c-BC2

Total	INDEL	4h	Ins	Del	HDR
49101	1.52%		0.92%	0.60%	0.00%

Typical seqs	Reads	Type	pct.
AGCTTCGTGACGTTGGA|TGG**AGG**GTTT	48357	WT	98.48%
AGCTTCGTGACGTTGGAtTGGAGGGTTT	334	+1 t	0.68%
AGCTTCGTGACGTTGGAaTGGAGGGTTT	102	+1 a	0.21%
AGCTTCGTGACGT----TGGAGGGTTT	102	-4	0.21%
AGCTTCGTGACGTTGGA-GGAGGGTTT	63	-1	0.13%
AGCTTCGTGACGTTGGA--GAGGGTTT	19	-2	0.04%
AGCTTCGTGACGTTG--TGGAGGGTTT	18	-2	0.04%
AGCTTCGTGACGTTGGAT-GAGGGTTT	17	-1	0.03%
AGCTTCG----------TGGAGGGTTT	10	-10	0.02%
AGCTTCGTGACGTTGGA----------	13	-13	0.03%

Total	INDEL	8h	Ins	Del	HDR
30593	4.09%		2.76%	1.33%	0.00%

Typical seqs	Reads	Type	pct.
AGCTTCGTGACGTTGGA|TGG**AGG**GTTT	29343	WT	95.91%
AGCTTCGTGACGTTGGAtTGGAGGGTTT	663	+1 t	2.17%
AGCTTCGTGACGT----TGGAGGGTTT	172	-4	0.56%
AGCTTCGTGACGTTGGAaTGGAGGGTTT	104	+1 a	0.34%
AGCTTCGTGACGTTGGA-GGAGGGTTT	76	-1	0.25%
AGCTTCGTGACGTTG--TGGAGGGTTT	37	-2	0.12%
AGCTTCGTGACGTTGGA--GAGGGTTT	16	-2	0.05%
AGCTTCG----------TGGAGGGTTT	13	-10	0.04%
AGCTTCGTGACGTTGG-TGGAGGGTTT	11	-1	0.04%
AGCCTCGTGACGTTGGATTGGAGGGTTT	10	+1 Ins	0.03%

Total	INDEL	12h	Ins	Del	HDR
16323	6.78%		3.39%	3.39%	0.00%

Typical seqs	Reads	Type	pct.
AGCTTCGTGACGTTGGA|TGG**AGG**GTTT	15217	WT	93.22%
AGCTTCGTGACGTTGGAtTGGAGGGTTT	446	+1 t	2.73%
AGCTTCGTGACGT----TGGAGGGTTT	295	-4	1.81%
AGCTTCGTGACGTTGGAaTGGAGGGTTT	72	+1 a	0.44%
AGCTTCGTGACGTTG--TGGAGGGTTT	50	-2	0.31%
AGCTTCGTGACGTTGGA-GGAGGGTTT	52	-1	0.32%
AGCTTCGTGACGTTGG-TGGAGGGTTT	15	-1	0.09%
AGCTTCGTGACGTTGGA--------TT	12	-8	0.07%
AGCTTCGT-------GATGGAGGGTTT	11	-7	0.07%
AGCTTCGTGACGTTGGA--GAGGGTTT	10	-2	0.06%

Total	INDEL	24h	Ins	Del	HDR
10623	21.13%		6.54%	14.59%	0.00%

Typical seqs	Reads	Type	pct.
AGCTTCGTGACGTTGGA|TGG**AGG**GTTT	8378	WT	78.87%
AGCTTCGTGACGT----TGGAGGGTTT	1034	-4	9.73%
AGCTTCGTGACGTTGGAtTGGAGGGTTT	538	+1 t	5.06%
AGCTTCGTGACGTTGGAaTGGAGGGTTT	79	+1 a	0.74%
AGCTTCGTGACGTTGGA-GGAGGGTTT	53	-1	0.50%
AGCTTCGTGACGTTGGAtCGGAGGGTTT	57	+1 t	0.54%
AGCTTCGTGACGTT-------GGGTTT	44	-7	0.41%
AGCTTCGTGAC------------GTTT	41	-12	0.39%
AGCTTCGTGACGTTG--TGGAGGGTTT	33	-2	0.31%
AGCTTCGTGACGTT--------GGTTT	29	-8	0.27%

Total	INDEL	48h	Ins	Del	HDR
45987	28.51%		8.35%	20.16%	0.00%

Typical seqs	Reads	Type	pct.
AGCTTCGTGACGTTGGA|TGG**AGG**GTTT	32876	WT	71.49%
AGCTTCGTGACGT----TGGAGGGTTT	6016	-4	13.08%
AGCTTCGTGACGTTGGAtTGGAGGGTTT	3136	+1 t	6.82%
AGCTTCGTGACGTTGGAaTGGAGGGTTT	368	+1 a	0.80%
AGCTTCGTGACGTT-------GGGTTT	313	-7	0.68%
AGCTTCGTGACGTTGGA-GGAGGGTTT	260	-1	0.57%
AGCTTCGTGACGTTG--TGGAGGGTTT	168	-2	0.37%
AGCTTCGTGAC------------GTTT	163	-12	0.35%
AGCTTCGTGACGTT--------GGTTT	108	-8	0.23%
AGCTTCG----------TGGAGGGTTT	107	-10	0.23%

P143-P147-YW(WT)-K562-RNP-KO-Syn94crBCL11A7d

4h

Total | INDEL	Ins	Del	HDR
8038 | 1.85%	1.58%	0.27%	0.00%

Typical seqs	Reads	Type	pct.
GCGTAGTGTTGGGTCCT|ACC**TGG**CCAC	7889	WT	98.15%
GCGTAGTGTTGGGTCCTtACCTGGCCAC	116	+1 t	1.44%
GCGTAGTGTTGGGT----CCTGGCCAC	8	-4	0.10%
GCGTAGTGTTGGGTCCTACCT-GCCAC	4	-1	0.05%
GCGTAGTGTTGGGTCCTCTACCTGGCCAC	4	+2	0.05%
GCGTAGTGTCGGGTCCTTACCTGGCCAC	4	+1 Ins	0.05%
GCGTAGTGTTGGGTCCTaACCTGGCCAC	3	+1 a	0.04%
GCGTAGTGTTGGGT--TACCTGGCCAC	3	-2	0.04%
GCGTAGTGTTGGGTCCT-----GCCAC	3	-5	0.04%
Total | INDEL	Ins	Del	HDR

8h

Total | INDEL	Ins	Del	HDR
8092 | 4.82%	4.20%	0.62%	0.00%

Typical seqs	Reads	Type	pct.
GCGTAGTGTTGGGTCCT|ACC**TGG**CCAC	7702	WT	95.18%
GCGTAGTGTTGGGTCCTtACCTGGCCAC	322	+1 t	3.98%
GCGTAGTGTTGGGT----CCTGGCCAC	15	-4	0.19%
GCGTAGTGTTGGGTCCTCTACCTGGCCAC	9	+2	0.11%
GCGTAGTGTTGGGT---ACCTGGCCAC	8	-3	0.10%
GCGTAGTGTTGGGTCC-ACCTGGCCAC	7	-1	0.09%
GCGTAGTGTTGGGTC-----------C	6	-11	0.07%
GCGTAGTGTTGGGTCCTACCT-GCCAC	5	-1	0.06%
GCGTAGTGTTGGGTCCTtGCCTGGCCAC	5	+1 t	0.06%
GCGTAGTGTTGGGTCCTaACCTGGCCAC	4	+1 a	0.05%

12h

Total | INDEL	Ins	Del	HDR
7217 | 6.86%	5.42%	1.44%	0.00%

Typical seqs	Reads	Type	pct.
GCGTAGTGTTGGGTCCT|ACC**TGG**CCAC	6722	WT	93.14%
GCGTAGTGTTGGGTCCTtACCTGGCCAC	370	+1 t	5.13%
GCGTAGTGTTGGGT----CCTGGCCAC	37	-4	0.51%
GCGTAGTGTTGGGTC--ACCTGGCCAC	18	-2	0.25%
GCGTAGTGTTGGGTCC-ACCTGGCCAC	8	-1	0.11%
GCGTAGTGTTGGGT---ACCTGGCCAC	8	-3	0.11%
GCGTAGTGTTGGGTCCTaACCTGGCCAC	8	+1 a	0.11%
GCGTAGTGTTGGGTCCTCTACCTGGCCAC	7	+2	0.10%
GCGTAGTGTTGGGTC-----------C	7	-11	0.10%
GCGTAGTGTTGGGTCCTACCT-GCCAC	5	-1	0.07%

24h

Total | INDEL	Ins	Del	HDR
2645 | 17.73%	10.06%	7.67%	0.00%

Typical seqs	Reads	Type	pct.
GCGTAGTGTTGGGTCCT|ACC**TGG**CCAC	2176	WT	82.27%
GCGTAGTGTTGGGTCCTtACCTGGCCAC	258	+1 t	9.75%
GCGTAGTGTTGGGT----CCTGGCCAC	107	-4	4.05%
GCGTAGTGTTGGGT----------CAC	14	-10	0.53%
GCGTAGTGT-----------TGGCCAC	14	-11	0.53%
GCGTAGTGTTGGGTC--ACCTGGCCAC	12	-2	0.45%
GCGTAGTGTTGGGTCC-ACCTGGCCAC	9	-1	0.34%
GCGTAGTGTTG----------GGCCAC	7	-10	0.26%
GCGTAGTGTTGGGTCCT--------AC	6	-8	0.23%
GCGTAGTGTTGGGTC-TACCTGGCCAC	6	-1	0.23%

48h

Total | INDEL	Ins	Del	HDR
16665 | 30.74%	13.73%	17.01%	0.00%

Typical seqs	Reads	Type	pct.
GCGTAGTGTTGGGTCCT|ACC**TGG**CCAC	11543	WT	69.26%
GCGTAGTGTTGGGTCCTtACCTGGCCAC	1981	+1 t	11.89%
GCGTAGTGTTGGGT----CCTGGCCAC	879	-4	5.27%
GCGTAGTGT-----------TGGCCAC	266	-11	1.60%
GCGTAGTGTTGGGT----------CAC	137	-10	0.82%
GCGTAGTG-------------------	124	-19	0.74%
GCGTAGTGTTG----------GGCCAC	106	-10	0.64%
GCGTAGTGTTGGGTC-----------C	98	-11	0.59%
GCGTAGTGTTGGGTC--ACCTGGCCAC	87	-2	0.52%
GCGTAGTGTTGGGTCC-ACCTGGCCAC	68	-1	0.41%

P143-P147-YW(WT)-K562-RNP-KO-Syn95crBCL11A8a

Total | INDEL	4h	Ins	Del	HDR
70864 | 0.40%		0.26%	0.14%	0.00%

Typical seqs	Reads	Type	pct.
GCTTCTGAGCGTCAAAC|TCGAGGAGCA	70580	WT	99.60%
GCTTCTGAGCGTCAAACcTCGAGGAGCA	108	+1 c	0.15%
GCTTCTGAGCGTCAAACtTCGAGGAGCA	49	+1 t	0.07%
GCTTCTGAGCGTCAAA--CGAGGAGCA	15	-2	0.02%
GCTTCTGAGCGTCAAAC-CGAGGAGCA	12	-1	0.02%
GCTTCTGAGCGTCAA--TCGAGGAGCA	10	-2	0.01%
GCTTCTGAGCG------TCGAGGAGCA	9	-6	0.01%
GCTTCTGAGCGTCAAACACTCGAGGAGCA	7	+2	0.01%
GCTTCTGAGCG-----------------	7	-18	0.01%
GCTTCTGAGCGTCAAACgTCGAGGAGCA	6	+1 g	0.01%

Total | INDEL	8h	Ins	Del	HDR
106013 | 1.67%		0.74%	0.93%	0.00%

Typical seqs	Reads	Type	pct.
GCTTCTGAGCGTCAAAC|TCGAGGAGCA	104239	WT	98.33%
GCTTCTGAGCGTCAAACcTCGAGGAGCA	429	+1 c	0.40%
GCTTCTGAGCGTCAAACtTCGAGGAGCA	233	+1 t	0.22%
GCTTCTGAGCGTCAA--TCGAGGAGCA	105	-2	0.10%
GCTTCTGAGCG------TCGAGGAGCA	108	-6	0.10%
GCTTCTGAGCGTCAAAC-CGAGGAGCA	104	-1	0.10%
GCTTCTGAGCGTCAAA--CGAGGAGCA	96	-2	0.09%
GCTTCT----------------GAGCA	62	-16	0.06%
GCTTCTGAGCGTCAAAC-------GCA	48	-7	0.05%
GCTTCTGAGCGTCAAACgTCGAGGAGCA	36	+1 g	0.03%

Total | INDEL	12h	Ins	Del	HDR
39569 | 2.01%		0.87%	1.14%	0.00%

Typical seqs	Reads	Type	pct.
GCTTCTGAGCGTCAAAC|TCGAGGAGCA	38772	WT	97.99%
GCTTCTGAGCGTCAAACcTCGAGGAGCA	222	+1 c	0.56%
GCTTCTGAGCGTCAAACtTCGAGGAGCA	87	+1 t	0.22%
GCTTCTGAGCG------TCGAGGAGCA	69	-6	0.17%
GCTTCTGAGCGTCAAAC-CGAGGAGCA	49	-1	0.12%
GCTTCTGAGCGTCAAA--CGAGGAGCA	36	-2	0.09%
GCTTCTGAGCGTCAA--TCGAGGAGCA	26	-2	0.07%
GCTTCTGAGCGTCAAAC----GGAGCA	25	-4	0.06%
GCTTCT----------------GAGCA	25	-16	0.06%
GCTTCTGAGCGTCAAA-TCGAGGAGCA	16	-1	0.04%

Total | INDEL	24h	Ins	Del	HDR
110890 | 6.67%		2.10%	4.57%	0.00%

Typical seqs	Reads	Type	pct.
GCTTCTGAGCGTCAAAC|TCGAGGAGCA	103489	WT	93.33%
GCTTCTGAGCGTCAAACcTCGAGGAGCA	1302	+1 c	1.17%
GCTTCTGAGCG------TCGAGGAGCA	768	-6	0.69%
GCTTCTGAGCGTCAAACtTCGAGGAGCA	596	+1 t	0.54%
GCTTCT----------------GAGCA	444	-16	0.40%
GCTTCTGAGCGTCAAA--CGAGGAGCA	357	-2	0.32%
GCTTCTGAGCGTCAA--TCGAGGAGCA	258	-2	0.23%
GCTTCTGAGCGTCAAAC-CGAGGAGCA	198	-1	0.18%
GCT--------------TCGAGGAGCA	175	-14	0.16%
GCTTCTGAGCGTCAAAC-------GCA	155	-7	0.14%

Total | INDEL	48h	Ins	Del	HDR
126878 | 11.97%		3.17%	8.80%	0.00%

Typical seqs	Reads	Type	pct.
GCTTCTGAGCGTCAAAC|TCGAGGAGCA	111695	WT	88.03%
GCTTCTGAGCGTCAAACcTCGAGGAGCA	2318	+1 c	1.83%
GCTTCTGAGCG------TCGAGGAGCA	1828	-6	1.44%
GCTTCT----------------GAGCA	1023	-16	0.81%
GCTTCTGAGCGTCAAACtTCGAGGAGCA	849	+1 t	0.67%
GCTTCTGAGCGTCAAA--CGAGGAGCA	744	-2	0.59%
GCTTCTGAGCGTCAAAC-CGAGGAGCA	420	-1	0.33%
GCT--------------TCGAGGAGCA	365	-14	0.29%
GCTTCTGAGCGTCAA--TCGAGGAGCA	397	-2	0.31%
GCTTCT-------------GAGGAGCA	243	-13	0.19%

P143-P147-YW(WT)-K562-RNP-KO-Syn96crBCL11A8b

Total | INDEL	4h	Ins	Del	HDR
8473 | 0.37%		0.07%	0.30%	0.00%

Typical seqs	Reads	Type	pct.
GCGGGCCACCCACATTC|ATT**GGG**GGAA	8442	WT	99.63%
GCGGGCCACCCAC----ATTGGGGGAA	15	-4	0.18%
GCGGGCCACCCACATTC----GGGGAA	4	-4	0.05%
GCGGGCCACCCACATTCcATTGGGGGAA	3	+1 c	0.04%
GCGGGCCACCCACATTCaATTGGGGGAA	3	+1 a	0.04%
GCGGGCCACCCACATTC--TGGGGGAA	3	-2	0.04%
GCGGGCCACCCACATTC-TTGGGGGAA	3	-1	0.04%

Total | INDEL	8h	Ins	Del	HDR
25706 | 2.05%		0.12%	1.93%	0.00%

Typical seqs	Reads	Type	pct.
GCGGGCCACCCACATTC|ATT**GGG**GGAA	25178	WT	97.95%
GCGGGCCACCCAC----ATTGGGGGAA	181	-4	0.70%
GCGGGCCACCCACATTC-TTGGGGGAA	39	-1	0.15%
GCGGGCCACCCACATTC--TGGGGGAA	29	-2	0.11%
GCGGGCCA--------CATTGGGGGAA	23	-8	0.09%
GCGGGCCACCCACATT-ATTGGGGGAA	20	-1	0.08%
GCGGGCCACCCACATTCaATTGGGGGAA	20	+1 a	0.08%
GCGGGC----------CATTGGGGGAA	16	-10	0.06%
GCGGGCCACCCACAT--ATTGGGGGAA	14	-2	0.05%
GCGGGCCACCCACATTC---GGGGGAA	13	-3	0.05%

Total | INDEL	12h	Ins	Del	HDR
13924 | 4.30%		0.22%	4.09%	0.00%

Typical seqs	Reads	Type	pct.
GCGGGCCACCCACATTC|ATT**GGG**GGAA	13325	WT	95.70%
GCGGGCCACCCAC----ATTGGGGGAA	220	-4	1.58%
GCGGGCCACCCACATTC-TTGGGGGAA	36	-1	0.26%
GCGGGCCACC-----ACATTGGGGGAA	22	-5	0.16%
GCGGGCCA--------CATTGGGGGAA	25	-8	0.18%
GCGGGCCACCCACATTC---GGGGGAA	18	-3	0.13%
GCGGGCCACCCACATT-ATTGGGGGAA	17	-1	0.12%
GCGGGCCACCCACATTC----GGGGAA	17	-4	0.12%
GCGGGCCACCCACATTC----------	29	-11	0.21%
GCGGGCCACCCACATTC--TGGGGGAA	13	-2	0.09%

Total | INDEL	24h	Ins	Del	HDR
7188 | 19.32%		0.54%	18.78%	0.00%

Typical seqs	Reads	Type	pct.
GCGGGCCACCCACATTC|ATT**GGG**GGAA	5799	WT	80.68%
GCGGGCCACCCAC----ATTGGGGGAA	731	-4	10.17%
GCGGGCCACCCACATTC-TTGGGGGAA	50	-1	0.70%
GCGGGCCACC-----ACATTGGGGGAA	42	-5	0.58%
GCGGGCCACCCACATT-ATTGGGGGAA	31	-1	0.43%
GCGGGCCACCCACATTC---GGGGGAA	26	-3	0.36%
GCGGGCCA--------CATTGGGGGAA	26	-8	0.36%
GCGGGCCACCCACATTCaATTGGGGGAA	21	+1 a	0.29%
GCGGGCCACCCACATTC--TGGGGGAA	21	-2	0.29%
GCGGGCCACCCAC--------------	21	-20	0.29%

Total | INDEL	48h	Ins	Del	HDR
35139 | 34.63%		0.92%	33.70%	0.00%

Typical seqs	Reads	Type	pct.
GCGGGCCACCCACATTC|ATT**GGG**GGAA	22972	WT	65.37%
GCGGGCCACCCAC----ATTGGGGGAA	6263	-4	17.82%
GC--------------------GGGAA	249	-20	0.71%
GCGGGCCACCCACATTC-TTGGGGGAA	228	-1	0.65%
GCGGGCC--------------------	314	-23	0.89%
GCGGGCCACC-----ACATTGGGGGAA	208	-5	0.59%
GCGGGCCACCC----------------	221	-19	0.63%
GCGGGCCACCCACATT-ATTGGGGGAA	191	-1	0.54%
GCGGGCCACCCAC--------------	203	-20	0.58%
GC-------------------GGGGAA	145	-19	0.41%

P143-P147-YW(WT)-K562-RNP-KO-Syn97crBCL11A8c

Total | INDEL	4h	Ins	Del	HDR
21231 | 0.15%		0.02%	0.12%	0.00%

Typical seqs	Reads	Type	pct.
CGCGGCAGAAACCTGTC|GTC**TGG**ACAC	21200	WT	99.85%
CGCGGCAGAAACCT---GTCTGGACAC	21	-3	0.10%
CGCGGCAGAAACCTGT-GTCTGGACAC	5	-1	0.02%
CGCGGCAGAAACCTGTCcGTCTGGACAC	5	+1 c	0.02%

Total | INDEL	8h	Ins	Del	HDR
31443 | 0.67%		0.04%	0.63%	0.00%

Typical seqs	Reads	Type	pct.
CGCGGCAGAAACCTGTC|GTC**TGG**ACAC	31232	WT	99.33%
CGCGGCAGAAACCT---GTCTGGACAC	100	-3	0.32%
CGCGGCAGAAACCTGTCGTCT-GACAC	23	-1	0.07%
CGCGGCAGAAACCTGTCcGTCTGGACAC	12	+1 c	0.04%
CGCGGCAGAAACCTGT-GTCTGGACAC	10	-1	0.03%
CGCGGCAGAAAC-------CTGGACAC	8	-7	0.03%
CGCGGCAGAAA-----CGTCTGGACAC	7	-5	0.02%
CGCGGCAGAAACCTG--GTCTGGACAC	8	-2	0.03%
CGCGGCAGAAAC--------------C	4	-14	0.01%
CGCGGCAGAAACCTGTC---------C	4	-9	0.01%

Total | INDEL	12h	Ins	Del	HDR
17217 | 0.98%		0.09%	0.89%	0.00%

Typical seqs	Reads	Type	pct.
CGCGGCAGAAACCTGTC|GTC**TGG**ACAC	17048	WT	99.02%
CGCGGCAGAAACCT---GTCTGGACAC	73	-3	0.42%
CGCGGCAGAAACCTGTCcGTCTGGACAC	15	+1 c	0.09%
CGCGGCAGAAACCTGTCGTCT-GACAC	13	-1	0.08%
CGCGGCAGAAACCTGTC----------	13	-12	0.08%
CGCGGCAGAAACCTGT-GTCTGGACAC	9	-1	0.05%
CGCGGCAGAAACC--TCGTCTGGACAC	6	-2	0.03%
CGCGGCAGAAA-----CGTCTGGACAC	6	-5	0.03%
CGCGGCAGAAACCT-------------	6	-15	0.03%
CGCGGCAGAAACCTGTC-------CAC	4	-7	0.02%

Total | INDEL	24h	Ins	Del	HDR
32019 | 2.41%		0.12%	2.29%	0.00%

Typical seqs	Reads	Type	pct.
CGCGGCAGAAACCTGTC|GTC**TGG**ACAC	31247	WT	97.59%
CGCGGCAGAAACCT---GTCTGGACAC	446	-3	1.39%
CGCGGCAGAAAC-------CTGGACAC	49	-7	0.15%
CGCGGCAGAAACCTGTCcGTCTGGACAC	31	+1 c	0.10%
CGCGGCAGAAACCT-------------	29	-15	0.09%
CGCGGCAGAAACCTG--GTCTGGACAC	15	-2	0.05%
CGCGGCAGAAACCTGTC----------	21	-11	0.07%
CGCGGCAGAAACCTGT-GTCTGGACAC	14	-1	0.04%
CGCGGCAGAAA-----CGTCTGGACAC	10	-5	0.03%
CGCGGCAGAAACCTGT--------CAC	10	-8	0.03%

Total | INDEL	48h	Ins	Del	HDR
60845 | 3.84%		0.22%	3.62%	0.00%

Typical seqs	Reads	Type	pct.
CGCGGCAGAAACCTGTC|GTC**TGG**ACAC	58509	WT	96.16%
CGCGGCAGAAACCT---GTCTGGACAC	1242	-3	2.04%
CGCGGCAGAAAC-------CTGGACAC	109	-7	0.18%
CGCGGCAGAAACCTGTCcGTCTGGACAC	77	+1 c	0.13%
CGCGGCAGAAACCT-------------	77	-15	0.13%
CGCGGCAGAAACCTGTCGTCT-GACAC	41	-1	0.07%
CGCGGCAGAAA-----CGTCTGGACAC	40	-5	0.07%
CGCGGCAGAA---------------AC	32	-15	0.05%
CGCGGCAGAA-------------ACAC	27	-13	0.04%
CGCGGCAGAAACCTGTC----------	64	-12	0.11%

P143-P147-YW(WT)-K562-RNP-KO-Syn98crBCL11A8d

Total | INDEL	4h	Ins	Del	HDR
68971 | 0.27%		0.12%	0.15%	0.00%

Typical seqs	Reads	Type	pct.
GCCTTGATTACTCAAAC|AAC**AGG**TTAC	68783	WT	99.73%
GCCTTGATTACTCAAACcAACAGGTTAC	76	+1 c	0.11%
GCCTTGATTACTCA---AACAGGTTAC	42	-3	0.06%
GCCTTGATTACTCAA--AACAGGTTAC	14	-2	0.02%
GCCTTGATTACT----CAACAGGTTAC	11	-4	0.02%
GCCTTGATTACTC-AACAACAGGTTAC	7	-1	0.01%
GCCTTGATT------ACAACAGGTTAC	5	-6	0.01%
GCCTTGATTACTCAAAC--CAGGTTAC	5	-2	0.01%
GCCT-----------------GGTTAC	4	-17	0.01%
GCCTTGA----------AACAGGTTAC	3	-10	0.00%

Total | INDEL	8h	Ins	Del	HDR
84382 | 0.96%		0.32%	0.64%	0.00%

Typical seqs	Reads	Type	pct.
GCCTTGATTACTCAAAC|AAC**AGG**TTAC	83571	WT	99.04%
GCCTTGATTACTCAAACcAACAGGTTAC	241	+1 c	0.29%
GCCTTGATTACTCA---AACAGGTTAC	218	-3	0.26%
GCCTTGATTACT----CAACAGGTTAC	27	-4	0.03%
GCCTTGATTACTCAA----------AC	27	-10	0.03%
GCCTTGATTACTCAA--AACAGGTTAC	15	-2	0.02%
GCCTTGATTACTC---CAACAGGTTAC	15	-3	0.02%
GCCTTGATTACTCAAAC-ACAGGTTAC	14	-1	0.02%
GCCTTGATT---------ACAGGTTAC	14	-9	0.02%
GCCTTGATTACTCAAAC-------TAC	14	-7	0.02%

Total | INDEL	12h	Ins	Del	HDR
37872 | 1.19%		0.39%	0.80%	0.00%

Typical seqs	Reads	Type	pct.
GCCTTGATTACTCAAAC|AAC**AGG**TTAC	37422	WT	98.81%
GCCTTGATTACTCAAACcAACAGGTTAC	136	+1 c	0.36%
GCCTTGATTACTCA---AACAGGTTAC	129	-3	0.34%
GCCTTGATTACTCAA--AACAGGTTAC	20	-2	0.05%
GCCTTGATTACTCAA----------AC	17	-10	0.04%
GCCTTGATTACT----CAACAGGTTAC	15	-4	0.04%
GCCTTGA----------------TTAC	12	-16	0.03%
GCCTTGATTACTCAAA-AACAGGTTAC	12	-1	0.03%
GCCTTGATTACTCA-------------	9	-14	0.02%
GCCTTGATTACTC---CAACAGGTTAC	9	-3	0.02%

Total | INDEL	24h	Ins	Del	HDR
91871 | 4.79%		0.90%	3.89%	0.00%

Typical seqs	Reads	Type	pct.
GCCTTGATTACTCAAAC|AAC**AGG**TTAC	87468	WT	95.21%
GCCTTGATTACTCA---AACAGGTTAC	1574	-3	1.71%
GCCTTGATTACTCAAACcAACAGGTTAC	668	+1 c	0.73%
GCCTTGATTACT----CAACAGGTTAC	172	-4	0.19%
GCCTTGA----------------TTAC	122	-16	0.13%
GCCTTGATTACTCAA----------AC	124	-10	0.13%
GCCTTGATTACTCAA--AACAGGTTAC	94	-2	0.10%
GCCTTGATTACT-------CAGGTTAC	88	-7	0.10%
GCCTTGATTACTCA-------------	82	-14	0.09%
GCCTTGATTACTCA------AGGTTAC	77	-6	0.08%

Total | INDEL	48h	Ins	Del	HDR
141734 | 6.88%		1.15%	5.73%	0.00%

Typical seqs	Reads	Type	pct.
GCCTTGATTACTCAAAC|AAC**AGG**TTAC	131988	WT	93.12%
GCCTTGATTACTCA---AACAGGTTAC	3858	-3	2.72%
GCCTTGATTACTCAAACcAACAGGTTAC	1148	+1 c	0.81%
GCCTTGATTACT----CAACAGGTTAC	376	-4	0.27%
GCCTTGA----------------TTAC	360	-16	0.25%
GCCTTGATTACTCAA--AACAGGTTAC	238	-2	0.17%
GCCTTGATTACTCA-------------	187	-14	0.13%
GCCTTGATTACTCA------AGGTTAC	172	-6	0.12%
GCCTTGATTACTCAA----------AC	182	-10	0.13%
GCCTTGATT---------ACAGGTTAC	126	-9	0.09%

P143-P147-YW(WT)-K562-RNP-KO-Syn99crBCL11A9a

4h — Total 43785 | INDEL 6.61% | Ins 5.76% | Del 0.85% | HDR 0.00%

Typical seqs	Reads	Type	pct.
GCGCAAACTCCCGTTCT|CCG**AGG**AGTG	40889	WT	93.39%
GCGCAAACTCCCGTTCTtCCGAGGAGTG	2364	+1 t	5.40%
GCGCAAACTC-------CCGAGGAGTG	58	-7	0.13%
GCGCAAACTCCCGTTCT--GAGGAGTG	52	-2	0.12%
GCGCAAACTCCCGT--TCCGAGGAGTG	48	-2	0.11%
GCGCAAACTCCCGTTCT-CGAGGAGTG	34	-1	0.08%
GCGCAAACTCCCGTT-TCCGAGGAGTG	26	-1	0.06%
GCGCAAACTCCCG---TCCGAGGAGTG	22	-3	0.05%
GCGCAAACTCCCGTTCTTTCCGAGGAGTG	21	+2	0.05%
GCGCAAGCTCCCGTTCTTCCGAGGAGTG	21	+1 Ins	0.05%

8h — Total 53383 | INDEL 16.21% | Ins 12.96% | Del 3.25% | HDR 0.00%

Typical seqs	Reads	Type	pct.
GCGCAAACTCCCGTTCT|CCG**AGG**AGTG	44731	WT	83.79%
GCGCAAACTCCCGTTCTtCCGAGGAGTG	6107	+1 t	11.44%
GCGCAAACTC-------CCGAGGAGTG	361	-7	0.68%
GCGCAAACTCCCGT--TCCGAGGAGTG	155	-2	0.29%
GCGCAAGCTCCCGTTCTTCCGAGGAGTG	115	+1 Ins	0.22%
GCGCAAACTCCCGTTCT-CGAGGAGTG	97	-1	0.18%
GCGCAAA---------------------	87	-20	0.16%
GCGCAAACTCCCGTT------------	76	-12	0.14%
GCGCAAA--------CTCCGAGGAGTG	55	-8	0.10%
GCGCAAACTCCCGTTCT--GAGGAGTG	50	-2	0.09%

12h — Total 20180 | INDEL 19.97% | Ins 15.40% | Del 4.56% | HDR 0.00%

Typical seqs	Reads	Type	pct.
GCGCAAACTCCCGTTCT|CCG**AGG**AGTG	16151	WT	80.03%
GCGCAAACTCCCGTTCTtCCGAGGAGTG	2807	+1 t	13.91%
GCGCAAACTC-------CCGAGGAGTG	240	-7	1.19%
GCGCAAACTCCCGT--TCCGAGGAGTG	97	-2	0.48%
GCGCAAGCTCCCGTTCTTCCGAGGAGTG	68	+1 Ins	0.34%
GCGCAAA--------CTCCGAGGAGTG	49	-8	0.24%
GCGCAAA---------------------	47	-20	0.23%
GCGCAAACTCCCGT-------------	44	-14	0.22%
GCGCAAACTCCCGTTCT--GAGGAGTG	42	-2	0.21%
GCGCAAACTCCCGTTCT-CGAGGAGTG	38	-1	0.19%

24h — Total 36451 | INDEL 43.42% | Ins 21.81% | Del 21.60% | HDR 0.00%

Typical seqs	Reads	Type	pct.
GCGCAAACTCCCGTTCT|CCG**AGG**AGTG	20625	WT	56.58%
GCGCAAACTCCCGTTCTtCCGAGGAGTG	7326	+1 t	20.10%
GCGCAAACTC-------CCGAGGAGTG	3827	-7	10.50%
GCGCAAACTCCCGT--TCCGAGGAGTG	434	-2	1.19%
GCGCAAA---------------------	334	-20	0.92%
GCGCAAA--------CTCCGAGGAGTG	260	-8	0.71%
GCGCAAACTCCCGT-------------	162	-14	0.44%
GCGCAAACTCCCGTTCT--GAGGAGTG	137	-2	0.38%
GCGCAAACTCCCG---TCCGAGGAGTG	135	-3	0.37%
GCGCAAACTCCCGTT------------	149	-12	0.41%

48h — Total 70691 | INDEL 57.90% | Ins 25.19% | Del 32.72% | HDR 0.00%

Typical seqs	Reads	Type	pct.
GCGCAAACTCCCGTTCT|CCG**AGG**AGTG	29760	WT	42.10%
GCGCAAACTCCCGTTCTtCCGAGGAGTG	15647	+1 t	22.13%
GCGCAAACTC-------CCGAGGAGTG	11084	-7	15.68%
GCGCAAACTCCCGT--TCCGAGGAGTG	1089	-2	1.54%
GCGCAAA--------CTCCGAGGAGTG	960	-8	1.36%
GCGCAAA---------------------	1136	-20	1.61%
GCGCAAACTCCCGT-------------	527	-14	0.75%
GCGCAAACTCCCGTTC-CCGAGGAGTG	363	-1	0.51%
GCGCAAACTCCCGTT------------	400	-12	0.57%
GCGCAAACTC-----------------	401	-19	0.57%

P143-P147-YW(WT)-K562-RNP-KO-Syn100crBCL11A9b

4h

Total | INDEL	Ins	Del	HDR
1606 | 1.81%	1.62%	0.19%	0.00%

Typical seqs	Reads	Type	pct.
GTCGGACTTGACCGTCA|TGG**GGG**ACGA	1577	WT	98.19%
GTCGGACTTGACCGTCAaTGGGGGACGA	21	+1 a	1.31%
GTCGGACTTGACCGTCAtTGGGGGACGA	5	+1 t	0.31%
GTCGGACTTGACCGTCAT-GGGGACGA	3	-1	0.19%

8h

Total | INDEL	Ins	Del	HDR
4857 | 5.85%	3.62%	2.22%	0.00%

Typical seqs	Reads	Type	pct.
GTCGGACTTGACCGTCA|TGG**GGG**ACGA	4573	WT	94.15%
GTCGGACTTGACCGTCAaTGGGGGACGA	135	+1 a	2.78%
GTCGGACTTGACCGTCAtTGGGGGACGA	36	+1 t	0.74%
GTCGGACTTGACCGTCA-GGGGGACGA	30	-1	0.62%
GTCGGACTTGACCGTCAT-GGGGACGA	26	-1	0.54%
GTCGGACTTGACCGTC-TGGGGGACGA	17	-1	0.35%
GTCGGACTT-------------GACGA	6	-13	0.12%
GTCGGACTTGACCGT--TGGGGGACGA	6	-2	0.12%
GTCGGACTTGACCGTC--GGGGGACGA	5	-2	0.10%
GTCGGACTTGACCGTCAgTGGGGGACGA	5	+1 g	0.10%

12h

Total | INDEL	Ins	Del	HDR
163 | 8.59%	5.52%	3.07%	0.00%

Typical seqs	Reads	Type	pct.
GTCGGACTTGACCGTCA|TGG**GGG**ACGA	149	WT	91.41%
GTCGGACTTGACCGTCAaTGGGGGACGA	9	+1 a	5.52%
GTCGGACTTGACCGTCA-GGGGGACGA	5	-1	3.07%

24h

Total | INDEL	Ins	Del	HDR
2799 | 26.44%	10.40%	16.04%	0.00%

Typical seqs	Reads	Type	pct.
GTCGGACTTGACCGTCA|TGG**GGG**ACGA	2059	WT	73.56%
GTCGGACTTGACCGTCAaTGGGGGACGA	216	+1 a	7.72%
GTCGGACTTGACCGTCAtTGGGGGACGA	46	+1 t	1.64%
GTCGGACTTGACCGTCA-GGGGGACGA	43	-1	1.54%
GTCGGACTT-------------GACGA	37	-13	1.32%
GTCGGACTTGACCGTC-TGGGGGACGA	34	-1	1.21%
GTC------------------GGACGA	34	-18	1.21%
GTCGGACT---------TGGGGGACGA	18	-9	0.64%
GTCGGACTTGACCGTCAT-GGGGACGA	17	-1	0.61%
GTCGGACTT----------------GA	16	-16	0.57%

48h

Total | INDEL	Ins	Del	HDR
180 | 36.11%	16.11%	20.00%	0.00%

Typical seqs	Reads	Type	pct.
GTCGGACTTGACCGTCA|TGG**GGG**ACGA	115	WT	63.89%
GTCGGACTTGACCGTCAaTGGGGGACGA	29	+1 a	16.11%
GTCGGACTTGACCGTCA-GGGGGACGA	7	-1	3.89%
GTCGGACTT-------------GACGA	6	-13	3.33%
GTC------------------GGACGA	6	-18	3.33%
GTCGGACTT----------------GA	7	-16	3.89%
GTCGGACTTGAC------------CGA	4	-12	2.22%
GTCGGACTTG------ATGGGGGACGA	3	-6	1.67%
GTCGGACTTGACCGTC-TGGGGGACGA	3	-1	1.67%

P143-P147-YW(WT)-K562-RNP-KO-Syn101crBCL11A9c

Total | INDEL	4h	Ins	Del	HDR
14159 | 2.97%		2.73%	0.24%	0.00%
**Typical seqs**		**Reads**	**Type**	**pct.**
GAGTACACGTTCTCCGT|GTT**GGG**CATC		13738	WT	97.03%
GAGTACACGTTCTCCGTtGTTGGGCATC		367	+1 t	2.59%
GAGTACACGTTCTCC--GTTGGGCATC		13	-2	0.09%
GAGTACACGTTCTCCGTtGCTGGGCATC		6	+1 t	0.04%
GAGTACACGTTCTCCGTGTT-GGCATC		6	-1	0.04%
GAGTACAC---------GTTGGGCATC		5	-9	0.04%
GAGTACACGTTCTCCGTtGTTGGGCGTC		4	+1 t	0.03%
GAGTACGCGTTCTCCGTTGTTGGGCATC		4	+1 Ins	0.03%
GAGTACACGTTCTCC-TGTTGGGCATC		4	-1	0.03%
GAGTACACGTTCTCCGCTGTTGGGCATC		3	+1 Ins	0.02%

Total | INDEL	8h	Ins	Del	HDR
3896 | 4.93%		4.52%	0.41%	0.00%
**Typical seqs**		**Reads**	**Type**	**pct.**
GAGTACACGTTCTCCGT|GTT**GGG**CATC		3704	WT	95.07%
GAGTACACGTTCTCCGTtGTTGGGCATC		176	+1 t	4.52%
GAGTACACGTTCTCC--GTTGGGCATC		5	-2	0.13%
GAGTACACGTTCTCC-TGTTGGGCATC		5	-1	0.13%
GAGTACACGTTCTCCGTGTT-GGCATC		3	-1	0.08%
GAGTACACGTTC-------TGGGCATC		3	-7	0.08%

Total | INDEL	12h	Ins	Del	HDR
6107 | 5.42%		4.42%	1.00%	0.00%
**Typical seqs**		**Reads**	**Type**	**pct.**
GAGTACACGTTCTCCGT|GTT**GGG**CATC		5776	WT	94.58%
GAGTACACGTTCTCCGTtGTTGGGCATC		267	+1 t	4.37%
GAGTACACGTTCTCC--GTTGGGCATC		18	-2	0.29%
GAGTACACGTTCTCC-TGTTGGGCATC		7	-1	0.11%
GAGTACAC---------GTTGGGCATC		6	-9	0.10%
GAGTACACGTTCTCCGTGTT-GGCATC		6	-1	0.10%
GAGTACACGTTCTCCGT-TTGGGCATC		4	-1	0.07%
GAGTACACGTTC----TGTTGGGCATC		4	-4	0.07%
--------------------------C		4	-27	0.07%
GAGTACACGTTCTC--TGTTGGGCATC		3	-2	0.05%

Total | INDEL	24h	Ins	Del	HDR
5932 | 7.50%		5.85%	1.65%	0.00%
**Typical seqs**		**Reads**	**Type**	**pct.**
GAGTACACGTTCTCCGT|GTT**GGG**CATC		5487	WT	92.50%
GAGTACACGTTCTCCGTtGTTGGGCATC		341	+1 t	5.75%
GAGTACACGTTCTCC--GTTGGGCATC		41	-2	0.69%
GAGTACAC---------GTTGGGCATC		15	-9	0.25%
GAGTACACGTTCTCC-TGTTGGGCATC		6	-1	0.10%
GA---------------GTTGGGCATC		5	-15	0.08%
GAGTACACGTTCTCC-------GCATC		5	-7	0.08%
GAGTACACGTTCTCCG---TGGGCATC		5	-3	0.08%
GAGTA---------------------C		5	-21	0.08%
GAGTACACGTTC----TGTTGGGCATC		4	-4	0.07%

Total | INDEL	48h	Ins	Del	HDR
14066 | 8.87%		6.31%	2.55%	0.00%
**Typical seqs**		**Reads**	**Type**	**pct.**
GAGTACACGTTCTCCGT|GTT**GGG**CATC		12819	WT	91.13%
GAGTACACGTTCTCCGTtGTTGGGCATC		842	+1 t	5.99%
GAGTACACGTTCTCC--GTTGGGCATC		88	-2	0.63%
GAGTACAC---------GTTGGGCATC		68	-9	0.48%
GAGTACACGTTCTCC-TGTTGGGCATC		18	-1	0.13%
GA---------------GTTGGGCATC		13	-15	0.09%
GAGTACACGTTCTCC------GGCATC		9	-6	0.06%
GAGTACACGTTC-------------TC		8	-13	0.06%
GAGTACACGTTCT---TGTTGGGCATC		7	-3	0.05%
GAGTACA-------------------C		6	-19	0.04%

P143-P147-YW(WT)-K562-RNP-KO-Syn105crBCL11A9g

Total | INDEL	4h	Ins	Del	HDR
11235 | 6.26%		**5.78%**	**0.48%**	**0.00%**

Typical seqs	Reads	Type	pct.
CAACTTACAAATACCCT|GCG**GGG**CATA	10532	WT	93.74%
CAACTTACAAATACCCTtGCGGGGCATA	608	+1 t	5.41%
CAACTTACAAATA-CCTGCGGGGCATA	20	-1	0.18%
CAACTTACAAATA--CTGCGGGGCATA	15	-2	0.13%
CAACTTACAAATACCCCTGCGGGGCATA	8	+1 Ins	0.07%
CAACTTACAAATACCCTGC-GGGCATA	7	-1	0.06%
CGACTTACAAATACCCTTGCGGGGCATA	5	+1 Ins	0.04%
CAACTTACAAATACCCTCTGCGGGGCATA	5	+2	0.04%
CAACTTACAAATACCCTcGCGGGGCATA	5	+1 c	0.04%
CAACTTACAAATACCCTCCTGCGGGGCATA	4	+3	0.04%

Total | INDEL	8h	Ins	Del	HDR
7118 | 8.92%		**8.04%**	**0.89%**	**0.00%**

Typical seqs	Reads	Type	pct.
CAACTTACAAATACCCT|GCG**GGG**CATA	6483	WT	91.08%
CAACTTACAAATACCCTtGCGGGGCATA	547	+1 t	7.68%
CAACTTACAAATA-CCTGCGGGGCATA	12	-1	0.17%
CAACTTACAAATA--CTGCGGGGCATA	11	-2	0.15%
CAACTTACAAATACCCTCTGCGGGGCATA	8	+2	0.11%
CAACTTACAAATA---TGCGGGGCATA	8	-3	0.11%
CAACTTACAAATACCCTtGCTGGGCATA	5	+1 t	0.07%
CAACTTACAAATACCCTGC-GGGCATA	5	-1	0.07%
CAACTTACAA------TGCGGGGCATA	4	-6	0.06%
CAACTTACAAATACCC-GCGGGGCATA	4	-1	0.06%

Total | INDEL	12h	Ins	Del	HDR
5748 | 18.32%		**14.94%**	**3.38%**	**0.00%**

Typical seqs	Reads	Type	pct.
CAACTTACAAATACCCT|GCG**GGG**CATA	4695	WT	81.68%
CAACTTACAAATACCCTtGCGGGGCATA	820	+1 t	14.27%
CAACTTACAAATA-CCTGCGGGGCATA	27	-1	0.47%
CAACTTACAAATA--CTGCGGGGCATA	18	-2	0.31%
CAACTTACAA--------------ATA	16	-14	0.28%
CAACTTACAAATA---TGCGGGGCATA	12	-3	0.21%
CAACTTACAAATACCCTCTGCGGGGCATA	11	+2	0.19%
CAACTTACAAATACC------------	11	-14	0.19%
CAACTTACAAATACCCTGACTGAGCTGGGC ATA	8	+6	0.14%
CAACTTACAAATACCCT--------TA	8	-8	0.14%

Total | INDEL	24h	Ins	Del	HDR
22770 | 18.79%		**14.08%**	**4.71%**	**0.00%**

Typical seqs	Reads	Type	pct.
CAACTTACAAATACCCT|GCG**GGG**CATA	18491	WT	81.21%
CAACTTACAAATACCCTtGCGGGGCATA	2843	+1 t	12.49%
CAACTTACAA--------------ATA	92	-14	0.40%
CAACTTACAAATA--CTGCGGGGCATA	68	-2	0.30%
CAACTTACAAATA---TGCGGGGCATA	65	-3	0.29%
CAACTTACAAATA-CCTGCGGGGCATA	51	-1	0.22%
CAACTTA----------------CATA	51	-16	0.22%
CAACTTACAAATACCCCTGCGGGGCATA	49	+1 Ins	0.22%
CAACT--------------------TA	46	-20	0.20%
CAACTTACAAATACC------------	45	-14	0.20%

Total | INDEL	48h	Ins	Del	HDR
23359 | 24.46%		**17.68%**	**6.79%**	**0.00%**

Typical seqs	Reads	Type	pct.
CAACTTACAAATACCCT|GCG**GGG**CATA	17645	WT	75.54%
CAACTTACAAATACCCTtGCGGGGCATA	3749	+1 t	16.05%
CAACTTACAAATA---TGCGGGGCATA	120	-3	0.51%
CAACTTACAA--------------ATA	116	-14	0.50%
CAACTTACAAATA--CTGCGGGGCATA	100	-2	0.43%
CAACTTACAAATA-CCTGCGGGGCATA	73	-1	0.31%
CAACT--------------------TA	65	-20	0.28%
CAACTTA----------------CATA	58	-16	0.25%
CAACTTACAAATACC------------	62	-14	0.27%
CAACTTACAA----------------A	39	-16	0.17%

P143-P147-YW(WT)-K562-RNP-KO-Syn106crBCL11A10a

4h

Total | INDEL	Ins	Del	HDR
15991 | 0.35%	0.12%	0.23%	0.00%

Typical seqs	Reads	Type	pct.
GCGCTAAGTCACTGTCG|TCG**AGG**CCCC	15935	WT	99.65%
GCGCTAAGTCACT---GTCGAGGCCCC	21	-3	0.13%
GCGCTAAGTCACTGTCGgTCGAGGCCCC	15	+1 g	0.09%
GCGCTAAGTCACTGTC-TCGAGGCCCC	5	-1	0.03%
G--------------CGTCGAGGCCCC	4	-14	0.03%
GCGCTAAGTCACTGTCGtTCGAGGCCCC	4	+1 t	0.03%
GCGCTAAGTCACTGTCG----------	4	-11	0.03%
GCGCTAAGTCACTGTCG-CGAGGCCCC	3	-1	0.02%

8h

Total | INDEL	Ins	Del	HDR
7577 | 0.70%	0.26%	0.44%	0.00%

Typical seqs	Reads	Type	pct.
GCGCTAAGTCACTGTCG|TCG**AGG**CCCC	7524	WT	99.30%
GCGCTAAGTCACT---GTCGAGGCCCC	26	-3	0.34%
GCGCTAAGTCACTGTCGgTCGAGGCCCC	13	+1 g	0.17%
GCGCTAAGTCACTGTCG-CGAGGCCCC	4	-1	0.05%
GCGCTAAGTCACTGTCGCGTCGAGGCCCC	4	+2	0.05%
GCGCTAAGTCACTGTCGtTCGAGGCCCC	3	+1 t	0.04%
GCGCTAAGTCACTGTC-TCGAGGCCCC	3	-1	0.04%

12h

Total | INDEL	Ins	Del	HDR
8422 | 1.14%	0.31%	0.83%	0.00%

Typical seqs	Reads	Type	pct.
GCGCTAAGTCACTGTCG|TCG**AGG**CCCC	8326	WT	98.86%
GCGCTAAGTCACT---GTCGAGGCCCC	53	-3	0.63%
GCGCTAAGTCACTGTCGgTCGAGGCCCC	11	+1 g	0.13%
GCGCTAAGTCACTGTCGtTCGAGGCCCC	9	+1 t	0.11%
GCGCTAAGTCACTGT--TCGAGGCCCC	5	-2	0.06%
GCGCTAAGTCACTGTC-TCGAGGCCCC	3	-1	0.04%
GCGCTAAGTCA---------------C	3	-15	0.04%
GCGCTAAGTCA-------------CCC	3	-13	0.04%
GCGCTAAGTCACTGTCGaTCGAGGCCCC	3	+1 a	0.04%
GCGCTAAGTCACTGTCG--GAGGCCCC	3	-2	0.04%

24h

Total | INDEL	Ins	Del	HDR
7996 | 4.23%	0.28%	3.95%	0.00%

Typical seqs	Reads	Type	pct.
GCGCTAAGTCACTGTCG|TCG**AGG**CCCC	7658	WT	95.77%
GCGCTAAGTCACT---GTCGAGGCCCC	220	-3	2.75%
GCGCTAAGTCACTGTC-TCGAGGCCCC	21	-1	0.26%
GCGCTAAGTCACTGTCGgTCGAGGCCCC	16	+1 g	0.20%
GCGCTAAGTCACTGT--TCGAGGCCCC	16	-2	0.20%
GCGCTAA---------GTCGAGGCCCC	14	-9	0.18%
GCGCTAAGTCACTGTCG----------	14	-11	0.18%
GCGCTAAGTCACTGTCG----------	14	-10	0.18%
GCGCTAAGTCA---------------C	6	-15	0.08%
GCGCTAAGTCACTGTCG---------C	5	-9	0.06%

48h

Total | INDEL	Ins	Del	HDR
15355 | 7.13%	0.66%	6.47%	0.00%

Typical seqs	Reads	Type	pct.
GCGCTAAGTCACTGTCG|TCG**AGG**CCCC	14260	WT	92.87%
GCGCTAAGTCACT---GTCGAGGCCCC	617	-3	4.02%
GCGCTAAGTCACTGTC-TCGAGGCCCC	62	-1	0.40%
GCGCTAAGTCACTGTCGgTCGAGGCCCC	52	+1 g	0.34%
GCGCTAA---------GTCGAGGCCCC	44	-9	0.29%
GCGCTAAGTCA---------------C	26	-15	0.17%
GCGCTAAGTCACTGTCGtTCGAGGCCCC	26	+1 t	0.17%
GCGCTAAGTCACTGT--TCGAGGCCCC	21	-2	0.14%
GCGCTAAGTCACTGTCG-CGAGGCCCC	21	-1	0.14%
GCGCTAA----------TCGAGGCCCC	11	-10	0.07%

P143-P147-YW(WT)-K562-RNP-KO-Syn108crBCL11A10c

Total | INDEL	4h	Ins	Del	HDR
6764 | 2.87%		2.57%	0.30%	0.00%

Typical seqs	Reads	Type	pct.
GTGGGAACTTTGCCGAT|ATG**AGG**ATGG	6570	WT	97.13%
GTGGGAACTTTGCCGATtATGAGGATGG	170	+1 t	2.51%
GTGGGAACTTTGCCG--ATGAGGATGG	9	-2	0.13%
GTGGGAACTTTGCTGATTATGAGGATGG	4	+1 Ins	0.06%
GTGGGAACTTTGCCG-TATGAGGATGG	4	-1	0.06%
GTGGGAACTTTGCC--TATGAGGATGG	4	-2	0.06%
GTGGGAACTT------TATGAGGATGG	3	-6	0.04%

Total | INDEL	8h	Ins	Del	HDR
4835 | 6.12%		5.05%	1.08%	0.00%

Typical seqs	Reads	Type	pct.
GTGGGAACTTTGCCGAT|ATG**AGG**ATGG	4539	WT	93.88%
GTGGGAACTTTGCCGATtATGAGGATGG	235	+1 t	4.86%
GTGGGAACTTTGCCG--ATGAGGATGG	17	-2	0.35%
GTGGGAACTTTGCCG-TATGAGGATGG	10	-1	0.21%
GTGGGAACTTTG----TATGAGGATGG	5	-4	0.10%
GTGGGAACTTTGCC--------GATGG	4	-8	0.08%
GTGGGA---------ATATGAGGATGG	4	-9	0.08%
GTGGGGACTTTGCCGATTATGAGGATGG	3	+1 Ins	0.06%
GTGGGAACTTTGCC--TATGAGGATGG	3	-2	0.06%
GTGGGAACTTTGCCGATtATGAGGGTGG	3	+1 t	0.06%

Total | INDEL	12h	Ins	Del	HDR
3384 | 9.75%		8.10%	1.65%	0.00%

Typical seqs	Reads	Type	pct.
GTGGGAACTTTGCCGAT|ATG**AGG**ATGG	3054	WT	90.25%
GTGGGAACTTTGCCGATtATGAGGATGG	271	+1 t	8.01%
GTGGGAACTTTGCCG--ATGAGGATGG	13	-2	0.38%
GTGGGAACTTTGCC--TATGAGGATGG	9	-2	0.27%
GTGGGAACTT------TATGAGGATGG	7	-6	0.21%
GTGGGAACTTTGCCGA-ATGAGGATGG	6	-1	0.18%
GTGGGAACTTTGC---TATGAGGATGG	5	-3	0.15%
GTGGGAACTTTGCC-------------	4	-13	0.12%
GTGGGAACTTTGCC-----GAGGATGG	3	-5	0.09%
GTGGGAAC--------TATGAGGATGG	3	-8	0.09%

Total | INDEL	24h	Ins	Del	HDR
4167 | 18.89%		12.07%	6.82%	0.00%

Typical seqs	Reads	Type	pct.
GTGGGAACTTTGCCGAT|ATG**AGG**ATGG	3380	WT	81.11%
GTGGGAACTTTGCCGATtATGAGGATGG	497	+1 t	11.93%
GTGGGAACTTTGCCG--ATGAGGATGG	45	-2	1.08%
GTGGGAACTTTGCC-----GAGGATGG	33	-5	0.79%
GTGGGAACTT--------TGAGGATGG	29	-8	0.70%
GTGGGAACTTTGC---TATGAGGATGG	17	-3	0.41%
GTGGGAACTTTGCC--TATGAGGATGG	14	-2	0.34%
GTGGGAACTTTGCC--------GATGG	12	-8	0.29%
G-----------------TGAGGATGG	12	-17	0.29%
GTGGGAACTTTGCCG-TATGAGGATGG	13	-1	0.31%

Total | INDEL	48h	Ins	Del	HDR
7313 | 28.78%		15.01%	13.77%	0.00%

Typical seqs	Reads	Type	pct.
GTGGGAACTTTGCCGAT|ATG**AGG**ATGG	5208	WT	71.22%
GTGGGAACTTTGCCGATtATGAGGATGG	1057	+1 t	14.45%
GTGGGAACTTTGCCG--ATGAGGATGG	141	-2	1.93%
GTGGGAACTTTGCC-----GAGGATGG	119	-5	1.63%
GTGGGAACTT--------TGAGGATGG	111	-8	1.52%
GTGGGAACTTTGCC--------GATGG	59	-8	0.81%
GTGGGAACTTTGCC-------------	57	-13	0.78%
G-----------------------TGG	45	-23	0.62%
GTGGGAACTTTGCCG-TATGAGGATGG	31	-1	0.42%
GTG------------------GGATGG	29	-18	0.40%

P151-YW(XY elec)-K562-RNP-Syn87crBCL11A5b-AAV6KI

Total | INDEL	4h	Ins	Del	HDR
9384 | 5.18%		1.02%	3.85%	0.31%

Typical seqs	Reads	Type	pct.
GGGATTACCGAGTCACC|ACC**AGG**CTGC	8898	WT	94.82%
GGGATTACCGAGTCAC-ACCAGGCTGC	163	-1	1.74%
GGGATTACCGAGTC---ACCAGGCTGC	161	-3	1.72%
GGGATTACCGAGTCACCcACCAGGCTGC	88	+1 c	0.94%
GGGATTACCGAGTCACCGTTTAAACACCAG GCTGC	29	+8 HDR	0.31%
GGGATTACCGAGTCAC--CCAGGCTGC	9	-2	0.10%
GGGATTACCGAGTCACCtACCAGGCTGC	5	+1 t	0.05%
GGGATTACCGAGTCACCA-CAGGCTGC	4	-1	0.04%
GGGATTACCGA---AGCACCAGGCTGC	3	-3	0.03%
GGGATTACCGAGTCACC-CCAGGCTGC	3	-1	0.03%

Total | INDEL	8h	Ins	Del	HDR
15372 | 11.79%		2.17%	8.52%	1.11%

Typical seqs	Reads	Type	pct.
GGGATTACCGAGTCACC|ACC**AGG**CTGC	13559	WT	88.21%
GGGATTACCGAGTC---ACCAGGCTGC	612	-3	3.98%
GGGATTACCGAGTCAC-ACCAGGCTGC	509	-1	3.31%
GGGATTACCGAGTCACCcACCAGGCTGC	271	+1 c	1.76%
GGGATTACCGAGTCACCGTTTAAACACCAG GCTGC	164	+8 HDR	1.07%
GGGATTACCGAGTCAC--CCAGGCTGC	29	-2	0.19%
GGGATTACCGAGT--CCACCAGGCTGC	15	-2	0.10%
GGGATTACCGAGTCACCA-CAGGCTGC	12	-1	0.08%
GGGATTACCGAGTCACCCCACCAGGCTGC	12	+2	0.08%
GGGATTACCGAGTCACCtACCAGGCTGC	12	+1 t	0.08%

Total | INDEL	12h	Ins	Del	HDR
19397 | 17.05%		2.42%	11.82%	2.82%

Typical seqs	Reads	Type	pct.
GGGATTACCGAGTCACC|ACC**AGG**CTGC	16089	WT	82.95%
GGGATTACCGAGTCAC-ACCAGGCTGC	1065	-1	5.49%
GGGATTACCGAGTC---ACCAGGCTGC	833	-3	4.29%
GGGATTACCGAGTCACCGTTTAAACACCAG GCTGC	515	+8 HDR	2.66%
GGGATTACCGAGTCACCcACCAGGCTGC	376	+1 c	1.94%
GGGATTACCGAGTCAC--CCAGGCTGC	51	-2	0.26%
GGGATTACCGAGT--CCACCAGGCTGC	38	-2	0.20%
GGGATTACCGAGTCACC-CCAGGCTGC	24	-1	0.12%
GGGATT-----------ACCAGGCTGC	25	-11	0.13%
GGGATTACCGAGTCACCtACCAGGCTGC	21	+1 t	0.11%

Total | INDEL	24h	Ins	Del	HDR
18486 | 33.58%		4.69%	20.09%	8.80%

Typical seqs	Reads	Type	pct.
GGGATTACCGAGTCACC|ACC**AGG**CTGC	12279	WT	66.42%
GGGATTACCGAGTCAC-ACCAGGCTGC	1621	-1	8.77%
GGGATTACCGAGTCACCGTTTAAACACCAG GCTGC	1530	+8 HDR	8.28%
GGGATTACCGAGTC---ACCAGGCTGC	1419	-3	7.68%
GGGATTACCGAGTCACCcACCAGGCTGC	641	+1 c	3.47%
GGGATT-----------ACCAGGCTGC	86	-11	0.47%
GGGATTACCGAGTCAC--CCAGGCTGC	69	-2	0.37%
GGGATTACCGAGTCACCaACCAGGCTGC	40	+1 a	0.22%
GGGATTACCGAGTCACCtACCAGGCTGC	35	+1 t	0.19%
GGGATTACCGAGT--CCACCAGGCTGC	29	-2	0.16%

Total | INDEL	48h	Ins	Del	HDR
16156 | 52.01%		5.86%	28.20%	17.96%

Typical seqs	Reads	Type	pct.
GGGATTACCGAGTCACC|ACC**AGG**CTGC	7753	WT	47.99%
GGGATTACCGAGTCACCGTTTAAACACCAG GCTGC	2683	+8 HDR	16.61%
GGGATTACCGAGTCAC-ACCAGGCTGC	1707	-1	10.57%
GGGATTACCGAGTC---ACCAGGCTGC	1670	-3	10.34%
GGGATTACCGAGTCACCcACCAGGCTGC	652	+1 c	4.04%
GGGATT-----------ACCAGGCTGC	272	-11	1.68%
GGGATTACCGAGTCAC--CCAGGCTGC	105	-2	0.65%
GGGATTACCGAGT--CCACCAGGCTGC	63	-2	0.39%
GGGATTACCGAGT------CAGGCTGC	59	-6	0.37%
GGGATTACCGAGTCACC-CCAGGCTGC	47	-1	0.29%

P150-YW(XY elec)-K562-RNP-Syn92crBCL11A7b-KO

Total | INDEL	4h	Ins	Del	HDR
3378 | 1.84%		1.75%	0.09%	0.00%

Typical seqs	Reads	Type	pct.
GCTTCGTGACGTTGGAT|GGA**GGG**TTTT	3316	WT	98.16%
GCTTCGTGACGTTGGATtGGAGGGTTTT	59	+1 t	1.75%
GCTTCGTGACGTT----GGAGGGTTTT	3	-4	0.09%

Total | INDEL	8h	Ins	Del	HDR
6202 | 4.35%		3.85%	0.50%	0.00%

Typical seqs	Reads	Type	pct.
GCTTCGTGACGTTGGAT|GGA**GGG**TTTT	5932	WT	95.65%
GCTTCGTGACGTTGGATtGGAGGGTTTT	233	+1 t	3.76%
GCTTCGTGACGTT----GGAGGGTTTT	11	-4	0.18%
GCTTCGTGACGTTGGAT-GAGGGTTTT	5	-1	0.08%
GCTTCGT-------GATGGAGGGTTTT	3	-7	0.05%
GCTTCGTGACGTTGGATCTGGAGGGTTTT	3	+2	0.05%
GCCTCGTGACGTTGGATTGGAGGGTTTT	3	+1 Ins	0.05%
GCTTCG----------TGGAGGGTTTT	3	-10	0.05%
GCTTCGTGACG-----TGGAGGGTTTT	3	-5	0.05%
GCTTCGTGACGTTGGA-GGAGGGTTTT	3	-1	0.05%

Total | INDEL	12h	Ins	Del	HDR
32578 | 6.93%		5.79%	1.13%	0.02%

Typical seqs	Reads	Type	pct.
GCTTCGTGACGTTGGAT|GGA**GGG**TTTT	30319	WT	93.07%
GCTTCGTGACGTTGGATtGGAGGGTTTT	1715	+1 t	5.26%
GCTTCGTGACGTT----GGAGGGTTTT	137	-4	0.42%
GCTTCGTGACGTTGGAT-GAGGGTTTT	25	-1	0.08%
GCTTCGTGACGTTG--TGGAGGGTTTT	20	-2	0.06%
GCTTCG----------TGGAGGGTTTT	18	-10	0.06%
GCTTCGTGACGTTGGA-GGAGGGTTTT	17	-1	0.05%
GCTTCGTGACGTTGGATATGGAGGGTTTT	15	+2	0.05%
GCTTCGTGAC------------GTTTT	15	-12	0.05%
GCTTCGTGACGTTGGATtGGGGGGTTTT	13	+1 t	0.04%

Total | INDEL	24h	Ins	Del	HDR
29775 | 13.73%		9.38%	4.31%	0.05%

Typical seqs	Reads	Type	pct.
GCTTCGTGACGTTGGAT|GGA**GGG**TTTT	25686	WT	86.27%
GCTTCGTGACGTTGGATtGGAGGGTTTT	2505	+1 t	8.41%
GCTTCGTGACGTT----GGAGGGTTTT	630	-4	2.12%
GCTTCGTGACGTT-------GGGTTTT	62	-7	0.21%
GCTTCGTGACGTTG--TGGAGGGTTTT	53	-2	0.18%
GCTTCGTGACGTTGGA-GGAGGGTTTT	46	-1	0.15%
GCTTCGTGAC------------GTTTT	28	-12	0.09%
GCTTCGTGACGTTGG-TGGAGGGTTTT	28	-1	0.09%
GCTTCGT-------GATGGAGGGTTTT	26	-7	0.09%
GCTTCGTGACGTT--------GGTTTT	26	-8	0.09%

Total | INDEL	48h	Ins	Del	HDR
18245 | 22.70%		12.32%	10.34%	0.04%

Typical seqs	Reads	Type	pct.
GCTTCGTGACGTTGGAT|GGA**GGG**TTTT	14104	WT	77.30%
GCTTCGTGACGTTGGATtGGAGGGTTTT	2039	+1 t	11.18%
GCTTCGTGACGTT----GGAGGGTTTT	1163	-4	6.37%
GCTTCGTGACGTT-------GGGTTTT	110	-7	0.60%
GCTTCGTGACGTT--------GGTTTT	61	-8	0.33%
GCTTCGTGAC------------GTTTT	56	-12	0.31%
GCTTCGTGACGTTGGA-GGAGGGTTTT	50	-1	0.27%
GCTTCGTGACGTTG--TGGAGGGTTTT	29	-2	0.16%
GCTTCGTGACGTTGGA-------TTTT	23	-7	0.13%
GCTTCGTGACGTTGGAATGGAGGGTTTT	23	+1 Ins	0.13%

P151-YW(XY elec)-K562-RNP-Syn92crBCL11A7b-AAV6KI

Total | INDEL	4h	Ins	Del	HDR
20911 | 1.98%		1.53%	0.24%	0.22%

Typical seqs	Reads	Type	pct.
GCTTCGTGACGTTGGAT|GGA**GGG**TTTT	20496	WT	98.02%
GCTTCGTGACGTTGGATtGGAGGGTTTT	302	+1 t	1.44%
GCTTCGTGACGTTGGATGTTTAAACGGAGG GTTTT	46	+8 HDR	0.22%
GCTTCGTGACGTT----GGAGGGTTTT	14	-4	0.07%
GCTTCGTGACGTTGG-TGGAGGGTTTT	8	-1	0.04%
GCTTCGTGACGTTG--TGGAGGGTTTT	6	-2	0.03%
GCTTCGTGACGTTGGAT-GAGGGTTTT	5	-1	0.02%
GCTTC-----------TGGAGGGTTTT	5	-11	0.02%
GCTTCGTGACGTTGGATgGGAGGGTTTT	5	+1 g	0.02%
GCTTCGTGACGTTGG--GGAGGGTTTT	5	-2	0.02%

Total | INDEL	8h	Ins	Del	HDR
18369 | 5.26%		3.91%	0.58%	0.77%

Typical seqs	Reads	Type	pct.
GCTTCGTGACGTTGGAT|GGA**GGG**TTTT	17402	WT	94.74%
GCTTCGTGACGTTGGATtGGAGGGTTTT	681	+1 t	3.71%
GCTTCGTGACGTTGGATGTTTAAACGGAGG GTTTT	141	+8 HDR	0.77%
GCTTCGTGACGTT----GGAGGGTTTT	56	-4	0.30%
GCTTCGTGACGTTG--TGGAGGGTTTT	9	-2	0.05%
GCTTCGTGACGTTGGAT-GAGGGTTTT	8	-1	0.04%
GCTTCGTGACGTTGGATtGGGGGGTTTT	6	+1 t	0.03%
GCTCCGTGACGTTGGATTGGAGGGTTTT	5	+1 Ins	0.03%
GCTTCGTG--------TGGAGGGTTTT	4	-8	0.02%
GCTTCGTGACGTTGGATATGGAGGGTTTT	4	+2	0.02%

Total | INDEL	12h	Ins	Del	HDR
32883 | 7.52%		5.36%	0.83%	1.33%

Typical seqs	Reads	Type	pct.
GCTTCGTGACGTTGGAT|GGA**GGG**TTTT	30410	WT	92.48%
GCTTCGTGACGTTGGATtGGAGGGTTTT	1584	+1 t	4.82%
GCTTCGTGACGTTGGATGTTTAAACGGAGG GTTTT	421	+8 HDR	1.28%
GCTTCGTGACGTT----GGAGGGTTTT	100	-4	0.30%
GCTTCGTGACGTTGGA-GGAGGGTTTT	29	-1	0.09%
GCTTCGTGACGTTG--TGGAGGGTTTT	19	-2	0.06%
GCTTCGTGACGTTGGAT-GAGGGTTTT	17	-1	0.05%
GCTTCGTGACGTTGG-TGGAGGGTTTT	15	-1	0.05%
GCTTCGTGACGTTGGATATGGAGGGTTTT	15	+2	0.05%
GCTTCGTGACGTTGGATtGGAGGGTCTT	13	+1 t	0.04%

Total | INDEL	24h	Ins	Del	HDR
19910 | 15.11%		8.87%	1.81%	4.42%

Typical seqs	Reads	Type	pct.
GCTTCGTGACGTTGGAT|GGA**GGG**TTTT	16902	WT	84.89%
GCTTCGTGACGTTGGATtGGAGGGTTTT	1549	+1 t	7.78%
GCTTCGTGACGTTGGATGTTTAAACGGAGG GTTTT	847	+8 HDR	4.25%
GCTTCGTGACGTT----GGAGGGTTTT	166	-4	0.83%
GCTTCGTGACGTTG--TGGAGGGTTTT	28	-2	0.14%
GCTTCGTGACGTTGGATATGGAGGGTTTT	28	+2	0.14%
GCTTCGTGACGTTGGA-GGAGGGTTTT	22	-1	0.11%
GCTTCGTGACGTTGGAT-GAGGGTTTT	21	-1	0.11%
GCTTCGTGACGTTGGATTTGGAGGGTTTT	16	+2	0.08%
GCTTCGTGACGTTGGATaGGAGGGTTTT	14	+1 a	0.07%

Total | INDEL	48h	Ins	Del	HDR
8111 | 21.42%		9.99%	4.11%	7.32%

Typical seqs	Reads	Type	pct.
GCTTCGTGACGTTGGAT|GGA**GGG**TTTT	6374	WT	78.58%
GCTTCGTGACGTTGGATtGGAGGGTTTT	725	+1 t	8.94%
GCTTCGTGACGTTGGATGTTTAAACGGAGG GTTTT	582	+8 HDR	7.18%
GCTTCGTGACGTT----GGAGGGTTTT	179	-4	2.21%
GCTTCGTGACGTTGGA-GGAGGGTTTT	19	-1	0.23%
GCTTCGTGACGTTG--TGGAGGGTTTT	19	-2	0.23%
GCTTCG----------TGGAGGGTTTT	11	-10	0.14%
GCTTCGTGACGT----TGGAGGTTTTT	9	-4	0.11%
GCTTCGTGACGTTGGAATGGAGGGTTTT	9	+1 Ins	0.11%
GCTTCGTGACGTTGGATTTTTAAACGGAGG GTTTT	8	+8	0.10%

P153-P157-YW-K562-RNP-Syn85BCL11A-KO

Total | INDEL	4h	Ins	Del	HDR
176616 | 0.54%		0.02%	0.52%	0.00%

Typical seqs	Reads	Type	pct.
TGGTCGCCTACGGAATC|TAC**AGG**AATC	175668	WT	99.46%
TGGTCGCCTAC----------GGAATC	265	-10	0.15%
TGGTCGC---------CTACAGGAATC	142	-9	0.08%
TGGTCGCCTACGGAA--TACAGGAATC	103	-2	0.06%
TGGTCGCCTACGGAATC-ACAGGAATC	101	-1	0.06%
TGGTCGCCTACGGAATC----------	53	-11	0.03%
TGGTCGCCTACGGAATC--CAGGAATC	35	-2	0.02%
TGGTCGCCTACGGAAT-TACAGGAATC	29	-1	0.02%
TGGTCGCCTACGGAATC-----GAATC	29	-5	0.02%
TGGTCGCCTACGGA---TACAGGAATC	27	-3	0.02%

Total | INDEL	8h	Ins	Del	HDR
86238 | 2.04%		0.05%	2.00%	0.00%

Typical seqs	Reads	Type	pct.
TGGTCGCCTACGGAATC|TAC**AGG**AATC	84477	WT	97.96%
TGGTCGCCTAC----------GGAATC	567	-10	0.66%
TGGTCGC---------CTACAGGAATC	357	-9	0.41%
TGGTCGCCTACGGAATC-ACAGGAATC	129	-1	0.15%
TGGTCGCCTACGGAA--TACAGGAATC	99	-2	0.11%
TGGTCGCCTACGGAATC----------	92	-11	0.11%
TGGTCGCCTACGGA---TACAGGAATC	51	-3	0.06%
TGGTCGCCTACGGAATC-----GAATC	47	-5	0.05%
TGGTCGCCTACGGAAT-TACAGGAATC	36	-1	0.04%
TGGTCG-----------TACAGGAATC	27	-11	0.03%

Total | INDEL	12h	Ins	Del	HDR
117202 | 5.02%		0.08%	4.93%	0.00%

Typical seqs	Reads	Type	pct.
TGGTCGCCTACGGAATC|TAC**AGG**AATC	111324	WT	94.98%
TGGTCGCCTAC----------GGAATC	2026	-10	1.73%
TGGTCGC---------CTACAGGAATC	1464	-9	1.25%
TGGTCGCCTACGGAATC-ACAGGAATC	396	-1	0.34%
TGGTCGCCTACGGAA--TACAGGAATC	215	-2	0.18%
TGGTCGCCTACGGAATC----------	176	-11	0.15%
TGGTCGCCTACGGAAT-TACAGGAATC	123	-1	0.10%
TGGTCGCCTACGGAATC-----GAATC	107	-5	0.09%
TGGTCGCCTACGGAAT---CAGGAATC	111	-3	0.09%
TGGTCGCCTACGGAATC--CAGGAATC	94	-2	0.08%

Total | INDEL	24h	Ins	Del	HDR
115549 | 24.77%		0.22%	24.55%	0.00%

Typical seqs	Reads	Type	pct.
TGGTCGCCTACGGAATC|TAC**AGG**AATC	86924	WT	75.23%
TGGTCGCCTAC----------GGAATC	12184	-10	10.54%
TGGTCGC---------CTACAGGAATC	9939	-9	8.60%
TGGTCGCCTACGGAATC-ACAGGAATC	631	-1	0.55%
TGGTCGCCTACGGAA--TACAGGAATC	506	-2	0.44%
TGGTCGCCTACGGAATC----------	469	-11	0.41%
TGGTCGCCTACGGAATC--CAGGAATC	282	-2	0.24%
TGGTCGCCTACGGAAT---CAGGAATC	241	-3	0.21%
TGGTCGCCTACGGA---TACAGGAATC	225	-3	0.19%
TGGTCGCCTACGGAATC-----GAATC	222	-5	0.19%

Total | INDEL	48h	Ins	Del	HDR
96083 | 32.19%		0.25%	31.94%	0.00%

Typical seqs	Reads	Type	pct.
TGGTCGCCTACGGAATC|TAC**AGG**AATC	65153	WT	67.81%
TGGTCGCCTAC----------GGAATC	14088	-10	14.66%
TGGTCGC---------CTACAGGAATC	9318	-9	9.70%
TGGTCGCCTACGGAATC-ACAGGAATC	910	-1	0.95%
TGGTCGCCTACGGAA--TACAGGAATC	484	-2	0.50%
TGGTCGCCTACGGAAT-TACAGGAATC	272	-1	0.28%
TGGTCGCCTACGGAATC-----GAATC	246	-5	0.26%
TGGTCGCCTACGGAAT---CAGGAATC	233	-3	0.24%
TGGTCGCCTACGGAATC----------	386	-11	0.40%
TGGTCG----------CTACAGGAATC	191	-10	0.20%

P153-P157-YW-K562-RNP-Syn102BCL11A-KO

4h

Total	INDEL	Ins	Del	HDR
59979	0.27%	0.21%	0.06%	0.00%

Typical seqs	Reads	Type	pct.
TCCGTGTTGGGCATCGC|GGC**CGG**GGGC	59820	WT	99.73%
TCCGTGTTGGGCATCGCcGGCCGGGGGC	69	+1 c	0.12%
TCCGTGTTGGGCATCGCaGGCCGGGGGC	22	+1 a	0.04%
TCCGTGTTGGGCATCGCgGGCCGGGGGC	13	+1 g	0.02%
TCCGTGTTG--------GGCCGGGGGC	11	-8	0.02%
TCCGTGTTGGGCATCGCtGGCCGGGGGC	10	+1 t	0.02%
TCCGTGTTGGGCAT--CGGCCGGGGGC	9	-2	0.02%
TCCGTGTTGGGCATC---GCCGGGGGC	8	-3	0.01%
TCCGTGTTGGGCATCGTGTTGGGTTCTCGT GTTGGGCCGGGGGC	5	+17	0.01%
TCCGTGTTGGGCATCGCATAGGCCGGGGGC	5	+3	0.01%

8h

Total	INDEL	Ins	Del	HDR
13555	0.94%	0.46%	0.48%	0.00%

Typical seqs	Reads	Type	pct.
TCCGTGTTGGGCATCGC|GGC**CGG**GGGC	13428	WT	99.06%
TCCGTGTTGGGCATCGCcGGCCGGGGGC	40	+1 c	0.30%
TCCGTGTTG--------GGCCGGGGGC	18	-8	0.13%
TCCGTGTTGGGCATCGCgGGCCGGGGGC	12	+1 g	0.09%
TCCGTGTTGGGCAT--CGGCCGGGGGC	9	-2	0.07%
TCCGTGTT---------GGCCGGGGGC	9	-9	0.07%
TCCGTGTTGGGCATC---GCCGGGGGC	8	-3	0.06%
TCCGTGTTGGGCATCGCaGGCCGGGGGC	7	+1 a	0.05%
TCCGTGTTGGGCATCG-GGCCGGGGGC	6	-1	0.04%
TCCGTGTTGGGCA--------------	5	-14	0.04%

12h

Total	INDEL	Ins	Del	HDR
16629	1.56%	0.62%	0.94%	0.00%

Typical seqs	Reads	Type	pct.
TCCGTGTTGGGCATCGC|GGC**CGG**GGGC	16370	WT	98.44%
TCCGTGTTGGGCATCGCcGGCCGGGGGC	66	+1 c	0.40%
TCCGTGTTG--------GGCCGGGGGC	33	-8	0.20%
TCCGTGTTGGGCATC---GCCGGGGGC	21	-3	0.13%
TCCGTGTT---------------GGGC	14	-15	0.08%
TCCGTGTTGGGCATCGCgGGCCGGGGGC	12	+1 g	0.07%
TCCGTGTTGGGCATCGCtGGCCGGGGGC	11	+1 t	0.07%
TCCGTGTTGGGCATCGCaGGCCGGGGGC	11	+1 a	0.07%
TCCGTGTTGGGCATCGC-GCCGGGGGC	10	-1	0.06%
TCCGTGTTGGGCAT--CGGCCGGGGGC	8	-2	0.05%

24h

Total	INDEL	Ins	Del	HDR
23703	8.16%	1.60%	6.56%	0.00%

Typical seqs	Reads	Type	pct.
TCCGTGTTGGGCATCGC|GGC**CGG**GGGC	21769	WT	91.84%
TCCGTGTTG--------GGCCGGGGGC	311	-8	1.31%
TCCGTGTTGGGCATC---GCCGGGGGC	281	-3	1.19%
TCCGTGT----------GGCCGGGGGC	207	-10	0.87%
TCCGTGTTGGGCATCGCcGGCCGGGGGC	162	+1 c	0.68%
TCCGTGTT---------------GGGC	85	-15	0.36%
TCCGTGTT---------GGCCGGGGGC	66	-9	0.28%
TCCGTGTTGGGCATCGCaGGCCGGGGGC	46	+1 a	0.19%
TCCGTGTTGGGCAT--CGGCCGGGGGC	46	-2	0.19%
TCCGTGTTGGGCATCGCtGGCCGGGGGC	42	+1 t	0.18%

48h

Total	INDEL	Ins	Del	HDR
15811	12.32%	2.08%	10.24%	0.00%

Typical seqs	Reads	Type	pct.
TCCGTGTTGGGCATCGC|GGC**CGG**GGGC	13863	WT	87.68%
TCCGTGTTG--------GGCCGGGGGC	373	-8	2.36%
TCCGTGTTGGGCATC---GCCGGGGGC	263	-3	1.66%
TCCGTGTT---------------GGGC	164	-15	1.04%
TCCGTGTTGGGCATCGCcGGCCGGGGGC	131	+1 c	0.83%
TCCGTGTT---------GGCCGGGGGC	75	-9	0.47%
TCCGTGTTGGGCAT--CGGCCGGGGGC	61	-2	0.39%
TCCGT-------------GCCGGGGGC	54	-13	0.34%
TCCGTGTTGGGCATCGCtGGCCGGGGGC	49	+1 t	0.31%
TCCGTGTTGGGCATCGCgGGCCGGGGGC	40	+1 g	0.25%

P153-P157-YW-K562-RNP-Syn103BCL11A-KO

Total | INDEL	4h	Ins	Del	HDR
29774 | 1.52%		**0.79%**	**0.73%**	**0.00%**

Typical seqs	Reads	Type	pct.
ATGACGGTCAAGTCCGA|CGA**CGG**TCTC	29321	WT	98.48%
ATGACGGTCAAGTCCGAaCGACGGTCTC	211	+1 a	0.71%
ATGACGGTCAAGTC---CGACGGTCTC	86	-3	0.29%
AT----------------GACGGTCTC	29	-16	0.10%
ATGACGGTCAAGTCCGA-GACGGTCTC	29	-1	0.10%
ATGACGGTCAAGTCCGAgCGACGGTCTC	18	+1 g	0.06%
ATGACGGTC--------CGACGGTCTC	16	-8	0.05%
ATGACGGTCAAGTCCG-CGACGGTCTC	11	-1	0.04%
ATGACGGTCAAGTCCGA----------	12	-11	0.04%
ATGACGGTCAAGTCCGA----------	12	-12	0.04%

Total | INDEL	8h	Ins	Del	HDR
6654 | 3.73%		**1.52%**	**2.21%**	**0.00%**

Typical seqs	Reads	Type	pct.
ATGACGGTCAAGTCCGA|CGA**CGG**TCTC	6406	WT	96.27%
ATGACGGTCAAGTCCGAaCGACGGTCTC	88	+1 a	1.32%
ATGACGGTCAAGTC---CGACGGTCTC	40	-3	0.60%
ATGACGGTCAAGTCCGA-GACGGTCTC	31	-1	0.47%
AT----------------GACGGTCTC	20	-16	0.30%
ATGACGGTCAAGTCCGA----------	5	-12	0.08%
ATGACGGTCAAGTCCG-CGACGGTCTC	5	-1	0.08%
ATGACGGTCAAGTCCGAtCGACGGTCTC	5	+1 t	0.08%
ATGACGGTCAAGTCCGAgCGACGGTCTC	5	+1 g	0.08%
ATGACGGTC-------ACGACGGTCTC	4	-7	0.06%

Total | INDEL	12h	Ins	Del	HDR
6148 | 6.90%		**2.67%**	**4.23%**	**0.00%**

Typical seqs	Reads	Type	pct.
ATGACGGTCAAGTCCGA|CGA**CGG**TCTC	5724	WT	93.10%
ATGACGGTCAAGTCCGAaCGACGGTCTC	148	+1 a	2.41%
ATGACGGTCAAGTC---CGACGGTCTC	81	-3	1.32%
AT----------------GACGGTCTC	65	-16	1.06%
ATGACGGTCAAGTCCGA-GACGGTCTC	29	-1	0.47%
ATGACGGTCAAGTCCG-CGACGGTCTC	18	-1	0.29%
ATGACGGTCAAGTCCGAgCGACGGTCTC	10	+1 g	0.16%
ATGACGGTCAAG---------------	10	-15	0.16%
ATGACGGTCAAGTCCGAtCGACGGTCTC	6	+1 t	0.10%
ATGACGGTCAAGTCCGA--ACGGTCTC	6	-2	0.10%

Total | INDEL	24h	Ins	Del	HDR
11186 | 24.26%		**4.78%**	**19.48%**	**0.00%**

Typical seqs	Reads	Type	pct.
ATGACGGTCAAGTCCGA|CGA**CGG**TCTC	8472	WT	75.74%
AT----------------GACGGTCTC	947	-16	8.47%
ATGACGGTCAAGTC---CGACGGTCTC	488	-3	4.36%
ATGACGGTCAAGTCCGAaCGACGGTCTC	458	+1 a	4.09%
ATGACGGTCAAGTCCGA-GACGGTCTC	105	-1	0.94%
ATGACGGTCAAG---------------	75	-15	0.67%
ATGACGGTCAA-----------GTCTC	63	-11	0.56%
ATGACGGTCAAGTCCG-CGACGGTCTC	54	-1	0.48%
ATGACGGTCAAGTCCG-----------	45	-14	0.40%
ATGACGGTCAAGT--------------	29	-18	0.26%

Total | INDEL	48h	Ins	Del	HDR
7266 | 41.36%		**4.76%**	**36.60%**	**0.00%**

Typical seqs	Reads	Type	pct.
ATGACGGTCAAGTCCGA|CGA**CGG**TCTC	4261	WT	58.64%
AT----------------GACGGTCTC	1317	-16	18.13%
ATGACGGTCAAGTC---CGACGGTCTC	485	-3	6.67%
ATGACGGTCAAGTCCGAaCGACGGTCTC	287	+1 a	3.95%
ATGACGGTCAAG---------------	136	-15	1.87%
ATGACGGTCAA-----------GTCTC	109	-11	1.50%
ATGACGGTCAAGTCCGA-GACGGTCTC	80	-1	1.10%
----------------ACGACGGTCTC	33	-16	0.45%
ATGACGGTCAAGTCCG-CGACGGTCTC	27	-1	0.37%
ATGACGGTCAAGT--------------	29	-18	0.40%

P153-P157-YW-K562-RNP-Syn104BCL11A-KO

Total | INDEL	4h	Ins	Del	HDR
214911 | 1.96%		0.09%	1.87%	0.00%

Typical seqs	Reads	Type	pct.
CATATTAGTGGTCCGGG|CCCGGGCAGG	210706	WT	98.04%
CATATTAGTGGT------CCGGGCAGG	981	-6	0.46%
CATATTAGTGGTCCGGG--CGGGCAGG	516	-2	0.24%
CATATTAGTGGTCC-GGCCCGGGCAGG	499	-1	0.23%
CATATTAGTGGTCCGGG-CCGGGCAGG	453	-1	0.21%
CATATTAGTGGTCC--GCCCGGGCAGG	214	-2	0.10%
CATATTAGTGGTCCGGGgCCCGGGCAGG	117	+1 g	0.05%
CATATTAGTGGTCCGG----GGGCAGG	93	-4	0.04%
CATATTAGTGGTCCGGG---GGGCAGG	82	-3	0.04%
CATATTAGTGGTCC-----------GG	78	-11	0.04%

Total | INDEL	8h	Ins	Del	HDR
47774 | 7.63%		0.18%	7.45%	0.00%

Typical seqs	Reads	Type	pct.
CATATTAGTGGTCCGGG|CCCGGGCAGG	44127	WT	92.37%
CATATTAGTGGT------CCGGGCAGG	1228	-6	2.57%
CATATTAGTGGTCC-GGCCCGGGCAGG	285	-1	0.60%
CATATTAGTGGTCCGGG--CGGGCAGG	288	-2	0.60%
CATATTAGTGGTCCGGG-CCGGGCAGG	252	-1	0.53%
CATATTAGTGGTCCG----------GG	119	-10	0.25%
CATATTAGTGGT-----CCCGGGCAGG	107	-5	0.22%
CATATTAGTGGTCC--GCCCGGGCAGG	100	-2	0.21%
CATATTAGT------GGCCCGGGCAGG	81	-6	0.17%
CATATTAGTGGTCCGGG---GGGCAGG	75	-3	0.16%

Total | INDEL	12h	Ins	Del	HDR
70073 | 12.34%		0.35%	11.99%	0.00%

Typical seqs	Reads	Type	pct.
CATATTAGTGGTCCGGG|CCCGGGCAGG	61427	WT	87.66%
CATATTAGTGGT------CCGGGCAGG	3187	-6	4.55%
CATATTAGTGGTCCGGG--CGGGCAGG	666	-2	0.95%
CATATTAGTGGTCC-GGCCCGGGCAGG	595	-1	0.85%
CATATTAGTGGTCCGGG-CCGGGCAGG	456	-1	0.65%
CATATTAGT------GGCCCGGGCAGG	257	-6	0.37%
CATATTAGTGGTCC--GCCCGGGCAGG	202	-2	0.29%
CATATTAGTGGTCCG----------GG	204	-10	0.29%
CATATTAGTGGTCCGGGgCCCGGGCAGG	194	+1 g	0.28%
CATATTAGTGGTCCG-----GGGCAGG	170	-5	0.24%

Total | INDEL	24h	Ins	Del	HDR
79241 | 45.13%		0.41%	44.72%	0.00%

Typical seqs	Reads	Type	pct.
CATATTAGTGGTCCGGG|CCCGGGCAGG	43480	WT	54.87%
CATATTAGTGGT------CCGGGCAGG	22131	-6	27.93%
CATATTAGTGGTCCGGG--CGGGCAGG	1225	-2	1.55%
CATATTAGT------GGCCCGGGCAGG	1086	-6	1.37%
CATATTAGTGGTCCGGG-CCGGGCAGG	988	-1	1.25%
CATATTAGTGGTCC-GGCCCGGGCAGG	835	-1	1.05%
CATATTAGTGGTCCG----------GG	542	-10	0.68%
CATATTAGTGGT-----CCCGGGCAGG	480	-5	0.61%
CATATTAGTGGTCCG-----GGGCAGG	359	-5	0.45%
CATATTAGTGGTCC--GCCCGGGCAGG	307	-2	0.39%

Total | INDEL	48h	Ins	Del	HDR
53993 | 55.37%		0.51%	54.86%	0.00%

Typical seqs	Reads	Type	pct.
CATATTAGTGGTCCGGG|CCCGGGCAGG	24098	WT	44.63%
CATATTAGTGGT------CCGGGCAGG	18298	-6	33.89%
CATATTAGT------GGCCCGGGCAGG	1074	-6	1.99%
CATATTAGTGGTCCGGG--CGGGCAGG	831	-2	1.54%
CATATTAGTGGTCCGGG-CCGGGCAGG	682	-1	1.26%
CATATTAGTGGTCC-GGCCCGGGCAGG	567	-1	1.05%
CATATTAGTGGT-----CCCGGGCAGG	426	-5	0.79%
CATATTAGTGGTCCG----------GG	378	-10	0.70%
CATATTAGTGGTCCG-----GGGCAGG	277	-5	0.51%
CATATTAGTGGTCC--GCCCGGGCAGG	276	-2	0.51%

P163-P165-YW-K562-Cas9-Syn102BCL11A-KO

Total | INDEL	4h	Ins	Del	HDR
36745 | 2.86%		2.25%	0.60%	0.00%

Typical seqs	Reads	Type	pct.
TCCGTGTTGGGCATCGC|GGC**CGG**GGGC	35695	WT	97.14%
TCCGTGTTGGGCATCGCcGGCCGGGGGC	535	+1 c	1.46%
TCCGTGTTGGGCATCGCtGGCCGGGGGC	93	+1 t	0.25%
TCCGTGTTGGGCATCGCgGGCCGGGGGC	72	+1 g	0.20%
TCCGTGTTGGGCATCG-GGCCGGGGGC	64	-1	0.17%
TCCGTGTTG--------GGCCGGGGGC	54	-8	0.15%
TCCGTGTTGGGCATC---GCCGGGGGC	51	-3	0.14%
TCCGTGTTGGGCATCGCaGGCCGGGGGC	44	+1 a	0.12%
TCCGTGTTGGGCATCGCGGGGCCGGGGGC	30	+2	0.08%
TCCGTGTTGGGCATCGCTAGGCCGGGGGC	21	+2	0.06%

Total | INDEL	8h	Ins	Del	HDR
32975 | 1.91%		1.13%	0.78%	0.00%

Typical seqs	Reads	Type	pct.
TCCGTGTTGGGCATCGC|GGC**CGG**GGGC	32344	WT	98.09%
TCCGTGTTGGGCATCGCcGGCCGGGGGC	154	+1 c	0.47%
TCCGTGTTGGGCATCGCgGGCCGGGGGC	67	+1 g	0.20%
TCCGTGTTGGGCATCGCtGGCCGGGGGC	64	+1 t	0.19%
TCCGTGTTG--------GGCCGGGGGC	33	-8	0.10%
TCCGTGTTGGGCAT--CGGCCGGGGGC	32	-2	0.10%
TCCGTGTTGGGCATCGCaGGCCGGGGGC	26	+1 a	0.08%
TCCGTGTTGGGCATC---GCCGGGGGC	19	-3	0.06%
TCCGTGTT---------GGCCGGGGGC	18	-9	0.05%
TCCGTGTTGGGCATCG-GGCCGGGGGC	17	-1	0.05%

Total | INDEL	12h	Ins	Del	HDR
94686 | 5.01%		2.65%	2.36%	0.00%

Typical seqs	Reads	Type	pct.
TCCGTGTTGGGCATCGC|GGC**CGG**GGGC	89940	WT	94.99%
TCCGTGTTGGGCATCGCcGGCCGGGGGC	1302	+1 c	1.38%
TCCGTGTTGGGCATCGCtGGCCGGGGGC	307	+1 t	0.32%
TCCGTGTTG--------GGCCGGGGGC	302	-8	0.32%
TCCGTGTTGGGCATCGCgGGCCGGGGGC	280	+1 g	0.30%
TCCGTGTTGGGCATCGCaGGCCGGGGGC	223	+1 a	0.24%
TCCGTGTTGGGCATC---GCCGGGGGC	211	-3	0.22%
TCCGTGTTGG-----GCGGCCGGGGGC	201	-5	0.21%
TCCGTGTTGGGCAT--CGGCCGGGGGC	156	-2	0.16%
TCCGTGTT---------------GGGC	148	-15	0.16%

P166-YW-K562-Cas9-puro-24h-Syn102crBCL11A9d	24h	Ins	Del	HDR
226201 | 27.13%		6.84%	20.29%	0.00%

Typical seqs	Reads	Type	pct.
TCCGTGTTGGGCATCGC|GGC**CGG**GGGC	164826	WT	72.87%
TCCGTGTTGGGCATC---GCCGGGGGC	8125	-3	3.59%
TCCGTGTTG--------GGCCGGGGGC	8068	-8	3.57%
TCCGTGTTGGGCATCGCcGGCCGGGGGC	6862	+1 c	3.03%
TCCGTGTT---------------GGGC	2827	-15	1.25%
TCCGTGTT---------GGCCGGGGGC	2422	-9	1.07%
TCCGTGTTGGGCATCGCaGGCCGGGGGC	1712	+1 a	0.76%
TCCGTGTTGGGCATCGCtGGCCGGGGGC	1676	+1 t	0.74%
TCCGTGTTGGGCATCGCgGGCCGGGGGC	1591	+1 g	0.70%
TCCGTGTTGG-----GCGGCCGGGGGC	1431	-5	0.63%

P167-YW-K562-Cas9-puro-48h-Syn102crBCL11A9d	48h	Ins	Del	HDR
12877 | 52.78%		9.89%	42.89%	0.00%

Typical seqs	Reads	Type	pct.
TCCGTGTTGGGCATCGC|GGC**CGG**GGGC	6080	WT	47.22%
TCCGTGTTGGGCATC---GCCGGGGGC	1298	-3	10.08%
TCCGTGTTG--------GGCCGGGGGC	1156	-8	8.98%
TCCGTGTTGGGCATCGCcGGCCGGGGGC	539	+1 c	4.19%
TCCGTGTT---------------GGGC	443	-15	3.44%
TCCGTGTT---------GGCCGGGGGC	207	-9	1.61%
TCCGT-------------GCCGGGGGC	204	-13	1.58%
TCCGTGTTGGGCAT--CGGCCGGGGGC	148	-2	1.15%
TCCGTGTTGG-----GCGGCCGGGGGC	132	-5	1.03%
TCCGTGTTGGGCATCGCgGGCCGGGGGC	132	+1 g	1.03%

P163-P165-YW-K562-Cas9-Syn103BCL11A-KO

Total | INDEL	4h	Ins	Del	HDR
11604 | 1.66%		0.63%	1.03%	0.00%

Typical seqs	Reads	Type	pct.
ATGACGGTCAAGTCCGA|CGA**CGG**TCTC	11411	WT	98.34%
ATGACGGTCAAGTC---CGACGGTCTC	62	-3	0.53%
ATGACGGTCAAGTCCGAaCGACGGTCTC	57	+1 a	0.49%
AT----------------GACGGTCTC	18	-16	0.16%
ATGACGGTCAAGTCCG-CGACGGTCTC	15	-1	0.13%
ATGACGGTCAAGTCCGA-GACGGTCTC	11	-1	0.09%
ATGACGGTCAAGTCCGAgCGACGGTCTC	8	+1 g	0.07%
ATGACGGTCAA-----------GTCTC	5	-11	0.04%
ATGACGGTCAAGTCCGAGACGACGGTCTC	4	+2	0.03%
ATGACGGTCAAGTCCGAcCGACGGTCTC	4	+1 c	0.03%

Total | INDEL	8h	Ins	Del	HDR
5114 | 14.00%		7.33%	6.67%	0.00%

Typical seqs	Reads	Type	pct.
ATGACGGTCAAGTCCGA|CGA**CGG**TCTC	4398	WT	86.00%
ATGACGGTCAAGTCCGAaCGACGGTCTC	301	+1 a	5.89%
ATGACGGTCAAGTC---CGACGGTCTC	110	-3	2.15%
ATGACGGTCAAGTCCGA-GACGGTCTC	47	-1	0.92%
ATGACGGTCAAGTCCG-CGACGGTCTC	31	-1	0.61%
AT----------------GACGGTCTC	24	-16	0.47%
ATGACGGTCAAGTCCGAgCGACGGTCTC	22	+1 g	0.43%
ATGACGGTCAAGTCCGAtCGACGGTCTC	15	+1 t	0.29%
ATGACGGTCAAGTCCGA--ACGGTCTC	13	-2	0.25%
ATGACGGTCAAGTCC--CGACGGTCTC	12	-2	0.23%

Total | INDEL	12h	Ins	Del	HDR
39924 | 29.27%		13.72%	15.55%	0.00%

Typical seqs	Reads	Type	pct.
ATGACGGTCAAGTCCGA|CGA**CGG**TCTC	28238	WT	70.73%
ATGACGGTCAAGTCCGAaCGACGGTCTC	4479	+1 a	11.22%
ATGACGGTCAAGTC---CGACGGTCTC	1703	-3	4.27%
AT----------------GACGGTCTC	774	-16	1.94%
ATGACGGTCAAGTCCGA-GACGGTCTC	579	-1	1.45%
ATGACGGTCAAGTCCG-CGACGGTCTC	313	-1	0.78%
ATGACGGTCAAGTCCGAgCGACGGTCTC	148	+1 g	0.37%
ATGACGGTCAAGT----CGACGGTCTC	139	-4	0.35%
ATGACGGTC-------ACGACGGTCTC	134	-7	0.34%
ATGACGGTC--------CGACGGTCTC	137	-8	0.34%

P166-YW-K562-Cas9-puro-24h-Syn103crBCL11A9e	24h	Ins	Del	HDR
187173 | 64.67%		21.13%	43.54%	0.00%

Typical seqs	Reads	Type	pct.
ATGACGGTCAAGTCCGA|CGA**CGG**TCTC	66122	WT	35.33%
ATGACGGTCAAGTCCGAaCGACGGTCTC	30846	+1 a	16.48%
AT----------------GACGGTCTC	21399	-16	11.43%
ATGACGGTCAAGTC---CGACGGTCTC	17013	-3	9.09%
ATGACGGTCAAGTCCGA-GACGGTCTC	5789	-1	3.09%
ATGACGGTCAAGTCCG-CGACGGTCTC	3241	-1	1.73%
ATGACGGTCAAG---------------	2493	-15	1.33%
ATGACGGTCAA-----------GTCTC	1803	-11	0.96%
ATGACGGTCAAGTCCGAgCGACGGTCTC	1323	+1 g	0.71%
ATGACGGTCAAGT----CGACGGTCTC	1171	-4	0.63%

P167-YW-K562-Cas9-puro-48h-Syn103crBCL11A9e	48h	Ins	Del	HDR
14313 | 89.53%		22.09%	67.44%	0.00%

Typical seqs	Reads	Type	pct.
ATGACGGTCAAGTCCGA|CGA**CGG**TCTC	1499	WT	10.47%
AT----------------GACGGTCTC	3805	-16	26.58%
ATGACGGTCAAGTCCGAaCGACGGTCTC	2476	+1 a	17.30%
ATGACGGTCAAGTC---CGACGGTCTC	1745	-3	12.19%
ATGACGGTCAAG---------------	441	-15	3.08%
ATGACGGTCAAGTCCGA-GACGGTCTC	421	-1	2.94%
ATGACGGTCAA-----------GTCTC	352	-11	2.46%
ATGACGGTCAAGTCCG-CGACGGTCTC	229	-1	1.60%
ATGACGGTCAAGTC------CGGTCTC	110	-6	0.77%
ATGACGGTCAAGTCCGAgCGACGGTCTC	102	+1 g	0.71%

P163-P165-YW-K562-Cas9-Syn104BCL11A-KO

Total | INDEL 110174 | 1.41%	4h	Ins 0.26%	Del 1.15%	HDR 0.00%
**Typical seqs**		**Reads**	**Type**	**pct.**
CATATTAGTGGTCCGGG|CCC**GGG**CAGG		108621	WT	98.59%
CATATTAGTGGT------CCGGGCAGG		802	-6	0.73%
CATATTAGTGGTCCGGG-CCGGGCAGG		211	-1	0.19%
CATATTAGTGGTCCGGGgCCCGGGCAGG		174	+1 g	0.16%
CATATTAGT------GGCCCGGGCAGG		52	-6	0.05%
CATATTAGTGGT-----CCCGGGCAGG		51	-5	0.05%
CATATTAGT-----------GGGCAGG		46	-11	0.04%
CATATTAGTGGTCCGGGcCCCGGGCAGG		34	+1 c	0.03%
CATATTAGTGGTCCGGGCAGGCCCGGGCAGG		32	+4	0.03%
CATATTAGTGGTCCGGACAGCCGGGCAGG		26	+2	0.02%

Total | INDEL 57141 | 3.48%	8h	Ins 0.19%	Del 3.28%	HDR 0.00%
**Typical seqs**		**Reads**	**Type**	**pct.**
CATATTAGTGGTCCGGG|CCC**GGG**CAGG		55154	WT	96.52%
CATATTAGTGGT------CCGGGCAGG		486	-6	0.85%
CATATTAGTGGTCC-GGCCCGGGCAGG		205	-1	0.36%
CATATTAGTGGTCCGGG-CCGGGCAGG		184	-1	0.32%
CATATTAGTGGTCCGGG--CGGGCAGG		173	-2	0.30%
CATATTAGTGGTCCGGGgCCCGGGCAGG		81	+1 g	0.14%
CATATTAGTGGTCC--GCCCGGGCAGG		75	-2	0.13%
CATATTAGTGGTCCG----------GG		62	-10	0.11%
CATATTAGTGGTCCG-----GGGCAGG		46	-5	0.08%
CATATTAGT------GGCCCGGGCAGG		41	-6	0.07%

Total | INDEL 316529 | 8.10%	12h	Ins 0.35%	Del 7.75%	HDR 0.00%
**Typical seqs**		**Reads**	**Type**	**pct.**
CATATTAGTGGTCCGGG|CCC**GGG**CAGG		290879	WT	91.90%
CATATTAGTGGT------CCGGGCAGG		7797	-6	2.46%
CATATTAGTGGTCCGGG--CGGGCAGG		2551	-2	0.81%
CATATTAGTGGTCC-GGCCCGGGCAGG		1957	-1	0.62%
CATATTAGTGGTCCGGG-CCGGGCAGG		1788	-1	0.56%
CATATTAGT------GGCCCGGGCAGG		874	-6	0.28%
CATATTAGTGGTCCG-----GGGCAGG		599	-5	0.19%
CATATTAGTGGT-----CCCGGGCAGG		557	-5	0.18%
CATATTAGTGGTCCG----------GG		560	-10	0.18%
CATATTAGTGGTCC--GCCCGGGCAGG		544	-2	0.17%

P166-YW-K562-Cas9-puro-24h-Syn104crBCL11A9f 372625 | 24.69%	24h	Ins 0.65%	Del 24.05%	HDR 0.00%
**Typical seqs**		**Reads**	**Type**	**pct.**
CATATTAGTGGTCCGGG|CCC**GGG**CAGG		280607	WT	75.31%
CATATTAGTGGT------CCGGGCAGG		46455	-6	12.47%
CATATTAGTGGTCCGGG--CGGGCAGG		4114	-2	1.10%
CATATTAGTGGTCC-GGCCCGGGCAGG		4048	-1	1.09%
CATATTAGTGGTCCGGG-CCGGGCAGG		3356	-1	0.90%
CATATTAGT------GGCCCGGGCAGG		2984	-6	0.80%
CATATTAGTGGT-----CCCGGGCAGG		1929	-5	0.52%
CATATTAGTGGTCCG-----GGGCAGG		1413	-5	0.38%
CATATTAGTGGTCC--GCCCGGGCAGG		1320	-2	0.35%
CATATTAGTGGTCCG----------GG		1247	-10	0.33%

P167-YW-K562-Cas9-puro-48h-Syn104crBCL11A9f 62087 | 40.20%	48h	Ins 0.62%	Del 39.58%	HDR 0.00%
**Typical seqs**		**Reads**	**Type**	**pct.**
CATATTAGTGGTCCGGG|CCC**GGG**CAGG		37128	WT	59.80%
CATATTAGTGGT------CCGGGCAGG		13725	-6	22.11%
CATATTAGTGGTCCGGG--CGGGCAGG		890	-2	1.43%
CATATTAGTGGTCC-GGCCCGGGCAGG		889	-1	1.43%
CATATTAGT------GGCCCGGGCAGG		860	-6	1.39%
CATATTAGTGGTCCGGG-CCGGGCAGG		686	-1	1.10%
CATATTAGTGGT-----CCCGGGCAGG		543	-5	0.87%
CATATTAGTGGTCCG-----GGGCAGG		355	-5	0.57%
CATATTAGTGGTCC--GCCCGGGCAGG		341	-2	0.55%
CATATTAGTGGTCCG----------GG		384	-10	0.62%

P161-P165-YW-K562-RNP-Syn20AAVS1-AAVKI

1h

Total | INDEL	Ins	Del	HDR
213397 | 2.19%	**2.06%**	**0.12%**	**0.01%**

Typical seqs	Reads	Type	pct.
TCTAACCCCCACCTCCT|GTT**AGG**CAGA	208732	WT	97.81%
TCTAACCCCCACCTCCTtGTTAGGCAGA	3922	+1 t	1.84%
TCTAACCCCCACCTCCTG-TAGGCAGA	50	-1	0.02%
TCTAACCCCCACCTCC--TTAGGCAGA	45	-2	0.02%
TCTAACCCCCACCCCCTTGTTAGGCAGA	26	+1 Ins	0.01%
TCTAACCCCCACCTC--GTTAGGCAGA	24	-2	0.01%
TCTACCCCCCACCTCCTTGTTAGGCAGA	23	+1 Ins	0.01%
TCTAACCCCCACCTCCTgGTTAGGCAGA	23	+1 g	0.01%
TCTAACCCC-------GTTAGGCAGA	23	-7	0.01%
TCTAACCCCCACCTC-TGTTAGGCAGA	21	-1	0.01%

2h

Total | INDEL	Ins	Del	HDR
230123 | 7.34%	**6.96%**	**0.37%**	**0.01%**

Typical seqs	Reads	Type	pct.
TCTAACCCCCACCTCCT|GTT**AGG**CAGA	213227	WT	92.66%
TCTAACCCCCACCTCCTtGTTAGGCAGA	14175	+1 t	6.16%
TCTAACCCCCACCT---GTTAGGCAGA	132	-3	0.06%
TCTAACCCCCCCCTCCTTGTTAGGCAGA	119	+1 Ins	0.05%
TCTAACCCCCACCTCC--TTAGGCAGA	117	-2	0.05%
TCTAACCCCCACCTC--GTTAGGCAGA	97	-2	0.04%
TCTAACCCCCACCTC-TGTTAGGCAGA	93	-1	0.04%
TCTAACCCCCACCCCCTTGTTAGGCAGA	92	+1 Ins	0.04%
TCTAACCCCCACCTCCTtGTTAGGCGGA	86	+1 t	0.04%
TCTAACCCCCACCTCCCTGTTAGGCAGA	84	+1 Ins	0.04%

3h

Total | INDEL	Ins	Del	HDR
298882 | 13.06%	**12.21%**	**0.74%**	**0.12%**

Typical seqs	Reads	Type	pct.
TCTAACCCCCACCTCCT|GTT**AGG**CAGA	259842	WT	86.94%
TCTAACCCCCACCTCCTtGTTAGGCAGA	32419	+1 t	10.85%
TCTAACCCCCACCTCC--TTAGGCAGA	592	-2	0.20%
TCTAACCCACGCGTAGTTTAAACTTAGGCAGA	343	+5 HDR	0.11%
TCTAACCCCCACCTC-TGTTAGGCAGA	337	-1	0.11%
TCTAACCCCCACCT---GTTAGGCAGA	163	-3	0.05%
TCTAGCCCCCACCTCCTTGTTAGGCAGA	158	+1 Ins	0.05%
TCTAACCCCCACCTCCTcGTTAGGCAGA	160	+1 c	0.05%
TCTAACCCCCACCTCCCTGTTAGG[illegible]	163	+1 Ins	0.05%
TCTAACC[illegible]TTAGGCAGA	161	+1 Ins	0.05%

48h

Total | INDEL	Ins	Del	HDR
204527 | 86.10%	**58.75%**	**13.40%**	**13.96%**

Typical seqs	Reads	Type	pct.
TCTAACCCCCACCTCCT|GTT**AGG**CAGA	28428	WT	13.90%
TCTAACCCCCACCTCCTtGTTAGGCAGA	104472	+1 t	51.08%
TCTAACCCACGCGTAGTTTAAACTTAGGCAGA	27087	+5 HDR	13.24%
TCTAACCCCCACCTCC--TTAGGCAGA	4666	-2	2.28%
TCTAACCCCCACCTC-TGTTAGGCAGA	2214	-1	1.08%
TCTAACCCCCACC--------------	1794	-15	0.88%
TCTAACCCCCACCT---GTTAGGCAGA	1494	-3	0.73%
TCTAACCCCCACCTC--GTTAGGCAGA	1343	-2	0.66%
TCTAACCCCCACCTCC-GTTAGGCAGA	1314	-1	0.64%
TCTAACCC[illegible]CTGTTAGGCAGA	989	-4	0.48%

4h

Total | INDEL	Ins	Del	HDR
135911 | 22.33%	**19.32%**	**2.02%**	**0.99%**

Typical seqs	Reads	Type	pct.
TCTAACCCCCACCTCCT|GTT**AGG**CAGA	105564	WT	77.67%
TCTAACCCCCACCTCCTtGTTAGGCAGA	22932	+1 t	16.87%
TCTAACCCACGCGTAGTTTAAACTTAGGCAGA	1295	+5 HDR	0.95%
TCTAACCCCCACCTCC--TTAGGCAGA	751	-2	0.55%
TCTAACCCCCACCTC-TGTTAGGCAGA	367	-1	0.27%
TCTAACCCCCACCT---GTTAGGCAGA	296	-3	0.22%
TCTAACCCCCACCTCCTCTGTTAGGCAGA	230	+2	0.17%
TCTAACCCCCACCTCCT-TTAGGCAGA	226	-1	0.17%
TCTAACCCCCACCTC--GTTAGGCAGA	146	-2	0.11%
TCTAACCCCCACCCCCTTGTTAGGCAGA	135	+1 Ins	0.10%

9h

Total | INDEL	Ins	Del	HDR
206773 | 45.87%	**36.91%**	**4.53%**	**4.43%**

Typical seqs	Reads	Type	pct.
TCTAACCCCCACCTCCT|GTT**AGG**CAGA	111923	WT	54.13%
TCTAACCCCCACCTCCTtGTTAGGCAGA	66999	+1 t	32.40%
TCTAACCCACGCGTAGTTTAAACTTAGGCAGA	8674	+5 HDR	4.19%
TCTAACCCCCACCTCC--TTAGGCAGA	2173	-2	1.05%
TCTAACCCCCACCTC-TGTTAGGCAGA	953	-1	0.46%
TCTAACCCCCACCTCC-GTTAGGCAGA	796	-1	0.38%
TCTAACCCCCACCTC--GTTAGGCAGA	647	-2	0.31%
TCTAACCCCCCCCTCCTTGTTAGGCAGA	436	+1 Ins	0.21%
TCTAACCCCCACCCCCTTGTTAGGCAGA	411	+1 Ins	0.20%
TCTAACCCCCGCCTCCTTGTTAGGCAGA	394	+1 Ins	0.19%

24h

Total | INDEL	Ins	Del	HDR
202092 | 73.21%	**52.25%**	**10.50%**	**10.46%**

Typical seqs	Reads	Type	pct.
TCTAACCCCCACCTCCT|GTT**AGG**CAGA	54150	WT	26.79%
TCTAACCCCCACCTCCTtGTTAGGCAGA	91551	+1 t	45.30%
TCTAACCCACGCGTAGTTTAAACTTAGGCAGA	20006	+5 HDR	9.90%
TCTAACCCCCACCTCC--TTAGGCAGA	3318	-2	1.64%
TCTAACCCCCACCTC-TGTTAGGCAGA	2208	-1	1.09%
TCTAACCCCCACCTCC-GTTAGGCAGA	1524	-1	0.75%
TCTAACCCCCA----CTGTTAGGCAGA	1109	-4	0.55%
TCTAACCCCCACCTC--GTTAGGCAGA	1096	-2	0.54%
TCTAACCCCCACCT---GTTAGGCAGA	894	-3	0.44%
TCTAACCCCCACCTCCT-TTAGGCAGA	712	-1	0.35%

P161-P165-YW-K562-RNP-Syn22AAVS1-AAVKI

1h

Total | INDEL	Ins	Del	HDR
150233 | 0.35%	**0.22%**	**0.12%**	**0.02%**

**Typical seqs**	**Reads**	**Type**	**pct.**
AATCTGCCTAACAGGAG|GTG**GGG**GTTA	149708	WT	99.65%
AATCTGCCTAACAGGAGgGTGGGGGTTA	179	+1 g	0.12%
AATCTGCCTAACAAGGAGGTGGGGGTTA	121	+1 Ins	0.08%
AATCTGCCTAACAGG---TGGGGGTTA	71	-3	0.05%
AATCTGCCTAAGTTTAAACTACGCGTGGGTTA	25	+5 HDR	0.02%
AATCTGCCTAAC----GGTGGGGGTTA	19	-4	0.01%
AATCTGCCTAACAGGA-GTGGGGGTTA	18	-1	0.01%
AATCTGCCTAACAGG-GGTGGGGGTTA	13	-1	0.01%
AATCTGCCTAACA-GAGGTGGGGGTTA	11	-1	0.01%
AATCTGCCTAACAGGAGaGTGGGGGTTA	10	+1 a	0.01%

2h

Total | INDEL	Ins	Del	HDR
248682 | 1.53%	**1.04%**	**0.48%**	**0.01%**

**Typical seqs**	**Reads**	**Type**	**pct.**
AATCTGCCTAACAGGAG|GTG**GGG**GTTA	244879	WT	98.47%
AATCTGCCTAACAGGAGgGTGGGGGTTA	1952	+1 g	0.78%
AATCTGCCTAACAGGA-GTGGGGGTTA	513	-1	0.21%
AATCTGCCTAACAAGGAGGTGGGGGTTA	201	+1 Ins	0.08%
AATCTGCCTAACAGG---TGGGGGTTA	192	-3	0.08%
AATCTGCCTAACAGGAGaGTGGGGGTTA	82	+1 a	0.03%
AATCTGCCTAAC----GGTGGGGGTTA	73	-4	0.03%
AATCTGCCTAACAGGAGCGGTGGGGGTTA	69	+2	0.03%
AATCTGCCTA-------GTGGGGGTTA	53	-7	0.02%
AATCTGCCTAACAG-----TGGGGTTA	40	-5	0.02%

3h

Total | INDEL	Ins	Del	HDR
131026 | 2.99%	**1.69%**	**1.21%**	**0.09%**

**Typical seqs**	**Reads**	**Type**	**pct.**
AATCTGCCTAACAGGAG|GTG**GGG**GTTA	127110	WT	97.01%
AATCTGCCTAACAGGAGgGTGGGGGTTA	1750	+1 g	1.34%
AATCTGCCTAACAGGA-GTGGGGGTTA	584	-1	0.45%
AATCTGCCTAACAGG---TGGGGGTTA	357	-3	0.27%
AATCTGCCTAAGTTTAAACTACGCGTGGGTTA	118	+5 HDR	0.09%
AATCTGCCTAAC----GGTGGGGGTTA	113	-4	0.09%
AATCTGCCTAACAAGGAGGTGGGGGTTA	96	+1 Ins	0.07%
AATCTGCCTAACA----GTGGGGGTTA	81	-4	0.06%
AATCTGCCTA-------GTGGGGGTTA	81	-7	0.06%
AATCTGCCTAACAGGAGAGGTGGGG[illegible]	78	+2	0.06%

48h

Total | INDEL	Ins	Del	HDR
150761 | 53.14%	**15.79%**	**20.80%**	**16.55%**

**Typical seqs**	**Reads**	**Type**	**pct.**
AATCTGCCTAACAGGAG|GTG**GGG**GTTA	70651	WT	46.86%
AATCTGCCTAAGTTTAAACTACGCGTGGGTTA	23631	+5 HDR	15.67%
AATCTGCCTAACAGGAGgGTGGGGGTTA	16960	+1 g	11.25%
AATCTGCCTAACAGGA-GTGGGGGTTA	7312	-1	4.85%
AATCTGCCTAACAGG---TGGGGGTTA	5292	-3	3.51%
AATCTGCCTAAC----GGTGGGGGTTA	1018	-4	0.68%
AATCTGCCTAACAG--GGTGGGGGTTA	985	-2	0.65%
AATCTGCCTAAC-----GTGGGGGTTA	867	-5	0.58%
AATCTGCCTAACAGG-GGTGGGGGTTA	860	-1	0.57%
AATCTGCCTAACAGGAGAGGTGGGGGTTA	704	+2	0.47%

4h

Total | INDEL	Ins	Del	HDR
135893 | 7.62%	**3.64%**	**3.31%**	**0.68%**

**Typical seqs**	**Reads**	**Type**	**pct.**
AATCTGCCTAACAGGAG|GTG**GGG**GTTA	125533	WT	92.38%
AATCTGCCTAACAGGAGgGTGGGGGTTA	3381	+1 g	2.49%
AATCTGCCTAACAGGA-GTGGGGGTTA	1704	-1	1.25%
AATCTGCCTAACAGG---TGGGGGTTA	1056	-3	0.78%
AATCTGCCTAAGTTTAAACTACGCGTGGGTTA	883	+5 HDR	0.65%
AATCTGCCTAACAAGGAGGTGGGGGTTA	503	+1 Ins	0.37%
AATCTGCCTAAC----GGTGGGGGTTA	187	-4	0.14%
AATCTGCCTAACAGGAGtGTGGGGGTTA	165	+1 t	0.12%
AATCTGCCTAACA----GTGGGGGTTA	124	-4	0.09%
AATCTGCCTAACAG--GGTGGGGGTTA	116	-2	0.09%

9h

Total | INDEL	Ins	Del	HDR
167089 | 19.17%	**7.61%**	**7.63%**	**3.94%**

**Typical seqs**	**Reads**	**Type**	**pct.**
AATCTGCCTAACAGGAG|GTG**GGG**GTTA	135053	WT	80.83%
AATCTGCCTAACAGGAGgGTGGGGGTTA	9377	+1 g	5.61%
AATCTGCCTAAGTTTAAACTACGCGTGGGTTA	6234	+5 HDR	3.73%
AATCTGCCTAACAGGA-GTGGGGGTTA	3862	-1	2.31%
AATCTGCCTAACAGG---TGGGGGTTA	2747	-3	1.64%
AATCTGCCTAACAAGGAGGTGGGGGTTA	742	+1 Ins	0.44%
AATCTGCCTAAC----GGTGGGGGTTA	695	-4	0.42%
AATCTGCCTAACAGG-GGTGGGGGTTA	379	-1	0.23%
AATCTGCCTAACAG--GGTGGGGGTTA	373	-2	0.22%
AATCTGCCTAACAGGAGtGTGGGGGTTA	255	+1 t	0.15%

24h

Total | INDEL	Ins	Del	HDR
64560 | 38.89%	**12.02%**	**14.68%**	**12.19%**

**Typical seqs**	**Reads**	**Type**	**pct.**
AATCTGCCTAACAGGAG|GTG**GGG**GTTA	39451	WT	61.11%
AATCTGCCTAAGTTTAAACTACGCGTGGGTTA	7410	+5 HDR	11.48%
AATCTGCCTAACAGGAGgGTGGGGGTTA	5419	+1 g	8.39%
AATCTGCCTAACAGGA-GTGGGGGTTA	2645	-1	4.10%
AATCTGCCTAACAGG---TGGGGGTTA	1385	-3	2.15%
AATCTGCCTAAC----GGTGGGGGTTA	427	-4	0.66%
AATCTGCCTAACAAGGAGGTGGGGGTTA	346	+1 Ins	0.54%
AATCTGCCTAACAG--GGTGGGGGTTA	296	-2	0.46%
AATCTGCCTAAC-----GTGGGGGTTA	289	-5	0.45%
AATCTGCCTAACA----GTGGGGGTTA	243	-4	0.38%

P161-P165-YW-K562-RNP-Syn23AAVS1-KO

1h

Total	INDEL	Ins	Del	HDR
103624	0.37%	0.18%	0.18%	0.02%

Typical seqs	Reads	Type	pct.
GAATCTGCCTAACAGGA|GGTGGGGGTT	103236	WT	99.63%
GAATCTGCCTAACAGGAaGGTGGGGGTT	76	+1 a	0.07%
GAATCTGCCTAACAAGGAGGTGGGGGTT	54	+1 Ins	0.05%
GAATCTGCCTAACCAGGAGGTGGGGGTT	30	+1 Ins	0.03%
GAATCTGCC--------GGTGGGGGTT	29	-8	0.03%
GAATCTGCCTAACAG--GGTGGGGGTT	24	-2	0.02%
GAATCTGCCTAACAGGAgGGTGGGGGTT	23	+1 g	0.02%
GAATCTGCCTAACAGGA-GTGGGGGTT	21	-1	0.02%
GAATCTGCCTA--AGGAGGTGGGGGTT	19	-2	0.02%
GAATCTGCCTAACA---GGTGGGGGTT	17	-3	0.02%

2h

Total	INDEL	Ins	Del	HDR
160636	0.76%	0.44%	0.32%	0.01%

Typical seqs	Reads	Type	pct.
GAATCTGCCTAACAGGA|GGTGGGGGTT	159409	WT	99.24%
GAATCTGCCTAACAGGAaGGTGGGGGTT	522	+1 a	0.32%
GAATCTGCCT-------GGTGGGGGTT	96	-7	0.06%
GAATCTGCCTAACA---GGTGGGGGTT	88	-3	0.05%
GAATCTGCCTAACAGG-GGTGGGGGTT	65	-1	0.04%
GAATCTGCCTAACAGGA-GTGGGGGTT	61	-1	0.04%
GAATCTGCCTAACAGGAgGGTGGGGGTT	55	+1 g	0.03%
GAATCTGCCTAAC----GGTGGGGGTT	51	-4	0.03%
GAATCTGCCTAACAAGGAGGTGGGGGTT	38	+1 Ins	0.02%
GAATCTGCCTAACA-GAGGTGGGGGTT	30	-1	0.02%

3h

Total	INDEL	Ins	Del	HDR
58760	2.04%	0.85%	1.18%	0.01%

Typical seqs	Reads	Type	pct.
GAATCTGCCTAACAGGA|GGTGGGGGTT	57561	WT	97.96%
GAATCTGCCTAACAGGAaGGTGGGGGTT	324	+1 a	0.55%
GAATCTGCCTAACA---GGTGGGGGTT	135	-3	0.23%
GAATCTGCCTAACAGGA-GTGGGGGTT	113	-1	0.19%
GAATCTGCCTAACAGG-GGTGGGGGTT	82	-1	0.14%
GAATCTGCCTAACAG--GGTGGGGGTT	81	-2	0.14%
GAATCTGCCTAACAGGAgGGTGGGGGTT	60	+1 g	0.10%
GAATCTGCCTAAC----GGTGGGGGTT	52	-4	0.09%
GAATCTGCCTAACAGGAcGGTGGGGGTT	38	+1 c	0.06%
GAATCTGCCTA-----AGGTGG[illegible]	26	-5	0.04%

48h

Total	INDEL	Ins	Del	HDR
91169	54.19%	10.39%	42.83%	0.98%

Typical seqs	Reads	Type	pct.
GAATCTGCCTAACAGGA|GGTGGGGGTT	41761	WT	45.81%
GAATCTGCCTAACA---GGTGGGGGTT	13487	-3	14.79%
GAATCTGCCTAACAGGAaGGTGGGGGTT	5359	+1 a	5.88%
GAATCTGCCTAACAGGA-GTGGGGGTT	2105	-1	2.31%
GAATCTGCCTAACAGG-GGTGGGGGTT	1360	-1	1.49%
GAATCTGCCTAACAG--GGTGGGGGTT	1222	-2	1.34%
GAATCTGCCTAAC----GGTGGGGGTT	1078	-4	1.18%
GAATCTGCCTAACAGGA--------TT	940	-8	1.03%
GAATCTGCCTAACAGGA--TGGGGGTT	905	-2	0.99%
GAATCTGCCTAAGTTTAAACTACGCGTGGGTT	856	+5 HDR	0.94%

4h

Total	INDEL	Ins	Del	HDR
47340	3.86%	1.37%	2.48%	0.01%

Typical seqs	Reads	Type	pct.
GAATCTGCCTAACAGGA|GGTGGGGGTT	45511	WT	96.14%
GAATCTGCCTAACAGGAaGGTGGGGGTT	390	+1 a	0.82%
GAATCTGCCTAACA---GGTGGGGGTT	245	-3	0.52%
GAATCTGCCTAACCAGGAGGTGGGGGTT	87	+1 Ins	0.18%
GAATCTGCCTAAC----GGTGGGGGTT	87	-4	0.18%
GAATCTGCCTAACAGG-GGTGGGGGTT	86	-1	0.18%
GAATCTGCCTAACAGGA-GTGGGGGTT	78	-1	0.16%
GAATCTGCCTAACAG--GGTGGGGGTT	59	-2	0.12%
GAATCTGCCT------AGGTGGGGGTT	48	-6	0.10%
GAATCTGCCTA--AGGAGGTGGGGGTT	47	-2	0.10%

9h

Total	INDEL	Ins	Del	HDR
52759	10.23%	3.32%	6.90%	0.01%

Typical seqs	Reads	Type	pct.
GAATCTGCCTAACAGGA|GGTGGGGGTT	47362	WT	89.77%
GAATCTGCCTAACAGGAaGGTGGGGGTT	1246	+1 a	2.36%
GAATCTGCCTAACA---GGTGGGGGTT	842	-3	1.60%
GAATCTGCCTAACAGGA-GTGGGGGTT	369	-1	0.70%
GAATCTGCCTAACAG--GGTGGGGGTT	262	-2	0.50%
GAATCTGCCTAAC----GGTGGGGGTT	180	-4	0.34%
GAATCTGC---------GGTGGGGGTT	175	-9	0.33%
GAATCTGCCTAACAGG-GGTGGGGGTT	153	-1	0.29%
GAATCTGCCTAACAGGA--TGGGGGTT	151	-2	0.29%
GAATCTGCCTA-----AGGTGGGGGTT	118	-5	0.22%

24h

Total	INDEL	Ins	Del	HDR
82276	28.73%	6.11%	22.62%	0.00%

Typical seqs	Reads	Type	pct.
GAATCTGCCTAACAGGA|GGTGGGGGTT	58636	WT	71.27%
GAATCTGCCTAACA---GGTGGGGGTT	4396	-3	5.34%
GAATCTGCCTAACAGGAaGGTGGGGGTT	3271	+1 a	3.98%
GAATCTGCCTAACAGGA-GTGGGGGTT	1522	-1	1.85%
GAATCTGCCTAACAG--GGTGGGGGTT	1163	-2	1.41%
GAATCTGCCTAAC----GGTGGGGGTT	1054	-4	1.28%
GAATCTGCCTAACAGG-GGTGGGGGTT	907	-1	1.10%
GAATCTGCCTA-----AGGTGGGGGTT	718	-5	0.87%
GAATCTGC---------GGTGGGGGTT	536	-9	0.65%
GAATCTGCCT------AGGTGGGGGTT	459	-6	0.56%

P161-P165-YW-K562-RNP-Syn23AAVS1-AAVKI

1h

Total	INDEL	Ins	Del	HDR
191496	0.32%	0.22%	0.08%	0.02%

Typical seqs	Reads	Type	pct.
GAATCTGCCTAACAGGA|GGT**GGG**GGTT	190885	WT	99.68%
GAATCTGCCTAACAAGGAGGTGGGGGTT	239	+1 Ins	0.12%
GAATCTGCCTAACAGGAaGGTGGGGGTT	115	+1 a	0.06%
GAATCTGCCTAAGTTTAAACTACGCGTGGGTT	31	+5 HDR	0.02%
GAATCTGCCTAACCAGGAGGTGGGGGTT	28	+1 Ins	0.01%
GAATCTGCCTAACA---GGTGGGGGTT	20	-3	0.01%
GAATCTGCCTAACAGGAgGGTGGGGGTT	18	+1 g	0.01%
GAATCTGCCTAACAGG-GGTGGGGGTT	18	-1	0.01%
GAATCTGCCTAACAGGA-GTGGGGGTT	17	-1	0.01%
GAATCTGCCTAACAGGAGG-GGGGGTT	15	-1	0.01%

2h

Total	INDEL	Ins	Del	HDR
177550	0.84%	0.49%	0.33%	0.01%

Typical seqs	Reads	Type	pct.
GAATCTGCCTAACAGGA|GGT**GGG**GGTT	176066	WT	99.16%
GAATCTGCCTAACAGGAaGGTGGGGGTT	495	+1 a	0.28%
GAATCTGCCTAACAAGGAGGTGGGGGTT	158	+1 Ins	0.09%
GAATCTGCCTAACA---GGTGGGGGTT	165	-3	0.09%
GAATCTGCCTAACAGGA--TGGGGGTT	95	-2	0.05%
GAATCTGCCTAACAGGAtGGTGGGGGTT	98	+1 t	0.06%
GAATCTGCCTAACAGGA-GTGGGGGTT	82	-1	0.05%
GAATCTGCCTAACAG--GGTGGGGGTT	47	-2	0.03%
GA--------------AGGTGGGGGTT	32	-14	0.02%
GAATCTGCCTAACAGG-GGTGGGGGTT	29	-1	0.02%

3h

Total	INDEL	Ins	Del	HDR
124756	1.89%	0.99%	0.83%	0.07%

Typical seqs	Reads	Type	pct.
GAATCTGCCTAACAGGA|GGT**GGG**GGTT	122393	WT	98.11%
GAATCTGCCTAACAGGAaGGTGGGGGTT	770	+1 a	0.62%
GAATCTGCCTAACAGGA-GTGGGGGTT	237	-1	0.19%
GAATCTGCCTAACAAGGAGGTGGGGGTT	115	+1 Ins	0.09%
GAATCTGCCTAACAGG-GGTGGGGGTT	102	-1	0.08%
GAATCTGCCTAACAG--GGTGGGGGTT	88	-2	0.07%
GAATCTGCCTAACA---GGTGGGGGTT	89	-3	0.07%
GAATCTGCCTAACAGGAcGGTGGGGGTT	81	+1 c	0.06%
GAATCTGCCTAAGTTTAAACTACGCGTGGGTT	88	+5 HDR	0.07%
GAATCTGCCTAACAGGAtGGTGGG[illegible]	80	+1 t	0.06%

48h

Total	INDEL	Ins	Del	HDR
200245	66.26%	11.14%	26.06%	29.06%

Typical seqs	Reads	Type	pct.
GAATCTGCCTAACAGGA|GGT**GGG**GGTT	67556	WT	33.74%
GAATCTGCCTAAGTTTAAACTACGCGTGGGTT	55187	+5 HDR	27.56%
GAATCTGCCTAACA---GGTGGGGGTT	11889	-3	5.94%
GAATCTGCCTAACAGGAaGGTGGGGGTT	10996	+1 a	5.49%
GAATCTGCCTAACAGGA-GTGGGGGTT	4153	-1	2.07%
GAATCTGCCTAACAG--GGTGGGGGTT	3017	-2	1.51%
GAATCTGCCTAACAGG-GGTGGGGGTT	2848	-1	1.42%
GAATCTGCCTAAC----GGTGGGGGTT	1906	-4	0.95%
GAATCTGCCT-----AGG[illegible]GGTT	1612	[illegible]	0.81%
GAATCTGCCTA[illegible]GGTT	1607	-2	0.80%

4h

Total	INDEL	Ins	Del	HDR
185156	4.88%	2.04%	1.91%	0.93%

Typical seqs	Reads	Type	pct.
GAATCTGCCTAACAGGA|GGT**GGG**GGTT	176127	WT	95.12%
GAATCTGCCTAACAGGAaGGTGGGGGTT	1683	+1 a	0.91%
GAATCTGCCTAAGTTTAAACTACGCGTGGGTT	1633	+5 HDR	0.88%
GAATCTGCCTAACAAGGAGGTGGGGGTT	1005	+1 Ins	0.54%
GAATCTGCCTAACA---GGTGGGGGTT	587	-3	0.32%
GAATCTGCCTAACAGGA-GTGGGGGTT	561	-1	0.30%
GAATCTGCCTAACAGG-GGTGGGGGTT	277	-1	0.15%
GAATCTGCCTAACAGGA---GGGGGTT	216	-3	0.12%
GAATCTGCCTA-----AGGTGGGGGTT	209	-5	0.11%
GAATCTGCCTAACAGGA--TGGGGGTT	185	-2	0.10%

9h

Total	INDEL	Ins	Del	HDR
147563	16.45%	4.26%	6.31%	5.87%

Typical seqs	Reads	Type	pct
GAATCTGCCTAACAGGA|GGT**GGG**GGTT	123292	WT	83.55%
GAATCTGCCTAAGTTTAAACTACGCGTGGGTT	8235	+5 HDR	5.58%
GAATCTGCCTAACAGGAaGGTGGGGGTT	2967	+1 a	2.01%
GAATCTGCCTAACA---GGTGGGGGTT	1858	-3	1.26%
GAATCTGCCTAACAGGA-GTGGGGGTT	952	-1	0.65%
GAATCTGCCTAACAAGGAGGTGGGGGTT	743	+1 Ins	0.50%
GAATCTGCCTAACAGG-GGTGGGGGTT	719	-1	0.49%
GAATCTGCCTAAC----GGTGGGGGTT	616	-4	0.42%
GAATCTGCCTAACAG--GGTGGGGGTT	553	-2	0.37%
GAATCTGCCTAACAGGA--TGGGGGTT	497	-2	0.34%

24h

Total	INDEL	Ins	Del	HDR
73651	45.53%	9.05%	16.71%	19.77%

Typical seqs	Reads	Type	pct.
GAATCTGCCTAACAGGA|GGT**GGG**GGTT	40117	WT	54.47%
GAATCTGCCTAAGTTTAAACTACGCGTGGGTT	13714	+5 HDR	18.62%
GAATCTGCCTAACAGGAaGGTGGGGGTT	3378	+1 a	4.59%
GAATCTGCCTAACA---GGTGGGGGTT	2279	-3	3.09%
GAATCTGCCTAAC----GGTGGGGGTT	946	-4	1.28%
GAATCTGCCTAACAG--GGTGGGGGTT	907	-2	1.23%
GAATCTGCCTAACAGGA-GTGGGGGTT	831	-1	1.13%
GAATCTGCCTAACAGG-GGTGGGGGTT	745	-1	1.01%
GAATCTGCCTAACAAGGAGGTGGGGGTT	451	+1 Ins	0.61%
GAATCTGCCTA-----AGGTGGGGGTT	447	-5	0.61%

P161-P165-YW-K562-RNP-Syn24AAVS1-KO

1h

Total	INDEL	Ins	Del	HDR
221015	0.18%	**0.06%**	**0.12%**	**0.00%**

Typical seqs	Reads	Type	pct.
GGAATCTGCCTAACAGG|AGG**TGG**GGGT	220610	WT	99.82%
GGAATCTGCCT-ACAGGAGGTGGGGGT	43	-1	0.02%
GGAATCTGCCTAACCAGGAGGTGGGGGT	42	+1 Ins	0.02%
GGAATCTGCCTAACAG-AGGTGGGGGT	40	-1	0.02%
GGAATCTGCCTAACAAGGAGGTGGGGGT	33	+1 Ins	0.01%
GGAATCTGCCTAAC---AGGTGGGGGT	32	-3	0.01%
GGAATCTGCCTAACAGGgAGGTGGGGGT	23	+1 g	0.01%
GGAATCTGCCTA--AGGAGGTGGGGGT	19	-2	0.01%
GGAATCTGCCTAACAGGAGG-GGGGGT	19	-1	0.01%
GGAATCTGCCTAACAGG-GGTGGGGGT	18	-1	0.01%

2h

Total	INDEL	Ins	Del	HDR
311314	0.66%	**0.09%**	**0.55%**	**0.01%**

Typical seqs	Reads	Type	pct.
GGAATCTGCCTAACAGG|AGG**TGG**GGGT	309272	WT	99.34%
GGAATCTGCCTAACA--AGGTGGGGGT	447	-2	0.14%
GGAATCTGCCTAAC---AGGTGGGGGT	295	-3	0.09%
GGAATCTGCCTAACAG-AGGTGGGGGT	206	-1	0.07%
GGAATCTGCCTAACAGG-GGTGGGGGT	148	-1	0.05%
GGAATCTGCCTAACAG--GGTGGGGGT	130	-2	0.04%
GGAATCTGCCTAA----AGGTGGGGGT	129	-4	0.04%
GGAATCTGCCTAACAGGgAGGTGGGGGT	102	+1 g	0.03%
GGAATCTGCCT-ACAGGAGGTGGGGGT	82	-1	0.03%
GGAATCTGCCTAACAAGGAGGTGGGGGT	70	+1 Ins	0.02%

3h

Total	INDEL	Ins	Del	HDR
120321	1.42%	**0.17%**	**1.25%**	**0.00%**

Typical seqs	Reads	Type	pct.
GGAATCTGCCTAACAGG|AGG**TGG**GGGT	118607	WT	98.58%
GGAATCTGCCTAAC---AGGTGGGGGT	498	-3	0.41%
GGAATCTGCCTAACA--AGGTGGGGGT	346	-2	0.29%
GGAATCTGCCTAA----AGGTGGGGGT	141	-4	0.12%
GGAATCTGCCTA-----AGGTGGGGGT	97	-5	0.08%
GGAATCTGCCTAACAG-AGGTGGGGGT	87	-1	0.07%
GGAATCTGCCTAACAGGgAGGTGGGGGT	87	+1 g	0.07%
GGAATCTGCCTAACAG--GGTGGGGGT	66	-2	0.05%
GGAATCTGCCTAACAGG-GGTGGGGGT	63	-1	0.05%
GGAATCTGCCTAACAGGtAGGTGG[illegible]	40	+1 t	0.03%

48h

Total	INDEL	Ins	Del	HDR
211849	59.84%	**3.47%**	**55.90%**	**0.47%**

Typical seqs	Reads	Type	pct.
GGAATCTGCCTAACAGG|AGG**TGG**GGGT	85078	WT	40.16%
GGAATCTGCCTAAC---AGGTGGGGGT	41692	-3	19.68%
GGAATCTGCCTAACA--AGGTGGGGGT	13011	-2	6.14%
GGAATCTGCCTAA----AGGTGGGGGT	6612	-4	3.12%
GGAATCTGCCTAACAG-AGGTGGGGGT	6064	-1	2.86%
GGAATCTGCCTAACAGG-GGTGGGGGT	4062	-1	1.92%
GGAATCTGCCTAACAG--GGTGGGGGT	3410	-2	1.61%
GGAATCTGCCTA-----AGGTGGGGGT	3372	-5	1.59%
GGAATCTGCCTAACAGGgAGGTGGGGGT	2369	+1 g	1.12%
GGAATCTGC--------AGGTGGGGGT	2072	-8	0.98%

4h

Total	INDEL	Ins	Del	HDR
119999	3.05%	**0.38%**	**2.67%**	**0.00%**

Typical seqs	Reads	Type	pct.
GGAATCTGCCTAACAGG|AGG**TGG**GGGT	116336	WT	96.95%
GGAATCTGCCTAACA--AGGTGGGGGT	577	-2	0.48%
GGAATCTGCCTAAC---AGGTGGGGGT	547	-3	0.46%
GGAATCTGCCTAACAG-AGGTGGGGGT	329	-1	0.27%
GGAATCTGCCTAACAGG-GGTGGGGGT	295	-1	0.25%
GGAATCTGCCTAA----AGGTGGGGGT	205	-4	0.17%
GGAATCTGCCTA-----AGGTGGGGGT	190	-5	0.16%
GGAATCTGCCTAACAGGgAGGTGGGGGT	164	+1 g	0.14%
GGAATCTGCCTAACAG--GGTGGGGGT	151	-2	0.13%
GGAATCTGCCTAACCAGGAGGTGGGGGT	104	+1 Ins	0.09%

9h

Total	INDEL	Ins	Del	HDR
230407	11.19%	**0.92%**	**10.26%**	**0.00%**

Typical seqs	Reads	Type	pct.
GGAATCTGCCTAACAGG|AGG**TGG**GGGT	204632	WT	88.81%
GGAATCTGCCTAAC---AGGTGGGGGT	5071	-3	2.20%
GGAATCTGCCTAACA--AGGTGGGGGT	3573	-2	1.55%
GGAATCTGCCTAACAG-AGGTGGGGGT	2548	-1	1.11%
GGAATCTGCCTAA----AGGTGGGGGT	2090	-4	0.91%
GGAATCTGCCTAACAGG-GGTGGGGGT	1721	-1	0.75%
GGAATCTGCCTA-----AGGTGGGGGT	1146	-5	0.50%
GGAATCTGCCTAACAG--GGTGGGGGT	1011	-2	0.44%
GGAATCTGCCTAACAGGgAGGTGGGGGT	817	+1 g	0.35%
GGAATCTGC--------AGGTGGGGGT	473	-8	0.21%

24h

Total	INDEL	Ins	Del	HDR
68564	34.04%	**2.15%**	**31.89%**	**0.00%**

Typical seqs	Reads	Type	pct.
GGAATCTGCCTAACAGG|AGG**TGG**GGGT	45222	WT	65.96%
GGAATCTGCCTAAC---AGGTGGGGGT	5410	-3	7.89%
GGAATCTGCCTAACA--AGGTGGGGGT	3209	-2	4.68%
GGAATCTGCCTAA----AGGTGGGGGT	1673	-4	2.44%
GGAATCTGCCTAACAG-AGGTGGGGGT	1484	-1	2.16%
GGAATCTGCCTAACAGG-GGTGGGGGT	1153	-1	1.68%
GGAATCTGCCTA-----AGGTGGGGGT	992	-5	1.45%
GGAATCTGCCTAACAG--GGTGGGGGT	740	-2	1.08%
GGAATCTGCCT------AGGTGGGGGT	704	-6	1.03%
GGAATCTGCCTAACAGGgAGGTGGGGGT	693	+1 g	1.01%

P161-P165-YW-K562-RNP-Syn24AAVS1-AAVKI

1h

Total | INDEL	Ins	Del	HDR
132577 | 0.33%	0.17%	0.16%	0.01%

Typical seqs	Reads	Type	pct.
GGAATCTGCCTAACAGG|AGG**TGG**GGGT	132138	WT	99.67%
GGAATCTGCCTAACAAGGAGGTGGGGGT	146	+1 Ins	0.11%
GGAATCTGCCTAACAG-AGGTGGGGGT	37	-1	0.03%
GGAATCTGCCTAAC---AGGTGGGGGT	36	-3	0.03%
GGAATCTGCCTAACAGGgAGGTGGGGGT	32	+1 g	0.02%
GGAATCTGCCT-ACAGGAGGTGGGGGT	29	-1	0.02%
GGAATCTGCCTAACCAGGAGGTGGGGGT	20	+1 Ins	0.02%
GGAATCTGCCTAACAGGaGGGTGGGGGT	15	+1 a	0.01%
GGAATCTGCCTAACAGGAGG-GGGGGT	12	-1	0.01%
GGAATCTGCCTA--AGGAGGTGGGGGT	9	-2	0.01%

2h

Total | INDEL	Ins	Del	HDR
197509 | 0.79%	0.20%	0.57%	0.02%

Typical seqs	Reads	Type	pct.
GGAATCTGCCTAACAGG|AGG**TGG**GGGT	195943	WT	99.21%
GGAATCTGCCTAACA--AGGTGGGGGT	233	-2	0.12%
GGAATCTGCCTAACAAGGAGGTGGGGGT	233	+1 Ins	0.12%
GGAATCTGCCTAAC---AGGTGGGGGT	217	-3	0.11%
GGAATCTGCCTAACAG-AGGTGGGGGT	200	-1	0.10%
GGAATCTGCCTAACAGG-GGTGGGGGT	72	-1	0.04%
GGAATCTGCCTAACAG--GGTGGGGGT	50	-2	0.03%
GGAATCTGCCT-ACAGGAGGTGGGGGT	47	-1	0.02%
GGAATCTGCCTAA----AGGTGGGGGT	47	-4	0.02%
GGAATCTGCCTAACCAGGAGGTGGGGGT	46	+1 Ins	0.02%

3h

Total | INDEL	Ins	Del	HDR
65599 | 1.34%	0.30%	0.97%	0.07%

Typical seqs	Reads	Type	pct.
GGAATCTGCCTAACAGG|AGG**TGG**GGGT	64718	WT	98.66%
GGAATCTGCCTAACA--AGGTGGGGGT	173	-2	0.26%
GGAATCTGCCTAAC---AGGTGGGGGT	145	-3	0.22%
GGAATCTGCCTAACAG-AGGTGGGGGT	72	-1	0.11%
GGAATCTGCCTAACAAGGAGGTGGGGGT	55	+1 Ins	0.08%
GGAATCTGCCTAAGTTTAAACTACGCGTGGGT	46	+5 HDR	0.07%
GGAATCTGCCTAACAG--GGTGGGGGT	44	-2	0.07%
GGAATCTGCCTAACAGGcAGGTGGGGGT	42	+1 c	0.06%
GGAATCTGCCTAACAGGgAGGTGGGGGT	40	+1 g	0.06%
GGAATCTGCCTAACAGG-GGTGGG[illegible]	38	-1	0.06%

48h

Total | INDEL	Ins	Del	HDR
227926 | 64.56%	5.58%	37.28%	21.69%

Typical seqs	Reads	Type	pct.
GGAATCTGCCTAACAGG|AGG**TGG**GGGT	80777	WT	35.44%
GGAATCTGCCTAAGTTTAAACTACGCGTGGGT	46652	+5 HDR	20.47%
GGAATCTGCCTAAC---AGGTGGGGGT	22502	-3	9.87%
GGAATCTGCCTAACA--AGGTGGGGGT	11831	-2	5.19%
GGAATCTGCCTAA----AGGTGGGGGT	5186	-4	2.28%
GGAATCTGCCTAACAG-AGGTGGGGGT	4590	-1	2.01%
GGAATCTGCCTA-----AGGTGGGGGT	4087	-5	1.79%
GGAATCTGCCTAACAGG-GGTGGGGGT	3328	-1	1.46%
GGAATCTGCCTAACAG--GGTGGGGGT	3007	-2	1.32%
GGAATCTGCCTAACAGGgAGGTGGGGGT	2538	+1 g	1.11%

4h

Total | INDEL	Ins	Del	HDR
146667 | 3.97%	1.03%	2.53%	0.41%

Typical seqs	Reads	Type	pct.
GGAATCTGCCTAACAGG|AGG**TG**[illegible]T	140846	WT	96.03%
GGAATCTGCCTAAC---AGGTGGGGGT	929	-3	0.63%
GGAATCTGCCTAACAAGGAGGTGGGGGT	631	+1 Ins	0.43%
GGAATCTGCCTAAGTTTAAACTACGCGTGG GT	580	+5 HDR	0.40%
GGAATCTGCCTAACA--AGGTGGGGGT	556	-2	0.38%
GGAATCTGCCTAACAG-AGGTGGGGGT	410	-1	0.28%
GGAATCTGCCTAACAGGgAGGTGGGGGT	277	+1 g	0.19%
GGAATCTGCCTA-----AGGTGGGGGT	244	-5	0.17%
GGAATCTGCCTAA----AGGTGGGGGT	226	-4	0.15%
GGAATCTGCCTAACAGG-GGTGGGGGT	237	-1	0.16%

9h

Total | INDEL	Ins	Del	HDR
250776 | 14.85%	1.98%	9.51%	3.36%

Typical seqs	Reads	Type	pct.
GGAATCTGCCTAACAGG|AGG**TGG**[illegible]	213528	WT	85.15%
GGAATCTGCCTAAGTTTAAACTACGCGTGG GT	7973	+5 HDR	3.18%
GGAATCTGCCTAAC---AGGTGGGGGT	4445	-3	1.77%
GGAATCTGCCTAACA--AGGTGGGGGT	4387	-2	1.75%
GGAATCTGCCTAACAG-AGGTGGGGGT	2514	-1	1.00%
GGAATCTGCCTAACAGG-GGTGGGGGT	1812	-1	0.72%
GGAATCTGCCTAA----AGGTGGGGGT	1759	-4	0.70%
GGAATCTGCCTAACAGGgAGGTGGGGGT	1293	+1 g	0.52%
GGAATCTGCCTAACAG--GGTGGGGGT	1240	-2	0.49%
GGAATCTGCCTAACAAGGAGGTGGGGGT	1227	+1 Ins	0.49%

24h

Total | INDEL	Ins	Del	HDR
59485 | 40.44%	3.54%	23.97%	12.92%

Typical seqs	Reads	Type	pct.
GGAATCTGCCTAACAGG|AGG**TGG**GGGT	35432	WT	59.56%
GGAATCTGCCTAAGTTTAAACTACGCGTGGGT	7212	+5 HDR	12.12%
GGAATCTGCCTAAC---AGGTGGGGGT	2989	-3	5.02%
GGAATCTGCCTAACA--AGGTGGGGGT	2208	-2	3.71%
GGAATCTGCCTAACAG-AGGTGGGGGT	1218	-1	2.05%
GGAATCTGCCTAA----AGGTGGGGGT	1035	-4	1.74%
GGAATCTGCCTAACAGG-GGTGGGGGT	834	-1	1.40%
GGAATCTGCCTAACAG--GGTGGGGGT	761	-2	1.28%
GGAATCTGCCTA-----AGGTGGGGGT	625	-5	1.05%
GGAATCTGCCT------AGGTGGGGGT	516	-6	0.87%

P161-P165-YW-K562-RNP-Syn25AAVS1-KO

1h

Total	INDEL	Ins	Del	HDR
190972	0.30%	0.12%	0.17%	0.00%

Typical seqs	Reads	Type	pct.
TAAGGAATCTGCCTAAC|AGGAGGTGGG	190407	WT	99.70%
TAAGGAATCTGCCTAACcAGGAGGTGGG	180	+1 c	0.09%
TAAGGAATCTGCCTA--AGGAGGTGGG	78	-2	0.04%
TAAGGAATCTGCCTA-CAGGAGGTGGG	66	-1	0.03%
TAAGGAATCTGCCTAACaAGGAGGTGGG	26	+1 a	0.01%
TAAGGAATCTGCCTAA-AGGAGGTGGG	30	-1	0.02%
TAAGGAATCTGCCTAACAGGA-GTGGG	22	-1	0.01%
TAAGGAATCTGCCTAACA-GAGGTGGG	19	-1	0.01%
TAAGGAATCTGCCTAACAGG-GGTGGG	15	-1	0.01%
TAAGGAATCTGCCTAAC---AGGTGGG	15	-3	0.01%

2h

Total	INDEL	Ins	Del	HDR
280205	1.78%	0.88%	0.89%	0.01%

Typical seqs	Reads	Type	pct.
TAAGGAATCTGCCTAAC|AGGAGGTGGG	275222	WT	98.22%
TAAGGAATCTGCCTAACcAGGAGGTGGG	2174	+1 c	0.78%
TAAGGAATCTGCCTA--AGGAGGTGGG	1063	-2	0.38%
TAAGGAATCTGCCTAA-AGGAGGTGGG	319	-1	0.11%
TAAGGAATCTGC-----AGGAGGTGGG	171	-5	0.06%
TAAGGAATCTGCCTA-CAGGAGGTGGG	166	-1	0.06%
TAAGGAATCTGCCTAAC--GAGGTGGG	131	-2	0.05%
TAAGGAATCTGCC----AGGAGGTGGG	111	-4	0.04%
TAAGGAATCTGCCTAAC-GGAGGTGGG	115	-1	0.04%
TAAGGAATCTGCCTAACaAGGAGGTGGG	92	+1 a	0.03%

3h

Total	INDEL	Ins	Del	HDR
175628	3.38%	1.50%	1.87%	0.01%

Typical seqs	Reads	Type	pct.
TAAGGAATCTGCCTAAC|AGGAGGTGGG	169696	WT	96.62%
TAAGGAATCTGCCTAACcAGGAGGTGGG	2377	+1 c	1.35%
TAAGGAATCTGCCTA--AGGAGGTGGG	1075	-2	0.61%
TAAGGAATCTGCCTAA-AGGAGGTGGG	453	-1	0.26%
TAAGGAATCTGCCTA-CAGGAGGTGGG	282	-1	0.16%
TAAGGAATCTGCCTAAC--GAGGTGGG	249	-2	0.14%
TAAGGAATCTGCC----AGGAGGTGGG	210	-4	0.12%
TAAGGAATCTGCCT---AGGAGGTGGG	156	-3	0.09%
TAAGGAATCTGCCTAAC-GGAGGTGGG	134	-1	0.08%
TAAGGAATCTGCCTAACaAGGAG[illegible]	86	+1 a	0.05%

48h

Total	INDEL	Ins	Del	HDR
176319	70.70%	17.13%	53.06%	0.50%

Typical seqs	Reads	Type	pct.
TAAGGAATCTGCCTAAC|AGGAGGTGGG	51669	WT	29.30%
TAAGGAATCTGCCTAACcAGGAGGTGGG	23244	+1 c	13.18%
TAAGGAATCTGCCTA--AGGAGGTGGG	15733	-2	8.92%
TA---------------AGGAGGTGGG	10362	-15	5.88%
TAAGGAATCTGCCTAAC--GAGGTGGG	6585	-2	3.73%
TAAGGAATCTGCCTAA-AGGAGGTGGG	6485	-1	3.68%
TAAGGAATCTGCCTA-CAGGAGGTGGG	6181	-1	3.51%
TAAGGAATCTGCCTAAC-GGAGGTGGG	4127	-1	2.34%
TAAGGAATCTGC-----AGGAGGTGGG	3078	-5	1.75%
TAAGGAATCTGCC----AGGAGGTGGG	2914	-4	1.65%

4h

Total	INDEL	Ins	Del	HDR
155684	6.68%	2.72%	3.96%	0.00%

Typical seqs	Reads	Type	pct.
TAAGGAATCTGCCTAAC|AGGAGGTGGG	145285	WT	93.32%
TAAGGAATCTGCCTAACcAGGAGGTGGG	3561	+1 c	2.29%
TAAGGAATCTGCCTA--AGGAGGTGGG	1812	-2	1.16%
TAAGGAATCTGCCTA-CAGGAGGTGGG	632	-1	0.41%
TAAGGAATCTGCCTAAC--GAGGTGGG	454	-2	0.29%
TAAGGAATCTGCCTAA-AGGAGGTGGG	461	-1	0.30%
TAAGGAATCTGCCTAAC-GGAGGTGGG	364	-1	0.23%
TAAGGAATCTGC-----AGGAGGTGGG	329	-5	0.21%
TAAGGAATCTGCCT---AGGAGGTGGG	324	-3	0.21%
TAAGGAATCTGCC----AGGAGGTGGG	273	-4	0.18%

9h

Total	INDEL	Ins	Del	HDR
193697	20.67%	6.50%	14.16%	0.00%

Typical seqs	Reads	Type	pct.
TAAGGAATCTGCCTAAC|AGGAGGTGGG	153656	WT	79.33%
TAAGGAATCTGCCTAACcAGGAGGTGGG	10323	+1 c	5.33%
TAAGGAATCTGCCTA--AGGAGGTGGG	7427	-2	3.83%
TAAGGAATCTGCCTAA-AGGAGGTGGG	2989	-1	1.54%
TAAGGAATCTGCCTAAC--GAGGTGGG	2397	-2	1.24%
TAAGGAATCTGCCTA-CAGGAGGTGGG	2270	-1	1.17%
TAAGGAATCTGCCTAAC-GGAGGTGGG	1619	-1	0.84%
TAAGGAATCTGC-----AGGAGGTGGG	1527	-5	0.79%
TAAGGAATCTGCCT---AGGAGGTGGG	1096	-3	0.57%
TAAGGAATCTGCC----AGGAGGTGGG	645	-4	0.33%

24h

Total	INDEL	Ins	Del	HDR
93271	49.95%	13.22%	36.72%	0.01%

Typical seqs	Reads	Type	pct.
TAAGGAATCTGCCTAAC|AGGAGGTGGG	46680	WT	50.05%
TAAGGAATCTGCCTAACcAGGAGGTGGG	10238	+1 c	10.98%
TAAGGAATCTGCCTA--AGGAGGTGGG	7924	-2	8.50%
TAAGGAATCTGCCTAAC--GAGGTGGG	3766	-2	4.04%
TAAGGAATCTGCCTAA-AGGAGGTGGG	2975	-1	3.19%
TAAGGAATCTGCCTA-CAGGAGGTGGG	2544	-1	2.73%
TAAGGAATCTGCCTAAC-GGAGGTGGG	1669	-1	1.79%
TA---------------AGGAGGTGGG	1427	-15	1.53%
TAAGGAATCTGC-----AGGAGGTGGG	1337	-5	1.43%
TAAGGAATCTGCC----AGGAGGTGGG	1284	-4	1.38%

P161-P165-YW-K562-RNP-Syn25AAVS1-AAVKI

1h

Total | INDEL	Ins	Del	HDR
73675 | 0.46%	0.24%	0.20%	0.02%

Typical seqs	Reads	Type	pct.
TAAGGAATCTGCCTAAC|AGG**AGG**TGGG	73337	WT	99.54%
TAAGGAATCTGCCTAACcAGGAGGTGGG	87	+1 c	0.12%
TAAGGAATCTGCCTAACaAGGAGGTGGG	81	+1 a	0.11%
TAAGGAATCTGCCTAAC--GAGGTGGG	31	-2	0.04%
TAAGGAATCTGCCTA--AGGAGGTGGG	26	-2	0.04%
TAAGGAATCTGCCTA-CAGGAGGTGGG	19	-1	0.03%
TAAGGAATCTGCCTAAGTTTAAACTACGCG TG	16	+5 HDR	0.02%
TAAGGAATCTGCCTAA-AGGAGGTGGG	13	-1	0.02%
TAAGGAATCTGCCTAACAGG-GGTGGG	11	-1	0.01%
TAAGGAATCTG-CTAACAGGAGGTGGG	7	-1	0.01%

2h

Total | INDEL	Ins	Del	HDR
122193 | 1.47%	0.87%	0.58%	0.02%

Typical seqs	Reads	Type	pct.
TAAGGAATCTGCCTAAC|AGG**AGG**TGGG	120400	WT	98.53%
TAAGGAATCTGCCTAACcAGGAGGTGGG	814	+1 c	0.67%
TAAGGAATCTGCCTAACaAGGAGGTGGG	178	+1 a	0.15%
TAAGGAATCTGCCTA--AGGAGGTGGG	165	-2	0.14%
TAAGGAATCTGCCTAA-AGGAGGTGGG	136	-1	0.11%
TAAGGAATCTGCCTA-CAGGAGGTGGG	67	-1	0.05%
TAAGGAATCTGCC----AGGAGGTGGG	62	-4	0.05%
TAAGGAATCTGCCTAAC--GAGGTGGG	60	-2	0.05%
TAAGGAATCTGCCTAAC-GGAGGTGGG	57	-1	0.05%
TAAGGAATCTGC-----AGGAGGTGGG	54	-5	0.04%

3h

Total | INDEL	Ins	Del	HDR
88799 | 3.21%	1.73%	1.41%	0.07%

Typical seqs	Reads	Type	pct.
TAAGGAATCTGCCTAAC|AGG**AGG**TGGG	85947	WT	96.79%
TAAGGAATCTGCCTAACcAGGAGGTGGG	1221	+1 c	1.38%
TAAGGAATCTGCCTA--AGGAGGTGGG	269	-2	0.30%
TAAGGAATCTGCCTA-CAGGAGGTGGG	208	-1	0.23%
TAAGGAATCTGCCTAA-AGGAGGTGGG	181	-1	0.20%
TAAGGAATCTGCCTAAC--GAGGTGGG	138	-2	0.16%
TAAGGAATCTGCCTAACaAGGAGGTGGG	111	+1 a	0.13%
TAAGGAATCTGCCT---AGGAGGTGGG	108	-3	0.12%
TAAGGAATCTGCCTAAC-GGAGGTGGG	76	-1	0.09%
TAAGGAATCTGCCTAAGTTTAAACTAC[illegible]	62	+5 HDR	0.07%

48h

Total | INDEL	Ins	Del	HDR
159104 | 76.23%	16.03%	36.00%	24.19%

Typical seqs	Reads	Type	pct.
TAAGGAATCTGCCTAAC|AGG**AGG**TGGG	37824	WT	23.77%
TAAGGAATCTGCCTAAGTTTAAACTACGCGTG	36195	+5 HDR	22.75%
TAAGGAATCTGCCTAACcAGGAGGTGGG	18165	+1 c	11.42%
TAAGGAATCTGCCTA--AGGAGGTGGG	11311	-2	7.11%
TAAGGAATCTGCCTAA-AGGAGGTGGG	5310	-1	3.34%
TAAGGAATCTGCCTAAC--GAGGTGGG	5013	-2	3.15%
TAAGGAATCTGCCTAAC-GGAGGTGGG	3701	-1	2.33%
TAAGGAATCTGCCTA-CAGGAGGTGGG	3063	-1	1.93%
TA---------------AGGAGGTGGG	2805	-15	1.76%
TAAGGAATCTGC-----AGGAGGTGGG	2367	-5	1.49%

4h

Total | INDEL	Ins	Del	HDR
106002 | 8.03%	3.52%	3.82%	0.69%

Typical seqs	Reads	Type	pct.
TAAGGAATCTGCCTAAC|AGG**A**[illegible]G	97494	WT	91.97%
TAAGGAATCTGCCTAACcAGGAGGTGGG	2693	+1 c	2.54%
TAAGGAATCTGCCTA--AGGAGGTGGG	1119	-2	1.06%
TAAGGAATCTGCCTAAGTTTAAACTACGCG TG	715	+5 HDR	0.67%
TAAGGAATCTGCCTAA-AGGAGGTGGG	604	-1	0.57%
TAAGGAATCTGCCTAACaAGGAGGTGGG	501	+1 a	0.47%
TAAGGAATCTGCCTAAC-GGAGGTGGG	381	-1	0.36%
TAAGGAATCTGCCTA-CAGGAGGTGGG	323	-1	0.30%
TAAGGAATCTGCCTAAC--GAGGTGGG	277	-2	0.26%
TAAGGAATCTGC-----AGGAGGTGGG	266	-5	0.25%

9h

Total | INDEL	Ins	Del	HDR
134469 | 26.75%	7.93%	13.72%	5.09%

Typical seqs	Reads	Type	pct.
TAAGGAATCTGCCTAAC|AGG**AGG**TGGG	98495	WT	73.25%
TAAGGAATCTGCCTAACcAGGAGGTGGG	8168	+1 c	6.07%
TAAGGAATCTGCCTAAGTTTAAACTACGCG TG	6542	+5 HDR	4.87%
TAAGGAATCTGCCTA--AGGAGGTGGG	5011	-2	3.73%
TAAGGAATCTGCCTAA-AGGAGGTGGG	1961	-1	1.46%
TAAGGAATCTGCCTAAC--GAGGTGGG	1667	-2	1.24%
TAAGGAATCTGCCTA-CAGGAGGTGGG	1457	-1	1.08%
TAAGGAATCTGCCTAAC-GGAGGTGGG	1232	-1	0.92%
TAAGGAATCTGC-----AGGAGGTGGG	1065	-5	0.79%
TAAGGAATCTGCCTAACaAGGAGGTGGG	900	+1 a	0.67%

24h

Total | INDEL	Ins	Del	HDR
91028 | 58.75%	13.34%	30.03%	15.38%

Typical seqs	Reads	Type	pct.
TAAGGAATCTGCCTAAC|AGG**AGG**TGGG	37550	WT	41.25%
TAAGGAATCTGCCTAAGTTTAAACTACGCGTG	13207	+5 HDR	14.51%
TAAGGAATCTGCCTAACcAGGAGGTGGG	9092	+1 c	9.99%
TAAGGAATCTGCCTA--AGGAGGTGGG	6930	-2	7.61%
TAAGGAATCTGCCTAA-AGGAGGTGGG	2583	-1	2.84%
TAAGGAATCTGCCTAAC-GGAGGTGGG	2068	-1	2.27%
TAAGGAATCTGCCTAAC--GAGGTGGG	1929	-2	2.12%
TAAGGAATCTGCCTA-CAGGAGGTGGG	1908	-1	2.10%
TAAGGAATCTGC-----AGGAGGTGGG	1163	-5	1.28%
TAAGGAATCTGCC----AGGAGGTGGG	1087	-4	1.19%

P212-YW-K562-Syn32crBCL11Gata-a1-48h-RNPko-BC1

P208-YW-K562-Syn32crBCL11Gata-a1-4h-RNPko-BC1 4h	Ins	Del	HDR
7385 | 0.26%	**0.26%**	**0.00%**	**0.00%**
**Typical seqs**	**Reads**	**Type**	**pct.**
CACGCCCCCACCCTAAT|CAG**AGG**CCAA	7366	WT	99.74%
CACGCCCCCACCCTAATtCAGAGGCCAA	7	+1 t	0.09%
CACGCCCCCCCCCTAATTCAGAGGCCAA	8	+1 Ins	0.11%
CACGCCCCCCCCCAATTCAGAGGCCAA	4	+1 Ins	0.05%

P209-YW-K562-Syn32crBCL11Gata-a1-8h-RNPko-BC1 8h	Ins	Del	HDR
4135 | 25.13%	**0.36%**	**24.76%**	**0.00%**
**Typical seqs**	**Reads**	**Type**	**pct.**
CACGCCCCCACCCTAAT|CAGAGGCCAA	3096	WT	74.87%
CACGCCCC---------CAGAGGCCAA	109	-9	2.64%
CACG-----------------------	260	-24	6.29%
CACG-----------------------	260	-29	6.29%
CA---------------CAGAGGCCAA	90	-15	2.18%
CACGCCC---------TCAGAGGCCAA	77	-9	1.86%
-----------------CAGAGGCCAA	68	-17	1.64%
CACG------------TCAGAGGCCAA	64	-12	1.55%
CACGC------------CAGAGGCCAA	58	-12	1.40%
CACG-----------------------	260	-28	6.29%

P210-YW-K562-Syn32crBCL11Gata-a1-12h-RNPko-BC1 12h	Ins	Del	HDR
6535 | 35.00%	**0.95%**	**34.05%**	**0.00%**
**Typical seqs**	**Reads**	**Type**	**pct.**
CACGCCCCCACCCTAAT|CAG**AGG**CCAA	4248	WT	65.00%
CACGCC---------------------	369	-27	5.65%
CACGCC---------------------	369	-22	5.65%
CACG-----------------------	251	-29	3.84%
CACGCCC----------CAGAGGCCAA	134	-10	2.05%
CACGCCCCCA-------CAGAGGCCAA	129	-7	1.97%
CACGC----------------------	232	-22	3.55%
CAC----------------GAGGCCAA	114	-16	1.74%
CACGC------------CAGAGGCCAA	111	-12	1.70%
CACGC----------------------	232	-28	3.55%

P211-YW-K562-Syn32crBCL11Gata-a1-24h-RNPko-BC1 24h	Ins	Del	HDR
20420 | 58.72%	**1.09%**	**57.63%**	**0.00%**
**Typical seqs**	**Reads**	**Type**	**pct.**
CACGCCCCCACCCTAAT|CAG**AGG**CCAA	8429	WT	41.28%
CACG-----------------------	3258	-29	15.95%
CACG-----------------------	3258	-25	15.95%
CACGCC---------------------	1106	-27	5.42%
-----------------------CCAA	527	-24	2.58%
CACG-----------------------	3258	-28	15.95%
CACG-------------CAGAGGCCAA	372	-13	1.82%
CACGC----------------------	816	-28	4.00%
CACGC----------------------	816	-23	4.00%
CACGCCC--------------------	489	-26	2.39%

P212-YW-K562-Syn32crBCL11Gata-a1-48h-RNPko-BC1 48h	Ins	Del	HDR
25303 | 69.07%	**2.18%**	**66.89%**	**0.00%**
**Typical seqs**	**Reads**	**Type**	**pct.**
CACGCCCCCACCCTAAT|CAG**AGG**CCAA	7826	WT	30.93%
CACGCC---------------------	1871	-27	7.39%
CACG-----------------------	3053	-29	12.07%
CACG----------------------A	737	-22	2.91%
CACG-----------------------	3053	-24	12.07%
CACGCCC--------------------	753	-26	2.98%
CACGC----------------------	1407	-28	5.56%
CAC------------------GCCAA	589	-19	2.33%
CACGCC---------------------	1871	-22	7.39%
CACG-----------------------	3053	-28	12.07%

P212-YW-K562-Syn34crBCL11Gata-a3-48h-RNPko-BC3

P208-YW-K562-Syn34crBCL11Gata-a3-4h-RNPko-BC3 **4h**	Ins	Del	HDR
4816 | 0.39%	**0.27%**	**0.12%**	**0.00%**
**Typical seqs**	**Reads**	**Type**	**pct.**
TTTATCACAGGCTCCAG|GAA**GGG**TTTG	4797	WT	99.61%
TTTATCACAGGCTCCAGgGAAGGGTTTG	13	+1 g	0.27%
TTTATCA-------CAGGAAGGGTTTG	3	-7	0.06%
TTTATCACAGGCTCCA-GAAGGGTTTG	3	-1	0.06%

P209-YW-K562-Syn34crBCL11Gata-a3-8h-RNPko-BC3 **8h**	Ins	Del	HDR
5015 | 0.92%	**0.50%**	**0.42%**	**0.00%**
**Typical seqs**	**Reads**	**Type**	**pct.**
TTTATCACAGGCTCCAG|GAA**GGG**TTTG	4969	WT	99.08%
TTTATCACAGGCTCCAGgGAAGGGTTTG	20	+1 g	0.40%
TTTATCACAGGCT--------------	9	-15	0.18%
TTTATCACA-----------------G	9	-17	0.18%
TTTATCACAGGCTCCAGgGAAGGGTTGG	5	+1 g	0.10%
TTT----------------AGGGTTTG	3	-16	0.06%

P210-YW-K562-Syn34crBCL11Gata-a3-12h-RNPko-BC3 **12h**	Ins	Del	HDR
7674 | 2.57%	**0.64%**	**1.93%**	**0.00%**
**Typical seqs**	**Reads**	**Type**	**pct.**
TTTATCACAGGCTCCAG|GAA**GGG**TTTG	7477	WT	97.43%
TTTATCACAGGCTCCAGgGAAGGGTTTG	38	+1 g	0.50%
TTTATCACAGGCTCCA-GAAGGGTTTG	34	-1	0.44%
TTTATCACAGGCT--------------	31	-15	0.40%
TTTATCAC-----------AGGGTTTG	15	-11	0.20%
TTTATCACAGGCTCC----AGGGTTTG	9	-4	0.12%
TTTATCACA-----------------G	12	-17	0.16%
TTTATCACAGGCTC-------------	10	-14	0.13%
TTTATCACAGGCTCCA-----------	7	-11	0.09%
TTTATCACAGGCTCCAGgGAAGGGTTGG	11	+1 g	0.14%

P211-YW-K562-Syn34crBCL11Gata-a3-24h-RNPko-BC3 **24h**	Ins	Del	HDR
15904 | 8.44%	**1.25%**	**7.19%**	**0.00%**
**Typical seqs**	**Reads**	**Type**	**pct.**
TTTATCACAGGCTCCAG|GAA**GGG**TTTG	14562	WT	91.56%
TTTATCACAGGCT--------------	156	-15	0.98%
TTTATCAC-----------AGGGTTTG	137	-11	0.86%
TTTATCACAGGCTCCAGgGAAGGGTTTG	103	+1 g	0.65%
TTTATCACAGGCTCCA-----------	48	-11	0.30%
TTTATCACAGGCTCC----AGGGTTTG	98	-4	0.62%
TTTATCACAGGCTCCA-GAAGGGTTTG	85	-1	0.53%
TTTATCACA-----------------G	70	-17	0.44%
TTTATCACAGG----------------	58	-18	0.36%
TTTATCACAGGCTC-------------	89	-17	0.56%

P212-YW-K562-Syn34crBCL11Gata-a3-48h-RNPko-BC3 **48h**	Ins	Del	HDR
34737 | 12.06%	**2.27%**	**9.80%**	**0.00%**
**Typical seqs**	**Reads**	**Type**	**pct.**
TTTATCACAGGCTCCAG|GAA**GGG**TTTG	30546	WT	87.94%
TTTATCACAGGCTCCAGgGAAGGGTTTG	544	+1 g	1.57%
TTTATCACAGGCT--------------	404	-15	1.16%
TTTATCAC-----------AGGGTTTG	408	-11	1.17%
TTTATCACAGGCTCCA-GAAGGGTTTG	255	-1	0.73%
TTTATCACAGGCTCC----AGGGTTTG	234	-4	0.67%
TTTATCACA-----------------G	267	-17	0.77%
TTTATCACAGGCTC-------------	214	-14	0.62%
TTTATCACAGGCTCC------------	119	-16	0.34%
TTTATCACAGG----------------	133	-18	0.38%

P212-YW-K562-Syn35crBCL11Gata-a4-48h-RNPko-BC4

P208-YW-K562-Syn35crBCL11Gata-a4-4h-RNPko-BC4 4h	Ins	Del	HDR
3881 | 2.50%	2.24%	0.26%	0.00%
**Typical seqs**	**Reads**	**Type**	**pct.**
TTTTATCACAGGCTCCA|GGA**AGG**GTTT	3784	WT	97.50%
TTTTATCACAGGCTCCAaGGAAGGGTTT	63	+1 a	1.62%
TTTTATCACAGGCTCCAaGGAAGGGTTG	20	+1 a	0.52%
TTTTATCACA------AGGAAGGGTTT	4	-6	0.10%
TTTTATCACAGGCTCCAaGGAAGGTTTG	4	+1 a	0.10%
TTTTATCA-------CAGGAAGGGTTT	3	-7	0.08%
TTTTATCACAGGCTC--GGAAGGGTTT	3	-2	0.08%

P209-YW-K562-Syn35crBCL11Gata-a4-8h-RNPko-BC4 8h	Ins	Del	HDR
6579 | 5.88%	4.26%	1.63%	0.00%
**Typical seqs**	**Reads**	**Type**	**pct.**
TTTTATCACAGGCTCCA|GGA**AGG**GTTT	6192	WT	94.12%
TTTTATCACAGGCTCCAaGGAAGGGTTT	187	+1 a	2.84%
TTTTATCACAGGCTCCAaGGAAGGGTTG	60	+1 a	0.91%
TTTTATCA-------CAGGAAGGGTTT	16	-7	0.24%
TTTTATCACAGGCTCCA----------	15	-13	0.23%
TTTTATCACAGGCTCCAaGGAAGGTTTT	13	+1 a	0.20%
TTTTATCACAGGCTCC-GGAAGGGTTT	13	-1	0.20%
TTTTATCACAGGCTCCACAGGAAGGGTTT	11	+2	0.17%
TTTTATCACAGGCTCCAaGGAAGGTTTG	6	+1 a	0.09%
TTTTATCACAGGCTCCA----------	15	-11	0.23%

P210-YW-K562-Syn35crBCL11Gata-a4-12h-RNPko-BC4 12h	Ins	Del	HDR
7938 | 14.55%	8.00%	6.55%	0.00%
**Typical seqs**	**Reads**	**Type**	**pct.**
TTTTATCACAGGCTCCA|GGA**AGG**GTTT	6783	WT	85.45%
TTTTATCACAGGCTCCAaGGAAGGGTTT	411	+1 a	5.18%
TTTTATCACA-----------------	109	-17	1.37%
TTTTATCACAGGCTCCAaGGAAGGGTTG	146	+1 a	1.84%
TTTTATCA-------CAGGAAGGGTTT	121	-7	1.52%
TTTTATCACAGGCTCC------GGTTT	54	-6	0.68%
TTTTATCACAGGCTC--GGAAGGGTTT	23	-2	0.29%
TTTTATCACA-----------GGTTT	20	-12	0.25%
TTTTATCACAGGCTCCAaGGAAGGTTTT	28	+1 a	0.35%
TTTTATCACAGGCT-------------	19	-15	0.24%

P211-YW-K562-Syn35crBCL11Gata-a4-24h-RNPko-BC4 24h	Ins	Del	HDR
18658 | 35.87%	14.29%	21.58%	0.00%
**Typical seqs**	**Reads**	**Type**	**pct.**
TTTTATCACAGGCTCCA|GGA**AGG**GTTT	11966	WT	64.13%
TTTTATCACAGGCTCCAaGGAAGGGTTT	1555	+1 a	8.33%
TTTTATCA-------CAGGAAGGGTTT	1304	-7	6.99%
TTTTATCACAGGCTCCAaGGAAGGGTTG	534	+1 a	2.86%
TTTTATCAC-----------AGGGTTT	476	-11	2.55%
TTTTATCACA-----------------	272	-17	1.46%
TTTTATCACAGG---------------	220	-18	1.18%
TTTTATCACAGGCT-------------	228	-15	1.22%
TTTTATCACAGGCTCCACAGGAAGGGTTT	171	+2	0.92%
TTTTATCACAGGCT-------------	228	-15	1.22%

P212-YW-K562-Syn35crBCL11Gata-a4-48h-RNPko-BC4 48h	Ins	Del	HDR
31508 | 53.27%	19.25%	34.02%	0.00%
**Typical seqs**	**Reads**	**Type**	**pct.**
TTTTATCACAGGCTCCA|GGA**AGG**GTTT	14725	WT	46.73%
TTTTATCACAGGCTCCAaGGAAGGGTTT	4762	+1 a	15.11%
TTTTATCA-------CAGGAAGGGTTT	3140	-7	9.97%
TTTTATCAC-----------AGGGTTT	1440	-11	4.57%
TTTTATCACAGGCT-------------	708	-15	2.25%
TTTTATCACA-----------------	574	-17	1.82%
TTTTATCACAGGCTCCAaGGAAGGGTTG	561	+1 a	1.78%
TTTTATCACAGG---------------	326	-18	1.03%
TTTTATCACAGGCTCC-GGAAGGGTTT	294	-1	0.93%
TTTTATCACA-----------GGTTT	288	-12	0.91%

P212-YW-K562-Syn37crBCL11Gata-a6-48h-RNPko-BC6

P208-YW-K562-Syn37crBCL11Gata-a6-4h-RNPko-BC6 4h	Ins	Del	HDR
5475 | 0.60%	**0.55%**	**0.05%**	**0.00%**
**Typical seqs**	**Reads**	**Type**	**pct.**
CTAACAGTTGCTTTTAT|CAC**AGG**CTCC	5442	WT	99.40%
CTAACAGTTGCTTTTATtCACAGGCTCC	30	+1 t	0.55%
CTAACAGTTG------TCACAGGCTCC	3	-6	0.05%

P209-YW-K562-Syn37crBCL11Gata-a6-8h-RNPko-BC6 8h	Ins	Del	HDR
12084 | 2.05%	**1.01%**	**1.04%**	**0.00%**
**Typical seqs**	**Reads**	**Type**	**pct.**
CTAACAGTTGCTTTTAT|CAC**AGG**CTCC	11836	WT	97.95%
CTAACAGTTGCTTTTATtCACAGGCTCC	122	+1 t	1.01%
CTA---------------ACAGGCTCC	20	-15	0.17%
CTAACAGTTGCTTTTAT-----GCTCC	16	-5	0.13%
CTAACAGTT-------------GCTCC	15	-13	0.12%
CTAACAGTTGC-----TAACAGGCTCC	10	-5	0.08%
CTAA-------------CACAGGCTCC	9	-13	0.07%
-----------------CACAGGCTCC	8	-17	0.07%
CTAACAGT--------TCACAGGCTCC	6	-8	0.05%
CTA------------ATCACAGGCTCC	6	-12	0.05%

P210-YW-K562-Syn37crBCL11Gata-a6-12h-RNPko-BC6 12h	Ins	Del	HDR
17233 | 4.56%	**1.61%**	**2.94%**	**0.00%**
**Typical seqs**	**Reads**	**Type**	**pct.**
CTAACAGTTGCTTTTAT|CAC**AGG**CTCC	16448	WT	95.44%
CTAACAGTTGCTTTTATtCACAGGCTCC	260	+1 t	1.51%
CTA---------------ACAGGCTCC	133	-15	0.77%
CTAACAGTT-------------GCTCC	91	-13	0.53%
CTAA-------------CACAGGCTCC	32	-13	0.19%
CTAACA----------------GCTCC	24	-16	0.14%
CTAACAGTTG-------CACAGGCTCC	20	-7	0.12%
CTAACAGTTGCTTT--TCACAGGCTCC	20	-2	0.12%
-------------------CAGGCTCC	14	-23	0.08%
CTA--------------CACAGGCTCC	10	-14	0.06%

P211-YW-K562-Syn37crBCL11Gata-a6-24h-RNPko-BC6 24h	Ins	Del	HDR
20755 | 15.46%	**3.28%**	**12.19%**	**0.00%**
**Typical seqs**	**Reads**	**Type**	**pct.**
CTAACAGTTGCTTTTAT|CAC**AGG**CTCC	17546	WT	84.54%
CTA---------------ACAGGCTCC	860	-15	4.14%
CTAACAGTTGCTTTTATtCACAGGCTCC	622	+1 t	3.00%
CTAACAGTT-------------GCTCC	340	-13	1.64%
CTAA-------------CACAGGCTCC	132	-13	0.64%
CTAACAGTTG-------CACAGGCTCC	90	-7	0.43%
-----------------CACAGGCTCC	46	-17	0.22%
CTAACAGTTGCTTTTAT--CAGGCTCC	69	-2	0.33%
CTAACAGT--------TCACAGGCTCC	42	-8	0.20%
----------------------GCTCC	37	-23	0.18%

P212-YW-K562-Syn37crBCL11Gata-a6-48h-RNPko-BC6 48h	Ins	Del	HDR
44311 | 17.17%	**4.48%**	**12.69%**	**0.00%**
**Typical seqs**	**Reads**	**Type**	**pct.**
CTAACAGTTGCTTTTAT|CAC**AGG**CTCC	36703	WT	82.83%
CTAACAGTTGCTTTTATtCACAGGCTCC	1806	+1 t	4.08%
CTA---------------ACAGGCTCC	1323	-15	2.99%
CTAACAGTT-------------GCTCC	832	-13	1.88%
CTAA-------------CACAGGCTCC	369	-13	0.83%
----------------------GCTCC	158	-23	0.36%
CTAACAGTTG-------CACAGGCTCC	166	-7	0.37%
CTAACA----------------GCTCC	109	-16	0.25%
CTAACAGTTGCTTTTAT-ACAGGCTCC	121	-1	0.27%
CTAACAGTTGCTTTT------------	163	-12	0.37%

P212-YW-K562-Syn26crCD326ac-RNPko-48h-BC1

P208-YW-K562-Syn26crCD326ac-RNPko-4h-BC1 4h	Ins	Del	HDR
3159 | 3.86%	**2.56%**	**1.30%**	**0.00%**
**Typical seqs**	**Reads**	**Type**	**pct.**
CCCTCCCGCGCCCCTCT|TCT**CGG**CGCG	3037	WT	96.14%
CCCTCCCGCGCCCCTCTtTCTCGGCGCG	73	+1 t	2.31%
CCCTCCCGCGCCCCTC-TCTCGGCGCG	19	-1	0.60%
CCCTCCCGCGCCCCTCTCTTCTCGGCGCG	8	+2	0.25%
CCCTCCCGCGCCCC---TCTCGGCGCG	8	-3	0.25%
CCCTCCCGCGCCCCT-TTCTCGGCGCG	6	-1	0.19%
CCCTCC-------------------CG	5	-19	0.16%
CCCTCCCGCGC----CTTCTCGGCGCG	3	-4	0.09%

P208-YW-K562-Syn26crCD326ac-RNPko-4h-BC1	Ins	Del	HDR
3159 | 3.86%	**2.56%**	**1.30%**	**0.00%**

P209-YW-K562-Syn26crCD326ac-RNPko-8h-BC1 8h	Ins	Del	HDR
8249 | 9.95%	**4.33%**	**5.62%**	**0.00%**
**Typical seqs**	**Reads**	**Type**	**pct.**
CCCTCCCGCGCCCCTCT|TCT**CGG**CGCG	7428	WT	90.05%
CCCTCCCGCGCCCCTCTtTCTCGGCGCG	338	+1 t	4.10%
CCCTCCCGCGCCCC---TCTCGGCGCG	77	-3	0.93%
CCCTCCCGCGCCCCT-TTCTCGGCGCG	69	-1	0.84%
CCCTCCCGCGCCCCTC-TCTCGGCGCG	63	-1	0.76%
CCCTCC---------------------	72	-23	0.87%
CCCTCC-------------------CG	45	-19	0.55%
CCCTCC---------------------	72	-21	0.87%
CCCTCCCGC------------------	22	-24	0.27%
CCCTCCCGCGCCC--CTTCTCGGCGCG	22	-2	0.27%

P210-YW-K562-Syn26crCD326ac-RNPko-12h-BC1 12h	Ins	Del	HDR
10043 | 16.00%	**5.68%**	**10.33%**	**0.00%**
**Typical seqs**	**Reads**	**Type**	**pct.**
CCCTCCCGCGCCCCTCT|TCT**CGG**CGCG	8436	WT	84.00%
CCCTCCCGCGCCCCTCTtTCTCGGCGCG	517	+1 t	5.15%
CCCTCCCGCGCCCCTC-TCTCGGCGCG	227	-1	2.26%
CCCTCCCGCGCCCC---TCTCGGCGCG	149	-3	1.48%
CCCTCC---------------------	156	-21	1.55%
---------------------------	72	-30	0.72%
CCCTC------------TCTCGGCGCG	44	-12	0.44%
CCCTCCCGCGCCCCTCT----------	84	-12	0.84%
--------------------------G	33	-28	0.33%
CCCTCCCGCGCCC--CTTCTCGGCGCG	26	-2	0.26%

P211-YW-K562-Syn26crCD326ac-RNPko-24h-BC1 24h	Ins	Del	HDR
13781 | 43.70%	**11.54%**	**32.13%**	**0.02%**
**Typical seqs**	**Reads**	**Type**	**pct.**
CCCTCCCGCGCCCCTCT|TCT**CGG**CGCG	7759	WT	56.30%
CCCTCCCGCGCCCCTCTtTCTCGGCGCG	1465	+1 t	10.63%
CCCTCCCGCGCCCCTC-TCTCGGCGCG	1093	-1	7.93%
CCCTCC---------------------	936	-21	6.79%
CCCTCCCGCGCCCC---TCTCGGCGCG	374	-3	2.71%
------------------------GCG	159	-26	1.15%
CCCTCC---------------------	936	-23	6.79%
CCCTC----------------------	232	-26	1.68%
CCCTCCCGCGC------TCTCGGCGCG	95	-6	0.69%
CCCTCCCGCGCCC-----CTCGGCGCG	94	-5	0.68%

P212-YW-K562-Syn26crCD326ac-RNPko-48h-BC1 48h	Ins	Del	HDR
13743 | 55.69%	**14.88%**	**40.76%**	**0.04%**
**Typical seqs**	**Reads**	**Type**	**pct.**
CCCTCCCGCGCCCCTCT|TCT**CGG**CGCG	6090	WT	44.31%
CCCTCCCGCGCCCCTCTtTCTCGGCGCG	1857	+1 t	13.51%
CCCTCC---------------------	1446	-21	10.52%
CCCTCCCGCGCCCCTC-TCTCGGCGCG	670	-1	4.88%
CCCTCCCGCGCCCC---TCTCGGCGCG	621	-3	4.52%
CCCTCC---------------------	1446	-23	10.52%
CCCTCCCGC------------------	177	-24	1.29%
CCC--------------TCTCGGCGCG	121	-14	0.88%
CCCTCCCGCGCCC-----CTCGGCGCG	111	-5	0.81%
CCCTCC-----------------CGCG	131	-17	0.95%

P212-YW-K562-Syn26crCD326ac-RNPaav-48h-BC7

P208-YW-K562-Syn26crCD326ac-RNPaav-4h-BC7 4h	Ins	Del	HDR
31413 | 6.68%	2.06%	1.53%	3.09%
**Typical seqs**	**Reads**	**Type**	**pct.**
CCCTCCCGCGCCCCTCT|TCTCGGCGCG	29314	WT	93.32%
CCCTCCCGCACGCGTAGTTTAAACCTCGGCGCG	944	+6 HDR	3.01%
CCCTCCCGCGCCCCTCTtTCTCGGCGCG	580	+1 t	1.85%
CCCTCCCGCGCCCCTC-TCTCGGCGCG	389	-1	1.24%
CCCTCCCGCGCCCCTCTATTCTCGGCGCG	52	+2	0.17%
CCCTCCCGCGCCCCTCT----------	49	-14	0.16%
CCCTCCCGCGCCCCT-TTCTCGGCGCG	38	-1	0.12%
CCCTCCCGCGCCCCTCT----------	49	-11	0.16%
CCCTCCCGCACGCTTAGTTTAAACCTCGGCGCG	6	+6	0.02%
CCCTCCCGCACGCGTCGTTTAAACCTCGGCGCG	3	+6	0.01%

P209-YW-K562-Syn26crCD326ac-RNPaav-8h-BC7 8h	Ins	Del	HDR
29105 | 25.22%	5.03%	3.59%	16.61%
**Typical seqs**	**Reads**	**Type**	**pct.**
CCCTCCCGCGCCCCTCT|TCTCGGCGCG	21764	WT	74.78%
CCCTCCCGCACGCGTAGTTTAAACCTCGGCGCG	4704	+6 HDR	16.16%
CCCTCCCGCGCCCCTCTtTCTCGGCGCG	1267	+1 t	4.35%
CCCTCCCGCGCCCCTC-TCTCGGCGCG	353	-1	1.21%
CCCTCCCGCGCCCC---TCTCGGCGCG	105	-3	0.36%
CC---------------TCTCGGCGCG	70	-15	0.24%
CCCTC----------CTTCTCGGCGCG	66	-10	0.23%
CCCTCC---------------------	65	-21	0.22%
CCCTCCCGCGCCC----TCTCGGCGCG	53	-4	0.18%
CCCTCCCGCGC------TCTCGGCGCG	52	-6	0.18%

P210-YW-K562-Syn26crCD326ac-RNPaav-12h-BC7 12h	Ins	Del	HDR
23758 | 46.44%	6.17%	6.92%	33.35%
**Typical seqs**	**Reads**	**Type**	**pct.**
CCCTCCCGCGCCCCTCT|TCTCGGCGCG	12725	WT	53.56%
CCCTCCCGCACGCGTAGTTTAAACCTCGGCGCG	7710	+6 HDR	32.45%
CCCTCCCGCGCCCCTCTtTCTCGGCGCG	1197	+1 t	5.04%
CCCTCCCGCGCCCCTC-TCTCGGCGCG	309	-1	1.30%
CCCTCCCGCGCCCC---TCTCGGCGCG	275	-3	1.16%
CCCTCC---------------------	216	-21	0.91%
--------------------------G	93	-28	0.39%
CCCTCC---------------------	216	-23	0.91%
C----------------TCTCGGCGCG	77	-16	0.32%
CCCTCCCGCGCCCC---TCTCG-----	75	-9	0.32%

P211-YW-K562-Syn26crCD326ac-RNPaav-24h-BC7 24h	Ins	Del	HDR
19114 | 76.11%	6.58%	10.41%	59.11%
**Typical seqs**	**Reads**	**Type**	**pct.**
CCCTCCCGCGCCCCTCT|TCTCGGCGCG	4567	WT	23.89%
CCCTCCCGCACGCGTAGTTTAAACCTCGGCGCG	10906	+6 HDR	57.06%
CCCTCCCGCGCCCCTCTtTCTCGGCGCG	830	+1 t	4.34%
CCCTCC---------------------	614	-21	3.21%
CCCTCCCGCGCCCCTC-TCTCGGCGCG	321	-1	1.68%
CCCTCCCGCGCCCC---TCTCGGCGCG	177	-3	0.93%
CCCTCC---------------------	614	-23	3.21%
CCCTCCCGCGCCC-----CTCGGCGCG	97	-5	0.51%
CCC--------------TCTCGGCGCG	60	-14	0.31%
CCCTCCCGCGCC---------------	100	-17	0.52%

P212-YW-K562-Syn26crCD326ac-RNPaav-48h-BC7 48h	Ins	Del	HDR
19455 | 86.36%	8.01%	13.97%	64.38%
**Typical seqs**	**Reads**	**Type**	**pct.**
CCCTCCCGCGCCCCTCT|TCTCGGCGCG	2654	WT	13.64%
CCCTCCCGCACGCGTAGTTTAAACCTCGGCGCG	12071	+6 HDR	62.05%
CCCTCCCGCGCCCCTCTtTCTCGGCGCG	990	+1 t	5.09%
CCCTCC---------------------	613	-21	3.15%
CCCTCCCGCGCCCCTC-TCTCGGCGCG	263	-1	1.35%
CCCTCCCGCGCCCC---TCTCGGCGCG	159	-3	0.82%
CCCTCC---------------------	613	-23	3.15%
CCCTCCCGC------------------	128	-24	0.66%
CCC--------------TCTCGGCGCG	125	-14	0.64%
CCCTCCC--------------------	91	-26	0.47%

P212-YW-K562-Syn28crCD326ae-RNPko-48h-BC3

P208-YW-K562-Syn28crCD326ae-RNPko-4h-BC3 **4h**	Ins	Del	HDR
10675 | 1.13%	**0.26%**	**0.87%**	**0.00%**
**Typical seqs**	**Reads**	**Type**	**pct.**
GCGCGCCGAGAAGAGGG|GCG**CGG**GAGG	10554	WT	98.87%
GCGCGCCGAGAAGA-GGGCGCGGGAGG	47	-1	0.44%
GCGCGCCGAGAAGA--GGCGCGGGAGG	29	-2	0.27%
GCGCGCCGAGAAGAGGGgGCGCGGGAGG	28	+1 g	0.26%
-----------------GCGCGGGAGG	11	-21	0.10%
GCGCGCCGAGAA-----------GAGG	6	-11	0.06%
P208-YW-K562-Syn28crCD326ae-RNPko-4h-BC3	Ins	Del	HDR
10675 | 1.13%	**0.26%**	**0.87%**	**0.00%**
**Typical seqs**	**Reads**	**Type**	**pct.**
GCGCGCCGAGAAGAGGG|GCG**CGG**GAGG	10554	WT	98.87%

P209-YW-K562-Syn28crCD326ae-RNPko-8h-BC3 **8h**	Ins	Del	HDR
14931 | 3.19%	**0.47%**	**2.73%**	**0.00%**
**Typical seqs**	**Reads**	**Type**	**pct.**
GCGCGCCGAGAAGAGGG|GCG**CGG**GAGG	14454	WT	96.81%
GCGCGCCGAGAAGA-GGGCGCGGGAGG	199	-1	1.33%
GCGCGCCGAGAAGA--GGCGCGGGAGG	120	-2	0.80%
GCGCGCCGAGAAGAGGGgGCGCGGGAGG	70	+1 g	0.47%
GCGCGCCGAGAAGAGGG--------AG	42	-8	0.28%
GCGCGCCGA-------GGCGCGGGAGG	22	-7	0.15%
GCGCGCCGAGAAGAGG---GCGGGAGG	13	-3	0.09%
GCGCGCCGAGA------GCGCGGGAGG	8	-6	0.05%
GCGCGCCGAGAGGA-GGGCGCGGGAGG	3	-1	0.02%
P209-YW-K562-Syn28crCD326ae-RNPko-8h-BC3	Ins	Del	HDR

P210-YW-K562-Syn28crCD326ae-RNPko-12h-BC3 **12h**	Ins	Del	HDR
28104 | 4.41%	**0.79%**	**3.62%**	**0.00%**
**Typical seqs**	**Reads**	**Type**	**pct.**
GCGCGCCGAGAAGAGGG|GCG**CGG**GAGG	26866	WT	95.59%
GCGCGCCGAGAAGA-GGGCGCGGGAGG	519	-1	1.85%
GCGCGCCGAGAAGA--GGCGCGGGAGG	147	-2	0.52%
GCGCGCCGAGAA----GGCGCGGGAGG	120	-4	0.43%
GCGCGCCGAGAAGAGGGgGCGCGGGAGG	116	+1 g	0.41%
-----------------GCGCGGGAGG	95	-21	0.34%
GCGCGCCGAGA-----GGCGCGGGAGG	86	-5	0.31%
GCGCGCCGAGAAGAGGGAGGGGCGCGCGGG AGG	46	+6	0.16%
GCGCGCCGAGAAGAGGGgCCGCGGGAGG	34	+1 g	0.12%
GCGCGCCGAGAAGA-------GGGAGG	19	-7	0.07%

P211-YW-K562-Syn28crCD326ae-RNPko-24h-BC3 **24h**	Ins	Del	HDR
33007 | 8.11%	**0.85%**	**7.23%**	**0.03%**
**Typical seqs**	**Reads**	**Type**	**pct.**
GCGCGCCGAGAAGAGGG|GCG**CGG**GAGG	30331	WT	91.89%
GCGCGCCGAGAAGA-GGGCGCGGGAGG	941	-1	2.85%
GCGCGCCGAGAAGA--GGCGCGGGAGG	262	-2	0.79%
GCGCGCCGAGAAGAGGGgGCGCGGGAGG	202	+1 g	0.61%
-----------------GCGCGGGAGG	220	-21	0.67%
GCGCGCCGAGAAGAGGGGCCCGGGA--	159	-4	0.48%
GCGCGCCGAGAAGA-------------	139	-18	0.42%
GCGCGCCGAGAAGAGGG----------	137	-13	0.42%
GCGCGCCGAGAAGA---GCGCGGGAGG	116	-3	0.35%
GCGCGCCGAGAAGAGGGaGCGCGGGAGG	80	+1 a	0.24%

P212-YW-K562-Syn28crCD326ae-RNPko-48h-BC3 **48h**	Ins	Del	HDR
40268 | 10.29%	**0.96%**	**9.32%**	**0.01%**
**Typical seqs**	**Reads**	**Type**	**pct.**
GCGCGCCGAGAAGAGGG|GCG**CGG**GAGG	36126	WT	89.71%
GCGCGCCGAGAAGA-GGGCGCGGGAGG	727	-1	1.81%
-----------------GCGCGGGAGG	830	-21	2.06%
GCGCGCCGAGAAGA--GGCGCGGGAGG	349	-2	0.87%
GCGCGCCGAGAAGAGGGgGCGCGGGAGG	288	+1 g	0.72%
GCGCGCCGAGAA-----------GAGG	273	-11	0.68%
GCGCGCCGAGAAGA-------------	332	-13	0.82%
GCGCGCCGAGAAGA-------------	332	-18	0.82%
-----------------GCGCGGGAGG	830	-19	2.06%
GCGCGCCGAGAAGA-------GGGAGG	167	-7	0.41%

P212-YW-K562-Syn28crCD326ae-RNPaav-48h-BC9

P208-YW-K562-Syn28crCD326ae-RNPaav-4h-BC9 **4h**	Ins	Del	HDR
60688 | 1.09%	**0.26%**	**0.72%**	**0.11%**
**Typical seqs**	**Reads**	**Type**	**pct.**
GCGCGCCGAGAAGAGGG|GCG**CGG**GAGG	60026	WT	98.91%
GCGCGCCGAGAAGA-GGGCGCGGGAGG	322	-1	0.53%
GCGCGCCGAGAAGAGGGgGCGCGGGAGG	156	+1 g	0.26%
GCGCGCCGAGA------GCGCGGGAGG	66	-6	0.11%
GCGCGCCGAGGTTTAAACTACGCGTGCGGG AGG	66	+6 HDR	0.11%
GCGCGCCGAGA-----GGCGCGGGAGG	49	-5	0.08%

P209-YW-K562-Syn28crCD326ae-RNPaav-8h-BC9 **8h**	Ins	Del	HDR
49712 | 3.16%	**0.75%**	**1.75%**	**0.67%**
**Typical seqs**	**Reads**	**Type**	**pct.**
GCGCGCCGAGAAGAGGG|GCG**CGG**GAGG	48140	WT	96.84%
GCGCGCCGAGAAGA-GGGCGCGGGAGG	585	-1	1.18%
GCGCGCCGAGGTTTAAACTACGCGTGCGGG AGG	327	+6 HDR	0.66%
GCGCGCCGAGAAGAGGGgGCGCGGGAGG	245	+1 g	0.49%
GCGCGCCGAGAAGA--GGCGCGGGAGG	127	-2	0.26%
GCGCGCCGAGA------GCGCGGGAGG	75	-6	0.15%
GCGCGCCGAGAAGAGGTGGCGCGGGAGG	66	+1 Ins	0.13%
GCGCGCCGAGA-------------AGG	58	-13	0.12%
GCGCGCCGAGAAGAGGGtGCGCGGGAGG	54	+1 t	0.11%
GCGCGCCGA-------GGCGCGGGAGG	16	-7	0.03%

P210-YW-K562-Syn28crCD326ae-RNPaav-12h-BC9 **12h**	Ins	Del	HDR
40843 | 6.11%	**0.70%**	**4.26%**	**1.16%**
**Typical seqs**	**Reads**	**Type**	**pct.**
GCGCGCCGAGAAGAGGG|GCG**CGG**GAGG	38348	WT	93.89%
GCGCGCCGAGAAGA-GGGCGCGGGAGG	929	-1	2.27%
GCGCGCCGAGGTTTAAACTACGCGTGCGGG AGG	461	+6 HDR	1.13%
GCGCGCCGAGAAGAGGGgGCGCGGGAGG	277	+1 g	0.68%
-----------------GCGCGGGAGG	283	-21	0.69%
GCGCGCCGAGAAGA--GGCGCGGGAGG	135	-2	0.33%
GCGCGCCGAGA------GCGCGGGAGG	90	-6	0.22%
GCGCGCCGAGAA-----------GAGG	71	-11	0.17%
GCGCGCCGAGAAGA---GCGCGGGAGG	68	-3	0.17%
GCGCGCCGAGAAGA------------G	48	-12	0.12%

P211-YW-K562-Syn28crCD326ae-RNPaav-24h-BC9 **24h**	Ins	Del	HDR
38309 | 11.45%	**1.13%**	**6.65%**	**3.67%**
**Typical seqs**	**Reads**	**Type**	**pct.**
GCGCGCCGAGAAGAGGG|GCG**CGG**GAGG	33924	WT	88.55%
GCGCGCCGAGGTTTAAACTACGCGTGCGGG AGG	1375	+6 HDR	3.59%
GCGCGCCGAGAAGA-GGGCGCGGGAGG	951	-1	2.48%
GCGCGCCGAGAAGA--GGCGCGGGAGG	461	-2	1.20%
GCGCGCCGAGAAGAGGGgGCGCGGGAGG	345	+1 g	0.90%
GCGCGCCGAGAA-----------GAGG	173	-11	0.45%
-----------------GCGCGGGAGG	225	-21	0.59%
GCGCGCCGAGAAGA-------GGGAGG	158	-7	0.41%
GCGCGCCGAGAAGA-------------	87	-18	0.23%
GCGCGCCGAGAA-----GCGCGGGAGG	73	-5	0.19%

P212-YW-K562-Syn28crCD326ae-RNPaav-48h-BC9 **48h**	Ins	Del	HDR
45661 | 10.28%	**0.91%**	**6.43%**	**2.94%**
**Typical seqs**	**Reads**	**Type**	**pct.**
GCGCGCCGAGAAGAGGG|GCG**CGG**GAGG	40967	WT	89.72%
GCGCGCCGAGGTTTAAACTACGCGTGCGGG AGG	1320	+6 HDR	2.89%
GCGCGCCGAGAAGA-GGGCGCGGGAGG	1064	-1	2.33%
GCGCGCCGAGAAGA--GGCGCGGGAGG	363	-2	0.79%
GCGCGCCGAGAAGAGGGgGCGCGGGAGG	354	+1 g	0.78%
GCGCGCCGAGAAGA-------------	157	-13	0.34%
-----------------GCGCGGGAGG	271	-21	0.59%
GCGCGCCGAGAAGA-----------G	116	-12	0.25%
GCGCGCCGAGAAGAG----------GG	126	-10	0.28%
-----------------GCGCGGGAGG	271	-19	0.59%

P212-YW-K562-Syn29crCD326af-RNPko-48h-BC4

P208-YW-K562-Syn29crCD326af-RNPko-4h-BC4 **4h**	Ins	Del	HDR
6136 | 5.87%	**4.97%**	**0.90%**	**0.00%**
**Typical seqs**	**Reads**	**Type**	**pct.**
TGCGCGCGCGCCGAGAA|GAG**GGG**CGCG	5776	WT	94.13%
TGCGCGCGCGCCGAGAAaGAGGGGCGCG	301	+1 a	4.91%
TGCGCGCGCGCCGAGAA-AGGGGCGCG	19	-1	0.31%
TGCGCGCGCGCCGAG-AGAGGGGCGCG	11	-1	0.18%
TGCGCGCGCGCC-----GAGGGGCGCG	10	-5	0.16%
TGCGC------------GAGGGGCGCG	5	-12	0.08%
TGCGCGCGCGCCGAGAA----------	5	-15	0.08%
TGCGCGCGCGCCGAGA--AGGGGCGCG	5	-2	0.08%
TGAGCGCGCGCCGAGAAAGAGGGGCGCG	4	+1 Ins	0.07%
P208-YW-K562-Syn29crCD326af-RNPko-4h-BC4	Ins	Del	HDR

P209-YW-K562-Syn29crCD326af-RNPko-8h-BC4 **8h**	Ins	Del	HDR
14961 | 26.98%	**19.27%**	**7.71%**	**0.00%**
**Typical seqs**	**Reads**	**Type**	**pct.**
TGCGCGCGCGCCGAGAA|GAG**GGG**CGCG	10924	WT	73.02%
TGCGCGCGCGCCGAGAAaGAGGGGCGCG	2772	+1 a	18.53%
TGCGCGCGCGCCGAG-AGAGGGGCGCG	226	-1	1.51%
T---------------------GCGCG	181	-21	1.21%
TGCGCGCGCGCCG---AGAGGGGCGCG	105	-3	0.70%
TGCGCGCGCGCCGAGA----GGGCGCG	78	-4	0.52%
TGCGCGCGCGCCGAGAA-AGGGGCGCG	65	-1	0.43%
TGCGCGCGCGCCGAGA--AGGGGCGCG	64	-2	0.43%
TGCGCGCGCGCCGAGAAGA-GGGCGCG	57	-1	0.38%
TGCGCGCGCGCCGAGAA---GGGCGCG	56	-3	0.37%

P210-YW-K562-Syn29crCD326af-RNPko-12h-BC4 **12h**	Ins	Del	HDR
15728 | 38.78%	**25.41%**	**13.35%**	**0.03%**
**Typical seqs**	**Reads**	**Type**	**pct.**
TGCGCGCGCGCCGAGAA|GAG**GGG**CGCG	9628	WT	61.22%
TGCGCGCGCGCCGAGAAaGAGGGGCGCG	3799	+1 a	24.15%
TGCGCGCGCGCCGAG-AGAGGGGCGCG	244	-1	1.55%
T---------------------GCGCG	241	-21	1.53%
TGCGCGCGCGCCG---AGAGGGGCGCG	155	-3	0.99%
T-----------------------GCG	104	-23	0.66%
TGCGCGCGCGCC---------------	97	-16	0.62%
T--------------------------	93	-32	0.59%
TGCGCG----------AGAGGGGCGCG	87	-10	0.55%
TGC-------------------GCGCG	80	-19	0.51%

P211-YW-K562-Syn29crCD326af-RNPko-24h-BC4 **24h**	Ins	Del	HDR
13331 | 82.12%	**47.87%**	**34.20%**	**0.05%**
**Typical seqs**	**Reads**	**Type**	**pct.**
TGCGCGCGCGCCGAGAA|GAG**GGG**CGCG	2383	WT	17.88%
TGCGCGCGCGCCGAGAAaGAGGGGCGCG	5872	+1 a	44.05%
T---------------------GCGCG	1582	-21	11.87%
T-----------------------GCG	262	-23	1.97%
T---------------AGAGGGGCGCG	175	-15	1.31%
TGC--------------GAGGGGCGCG	173	-14	1.30%
T--------------------------	155	-29	1.16%
---------------------------	154	-32	1.16%
TGCGCGCGCGCCGAGAA----------	167	-16	1.25%
TGCGCATGC------------TGCGCG	113	-12	0.85%

P212-YW-K562-Syn29crCD326af-RNPko-48h-BC4 **48h**	Ins	Del	HDR
13931 | 92.02%	**46.36%**	**45.63%**	**0.04%**
**Typical seqs**	**Reads**	**Type**	**pct.**
TGCGCGCGCGCCGAGAA|GAG**GGG**CGCG	1111	WT	7.98%
TGCGCGCGCGCCGAGAAaGAGGGGCGCG	5968	+1 a	42.84%
T---------------------GCGCG	2327	-21	16.70%
T-----------------------GCG	619	-23	4.44%
T--------------------------	721	-32	5.18%
TGCGCGCGCGCCGAG-AGAGGGGCGCG	170	-1	1.22%
T--------------------------	721	-26	5.18%
TGC------------------------	250	-30	1.79%
TGC--------------GAGGGGCGCG	106	-14	0.76%
T--------------------------	721	-31	5.18%

P212-YW-K562-Syn29crCD326af-RNPaav-48h-BC10

P208-YW-K562-Syn29crCD326af-RNPaav-4h-BC10 4h<br>54221 | 22.94%	Ins<br>13.61%	Del<br>1.37%	HDR<br>7.96%
**Typical seqs**	**Reads**	**Type**	**pct.**
TGCGCGCGCGCCGAGAA|GAGGGGCGCG	41782	WT	77.06%
TGCGCGCGCGCCGAGAAaGAGGGGCGCG	6965	+1 a	12.85%
TGCGCGCGCGCCGAGGTTTAAACTACGCGT GCG	4155	+6 HDR	7.66%
TGCG------------AGAGGGGCGCG	153	-12	0.28%
TGCGCGCGCGCCGAGAA-AGGGGCGCG	119	-1	0.22%
TGCGCGCGC-------AGAGGGGCGCG	82	-7	0.15%
T---------------AGAGGGGCGCG	76	-15	0.14%
----------------AGAGGGGCGCG	51	-16	0.09%
TGCGCGCGCGCCGAGAA-------GCG	48	-7	0.09%
TGCGCGCGCGCCGAGAAAAGAGGGGCGCG	47	+2	0.09%

P209-YW-K562-Syn29crCD326af-RNPaav-8h-BC10 8h<br>46892 | 51.54%	Ins<br>17.78%	Del<br>4.51%	HDR<br>29.25%
**Typical seqs**	**Reads**	**Type**	**pct.**
TGCGCGCGCGCCGAGAA|GAGGGGCGCG	22725	WT	48.46%
TGCGCGCGCGCCGAGGTTTAAACTACGCGT GCG	13219	+6 HDR	28.19%
TGCGCGCGCGCCGAGAAaGAGGGGCGCG	7349	+1 a	15.67%
TGCGCGCGCGCCGAG-AGAGGGGCGCG	323	-1	0.69%
TGCGCGCGCGCCGAGAA-AGGGGCGCG	262	-1	0.56%
TGCG---------------GGGGCGCG	160	-15	0.34%
TGCGCGCGCGCCGAGAAGA-GGGCGCG	160	-1	0.34%
----------------AGAGGGGCGCG	129	-16	0.28%
TGCGCG----------AGAGGGGCGCG	127	-10	0.27%
TGCGCGCGCGCCG---AGAGGGGCGCG	98	-3	0.21%

P210-YW-K562-Syn29crCD326af-RNPaav-12h-BC10 12h<br>38507 | 70.50%	Ins<br>19.21%	Del<br>6.80%	HDR<br>44.49%
**Typical seqs**	**Reads**	**Type**	**pct.**
TGCGCGCGCGCCGAGAA|GAGGGGCGCG	11360	WT	29.50%
TGCGCGCGCGCCGAGGTTTAAACTACGCGT GCG	16405	+6 HDR	42.60%
TGCGCGCGCGCCGAGAAaGAGGGGCGCG	6393	+1 a	16.60%
TGCGCGCGCGCCGAG-AGAGGGGCGCG	418	-1	1.09%
T---------------------GCGCG	274	-21	0.71%
TGC-------------------GCGCG	177	-19	0.46%
T-----------------------GCG	145	-23	0.38%
TGCGCGCGCGCCGAGAA----------	154	-15	0.40%
TGCGC-----------AGAGGGGCGCG	138	-11	0.36%
TGCGCGCGCGCCG---AGAGGGGCGCG	120	-3	0.31%

P211-YW-K562-Syn29crCD326af-RNPaav-24h-BC10 24h<br>35598 | 93.88%	Ins<br>21.12%	Del<br>6.39%	HDR<br>66.36%
**Typical seqs**	**Reads**	**Type**	**pct.**
TGCGCGCGCGCCGAGAA|GAGGGGCGCG	2179	WT	6.12%
TGCGCGCGCGCCGAGGTTTAAACTACGCGT GCG	22595	+6 HDR	63.47%
TGCGCGCGCGCCGAGAAaGAGGGGCGCG	6025	+1 a	16.93%
TGCGCGCGCGCCGAGGTTTAAACTACGCGC GCG	329	+6	0.92%
T---------------------GCGCG	355	-21	1.00%
TGCGCGCGCGCCGAG-AGAGGGGCGCG	226	-1	0.63%
T----------------GAGGGGCGCG	164	-16	0.46%
T---------------AGAGGGGCGCG	144	-15	0.40%
TGCGCGCGCGCCGAGAA-AGGGGCGCG	132	-1	0.37%
TGCGCGCGCGCCG---AGAGGGGCGCG	101	-3	0.28%

P212-YW-K562-Syn29crCD326af-RNPaav-48h-BC10 48h<br>36487 | 91.62%	Ins<br>19.00%	Del<br>10.03%	HDR<br>62.59%
**Typical seqs**	**Reads**	**Type**	**pct.**
TGCGCGCGCGCCGAGAA|GAGGGGCGCG	3057	WT	8.38%
TGCGCGCGCGCCGAGGTTTAAACTACGCGT GCG	21931	+6 HDR	60.11%
TGCGCGCGCGCCGAGAAaGAGGGGCGCG	5577	+1 a	15.28%
T---------------------GCGCG	478	-21	1.31%
TGCGCGCGCGCCGAG-AGAGGGGCGCG	398	-1	1.09%
TGCGCGCGCGCCG---AGAGGGGCGCG	332	-3	0.91%
TGCGCGCGCGCCGAGAA-AGGGGCGCG	163	-1	0.45%
TGCGCGCGCGCCGAGGTTTAAACTACGCGC GCG	161	+6	0.44%
T--------------------------	194	-32	0.53%
TGCGCGCGCGCCGA-------------	154	-14	0.42%

P212-YW-K562-Syn30crCD326ag-RNPko-48h-BC5

P208-YW-K562-Syn30crCD326ag-RNPko-4h-BC5 4h<br>12292 | 0.84%	Ins<br>0.46%	Del<br>0.37%	HDR<br>0.00%
**Typical seqs**	**Reads**	**Type**	**pct.**
CTGCGCGCGCGCCGAGA|AGA**GGG**GCGC	12189	WT	99.16%
CTGCGCGCGCGCCGAGAaAGAGGGGCGC	57	+1 a	0.46%
CTGCGCGCGCGCCGAG-AGAGGGGCGC	30	-1	0.24%
CTGCGCGCGCGCCGAGA--AGGGGCGC	7	-2	0.06%
CTGCGCGCGCGCCG---AGAGGGGCGC	6	-3	0.05%
CTGCGCGCGCGCCGAGA----GGGCGC	3	-4	0.02%

P209-YW-K562-Syn30crCD326ag-RNPko-8h-BC5 8h<br>12821 | 2.07%	Ins<br>0.60%	Del<br>1.47%	HDR<br>0.00%
**Typical seqs**	**Reads**	**Type**	**pct.**
CTGCGCGCGCGCCGAGA|AGA**GGG**GCGC	12555	WT	97.93%
CTGCGCGCGCGCCGAG-AGAGGGGCGC	74	-1	0.58%
CTGCGCGCGCGCCGAGAaAGAGGGGCGC	72	+1 a	0.56%
CTGCGCGCGCGCCG---AGAGGGGCGC	52	-3	0.41%
CTGCGCG----------AGAGGGGCGC	13	-10	0.10%
CTGC-------------------GCGC	13	-19	0.10%
CTGCGCGCGCGCC----AGAGGGGCGC	12	-4	0.09%
-----------------------GCGCG	10	-23	0.08%
C----------------AGAGGGGCGC	7	-16	0.05%
CTGCGCGCGCGCCGA------------	5	-14	0.04%

P210-YW-K562-Syn30crCD326ag-RNPko-12h-BC5 12h<br>22744 | 7.33%	Ins<br>1.76%	Del<br>5.58%	HDR<br>0.00%
**Typical seqs**	**Reads**	**Type**	**pct.**
CTGCGCGCGCGCCGAGA|AGA**GGG**GCGC	21076	WT	92.67%
CTGCGCGCGCGCCGAG-AGAGGGGCGC	373	-1	1.64%
CTGCGCGCGCGCCGAGAaAGAGGGGCGC	360	+1 a	1.58%
CTGCGCGCGCGCCG---AGAGGGGCGC	137	-3	0.60%
CT-----------------------GC	87	-23	0.38%
C-------------------CGGGCGC	64	-19	0.28%
CT-------------------------	109	-27	0.48%
CT---------------------GCGC	54	-21	0.24%
CT-------------------------	109	-29	0.48%
CTGCG------------AGAGGGGCGC	41	-12	0.18%

P211-YW-K562-Syn30crCD326ag-RNPko-24h-BC5 24h<br>16939 | 25.10%	Ins<br>2.86%	Del<br>22.20%	HDR<br>0.04%
**Typical seqs**	**Reads**	**Type**	**pct.**
CTGCGCGCGCGCCGAGA|AGA**GGG**GCGC	12688	WT	74.90%
CT---------------------GCGC	1037	-21	6.12%
CTGCGCGCGCGCCGAG-AGAGGGGCGC	559	-1	3.30%
CT-----------------------GC	523	-23	3.09%
CTGCGCGCGCGCCGAGAaAGAGGGGCGC	428	+1 a	2.53%
-----------------------GCGC	258	-24	1.52%
-------------------------GC	213	-26	1.26%
CTGCGCGCATGC----------TGCGC	137	-10	0.81%
CT---------------AGAGGGGCGC	103	-15	0.61%
CTGCGCGCGCGCCG---AGAGGGGCGC	85	-3	0.50%

P212-YW-K562-Syn30crCD326ag-RNPko-48h-BC5 48h<br>14564 | 46.49%	Ins<br>5.53%	Del<br>40.96%	HDR<br>0.00%
**Typical seqs**	**Reads**	**Type**	**pct.**
CTGCGCGCGCGCCGAGA|AGA**GGG**GCGC	7793	WT	53.51%
CT---------------------GCGC	1330	-21	9.13%
CT-----------------------GC	1289	-23	8.85%
CTGCGCGCGCGCCGAGAaAGAGGGGCGC	725	+1 a	4.98%
-----------------------GCGC	463	-24	3.18%
CTGCGCGCGCGCCGAG-AGAGGGGCGC	339	-1	2.33%
-------------------------GC	332	-26	2.28%
CTGC-------------------GCGC	181	-19	1.24%
CTGC-----------------------	266	-28	1.83%
CTG--------------AGAGGGGCGC	121	-14	0.83%

P212-YW-K562-Syn30crCD326ag-RNPaav-48h-BC11

P208-YW-K562-Syn30crCD326ag-RNPaav-4h-BC11 **4h**	Ins	Del	HDR
66085 | 3.84%	**0.22%**	**1.04%**	**2.58%**
**Typical seqs**	**Reads**	**Type**	**pct.**
CTGCGCGCGCGCCGAGA|AGA**GGG**GCGC	63550	WT	96.16%
CTGCGCGCGCGCCGAGGTTTAAACTACGCG TGC	1547	+6 HDR	2.34%
CT----------------------GC	209	-23	0.32%
CTGCGC----------AAGAGGGGCGC	209	-10	0.32%
CTGCGCGCGCGCCGAG-AGAGGGGCGC	174	-1	0.26%
CTGCGCGCGCGCCGAGAaAGAGGGGCGC	113	+1 a	0.17%
CTGCGCGC---------AGAGGGGCGC	35	-9	0.05%
CTGCGCGCGCGCCG---AGAGGGGCGC	34	-3	0.05%
CTGCGCGCGCGCCGAGA-----GGCGC	27	-5	0.04%
CTGCGCGCGCGCCGAGGTTTAAACTACGAG TGC	11	+6	0.02%

P209-YW-K562-Syn30crCD326ag-RNPaav-8h-BC11 **8h**	Ins	Del	HDR
48044 | 13.03%	**1.69%**	**1.51%**	**9.83%**
**Typical seqs**	**Reads**	**Type**	**pct.**
CTGCGCGCGCGCCGAGA|AGA**GGG**GCGC	41782	WT	86.97%
CTGCGCGCGCGCCGAGGTTTAAACTACGCG TGC	4557	+6 HDR	9.49%
CTGCGCGCGCGCCGAGAaAGAGGGGCGC	524	+1 a	1.09%
CTGCGCGCGCGCCGAG-AGAGGGGCGC	234	-1	0.49%
CTGCGCGCGCGCCGAGAtAGAGGGGCGC	96	+1 t	0.20%
CTGCGCGCGCGCCGAGA----------	133	-15	0.28%
CT---------------------GCGC	57	-21	0.12%
CT----------------------GC	52	-23	0.11%
CTGCGCGCGCGCCGAGAcAGAGGGGCGC	50	+1 c	0.10%
CTGCGCGCGCGCCGAGA----------	133	-17	0.28%

P210-YW-K562-Syn30crCD326ag-RNPaav-12h-BC11 **12h**	Ins	Del	HDR
45010 | 20.84%	**2.31%**	**4.73%**	**13.80%**
**Typical seqs**	**Reads**	**Type**	**pct.**
CTGCGCGCGCGCCGAGA|AGA**GGG**GCGC	35629	WT	79.16%
CTGCGCGCGCGCCGAGGTTTAAACTACGCG TGC	5973	+6 HDR	13.27%
CTGCGCGCGCGCCGAG-AGAGGGGCGC	672	-1	1.49%
CTGCGCGCGCGCCGAGAaAGAGGGGCGC	633	+1 a	1.41%
CT---------------------GCGC	325	-21	0.72%
CTGCGCGCGCGCCG---AGAGGGGCGC	199	-3	0.44%
CT----------------------GC	131	-23	0.29%
CTGCGCGCGCGCCGA------------	97	-14	0.22%
CTGC-------------------GCGC	92	-19	0.20%
CTGCG------------AGAGGGGCGC	79	-12	0.18%

P211-YW-K562-Syn30crCD326ag-RNPaav-24h-BC11 **24h**	Ins	Del	HDR
36610 | 53.93%	**3.44%**	**7.07%**	**43.42%**
**Typical seqs**	**Reads**	**Type**	**pct.**
CTGCGCGCGCGCCGAGA|AGA**GGG**GCGC	16868	WT	46.07%
CTGCGCGCGCGCCGAGGTTTAAACTACGCG TGC	15220	+6 HDR	41.57%
CTGCGCGCGCGCCGAG-AGAGGGGCGC	654	-1	1.79%
CTGCGCGCGCGCCGAGAaAGAGGGGCGC	475	+1 a	1.30%
CT---------------------GCGC	409	-21	1.12%
CT----------------------GC	275	-23	0.75%
CTGCGCGCGCGCCG---AGAGGGGCGC	175	-3	0.48%
CTGCGCGCGCGCCGAGAtAGAGGGGCGC	151	+1 t	0.41%
CTGC-------------------GCGC	144	-19	0.39%
CTGCGCGCGCGCCGAG-AGA-------	140	-13	0.38%

P212-YW-K562-Syn30crCD326ag-RNPaav-48h-BC11 **48h**	Ins	Del	HDR
30062 | 54.44%	**3.97%**	**15.22%**	**35.25%**
**Typical seqs**	**Reads**	**Type**	**pct.**
CTGCGCGCGCGCCGAGA|AGA**GGG**GCGC	13696	WT	45.56%
CTGCGCGCGCGCCGAGGTTTAAACTACGCG TGC	10169	+6 HDR	33.83%
CT---------------------GCGC	800	-21	2.66%
CTGCGCGCGCGCCGAG-AGAGGGGCGC	779	-1	2.59%
CTGCGCGCGCGCCGAGAaAGAGGGGCGC	615	+1 a	2.05%
CTGCGCGCGCGCCG---AGAGGGGCGC	443	-3	1.47%
CT----------------------GC	417	-23	1.39%
CTGCGC-----------------GCGC	166	-17	0.55%
-----------------------GCGC	162	-24	0.54%
CTGCGCGCGCGCCGAGGTTTAAACTACGCG CGC	117	+6	0.39%

P212-YW-K562-Syn31crCD326ah-RNPko-48h-BC6

P208-YW-K562-Syn31crCD326ah-RNPko-4h-BC6 4h	Ins	Del	HDR
7088 | 0.87%	0.35%	0.52%	0.00%
**Typical seqs**	**Reads**	**Type**	**pct.**
GCTGCGCGCGCGCCGAG|AAG**AGG**GGCG	7026	WT	99.13%
GCTGCGCGCGCGCCGAGgAAGAGGGGCG	20	+1 g	0.28%
GCTGCGC----------AAGAGGGGCG	9	-10	0.13%
GCTGCGCGCGCGCCGA------GGGCG	7	-6	0.10%
GCTGCGCGC--------AAGAGGGGCG	5	-8	0.07%
GCTGCGC-----TCGGGAAGAGGGGCG	5	-5	0.07%
GCTGCGCGCGCGCCGAGaAAGAGGGGCG	5	+1 a	0.07%
GCTGCGCGCGCGCC---AAGAGGGGCG	4	-3	0.06%
GCTGCGCTC-------GAAGAGGGGCG	4	-7	0.06%
GCTGC----------------GGGGCG	3	-16	0.04%

P209-YW-K562-Syn31crCD326ah-RNPko-8h-BC6 8h	Ins	Del	HDR
15914 | 3.97%	1.36%	2.60%	0.00%
**Typical seqs**	**Reads**	**Type**	**pct.**
GCTGCGCGCGCGCCGAG|AAG**AGG**GGCG	15283	WT	96.03%
GCTGCGCGCGCGCCGAGgAAGAGGGGCG	204	+1 g	1.28%
GCTGCGCGCGCGCC--GAAGAGGGGCG	60	-2	0.38%
GCT-----------------------G	41	-23	0.26%
GCTGCGCGCGCGCCG---AGAGGGGCG	30	-3	0.19%
GCTGCGCGCGCGCCG-GAAGAGGGGCG	27	-1	0.17%
GCTGCGCGCGCGCC-----GAGGGGCG	26	-5	0.16%
GCT---------------------GCG	25	-21	0.16%
GCTGCGCGCGCGCCGA-AAGAGGGGCG	24	-1	0.15%
GCT-------------GAAGAGGGGCG	18	-13	0.11%

P210-YW-K562-Syn31crCD326ah-RNPko-12h-BC6 12h	Ins	Del	HDR
29454 | 7.84%	1.89%	5.93%	0.01%
**Typical seqs**	**Reads**	**Type**	**pct.**
GCTGCGCGCGCGCCGAG|AAG**AGG**GGCG	27146	WT	92.16%
GCTGCGCGCGCGCCGAGgAAGAGGGGCG	545	+1 g	1.85%
GCT---------------------GCG	254	-21	0.86%
GCTGCGCGCGCGCCGA-AAGAGGGGCG	131	-1	0.44%
GCTGCGCGCGCGCC--GAAGAGGGGCG	119	-2	0.40%
GCT-----------------------G	106	-23	0.36%
GCTGCGC----------AAGAGGGGCG	99	-10	0.34%
GCTGCGCGCGCGCC-----GAGGGGCG	97	-5	0.33%
GCTGC------------AAGAGGGGCG	71	-12	0.24%
GCTGCGCGCGCGCC---AAGAGGGGCG	58	-3	0.20%

P211-YW-K562-Syn31crCD326ah-RNPko-24h-BC6 24h	Ins	Del	HDR
16382 | 40.49%	3.44%	37.02%	0.02%
**Typical seqs**	**Reads**	**Type**	**pct.**
GCTGCGCGCGCGCCGAG|AAG**AGG**GGCG	9749	WT	59.51%
GCT---------------------GCG	1294	-21	7.90%
GCT-----------------------G	666	-23	4.07%
GCT------------------------	499	-26	3.05%
GCTGCGCGCGCGCCGAGgAAGAGGGGCG	434	+1 g	2.65%
---------------------------	635	-29	3.88%
G--------------------------	262	-31	1.60%
---------------------------	635	-30	3.88%
------------------------GCG	236	-24	1.44%
GCTGC------------AAGAGGGGCG	212	-12	1.29%

P212-YW-K562-Syn31crCD326ah-RNPko-48h-BC6 48h	Ins	Del	HDR
15092 | 62.76%	3.83%	58.85%	0.08%
**Typical seqs**	**Reads**	**Type**	**pct.**
GCTGCGCGCGCGCCGAG|AAG**AGG**GGCG	5621	WT	37.24%
GCT---------------------GCG	2460	-21	16.30%
GCT-----------------------G	1679	-23	11.13%
GCTGCGCGCGCGCCGAGgAAGAGGGGCG	463	+1 g	3.07%
------------------------GCG	386	-24	2.56%
--------------------------G	381	-26	2.52%
GCT------------------------	538	-27	3.56%
GCTGC-------------------GCG	268	-19	1.78%
---------------------------	400	-30	2.65%
---------------------------	400	-32	2.65%

P212-YW-K562-Syn31crCD326ah-RNPaav-48h-BC12

P208-YW-K562-Syn31crCD326ah-RNPaav-4h-BC12 **4h**	Ins	Del	HDR
52027 | 5.71%	**1.00%**	**1.25%**	**3.46%**
**Typical seqs**	**Reads**	**Type**	**pct.**
GCTGCGCGCGCGCCGAG|AAG**AGG**GGCG	49055	WT	94.29%
GCTGCGCGCGCGCCGAGGTTTAAACTACGC GTG	1757	+6 HDR	3.38%
GCTGCGCGCGCGCCGAGgAAGAGGGGCG	469	+1 g	0.90%
GCTGC-------------------GCG	288	-19	0.55%
----------------GAAGAGGGGCG	211	-16	0.41%
GCTGCGCGCGCGCCGA-AAGAGGGGCG	51	-1	0.10%
GCTGCGCGC-------GAAGAGGGGCG	50	-7	0.10%
GCTGCGCGCGCGCC--GAAGAGGGGCG	31	-2	0.06%
GCTGCGCGCGCGCCGAGGTTTAAACTACGA GTG	17	+6	0.03%
GCTGCGCGCGCGCCGAG--GAGGGGCG	11	-2	0.02%

P209-YW-K562-Syn31crCD326ah-RNPaav-8h-BC12 **8h**	Ins	Del	HDR
35767 | 14.61%	**1.79%**	**2.37%**	**10.44%**
**Typical seqs**	**Reads**	**Type**	**pct.**
GCTGCGCGCGCGCCGAG|AAG**AGG**GGCG	30543	WT	85.39%
GCTGCGCGCGCGCCGAGGTTTAAACTACGC GTG	3598	+6 HDR	10.06%
GCTGCGCGCGCGCCGAGgAAGAGGGGCG	454	+1 g	1.27%
---------------------------	154	-30	0.43%
----------------GAAGAGGGGCG	101	-16	0.28%
GCTGC------------AAGAGGGGCG	90	-12	0.25%
GCTGCGCTC-------GAAGAGGGGCG	61	-7	0.17%
GCTGCGCGCGCGCCGA-----------	58	-14	0.16%
GCTGCGCTCG------GAAGAGGGGCG	52	-6	0.15%
GCTGCGCGCGCGCC--GAAGAGGGGCG	37	-2	0.10%

P210-YW-K562-Syn31crCD326ah-RNPaav-12h-BC12 **12h**	Ins	Del	HDR
36551 | 20.37%	**2.83%**	**3.44%**	**14.10%**
**Typical seqs**	**Reads**	**Type**	**pct.**
GCTGCGCGCGCGCCGAG|AAG**AGG**GGCG	29107	WT	79.63%
GCTGCGCGCGCGCCGAGGTTTAAACTACGC GTG	4984	+6 HDR	13.64%
GCTGCGCGCGCGCCGAGgAAGAGGGGCG	774	+1 g	2.12%
---------------------------	177	-28	0.48%
GCT---------------------GCG	124	-21	0.34%
GCTGCGCGCGCGCCGA-AAGAGGGGCG	101	-1	0.28%
GCTGCGCGCGCGCC--GAAGAGGGGCG	93	-2	0.25%
GCAT--------------------GCG	85	-20	0.23%
GCTGC-----------GAAGAGGGGCG	83	-11	0.23%
--------------------------G	67	-26	0.18%

P211-YW-K562-Syn31crCD326ah-RNPaav-24h-BC12 **24h**	Ins	Del	HDR
25379 | 55.67%	**4.42%**	**10.83%**	**40.42%**
**Typical seqs**	**Reads**	**Type**	**pct.**
GCTGCGCGCGCGCCGAG|AAG**AGG**GGCG	11250	WT	44.33%
GCTGCGCGCGCGCCGAGGTTTAAACTACGC GTG	9790	+6 HDR	38.58%
GCTGCGCGCGCGCCGAGgAAGAGGGGCG	719	+1 g	2.83%
GCT---------------------GCG	334	-21	1.32%
GCT-----------------------G	256	-23	1.01%
------------------------GCG	177	-24	0.70%
GCTGCGCGCGCC---------------	161	-16	0.63%
GCTGC-------------------GCG	128	-19	0.50%
GC-----------------------CG	110	-23	0.43%
GCTGCGCGCGCGCCGAG--GAGGGGCG	108	-2	0.43%

P212-YW-K562-Syn31crCD326ah-RNPaav-48h-BC12 **48h**	Ins	Del	HDR
20946 | 62.97%	**4.60%**	**14.42%**	**43.95%**
**Typical seqs**	**Reads**	**Type**	**pct.**
GCTGCGCGCGCGCCGAG|AAG**AGG**GGCG	7757	WT	37.03%
GCTGCGCGCGCGCCGAGGTTTAAACTACGC GTG	8867	+6 HDR	42.33%
GCTGCGCGCGCGCCGAGgAAGAGGGGCG	531	+1 g	2.54%
GCT---------------------GCG	485	-21	2.32%
GCT-----------------------G	354	-23	1.69%
GCTGCGCGCGCGCC--GAAGAGGGGCG	194	-2	0.93%
GCTGCGCGCGCGCC-----GAGGGGCG	131	-5	0.63%
GCTGC-------------------GCG	120	-19	0.57%
GCTGCGCGCGCGCCGA-AAGAGGGGCG	72	-1	0.34%
---------------------------	120	-30	0.57%

P212-YW-K562-Syn47crHBG200g-RNPko-48h-BC1

P208-YW-K562-Syn47crHBG200g-RNPko-4h-BC1 4h	Ins	Del	HDR
25603 | 9.72%	3.36%	6.35%	0.00%
**Typical seqs**	**Reads**	**Type**	**pct.**
GCATTGAGATAGTGTGG|GGA**AGG**GGCC	23115	WT	90.28%
GCATTGAGATAGTGT-GGGAAGGGGCC	873	-1	3.41%
GCATTGAGATAGTGTGGgGGAAGGGGCC	766	+1 g	2.99%
GCATTGAGATAGTGT--GGAAGGGGCC	338	-2	1.32%
GCATTGAGATA-----GGGAAGGGGCC	55	-5	0.21%
GCATTGAGATAGTGTGGGGGGAAGGGGCC	30	+2	0.12%
GCATT-----------GGGAAGGGGCC	27	-11	0.11%
GCATTGAGAT-------GGAAGGGGCC	25	-7	0.10%
GCATTGAGATAGTGT--------GGCC	25	-8	0.10%
GCATTGAGATAGTGTGG----------	50	-13	0.20%

P209-YW-K562-Syn47crHBG200g-RNPko-8h-BC1 8h	Ins	Del	HDR
29254 | 16.39%	5.99%	10.39%	0.02%
**Typical seqs**	**Reads**	**Type**	**pct.**
GCATTGAGATAGTGTGG|GGA**AGG**GGCC	24458	WT	83.61%
GCATTGAGATAGTGTGGgGGAAGGGGCC	1508	+1 g	5.15%
GCATTGAGATAGTGT-GGGAAGGGGCC	964	-1	3.30%
GCATTGAGATAGTGT--GGAAGGGGCC	868	-2	2.97%
GCATTGAGATAGTGT--------GGCC	128	-8	0.44%
GCATTGAGATAGT----GGAAGGGGCC	103	-4	0.35%
GCATTGAGATAGT---GGGAAGGGGCC	101	-3	0.35%
GCATTGAGATAGTGT-------GGGCC	81	-7	0.28%
GCATTGAGATA------GGAAGGGGCC	68	-6	0.23%
GCATTGA-----------GAAGGGGCC	56	-11	0.19%

P210-YW-K562-Syn47crHBG200g-RNPko-12h-BC1 12h	Ins	Del	HDR
28764 | 24.14%	6.65%	17.45%	0.05%
**Typical seqs**	**Reads**	**Type**	**pct.**
GCATTGAGATAGTGTGG|GGA**AGG**GGCC	21820	WT	75.86%
GCATTGAGATAGTGT-GGGAAGGGGCC	1685	-1	5.86%
GCATTGAGATAGTGTGGgGGAAGGGGCC	1605	+1 g	5.58%
GCATTGAGATAGTGT--GGAAGGGGCC	1197	-2	4.16%
GCATTGAGATAGTGT--------GGCC	162	-8	0.56%
GCATTGAGATAGTGT------GGGGCC	116	-6	0.40%
GCATTGAGATAGT----GGAAGGGGCC	106	-4	0.37%
GCATTGAGATAGTGT-------GGGCC	104	-7	0.36%
GCATT----------------------	113	-27	0.39%
GCATTGAGATAGTGT------------	97	-19	0.34%

P211-YW-K562-Syn47crHBG200g-RNPko-24h-BC1 24h	Ins	Del	HDR
36992 | 46.21%	10.93%	35.23%	0.05%
**Typical seqs**	**Reads**	**Type**	**pct.**
GCATTGAGATAGTGTGG|GGA**AGG**GGCC	19899	WT	53.79%
GCATTGAGATAGTGTGGgGGAAGGGGCC	3277	+1 g	8.86%
GCATTGAGATAGTGT--GGAAGGGGCC	2927	-2	7.91%
GCATTGAGATAGTGT-GGGAAGGGGCC	2525	-1	6.83%
GCATTGAGATAGTGT--------GGCC	755	-8	2.04%
GCATTGAGATAGTGT-------GGGCC	637	-7	1.72%
GCATTGAGATAGT----GGAAGGGGCC	264	-4	0.71%
GCATTGAGATAGTGTGGtGGAAGGGGCC	194	+1 t	0.52%
GCATTGAGATA------GGAAGGGGCC	191	-6	0.52%
GCATTGAGAT----------AGGGGCC	179	-10	0.48%

P212-YW-K562-Syn47crHBG200g-RNPko-48h-BC1 48h	Ins	Del	HDR
49029 | 57.49%	12.57%	44.87%	0.06%
**Typical seqs**	**Reads**	**Type**	**pct.**
GCATTGAGATAGTGTGG|GGA**AGG**GGCC	20840	WT	42.51%
GCATTGAGATAGTGT--GGAAGGGGCC	4985	-2	10.17%
GCATTGAGATAGTGTGGgGGAAGGGGCC	4926	+1 g	10.05%
GCATTGAGATAGTGT-GGGAAGGGGCC	3500	-1	7.14%
GCATTGAGATAGTGT--------GGCC	1355	-8	2.76%
GCATTGAGATAGTGT-------GGGCC	937	-7	1.91%
GCATTGA-----------GAAGGGGCC	401	-11	0.82%
GCATTG---------------------	461	-25	0.94%
GCATTGAGATAGTGT---------GCC	332	-9	0.68%
GCATTGAGATA------GGAAGGGGCC	326	-6	0.66%

P212-YW-K562-Syn47crHBG200g-RNPaav-48h-BC5

P208-YW-K562-Syn47crHBG200g-RNPaav-4h-BC5 4h	Ins	Del	HDR
47762 | 10.15%	4.59%	5.22%	0.34%
**Typical seqs**	**Reads**	**Type**	**pct.**
GCATTGAGATAGTGTGG|GGA**AGG**GGCC	42915	WT	89.85%
GCATTGAGATAGTGTGGgGGAAGGGGCC	1931	+1 g	4.04%
GCATTGAGATAGTGT-GGGAAGGGGCC	1407	-1	2.95%
GCATTGAGATAGTGT--GGAAGGGGCC	484	-2	1.01%
GCATTGAGATAGGTTT---------------AAAC	163	-7 HDR	0.34%
GCATTGAGATA------GGAAGGGGCC	82	-6	0.17%
GCATTGAGATAGT----GGAAGGGGCC	60	-4	0.13%
GCATTGAGATAGTGTGGtGGAAGGGGCC	55	+1 t	0.12%
GCATTGAGATAGTGT---GAAGGGGCC	54	-3	0.11%
GCATTGAGATA--GTGGGGAAGGGGCC	52	-2	0.11%

P209-YW-K562-Syn47crHBG200g-RNPaav-8h-BC5 8h	Ins	Del	HDR
43730 | 18.66%	6.17%	10.56%	1.93%
**Typical seqs**	**Reads**	**Type**	**pct.**
GCATTGAGATAGTGTGG|GGA**AGG**GGCC	35572	WT	81.34%
GCATTGAGATAGTGTGGgGGAAGGGGCC	2221	+1 g	5.08%
GCATTGAGATAGTGT-GGGAAGGGGCC	1942	-1	4.44%
GCATTGAGATAGTGT--GGAAGGGGCC	1099	-2	2.51%
GCATTGAGATAGGTTT---------------AAAC	827	-7 HDR	1.89%
GCATTGAGATAGT---GGGAAGGGGCC	114	-3	0.26%
GCATTGAGATAGTGTGGtGGAAGGGGCC	102	+1 t	0.23%
GCATTGAGATAGTGTGGGGGGAAGGGGCC	98	+2	0.22%
GCATTGAGATAGTGT-------GGGCC	94	-7	0.21%
GCATTGAGATA-------GAAGGGGCC	86	-7	0.20%

P210-YW-K562-Syn47crHBG200g-RNPaav-12h-BC5 12h	Ins	Del	HDR
35076 | 28.80%	7.79%	15.27%	5.74%
**Typical seqs**	**Reads**	**Type**	**pct.**
GCATTGAGATAGTGTGG|GGA**AGG**GGCC	24975	WT	71.20%
GCATTGAGATAGTGTGGgGGAAGGGGCC	2193	+1 g	6.25%
GCATTGAGATAGGTTT---------------AAAC	1963	-7 HDR	5.60%
GCATTGAGATAGTGT--GGAAGGGGCC	1726	-2	4.92%
GCATTGAGATAGTGT-GGGAAGGGGCC	1664	-1	4.74%
GCATTGAGATAGTGT-------GGGCC	161	-7	0.46%
GCATTGAGATAGTGT--------GGCC	158	-8	0.45%
GCATTGAGATAGT---GGGAAGGGGCC	128	-3	0.36%
GCATTGAGATAGTGTGGtGGAAGGGGCC	106	+1 t	0.30%
GCATTGAGATA------GGAAGGGGCC	87	-6	0.25%

P211-YW-K562-Syn47crHBG200g-RNPaav-24h-BC5 24h	Ins	Del	HDR
34517 | 45.17%	9.36%	24.42%	11.39%
**Typical seqs**	**Reads**	**Type**	**pct.**
GCATTGAGATAGTGTGG|GGA**AGG**GGCC	18927	WT	54.83%
GCATTGAGATAGGTTT---------------AAAC	3845	-7 HDR	11.14%
GCATTGAGATAGTGTGGgGGAAGGGGCC	2652	+1 g	7.68%
GCATTGAGATAGTGT--GGAAGGGGCC	1866	-2	5.41%
GCATTGAGATAGTGT-GGGAAGGGGCC	1738	-1	5.04%
GCATTGAGATAGTGT--------GGCC	428	-8	1.24%
GCATTGAGATAGTGT-------GGGCC	244	-7	0.71%
GCATTGAGATAGTGTGG---------C	135	-9	0.39%
------------------------GCC	121	-24	0.35%
GCATT-----------GGGAAGGGGCC	120	-11	0.35%

P212-YW-K562-Syn47crHBG200g-RNPaav-48h-BC5 48h	Ins	Del	HDR
33685 | 58.92%	12.80%	32.95%	13.17%
**Typical seqs**	**Reads**	**Type**	**pct.**
GCATTGAGATAGTGTGG|GGA**AGG**GGCC	13838	WT	41.08%
GCATTGAGATAGGTTT---------------AAAC	4349	-7 HDR	12.91%
GCATTGAGATAGTGTGGgGGAAGGGGCC	3393	+1 g	10.07%
GCATTGAGATAGTGT--GGAAGGGGCC	2937	-2	8.72%
GCATTGAGATAGTGT-GGGAAGGGGCC	2506	-1	7.44%
GCATTGAGATAGTGT--------GGCC	483	-8	1.43%
GCATTGAGATAGTGT-------GGGCC	361	-7	1.07%
GCATTGAGATAGT----GGAAGGGGCC	198	-4	0.59%
GCATTGAGATAGT---GGGAAGGGGCC	192	-3	0.57%
GCATT----------------------	231	-27	0.69%

P212-YW-K562-Syn48crHBG200h-RNPko-48h-BC2

P208-YW-K562-Syn48crHBG200h-RNPko-4h-BC2 4h	Ins	Del	HDR
26472 | 10.97%	9.55%	1.42%	0.00%
Typical seqs	Reads	Type	pct.
ATTTGCATTGAGATAGT|GTGGGGAAGG	23568	WT	89.03%
ATTTGCATTGAGATAGTtGTGGGGAAGG	2390	+1 t	9.03%
ATTTGCATTGAGA---TGTGGGGAAGG	67	-3	0.25%
ATTTGCATTGAGAT--TGTGGGGAAGG	63	-2	0.24%
ATTTGCATTGAGATA--GTGGGGAAGG	56	-2	0.21%
ATTTGCATTGAGATA-TGTGGGGAAGG	25	-1	0.09%
ATTTGCATT------GTGTGGGGAAGG	23	-6	0.09%
ATTTGCATTGAGATAGT---------G	16	-9	0.06%
ATTTGCATTGAGATAGTGT-GGGAAGG	14	-1	0.05%
ATTTGCATTGA------GGGGGGAAGG	13	-6	0.05%

P209-YW-K562-Syn48crHBG200h-RNPko-8h-BC2 8h	Ins	Del	HDR
30786 | 18.91%	15.78%	3.14%	0.00%
Typical seqs	Reads	Type	pct.
ATTTGCATTGAGATAGT|GTGGGGAAGG	24963	WT	81.09%
ATTTGCATTGAGATAGTtGTGGGGAAGG	4512	+1 t	14.66%
ATTTGCATTGAGATA--GTGGGGAAGG	146	-2	0.47%
ATTTGCATTGAGATA-TGTGGGGAAGG	87	-1	0.28%
ATTTGCATTGAGA---TGTGGGGAAGG	86	-3	0.28%
ATTTGCATTGAGAT--TGTGGGGAAGG	73	-2	0.24%
ATTTGCATT------GTGTGGGGAAGG	58	-6	0.19%
ATTTGCATT------------------	49	-19	0.16%
ATTTGCATTGAGATAG-GTGGGGAAGG	42	-1	0.14%
ATTTGCA---------TGTGGGGAAGG	40	-9	0.13%

P210-YW-K562-Syn48crHBG200h-RNPko-12h-BC2 12h	Ins	Del	HDR
24582 | 26.23%	20.27%	5.96%	0.00%
Typical seqs	Reads	Type	pct.
ATTTGCATTGAGATAGT|GTGGGGAAGG	18134	WT	73.77%
ATTTGCATTGAGATAGTtGTGGGGAAGG	4599	+1 t	18.71%
ATTTGCATTGAGATA--GTGGGGAAGG	240	-2	0.98%
ATTTGCATTGAGATA-TGTGGGGAAGG	87	-1	0.35%
ATTTGCATTGAGA---TGTGGGGAAGG	84	-3	0.34%
ATTTGCATTGAGAT--TGTGGGGAAGG	77	-2	0.31%
ATTTGCAT--------TGTGGGGAAGG	67	-8	0.27%
ATTTGCAT----------TGGGGAAGG	61	-10	0.25%
ATTTGCATT------GTGTGGGGAAGG	45	-6	0.18%
ATTTGCATT-----------GGGAAGG	42	-11	0.17%

P211-YW-K562-Syn48crHBG200h-RNPko-24h-BC2 24h	Ins	Del	HDR
39056 | 44.89%	31.63%	13.26%	0.00%
Typical seqs	Reads	Type	pct.
ATTTGCATTGAGATAGT|GTGGGGAAGG	21524	WT	55.11%
ATTTGCATTGAGATAGTtGTGGGGAAGG	11274	+1 t	28.87%
ATTTGCATTGAGATA--GTGGGGAAGG	631	-2	1.62%
ATTTGCATTGAGAT--TGTGGGGAAGG	434	-2	1.11%
ATTTGCATTGAGA---TGTGGGGAAGG	208	-3	0.53%
ATTTGCATT------GTGTGGGGAAGG	203	-6	0.52%
ATTTGCATT------------------	327	-19	0.84%
ATTTGCATTGAGATA------------	206	-13	0.53%
ATTT-----------------------	177	-24	0.45%
ATTTG----------------------	146	-28	0.37%

P212-YW-K562-Syn48crHBG200h-RNPko-48h-BC2 48h	Ins	Del	HDR
46762 | 57.68%	40.37%	17.31%	0.00%
Typical seqs	Reads	Type	pct.
ATTTGCATTGAGATAGT|GTGGGGAAGG	19789	WT	42.32%
ATTTGCATTGAGATAGTtGTGGGGAAGG	17433	+1 t	37.28%
ATTTGCATTGAGATA--GTGGGGAAGG	699	-2	1.49%
ATTTGCATTGAGAT--TGTGGGGAAGG	380	-2	0.81%
ATTTGCATTGAGA---TGTGGGGAAGG	332	-3	0.71%
ATTTGCATT------------------	480	-19	1.03%
ATTTGCATTGAGATA------------	431	-13	0.92%
ATTT-----------------------	325	-24	0.70%
ATTTGCAT----------TGGGGAAGG	226	-10	0.48%
ATTTG----------------------	312	-28	0.67%

P212-YW-K562-Syn49crHBG200i-RNPko-48h-BC3

P208-YW-K562-Syn49crHBG200i-RNPko-4h-BC3 4h	Ins	Del	HDR
25578 | 3.14%	0.19%	2.95%	0.00%
**Typical seqs**	**Reads**	**Type**	**pct.**
TATTTGCATTGAGATAG|TGT**GGG**GAAG	24775	WT	96.86%
TATTTGCATTGAGA---TGTGGGGAAG	210	-3	0.82%
TATTTGCATTGAGATA--GTGGGGAAG	159	-2	0.62%
TATTTGCATTGAGATAG-GTGGGGAAG	119	-1	0.47%
TATTTGCATTGAGATA-TGTGGGGAAG	103	-1	0.40%
TATTTGCATTGAGAT--TGTGGGGAAG	43	-2	0.17%
TATTTGCATT------GTGTGGGGAAG	41	-6	0.16%
TATTTGCATTGAGATAGtTGTGGGGAAG	25	+1 t	0.10%
TATTTGCATTGAGATAGgTGTGGGGAAG	23	+1 g	0.09%
TA---------------TGTGGGGAAG	17	-15	0.07%

P209-YW-K562-Syn49crHBG200i-RNPko-8h-BC3 8h	Ins	Del	HDR
25225 | 9.82%	1.17%	8.65%	0.00%
**Typical seqs**	**Reads**	**Type**	**pct.**
TATTTGCATTGAGATAG|TGT**GGG**GAAG	22749	WT	90.18%
TATTTGCATTGAGATA-TGTGGGGAAG	473	-1	1.88%
TATTTGCATTGAGA---TGTGGGGAAG	386	-3	1.53%
TATTTGCATTGAGAT--TGTGGGGAAG	369	-2	1.46%
TATTTGCATTGAGATA--GTGGGGAAG	204	-2	0.81%
TATTTGCATTGAGATAG-GTGGGGAAG	143	-1	0.57%
TATTTGCAT--------TGTGGGGAAG	95	-8	0.38%
TATTTGCATTGAGATAGTGGGGTTGTGGGGAAG	87	+6	0.34%
TATTTGCATTGAGATAGtTGTGGGGAAG	75	+1 t	0.30%
TATTTGCATTGAGATAG---GGGGAAG	69	-3	0.27%

P210-YW-K562-Syn49crHBG200i-RNPko-12h-BC3 12h	Ins	Del	HDR
18330 | 15.39%	1.60%	13.79%	0.00%
**Typical seqs**	**Reads**	**Type**	**pct.**
TATTTGCATTGAGATAG|TGT**GGG**GAAG	15509	WT	84.61%
TATTTGCATTGAGAT--TGTGGGGAAG	543	-2	2.96%
TATTTGCATTGAGA---TGTGGGGAAG	209	-3	1.14%
TATTTGCAT--------TGTGGGGAAG	183	-8	1.00%
TATTTGCATTGAGATA--GTGGGGAAG	176	-2	0.96%
TATTTGCATTGAGATAG-GTGGGGAAG	160	-1	0.87%
TATTTGCATTGAGATA-TGTGGGGAAG	157	-1	0.86%
TATTTGCATT------GTGTGGGGAAG	106	-6	0.58%
TATTTGCATTGAGATA-----------	172	-13	0.94%
TATTTGCATTGAGATAGtTGTGGGGAAG	71	+1 t	0.39%

P211-YW-K562-Syn49crHBG200i-RNPko-24h-BC3 24h	Ins	Del	HDR
31719 | 33.71%	3.24%	30.46%	0.00%
**Typical seqs**	**Reads**	**Type**	**pct.**
TATTTGCATTGAGATAG|TGT**GGG**GAAG	21028	WT	66.29%
TATTTGCAT--------TGTGGGGAAG	1282	-8	4.04%
TATTTGCATTGAGA---TGTGGGGAAG	804	-3	2.53%
TATTTGCATTGAGAT--TGTGGGGAAG	761	-2	2.40%
TATTTGCATTGAGATA--GTGGGGAAG	536	-2	1.69%
TATTTGCATTGAGATA-TGTGGGGAAG	485	-1	1.53%
TATTTGCATTGAGATAG-GTGGGGAAG	362	-1	1.14%
TATTTGCAT----------TGGGGAAG	298	-10	0.94%
TATTTGCATTGA-----------GAAG	242	-11	0.76%
TATTTGCATTG----AGTGTGGGGAAG	225	-4	0.71%

P212-YW-K562-Syn49crHBG200i-RNPko-48h-BC3 48h	Ins	Del	HDR
38730 | 46.05%	4.95%	41.10%	0.00%
**Typical seqs**	**Reads**	**Type**	**pct.**
TATTTGCATTGAGATAG|TGT**GGG**GAAG	20893	WT	53.95%
TATTTGCATTGAGAT--TGTGGGGAAG	2043	-2	5.27%
TATTTGCAT--------TGTGGGGAAG	1407	-8	3.63%
TATTTGCATTGAGA---TGTGGGGAAG	1283	-3	3.31%
TATTT----------------------	897	-24	2.32%
TATTTGCATTGAGATA--GTGGGGAAG	594	-2	1.53%
TATTTGCATTGAGATA-TGTGGGGAAG	536	-1	1.38%
TATTTGCATTGAGATAG-GTGGGGAAG	485	-1	1.25%
TATTTGCA---------TGTGGGGAAG	466	-9	1.20%
TATTTGCATT------GTGTGGGGAAG	423	-6	1.09%

P212-YW-K562-Syn50crHBG200j-RNPko-48h-BC4

P208-YW-K562-Syn50crHBG200j-RNPko-4h-BC4 4h	Ins	Del	HDR
21433 | 2.75%	1.85%	0.90%	0.00%
**Typical seqs**	**Reads**	**Type**	**pct.**
ATATTTGCATTGAGATA|GTG**TGG**GGAA	20844	WT	97.25%
ATATTTGCATTGAGATAaGTGTGGGGAA	343	+1 a	1.60%
ATATTTGCATTGAGAT-GTGTGGGGAA	37	-1	0.17%
ATATTTGCA-------AGTGTGGGGAA	28	-7	0.13%
ATATTTGCATTG----AGTGTGGGGAA	25	-4	0.12%
ATATTTGCATTGA---AGTGTGGGGAA	23	-3	0.11%
ATATTTGCATTGAGATATAGTGTGGGGAA	23	+2	0.11%
ATATTTGCATTGAGATAgTTGTGGGGAA	16	+1 g	0.07%
ATATTTGCATTGAGAT----------A	12	-10	0.06%
ATATTTGCATTGAGATAtGTGTGGGGAA	10	+1 t	0.05%

P209-YW-K562-Syn50crHBG200j-RNPko-8h-BC4 8h	Ins	Del	HDR
23035 | 6.13%	4.19%	1.94%	0.00%
**Typical seqs**	**Reads**	**Type**	**pct.**
ATATTTGCATTGAGATA|GTG**TGG**GGAA	21623	WT	93.87%
ATATTTGCATTGAGATAaGTGTGGGGAA	809	+1 a	3.51%
ATATTTGCATTGAGAT-GTGTGGGGAA	99	-1	0.43%
ATATTTGCATTG----AGTGTGGGGAA	56	-4	0.24%
ATATTTGCATTGAGATATAGTGTGGGGAA	53	+2	0.23%
ATATTTGCATT-------------GAA	27	-13	0.12%
ATATTTGCATTGAG--AGTGTGGGGAA	25	-2	0.11%
ATATTTGCATT-----------GGGAA	18	-11	0.08%
ATATTTGCATTGAGATAgTTGTGGGGAA	18	+1 g	0.08%
ATATTTGCATTGAGATA--GTGGGGAA	18	-2	0.08%

P210-YW-K562-Syn50crHBG200j-RNPko-12h-BC4 12h	Ins	Del	HDR
18075 | 9.75%	4.89%	4.86%	0.00%
**Typical seqs**	**Reads**	**Type**	**pct.**
ATATTTGCATTGAGATA|GTG**TGG**GGAA	16313	WT	90.25%
ATATTTGCATTGAGATAaGTGTGGGGAA	685	+1 a	3.79%
ATATTTGCATTGAGATATAGTGTGGGGAA	99	+2	0.55%
ATATTTGCATTGAGAT-GTGTGGGGAA	78	-1	0.43%
ATATTTGCATTG----AGTGTGGGGAA	77	-4	0.43%
ATATTTGCAT--------TGTGGGGAA	68	-8	0.38%
ATATTTGCATTGAG--AGTGTGGGGAA	37	-2	0.20%
ATATTTGC-----------------AA	34	-17	0.19%
ATATTT---------------------	33	-24	0.18%
ATATTTGCATTGA-----------GAA	27	-11	0.15%

P211-YW-K562-Syn50crHBG200j-RNPko-24h-BC4 24h	Ins	Del	HDR
32301 | 23.90%	10.02%	13.87%	0.00%
**Typical seqs**	**Reads**	**Type**	**pct.**
ATATTTGCATTGAGATA|GTG**TGG**GGAA	24582	WT	76.10%
ATATTTGCATTGAGATAaGTGTGGGGAA	2529	+1 a	7.83%
ATATTTGCATTGAGAT-GTGTGGGGAA	350	-1	1.08%
ATATTTGCATTG----AGTGTGGGGAA	278	-4	0.86%
ATATTTGCATTGAGATATAGTGTGGGGAA	253	+2	0.78%
ATATTTGCATTGAGATA-TGTGGGGAA	227	-1	0.70%
ATATTTGCATTGA-----------GAA	220	-11	0.68%
ATATTTGCAT--------TGTGGGGAA	216	-8	0.67%
ATATTTGCATT------GTGTGGGGAA	211	-6	0.65%
ATATTTGCATT-------------GAA	176	-13	0.54%

P212-YW-K562-Syn50crHBG200j-RNPko-48h-BC4 48h	Ins	Del	HDR
35552 | 36.22%	14.65%	21.57%	0.00%
**Typical seqs**	**Reads**	**Type**	**pct.**
ATATTTGCATTGAGATA|GTG**TGG**GGAA	22676	WT	63.78%
ATATTTGCATTGAGATAaGTGTGGGGAA	3776	+1 a	10.62%
ATATTTGCATTGAGATATAGTGTGGGGAA	641	+2	1.80%
ATATTTGCATTG----AGTGTGGGGAA	452	-4	1.27%
ATATTTGCATT------GTGTGGGGAA	453	-6	1.27%
ATATTTGCAT--------TGTGGGGAA	362	-8	1.02%
ATATTTGCATTGAGAT-GTGTGGGGAA	358	-1	1.01%
ATATTT---------------------	558	-24	1.57%
ATATTTGCATT-------------GAA	198	-13	0.56%
ATATTTGCATTGA-----------GAA	193	-11	0.54%

P201-YW-TCs-Syn20crAAVS1c-KO

P201-YW-TCs-Syn20crAAVS1c-KO-DMSO-BC1	Ins	Del	HDR
47861 | 76.23%	**39.04%**	**36.70%**	**0.49%**
**Typical seqs**	**Reads**	**Type**	**pct.**
TCTAACCCCCACCTCCT|GTT**AGG**CAGA	11376	WT	23.77%
TCTAACCCCCACCTCCTtGTTAGGCAGA	16754	+1 t	35.01%
TCTAACCCCCA----------------	3135	-18	6.55%
TCTAACCCCCACC--------------	2476	-15	5.17%
TCTAACCCCCACCTCC--TTAGGCAGA	660	-2	1.38%
TCTAACCCCCACCT-CTGTTAGGCAGA	604	-1	1.26%
TCTAA----------------------	589	-24	1.23%
---------------------------	619	-28	1.29%
TCTAACCCCCACCTCC-GTTAGGCAGA	459	-1	0.96%
TCTAACCCCCA---CCTGTTAGGCAGA	396	-3	0.83%

P201-YW-TCs-Syn20crAAVS1c-KO-CRISPYmix-BC4	Ins	Del	HDR
26306 | 66.67%	**29.15%**	**36.85%**	**0.67%**
**Typical seqs**	**Reads**	**Type**	**pct.**
TCTAACCCCCACCTCCT|GTT**AGG**CAGA	8767	WT	33.33%
TCTAACCCCCACCTCCTtGTTAGGCAGA	6901	+1 t	26.23%
TCTAACCCCCA----------------	1662	-18	6.32%
TCTAACCCCCACC--------------	1079	-15	4.10%
TCTAA----------------------	577	-24	2.19%
---------------------------	544	-28	2.07%
TCTAACCCCCACCT-CTGTTAGGCAGA	340	-1	1.29%
TCTAACCCCCACCTCC--TTAGGCAGA	315	-2	1.20%
TCTAACCCCCA---CCTGTTAGGCAGA	257	-3	0.98%
TCTAACCCCCAC-----------CAGA	236	-11	0.90%

P201-YW-TCs-Syn20crAAVS1c-KO-M3814-BC2	Ins	Del	HDR
23374 | 67.28%	**3.31%**	**63.43%**	**0.55%**
**Typical seqs**	**Reads**	**Type**	**pct.**
TCTAACCCCCACCTCCT|GTT**AGG**CAGA	7647	WT	32.72%
TCTAACCCCCA----------------	4097	-18	17.53%
TCTAACCCCCACC--------------	2638	-15	11.29%
---------------------------	638	-28	2.73%
TCTAA----------------------	584	-24	2.50%
TCTAACCCCCAC-----------CAGA	405	-11	1.73%
TCTAACCCCCACCTCCTtGTTAGGCAGA	349	+1 t	1.49%
TCTAACCCC-------------CAGA	303	-14	1.30%
TCTAAC-----------------CAGA	230	-17	0.98%
TCTAACCCCCACC-----TTAGGCAGA	220	-5	0.94%

P201-YW-TCs-Syn20crAAVS1c-KO-NaB-BC3	Ins	Del	HDR
33580 | 73.84%	**38.40%**	**34.85%**	**0.59%**
**Typical seqs**	**Reads**	**Type**	**pct.**
TCTAACCCCCACCTCCT|GTT**AGG**CAGA	8784	WT	26.16%
TCTAACCCCCACCTCCTtGTTAGGCAGA	11609	+1 t	34.57%
TCTAACCCCCA----------------	1837	-18	5.47%
TCTAACCCCCACC--------------	1422	-15	4.23%
TCTAA----------------------	497	-24	1.48%
TCTAACCCCCACCTCC--TTAGGCAGA	416	-2	1.24%
---------------------------	437	-28	1.30%
TCTAACCCCCACCTCC-GTTAGGCAGA	399	-1	1.19%
TCTAACCCCCACCT-CTGTTAGGCAGA	320	-1	0.95%
TCTAACCCCCA---CCTGTTAGGCAGA	294	-3	0.88%

P201-YW-TCs-Syn20crAAVS1c-AAV6KI

P201-YW-TCs-Syn20crAAVS1c-AAV6KI-DMSO-BC9	Ins	Del	HDR
55645 | 89.29%	34.90%	14.42%	39.97%
**Typical seqs**	**Reads**	**Type**	**pct.**
TCTAACCCCCACCTCCT|GTT**AGG**CAGA	5959	WT	10.71%
TCTAACCCACGCGTAGTTTAAACTTAGGCAGA	21429	+5 HDR	38.51%
TCTAACCCCCACCTCCTtGTTAGGCAGA	16995	+1 t	30.54%
TCTAACCCCCACC--------------	679	-15	1.22%
TCTAACCCCCA-----------------	618	-18	1.11%
TCTAACCCCCACCTCC--TTAGGCAGA	571	-2	1.03%
TCTAACCCCCACCTCC-GTTAGGCAGA	417	-1	0.75%
TCTAACCCCCA---CCTGTTAGGCAGA	409	-3	0.74%
TCTAACCCCCACCTC--GTTAGGCAGA	305	-2	0.55%
TCTAACCCCCACCT--TGTTAGGCAGA	225	-2	0.40%

P201-YW-TCs-Syn20crAAVS1c-AAV6KI-CRISPYmix-BC12	Ins	Del	HDR
43713 | 90.48%	30.04%	13.02%	47.42%
**Typical seqs**	**Reads**	**Type**	**pct.**
TCTAACCCCCACCTCCT|GTT**AGG**CAGA	4163	WT	9.52%
TCTAACCCACGCGTAGTTTAAACTTAGGCAGA	20004	+5 HDR	45.76%
TCTAACCCCCACCTCCTtGTTAGGCAGA	11146	+1 t	25.50%
TCTAACCCCCA-----------------	489	-18	1.12%
TCTAACCCCCACC--------------	480	-15	1.10%
TCTAACCCCCACCT-CTGTTAGGCAGA	330	-1	0.75%
TCTAACCCCCACCTCC--TTAGGCAGA	306	-2	0.70%
TCTAACCCCCACCTC--GTTAGGCAGA	287	-2	0.66%
TCTAACCCCCA---CCTGTTAGGCAGA	248	-3	0.57%
TCTAACCCCCACCTCC-GTTAGGCAGA	221	-1	0.51%

P201-YW-TCs-Syn20crAAVS1c-AAV6KI-M3814-BC10	Ins	Del	HDR
47974 | 94.21%	4.67%	8.08%	81.46%
**Typical seqs**	**Reads**	**Type**	**pct.**
TCTAACCCCCACCTCCT|GTT**AGG**CAGA	2778	WT	5.79%
TCTAACCCACGCGTAGTTTAAACTTAGGCAGA	37663	+5 HDR	78.51%
TCTAACCCCCA-----------------	823	-18	1.72%
TCTAACCCCCACC--------------	801	-15	1.67%
TCTAACCCCCACCTCCTtGTTAGGCAGA	657	+1 t	1.37%
TCTAACCCACGCGTAGTTTAACC-----TTAGGCAGA	215	+5	0.45%
TCTAA----------------------	149	-24	0.31%
--------------------------	150	-28	0.31%
TCTAACCCCCAC-----------CAGA	109	-11	0.23%
TCTAACCCACGCGTAGTTTACACTTAGGCAGA	104	+5	0.22%

P201-YW-TCs-Syn20crAAVS1c-AAV6KI-NaB-BC11	Ins	Del	HDR
61633 | 91.43%	35.72%	12.75%	42.96%
**Typical seqs**	**Reads**	**Type**	**pct.**
TCTAACCCCCACCTCCT|GTT**AGG**CAGA	5284	WT	8.57%
TCTAACCCACGCGTAGTTTAAACTTAGGCAGA	25539	+5 HDR	41.44%
TCTAACCCCCACCTCCTtGTTAGGCAGA	19273	+1 t	31.27%
TCTAACCCCCA-----------------	729	-18	1.18%
TCTAACCCCCACCTCC--TTAGGCAGA	590	-2	0.96%
TCTAACCCCCACC--------------	591	-15	0.96%
TCTAACCCCCACCT-CTGTTAGGCAGA	463	-1	0.75%
TCTAACCCCCACCTCC-GTTAGGCAGA	410	-1	0.67%
TCTAACCCCCACCTC--GTTAGGCAGA	282	-2	0.46%
TCTAACCCCCA---CCTGTTAGGCAGA	267	-3	0.43%

P201-YW-TCs-Syn25crAAVS1h-KO

P201-YW-TCs-Syn25crAAVS1h-KO-DMSO-BC5<br>58908 | 57.31%	Ins<br>7.43%	Del<br>49.40%	HDR<br>0.48%
**Typical seqs**	**Reads**	**Type**	**pct.**
TAAGGAATCTGCCTAAC|AGG**AGG**TGGG	25150	WT	42.69%
TA---------------AGGAGGTGGG	8954	-15	15.20%
TAAGGAATCTGCCTAACcAGGAGGTGGG	2939	+1 c	4.99%
TA------------------AGGTGGG	2847	-18	4.83%
TAAGGAATCTGCCTA--AGGAGGTGGG	2246	-2	3.81%
TAAGGAATCTGCCTAA-AGGAGGTGGG	1435	-1	2.44%
TAAGGAATCTGCCT-ACAGGAGGTGGG	976	-1	1.66%
TAAGGAATCTG-----CAGGAGGTGGG	796	-5	1.35%
TAAGGAATCTGC----CAGGAGGTGGG	762	-4	1.29%
TAAGGAATCTGCCTAAC--GAGGTGGG	576	-2	0.98%

P201-YW-TCs-Syn25crAAVS1h-KO-CRISPYmix-BC8<br>71023 | 51.15%	Ins<br>5.52%	Del<br>45.19%	HDR<br>0.44%
**Typical seqs**	**Reads**	**Type**	**pct.**
TAAGGAATCTGCCTAAC|AGG**AGG**TGGG	34693	WT	48.85%
TA---------------AGGAGGTGGG	8097	-15	11.40%
TA------------------AGGTGGG	3418	-18	4.81%
TAAGGAATCTGCCTAACcAGGAGGTGGG	2644	+1 c	3.72%
TAAGGAATCTGCCTA--AGGAGGTGGG	2117	-2	2.98%
TAAGGAATCTGCCT-ACAGGAGGTGGG	1592	-1	2.24%
TAAGGAATCTGCCTAA-AGGAGGTGGG	1459	-1	2.05%
TAAG-----------------------	910	-28	1.28%
TAAGGAATCTGCCT---AGGAGGTGGG	896	-3	1.26%
TAA------------------------	853	-24	1.20%

P201-YW-TCs-Syn25crAAVS1h-KO-M3814-BC6<br>35710 | 61.55%	Ins<br>1.98%	Del<br>59.05%	HDR<br>0.52%
**Typical seqs**	**Reads**	**Type**	**pct.**
TAAGGAATCTGCCTAAC|AGG**AGG**TGGG	13730	WT	38.45%
TA---------------AGGAGGTGGG	9656	-15	27.04%
TA------------------AGGTGGG	3359	-18	9.41%
TAAG-----------------------	452	-28	1.27%
TAAGGAATCTGCCTA--AGGAGGTGGG	419	-2	1.17%
TAA------------------------	389	-24	1.09%
TAAGGAATCT-----------GGTGGG	350	-11	0.98%
TA---------------AGGAGGGGGG	308	-15	0.86%
TAAGGAATCT--------GGAGGTGGG	252	-8	0.71%
TAAGGAATC--------------TGGG	266	-14	0.74%

P201-YW-TCs-Syn25crAAVS1h-KO-NaB-BC7<br>61389 | 55.28%	Ins<br>6.52%	Del<br>48.27%	HDR<br>0.49%
**Typical seqs**	**Reads**	**Type**	**pct.**
TAAGGAATCTGCCTAAC|AGG**AGG**TGGG	27455	WT	44.72%
TA---------------AGGAGGTGGG	8020	-15	13.06%
TA------------------AGGTGGG	3226	-18	5.26%
TAAGGAATCTGCCTAACcAGGAGGTGGG	2461	+1 c	4.01%
TAAGGAATCTGCCTA--AGGAGGTGGG	2370	-2	3.86%
TAAGGAATCTGCCTAA-AGGAGGTGGG	1786	-1	2.91%
TAAGGAATCTGCCT-ACAGGAGGTGGG	1137	-1	1.85%
TAAGGAATCTG-----CAGGAGGTGGG	674	-5	1.10%
TAAGGAATCTGCCTAAC--GAGGTGGG	632	-2	1.03%
TAAGGAATCTGC----CAGGAGGTGGG	600	-4	0.98%

P201-YW-TCs-Syn25crAAVS1h-AAV6KI

P201-YW-TCs-Syn25crAAVS1h-AAV6KI-DMSO-BC13	Ins	Del	HDR
49049 | 70.69%	**6.72%**	**26.40%**	**37.57%**
**Typical seqs**	**Reads**	**Type**	**pct.**
TAAGGAATCTGCCTAAC|AGG**AGG**TGGG	14377	WT	29.31%
TAAGGAATCTGCCTAAGTTTAAACTACGCGTG	17502	+5 HDR	35.68%
TA---------------AGGAGGTGGG	2551	-15	5.20%
TAAGGAATCTGCCTAACcAGGAGGTGGG	1927	+1 c	3.93%
TAAGGAATCTGCCTA--AGGAGGTGGG	1468	-2	2.99%
TAAGGAATCTGCCTAA-AGGAGGTGGG	1186	-1	2.42%
TA------------------AGGTGGG	740	-18	1.51%
TAAGGAATCTGCCT-ACAGGAGGTGGG	740	-1	1.51%
TAAGGAATCTG-----CAGGAGGTGGG	551	-5	1.12%
TAAGGAATCTGC----CAGGAGGTGGG	463	-4	0.94%

P201-YW-TCs-Syn25crAAVS1h-AAV6KI-CRISPYmix-BC16	Ins	Del	HDR
58158 | 74.74%	**6.64%**	**22.86%**	**45.24%**
**Typical seqs**	**Reads**	**Type**	**pct.**
TAAGGAATCTGCCTAAC|AGG**AGG**TGGG	14690	WT	25.26%
TAAGGAATCTGCCTAAGTTTAAACTACGCGTG	24961	+5 HDR	42.92%
TAAGGAATCTGCCTAACcAGGAGGTGGG	1943	+1 c	3.34%
TAAGGAATCTGCCTA--AGGAGGTGGG	1781	-2	3.06%
TA---------------AGGAGGTGGG	1804	-15	3.10%
TAAGGAATCTGCCTAA-AGGAGGTGGG	1103	-1	1.90%
TAAGGAATCTGCCT-ACAGGAGGTGGG	1100	-1	1.89%
TA------------------AGGTGGG	614	-18	1.06%
TAAGGAATCTGC----CAGGAGGTGGG	573	-4	0.99%
TAAGGAATCTG-----CAGGAGGTGGG	565	-5	0.97%

P201-YW-TCs-Syn25crAAVS1h-AAV6KI-M3814-BC14	Ins	Del	HDR
35907 | 83.35%	**4.04%**	**14.06%**	**65.25%**
**Typical seqs**	**Reads**	**Type**	**pct.**
TAAGGAATCTGCCTAAC|AGG**AGG**TGGG	5979	WT	16.65%
TAAGGAATCTGCCTAAGTTTAAACTACGCGTG	22180	+5 HDR	61.77%
TA---------------AGGAGGTGGG	1863	-15	5.19%
TA------------------AGGTGGG	714	-18	1.99%
TAAGGAATCTGCCT-CCAGGAGGTGGG	241	-1	0.67%
TAAGGAATCTGCCTAACcAGGAGGTGGG	240	+1 c	0.67%
TAAGGAATCTGCCTA--AGGAGGTGGG	232	-2	0.65%
TAAGGAATCTGCCTAACaAGGAGGTGGG	147	+1 a	0.41%
TAAGGAATCTGCCTA--AGGTTAAACTACGCGTG	122	+5	0.34%
TAA------------------------	122	-24	0.34%

P201-YW-TCs-Syn25crAAVS1h-AAV6KI-NaB-BC15	Ins	Del	HDR
40230 | 72.40%	**7.14%**	**25.24%**	**40.01%**
**Typical seqs**	**Reads**	**Type**	**pct.**
TAAGGAATCTGCCTAAC|AGG**AGG**TGGG	11105	WT	27.60%
TAAGGAATCTGCCTAAGTTTAAACTACGCGTG	15268	+5 HDR	37.95%
TAAGGAATCTGCCTAACcAGGAGGTGGG	1630	+1 c	4.05%
TAAGGAATCTGCCTA--AGGAGGTGGG	1454	-2	3.61%
TA---------------AGGAGGTGGG	1381	-15	3.43%
TAAGGAATCTGCCTAA-AGGAGGTGGG	1044	-1	2.60%
TAAGGAATCTGCCT-ACAGGAGGTGGG	655	-1	1.63%
TA------------------AGGTGGG	505	-18	1.26%
TAAGGAATCTGC----CAGGAGGTGGG	414	-4	1.03%
TAAGGAATCTG-----CAGGAGGTGGG	414	-5	1.03%

P204-XY-TC-syn90crBCL11A-6c-RNPko

4h

6h

12h

P204-XY-TC-syn90crBCL11A-6c-RNPko-6h	Ins	Del	HDR
92353 | 3.36%	**1.97%**	**1.39%**	**0.00%**
**Typical seqs**	**Reads**	**Type**	**pct.**
CTGCCTACCGGCACGCA|TGC**AGG**ATCA	89246	WT	96.64%
CTGCCTACCGGCACGCAaTGCAGGATCA	859	+1 a	0.93%
CTGCCTACCGGCACGCAtTGCAGGATCA	615	+1 t	0.67%
CTGCCTACCGGCAC----GCAGGATCA	476	-4	0.52%
CTGCCTACCG--------GCAGGATCA	219	-8	0.24%
CTGCCTACCGGCACGCAgTGCAGGATCA	86	+1 g	0.09%
CTGCCTACCGGCACGCAcTGCAGGATCA	63	+1 c	0.07%
CTGCCTACCGGCACGCA--CAGGATCA	44	-2	0.05%
CTGCCTACCG----GCATGCAGGATCA	40	-4	0.04%
C----------------TGCAGGATCA	38	-16	0.04%

24h

P205-XY-TC-syn90crBCL11A-6c-RNPko-24h	Ins	Del	HDR
79514 | 20.31%	**6.52%**	**13.80%**	**0.00%**
**Typical seqs**	**Reads**	**Type**	**pct.**
CTGCCTACCGGCACGCA|TGC**AGG**ATCA	63362	WT	79.69%
CTGCCTACCGGCAC----GCAGGATCA	3650	-4	4.59%
CTGCCTACCGGCACGCAaTGCAGGATCA	2328	+1 a	2.93%
CTGCCTACCG--------GCAGGATCA	2143	-8	2.70%
CTGCCTACCGGCACGCAtTGCAGGATCA	1740	+1 t	2.19%
CTGCCTACCG----GCATGCAGGATCA	518	-4	0.65%
CTGCCTACC------------GGATCA	394	-12	0.50%
CTGCCTACCGGCACGCAgTGCAGGATCA	271	+1 g	0.34%
C----------------TGCAGGATCA	217	-16	0.27%
CTGCCTACCGGCAC--------GATCA	178	-8	0.22%

48h

P206-XY-TC-syn90crBCL11A-6c-RNPko-48h	Ins	Del	HDR
83808 | 25.92%	**8.15%**	**17.77%**	**0.00%**
**Typical seqs**	**Reads**	**Type**	**pct.**
CTGCCTACCGGCACGCA|TGC**AGG**ATCA	62085	WT	74.08%
CTGCCTACCGGCAC----GCAGGATCA	4895	-4	5.84%
CTGCCTACCGGCACGCAaTGCAGGATCA	3131	+1 a	3.74%
CTGCCTACCG--------GCAGGATCA	3018	-8	3.60%
CTGCCTACCGGCACGCAtTGCAGGATCA	2183	+1 t	2.60%
CTGCCTACCG----GCATGCAGGATCA	651	-4	0.78%
CTGCCTACC------------GGATCA	504	-12	0.60%
CTGCCTACCGGCACGCAgTGCAGGATCA	383	+1 g	0.46%
C----------------TGCAGGATCA	273	-16	0.33%
CTGCCTACCGGCA-----GCAGGATCA	235	-5	0.28%

P205-XY-TC-syn95crBCL11A-8a-RNPko

4h

6h

P204-XY-TC-syn95crBCL11A-8a-RNPko-6h	Ins	Del	HDR
114325 | 1.88%	**0.51%**	**1.37%**	**0.00%**
**Typical seqs**	**Reads**	**Type**	**pct.**
GCTTCTGAGCGTCAAAC|TCG**AGG**AGCA	112173	WT	98.12%
GCTTCTGAGCGTCAAACcTCGAGGAGCA	248	+1 c	0.22%
GCTTCTGAGCGTCAAACtTCGAGGAGCA	224	+1 t	0.20%
GCTTCTGAGCG------TCGAGGAGCA	205	-6	0.18%
GCTTCT----------------GAGCA	136	-16	0.12%
GCTTCTGAGCGTCAAA-TCGAGGAGCA	84	-1	0.07%
GCTTCTGAGCGTCAAA--CGAGGAGCA	76	-2	0.07%
GCTTCTGAGCGTCAAAC-CGAGGAGCA	59	-1	0.05%
GCTTCTGAGCGTC-AACTCGAGGAGCA	58	-1	0.05%
GCTTCT-------------GAGGAGCA	56	-13	0.05%

12h

24h

P205-XY-TC-syn95crBCL11A-8a-RNPko-24h	Ins	Del	HDR
100239 | 9.13%	**1.66%**	**7.47%**	**0.00%**
**Typical seqs**	**Reads**	**Type**	**pct.**
GCTTCTGAGCGTCAAAC|TCG**AGG**AGCA	91089	WT	90.87%
GCTTCT----------------GAGCA	1262	-16	1.26%
GCTTCTGAGCG------TCGAGGAGCA	869	-6	0.87%
GCTTCTGAGCGTCAAACcTCGAGGAGCA	696	+1 c	0.69%
GCTTCTGAGCGTCAAACtTCGAGGAGCA	455	+1 t	0.45%
GCTTCTG-------------------A	337	-19	0.34%
GCTTCTGAG---------CGAGGAGCA	296	-9	0.30%
GCT--------------TCGAGGAGCA	291	-14	0.29%
GCTTCT-------------GAGGAGCA	259	-13	0.26%
GCTTCTGAGCGTCAA--TCGAGGAGCA	218	-2	0.22%

48h

P206-XY-TC-syn95crBCL11A-8a-RNPko-48h	Ins	Del	HDR
130475 | 11.51%	**2.20%**	**9.31%**	**0.00%**
**Typical seqs**	**Reads**	**Type**	**pct.**
GCTTCTGAGCGTCAAAC|TCG**AGG**AGCA	115460	WT	88.49%
GCTTCT----------------GAGCA	2042	-16	1.57%
GCTTCTGAGCG------TCGAGGAGCA	1508	-6	1.16%
GCTTCTGAGCGTCAAACcTCGAGGAGCA	1274	+1 c	0.98%
GCTTCTGAGCGTCAAACtTCGAGGAGCA	702	+1 t	0.54%
GCTTCT-------------GAGGAGCA	466	-13	0.36%
GCTTCTG-------------------A	462	-19	0.35%
GCTTCTGAG---------CGAGGAGCA	411	-9	0.32%
GCT--------------TCGAGGAGCA	384	-14	0.29%
GCTTCTGAGCGTCAA--TCGAGGAGCA	353	-2	0.27%

P206-XY-TC-syn97crBCL11A-8c-RNPko

4h

12h

P204-XY-TC-syn97crBCL11A-8c-RNPko-6h (6h) 40320 | 1.01%	Ins 0.11%	Del 0.90%	HDR 0.00%
**Typical seqs**	**Reads**	**Type**	**pct.**
CGCGGCAGAAACCTGTC|GTC**TGG**ACAC	39914	WT	98.99%
CGCGGCAGAAACCT---GTCTGGACAC	139	-3	0.34%
CGCGGCAGAAAC-------CTGGACAC	60	-7	0.15%
CGCGGCAGAAACCTGT-GTCTGGACAC	21	-1	0.05%
CGCGGCAGAAACCTGTCcGTCTGGACAC	21	+1 c	0.05%
CGCGGCAGAAA-----CGTCTGGACAC	10	-5	0.02%
CGCGGCAGAAAC--------------C	9	-14	0.02%
CGCGG-------------------CAC	9	-19	0.02%
CGCGGCAGAAACCT--------GACAC	8	-8	0.02%
CGCGGCAGAAACCT-------------	8	-15	0.02%

P205-XY-TC-syn97crBCL11A-8c-RNPko-24h (24h) 45483 | 4.50%	Ins 0.31%	Del 4.19%	HDR 0.00%
**Typical seqs**	**Reads**	**Type**	**pct.**
CGCGGCAGAAACCTGTC|GTC**TGG**ACAC	43436	WT	95.50%
CGCGGCAGAAACCT---GTCTGGACAC	739	-3	1.62%
CGCGGCAGAAAC-------CTGGACAC	334	-7	0.73%
CGCGGCAGAAAC--------------C	97	-14	0.21%
CGCGGCAGAAACCT-------------	78	-15	0.17%
CGCGGCAGAAACCTGTCcGTCTGGACAC	39	+1 c	0.09%
CGCGGCAGAAACCTGT-GTCTGGACAC	36	-1	0.08%
CGCGGCAGAAACCTG--GTCTGGACAC	29	-2	0.06%
CGCGGCAGAAAC-------CTGGTCAC	23	-7	0.05%
CGCGGCAGAAACCTGTCAT----ACAC	22	-4	0.05%

P206-XY-TC-syn97crBCL11A-8c-RNPko-48h (48h) 40947 | 5.19%	Ins 0.29%	Del 4.90%	HDR 0.00%
**Typical seqs**	**Reads**	**Type**	**pct.**
CGCGGCAGAAACCTGTC|GTC**TGG**ACAC	38821	WT	94.81 %
CGCGGCAGAAACCT---GTCTGGACAC	815	-3	1.99%
CGCGGCAGAAAC-------CTGGACAC	377	-7	0.92%
CGCGGCAGAAAC--------------C	85	-14	0.21%
CGCGGCAGAAACCT-------------	74	-15	0.18%
CGCGGCAGAAACCTGTCcGTCTGGACAC	61	+1 c	0.15%
CGCGGCA---------------GACAC	30	-15	0.07%
CGCGGCAGAAACCTGT-GTCTGGACAC	27	-1	0.07%
CGCGGCAGAA-------------ACAC	27	-13	0.07%
CGCGG----------------------	26	-26	0.06%

P204-XY-TC-syn98crBCL11A-8d-RNPko

4h

12h

6h

P204-XY-TC-syn98crBCL11A-8d-RNPko-6h	Ins	Del	HDR
56594 | 1.79%	**0.44%**	**1.35%**	**0.00%**
**Typical seqs**	**Reads**	**Type**	**pct.**
GCCTTGATTACTCAAAC|AAC**AGG**TTAC	55579	WT	98.21%
GCCTTGATTACTCA---AACAGGTTAC	179	-3	0.32%
GCCTTGATTACTCAAACcAACAGGTTAC	159	+1 c	0.28%
GCCTTGATTACT----CAACAGGTTAC	88	-4	0.16%
GCCTTGA----------------TTAC	59	-16	0.10%
GCCTTGATTACTCAA--AACAGGTTAC	45	-2	0.08%
GCCTTGATT---------ACAGGTTAC	37	-9	0.07%
GCCTTGATTACTCA------AGGTTAC	26	-6	0.05%
GCCTTGATTACTC-AACAACAGGTTAC	24	-1	0.04%
GCCTTGATTACTCAAAC--CAGGTTAC	21	-2	0.04%

24h

P205-XY-TC-syn98crBCL11A-8d-RNPko-24h	Ins	Del	HDR
78224 | 8.60%	**1.48%**	**7.12%**	**0.00%**
**Typical seqs**	**Reads**	**Type**	**pct.**
GCCTTGATTACTCAAAC|AAC**AGG**TTAC	71496	WT	91.40%
GCCTTGATTACTCA---AACAGGTTAC	1237	-3	1.58%
GCCTTGA----------------TTAC	913	-16	1.17%
GCCTTGATTACTCAAACcAACAGGTTAC	705	+1 c	0.90%
GCCTTGATT---------ACAGGTTAC	331	-9	0.42%
GCCTTGATTACT----CAACAGGTTAC	286	-4	0.37%
GCCTTGATTACT-------CAGGTTAC	204	-7	0.26%
GCCTTGATTACTCA------AGGTTAC	189	-6	0.24%
GCCTTGATTACTCAA--AACAGGTTAC	153	-2	0.20%
GCCTTGATTACTCA-------------	121	-14	0.15%

48h

P206-XY-TC-syn98crBCL11A-8d-RNPko-48h	Ins	Del	HDR
80576 | 11.27%	**1.86%**	**9.41%**	**0.00%**
**Typical seqs**	**Reads**	**Type**	**pct.**
GCCTTGATTACTCAAAC|AAC**AGG**TTAC	71495	WT	88.73%
GCCTTGATTACTCA---AACAGGTTAC	2034	-3	2.52%
GCCTTGA----------------TTAC	1406	-16	1.74%
GCCTTGATTACTCAAACcAACAGGTTAC	868	+1 c	1.08%
GCCTTGATT---------ACAGGTTAC	390	-9	0.48%
GCCTTGATTACT----CAACAGGTTAC	383	-4	0.48%
GCCTTGATTACT-------CAGGTTAC	282	-7	0.35%
GCCTTGATTACTCA------AGGTTAC	225	-6	0.28%
GCCTTGATTACTCAA--AACAGGTTAC	148	-2	0.18%
GCCTTGATTACTC--------------	209	-16	0.26%

P206-XY-TC-syn106crBCL11A-10a-RNPko

4h

6h

P204-XY-TC-syn106crBCL11A-10a-RNPko-6h	Ins	Del	HDR
53096 | 1.07%	**0.02%**	**1.04%**	**0.00%**
**Typical seqs**	**Reads**	**Type**	**pct.**
GCGCTAAGTCACTGTCG|TCG**AGG**CCCC	52530	WT	98.93%
GCGCTAAGTCACTGTCG--GAGGCCCC	390	-2	0.73%
GCGCTAAGTCACT---GTCGAGGCCCC	103	-3	0.19%
GCGCTAAGTCACTGTC-----AAACCC	10	-5	0.02%
GCGCTA--------------AGGCCCC	7	-14	0.01%
GCGCTAA---------GTCGAGGCCCC	6	-9	0.01%
GCGCTAAGTCAC-----TCGAGGCCCC	5	-5	0.01%
GCGCTAAGTCACTGT--------CCCC	5	-8	0.01%
GCGCTAAGTCACTGTCGgTCGAGGCCCC	5	+1 g	0.01%
GCGCTAAGTCACTGTCGaTCGAGGCCCC	4	+1 a	0.01%

12h

24h

P205-XY-TC-syn106crBCL11A-10a-RNPko-24h	Ins	Del	HDR
66460 | 3.61%	**0.29%**	**3.32%**	**0.00%**
**Typical seqs**	**Reads**	**Type**	**pct.**
GCGCTAAGTCACTGTCG|TCG**AGG**CCCC	64061	WT	96.39%
GCGCTAAGTCACT---GTCGAGGCCCC	1231	-3	1.85%
GCGCTAAGTCA---------------C	75	-15	0.11%
GCGCTAA---------GTCGAGGCCCC	67	-9	0.10%
GCGCTAAGTCACTGTCGgTCGAGGCCCC	56	+1 g	0.08%
GCGCTA--------------AGGCCCC	55	-14	0.08%
GCGCTAAGTCACTGGCTTAGCGCTAAGGGG CTAAGTATCAGCCCC	35	+18	0.05%
GCGCTAAGTCA-----------------	35	-21	0.05%
GCGCTAAGTCACTGTC-TCGAGGCCCC	33	-1	0.05%
GCGCTAA---------------GCCCC	33	-15	0.05%

48h

P206-XY-TC-syn106crBCL11A-10a-RNPko-48h	Ins	Del	HDR
59750 | 4.78%	**0.27%**	**4.52%**	**0.00%**
**Typical seqs**	**Reads**	**Type**	**pct.**
GCGCTAAGTCACTGTCG|TCG**AGG**CCCC	56892	WT	95.22%
GCGCTAAGTCACT---GTCGAGGCCCC	899	-3	1.50%
GCGCTAAGTCACTGTCGCT----GCCCC	551	-3	0.92%
GCGCTAAGTCA---------------C	413	-15	0.69%
GCGCTAA---------------GCCCC	90	-15	0.15%
GCGCTAAGTCAC---------AGCCCC	58	-9	0.10%
GCGCTAAGTCACTGTCGgTCGAGGCCCC	54	+1 g	0.09%
GCGCTAA---------GTCGAGGCCCC	48	-9	0.08%
GCGCTA--------------AGGCCCC	34	-14	0.06%
GCGCTAAGTCA----------------	32	-21	0.05%

P204-XY-TC-syn35crBCL11Gata-a4-RNPko

P204-XY-TC-syn35crBCL11Gata-a4-RNPko-4h-BC1 4h	Ins	Del	HDR
14553 | 2.84%	2.31%	0.54%	0.00%
**Typical seqs**	**Reads**	**Type**	**pct.**
TTTTATCACAGGCTCCA|GGA**AGG**GTTT	14139	WT	97.16%
TTTTATCACAGGCTCCAaGGAAGGGTTT	281	+1 a	1.93%
TTTTATCA-------CAGGAAGGGTTT	25	-7	0.17%
TTTTATCACAGGCTCCAaGGAAGGGTTG	34	+1 a	0.23%
TTTTATCAC-----------AGGGTTT	12	-11	0.08%
TTTTATCACAGGCTCC-GGAAGGGTTT	8	-1	0.05%
TTTTATCACAGGCTCCAaGGAAGGTTTT	8	+1 a	0.05%
TTTTATCACAGG----AGGAAGGGTTT	7	-4	0.05%
TTTTATCACAGGCTCCACAGGAAGGGTTT	6	+2	0.04%
TTTTATCACAGGCTCCA----------	5	-13	0.03%

P204-XY-TC-syn35crBCL11Gata-a4-RNPko-8h-BC3 6h	Ins	Del	HDR
66939 | 6.80%	3.59%	3.20%	0.00%
**Typical seqs**	**Reads**	**Type**	**pct.**
TTTTATCACAGGCTCCA|GGA**AGG**GTTT	62390	WT	93.20%
TTTTATCACAGGCTCCAaGGAAGGGTTT	1941	+1 a	2.90%
TTTTATCAC-----------AGGGTTT	487	-11	0.73%
TTTTATCA-------CAGGAAGGGTTT	347	-7	0.52%
TTTTATCACA-----------------	276	-17	0.41%
TTTTATCACAGGCTCCAaGGAAGGGTTG	239	+1 a	0.36%
TTTTATCACAGG---------------	153	-18	0.23%
TTTTATCACA------------GGTTT	75	-12	0.11%
TTTTATCACAGGCTCC-GGAAGGGTTT	74	-1	0.11%
TTTTATCACAGGCTC--GGAAGGGTTT	67	-2	0.10%

P204-XY-TC-syn35crBCL11Gata-a4-RNPko-12h-BC5 12h	Ins	Del	HDR
70590 | 8.40%	3.59%	4.81%	0.00%
**Typical seqs**	**Reads**	**Type**	**pct.**
TTTTATCACAGGCTCCA|GGA**AGG**GTTT	64660	WT	91.60%
TTTTATCACAGGCTCCAaGGAAGGGTTT	1994	+1 a	2.82%
TTTTATCAC-----------AGGGTTT	763	-11	1.08%
TTTTATCA-------CAGGAAGGGTTT	649	-7	0.92%
TTTTATCACA-----------------	392	-17	0.56%
TTTTATCACAGG---------------	238	-18	0.34%
TTTTATCACAGGCTCCAaGGAAGGGTTG	276	+1 a	0.39%
TTTTATCACAGGCTCC-GGAAGGGTTT	98	-1	0.14%
TTTTATCACA------------GGTTT	112	-12	0.16%
TTTTATCACAGGC-----------TTT	107	-11	0.15%

P204-XY-TC-syn35crBCL11Gata-a4-RNPko-24h-BC7 24h	Ins	Del	HDR
79108 | 19.97%	6.69%	13.28%	0.00%
**Typical seqs**	**Reads**	**Type**	**pct.**
TTTTATCACAGGCTCCA|GGA**AGG**GTTT	63312	WT	80.03%
TTTTATCACAGGCTCCAaGGAAGGGTTT	4138	+1 a	5.23%
TTTTATCAC-----------AGGGTTT	2338	-11	2.96%
TTTTATCA-------CAGGAAGGGTTT	1908	-7	2.41%
TTTTATCACA-----------------	1541	-17	1.95%
TTTTATCACAGG---------------	727	-18	0.92%
TTTTATCACA------------GGTTT	454	-12	0.57%
TTTTATCACAGGCTCCAaGGAAGGGTTG	481	+1 a	0.61%
TTTTATCACAGGC-----------TTT	227	-11	0.29%
TTTTATCACAG---------AGGGTTT	184	-9	0.23%

P204-XY-TC-syn35crBCL11Gata-a4-RNPko-48h-BC16 48h	Ins	Del	HDR
32397 | 26.47%	9.22%	17.24%	0.00%
**Typical seqs**	**Reads**	**Type**	**pct.**
TTTTATCACAGGCTCCA|GGA**AGG**GTTT	23823	WT	73.53%
TTTTATCACAGGCTCCAaGGAAGGGTTT	2284	+1 a	7.05%
TTTTATCAC-----------AGGGTTT	1291	-11	3.98%
TTTTATCA-------CAGGAAGGGTTT	1100	-7	3.40%
TTTTATCACA-----------------	834	-17	2.57%
TTTTATCACAGG---------------	302	-18	0.93%
TTTTATCACA------------GGTTT	251	-12	0.77%
TTTTATCACAGGCTCCAaGGAAGGGTTG	296	+1 a	0.91%
TTTTATCACAGGC-----------TTT	143	-11	0.44%
TTTTATCACAG---------AGGGTTT	104	-9	0.32%

P204-XY-TC-syn35crBCL11Gata-a4-AAV6KI

P204-XY-TC-syn35crBCL11Gata-a4-AAV6KI-4h-BC9 4h	Ins	Del	HDR
81435 | 5.47%	3.70%	1.05%	0.71%
**Typical seqs**	**Reads**	**Type**	**pct.**
TTTTATCACAGGCTCCA|GGA**AGG**GTTT	76982	WT	94.53%
TTTTATCACAGGCTCCAaGGAAGGGTTT	2453	+1 a	3.01%
TTTTATCACAGGTTTAAACT-----ACGCGT--	562	-5 HDR	0.69%
TTTTATCACAGGCTCCAaGGAAGGGTTG	302	+1 a	0.37%
TTTTATCACAGGCTCC-GGAAGGGTTT	65	-1	0.08%
TTTTATCACAGGCTCCAaGGAAGGTTTT	60	+1 a	0.07%
TTTTATCACAGG---CAGGAAGGGTTT	43	-3	0.05%
TTTTATCACA-----------------	43	-17	0.05%
TTTTATCACAGGCTCCA-GAAGGGTTT	43	-1	0.05%
TTTTATCA-------CAGGAAGGGTTT	48	-7	0.06%

P204-XY-TC-syn35crBCL11Gata-a4-AAV6KI-8h-BC12 6h	Ins	Del	HDR
86290 | 10.57%	5.37%	2.60%	2.59%
**Typical seqs**	**Reads**	**Type**	**pct.**
TTTTATCACAGGCTCCA|GGA**AGG**GTTT	77168	WT	89.43%
TTTTATCACAGGCTCCAaGGAAGGGTTT	3673	+1 a	4.26%
TTTTATCACAGGTTTAAACT-----ACGCGT--	2141	-5 HDR	2.48%
TTTTATCACAGGCTCCAaGGAAGGGTTG	548	+1 a	0.64%
TTTTATCAC-----------AGGGTTT	261	-11	0.30%
TTTTATCACA-----------------	191	-17	0.22%
TTTTATCA-------CAGGAAGGGTTT	208	-7	0.24%
TTTTATCACAGGCTCC-GGAAGGGTTT	157	-1	0.18%
TTTTATCACAGG---------------	116	-18	0.13%
TTTTATCACAGGTTTAAACTACGCTT-	121	-5	0.14%

P204-XY-TC-syn35crBCL11Gata-a4-AAV6KI-12h-BC14 12h	Ins	Del	HDR
86596 | 15.16%	6.41%	4.79%	3.96%
**Typical seqs**	**Reads**	**Type**	**pct.**
TTTTATCACAGGCTCCA|GGA**AGG**GTTT	73470	WT	84.84%
TTTTATCACAGGCTCCAaGGAAGGGTTT	4314	+1 a	4.98%
TTTTATCACAGGTTTAAACT-----ACGCGT--	3263	-5 HDR	3.77%
TTTTATCAC-----------AGGGTTT	576	-11	0.67%
TTTTATCA-------CAGGAAGGGTTT	571	-7	0.66%
TTTTATCACAGGCTCCAaGGAAGGGTTG	613	+1 a	0.71%
TTTTATCACA-----------------	361	-17	0.42%
TTTTATCACAGG---------------	250	-18	0.29%
TTTTATCACAGGCTCC-GGAAGGGTTT	201	-1	0.23%
TTTTATCACA------------GGTTT	214	-12	0.25%

P205-XY-TC-syn35crBCL11Gata-a4-AAV6KI-24h-BC18 24h	Ins	Del	HDR
23266 | 24.26%	8.67%	8.83%	6.76%
**Typical seqs**	**Reads**	**Type**	**pct.**
TTTTATCACAGGCTCCA|GGA**AGG**GTTT	17621	WT	75.74%
TTTTATCACAGGCTCCAaGGAAGGGTTT	1621	+1 a	6.97%
TTTTATCACAGGTTTAAACT-----ACGCGT--	1501	-5 HDR	6.45%
TTTTATCAC-----------AGGGTTT	425	-11	1.83%
TTTTATCA-------CAGGAAGGGTTT	405	-7	1.74%
TTTTATCACA-----------------	254	-17	1.09%
TTTTATCACAGGCTCCAaGGAAGGGTTG	221	+1 a	0.95%
TTTTATCACAGG---------------	114	-18	0.49%
TTTTATCACA------------GGTTT	83	-12	0.36%
TTTTATCACAGGCTCC-GGAAGGGTTT	64	-1	0.28%

P204-XY-TC-syn35crBCL11Gata-a4-AAV6KI-48h-BC18 48h	Ins	Del	HDR
14035 | 29.21%	10.38%	10.27%	8.56%
**Typical seqs**	**Reads**	**Type**	**pct.**
TTTTATCACAGGCTCCA|GGA**AGG**GTTT	9935	WT	70.79%
TTTTATCACAGGCTCCAaGGAAGGGTTT	1186	+1 a	8.45%
TTTTATCACAGGTTTAAACT-----ACGCGT--	1160	-5 HDR	8.27%
TTTTATCAC-----------AGGGTTT	267	-11	1.90%
TTTTATCA-------CAGGAAGGGTTT	283	-7	2.02%
TTTTATCACA-----------------	151	-17	1.08%
TTTTATCACAGGCTCCAaGGAAGGGTTG	144	+1 a	1.03%
TTTTATCACAGG---------------	105	-18	0.75%
TTTTATCACA------------GGTTT	58	-12	0.41%
TTTTATCACAGGTTTAAACTACGCTT-	52	-5	0.37%

P204-XY-TC-syn37crBCL11Gata-a6-RNPko

P204-XY-TC-syn37crBCL11Gata-a6-RNPko-4h-BC2 4h	Ins	Del	HDR
90739 | 0.91%	**0.41%**	**0.51%**	**0.00%**
**Typical seqs**	**Reads**	**Type**	**pct.**
CTAACAGTTGCTTTTAT|CAC**AGG**CTCC	89911	WT	99.09%
CTAACAGTTGCTTTTATtCACAGGCTCC	332	+1 t	0.37%
CTA---------------ACAGGCTCC	85	-15	0.09%
CTAACAGTT-------------GCTCC	62	-13	0.07%
CTAACAGTTG-------CACAGGCTCC	42	-7	0.05%
CTAACAGTTGCTTT--TCACAGGCTCC	31	-2	0.03%
CTAACAGTTGCTTTTAT--CAGGCTCC	23	-2	0.03%
CTAACAGTTGCTTTTA-CACAGGCTCC	20	-1	0.02%
CTAACAGTTGC-TTTATCACAGGCTCC	16	-1	0.02%
CTAA-------------CACAGGCTCC	16	-13	0.02%

P204-XY-TC-syn37crBCL11Gata-a6-RNPko-8h-BC4 6h	Ins	Del	HDR
71488 | 2.50%	**0.09%**	**2.41%**	**0.00%**
**Typical seqs**	**Reads**	**Type**	**pct.**
CTAACAGTTGCTTTTAT|CAC**AGG**CTCC	69698	WT	97.50%
CTA---------------ACAGGCTCC	464	-15	0.65%
CTAACAGTTGCTTTTAT-----GCTCC	384	-5	0.54%
CTAACAGTTGCTTTTAT---AGGCTCC	378	-3	0.53%
CTAACAGTTGCT----TCACAGGCTCC	320	-4	0.45%
CTAACAGTT-------------GCTCC	79	-13	0.11%
CTAACAGTTGCTTTTATtCACAGGCTCC	60	+1 t	0.08%
CTAACAGTTGC-TTTATCACAGGCTCC	12	-1	0.02%
CTAACAGTTG-------CACAGGCTCC	9	-7	0.01%
CTAACAGTTGCTTT--TCACAGGCTCC	7	-2	0.01%

P204-XY-TC-syn37crBCL11Gata-a6-RNPko-12h-BC6 12h	Ins	Del	HDR
80402 | 5.30%	**0.80%**	**4.51%**	**0.00%**
**Typical seqs**	**Reads**	**Type**	**pct.**
CTAACAGTTGCTTTTAT|CAC**AGG**CTCC	76137	WT	94.70%
CTA---------------ACAGGCTCC	1189	-15	1.48%
CTAACAGTT-------------GCTCC	839	-13	1.04%
CTAACAGTTGCTTTTATtCACAGGCTCC	566	+1 t	0.70%
CTAA-------------CACAGGCTCC	109	-13	0.14%
CTAACAGTTG-------CACAGGCTCC	94	-7	0.12%
CTAACAGTTGCTTT--TCACAGGCTCC	75	-2	0.09%
CTAA----------------------C	68	-22	0.08%
----------------------GCTCC	57	-23	0.07%
CTAACAGTTG---------CAGGCTCC	52	-9	0.06%

P204-XY-TC-syn37crBCL11Gata-a6-RNPko-24h-BC8 24h	Ins	Del	HDR
70616 | 10.74%	**1.42%**	**9.32%**	**0.00%**
**Typical seqs**	**Reads**	**Type**	**pct.**
CTAACAGTTGCTTTTAT|CAC**AGG**CTCC	63034	WT	89.26%
CTA---------------ACAGGCTCC	2154	-15	3.05%
CTAACAGTT-------------GCTCC	1506	-13	2.13%
CTAACAGTTGCTTTTATtCACAGGCTCC	828	+1 t	1.17%
CTAACAGTTG-------CACAGGCTCC	265	-7	0.38%
CTAA-------------CACAGGCTCC	168	-13	0.24%
----------------------GCTCC	140	-23	0.20%
CTAA----------------------C	136	-22	0.19%
-------------------CAGGCTCC	99	-23	0.14%
CTAACAGTTG---------CAGGCTCC	81	-9	0.11%

P204-XY-TC-syn37crBCL11Gata-a6-RNPko-48h-BC17 48h	Ins	Del	HDR
30286 | 16.25%	**2.19%**	**14.07%**	**0.00%**
**Typical seqs**	**Reads**	**Type**	**pct.**
CTAACAGTTGCTTTTAT|CAC**AGG**CTCC	25364	WT	83.75%
CTA---------------ACAGGCTCC	1388	-15	4.58%
CTAACAGTT-------------GCTCC	1011	-13	3.34%
CTAACAGTTGCTTTTATtCACAGGCTCC	581	+1 t	1.92%
CTAA-------------CACAGGCTCC	169	-13	0.56%
CTAACAGTTG-------CACAGGCTCC	132	-7	0.44%
CTAA----------------------C	99	-22	0.33%
----------------------GCTCC	88	-23	0.29%
CTAACAGTTGCTTT--TCACAGGCTCC	97	-2	0.32%
-------------------CAGGCTCC	70	-23	0.23%

P206-XY-TC-syn84crBCL11A-4c-KO

P206-XY-TC-syn84crBCL11A-4c-KO-DMSO-BC1	Ins	Del	HDR	P206-XY-TC-syn84crBCL11A-4c-KO-M3814-BC2	Ins	Del	HDR	P206-XY-TC-syn84crBCL11A-4c-KO-M3814+NaB-BC5	Ins	Del	HDR
7001 | 80.69%	**1.64%**	**79.05%**	**0.00%**	34943 | 82.41%	**3.04%**	**79.37%**	**0.00%**	21327 | 73.17%	**2.71%**	**70.46%**	**0.00%**
**Typical seqs**	**Reads**	**Type**	**pct.**	**Typical seqs**	**Reads**	**Type**	**pct.**	**Typical seqs**	**Reads**	**Type**	**pct.**
GTAGGCGACCAACATGG|GGG**GGG**GGGG	1352	WT	19.31%	GTAGGCGACCAACATGG|GGG**GGG**GGGG	6146	WT	17.59%	GTAGGCGACCAACATGG|GGG**GGG**GGGG	5722	WT	26.83%
GTAGGCGACCAACAT--GGTGGGGGTG	1110	-2	15.85%	GTAGGCGACCAACA-----TGGGGGTG	4693	-5	13.43%	GTAGGCGACCAACA-----TGGGGGTG	2427	-5	11.38%
GTAGGCGACCAACA-----TGGGGGTG	615	-5	8.78%	GTAGGCGACCAACAT--GGTGGGGGTG	4087	-2	11.70%	GTAGGCGACCAACAT--GGTGGGGGTG	2100	-2	9.85%
GTAGGCGACCAACA-----------TG	242	-11	3.46%	GTAGGCGACCAACA-----------TG	1706	-11	4.88%	GTAGGCGACCAACA-----------TG	1031	-11	4.83%
GTAGGC----------------------	205	-24	2.93%	GTAGGCGACCAACAT--------GGTG	1348	-8	3.86%	GTAGGCGACCAACAT-------GGGTG	637	-7	2.99%
GTAGGCGACCAACAT--------GGTG	164	-8	2.34%	GTAGGC----------------------	1440	-24	4.12%	GTAGGC----------------------	543	-24	2.55%
GTAGGCGACCAACAT-------GGGTG	152	-7	2.17%	GTAGGCGACCAACA-------------	1106	-19	3.17%	GTAGGCGACCAACAT--------GGTG	458	-8	2.15%
GTA-----------------------G	123	-23	1.76%	GTAGGCGACCAACAT-------GGGTG	729	-7	2.09%	GTAGGCGACCAACAT---GTGGGGGTG	381	-3	1.79%
GTA--------------GGTGGGGGTG	123	-14	1.76%	GTA--------------------GGTG	609	-20	1.74%	GTA--------------GGTGGGGGTG	354	-14	1.66%
GTA------------------GGGTG	119	-19	1.70%	GTAGGCGACCAACAT---GTGGGGGTG	573	-3	1.64%	GTAGGCGACCAACAT------------	625	-13	2.93%

P206-XY-TC-syn84crBCL11A-4c-KO-M3814+TSA-BC4	Ins	Del	HDR	P206-XY-TC-syn84crBCL11A-4c-KO-Nu7026+TSA-BC3	Ins	Del	HDR
30370 | 83.94%	**3.14%**	**80.80%**	**0.00%**	17973 | 85.57%	**3.71%**	**81.86%**	**0.00%**
**Typical seqs**	**Reads**	**Type**	**pct.**	**Typical seqs**	**Reads**	**Type**	**pct.**
GTAGGCGACCAACATGG|GGG**GGG**GGGG	4877	WT	16.06%	GTAGGCGACCAACATGG|GGG**GGG**GGGG	2594	WT	14.43%
GTAGGCGACCAACA-----TGGGGGTG	3403	-5	11.21%	GTAGGCGACCAACAT--GGTGGGGGTG	2711	-2	15.08%
GTAGGCGACCAACAT--GGTGGGGGTG	3303	-2	10.88%	GTAGGCGACCAACA-----TGGGGGTG	1716	-5	9.55%
GTAGGCGACCAACA-----------TG	1489	-11	4.90%	GTAGGCGACCAACA-----------TG	725	-11	4.03%
GTAGGC----------------------	1517	-24	5.00%	GTAGGCGACCAACAT--------GGTG	714	-8	3.97%
GTAGGCGACCAACAT--------GGTG	1040	-8	3.42%	GTAGGC----------------------	531	-24	2.95%
GTAGGCGACCA-----------------	919	-20	3.03%	GTAGGCGACCAACAT-------GGGTG	398	-7	2.21%
GTAGGCGACCAACAT-------GGGTG	828	-7	2.73%	GTAGGCGACCAACATGGGG-GGGGGGG	318	-1	1.77%
GTAGGCGACCAACAT------------	1703	-13	5.61%	GTA--------------GGTGGGGGTG	305	-14	1.70%
GTAGGCGACCAACA-------------	830	-19	2.73%	GTAGGCGACCAAC----GGTGGGGGTG	264	-4	1.47%

P206-XY-TC-syn87crBCL11A-5b-KO

P206-XY-TC-syn87crBCL11A-5b-KO-DMSO-BC1	Ins	Del	HDR
818 | 96.70%	1.59%	95.11%	0.00%
**Typical seqs**	**Reads**	**Type**	**pct.**
GGGATTACCGAGTCACC|ACC**AGG**CTGC	27	WT	3.30%
GGGATT-----------ACCAGGCTGC	298	-11	36.43%
GGGATTACCGAGT------CAGGCTGC	211	-6	25.79%
GGGATTACCGAGTCA-CACCAGGCTGC	89	-1	10.88%
GGGATTACCGAGT---CACCAGGCTGC	80	-3	9.78%
GGGATTACCG---------CAGGCTGC	43	-9	5.26%
GGGATTAC------------------C	16	-18	1.96%
GGGATTACCGAGTC-------GG----	12	-14	1.47%
GGGATTACCGAGTCACCcACCAGGCTGC	10	+1 c	1.22%
GGGATTACC---------ACAGGCTGC	5	-9	0.61%

P206-XY-TC-syn87crBCL11A-5b-KO-M3814-BC2	Ins	Del	HDR
59925 | 84.03%	2.09%	81.93%	0.00%
**Typical seqs**	**Reads**	**Type**	**pct.**
GGGATTACCGAGTCACC|ACC**AGG**CTGC	9573	WT	15.97%
GGGATT-----------ACCAGGCTGC	18911	-11	31.56%
GGGATTACCGAGT------CAGGCTGC	10031	-6	16.74%
GGGATTACCGAGT---CACCAGGCTGC	4809	-3	8.03%
GGGATTACCGAGT-----TCAGGCTGC	918	-5	1.53%
GGGATTAC---------------CTGC	900	-15	1.50%
------------------------TGC	801	-26	1.34%
GGGATTACCGAGTCA-CACCAGGCTGC	799	-1	1.33%
GGGATTACCG----------AGGCTGC	649	-10	1.08%
GGGATTA----------------CTGC	637	-16	1.06%

P206-XY-TC-syn87crBCL11A-5b-KO-M3814+NaB-BC5	Ins	Del	HDR
34004 | 79.22%	2.05%	77.17%	0.00%
**Typical seqs**	**Reads**	**Type**	**pct.**
GGGATTACCGAGTCACC|ACC**AGG**CTGC	7067	WT	20.78%
GGGATT-----------ACCAGGCTGC	11139	-11	32.76%
GGGATTACCGAGT------CAGGCTGC	4326	-6	12.72%
GGGATTACCGAGT---CACCAGGCTGC	2463	-3	7.24%
GGGATTACCG----------AGGCTGC	682	-10	2.01%
GGGATTACCGAGTCA-CACCAGGCTGC	607	-1	1.79%
GGGATTAC------------------C	420	-18	1.24%
GGGATTAC---------------CTGC	427	-15	1.26%
------------------------TGC	388	-26	1.14%
GGGATTACCGAGT-----TCAGGCTGC	326	-5	0.96%

P206-XY-TC-syn87crBCL11A-5b-KO-M3814+TSA-BC4	Ins	Del	HDR
54656 | 82.76%	2.11%	80.65%	0.00%
**Typical seqs**	**Reads**	**Type**	**pct.**
GGGATTACCGAGTCACC|ACC**AGG**CTGC	9425	WT	17.24%
GGGATT-----------ACCAGGCTGC	18970	-11	34.71%
GGGATTACCGAGT------CAGGCTGC	7817	-6	14.30%
GGGATTACCGAGT---CACCAGGCTGC	3680	-3	6.73%
GGGATTACCGAGT-----TCAGGCTGC	795	-5	1.45%
------------------------TGC	811	-26	1.48%
GGGATTACCG----------AGGCTGC	712	-10	1.30%
GGGATTAC------------------C	687	-18	1.26%
GGGATTAC---------------CTGC	634	-15	1.16%
GGGATTACCGAGTCA-CACCAGGCTGC	561	-1	1.03%

P206-XY-TC-syn87crBCL11A-5b-KO-Nu7026+TSA-BC3	Ins	Del	HDR
39818 | 87.53%	6.22%	81.31%	0.00%
**Typical seqs**	**Reads**	**Type**	**pct.**
GGGATTACCGAGTCACC|ACC**AGG**CTGC	4965	WT	12.47%
GGGATTACCGAGTCA-CACCAGGCTGC	9726	-1	24.43%
GGGATTACCGAGT---CACCAGGCTGC	6528	-3	16.39%
GGGATT-----------ACCAGGCTGC	6131	-11	15.40%
GGGATTACCGAGT------CAGGCTGC	2317	-6	5.82%
GGGATTACCGAGTCACCcACCAGGCTGC	1752	+1 c	4.40%
GGGATTACCGAGTCAC--CCAGGCTGC	375	-2	0.94%
GGGATTACCGAG----CACCAGGCTGC	317	-4	0.80%
GGGATTACCG----------AGGCTGC	315	-10	0.79%
------------------------TGC	244	-26	0.61%

P207-XY-TC-syn87crBCL11A-5b-KI

P207-XY-TC-syn87crBCL11A-5b-KI-DMSO-BC1	Ins	Del	HDR
35971 | 90.02%	35.63%	54.39%	28.82%
**Typical seqs**	**Reads**	**Type**	**pct.**
GGGATTACCGAGTCACC|ACC**AGG**CTGC	3589	WT	9.98%
GGGATTACCGAGTCACCGTTTAAACACCAGGCTGC	10367	+8	28.82%
GGGATTACCGAGTCA-CACCAGGCTGC	9101	-1	25.30%
GGGATTACCGAGT---CACCAGGCTGC	5067	-3	14.09%
GGGATT-----------ACCAGGCTGC	1545	-11	4.30%
GGGATTACCGAGTCACCcACCAGGCTGC	1368	+1 c	3.80%
GGGATTACCGAGT------CAGGCTGC	332	-6	0.92%
GGGATTACCGAGTCAC--CCAGGCTGC	255	-2	0.71%
GGGATTACCGAGTCACCtACCAGGCTGC	207	+1 t	0.58%
GGGATTACCGAG----CACCAGGCTGC	138	-4	0.38%

P207-XY-TC-syn87crBCL11A-5b-KI-M3814-BC2	Ins	Del	HDR
115757 | 91.27%	59.14%	32.13%	52.62%
**Typical seqs**	**Reads**	**Type**	**pct.**
GGGATTACCGAGTCACC|ACC**AGG**CTGC	10103	WT	8.73%
GGGATTACCGAGTCACCGTTTAAACACCAGGCTGC	60908	+8	52.62%
GGGATTACCGAGTCA-CACCAGGCTGC	10114	-1	8.74%
GGGATT-----------ACCAGGCTGC	7249	-11	6.26%
GGGATTACCGAGT---CACCAGGCTGC	6922	-3	5.98%
GGGATTACCGAGT------CAGGCTGC	2572	-6	2.22%
GGGATTACCGAGTCACCcACCAGGCTGC	2472	+1 c	2.14%
GGGATTACCGAGT--CCACCAGGCTGC	688	-2	0.59%
GGGATTACCG-------ACCAGGCTGC	606	-7	0.52%
GGGATTACCGAGTCAC--CCAGGCTGC	457	-2	0.39%

P207-XY-TC-syn87crBCL11A-5b-KI-M3814+NaB-BC5	Ins	Del	HDR
5659 | 91.08%	63.83%	27.25%	57.54%
**Typical seqs**	**Reads**	**Type**	**pct.**
GGGATTACCGAGTCACC|ACC**AGG**CTGC	505	WT	8.92%
GGGATTACCGAGTCACCGTTTAAACACCAGGCTGC	3256	+8	57.54%
GGGATTACCGAGTCA-CACCAGGCTGC	551	-1	9.74%
GGGATTACCGAGT---CACCAGGCTGC	330	-3	5.83%
GGGATT-----------ACCAGGCTGC	283	-11	5.00%
GGGATTACCGAGT------CAGGCTGC	106	-6	1.87%
GGGATTACCGAGTCACCcACCAGGCTGC	105	+1 c	1.86%
GGGATTACCGAGTCAC--CCAGGCTGC	32	-2	0.57%
GGGATTACCGAGT--CCACCAGGCTGC	27	-2	0.48%
GGGATTACCGAGTCA--ACCAGGCTGC	24	-2	0.42%

P207-XY-TC-syn87crBCL11A-5b-KI-M3814+TSA-BC4	Ins	Del	HDR
108476 | 91.29%	56.37%	34.92%	50.00%
**Typical seqs**	**Reads**	**Type**	**pct.**
GGGATTACCGAGTCACC|ACC**AGG**CTGC	9446	WT	8.71%
GGGATTACCGAGTCACCGTTTAAACACCAGGCTGC	54242	+8	50.00%
GGGATTACCGAGTCA-CACCAGGCTGC	12514	-1	11.54%
GGGATTACCGAGT---CACCAGGCTGC	7347	-3	6.77%
GGGATT-----------ACCAGGCTGC	6134	-11	5.65%
GGGATTACCGAGT------CAGGCTGC	2769	-6	2.55%
GGGATTACCGAGTCACCcACCAGGCTGC	2394	+1 c	2.21%
GGGATTACCGAGTCAC--CCAGGCTGC	482	-2	0.44%
GGGATTACCGAGT----ACCAGGCTGC	407	-4	0.38%
GGGATTACCGAGTCA--ACCAGGCTGC	409	-2	0.38%

P207-XY-TC-syn87crBCL11A-5b-KI-Nu7026+TSA-BC3	Ins	Del	HDR
47069 | 91.50%	36.21%	55.29%	30.22%
**Typical seqs**	**Reads**	**Type**	**pct.**
GGGATTACCGAGTCACC|ACC**AGG**CTGC	4000	WT	8.50%
GGGATTACCGAGTCACCGTTTAAACACCAGGCTGC	14226	+8	30.22%
GGGATTACCGAGTCA-CACCAGGCTGC	11627	-1	24.70%
GGGATTACCGAGT---CACCAGGCTGC	7120	-3	15.13%
GGGATT-----------ACCAGGCTGC	2069	-11	4.40%
GGGATTACCGAGTCACCcACCAGGCTGC	1623	+1 c	3.45%
GGGATTACCGAGT------CAGGCTGC	589	-6	1.25%
GGGATTACCGAGTCAC--CCAGGCTGC	293	-2	0.62%
GGGATTACCGAGT--CCACCAGGCTGC	261	-2	0.55%
GGGATTACCGAG----CACCAGGCTGC	230	-4	0.49%

P206-XY-TC-syn105crBCL11A-9g-KO

P206-XY-TC-syn105crBCL11A-9g-KO-DMSO-BC1	Ins	Del	HDR	P206-XY-TC-syn105crBCL11A-9g-KO-M3814-BC2	Ins	Del	HDR	P206-XY-TC-syn105crBCL11A-9g-KO-M3814+NaB-BC5	Ins	Del	HDR
12047 | 43.35%	**31.47%**	**11.88%**	**0.00%**	67509 | 35.63%	**4.96%**	**30.66%**	**0.01%**	42779 | 32.29%	**4.91%**	**27.38%**	**0.00%**
**Typical seqs**	**Reads**	**Type**	**pct.**	**Typical seqs**	**Reads**	**Type**	**pct.**	**Typical seqs**	**Reads**	**Type**	**pct.**
CAACTTACAAATACCCT|GCG**GGG**CATA	6825	WT	56.65%	CAACTTACAAATACCCT|GCG**GGG**CATA	43453	WT	64.37%	CAACTTACAAATACCCT|GCG**GGG**CATA	28966	WT	67.71%
CAACTTACAAATACCCTtGCGGGGCATA	3526	+1 t	29.27%	CAACTTACAA--------------ATA	2963	-14	4.39%	CAACTTACAA--------------ATA	1706	-14	3.99%
CAACTTACAA--------------ATA	177	-14	1.47%	CAACTTA----------------CATA	2870	-16	4.25%	CAACTTA----------------CATA	1599	-16	3.74%
CAACTTA----------------CATA	152	-16	1.26%	CAACTTACAAATACCCTtGCGGGGCATA	1710	+1 t	2.53%	CAACTTACAAATACCCTtGCGGGGCATA	1161	+1 t	2.71%
CAACTTACAAATA---TGCGGGGCATA	101	-3	0.84%	CAACTTACAAATA---TGCGGGGCATA	1688	-3	2.50%	CAACTTACAAATA---TGCGGGGCATA	1075	-3	2.51%
CAACTTACAAATA-CCTGCGGGGCATA	61	-1	0.51%	CAACT--------------------TA	841	-20	1.25%	CAACT--------------------TA	407	-20	0.95%
CAACT--------------------TA	52	-20	0.43%	CAACTTACAAATA----------CATA	740	-10	1.10%	CAACTTA--------------------	362	-25	0.85%
CAACTTACAAATA--CTGCGGGGCATA	51	-2	0.42%	CAACTTA--------------------	664	-25	0.98%	CA-------------------------	276	-31	0.65%
CAACTTA--------------------	48	-25	0.40%	-----------------------CATA	509	-23	0.75%	CAACTTACAAATA----------CATA	222	-10	0.52%
CAACTTACAAAT----TGCGGGGCATA	38	-4	0.32%	CA-------------------------	484	-31	0.72%	-----------------------CATA	213	-23	0.50%

P206-XY-TC-syn105crBCL11A-9g-KO-M3814+TSA-BC4	Ins	Del	HDR	P206-XY-TC-syn105crBCL11A-9g-KO-Nu7026+TSA-BC3	Ins	Del	HDR
42823 | 33.96%	**4.87%**	**29.05%**	**0.04%**	39251 | 44.12%	**28.87%**	**15.24%**	**0.01%**
**Typical seqs**	**Reads**	**Type**	**pct.**	**Typical seqs**	**Reads**	**Type**	**pct.**
CAACTTACAAATACCCT|GCG**GGG**CATA	28282	WT	66.04%	CAACTTACAAATACCCT|GCG**GGG**CATA	21933	WT	55.88%
CAACTTACAA--------------ATA	1925	-14	4.50%	CAACTTACAAATACCCTtGCGGGGCATA	10348	+1 t	26.36%
CAACTTA----------------CATA	1648	-16	3.85%	CAACTTACAA--------------ATA	662	-14	1.69%
CAACTTACAAATACCCTtGCGGGGCATA	1115	+1 t	2.60%	CAACTTA----------------CATA	616	-16	1.57%
CAACTTACAAATA---TGCGGGGCATA	951	-3	2.22%	CAACTTACAAATA---TGCGGGGCATA	322	-3	0.82%
CAACT--------------------TA	450	-20	1.05%	CAACT--------------------TA	226	-20	0.58%
CA-------------------------	402	-31	0.94%	CAACTTACAAATA-CCTGCGGGGCATA	199	-1	0.51%
CAACTTACAAATA-----CGGGGCATA	323	-5	0.75%	CAACTTACAAATACCCCTGCGGGGCATA	142	+1 Ins	0.36%
CAACTTA--------------------	372	-25	0.87%	CA-------------------------	141	-31	0.36%
CAACTTACAAATA----------CATA	281	-10	0.66%	CAACTTACAAATA----------CATA	130	-10	0.33%

P207-XY-TC-syn105crBCL11A-9g-KI

P207-XY-TC-syn105crBCL11A-9g-KI-DMSO-BC1	Ins	Del	HDR
8303 | 45.04%	**31.28%**	**6.72%**	**7.05%**
**Typical seqs**	**Reads**	**Type**	**pct.**
CAACTTACAAATACCCT|GCG**GGG**CATA	4563	WT	54.96%
CAACTTACAAATACCCTtGCGGGGCATA	2467	+1 t	29.71%
CAACTTACAAATACCCTGTTTAAACGCGGG GCATA	582	+8 HDR	7.01%
CAACTTACAA--------------ATA	58	-14	0.70%
CAACTTACAAATA---TGCGGGGCATA	57	-3	0.69%
CAACTTACAAATA-CCTGCGGGGCATA	36	-1	0.43%
CAACTTA----------------CATA	30	-16	0.36%
CAACT--------------------TA	27	-20	0.33%
CAACTTACAAATACCCCTGCGGGGCATA	25	+1 Ins	0.30%
CAACTTACAAAT----TGCGGGGCATA	23	-4	0.28%

P207-XY-TC-syn105crBCL11A-9g-KI-M3814-BC2	Ins	Del	HDR
59103 | 51.38%	**11.01%**	**15.07%**	**25.30%**
**Typical seqs**	**Reads**	**Type**	**pct.**
CAACTTACAAATACCCT|GCG**GGG**CATA	28735	WT	48.62%
CAACTTACAAATACCCTGTTTAAACGCGGG GCATA	14520	+8 HDR	24.57%
CAACTTACAAATACCCTtGCGGGGCATA	5101	+1 t	8.63%
CAACTTACAA--------------ATA	1213	-14	2.05%
CAACTTA----------------CATA	1044	-16	1.77%
CAACTTACAAATA---TGCGGGGCATA	618	-3	1.05%
CAACT--------------------TA	308	-20	0.52%
CAACTTA---------------------	234	-25	0.40%
CA--------------------------	153	-31	0.26%
CAACTTA--------CTGCGGGGCATA	150	-8	0.25%

P207-XY-TC-syn105crBCL11A-9g-KI-M3814+NaB-BC5	Ins	Del	HDR
1680 | 68.51%	**7.56%**	**5.24%**	**55.71%**
**Typical seqs**	**Reads**	**Type**	**pct.**
CAACTTACAAATACCCT|GCG**GGG**CATA	529	WT	31.49%
CAACTTACAAATACCCTGTTTAAACGCGGG GCATA	925	+8 HDR	55.06%
CAACTTACAAATACCCTtGCGGGGCATA	121	+1 t	7.20%
CAACTTACAA--------------ATA	18	-14	1.07%
CAACTTA----------------CATA	14	-16	0.83%
CAACTTACAAATA---TGCGGGGCATA	10	-3	0.60%
CAACT--------------------TA	7	-20	0.42%
CAACTTA---------------------	6	-25	0.36%
CAACTTACAAATA-CCTGCGGGGCATA	5	-1	0.30%
CAACTTACAAATAC-------------	4	-15	0.24%

P207-XY-TC-syn105crBCL11A-9g-KI-M3814+TSA-BC4	Ins	Del	HDR
44385 | 51.66%	**12.38%**	**15.44%**	**23.84%**
**Typical seqs**	**Reads**	**Type**	**pct.**
CAACTTACAAATACCCT|GCG**GGG**CATA	21454	WT	48.34%
CAACTTACAAATACCCTGTTTAAACGCGGG GCATA	10198	+8 HDR	22.98%
CAACTTACAAATACCCTtGCGGGGCATA	4490	+1 t	10.12%
CAACTTA----------------CATA	923	-16	2.08%
CAACTTACAA--------------ATA	861	-14	1.94%
CAACTTACAAATA---TGCGGGGCATA	469	-3	1.06%
CAACT--------------------TA	239	-20	0.54%
CAACTTACAA----------------A	160	-16	0.36%
CAACTTACAAATA-CCTGCGGGGCATA	156	-1	0.35%
CAACTTA---------------------	205	-25	0.46%

P207-XY-TC-syn105crBCL11A-9g-KI-Nu7026+TSA-BC3	Ins	Del	HDR
30978 | 49.63%	**30.36%**	**8.89%**	**10.38%**
**Typical seqs**	**Reads**	**Type**	**pct.**
CAACTTACAAATACCCT|GCG**GGG**CATA	15604	WT	50.37%
CAACTTACAAATACCCTtGCGGGGCATA	8774	+1 t	28.32%
CAACTTACAAATACCCTGTTTAAACGCGGG GCATA	3152	+8 HDR	10.17%
CAACTTACAA--------------ATA	363	-14	1.17%
CAACTTA----------------CATA	227	-16	0.73%
CAACTTACAAATA---TGCGGGGCATA	167	-3	0.54%
CAACTTACAAATA-CCTGCGGGGCATA	139	-1	0.45%
CAACT--------------------TA	101	-20	0.33%
CAACTTACAAATACCCCTGCGGGGCATA	87	+1 Ins	0.28%
CAACTTA---------------------	92	-25	0.30%
