# Supplementary material for: Dynamics and competition of CRISPR–Cas9 ribonucleoproteins and AAV donor-mediated NHEJ, MMEJ and HDR editing
Source: Nucleic Acids Res. 2021 Jan 4;49(2):969–85. doi: 10.1093/nar/gkaa1251 (PMC7826255; doi:10.1093/nar/gkaa1251)
Supplement: gkaa1251_Supplemental_Files [file gkaa1251_supplemental_files.zip › NAR_RNP-AAV_editing_dynamics_-_Supplementary_Figures_12-10-2020.pptx]

## Slide 1
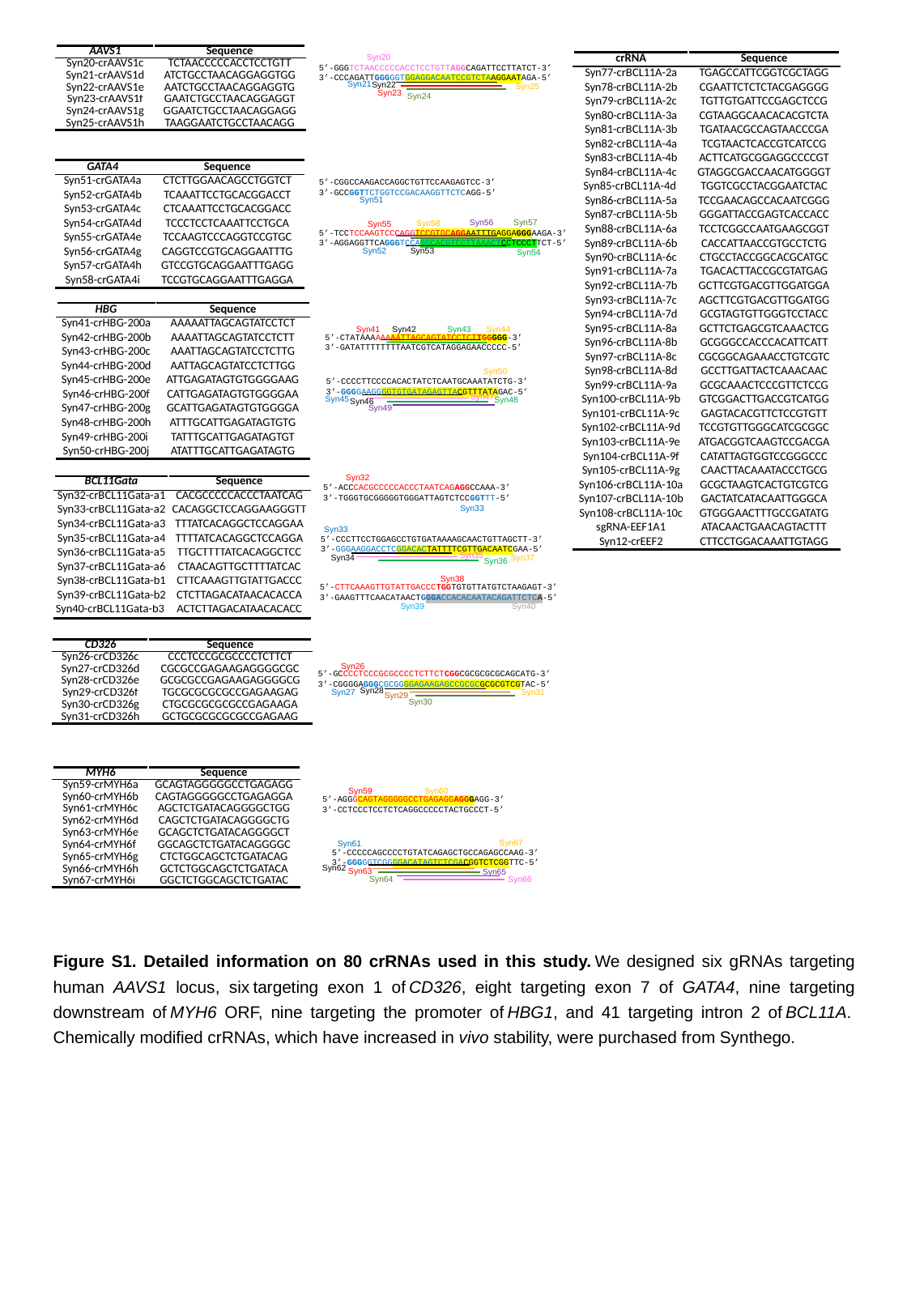

| AAVS1 | Sequence |
| --- | --- |
| Syn20-crAAVS1c | TCTAACCCCCACCTCCTGTT |
| Syn21-crAAVS1d | ATCTGCCTAACAGGAGGTGG |
| Syn22-crAAVS1e | AATCTGCCTAACAGGAGGTG |
| Syn23-crAAVS1f | GAATCTGCCTAACAGGAGGT |
| Syn24-crAAVS1g | GGAATCTGCCTAACAGGAGG |
| Syn25-crAAVS1h | TAAGGAATCTGCCTAACAGG |
Syn20
5’-GGGTCTAACCCCCACCTCCTGTTAGGCAGATTCCTTATCT-3’
3’-CCCAGATTGGGGGTGGAGGACAATCCGTCTAAGGAATAGA-5’
Syn21
Syn22
Syn25
Syn23
Syn24
| crRNA | Sequence |
| --- | --- |
| Syn77-crBCL11A-2a | TGAGCCATTCGGTCGCTAGG |
| Syn78-crBCL11A-2b | CGAATTCTCTCTACGAGGGG |
| Syn79-crBCL11A-2c | TGTTGTGATTCCGAGCTCCG |
| Syn80-crBCL11A-3a | CGTAAGGCAACACACGTCTA |
| Syn81-crBCL11A-3b | TGATAACGCCAGTAACCCGA |
| Syn82-crBCL11A-4a | TCGTAACTCACCGTCATCCG |
| Syn83-crBCL11A-4b | ACTTCATGCGGAGGCCCCGT |
| Syn84-crBCL11A-4c | GTAGGCGACCAACATGGGGT |
| Syn85-crBCL11A-4d | TGGTCGCCTACGGAATCTAC |
| Syn86-crBCL11A-5a | TCCGAACAGCCACAATCGGG |
| Syn87-crBCL11A-5b | GGGATTACCGAGTCACCACC |
| Syn88-crBCL11A-6a | TCCTCGGCCAATGAAGCGGT |
| Syn89-crBCL11A-6b | CACCATTAACCGTGCCTCTG |
| Syn90-crBCL11A-6c | CTGCCTACCGGCACGCATGC |
| Syn91-crBCL11A-7a | TGACACTTACCGCGTATGAG |
| Syn92-crBCL11A-7b | GCTTCGTGACGTTGGATGGA |
| Syn93-crBCL11A-7c | AGCTTCGTGACGTTGGATGG |
| Syn94-crBCL11A-7d | GCGTAGTGTTGGGTCCTACC |
| Syn95-crBCL11A-8a | GCTTCTGAGCGTCAAACTCG |
| Syn96-crBCL11A-8b | GCGGGCCACCCACATTCATT |
| Syn97-crBCL11A-8c | CGCGGCAGAAACCTGTCGTC |
| Syn98-crBCL11A-8d | GCCTTGATTACTCAAACAAC |
| Syn99-crBCL11A-9a | GCGCAAACTCCCGTTCTCCG |
| Syn100-crBCL11A-9b | GTCGGACTTGACCGTCATGG |
| Syn101-crBCL11A-9c | GAGTACACGTTCTCCGTGTT |
| Syn102-crBCL11A-9d | TCCGTGTTGGGCATCGCGGC |
| Syn103-crBCL11A-9e | ATGACGGTCAAGTCCGACGA |
| Syn104-crBCL11A-9f | CATATTAGTGGTCCGGGCCC |
| Syn105-crBCL11A-9g | CAACTTACAAATACCCTGCG |
| Syn106-crBCL11A-10a | GCGCTAAGTCACTGTCGTCG |
| Syn107-crBCL11A-10b | GACTATCATACAATTGGGCA |
| Syn108-crBCL11A-10c | GTGGGAACTTTGCCGATATG |
| sgRNA-EEF1A1 | ATACAACTGAACAGTACTTT |
| Syn12-crEEF2 | CTTCCTGGACAAATTGTAGG |
| GATA4 | Sequence |
| --- | --- |
| Syn51-crGATA4a | CTCTTGGAACAGCCTGGTCT |
| Syn52-crGATA4b | TCAAATTCCTGCACGGACCT |
| Syn53-crGATA4c | CTCAAATTCCTGCACGGACC |
| Syn54-crGATA4d | TCCCTCCTCAAATTCCTGCA |
| Syn55-crGATA4e | TCCAAGTCCCAGGTCCGTGC |
| Syn56-crGATA4g | CAGGTCCGTGCAGGAATTTG |
| Syn57-crGATA4h | GTCCGTGCAGGAATTTGAGG |
| Syn58-crGATA4i | TCCGTGCAGGAATTTGAGGA |
5’-CGGCCAAGACCAGGCTGTTCCAAGAGTCC-3’
3’-GCCGGTTCTGGTCCGACAAGGTTCTCAGG-5’
Syn51
Syn57
Syn56
Syn58
Syn55
5’-TCCTCCAAGTCCCAGGTCCGTGCAGGAATTTGAGGAGGGAAGA-3’
3’-AGGAGGTTCAGGGTCCAGGCACGTCCTTAAACTCCTCCCTTCT-5’
Syn52
Syn53
Syn54
| HBG | Sequence |
| --- | --- |
| Syn41-crHBG-200a | AAAAATTAGCAGTATCCTCT |
| Syn42-crHBG-200b | AAAATTAGCAGTATCCTCTT |
| Syn43-crHBG-200c | AAATTAGCAGTATCCTCTTG |
| Syn44-crHBG-200d | AATTAGCAGTATCCTCTTGG |
| Syn45-crHBG-200e | ATTGAGATAGTGTGGGGAAG |
| Syn46-crHBG-200f | CATTGAGATAGTGTGGGGAA |
| Syn47-crHBG-200g | GCATTGAGATAGTGTGGGGA |
| Syn48-crHBG-200h | ATTTGCATTGAGATAGTGTG |
| Syn49-crHBG-200i | TATTTGCATTGAGATAGTGT |
| Syn50-crHBG-200j | ATATTTGCATTGAGATAGTG |
Syn41
Syn42
Syn44
Syn43
5’-CTATAAAAAAAATTAGCAGTATCCTCTTGGGGG-3’
3’-GATATTTTTTTTAATCGTCATAGGAGAACCCCC-5’
Syn50
5’-CCCCTTCCCCACACTATCTCAATGCAAATATCTG-3’
3’-GGGGAAGGGGTGTGATAGAGTTACGTTTATAGAC-5’
Syn47
Syn45
Syn48
Syn46
Syn49
Syn32
5’-ACCCACGCCCCCACCCTAATCAGAGGCCAAA-3’
3’-TGGGTGCGGGGGTGGGATTAGTCTCCGGTTT-5’
Syn33
| BCL11Gata | Sequence |
| --- | --- |
| Syn32-crBCL11Gata-a1 | CACGCCCCCACCCTAATCAG |
| Syn33-crBCL11Gata-a2 | CACAGGCTCCAGGAAGGGTT |
| Syn34-crBCL11Gata-a3 | TTTATCACAGGCTCCAGGAA |
| Syn35-crBCL11Gata-a4 | TTTTATCACAGGCTCCAGGA |
| Syn36-crBCL11Gata-a5 | TTGCTTTTATCACAGGCTCC |
| Syn37-crBCL11Gata-a6 | CTAACAGTTGCTTTTATCAC |
| Syn38-crBCL11Gata-b1 | CTTCAAAGTTGTATTGACCC |
| Syn39-crBCL11Gata-b2 | CTCTTAGACATAACACACCA |
| Syn40-crBCL11Gata-b3 | ACTCTTAGACATAACACACC |
Syn33
5’-CCCTTCCTGGAGCCTGTGATAAAAGCAACTGTTAGCTT-3’
3’-GGGAAGGACCTCGGACACTATTTTCGTTGACAATCGAA-5’
Syn35
Syn37
Syn34
Syn36
Syn38
5’-CTTCAAAGTTGTATTGACCCTGGTGTGTTATGTCTAAGAGT-3’
3’-GAAGTTTCAACATAACTGGGACCACACAATACAGATTCTCA-5’
Syn39
Syn40
| CD326 | Sequence |
| --- | --- |
| Syn26-crCD326c | CCCTCCCGCGCCCCTCTTCT |
| Syn27-crCD326d | CGCGCCGAGAAGAGGGGCGC |
| Syn28-crCD326e | GCGCGCCGAGAAGAGGGGCG |
| Syn29-crCD326f | TGCGCGCGCGCCGAGAAGAG |
| Syn30-crCD326g | CTGCGCGCGCGCCGAGAAGA |
| Syn31-crCD326h | GCTGCGCGCGCGCCGAGAAG |
Syn26
5’-GCCCCTCCCGCGCCCCTCTTCTCGGCGCGCGCGCAGCATG-3’
3’-CGGGGAGGGCGCGGGGAGAAGAGCCGCGCGCGCGTCGTAC-5’
Syn28
Syn27
Syn31
Syn29
Syn30
| MYH6 | Sequence |
| --- | --- |
| Syn59-crMYH6a | GCAGTAGGGGGCCTGAGAGG |
| Syn60-crMYH6b | CAGTAGGGGGCCTGAGAGGA |
| Syn61-crMYH6c | AGCTCTGATACAGGGGCTGG |
| Syn62-crMYH6d | CAGCTCTGATACAGGGGCTG |
| Syn63-crMYH6e | GCAGCTCTGATACAGGGGCT |
| Syn64-crMYH6f | GGCAGCTCTGATACAGGGGC |
| Syn65-crMYH6g | CTCTGGCAGCTCTGATACAG |
| Syn66-crMYH6h | GCTCTGGCAGCTCTGATACA |
| Syn67-crMYH6i | GGCTCTGGCAGCTCTGATAC |
Syn60
Syn59
5’-AGGGCAGTAGGGGGCCTGAGAGGAGGGAGG-3’
3’-CCTCCCTCCTCTCAGGCCCCCTACTGCCCT-5’
Syn67
Syn61
 5’-CCCCCAGCCCCTGTATCAGAGCTGCCAGAGCCAAG-3’
 3’-GGGGGTCGGGGACATAGTCTCGACGGTCTCGGTTC-5’
Syn62
Syn63
Syn65
Syn64
Syn66
Figure S1. Detailed information on 80 crRNAs used in this study. We designed six gRNAs targeting human AAVS1 locus, six targeting exon 1 of CD326, eight targeting exon 7 of GATA4, nine targeting downstream of MYH6 ORF, nine targeting the promoter of HBG1, and 41 targeting intron 2 of BCL11A.  Chemically modified crRNAs, which have increased in vivo stability, were purchased from Synthego.

## Slide 2
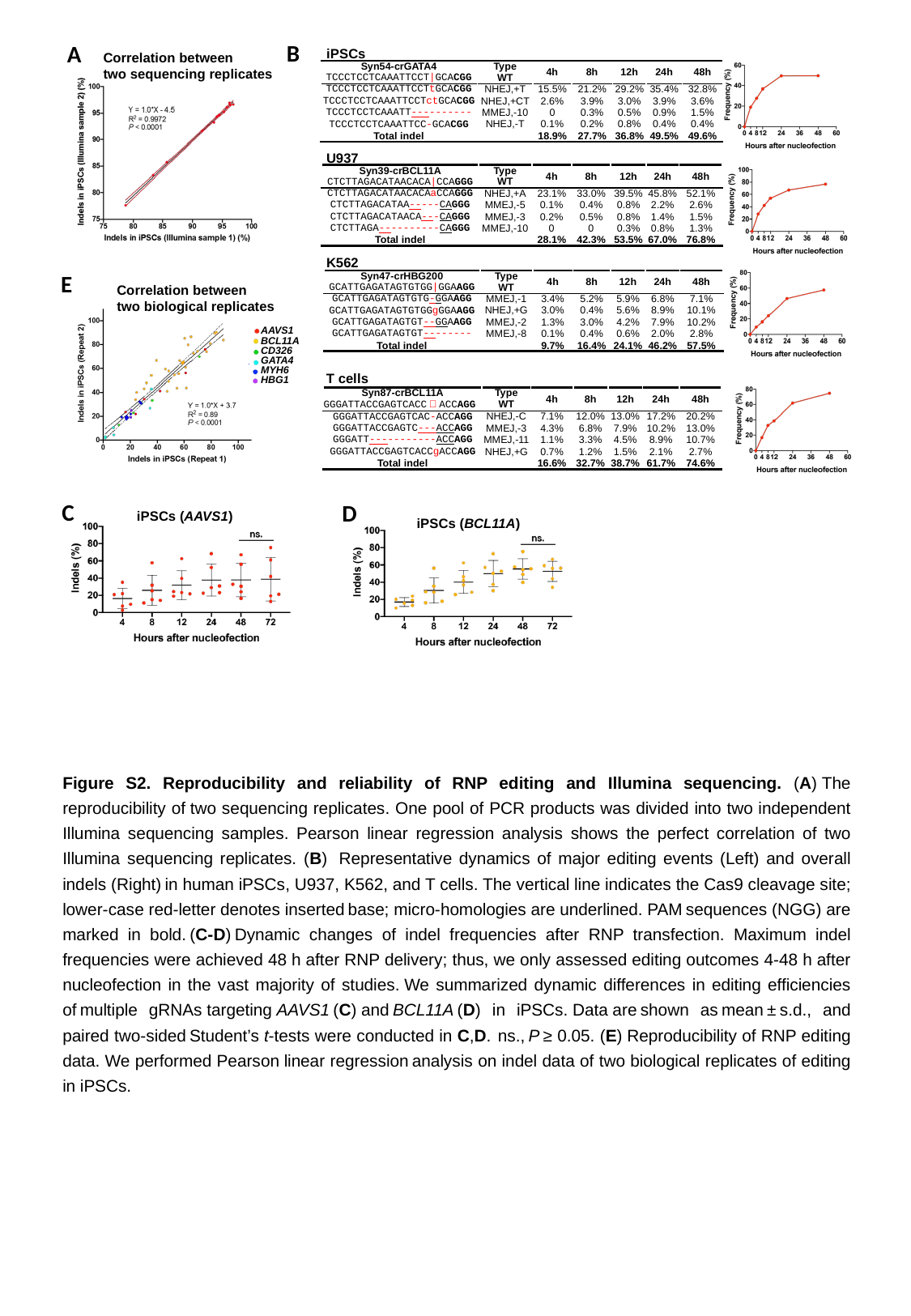

B
A
iPSCs
Correlation between two sequencing replicates
| Syn54-crGATA4 TCCCTCCTCAAATTCCT|GCACGG | Type WT | 4h | 8h | 12h | 24h | 48h |
| --- | --- | --- | --- | --- | --- | --- |
| TCCCTCCTCAAATTCCTtGCACGG | NHEJ,+T | 15.5% | 21.2% | 29.2% | 35.4% | 32.8% |
| TCCCTCCTCAAATTCCTctGCACGG | NHEJ,+CT | 2.6% | 3.9% | 3.0% | 3.9% | 3.6% |
| TCCCTCCTCAAATT---------- | MMEJ,-10 | 0 | 0.3% | 0.5% | 0.9% | 1.5% |
| TCCCTCCTCAAATTCC-GCACGG | NHEJ,-T | 0.1% | 0.2% | 0.8% | 0.4% | 0.4% |
| Total indel | | 18.9% | 27.7% | 36.8% | 49.5% | 49.6% |
U937
| Syn39-crBCL11A CTCTTAGACATAACACA|CCAGGG | Type WT | 4h | 8h | 12h | 24h | 48h |
| --- | --- | --- | --- | --- | --- | --- |
| CTCTTAGACATAACACAaCCAGGG | NHEJ,+A | 23.1% | 33.0% | 39.5% | 45.8% | 52.1% |
| CTCTTAGACATAA-----CAGGG | MMEJ,-5 | 0.1% | 0.4% | 0.8% | 2.2% | 2.6% |
| CTCTTAGACATAACA---CAGGG | MMEJ,-3 | 0.2% | 0.5% | 0.8% | 1.4% | 1.5% |
| CTCTTAGA----------CAGGG | MMEJ,-10 | 0 | 0 | 0.3% | 0.8% | 1.3% |
| Total indel | | 28.1% | 42.3% | 53.5% | 67.0% | 76.8% |
K562
E
| Syn47-crHBG200 GCATTGAGATAGTGTGG|GGAAGG | Type WT | 4h | 8h | 12h | 24h | 48h |
| --- | --- | --- | --- | --- | --- | --- |
| GCATTGAGATAGTGTG-GGAAGG | MMEJ,-1 | 3.4% | 5.2% | 5.9% | 6.8% | 7.1% |
| GCATTGAGATAGTGTGGgGGAAGG | NHEJ,+G | 3.0% | 0.4% | 5.6% | 8.9% | 10.1% |
| GCATTGAGATAGTGT--GGAAGG | MMEJ,-2 | 1.3% | 3.0% | 4.2% | 7.9% | 10.2% |
| GCATTGAGATAGTGT-------- | MMEJ,-8 | 0.1% | 0.4% | 0.6% | 2.0% | 2.8% |
| Total indel | | 9.7% | 16.4% | 24.1% | 46.2% | 57.5% |
Correlation between two biological replicates
AAVS1
BCL11A
CD326
GATA4
MYH6
HBG1
T cells
| Syn87-crBCL11A GGGATTACCGAGTCACC｜ACCAGG | Type WT | 4h | 8h | 12h | 24h | 48h |
| --- | --- | --- | --- | --- | --- | --- |
| GGGATTACCGAGTCAC-ACCAGG | NHEJ,-C | 7.1% | 12.0% | 13.0% | 17.2% | 20.2% |
| GGGATTACCGAGTC---ACCAGG | MMEJ,-3 | 4.3% | 6.8% | 7.9% | 10.2% | 13.0% |
| GGGATT-----------ACCAGG | MMEJ,-11 | 1.1% | 3.3% | 4.5% | 8.9% | 10.7% |
| GGGATTACCGAGTCACCgACCAGG | NHEJ,+G | 0.7% | 1.2% | 1.5% | 2.1% | 2.7% |
| Total indel | | 16.6% | 32.7% | 38.7% | 61.7% | 74.6% |
C
D
iPSCs (AAVS1)
iPSCs (BCL11A)
Figure S2. Reproducibility and reliability of RNP editing and Illumina sequencing. (A) The reproducibility of two sequencing replicates. One pool of PCR products was divided into two independent Illumina sequencing samples. Pearson linear regression analysis shows the perfect correlation of two Illumina sequencing replicates. (B)  Representative dynamics of major editing events (Left) and overall indels (Right) in human iPSCs, U937, K562, and T cells. The vertical line indicates the Cas9 cleavage site; lower-case red-letter denotes inserted base; micro-homologies are underlined. PAM sequences (NGG) are marked in bold. (C-D) Dynamic changes of indel frequencies after RNP transfection. Maximum indel frequencies were achieved 48 h after RNP delivery; thus, we only assessed editing outcomes 4-48 h after nucleofection in the vast majority of studies. We summarized dynamic differences in editing efficiencies of multiple gRNAs targeting AAVS1 (C) and BCL11A (D) in iPSCs. Data are shown as mean ± s.d., and paired two-sided Student’s t-tests were conducted in C,D.  ns., P ≥ 0.05. (E) Reproducibility of RNP editing data. We performed Pearson linear regression analysis on indel data of two biological replicates of editing in iPSCs.

## Slide 3
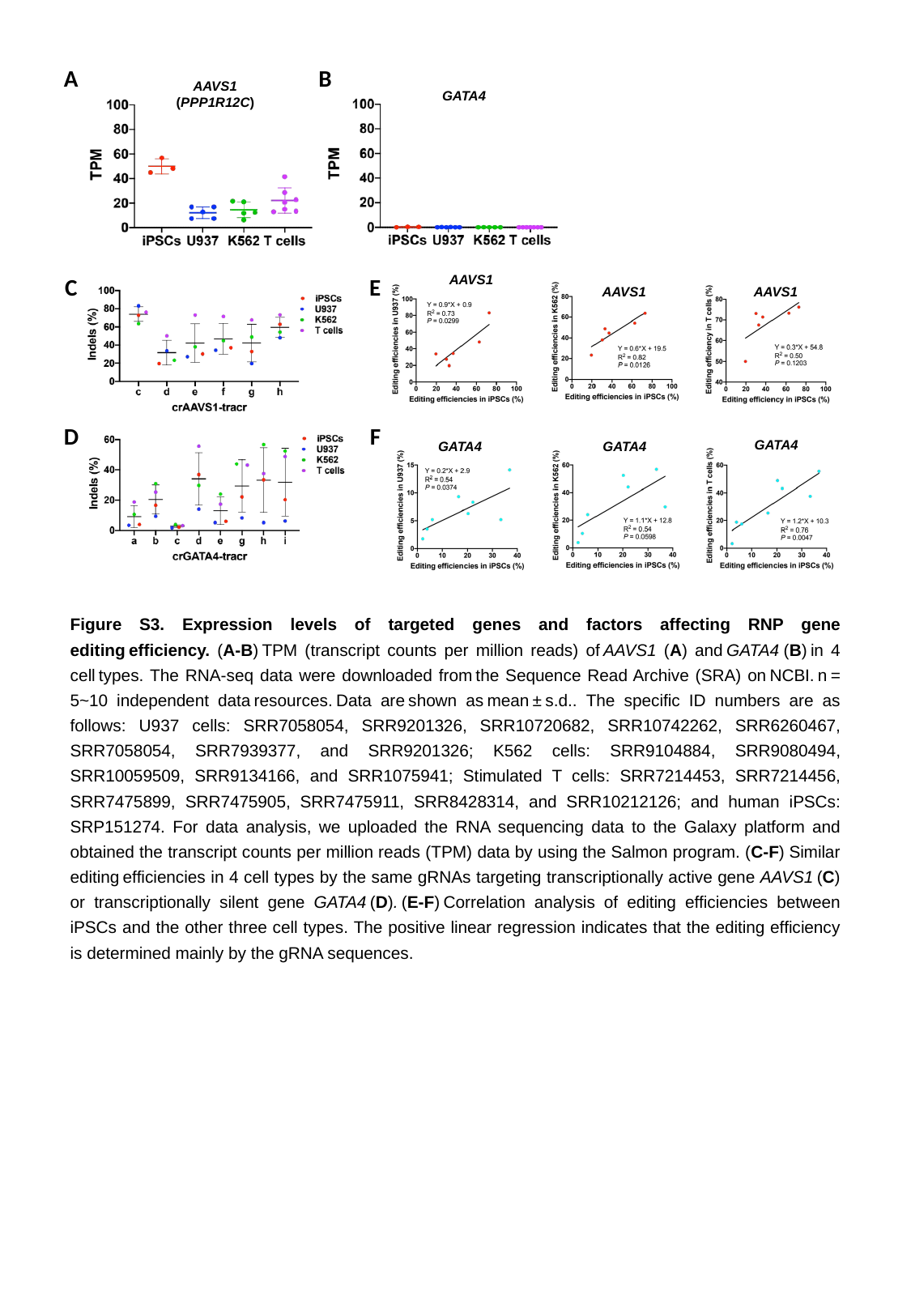

B
A
AAVS1
(PPP1R12C)
GATA4
AAVS1
C
E
AAVS1
AAVS1
D
F
GATA4
GATA4
GATA4
Figure S3. Expression levels of targeted genes and factors affecting RNP gene editing efficiency. (A-B) TPM (transcript counts per million reads) of AAVS1 (A) and GATA4 (B) in 4 cell types. The RNA-seq data were downloaded from the Sequence Read Archive (SRA) on NCBI. n = 5~10 independent data resources. Data are shown as mean ± s.d.. The specific ID numbers are as follows: U937 cells: SRR7058054, SRR9201326, SRR10720682, SRR10742262, SRR6260467, SRR7058054, SRR7939377, and SRR9201326; K562 cells: SRR9104884, SRR9080494, SRR10059509, SRR9134166, and SRR1075941; Stimulated T cells: SRR7214453, SRR7214456, SRR7475899, SRR7475905, SRR7475911, SRR8428314, and SRR10212126; and human iPSCs: SRP151274. For data analysis, we uploaded the RNA sequencing data to the Galaxy platform and obtained the transcript counts per million reads (TPM) data by using the Salmon program. (C-F) Similar editing efficiencies in 4 cell types by the same gRNAs targeting transcriptionally active gene AAVS1 (C) or transcriptionally silent gene GATA4 (D). (E-F) Correlation analysis of editing efficiencies between iPSCs and the other three cell types. The positive linear regression indicates that the editing efficiency is determined mainly by the gRNA sequences.

## Slide 4
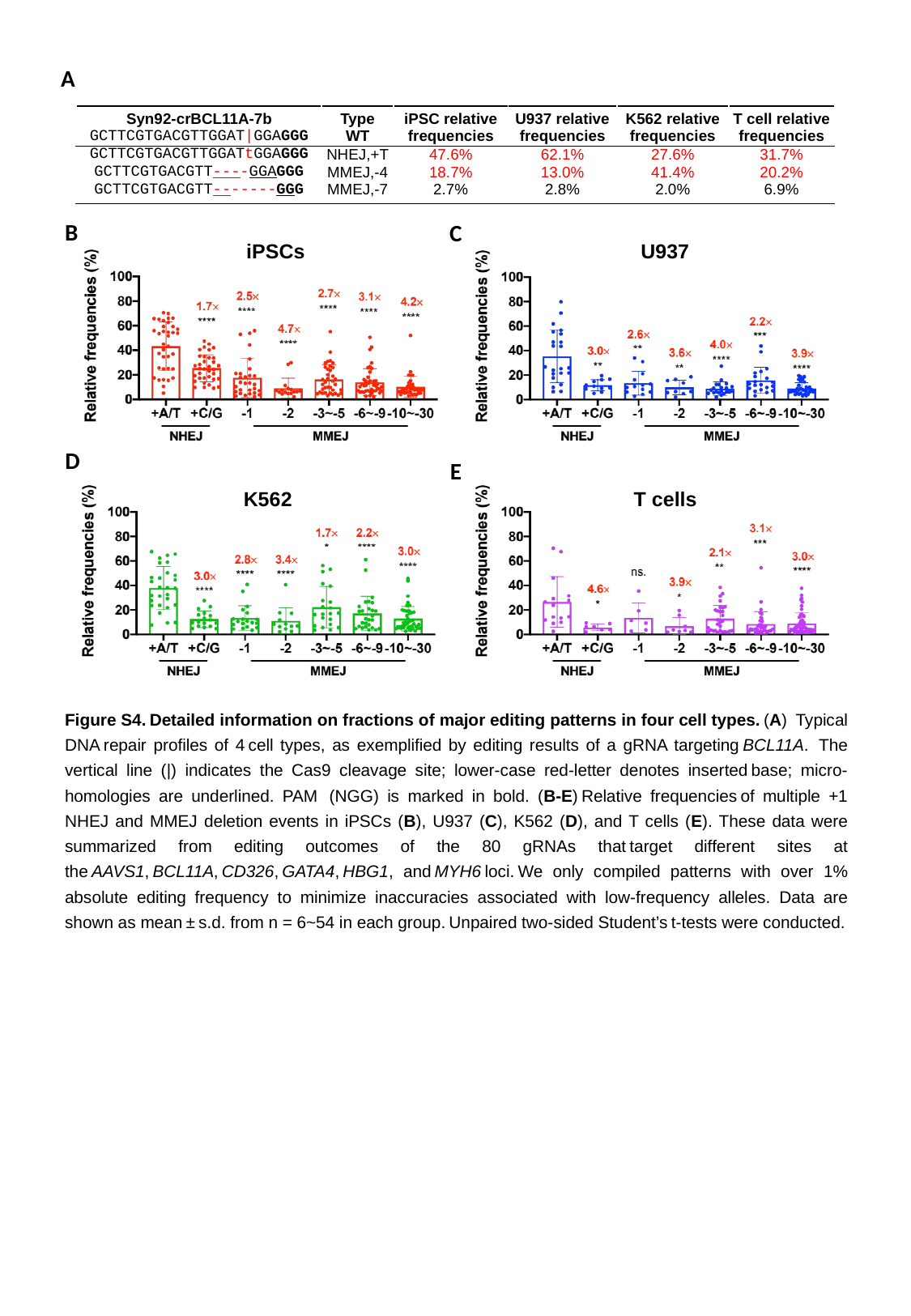

A
| Syn92-crBCL11A-7b GCTTCGTGACGTTGGAT|GGAGGG | Type WT | iPSC relative frequencies | U937 relative frequencies | K562 relative frequencies | T cell relative frequencies |
| --- | --- | --- | --- | --- | --- |
| GCTTCGTGACGTTGGATtGGAGGG | NHEJ,+T | 47.6% | 62.1% | 27.6% | 31.7% |
| GCTTCGTGACGTT----GGAGGG | MMEJ,-4 | 18.7% | 13.0% | 41.4% | 20.2% |
| GCTTCGTGACGTT-------GGG | MMEJ,-7 | 2.7% | 2.8% | 2.0% | 6.9% |
B
C
iPSCs
U937
D
E
K562
T cells
Figure S4. Detailed information on fractions of major editing patterns in four cell types. (A)  Typical DNA repair profiles of 4 cell types, as exemplified by editing results of a gRNA targeting BCL11A.  The vertical line (|) indicates the Cas9 cleavage site; lower-case red-letter denotes inserted base; micro-homologies are underlined. PAM  (NGG) is marked in bold. (B-E) Relative frequencies of multiple +1 NHEJ and MMEJ deletion events in iPSCs (B), U937 (C), K562 (D), and T cells (E). These data were summarized from editing outcomes of the 80 gRNAs that target different sites at the AAVS1, BCL11A, CD326, GATA4, HBG1, and MYH6 loci. We only compiled patterns with over 1% absolute editing frequency to minimize inaccuracies associated with low-frequency alleles. Data are shown as mean ± s.d. from n = 6~54 in each group. Unpaired two-sided Student’s t-tests were conducted.

## Slide 5
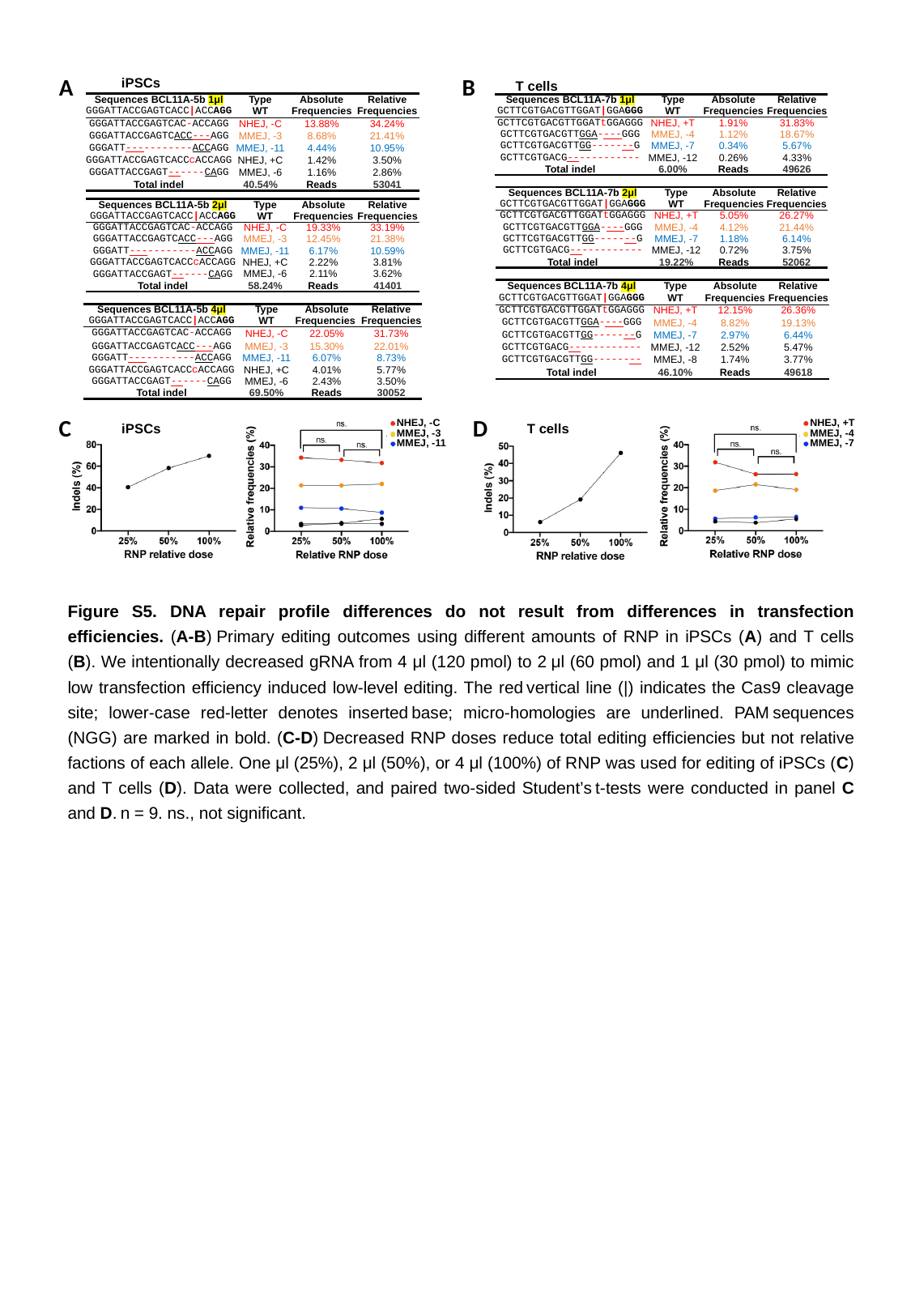

A
B
iPSCs
T cells
| Sequences BCL11A-5b 1μl GGGATTACCGAGTCACC|ACCAGG | Type WT | Absolute Frequencies | Relative Frequencies |
| --- | --- | --- | --- |
| GGGATTACCGAGTCAC-ACCAGG | NHEJ, -C | 13.88% | 34.24% |
| GGGATTACCGAGTCACC---AGG | MMEJ, -3 | 8.68% | 21.41% |
| GGGATT-----------ACCAGG | MMEJ, -11 | 4.44% | 10.95% |
| GGGATTACCGAGTCACCcACCAGG | NHEJ, +C | 1.42% | 3.50% |
| GGGATTACCGAGT------CAGG | MMEJ, -6 | 1.16% | 2.86% |
| Total indel | 40.54% | Reads | 53041 |
| Sequences BCL11A-7b 1μl GCTTCGTGACGTTGGAT|GGAGGG | Type WT | Absolute Frequencies | Relative Frequencies |
| --- | --- | --- | --- |
| GCTTCGTGACGTTGGATtGGAGGG | NHEJ, +T | 1.91% | 31.83% |
| GCTTCGTGACGTTGGA----GGG | MMEJ, -4 | 1.12% | 18.67% |
| GCTTCGTGACGTTGG-------G | MMEJ, -7 | 0.34% | 5.67% |
| GCTTCGTGACG------------ | MMEJ, -12 | 0.26% | 4.33% |
| Total indel | 6.00% | Reads | 49626 |
| Sequences BCL11A-7b 2μl GCTTCGTGACGTTGGAT|GGAGGG | Type WT | Absolute Frequencies | Relative Frequencies |
| --- | --- | --- | --- |
| GCTTCGTGACGTTGGATtGGAGGG | NHEJ, +T | 5.05% | 26.27% |
| GCTTCGTGACGTTGGA----GGG | MMEJ, -4 | 4.12% | 21.44% |
| GCTTCGTGACGTTGG-------G | MMEJ, -7 | 1.18% | 6.14% |
| GCTTCGTGACG------------ | MMEJ, -12 | 0.72% | 3.75% |
| Total indel | 19.22% | Reads | 52062 |
| Sequences BCL11A-5b 2μl GGGATTACCGAGTCACC|ACCAGG | Type WT | Absolute Frequencies | Relative Frequencies |
| --- | --- | --- | --- |
| GGGATTACCGAGTCAC-ACCAGG | NHEJ, -C | 19.33% | 33.19% |
| GGGATTACCGAGTCACC---AGG | MMEJ, -3 | 12.45% | 21.38% |
| GGGATT-----------ACCAGG | MMEJ, -11 | 6.17% | 10.59% |
| GGGATTACCGAGTCACCcACCAGG | NHEJ, +C | 2.22% | 3.81% |
| GGGATTACCGAGT------CAGG | MMEJ, -6 | 2.11% | 3.62% |
| Total indel | 58.24% | Reads | 41401 |
| Sequences BCL11A-7b 4μl GCTTCGTGACGTTGGAT|GGAGGG | Type WT | Absolute Frequencies | Relative Frequencies |
| --- | --- | --- | --- |
| GCTTCGTGACGTTGGATtGGAGGG | NHEJ, +T | 12.15% | 26.36% |
| GCTTCGTGACGTTGGA----GGG | MMEJ, -4 | 8.82% | 19.13% |
| GCTTCGTGACGTTGG-------G | MMEJ, -7 | 2.97% | 6.44% |
| GCTTCGTGACG------------ | MMEJ, -12 | 2.52% | 5.47% |
| GCTTCGTGACGTTGG-------- | MMEJ, -8 | 1.74% | 3.77% |
| Total indel | 46.10% | Reads | 49618 |
| Sequences BCL11A-5b 4μl GGGATTACCGAGTCACC|ACCAGG | Type WT | Absolute Frequencies | Relative Frequencies |
| --- | --- | --- | --- |
| GGGATTACCGAGTCAC-ACCAGG | NHEJ, -C | 22.05% | 31.73% |
| GGGATTACCGAGTCACC---AGG | MMEJ, -3 | 15.30% | 22.01% |
| GGGATT-----------ACCAGG | MMEJ, -11 | 6.07% | 8.73% |
| GGGATTACCGAGTCACCcACCAGG | NHEJ, +C | 4.01% | 5.77% |
| GGGATTACCGAGT------CAGG | MMEJ, -6 | 2.43% | 3.50% |
| Total indel | 69.50% | Reads | 30052 |
D
C
iPSCs
T cells
NHEJ, +T
MMEJ, -4
MMEJ, -7
NHEJ, -C
MMEJ, -3
MMEJ, -11
Figure S5. DNA repair profile differences do not result from differences in transfection efficiencies. (A-B) Primary editing outcomes using different amounts of RNP in iPSCs (A) and T cells (B). We intentionally decreased gRNA from 4 μl (120 pmol) to 2 μl (60 pmol) and 1 μl (30 pmol) to mimic low transfection efficiency induced low-level editing. The red vertical line (|) indicates the Cas9 cleavage site; lower-case red-letter denotes inserted base; micro-homologies are underlined. PAM sequences (NGG) are marked in bold. (C-D) Decreased RNP doses reduce total editing efficiencies but not relative factions of each allele. One μl (25%), 2 μl (50%), or 4 μl (100%) of RNP was used for editing of iPSCs (C) and T cells (D). Data were collected, and paired two-sided Student’s t-tests were conducted in panel C and D. n = 9. ns., not significant.

## Slide 6
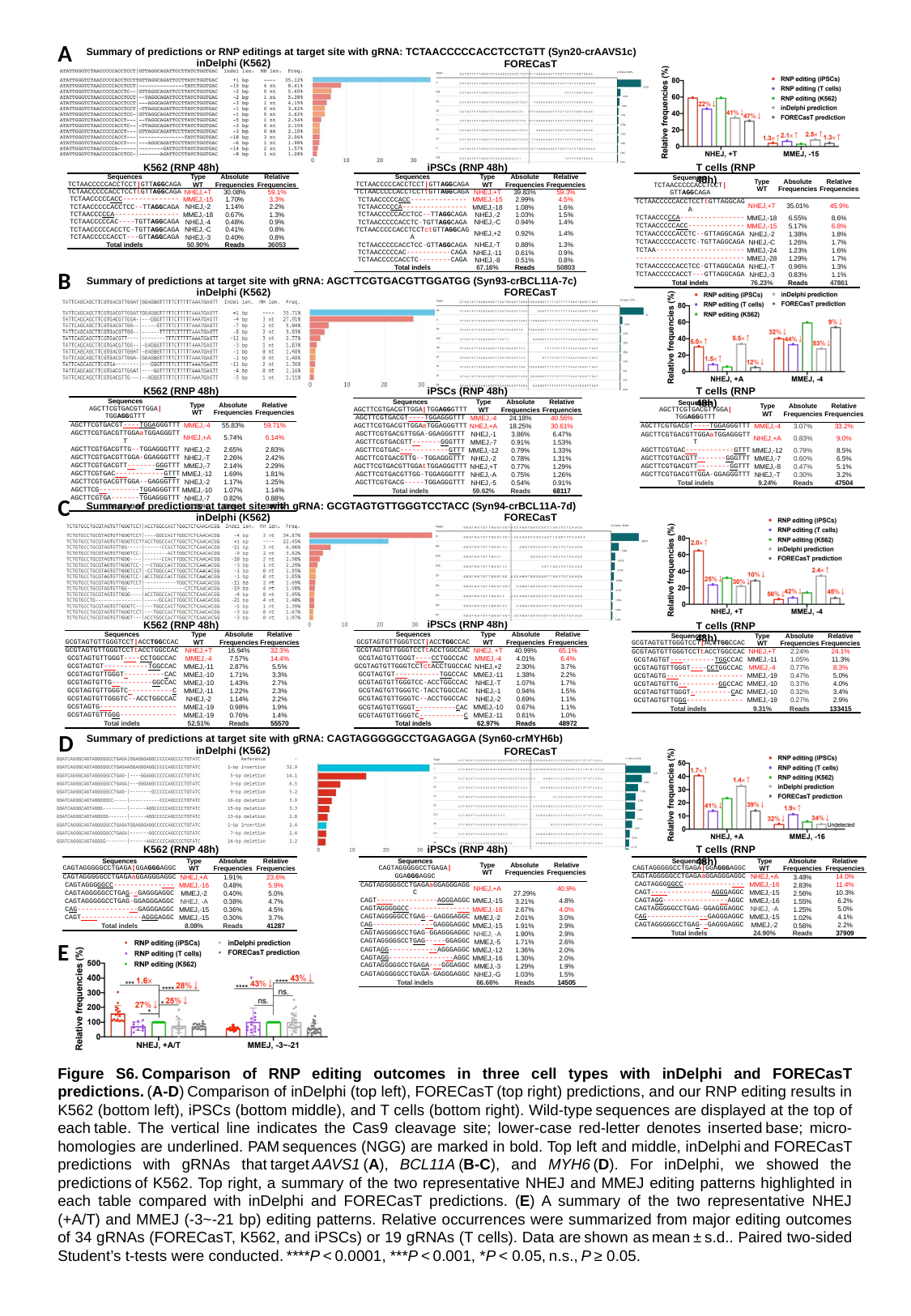

Syn93-crBCL11A-7c
A
Summary of predictions or RNP editings at target site with gRNA: TCTAACCCCCACCTCCTGTT (Syn20-crAAVS1c)
inDelphi (K562)
FORECasT
iPSCs (RNP 48h)
K562 (RNP 48h)
T cells (RNP 48h)
| Sequences TCTAACCCCCACCTCCT|GTTAGGCAGA | Type WT | Absolute Frequencies | Relative Frequencies |
| --- | --- | --- | --- |
| TCTAACCCCCACCTCCTtGTTAGGCAGA | NHEJ,+T | 30.08% | 59.1% |
| TCTAACCCCCACC-------------- | MMEJ,-15 | 1.70% | 3.3% |
| TCTAACCCCCACCTCC--TTAGGCAGA | NHEJ,-2 | 1.14% | 2.2% |
| TCTAACCCCCA---------------- | MMEJ,-18 | 0.67% | 1.3% |
| TCTAACCCCCAC----TGTTAGGCAGA | NHEJ,-4 | 0.48% | 0.9% |
| TCTAACCCCCACCTC-TGTTAGGCAGA | NHEJ,-C | 0.41% | 0.8% |
| TCTAACCCCCACCT---GTTAGGCAGA | NHEJ,-3 | 0.40% | 0.8% |
| Total indels | 50.90% | Reads | 36053 |
| Sequences TCTAACCCCCACCTCCT|GTTAGGCAGA | Type WT | Absolute Frequencies | Relative Frequencies |
| --- | --- | --- | --- |
| TCTAACCCCCACCTCCTtGTTAGGCAGA | NHEJ,+T | 39.83% | 59.3% |
| TCTAACCCCCACC-------------- | MMEJ,-15 | 2.99% | 4.5% |
| TCTAACCCCCA---------------- | MMEJ,-18 | 1.08% | 1.6% |
| TCTAACCCCCACCTCC--TTAGGCAGA | NHEJ,-2 | 1.03% | 1.5% |
| TCTAACCCCCACCTC-TGTTAGGCAGA | NHEJ,-C | 0.94% | 1.4% |
| TCTAACCCCCACCTCCTctGTTAGGCAGA | NHEJ,+2 | 0.92% | 1.4% |
| TCTAACCCCCACCTCC-GTTAGGCAGA | NHEJ,-T | 0.88% | 1.3% |
| TCTAACCCCCAC-----------CAGA | NHEJ,-11 | 0.61% | 0.9% |
| TCTAACCCCCACCTC--------CAGA | NHEJ,-8 | 0.51% | 0.8% |
| Total indels | 67.16% | Reads | 50803 |
| Sequences TCTAACCCCCACCTCCT|GTTAGGCAGA | Type WT | Absolute Frequencies | Relative Frequencies |
| --- | --- | --- | --- |
| TCTAACCCCCACCTCCTtGTTAGGCAGA | NHEJ,+T | 35.01% | 45.9% |
| TCTAACCCCCA---------------- | MMEJ,-18 | 6.55% | 8.6% |
| TCTAACCCCCACC-------------- | MMEJ,-15 | 5.17% | 6.8% |
| TCTAACCCCCACCTC--GTTAGGCAGA | NHEJ,-2 | 1.38% | 1.8% |
| TCTAACCCCCACCTC-TGTTAGGCAGA | NHEJ,-C | 1.26% | 1.7% |
| TCTAA---------------------- | MMEJ,-24 | 1.23% | 1.6% |
| --------------------------- | MMEJ,-28 | 1.29% | 1.7% |
| TCTAACCCCCACCTCC-GTTAGGCAGA | NHEJ,-T | 0.96% | 1.3% |
| TCTAACCCCCACCT---GTTAGGCAGA | NHEJ,-3 | 0.83% | 1.1% |
| Total indels | 76.23% | Reads | 47861 |
B
Summary of predictions at target site with gRNA: AGCTTCGTGACGTTGGATGG (Syn93-crBCL11A-7c)
FORECasT
inDelphi (K562)
K562 (RNP 48h)
T cells (RNP 48h)
iPSCs (RNP 48h)
| Sequences AGCTTCGTGACGTTGGA|TGGAGGGTTT | Type WT | Absolute Frequencies | Relative Frequencies |
| --- | --- | --- | --- |
| AGCTTCGTGACGT----TGGAGGGTTT | MMEJ,-4 | 55.83% | 59.71% |
| AGCTTCGTGACGTTGGAaTGGAGGGTTT | NHEJ,+A | 5.74% | 6.14% |
| AGCTTCGTGACGTTG--TGGAGGGTTT | NHEJ,-2 | 2.65% | 2.83% |
| AGCTTCGTGACGTTGGA-GGAGGGTTT | NHEJ,-T | 2.26% | 2.42% |
| AGCTTCGTGACGTT-------GGGTTT | MMEJ,-7 | 2.14% | 2.29% |
| AGCTTCGTGAC------------GTTT | MMEJ,-12 | 1.69% | 1.81% |
| AGCTTCGTGACGTTGGA--GAGGGTTT | NHEJ,-2 | 1.17% | 1.25% |
| AGCTTCG----------TGGAGGGTTT | MMEJ,-10 | 1.07% | 1.14% |
| AGCTTCGTGA-------TGGAGGGTTT | NHEJ,-7 | 0.82% | 0.88% |
| Total indels | 93.50% | Reads | 34674 |
| Sequences AGCTTCGTGACGTTGGA|TGGAGGGTTT | Type WT | Absolute Frequencies | Relative Frequencies |
| --- | --- | --- | --- |
| AGCTTCGTGACGT----TGGAGGGTTT | MMEJ,-4 | 24.18% | 40.56% |
| AGCTTCGTGACGTTGGAaTGGAGGGTTT | NHEJ,+A | 18.25% | 30.61% |
| AGCTTCGTGACGTTGGA-GGAGGGTTT | NHEJ,-1 | 3.86% | 6.47% |
| AGCTTCGTGACGTT-------GGGTTT | MMEJ,-7 | 0.91% | 1.53% |
| AGCTTCGTGAC------------GTTT | MMEJ,-12 | 0.79% | 1.33% |
| AGCTTCGTGACGTTG--TGGAGGGTTT | NHEJ,-2 | 0.78% | 1.31% |
| AGCTTCGTGACGTTGGAtTGGAGGGTTT | NHEJ,+T | 0.77% | 1.29% |
| AGCTTCGTGACGTTGG-TGGAGGGTTT | NHEJ,-A | 0.75% | 1.26% |
| AGCTTCGTGACG-----TGGAGGGTTT | NHEJ,-5 | 0.54% | 0.91% |
| Total indels | 59.62% | Reads | 68117 |
| Sequences AGCTTCGTGACGTTGGA|TGGAGGGTTT | Type WT | Absolute Frequencies | Relative Frequencies |
| --- | --- | --- | --- |
| AGCTTCGTGACGT----TGGAGGGTTT | MMEJ,-4 | 3.07% | 33.2% |
| AGCTTCGTGACGTTGGAaTGGAGGGTTT | NHEJ,+A | 0.83% | 9.0% |
| AGCTTCGTGAC------------GTTT | MMEJ,-12 | 0.79% | 8.5% |
| AGCTTCGTGACGTT-------GGGTTT | MMEJ,-7 | 0.60% | 6.5% |
| AGCTTCGTGACGTT--------GGTTT | MMEJ,-8 | 0.47% | 5.1% |
| AGCTTCGTGACGTTGGA-GGAGGGTTT | NHEJ,-T | 0.30% | 3.2% |
| Total indels | 9.24% | Reads | 47504 |
C
Summary of predictions at target site with gRNA: GCGTAGTGTTGGGTCCTACC (Syn94-crBCL11A-7d)
inDelphi (K562)
FORECasT
iPSCs (RNP 48h)
K562 (RNP 48h)
T cells (RNP 48h)
| Sequences GCGTAGTGTTGGGTCCT|ACCTGGCCAC | Type WT | Absolute Frequencies | Relative Frequencies |
| --- | --- | --- | --- |
| GCGTAGTGTTGGGTCCTtACCTGGCCAC | NHEJ,+T | 16.94% | 32.3% |
| GCGTAGTGTTGGGT----CCTGGCCAC | MMEJ,-4 | 7.57% | 14.4% |
| GCGTAGTGT-----------TGGCCAC | MMEJ,-11 | 2.87% | 5.5% |
| GCGTAGTGTTGGGT----------CAC | MMEJ,-10 | 1.71% | 3.3% |
| GCGTAGTGTTG----------GGCCAC | MMEJ,-10 | 1.43% | 2.7% |
| GCGTAGTGTTGGGTC-----------C | MMEJ,-11 | 1.22% | 2.3% |
| GCGTAGTGTTGGGTC--ACCTGGCCAC | NHEJ,-2 | 1.14% | 2.2% |
| GCGTAGTG------------------- | MMEJ,-19 | 0.98% | 1.9% |
| GCGTAGTGTTGGG-------------- | MMEJ,-19 | 0.76% | 1.4% |
| Total indels | 52.51% | Reads | 55570 |
| Sequences GCGTAGTGTTGGGTCCT|ACCTGGCCAC | Type WT | Absolute Frequencies | Relative Frequencies |
| --- | --- | --- | --- |
| GCGTAGTGTTGGGTCCTtACCTGGCCAC | NHEJ, +T | 40.99% | 65.1% |
| GCGTAGTGTTGGGT----CCTGGCCAC | MMEJ,-4 | 4.01% | 6.4% |
| GCGTAGTGTTGGGTCCTctACCTGGCCAC | NHEJ,+2 | 2.30% | 3.7% |
| GCGTAGTGT-----------TGGCCAC | MMEJ,-11 | 1.38% | 2.2% |
| GCGTAGTGTTGGGTCC-ACCTGGCCAC | NHEJ,-T | 1.07% | 1.7% |
| GCGTAGTGTTGGGTC-TACCTGGCCAC | NHEJ,-1 | 0.94% | 1.5% |
| GCGTAGTGTTGGGTC--ACCTGGCCAC | NHEJ,-2 | 0.69% | 1.1% |
| GCGTAGTGTTGGGT----------CAC | MMEJ,-10 | 0.67% | 1.1% |
| GCGTAGTGTTGGGTC-----------C | MMEJ,-11 | 0.61% | 1.0% |
| Total indels | 62.97% | Reads | 48972 |
| Sequences GCGTAGTGTTGGGTCCT|ACCTGGCCAC | Type WT | Absolute Frequencies | Relative Frequencies |
| --- | --- | --- | --- |
| GCGTAGTGTTGGGTCCTtACCTGGCCAC | NHEJ,+T | 2.24% | 24.1% |
| GCGTAGTGT-----------TGGCCAC | MMEJ,-11 | 1.05% | 11.3% |
| GCGTAGTGTTGGGT----CCTGGCCAC | MMEJ,-4 | 0.77% | 8.3% |
| GCGTAGTG------------------- | MMEJ,-19 | 0.47% | 5.0% |
| GCGTAGTGTTG----------GGCCAC | MMEJ,-10 | 0.37% | 4.0% |
| GCGTAGTGTTGGGT----------CAC | MMEJ,-10 | 0.32% | 3.4% |
| GCGTAGTGTTGGG-------------- | MMEJ,-19 | 0.27% | 2.9% |
| Total indels | 9.31% | Reads | 133415 |
D
Summary of predictions at target site with gRNA: CAGTAGGGGGCCTGAGAGGA (Syn60-crMYH6b)
inDelphi (K562)
FORECasT
K562 (RNP 48h)
T cells (RNP 48h)
iPSCs (RNP 48h)
| Sequences CAGTAGGGGGCCTGAGA|GGAGGGAGGC | Type WT | Absolute Frequencies | Relative Frequencies |
| --- | --- | --- | --- |
| CAGTAGGGGGCCTGAGAaGGAGGGAGGC | NHEJ,+A | 3.48% | 14.0% |
| CAGTAGGGGGCC--------------- | MMEJ,-16 | 2.83% | 11.4% |
| CAGT---------------AGGGAGGC | MMEJ,-15 | 2.56% | 10.3% |
| CAGTAGG----------------AGGC | MMEJ,-16 | 1.55% | 6.2% |
| CAGTAGGGGGCCTGAG-GGAGGGAGGC | NHEJ, -A | 1.25% | 5.0% |
| CAG---------------GAGGGAGGC | MMEJ,-15 | 1.02% | 4.1% |
| CAGTAGGGGGCCTGAG--GAGGGAGGC | MMEJ,-2 | 0.56% | 2.2% |
| Total indels | 24.90% | Reads | 37909 |
| Sequences CAGTAGGGGGCCTGAGA|GGAGGGAGGC | Type WT | Absolute Frequencies | Relative Frequencies |
| --- | --- | --- | --- |
| CAGTAGGGGGCCTGAGAaGGAGGGAGGC | NHEJ,+A | 27.29% | 40.9% |
| CAGT---------------AGGGAGGC | MMEJ,-15 | 3.21% | 4.8% |
| CAGTAGGGGGCC--------------- | MMEJ,-16 | 2.67% | 4.0% |
| CAGTAGGGGGCCTGAG--GAGGGAGGC | MMEJ,-2 | 2.01% | 3.0% |
| CAG---------------GAGGGAGGC | MMEJ,-15 | 1.91% | 2.9% |
| CAGTAGGGGGCCTGAG-GGAGGGAGGC | NHEJ, -A | 1.90% | 2.9% |
| CAGTAGGGGGCCTGAG-----GGAGGC | MMEJ,-5 | 1.71% | 2.6% |
| CAGTAGG------------AGGGAGGC | MMEJ,-12 | 1.36% | 2.0% |
| CAGTAGG----------------AGGC | MMEJ,-16 | 1.30% | 2.0% |
| CAGTAGGGGGCCTGAGA---GGGAGGC | MMEJ,-3 | 1.29% | 1.9% |
| CAGTAGGGGGCCTGAGA-GAGGGAGGC | NHEJ,-G | 1.03% | 1.5% |
| Total indels | 66.66% | Reads | 14505 |
| Sequences CAGTAGGGGGCCTGAGA|GGAGGGAGGC | Type WT | Absolute Frequencies | Relative Frequencies |
| --- | --- | --- | --- |
| CAGTAGGGGGCCTGAGAaGGAGGGAGGC | NHEJ,+A | 1.91% | 23.6% |
| CAGTAGGGGGCC--------------- | MMEJ,-16 | 0.48% | 5.9% |
| CAGTAGGGGGCCTGAG--GAGGGAGGC | MMEJ,-2 | 0.40% | 5.0% |
| CAGTAGGGGGCCTGAG-GGAGGGAGGC | NHEJ, -A | 0.38% | 4.7% |
| CAG---------------GAGGGAGGC | MMEJ,-15 | 0.36% | 4.5% |
| CAGT---------------AGGGAGGC | MMEJ,-15 | 0.30% | 3.7% |
| Total indels | 8.08% | Reads | 41287 |
E
Figure S6. Comparison of RNP editing outcomes in three cell types with inDelphi and FORECasT predictions. (A-D) Comparison of inDelphi (top left), FORECasT (top right) predictions, and our RNP editing results in K562 (bottom left), iPSCs (bottom middle), and T cells (bottom right). Wild-type sequences are displayed at the top of each table. The vertical line indicates the Cas9 cleavage site; lower-case red-letter denotes inserted base; micro-homologies are underlined. PAM sequences (NGG) are marked in bold. Top left and middle, inDelphi and FORECasT predictions with gRNAs that target AAVS1 (A), BCL11A (B-C), and MYH6 (D). For inDelphi, we showed the predictions of K562. Top right, a summary of the two representative NHEJ and MMEJ editing patterns highlighted in each table compared with inDelphi and FORECasT predictions. (E) A summary of the two representative NHEJ (+A/T) and MMEJ (-3~-21 bp) editing patterns. Relative occurrences were summarized from major editing outcomes of 34 gRNAs (FORECasT, K562, and iPSCs) or 19 gRNAs (T cells). Data are shown as mean ± s.d.. Paired two-sided Student’s t-tests were conducted. ****P < 0.0001, ***P < 0.001, *P < 0.05, n.s., P ≥ 0.05.

## Slide 7
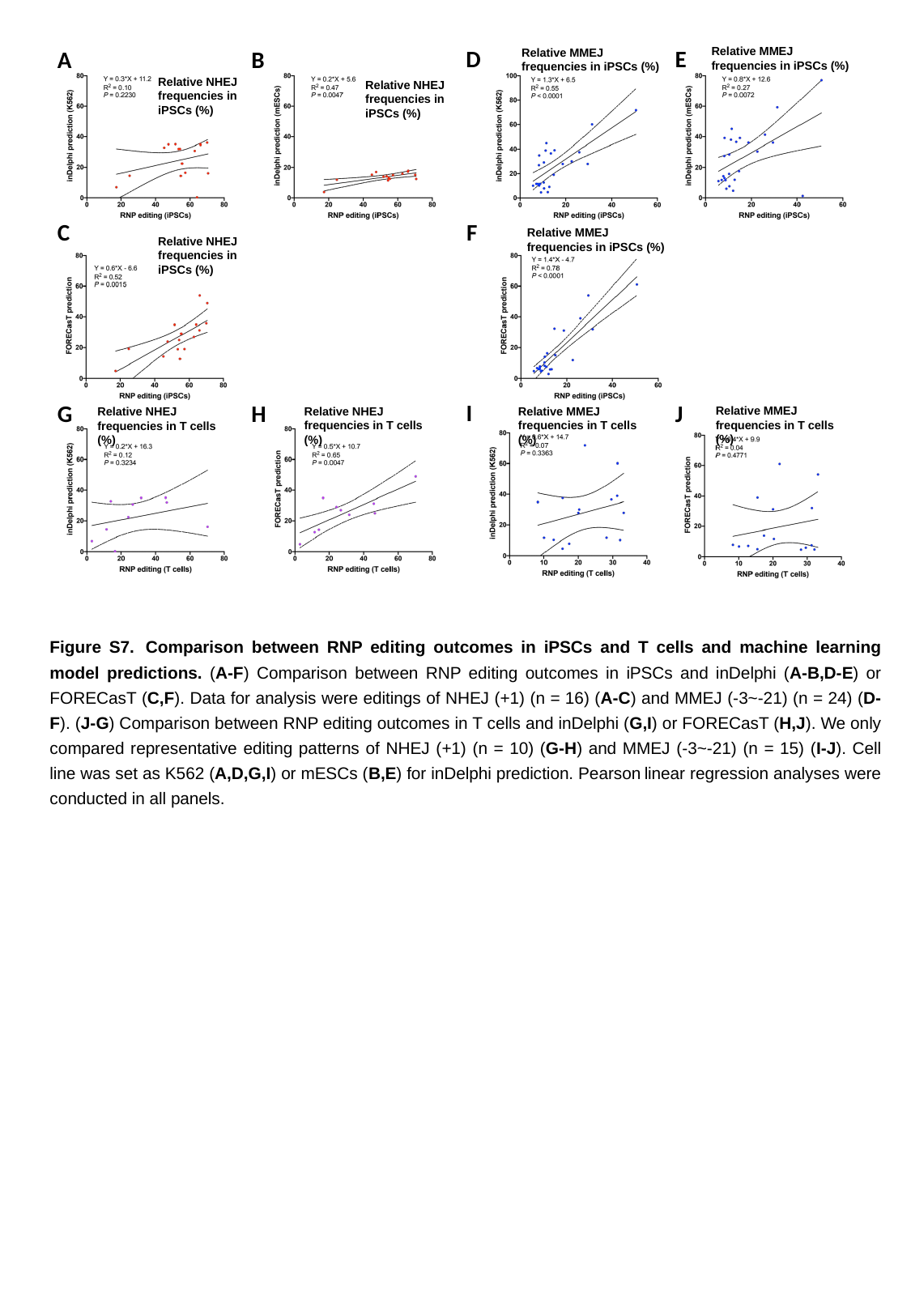

Syn93-crBCL11A-7c
Relative MMEJ frequencies in iPSCs (%)
E
D
B
A
Relative MMEJ frequencies in iPSCs (%)
Relative NHEJ frequencies in iPSCs (%)
Relative NHEJ frequencies in iPSCs (%)
C
F
Relative MMEJ frequencies in iPSCs (%)
Relative NHEJ frequencies in iPSCs (%)
I
J
G
H
Relative MMEJ frequencies in T cells (%)
Relative NHEJ frequencies in T cells (%)
Relative MMEJ frequencies in T cells (%)
Relative NHEJ frequencies in T cells (%)
Figure S7.  Comparison between RNP editing outcomes in iPSCs and T cells and machine learning model predictions. (A-F) Comparison between RNP editing outcomes in iPSCs and inDelphi (A-B,D-E) or FORECasT (C,F). Data for analysis were editings of NHEJ (+1) (n = 16) (A-C) and MMEJ (-3~-21) (n = 24) (D-F). (J-G) Comparison between RNP editing outcomes in T cells and inDelphi (G,I) or FORECasT (H,J). We only compared representative editing patterns of NHEJ (+1) (n = 10) (G-H) and MMEJ (-3~-21) (n = 15) (I-J). Cell line was set as K562 (A,D,G,I) or mESCs (B,E) for inDelphi prediction. Pearson linear regression analyses were conducted in all panels.

## Slide 8
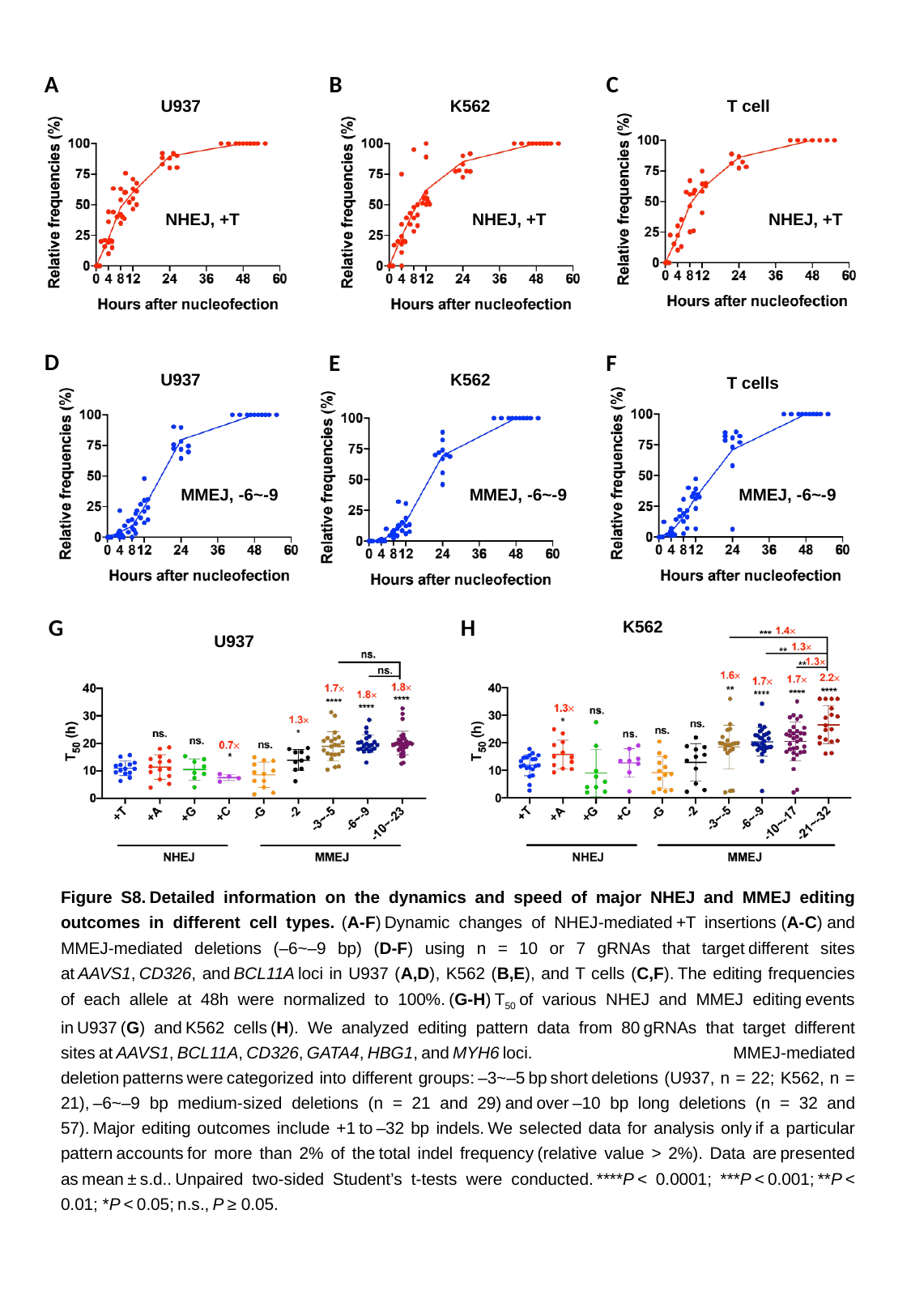

A
C
B
K562
T cell
U937
 ​NHEJ, +T
 ​NHEJ, +T
 ​NHEJ, +T
D
F
E
K562
U937
T cells
MMEJ, -6~-9
MMEJ, -6~-9
MMEJ, -6~-9
G
H
K562
U937
Figure S8. Detailed information on the dynamics and speed of major NHEJ and MMEJ editing outcomes in different cell types.  (A-F) Dynamic changes of NHEJ-mediated +T insertions (A-C) and MMEJ-mediated deletions (–6~–9 bp) (D-F) using n = 10 or 7 gRNAs that target different sites at AAVS1, CD326, and BCL11A loci in U937 (A,D), K562 (B,E), and T cells (C,F). The editing frequencies of each allele at 48h were normalized to 100%. (G-H) T50 of various NHEJ and MMEJ editing events in U937 (G) and K562 cells (H). We analyzed editing pattern data from 80 gRNAs that target different sites at AAVS1, BCL11A, CD326, GATA4, HBG1, and MYH6 loci. MMEJ-mediated deletion patterns were categorized into different groups: –3~–5 bp short deletions (U937, n = 22; K562, n = 21), –6~–9 bp medium-sized deletions (n = 21 and 29) and over –10 bp long deletions (n = 32 and 57). Major editing outcomes include +1 to –32 bp indels. We selected data for analysis only if a particular pattern accounts for more than 2% of the total indel frequency (relative value > 2%).  Data are presented as mean ± s.d.. Unpaired two-sided Student’s t-tests were conducted. ****P < 0.0001; ***P < 0.001; **P < 0.01; *P < 0.05; n.s., P ≥ 0.05.

## Slide 9
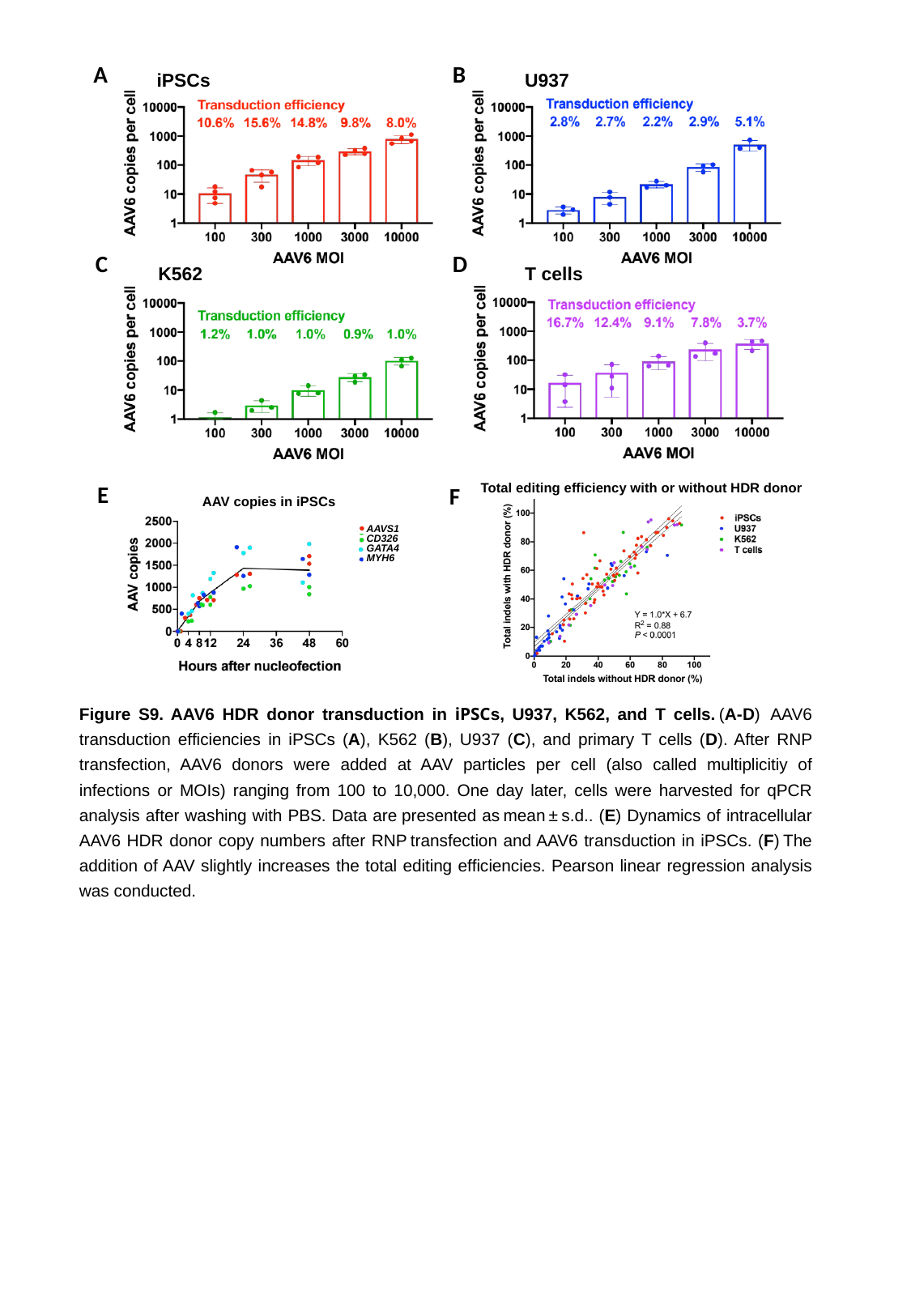

A
B
U937
iPSCs
C
D
T cells
K562
Total editing efficiency with or without HDR donor
E
F
AAV copies in iPSCs
AAVS1
CD326
GATA4
MYH6
Figure S9. AAV6 HDR donor transduction in iPSCs, U937, K562, and T cells. (A-D)  AAV6 transduction efficiencies in iPSCs (A), K562 (B), U937 (C), and primary T cells (D). After RNP transfection, AAV6 donors were added at AAV particles per cell (also called multiplicitiy of infections or MOIs) ranging from 100 to 10,000. One day later, cells were harvested for qPCR analysis after washing with PBS. Data are presented as mean ± s.d.. (E) Dynamics of intracellular AAV6 HDR donor copy numbers after RNP transfection and AAV6 transduction in iPSCs. (F) The addition of AAV slightly increases the total editing efficiencies. Pearson linear regression analysis was conducted.

## Slide 10
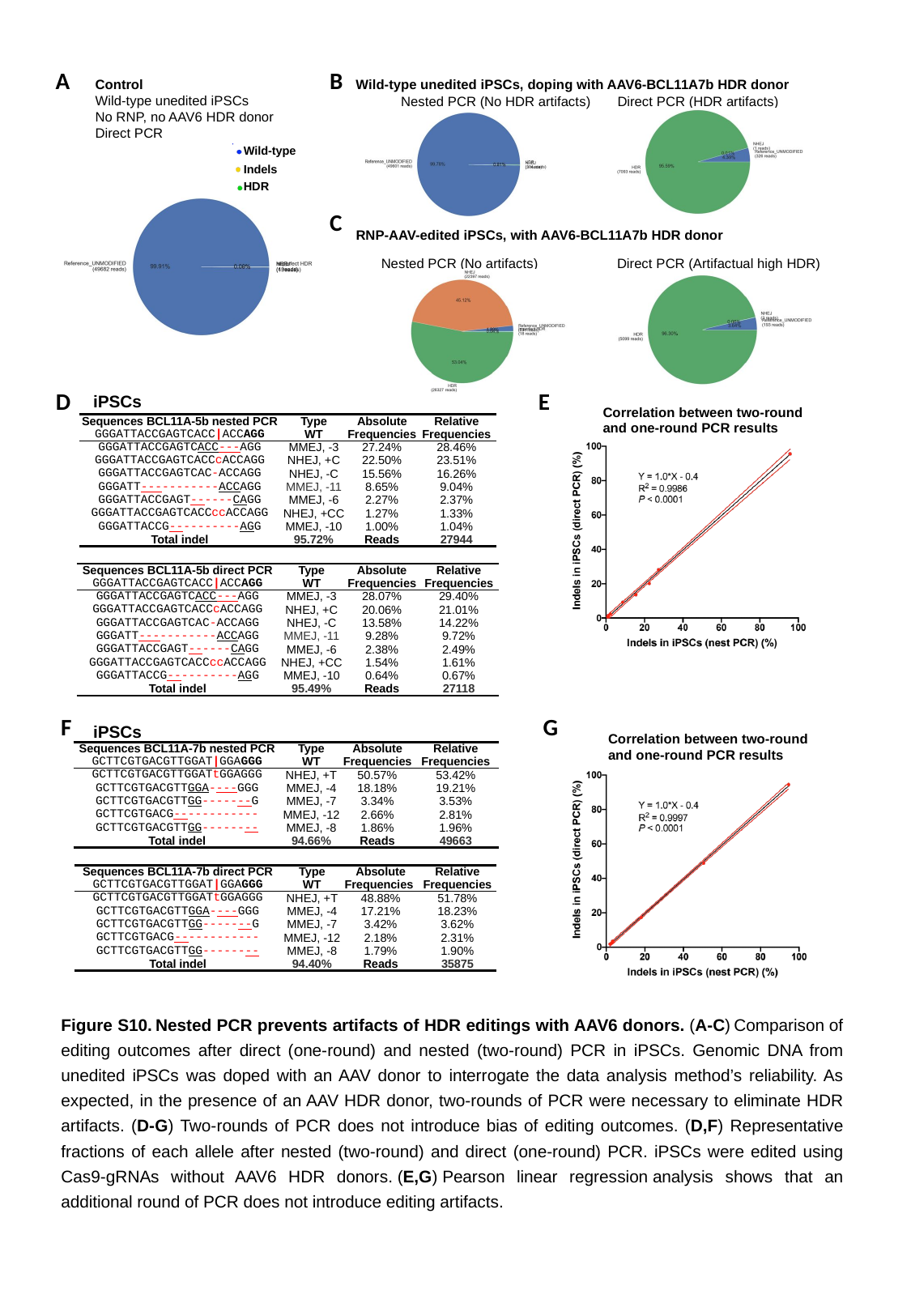

A
B
Control
Wild-type unedited iPSCs
No RNP, no AAV6 HDR donor
Direct PCR
Wild-type unedited iPSCs, doping with AAV6-BCL11A7b HDR donor
Nested PCR (No HDR artifacts)
Direct PCR (HDR artifacts)
Wild-type
Indels
HDR
C
RNP-AAV-edited iPSCs, with AAV6-BCL11A7b HDR donor
Nested PCR (No artifacts)
Direct PCR (Artifactual high HDR)
D
E
iPSCs
Correlation between two-round and one-round PCR results
| Sequences BCL11A-5b nested PCR GGGATTACCGAGTCACC|ACCAGG | Type WT | Absolute Frequencies | Relative Frequencies |
| --- | --- | --- | --- |
| GGGATTACCGAGTCACC---AGG | MMEJ, -3 | 27.24% | 28.46% |
| GGGATTACCGAGTCACCcACCAGG | NHEJ, +C | 22.50% | 23.51% |
| GGGATTACCGAGTCAC-ACCAGG | NHEJ, -C | 15.56% | 16.26% |
| GGGATT-----------ACCAGG | MMEJ, -11 | 8.65% | 9.04% |
| GGGATTACCGAGT------CAGG | MMEJ, -6 | 2.27% | 2.37% |
| GGGATTACCGAGTCACCccACCAGG | NHEJ, +CC | 1.27% | 1.33% |
| GGGATTACCG----------AGG | MMEJ, -10 | 1.00% | 1.04% |
| Total indel | 95.72% | Reads | 27944 |
| Sequences BCL11A-5b direct PCR GGGATTACCGAGTCACC|ACCAGG | Type WT | Absolute Frequencies | Relative Frequencies |
| --- | --- | --- | --- |
| GGGATTACCGAGTCACC---AGG | MMEJ, -3 | 28.07% | 29.40% |
| GGGATTACCGAGTCACCcACCAGG | NHEJ, +C | 20.06% | 21.01% |
| GGGATTACCGAGTCAC-ACCAGG | NHEJ, -C | 13.58% | 14.22% |
| GGGATT-----------ACCAGG | MMEJ, -11 | 9.28% | 9.72% |
| GGGATTACCGAGT------CAGG | MMEJ, -6 | 2.38% | 2.49% |
| GGGATTACCGAGTCACCccACCAGG | NHEJ, +CC | 1.54% | 1.61% |
| GGGATTACCG----------AGG | MMEJ, -10 | 0.64% | 0.67% |
| Total indel | 95.49% | Reads | 27118 |
F
G
iPSCs
Correlation between two-round and one-round PCR results
| Sequences BCL11A-7b nested PCR GCTTCGTGACGTTGGAT|GGAGGG | Type WT | Absolute Frequencies | Relative Frequencies |
| --- | --- | --- | --- |
| GCTTCGTGACGTTGGATtGGAGGG | NHEJ, +T | 50.57% | 53.42% |
| GCTTCGTGACGTTGGA----GGG | MMEJ, -4 | 18.18% | 19.21% |
| GCTTCGTGACGTTGG-------G | MMEJ, -7 | 3.34% | 3.53% |
| GCTTCGTGACG------------ | MMEJ, -12 | 2.66% | 2.81% |
| GCTTCGTGACGTTGG-------- | MMEJ, -8 | 1.86% | 1.96% |
| Total indel | 94.66% | Reads | 49663 |
| Sequences BCL11A-7b direct PCR GCTTCGTGACGTTGGAT|GGAGGG | Type WT | Absolute Frequencies | Relative Frequencies |
| --- | --- | --- | --- |
| GCTTCGTGACGTTGGATtGGAGGG | NHEJ, +T | 48.88% | 51.78% |
| GCTTCGTGACGTTGGA----GGG | MMEJ, -4 | 17.21% | 18.23% |
| GCTTCGTGACGTTGG-------G | MMEJ, -7 | 3.42% | 3.62% |
| GCTTCGTGACG------------ | MMEJ, -12 | 2.18% | 2.31% |
| GCTTCGTGACGTTGG-------- | MMEJ, -8 | 1.79% | 1.90% |
| Total indel | 94.40% | Reads | 35875 |
Figure S10. Nested PCR prevents artifacts of HDR editings with AAV6 donors. (A-C) Comparison of editing outcomes after direct (one-round) and nested (two-round) PCR in iPSCs. Genomic DNA from unedited iPSCs was doped with an AAV donor to interrogate the data analysis method’s reliability. As expected, in the presence of an AAV HDR donor, two-rounds of PCR were necessary to eliminate HDR artifacts. (D-G) Two-rounds of PCR does not introduce bias of editing outcomes. (D,F) Representative fractions of each allele after nested (two-round) and direct (one-round) PCR. iPSCs were edited using Cas9-gRNAs without AAV6 HDR donors. (E,G) Pearson linear regression analysis shows that an additional round of PCR does not introduce editing artifacts.

## Slide 11
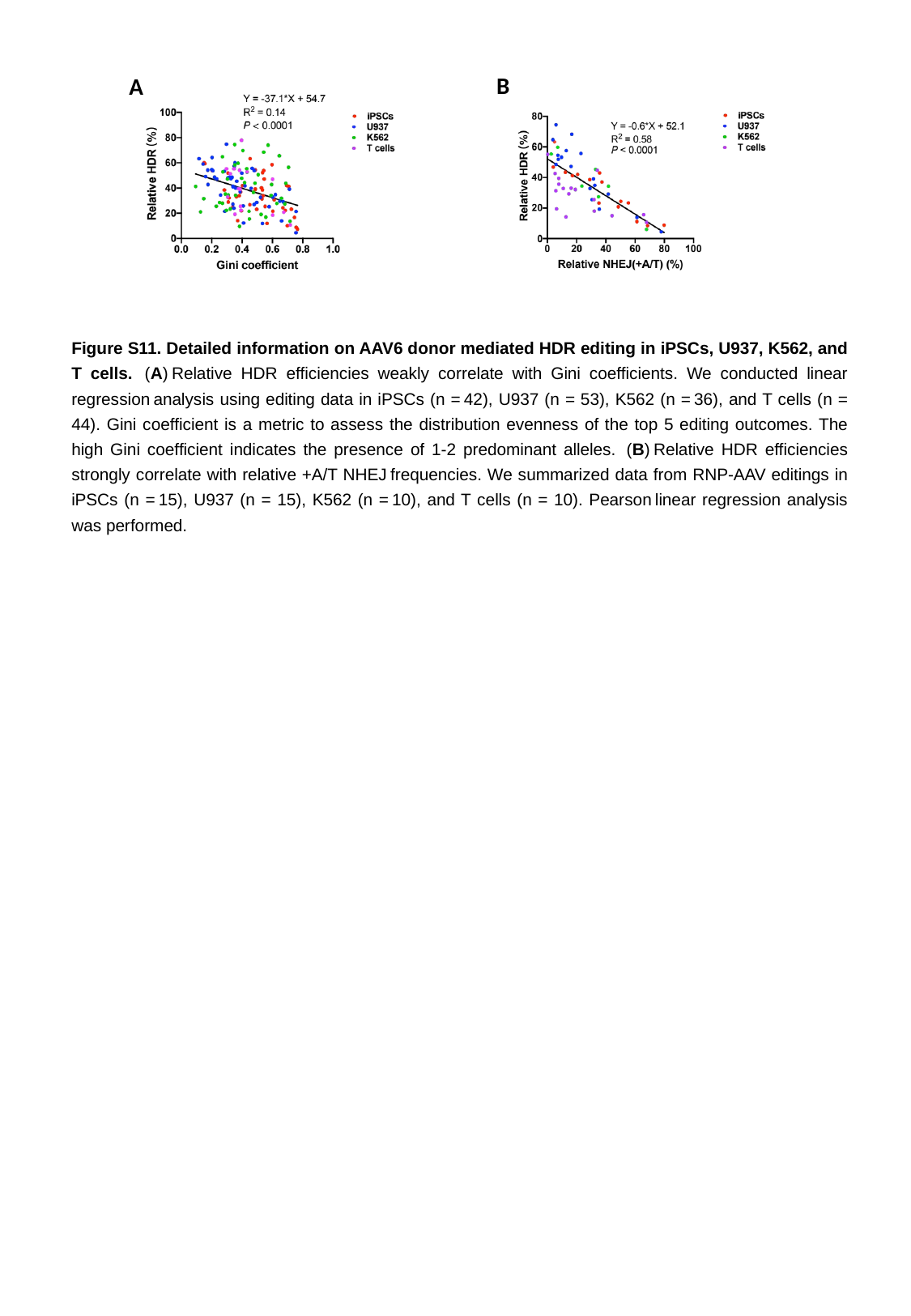

B
A
Figure S11. Detailed information on AAV6 donor mediated HDR editing in iPSCs, U937, K562, and T cells.  (A) Relative HDR efficiencies weakly correlate with Gini coefficients. We conducted linear regression analysis using editing data in iPSCs (n = 42), U937 (n = 53), K562 (n = 36), and T cells (n = 44). Gini coefficient is a metric to assess the distribution evenness of the top 5 editing outcomes. The high Gini coefficient indicates the presence of 1-2 predominant alleles.  (B) Relative HDR efficiencies strongly correlate with relative +A/T NHEJ frequencies. We summarized data from RNP-AAV editings in iPSCs (n = 15), U937 (n = 15), K562 (n = 10), and T cells (n = 10). Pearson linear regression analysis was performed.

## Slide 12
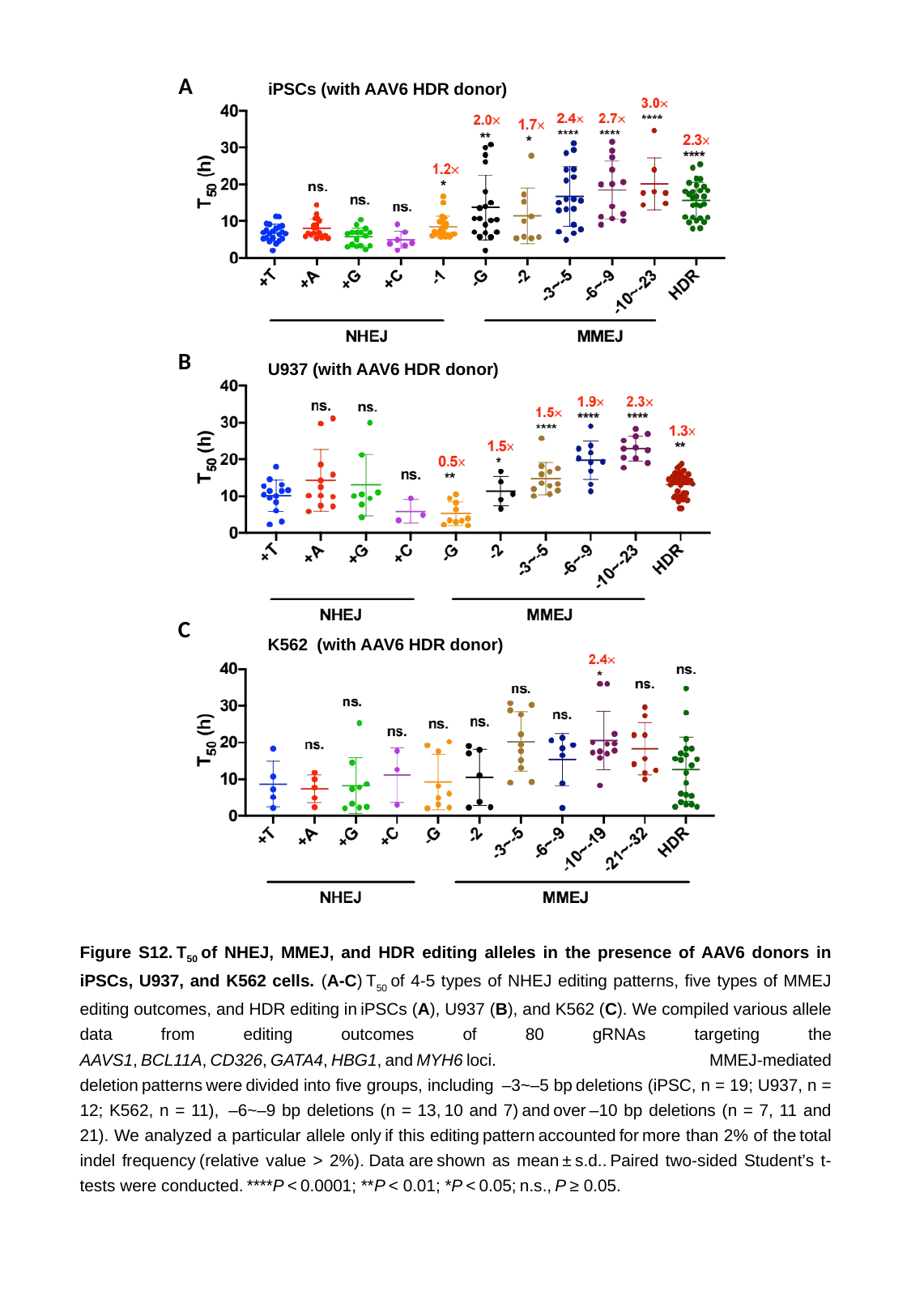

A
iPSCs (with AAV6 HDR donor)
B
U937 (with AAV6 HDR donor)
C
K562  (with AAV6 HDR donor)
Figure S12. T50 of NHEJ, MMEJ, and HDR editing alleles in the presence of AAV6 donors in iPSCs, U937, and K562 cells.  (A-C) T50 of 4-5 types of NHEJ editing patterns, five types of MMEJ editing outcomes, and HDR editing in iPSCs (A), U937 (B), and K562 (C). We compiled various allele data from editing outcomes of 80 gRNAs targeting the AAVS1, BCL11A, CD326, GATA4, HBG1, and MYH6 loci. MMEJ-mediated deletion patterns were divided into five groups, including  –3~–5 bp deletions (iPSC, n = 19; U937, n = 12; K562, n = 11),  –6~–9 bp deletions (n = 13, 10 and 7) and over –10 bp deletions (n = 7, 11 and 21). We analyzed a particular allele only if this editing pattern accounted for more than 2% of the total indel frequency (relative value > 2%). Data are shown as mean ± s.d.. Paired two-sided Student’s t-tests were conducted. ****P < 0.0001; **P < 0.01; *P < 0.05; n.s., P ≥ 0.05.

## Slide 13
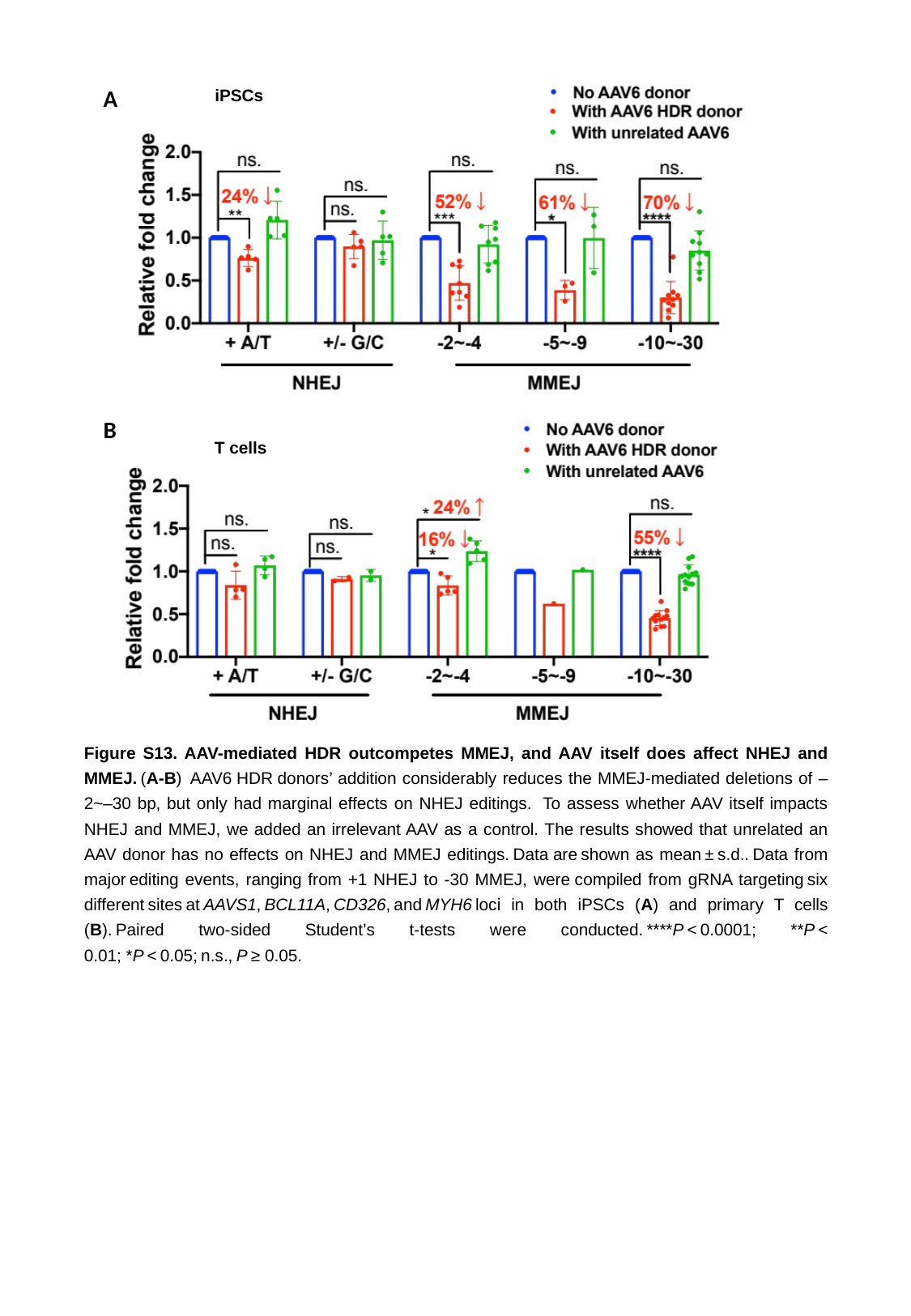

A
iPSCs
B
T cells
Figure S13. AAV-mediated HDR outcompetes MMEJ, and AAV itself does affect NHEJ and MMEJ. (A-B)  AAV6 HDR donors’ addition considerably reduces the MMEJ-mediated deletions of –2~–30 bp, but only had marginal effects on NHEJ editings.  To assess whether AAV itself impacts NHEJ and MMEJ, we added an irrelevant AAV as a control. The results showed that unrelated an AAV donor has no effects on NHEJ and MMEJ editings. Data are shown as mean ± s.d.. Data from major editing events, ranging from +1 NHEJ to -30 MMEJ, were compiled from gRNA targeting six different sites at AAVS1, BCL11A, CD326, and MYH6 loci in both iPSCs (A) and primary T cells (B). Paired two-sided Student’s t-tests were conducted. ****P < 0.0001; **P < 0.01; *P < 0.05; n.s., P ≥ 0.05.

## Slide 14
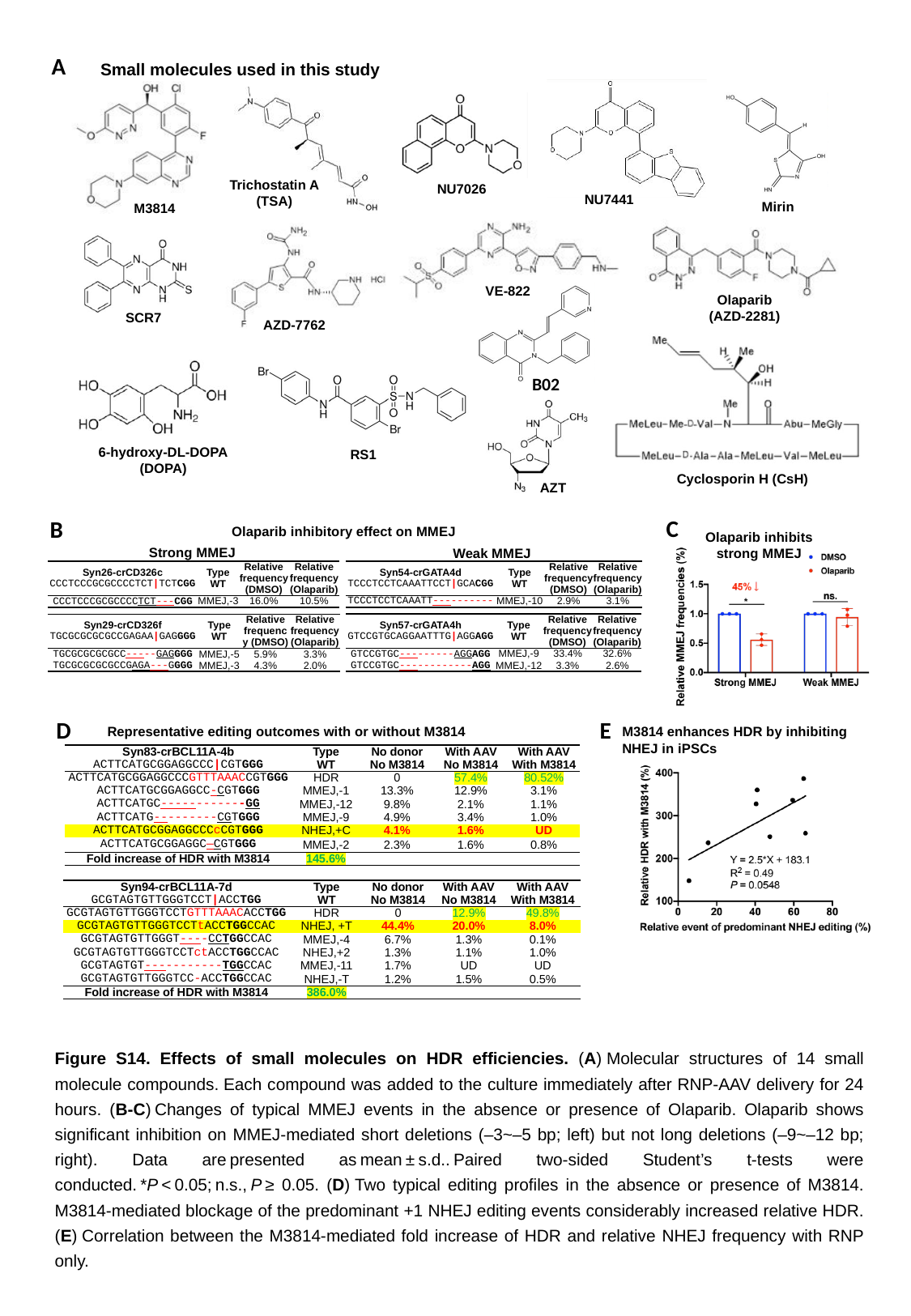

A
Small molecules​ used in this study
Trichostatin A
(TSA)
NU7026
NU7441
Mirin
M3814
VE-822
Olaparib
(AZD-2281)
SCR7
AZD-7762
B02
6-hydroxy-DL-DOPA
(DOPA)
RS1
Cyclosporin H (CsH)
AZT
C
B
Olaparib inhibitory effect on MMEJ
Olaparib inhibits
strong MMEJ
Strong MMEJ
Weak MMEJ
| Syn54-crGATA4d TCCCTCCTCAAATTCCT|GCACGG | Type WT | Relative frequency (DMSO) | Relative frequency (Olaparib) |
| --- | --- | --- | --- |
| TCCCTCCTCAAATT---------- | MMEJ,-10 | 2.9% | 3.1% |
| Syn26-crCD326c CCCTCCCGCGCCCCTCT|TCTCGG | Type WT | Relative frequency (DMSO) | Relative frequency (Olaparib) |
| --- | --- | --- | --- |
| CCCTCCCGCGCCCCTCT---CGG | MMEJ,-3 | 16.0% | 10.5% |
| Syn57-crGATA4h GTCCGTGCAGGAATTTG|AGGAGG | Type WT | Relative frequency (DMSO) | Relative frequency (Olaparib) |
| --- | --- | --- | --- |
| GTCCGTGC---------AGGAGG | MMEJ,-9 | 33.4% | 32.6% |
| GTCCGTGC------------AGG | MMEJ,-12 | 3.3% | 2.6% |
| Syn29-crCD326f TGCGCGCGCGCCGAGAA|GAGGGG | Type WT | Relative frequency (DMSO) | Relative frequency (Olaparib) |
| --- | --- | --- | --- |
| TGCGCGCGCGCC-----GAGGGG | MMEJ,-5 | 5.9% | 3.3% |
| TGCGCGCGCGCCGAGA---GGGG | MMEJ,-3 | 4.3% | 2.0% |
D
E
Representative editing outcomes with or without M3814
M3814 enhances HDR by inhibiting NHEJ in iPSCs
| Syn83-crBCL11A-4b ACTTCATGCGGAGGCCC|CGTGGG | Type WT | No donor No M3814 | With AAV No M3814 | With AAV With M3814 |
| --- | --- | --- | --- | --- |
| ACTTCATGCGGAGGCCCGTTTAAACCGTGGG | HDR | 0 | 57.4% | 80.52% |
| ACTTCATGCGGAGGCC-CGTGGG | MMEJ,-1 | 13.3% | 12.9% | 3.1% |
| ACTTCATGC------------GG | MMEJ,-12 | 9.8% | 2.1% | 1.1% |
| ACTTCATG---------CGTGGG | MMEJ,-9 | 4.9% | 3.4% | 1.0% |
| ACTTCATGCGGAGGCCCcCGTGGG | NHEJ,+C | 4.1% | 1.6% | UD |
| ACTTCATGCGGAGGC—CGTGGG | MMEJ,-2 | 2.3% | 1.6% | 0.8% |
| Fold increase of HDR with M3814 | 145.6% | | | |
| Syn94-crBCL11A-7d GCGTAGTGTTGGGTCCT|ACCTGG | Type WT | No donor No M3814 | With AAV No M3814 | With AAV With M3814 |
| --- | --- | --- | --- | --- |
| GCGTAGTGTTGGGTCCTGTTTAAACACCTGG | HDR | 0 | 12.9% | 49.8% |
| GCGTAGTGTTGGGTCCTtACCTGGCCAC | NHEJ, +T | 44.4% | 20.0% | 8.0% |
| GCGTAGTGTTGGGT----CCTGGCCAC | MMEJ,-4 | 6.7% | 1.3% | 0.1% |
| GCGTAGTGTTGGGTCCTctACCTGGCCAC | NHEJ,+2 | 1.3% | 1.1% | 1.0% |
| GCGTAGTGT-----------TGGCCAC | MMEJ,-11 | 1.7% | UD | UD |
| GCGTAGTGTTGGGTCC-ACCTGGCCAC | NHEJ,-T | 1.2% | 1.5% | 0.5% |
| Fold increase of HDR with M3814 | 386.0% | | | |
Figure S14. Effects of small molecules on HDR efficiencies. (A) Molecular structures of 14 small molecule compounds. Each compound was added to the culture immediately after RNP-AAV delivery for 24 hours. (B-C) Changes of typical MMEJ events in the absence or presence of Olaparib. Olaparib shows significant inhibition on MMEJ-mediated short deletions (–3~–5 bp; left) but not long deletions (–9~–12 bp; right). Data are presented as mean ± s.d.. Paired two-sided Student’s t-tests were conducted. *P < 0.05; n.s., P ≥ 0.05. ​(D) Two typical editing profiles in the absence or presence of M3814. M3814-mediated blockage of the predominant +1 NHEJ editing events considerably increased relative HDR. (E) Correlation between the M3814-mediated fold increase of HDR and relative NHEJ frequency with RNP only.

## Slide 15
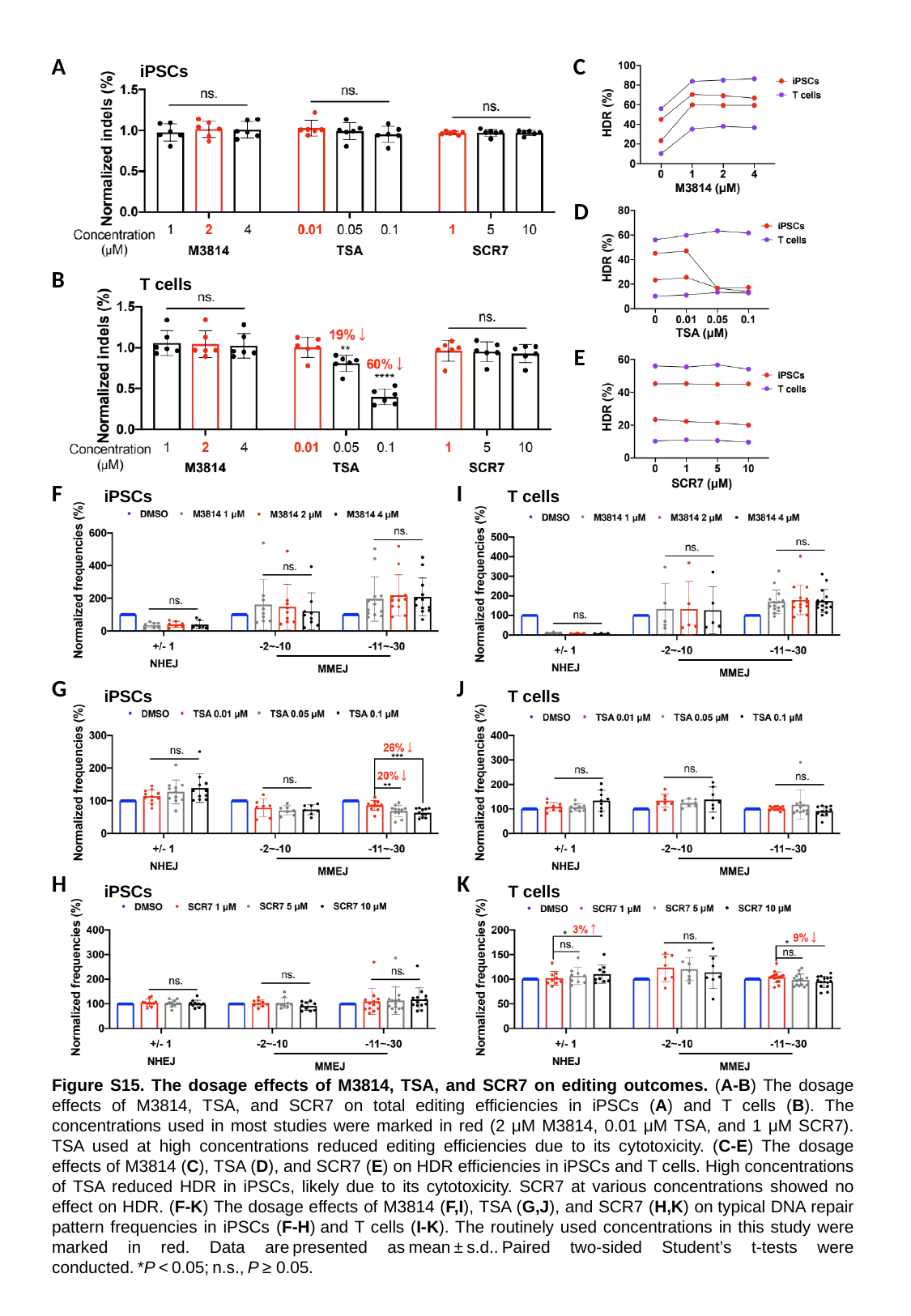

A
C
iPSCs
D
B
T cells
E
F
I
T cells
iPSCs
G
J
T cells
iPSCs
H
K
T cells
iPSCs
Figure S15. The dosage effects of M3814, TSA, and SCR7 on editing outcomes. (A-B) The dosage effects of M3814, TSA, and SCR7 on total editing efficiencies in iPSCs (A) and T cells (B). The concentrations used in most studies were marked in red (2 μM M3814, 0.01 μM TSA, and 1 μM SCR7). TSA used at high concentrations reduced editing efficiencies due to its cytotoxicity. (C-E) The dosage effects of M3814 (C), TSA (D), and SCR7 (E) on HDR efficiencies in iPSCs and T cells. High concentrations of TSA reduced HDR in iPSCs, likely due to its cytotoxicity. SCR7 at various concentrations showed no effect on HDR. (F-K) The dosage effects of M3814 (F,I), TSA (G,J), and SCR7 (H,K) on typical DNA repair pattern frequencies in iPSCs (F-H) and T cells (I-K). The routinely used concentrations in this study were marked in red. Data are presented as mean ± s.d.. Paired two-sided Student’s t-tests were conducted. *P < 0.05; n.s., P ≥ 0.05.

## Slide 16
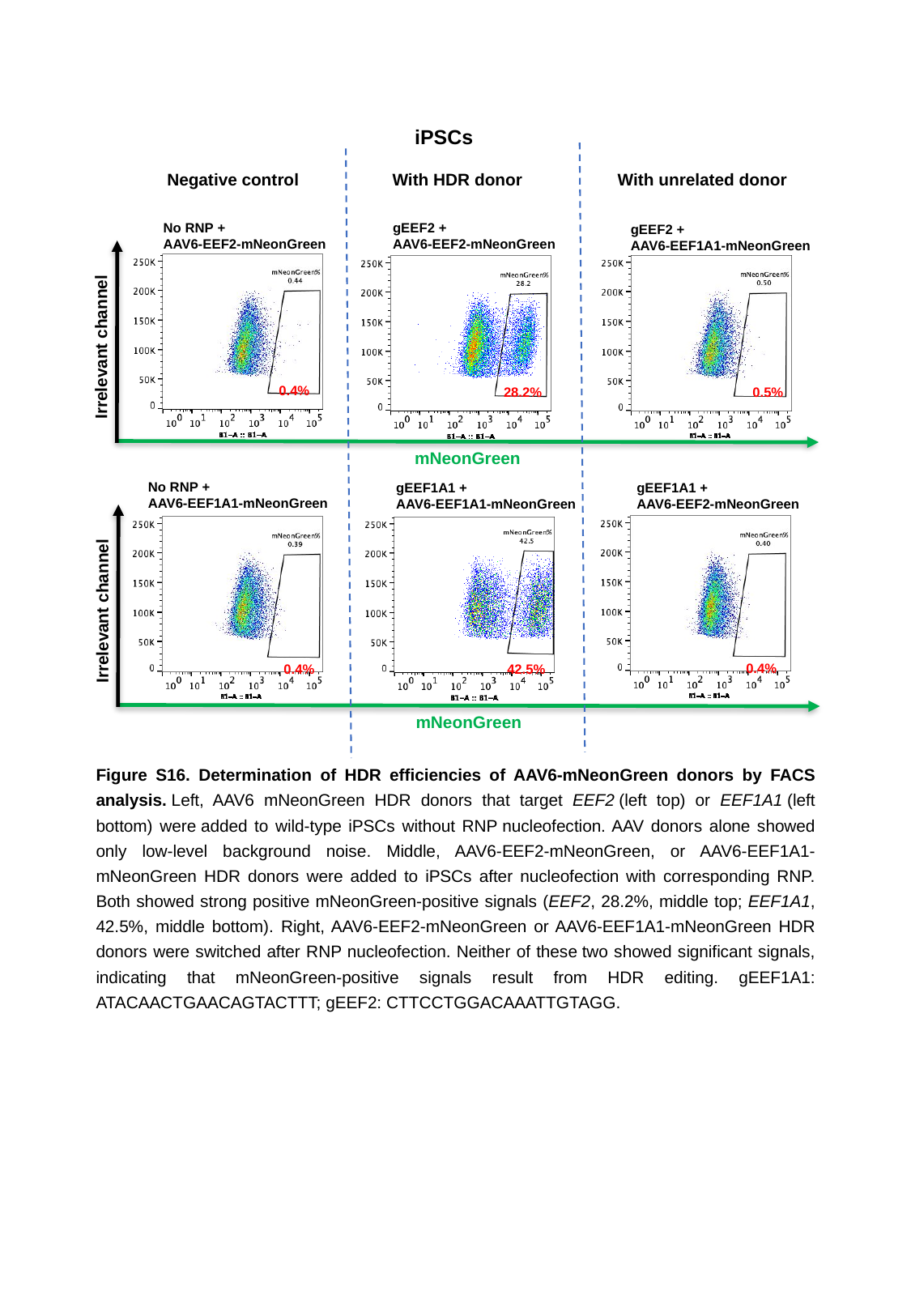

iPSCs
Negative control
With HDR donor
With unrelated donor
No RNP +
AAV6-EEF2-mNeonGreen
gEEF2 +
AAV6-EEF2-mNeonGreen
gEEF2 +
AAV6-EEF1A1-mNeonGreen
Irrelevant channel
mNeonGreen
0.4%
28.2%
0.5%
No RNP +
AAV6-EEF1A1-mNeonGreen
gEEF1A1 +
AAV6-EEF1A1-mNeonGreen
gEEF1A1 +
AAV6-EEF2-mNeonGreen
Irrelevant channel
mNeonGreen
0.4%
42.5%
0.4%
Figure S16. Determination of HDR efficiencies of AAV6-mNeonGreen donors by FACS analysis. Left, AAV6 mNeonGreen HDR donors that target EEF2 (left top) or EEF1A1 (left bottom) were added to wild-type iPSCs without RNP nucleofection. AAV donors alone showed only low-level background noise. Middle, AAV6-EEF2-mNeonGreen, or AAV6-EEF1A1-mNeonGreen HDR donors were added to iPSCs after nucleofection with corresponding RNP. Both showed strong positive mNeonGreen-positive signals (EEF2, 28.2%, middle top; EEF1A1, 42.5%, middle bottom). Right, AAV6-EEF2-mNeonGreen or AAV6-EEF1A1-mNeonGreen HDR donors were switched after RNP nucleofection. Neither of these two showed significant signals, indicating that mNeonGreen-positive signals result from HDR editing. gEEF1A1: ATACAACTGAACAGTACTTT; gEEF2: CTTCCTGGACAAATTGTAGG.

## Slide 17
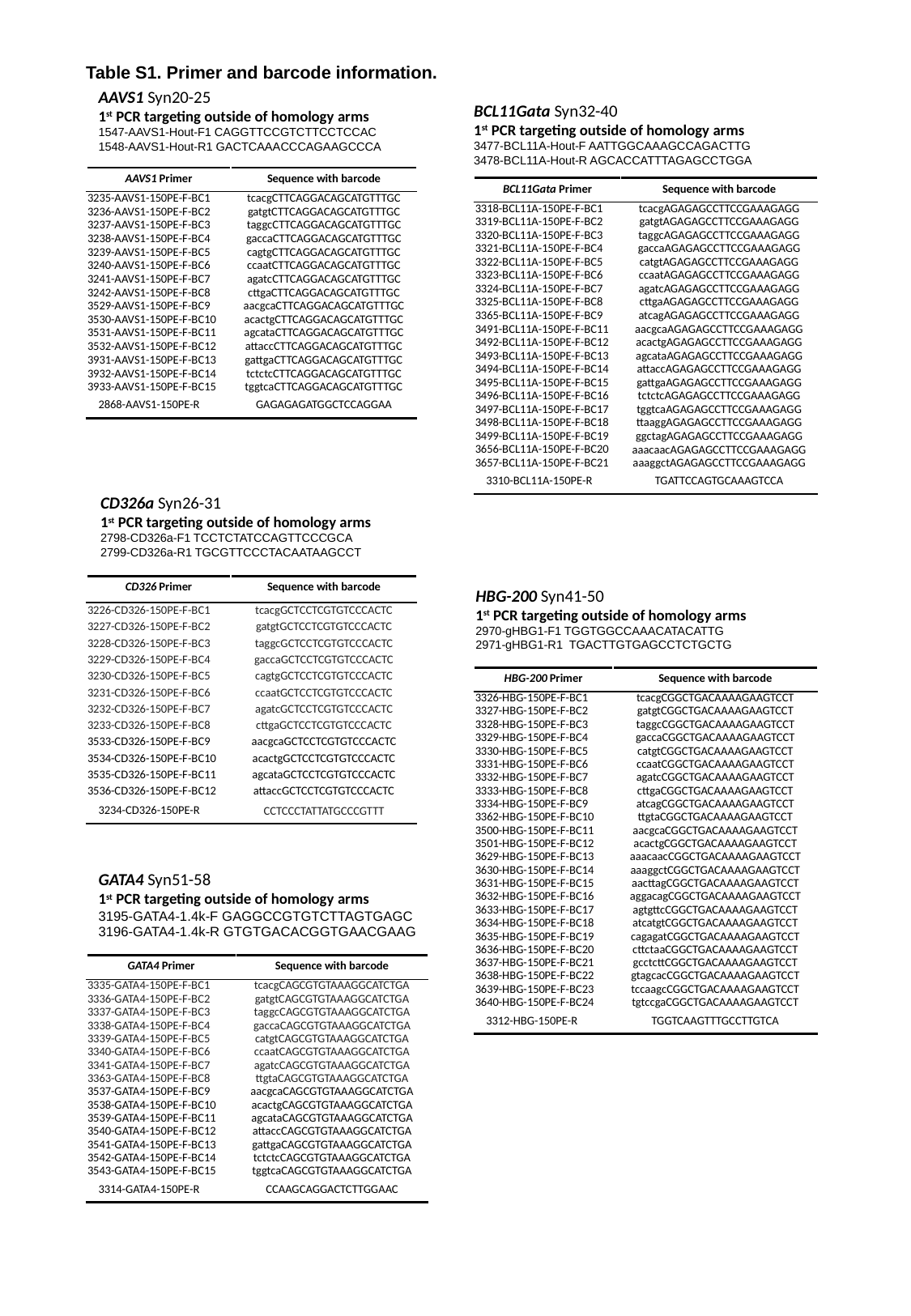

Table S1. Primer and barcode information.
AAVS1 Syn20-25
1st PCR targeting outside of homology arms
1547-AAVS1-Hout-F1 CAGGTTCCGTCTTCCTCCAC
1548-AAVS1-Hout-R1 GACTCAAACCCAGAAGCCCA
BCL11Gata Syn32-40
1st PCR targeting outside of homology arms
3477-BCL11A-Hout-F AATTGGCAAAGCCAGACTTG
3478-BCL11A-Hout-R AGCACCATTTAGAGCCTGGA
| AAVS1 Primer | Sequence with barcode |
| --- | --- |
| 3235-AAVS1-150PE-F-BC1 | tcacgCTTCAGGACAGCATGTTTGC |
| 3236-AAVS1-150PE-F-BC2 | gatgtCTTCAGGACAGCATGTTTGC |
| 3237-AAVS1-150PE-F-BC3 | taggcCTTCAGGACAGCATGTTTGC |
| 3238-AAVS1-150PE-F-BC4 | gaccaCTTCAGGACAGCATGTTTGC |
| 3239-AAVS1-150PE-F-BC5 | cagtgCTTCAGGACAGCATGTTTGC |
| 3240-AAVS1-150PE-F-BC6 | ccaatCTTCAGGACAGCATGTTTGC |
| 3241-AAVS1-150PE-F-BC7 | agatcCTTCAGGACAGCATGTTTGC |
| 3242-AAVS1-150PE-F-BC8 | cttgaCTTCAGGACAGCATGTTTGC |
| 3529-AAVS1-150PE-F-BC9 | aacgcaCTTCAGGACAGCATGTTTGC |
| 3530-AAVS1-150PE-F-BC10 | acactgCTTCAGGACAGCATGTTTGC |
| 3531-AAVS1-150PE-F-BC11 | agcataCTTCAGGACAGCATGTTTGC |
| 3532-AAVS1-150PE-F-BC12 | attaccCTTCAGGACAGCATGTTTGC |
| 3931-AAVS1-150PE-F-BC13 | gattgaCTTCAGGACAGCATGTTTGC |
| 3932-AAVS1-150PE-F-BC14 | tctctcCTTCAGGACAGCATGTTTGC |
| 3933-AAVS1-150PE-F-BC15 | tggtcaCTTCAGGACAGCATGTTTGC |
| 2868-AAVS1-150PE-R | GAGAGAGATGGCTCCAGGAA |
| BCL11Gata Primer | Sequence with barcode |
| --- | --- |
| 3318-BCL11A-150PE-F-BC1 | tcacgAGAGAGCCTTCCGAAAGAGG |
| 3319-BCL11A-150PE-F-BC2 | gatgtAGAGAGCCTTCCGAAAGAGG |
| 3320-BCL11A-150PE-F-BC3 | taggcAGAGAGCCTTCCGAAAGAGG |
| 3321-BCL11A-150PE-F-BC4 | gaccaAGAGAGCCTTCCGAAAGAGG |
| 3322-BCL11A-150PE-F-BC5 | catgtAGAGAGCCTTCCGAAAGAGG |
| 3323-BCL11A-150PE-F-BC6 | ccaatAGAGAGCCTTCCGAAAGAGG |
| 3324-BCL11A-150PE-F-BC7 | agatcAGAGAGCCTTCCGAAAGAGG |
| 3325-BCL11A-150PE-F-BC8 | cttgaAGAGAGCCTTCCGAAAGAGG |
| 3365-BCL11A-150PE-F-BC9 | atcagAGAGAGCCTTCCGAAAGAGG |
| 3491-BCL11A-150PE-F-BC11 | aacgcaAGAGAGCCTTCCGAAAGAGG |
| 3492-BCL11A-150PE-F-BC12 | acactgAGAGAGCCTTCCGAAAGAGG |
| 3493-BCL11A-150PE-F-BC13 | agcataAGAGAGCCTTCCGAAAGAGG |
| 3494-BCL11A-150PE-F-BC14 | attaccAGAGAGCCTTCCGAAAGAGG |
| 3495-BCL11A-150PE-F-BC15 | gattgaAGAGAGCCTTCCGAAAGAGG |
| 3496-BCL11A-150PE-F-BC16 | tctctcAGAGAGCCTTCCGAAAGAGG |
| 3497-BCL11A-150PE-F-BC17 | tggtcaAGAGAGCCTTCCGAAAGAGG |
| 3498-BCL11A-150PE-F-BC18 | ttaaggAGAGAGCCTTCCGAAAGAGG |
| 3499-BCL11A-150PE-F-BC19 | ggctagAGAGAGCCTTCCGAAAGAGG |
| 3656-BCL11A-150PE-F-BC20 | aaacaacAGAGAGCCTTCCGAAAGAGG |
| 3657-BCL11A-150PE-F-BC21 | aaaggctAGAGAGCCTTCCGAAAGAGG |
| 3310-BCL11A-150PE-R | TGATTCCAGTGCAAAGTCCA |
CD326a Syn26-31
1st PCR targeting outside of homology arms
2798-CD326a-F1 TCCTCTATCCAGTTCCCGCA
2799-CD326a-R1 TGCGTTCCCTACAATAAGCCT
| CD326 Primer | Sequence with barcode |
| --- | --- |
| 3226-CD326-150PE-F-BC1 | tcacgGCTCCTCGTGTCCCACTC |
| 3227-CD326-150PE-F-BC2 | gatgtGCTCCTCGTGTCCCACTC |
| 3228-CD326-150PE-F-BC3 | taggcGCTCCTCGTGTCCCACTC |
| 3229-CD326-150PE-F-BC4 | gaccaGCTCCTCGTGTCCCACTC |
| 3230-CD326-150PE-F-BC5 | cagtgGCTCCTCGTGTCCCACTC |
| 3231-CD326-150PE-F-BC6 | ccaatGCTCCTCGTGTCCCACTC |
| 3232-CD326-150PE-F-BC7 | agatcGCTCCTCGTGTCCCACTC |
| 3233-CD326-150PE-F-BC8 | cttgaGCTCCTCGTGTCCCACTC |
| 3533-CD326-150PE-F-BC9 | aacgcaGCTCCTCGTGTCCCACTC |
| 3534-CD326-150PE-F-BC10 | acactgGCTCCTCGTGTCCCACTC |
| 3535-CD326-150PE-F-BC11 | agcataGCTCCTCGTGTCCCACTC |
| 3536-CD326-150PE-F-BC12 | attaccGCTCCTCGTGTCCCACTC |
| 3234-CD326-150PE-R | CCTCCCTATTATGCCCGTTT |
HBG-200 Syn41-50
1st PCR targeting outside of homology arms
2970-gHBG1-F1 TGGTGGCCAAACATACATTG
2971-gHBG1-R1  TGACTTGTGAGCCTCTGCTG
| HBG-200 Primer | Sequence with barcode |
| --- | --- |
| 3326-HBG-150PE-F-BC1 | tcacgCGGCTGACAAAAGAAGTCCT |
| 3327-HBG-150PE-F-BC2 | gatgtCGGCTGACAAAAGAAGTCCT |
| 3328-HBG-150PE-F-BC3 | taggcCGGCTGACAAAAGAAGTCCT |
| 3329-HBG-150PE-F-BC4 | gaccaCGGCTGACAAAAGAAGTCCT |
| 3330-HBG-150PE-F-BC5 | catgtCGGCTGACAAAAGAAGTCCT |
| 3331-HBG-150PE-F-BC6 | ccaatCGGCTGACAAAAGAAGTCCT |
| 3332-HBG-150PE-F-BC7 | agatcCGGCTGACAAAAGAAGTCCT |
| 3333-HBG-150PE-F-BC8 | cttgaCGGCTGACAAAAGAAGTCCT |
| 3334-HBG-150PE-F-BC9 | atcagCGGCTGACAAAAGAAGTCCT |
| 3362-HBG-150PE-F-BC10 | ttgtaCGGCTGACAAAAGAAGTCCT |
| 3500-HBG-150PE-F-BC11 | aacgcaCGGCTGACAAAAGAAGTCCT |
| 3501-HBG-150PE-F-BC12 | acactgCGGCTGACAAAAGAAGTCCT |
| 3629-HBG-150PE-F-BC13 | aaacaacCGGCTGACAAAAGAAGTCCT |
| 3630-HBG-150PE-F-BC14 | aaaggctCGGCTGACAAAAGAAGTCCT |
| 3631-HBG-150PE-F-BC15 | aacttagCGGCTGACAAAAGAAGTCCT |
| 3632-HBG-150PE-F-BC16 | aggacagCGGCTGACAAAAGAAGTCCT |
| 3633-HBG-150PE-F-BC17 | agtgttcCGGCTGACAAAAGAAGTCCT |
| 3634-HBG-150PE-F-BC18 | atcatgtCGGCTGACAAAAGAAGTCCT |
| 3635-HBG-150PE-F-BC19 | cagagatCGGCTGACAAAAGAAGTCCT |
| 3636-HBG-150PE-F-BC20 | cttctaaCGGCTGACAAAAGAAGTCCT |
| 3637-HBG-150PE-F-BC21 | gcctcttCGGCTGACAAAAGAAGTCCT |
| 3638-HBG-150PE-F-BC22 | gtagcacCGGCTGACAAAAGAAGTCCT |
| 3639-HBG-150PE-F-BC23 | tccaagcCGGCTGACAAAAGAAGTCCT |
| 3640-HBG-150PE-F-BC24 | tgtccgaCGGCTGACAAAAGAAGTCCT |
| 3312-HBG-150PE-R | TGGTCAAGTTTGCCTTGTCA |
GATA4 Syn51-58
1st PCR targeting outside of homology arms
3195-GATA4-1.4k-F GAGGCCGTGTCTTAGTGAGC
3196-GATA4-1.4k-R GTGTGACACGGTGAACGAAG
| GATA4 Primer | Sequence with barcode |
| --- | --- |
| 3335-GATA4-150PE-F-BC1 | tcacgCAGCGTGTAAAGGCATCTGA |
| 3336-GATA4-150PE-F-BC2 | gatgtCAGCGTGTAAAGGCATCTGA |
| 3337-GATA4-150PE-F-BC3 | taggcCAGCGTGTAAAGGCATCTGA |
| 3338-GATA4-150PE-F-BC4 | gaccaCAGCGTGTAAAGGCATCTGA |
| 3339-GATA4-150PE-F-BC5 | catgtCAGCGTGTAAAGGCATCTGA |
| 3340-GATA4-150PE-F-BC6 | ccaatCAGCGTGTAAAGGCATCTGA |
| 3341-GATA4-150PE-F-BC7 | agatcCAGCGTGTAAAGGCATCTGA |
| 3363-GATA4-150PE-F-BC8 | ttgtaCAGCGTGTAAAGGCATCTGA |
| 3537-GATA4-150PE-F-BC9 | aacgcaCAGCGTGTAAAGGCATCTGA |
| 3538-GATA4-150PE-F-BC10 | acactgCAGCGTGTAAAGGCATCTGA |
| 3539-GATA4-150PE-F-BC11 | agcataCAGCGTGTAAAGGCATCTGA |
| 3540-GATA4-150PE-F-BC12 | attaccCAGCGTGTAAAGGCATCTGA |
| 3541-GATA4-150PE-F-BC13 | gattgaCAGCGTGTAAAGGCATCTGA |
| 3542-GATA4-150PE-F-BC14 | tctctcCAGCGTGTAAAGGCATCTGA |
| 3543-GATA4-150PE-F-BC15 | tggtcaCAGCGTGTAAAGGCATCTGA |
| 3314-GATA4-150PE-R | CCAAGCAGGACTCTTGGAAC |

## Slide 18
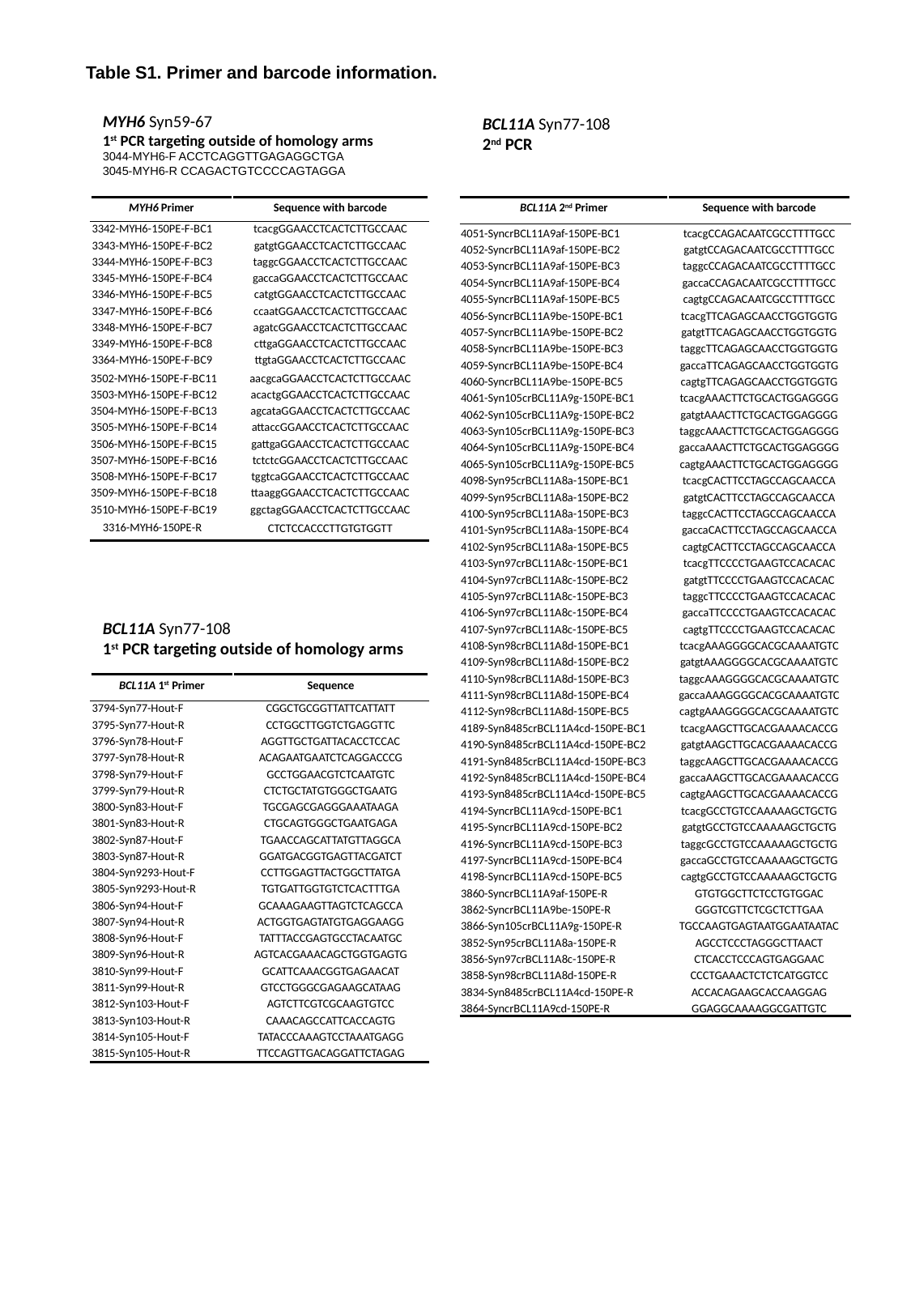

Table S1. Primer and barcode information.
MYH6 Syn59-67
1st PCR targeting outside of homology arms
3044-MYH6-F ACCTCAGGTTGAGAGGCTGA
3045-MYH6-R CCAGACTGTCCCCAGTAGGA
BCL11A Syn77-108
2nd PCR
| BCL11A 2nd Primer | Sequence with barcode |
| --- | --- |
| 4051-SyncrBCL11A9af-150PE-BC1 | tcacgCCAGACAATCGCCTTTTGCC |
| 4052-SyncrBCL11A9af-150PE-BC2 | gatgtCCAGACAATCGCCTTTTGCC |
| 4053-SyncrBCL11A9af-150PE-BC3 | taggcCCAGACAATCGCCTTTTGCC |
| 4054-SyncrBCL11A9af-150PE-BC4 | gaccaCCAGACAATCGCCTTTTGCC |
| 4055-SyncrBCL11A9af-150PE-BC5 | cagtgCCAGACAATCGCCTTTTGCC |
| 4056-SyncrBCL11A9be-150PE-BC1 | tcacgTTCAGAGCAACCTGGTGGTG |
| 4057-SyncrBCL11A9be-150PE-BC2 | gatgtTTCAGAGCAACCTGGTGGTG |
| 4058-SyncrBCL11A9be-150PE-BC3 | taggcTTCAGAGCAACCTGGTGGTG |
| 4059-SyncrBCL11A9be-150PE-BC4 | gaccaTTCAGAGCAACCTGGTGGTG |
| 4060-SyncrBCL11A9be-150PE-BC5 | cagtgTTCAGAGCAACCTGGTGGTG |
| 4061-Syn105crBCL11A9g-150PE-BC1 | tcacgAAACTTCTGCACTGGAGGGG |
| 4062-Syn105crBCL11A9g-150PE-BC2 | gatgtAAACTTCTGCACTGGAGGGG |
| 4063-Syn105crBCL11A9g-150PE-BC3 | taggcAAACTTCTGCACTGGAGGGG |
| 4064-Syn105crBCL11A9g-150PE-BC4 | gaccaAAACTTCTGCACTGGAGGGG |
| 4065-Syn105crBCL11A9g-150PE-BC5 | cagtgAAACTTCTGCACTGGAGGGG |
| 4098-Syn95crBCL11A8a-150PE-BC1 | tcacgCACTTCCTAGCCAGCAACCA |
| 4099-Syn95crBCL11A8a-150PE-BC2 | gatgtCACTTCCTAGCCAGCAACCA |
| 4100-Syn95crBCL11A8a-150PE-BC3 | taggcCACTTCCTAGCCAGCAACCA |
| 4101-Syn95crBCL11A8a-150PE-BC4 | gaccaCACTTCCTAGCCAGCAACCA |
| 4102-Syn95crBCL11A8a-150PE-BC5 | cagtgCACTTCCTAGCCAGCAACCA |
| 4103-Syn97crBCL11A8c-150PE-BC1 | tcacgTTCCCCTGAAGTCCACACAC |
| 4104-Syn97crBCL11A8c-150PE-BC2 | gatgtTTCCCCTGAAGTCCACACAC |
| 4105-Syn97crBCL11A8c-150PE-BC3 | taggcTTCCCCTGAAGTCCACACAC |
| 4106-Syn97crBCL11A8c-150PE-BC4 | gaccaTTCCCCTGAAGTCCACACAC |
| 4107-Syn97crBCL11A8c-150PE-BC5 | cagtgTTCCCCTGAAGTCCACACAC |
| 4108-Syn98crBCL11A8d-150PE-BC1 | tcacgAAAGGGGCACGCAAAATGTC |
| 4109-Syn98crBCL11A8d-150PE-BC2 | gatgtAAAGGGGCACGCAAAATGTC |
| 4110-Syn98crBCL11A8d-150PE-BC3 | taggcAAAGGGGCACGCAAAATGTC |
| 4111-Syn98crBCL11A8d-150PE-BC4 | gaccaAAAGGGGCACGCAAAATGTC |
| 4112-Syn98crBCL11A8d-150PE-BC5 | cagtgAAAGGGGCACGCAAAATGTC |
| 4189-Syn8485crBCL11A4cd-150PE-BC1 | tcacgAAGCTTGCACGAAAACACCG |
| 4190-Syn8485crBCL11A4cd-150PE-BC2 | gatgtAAGCTTGCACGAAAACACCG |
| 4191-Syn8485crBCL11A4cd-150PE-BC3 | taggcAAGCTTGCACGAAAACACCG |
| 4192-Syn8485crBCL11A4cd-150PE-BC4 | gaccaAAGCTTGCACGAAAACACCG |
| 4193-Syn8485crBCL11A4cd-150PE-BC5 | cagtgAAGCTTGCACGAAAACACCG |
| 4194-SyncrBCL11A9cd-150PE-BC1 | tcacgGCCTGTCCAAAAAGCTGCTG |
| 4195-SyncrBCL11A9cd-150PE-BC2 | gatgtGCCTGTCCAAAAAGCTGCTG |
| 4196-SyncrBCL11A9cd-150PE-BC3 | taggcGCCTGTCCAAAAAGCTGCTG |
| 4197-SyncrBCL11A9cd-150PE-BC4 | gaccaGCCTGTCCAAAAAGCTGCTG |
| 4198-SyncrBCL11A9cd-150PE-BC5 | cagtgGCCTGTCCAAAAAGCTGCTG |
| 3860-SyncrBCL11A9af-150PE-R | GTGTGGCTTCTCCTGTGGAC |
| 3862-SyncrBCL11A9be-150PE-R | GGGTCGTTCTCGCTCTTGAA |
| 3866-Syn105crBCL11A9g-150PE-R | TGCCAAGTGAGTAATGGAATAATAC |
| 3852-Syn95crBCL11A8a-150PE-R | AGCCTCCCTAGGGCTTAACT |
| 3856-Syn97crBCL11A8c-150PE-R | CTCACCTCCCAGTGAGGAAC |
| 3858-Syn98crBCL11A8d-150PE-R | CCCTGAAACTCTCTCATGGTCC |
| 3834-Syn8485crBCL11A4cd-150PE-R | ACCACAGAAGCACCAAGGAG |
| 3864-SyncrBCL11A9cd-150PE-R | GGAGGCAAAAGGCGATTGTC |
| MYH6 Primer | Sequence with barcode |
| --- | --- |
| 3342-MYH6-150PE-F-BC1 | tcacgGGAACCTCACTCTTGCCAAC |
| 3343-MYH6-150PE-F-BC2 | gatgtGGAACCTCACTCTTGCCAAC |
| 3344-MYH6-150PE-F-BC3 | taggcGGAACCTCACTCTTGCCAAC |
| 3345-MYH6-150PE-F-BC4 | gaccaGGAACCTCACTCTTGCCAAC |
| 3346-MYH6-150PE-F-BC5 | catgtGGAACCTCACTCTTGCCAAC |
| 3347-MYH6-150PE-F-BC6 | ccaatGGAACCTCACTCTTGCCAAC |
| 3348-MYH6-150PE-F-BC7 | agatcGGAACCTCACTCTTGCCAAC |
| 3349-MYH6-150PE-F-BC8 | cttgaGGAACCTCACTCTTGCCAAC |
| 3364-MYH6-150PE-F-BC9 | ttgtaGGAACCTCACTCTTGCCAAC |
| 3502-MYH6-150PE-F-BC11 | aacgcaGGAACCTCACTCTTGCCAAC |
| 3503-MYH6-150PE-F-BC12 | acactgGGAACCTCACTCTTGCCAAC |
| 3504-MYH6-150PE-F-BC13 | agcataGGAACCTCACTCTTGCCAAC |
| 3505-MYH6-150PE-F-BC14 | attaccGGAACCTCACTCTTGCCAAC |
| 3506-MYH6-150PE-F-BC15 | gattgaGGAACCTCACTCTTGCCAAC |
| 3507-MYH6-150PE-F-BC16 | tctctcGGAACCTCACTCTTGCCAAC |
| 3508-MYH6-150PE-F-BC17 | tggtcaGGAACCTCACTCTTGCCAAC |
| 3509-MYH6-150PE-F-BC18 | ttaaggGGAACCTCACTCTTGCCAAC |
| 3510-MYH6-150PE-F-BC19 | ggctagGGAACCTCACTCTTGCCAAC |
| 3316-MYH6-150PE-R | CTCTCCACCCTTGTGTGGTT |
BCL11A Syn77-108
1st PCR targeting outside of homology arms
| BCL11A 1st Primer | Sequence |
| --- | --- |
| 3794-Syn77-Hout-F | CGGCTGCGGTTATTCATTATT |
| 3795-Syn77-Hout-R | CCTGGCTTGGTCTGAGGTTC |
| 3796-Syn78-Hout-F | AGGTTGCTGATTACACCTCCAC |
| 3797-Syn78-Hout-R | ACAGAATGAATCTCAGGACCCG |
| 3798-Syn79-Hout-F | GCCTGGAACGTCTCAATGTC |
| 3799-Syn79-Hout-R | CTCTGCTATGTGGGCTGAATG |
| 3800-Syn83-Hout-F | TGCGAGCGAGGGAAATAAGA |
| 3801-Syn83-Hout-R | CTGCAGTGGGCTGAATGAGA |
| 3802-Syn87-Hout-F | TGAACCAGCATTATGTTAGGCA |
| 3803-Syn87-Hout-R | GGATGACGGTGAGTTACGATCT |
| 3804-Syn9293-Hout-F | CCTTGGAGTTACTGGCTTATGA |
| 3805-Syn9293-Hout-R | TGTGATTGGTGTCTCACTTTGA |
| 3806-Syn94-Hout-F | GCAAAGAAGTTAGTCTCAGCCA |
| 3807-Syn94-Hout-R | ACTGGTGAGTATGTGAGGAAGG |
| 3808-Syn96-Hout-F | TATTTACCGAGTGCCTACAATGC |
| 3809-Syn96-Hout-R | AGTCACGAAACAGCTGGTGAGTG |
| 3810-Syn99-Hout-F | GCATTCAAACGGTGAGAACAT |
| 3811-Syn99-Hout-R | GTCCTGGGCGAGAAGCATAAG |
| 3812-Syn103-Hout-F | AGTCTTCGTCGCAAGTGTCC |
| 3813-Syn103-Hout-R | CAAACAGCCATTCACCAGTG |
| 3814-Syn105-Hout-F | TATACCCAAAGTCCTAAATGAGG |
| 3815-Syn105-Hout-R | TTCCAGTTGACAGGATTCTAGAG |

## Slide 19
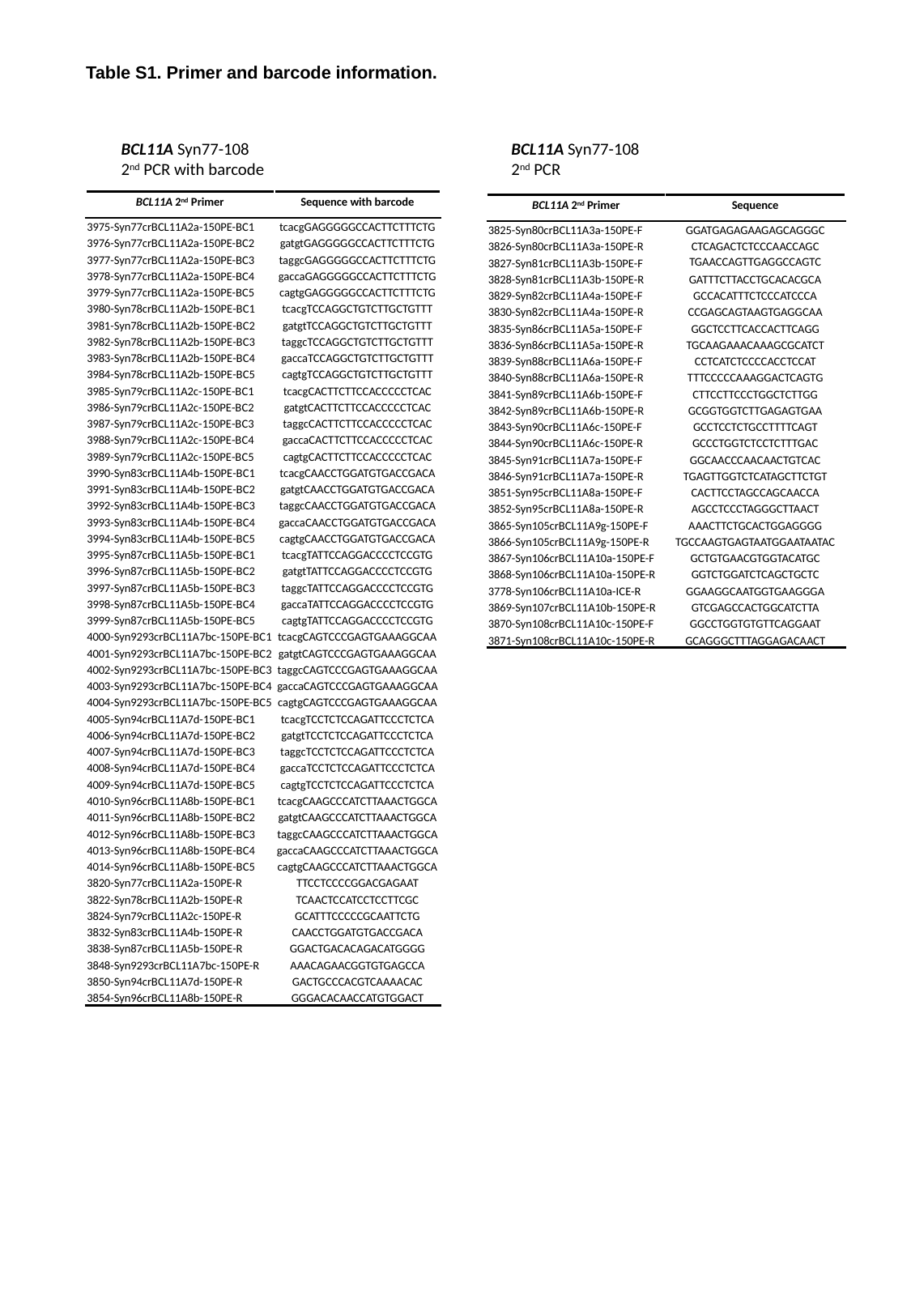

Table S1. Primer and barcode information.
BCL11A Syn77-108
2nd PCR with barcode
BCL11A Syn77-108
2nd PCR
| BCL11A 2nd Primer | Sequence with barcode |
| --- | --- |
| 3975-Syn77crBCL11A2a-150PE-BC1 | tcacgGAGGGGGCCACTTCTTTCTG |
| 3976-Syn77crBCL11A2a-150PE-BC2 | gatgtGAGGGGGCCACTTCTTTCTG |
| 3977-Syn77crBCL11A2a-150PE-BC3 | taggcGAGGGGGCCACTTCTTTCTG |
| 3978-Syn77crBCL11A2a-150PE-BC4 | gaccaGAGGGGGCCACTTCTTTCTG |
| 3979-Syn77crBCL11A2a-150PE-BC5 | cagtgGAGGGGGCCACTTCTTTCTG |
| 3980-Syn78crBCL11A2b-150PE-BC1 | tcacgTCCAGGCTGTCTTGCTGTTT |
| 3981-Syn78crBCL11A2b-150PE-BC2 | gatgtTCCAGGCTGTCTTGCTGTTT |
| 3982-Syn78crBCL11A2b-150PE-BC3 | taggcTCCAGGCTGTCTTGCTGTTT |
| 3983-Syn78crBCL11A2b-150PE-BC4 | gaccaTCCAGGCTGTCTTGCTGTTT |
| 3984-Syn78crBCL11A2b-150PE-BC5 | cagtgTCCAGGCTGTCTTGCTGTTT |
| 3985-Syn79crBCL11A2c-150PE-BC1 | tcacgCACTTCTTCCACCCCCTCAC |
| 3986-Syn79crBCL11A2c-150PE-BC2 | gatgtCACTTCTTCCACCCCCTCAC |
| 3987-Syn79crBCL11A2c-150PE-BC3 | taggcCACTTCTTCCACCCCCTCAC |
| 3988-Syn79crBCL11A2c-150PE-BC4 | gaccaCACTTCTTCCACCCCCTCAC |
| 3989-Syn79crBCL11A2c-150PE-BC5 | cagtgCACTTCTTCCACCCCCTCAC |
| 3990-Syn83crBCL11A4b-150PE-BC1 | tcacgCAACCTGGATGTGACCGACA |
| 3991-Syn83crBCL11A4b-150PE-BC2 | gatgtCAACCTGGATGTGACCGACA |
| 3992-Syn83crBCL11A4b-150PE-BC3 | taggcCAACCTGGATGTGACCGACA |
| 3993-Syn83crBCL11A4b-150PE-BC4 | gaccaCAACCTGGATGTGACCGACA |
| 3994-Syn83crBCL11A4b-150PE-BC5 | cagtgCAACCTGGATGTGACCGACA |
| 3995-Syn87crBCL11A5b-150PE-BC1 | tcacgTATTCCAGGACCCCTCCGTG |
| 3996-Syn87crBCL11A5b-150PE-BC2 | gatgtTATTCCAGGACCCCTCCGTG |
| 3997-Syn87crBCL11A5b-150PE-BC3 | taggcTATTCCAGGACCCCTCCGTG |
| 3998-Syn87crBCL11A5b-150PE-BC4 | gaccaTATTCCAGGACCCCTCCGTG |
| 3999-Syn87crBCL11A5b-150PE-BC5 | cagtgTATTCCAGGACCCCTCCGTG |
| 4000-Syn9293crBCL11A7bc-150PE-BC1 | tcacgCAGTCCCGAGTGAAAGGCAA |
| 4001-Syn9293crBCL11A7bc-150PE-BC2 | gatgtCAGTCCCGAGTGAAAGGCAA |
| 4002-Syn9293crBCL11A7bc-150PE-BC3 | taggcCAGTCCCGAGTGAAAGGCAA |
| 4003-Syn9293crBCL11A7bc-150PE-BC4 | gaccaCAGTCCCGAGTGAAAGGCAA |
| 4004-Syn9293crBCL11A7bc-150PE-BC5 | cagtgCAGTCCCGAGTGAAAGGCAA |
| 4005-Syn94crBCL11A7d-150PE-BC1 | tcacgTCCTCTCCAGATTCCCTCTCA |
| 4006-Syn94crBCL11A7d-150PE-BC2 | gatgtTCCTCTCCAGATTCCCTCTCA |
| 4007-Syn94crBCL11A7d-150PE-BC3 | taggcTCCTCTCCAGATTCCCTCTCA |
| 4008-Syn94crBCL11A7d-150PE-BC4 | gaccaTCCTCTCCAGATTCCCTCTCA |
| 4009-Syn94crBCL11A7d-150PE-BC5 | cagtgTCCTCTCCAGATTCCCTCTCA |
| 4010-Syn96crBCL11A8b-150PE-BC1 | tcacgCAAGCCCATCTTAAACTGGCA |
| 4011-Syn96crBCL11A8b-150PE-BC2 | gatgtCAAGCCCATCTTAAACTGGCA |
| 4012-Syn96crBCL11A8b-150PE-BC3 | taggcCAAGCCCATCTTAAACTGGCA |
| 4013-Syn96crBCL11A8b-150PE-BC4 | gaccaCAAGCCCATCTTAAACTGGCA |
| 4014-Syn96crBCL11A8b-150PE-BC5 | cagtgCAAGCCCATCTTAAACTGGCA |
| 3820-Syn77crBCL11A2a-150PE-R | TTCCTCCCCGGACGAGAAT |
| 3822-Syn78crBCL11A2b-150PE-R | TCAACTCCATCCTCCTTCGC |
| 3824-Syn79crBCL11A2c-150PE-R | GCATTTCCCCCGCAATTCTG |
| 3832-Syn83crBCL11A4b-150PE-R | CAACCTGGATGTGACCGACA |
| 3838-Syn87crBCL11A5b-150PE-R | GGACTGACACAGACATGGGG |
| 3848-Syn9293crBCL11A7bc-150PE-R | AAACAGAACGGTGTGAGCCA |
| 3850-Syn94crBCL11A7d-150PE-R | GACTGCCCACGTCAAAACAC |
| 3854-Syn96crBCL11A8b-150PE-R | GGGACACAACCATGTGGACT |
| BCL11A 2nd Primer | Sequence |
| --- | --- |
| 3825-Syn80crBCL11A3a-150PE-F | GGATGAGAGAAGAGCAGGGC |
| 3826-Syn80crBCL11A3a-150PE-R | CTCAGACTCTCCCAACCAGC |
| 3827-Syn81crBCL11A3b-150PE-F | TGAACCAGTTGAGGCCAGTC |
| 3828-Syn81crBCL11A3b-150PE-R | GATTTCTTACCTGCACACGCA |
| 3829-Syn82crBCL11A4a-150PE-F | GCCACATTTCTCCCATCCCA |
| 3830-Syn82crBCL11A4a-150PE-R | CCGAGCAGTAAGTGAGGCAA |
| 3835-Syn86crBCL11A5a-150PE-F | GGCTCCTTCACCACTTCAGG |
| 3836-Syn86crBCL11A5a-150PE-R | TGCAAGAAACAAAGCGCATCT |
| 3839-Syn88crBCL11A6a-150PE-F | CCTCATCTCCCCACCTCCAT |
| 3840-Syn88crBCL11A6a-150PE-R | TTTCCCCCAAAGGACTCAGTG |
| 3841-Syn89crBCL11A6b-150PE-F | CTTCCTTCCCTGGCTCTTGG |
| 3842-Syn89crBCL11A6b-150PE-R | GCGGTGGTCTTGAGAGTGAA |
| 3843-Syn90crBCL11A6c-150PE-F | GCCTCCTCTGCCTTTTCAGT |
| 3844-Syn90crBCL11A6c-150PE-R | GCCCTGGTCTCCTCTTTGAC |
| 3845-Syn91crBCL11A7a-150PE-F | GGCAACCCAACAACTGTCAC |
| 3846-Syn91crBCL11A7a-150PE-R | TGAGTTGGTCTCATAGCTTCTGT |
| 3851-Syn95crBCL11A8a-150PE-F | CACTTCCTAGCCAGCAACCA |
| 3852-Syn95crBCL11A8a-150PE-R | AGCCTCCCTAGGGCTTAACT |
| 3865-Syn105crBCL11A9g-150PE-F | AAACTTCTGCACTGGAGGGG |
| 3866-Syn105crBCL11A9g-150PE-R | TGCCAAGTGAGTAATGGAATAATAC |
| 3867-Syn106crBCL11A10a-150PE-F | GCTGTGAACGTGGTACATGC |
| 3868-Syn106crBCL11A10a-150PE-R | GGTCTGGATCTCAGCTGCTC |
| 3778-Syn106crBCL11A10a-ICE-R | GGAAGGCAATGGTGAAGGGA |
| 3869-Syn107crBCL11A10b-150PE-R | GTCGAGCCACTGGCATCTTA |
| 3870-Syn108crBCL11A10c-150PE-F | GGCCTGGTGTGTTCAGGAAT |
| 3871-Syn108crBCL11A10c-150PE-R | GCAGGGCTTTAGGAGACAACT |

## Slide 20
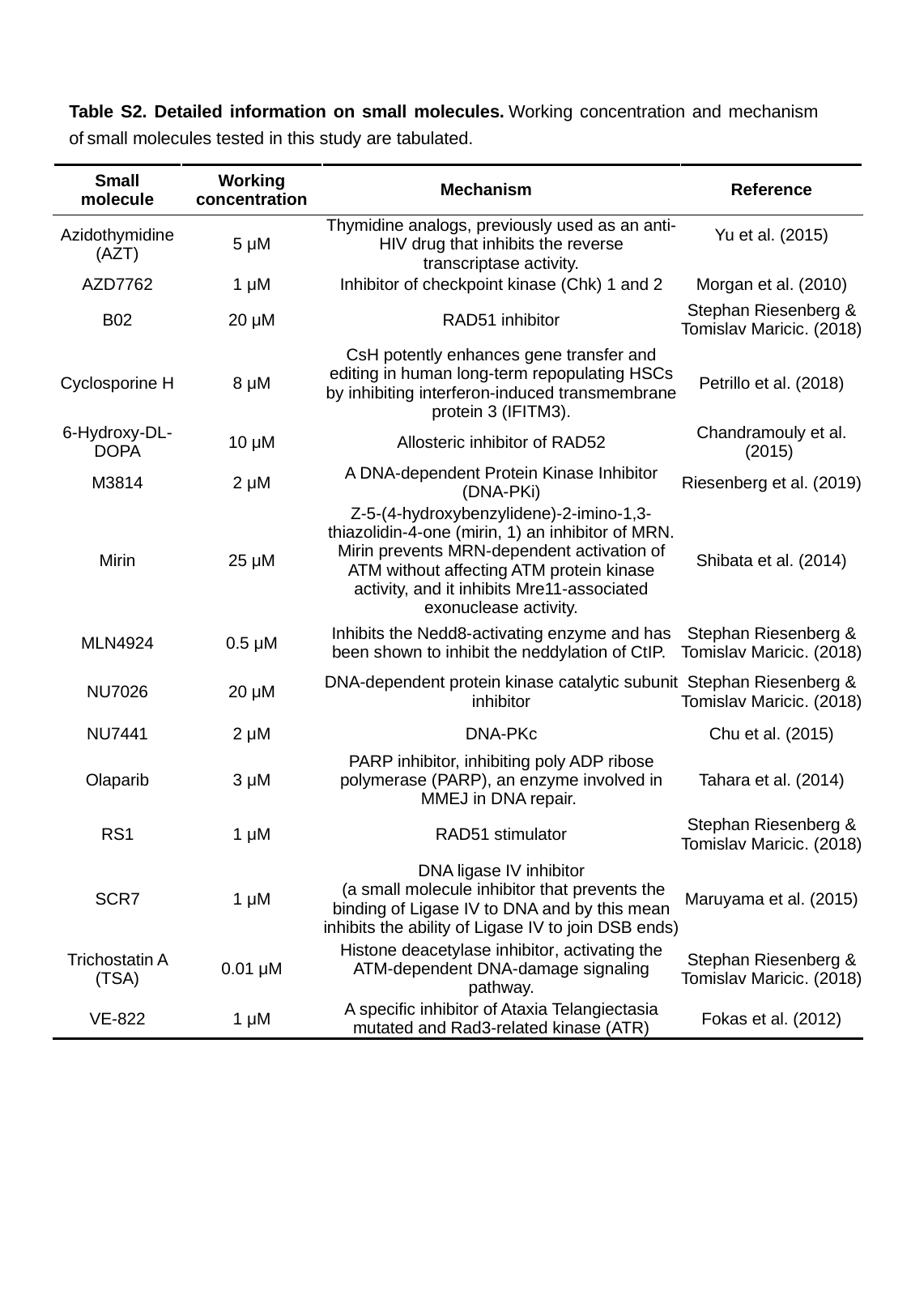

Table S2. Detailed information on small molecules. Working concentration and mechanism of small molecules tested in this study are tabulated.
| Small molecule | Working concentration | Mechanism | Reference |
| --- | --- | --- | --- |
| Azidothymidine (AZT) | 5 μM | Thymidine analogs, previously used as an anti-HIV drug that inhibits the reverse transcriptase activity. | Yu et al. (2015) |
| AZD7762 | 1 μM | Inhibitor of checkpoint kinase (Chk) 1 and 2 | Morgan et al. (2010) |
| B02 | 20 μM | RAD51 inhibitor | Stephan Riesenberg & Tomislav Maricic. (2018) |
| Cyclosporine H | 8 μM | CsH potently enhances gene transfer and editing in human long-term repopulating HSCs by inhibiting interferon-induced transmembrane protein 3 (IFITM3). | Petrillo et al. (2018) |
| 6-Hydroxy-DL-DOPA | 10 μM | Allosteric inhibitor of RAD52 | Chandramouly et al. (2015) |
| M3814 | 2 μM | A DNA-dependent Protein Kinase Inhibitor (DNA-PKi) | Riesenberg et al. (2019) |
| Mirin | 25 μM | Z-5-(4-hydroxybenzylidene)-2-imino-1,3-thiazolidin-4-one (mirin, 1) an inhibitor of MRN. Mirin prevents MRN-dependent activation of ATM without affecting ATM protein kinase activity, and it inhibits Mre11-associated exonuclease activity. | Shibata et al. (2014) |
| MLN4924 | 0.5 μM | Inhibits the Nedd8-activating enzyme and has been shown to inhibit the neddylation of CtIP. | Stephan Riesenberg & Tomislav Maricic. (2018) |
| NU7026 | 20 μM | DNA-dependent protein kinase catalytic subunit inhibitor | Stephan Riesenberg & Tomislav Maricic. (2018) |
| NU7441 | 2 μM | DNA-PKc | Chu et al. (2015) |
| Olaparib | 3 μM | PARP inhibitor, inhibiting poly ADP ribose polymerase (PARP), an enzyme involved in MMEJ in DNA repair. | Tahara et al. (2014) |
| RS1 | 1 μM | RAD51 stimulator | Stephan Riesenberg & Tomislav Maricic. (2018) |
| SCR7 | 1 μM | DNA ligase IV inhibitor ‎ (a small molecule inhibitor that prevents the binding of Ligase IV to DNA and by this mean inhibits the ability of Ligase IV to join DSB ends) | Maruyama et al. (2015) |
| Trichostatin A (TSA) | 0.01 μM | Histone deacetylase inhibitor, activating the ATM-dependent DNA-damage signaling pathway. | Stephan Riesenberg & Tomislav Maricic. (2018) |
| VE-822 | 1 μM | A specific inhibitor of Ataxia Telangiectasia mutated and Rad3-related kinase (ATR) | Fokas et al. (2012) |
